# Carbon Atom Insertion into Pyrroles and Indoles Promoted by Chlorodiazirines

**DOI:** 10.1021/jacs.1c06287

**Published:** 2021-07-21

**Authors:** Balu D. Dherange, Patrick Q. Kelly, Jordan P. Liles, Matthew S. Sigman, Mark D. Levin

**Affiliations:** † Department of Chemistry, University of Chicago, Chicago, Illinois 60637, United States; ‡ Department of Chemistry, University of Utah, Salt Lake City, Utah 84112, United States

## Abstract

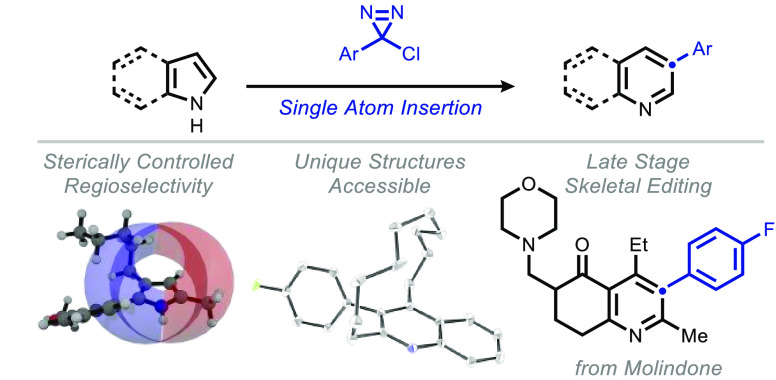

Herein, we report a reaction that selectively generates 3-arylpyridine and quinoline motifs by inserting aryl carbynyl cation equivalents into pyrrole and indole cores, respectively. By employing α-chlorodiazirines as thermal precursors to the corresponding chlorocarbenes, the traditional haloform-based protocol central to the parent Ciamician-Dennstedt rearrangement can be modified to directly afford 3-(hetero)arylpyridines and quinolines. Chlorodiazirines are conveniently prepared in a single step by oxidation of commercially available amidinium salts. Selectivity as a function of pyrrole substitution pattern was examined, and a predictive model based on steric effects is put forward, with DFT calculations supporting a selectivity-determining cyclopropanation step. Computations surprisingly indicate that the stereochemistry of cyclopropanation is of little consequence to the subsequent electrocyclic ring opening that forges the pyridine core, due to a compensatory homoaromatic stabilization that counterbalances orbital-controlled torquoselectivity effects. The utility of this skeletal transform is further demonstrated through the preparation of quinolinophanes and the skeletal editing of pharmaceutically relevant pyrroles.

In recent years, molecular editing has taken root as an approach to diversify the suite of complexity-building reactions available to the synthetic community.^1−5^ This paradigm has so far chiefly focused on C–H functionalization (i.e., peripheral editing, Figure 1A^6−8^), which, while effective, does not harness the immense potential manifest in the underlying molecular skeleton. Indeed, by their nature, C–H bonds are necessarily peripheral sites for reactivity, and the development of a complementary set of skeletally focused (i.e., C–C, C–N, C–O editing) reactions would have a synergistic effect on access to complex molecular scaffolds.^9,10^

**Figure 1:**
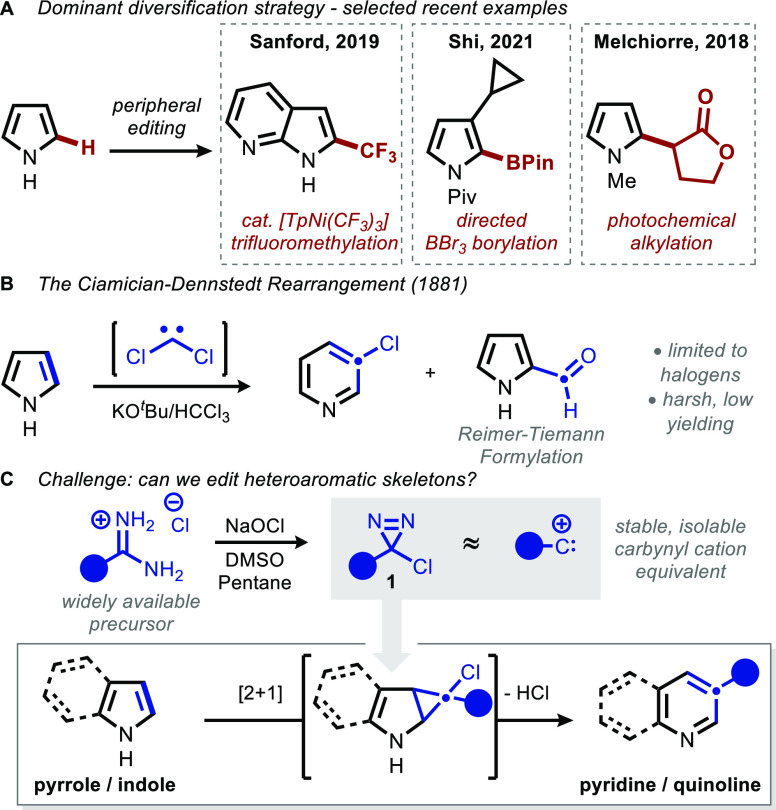
Introduction. (A) selected recent examples of peripheral editing of pyrroles and indoles; (B) the classical Ciamician–Dennstedt Rearrangement; (C) skeletal editing logic for heterocycle diversification (this work).

In this vein, “single-atom” manipulations of ring systems (i.e., targeted insertions or deletions) are of particular interest, in part due to their retrosynthetic simplicity.^11−14^ Such reactions are known for a limited subset of molecules, including venerable carbonyl rearrangements such as the Bayer–Villiger, Beckmann, and Wolff rearrangements.^15−18^ However, the practical attractiveness of these classic reactions varies greatly from case to case by virtue of their conditions and limitations. The Ciamician–Dennstedt rearrangement (Figure 1B) represents a stark example of such a transformation; the attractive underlying retrosynthetic logic is hindered by practical limitations that have largely precluded its widespread adoption.^19,20^ The reaction is principally limited to the production of 3-halopyridines through haloform-derived carbenes, and typical yields and functional group tolerances are low, due in part to competitive Reimer–Tiemann formylation.^21^ The potential of the underlying transformation, however, spurred us to identify an alternative protocol to access polysubstituted pyridines and quinolines. These targets are prevalent motifs among medicinal compounds, with contributions from numerous laboratories to their synthesis in recent years.^22−36^

The key intermediate for the desired transformation is a carbenic center bearing an appropriate leaving group (i.e., a carbynyl cation equivalent). Though benzal halides have been employed toward this purpose, the procedures are typically low yielding.^37^ α-halo diazoalkanes have similarly been reported, but their intrinsic instability has limited their use.^38−40^ Suero has recently reported the related α-iodonium diazo compounds as surprisingly stable, isolable carbynyl cation equivalents, though despite increased stability relative to the parent α-halo compounds, Suero reagents retain the requirement of a stabilizing electron-withdrawing group.^41−44^ Moreover, the associated oxidizing capacity of iodine(III) limits their application to reducing substrates such as pyrroles and indoles.

Aware of these limitations, we turned our attention to diazirines, which are the cyclic valence isomers of diazo compounds.^45^ Though similarly capable of serving as carbene precursors through extrusion of N_2_, diazirines are typically more stable, allowing isolation of carbene precursors lacking electron-withdrawing functionality.^46−50^ The most commonly encountered diazirines are the trifluoromethyl derivatives, which are often applied as photoaffinity probes in biological applications.^51,52^ However, importantly for our purposes, the corresponding α-chlorodiazirines (**1**) are much more easily prepared than their trifluoromethyl analogues via the single-step Graham oxidation of amidine precursors (Figure 1C).^53,54^ Simple treatment with bleach directly affords a diverse range of chlorodiazirines (see the experimental Supporting Information (SI) for details). Indeed, hundreds of amidine precursors bearing diverse substitution patterns are commercially available, enabling the straightforward preparation of a library of reagents.^55^

With these compounds in hand, we examined their potential for Ciamician–Dennstedt-type ring expansions, initially with indole substrates (Figure 2). Optimization revealed that sodium carbonate in acetonitrile afforded high yields, with inorganic bases proving critical for the formation of the desired quinoline products (**3**). We suspect this beneficial effect to be a consequence of chloride-scavenging by sodium, given that addition of Bu_4_NCl causes dramatic decreases in the isolated yield of **3**, with attendant formation of benzal chloride (see the experimental SI Section VIA).^56−59^ Solvents other than acetonitrile afforded varying quantities of carbene-trapping side products.^60^ Though the reaction proceeded with similar yields at a range of temperatures, heating at 50 °C allowed the process to proceed at a convenient rate, generally reaching full conversion in 12 h.

**Figure 2:**
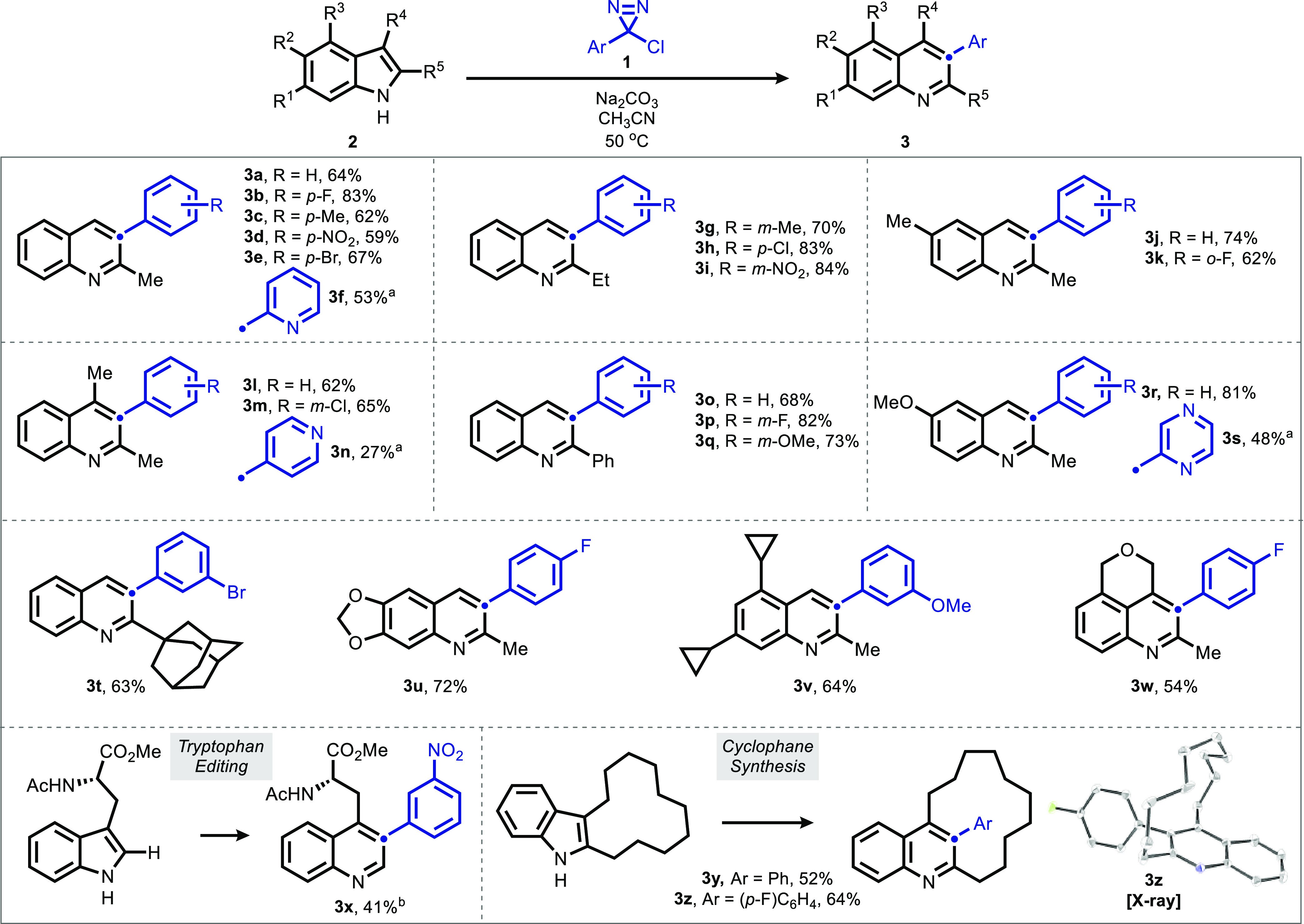
Scope of the indole-to-quinoline ring expansion. Conditions: **2** (1 equiv), **1** (3 equiv), Na_2_CO_3_ (3 equiv), CH_3_CN (0.1 M), 50 °C, 12 h. Isolated yields, 0.1–0.3 mmol scale. ^*a*^5 equiv of **1**. ^*b*^48 h.

Indoles substituted at the 2-position were found to be particularly effective substrates, though substitution at multiple positions was well-tolerated provided that the indole was relatively electron-rich. This allowed for the preparation of diversely substituted quinolines (entries **3a**–**3x**). Though a protected tryptophan derivative could be converted to the corresponding quinoline **3x** in 41% yield, in the absence of a 2-substituent, yields were generally lower (see the experimental SI for additional examples). We suspect decomposition via pyridinium ylide intermediates is a deleterious pathway, as addition of 3 equiv of quinoline to the reaction of 2-phenyl indole with phenylchlorodiazirine afforded **3o** in 15% yield, compared to 68% in its absence.^61^

The synthesis of cyclophanes exemplifies the unique retrosynthetic logic enabled by this protocol. 2,3-Ring-fused indoles, easily prepared from cycloalkanones via a Fischer indole synthesis, afford ring expanded quinolinophanes **3y** and **3z**, providing ready access to an otherwise challenging class of compounds.^62−64^

Various diazirenes were found to be effective coupling partners, including *ortho*, *meta*, and *para* substituted arenes, as well as several heteroaryl carbene precursors. Products such as **3f**, **3n**, and **3s**, which bear heteroaryl-heteroaryl linkages, are considered challenging to prepare using cross-coupling; by formally moving the retrosynthetic disconnection inward by one carbon, indoles can be employed as analogues to 3-quinolyl nucleophiles.^65^ Even in cases where such heterocyclic diazirines are not employed, this method may offer an advantage—sequential application of the classical Cicamician–Dennstedt (excess CHCl_3_, aq. NaOH, BnEt_3_NCl) followed by Suzuki coupling with 3-fluorophenylboronic acid afforded **3k** in 22% yield over 2 steps, compared with 82% under the title conditions. A limitation was observed in moving to electron-rich diazirines, which exclusively afforded the corresponding aldehydes.^66,67^ Aliphatic diazirines were similarly poor coupling partners, either isomerizing to vinyl chlorides or undergoing competitive dimerization (see the experimental SI section IV).^68−71^

Pyrroles (**4**) represent a more complex substrate class due to the potential for regioisomeric products derived from insertion into the two “olefinic” sites of the substrate (Figure 3).^72^ The reaction was found to proceed efficiently with a range of pyrroles (though again displaying the 2-substitution constraint observed for indoles), affording good yields of the corresponding pyridines with tolerance for ester (**4d** and **4w**), thiophene (**4o**), and amide (**4p** and **4r**) functionality. Free alcohols, halopyrroles, and trialkyl pyrroles were not tolerated (see the experimental SI for details). In addition to symmetric pyrroles such as **4a** (which do not pose a regiochemical question), trisubstituted pyrroles (**4b**–**4e**) were found to give exquisite selectivity for insertion into the less-substituted side of the pyrrole.

**Figure 3:**
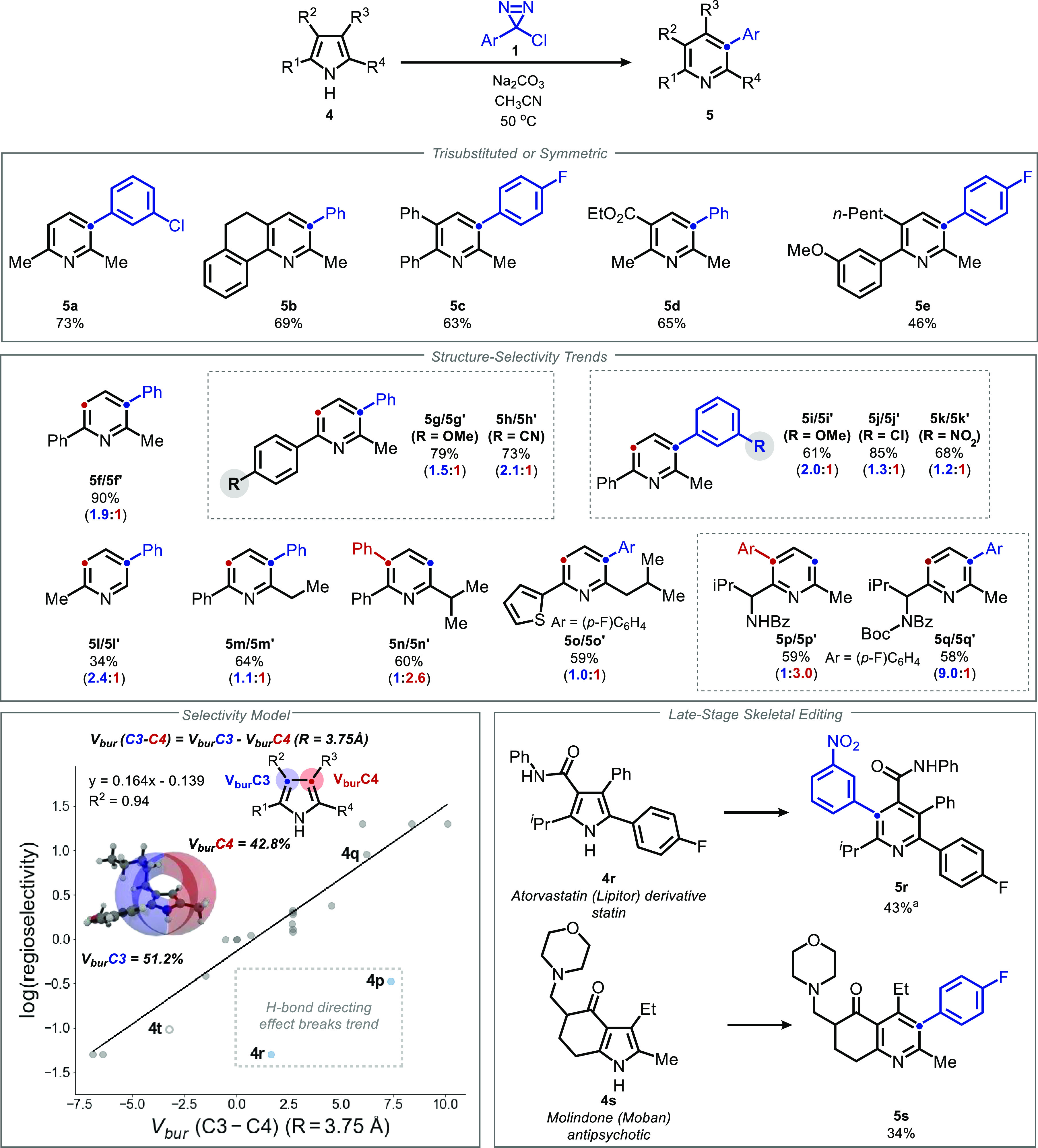
Scope and selectivity of the pyrrole-to-pyridine ring expansion. Conditions: **4** (1 equiv), **1** (3 equiv), Na_2_CO_3_ (3 equiv), CH_3_CN (0.1 M), 50 °C, 12 h. Isolated yields, 0.1–0.3 mmol scale. ^*a*^48 h. Regioisomer assignments supported by ^1^H-NOE. Selectivity model based on the difference between the Boltzmann averaged buried volume in a 3.75 Å sphere at C3 vs C4.

In asymmetric disubstituted pyrroles, mixtures of products were observed, allowing for structure-selectivity trends to be discerned. Though inspection of subsets of the pyrroles (e.g., {**4f**, **4m**, **4n**} vs {**4f**, **4g**, **4h**}) suggests a more significant role for steric effects than electronic effects, we sought a quantitative, predictive model applicable to the full data set and potentially of use to those seeking to adopt this method to other pyrroles.^73^ Molecular descriptors capturing steric and electronic features of the pyrroles were extracted as Boltzmann averages from Density Functional Theory (DFT) optimized conformers and correlated against the experimental product distribution. To maintain generality beyond the present data set, descriptors derived from either a difference or quotient of properties representing each side of the pyrrole were calculated. Accordingly, the best model was found to be a difference in buried volume at C3 and C4 of the pyrrole at a radius of 3.75 Å. This univariate model not only captured the high selectivity of trisubstituted pyrroles but also was able to accurately predict low-selectivity substrates such as **4o**.

Substrate **4p** was observed as an outlier in most models surveyed, and we hypothesized that this was due to hydrogen bonding between the −NHBz moiety and the carbene in the selectivity-determining step.^74−76^ To probe this, we prepared the doubly protected analogue **4q**, which blocked such hydrogen bonding effects. This substrate was effectively predicted by the steric model, consistent with the hydrogen-bonding hypothesis.

Armed with this insight into selectivity, we examined the late-stage skeletal editing of **4r** (N-des-alkyl Lipitor) and **4s** (Molindone). Both compounds afforded one major isomer—**5r** showing hydrogen-bond-donor-controlled selectivity and **5s** with a regioselectivity that was accurately predicted by our quantitative model. We note despite our moderate yields that the classical Ciamician–Dennstedt induces decomposition of molindone with no detectable pyridine formation. These examples showcase the potential for skeletal editing approaches to offer access to new chemical space in a medicinal chemistry campaign.

For some substrates, unusual *ortho* and *para* insertion products were observed (**5t**, **5v**, **5w**). These cannot be accounted for by a 2,3-cyclopropanation mechanism alone, forcing us to reexamine the potential reaction pathways (Figure 4).^77,78^ We considered the possibility that cyclopropanation is followed by cyclization to afford an azabenzvalene intermediate.^79−82^ However, DFT computations suggest that such a mechanism is implausible. The transition state for azabenzvalene formation from the exo-chlorocyclopropane **6** is predicted to be ∼16 kcal/mol higher in energy than the corresponding electrocyclic ring opening to afford **5t**. Instead, we suggest that cyclopropanation (or aziridination) of the 3,4 (or 1,2) linkage (respectively) is operative in the generation of the unusual regioisomeric products **5t′**, **5v′**, and **5w**. A plausible pathway was located computationally in which metastable zwitterionic 3,4-cyclopropane **7** forms through stepwise attack and ring closure (see the computational SI, Figure S6 for details). Intermediate **7** is likely stabilized by its phenyl substituent, as evidenced by the exclusive formation of the typical *meta* isomer from di-*tert*-butylpyrrole **4u**.

**Figure 4:**
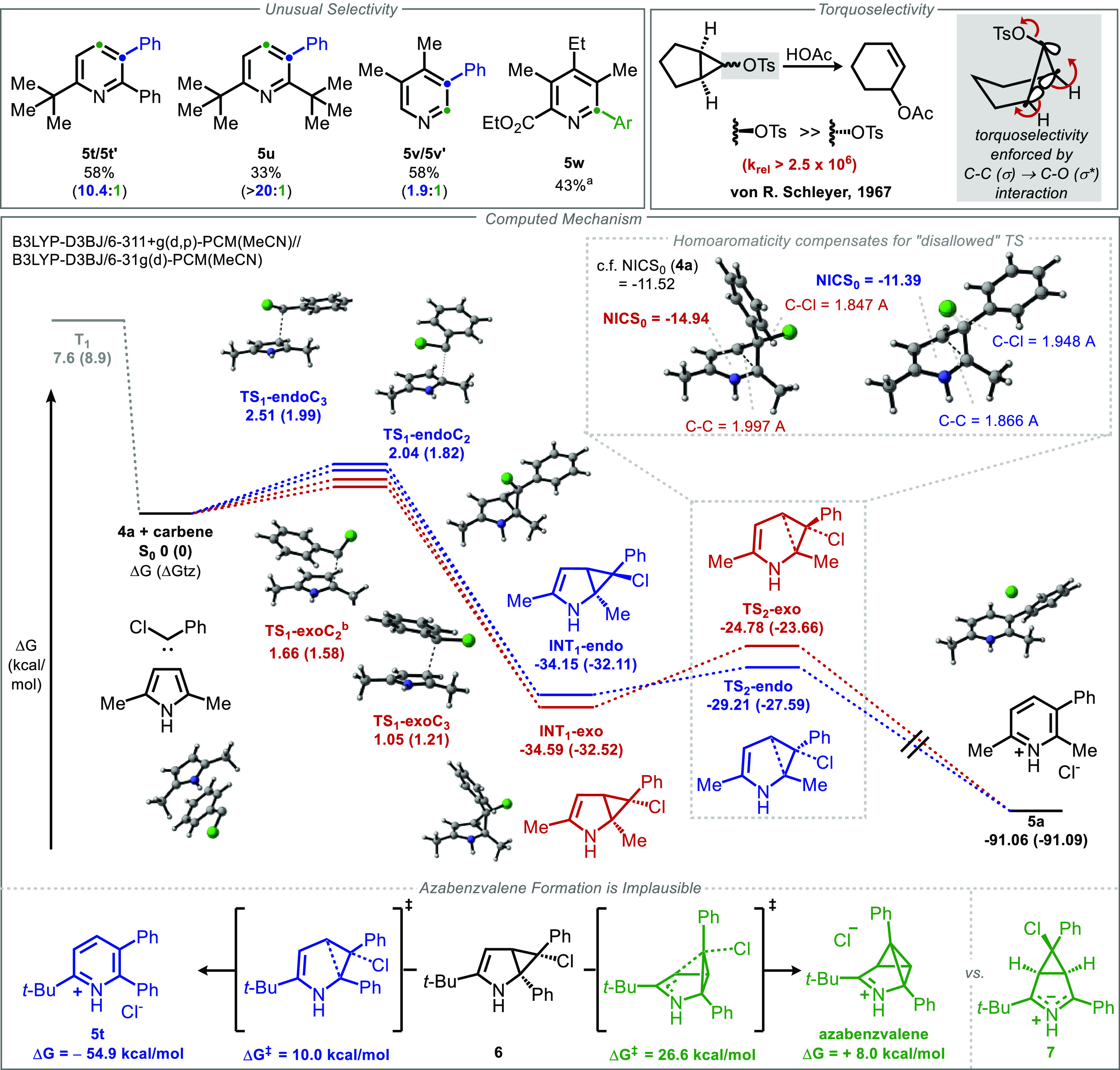
Unusual *ortho* and *para* isomers and computational investigation of their mechanism of formation. Conditions: **4** (1 equiv), **1** (3 equiv), Na_2_CO_3_ (3 equiv), CH_3_CN (0.1 M), 50 °C, 12 h. Isolated yields, 0.1–0.3 mmol scale. ^*a*^Unassigned minor isomer detected. *^b^*Carbene-C2 bond was frozen at length from B3LYP-D3/6-31g(d) optimization.

Finally, because our reagent generates a monochlorocarbene, cyclopropanation can in principle afford diastereomeric cyclopropanes, unlike the classical use of dichlorocarbene. Based on precedent in cyclopropyltosylate solvolyses, these diastereomers were expected to exhibit dramatically different rates of ring opening.^83−85^ Our computational investigations suggest that the intrinsic diastereoselectivity of the initial cyclopropanation is quite low, such that both diastereomers are likely formed under the reaction conditions. Despite these considerations, no cyclopropane byproducts have been detected, and experimental yields range as high as 90%. Moreover, the computationally predicted barrier for ring opening by the putatively forbidden pathway is surprisingly low.

In order to better understand this unexpected phenomenon, we analyzed the bond lengths and Nucleus Independent Chemical Shift (NICS) of each transition state.^86,87^ As expected, the disallowed transition state (**TS**_**2**_**-exo**, red) shows a lesser degree of C–Cl bond breaking than the allowed transition state (**TS**_**2**_**-endo**, blue), 1.85 Å vs 1.95 Å. However, this is accompanied by a greater degree of cyclopropane C–C bond-breaking (2.00 Å vs 1.86 Å), and a far more negative NICS_0_ value (−14.9 vs −11.4, compared to −11.5 for the parent pyrrole) indicating a greater degree of aromaticity in the disallowed transition state. Taken together, these results indicate that a substantial degree of homoaromaticity in the pyrrolic ring of the disallowed transition state compensates for the lack of C–C (σ) → C–Cl (σ*) interaction in the transition state.^88,89^

In conclusion, we have demonstrated that chlorodiazirine reagents enable a versatile new ring expansion reaction of pyrrole and indole substrates through the generation of aryl carbynyl cation equivalents. Mechanistic experiments and computations indicate that the regioselectivity is controlled by steric effects in a selectivity-determining cyclopropanation step, with diminished torquoselectivity effects in the subsequent ring opening due to homopyrrole character in the product-forming transition state. Ring expansion of fused indoles allows access to otherwise challenging quinolinophanes, and the method is applicable to the skeletal editing of medicinally relevant compounds. This method, coupled with the predictive model for its deployment, promises to enable direct interrogation of aromatic heterocycle skeletal editing as an innovative approach to synthetic and structural optimization campaigns.

## Supporting Information Available

Experimental procedures and characterization data (PDF)
Computational procedures and data (PDF)

## Accession Codes

CCDC 2090443 contains the supplementary crystallographic data for this paper. These data can be obtained free of charge via www.ccdc.cam.ac.uk/data_request/cif, or by emailing data_request@ccdc.cam.ac.uk, or by contacting The Cambridge Crystallographic Data Centre, 12 Union Road, Cambridge CB2 1EZ, UK; fax: +44 1223 336033.
