# Supplementary material for: Carbon Atom Insertion into Pyrroles and Indoles Promoted by Chlorodiazirines
Source: J Am Chem Soc. 2021 Jul 21;143(30):11337–44. doi: 10.1021/jacs.1c06287 (PMC8343525; doi:10.1021/jacs.1c06287)
Supplement: Supplementary file 1 — ja1c06287_si_001.pdf [file ja1c06287_si_001.pdf]

Supporting Information 1

# Carbon Atom Insertion into Pyrroles and Indoles Promoted by Chlorodiazirines

# Experimental Details and Spectra

Balu D. Dherange[1], Patrick Q. Kelly[1], Jordan P. Liles[2], Matthew S. Sigman[2], and Mark D. Levin[1,*]

[1]Department of Chemistry, University of Chicago, Chicago, IL 60637, United States

[2] Department of Chemistry, University of Utah, Salt Lake City, Utah 84112, United States

**Table of Contents**

I. Material and Methods: S1-2

II. General Procedure for synthesis of 3-substituted quinolines (3) / pyridines: S1-3

III. Synthesis of Starting Materials

IIIA. General procedure for synthesis of chlorodiazirine 1: S1-21

IIIB. Synthesis of indoles and pyrroles: S1-25

IV. Limitations: S1-30

V. Comparison with Ciamician-Denstedt Reaction: S1-31

VI. Mechanistic Experiments

VIA. Effect of addition of tetrabutyl ammonium chloride: S1-33

VIB. Effect of addition of quinoline: S1-34

VIII. X-Ray-derived ORTEP of 3z: S1-35

VIII. References: S1-37

IX. Spectra S1-39

## I. Material and Methods

Unless noted otherwise, all reactions were performed in oven-dried or flame-dried glassware under an atmosphere of dry $N_2$. $CH_3CN$, THF, $Et_2O$, $CH_2Cl_2$, toluene, and $Et_3N$ were dried by passing these previously degassed solvents through a PPT Solvent Purification System, and all other solvents were dried over molecular sieves (4A) and degassed prior to use or purchased an- hydrous and sealed under $N_2$ (e.g. VWR Dri-solv or equivalent). Reaction temperatures were reported as the temperatures of the bath surrounding the flasks or vials. Sensitive reagents and solvents were transferred under nitrogen into a nitrogen-filled glovebox with standard techniques. Unless otherwise noted, all reagents were used as received. Analytical thin-layer chromatography (TLC) was carried out using 0.2 mm commercial silica gel plates (silica gel 60, F254) and visualized by UV irradiation or staining as indicated.

High resolution mass spectra were recorded on either an Agilent 6224 TOF High Resolution Accurate MS with electrospray ionization or an Agilent 7200B QTOF High Resolution Accurate Mass GCMS using an Agilent HP-5MS column with a temperature gradient of 50 $^oC$ to 200 $^oC$ over 15 minutes and electron impact ionization. All mass spectra were processed with an Agilent MassHunter Operating System. X-Ray crystallographic analysis data were collected using a Bruker D8 VENTURE with PHOTON 100 CMOS detector system. Nuclear magnetic resonance spectra ($^1H$-NMR, $^{13}C$-NMR and $^{19}F$-NMR) were recorded with Bruker spectrometers operating at 400 or 500 MHz for 1H. Chemical shifts are reported in parts per million (ppm, δ), downfield from tetramethylsilane (TMS, δ=0.00 ppm) and are referenced to residual solvent (CDCl3, δ=7.26 ppm (1H) and 77.160 ppm (13C)). Coupling constants were reported in Hertz (Hz). Data for 1H-NMR spectra were reported as follows: chem- ical shift (ppm, s = singlet, d = doublet, t = triplet, q = quartet, quin = quintet, dd = doublet of doublets, td = triplet of doublets, ddd = doublet of doublet of doublets, m = multiplet, coupling constant (Hz), and integration).

**CAUTION!** Diazirines are reactive compounds that release gaseous byproducts. Though they are typically less reactive than the isomeric diazos and we have not encountered any stability issues with arylchlorocarbenes to date, lower molecular weight diazirines (e.g. methylchlorodiazirine) have been reported to detonate. Operations should be conducted behind a blast shield and scales should be limited whenever possible.

**II. General Procedure for synthesis of 3-substituted quinolines (3) and pyridines (5) from indoles (2) and pyrroles (4)**

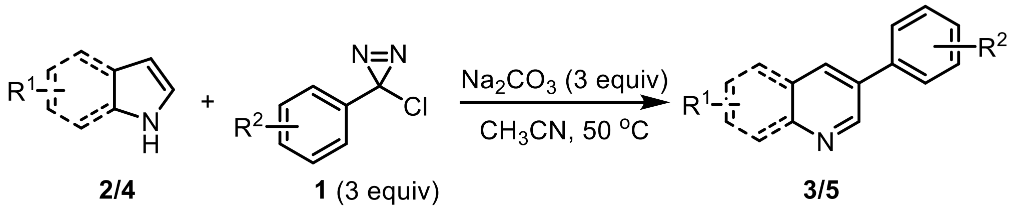

Oven dried 1-dram screw cap vial equipped with a stir bar and PTFE/white silicone septum was added indole **2** /pyrrole **4** (0.2 mmol.) and dry $CH_3CN$ (1 mL), followed by diazirine **1** (0.6 mmol) and $Na_2CO_3$ (0.6 mmol). The vial then sealed with electric tape. The mixture was then allowed to stir at 50 °C for 12 h and then allowed to cool to 25 °C. The reaction mixture was quenched by the addition of saturated aqueous $NH_4Cl$ solution (1 mL), and the two phases were separated. The aqueous layer was extracted with EtOAc (3 × 3 mL), and the combined organic layers were dried over anhydrous $Na_2SO_4$, filtered, and concentrated under reduced pressure. The obtained residue was purified by flash column chromatography to afford 3-substituted quinoline **3**/pyridine **5**.

**2-methyl-3-phenylquinoline (3a):**

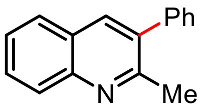

**3a**

Following the general procedure II, product **3a** was isolated in 64% yield as a colorless liquid. **3a**: $R_f$ = 0.6 (silica gel, 20% EtOAc in hexanes); **$^1$H NMR** (400 MHz, $CDCl_3$) δ 8.07 (dt, *J* = 8.4, 1.0 Hz, 1H), 7.97 (s, 1H), 7.80 (dd, *J* = 8.1, 1.4 Hz, 1H), 7.70 (ddd, *J* = 8.5, 6.9, 1.5 Hz, 1H), 7.54 – 7.37 (m, 6H), 2.68 (s, 3H). Spectroscopic data are in agreement with those in the literature.[1]

**3-(4-fluorophenyl)-2-methylquinoline (3b):**

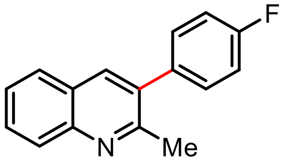

**3b**

Following the general procedure II, product **3b** was isolated in 83% yield as a pale yellow liquid. **3b**: $R_f$ = 0.5 (silica gel, 20% EtOAc in hexanes); **$^1$H NMR** (400 MHz, $CDCl_3$) δ 8.06 (dd, *J* = 8.5, 1.1 Hz, 1H), 7.94 (s, 1H), 7.79 (dd, *J* = 8.1, 1.4 Hz, 1H), 7.71 (ddd, *J* = 8.5, 6.9, 1.5 Hz, 1H), 7.51 (ddd, *J* = 8.1, 6.8, 1.1 Hz, 1H), 7.42 – 7.34 (m, 2H), 7.20 – 7.13 (m, 2H), 2.65 (s, 3H). Spectroscopic data are in agreement with those in the literature.[2]

**2-methyl-3-(*p*-tolyl)quinoline (3c):**

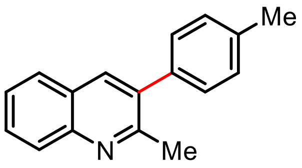


**3c**

Following the general procedure II, product **3c** was isolated in 62% yield as a yellow viscous liquid. **3c**: $R_f = 0.3$ (silica gel, 10% EtOAc in hexanes); **$^{1}$H NMR** (400 MHz, $CDCl_3$) δ 8.09 – 8.03 (m, 1H), 7.94 (s, 1H), 7.79 (dd, $J$ = 8.0, 1.4 Hz, 1H), 7.69 (ddd, $J$ = 8.4, 6.9, 1.5 Hz, 1H), 7.50 (ddd, $J$ = 8.1, 6.9, 1.2 Hz, 1H), 7.33 – 7.27 (m, 4H), 2.68 (s, 3H), 2.44 (s, 3H); **$^{13}$C{$^{1}$H} NMR** (101 MHz, $CDCl_3$) δ 157.7, 147.1, 137.5, 137.1, 136.2, 135.9, 129.4, 129.3, 129.2, 128.5, 127.6, 127.1, 126.1, 24.8, 21.4; **HRMS** (ESI-TOF) calcd for $C_{17}H_{16}N^+$ $[M+H]^+$ 234.1277, found 234.1284.

**2-methyl-3-(4-nitrophenyl)quinoline (3d):**

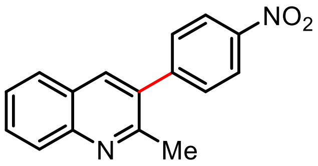


**3d**

Following the general procedure II, product **3d** was isolated in 59% yield as a yellow liquid along with 9% recovered starting material. **3d**: $R_f = 0.3$ (silica gel, 20% EtOAc in hexanes); **$^{1}$H NMR** (400 MHz, $CDCl_3$) δ 8.36 (d, $J$ = 8.7 Hz, 2H), 8.08 (dd, $J$ = 8.6, 1.1 Hz, 1H), 7.99 (s, 1H), 7.83 (dd, $J$ = 8.1, 1.4 Hz, 1H), 7.75 (ddd, $J$ = 8.5, 6.9, 1.5 Hz, 1H), 7.61 (d, $J$ = 8.7 Hz, 2H), 7.55 (ddd, $J$ = 8.1, 6.8, 1.2 Hz, 1H), 2.67 (s, 3H); **$^{13}$C{$^{1}$H} NMR** (101 MHz, $CDCl_3$) δ 156.2, 147.7, 147.5, 146.9, 136.5, 133.6, 130.4, 130.3, 128.7, 127.7, 126.7, 126.6, 123.9, 24.7; **HRMS** (ESI-TOF) calcd for $C_{16}H_{13}N_2O_2^+$ $[M+H]^+$ 265.0972, found 265.0975.

**3-(4-bromophenyl)-2-methylquinoline (3e):**

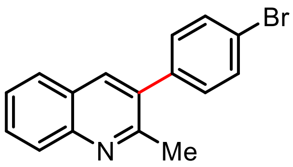


**3e**

Following the general procedure II, product **3e** was isolated in 67% yield as a yellow solid. **3e**: $R_f = 0.5$ (silica gel, 20% EtOAc in hexanes); **$^{1}$H NMR** (400 MHz, $CDCl_3$) δ 8.09 – 8.02 (m, 1H), 7.94 (s, 1H), 7.79 (dd, $J$ = 8.1, 1.4 Hz, 1H), 7.71 (ddd, $J$ = 8.5, 6.9, 1.5 Hz, 1H), 7.65 – 7.58 (m, 2H), 7.52 (ddd, $J$ = 8.1, 6.9, 1.2 Hz, 1H), 7.31 – 7.27 (m, 2H), 2.65 (s, 3H); **$^{13}$C{$^{1}$H} NMR** (101 MHz, $CDCl_3$) δ 157.1, 147.3, 139.0, 136.2, 134.6, 131.8, 131.0, 129.8, 128.6, 127.6, 126.9, 126.4, 122.1, 24.7; **HRMS** (ESI-TOF) calcd for $C_{16}H_{13}BrN^+$ $[M+H]^+$ 298.0226, found 298.0235.

**2-methyl-3-(pyridin-2-yl)quinoline (3f):**

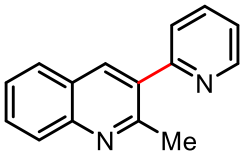


**3f**

Following the general procedure II,* product **3f** was isolated in 53% yield as a yellow solid along with 16% recovered starting material. **3f**: $R_f = 0.3$ (silica gel, 20% EtOAc in hexanes); **$^{1}$H NMR** (400 MHz, $CDCl_3$) δ 8.77 (ddd, $J$ = 4.8, 1.9, 1.0 Hz, 1H), 8.17 (s, 1H), 8.07 (dd, $J$ = 8.5, 1.2 Hz, 1H), 7.86 – 7.80 (m, 2H), 7.72 (ddd, $J$ = 8.4, 6.9, 1.4 Hz, 1H), 7.53 (dt, $J$ = 7.7, 1.1 Hz, 2H), 7.34 (ddd, $J$ = 7.6, 4.9, 1.1 Hz,

1H), 2.78 (s, 3H); **$^{13}$C{$^1$H} NMR** (101 MHz, $CDCl_3$) δ 158.4, 157.3, 149.7, 147.5, 136.9, 136.7, 134.5, 130.0, 128.6, 127.9, 126.8, 126.3, 124.4, 122.5, 24.7; **HRMS** (ESI-TOF) calcd for $C_{15}H_{13}N_2^+$ $[M+H]^+$ 221.1073, found 221.1081.

** 5 equivalents of corresponding halo diazirine was used.*

**2-ethyl-3-(*m*-tolyl)quinoline (3g):**

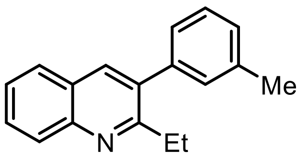


**3g**

Following the general procedure II, product **3g** was isolated in 70% yield as a colorless liquid. **3g**: $R_f = 0.6$ (silica gel, 20% EtOAc in hexanes); **$^1$H NMR** (400 MHz, $CDCl_3$) δ 8.10 (d, *J* = 8.5 Hz, 1H), 7.93 (s, 1H), 7.78 (dd, *J* = 8.1, 1.5 Hz, 1H), 7.69 (ddd, *J* = 8.5, 6.9, 1.5 Hz, 1H), 7.50 (ddd, *J* = 8.1, 6.8, 1.2 Hz, 1H), 7.36 (t, *J* = 7.5 Hz, 1H), 7.26 – 7.15 (m, 3H), 2.97 (q, *J* = 7.5 Hz, 2H), 2.44 (s, 3H), 1.24 (t, *J* = 7.5 Hz, 3H); **$^{13}$C{$^1$H} NMR** (101 MHz, $CDCl_3$) δ 162.3, 147.4, 140.1, 138.2, 136.5, 135.7, 130.1, 129.3, 128.8, 128.4, 128.3, 127.6, 126.8, 126.5, 126.1, 29.9, 21.6, 13.9; **HRMS** (ESI-TOF) calcd for $C_{18}H_{18}N^+$ $[M+H]^+$ 248.1434, found 248.1437.

**3-(4-chlorophenyl)-2-ethylquinoline (3h):**

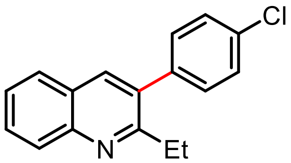


**3h**

Following the general procedure II, product **3h** was isolated in 83% yield as a yellow solid. **3h**: $R_f = 0.5$ (silica gel, 20% EtOAc in hexanes); **$^1$H NMR** (400 MHz, $CDCl_3$δ 8.10 (dd, *J* = 8.5, 1.1 Hz, 1H), 7.95 – 7.87 (m, 1H), 7.79 (dd, *J* = 8.1, 1.4 Hz, 1H), 7.71 (ddd, *J* = 8.4, 6.9, 1.5 Hz, 1H), 7.51 (ddd, *J* = 8.1, 6.9, 1.2 Hz, 1H), 7.48 – 7.42 (m, 2H), 7.37 – 7.30 (m, 2H), 2.95 (q, *J* = 7.5 Hz, 2H), 1.23 (t, *J* = 7.5 Hz, 3H); **$^{13}$C{$^1$H} NMR** (101 MHz, $CDCl_3$) δ 161.9, 147.5, 138.6, 136.6, 134.4, 133.9, 130.7, 129.6, 128.8, 128.7, 127.6, 126.7, 126.3, 29.9, 13.9; **HRMS** (ESI-TOF) calcd for $C_{17}H_{15}ClN^+$ $[M+H]^+$ 268.0888, found 268.0894.

**2-ethyl-3-(3-nitrophenyl)quinoline (3i):**

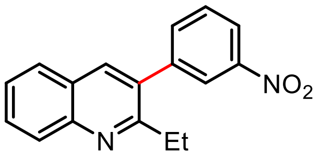


**3i**

Following the general procedure II, product **3i** was isolated in 83% yield as a yellow viscous liquid. **3i**: $R_f = 0.4$ (silica gel, 20% EtOAc in hexanes); **$^1$H NMR** (400 MHz, $CDCl_3$) δ 8.30 (pd, *J* = 2.3, 1.2 Hz, 2H), 8.15 – 8.08 (m, 1H), 7.98 (s, 1H), 7.81 (dd, *J* = 8.1, 1.4 Hz, 1H), 7.75 (ddd, *J* = 8.6, 6.8, 1.4 Hz, 2H), 7.67 (dd, *J* = 8.8, 7.6 Hz, 1H), 7.55 (ddd, *J* = 8.1, 6.8, 1.2 Hz, 1H), 2.94 (q, *J* = 7.5 Hz, 2H), 1.26 (t, *J* = 7.5 Hz, 3H); **$^{13}$C{$^1$H} NMR** (101 MHz, $CDCl_3$) δ 161.1, 148.4, 147.8, 141.8, 136.9, 135.5, 133.0, 130.1, 129.6, 128.9, 127.6, 126.6, 126.5, 124.3, 122.7, 29.9, 13.7; **HRMS** (ESI-TOF) calcd for $C_{17}H_{15}N_2O_2^+$ $[M+H]^+$ 279.1128, found 279.1124.

**2,6-dimethyl-3-phenylquinoline (3j):**

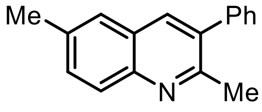

**3j**

Following the general procedure II, product **3j** was isolated in 74% yield colorless oil. **3j**: $R_f$ = 0.5 (silica gel, 20% EtOAc in hexanes); $^1$**H NMR** (400 MHz,) δ 7.96 (d, *J* = 8.4 Hz, 1H), 7.87 (s, 1H), 7.57 – 7.36 (m, 8H), 2.65 (s, 3H), 2.53 (s, 3H). Spectroscopic data are in agreement with those in the literature.[3]

**3-(2-fluorophenyl)-2,6-dimethylquinoline (3k):**

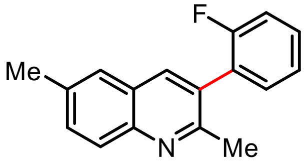

**3k**

Following the general procedure II, product **3k** was isolated in 62% yield as a yellow viscous liquid. **3k**: $R_f$ = 0.5 (silica gel, 20% EtOAc in hexanes); $^1$**H NMR** (400 MHz, $CDCl_3$) δ 7.98 (d, *J* = 9.1 Hz, 1H), 7.90 (s, 1H), 7.58 – 7.53 (m, 2H), 7.47 – 7.39 (m, 1H), 7.32 (td, *J* = 7.5, 2.0 Hz, 1H), 7.29 – 7.23 (m, 1 H), 7.19 (ddd, *J* = 9.6, 8.2, 1.1 Hz, 1H), 2.60 (d, *J* = 1.2 Hz, 3H), 2.53 (s, 3H); $^{13}$**C{$^1$H} NMR** (101 MHz, $CDCl_3$) δ 159.93 (d, *J* = 246.5 Hz), 156.9, 146.0, 136.6, 136.1, 132.1, 131.70 (d, *J* = 2.6 Hz), 130.02 (d, *J* = 8.2 Hz), 129.8, 128.3, 127.6 (d, *J* = 16.4 Hz), 126.7, 126.5, 124.5, 115.92 (d, *J* = 22.1 Hz), 23.9, 21.7; $^{19}$**F NMR** (376 MHz, $CDCl_3$) δ −114.20; **HRMS** (ESI-TOF) calcd for $C_{17}H_{15}FN^+$ $[M+H]^+$ 252.1183, found 252.1189.

**2,4-dimethyl-3-phenylquinoline (3l):**

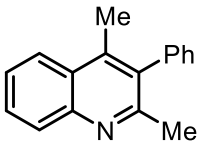

**3l**

Following the general procedure II, product **3l** was isolated in 62% yield as a colorless viscous liquid. **3l**: $R_f$ = 0.3 (silica gel, 10% EtOAc in hexanes); $^1$**H NMR** (400 MHz, $CDCl_3$) δ 8.07 (dt, *J* = 8.4, 1.0 Hz, 1H), 8.00 (ddd, *J* = 8.4, 1.4, 0.6 Hz, 1H), 7.70 (ddd, *J* = 8.3, 6.8, 1.4 Hz, 1H), 7.58 – 7.39 (m, 4H), 7.24 – 7.19 (m, 2H), 2.43 (s, 3H), 2.40 (s, 3H). Spectroscopic data are in agreement with those in the literature.[4]

**3-(3-chlorophenyl)-2,4-dimethylquinoline (3m):**

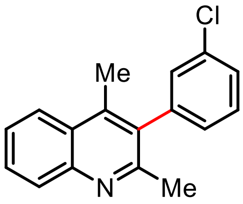

**3m**

Following the general procedure II, product **3m** was isolated in 65% yield as an off white solid. **3m**: $R_f$ = 0.4 (silica gel, 10% EtOAc in hexanes); $^1$**H NMR** (400 MHz, $CDCl_3$) δ 8.08 – 8.03 (m, 1H), 8.00 (ddd, *J* = 8.4, 1.4, 0.6 Hz, 1H), 7.71 (ddd, *J* = 8.4, 6.9, 1.4 Hz, 1H), 7.55 (ddd, *J* = 8.3, 6.9, 1.3 Hz, 1H), 7.44 – 7.41 (m, 2H), 7.23 (td, *J* = 1.7, 0.8 Hz, 1H), 7.11 (ddd, *J* = 6.4, 2.5, 1.6 Hz, 1H), 2.43 (s, 3H), 2.40 (s, 3H); $^{13}$**C{$^1$H} NMR** (101 MHz, $CDCl_3$) δ 157.2, 146.9, 141.5, 134.8, 133.6, 130.2, 129.6, 129.4, 129.3, 127.84, 127.79, 126.7, 126.1, 124.2, 25.5, 16.1; **HRMS** (ESI-TOF) calcd for $C_{17}H_{15}ClN^+$ $[M+H]^+$ 268.0888, found 268.0895.

**2,4-dimethyl-3-(pyridin-4-yl)quinoline (3n):**

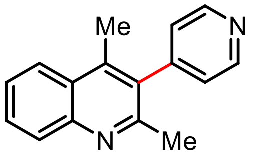

**3n**

Following the general procedure II,* product **3n** was isolated in 27% yield as a yellow solid. **3n**: $R_f = 0.4$ (silica gel, 20% EtOAc in hexanes); **$^1$H NMR** (400 MHz, $CDCl_3$) δ 8.76 (d, *J* = 4.9 Hz, 2H), 8.11 – 8.04 (m, 1H), 8.01 (dt, *J* = 8.4, 0.9 Hz, 1H), 7.73 (ddd, *J* = 8.4, 6.9, 1.4 Hz, 1H), 7.57 (ddd, *J* = 8.3, 6.9, 1.3 Hz, 1H), 7.23 – 7.15 (m, 2H), 2.42 (s, 3H), 2.40 (s, 3H); **$^{13}$C{$^1$H} NMR** (101 MHz, $CDCl_3$) δ 156.1, 150.5, 148.1, 147.0, 141.2, 132.4, 129.6, 129.4, 126.5, 126.3, 124.8, 124.2, 25.4, 16.1; **HRMS** (ESI-TOF) calcd for $C_{16}H_{15}N_2^+$ [M+H]$^+$ 235.1230, found 235.1238.

* *5 equivalents of corresponding halo diazirine was used.*

**2,3-diphenylquinoline (3o):**

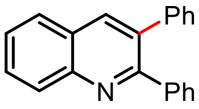

**3o**

Following the general procedure II, product **3o** was isolated in 68% yield as a colorless viscous liquid. **3o**: $R_f = 0.4$ (silica gel, 20% EtOAc in hexanes); **$^1$H NMR** (400 MHz, $CDCl_3$) δ 8.24 – 8.16 (m, 2H), 7.88 (dd, *J* = 8.1, 1.4 Hz, 1H), 7.74 (ddd, *J* = 8.4, 6.8, 1.5 Hz, 1H), 7.57 (ddd, *J* = 8.1, 6.8, 1.2 Hz, 1H), 7.49 – 7.42 (m, 2H), 7.34 – 7.21 (m, 8 H). Spectroscopic data are in agreement with those in the literature.[5]

**3-(3-fluorophenyl)-2-phenylquinoline (3p):**

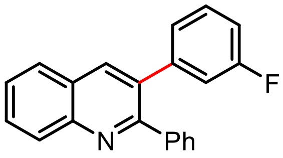

**3p**

Following the general procedure II, product **3p** was isolated in 82% yield as a yellow solid. **3p**: $R_f = 0.5$ (silica gel, 20% EtOAc in hexanes); **$^1$H NMR** (400 MHz, $CDCl_3$) δ 8.21 (d, *J* = 8.5 Hz, 1H), 8.17 (s, 1H), 7.88 (dd, *J* = 8.2, 1.4 Hz, 1H), 7.76 (ddd, *J* = 8.4, 6.8, 1.4 Hz, 1H), 7.58 (td, *J* = 7.5, 6.9, 1.1 Hz, 1H), 7.49 – 7.40 (m, 2H), 7.30 (dd, *J* = 5.2, 1.9 Hz, 3H), 7.26 – 7.20 (m, 1H), 7.04 – 6.87 (m, 3H); **$^{13}$C{$^1$H} NMR** (101 MHz, $CDCl_3$) δ 162.7 (d, *J* = 246.2 Hz), 158.3, 147.6, 142.4 (d, *J* = 7.7 Hz), 140.2, 137.8, 133.4 (d, *J* = 2.1 Hz), 130.1, 129.9, 129.8, 129.6, 128.4, 128.2, 127.7, 127.2, 127.1, 125.8 (d, *J* = 2.9 Hz), 116.8 (d, *J* = 22.0 Hz), 114.3 (d, *J* = 20.9 Hz); **$^{19}$F NMR** (376 MHz, $CDCl_3$) δ -113.07; **HRMS** (ESI-TOF) calcd for $C_{21}H_{15}FN^+$ [M+H]$^+$ 300.1183, found 300.1171.

**3-(3-methoxyphenyl)-2-phenylquinoline (3q):**

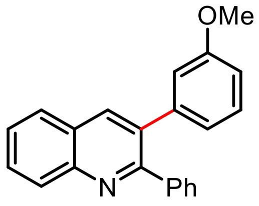


**3q**

Following the general procedure II, product **3q** was isolated in 73% yield as a yellow viscous liquid. **3q**: $R_f = 0.4$ (silica gel, 10% EtOAc in hexanes); **$^1$H NMR** (400 MHz, $CDCl_3$) δ 8.21 (d, *J* = 9.0 Hz, 2H), 7.88 (dd, *J* = 8.1, 1.4 Hz, 1H), 7.74 (ddd, *J* = 8.5, 6.9, 1.5 Hz, 1H), 7.57 (ddd, *J* = 8.0, 6.9, 1.1 Hz, 1H), 7.51 – 7.42 (m, 2H), 7.33 – 7.27 (m, 3H), 7.21 (t, *J* = 7.9 Hz, 1H), 6.90 – 6.81 (m, 2H), 6.76 (dd, *J* = 2.6, 1.6 Hz, 1H), 3.66 (s, 3H); **$^{13}$C{$^1$H} NMR** (101 MHz, $CDCl_3$) δ 159.4, 158.5, 147.4, 141.4, 140.6, 137.6, 134.5, 130.0, 129.8, 129.6, 129.4, 128.1, 128.1, 127.6, 127.3, 126.9, 122.4, 115.3, 113.3, 55.3; **HRMS** (ESI-TOF) calcd for $C_{22}H_{18}NO^+$ $[M+H]^+$ 312.1383, found 312.1390.

**6-methoxy-2-methyl-3-phenylquinoline (3r):**

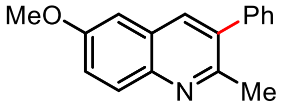


**3r**

Following the general procedure II, product **3r** was isolated in 81% yield as a yellow solid. **3r**: $R_f = 0.4$ (silica gel, 10% EtOAc in hexanes); **$^1$H NMR** (400 MHz, $CDCl_3$) δ 7.96 (d, *J* = 9.2 Hz, 1H), 7.86 (s, 1H), 7.50 – 7.38 (m, 5H), 7.35 (dd, *J* = 9.2, 2.8 Hz, 1H), 7.06 (d, *J* = 2.8 Hz, 1H), 3.92 (s, 3H), 2.63 (s, 3H); **$^{13}$C{$^1$H} NMR** (101 MHz, $CDCl_3$) δ 157.6, 154.8, 143.3, 140.2, 136.1, 135.2, 130.0, 129.3, 128.5, 127.8, 127.7, 122.1, 105.1, 55.7, 24.4; **HRMS** (ESI-TOF) calcd for $C_{17}H_{16}NO^+$ $[M+H]^+$ 250.1226, found 250.1232.

**6-methoxy-2-methyl-3-(pyrazin-2-yl)quinoline (3s):**

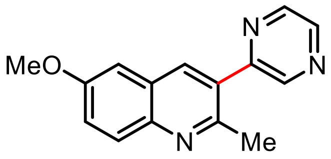


**3s**

Following the general procedure II,* product **3s** was isolated in 48% yield as a pale yellow liquid along with 29% of recovered starting material. **3s**: $R_f = 0.3$ (silica gel, 20% EtOAc in hexanes); **$^1$H NMR** (400 MHz, $CDCl_3$) δ 8.84 (d, *J* = 1.5 Hz, 1H), 8.73 (dd, *J* = 2.5, 1.6 Hz, 1H), 8.63 (d, *J* = 2.5 Hz, 1H), 8.11 (s, 1H), 7.98 (d, *J* = 9.2 Hz, 1H), 7.41 (dd, *J* = 9.2, 2.8 Hz, 1H), 7.11 (d, *J* = 2.9 Hz, 1H), 3.94 (s, 3H), 2.77 (s, 3H); **$^{13}$C{$^1$H} NMR** (101 MHz, $CDCl_3$) δ 164.2, 157.9, 154.4, 145.2, 144.2, 144.0, 143.4, 136.4, 131.1, 130.1, 127.5, 123.3, 105.4, 55.7, 24.3; **HRMS** (ESI-TOF) calcd for $C_{15}H_{14}N_3O^+$ $[M+H]^+$ 252.1131, found 252.1141.

** 5 equivalents of corresponding halo diazirine was used.*

**2-(adamantan-1-yl)-3-(3-bromophenyl)quinoline (3t):**

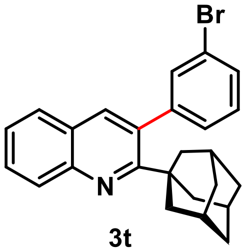

3t

Following the general procedure II, product **3t** was isolated in 63% yield as a white solid. **3t**: $R_f = 0.4$ (silica gel, 20% EtOAc in hexanes); **$^1$H NMR** (400 MHz, $CDCl_3$) δ 8.08 (d, *J* = 8.4 Hz, 1H), 7.70 (dd, *J* = 16.7, 8.1 Hz, 3H), 7.57 – 7.47 (m, 3H), 7.28 (d, *J* = 6.8 Hz, 2 H), 2.13 – 1.92 (m, 8 H), 1.74 – 1.44 (m, 7H); **$^{13}$C{$^1$H} NMR** (101 MHz, $CDCl_3$) δ 165.0, 146.7, 145.1, 139.1, 134.1, 133.2, 130.4, 129.45, 129.36, 129.1, 129.0, 127.0, 126.5, 125.4, 121.7, 43.2, 42.1, 36.9, 29.1; **HRMS** (ESI-TOF) calcd for $C_{25}H_{25}BrN^+$ $[M+H]^+$ 418.1165, found 418.1173.

**7-(4-fluorophenyl)-6-phenyl-[1,3]dioxolo[4,5-*g*]quinoline (3u):**

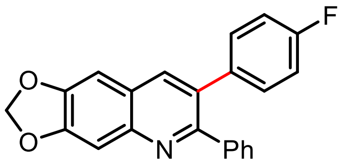

3u

Following the general procedure II, product **3u** was isolated in 72% yield as a yellow solid. **3u**: $R_f = 0.4$ (silica gel, 10% EtOAc in hexanes); **$^1$H NMR** (400 MHz, $CDCl_3$) δ 7.95 (s, 1H), 7.48 (s, 1H), 7.39 (dd, *J* = 6.8, 3.0 Hz, 2H), 7.30 – 7.25 (m, 3 H), 7.21 – 7.12 (m, 2H), 7.10 (s, 1H), 6.97 (t, *J* = 8.7 Hz, 2H), 6.13 (s, 2H); **$^{13}$C{$^1$H} NMR** (101 MHz, $CDCl_3$)) δ 162.2 (d, *J* = 246.6 Hz), 156.1, 151.1, 148.3, 145.8, 140.5, 136.6, 136.3, 131.8, 131.5 (d, *J* = 7.8 Hz), 130.1, 128.2, 128.0, 124.3, 115.3 (d, *J* = 21.4 Hz), 106.0, 102.5, 101.9; **$^{19}$F NMR** (376 MHz, $CDCl_3$) δ −115.38; **HRMS** (ESI-TOF) calcd for $C_{22}H_{15}FNO_2^+$ $[M+H]^+$ 344.1081, found 344.1092.

**5,7-dicyclopropyl-3-(3-methoxyphenyl)-2-methylquinoline (3v):**

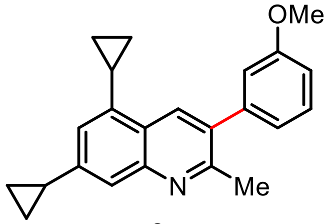

3v

Following the general procedure II, product **3v** was isolated in 64% yield as a pale yellow viscous liquid. **3v**: $R_f = 0.4$ (silica gel, 10% EtOAc in hexanes); **$^1$H NMR** (400 MHz, $CDCl_3$) δ 8.41 (s, 1H), 7.53 (d, *J* = 1.6 Hz, 1H), 7.44 – 7.37 (m, 1H), 7.06 (t, *J* = 1.3 Hz, 1H), 7.01 (dt, *J* = 7.6, 1.2 Hz, 1H), 6.97 (dt, *J* = 6.8, 1.6 Hz, 2H), 3.87 (s, 3H), 2.65 (s, 3H), 2.25 (ddd, *J* = 13.8, 8.5, 5.3 Hz, 1H), 2.03 (tt, *J* = 8.3, 5.1 Hz, 1H), 1.04 (dddd, *J* = 12.7, 8.5, 6.4, 4.4 Hz, 4H), 0.86 (dt, *J* = 6.8, 4.6 Hz, 2H), 0.81 – 0.72 (m, 2H); **$^{13}$C{$^1$H} NMR** (101 MHz, $CDCl_3$) δ 159.6, 156.8, 147.7, 145.7, 142.0, 139.3, 134.2, 133.0, 129.6, 125.3, 123.5, 122.0, 121.4, 115.5, 112.8, 55.5, 24.4, 16.1, 12.7, 10.0, 6.8; **HRMS** (ESI-TOF) calcd for $C_{23}H_{24}NO^+$ $[M+H]^+$ 330.1852, found 330.1862.

**3-(4-fluorophenyl)-2-phenyl-4*H*,6*H*-pyrano[3,4,5-de]quinoline (3w):**

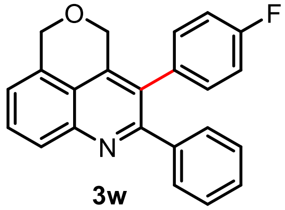

**3w**

Following the general procedure II, product **3w** was isolated in 54% yield as a yellow viscous liquid. **3w**: $R_f$ = 0.5 (silica gel, 20% EtOAc in hexanes); **$^1$H NMR** (400 MHz, $CDCl_3$) δ 8.08 (dd, *J* = 8.6, 1.0 Hz, 1H), 7.70 (dd, *J* = 8.6, 7.0 Hz, 1H), 7.34 – 7.26 (m, 3H), 7.24 (dd, *J* = 5.1, 1.9 Hz, 3H), 7.10 – 6.89 (m, 4H), 5.06 (d, *J* = 1.1 Hz, 2H), 4.86 (s, 2H); **$^{13}$C{$^1$H} NMR** (101 MHz, $CDCl_3$) δ 162.3 (d, *J* = 247.6 Hz), 158.9, 146.7, 140.5, 140.4, 132.9, 132.8, 131.9 (d, *J* = 8.1 Hz), 130.0, 129.6, 128.2, 128.1, 128.0 (2C), 121.1, 120.8, 115.6 (d, *J* = 21.5 Hz), 68.7, 67.5; **$^{19}$F NMR** (376 MHz, $CDCl_3$) δ -114.02; **HRMS** (ESI-TOF) calcd for $C_{23}H_{17}FNO^+$ $[M+H]^+$ 342.1289, found 342.1294.

**methyl (*S*)-2-acetamido-3-(3-(3-nitrophenyl)quinolin-4-yl)propanoate (3x):**

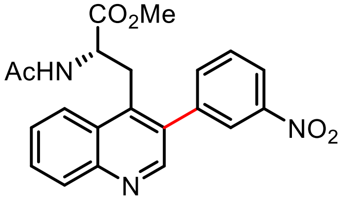

**3x**

Following the general procedure II,* product **3x** was isolated in 41% yield as a pale yellow solid. **3x**: $R_f$ = 0.6 (silica gel, 50% EtOAc in hexanes); **$^1$H NMR** (400 MHz, $CDCl_3$) δ 8.73 (s, 1H), 8.40 – 8.30 (m, 2H), 8.25 (p, *J* = 1.0 Hz, 1H), 8.19 (dd, *J* = 8.4, 1.3 Hz, 1H), 7.80 (ddd, *J* = 8.4, 6.8, 1.4 Hz, 1H), 7.76 – 7.68 (m, 3H), 5.87 (d, *J* = 8.0 Hz, 1H), 4.83 (q, *J* = 7.6 Hz, 1H), 3.63 (dd, *J* = 13.8, 7.5 Hz, 1H), 3.55 – 3.46 (m, 4H), 1.82 (s, 3H); **$^{13}$C{$^1$H} NMR** (101 MHz, $CDCl_3$) δ 171.6, 169.7, 150.6, 148.5, 148.1, 140.0, 139.7, 136.1, 133.2, 130.7, 130.1, 130.0, 128.0, 127.0, 124.8, 124.2, 123.2, 52.8, 52.6, 31.8, 23.1; **HRMS** (ESI-TOF) calcd for $C_{21}H_{20}N_3O_5^+$ $[M+H]^+$ 394.1397, found 394.1405.

* *Reaction was continued for 48 h.*

**1$^3$-phenyl-1(2,4)-quinolinacycloundecaphane (3y):**

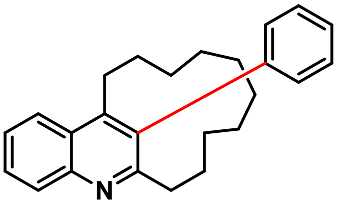

**3y**

Following the general procedure II, product **3y** was isolated in 52% yield as a colorless viscous liquid. **3y**: $R_f$ = 0.6 (silica gel, 20% EtOAc in hexanes); **$^1$H NMR** (400 MHz, $CDCl_3$) δ 8.17 – 8.02 (m, 2H), 7.84 – 7.76 (m, 1H), 7.69 (ddd, *J* = 8.3, 6.8, 1.4 Hz, 1H), 7.61 – 7.49 (m, 2H), 7.45 – 7.34 (m, 2H), 6.88 (dt, *J* = 7.2, 1.8 Hz, 1H), 3.24 (dt, *J* = 13.7, 4.2 Hz, 1H), 2.90 – 2.80 (m, 1H), 2.78 – 2.59 (m, 2H), 2.04 – 1.93 (m, 1H), 1.89 (dq, *J* = 10.8, 6.3, 4.7 Hz, 2H), 1.83 – 1.69 (m, 1H), 1.65 (s, 1H), 1.55 – 1.42 (m, 1H), 1.32 (dddd, *J* = 20.2, 17.1, 7.9, 5.7 Hz, 2H), 1.23 – 1.04 (m, 2H), 1.04 – 0.83 (m, 2H), 0.60 (ttd, *J* = 24.1, 11.7, 11.0, 5.5 Hz, 2H), 0.25 – -0.03 (m, 2H); **$^{13}$C{$^1$H} NMR** (101 MHz, $CDCl_3$) δ 161.9, 139.6, 135.8, 131.1, 131.1, 129.6, 128.9, 128.7, 127.9, 127.3, 126.3, 125.6, 124.8, 36.7, 29.8, 28.2, 28.1, 27.9, 26.9, 26.9, 26.3, 26.2, 26.2; **HRMS** (ESI-TOF) calcd for $C_{25}H_{30}N^+$ $[M+H]^+$ 344.2373, found 344.2381.

**1³-(4-fluorophenyl)-1(2,4)-quinolinacycloundecaphane (3z):**

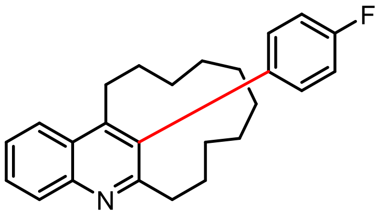

**3z**

Following the general procedure II, product **3z** was isolated in 64% yield as an off white solid. **3z**: $R_f = 0.5$ (silica gel, 20% EtOAc in hexanes); **$^1$H NMR** (400 MHz, $CDCl_3$) δ 8.14 – 8.02 (m, 2H), 7.75 (ddd, $J$ = 8.1, 5.3, 2.4 Hz, 1H), 7.70 (ddd, $J$ = 8.3, 6.8, 1.3 Hz, 1H), 7.53 (ddd, $J$ = 8.3, 6.8, 1.3 Hz, 1H), 7.31 – 7.23 (m, 1H), 7.07 (td, $J$ = 8.5, 2.8 Hz, 1H), 6.85 (ddd, $J$ = 8.1, 5.4, 2.3 Hz, 1H), 3.24 (dt, $J$ = 13.8, 4.2 Hz, 1H), 2.84 (dt, $J$ = 13.3, 5.1 Hz, 1H), 2.77 – 2.56 (m, 2H), 1.98 (dp, $J$ = 14.8, 4.5 Hz, 1H), 1.93 – 1.82 (m, 2H), 1.75 (ddq, $J$ = 14.9, 11.4, 4.6 Hz, 1H), 1.48 (dtd, $J$ = 13.9, 10.8, 6.7 Hz, 1H), 1.37 – 1.19 (m, 4H), 1.11 (dddd, $J$ = 26.1, 12.3, 6.4, 3.2 Hz, 1H), 0.97 (ttd, $J$ = 16.8, 10.2, 8.8, 5.5 Hz, 2H), 0.59 (dddd, $J$ = 27.5, 13.5, 10.7, 5.6 Hz, 2H), 0.21 – -0.05 (m, 2 H); **$^{13}$C{$^1$H} NMR** (101 MHz, $CDCl_3$) δ 162.2 (d, $J$ = 247.1 Hz), 162.0, 147.2, 145.9, 135.5, 134.8, 133.0 (d, $J$ = 7.6 Hz), 132.6 (d, $J$ = 7.9 Hz), 129.7, 129.1, 126.3, 125.9, 124.9, 115.8 (d, $J$ = 21.1 Hz), 115.0 (d, $J$ = 21.4 Hz), 36.8, 29.9, 28.3 (2C), 28.0, 27.0, 26.9, 26.4 (2C), 26.2; **$^{19}$F NMR** (376 MHz, $CDCl_3$) δ −115.1; **HRMS** (ESI-TOF) calcd for $C_{25}H_{29}FN^+$ $[M+H]^+$ 362.2279, found 362.2283.

**3-(3-chlorophenyl)-2,6-dimethylpyridine (5a):**

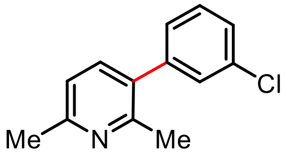

**5a**

Following the general procedure II, product **5a** was isolated in 73% yield as a viscous brown liquid. **5a**: $R_f = 0.4$ (silica gel, 10% EtOAc in hexanes); **$^1$H NMR** (400 MHz, $CDCl_3$) δ 7.39 (d, $J$ = 7.7 Hz, 1H), 7.35 (dd, $J$ = 4.8, 1.9 Hz, 2H), 7.30 (q, $J$ = 1.5 Hz, 1H), 7.19 (ddd, $J$ = 6.2, 2.9, 1.7 Hz, 1H), 7.05 (d, $J$ = 7.8 Hz, 1H), 2.57 (s, 3H), 2.47 (s, 3H); **$^{13}$C{$^1$H} NMR** (101 MHz, $CDCl_3$) δ 157.3, 155.0, 142.1, 137.6, 134.4, 132.8, 129.8, 129.3, 127.6, 127.5, 120.7, 24.4, 23.5; **HRMS** (ESI-TOF) calcd for $C_{13}H_{13}ClN^+$ $[M+H]^+$ 218.0731, found 218.0731.

**2-methyl-3-phenyl-5,6-dihydrobenzo[*h*]quinoline (5b):**

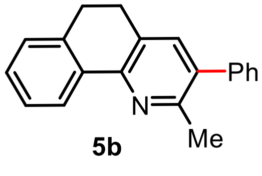

**5b**

Following the general procedure II, product **5b** was isolated in 69% yield as a yellow iol. **5b**: $R_f = 0.5$ (silica gel, 20% EtOAc in hexanes); **$^1$H NMR** (400 MHz, $CDCl_3$) δ 8.38 (dd, $J$ = 7.7, 1.5 Hz, 1H), 7.45 (ddt, $J$ = 7.8, 5.0, 1.3 Hz, 2H), 7.37 (dtd, $J$ = 7.9, 4.5, 2.0 Hz, 5H), 7.31 (td, $J$ = 7.4, 1.5 Hz, 1H), 7.24 (dd, $J$ = 7.4, 1.4 Hz, 1H), 2.95 (q, $J$ = 2.4 Hz, 4H), 2.57 (s, 3H). Spectroscopic data are in agreement with those in the literature.[6]

**3-(4-fluorophenyl)-2-methyl-5,6-diphenylpyridine (5c):**

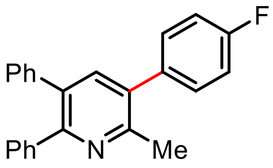


**5c**

Following the general procedure II, product **5c** was isolated in 63% yield as a off white solid. **5c**: $R_f$ = 0.6 (silica gel, 20% EtOAc in hexanes); **$^1$H NMR** (400 MHz, $CDCl_3$) δ 7.49 (s, 1H), 7.38 – 7.28 (m, 4H), 7.18 (td, *J* = 3.5, 1.8 Hz, 6H), 7.14 – 6.99 (m, 4H), 2.53 (s, 3H); **$^{13}$C{$^1$H} NMR** (101 MHz, $CDCl_3$) δ 162.4 (d, *J* = 247.0 Hz), 155.5, 154.5, 140.1, 139.9, 139.8, 135.7 (d, *J* = 3.5 Hz), 134.6, 133.6, 130.9 (d, *J* = 8.1 Hz), 130.1, 129.7, 128.4, 128.1, 127.9, 127.2, 115.6 (d, *J* = 21.5 Hz), 23.4; **$^{19}$F NMR** (376 MHz, $CDCl_3$) δ -114.7; **HRMS** (ESI-TOF) calcd for $C_{24}H_{19}FN^+$ $[M+H]^+$ 340.1496, found 340.1502.

**ethyl 2,6-dimethyl-5-phenylnicotinate (5d):**

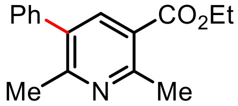


**5d**

Following the general procedure II, product **5d** was isolated in 65% yield as a yellow solid. **5d**: $R_f$ = 0.3 (silica gel, 20% EtOAc in hexanes); **$^1$H NMR** (400 MHz, $CDCl_3$) δ 8.05 (s, 1H), 7.48 – 7.42 (m, 2H), 7.42 – 7.37 (m, 1H), 7.35 – 7.30 (m, 2H), 4.36 (q, *J* = 7.1 Hz, 2H), 2.86 (s, 3H), 2.52 (s, 3H), 1.38 (t, *J* = 7.1 Hz, 3H); **$^{13}$C{$^1$H} NMR** (101 MHz, $CDCl_3$) δ 166.8, 158.7, 158.1, 139.6, 139.2, 134.4, 129.2, 128.6, 127.8, 123.1, 61.3, 24.6, 23.7, 14.4; **HRMS** (ESI-TOF) calcd for $C_{16}H_{18}NO_2^+$ $[M+H]^+$ 256.1332, found 256.1339.

**3-(4-fluorophenyl)-6-(3-methoxyphenyl)-2-methyl-5-pentylpyridine (5e):**

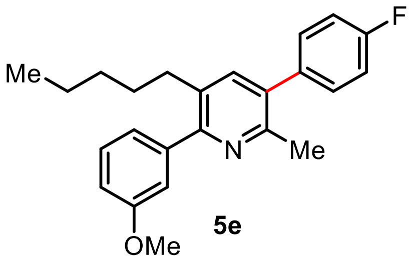


**5e**

Following the general procedure II, product **5e** was isolated in 46% yield as a white solid. **5e**: $R_f$ = 0.4 (silica gel, 10% EtOAc in hexanes); **$^1$H NMR** (400 MHz, $CDCl_3$) δ 7.41 (s, 1H), 7.38 – 7.32 (m, 3H), 7.19 – 7.12 (m, 2H), 7.08 – 7.02 (m, 2H), 6.94 (ddd, *J* = 8.3, 2.6, 1.0 Hz, 1H), 3.85 (s, 3H), 2.67 – 2.58 (m, 2H), 2.51 (s, 3H), 1.52 (p, *J* = 7.1 Hz, 2H), 1.23 (dtd, *J* = 7.2, 4.8, 4.1, 2.1 Hz, 4H), 0.87 – 0.78 (m, 3H); **$^{13}$C{$^1$H} NMR** (101 MHz, $CDCl_3$) δ 162.4 (d, *J* = 246.6 Hz), 159.6, 157.2, 152.6, 138.7, 134.7, 133.1, 130.9, 130.8, 129.4, 121.5, 115.6, 115.4, 114.5, 113.9, 55.5, 32.0, 31.7, 30.9, 23.2, 22.5, 14.1. $^{19}$F NMR (376 MHz, $CDCl_3$) δ -115.08; **HRMS** (ESI-TOF) calcd for $C_{24}H_{27}FNO^+$ $[M+H]^+$ 364.2071, found 364.2080.

**2-methyl-3,6-diphenylpyridine (5f) and 6-methyl-2,3-diphenylpyridine (5f')**:

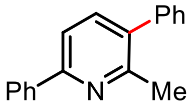


**5f**

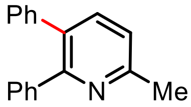


**5f'**

Following the general procedure II, products **5f** and **5f'** were obtained in 90% yield in 1.9:1 ratio as a pale yellow solid and yellow viscous liquid respectively. **5f**: $R_f = 0.35$ (silica gel, 10% EtOAc in hexanes); **$^1$H NMR** (400 MHz, $CDCl_3$) δ 8.08 – 8.01 (m, 2H), 7.66 – 7.57 (m, 2H), 7.52 – 7.43 (m, 4H), 7.42 – 7.37 (m, 4H), 2.60 (s, 3H). Spectroscopic data are in agreement with those in the literature.[7]

**5f'**: $R_f = 0.3$ (silica gel, 10% EtOAc in hexanes); $^1$H NMR (400 MHz, $CDCl_3$) δ 7.62 (d, *J* = 7.8 Hz, 1H), 7.34 (dd, *J* = 6.8, 3.0 Hz, 2H), 7.25 – 7.10 (m, 9H), 2.66 (s, 3H). Spectroscopic data are in agreement with those in the literature.[8]

**6-(4-methoxyphenyl)-2-methyl-3-phenylpyridine (5g) and 2-(4-methoxyphenyl)-6-methyl-3-phenylpyridine (5g')**:

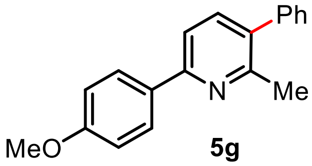


**5g**

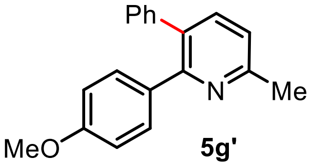


**5g'**

Following the general procedure II, products **5g** and **5g'** were obtained in 79% yield in 1.5:1 ratio as a white solid and pale yellow viscous liquid respectively. **5g**: $R_f = 0.4$ (silica gel, 10% EtOAc in hexanes); **$^1$H NMR** (400 MHz, $CDCl_3$) δ 8.05 – 7.96 (m, 2H), 7.56 (s, 2H), 7.50 – 7.41 (m, 2H), 7.41 – 7.34 (m, 3H), 7.06 – 6.96 (m, 2H), 3.87 (s, 3H), 2.58 (s, 3H); **$^{13}$C{$^1$H} NMR** (101 MHz, $CDCl_3$) δ 160.5, 155.6, 155.4, 140.3, 138.0, 134.8, 132.2, 129.2, 128.5, 128.3, 127.4, 117.2, 114.2, 55.5, 23.9; **HRMS** (ESI-TOF) calcd for $C_{19}H_{18}NO^+$ $[M+H]^+$ 276.1383, found 276.1382.

**5g'**: $R_f = 0.35$ (silica gel, 10% EtOAc in hexanes); **$^1$H NMR** (400 MHz, $CDCl_3$) δ 7.58 (d, *J* = 7.8 Hz, 1H), 7.27 (dtd, *J* = 8.3, 6.1, 2.7 Hz, 5H), 7.21 – 7.11 (m, 3H), 6.76 (d, *J* = 8.7 Hz, 2H), 3.77 (s, 3H), 2.65 (s, 3H); **$^{13}$C{$^1$H} NMR** (101 MHz, $CDCl_3$) δ 159.3, 157.1, 156.2, 140.5, 139.0, 133.1, 132.9, 131.4, 129.7, 128.4, 126.9, 121.4, 113.5, 55.4, 24.6; **HRMS** (ESI-TOF) calcd for $C_{19}H_{18}NO^+$ $[M+H]^+$ 276.1383, found 276.1378.

**4-(6-methyl-5-phenylpyridin-2-yl)benzonitrile (5h) and 4-(6-methyl-3-phenylpyridin-2-yl)benzonitrile (5h')**:

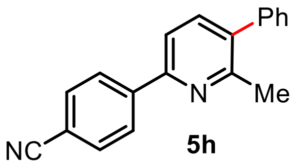


**5h**

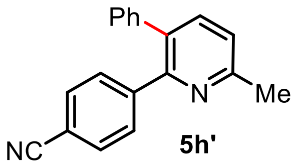


**5h'**

Following the general procedure II, products **5h** and **5h'** were obtained in 73% yield in 2.1:1 ratio as a colorless solid and white solid respectively. **5h:** $R_f = 0.4$ (silica gel, 20% EtOAc in hexanes); **$^1$H NMR** (400 MHz, $CDCl_3$) δ 8.17 – 8.04 (m, 2H), 7.79 – 7.66 (m, 2H), 7.60 (d, *J* = 8.1 Hz, 1H), 7.57 (d, *J* = 8.0

Hz, 1H), 7.43 – 7.27 (m, 5H), 2.53 (s, 3H); **$^{13}$C{$^1$H} NMR** (101 MHz, $CDCl_3$) δ 156.5, 153.4, 143.7, 139.6, 138.3, 136.9, 132.7, 129.1, 128.7, 127.8, 127.5, 119.1, 118.4, 112.3, 23.9; **HRMS** (ESI-TOF) calcd for $C_{19}H_{15}N_2^+$ [M+H]$^+$ 271.1230, found 271.1234.

**5h':** $R_f$ = 0.35 (silica gel, 20% EtOAc in hexanes); **$^1$H NMR** (400 MHz, $CDCl_3$) δ 7.65 (d, *J* = 7.9 Hz, 1H), 7.52 (d, *J* = 8.3 Hz, 2H), 7.46 (d, *J* = 8.3 Hz, 2H), 7.31 – 7.26 (m, 3H), 7.11 (dt, *J* = 5.4, 2.0 Hz, 2H), 2.66 (s, 3H); **$^{13}$C{$^1$H} NMR** (101 MHz, $CDCl_3$) δ 157.8, 154.4, 145.2, 139.3, 139.2, 133.7, 131.9, 130.8, 129.7, 128.7, 127.6, 122.8, 119.1, 111.4, 24.5; **HRMS** (ESI-TOF) calcd for $C_{19}H_{15}N_2^+$ [M+H]$^+$ 271.1230, found 271.1241.

**3-(3-methoxyphenyl)-2-methyl-6-phenylpyridine (5i) and 3-(3-methoxyphenyl)-6-methyl-2-phenylpyridine (5i'):**

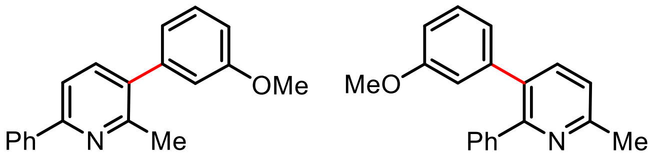

**5i** **5i'**

Following the general procedure II, products **5i** and **5i'** were obtained in 61% yield in 2:1 ratio as a white solid and colorless viscous liquid respectively. **5i:** $R_f$ = 0.6 (silica gel, 20% EtOAc in hexanes); **$^1$H NMR** (400 MHz, $CDCl_3$) δ 8.10 – 7.97 (m, 2H), 7.73 – 7.58 (m, 2H), 7.52 – 7.44 (m, 2H), 7.44 – 7.33 (m, 2H), 6.99 – 6.85 (m, 3H), 3.86 (s, 3H), 2.60 (s, 3H); **$^{13}$C{$^1$H} NMR** (101 MHz, $CDCl_3$) δ 159.6, 155.9, 155.8, 141.5, 139.6, 138.0, 135.4, 129.6, 128.9, 128.9, 127.1, 121.7, 117.9, 115.0, 112.9, 55.5, 23.9; **HRMS** (ESI-TOF) calcd for $C_{19}H_{18}NO^+$ [M+H]$^+$ 276.1383, found 276.1389.

**5i':** $R_f$ = 0.55 (silica gel, 20% EtOAc in hexanes); **$^1$H NMR** (400 MHz, $CDCl_3$) δ 7.63 (d, *J* = 7.8 Hz, 1H), 7.39 – 7.32 (m, 2H), 7.25 – 7.22 (m, 3H), 7.20 – 7.13 (m, 2H), 6.79 – 6.73 (m, 2H), 6.66 (dd, *J* = 2.6, 1.6 Hz, 1H), 3.63 (s, 3H), 2.66 (s, 3H); **$^{13}$C{$^1$H} NMR** (101 MHz, $CDCl_3$) δ 159.4, 157.3, 156.7, 141.5, 140.6, 138.7, 133.1, 130.0, 129.3, 128.1, 127.7, 122.2, 121.8, 115.2, 113.0, 55.3, 24.6; **HRMS** (ESI-TOF) calcd for $C_{19}H_{18}NO^+$ [M+H]$^+$ 276.1383, found 276.1391.

**3-(3-chlorophenyl)-2-methyl-6-phenylpyridine (5j) and 3-(3-chlorophenyl)-6-methyl-2-phenylpyridine (5j'):**

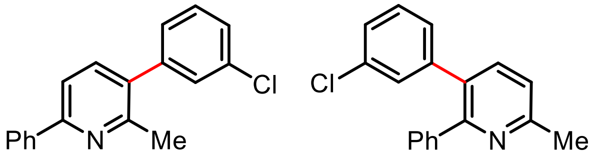

**5j** **5j'**

Following the general procedure II, products **5j** and **5j'** were obtained in 85% yield in 1.3:1 ratio as a colorless viscous liquid and white solid respectively. **5j:** $R_f$ = 0.5 (silica gel, 20% EtOAc in hexanes); **$^1$H NMR** (400 MHz, $CDCl_3$) δ 8.08 – 7.99 (m, 2H), 7.62 (d, *J* = 8.0 Hz, 1H), 7.57 (d, *J* = 8.0 Hz, 1H), 7.53 – 7.46 (m, 2H), 7.45 – 7.40 (m, 1H), 7.40 – 7.34 (m, 3H), 7.28 – 7.24 (m, 1H), 2.59 (s, 3H); **$^{13}$C{$^1$H} NMR**

(101 MHz, $CDCl_3$) δ 156.3, 155.7, 141.9, 139.4, 138.0, 134.4, 134.1, 129.8, 129.3, 129.1, 128.9, 127.7, 127.5, 127.1, 118.0, 23.8; **HRMS** (ESI-TOF) calcd for $C_{18}H_{15}ClN^+$ $[M+H]^+$ 280.0888, found 280.0897.

**5j':** $R_f$ = 0.45 (silica gel, 20% EtOAc in hexanes); **$^1$H NMR** (400 MHz, $CDCl_3$) δ 7.60 (d, *J* = 7.8 Hz, 1H), 7.33 (dd, *J* = 6.7, 3.1 Hz, 2H), 7.26 – 7.18 (m, 6H), 7.17 – 7.10 (m, 1H), 6.96 (dt, *J* = 7.6, 1.4 Hz, 1H), 2.67 (s, 3H); **$^{13}$C{$^1$H} NMR** (101 MHz, $CDCl_3$) δ 157.8, 156.7, 142.1, 140.1, 138.7, 134.2, 131.9, 130.0, 129.6, 129.5, 128.2, 128.1, 128.0, 127.2, 121.9, 24.6; **HRMS** (ESI-TOF) calcd for $C_{18}H_{15}ClN^+$ $[M+H]^+$ 280.0888, found 280.0883.

**2-methyl-3-(3-nitrophenyl)-6-phenylpyridine (5k) and 6-methyl-3-(3-nitrophenyl)-2-phenylpyridine (5k'):**

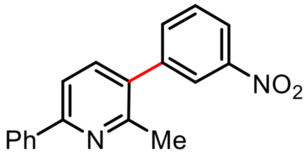

**5k**

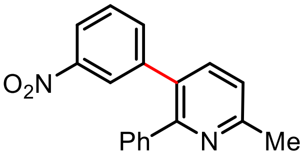

**5k'**

Following the general procedure II, products **5k** and **5k'** were obtained in 68% yield in 1.2:1 ratio as a pale yellow solid and yellow viscous liquid respectively. **5k:** $R_f$ = 0.3 (silica gel, 20% EtOAc in hexanes); **$^1$H NMR** (400 MHz, $CDCl_3$) δ 8.27 (pd, *J* = 2.3, 1.2 Hz, 2H), 8.10 – 7.99 (m, 2H), 7.73 (dt, *J* = 7.7, 1.4 Hz, 1H), 7.69 – 7.64 (m, 2H), 7.64 – 7.60 (m, 1H), 7.54 – 7.47 (m, 2H), 7.47 – 7.40 (m, 1H), 2.60 (s, 3H); **$^{13}$C{$^1$H} NMR** (101 MHz, $CDCl_3$) δ 156.9, 155.6, 148.5, 141.8, 139.1, 138.1, 135.3, 133.0, 129.6, 129.3, 129.0, 127.2, 124.2, 122.6, 118.2, 23.8; **HRMS** (ESI-TOF) calcd for $C_{18}H_{15}N_2O_2^+$ $[M+H]^+$ 291.1128, found 291.1137.

**5k':** $R_f$ = 0.25 (silica gel, 20% EtOAc in hexanes); **$^1$H NMR** (400 MHz, $CDCl_3$) δ 8.10 (dd, *J* = 6.1, 2.1 Hz, 2H), 7.66 (d, *J* = 7.8 Hz, 1H), 7.42 – 7.36 (m, 2H), 7.33 – 7.28 (m, 2H), 7.27 – 7.22 (m, 4H), 2.69 (s, 3H); **$^{13}$C{$^1$H} NMR** (101 MHz, $CDCl_3$) δ 158.6, 156.9, 148.4, 142.0, 139.6, 138.7, 136.0, 130.9, 130.0, 129.2, 128.4, 128.3, 124.4, 122.2, 122.1, 24.6; **HRMS** (ESI-TOF) calcd for $C_{18}H_{15}N_2O_2^+$ $[M+H]^+$ 291.1128, found 291.1141.

**2-methyl-5-phenylpyridine (5l) and 2-methyl-3-phenylpyridine (5l'):**

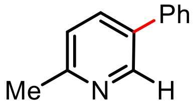

**5l**

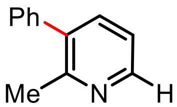

**5l'**

Following the general procedure II, products **5l** and **5l'** were obtained in 34% yield in 2.4:1 ratio respectively as a pale-yellow liquid. **5l**: $R_f$ = 0.4 (silica gel, 20% EtOAc in hexanes); **$^1$H NMR** (400 MHz, $CDCl_3$) δ 8.73 (dd, *J* = 2.5, 0.8 Hz, 1H), 7.75 (dd, *J* = 8.0, 2.4 Hz, 1H), 7.55 (d, J = 7.6 Hz, 2H), 7.45 7.20 (m, 4H), 2.59 (s, 3H). Spectroscopic data are in agreement with those in the literature.[9]

**5l'**: $R_f$ = 0.35 (silica gel, 20% EtOAc in hexanes); **$^1$H NMR** (400 MHz, $CDCl_3$) δ 8.49 (s, dd, *J* = 5.0, 1.8 Hz, 1H), 7.49-7.15 (m, 7H), 2.50 (3H, s, $CH_3$). Spectroscopic data are in agreement with those in the literature.[10]

**2-ethyl-3,6-diphenylpyridine (5m) and 6-ethyl-2,3-diphenylpyridine (5m'):**

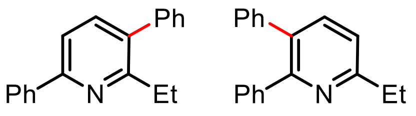


Following the general procedure II, products **5m** and **5m'** were obtained in 64% yield in 1.1:1 ratio respectively as a white solid. **5m:** $R_f$ = 0.5 (silica gel, 20% EtOAc in hexanes); **$^1$H NMR** (400 MHz, $CDCl_3$) δ 8.15 – 8.02 (m, 2H), 7.62 (d, *J* = 8.0 Hz, 1H), 7.56 (d, *J* = 8.0 Hz, 1H), 7.52 – 7.33 (m, 8H), 2.86 (q, *J* = 7.5 Hz, 2H), 1.29 (t, *J* = 7.5 Hz, 3H); **$^{13}$C{$^1$H} NMR** (101 MHz, $CDCl_3$) δ 160.4, 155.7, 140.2, 139.7, 138.2, 135.1, 129.3, 128.8, 128.8, 128.5, 127.5, 127.0, 117.5, 29.0, 14.0; **HRMS** (ESI-TOF) calcd for $C_{19}H_{18}N^+$ $[M+H]^+$ 260.1434, found 260.1440. **5m':** $R_f$ = 0.45 (silica gel, 20% EtOAc in hexanes); **$^1$H NMR** (400 MHz, Chloroform-*d*) δ 7.64 (d, *J* = 7.9 Hz, 1H), 7.35 (ddd, *J* = 6.6, 3.1, 1.5 Hz, 2H), 7.26 – 7.21 (m, 7H), 7.17 – 7.12 (m, 2H), 2.94 (q, *J* = 7.6 Hz, 2H), 1.39 (t, *J* = 7.6 Hz, 3H); **$^{13}$C{$^1$H} NMR** (101 MHz, $CDCl_3$) δ 162.4, 156.5, 140.7, 140.3, 139.0, 133.4, 130.1, 129.7, 128.3, 128.0, 127.7, 127.0, 120.4, 31.4, 14.2; **HRMS** (ESI-TOF) calcd for $C_{19}H_{18}N^+$ $[M+H]^+$ 260.1434, found 260.1439.

**2-isopropyl-3,6-diphenylpyridine (5n) and 6-isopropyl-2,3-diphenylpyridine (5n'):**

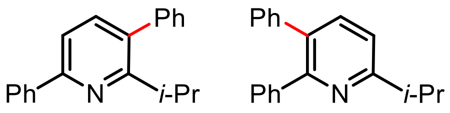


Following the general procedure II, products **5n** and **5n'** were obtained in 60% yield in 1:2.6 ratio respectively as a white solid. **5n:** $R_f$ = 0.6 (silica gel, 20% EtOAc in hexanes); **$^1$H NMR** (400 MHz, $CDCl_3$) δ 8.39 – 8.08 (m, 2H), 7.61 (d, *J* = 8.0 Hz, 1H), 7.53 (d, *J* = 8.0 Hz, 1H), 7.51 – 7.38 (m, 6H), 7.37 – 7.31 (m, 2H), 3.26 (p, *J* = 6.7 Hz, 1H), 1.29 (d, *J* = 6.7 Hz, 6H). Spectroscopic data are in agreement with those in the literature.[11]

**5n':** $R_f$ = 0.55 (silica gel, 20% EtOAc in hexanes); **$^1$H NMR** (400 MHz, $CDCl_3$) δ 7.64 (d, *J* = 7.9 Hz, 1H), 7.43 – 7.33 (m, 2H), 7.23 (td, *J* = 5.2, 4.4, 2.2 Hz, 7H), 7.16 (dd, *J* = 7.3, 2.3 Hz, 2H), 3.18 (h, *J* = 7.0 Hz, 1H), 1.38 (d, *J* = 6.9 Hz, 6H); **$^{13}$C{$^1$H} NMR** (101 MHz, $CDCl_3$) δ 166.2, 156.0, 140.7, 140.5, 139.0, 133.4, 130.2, 129.7, 128.3, 127.9, 127.7, 127.0, 118.7, 36.4, 22.9; **HRMS** (ESI-TOF) calcd for $C_{20}H_{20}N^+$ $[M+H]^+$ 274.1590, found 274.1594.

**3-(4-fluorophenyl)-2-isobutyl-6-(thiophen-2-yl)pyridine (5o) and 3-(4-fluorophenyl)-6-isobutyl-2-(thiophen-2-yl)pyridine (5o'):**

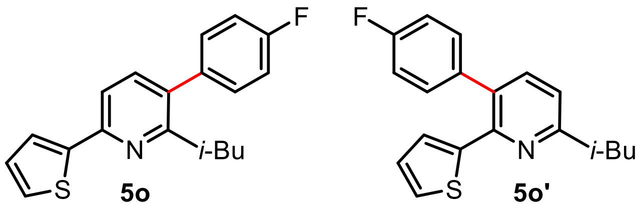

Following the general procedure II, products **5o** and **5o'** were obtained in 59% yield in 1:1 ratio as a yellow solid and pale yellow solid respectively. **5o:** $R_f$ = 0.45 (silica gel, 20% EtOAc in hexanes); **$^1$H NMR** (400 MHz, $CDCl_3$) δ 7.60 (dd, *J* = 3.7, 1.1 Hz, 1H), 7.51 (d, *J* = 8.0 Hz, 1H), 7.46 (d, *J* = 8.0 Hz, 1H), 7.38 (dd, *J* = 5.1, 1.2 Hz, 1H), 7.30 – 7.25 (m, 2H), 7.16 – 7.09 (m, 3H), 2.65 (d, *J* = 7.1 Hz, 2H), 2.23 (dp, *J* = 13.7, 6.8 Hz, 1H), 0.83 (d, *J* = 6.6 Hz, 6H); **$^{13}$C{$^1$H} NMR** (101 MHz, $CDCl_3$) δ 162.3 (d, *J* = 246.6 Hz), 158.8, 151.0, 145.6, 138.1, 136.2 (d, *J* = 3.4 Hz), 134.7, 131.1 (d, *J* = 7.9 Hz), 128.1, 127.5, 124.3, 115.7, 115.4 (d, *J* = 21.4 Hz), 43.9, 28.6, 22.6; **$^{19}$F NMR** (376 MHz, $CDCl_3$) δ -115.17; **HRMS** (ESI-TOF) calcd for $C_{19}H_{19}FNS^+$ $[M+H]^+$ 312.1217, found 312.1225.

**5o':** $R_f$ = 0.4 (silica gel, 20% EtOAc in hexanes); **$^1$H NMR** (400 MHz, $CDCl_3$) δ 7.42 (d, *J* = 7.7 Hz, 1H), 7.32 – 7.25 (m, 3H), 7.15 – 7.07 (m, 2H), 7.02 (d, *J* = 7.8 Hz, 1H), 6.81 (dd, *J* = 5.1, 3.7 Hz, 1H), 6.57 (dd, *J* = 3.8, 1.1 Hz, 1H), 2.71 (d, *J* = 7.2 Hz, 2H), 2.23 (dp, *J* = 13.6, 6.8 Hz, 1H), 1.01 (d, *J* = 6.6 Hz, 6H); **$^{13}$C{$^1$H} NMR** (101 MHz, $CDCl_3$) δ 162.6 (d, *J* = 246.9 Hz), 160.5, 149.5, 145.1, 138.9, 136.4 (d, *J* = 3.4 Hz), 131.2 (d, *J* = 8.1 Hz), 130.9, 127.6 (d, *J* = 23.9 Hz), 127.5, 121.4, 115.9, 115.7, 47.2, 28.9, 22.7; **$^{19}$F NMR** (376 MHz, $CDCl_3$) δ -114.6; **HRMS** (ESI-TOF) calcd for $C_{19}H_{19}FNS^+$ $[M+H]^+$ 312.1217, found 312.1225.

***N*-(2-(5-(4-fluorophenyl)-6-methylpyridin-2-yl)-3-methylbutyl)benzamide (5p) and tert-butyl benzoyl(2-(3-(4-fluorophenyl)-6-methylpyridin-2-yl)-3-methylbutyl)carbamate (5p'):**

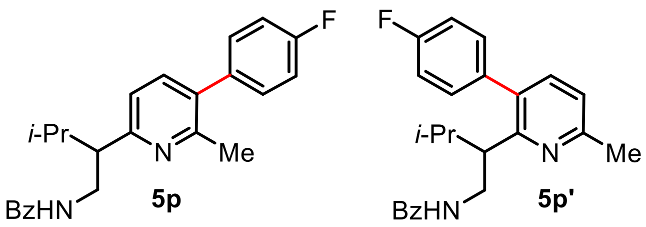

Following the general procedure II, products **5p** and **5p'** were obtained in 59% yield in 1:3 ratio respectively as a white solid. **5p:** $R_f$ = 0.25 (silica gel, 40% EtOAc in hexanes); **$^1$H NMR** (400 MHz, $CDCl_3$) δ 8.25 (d, *J* = 7.0 Hz, 1H), 7.90 – 7.70 (m, 2H), 7.42 – 7.32 (m, 4H), 7.25 – 7.18 (m, 2H), 7.10 – 7.02 (m, 2H), 6.98 (d, *J* = 7.7 Hz, 1H), 4.14 (ddd, *J* = 13.3, 7.2, 5.8 Hz, 1H), 3.55 – 3.30 (m, 1H), 2.59 (ddd, *J* = 9.1, 5.6, 2.9 Hz, 1H), 2.45 (s, 3H), 2.18 (dp, *J* = 9.5, 6.6 Hz, 1H), 1.03 (d, *J* = 6.6 Hz, 3H), 0.72 (d, *J* = 6.6 Hz, 3H); **$^{13}$C{$^1$H} NMR** (101 MHz, $CDCl_3$) δ 167.2, 162.4 (d, *J* = 246.9 Hz), 162.1, 155.1, 137.9, 135.8 (d, *J* = 3.2 Hz), 135.2, 134.1, 131.3, 130.8 (d, *J* = 8.1 Hz), 128.6, 127.1, 121.6, 115.6 (d, *J* = 21.3 Hz), 52.4, 41.4, 30.0, 23.8, 21.7, 20.7; **$^{19}$F**

**NMR** (376 MHz, CDCl3) δ -114.8; **HRMS** (ESI-TOF) calcd for $C_{24}H_{26}FN_2O^+$ $[M+H]^+$ 377.2024, found 377.2034.

**5p':** $R_f$ = 0.3 (silica gel, 40% EtOAc in hexanes); **$^1$H NMR** (400 MHz, $CDCl_3$) δ 8.72 (d, $J$ = 7.1 Hz, 1H), 8.07 – 7.76 (m, 2H), 7.50 – 7.37 (m, 4H), 7.20 – 7.14 (m, 2H), 7.13 – 7.05 (m, 3H), 4.18 (ddd, $J$ = 13.5, 7.3, 4.5 Hz, 1H), 3.35 (dt, $J$ = 13.6, 2.3 Hz, 1H), 2.79 (ddd, $J$ = 10.2, 4.4, 2.6 Hz, 1H), 2.63 (s, 3H), 2.29 (dp, $J$ = 10.1, 6.6 Hz, 1H), 0.92 (d, $J$ = 6.7 Hz, 3H), 0.54 (d, $J$ = 6.7 Hz, 3H); **$^{13}$C{$^1$H} NMR** (101 MHz, $CDCl_3$) δ 167.1, 162.3 (d, $J$ = 247.0 Hz), 160.4, 156.7, 138.3, 135.5 (d, $J$ = 3.4 Hz), 135.0, 134.2, 131.2, 131.0 (d, $J$ = 7.9 Hz), 128.4, 127.0, 120.8, 115.5 (d, $J$ = 21.3 Hz), 46.5, 41.4, 30.0, 24.5, 21.3, 20.3; **$^{19}$F NMR** (376 MHz, $CDCl_3$) δ -114.7; **HRMS** (ESI-TOF) calcd for $C_{24}H_{26}FN_2O^+$ $[M+H]^+$ 377.2024, found 377.2031.

***tert*-butyl benzoyl(2-(5-(4-fluorophenyl)-6-methylpyridin-2-yl)-3-methylbutyl)carbamate (5q) III-191) and *tert*-butyl benzoyl(2-(3-(4-fluorophenyl)-6-methylpyridin-2-yl)-3-methylbutyl)carbamate (5q'):**

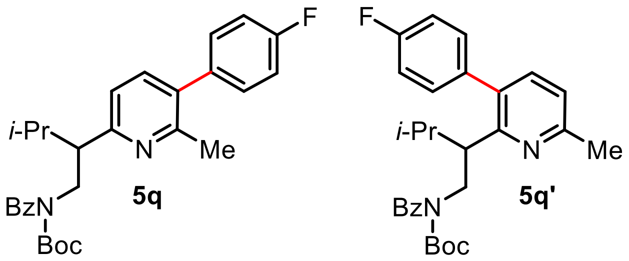


Following the general procedure II, products **5q** and **5q'** were obtained in 58% yield in 9:1 ratio as a pale yellow viscous liquid and pale yellow solid respectively. **5q:** $R_f$ = 0.45 (silica gel, 30% EtOAc in hexanes); **$^1$H NMR** (400 MHz, $CDCl_3$) δ 7.40 – 7.31 (m, 2H), 7.22 (t, $J$ = 7.6 Hz, 2H), 7.15 – 7.02 (m, 7H), 4.52 (dd, $J$ = 13.4, 11.0 Hz, 1H), 4.07 (dd, $J$ = 13.4, 4.9 Hz, 1H), 3.24 (d, $J$ = 11.5 Hz, 1H), 2.29 (s, 3H), 2.08 (dt, $J$ = 16.4, 8.1 Hz, 1H), 1.15 (d, $J$ = 6.7 Hz, 3H), 1.06 (s, 9H), 0.85 (d, $J$ = 6.7 Hz, 3H); **$^{13}$C{$^1$H} NMR** (101 MHz, $CDCl_3$) δ 173.6, 162.2 (d, $J$ = 247.0 Hz), 160.6, 155.1, 153.5, 137.9, 137.1, 136.2, 133.6, 131.0, 130.7 (d, $J$ = 8.1 Hz), 128.2, 127.9, 127.8, 127.7, 121.4, 115.3 (d, $J$ = 21.5 Hz), 115.2, 82.4, 53.9, 48.4, 31.8, 29.8, 27.5, 23.2, 21.2, 20.9; **$^{19}$F NMR** (376 MHz, $CDCl_3$) δ -115.35; **HRMS** (ESI-TOF) calcd for $C_{29}H_{34}FN_2O_3^+$ $[M+H]^+$ 477.2548, found 477.2548.

**5q'**: $R_f$ = 0.5 (silica gel, 30% EtOAc in hexanes); **$^1$H NMR** (400 MHz, $CDCl_3$) δ 7.42 – 7.35 (m, 1H), 7.32 – 7.25 (m, 5H), 7.20 – 7.13 (m, 2H), 7.09 (t, $J$ = 8.7 Hz, 2H), 6.89 (d, $J$ = 7.8 Hz, 1H), 4.58 (dd, $J$ = 13.2, 9.8 Hz, 1H), 3.96 (dd, $J$ = 13.2, 4.4 Hz, 1H), 3.44 (ddd, $J$ = 9.7, 8.2, 4.4 Hz, 1H), 2.36 (s, 3H), 2.09 – 1.90 (m, 1H), 1.02 (s, 9H), 0.95 (d, $J$ = 6.8 Hz, 3H), 0.65 (d, $J$ = 6.7 Hz, 3H); **$^{13}$C{$^1$H} NMR** (101 MHz, $CDCl_3$) δ 173.4, 162.3 (d, $J$ = 246.7 Hz), 158.7, 157.3, 153.7, 137.9, 137.7, 136.3, 134.8, 131.5 (d, $J$ = 7.9 Hz), 130.9, 127.9, 127.5, 120.3, 115.2 (d, $J$ = 21.4 Hz), 82.4, 48.8, 48.3, 32.4, 27.4, 24.2, 21.2, 20.5; **$^{19}$F NMR** (376 MHz, $CDCl_3$) δ -115.7; **HRMS** (ESI-TOF) calcd for $C_{29}H_{34}FN_2O_3^+$ $[M+H]^+$ 477.2548, found 477.2547.

**2-(4-fluorophenyl)-6-isopropyl-5-(3-nitrophenyl)-*N*,3-diphenylisonicotinamide (5r):**

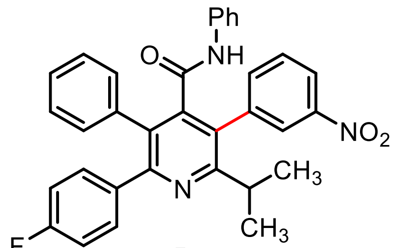

**5r**

Following the general procedure II,* product **5r** was isolated in 43% yield as a white solid, while 24% of starting material **4r** was recovered. **5r**: $R_f$ = 0.45 (silica gel, 30% EtOAc in hexanes); **$^1$H NMR** (400 MHz, $CDCl_3$) δ 8.31 (t, $J$ = 2.0 Hz, 1H), 8.26 (ddd, $J$ = 8.2, 2.3, 1.1 Hz, 1H), 7.78 (dt, $J$ = 7.7, 1.3 Hz, 1H), 7.62 (t, $J$ = 7.9 Hz, 1H), 7.42 – 7.35 (m, 2H), 7.30 – 7.25 (s, 4H), 7.11 (dd, $J$ = 8.4, 6.9 Hz, 2H), 7.03 – 6.98 (m, 1H), 6.95 – 6.86 (m, 2H), 6.73 – 6.65 (m, 3H), 2.95 (hept, $J$ = 6.7 Hz, 1H), 1.31 (d, $J$ = 6.7 Hz, 3H), 1.26 (d, $J$ = 6.6 Hz, 3H); **$^{13}$C{$^1$H} NMR** (101 MHz, $CDCl_3$) δ 165.0, 163.9, 162.8 (d, $J$ = 248.2 Hz), 156.4, 148.1, 145.6, 138.6, 136.8, 136.0, 136.0, 135.9 (d, $J$ = 3.1 Hz), 132.1 (d, $J$ = 8.2 Hz), 130.0, 129.6, 129.0, 129.0, 128.9, 128.8, 128.2, 125.6, 124.4, 123.2, 121.2, 114.9 (d, $J$ = 21.6 Hz), 32.5, 22.6, 22.5; **$^{19}$F NMR** (376 MHz, $CDCl_3$) δ -113.60; **HRMS** (ESI-TOF) calcd for $C_{33}H_{27}FN_3O_3^+$ $[M+H]^+$ 532.2031, found 532.2045.

** Reaction was continued for 48 h.*

**4-ethyl-3-(4-fluorophenyl)-2-methyl-6-(morpholinomethyl)-7,8-dihydroquinolin-5(6*H*)-one (5s):**

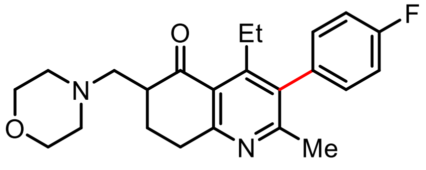

**5s**

Following the general procedure II, product **5s** was isolated in 34% yield as a brown solid. **5s:** $R_f$ = 0.3 (silica gel, 30% EtOAc in hexanes); **$^1$H NMR** (400 MHz, $CDCl_3$) δ 7.21 – 7.04 (m, 4H), 3.70 (br. s, 5H), 3.19 (tq, $J$ = 15.5, 5.6 Hz, 2H), 2.93 – 2.74 (m, 2H), 2.69 (dq, $J$ = 11.9, 7.3 Hz, 1H), 2.58 – 2.34 (m, 5H), 2.21 (s, 3H), 2.02 – 1.90 (m, 1H), 1.76 – 1.56 (m, 1H), 0.96 (t, $J$ = 7.3 Hz, 3H); **$^{13}$C{$^1$H} NMR** (101 MHz, $CDCl_3$) δ 201.2, 162.6, 162.2 (d, $J$ = 247.0 Hz), 160.2, 154.1, 135.4, 134.0, 130.9 (d, $J$ = 8.4 Hz), 130.8, 124.7, 115.8 (d, $J$ = 21.4 Hz), 115.78 (d, $J$ = 21.5 Hz), 67.0, 58.4, 54.0, 46.5, 32.2, 24.7, 24.0, 14.8; **$^{19}$F NMR** (376 MHz, $CDCl_3$) δ -114.5; **HRMS** (ESI-TOF) calcd for $C_{23}H_{28}FN_2O_2^+$ $[M+H]^+$ 383.2129, found 383.2129.

**6-(tert-butyl)-2,3-diphenylpyridine (5t) and 2-(tert-butyl)-4,6-diphenylpyridine (5t'):**

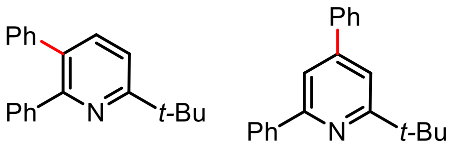

**5t** **5t'**

Following the general procedure II, products **5t** and **5t'** were obtained in 58% yield in 10.4:1 ratio respectively as a white solid. **5t:** $R_f$ = 0.3 (silica gel, 10% EtOAc in hexanes); **$^1$H NMR** (400 MHz, $CDCl_3$) δ 7.55 (dd, $J$ = 8.0, 0.9 Hz, 1H), 7.38 – 7.32 (m, 2H), 7.28 (d, $J$ = 8.0 Hz, 1H), 7.22 – 7.09 (m, 2H), 7.10 (dd, $J$ = 7.4, 2.2 Hz, 2H), 1.37 (d, $J$ = 0.9 Hz, 9H); **$^{13}$C{$^1$H} NMR** (101 MHz, $CDCl_3$) δ 167.9, 155.1, 140.9, 140.7, 138.8, 132.8, 130.4, 129.7, 128.4, 127.7, 127.6, 127.0, 117.4, 37.6, 30.4; **HRMS** (ESI-TOF) calcd for $C_{21}H_{22}N^+$ $[M+H]^+$ 288.1747, found 288.1753.

**5t’:** $R_f$ = 0.6 (silica gel, 10% EtOAc in hexanes); **$^1$H NMR** (400 MHz, $CDCl_3$) δ 8.20 – 8.10 (m, 2H), 7.75 (d, *J* = 1.4 Hz, 1H), 7.71 – 7.65 (m, 2H), 7.54 – 7.35 (m, 7 H), 1.48 (s, 9H). Spectroscopic data are in agreement with those in the literature.[12]

**2,6-di-tert-butyl-3-phenylpyridine (5u):**

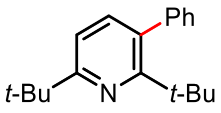

**5u**

Following the general procedure II, product **5u** was isolated in 33% yield as a yellow viscous liquid, while 25% of starting material was recovered. **5u:** $R_f$ = 0.4 (silica gel, 10% EtOAc in hexanes); **$^1$H NMR** (400 MHz, $CDCl_3$) δ 7.36 – 7.31 (m, 3H), 7.26 – 7.20 (m, 3H), 7.10 (d, *J* = 7.9 Hz, 1H), 1.39 (s, 9H), 1.20 (s, 9H); **$^{13}$C{$^1$H} NMR** (101 MHz, $CDCl_3$) δ 166.0, 162.9, 143.3, 139.9, 133.1, 130.0, 127.6, 126.9, 114.8, 40.4, 37.7, 31.7, 30.3; **HRMS** (ESI-TOF) calcd for $C_{19}H_{26}N^+$ $[M+H]^+$ 268.2060, found 268.2058.

**3,4-dimethyl-5-phenylpyridine (5v) and 4,5-dimethyl-2-phenylpyridine (5v’):**

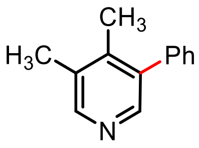

**5v**

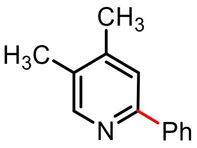

**5v'**

Following the general procedure II, products **5v** and **5v’** were obtained in 58% yield in 1.9:1 ratio respectively as a brown solid. **5v:** $R_f$ = 0.3 (silica gel, 30% EtOAc in hexanes); **$^1$H NMR** (400 MHz, $CDCl_3$) δ 8.33 (s, 1H), 8.29 (s, 1H), 7.44 (ddt, *J* = 8.1, 6.5, 1.2 Hz, 2H), 7.41 – 7.35 (m, 1H), 7.31 – 7.27 (m, 2H), 2.31 (s, 3H), 2.18 (s, 3H); **$^{13}$C{$^1$H} NMR** (101 MHz, $CDCl_3$) δ 149.0, 148.0, 143.3, 138.5, 137.6, 132.3, 129.6, 128.5, 127.6, 17.2, 16.5; **HRMS** (ESI-TOF) calcd for $C_{13}H_{14}N^+$ $[M+H]^+$ 184.1121, found 184.1120.

**5v’**: $R_f$ = 0.7 (silica gel, 30% EtOAc in hexanes); **$^1$H NMR** (400 MHz, $CDCl_3$) δ 8.41 (s, 1H), 8.00 – 7.91 (m, 2H), 7.50 (s, 1H), 7.49 – 7.42 (m, 2H), 7.42 – 7.35 (m, 1H), 2.34 (s, 3H), 2.29 (s, 3H). Spectroscopic data are in agreement with those in the literature.[13]

**Ethyl 4-ethyl-6-(4-fluorophenyl)-3,5-dimethylpicolinate (5w):**

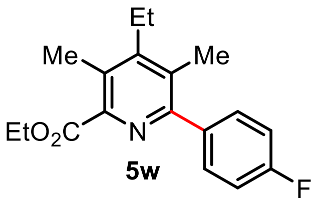

**5w**

Following the general procedure II, product **5w** was isolated in 43% yield as a viscous yellow liquid. **5w**: $R_f$ = 0.3 (silica gel, 30% EtOAc in hexanes); **$^1$H NMR** (400 MHz, $CDCl_3$) δ 7.16 – 7.06 (m, 4H), 4.06 (q, *J* = 7.1 Hz, 2H), 2.74 (q, *J* = 7.6 Hz, 2H), 2.64 (s, 3H), 2.07 (s, 3H), 1.16 (t, *J* = 7.5 Hz, 3H), 0.99 (t, *J* = 7.1 Hz, 3H); **$^{13}$C{$^1$H} NMR** (101 MHz, $CDCl_3$) δ 167.5, 162.4 (d, *J* = 246.7 Hz), 155.7, 146.9, 144.5, 138.3, 134.0 (d, *J* = 3.5 Hz), 133.8, 131.1 (d, *J* = 7.9 Hz), 115.3 (d, *J* = 21.4 Hz), 61.4, 22.9, 22.8, 16.4, 13.9, 12.9; **$^{19}$F NMR** (376 MHz, $CDCl_3$) δ -114.8; **HRMS** (ESI-TOF) calcd for $C_{18}H_{21}FNO_2^+$ $[M+H]^+$ 302.1551, found 302.1559.

**III. Synthesis of Starting Materials**

**IIIA. General procedure for synthesis of chloro diazirine 1**

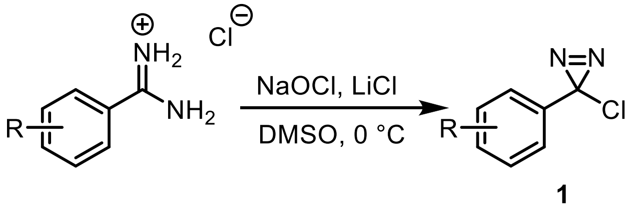

Diazerenes were prepared according to literature procedure with slight variation.[15] A solution of LiCl (5.30 g, 0.125 mol) in DMSO (87 mL) was rapidly stirred within a 1-L Erlenmeyer flask bearing a ground joint. Meanwhile, NaCl (31.7 g, 0.542 mol) was dissolved in cold 0.56 M NaOCl (296 mL, 0.166 mol) in another 1-L Erlenmeyer flask. The salty chlorine bleach solution was transferred to a 500-mL dropping funnel equipped with an equilibrating sidearm and ground joints. Amidine hydrochloride hydrate (23.7 mmol) was added to the 1-L Erlenmeyer reaction flask, and then pentane (50 mL) was poured in. The reaction flask was submerged into an ice bath, and rapid stirring was continued. The NaOCl/NaCl solution was dripped in within 15 min. The reaction mixture was stirred for an additional 30 min in the ice bath. The pentane layer was collected using a 1-L separatory funnel. Next, the aqueous DMSO layer was extracted with $Et_2O$ (4 × 25 mL). The combined organic extracts were washed with water (2 × 20 mL) and brine (20 mL). The organic layer was dried over anhydrous $MgSO_4$ and then carefully rotary-evaporated. The residual oil was chromatographed using pentane (or diethyl ether/pentane) as eluant followed by careful rotary evaporation (ca. 30 min) to afford pure diazerene in 20 – 60% yield.

***CAUTION! Perform behind a safety shield under dim lighting.***

**3-chloro-3-phenyl-3*H*-diazirine (1a):**

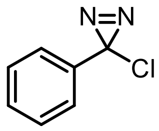

**1a**

Following the general procedure IIIA, product **1a** was isolated in 64% yield as a colorless liquid. **1a:** $R_f = 0.5$ (silica gel, 5% EtOAc in hexanes); **$^1$H NMR** (400 MHz, $CDCl_3$) δ 7.46 – 7.33 (m, 3H), 7.18 – 7.07 (m, 2H). Spectroscopic data are in agreement with those in the literature.[15]

**2-(3-chloro-3*H*-diazirin-3-yl)pyridine (1b):**

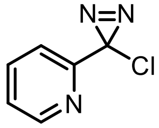

**1b**

Following the general procedure IIIA, product **1b** was isolated in 36% yield as a colorless liquid. **1b:** $R_f = 0.4$ (silica gel, 10% EtOAc in hexanes); **$^1$H NMR** (400 MHz, $CDCl_3$) δ 8.53 (ddd, $J$ = 4.8, 1.8, 0.9 Hz, 1H), 7.80 (td, $J$ = 7.8, 1.7 Hz, 1H), 7.69 (dt, $J$ = 8.0, 1.1 Hz, 1H), 7.30 (ddd, $J$ = 7.6, 4.8, 1.1 Hz, 1H). Spectroscopic data are in agreement with those in the literature.[16]

**3-chloro-3-(4-nitrophenyl)-3*H*-diazirine (1c):**

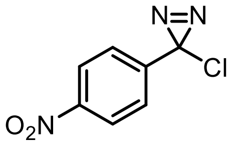


**1c**

Following the general procedure IIIA, product **1c** was isolated in 14% yield as a pale yellow solid. **1c:** $R_f$ = 0.4 (silica gel, 10% EtOAc in hexanes); $^1$**H NMR** (400 MHz, $CDCl_3$) δ 8.26 (d, *J* = 8.9 Hz, 2 H), 7.30 (d, *J* = 9.0 Hz, 2 H). Spectroscopic data are in agreement with those in the literature.[15]

**3-chloro-3-(3-chlorophenyl)-3*H*-diazirine (1d):**

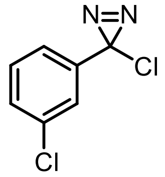


**1d**

Following the general procedure IIIA, product **1d** was isolated in 61% yield as a pale-yellow liquid. **1d:** $R_f$ = 0.4 (silica gel, 5% EtOAc in hexanes); $^1$**H NMR** (400 MHz, $CDCl_3$) δ 7.38 (ddd, *J* = 8.0, 2.0, 1.2 Hz, 1H), 7.35 – 7.29 (m, 1H), 7.16 (td, *J* = 1.9, 0.5 Hz, 1H), 6.95 (ddd, *J* = 7.7, 1.9, 1.2 Hz, 1H); $^{13}$**C{$^1$H} NMR** (101 MHz, $CDCl_3$) δ 137.8, 135.0, 129.9, 129.7, 126.6, 124.2, 46.3; **HRMS** (ESI-TOF) calcd for $C_7H_4Cl_2^+$ $[M\text{-}N_2]^+$ 157.9685, found 157.9693.

**3-chloro-3-(4-fluorophenyl)-3*H*-diazirine (1e):**

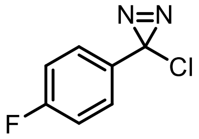


**1e**

Following the general procedure IIIA, product **1e** was isolated in 63% yield as a colorless liquid. **1e:** $R_f$ = 0.4 (silica gel, 5% EtOAc in hexanes); $^1$**H NMR** (400 MHz, $CDCl_3$) δ 7.14 – 7.04 (m, 4H); $^{13}$**C{$^1$H} NMR** (101 MHz, $CDCl_3$) δ 163.29 (d, *J* = 250.1 Hz), 131.6, 127.95 (d, *J* = 8.7 Hz), 115.67 (d, *J* = 22.3 Hz), 46.6; $^{19}$F NMR (376 MHz, $CDCl_3$) δ −111.45; **HRMS** (ESI-TOF) calcd for $C_7H_4ClF^+$ $[M\text{-}N_2]^+$ 141.9980, found 141.9995.

**3-chloro-3-(*m*-tolyl)-3*H*-diazirine (1f):**

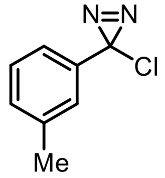


**1f**

Following the general procedure IIIA, product **1f** was isolated in 58% yield as a colorless liquid. **1f:** $R_f$ = 0.6 (silica gel, 5% EtOAc in hexanes); $^1$**H NMR** (400 MHz, $CDCl_3$) δ 7.29 (d, *J* = 7.8 Hz, 1H), 7.20 (d, *J* = 7.6 Hz, 1H), 6.91 (dt, *J* = 4.0, 1.7 Hz, 2H), 2.37 (s, 3H); $^{13}$**C{$^1$H} NMR** (101 MHz, $CDCl_3$) δ 138.6, 135.8, 130.2, 128.6, 126.7, 123.3, 47.3, 21.5; **HRMS** (ESI-TOF) calcd for $C_8H_8Cl^+$ $[M\text{-}N_2\text{+}H]^+$ 139.0309, found 139.0315.

**3-(3-bromophenyl)-3-chloro-3*H*-diazirine (1g):**

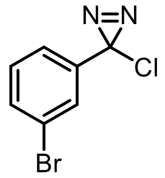


**1g**

Following the general procedure IIIA, product **1g** was isolated in 55% yield as a pale yellow liquid. **1g:** $R_f$ = 0.5 (silica gel, 5% EtOAc in hexanes); $^1$**H NMR** (400 MHz, $CDCl_3$) δ 7.53 (ddd, *J* = 8.0, 1.9, 1.0 Hz, 1H), 7.31 (t, *J* = 1.9 Hz, 1H), 7.29 – 7.23 (m, 1H), 7.00 (ddd, *J* = 8.0, 1.9, 1.0 Hz, 1H); $^{13}$**C{$^1$H} NMR** (101 MHz, $CDCl_3$) δ 137.9, 132.6, 130.1, 129.4, 124.7, 123.0, 46.2; **HRMS** (ESI-TOF) calcd for $C_7H_4BrCl^+$ $[M\text{-}N_2]^+$ 201.9185, found 201.9190.

**2-(3-chloro-3*H*-diazirin-3-yl)pyrazine (1h):**

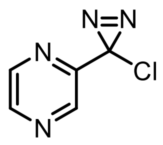

**1h**

Following the general procedure IIIA, product **1h** was isolated in 23% yield as a pale yellow solid. **1h:** $R_f$ = 0.4 (silica gel, 10% EtOAc in hexanes); **$^1$H NMR** (400 MHz, $CDCl_3$) δ 9.02 (d, *J* = 1.5 Hz, 1H), 8.61 (d, *J* = 2.5 Hz, 1H), 8.49 (dd, *J* = 2.5, 1.5 Hz, 1H); **$^{13}$C{$^1$H} NMR** (101 MHz, $CDCl_3$) δ 149.7, 144.4 (2C), 143.4, 45.4; **HRMS** (ESI-TOF) calcd for $C_5H_3ClN_4Na^+$ $[M+Na]^+$ 176.9938, found 176.9938.

**3-chloro-3-(2-fluorophenyl)-3*H*-diazirine (1i):**

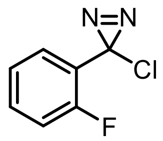

**1i**

Following the general procedure IIIA, product **1i** was isolated in 61% yield as a colorless liquid. **1i:** $R_f$ = 0.4 (silica gel, 5% EtOAc in hexanes); **$^1$H NMR** (400 MHz, $CDCl_3$) δ 7.51 (td, *J* = 7.6, 1.8 Hz, 1H), 7.43 – 7.35 (m, 1H), 7.18 (td, *J* = 7.6, 1.2 Hz, 1H), 7.08 (ddd, *J* = 10.8, 8.3, 1.1 Hz, 1H); **$^{13}$C{$^1$H} NMR** (101 MHz, $CDCl_3$) δ 160.57 (d, *J* = 254.0 Hz), 132.10 (d, *J* = 8.2 Hz), 129.12, 124.60 (d, *J* = 3.9 Hz), 122.75 (d, *J* = 11.4 Hz), 116.77 (d, *J* = 20.6 Hz), 43.06; **$^{19}$F NMR** (376 MHz, $CDCl_3$) δ −113.40; **HRMS** (ESI-TOF) calcd for C7H5ClF$^+$ $[M-N_2+H]^+$ 143.0058, found 143.0063.

**3-chloro-3-(3-methoxyphenyl)-3*H*-diazirine (1j):**

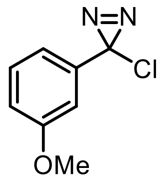

**1j**

Following the general procedure IIIA, product **1j** was isolated in 64% yield as a pale yellow liquid. **1j:** $R_f$ = 0.5 (silica gel, 5% EtOAc in hexanes); **$^1$H NMR** (400 MHz, $CDCl_3$) δ 7.29 (t, *J* = 8.0 Hz, 1H), 6.93 (ddd, *J* = 8.3, 2.5, 0.9 Hz, 1H), 6.69 (t, *J* = 2.2 Hz, 1H), 6.62 (ddd, *J* = 7.8, 1.9, 0.9 Hz, 1H), 3.82 (s, 3H); **$^{13}$C{$^1$H} NMR** (101 MHz, $CDCl_3$) δ 159.8, 137.4, 129.7, 118.3, 115.1, 112.0, 55.5, 47.1; **HRMS** (ESI-TOF) calcd for $C_8H_7ClN_2O^+$ $[M]^+$ 182.0241, found 182.0245.

**3-chloro-3-(3-nitrophenyl)-3*H*-diazirine (1k):**

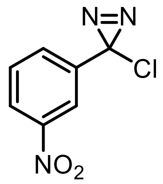

**xx**

Following the general procedure IIIA, product **1k** was isolated in 51% yield as a colorless liquid. **1k:** $R_f$ = 0.4 (silica gel, 10% EtOAc in hexanes); **$^1$H NMR** (400 MHz, $CDCl_3$) δ 8.27 (ddd, *J* = 8.2, 2.2, 1.0 Hz, 1H), 8.05 (t, *J* = 2.1 Hz, 1H), 7.61 (t, *J* = 8.1 Hz, 1H), 7.42 (ddd, *J* = 7.9, 2.0, 1.0 Hz, 1H); **$^{13}$C{$^1$H} NMR** (101 MHz, $CDCl_3$) δ 148.5, 138.0, 131.6, 129.9, 124.3, 121.5, 46.0; **HRMS** (ESI-TOF) calcd for $C_7H_5ClNO_2^+$ $[M-N_2+H]^+$ 170.0003, found 170.0012.

**4-chloro-3-(3-chlorophenyl)-3*H*-diazirine (1l):**

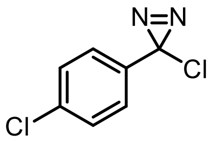


**1l**

Following the general procedure IIIA, product **1l** was isolated in 62% yield as a colorless liquid. **1l:** $R_f = 0.6$ (silica gel, 5% EtOAc in hexanes); **$^1$H NMR** (400 MHz, $CDCl_3$) δ 7.37 (d, *J* = 8.0 Hz, 2H), 7.05 (d, *J* = 8.2 Hz, 2H). Spectroscopic data are in agreement with those in the literature.[15]

**3-chloro-3-(3-fluorophenyl)-3*H*-diazirine (1m):**

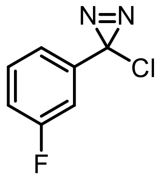


**1m**

Following the general procedure IIIA, product **1m** was isolated in 61% yield as a colorless liquid. **1m:** $R_f = 0.5$ (silica gel, 5% EtOAc in hexanes); **$^1$H NMR** (400 MHz, $CDCl_3$) δ 7.36 (td, *J* = 8.1, 5.7 Hz, 1H), 7.10 (tdd, *J* = 8.3, 2.5, 0.9 Hz, 1H), 6.92 (dt, *J* = 9.7, 2.2 Hz, 1H), 6.82 (ddd, *J* = 7.9, 1.9, 1.0 Hz, 1H); **$^{13}$C{$^1$H} NMR** (101 MHz, $CDCl_3$) δ 162.8 (d, *J* = 247.9 Hz), 138.3 (d, J = 8.2 Hz), 130.3 (d, *J* = 8.4 Hz), 121.6 (d, *J* = 3.1 Hz), 116.6 (d, *J* = 21.2 Hz), 113.9 (d, *J* = 24.7 Hz), 46.4; **$^{19}$F NMR** (376 MHz, $CDCl_3$) δ -111.54; **HRMS** (ESI-TOF) calcd for $C_7H_4ClFN_2^+$ $[M]^+$ 170.0047, found 170.0019.

**3-chloro-3-(p-tolyl)-3*H*-diazirine (1n):**

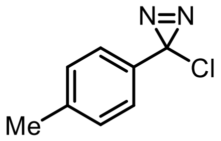


**1n**

Following the general procedure IIIA, product **1n** was isolated in 56% yield as a colorless liquid. **1n:** $R_f = 0.6$ (silica gel, 5% EtOAc in hexanes); **$^1$H NMR** (400 MHz, $CDCl_3$) δ 7.22 – 7.16 (m, 2H), 7.00 (d, *J* = 8.3 Hz, 2H), 2.38 (s, 3H). Spectroscopic data are in agreement with those in the literature.[17]

**4-(3-chloro-3*H*-diazirin-3-yl)pyridine (1o):**

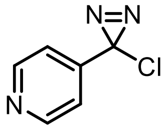


**1o**

Following the general procedure IIIA, product **1o** was isolated in 24% yield as a colorless liquid. **1o:** $R_f = 0.35$ (silica gel, 10% EtOAc in hexanes); **$^1$H NMR** (400 MHz, $CDCl_3$) δ 8.77 – 8.51 (m, 2H), 7.06 – 6.95 (m, 2H); **$^{13}$C{$^1$H} NMR** (101 MHz, $CDCl_3$) δ 150.1, 144.5, 120.5, 45.4; **HRMS** (ESI-TOF) calcd for $C_6H_4ClN_3^+$ $[M+H]^+$ 153.0094, found 153.0099.

**3-(4-bromophenyl)-3-chloro-3*H*-diazirine (1p):**

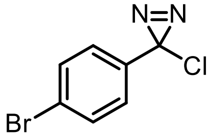


**1p**

Following the general procedure IIIA, product **1p** was isolated in 59% yield as a colorless liquid. **1p:** $R_f = 0.5$ (silica gel, 5% EtOAc in hexanes); **$^1$H NMR** (400 MHz, $CDCl_3$) δ 7.62 – 7.43 (m, 2H), 7.05 – 6.90 (m, 2H); **$^{13}$C{$^1$H} NMR** (101 MHz, $CDCl_3$) δ 134.9, 131.9, 127.7, 124.0, 46.7; **HRMS** (ESI-TOF) calcd for $C_7H_4BrCl^+$ $[M+H]^+$ 201.9179, found 201.9193.

**IIIB. Synthesis of indoles and pyrroles**

**4,6-dicyclopropyl-2-methyl-1*H*-indole (2v):**

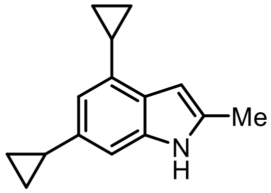

**2v**

To a degassed suspension of 4,6-dichloro-2-methyl-1*H*-indole (100 mg, 0.50 mmol, 1.0 equiv), cyclopropylboronic acid (171 mg, 2.0 mmol, 4.0 equiv), $K_3PO_4$ (690 mg, 3.0 mmol, 6.0 equiv ), tricyclohexyl phosphine (28 mg, 0.10 mmol), and water (0.5 mL) in toluene (5.0 mL) was added palladium (II) acetate (11 mg, 50 µmol). The reaction mixture was stirred at 100 °C for 36 h. The reaction mixture was allowed to cool to 23 °C and filtered through a pad of celite. The filtrate was concentrated under reduced pressure and the obtained residue was purified by flash column chromatography (silica gel, 2% EtOAc in hexanes) to afford pure **2v** (74 mg, 0.35 mmol, 70% yield) as a brown amorphous solid. **2v**: $R_f = 0.60$ (silica gel, 10% EtOAc in hexanes). **2v: $^1$H NMR** (400 MHz, $CD_3CN$) δ 8.86 (s, 1H), 6.78 (t, *J* = 1.1 Hz, 1H), 6.33 (d, *J* = 1.4 Hz, 1H), 6.19 (dt, *J* = 2.2, 1.1 Hz, 1H), 2.35 (d, *J* = 1.0 Hz, 3H), 2.11 – 2.03 (m, 1H), 1.90 – 1.81 (m, 1H), 0.93 – 0.88 (m, 2 H), 0.88 – 0.83 (m, 2 H), 0.75 – 0.67 (m, 2H), 0.64 – 0.56 (m, 2H); **$^{13}$C{$^1$H} NMR** (101 MHz, $CD_3CN$) δ 137.4, 135.4, 134.8, 127.8, 118.0, 113.6, 105.4, 98.6, 16.3, 13.7, 13.5, 9.3, 8.0; **HRMS** (ESI-TOF) calcd for $C_{15}H_{18}N^+$ $[M+H]^+$ 212.1434, found 212.1444.

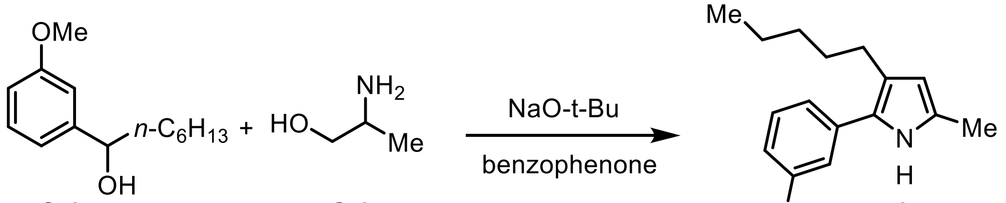


**S-1** **S-2** **4e**

**2-(3-methoxyphenyl)-5-methyl-3-pentyl-1H-pyrrole (4e)[18]:**

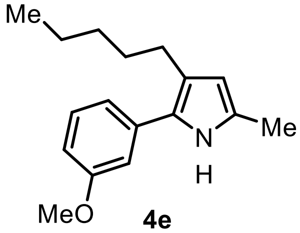

**4e**

To a round-bottom flask (10 mL) equipped with a condenser and stirring bar were added 1-(3-methoxyphenyl)heptan-1-ol (**S-1**) (100 mg, 0.45 mmol), 2-aminopropan-1-ol (**S-2**) (36 mg, 0.5 mmol), NaO-*t*-Bu (48 mg, 0.5 mmol), benzophenone (495 mg, 2.7 mmol) in dry toluene (6 mL). The reaction mixture was purged with argon and refluxed (110 °C, oil bath) overnight (~18 h). After the reaction mixture had been cooled to room temperature, it was added directly to a silica column to afford pyrrole **4e** (31 mg, 27%, 0.12 mmol) as a yellow oil. **4e**: $R_f = 0.3$ (silica gel, 10% EtOAc in hexanes); **$^1$H NMR** (400 MHz, $C_6D_6$) δ 7.20 (d, *J* = 8.0 Hz, 1H), 7.06 (dd, *J* = 2.6, 1.6 Hz, 1H), 6.97 (dt, *J* = 7.7, 1.3 Hz, 1H), 6.72 (ddd, *J* = 8.3, 2.6, 1.0 Hz, 1H), 5.97 (d, *J* = 2.9 Hz, 1H), 3.40 (s, 3H), 2.83 – 2.71 (m, 2H), 1.94 (d, *J* = 0.8 Hz, 3H), 1.76 – 1.70 (m, 2H), 1.38 – 1.23 (m, 4H), 0.86 – 0.84 (m, 3H); **$^{13}$C{$^1$H} NMR** (101 MHz, $C_6D_6$) δ 160.5, 136.3, 129.8, 127.2, 126.8, 122.6, 119.5, 113.0, 111.2, 109.3, 54.8, 32.3, 31.6, 27.2, 23.1, 14.4, 13.1; **HRMS** (ESI-TOF) calcd for $C_{17}H_{24}N^+$ $[M+H]^+$ 258.1852, found 258.1844.

**4-(5-methyl-1*H*-pyrrol-2-yl)benzonitrile (4h)[19]:**

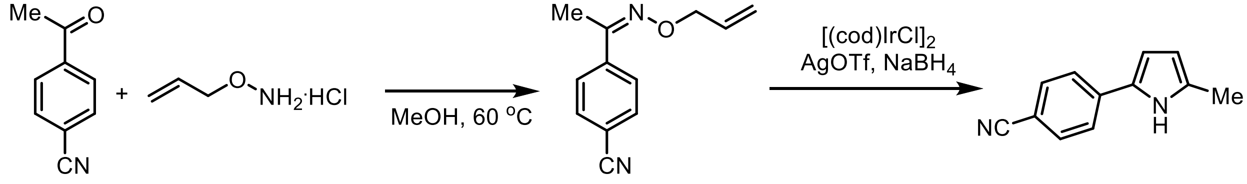

S-3 S-4 4h

***Step I***- A 25 mL round-bottom flask was charged with allylhydroxylamine hydrochloride salt (225 mg, 2.07 mmol, 1 equiv), NaOAc (170 mg, 2.07 mmol, 1 equiv) and MeOH (5 mL). The resulting slurry was allowed to stir at 25 °C for 30 min. At this time, 4-cyanoacetophenone **S-3** (300 mg, 2.07 mmol, 1 equiv), was dissolved in 2 mL of MeOH and added dropwise to the reaction slurry. The reaction mixture was then allowed to stir at 60 °C for 6 h. After completion of reaction, indicated by TLC, reaction mixture was concentrated in vacuo, 10 mL of water was added to the flask and extracted using EtOAc (3 × 10 mL). The organic layers were then combined, dried over $MgSO_4$, filtered and concentrated to afford **S-4** (400 mg, 2 mmol) as white solid which was used for next step without purification.

***Step II***- In a glove box, a 20 mL scintillation vial was charged with $[(cod)IrCl]_2$ (57 mg, 85 µmol, 0.05 equiv), AgOTf (43 mg, 0.17 mmol, 0.1 equiv), $NaBH_4$ (6.5 mg, 0.17 mmol, 0.1 equiv), and 3 mL of THF. This mixture was then allowed to stir at 25 °C for 20 min. O-Allyl oxime **S-4** (340 mg, 1.7 mmol, 1 equiv) was mixed with 2 mL of THF in a small vial. The oxime solution was then transferred to the scintillation vial containing the 5 mol % iridium mixture and allowed to stir at 25 °C for 24 h. Then the reaction mixture was transferred to 10 mL Teflon-sealed reaction flasks, charged with ~15 4 Å molecular sieves, and heated to 75 °C for 15 h. The reaction mixture was then transferred to scintillation vials and dry-loaded on ~3 mL of silica gel. The product was then purified by flash chromatography to afford pyrrole **4h** (120 mg, 0.66 mmol) as a brown solid. **4h:** $R_f = 0.45$ (silica gel, 20% EtOAc in hexanes); **$^1$H NMR** (400 MHz, $CDCl_3$) δ

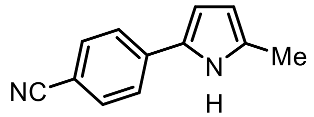

**4h**

8.28 (s, 1H), 7.66 – 7.54 (m, 2H), 7.52 – 7.41 (m, 2H), 6.56 (t, *J* = 3.1 Hz, 1H), 6.01 (ddd, *J* = 3.6, 2.5, 1.0 Hz, 1H), 2.36 (d, *J* = 0.8 Hz, 3H); **$^{13}$C{$^1$H} NMR** (101 MHz, $CDCl_3$) δ 136.9, 132.9, 131.7, 128.9, 123.1, 119.5, 109.5, 109.2, 108.1, 13.4; **HRMS** (ESI-TOF) calcd for $C_{12}H_{11}N_2^+$ $[M+H]^+$ 183.0917, found 183.0916.

**2-isobutyl-5-(thiophen-2-yl)-1$H$-pyrrole (4o):**

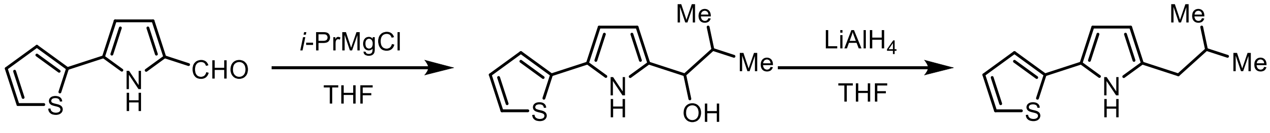


***Step I***- To a magnetically stirred solution of aldehyde **S-5** (240 mg, 1.35 mmol) in THF (5 mL) at 0 °C was added isopropylmagnesium chloride solution (2 mL 1.3 M solution in THF, 2.7 mmol). The reaction mixture was slowly warmed to 23 °C and stirred for additional 1 h. After completion of reaction, indicated by TLC the reaction mixture was cooled to 0 °C and quenched with saturated solution of $NH_4Cl$ and organic layer was extracted in EtOAc (3 × 10 mL). Combined organic layers were washed with brine, dried over Na2SO4 and concentrated in vacuo to afford crude alcohol **S-6** as a pale-yellow oil which was used in next step without purification.

***Step II***- To a stirred solution of the so-obtained crude residue **S-6** in THF (5 mL) at 0 °C was added $LiAlH_4$ (86 mg, 2.26 mmol). After 5 min, the reaction mixture was heated to reflux. After completion of reaction, indicated by TLC, reaction mixture cooled to 0 °C and quenched by slow addition of EtOAc (2 mL) and then water (3 mL), and allowed to warm to 25 °C. The two phases were separated, and the aqueous layer was extracted with EtOAc (3 × 10 mL). The combined organic layers were dried over anhydrous $Na_2SO_4$, filtered, and concentrated under reduced pressure. The obtained residue was automated silica gel column chromatography to afford pure pyrrole **4o** (43 mg, 194 µmol, 14% yield for two steps) as pale-yellow oil.

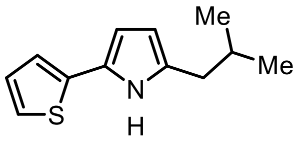


**4o**: $R_f$ = 0.6 (silica gel, 20% EtOAc in hexanes); **$^1$H NMR** (400 MHz, $CDCl_3$) δ 7.94 (br. s, 1H), 7.10 (dd, $J$ = 5.0, 1.2 Hz, 1H), 7.03 – 6.91 (m, 2H), 6.32 (t, $J$ = 3.0 Hz, 1H), 5.99 – 5.83 (m, 1H), 2.49 (d, $J$ = 7.1 Hz, 2H), 1.88 (dh, $J$ = 13.7, 6.8 Hz, 1H), 0.97 (d, $J$ = 6.6 Hz, 6H); **$^{13}$C{$^1$H} NMR** (101 MHz, $CDCl_3$) δ 136.9, 133.0, 127.7, 125.2, 122.0, 120.1, 108.0, 107.0, 37.4, 29.4, 22.6; **HRMS** (ESI-TOF) calcd for $C_{12}H_{16}NS^+$ $[M+H]^+$ 206.0998, found 206.0990.

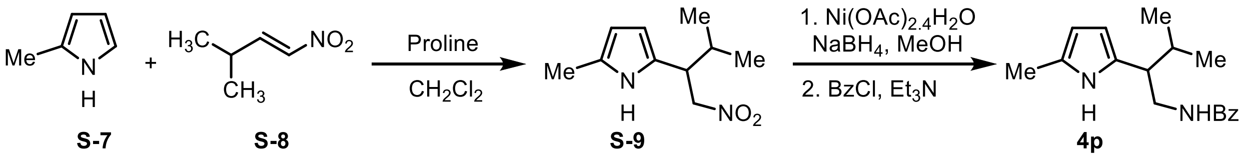


**(*rac*)-2-methyl-5-(3-methyl-1-nitrobutan-2-yl)-1*H*-pyrrole (S-9):** To a magnetically stirred solution of 2-methyl pyrrole **S-7** (200 mg, 2.47 mmol) in $CH_2Cl_2$ (5 mL) was added (*E*)-3-methyl-1-nitrobut-1-ene **S-8** (567 mg, 4.94 mmol) followed by (±)-proline (56 mg, 0.49 mmol) at 23 °C and the reaction mixture stirred for 12 h. After completion of reaction, indicated by TLC, reaction was quenched by sat. $NaHCO_3$ (2 mL) and water (3 mL). The two phases were separated, and the aqueous layer was extracted with $CH_2Cl_2$ (3 × 10 mL). The combined organic layers were dried over anhydrous $Na_2SO_4$, filtered, and concentrated under reduced pressure. The obtained residue was automated silica gel column chromatography to afford pyrrole **S-9** (358 mg, 1.82 mmol, 74% yield) as pale-yellow oil. **S-9**: $R_f = 0.35$ (silica gel, 20% EtOAc in hexanes); **$^1$H NMR** (400 MHz, $CDCl_3$) δ 7.66 (br s, 1H), 5.82 (t, *J* = 3.0 Hz, 1H), 5.81 – 5.77 (m, 1H), 4.64 (dd, *J* = 12.6, 6.1 Hz, 1H), 4.56 (dd, *J* = 12.6, 9.0 Hz, 1H), 3.28 (dt, *J* = 9.0, 6.3 Hz, 1H), 2.22 (d, *J* = 0.9 Hz, 3H), 1.93 (h, *J* = 6.7 Hz, 1H), 0.96 (d, *J* = 6.8 Hz, 3H), 0.91 (d, *J* = 6.7 Hz, 3H); **$^{13}$C{$^1$H} NMR** (101 MHz, $CDCl_3$) δ 127.2, 127.1, 106.4, 106.4, 78.5, 44.2, 30.8, 20.7, 19.6, 13.1; **HRMS** (ESI-TOF) calcd for $C_{10}H_{17}N_2O_2^+$ $[M+H]^+$ 197.1285, found 197.1290.

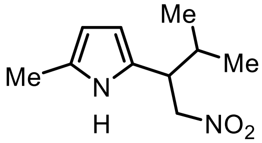


**S-9**

**(*rac*)-*N*-(3-methyl-2-(5-methyl-1*H*-pyrrol-2-yl)butyl)benzamide (4p):**

***Step1:*** Pyrrole **S-9** (200 mg, 1.02 mmol) was dissolved in methanol (5 mL), followed by the addition of $Ni(OAc)_2 \cdot 4H_2O$ (254 mg, 1.02 mmol) in one portion. Sodium borohydride (97 mg, 2.55 mmol) was added to the solution in three portions over 15 min at 0 °C. The reaction mixture turned black. After 2 h, the starting material was consumed as based on TLC. The solvent was removed by rotary evaporation, and the residue was redissolved in $CH_2Cl_2$ (15 mL) with the subsequent addition of concentrated ammonium hydroxide (15 mL). The mixture was stirred for 1.5 h, and two layers were separated. The aqueous layer was extracted with $CH_2Cl_2$ (3 × 15 mL). The combined organic layers were dried with $MgSO_4$. The filtration of the drying agent, followed by concentration via rotary evaporation, afforded a crude residue which was used directly for next step.

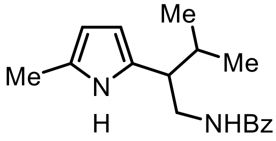


**4p**

***Step 2:*** To a magnetically stirred solution of so obtained crude amine in $CH_2Cl_2$ (5 mL) was added triethyl amine (212 µL, 1.53 mmol) followed by dropwise addition of benzoyl chloride (124 µL, 1.07 mmol) at 0 °C and the reaction mixture stirred for additional 2 h at same temperature. After completion of reaction, indicated by TLC, reaction was quenched by sat. $NaHCO_3$ (2 mL) and water (3 mL). The two phases were

separated, and the aqueous layer was extracted with $CH_2Cl_2$ (3 × 10 mL). The combined organic layers were dried over anhydrous $Na_2SO_4$, filtered, and concentrated under reduced pressure. The obtained residue was automated silica gel column chromatography to afford pyrrole **4p** (174 mg, 0.64 mmol, 63% yield) as pale-yellow viscous liquid. **4p**: $R_f$ = 0.45 (silica gel, 30% EtOAc in hexanes); **$^1$H NMR** (400 MHz, $CDCl_3$) δ 7.99 (br. s, 1H), 7.64 – 7.56 (m, 2H), 7.50 – 7.42 (m, 1H), 7.37 (dd, *J* = 8.2, 6.7 Hz, 2H), 6.15 (br. s, 1H), 5.86 (t, *J* = 3.0 Hz, 1H), 5.83 – 5.77 (m, 1H), 3.95 (ddd, *J* = 13.2, 7.0, 4.9 Hz, 1H), 3.35 (ddd, *J* = 13.2, 10.6, 4.3 Hz, 1H), 2.65 (ddd, *J* = 10.6, 7.2, 4.9 Hz, 1H), 2.23 (d, *J* = 0.9 Hz, 3H), 1.87 (h, *J* = 6.8 Hz, 1H), 1.00 (d, *J* = 6.7 Hz, 3H), 0.88 (d, *J* = 6.7 Hz, 3H); **$^{13}$C{$^1$H} NMR** (101 MHz, $CDCl_3$) δ 167.7, 134.8, 131.5, 130.2, 128.6, 127.0, 126.9, 106.4, 105.9, 46.1, 42.2, 31.6, 20.9, 20.5, 13.2; **HRMS** (ESI-TOF) calcd for $C_{17}H_{23}N_2O^+$ [M+H]$^+$ 271.1805, found 271.1811.

***(rac)-tert*-butyl benzoyl(3-methyl-2-(5-methyl-1*H*-pyrrol-2-yl)butyl)carbamate (4q):**

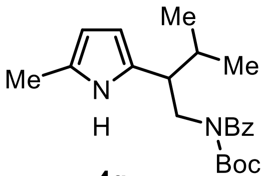


**4q**

To a magnetically stirred solution of pyrrole **4p** (42 mg, 150 μmol) in $CH_2Cl_2$ (2 mL) was added di-*tert*-butyl dicarbonate (98 mg, 0.45 mmol) followed by 4-dimethylaminopyridine (9 mg, 75 μmol) at 23 °C. The reaction mixture was heated to 60 °C and stirred for additional 30 at same temperature. After completion of reaction, indicated by TLC, reaction mixture was cooled to 0 °C, quenched by sat. $NaHCO_3$ (2 mL) and water (3 mL). The two phases were separated, and the aqueous layer was extracted with EtOAc (3 × 5 mL). The combined organic layers were dried over anhydrous $Na_2SO_4$, filtered, and concentrated under reduced pressure. The obtained residue was automated silica gel column chromatography to afford pyrrole **4q** (51 mg, 137 μmol, 89% yield) as yellow solid. **4q**: $R_f$ = 0.35 (silica gel, 20% EtOAc in hexanes); **$^1$H NMR** (400 MHz, $CDCl_3$) δ 7.95 (br. s, 1H), 7.42 – 7.33 (m, 1H), 7.25 (t, *J* = 7.8 Hz, 2H), 7.12 – 6.93 (m, 2H), 5.75 (t, *J* = 2.9 Hz, 1H), 5.72 (dt, *J* = 3.9, 2.0 Hz, 1H), 4.20 (dd, *J* = 13.7, 11.5 Hz, 1H), 3.98 (dd, *J* = 13.7, 6.2 Hz, 1H), 3.11 (dt, *J* = 11.9, 6.2 Hz, 1H), 2.16 (d, *J* = 0.9 Hz, 3H), 1.88 – 1.75 (m, 1H), 1.03 (s, 9H), 1.00 (d, *J* = 6.8 Hz, 3H), 0.87 (d, *J* = 6.7 Hz, 3H); **$^{13}$C{$^1$H} NMR** (101 MHz, $CDCl_3$) δ 174.0, 154.0, 138.4, 130.8, 129.0, 127.9, 127.5, 126.3, 108.4, 105.6, 83.0, 47.1, 43.9, 31.9, 27.4, 21.2, 20.0, 13.1; **HRMS** (ESI-TOF) calcd for $C^{22}H^{31}N^{2}O^{3+}$ [M+H]$^+$ 371.2329, found 371.2333.

## IV. Limitations

**Fig. S1.** Low yielding substrates with no 2-position substitution on indoles/pyrroles.

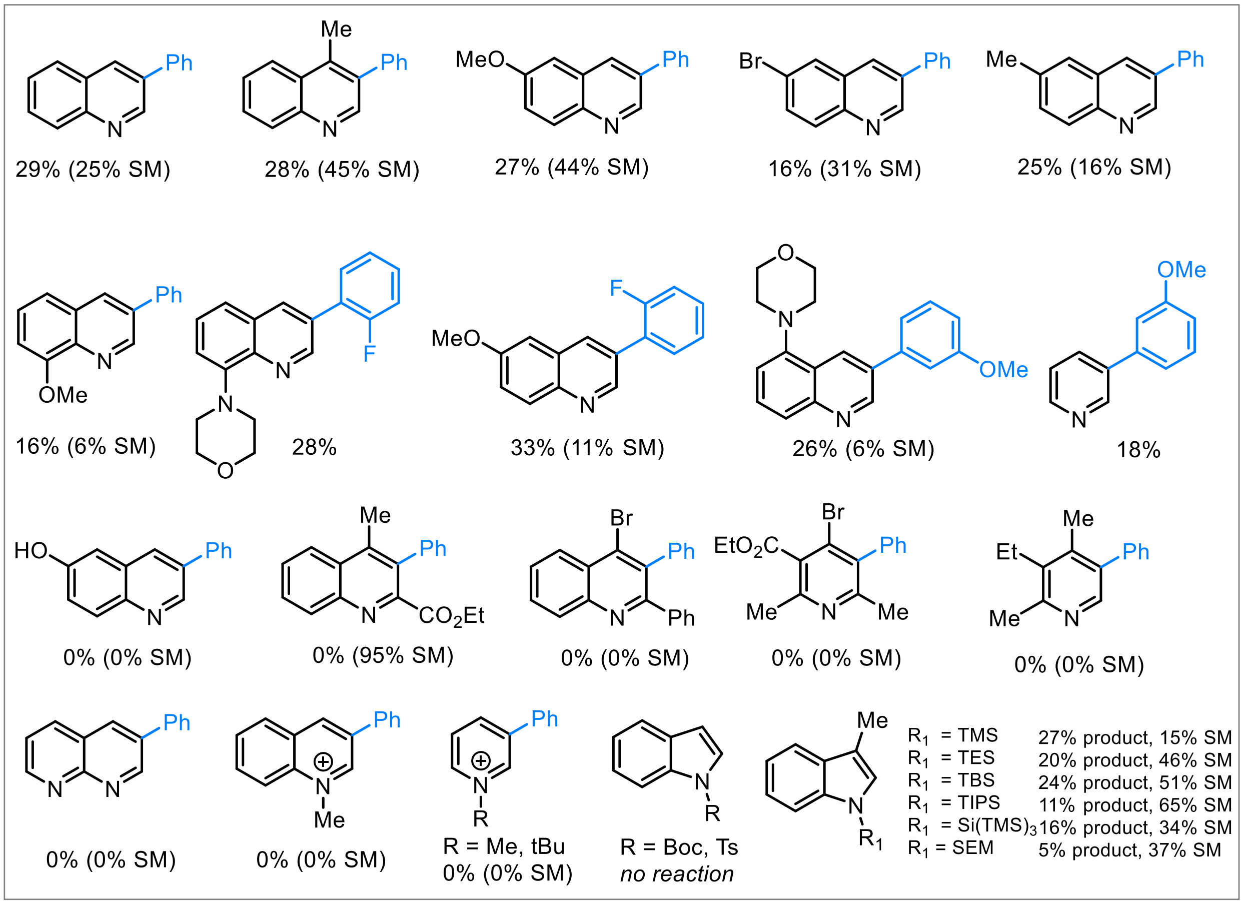


**Fig. S2.** Limitations of diazirines (A) electron rich aromatic halo diazirine were converted to corresponding aldehyde. (B) Aliphatic diazirines bearing α-hydrogens were isomerized to chloro-olefins (C) Aliphatic *tert*-alkyl diazirines underwent competitive dimerization.

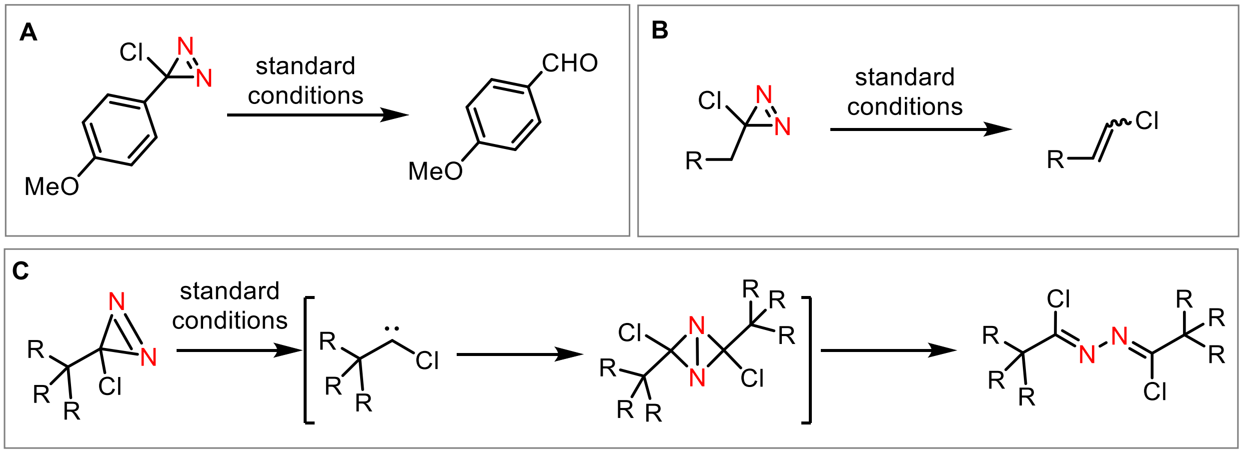

### V. Comparison with Ciamician-Denstedt Reaction

**i) Under standard conditions:**

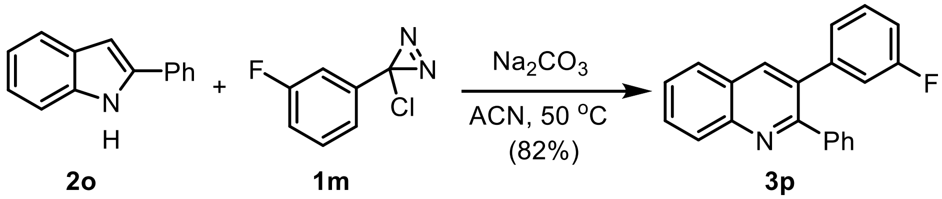


Following the general procedure II, product **3p** was isolated in 82% yield as a yellow solid from 2-phenyl indole **2o** and diazirine **1m**. **3p**: $^{1}$**H NMR** (400 MHz, $CDCl_3$) δ 8.21 (d, *J* = 8.5 Hz, 1H), 8.17 (s, 1H), 7.88 (dd, *J* = 8.2, 1.4 Hz, 1H), 7.76 (ddd, *J* = 8.4, 6.8, 1.4 Hz, 1H), 7.58 (td, *J* = 7.5, 6.9, 1.1 Hz, 1H), 7.49 – 7.40 (m, 2H), 7.30 (dd, *J* = 5.2, 1.9 Hz, 3H), 7.26 – 7.20 (m, 1H), 7.04 – 6.87 (m, 3H).

**ii) Under Ciamician-Denstedt followed by Suzuki coupling conditions:**

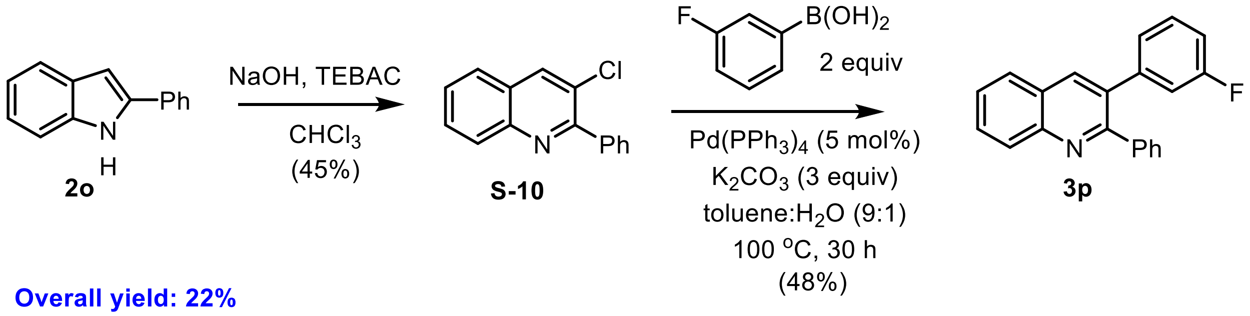


**Overall yield: 22%**

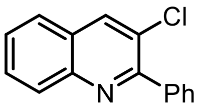

**S-10**

***Step I***- The reaction was performed according to reported literature procedure.[20] NaOH (104 mg, 2.6 mmol) in water (0.5 mL) was added to a vigorously stirred solution of 2-phynylindole **2o** (50 mg, 0.26 mmol, 1 eq) and benzyltriethylammonium chloride (10 mg, 26 μmol, 10 mol%) in chloroform (3 mL) under ice-cooling bath. The reaction mixture was stirred at 0 °C for 3 h and left overnight at room temperature. The aqueous layer was separated and extracted with chloroform (3 × 5 mL). The organic layers were dried over $MgSO_4$. After filtration and evaporation, the crude was purified by flash chromatography (10 % EtOAc/Hexanes) affording 3-chloro-2-phenyl quinoline **S-10** as a brown oil (28 mg, 116 μmol, 45%) and 18% of starting material **2o** was recovered. $R_f$ = 0.61 (20% Ethyl acetate/Hexanes). $^{1}$**H NMR** (400 MHz, $CDCl_3$) δ 8.19 (d, *J* = 0.8 Hz, 1H), 8.06 (dq, *J* = 8.5, 0.9 Hz, 1H), 7.74 – 7.67 (m, 3H), 7.64 (ddd, *J* = 8.5, 6.9, 1.5 Hz, 1H), 7.49 (ddd, *J* = 8.1, 6.9, 1.2 Hz, 1H), 7.43 – 7.38 (m, 3H). Spectroscopic data are in agreement with those in the literature.[21]

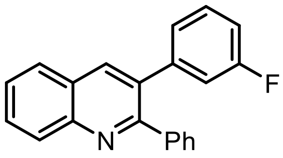

**3p**

***Step II***- To an oven dried 1 dram screw cap vial equipped with a stir bar and PTFE/white silicone septum was added quinoline **S-10** (28 mg, 0.116 mmol), 3-fluoroboronic acid (32.6 mg, 0.23 mmol), $Pd(PPh_3)_4$ (6.7 mg, 5.8 µmol) and $K_2CO_3$ (48 mg, 0.348 mmol). The vial was evacuated/backfilled with nitrogen twice, and then toluene (0.9 mL) and water (0.1 mL) (sparged with nitrogen for 15 min) were added via syringe. The vial was stirred vigorously (1000 rpm) at 100 °C. After 30 h, vial was cooled to room temperature, reaction was quenched by sat. $NH_4Cl$ (2 mL) and water (2 mL). The two phases were separated, and the aqueous layer was extracted with EtOAc (3 × 3 mL). The combined organic layers were dried over anhydrous $Na_2SO_4$, filtered, and concentrated under reduced pressure. The obtained residue was automated silica gel column chromatography to afford quinoline **3p** (16.8 mg, 56 µmol, 48% yield) as yellow solid, while 21% of starting material (6 mg, 25 µmol) was recovered back.

## VI. Mechanistic Experiments

### VIA. Effect of tetrabutyl ammonium chloride

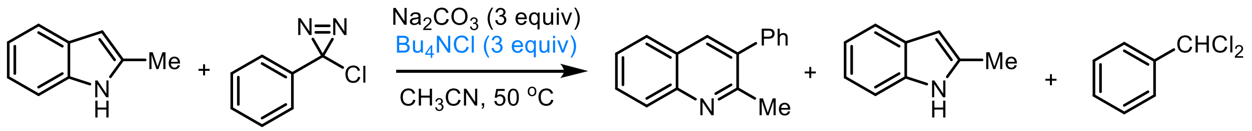


**2a** **1a** **3a**, 21% **2a**, 69% **S-11**, 11% (*w.r.t* 3 equiv **1a**)

Without $Bu_4NCl$, yield of **3a** is 64%

Oven dried 1-dram screw capped vial was degassed and charged with 2-methylindole **2a** (10.0 mg, 76.2 µmol, 1.0 equiv), diazirine **1a** (35.0 mg, 229 µmol, 3.0 equiv), $Na_2CO_3$ (24.0 mg, 229 µmol, 3.0 equiv), $Bu_4NCl$ (63.0 mg, 229 µmol, 3.0 equiv) and 1 mL of dry acetonitrile. This mixture was then allowed to stir at 50 °C for 12 h and then allowed to cool to 25 °C. The reaction mixture was quenched by the addition of saturated aqueous $NH_4Cl$ solution (1 mL), and the two phases were separated. The aqueous layer was extracted with EtOAc (3 × 3 mL), and the combined organic layers were dried over anhydrous $Na_2SO_4$, filtered, and concentrated under reduced pressure. Crude NMR showed the presence of quinoline **3a** (21%), starting material **2a** (69%) and benzal chloride **S-11** 11% (*w.r.t.* 3 equiv. of diazirine **1a**).

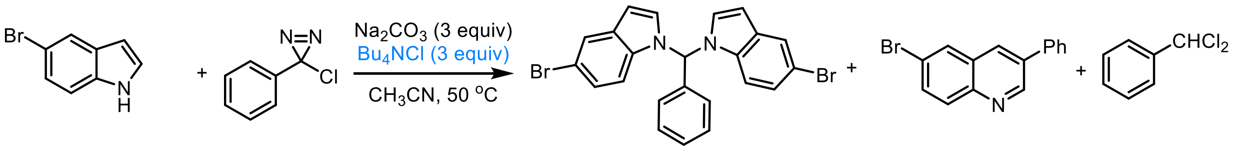


**S-12** **1a** **S-13**, 67% (52% isolated) **S-12**, <3% **S-11**, 34% (*w.r.t* 3 equiv **1a**)

Without $Bu_4NCl$, yield of **S-12** is 16%

Oven dried 1-dram screw capped vial was degassed and charged with 5-bromoindole **S-11** (10.0 mg, 47 µmol, 1.0 equiv), diazirine **1a** (22.0 mg, 143 µmol, 3.0 equiv), $Na_2CO_3$ (15.0 mg, 143 µmol, 3.0 equiv), $Bu_4NCl$ (39.7 mg, 143 µmol, 3.0 equiv) and 1 mL of dry acetonitrile. This mixture was then allowed to stir at 50 °C for 12 h and then allowed to cool to 25 °C. The reaction mixture was quenched by the addition of saturated aqueous $NH_4Cl$ solution (1 mL), and the two phases were separated. The aqueous layer was extracted with EtOAc (3 × 3 mL), and the combined organic layers were dried over anhydrous $Na_2SO_4$, filtered, and concentrated under reduced pressure. Crude NMR showed the presence of **S-13** (67%), quinoline **S-12** (<3%), and benzal chloride **S-11**, 34% (*w.r.t.* 3 equiv. of diazirine **1a**). Purification of crude material on automated column chromatography afforded **S-13** (6.0 mg, 13 µmol, 52%) as a yellow viscous liquid.

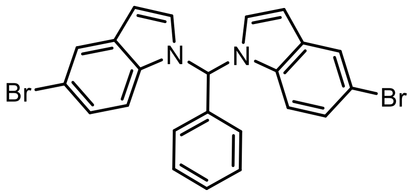


**S-13**

**1,1'-(phenylmethylene)bis(5-bromo-1*H*-indole) (S-13) BD1-153:** **$^1$H NMR** (400 MHz, $CDCl_3$) δ 7.84 (s, 1H), 7.78 (d, *J* = 1.9 Hz, 2H), 7.44 – 7.37 (m, 3H), 7.24 (dd, *J* = 8.7, 1.9 Hz, 2H), 7.11 – 7.01 (m, 4H), 6.73 (d, *J* = 3.4 Hz, 2H), 6.47 (dd, *J* = 3.4, 0.8 Hz, 2H); **$^{13}$C{$^1$H} NMR** (101 MHz, $CDCl_3$) δ 135.7, 134.7, 131.0, 129.9, 129.5, 127.3, 126.8, 125.5, 124.0, 114.1, 111.1, 102.9, 69.6; **HRMS** (ESI-TOF) calcd for $C_{15}H_{11}BrN^+$ $[M-C_8H_5BrN]^+$ 284.0069, found 284.0069.

## VIB. Effect of addition of quinoline

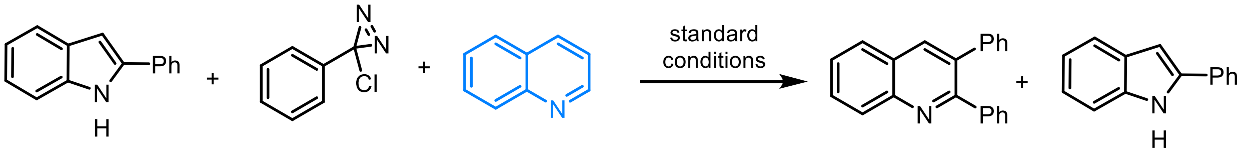


**2o** (1 equiv) **1a** (3 equiv) (3 equiv) **3o**, 15% **2o**, 38%

Without quinoline, yield of **3o** is 68%

Oven dried 1-dram screw capped vial was degassed and charged with 2-phenylindole **2a** (10.0 mg, 52 μmol, 1.0 equiv), diazirine **1a** (24 mg, 155 μmol, 3.0 equiv), $Na_2CO_3$ (16 mg, 155 μmol, 3.0 equiv), quinoline (20.0 mg, 155 μmol, 3.0 equiv) and 1 mL of dry acetonitrile. This mixture was then allowed to stir at 50 °C for 12 h and then allowed to cool to 25 °C. The reaction mixture was quenched by the addition of saturated aqueous $NH_4Cl$ solution (1 mL), and the two phases were separated. The aqueous layer was extracted with EtOAc (3 × 3 mL), and the combined organic layers were dried over anhydrous $Na_2SO_4$, filtered, and concentrated under reduced pressure. Crude NMR showed the presence of 2,3-diphenylquinoline **3o** (15%) and starting material **2o** (38%).

## VII.     X-Ray-derived ORTEP of 3z

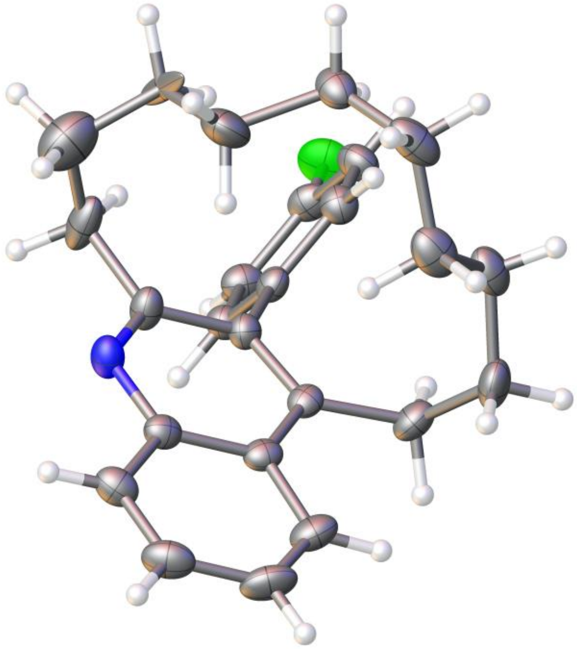

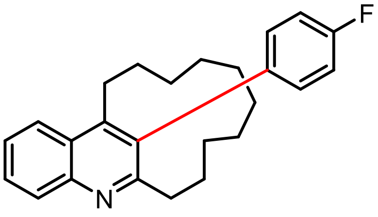


**3z**

**ORTEP drawing of 3z**

Cystallographic data is available free of charge from the Cambridge Crystallographic Data Centre (https://summary.ccdc.cam.ac.uk/structure-summary-form under deposition number xxxxx).

## Datablock: mo_0896_levin_0m

Bond precision: C-C = 0.0023 A Wavelength=0.71073

| | | | |
|---|---|---|---|
| Cell: | a=9.2870(4) | b=14.2383(6) | c=14.6599(7) |
| | alpha=90 | beta=91.126(1) | gamma=90 |
| Temperature: | 100 K | | |

| | Calculated | Reported |
|---|---|---|
| Volume | 1938.12(15) | 1938.12(15) |
| Space group | P 21/n | P 1 21/n 1 |
| Hall group | -P 2yn | -P 2yn |
| Moiety formula | C25 H28 F N | C25 H28 F N |
| Sum formula | C25 H28 F N | C25 H28 F N |
| Mr | 361.48 | 361.48 |
| Dx,g cm-3 | 1.239 | 1.239 |
| Z | 4 | 4 |
| Mu (mm-1) | 0.078 | 0.078 |
| F000 | 776.0 | 776.0 |
| F000' | 776.31 | |
| h,k,lmax | 12,18,19 | 12,18,19 |
| Nref | 4693 | 4202 |
| Tmin,Tmax | 0.979,0.992 | 0.698,0.746 |
| Tmin' | 0.966 | |

Correction method= # Reported T Limits: Tmin=0.698 Tmax=0.746
AbsCorr = MULTI-SCAN

Data completeness= 0.895 Theta(max)= 28.040

R(reflections)= 0.0549( 2988) wR2(reflections)= 0.1305( 4202)

S = 1.034 Npar= 281

**References:**

1. Majumder, S.; Gipson, K. R.; Odom A. L. A Multicomponent Coupling Sequence for Direct Access to Substituted Quinolines *Org. Lett.* **2009**, *11*, 4720-4723 (3a)

2. Tseng, C.-H.; Lin, C.-K.; Chen, Y.-L.; Hsu, C.-Y. Wu, H.-N.; Tseng, C.-K.; Lee, J.-C. Synthesis, antiproliferative and anti-dengue virus evaluations of 2-aroyl-3-arylquinoline derivatives *Eur. J. Med. Chem.* **2014**, *79*, 66-76. (3b: )

3. McDaniel, T. J.; Lansdell, T. A.; Dissanayake, A. A.; Azevedo, L. M.; Claes, J.; Odom, A. L.; Tepe, J. J. Substituted quinolines as noncovalent proteasome inhibitor *Bioorg. Med. Chem.* **2016**, *24*, 2441–2450. (**3j**)

4. Kong, L.; Yu, S.; Zhou, X.; Li, X. Redox-Neutral Couplings between Amides and Alkynes via Cobalt(III)-Catalyzed C-H Activation *Org. Lett.* **2016**, *18*, 588–591. (**3l:**)

5. Saunthwal, R. K.; Patel, M.; Verma, A. K. Metal- and Protection-Free [4 + 2] Cycloadditions of Alkynes with Azadienes: Assembly of Functionalized Quinolines *Org. Lett.* **2016**, *18*, 2200−2203. **(3o:)**

6. Wei, Y.; Yoshikai, N. Modular Pyridine Synthesis from Oximes and Enals through Synergistic Copper/Iminium Catalysis *J. Am. Chem. Soc.* **2013**, *135*, 3756-3759. ( **5b:**)

7. Wang, K.; Meng, L.-G.; Wang, L. Visible-Light-Promoted [2 + 2 + 2] Cyclization of Alkynes with Nitriles to Pyridines Using Pyrylium Salts as Photoredox Catalysts *Org. Lett.* **2017**, *19*, 1958−1961. (**5f: Xx:**)

8. Parthasarathy, K.; Jeganmohan, M.; Cheng, C.-H. Rhodium-Catalyzed One-Pot Synthesis of Substituted Pyridine Derivatives from α, β-Unsaturated Ketoximes and Alkynes *Org. Lett.* **2008**, *10*, 325–328. (**5f': Xx'**:)

9. Zhu, T.; Li, X.; Chang, H.; Gao, W.; Wei, W. Nano-Pd/$Al(OH)_3$-Catalyzed Suzuki–Miyaura Coupling Reaction of Potassium Aryl- and Heteroaryltrifluoroborates with Electrophiles in Alcohols *Synlett* **2016**, *27*, 880-887. (**5l:)**

10. Craig, D.; Paina, F.; Smith, S. C. Double decarboxylative Claisen rearrangement reactions: microwave-assisted de novo synthesis of pyridines *Chem. Commun.* **2008**, 3408–3410. (**5l':** Xx':)

11. Wang, K.; Meng, L.-G. Wang, L. Visible-Light-Promoted [2 + 2 + 2] Cyclization of Alkynes with Nitriles to Pyridines Using Pyrylium Salts as Photoredox Catalysts *Org. Lett.* **2017**, *19*, 1958–1961. (**5n:)**

12. Huang, Z.; Zhang, W.-X.; Xi, Z. Lewis Acid-Promoted Ring-Contraction of 2,4,6,8-Tetrasubstituted 1,5-Diazacyclooctatetraenes to 2,4,6-Trisubstituted Pyridines *Org. Lett.* **2018**, *20*, 485–488. (**5t': Xx':)**

13. Ohashi, M.; Takeda, I.; Ikawa, M.; Ogoshi, S. Nickel-Catalyzed Dehydrogenative [4 + 2] Cycloaddition of 1,3-Dienes with Nitriles *J. Am. Chem. Soc.* **2011**, *133*, 18018–18021. (**5v')**

14. Błachut, D.; Czarnocki, Z.; Wojtasiewicz, K. A Suzuki-Miyaura Approach to a Series of Forensically Relevant Pyridines *Synthesis* **2006**, *17*, 2855-2864. (**5x)**

15. a) Graham, W. H. The Halogenation of Amidines. I. Synthesis of 3-Halo- and Other Negatively Substituted Diazirines *J. Am. Chem. Soc.* **1965**, *87*, 4396-4397. b) Moss, R. A.; Tian, J. Hammett studies of aryldichloromethide carbanion reactions *Org. Lett.* **2006**, *8*, 1245-1247. (general procedure, 1a, 1c, 1l)

16. Baird, M. S.; Bruce, I. Generation and trapping of 2- and 3-pyridylhalocarbenes from diazirines *J. Chem. Research (S)* **1989**, *12*, 374-375. (1b)

17. Martinu, T.; Dailey, W. P. Reactivity of 1-Chloro-3-phenyldiazirines *J. Org. Chem.* **2006**, *71*, 5012-5015. (1n)

18. Krasniqi, B.; Geerts, K.; Dehaen, W. General Transition Metal-Free Synthesis of NH-Pyrroles from Secondary Alcohols and 2-Aminoalcohols *J. Org. Chem.* **2019**, *84*, 5027−5034. (4e:)

19. Wang, H.-Y.; Mueller, D. S.; Sachwani, R. M.; Kapadia, R.; Londino, H. N.; Anderson, L. L. Regioselective Synthesis of 2,3,4- or 2,3,5-Trisubstituted Pyrroles via [3,3] or [1,3] Rearrangements of O-Vinyl Oximes *J. Org. Chem.* **2011**, *76*, 3203–3221. (4h:)

20. The reaction was performed using literature reported procedure as follow: Rousseaux, S.; Davi, M.; Sofack-Kreutzer, J.; Pierre, C.; Kefalidis, C. E.; Clot, E.; Fagnou, K.; Baudoin O. *J. Am. Chem. Soc.* **2010**, *132*, 10706–10716.

21. Akhil Mahajan, Anju Arya, and Tejpal Singh Chundawat Green synthesis of silver nanoparticles using green alga (Chlorella vulgaris) and its application for synthesis of quinolines derivatives *Synth. Commun.* **2019**, *49*, 1926–1937.

# VII. $^{1}$H, $^{13}$C, $^{19}$F and 2D NMR Spectra

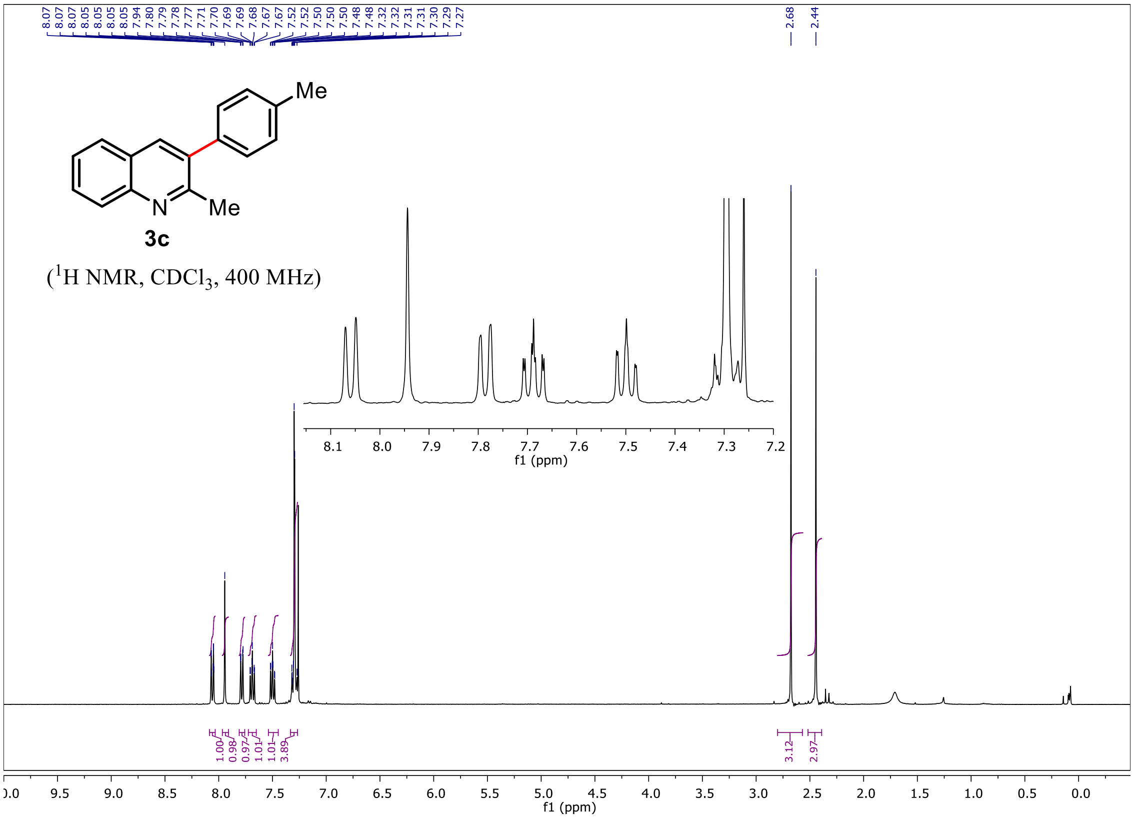


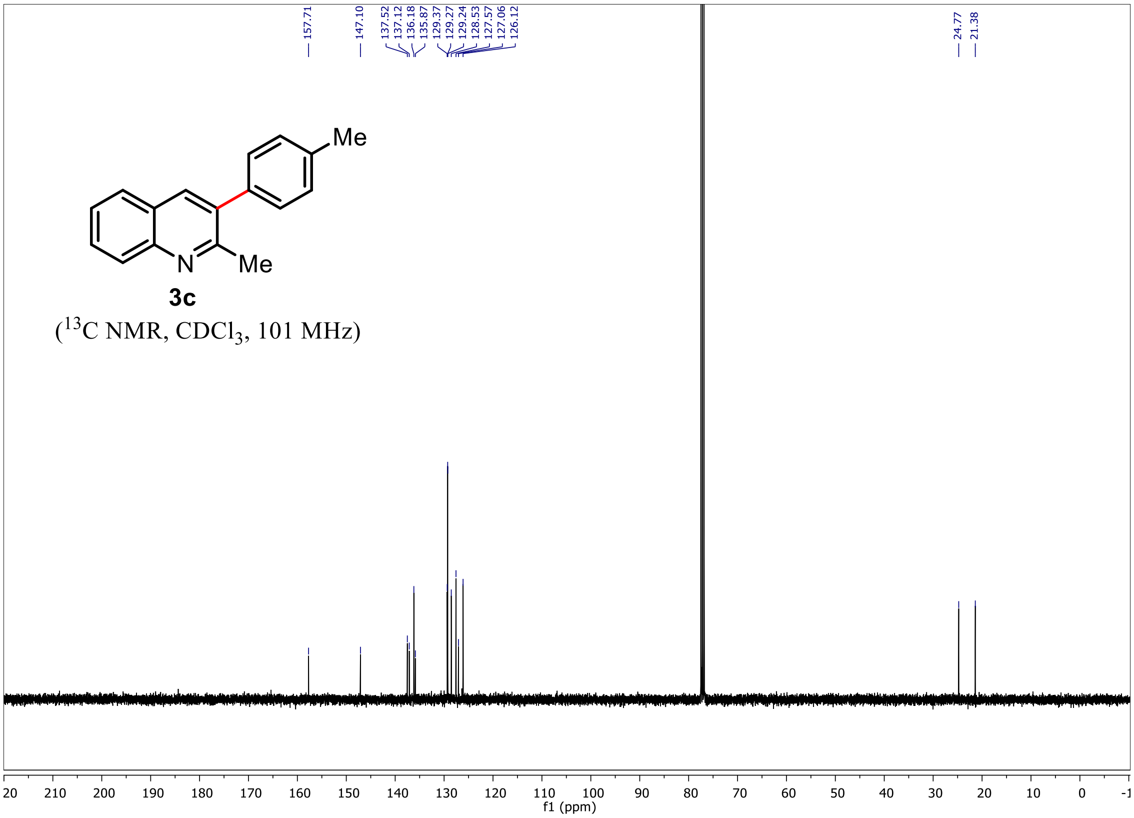

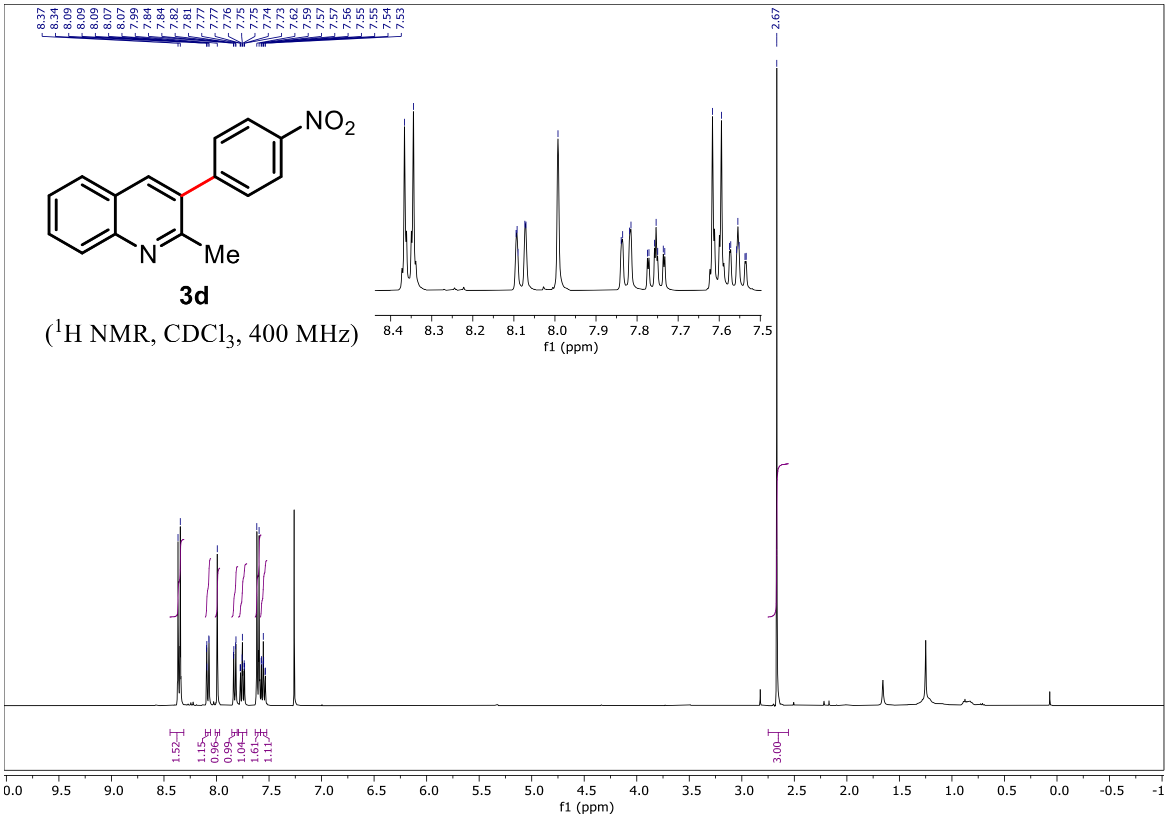

NO2
N
Me
3d
(1H NMR, CDCl3, 400 MHz)
f1 (ppm)


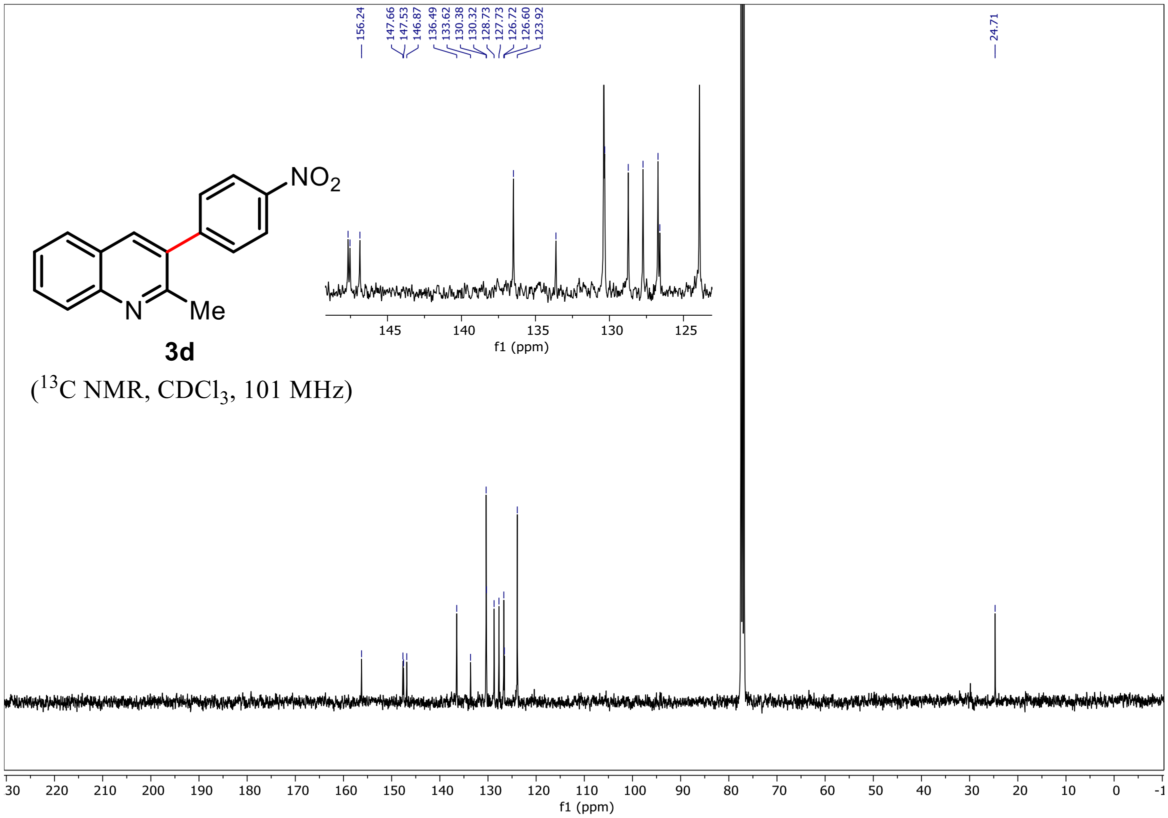

NO2
N
Me
3d
(13C NMR, CDCl3, 101 MHz)
f1 (ppm)

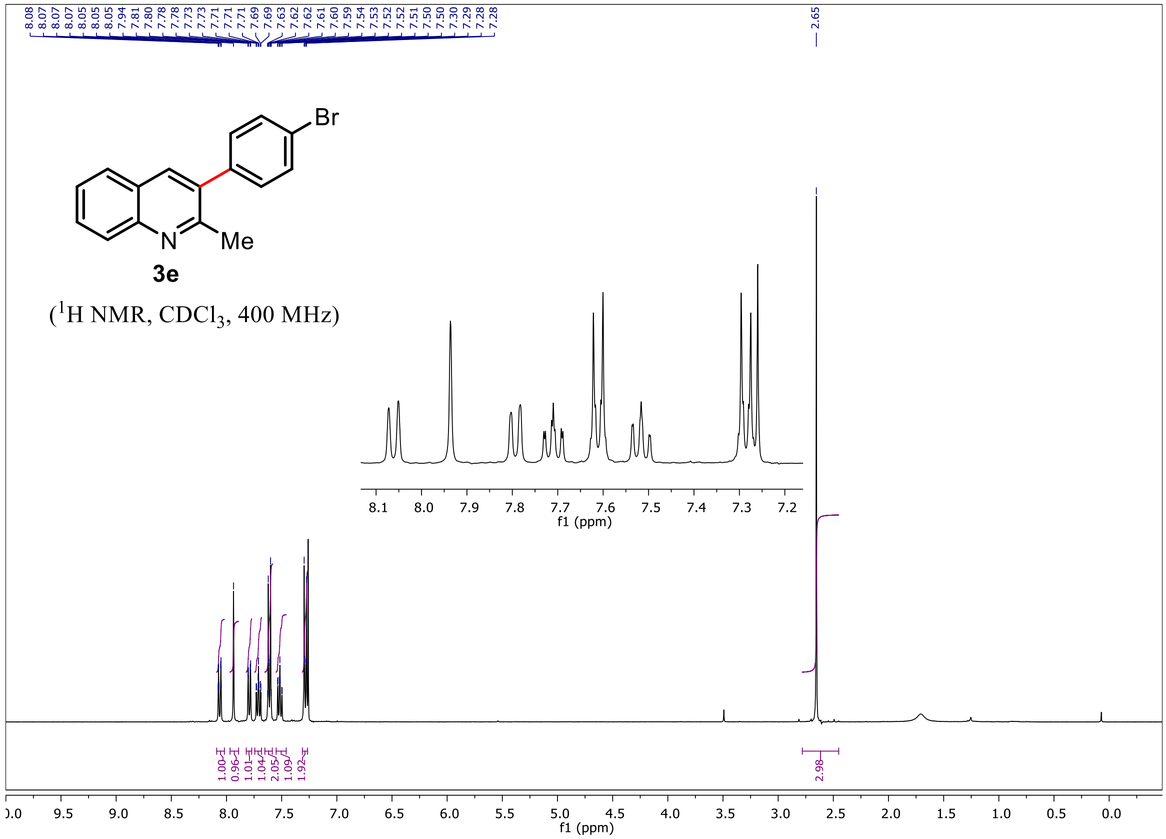

8.08 8.07 8.07 8.05 8.05 8.05 7.94 7.81 7.80 7.78 7.78 7.73 7.73 7.71 7.71 7.71 7.69 7.69 7.63 7.62 7.62 7.61 7.60 7.59 7.54 7.53 7.52 7.52 7.51 7.50 7.50 7.30 7.29 7.28 7.28
2.65
Br
N
Me
3e
($^1$H NMR, CDCl$_3$, 400 MHz)
1.00 0.96 1.01 1.04 2.05 1.09 1.92
2.98
f1 (ppm)


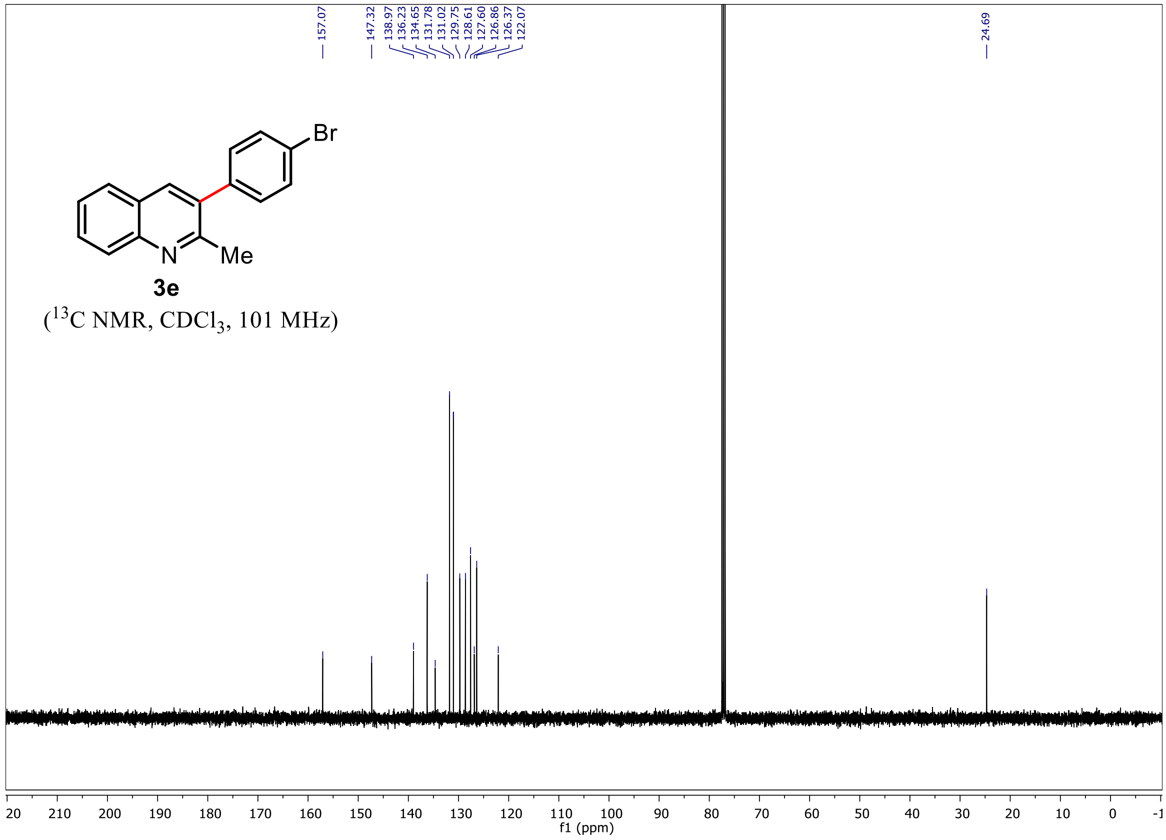

157.07
147.32 138.97 136.23 134.65 131.78 131.02 129.75 128.61 127.60 126.86 126.37 122.07
24.69
Br
N
Me
3e
($^{13}$C NMR, CDCl$_3$, 101 MHz)
f1 (ppm)

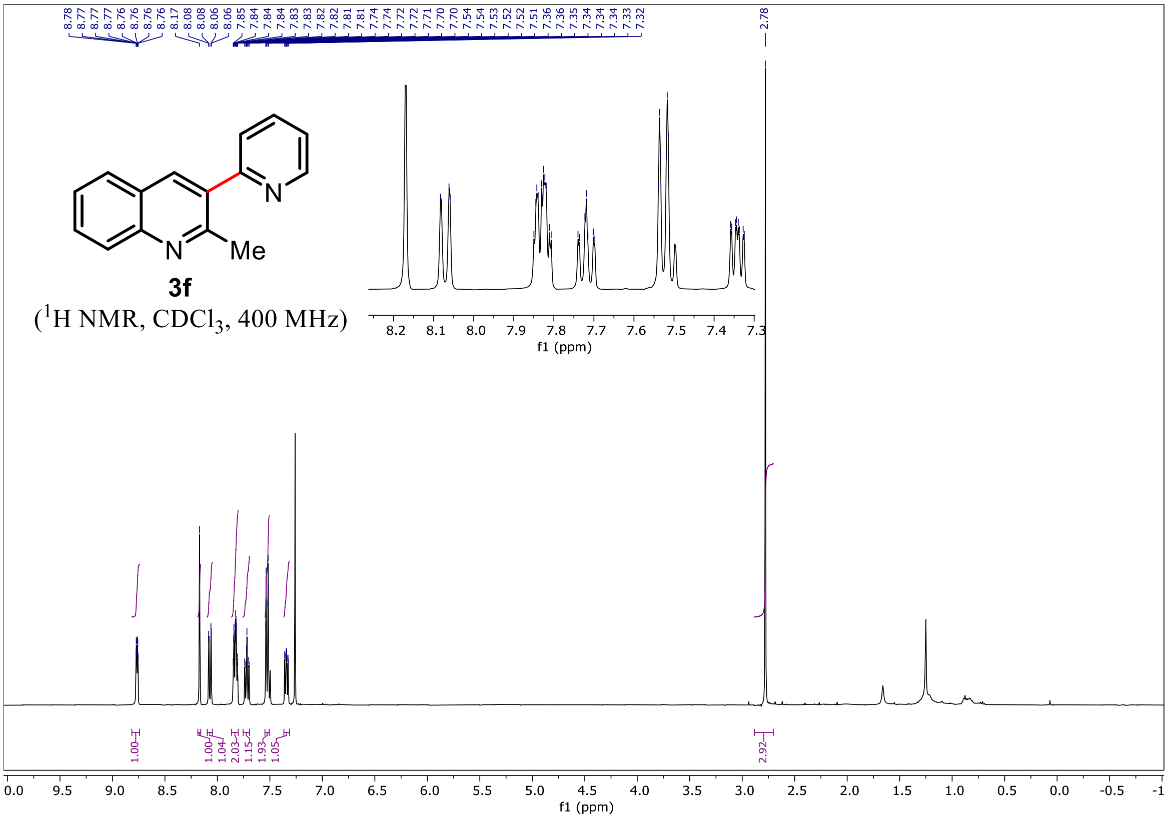
3f
(1H NMR, CDCl3, 400 MHz)
f1 (ppm)

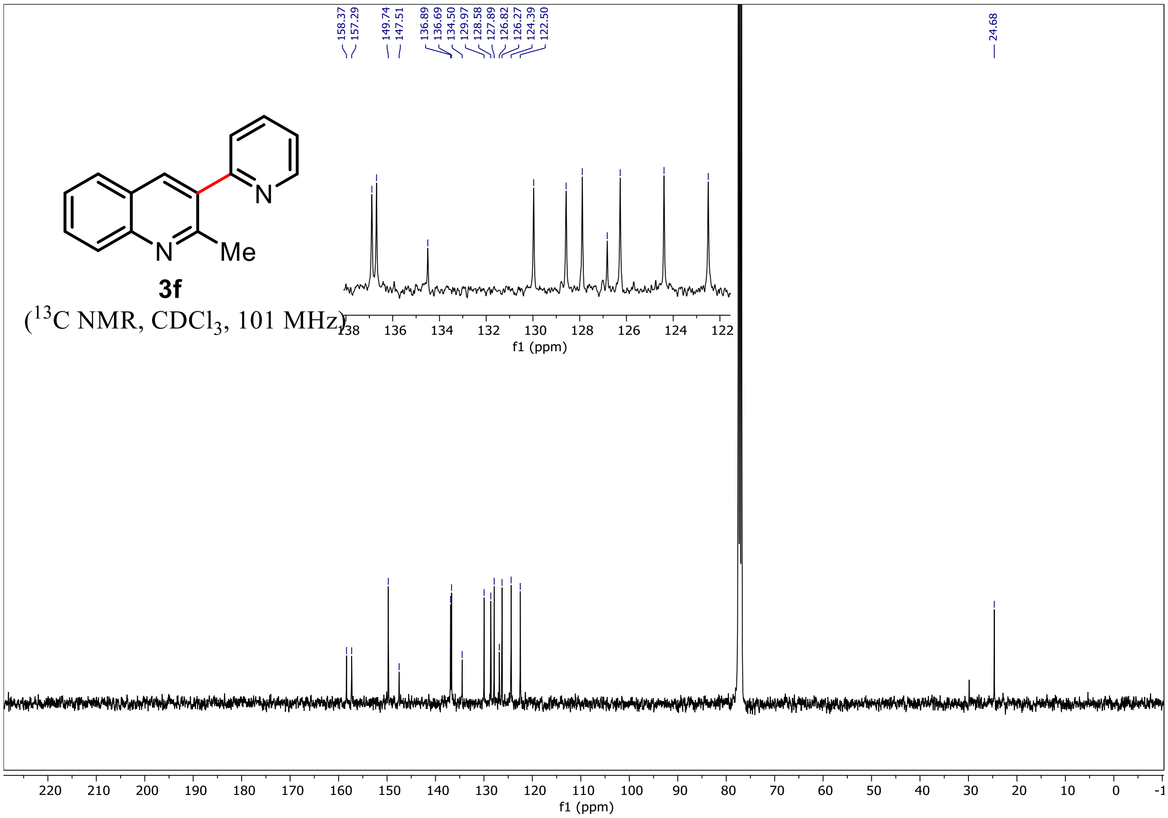
3f
(13C NMR, CDCl3, 101 MHz)
f1 (ppm)

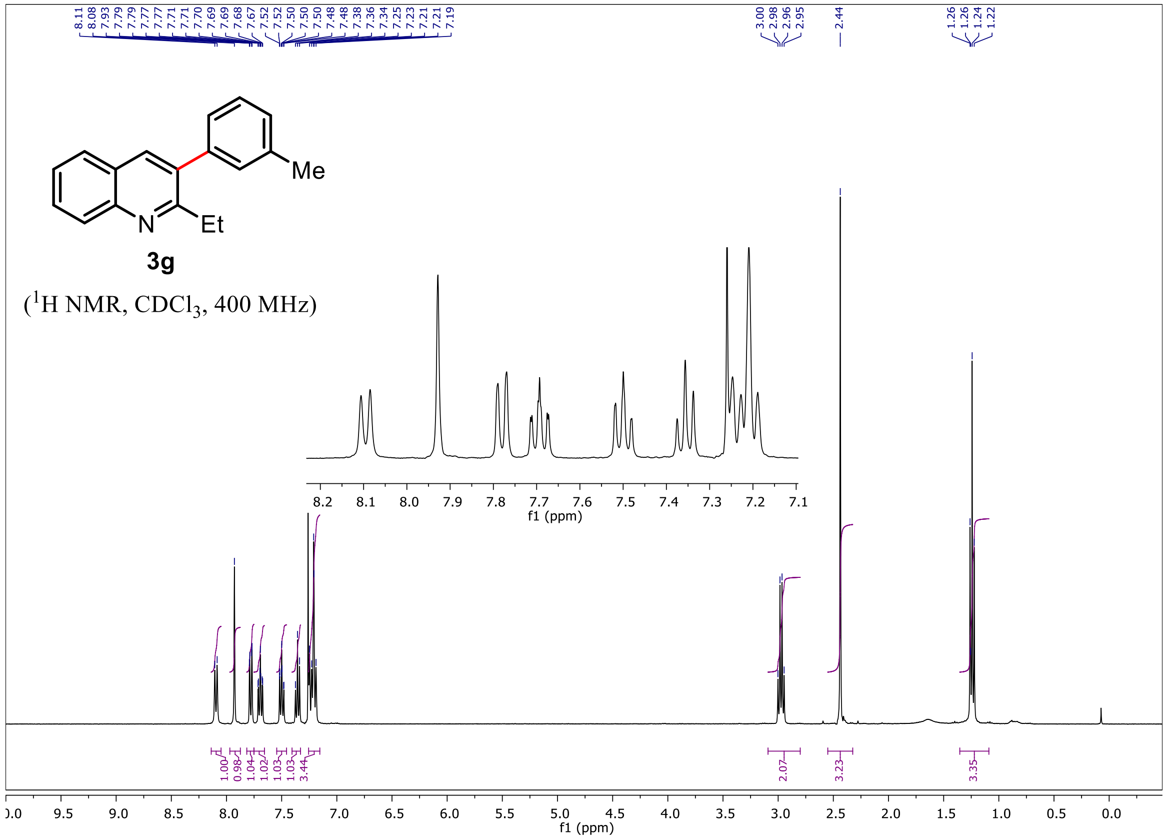
Me
N
Et
3g
(1H NMR, CDCl3, 400 MHz)
f1 (ppm)

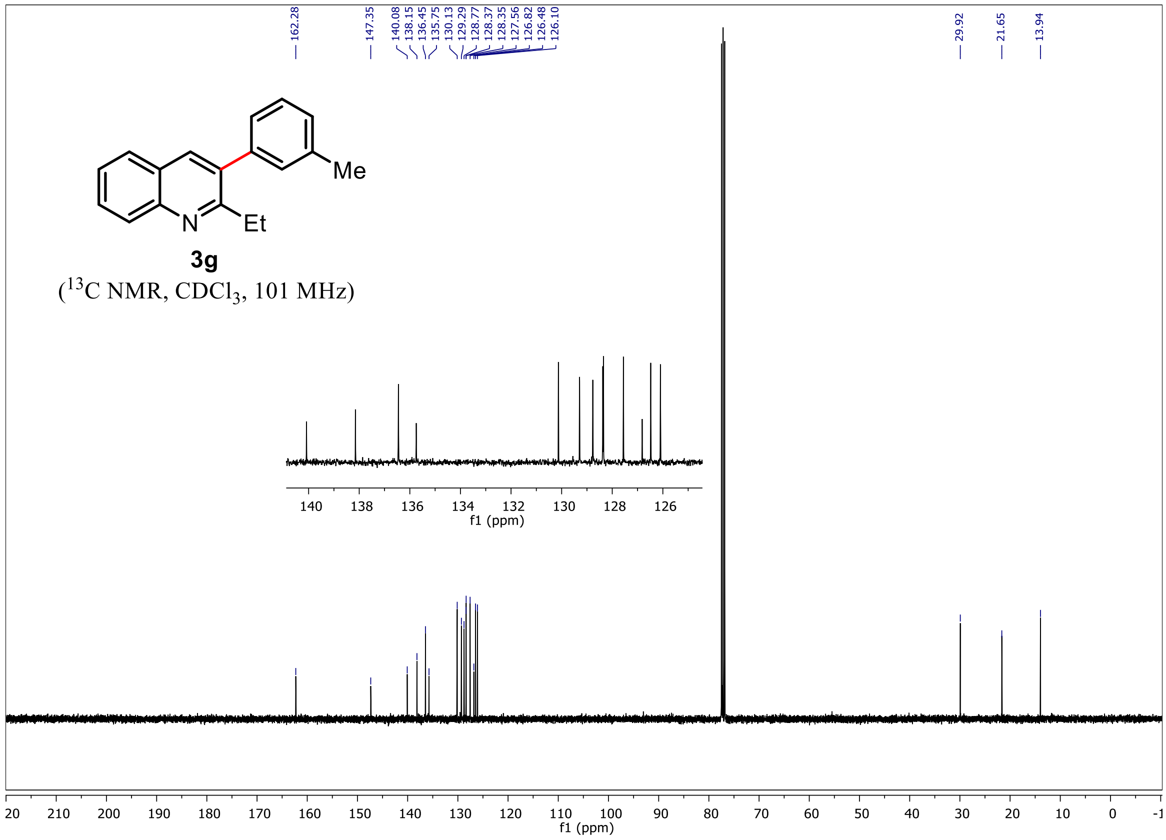
Me
N
Et
3g
(13C NMR, CDCl3, 101 MHz)
f1 (ppm)

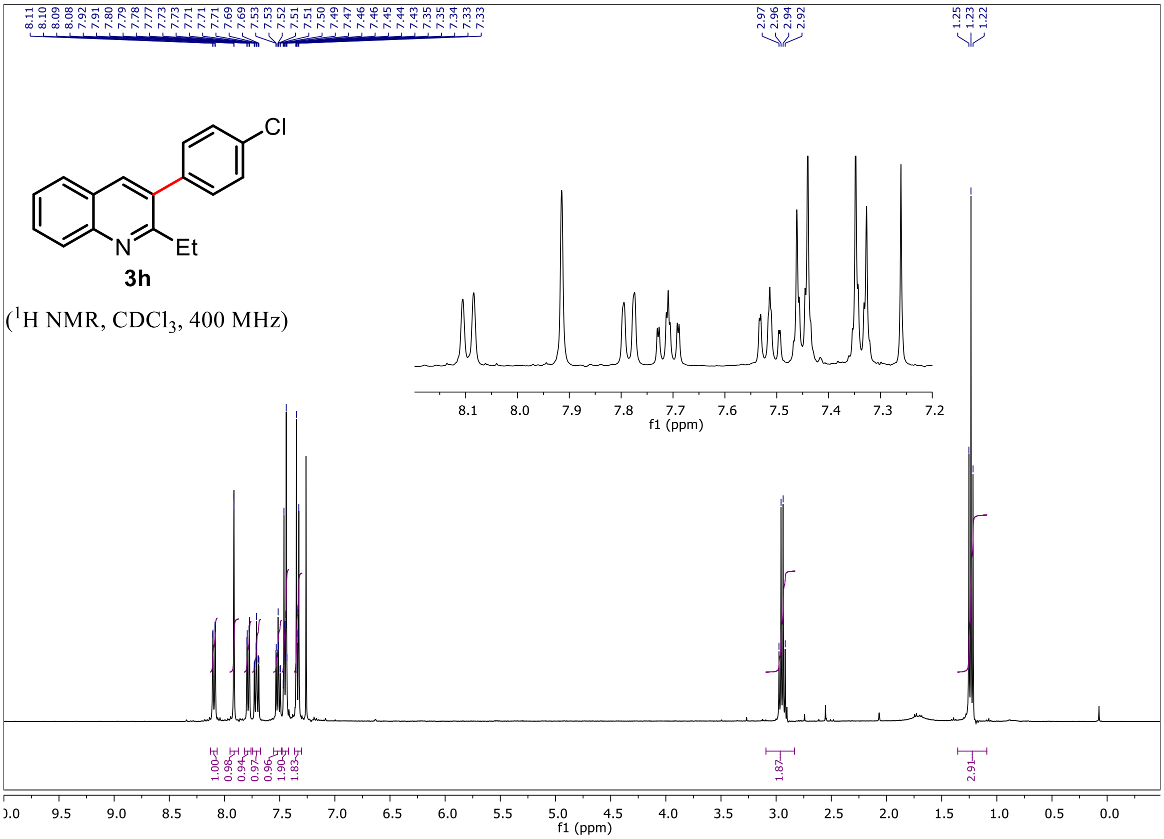

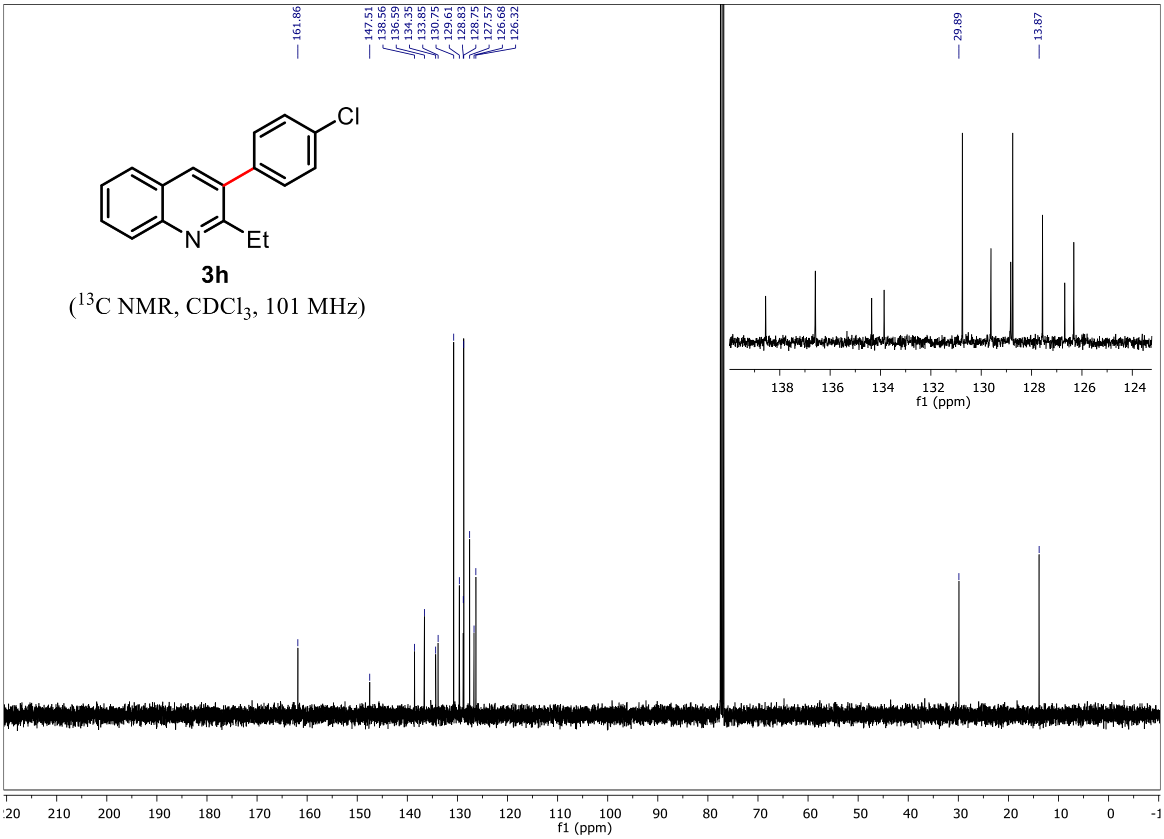

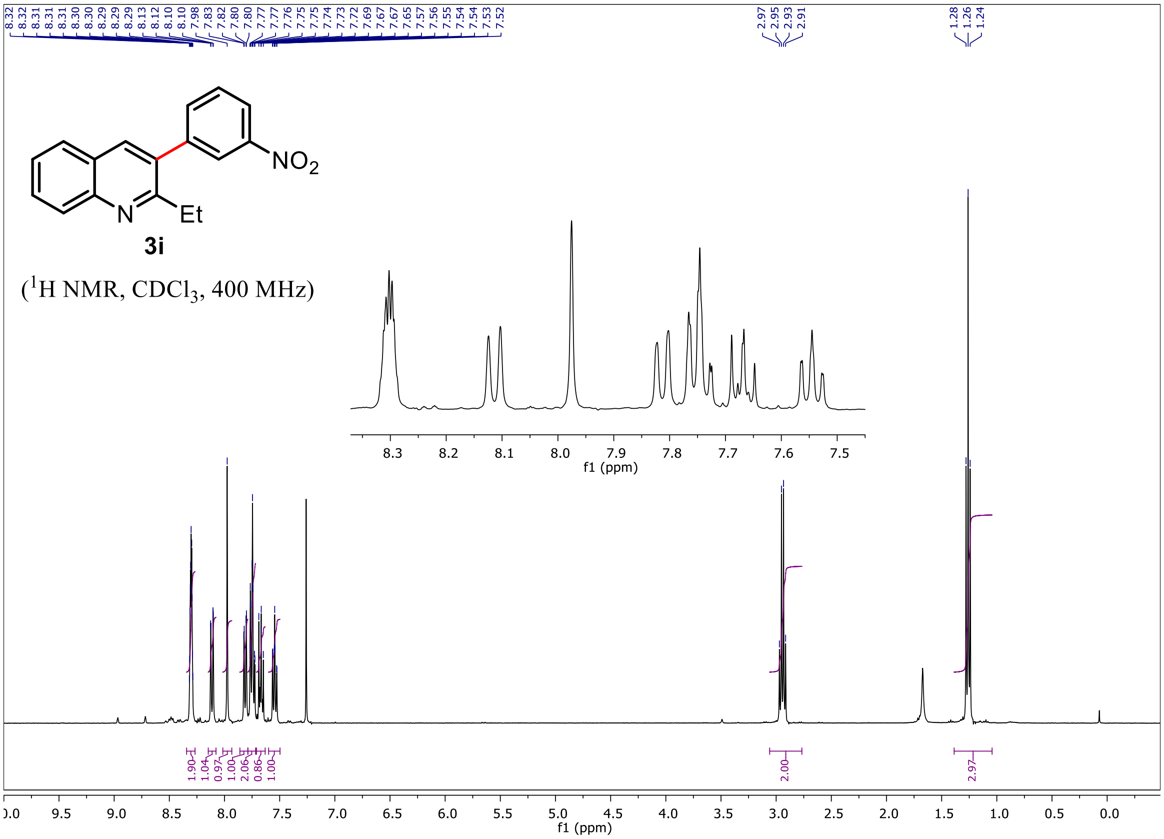
8.32 8.32 8.31 8.31 8.30 8.29 8.29 8.13 8.12 8.10 7.98 7.83 7.82 7.80 7.80 7.77 7.77 7.76 7.75 7.75 7.74 7.73 7.72 7.69 7.67 7.67 7.65 7.57 7.56 7.55 7.54 7.54 7.53 7.52
2.97 2.95 2.93 2.91
1.28 1.26 1.24
NO2
N
Et
3i
(1H NMR, CDCl3, 400 MHz)
1.90 1.04 0.97 1.00 2.06 0.86 1.00
2.00
2.97
f1 (ppm)

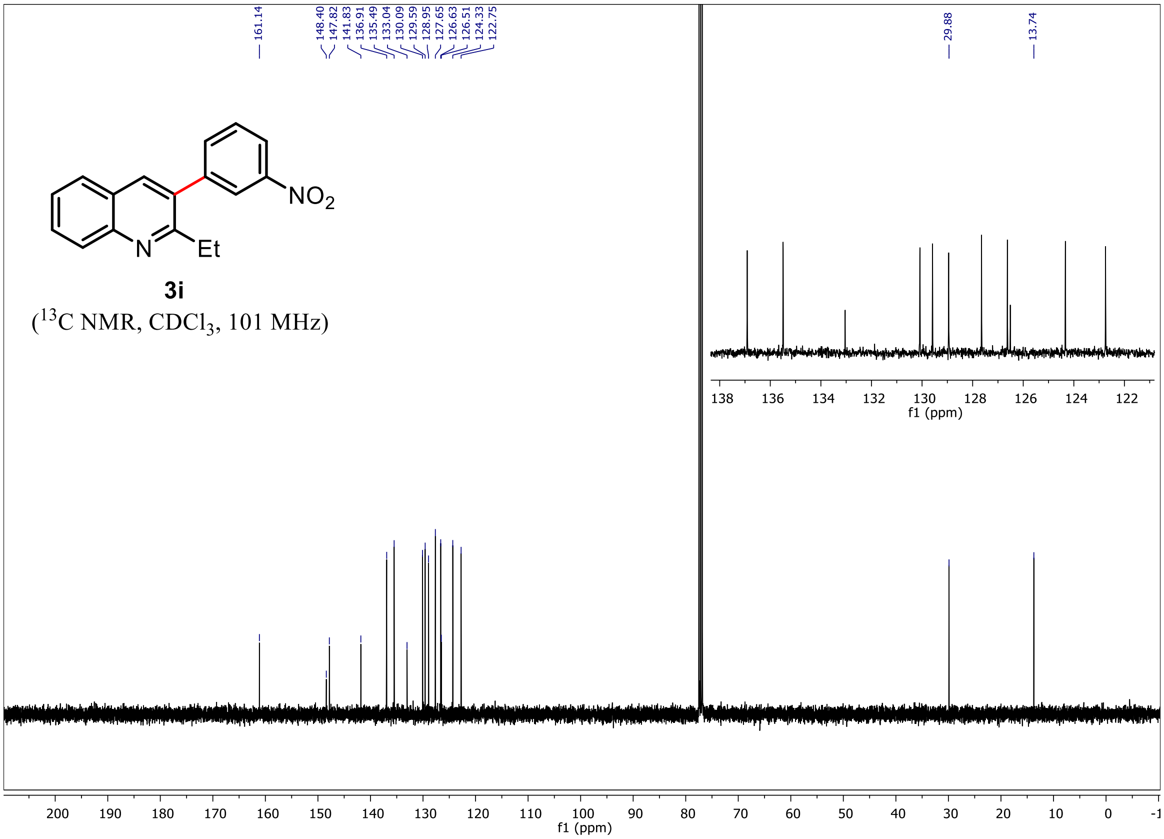
161.14
148.40 147.82 141.83 136.91 135.49 133.04 130.09 129.59 128.95 127.65 126.63 126.51 124.33 122.75
29.88
13.74
NO2
N
Et
3i
(13C NMR, CDCl3, 101 MHz)
f1 (ppm)

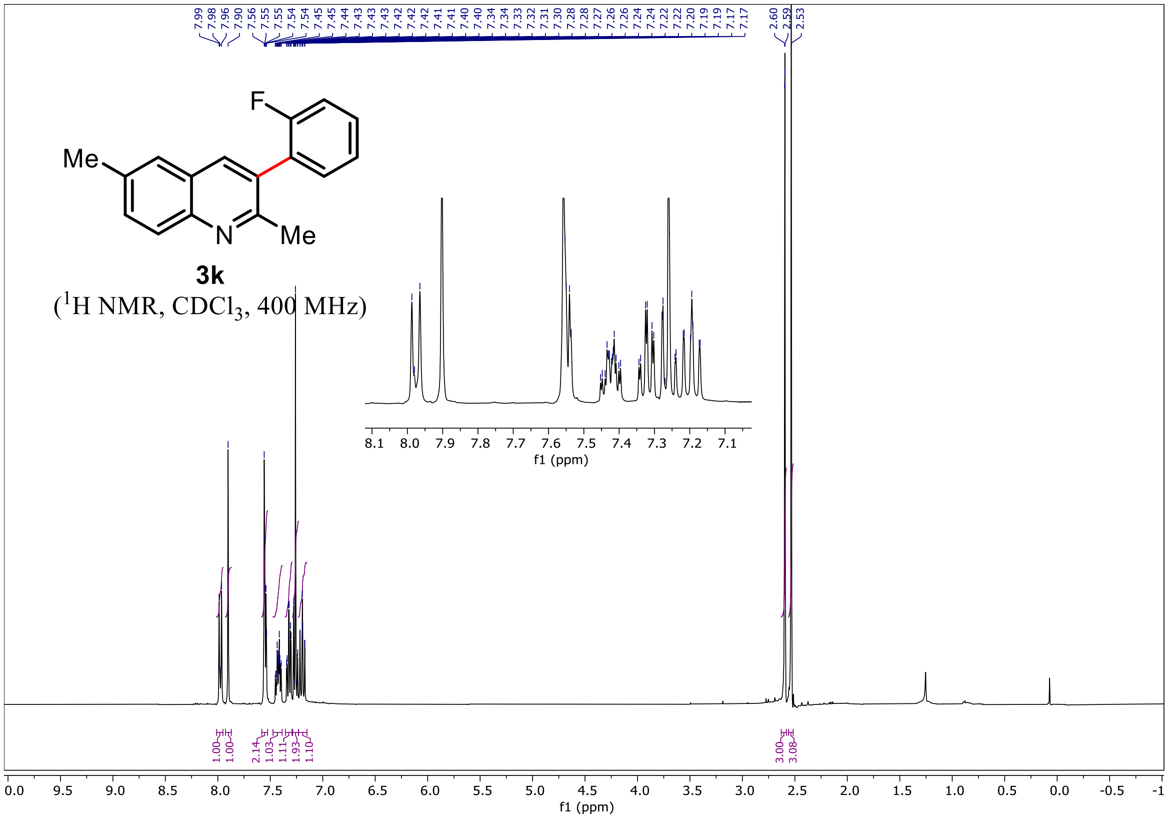
Me
F
N
Me
3k
(1H NMR, CDCl3, 400 MHz)
f1 (ppm)

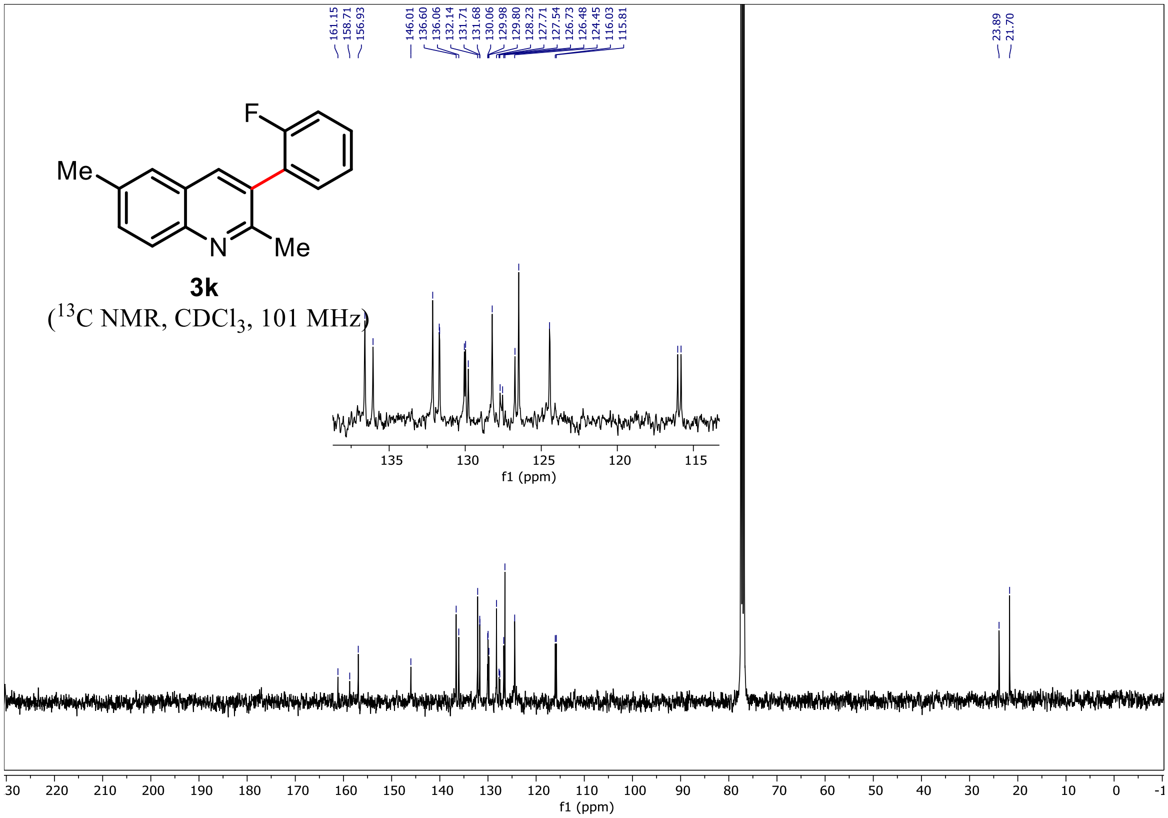
Me
F
N
Me
3k
(13C NMR, CDCl3, 101 MHz)
f1 (ppm)

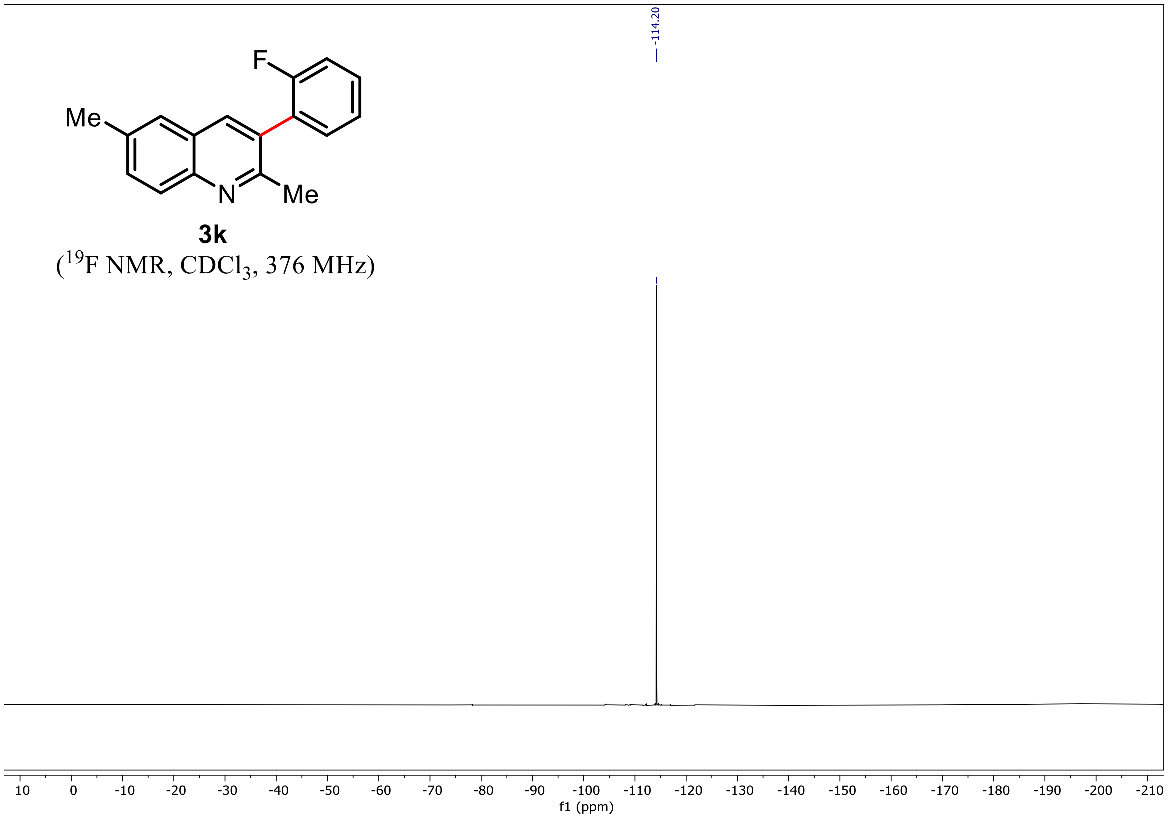

**3k**

($^{19}$F NMR, $CDCl_3$, 376 MHz)

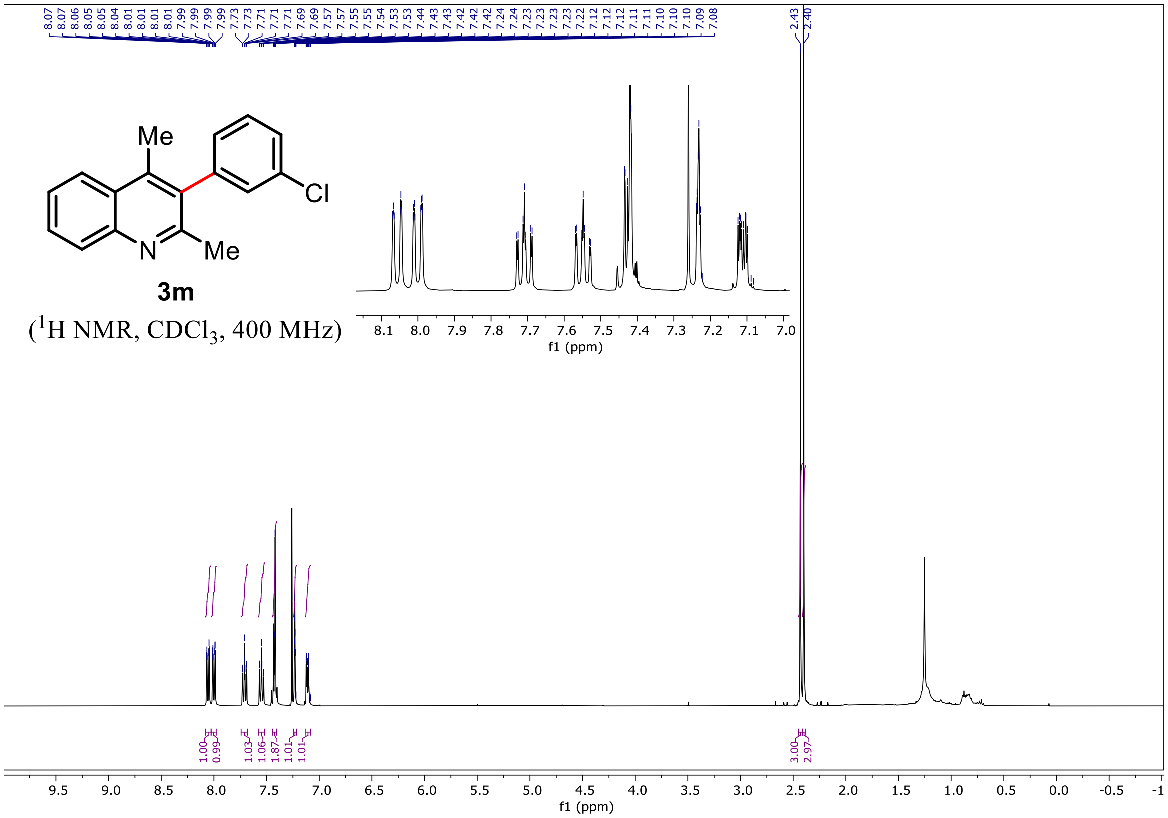

**3m**

($^{1}$H NMR, $CDCl_3$, 400 MHz)

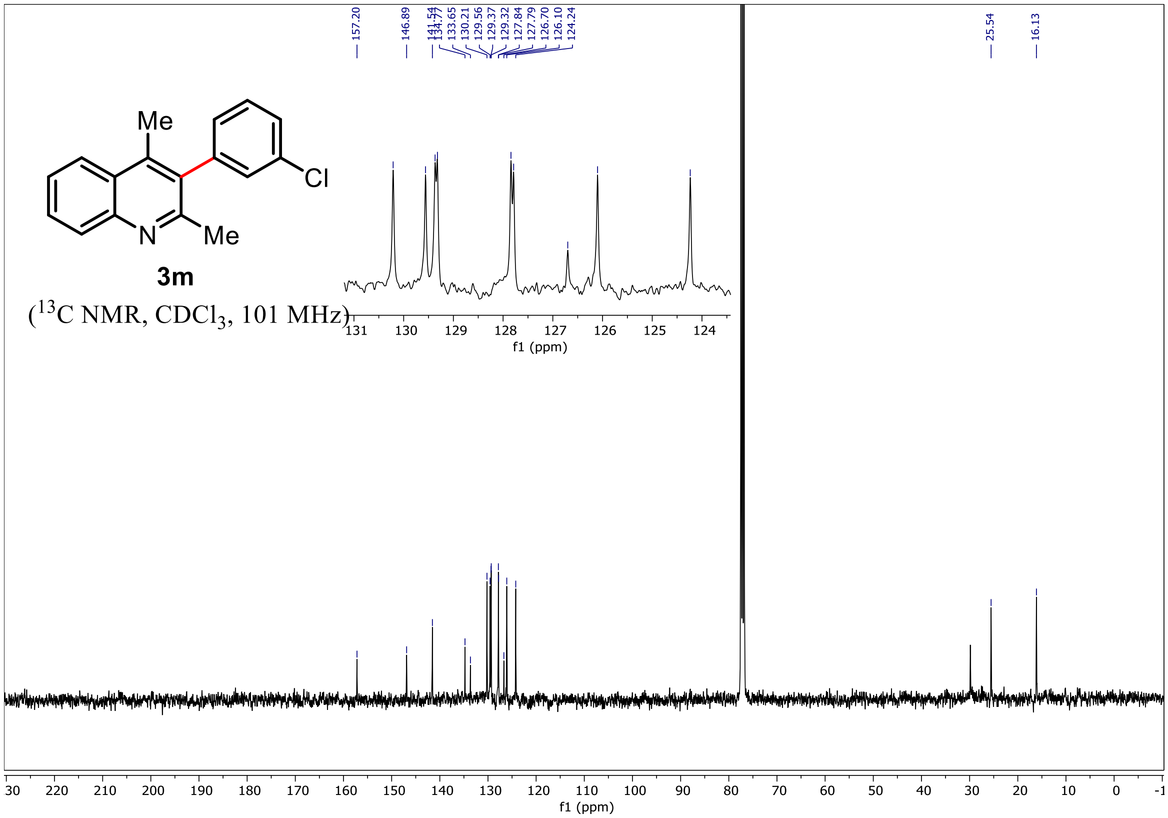
Me
Cl
N
Me
3m
(13C NMR, CDCl3, 101 MHz)
157.20
146.89
141.54
134.77
133.65
130.21
129.56
129.37
129.32
127.84
127.79
126.70
126.10
124.24
25.54
16.13
f1 (ppm)

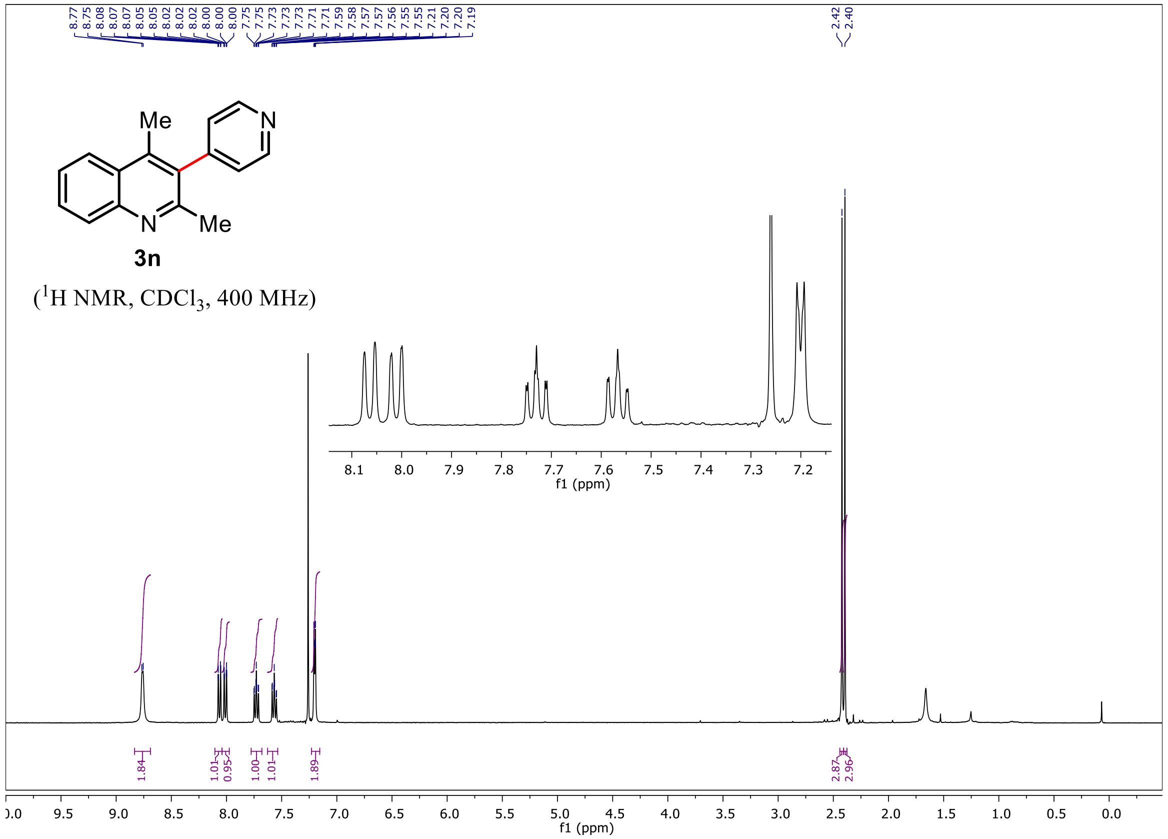
8.77
8.75
8.08
8.07
8.07
8.05
8.05
8.02
8.02
8.02
8.00
8.00
8.00
7.75
7.75
7.73
7.73
7.73
7.71
7.71
7.59
7.58
7.57
7.57
7.56
7.55
7.55
7.21
7.20
7.20
7.19
2.42
2.40
Me
N
N
Me
3n
(1H NMR, CDCl3, 400 MHz)
1.84
1.01
0.95
1.00
1.01
1.89
2.87
2.96
f1 (ppm)

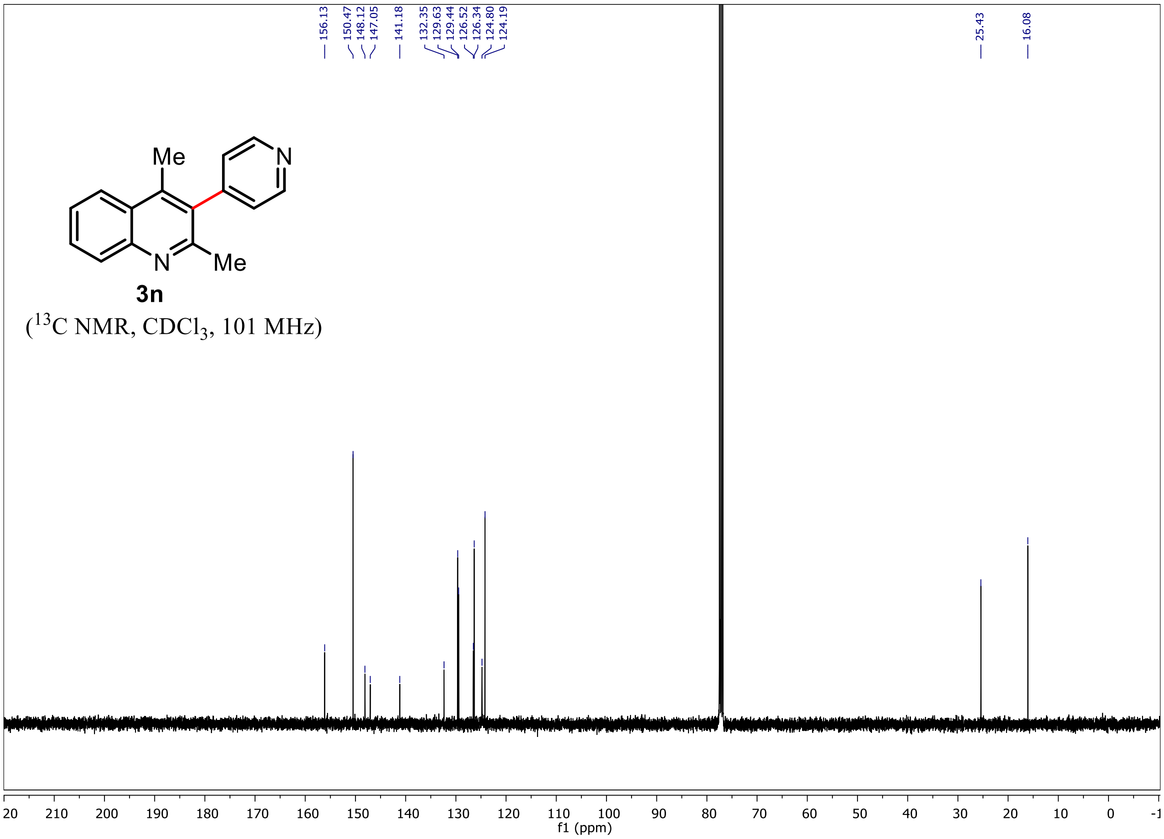

156.13
150.47
148.12
147.05
141.18
132.35
129.63
129.44
126.52
126.34
124.80
124.19
25.43
16.08
Me
N
Me
3n
($^{13}$C NMR, $CDCl_3$, 101 MHz)
f1 (ppm)


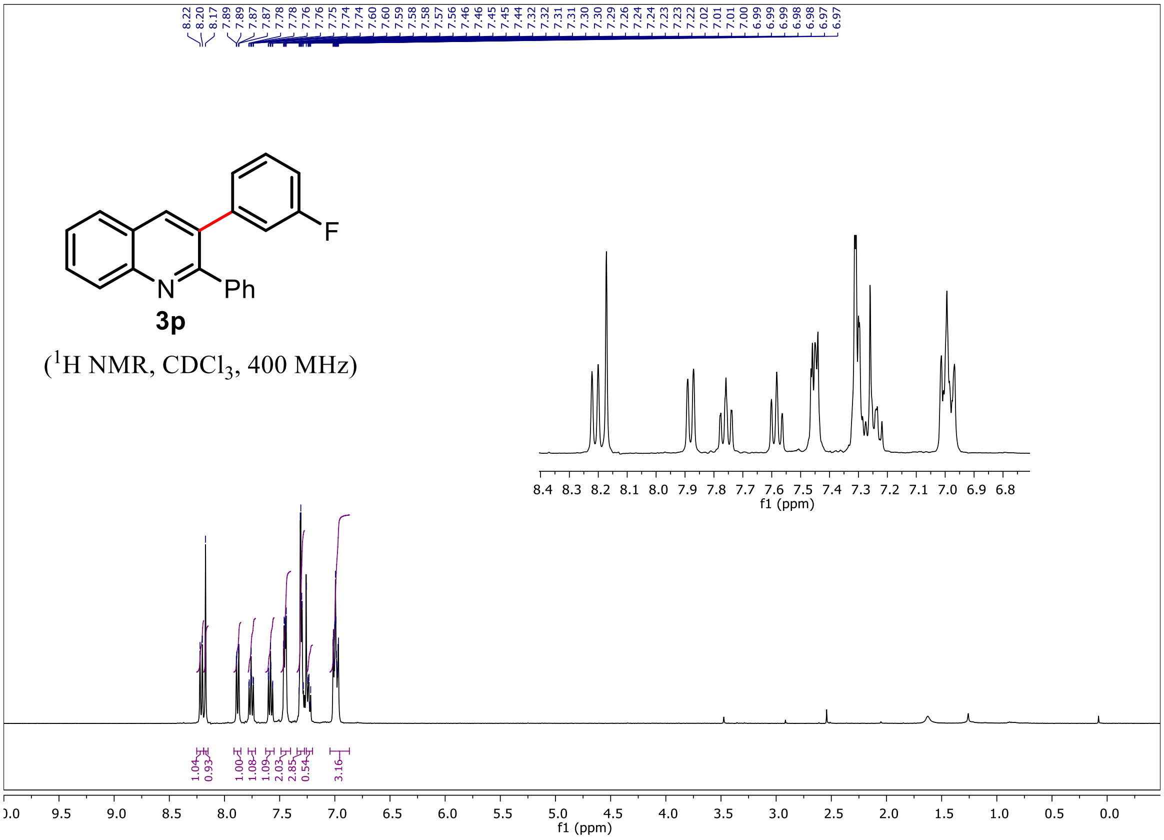

F
N
Ph
3p
($^{1}$H NMR, $CDCl_3$, 400 MHz)
1.04
0.93
1.00
1.08
1.09
2.03
2.85
0.54
3.16
f1 (ppm)

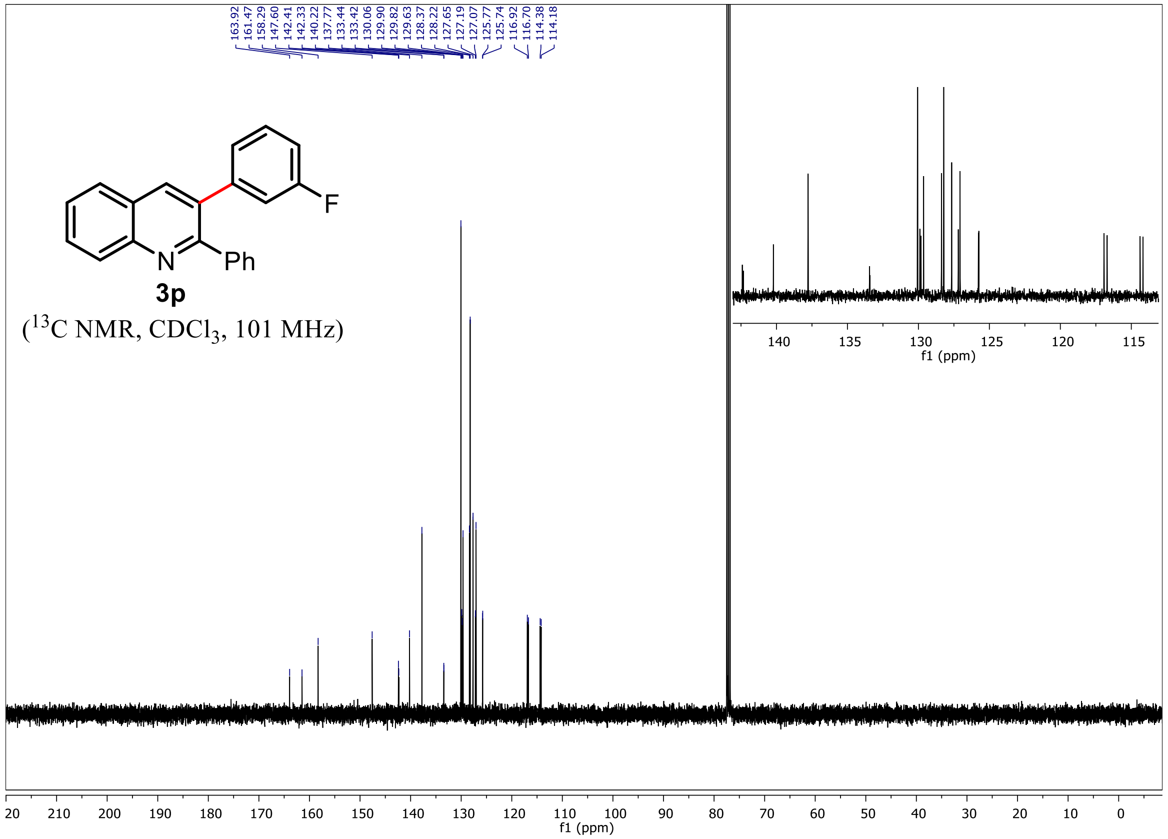
163.92
161.47
158.29
147.60
142.41
142.33
140.22
137.77
133.44
133.42
130.06
129.90
129.82
129.63
128.37
128.22
127.65
127.19
127.07
125.77
125.74
116.92
116.70
114.38
114.18
F
N
Ph
3p
(13C NMR, CDCl3, 101 MHz)
f1 (ppm)

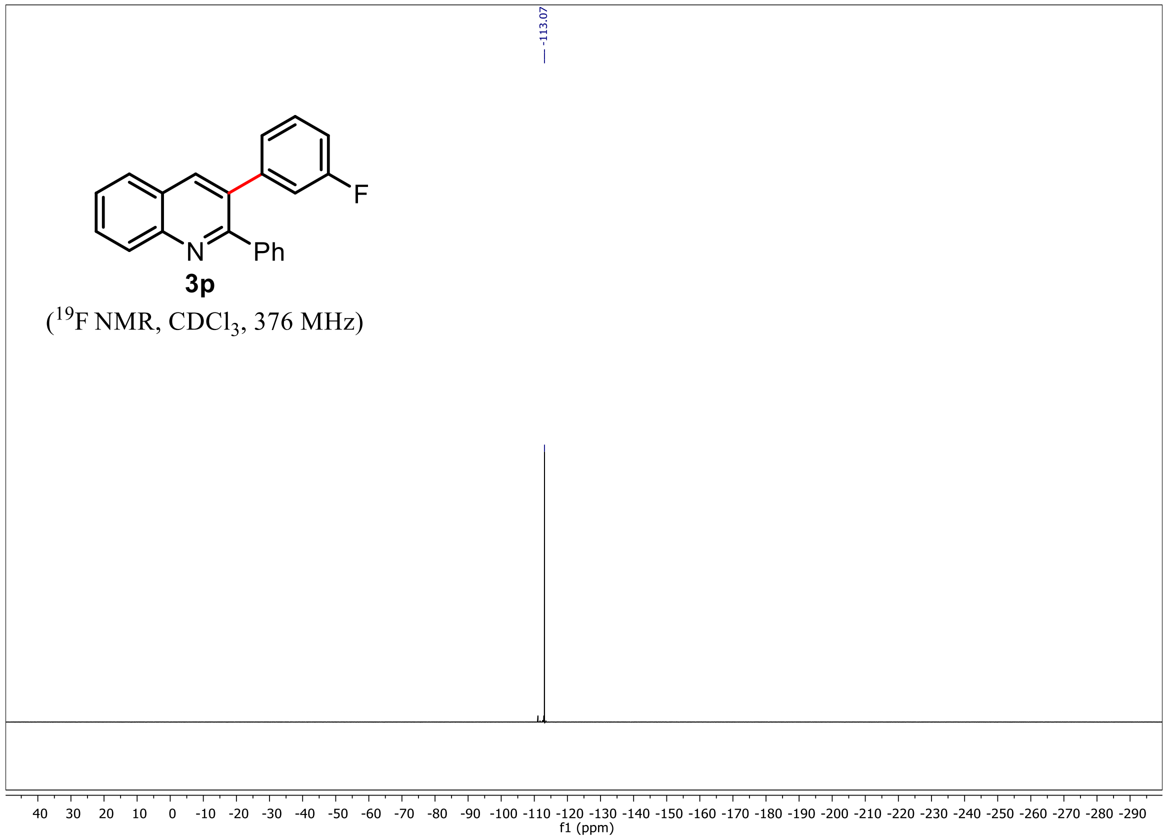
-113.07
F
N
Ph
3p
(19F NMR, CDCl3, 376 MHz)
f1 (ppm)

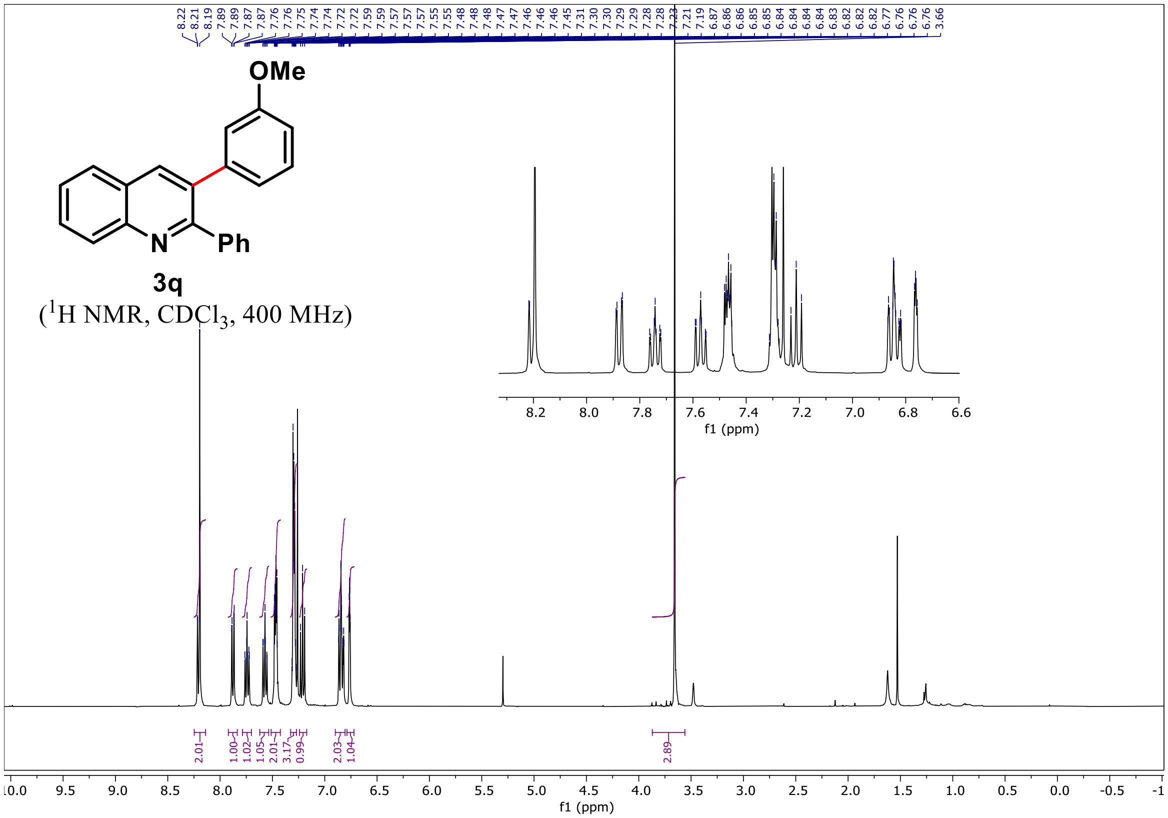
OMe
N
Ph
3q
(1H NMR, CDCl3, 400 MHz)
f1 (ppm)

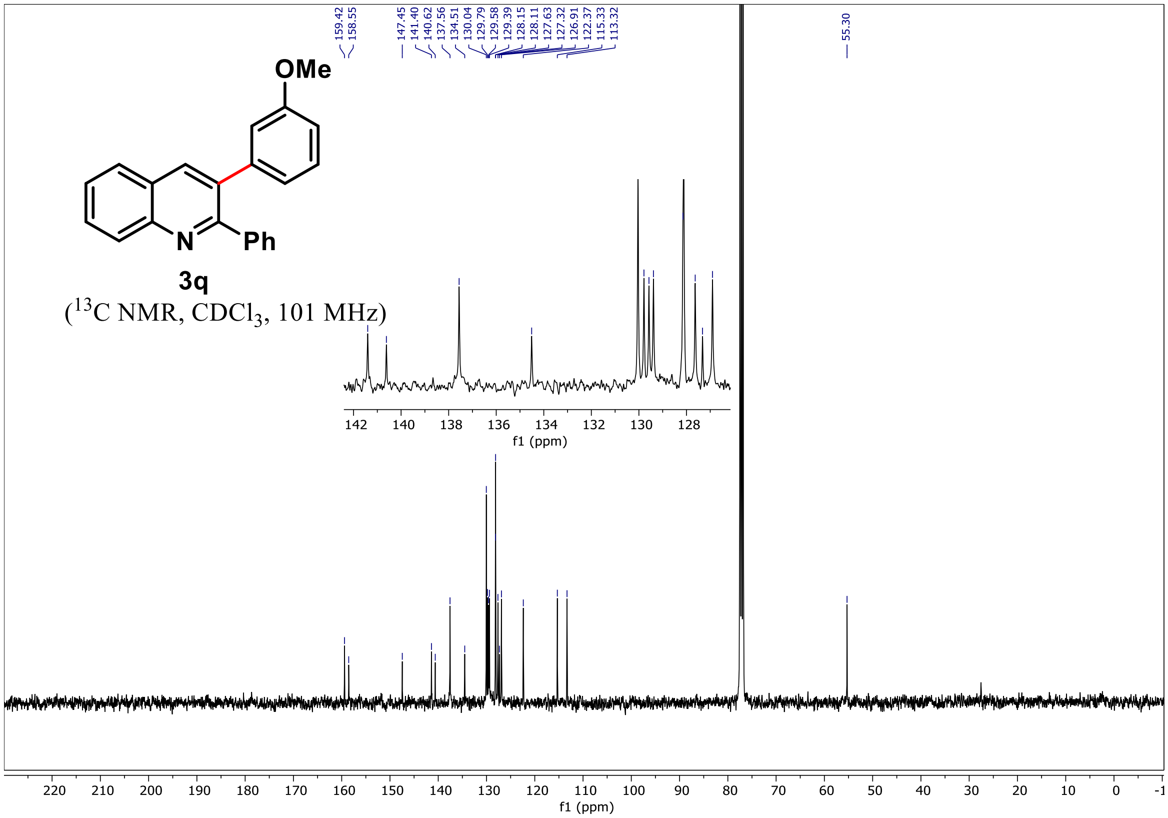
OMe
N
Ph
3q
(13C NMR, CDCl3, 101 MHz)
f1 (ppm)

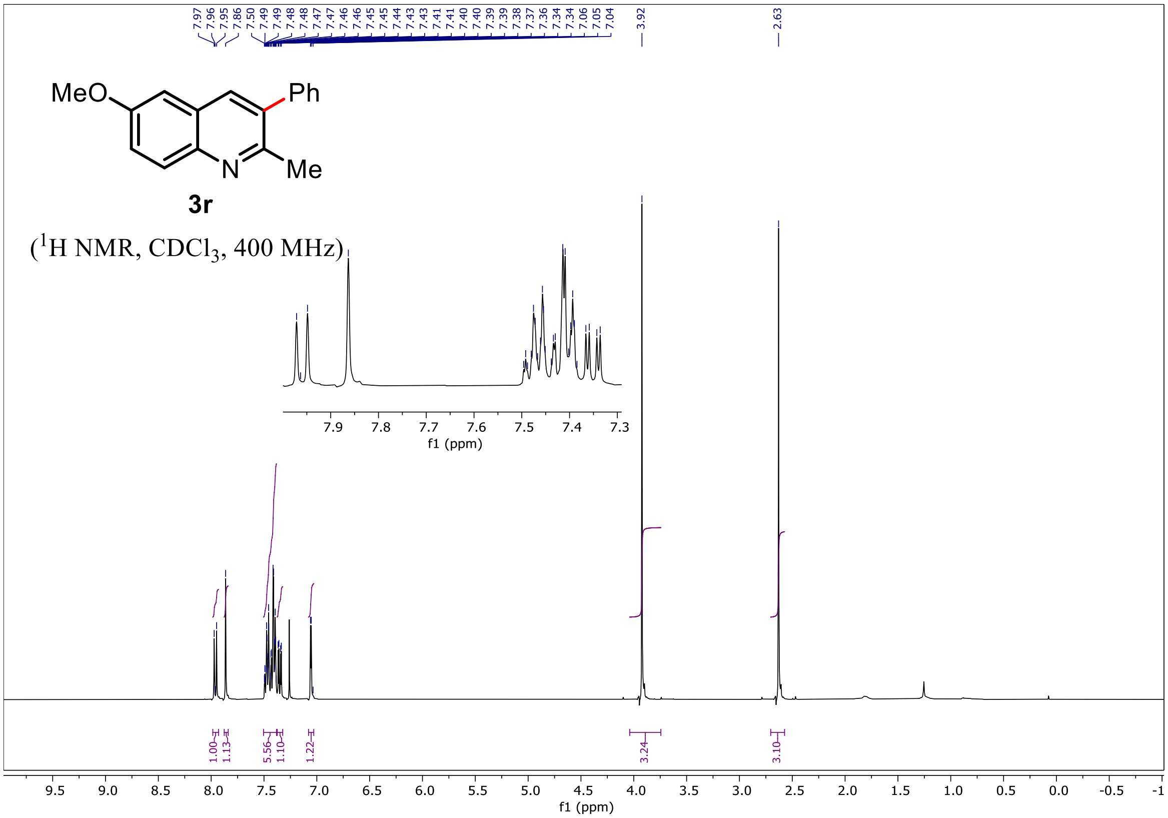
MeO
Ph
N
Me
3r
(1H NMR, CDCl3, 400 MHz)
f1 (ppm)

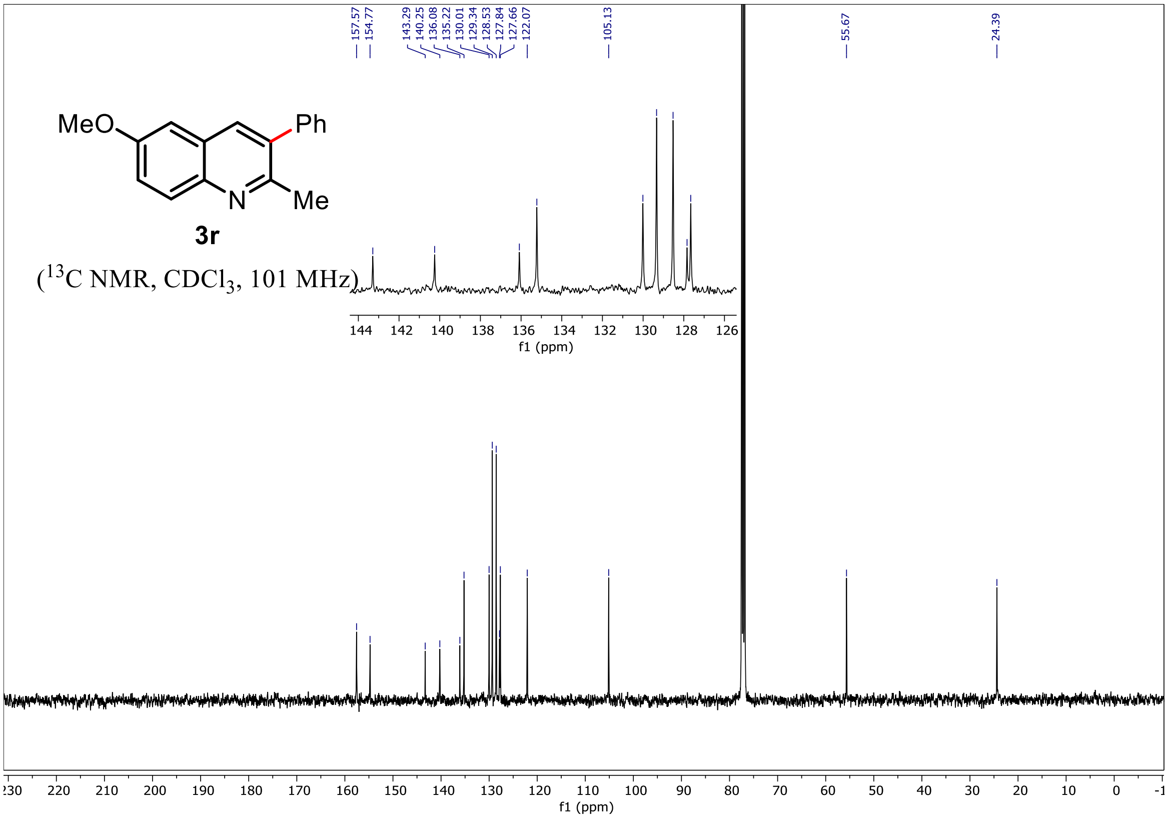
MeO
Ph
N
Me
3r
(13C NMR, CDCl3, 101 MHz)
f1 (ppm)

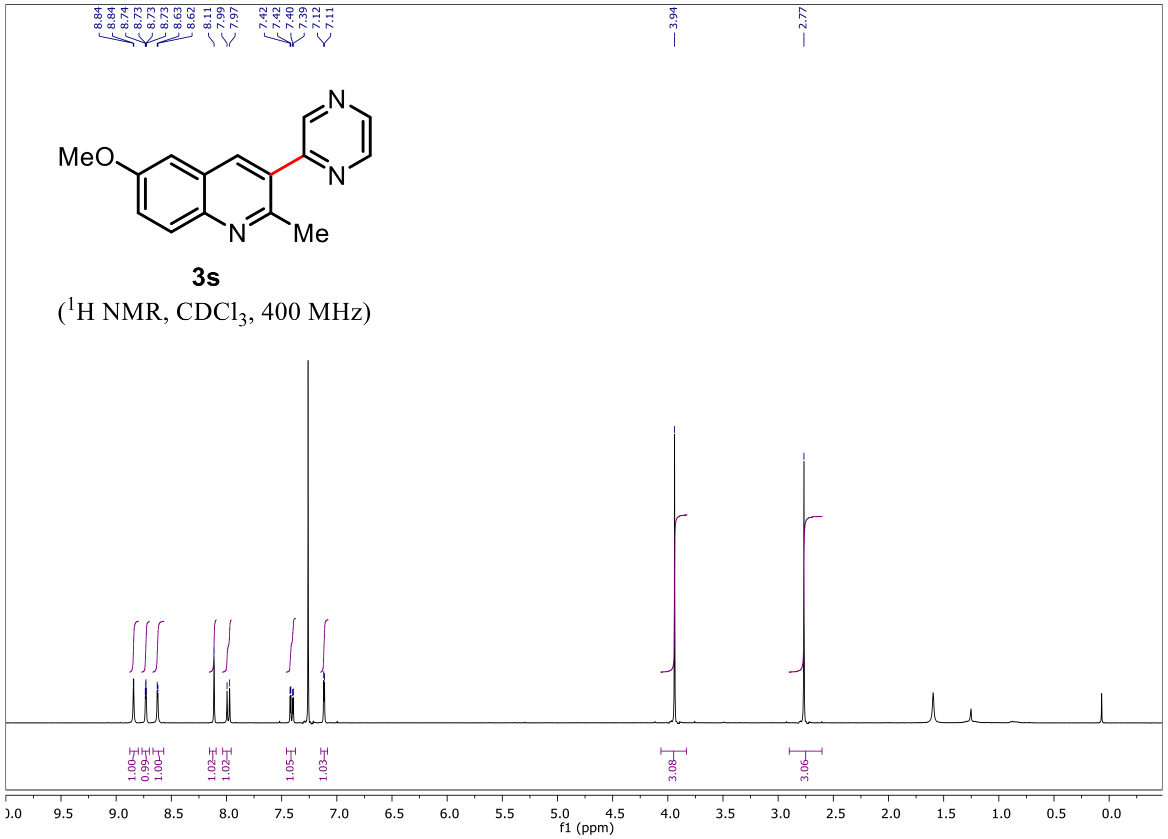
8.84
8.84
8.74
8.73
8.73
8.73
8.63
8.62
8.11
7.99
7.97
7.42
7.42
7.40
7.39
7.12
7.11
3.94
2.77
MeO
N
N
N
Me
3s
(¹H NMR, CDCl₃, 400 MHz)
1.00
0.99
1.00
1.02
1.02
1.05
1.03
3.08
3.06
f1 (ppm)

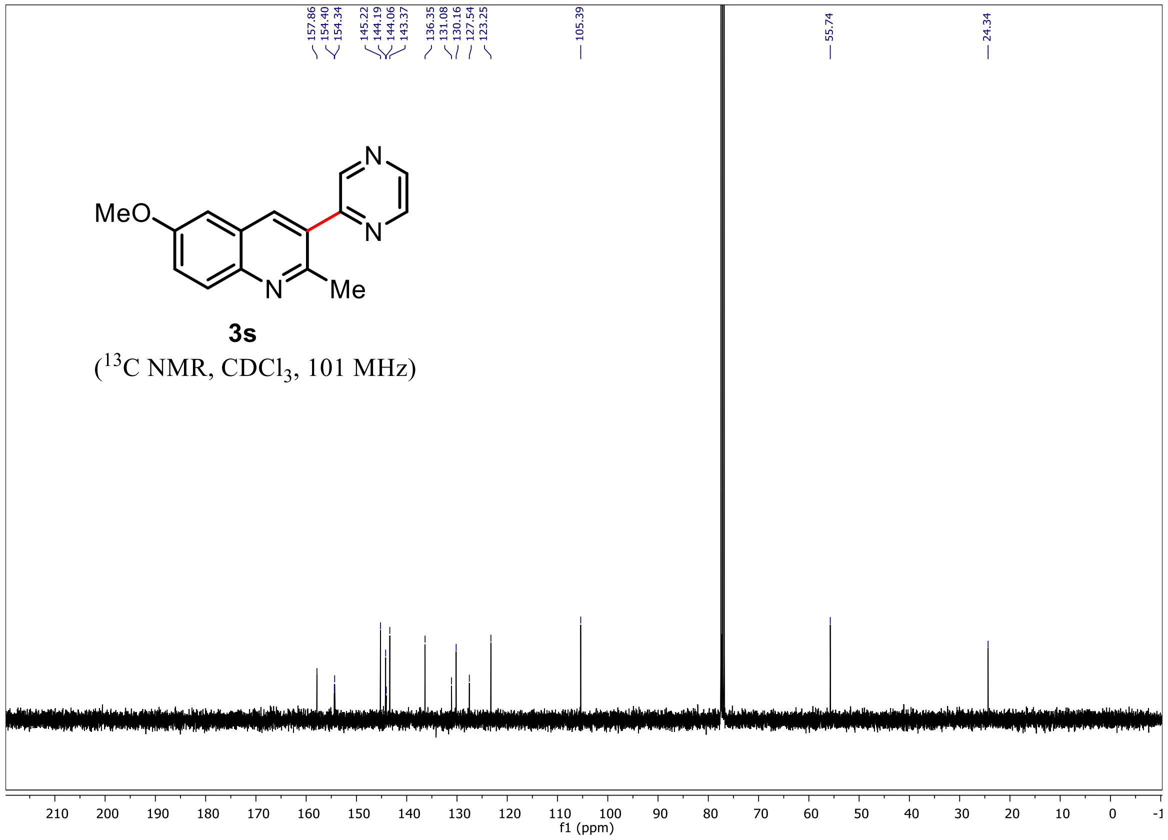
157.86
154.40
154.34
145.22
144.19
144.06
143.37
136.35
131.08
130.16
127.54
123.25
105.39
55.74
24.34
MeO
N
N
N
Me
3s
(¹³C NMR, CDCl₃, 101 MHz)
f1 (ppm)

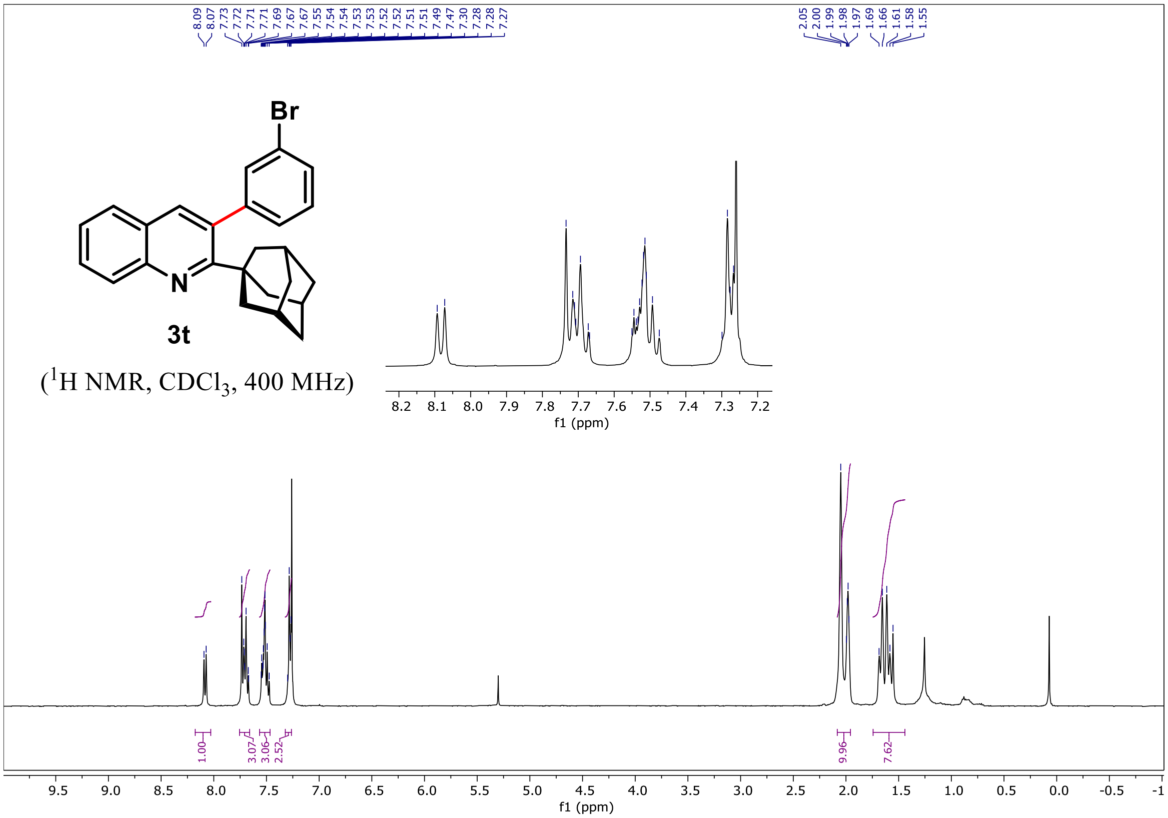
8.09 8.07 7.73 7.72 7.71 7.71 7.69 7.67 7.67 7.55 7.54 7.54 7.53 7.53 7.52 7.52 7.51 7.51 7.49 7.47 7.30 7.28 7.28 7.27
2.05 2.00 1.99 1.98 1.97 1.69 1.66 1.61 1.58 1.55
Br
N
3t
(1H NMR, CDCl3, 400 MHz)
8.2 8.1 8.0 7.9 7.8 7.7 7.6 7.5 7.4 7.3 7.2
f1 (ppm)
1.00 3.07 3.06 2.52 9.96 7.62
9.5 9.0 8.5 8.0 7.5 7.0 6.5 6.0 5.5 5.0 4.5 4.0 3.5 3.0 2.5 2.0 1.5 1.0 0.5 0.0 -0.5 -1
f1 (ppm)

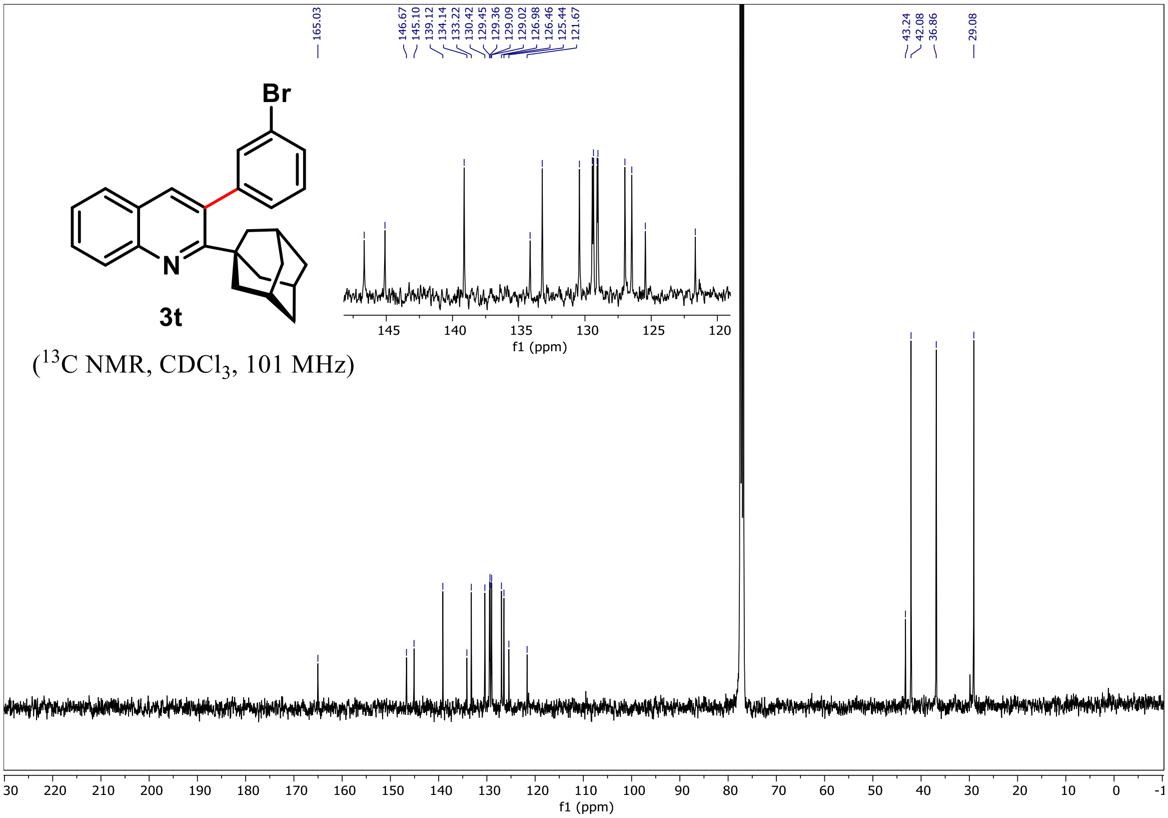
165.03
146.67 145.10 139.12 134.14 133.22 130.42 129.45 129.36 129.09 129.02 126.98 126.46 125.44 121.67
43.24 42.08 36.86 29.08
Br
N
3t
(13C NMR, CDCl3, 101 MHz)
145 140 135 130 125 120
f1 (ppm)
30 220 210 200 190 180 170 160 150 140 130 120 110 100 90 80 70 60 50 40 30 20 10 0 -1
f1 (ppm)

1D NOE NMR spectrum of **3t**, 500 MHz, $C_6D_6$

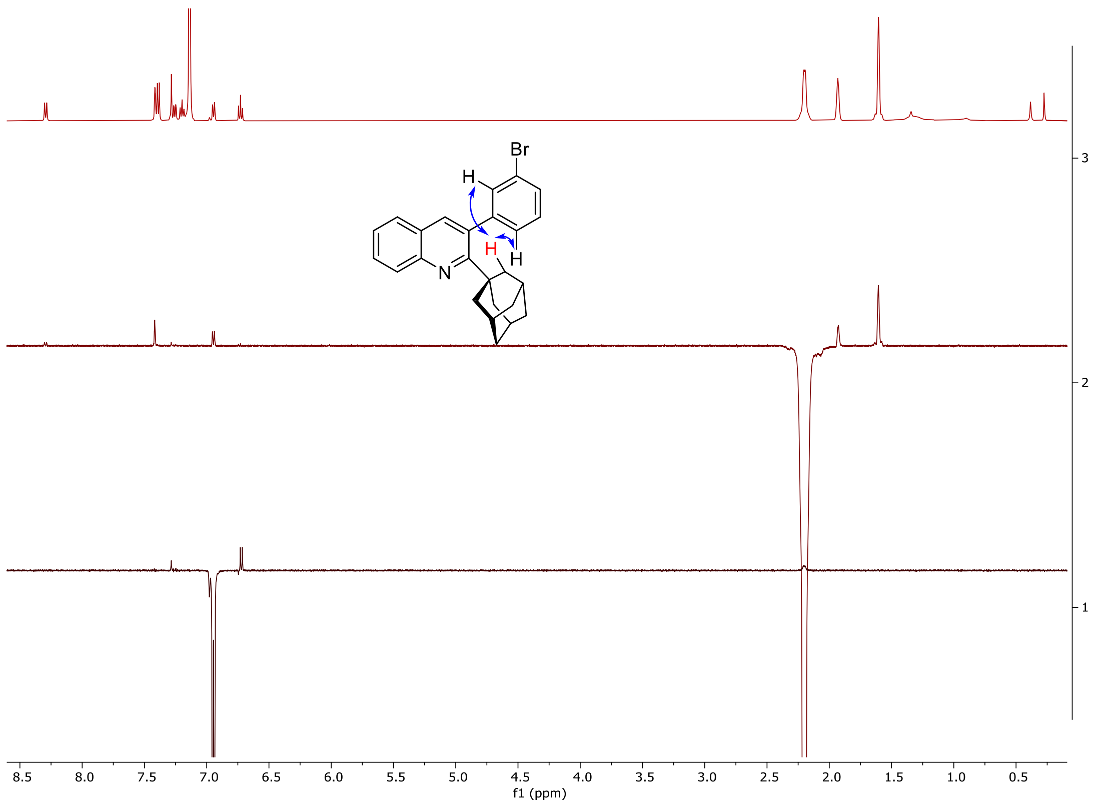


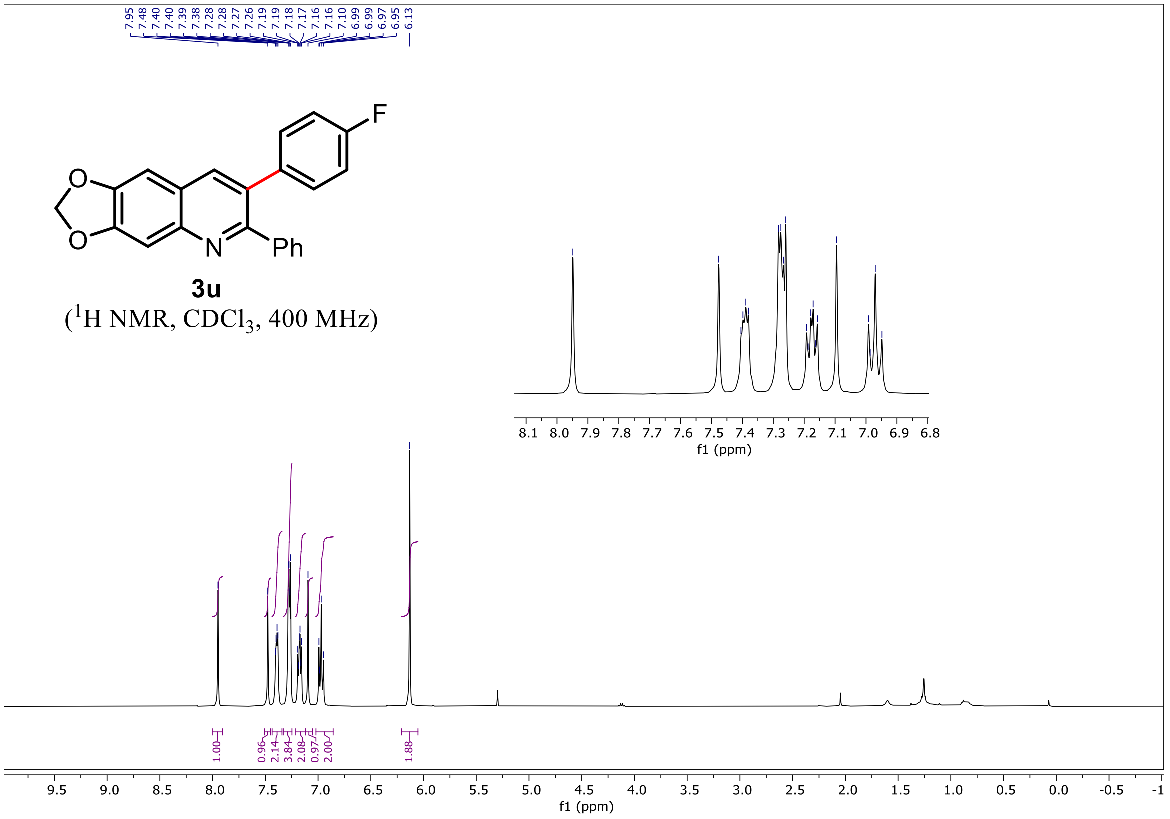

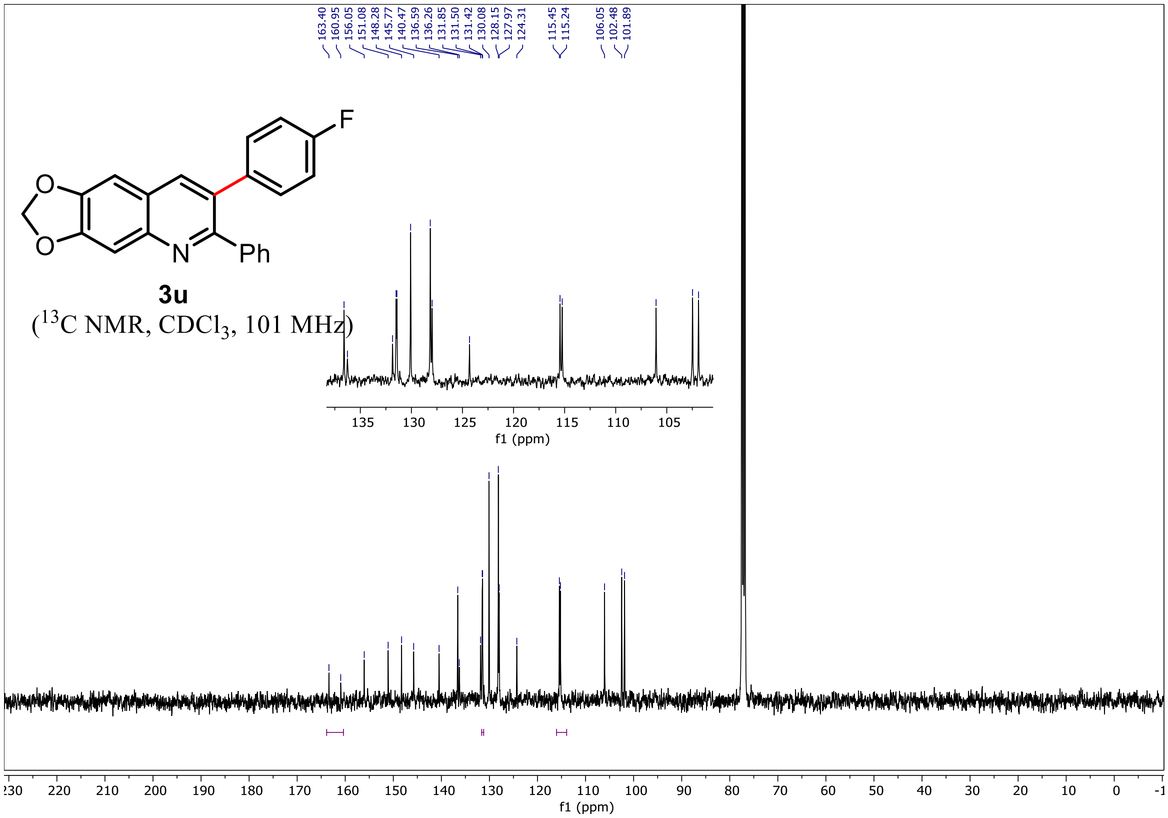


**3u**
($^{13}$C NMR, $CDCl_3$, 101 MHz)

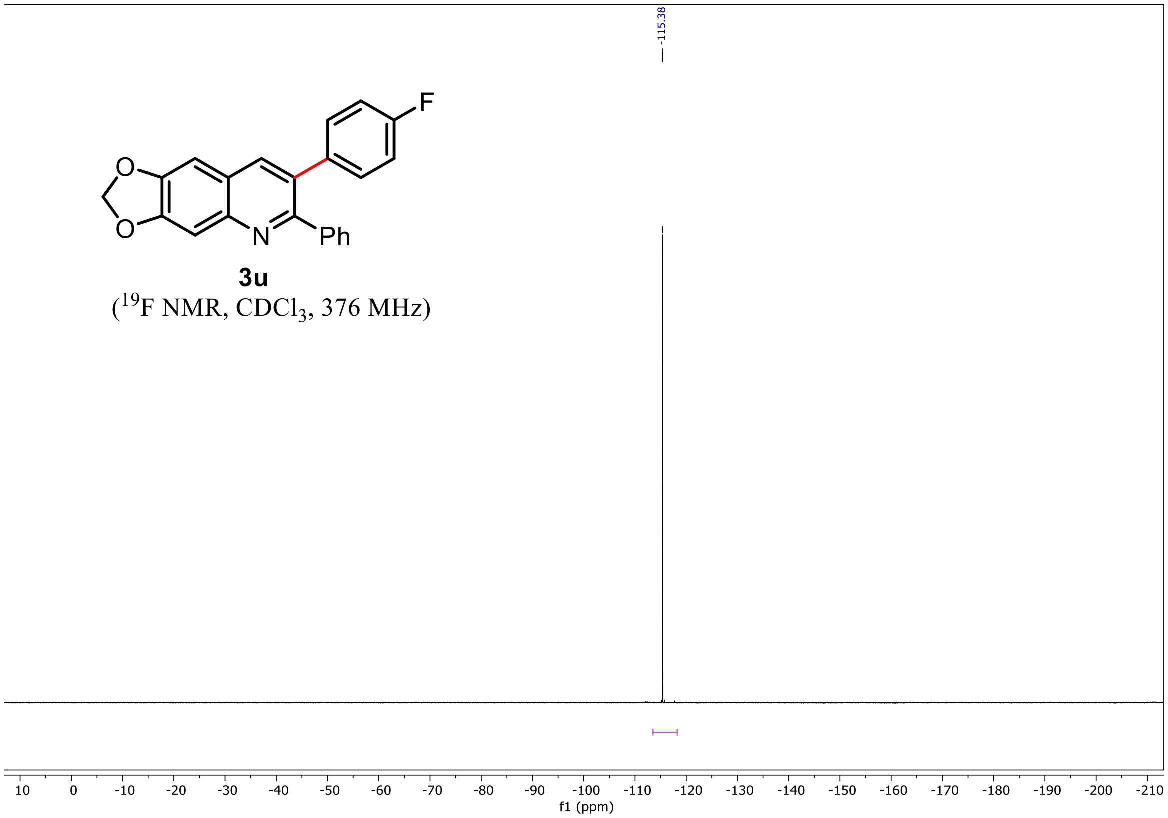


**3u**
($^{19}$F NMR, $CDCl_3$, 376 MHz)

1D NOE NMR spectrum of **3u**, 500 MHz, $CDCl_3$

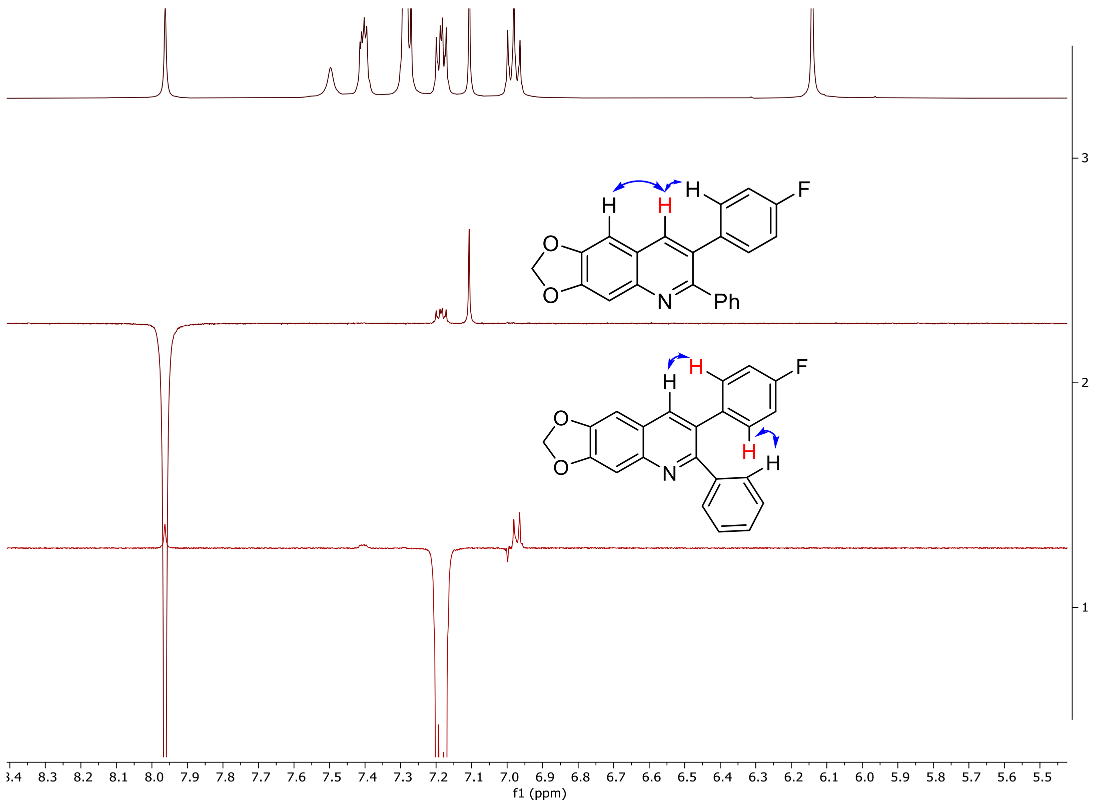

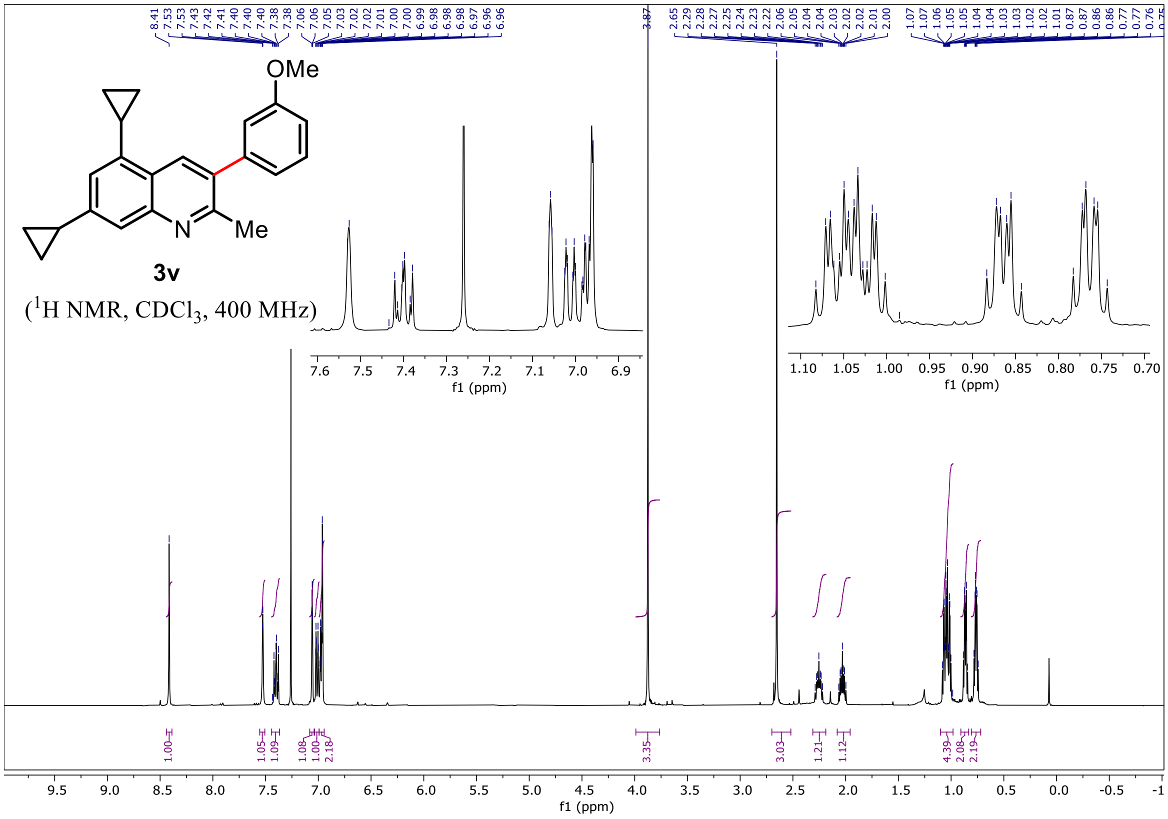

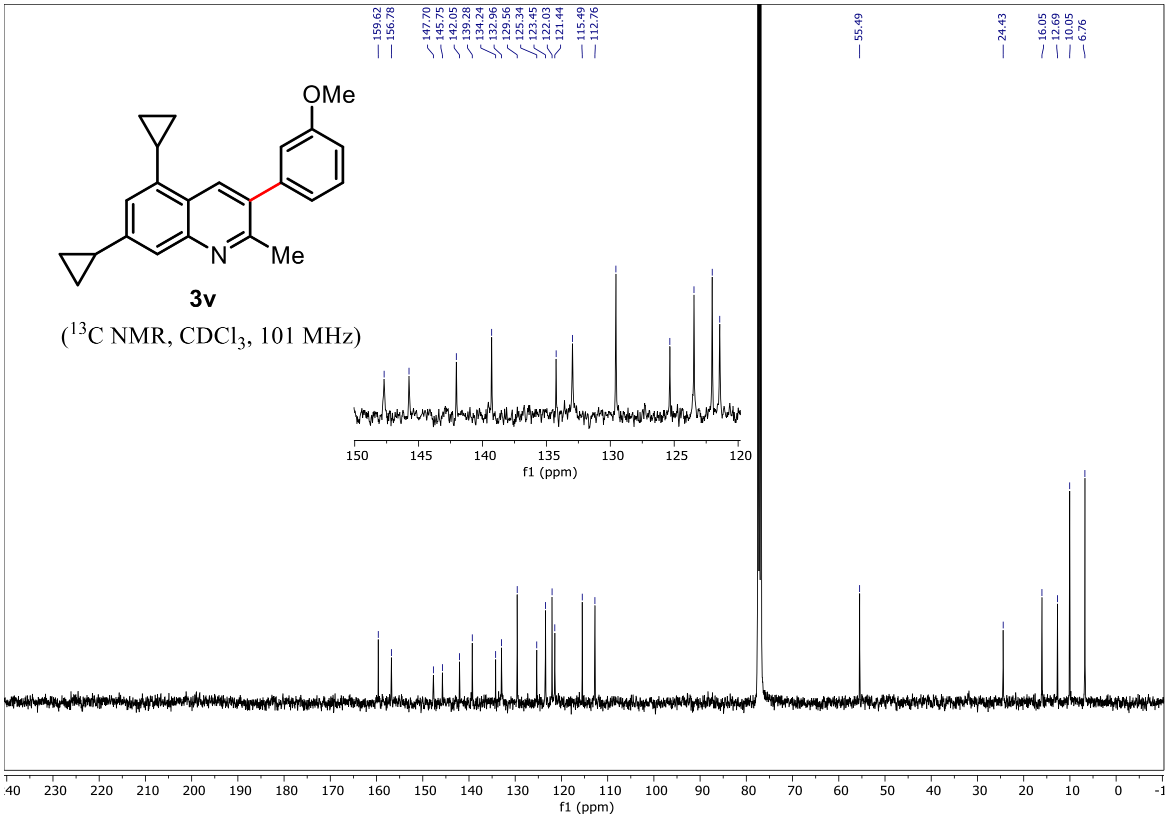


1D NOE NMR spectrum of **3v**, 500 MHz, $CDCl_3$

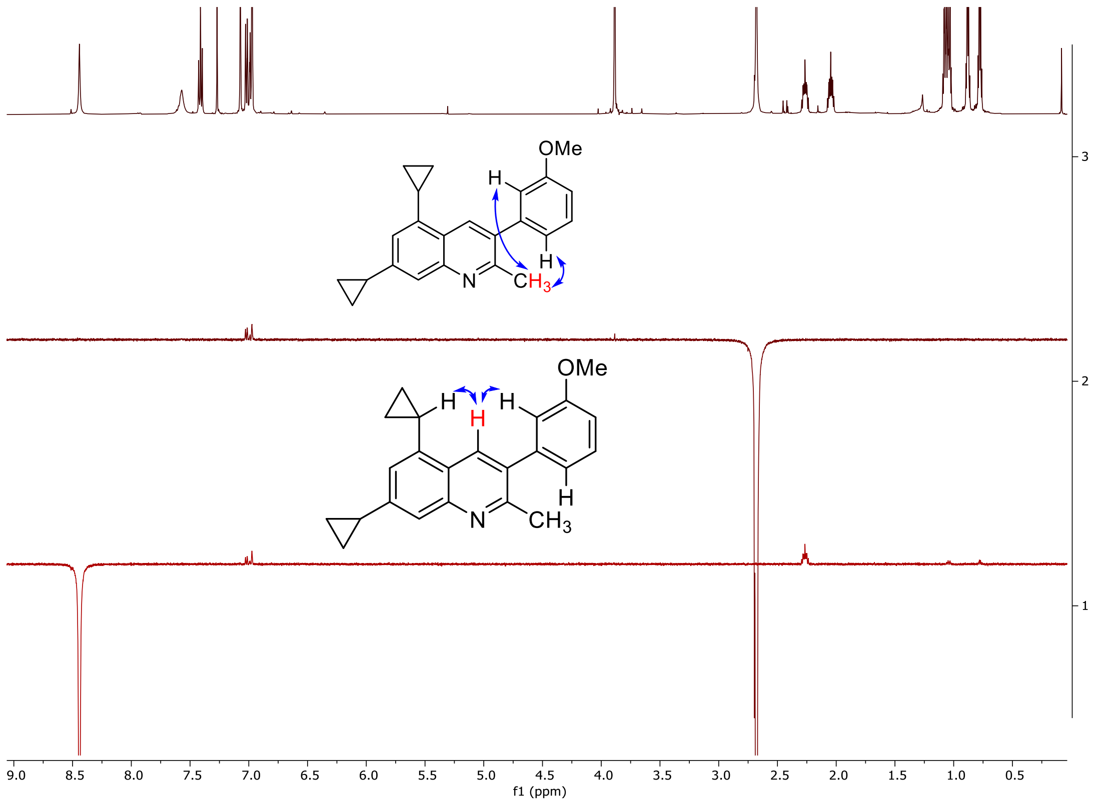

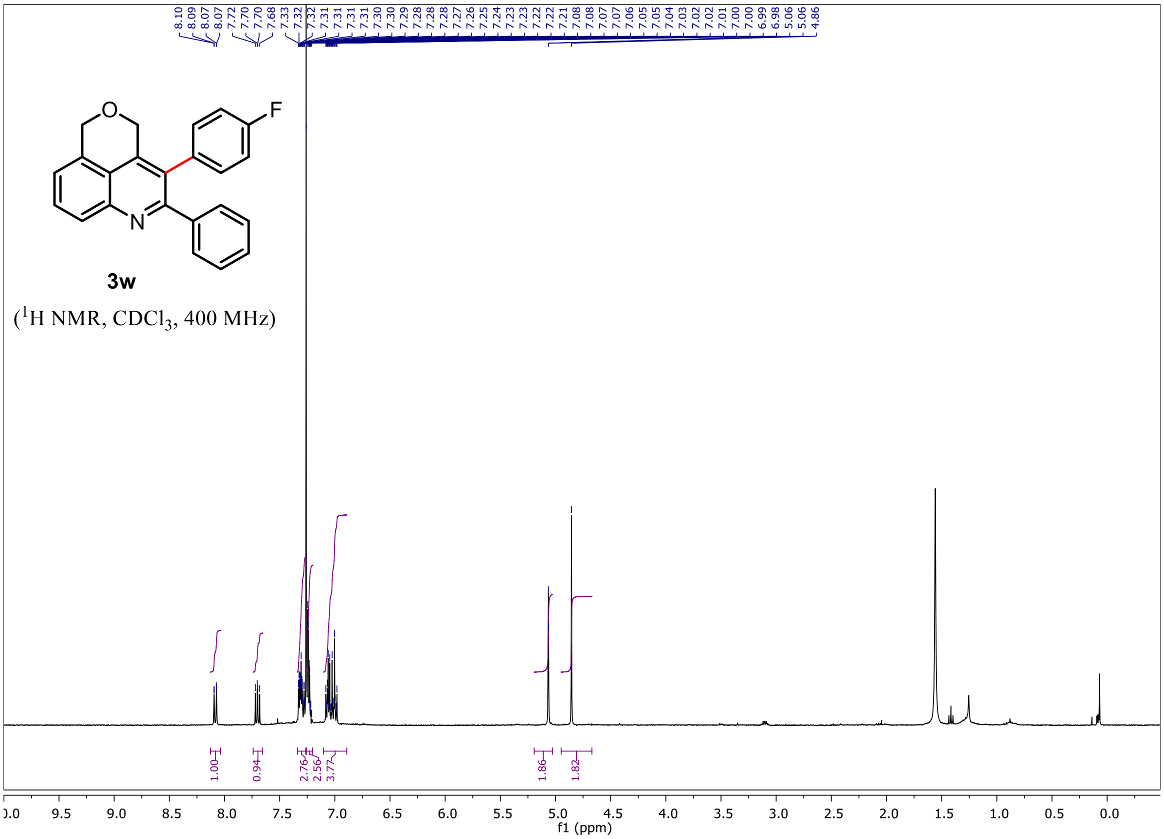


**3w**

($^1$H NMR, $CDCl_3$, 400 MHz)

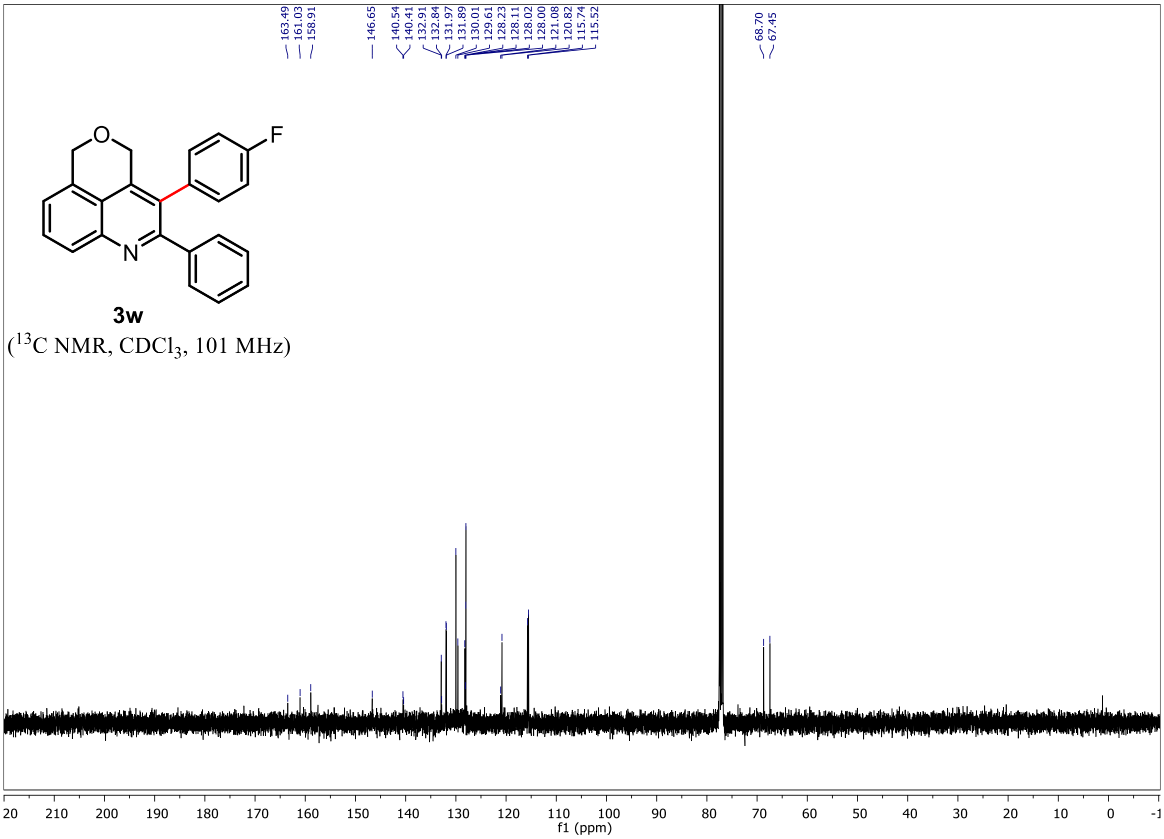


**3w**

($^{13}$C NMR, $CDCl_3$, 101 MHz)

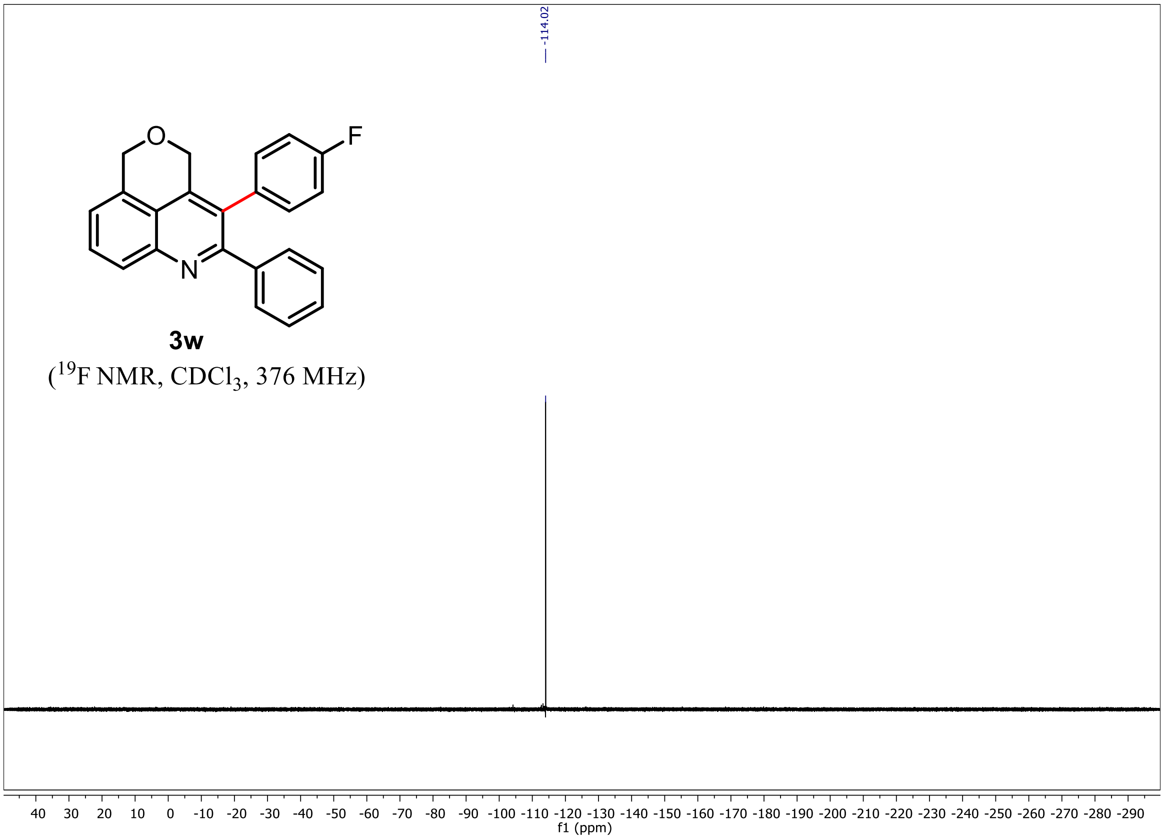
-114.02
3w
($^{19}$F NMR, CDCl$_3$, 376 MHz)
f1 (ppm)

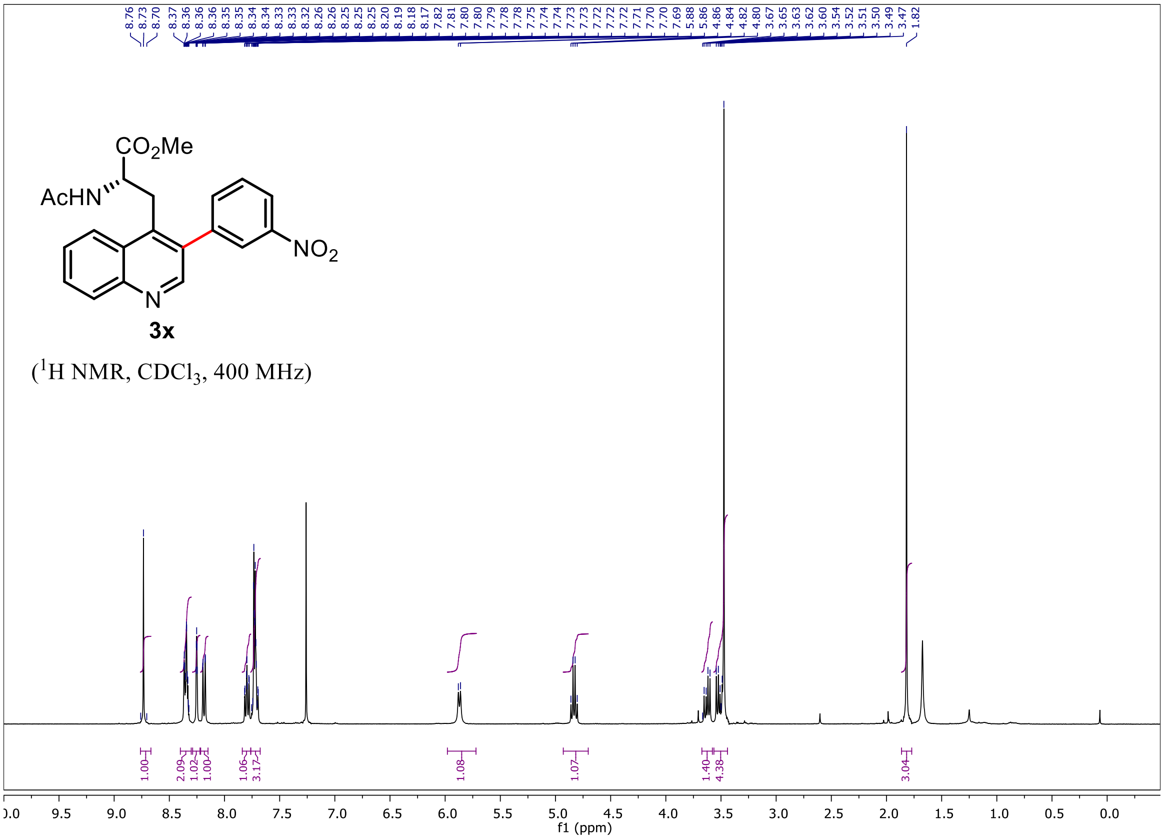
8.76 8.73 8.70 8.37 8.36 8.36 8.36 8.35 8.35 8.34 8.34 8.33 8.33 8.32 8.26 8.26 8.25 8.25 8.25 8.20 8.19 8.18 8.17 7.82 7.81 7.80 7.80 7.79 7.78 7.78 7.75 7.74 7.74 7.73 7.73 7.72 7.72 7.72 7.71 7.70 7.70 7.69 5.88 5.86 4.86 4.84 4.82 4.80 3.67 3.65 3.63 3.62 3.60 3.54 3.52 3.51 3.50 3.49 3.47 1.82
3x
($^{1}$H NMR, CDCl$_3$, 400 MHz)
1.00 2.09 1.02 1.00 1.06 3.17 1.08 1.07 1.40 4.38 3.04
f1 (ppm)

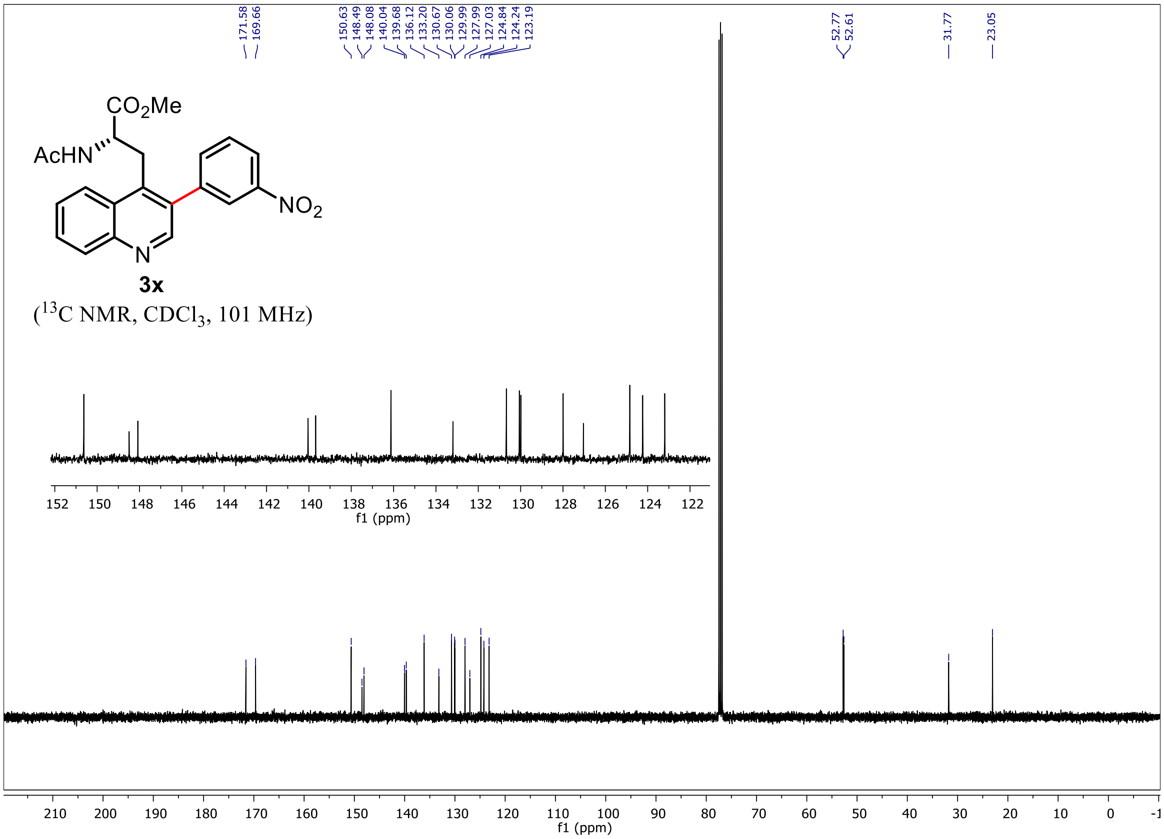

171.58
169.66
150.63
148.49
148.08
140.04
139.68
136.12
133.20
130.67
130.06
129.99
127.99
127.03
124.84
124.24
123.19
52.77
52.61
31.77
23.05
CO2Me
AcHN
NO2
N
3x
(13C NMR, CDCl3, 101 MHz)
f1 (ppm)


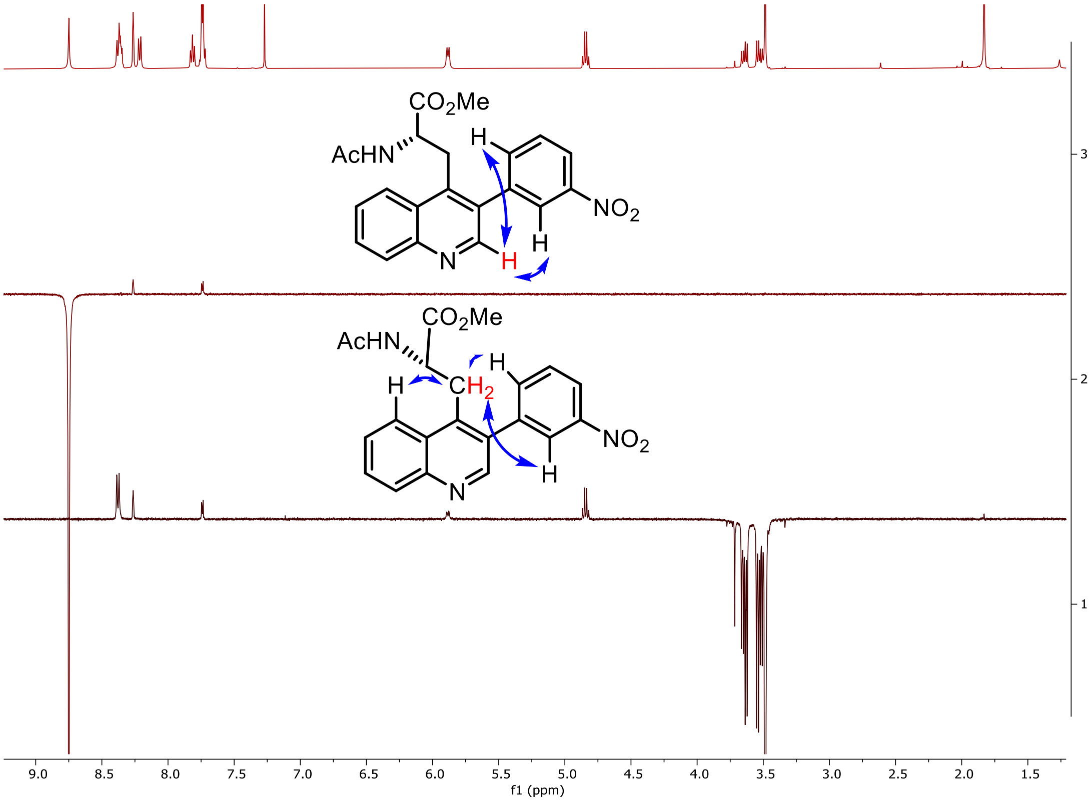

CO2Me
AcHN
H
NO2
N
H
H
CO2Me
AcHN
H
CH2
H
NO2
N
H
f1 (ppm)

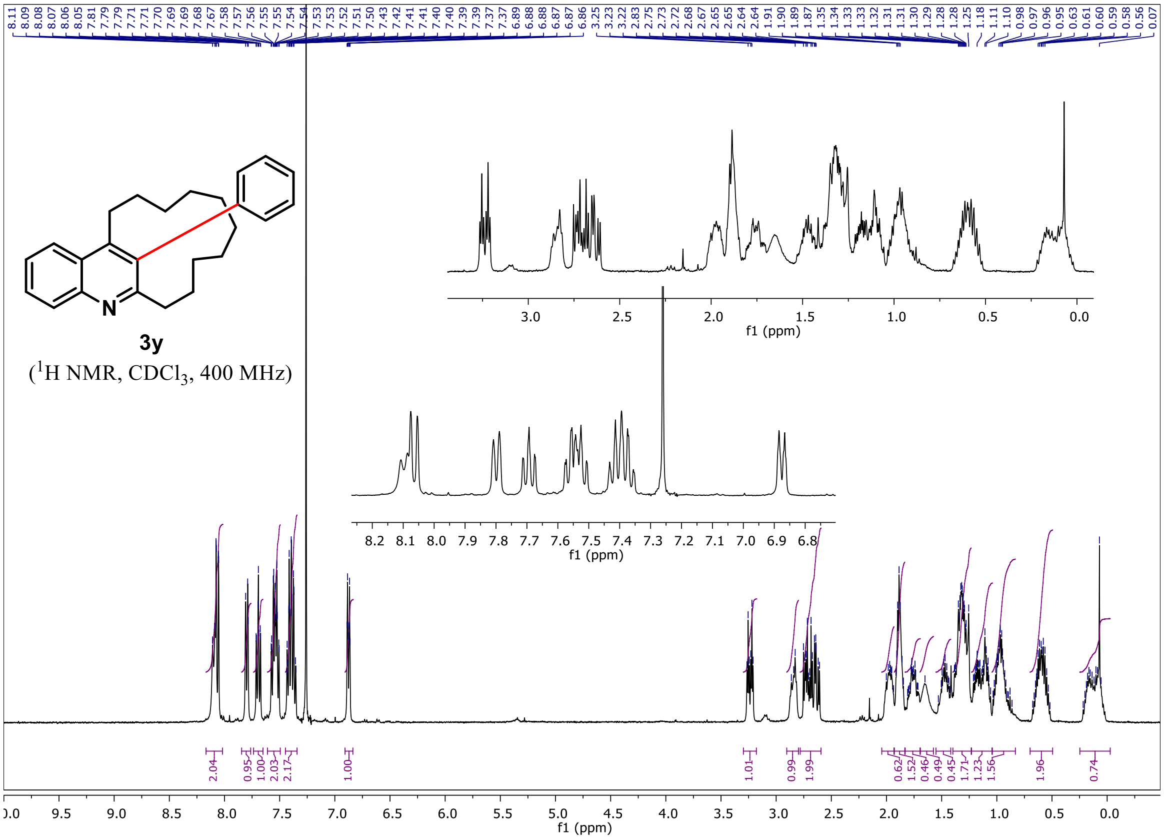
8.11 8.09 8.08 8.07 8.06 8.05 7.81 7.79 7.79 7.71 7.71 7.70 7.69 7.69 7.68 7.67 7.58 7.57 7.56 7.55 7.55 7.54 7.54 7.53 7.53 7.52 7.51 7.50 7.43 7.42 7.41 7.41 7.40 7.39 7.39 7.37 7.37 6.89 6.88 6.88 6.87 6.87 6.86 3.25 3.23 3.22 2.83 2.75 2.73 2.72 2.68 2.67 2.65 2.65 2.64 2.64 1.91 1.90 1.89 1.87 1.35 1.34 1.33 1.33 1.32 1.31 1.31 1.30 1.29 1.28 1.28 1.25 1.18 1.11 1.10 0.98 0.97 0.96 0.95 0.63 0.61 0.60 0.59 0.58 0.56 0.07
N
3y
(1H NMR, CDCl3, 400 MHz)
2.04 0.95 1.00 2.03 2.17 1.00 1.01 0.99 1.99 0.62 1.52 0.46 0.49 0.45 1.71 1.23 1.56 1.96 0.74
f1 (ppm)

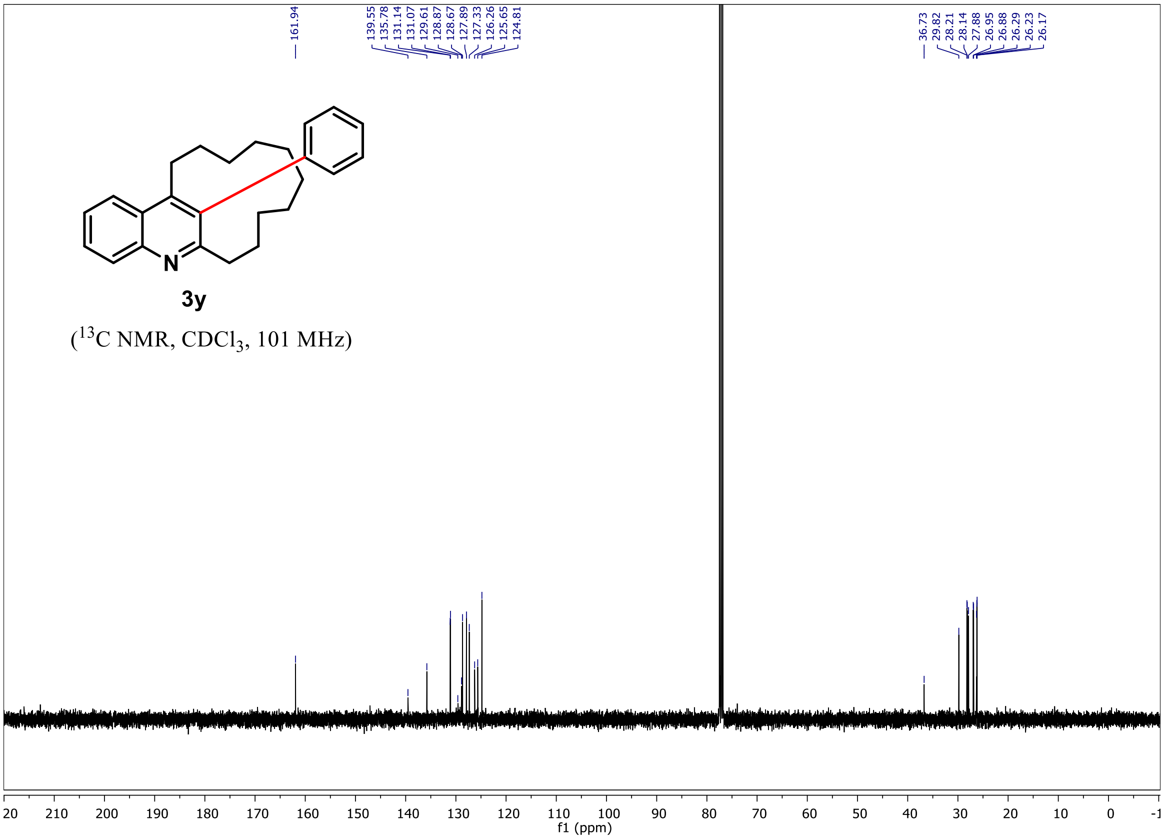
161.94
139.55 135.78 131.14 131.07 129.61 128.67 127.89 127.33 126.26 125.65 124.81
36.73 29.82 28.21 28.14 27.88 26.95 26.88 26.29 26.23 26.17
N
3y
(13C NMR, CDCl3, 101 MHz)
f1 (ppm)

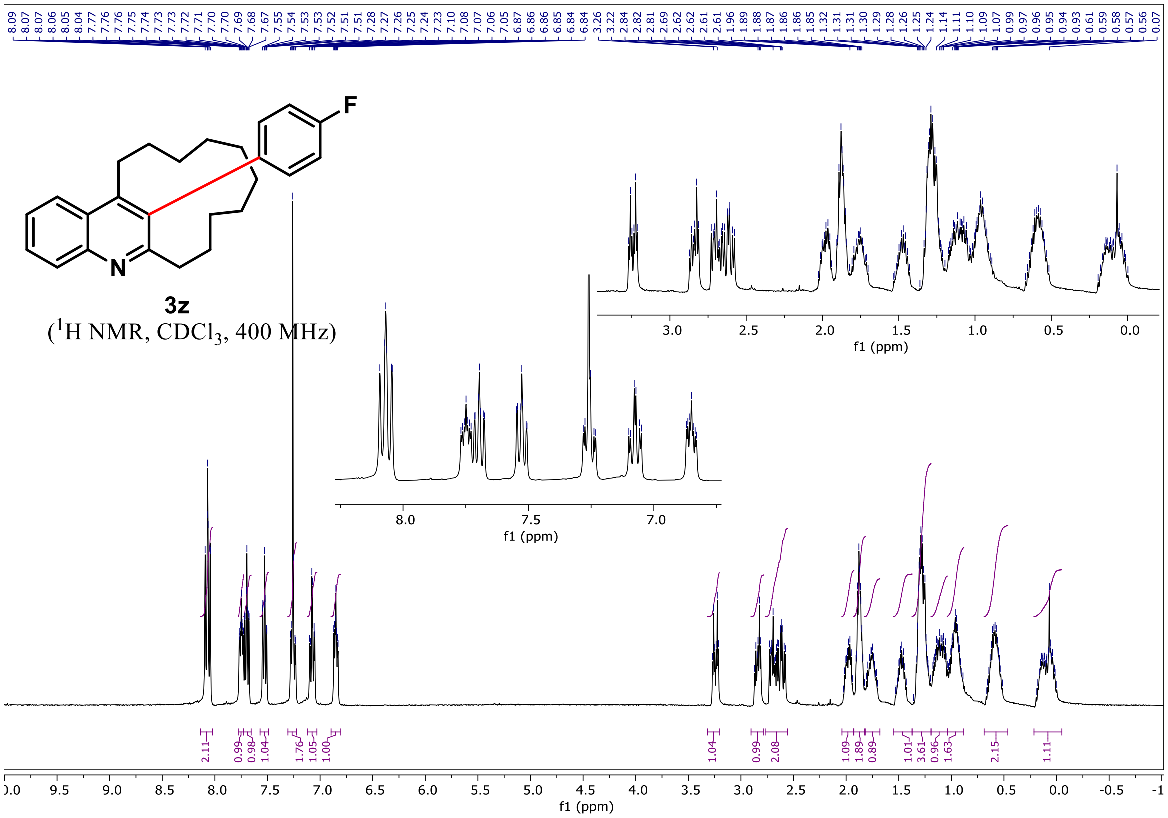
3z
(¹H NMR, CDCl₃, 400 MHz)
f1 (ppm)

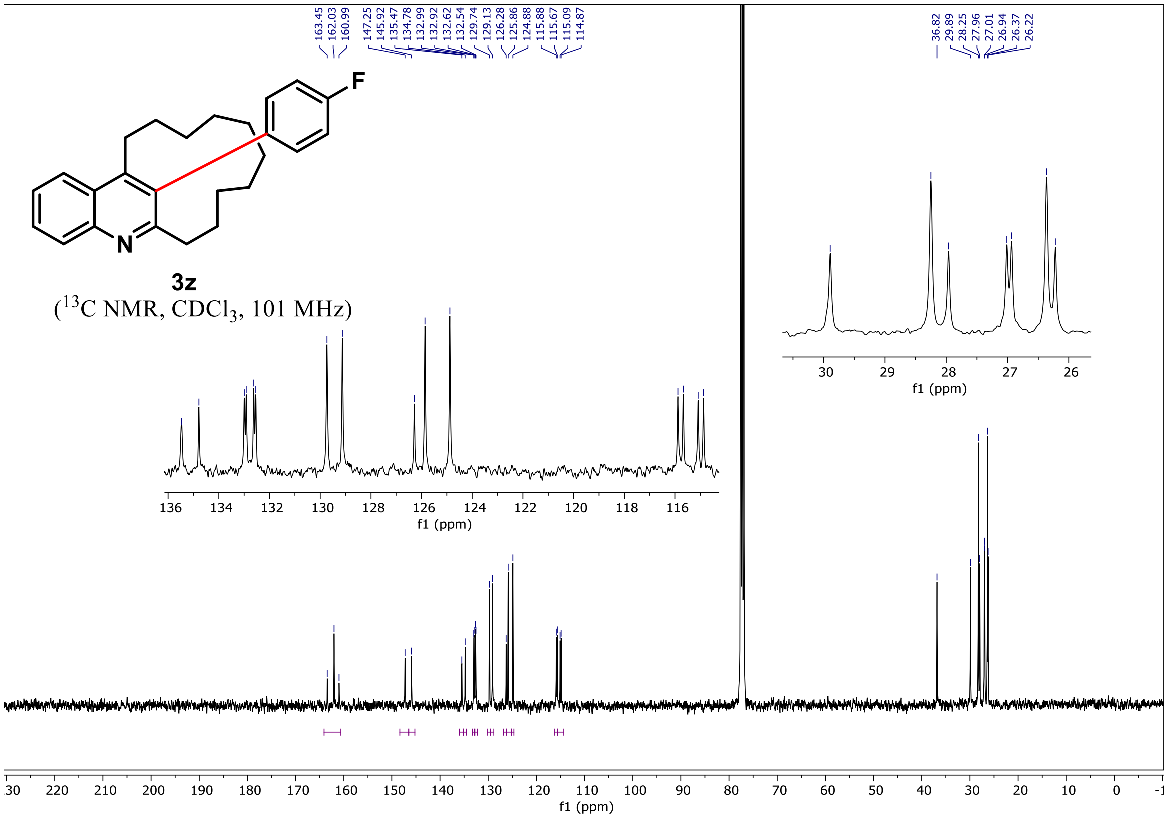
3z
(¹³C NMR, CDCl₃, 101 MHz)
f1 (ppm)

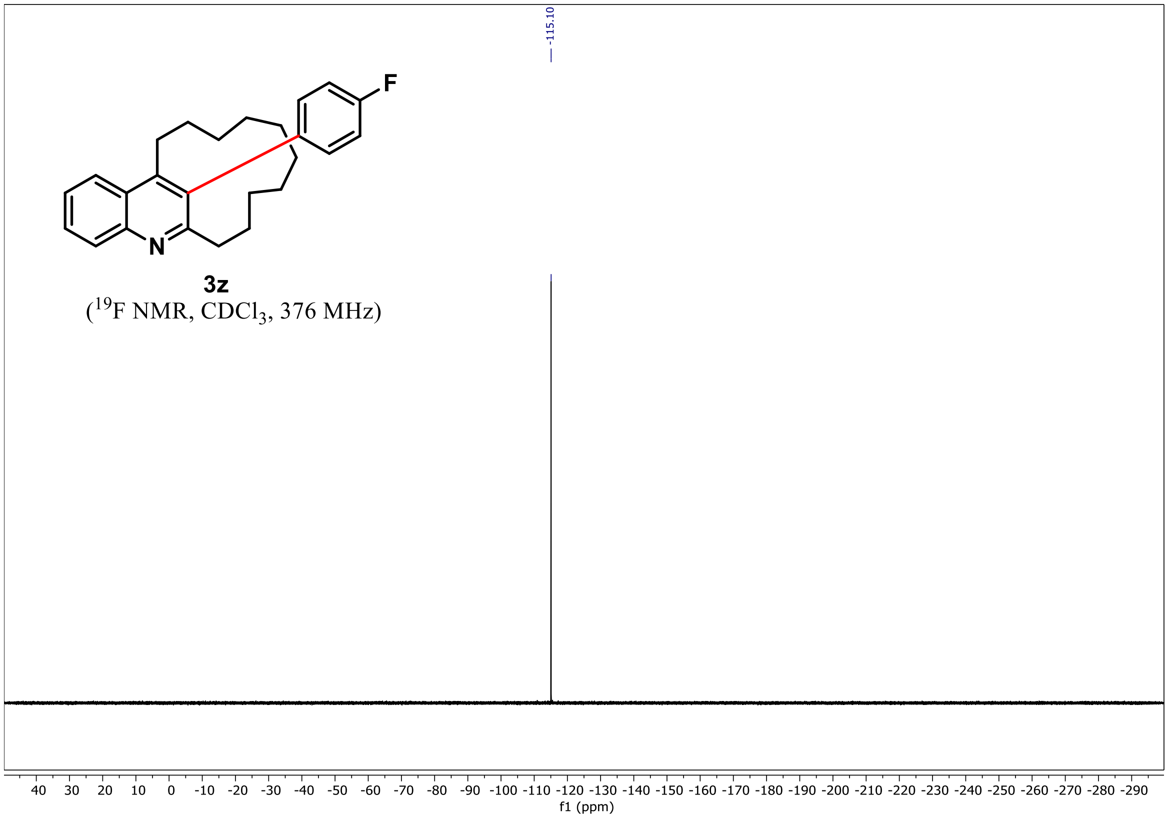
-115.10
F
N
3z
($^{19}$F NMR, CDCl$_3$, 376 MHz)
f1 (ppm)

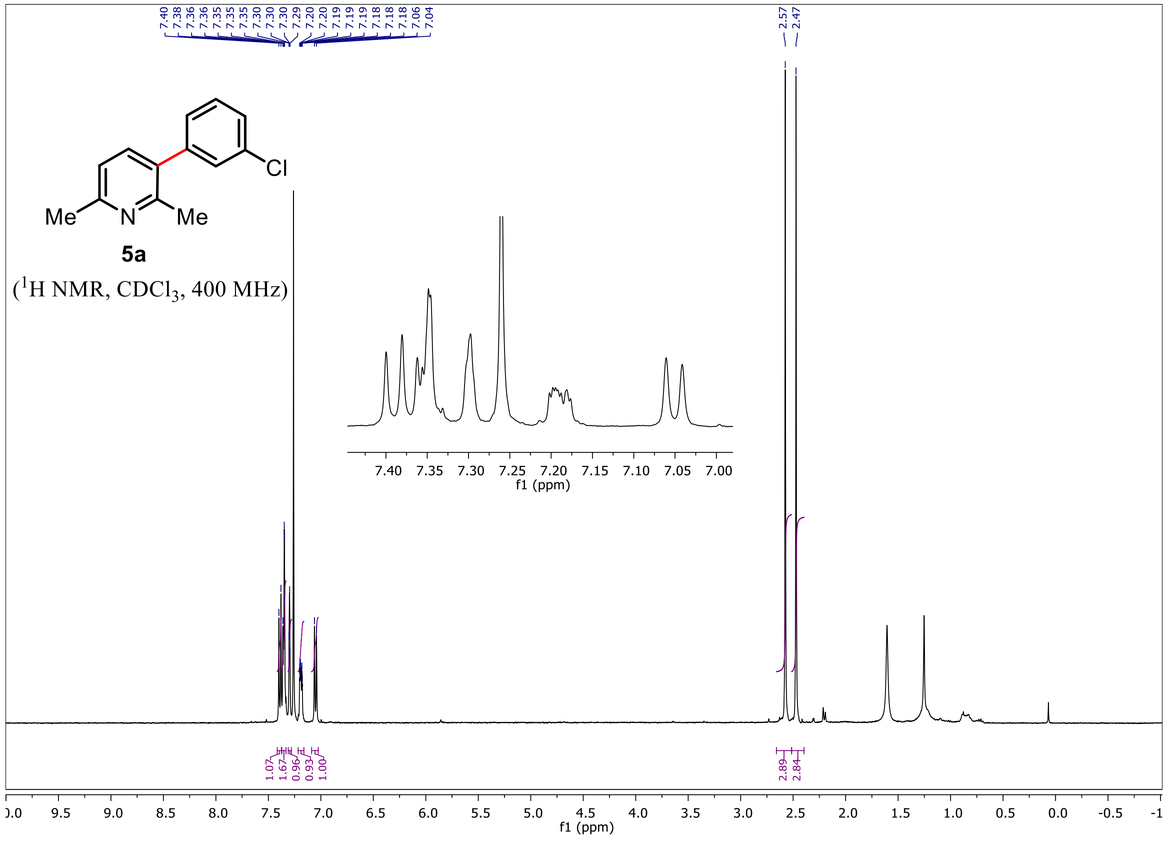
Cl
Me
N
Me
5a
($^{1}$H NMR, CDCl$_3$, 400 MHz)
f1 (ppm)

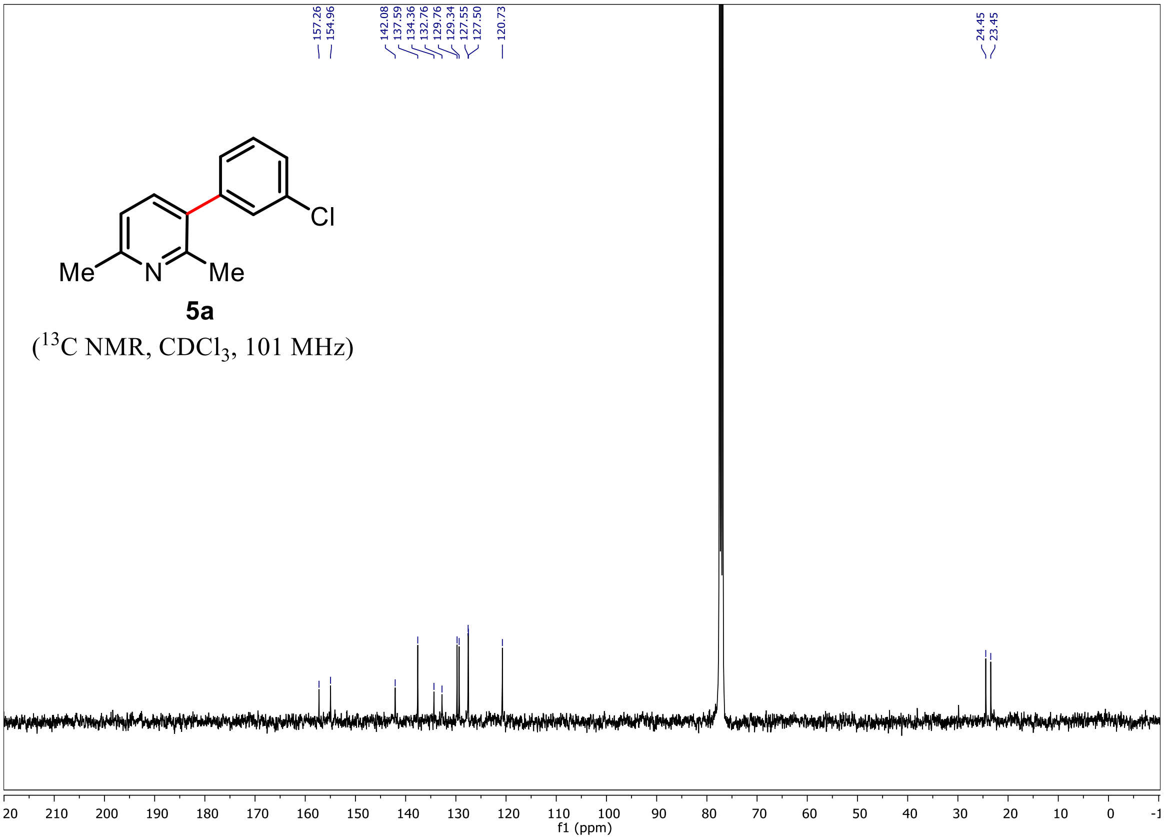
157.26
154.96
142.08
137.59
134.36
132.76
129.76
129.34
127.55
127.50
120.73
24.45
23.45
Cl
Me
N
Me
5a
(13C NMR, CDCl3, 101 MHz)
f1 (ppm)

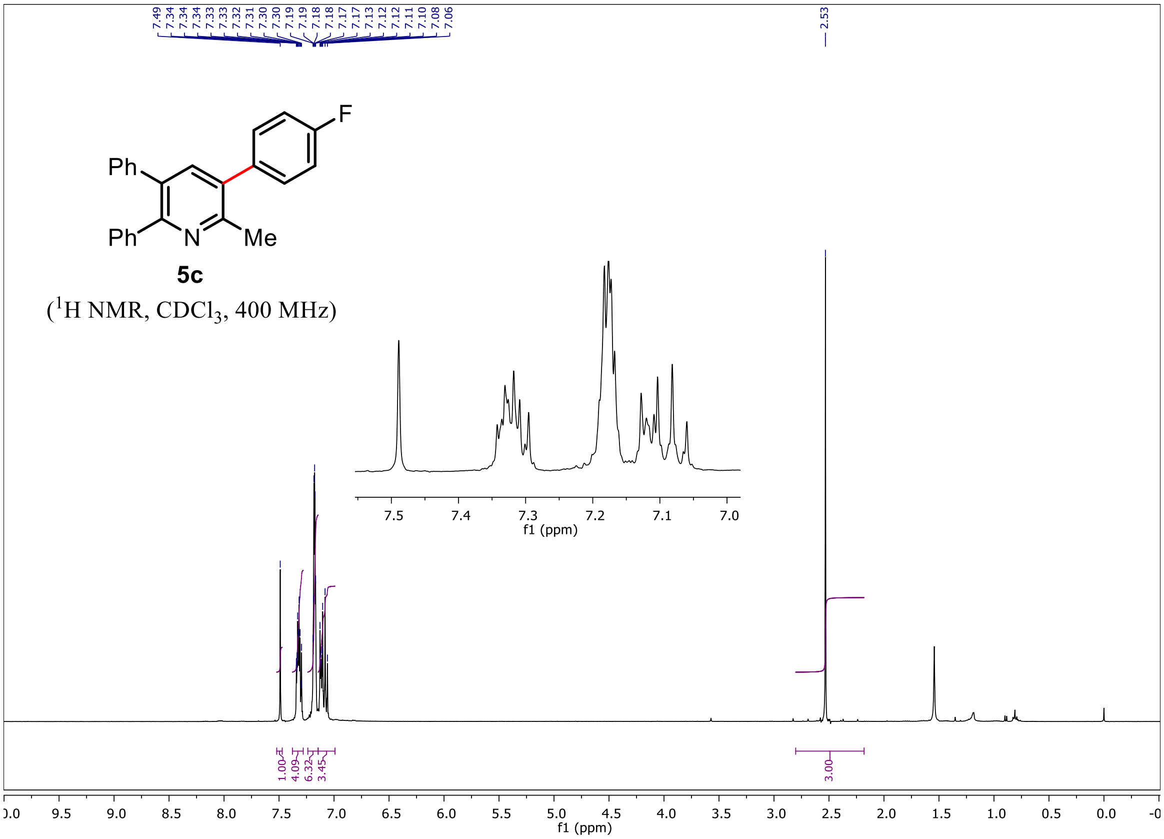
7.49
7.34
7.34
7.34
7.33
7.33
7.32
7.31
7.30
7.30
7.19
7.19
7.18
7.18
7.17
7.17
7.13
7.12
7.12
7.11
7.10
7.08
7.06
2.53
F
Ph
Ph
N
Me
5c
(1H NMR, CDCl3, 400 MHz)
1.00
4.09
6.32
3.45
3.00
f1 (ppm)

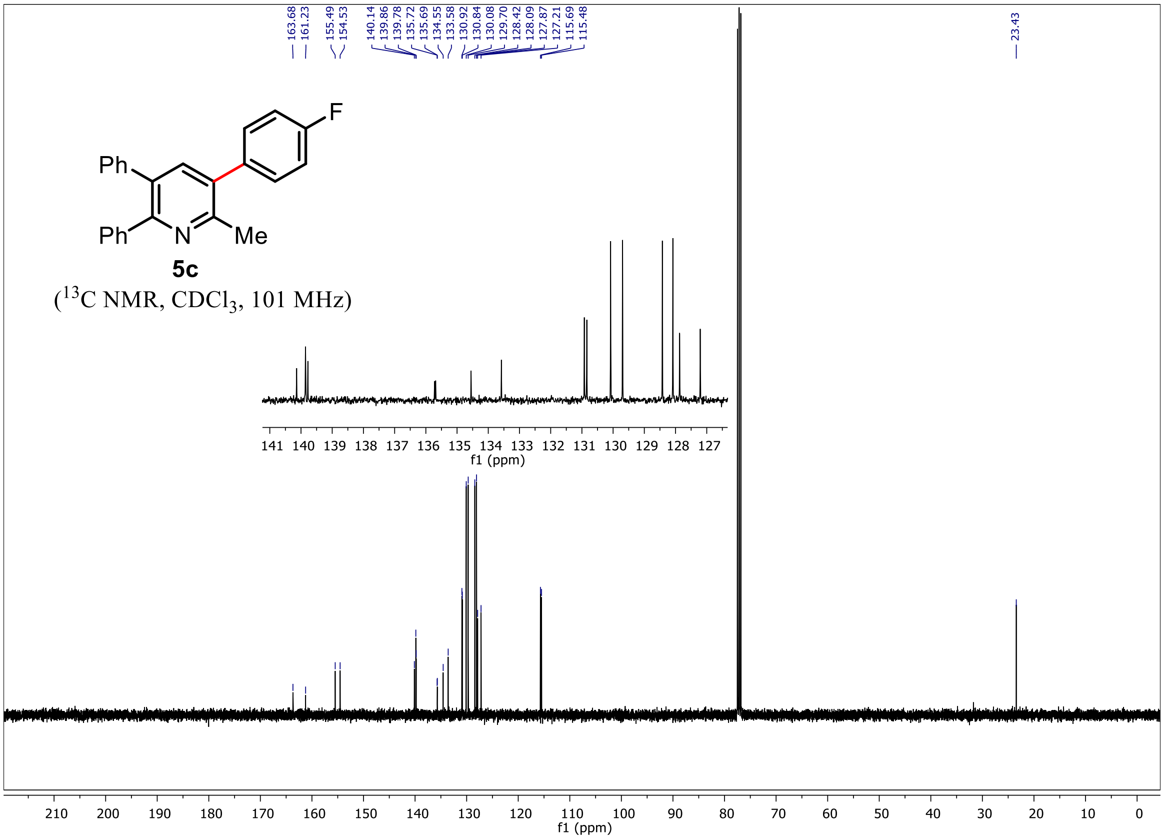
163.68
161.23
155.49
154.53
140.14
139.86
139.78
135.72
135.69
134.55
133.58
130.92
130.84
130.08
129.70
128.42
128.09
127.87
127.21
115.69
115.48
23.43
Ph
Ph
N
Me
F
5c
($^{13}$C NMR, $CDCl_3$, 101 MHz)
f1 (ppm)

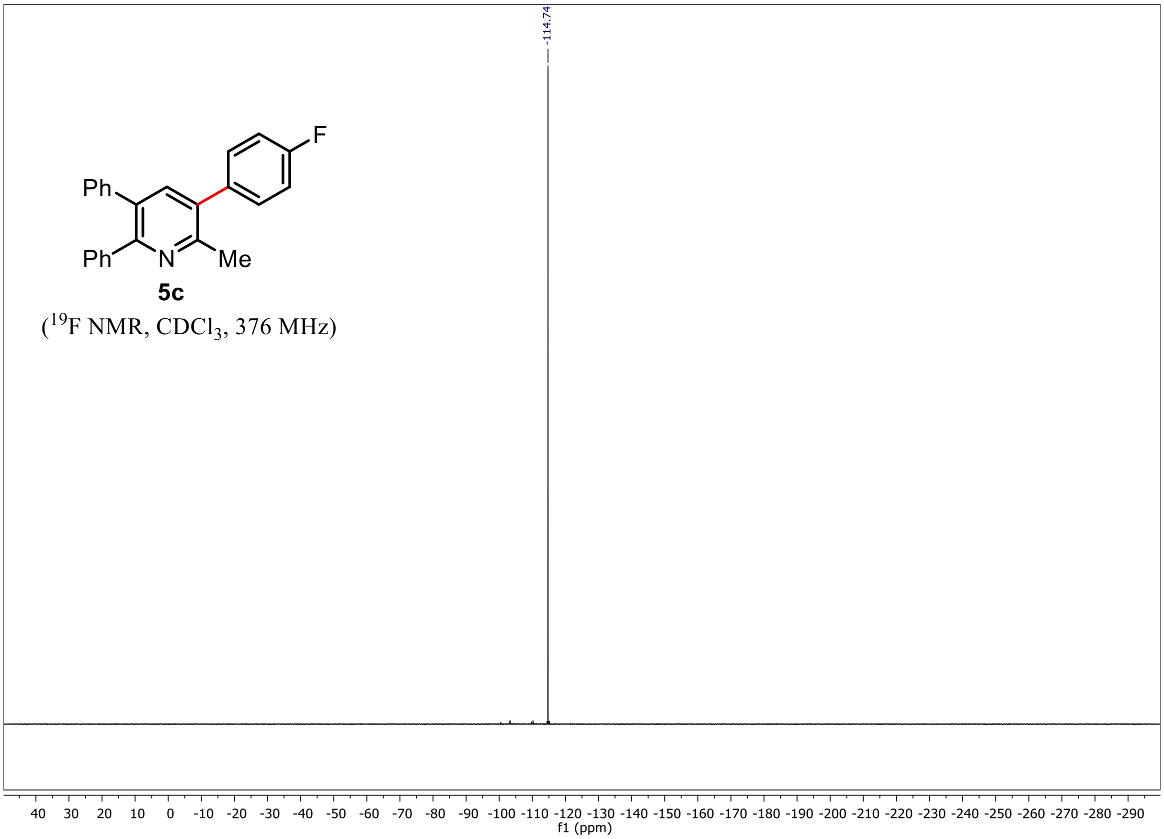
-114.74
Ph
Ph
N
Me
F
5c
($^{19}$F NMR, $CDCl_3$, 376 MHz)
f1 (ppm)

1D NOE NMR spectrum of **5c**, 500 MHz, benzene-*d6*

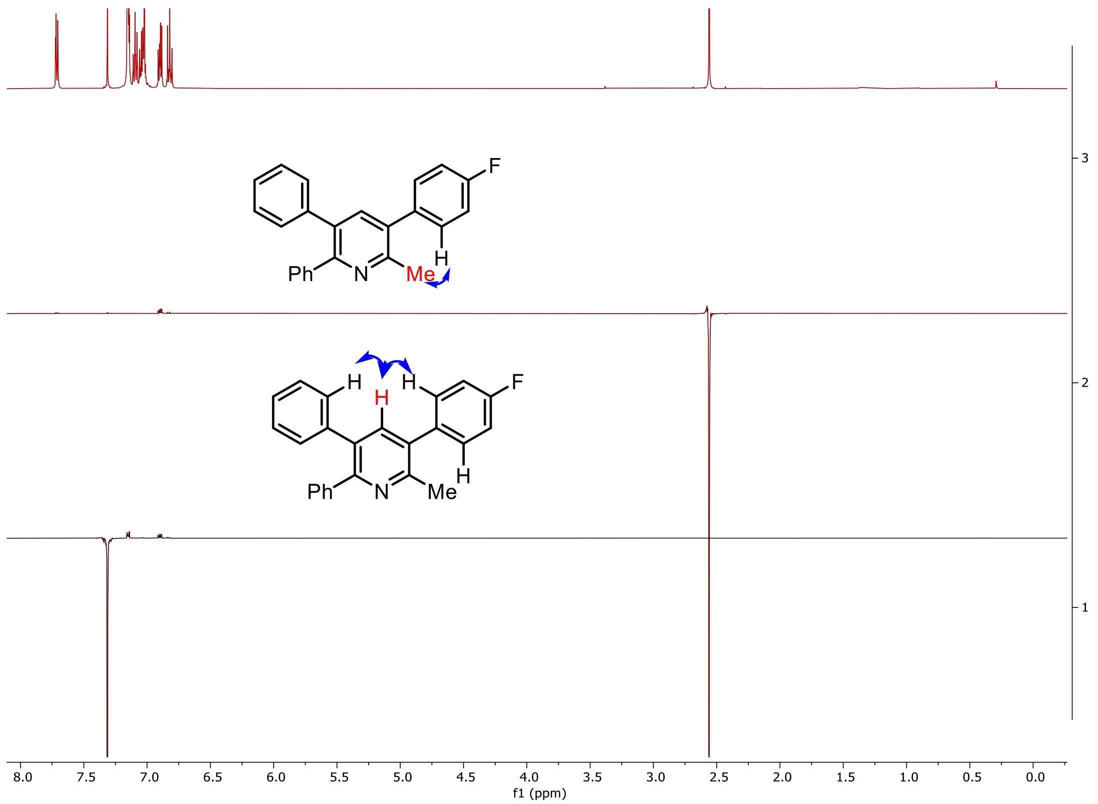

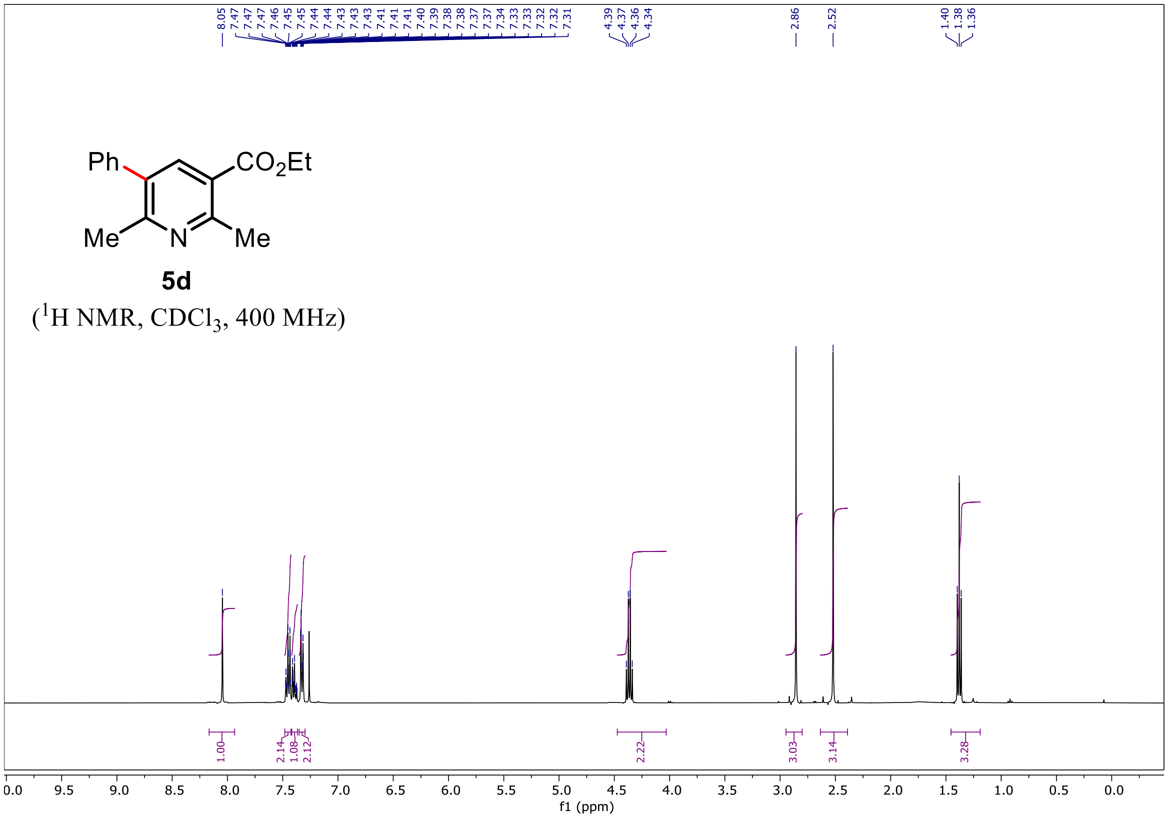

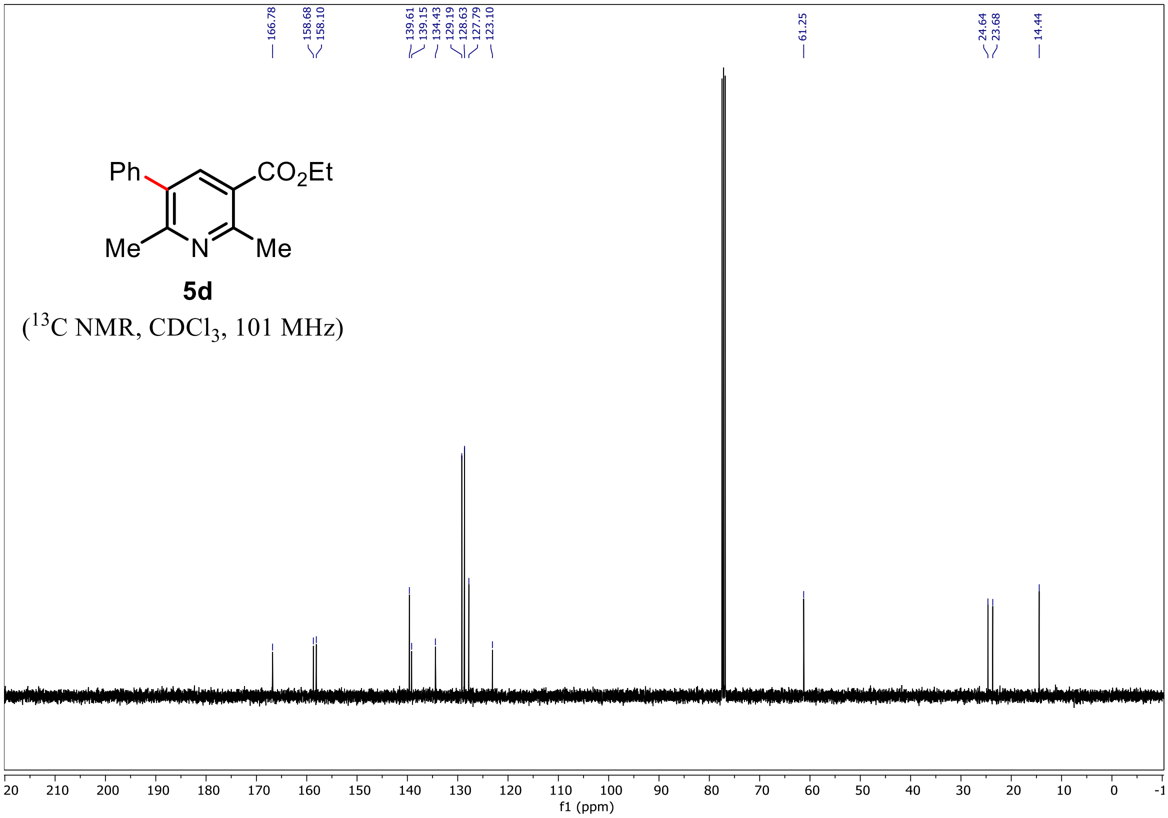


1D NOE NMR spectrum of **5d**, 500 MHz, $CDCl_3$

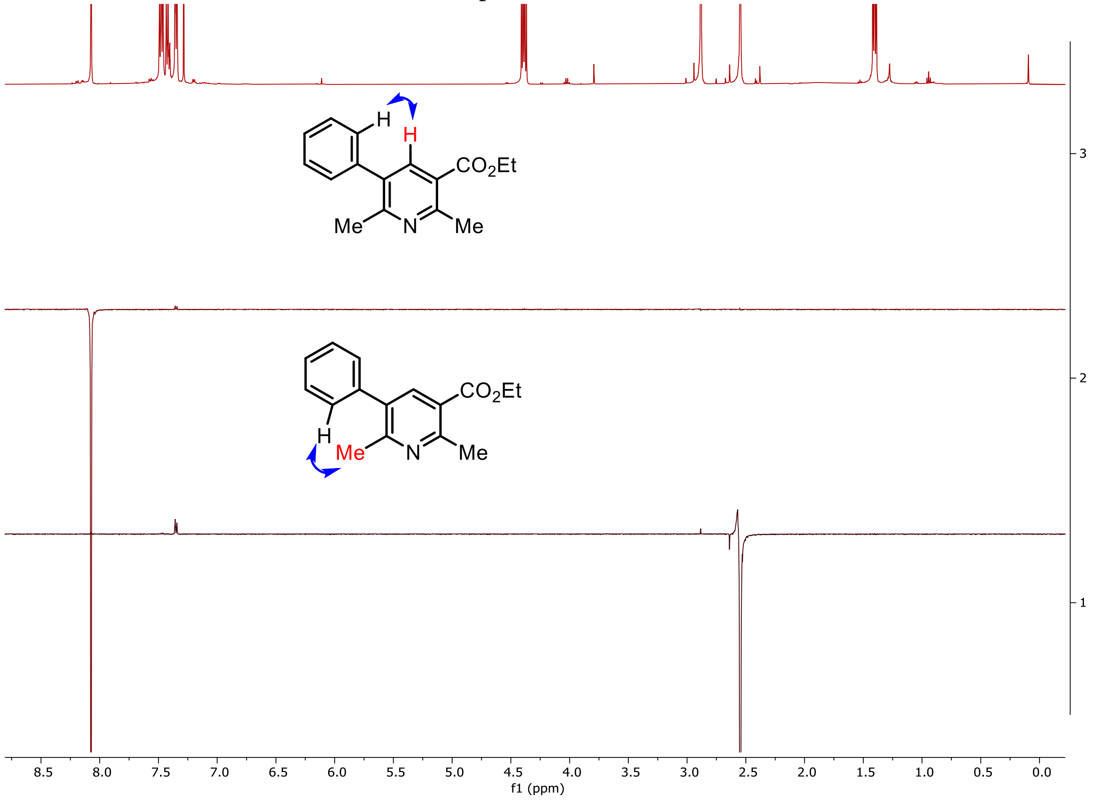

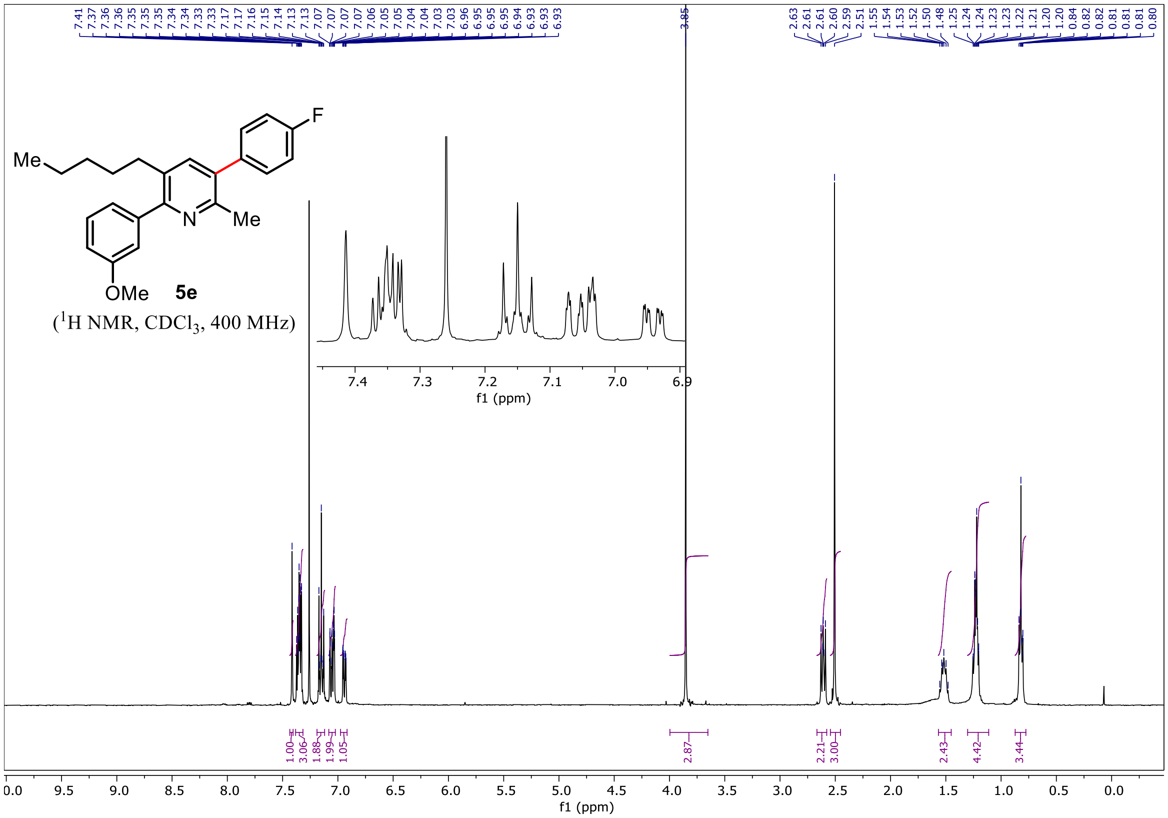
Me
F
N
Me
OMe
5e
(¹H NMR, CDCl₃, 400 MHz)
f1 (ppm)

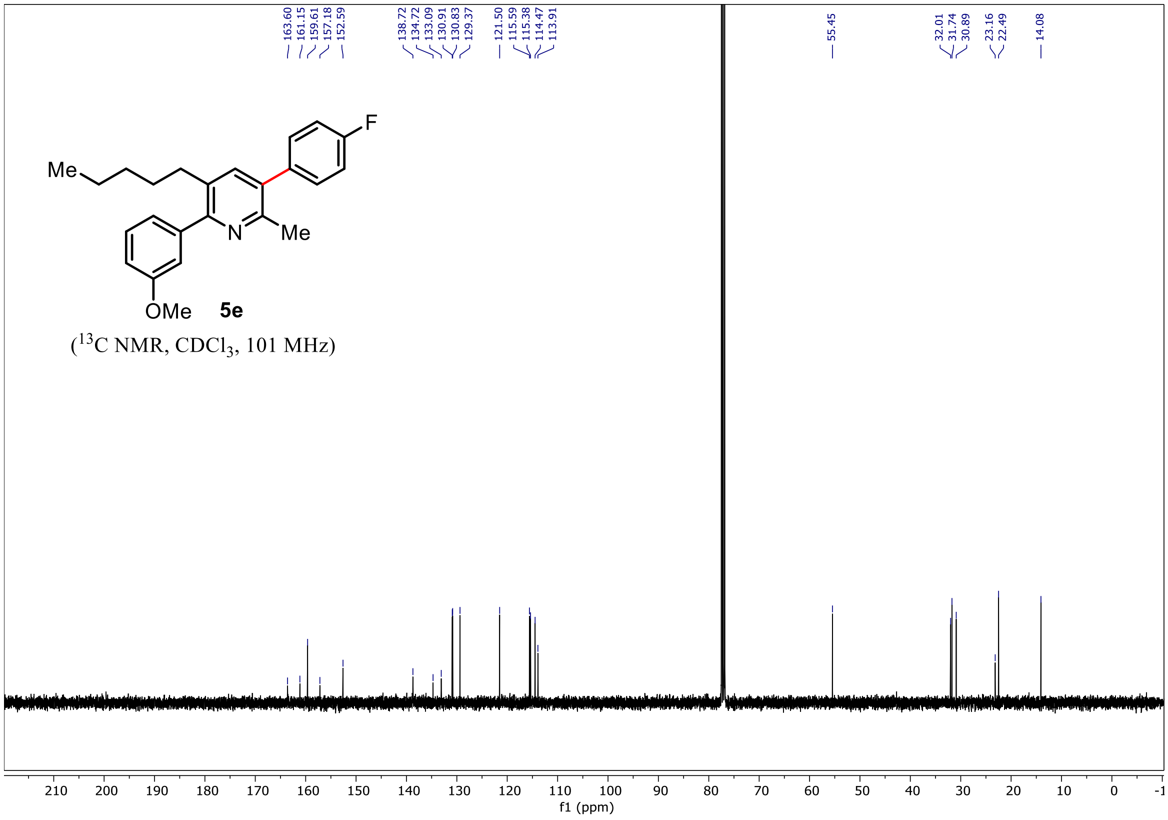
163.60
161.15
159.61
157.18
152.59
138.72
134.72
133.09
130.91
130.83
129.37
121.50
115.59
115.38
114.47
113.91
55.45
32.01
31.74
30.89
23.16
22.49
14.08
Me
F
N
Me
OMe
5e
(¹³C NMR, CDCl₃, 101 MHz)
f1 (ppm)

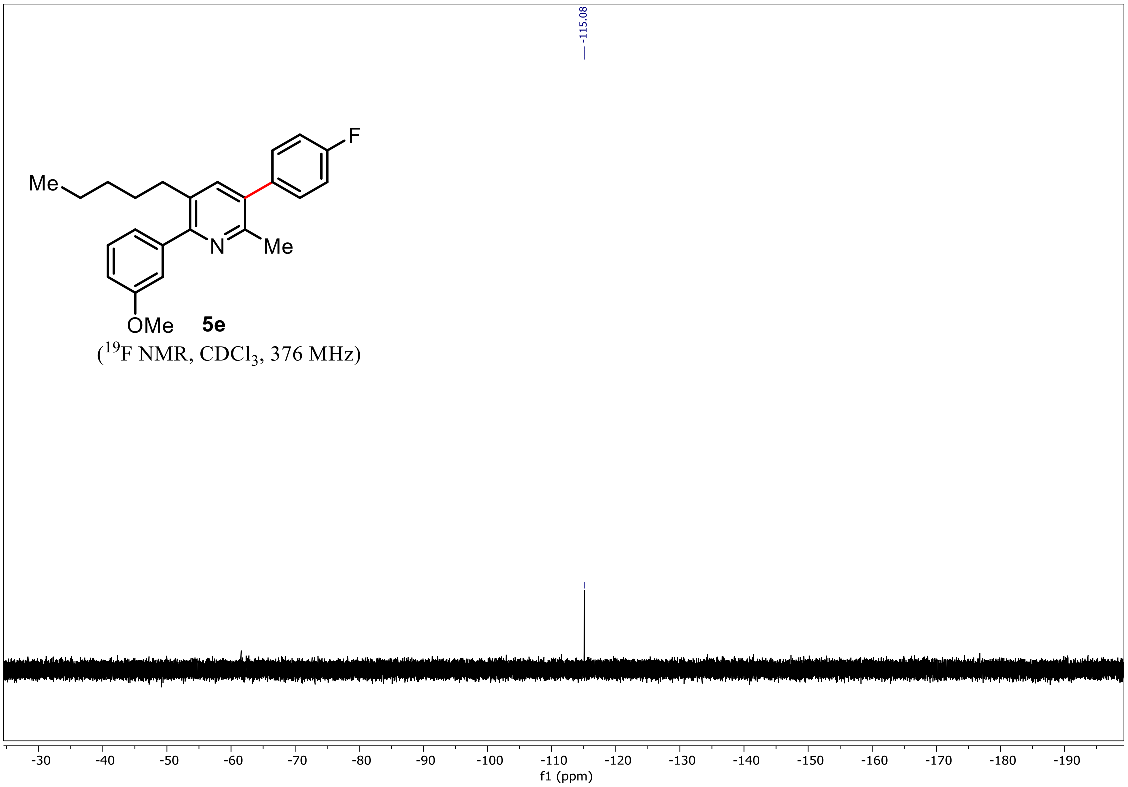


1D NOE NMR spectrum of **5e**, 500 MHz, $CDCl_3$

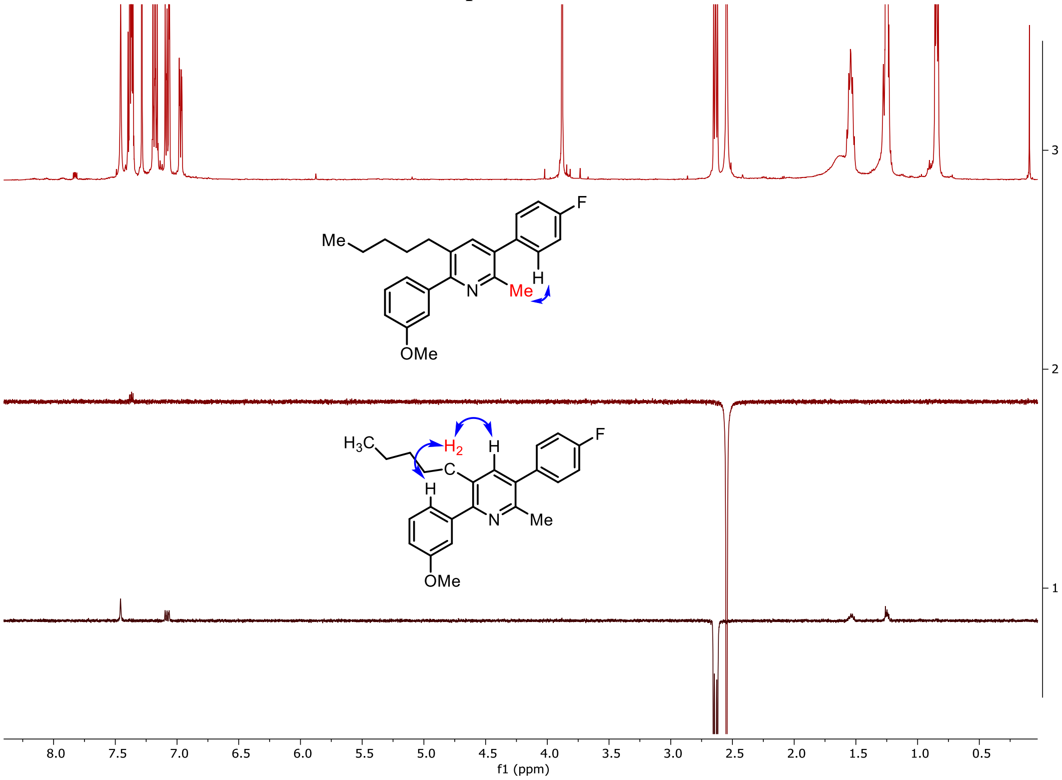

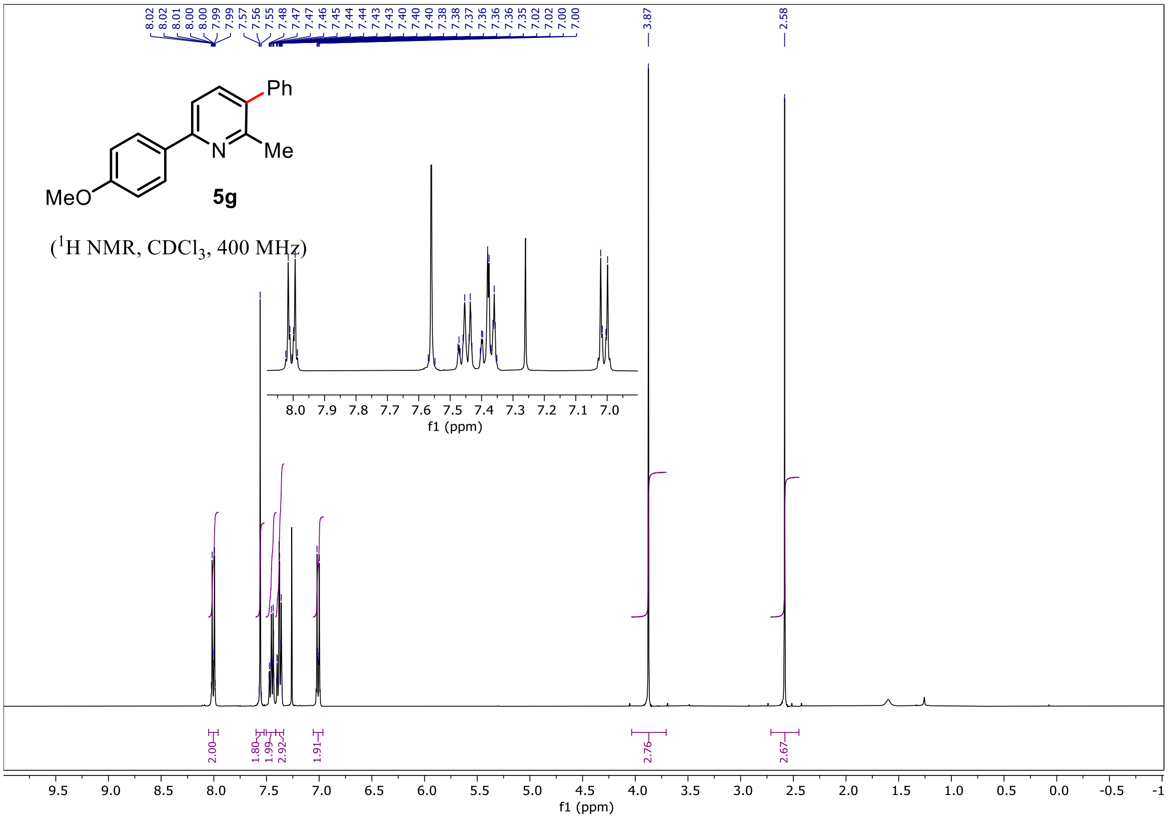
8.02
8.02
8.01
8.00
8.00
7.99
7.99
7.57
7.56
7.55
7.48
7.47
7.47
7.46
7.45
7.44
7.44
7.43
7.43
7.40
7.40
7.38
7.38
7.37
7.36
7.36
7.36
7.35
7.02
7.02
7.00
7.00
3.87
2.58
Ph
N
Me
MeO
5g
(1H NMR, CDCl3, 400 MHz)
2.00
1.80
1.99
2.92
1.91
2.76
2.67
f1 (ppm)

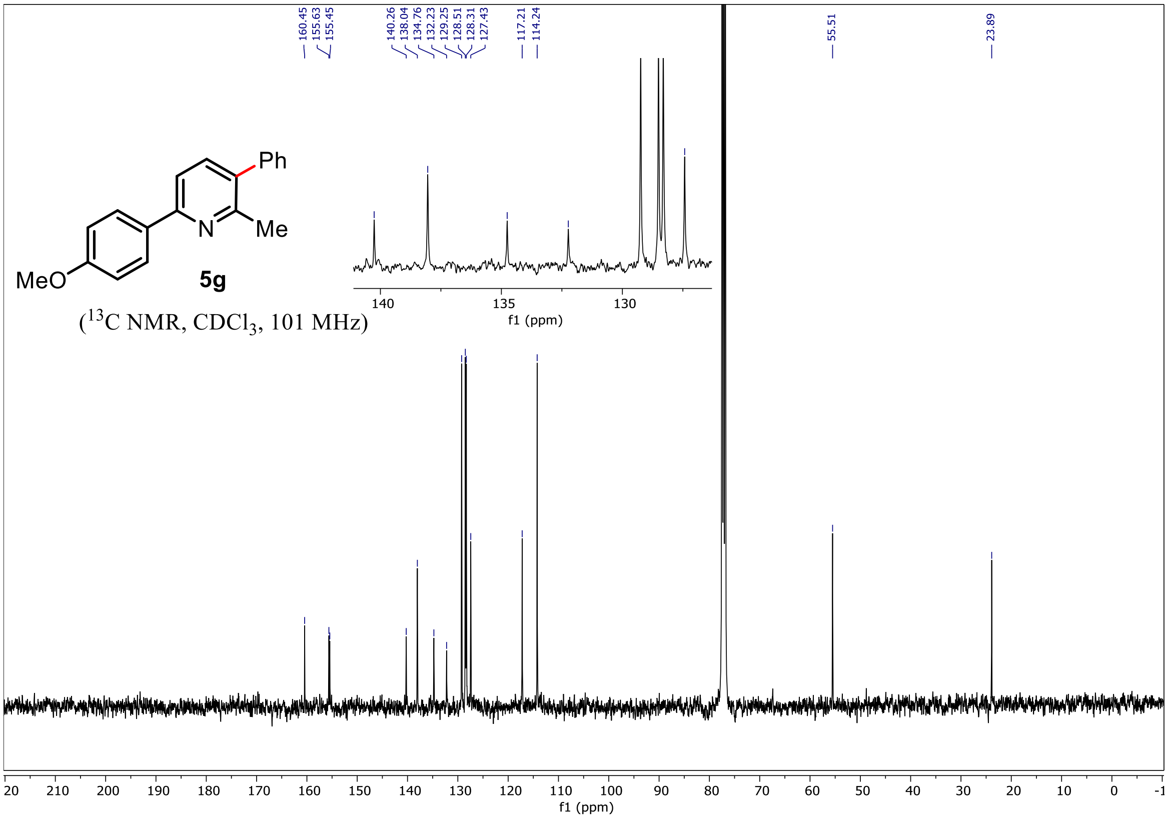
160.45
155.63
155.45
140.26
138.04
134.76
132.23
129.25
128.51
128.31
127.43
117.21
114.24
55.51
23.89
Ph
N
Me
MeO
5g
(13C NMR, CDCl3, 101 MHz)
f1 (ppm)

1D NOE NMR spectrum of **5g**, 500 MHz, $C_6D_6$

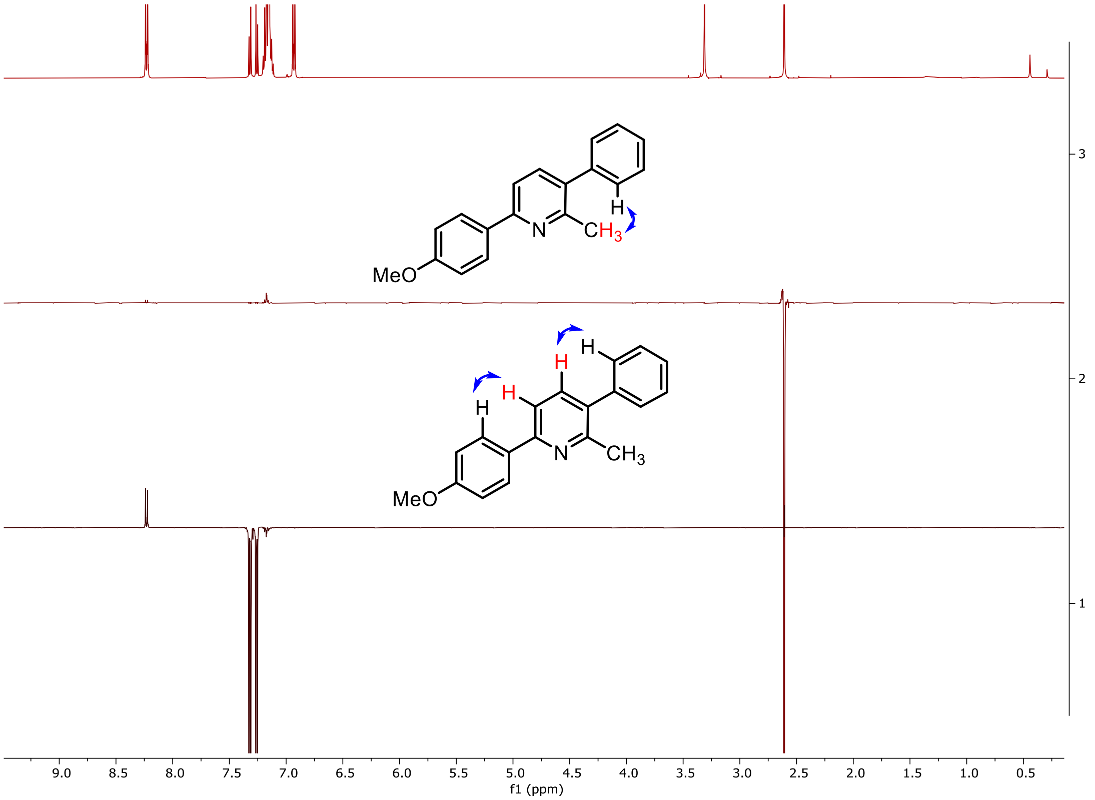

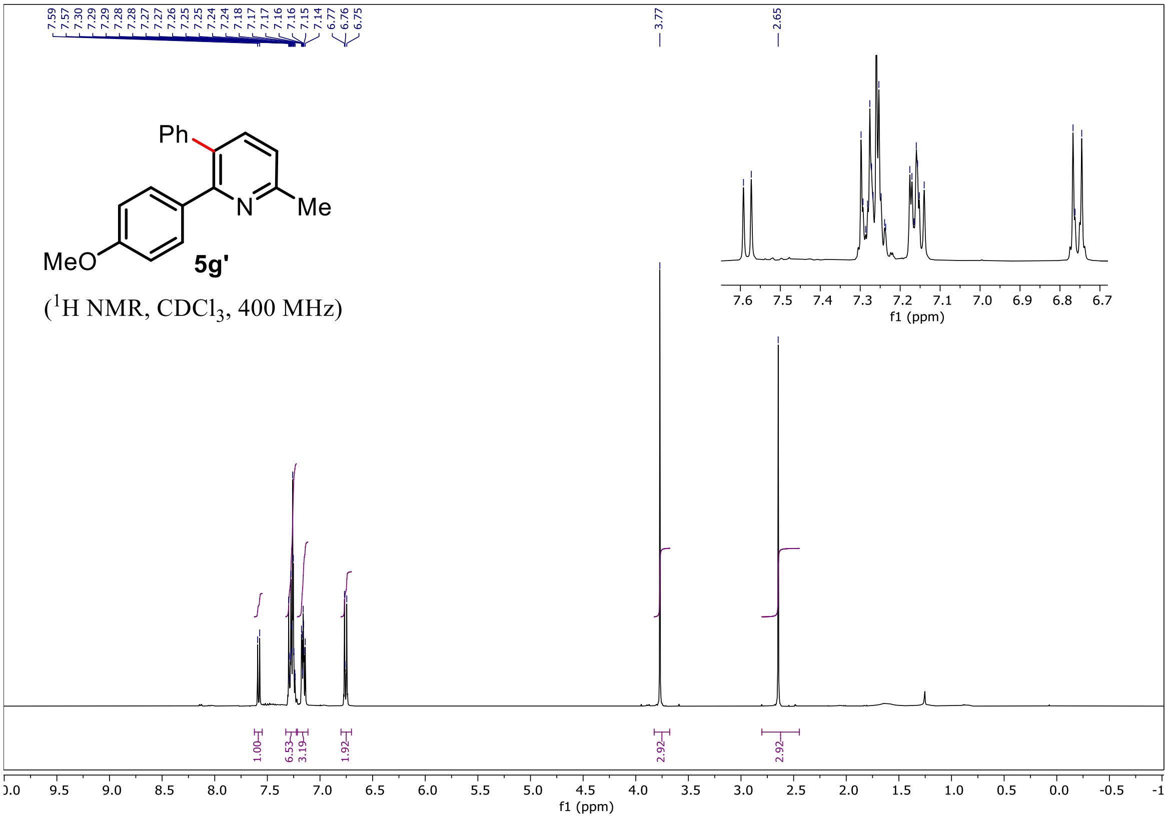

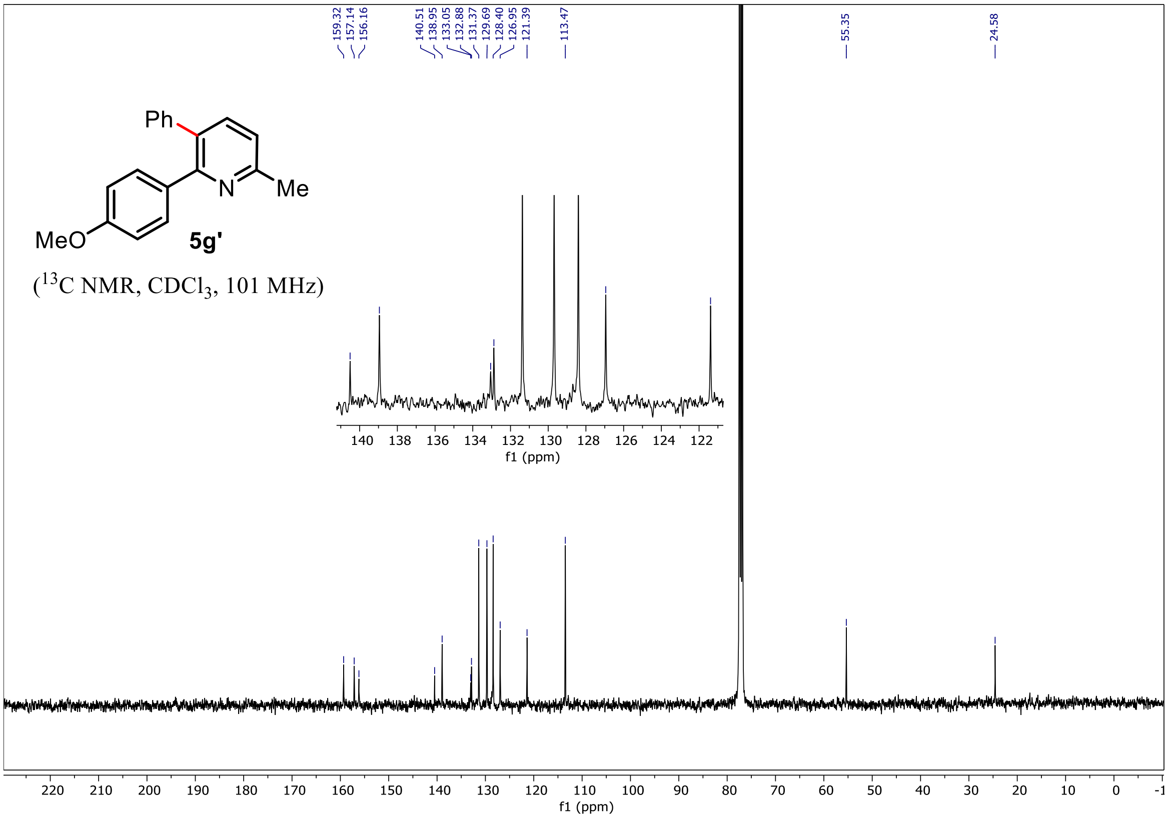


1D NOE NMR spectrum of **5g'**, 500 MHz, $C_6D_6$

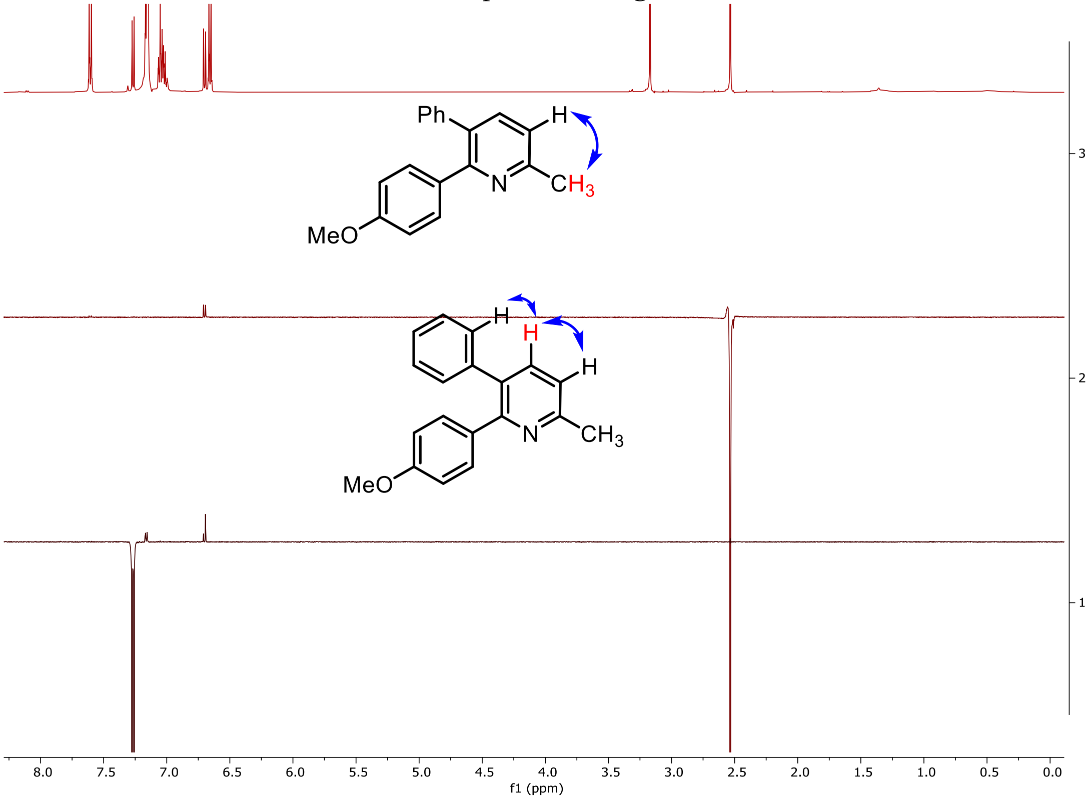

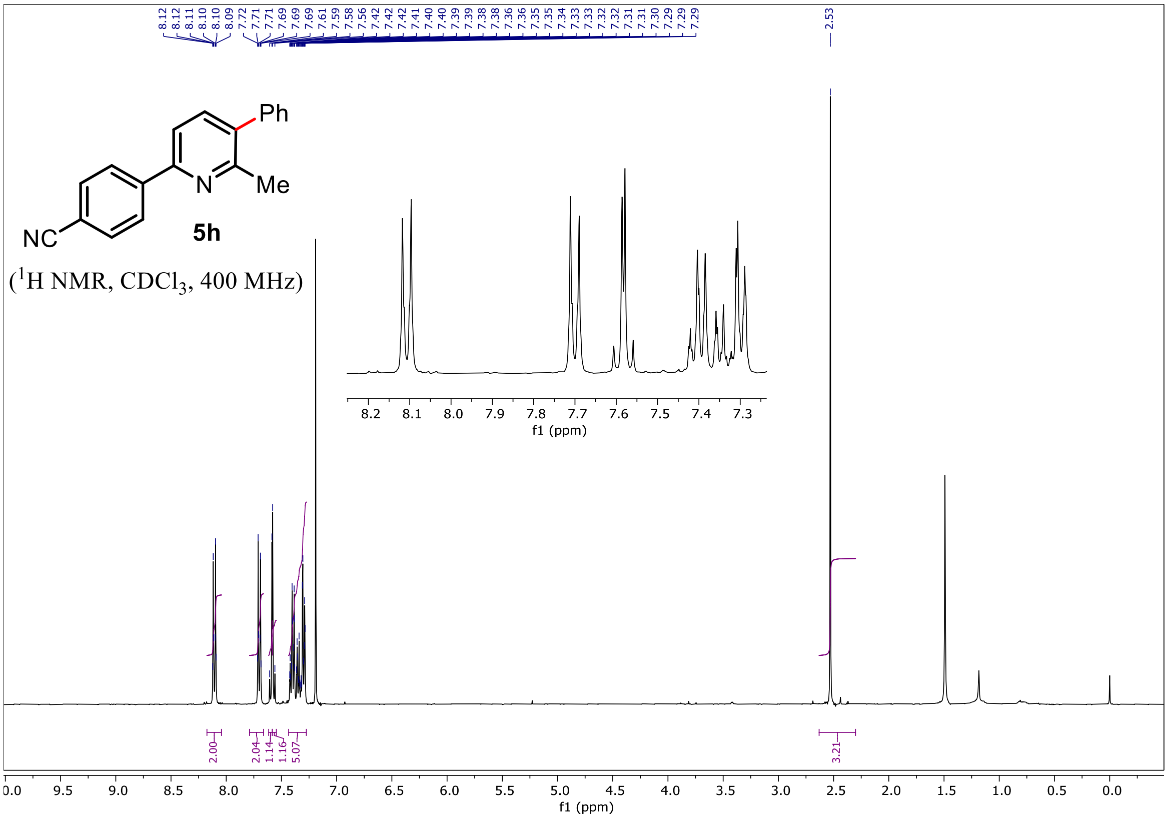
Ph
N
Me
NC
5h
(1H NMR, CDCl3, 400 MHz)
f1 (ppm)

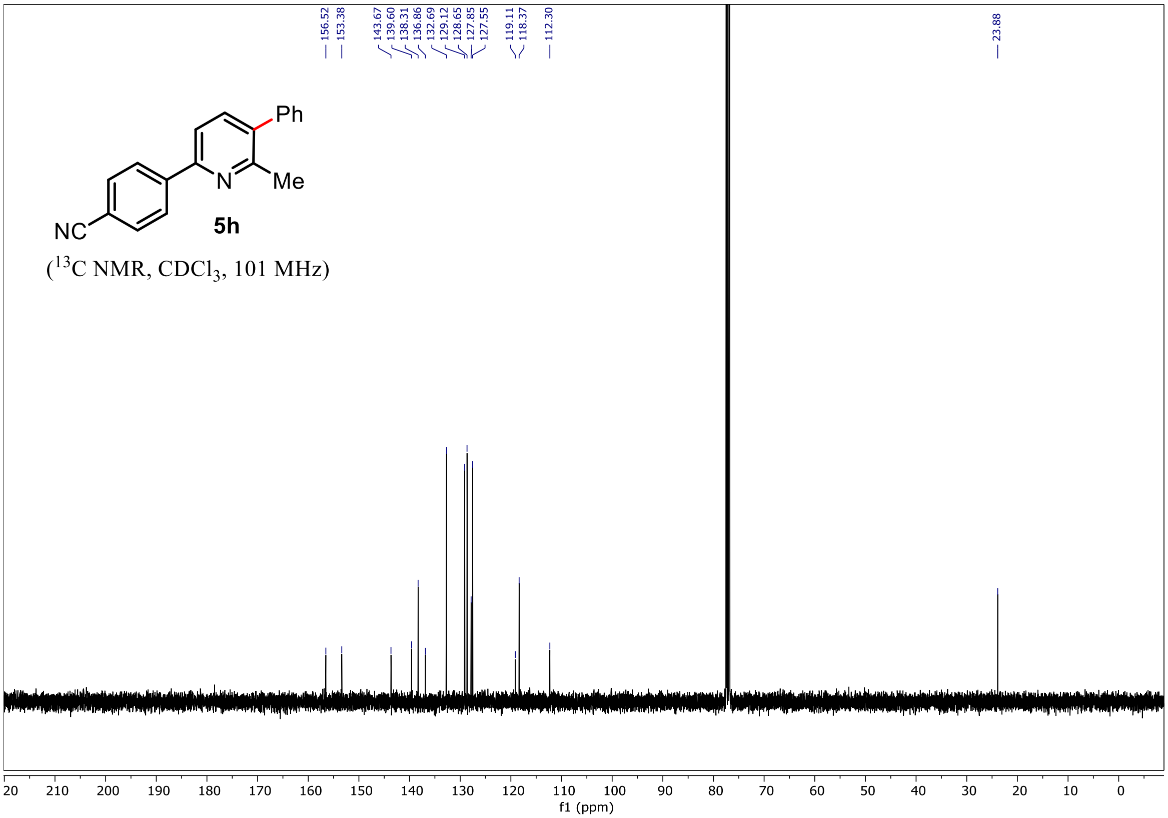
Ph
N
Me
NC
5h
(13C NMR, CDCl3, 101 MHz)
f1 (ppm)

1D NOE NMR spectrum of **5h**, 500 MHz, $CDCl_3$

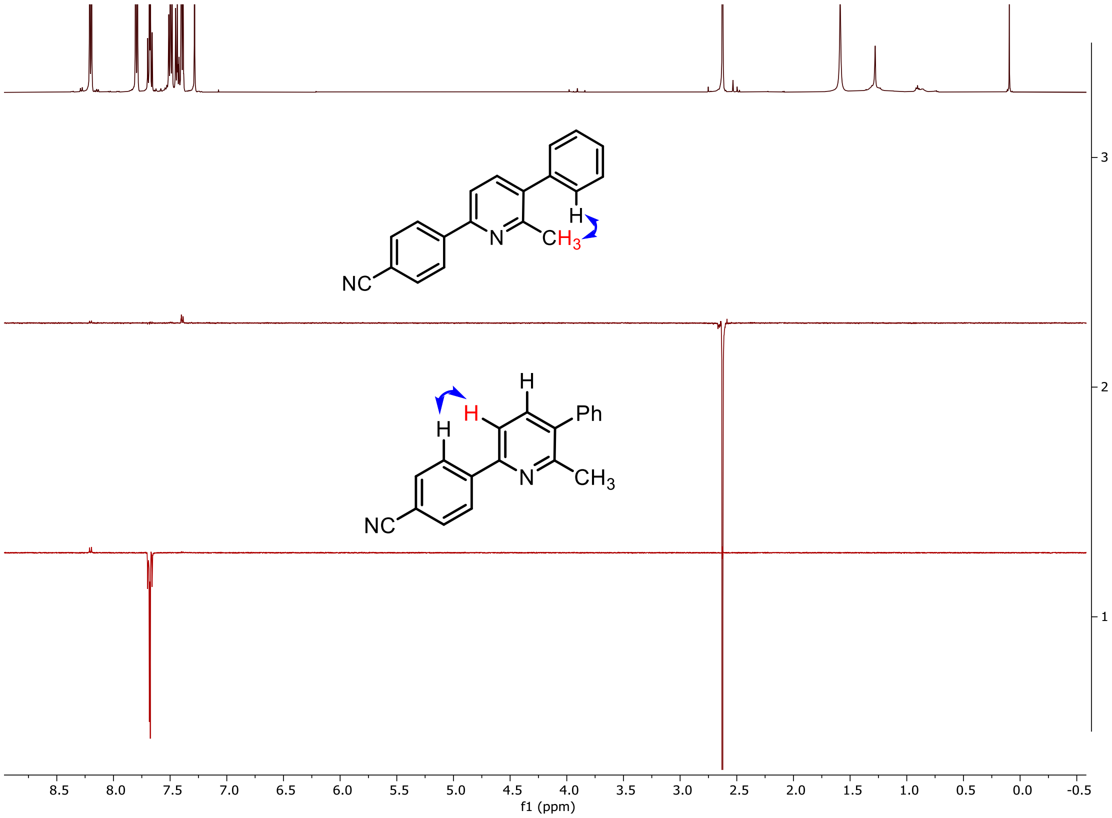


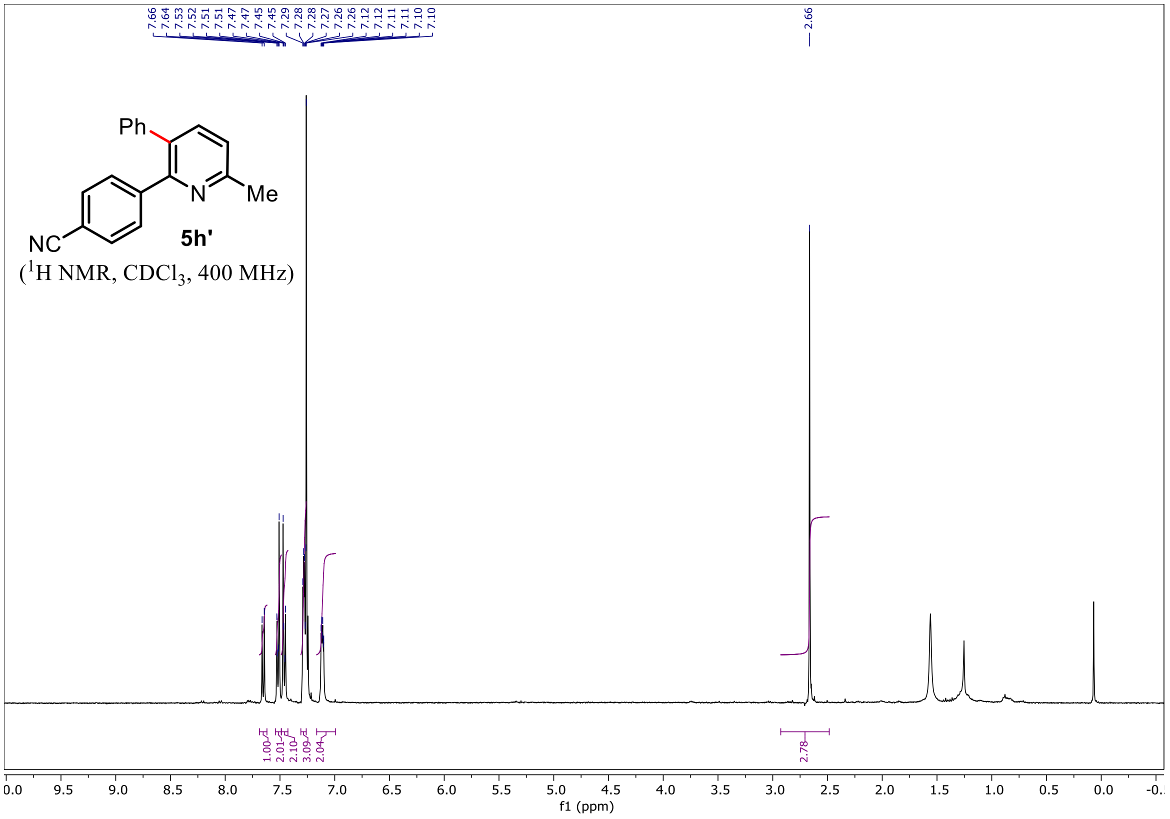

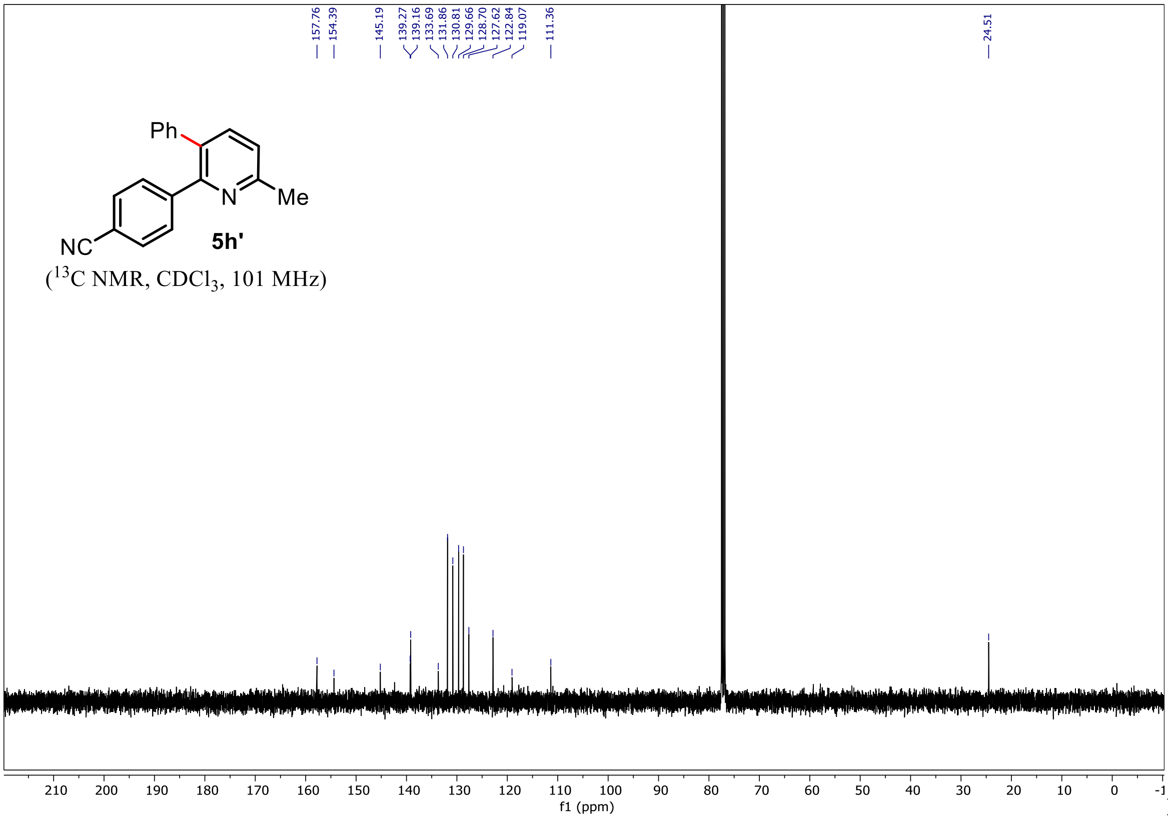


1D

NOE NMR spectrum of **5h’**, 500 MHz, $CDCl_3$

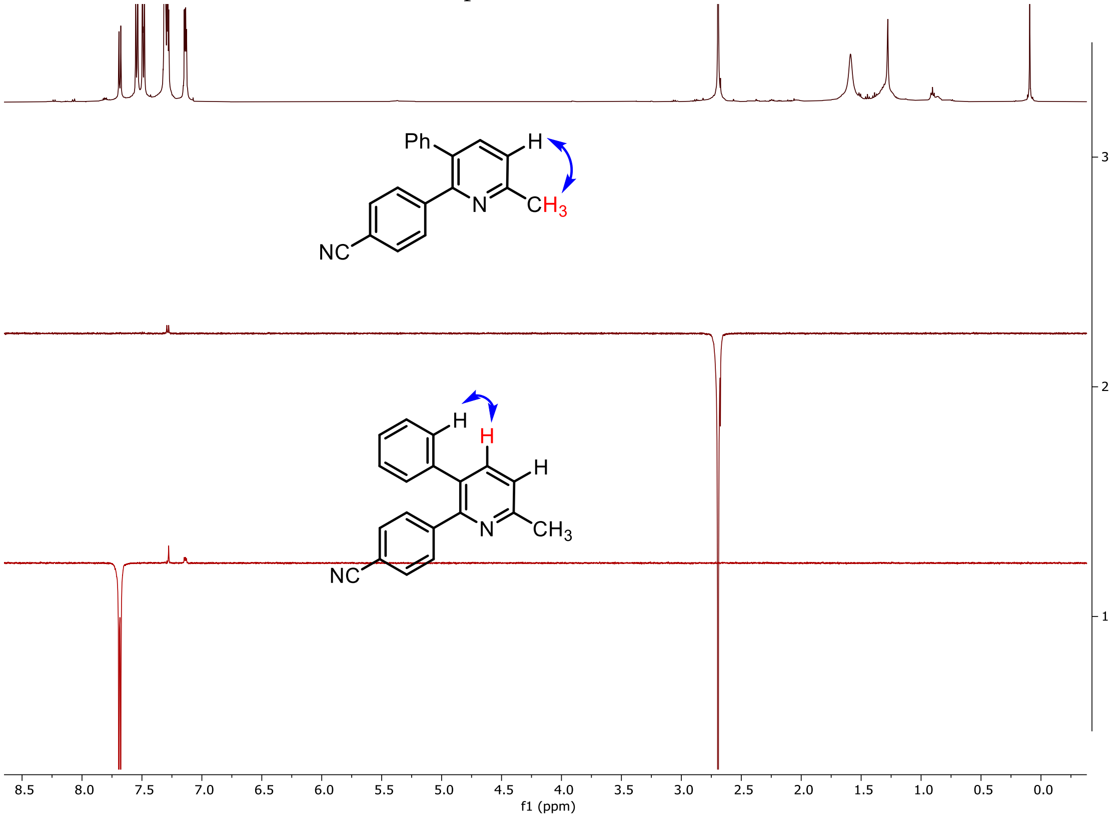

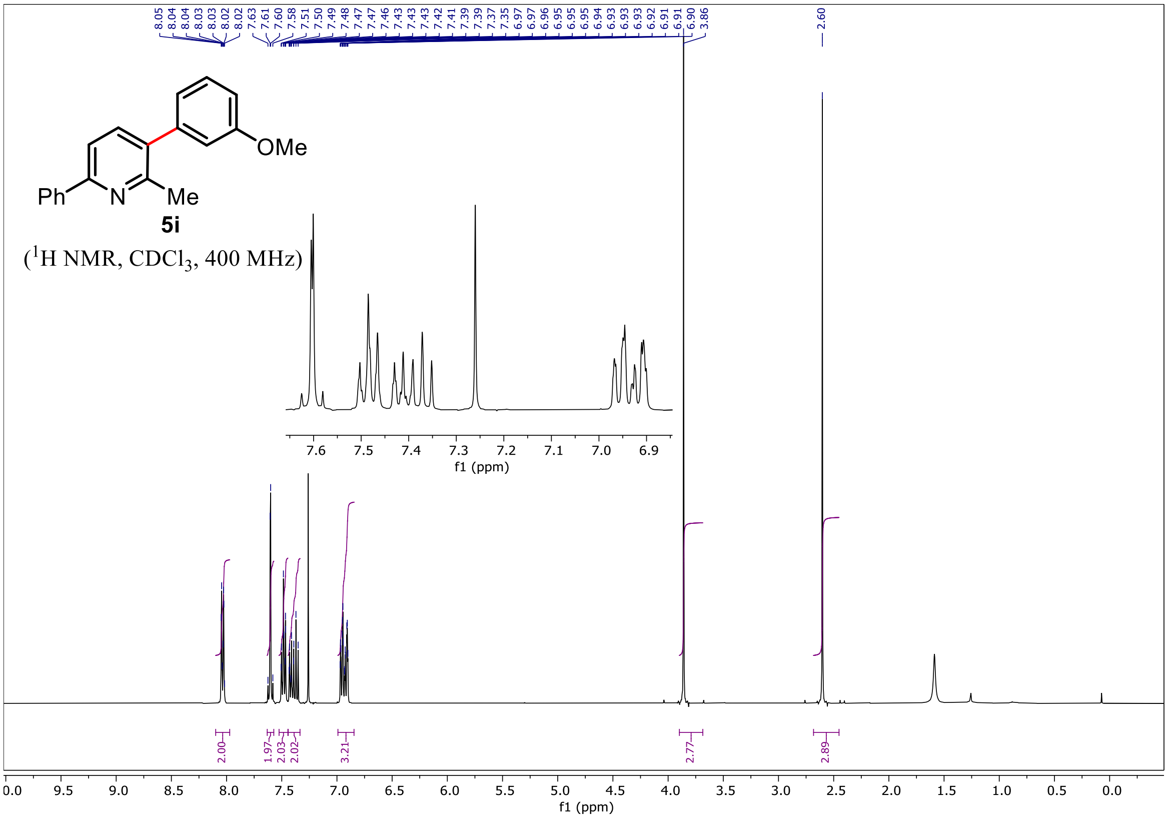
8.05
8.04
8.04
8.03
8.03
8.02
8.02
7.63
7.61
7.60
7.58
7.51
7.50
7.49
7.48
7.47
7.47
7.46
7.43
7.43
7.43
7.42
7.41
7.39
7.39
7.37
7.35
6.97
6.97
6.96
6.95
6.95
6.95
6.94
6.93
6.93
6.93
6.92
6.91
6.91
6.90
3.86
2.60
OMe
Ph
N
Me
5i
(¹H NMR, CDCl3, 400 MHz)
f1 (ppm)
2.00
1.97
2.03
2.02
3.21
2.77
2.89

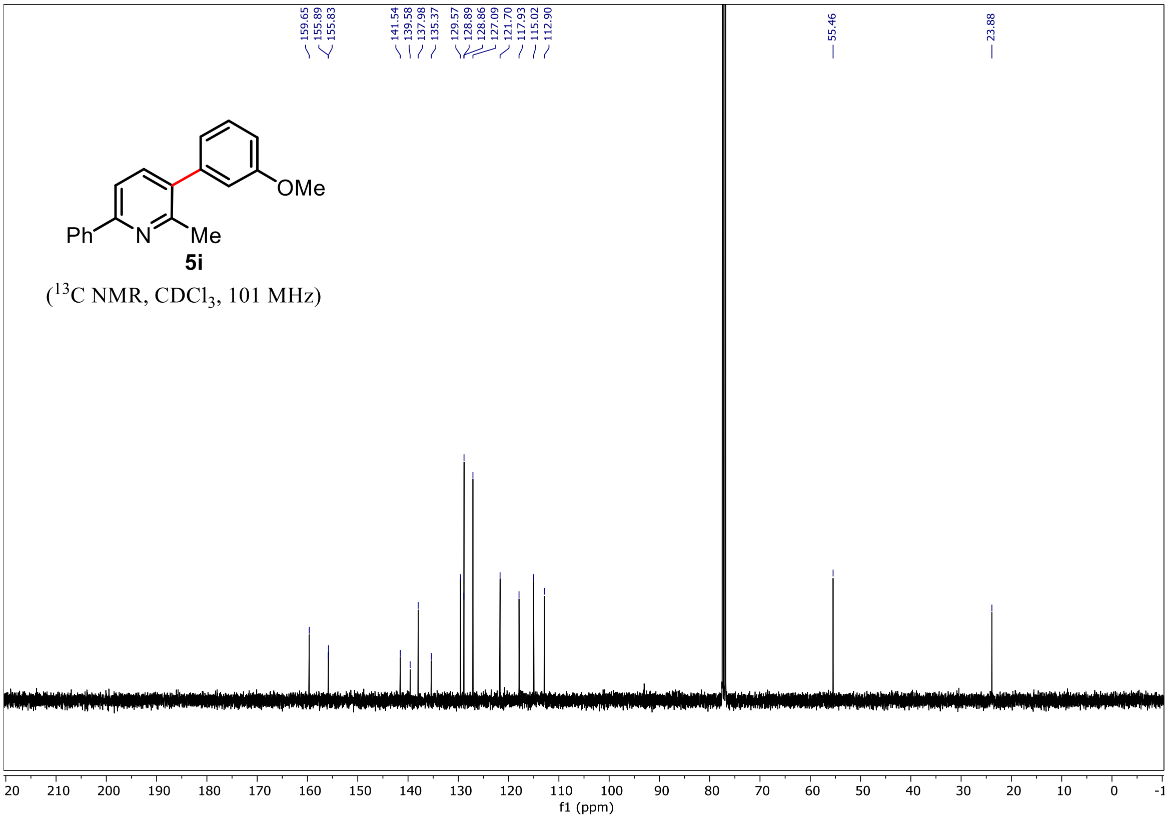
159.65
155.89
155.83
141.54
139.58
137.98
135.37
129.57
128.89
128.86
127.09
121.70
117.93
115.02
112.90
55.46
23.88
OMe
Ph
N
Me
5i
(¹³C NMR, CDCl3, 101 MHz)
f1 (ppm)

1D NOE NMR spectrum of **5i**, 500 MHz, $CDCl_3$

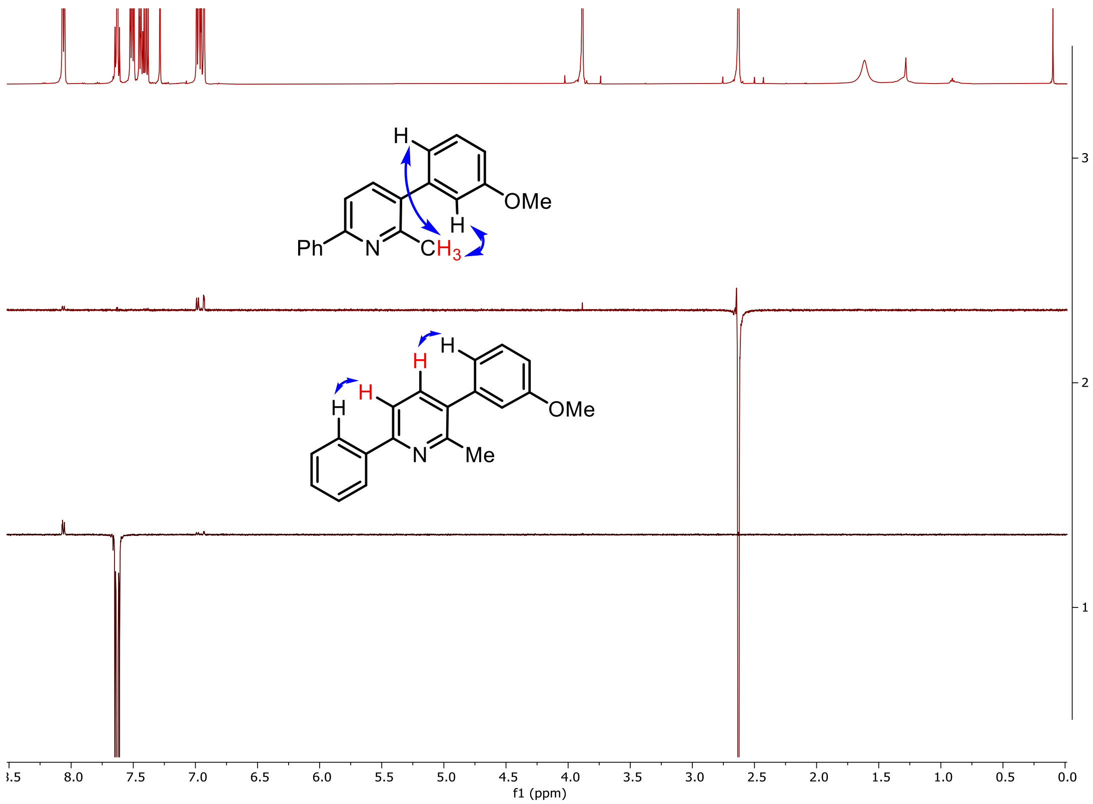

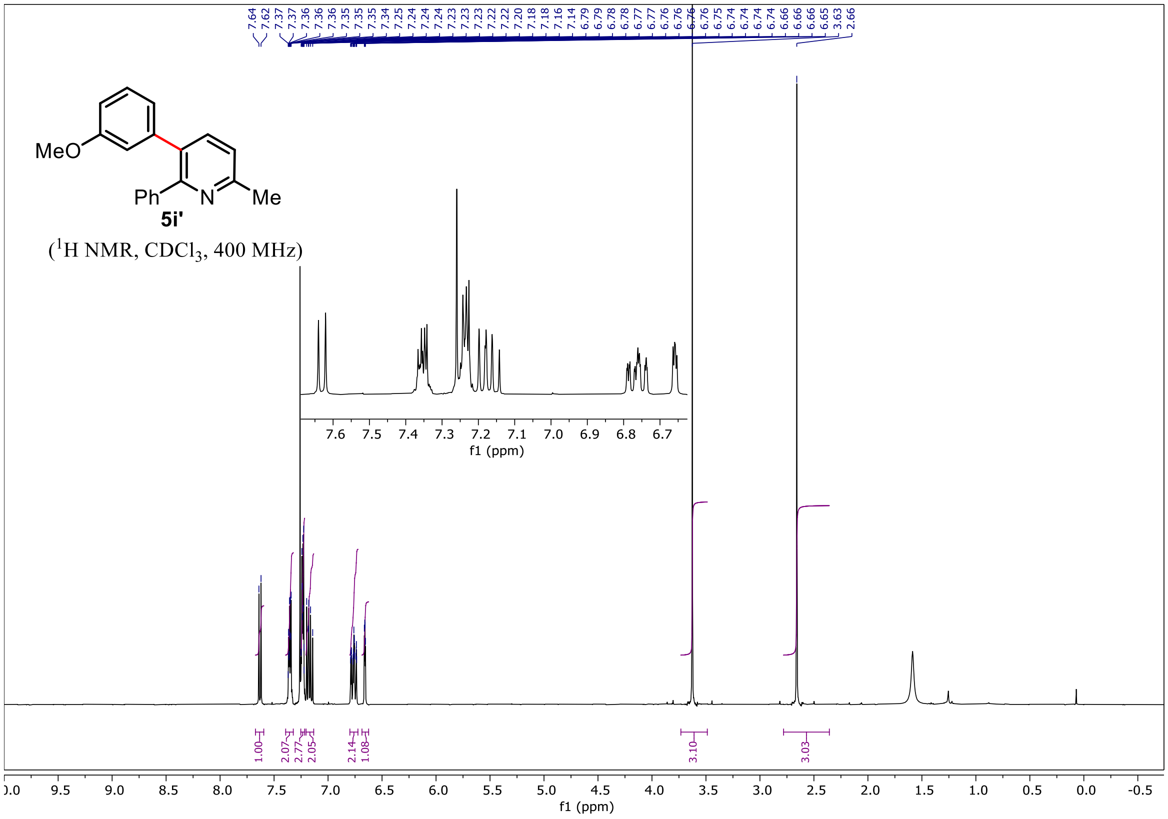

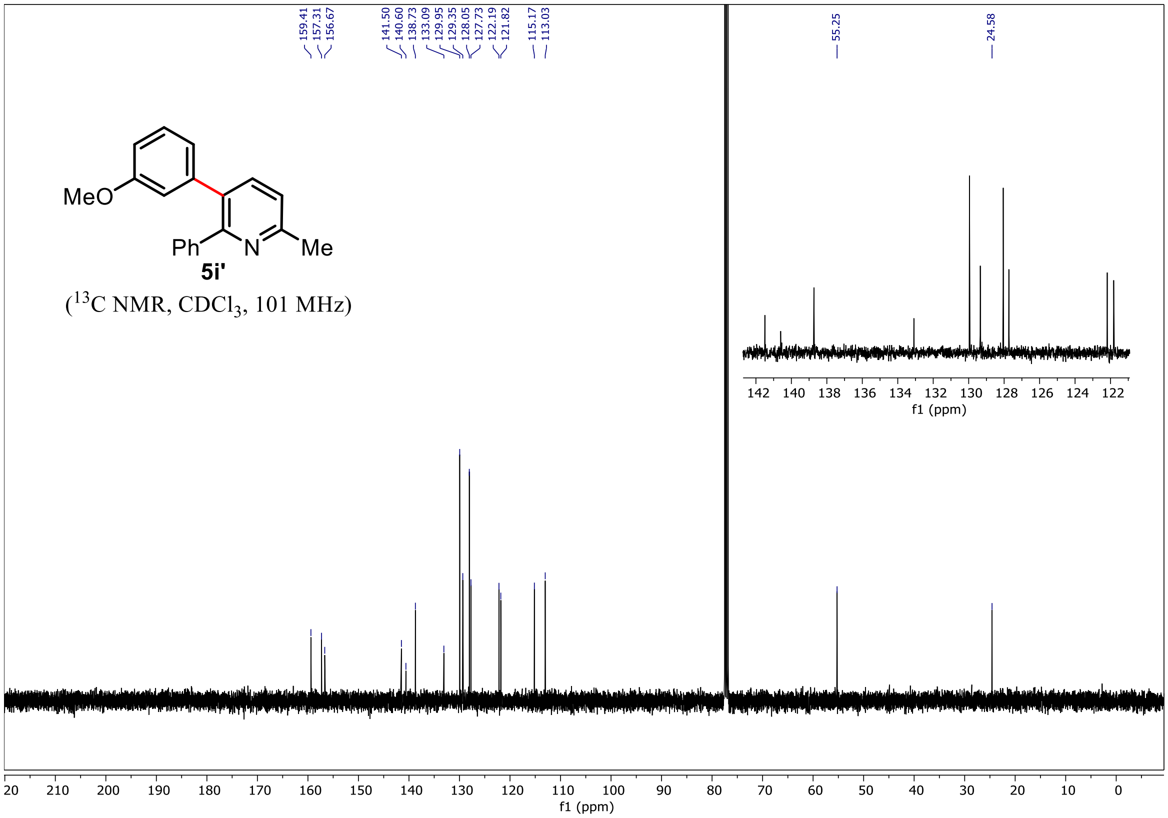


1D NOE NMR spectrum of **5i'**, 500 MHz, $CDCl_3$

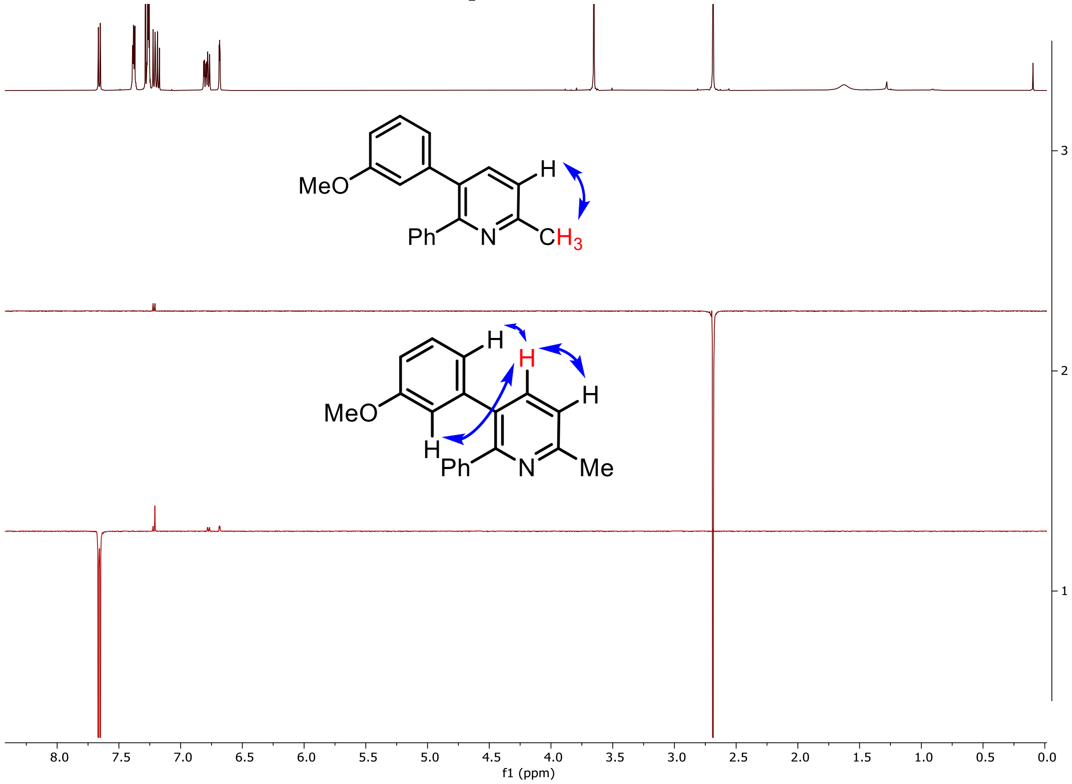

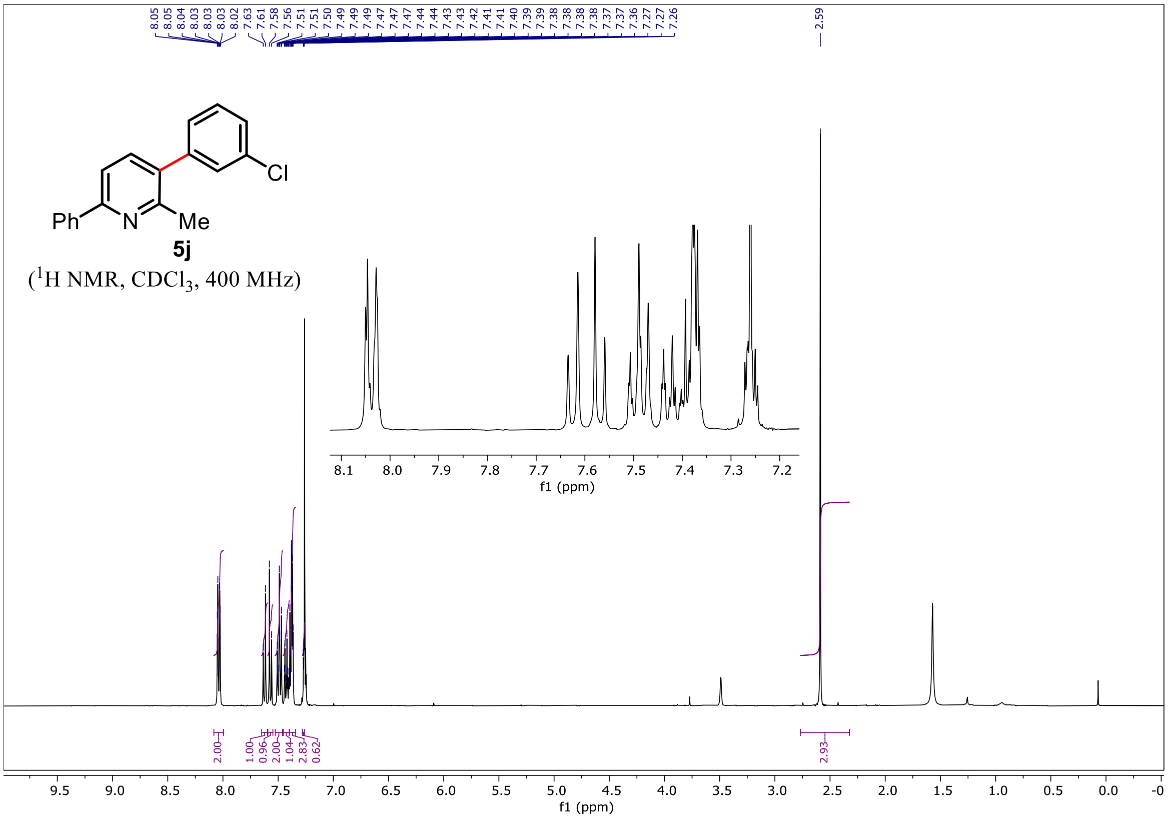
Ph
N
Me
Cl
5j
(1H NMR, CDCl3, 400 MHz)
f1 (ppm)

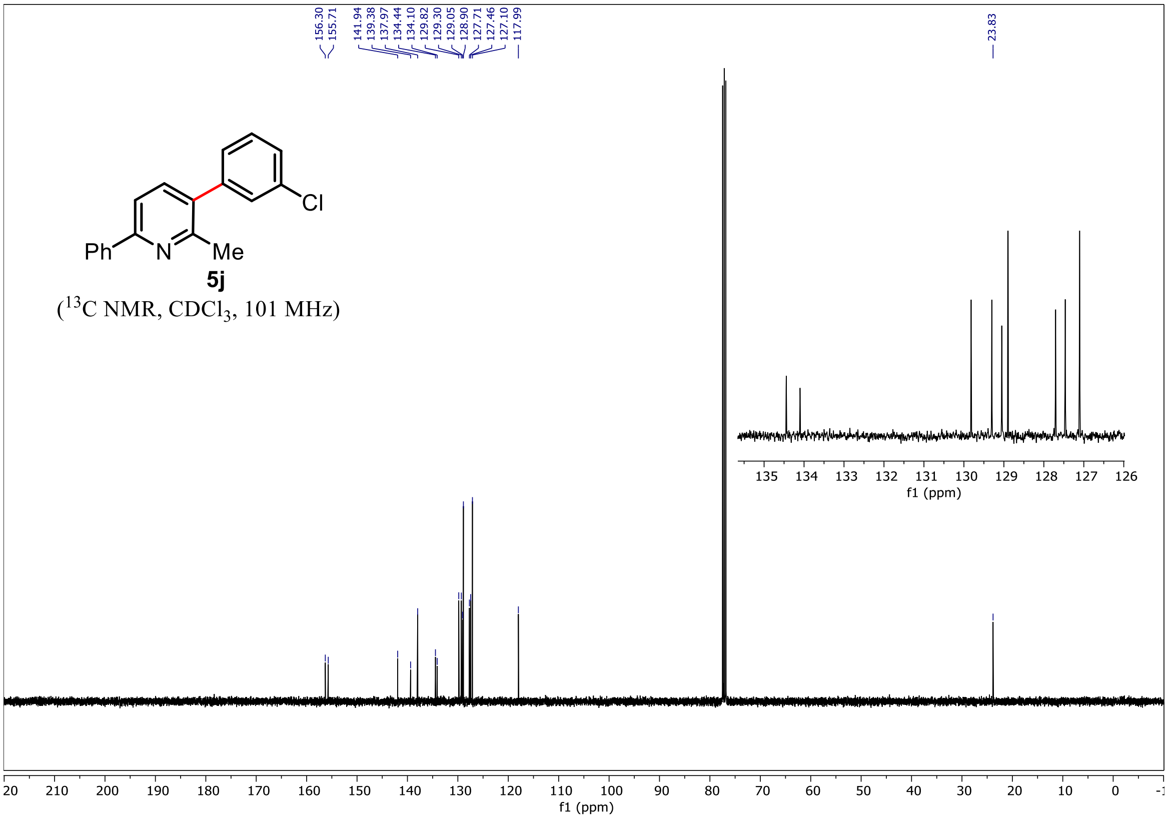
Ph
N
Me
Cl
5j
(13C NMR, CDCl3, 101 MHz)
f1 (ppm)

1D NOE NMR spectrum of **5j**, 500 MHz, $CDCl_3$

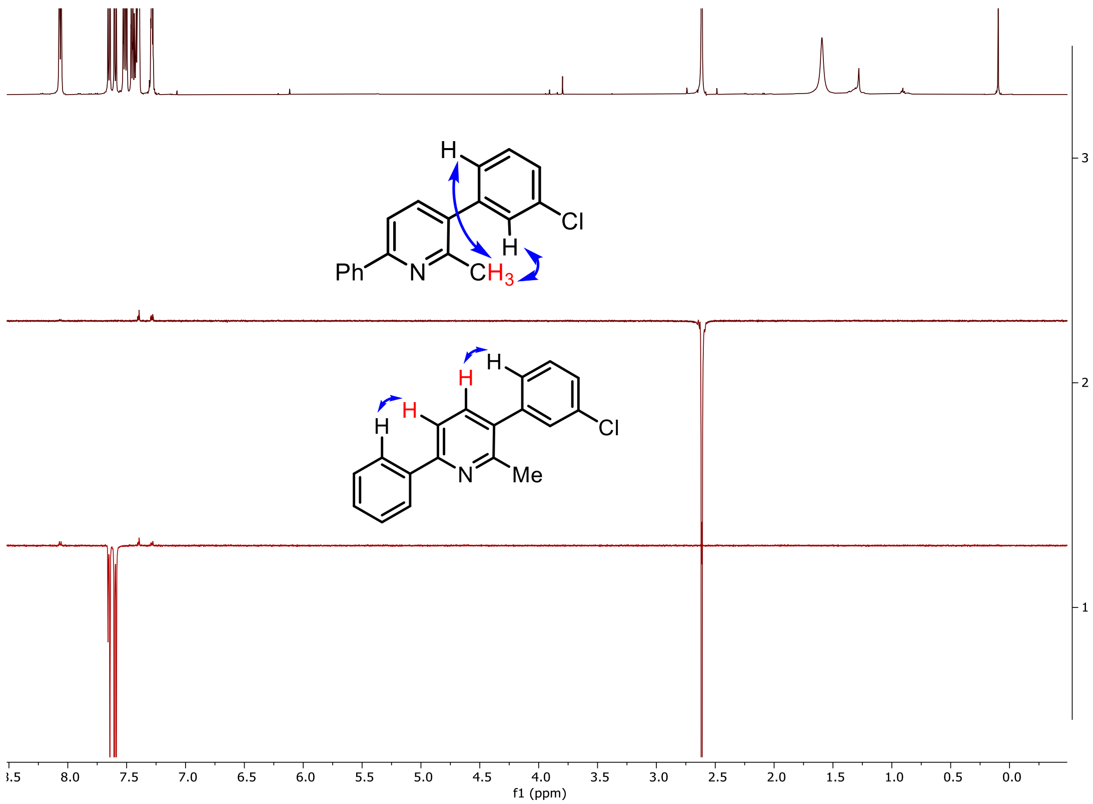


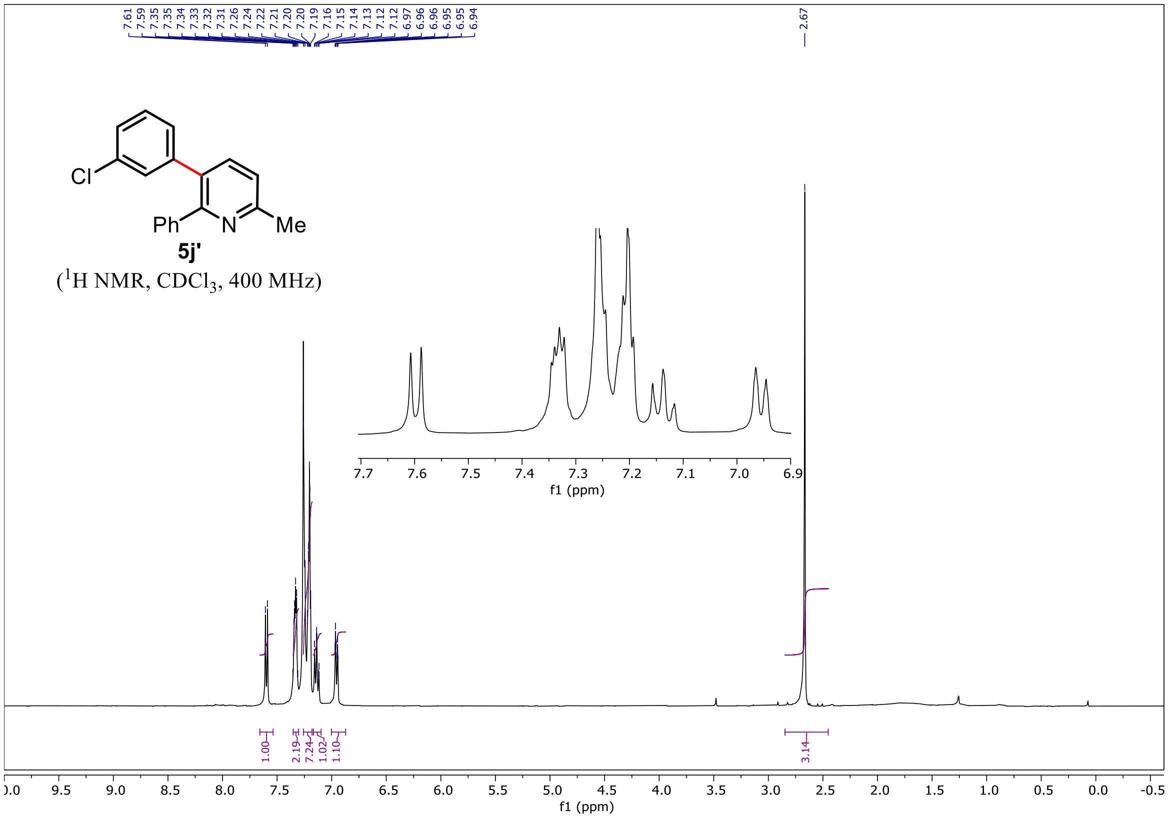

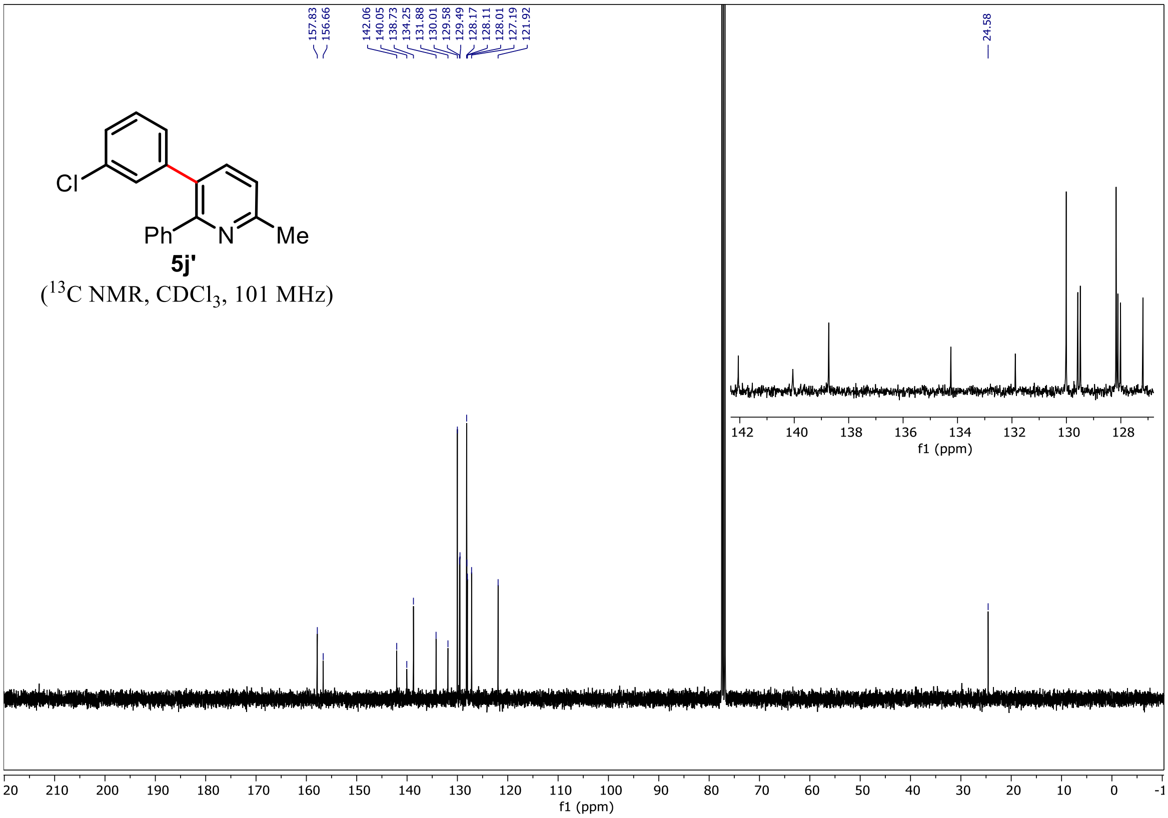


1D NOE NMR spectrum of **5j’**, 500 MHz, $C_6D_6$

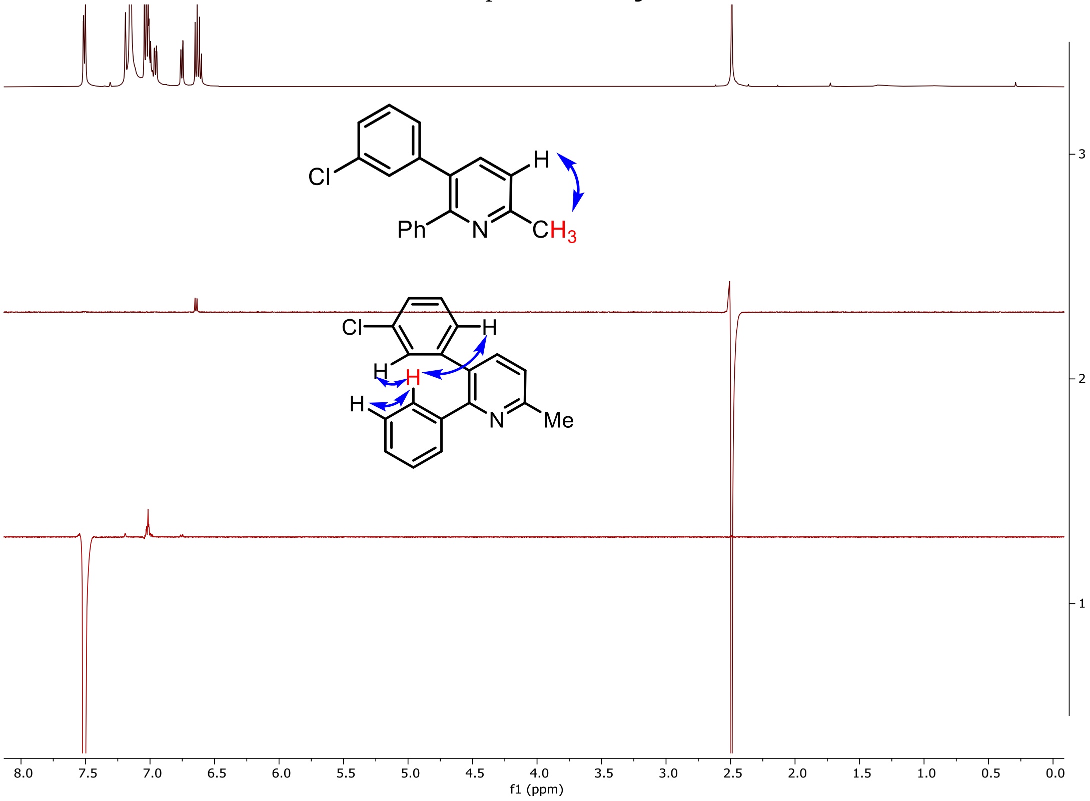

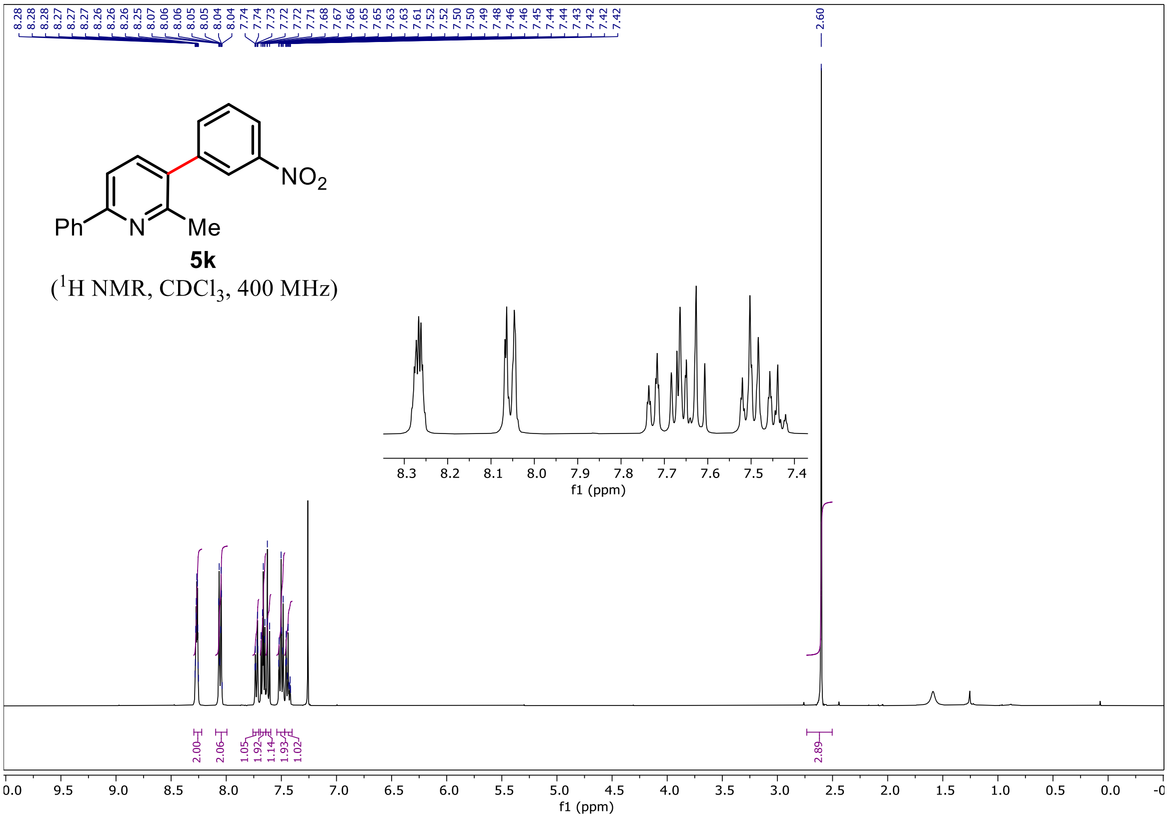
8.28
8.28
8.28
8.27
8.27
8.26
8.26
8.25
8.07
8.06
8.06
8.05
8.05
8.04
8.04
7.74
7.74
7.73
7.72
7.72
7.71
7.68
7.67
7.66
7.65
7.63
7.63
7.61
7.52
7.52
7.50
7.49
7.48
7.46
7.45
7.44
7.43
7.42
7.42
7.42
2.60
NO2
Ph
N
Me
5k
(1H NMR, CDCl3, 400 MHz)
2.00
2.06
1.05
1.92
1.14
1.93
1.02
2.89
f1 (ppm)

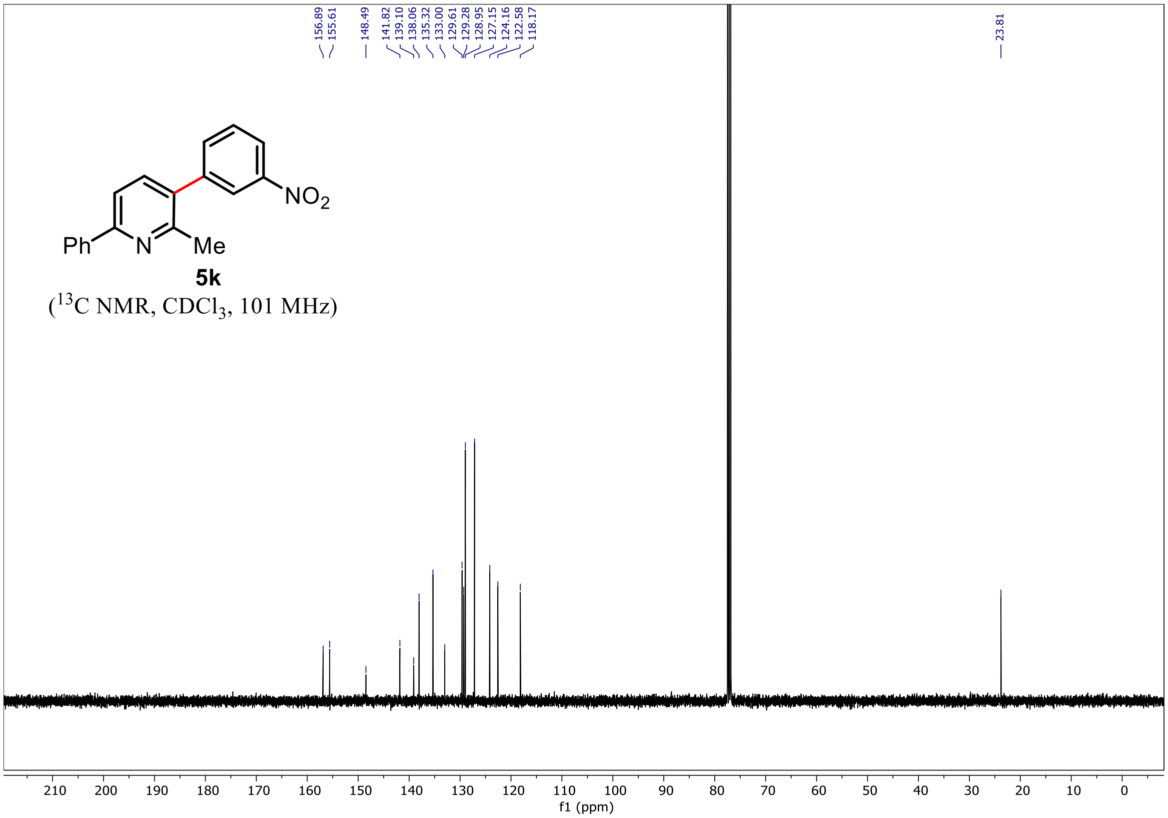
156.89
155.61
148.49
141.82
139.10
138.06
135.32
133.00
129.61
129.28
128.95
127.15
124.16
122.58
118.17
23.81
NO2
Ph
N
Me
5k
(13C NMR, CDCl3, 101 MHz)
f1 (ppm)

1D NOE NMR spectrum of **5k**, 500 MHz, $C_6D_6$

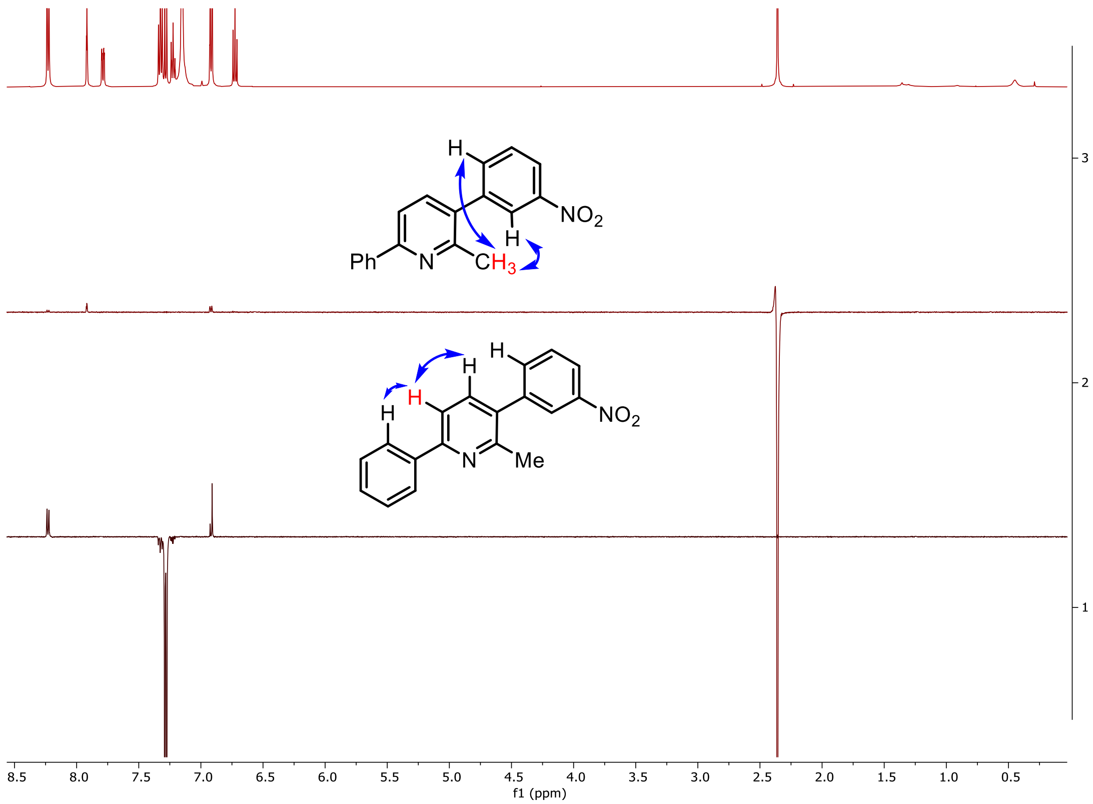


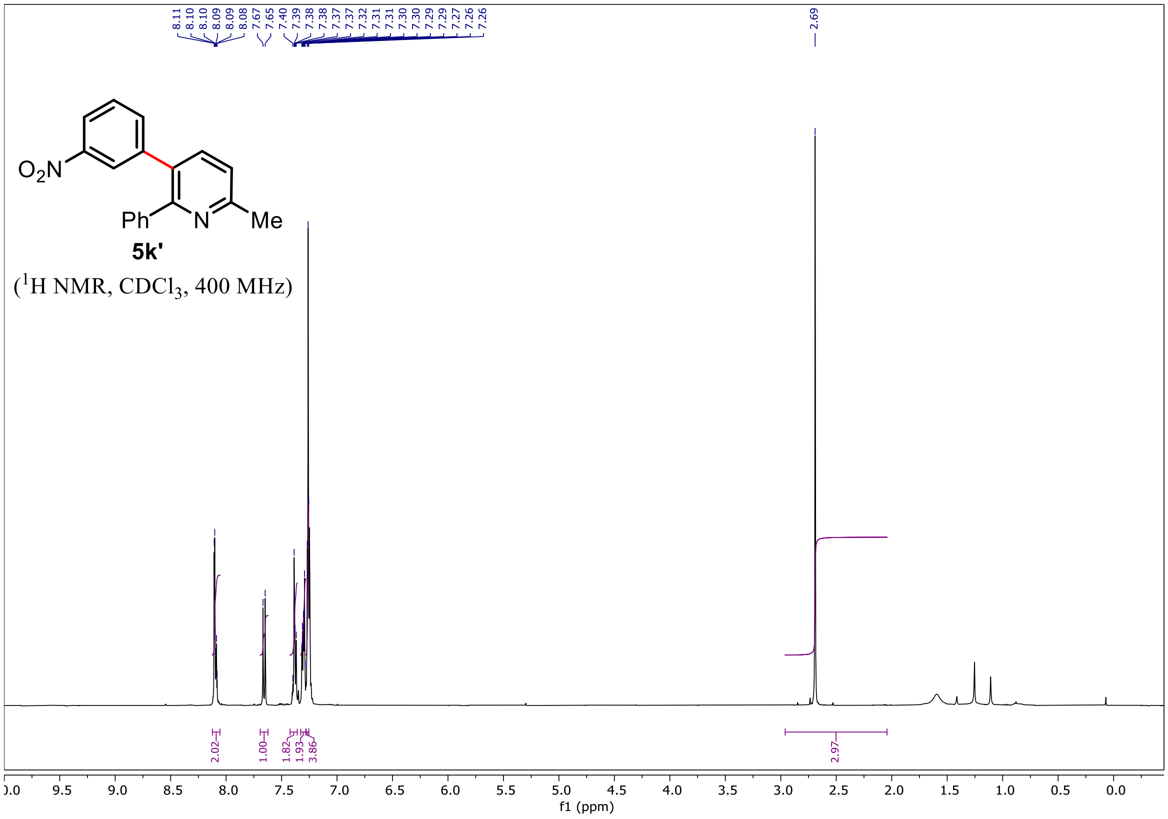

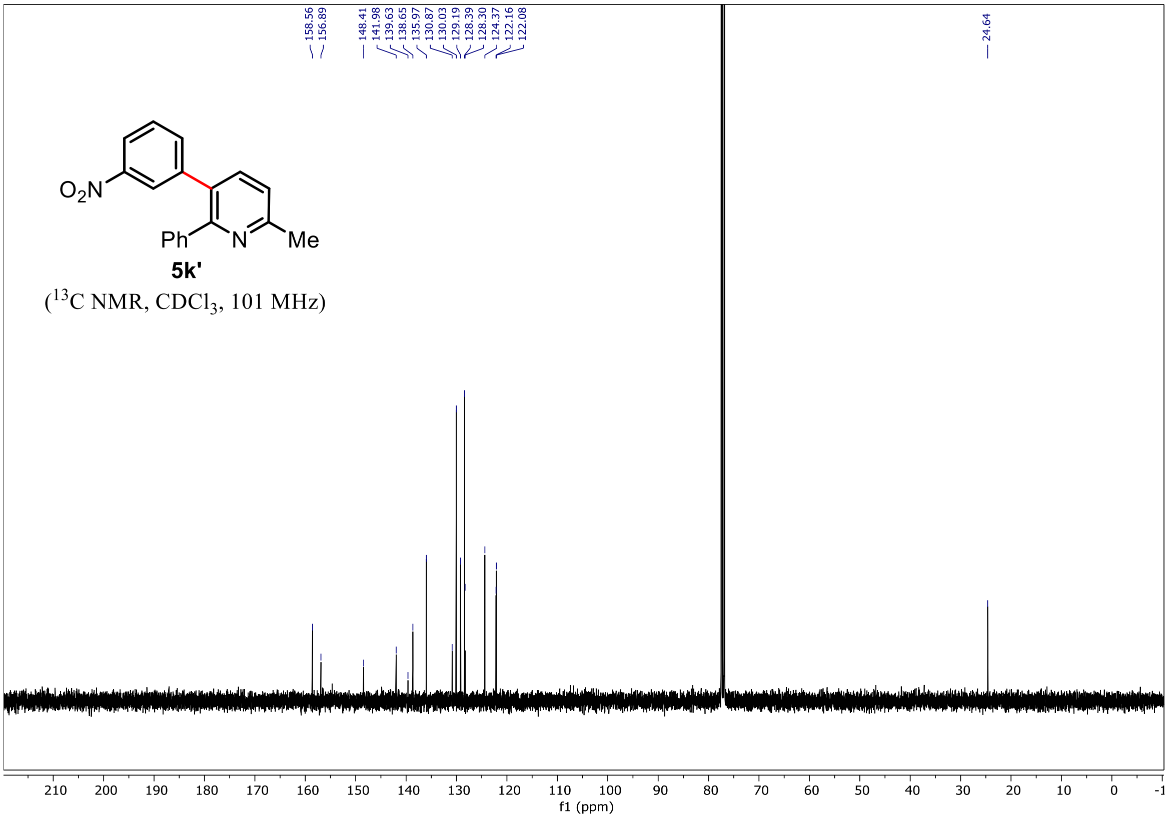


1D NOE NMR spectrum of **5k'**, 500 MHz, $C_6D_6$

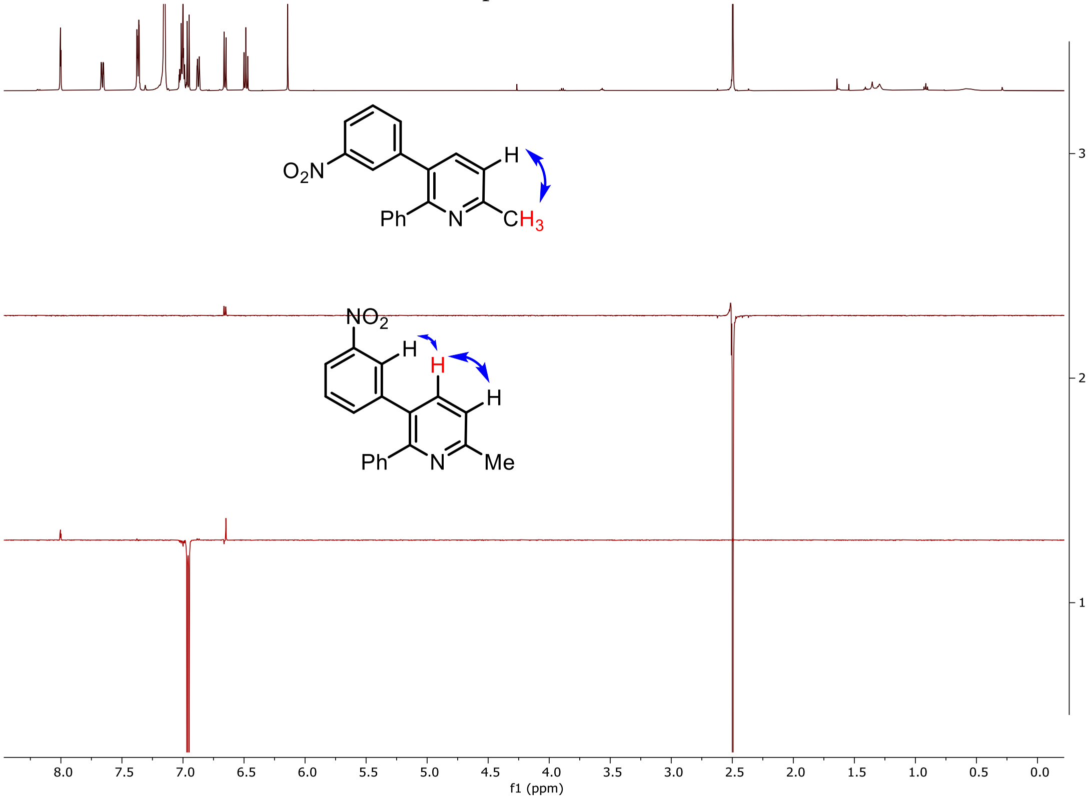

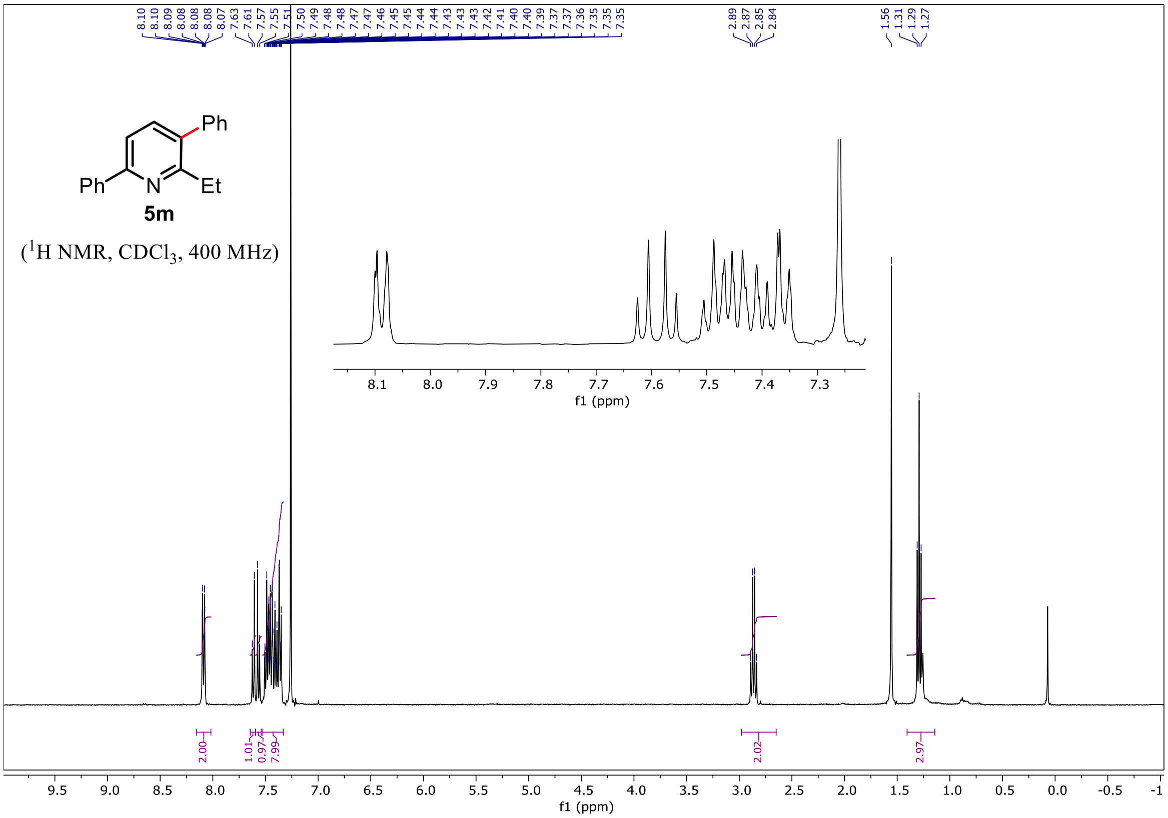
8.10 8.10 8.09 8.08 8.08 8.07 7.63 7.61 7.57 7.55 7.51 7.50 7.49 7.48 7.48 7.47 7.46 7.45 7.45 7.44 7.44 7.43 7.43 7.43 7.42 7.41 7.40 7.39 7.37 7.37 7.36 7.35 7.35 7.35
2.89 2.87 2.85 2.84
1.56 1.31 1.29 1.27
Ph
Ph
N
Et
5m
(¹H NMR, CDCl3, 400 MHz)
2.00
1.01
0.97
7.99
2.02
2.97
f1 (ppm)

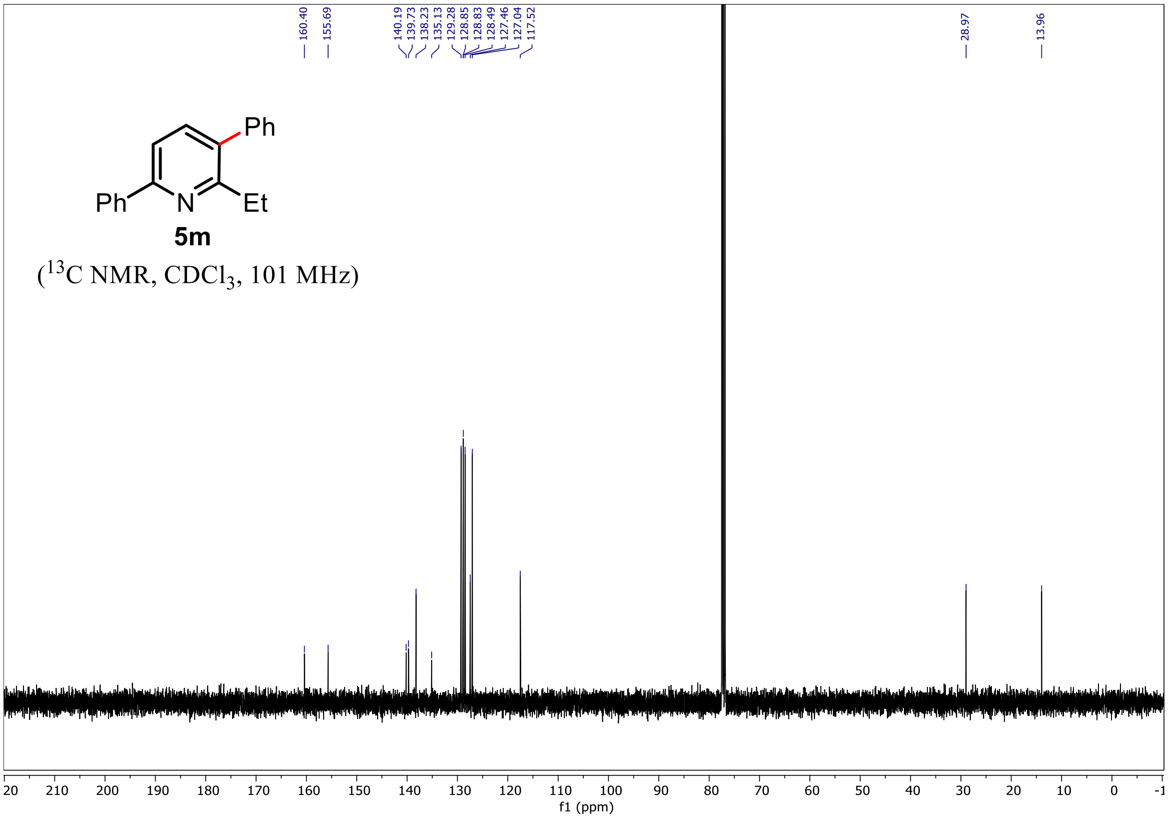
160.40
155.69
140.19 139.73 138.23 135.13 129.28 128.85 128.83 128.49 127.46 127.04 117.52
28.97
13.96
Ph
Ph
N
Et
5m
(¹³C NMR, CDCl3, 101 MHz)
f1 (ppm)

1D NOE NMR spectrum of **5m**, 500 MHz, $CDCl_3$

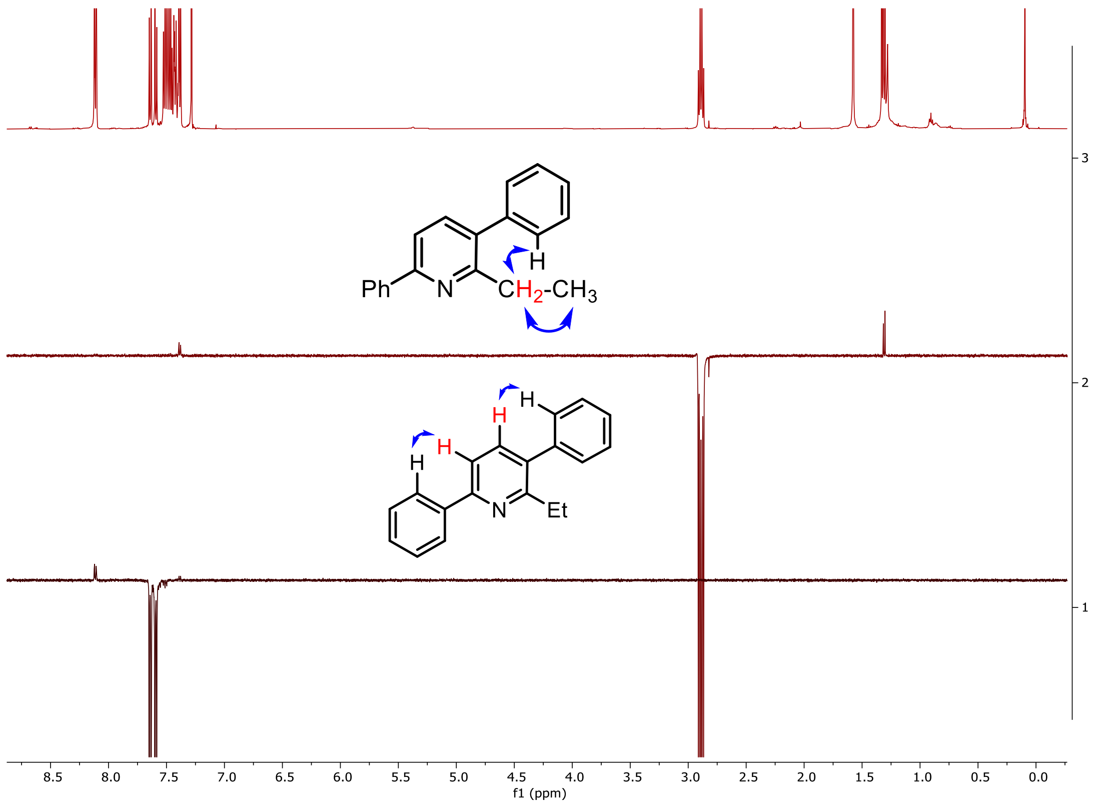

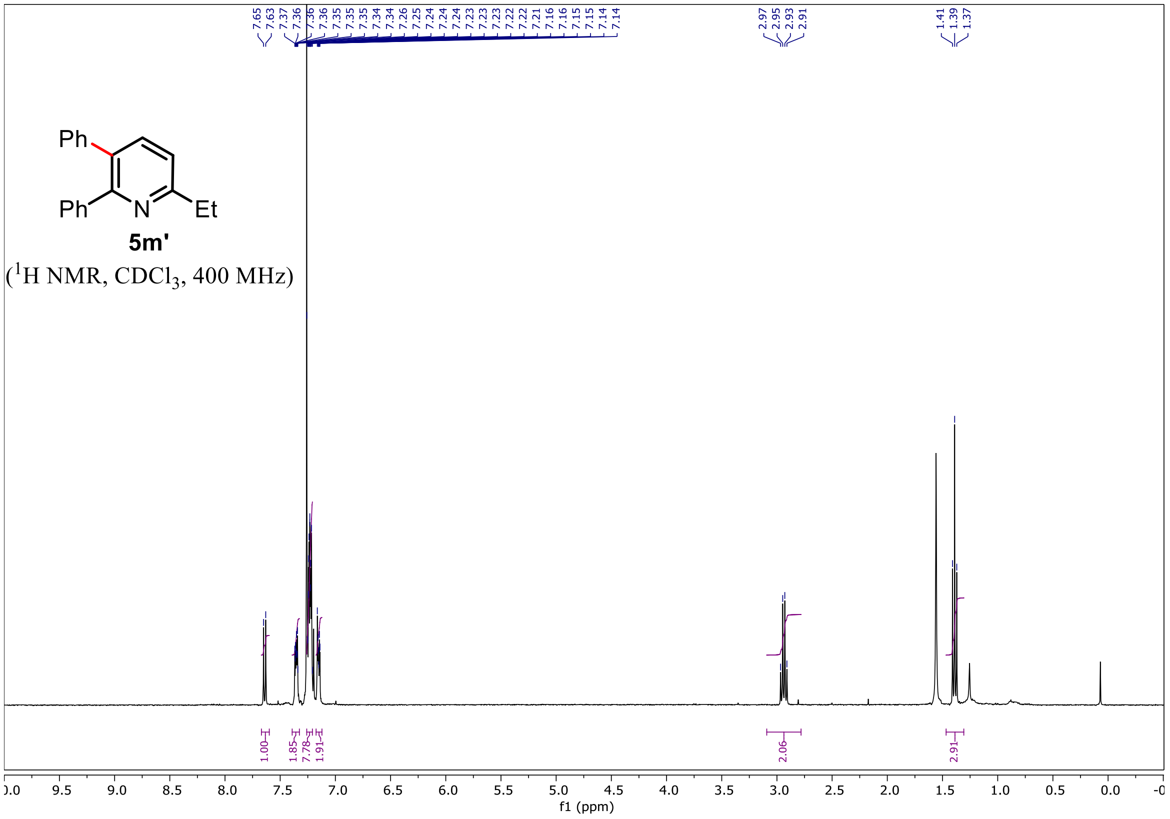

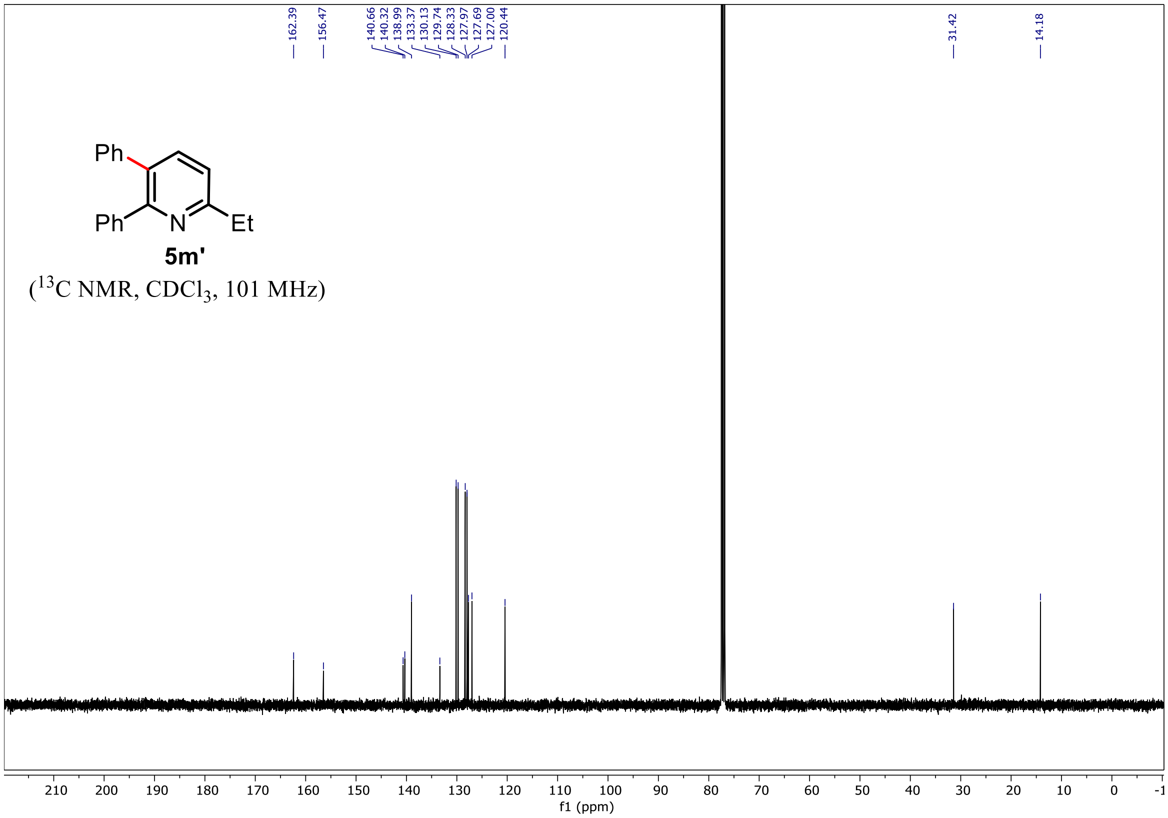


1D NOE NMR spectrum of **5m’**, 500 MHz, $C_6D_6$

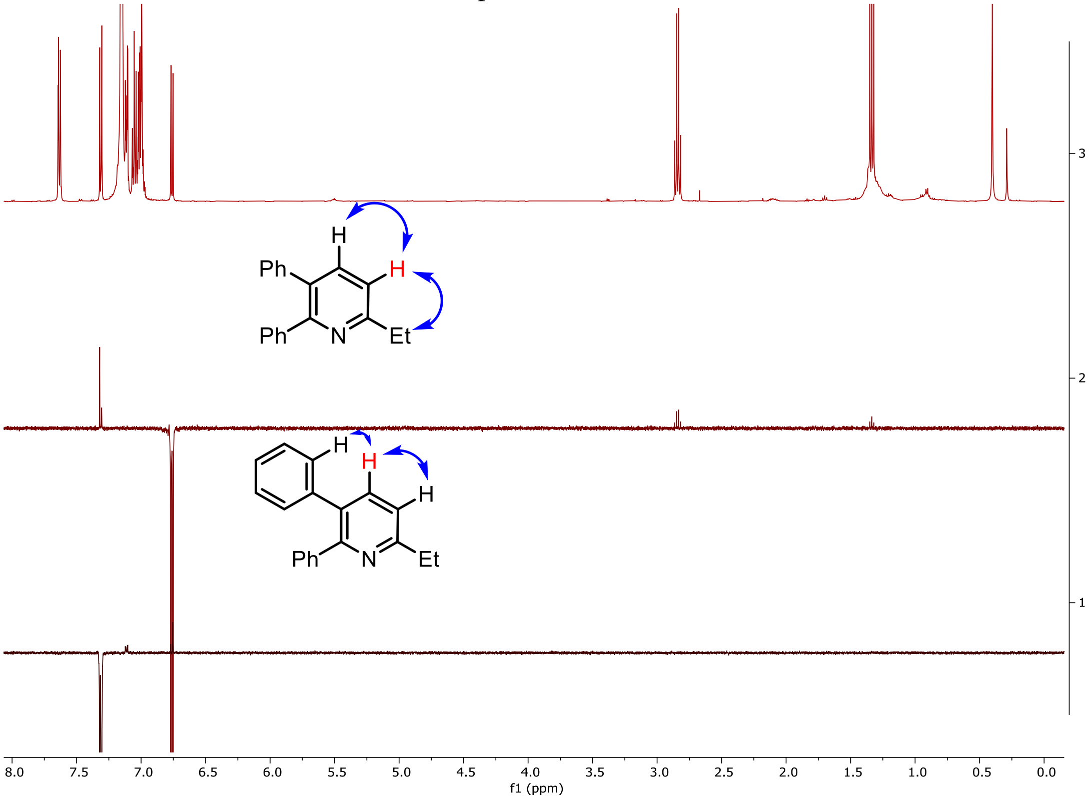

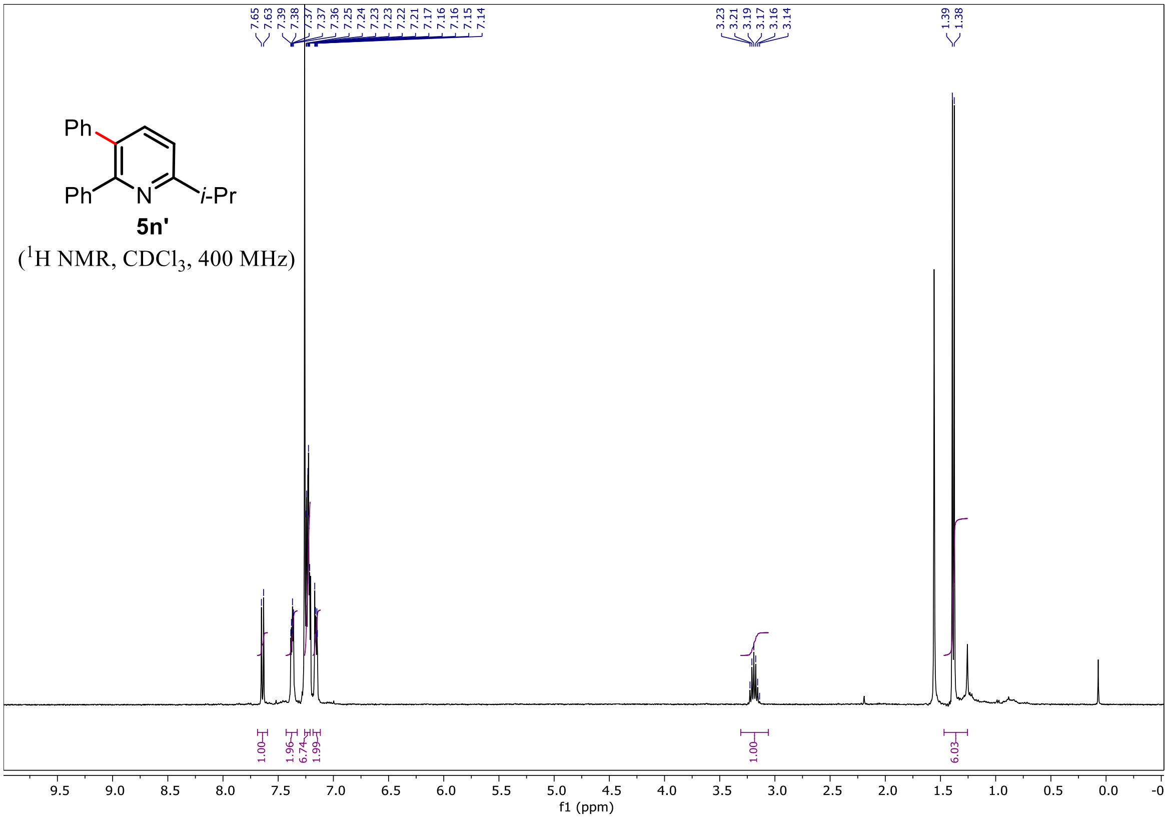
7.65
7.63
7.39
7.38
7.37
7.37
7.36
7.25
7.24
7.23
7.23
7.22
7.21
7.17
7.16
7.16
7.15
7.14
3.23
3.21
3.19
3.17
3.16
3.14
1.39
1.38
Ph
Ph
N
i-Pr
5n'
(1H NMR, CDCl3, 400 MHz)
1.00
1.96
6.74
1.99
1.00
6.03
f1 (ppm)

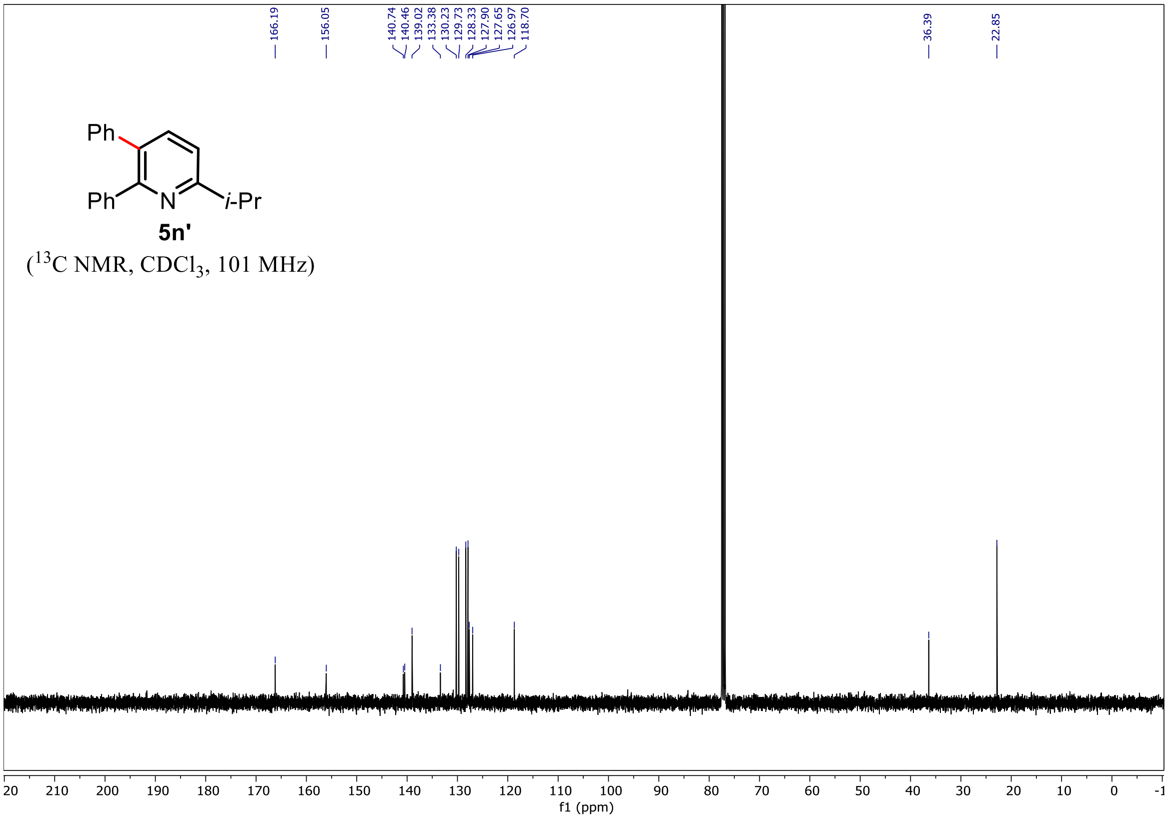
166.19
156.05
140.74
140.46
139.02
133.38
130.23
129.73
128.33
127.90
127.65
126.97
118.70
36.39
22.85
Ph
Ph
N
i-Pr
5n'
(13C NMR, CDCl3, 101 MHz)
f1 (ppm)

1D NOE NMR spectrum of **5n'**, 500 MHz, $C_6D_6$

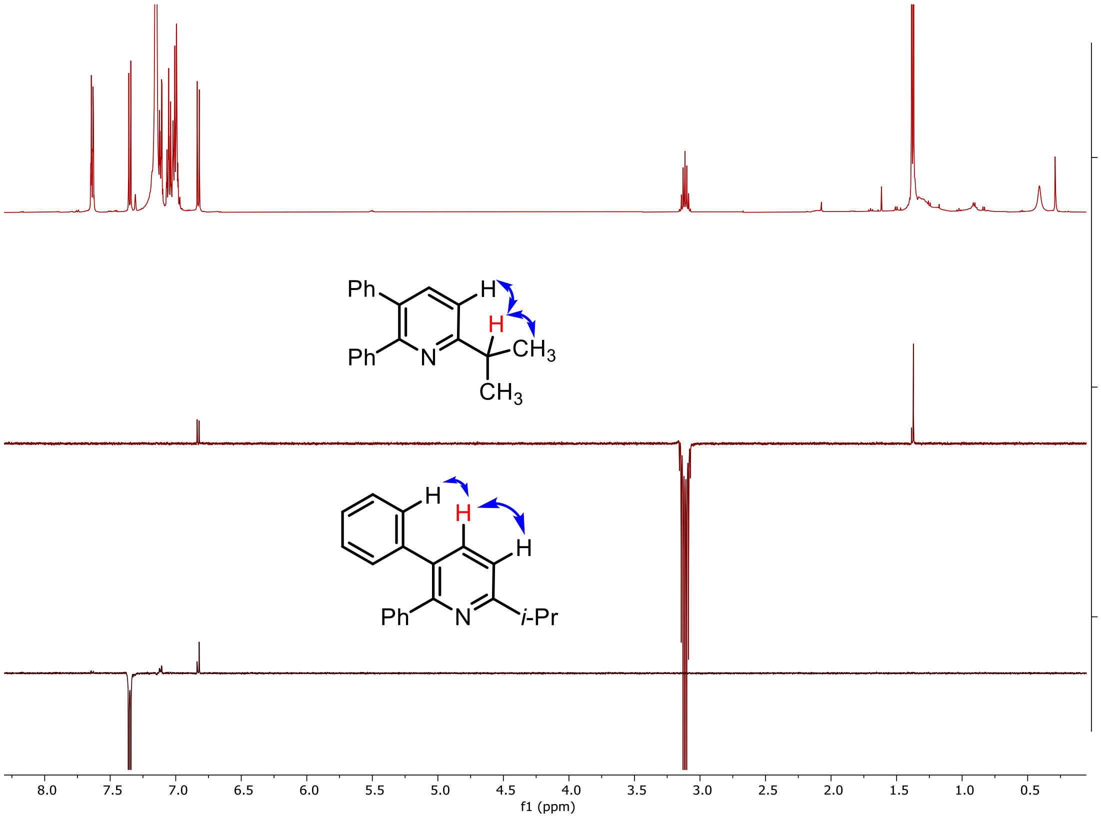


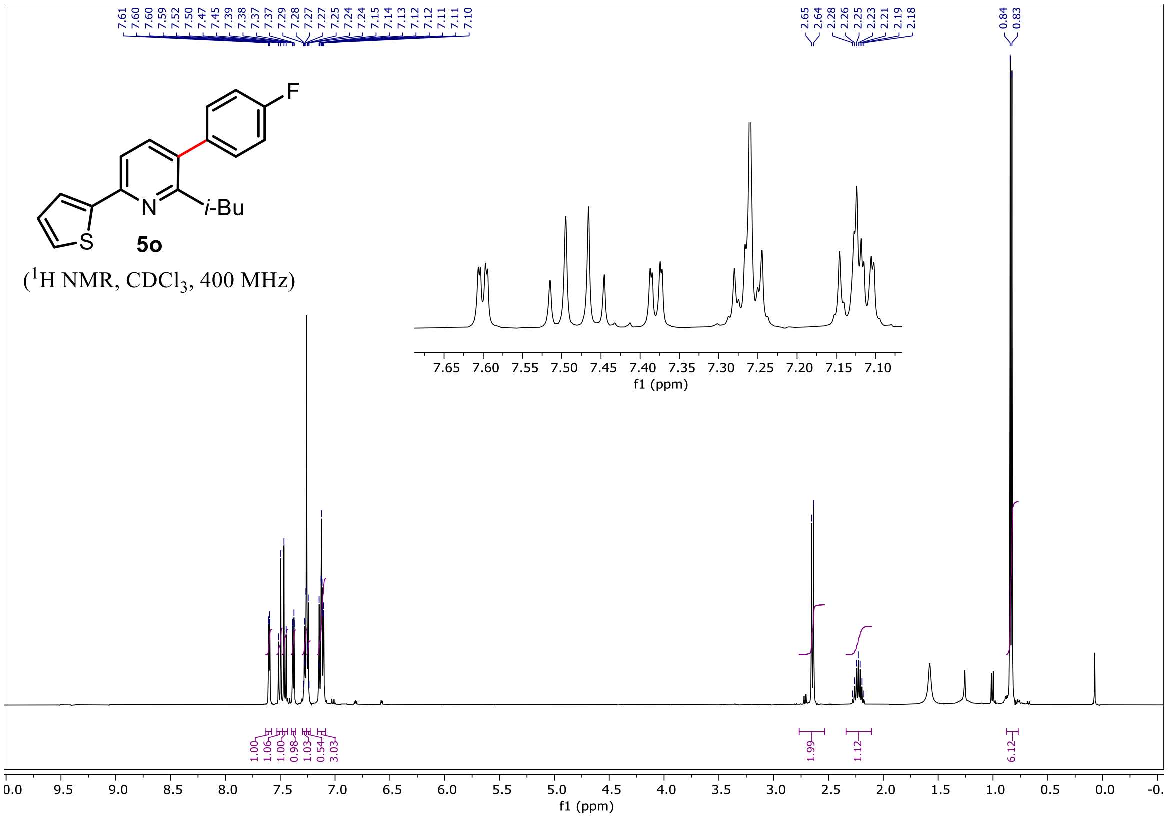

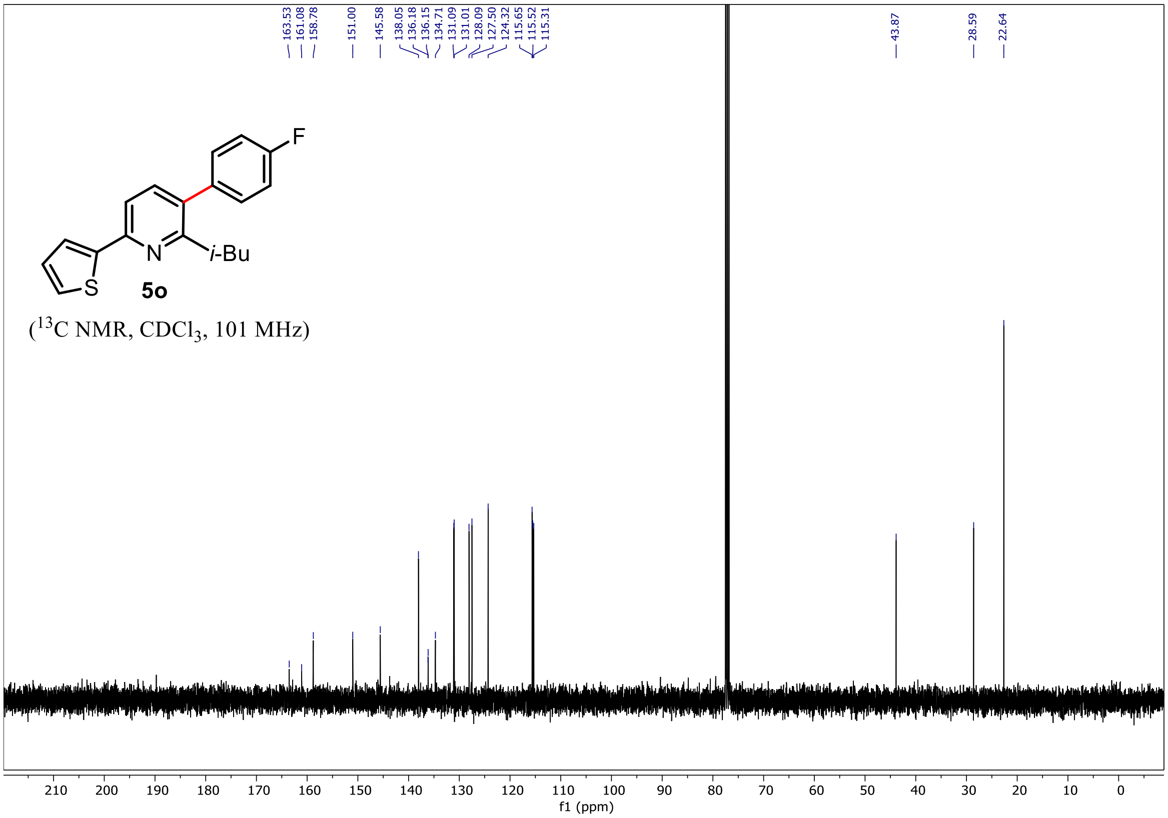
163.53
161.08
158.78
151.00
145.58
138.05
136.18
136.15
134.71
131.09
131.01
128.09
127.50
124.32
115.65
115.52
115.31
43.87
28.59
22.64
F
N
*i*-Bu
S
5o
($^{13}$C NMR, $CDCl_3$, 101 MHz)
210 200 190 180 170 160 150 140 130 120 110 100 90 80 70 60 50 40 30 20 10 0
f1 (ppm)

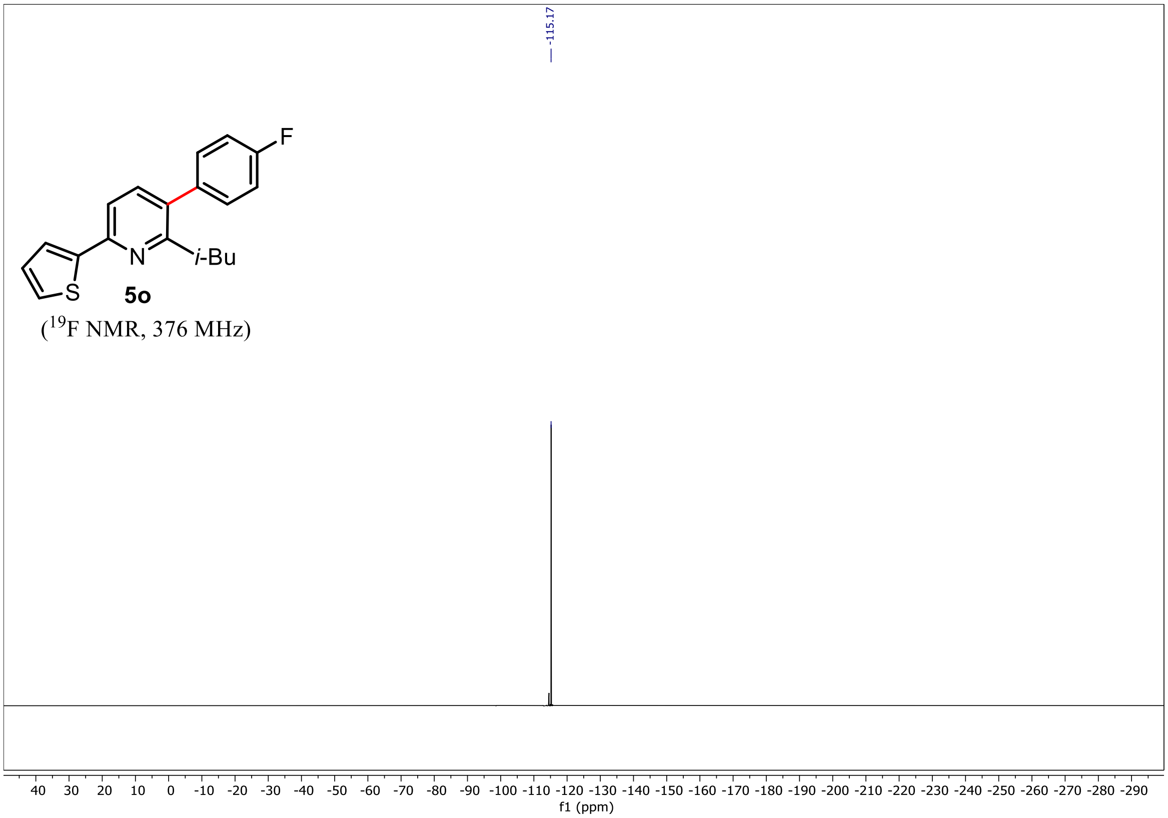
-115.17
F
N
*i*-Bu
S
5o
($^{19}$F NMR, 376 MHz)
40 30 20 10 0 -10 -20 -30 -40 -50 -60 -70 -80 -90 -100 -110 -120 -130 -140 -150 -160 -170 -180 -190 -200 -210 -220 -230 -240 -250 -260 -270 -280 -290
f1 (ppm)

1D NOE NMR spectrum of **5o**, 500 MHz, $CDCl_3$

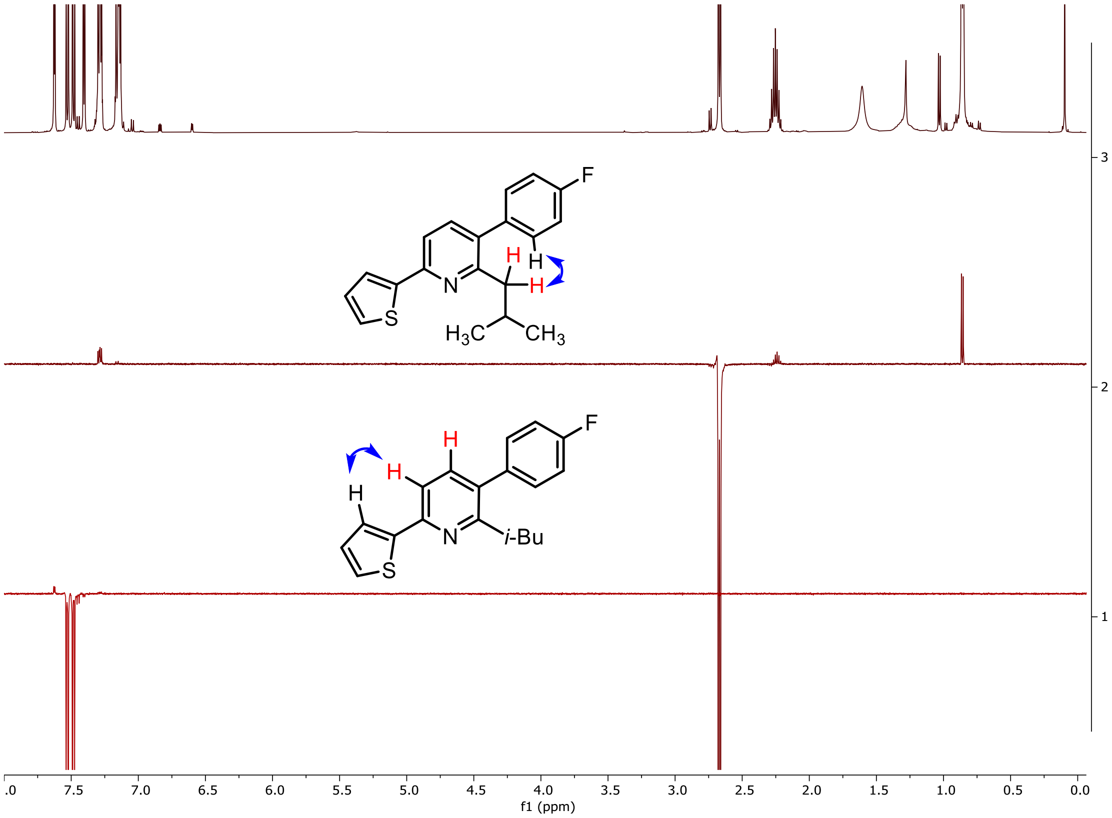


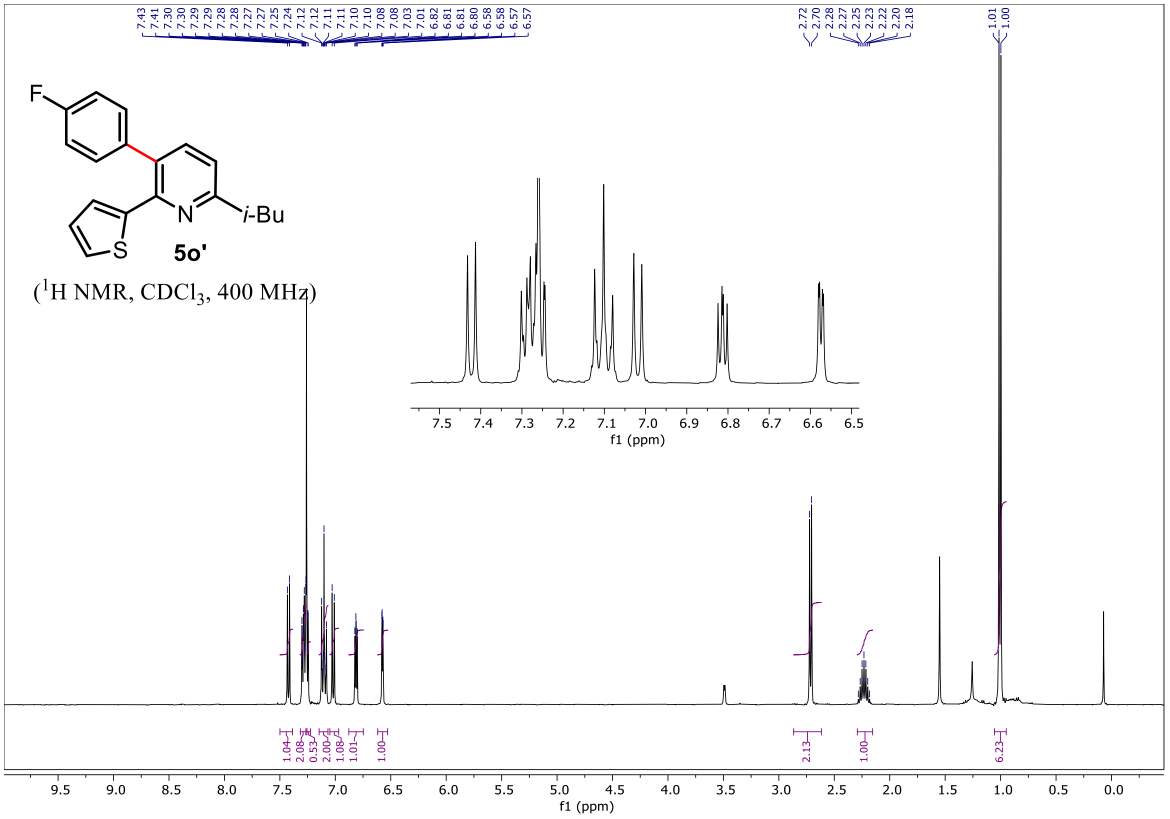

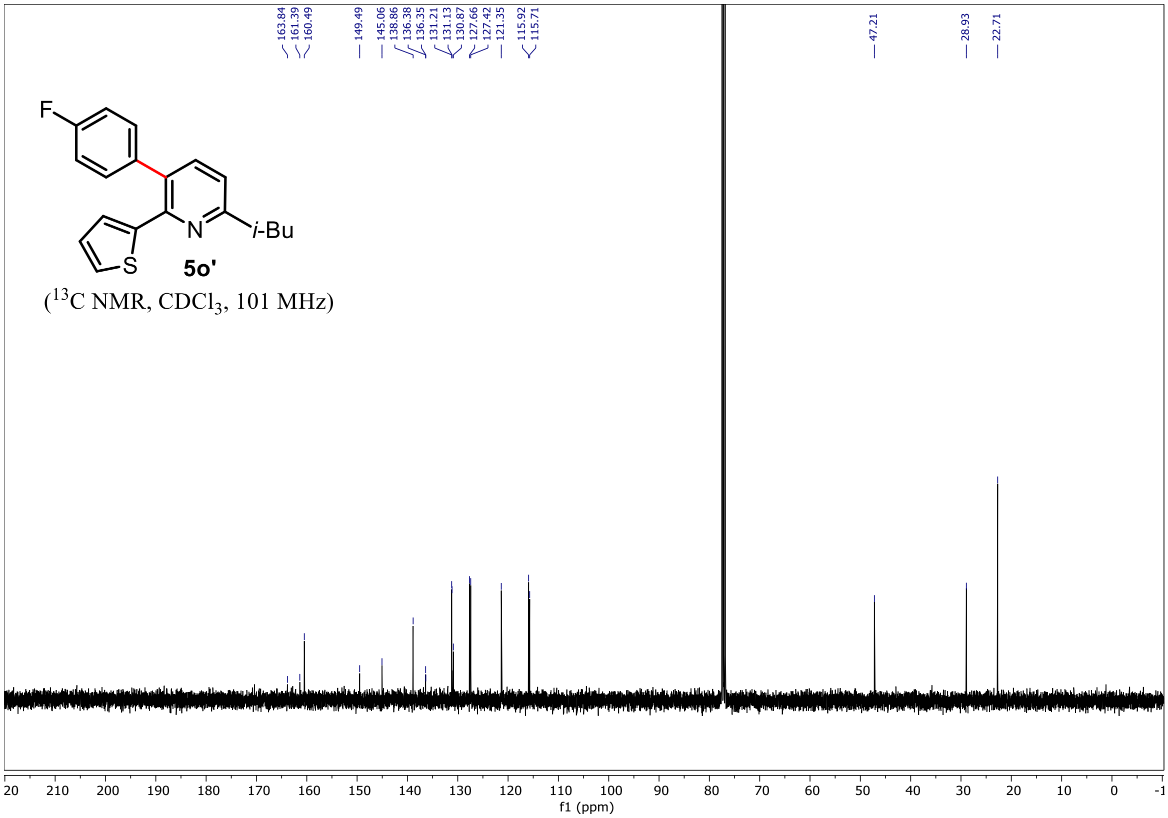
163.84
161.39
160.49
149.49
145.06
138.86
136.38
136.35
131.21
131.13
130.87
127.66
127.42
121.35
115.92
115.71
47.21
28.93
22.71
F
N
i-Bu
S
5o'
(13C NMR, CDCl3, 101 MHz)
f1 (ppm)

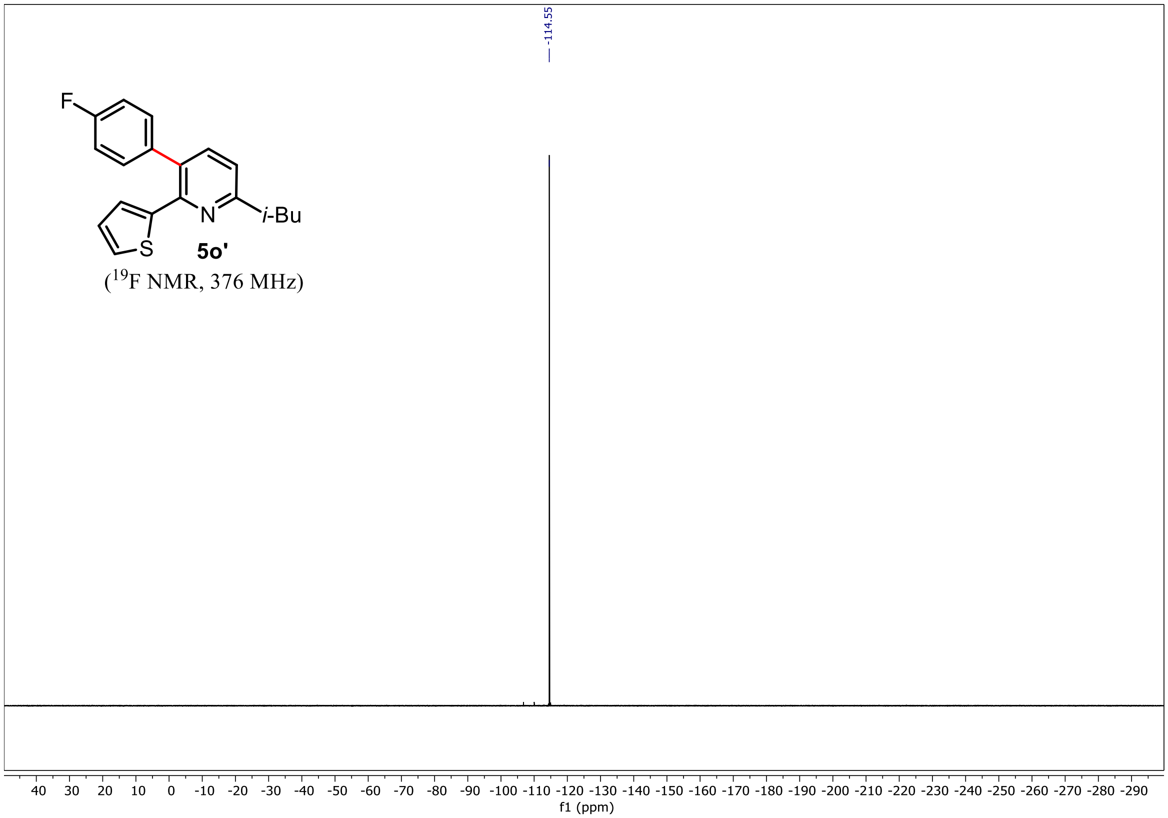
-114.55
F
N
i-Bu
S
5o'
(19F NMR, 376 MHz)
f1 (ppm)

1D NOE NMR spectrum of **5o’**, 500 MHz, $CDCl_3$

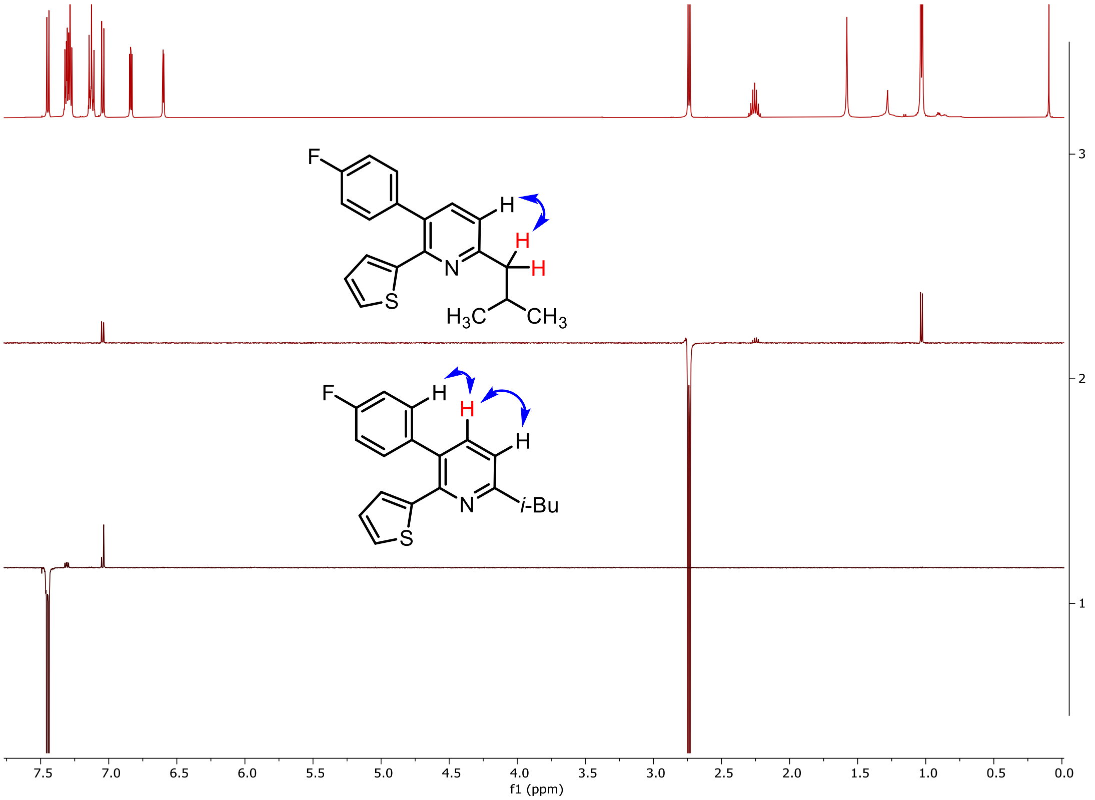


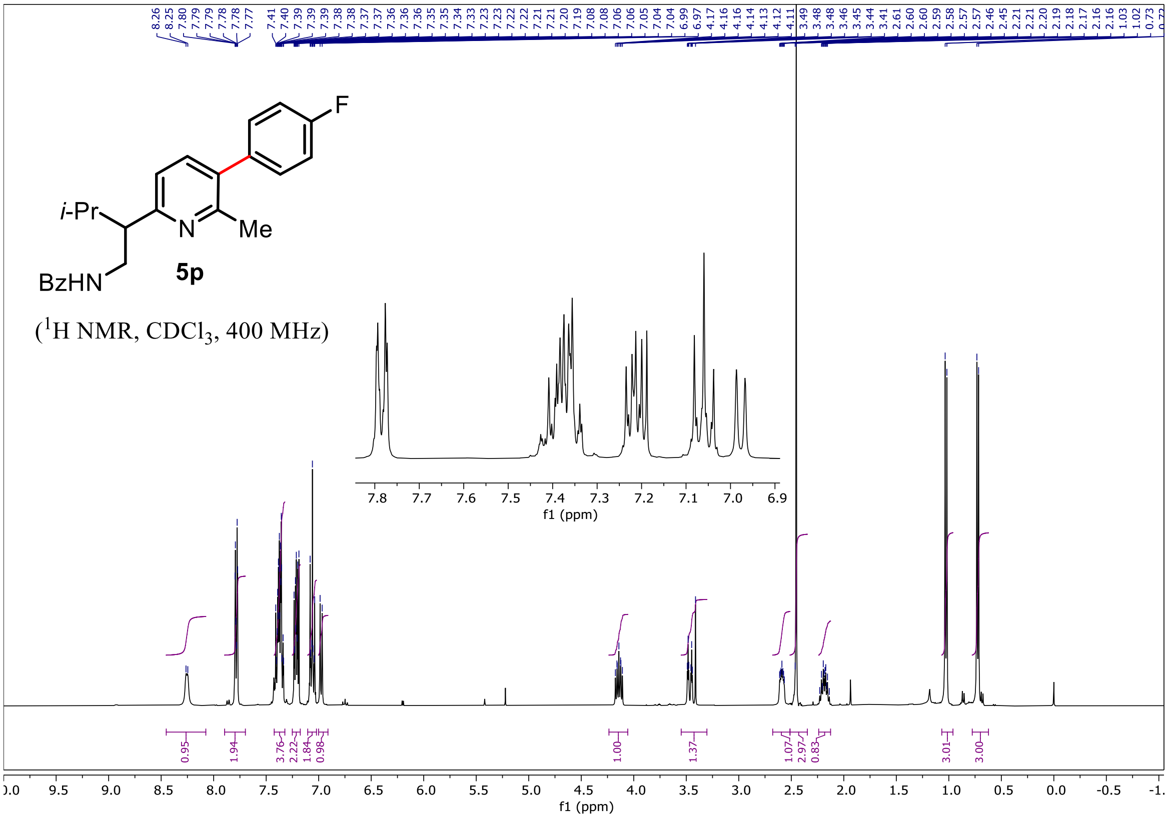

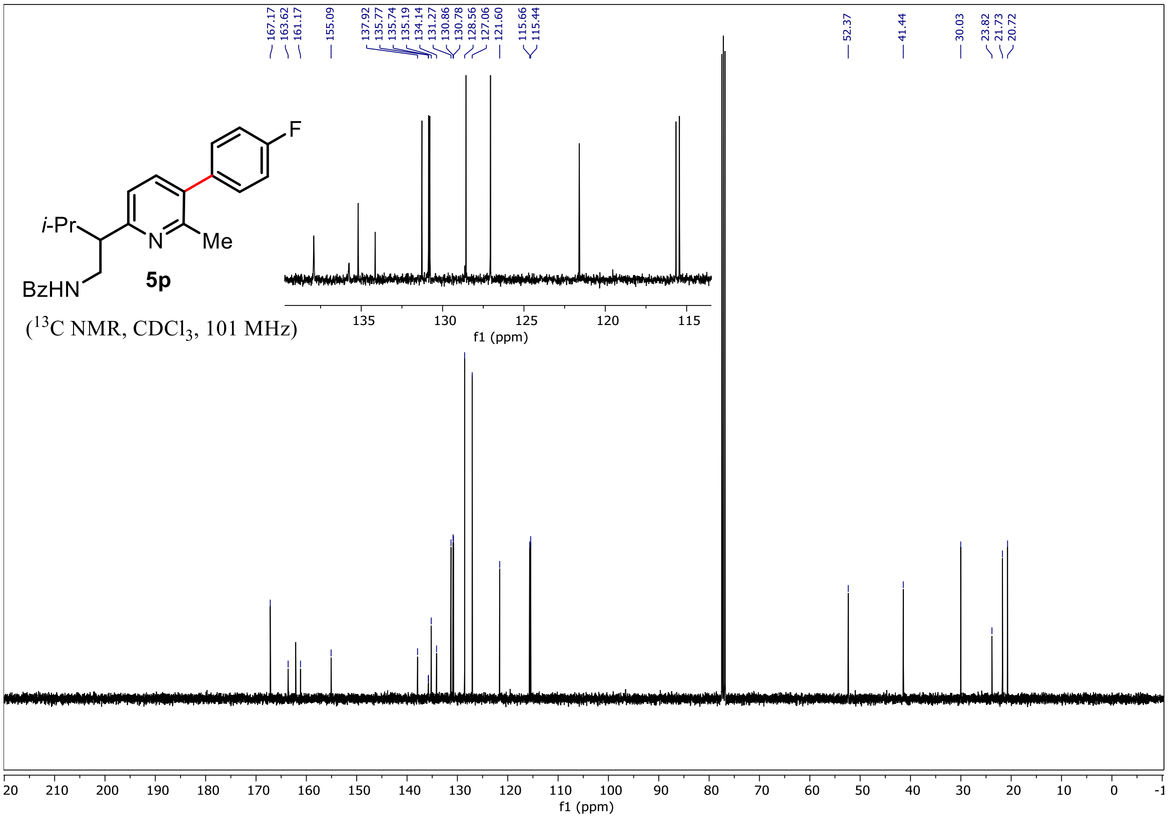
167.17
163.62
161.17
155.09
137.92
135.77
135.74
135.19
134.14
131.27
130.86
130.78
128.56
127.06
121.60
115.66
115.44
52.37
41.44
30.03
23.82
21.73
20.72
i-Pr
BzHN
N
Me
F
5p
($^{13}$C NMR, $CDCl_3$, 101 MHz)
f1 (ppm)

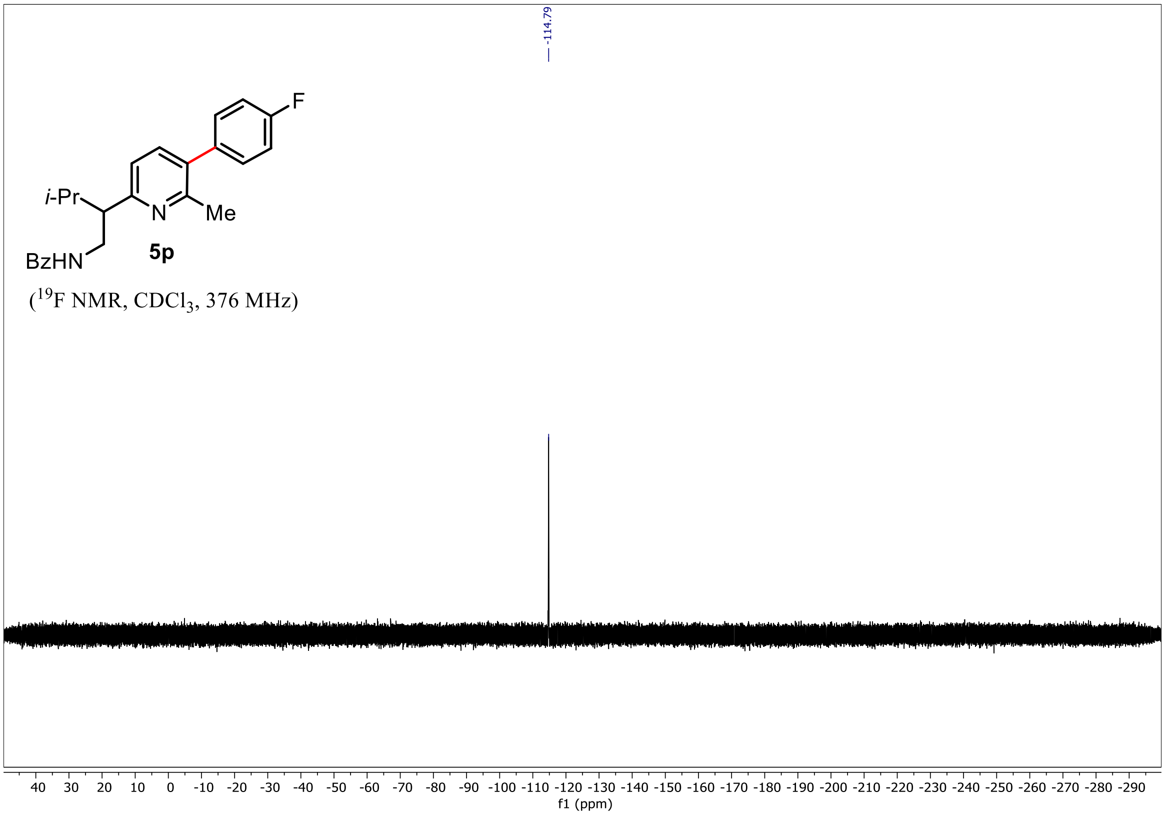
-114.79
i-Pr
BzHN
N
Me
F
5p
($^{19}$F NMR, $CDCl_3$, 376 MHz)
f1 (ppm)

1D NOE NMR spectrum of **5p**, 500 MHz, $C_6D_6$

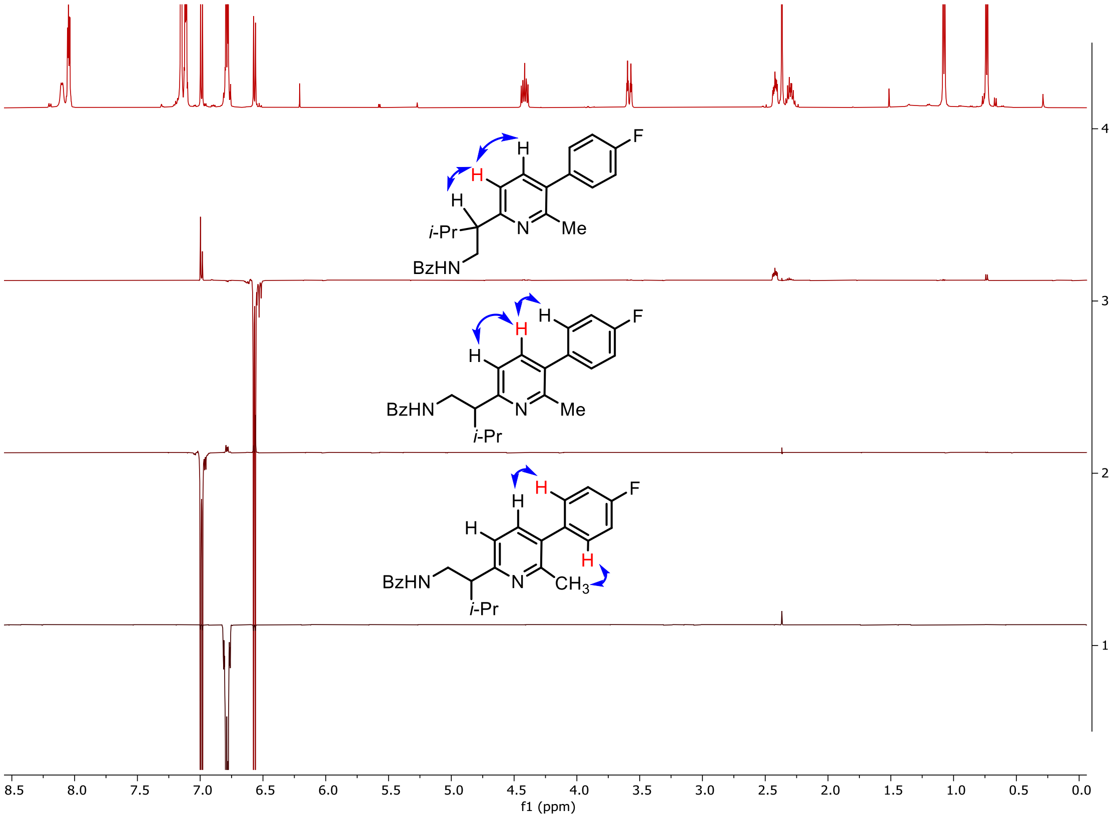

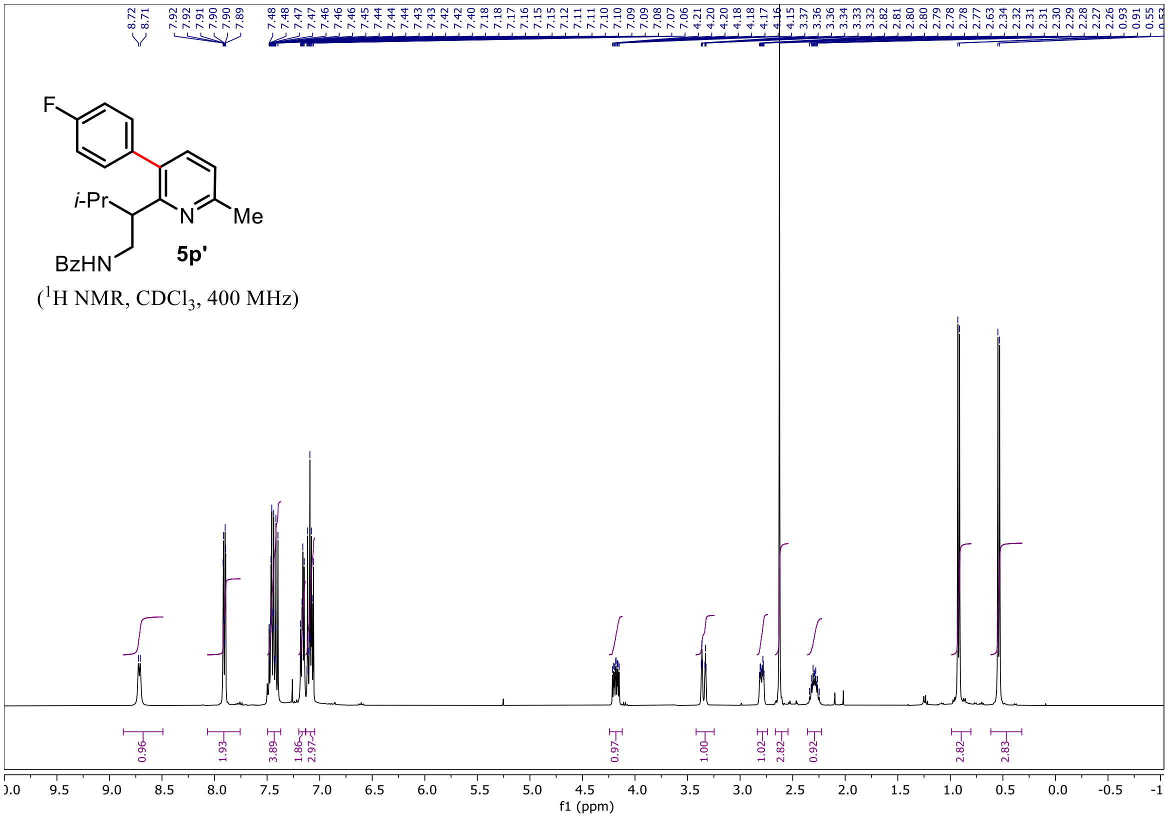

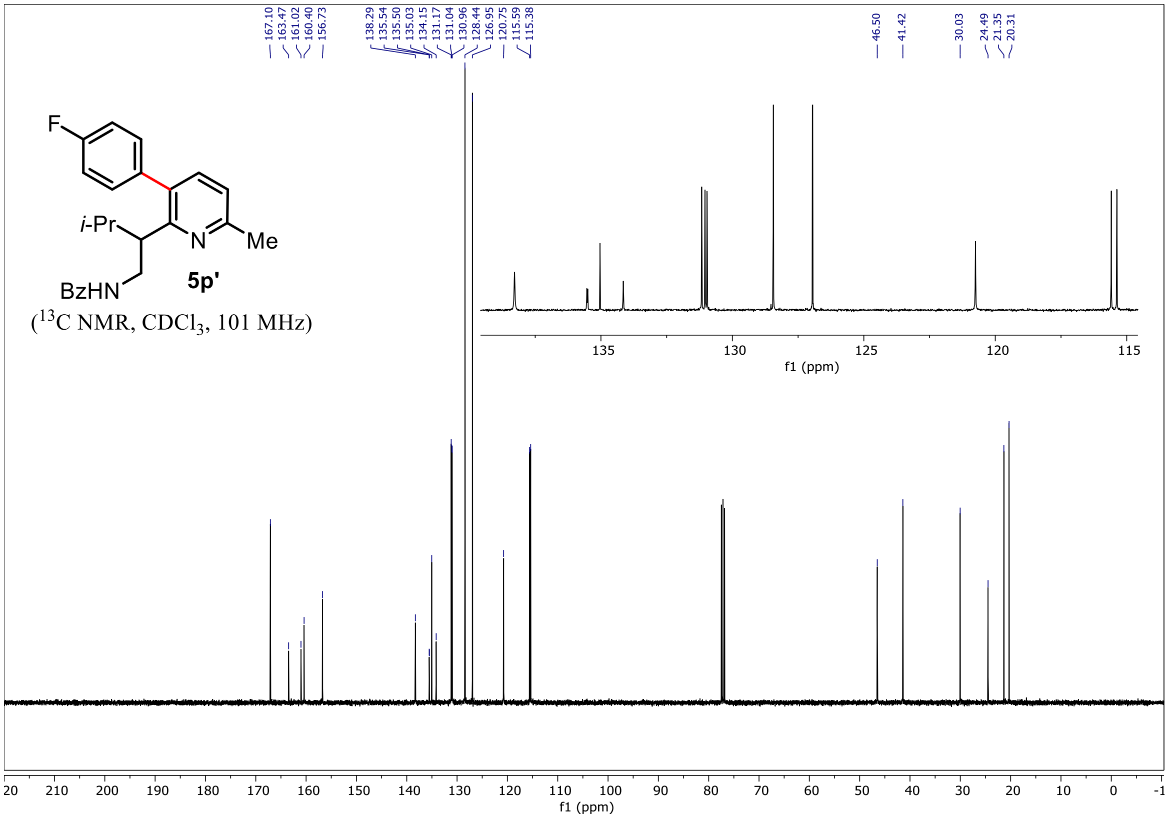
5p'
(13C NMR, CDCl3, 101 MHz)
f1 (ppm)

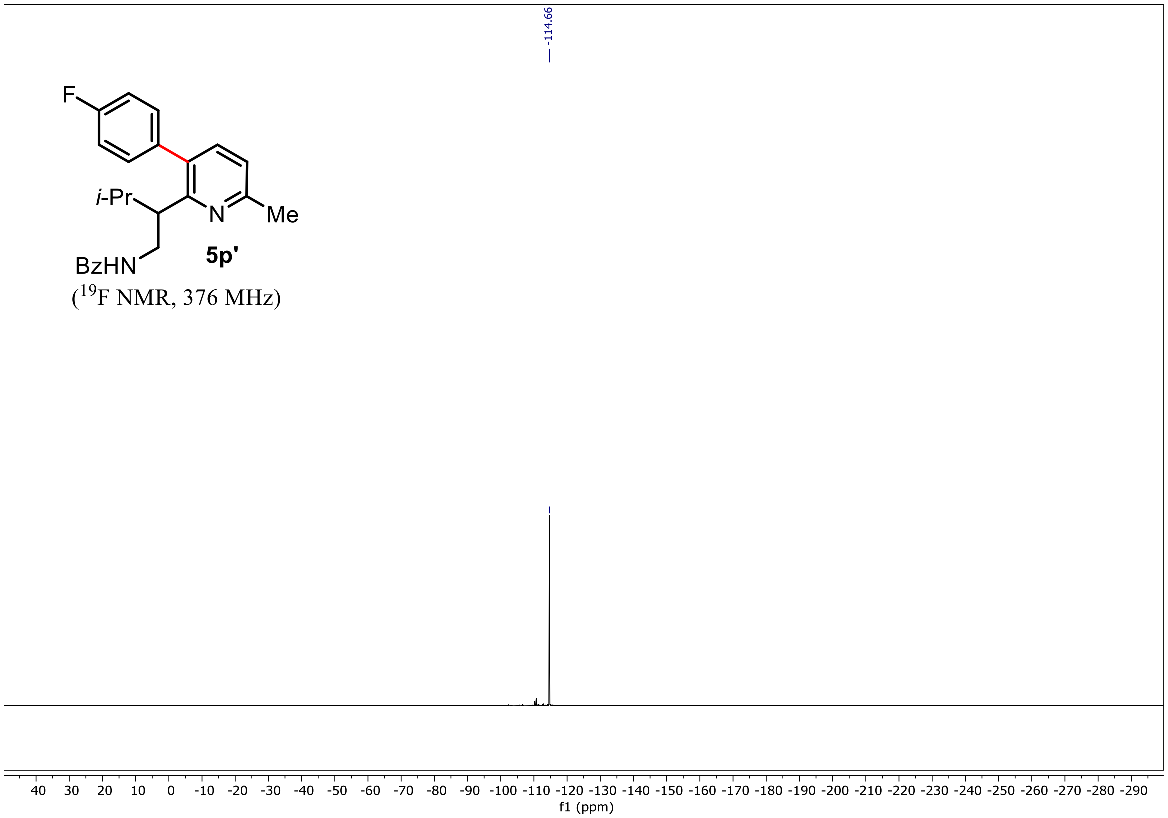
5p'
(19F NMR, 376 MHz)
-114.66
f1 (ppm)

1D NOE NMR spectrum of **5p'**, 500 MHz, $C_6D_6$

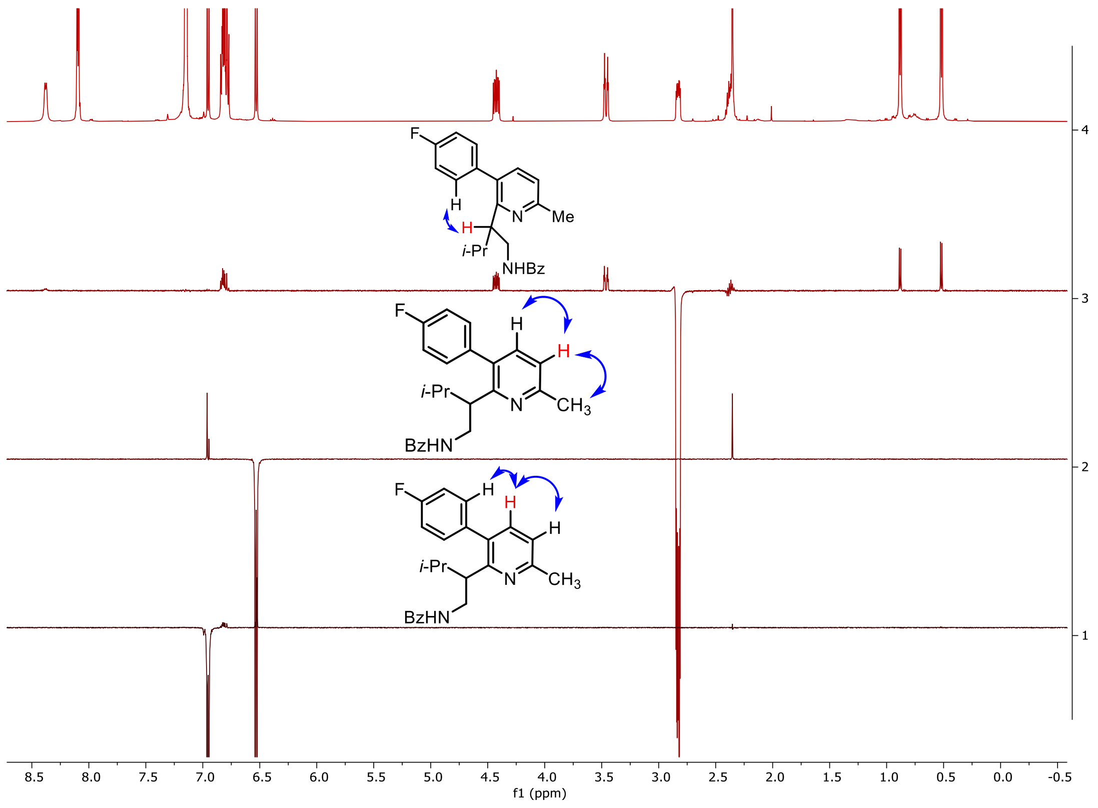


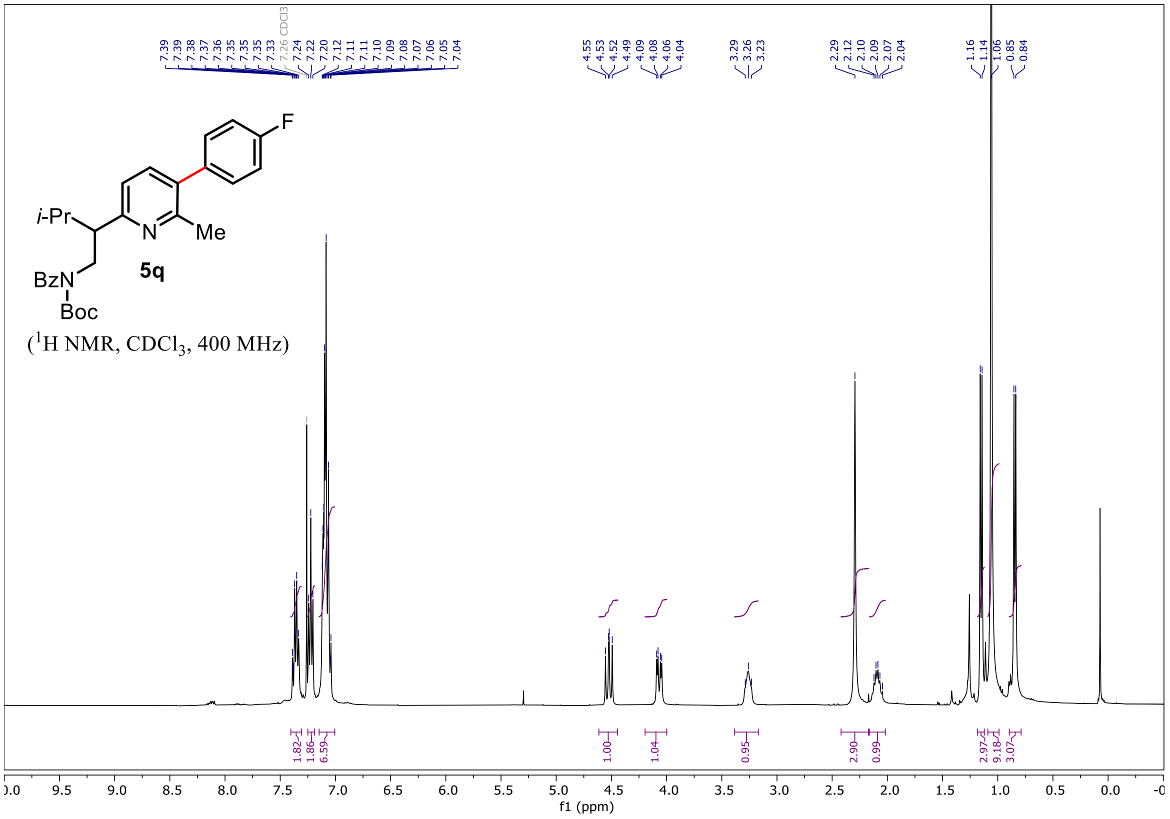

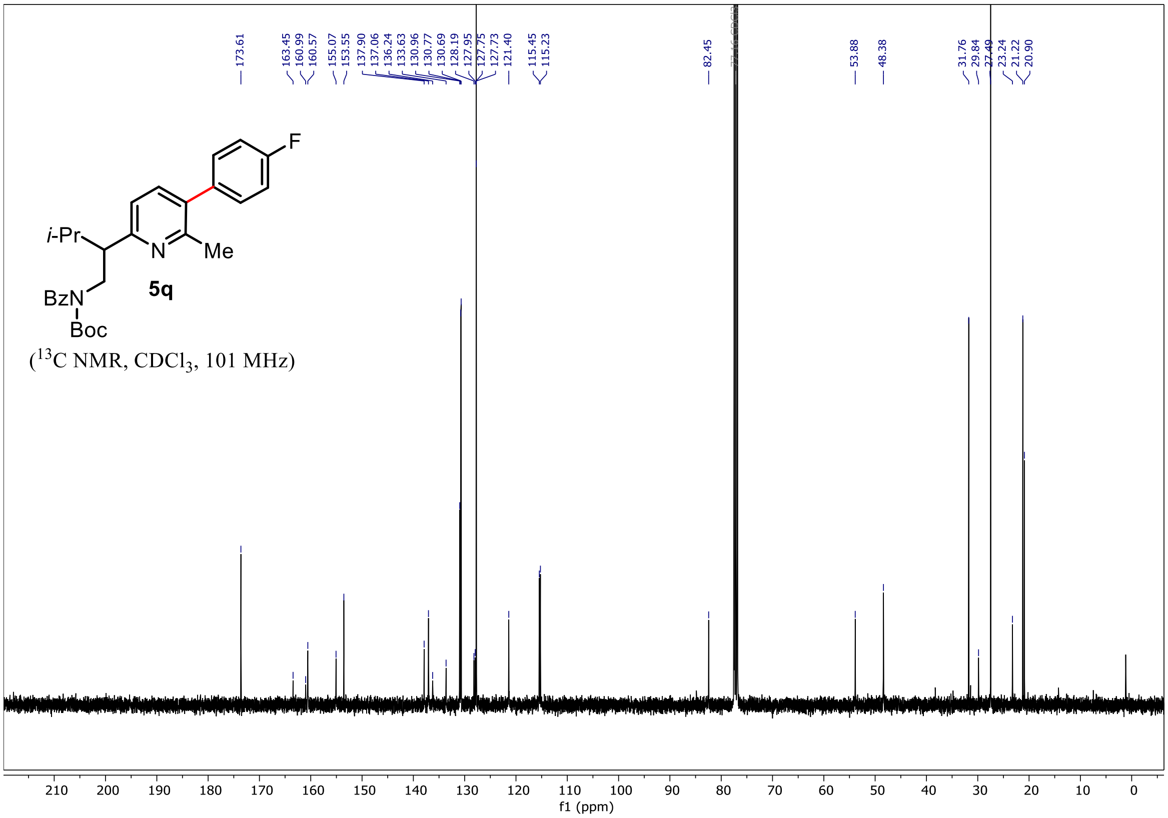

(13C NMR, $CDCl_3$, 101 MHz)

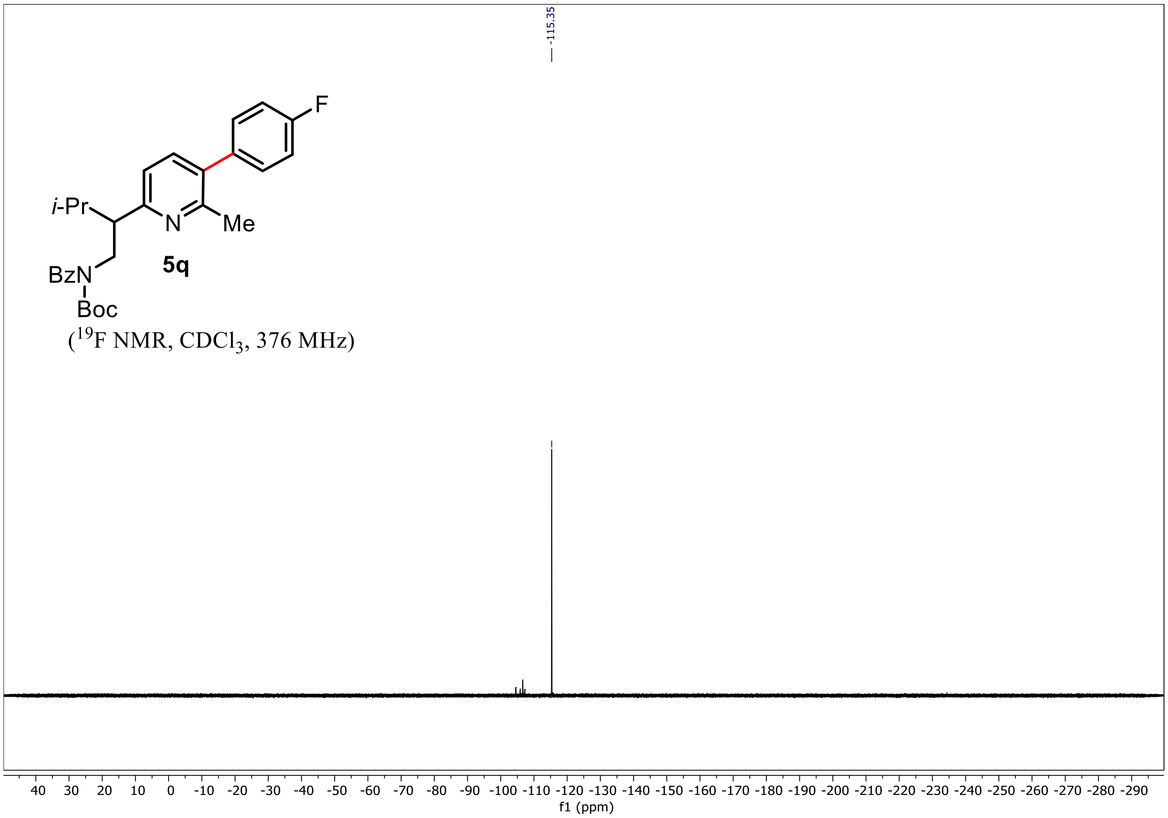

(19F NMR, $CDCl_3$, 376 MHz)

1D NOE NMR spectrum of **5q**, 500 MHz, $C_6D_6$

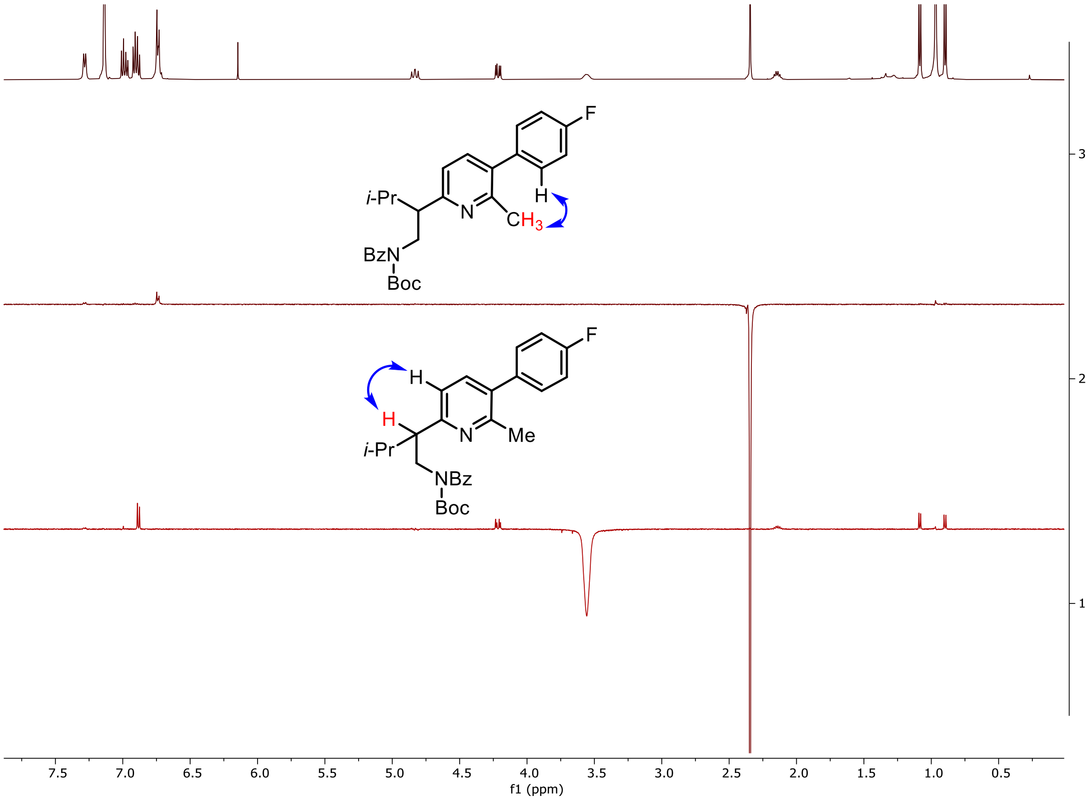


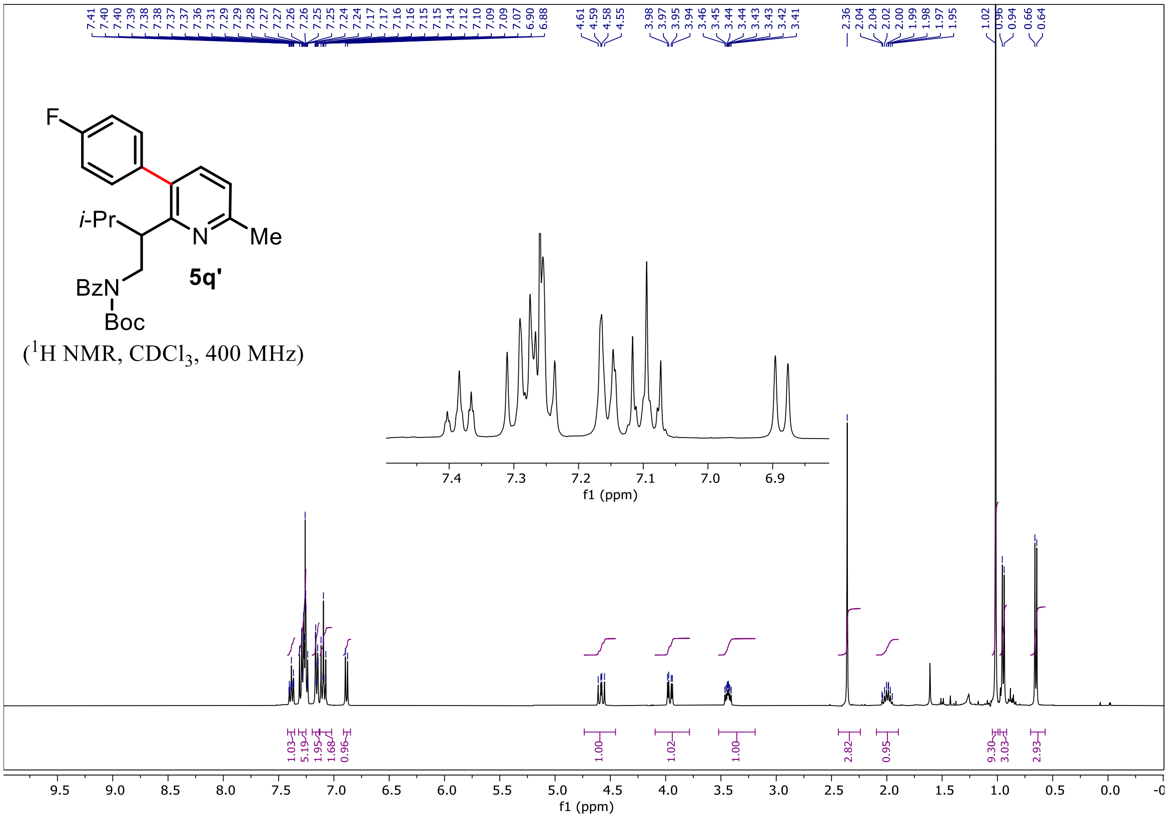

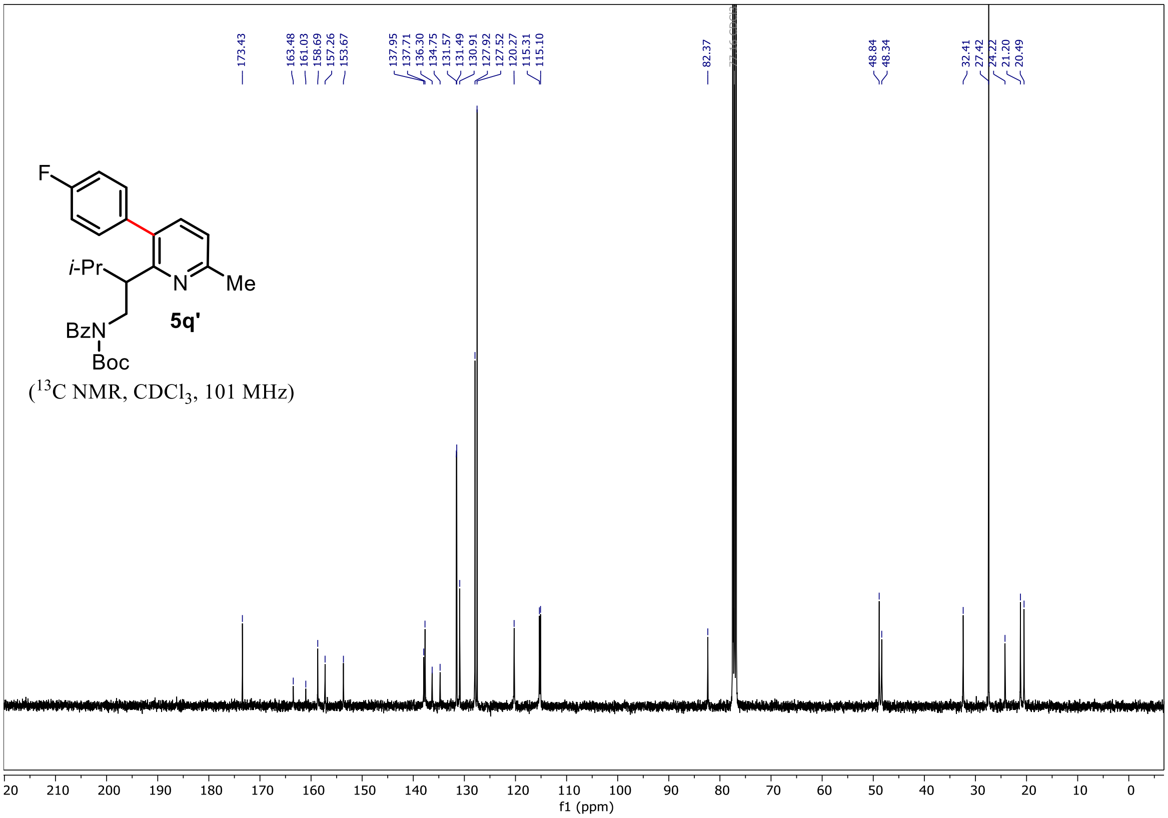
173.43
163.48
161.03
158.69
157.26
153.67
137.95
137.71
136.30
134.75
131.57
131.49
130.91
127.92
127.52
120.27
115.31
115.10
82.37
48.84
48.34
32.41
27.42
24.22
21.20
20.49
F
i-Pr
N
Me
BzN
Boc
5q'
($^{13}$C NMR, $CDCl_3$, 101 MHz)
f1 (ppm)

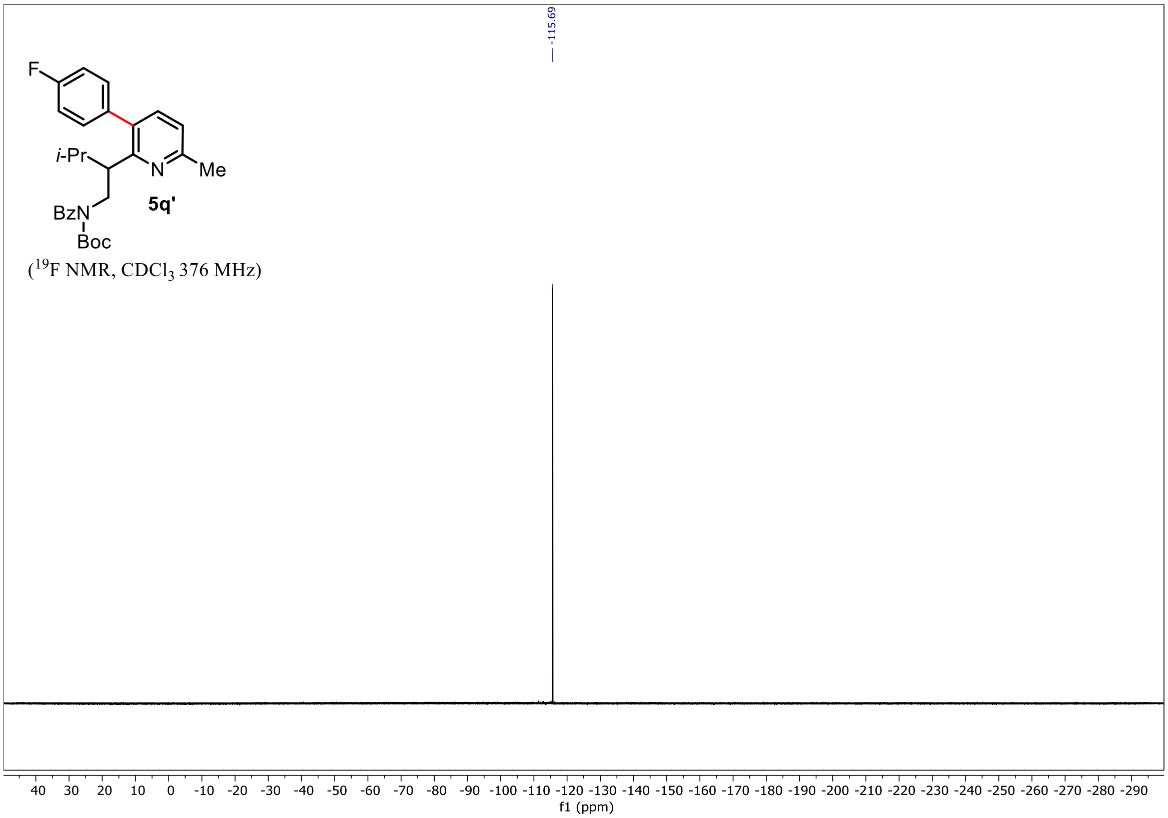
-115.69
F
i-Pr
N
Me
BzN
Boc
5q'
($^{19}$F NMR, $CDCl_3$ 376 MHz)
f1 (ppm)

1D NOE NMR spectrum of **5q'**, 500 MHz, $CDCl_3$

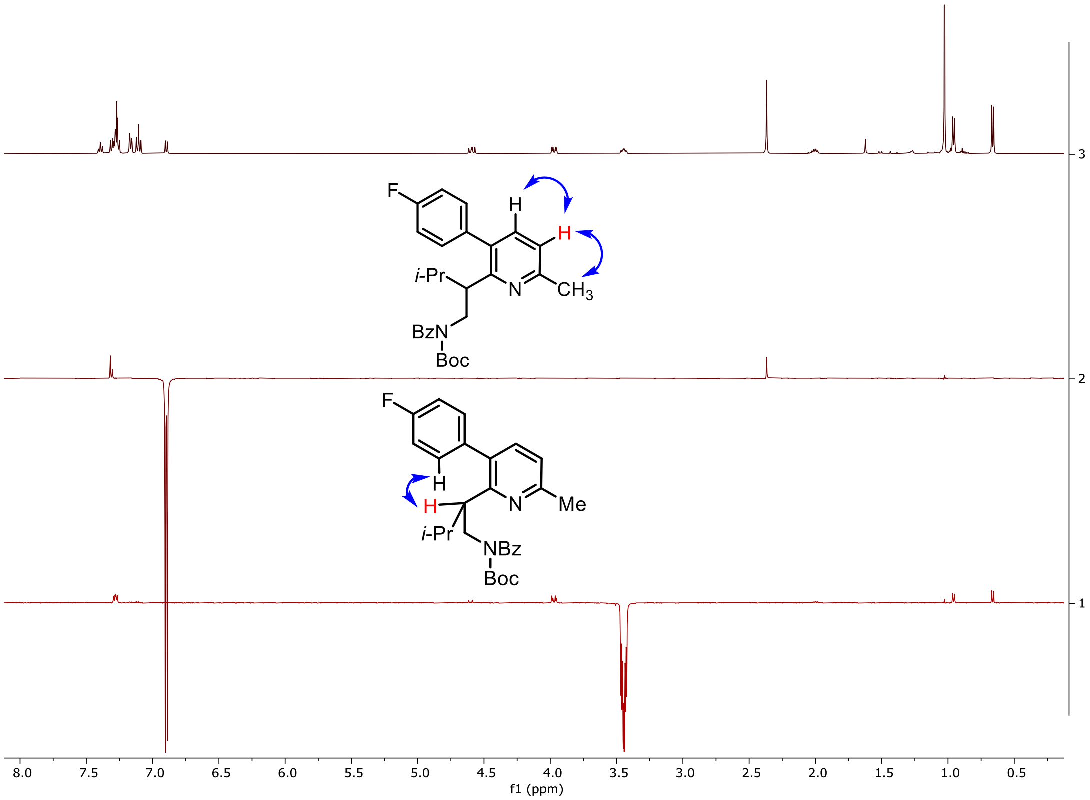


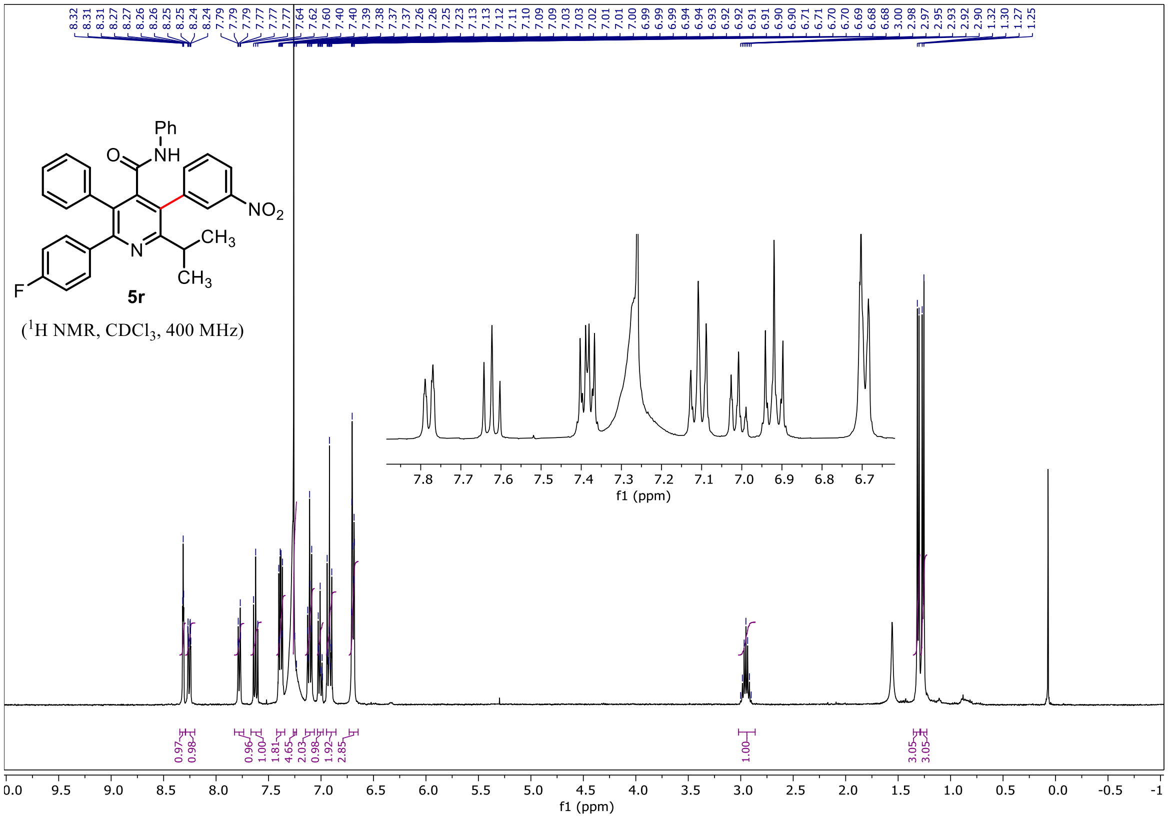

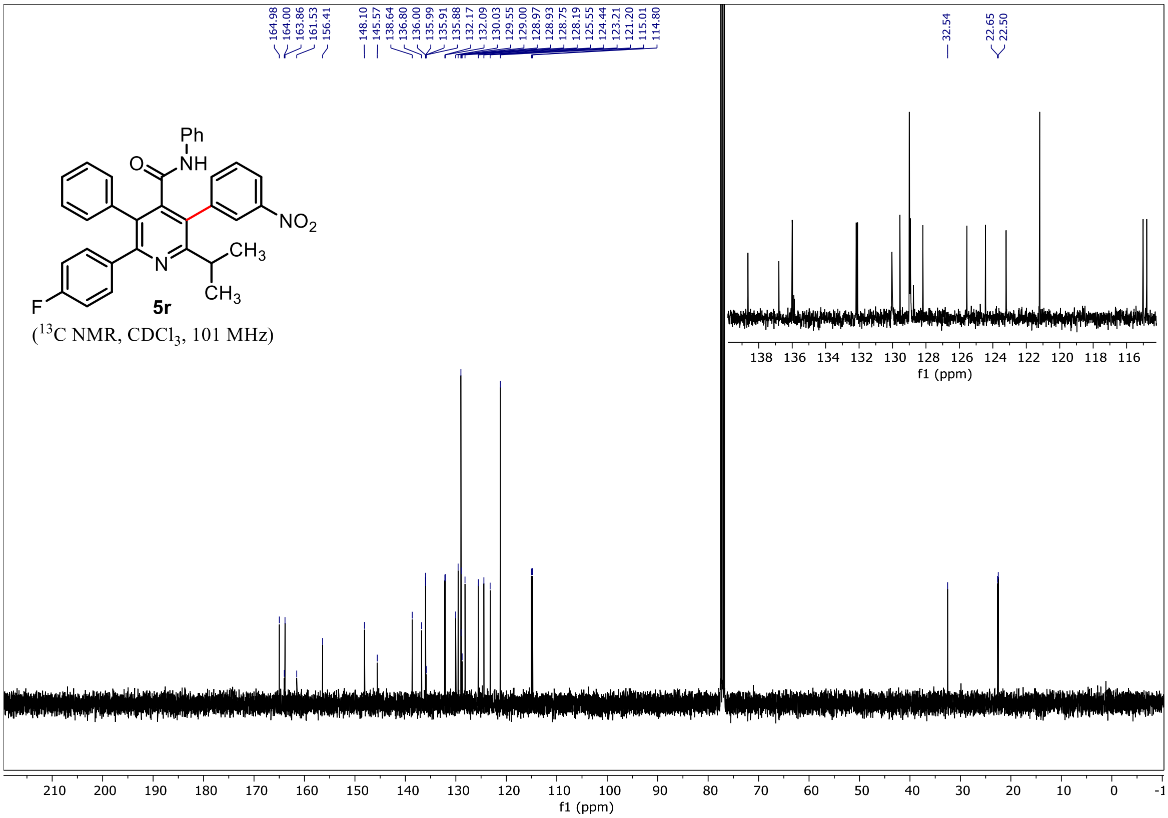

Ph
NH
O
NO2
CH3
CH3
N
F
5r
(13C NMR, CDCl3, 101 MHz)
164.98
164.00
163.86
161.53
156.41
148.10
145.57
138.64
136.80
136.00
135.99
135.91
135.88
132.17
132.09
130.03
129.55
129.00
128.97
128.93
128.75
128.19
125.55
124.44
123.21
121.20
115.01
114.80
32.54
22.65
22.50
f1 (ppm)


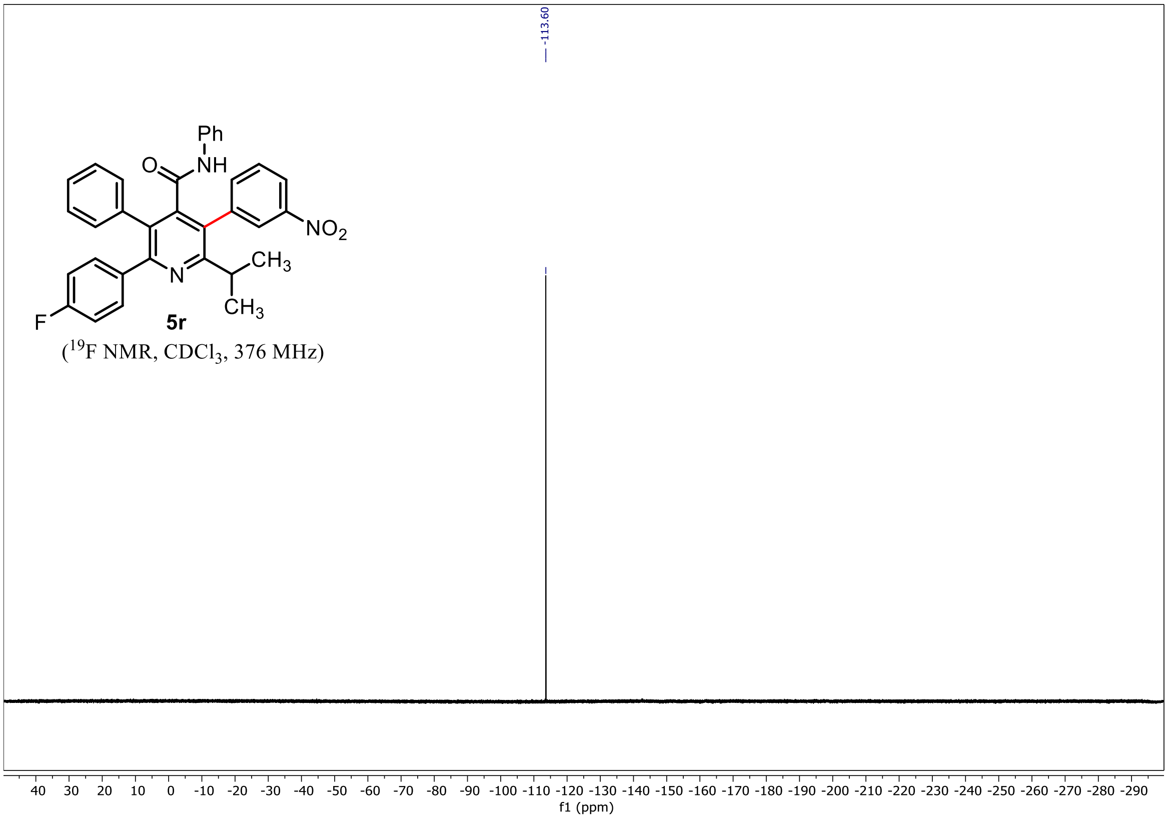

-113.60
Ph
NH
O
NO2
CH3
CH3
N
F
5r
(19F NMR, CDCl3, 376 MHz)
f1 (ppm)

1D NOE NMR spectrum of **5r**, 500 MHz, $CDCl_3$

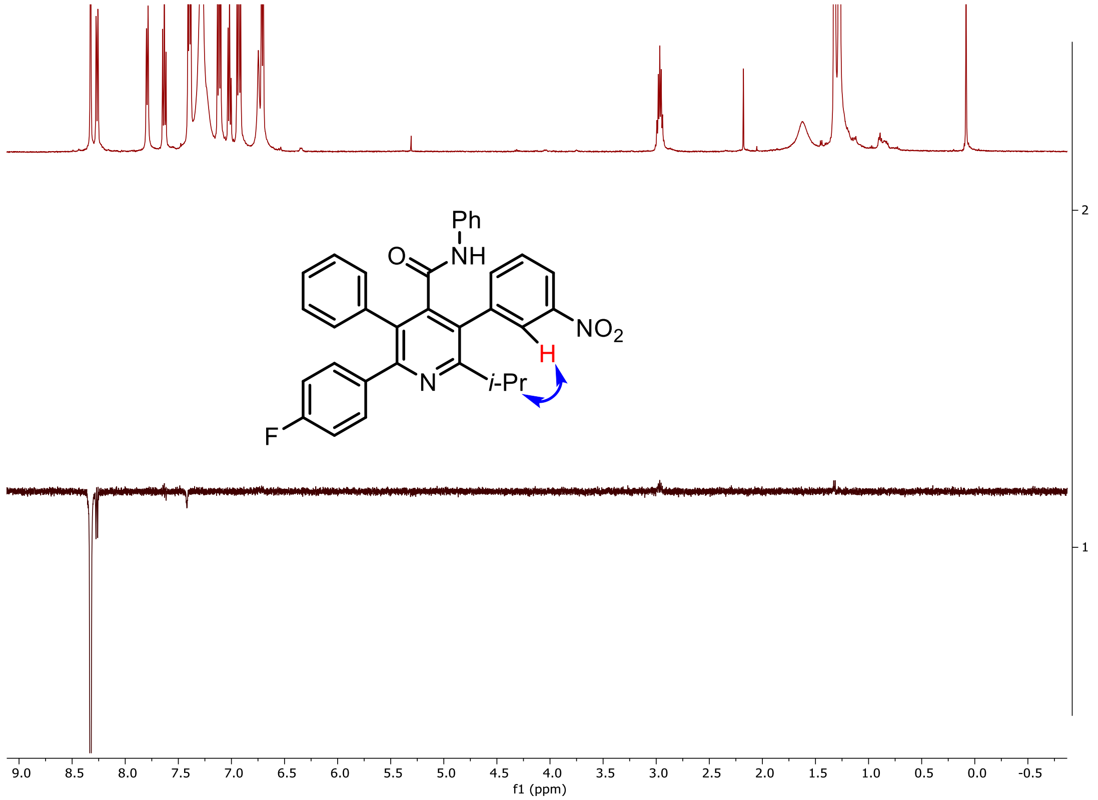


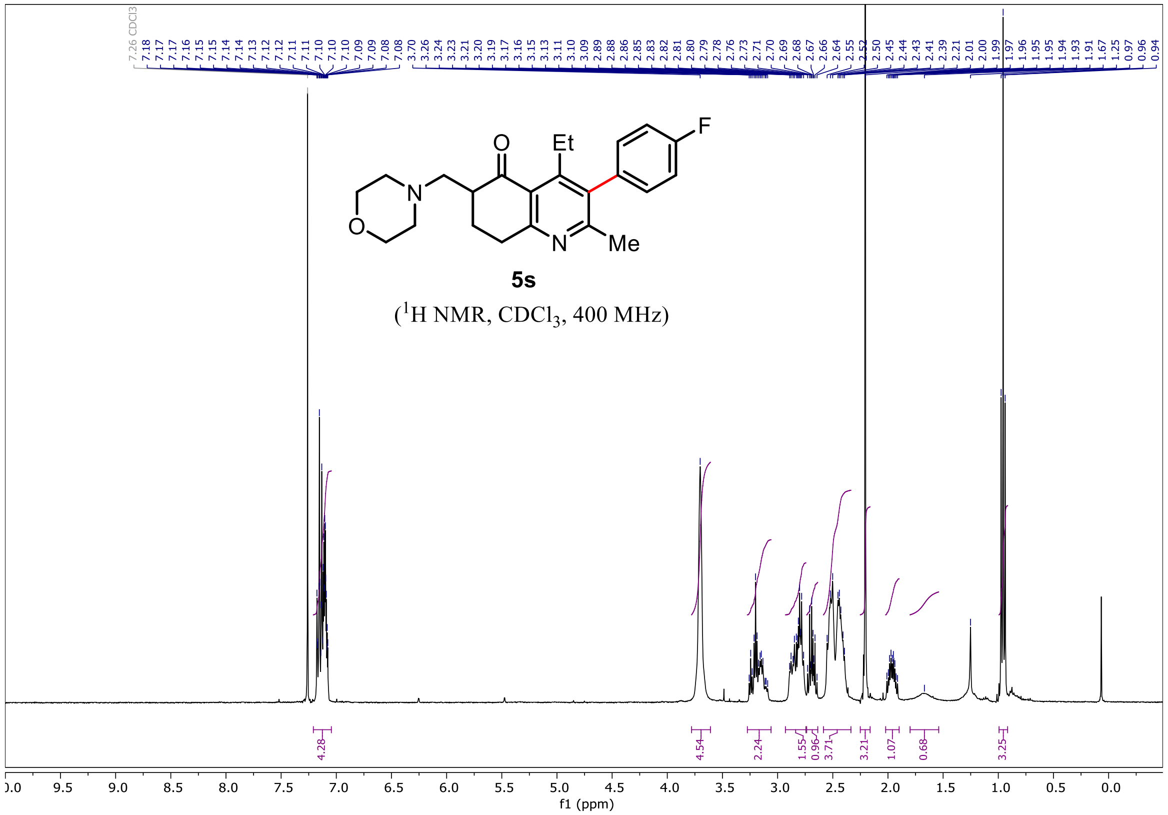

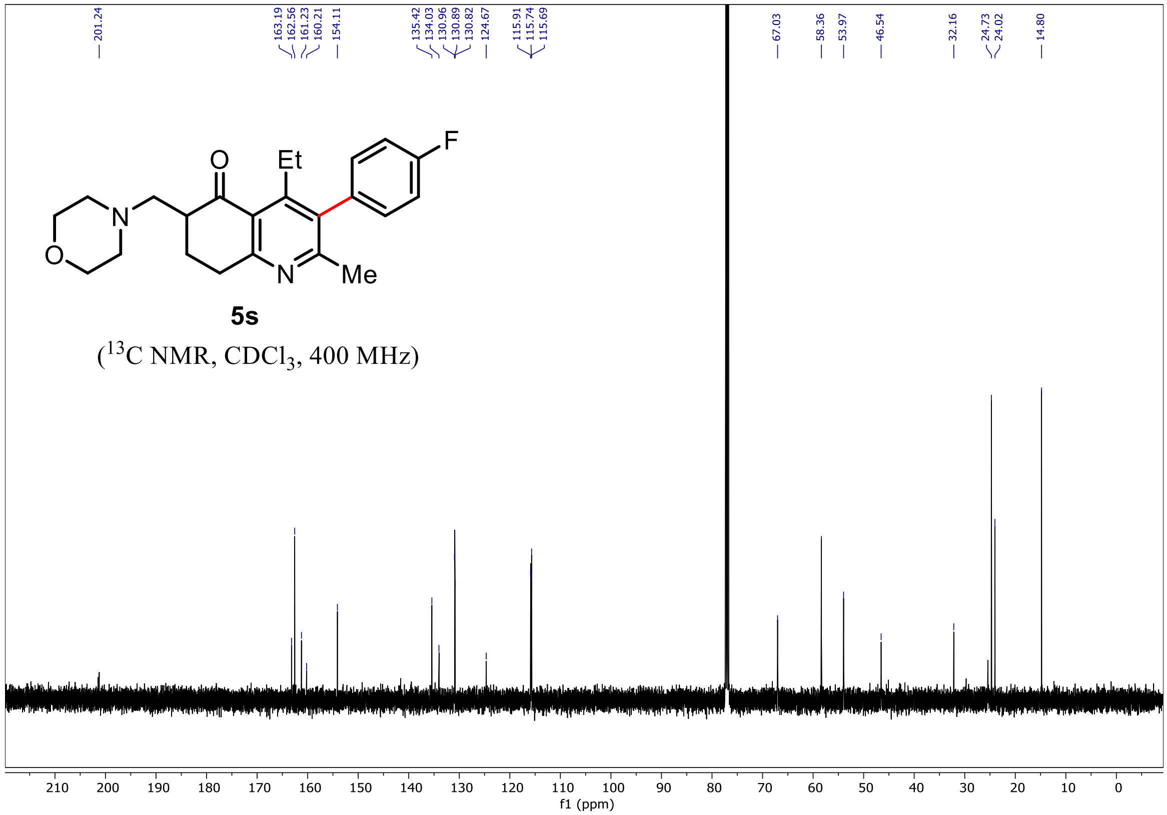


(13C NMR, $CDCl_3$, 400 MHz)

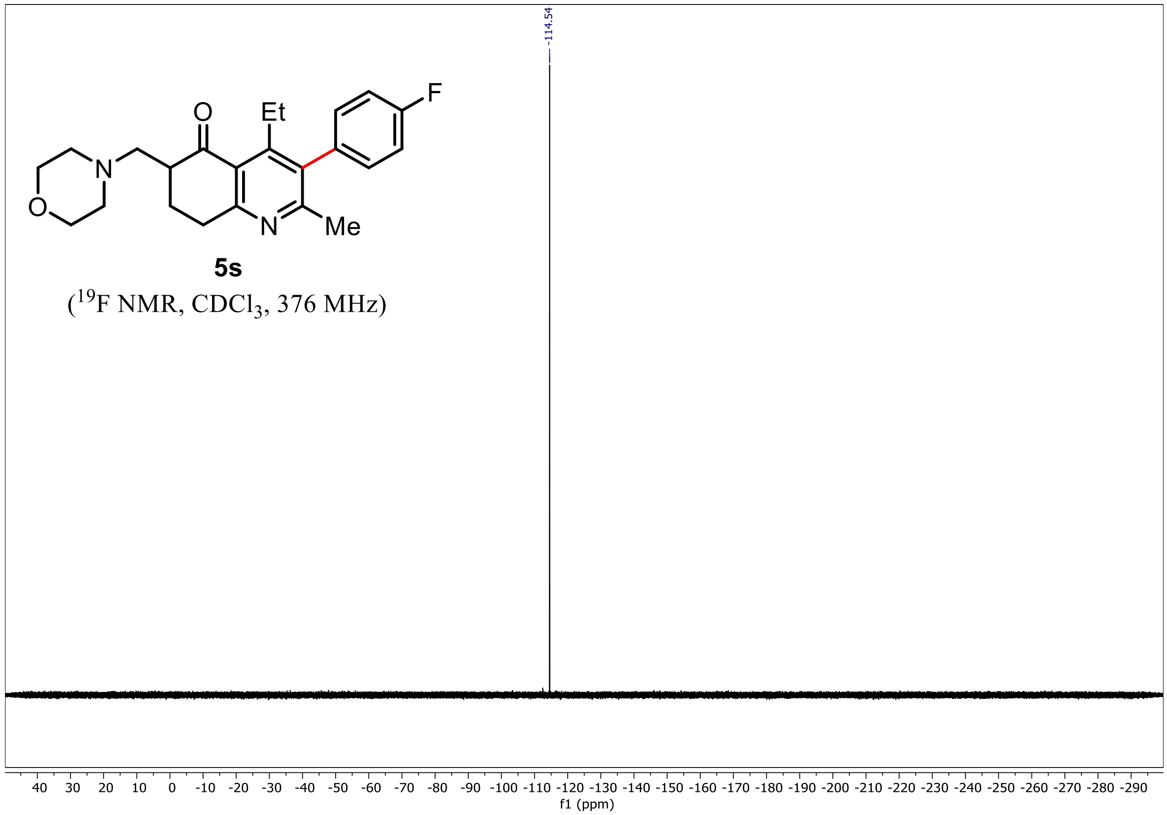


(19F NMR, $CDCl_3$, 376 MHz)

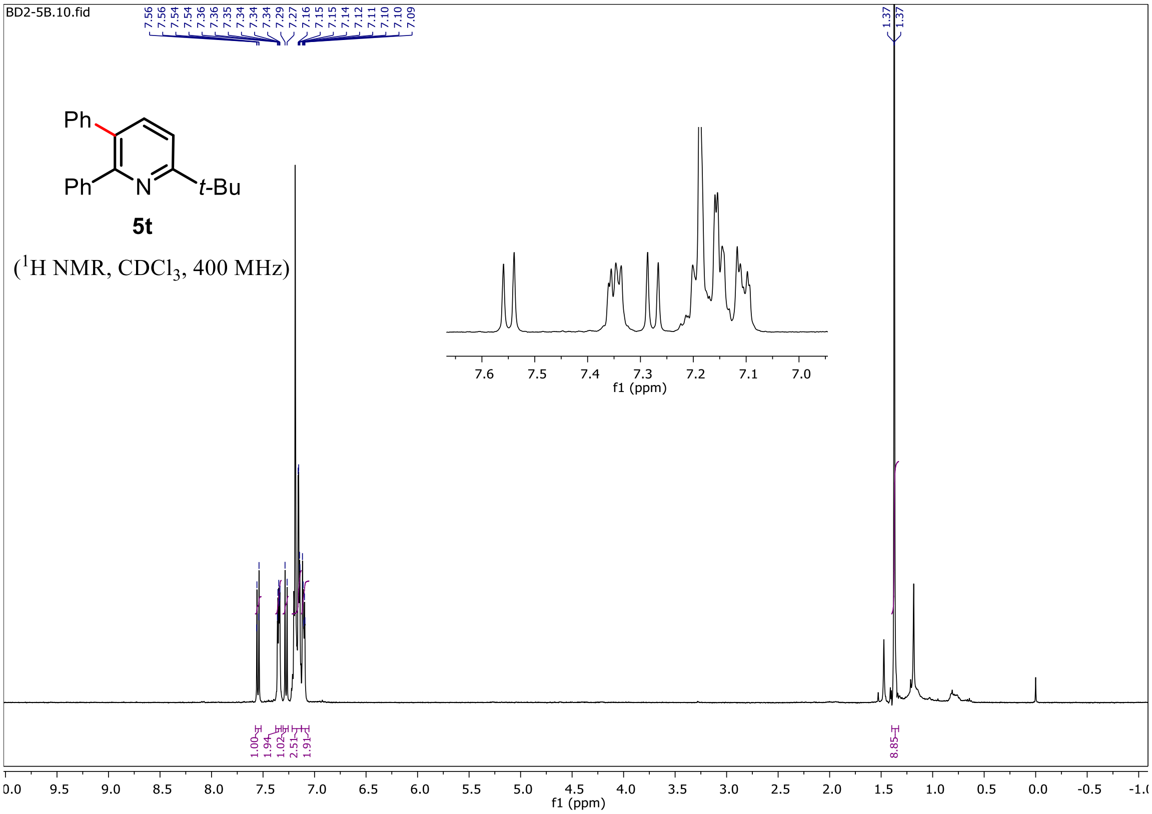
BD2-5B.10.fid
5t
(1H NMR, CDCl3, 400 MHz)
f1 (ppm)

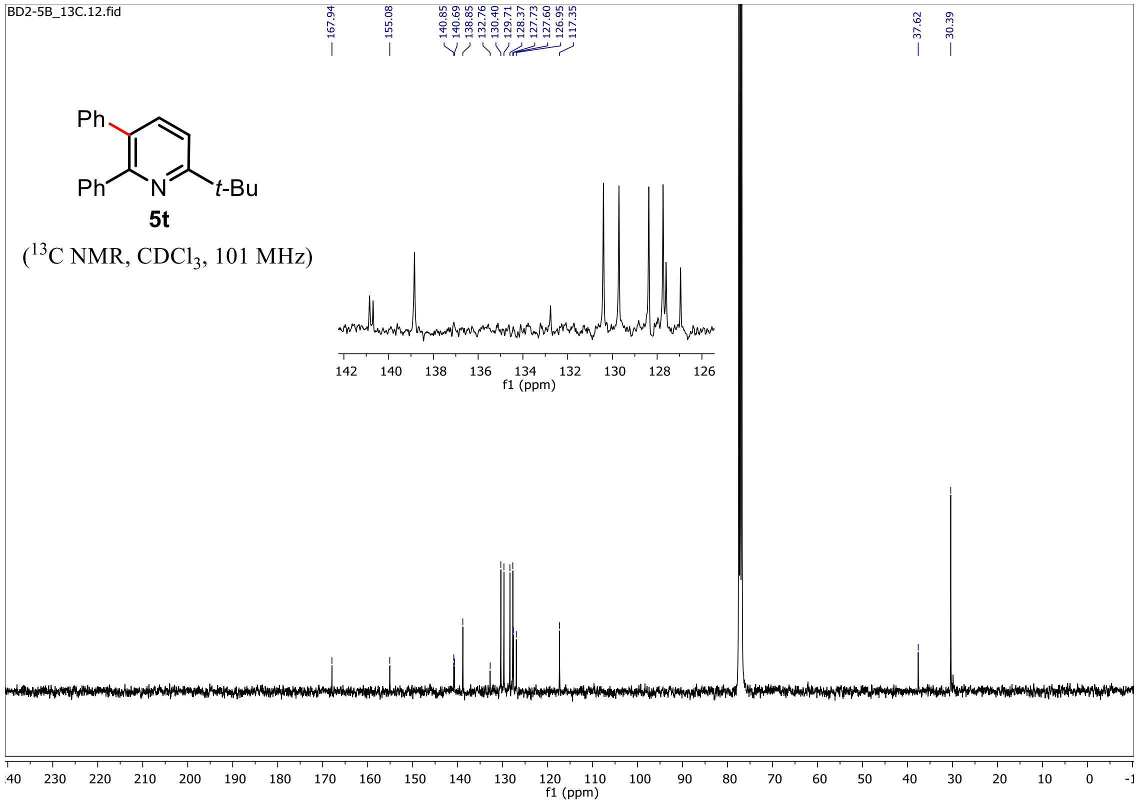
BD2-5B_13C.12.fid
5t
(13C NMR, CDCl3, 101 MHz)
f1 (ppm)

1D NOE NMR spectrum of **5t**, 500 MHz, $CDCl_3$

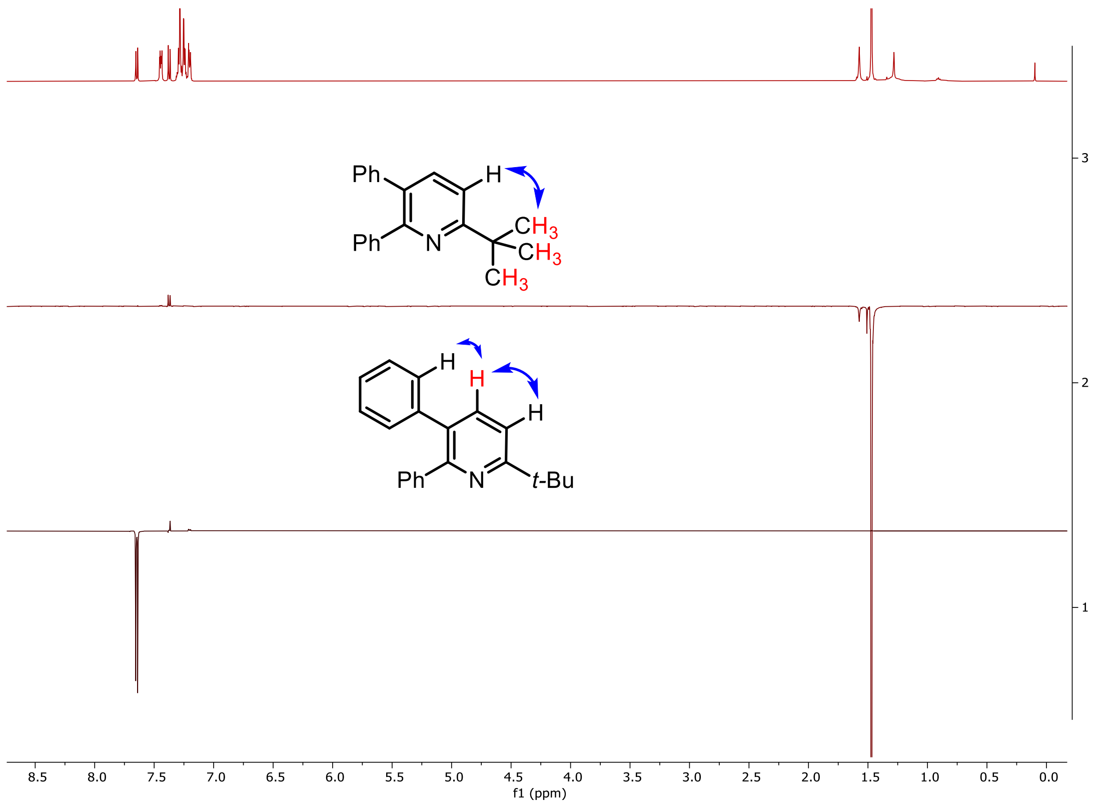


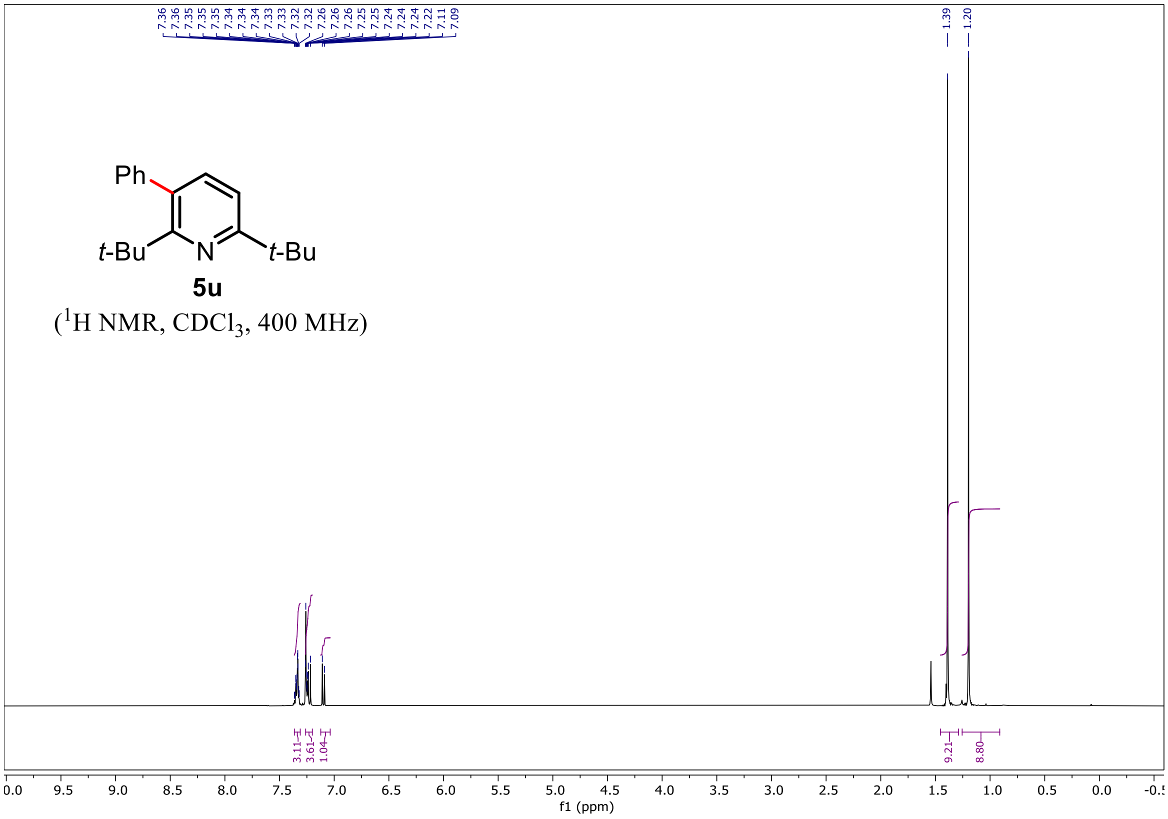

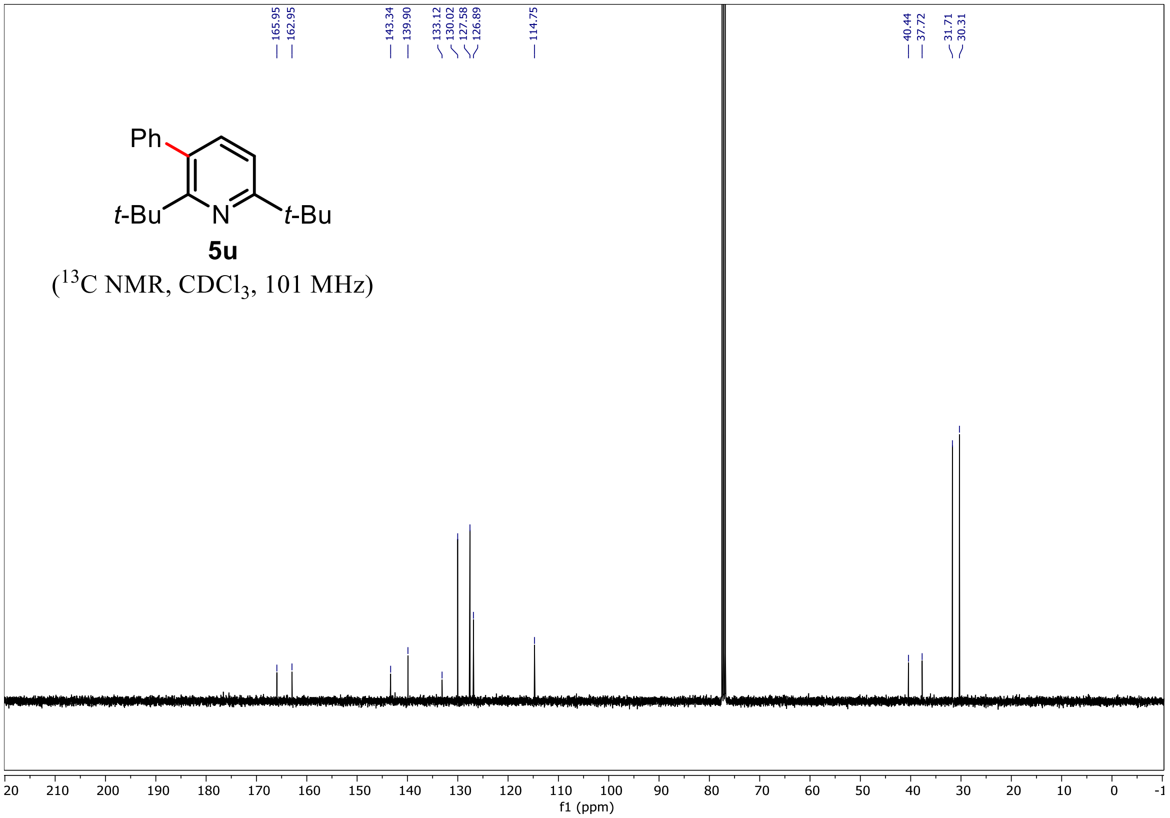


1D NOE NMR spectrum of **5u**, 500 MHz, $CDCl_3$

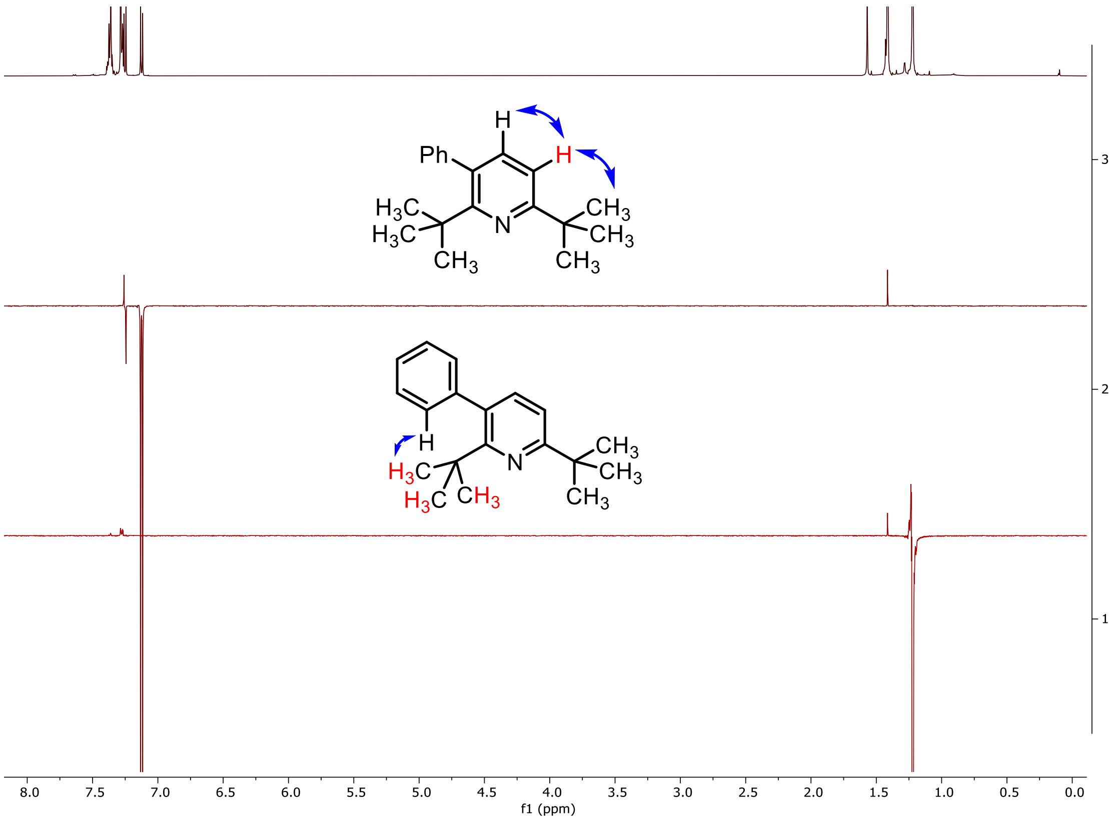

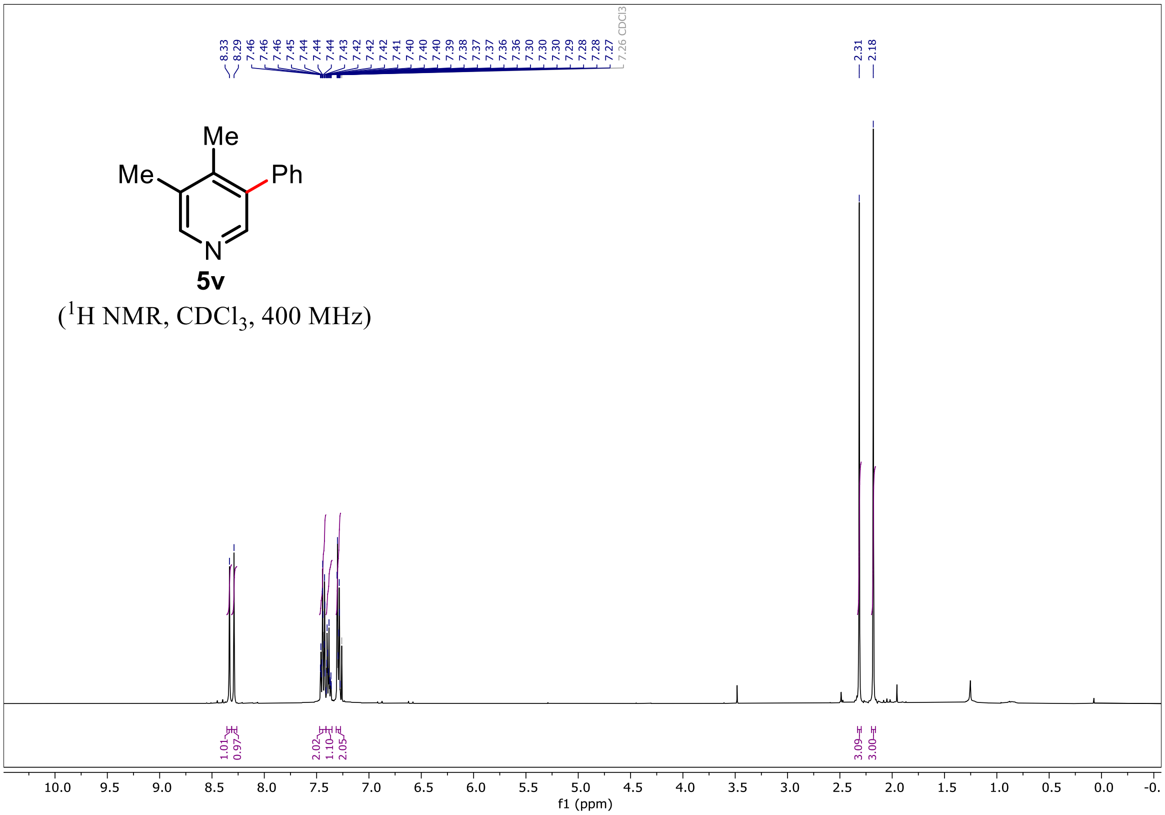


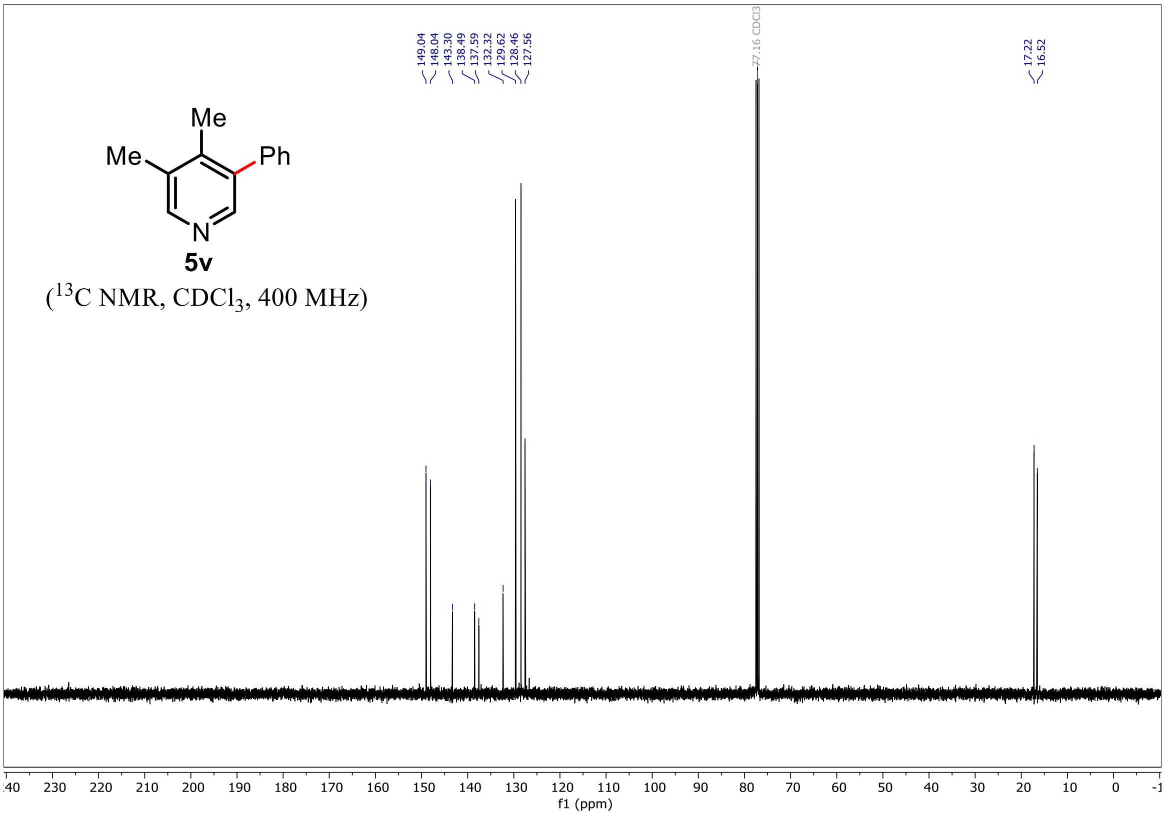


1D NOE NMR spectrum of **5v**, 500 MHz, $CDCl_3$

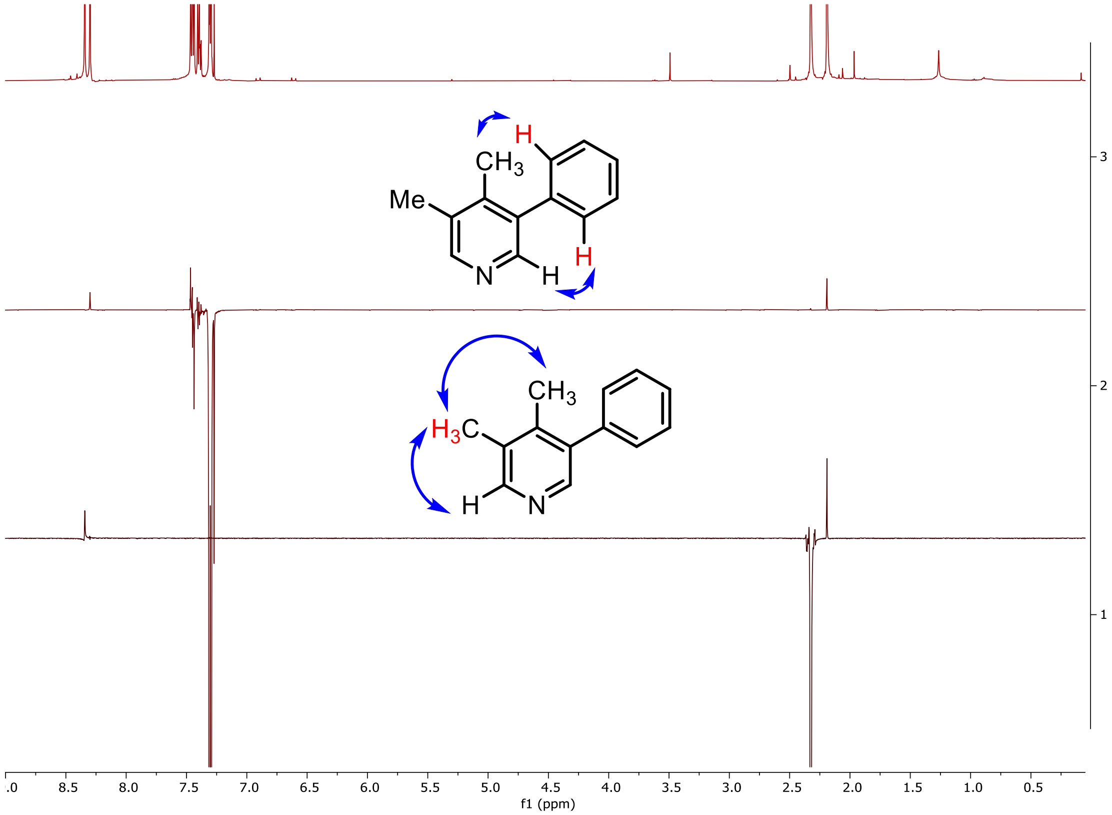

CH3
Me
H
N
H
H
H3C
CH3
H
N
f1 (ppm)


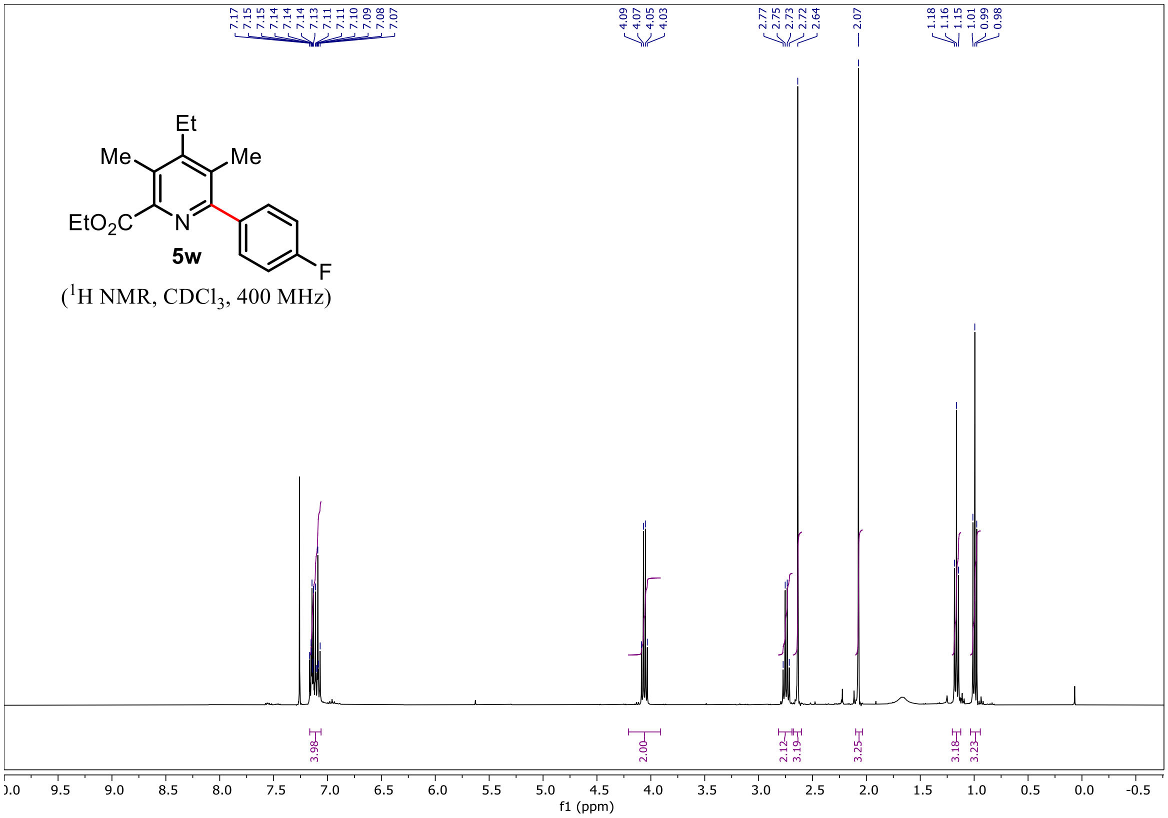

Et
Me
Me
EtO2C
N
5w
F
(1H NMR, CDCl3, 400 MHz)
f1 (ppm)

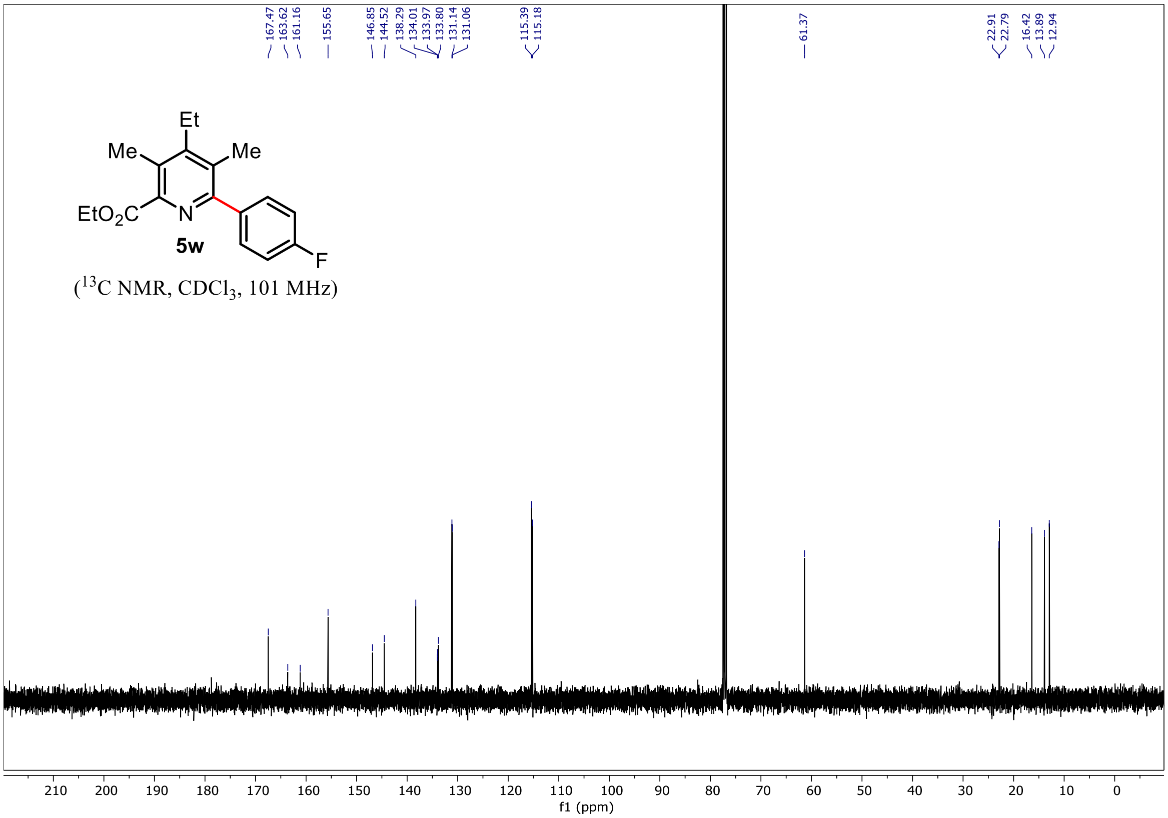
167.47
163.62
161.16
155.65
146.85
144.52
138.29
134.01
133.97
133.80
131.14
131.06
115.39
115.18
61.37
22.91
22.79
16.42
13.89
12.94
Et
Me
Me
EtO2C
N
F
5w
(13C NMR, CDCl3, 101 MHz)
210 200 190 180 170 160 150 140 130 120 110 100 90 80 70 60 50 40 30 20 10 0
f1 (ppm)

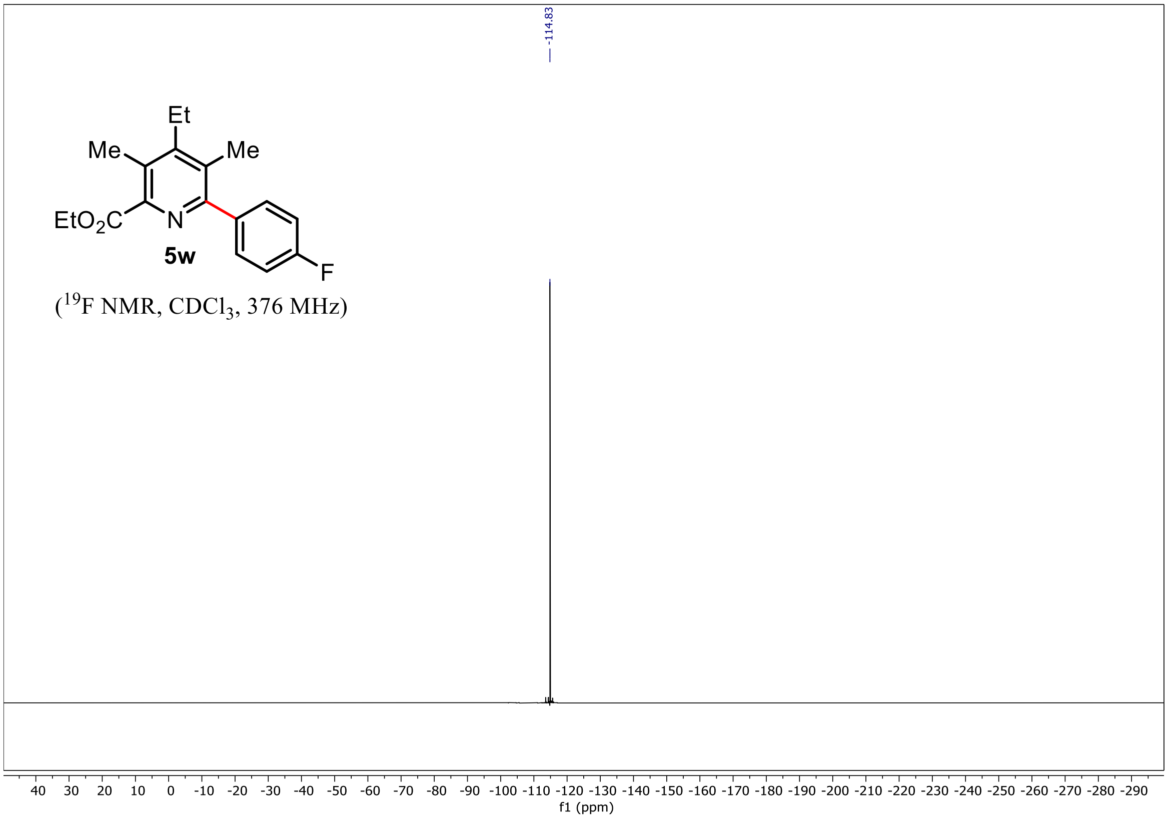
-114.83
Et
Me
Me
EtO2C
N
F
5w
(19F NMR, CDCl3, 376 MHz)
40 30 20 10 0 -10 -20 -30 -40 -50 -60 -70 -80 -90 -100 -110 -120 -130 -140 -150 -160 -170 -180 -190 -200 -210 -220 -230 -240 -250 -260 -270 -280 -290
f1 (ppm)

1D NOE NMR spectrum of **5w**, 500 MHz, $CDCl_3$

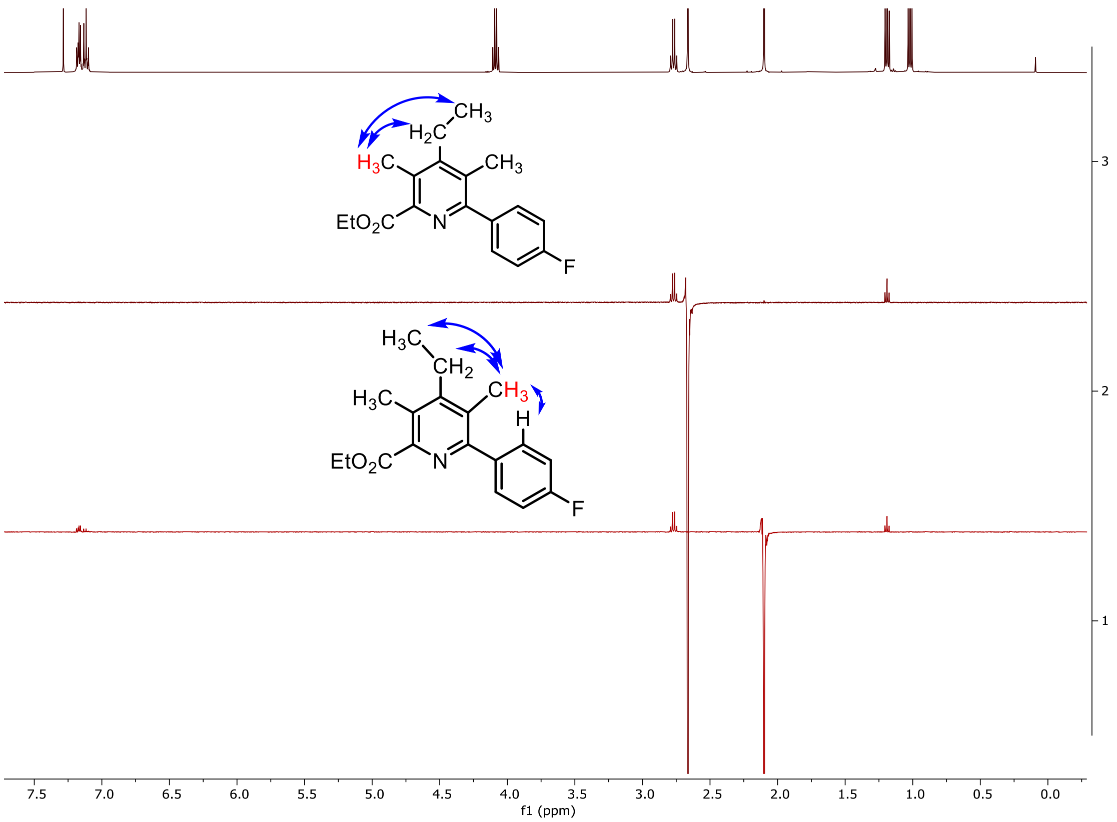


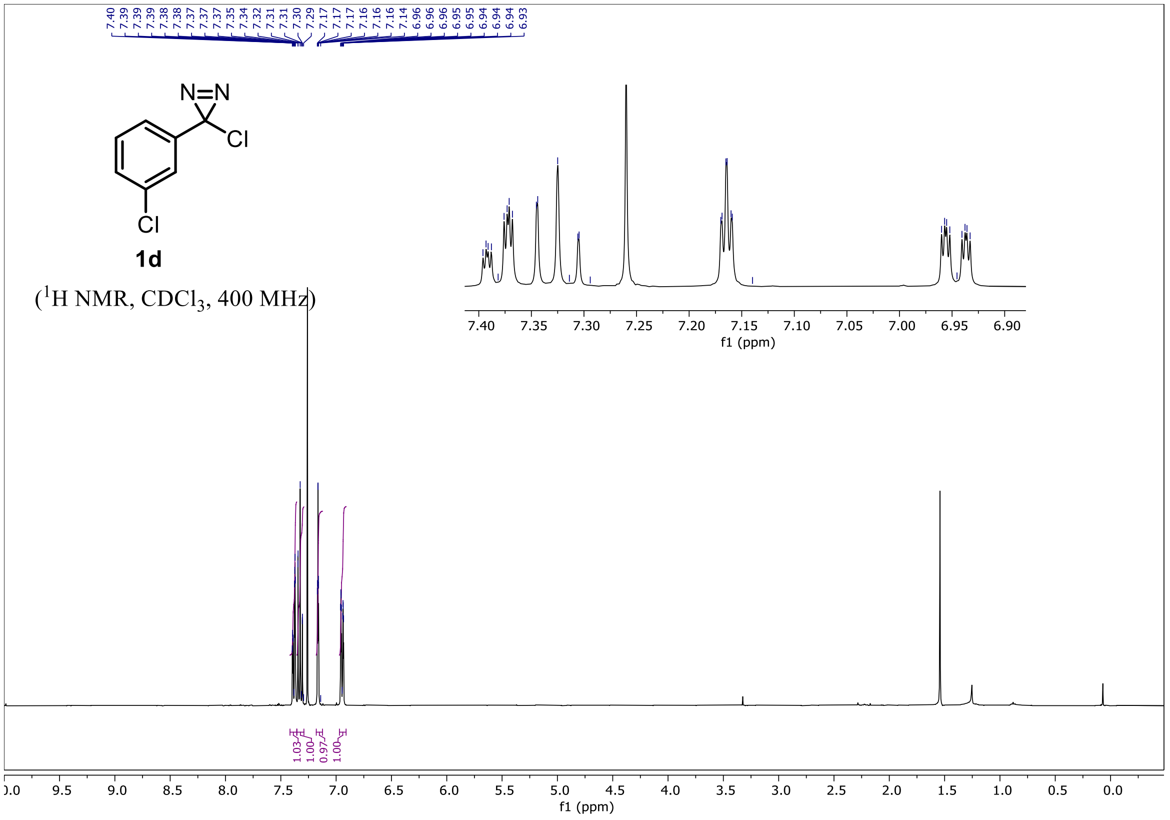

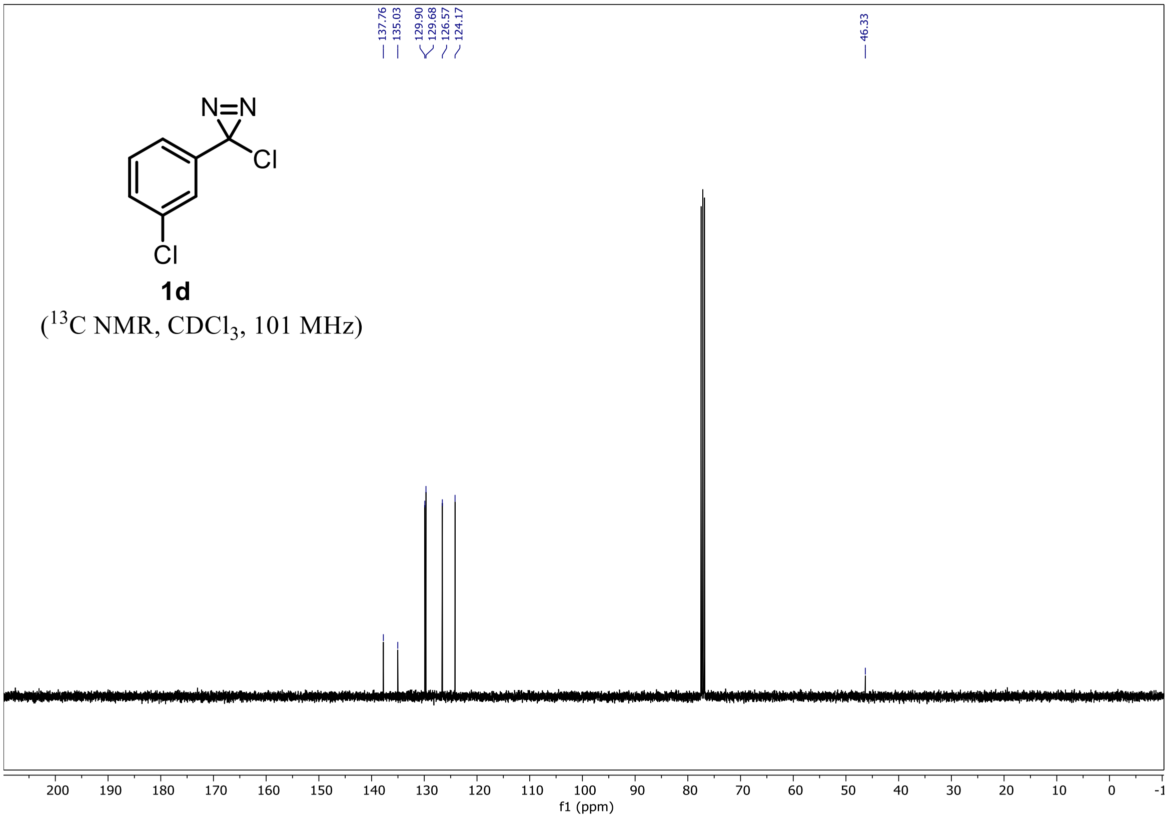


**1d**

($^{13}$C NMR, $CDCl_3$, 101 MHz)

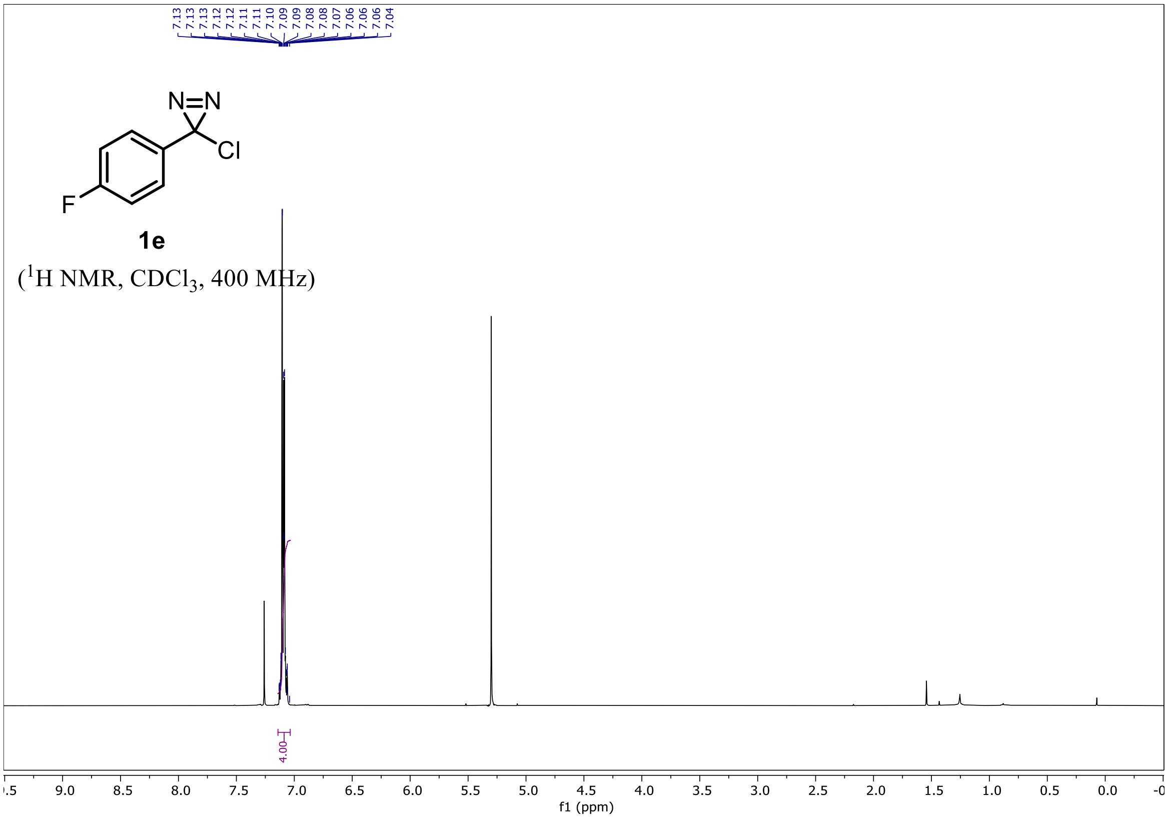


**1e**

($^{1}$H NMR, $CDCl_3$, 400 MHz)

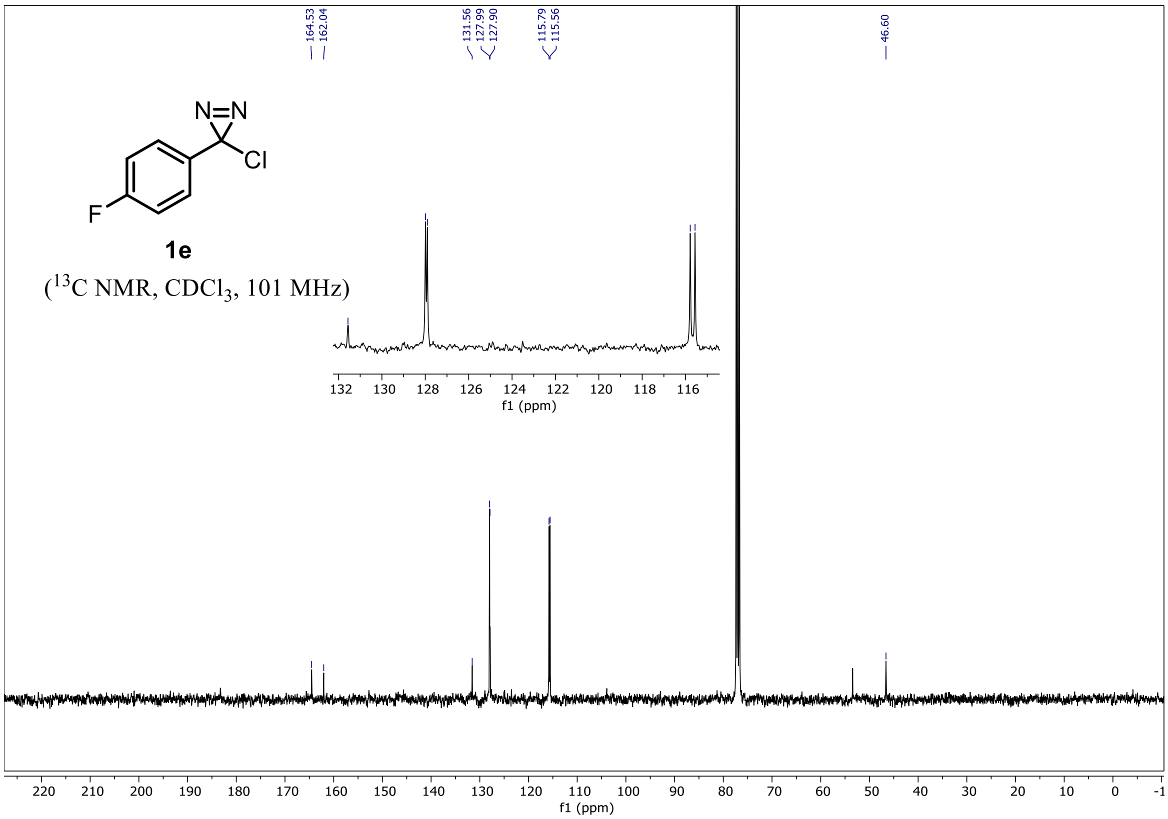
164.53
162.04
131.56
127.99
127.90
115.79
115.56
46.60
1e
(13C NMR, CDCl3, 101 MHz)
f1 (ppm)

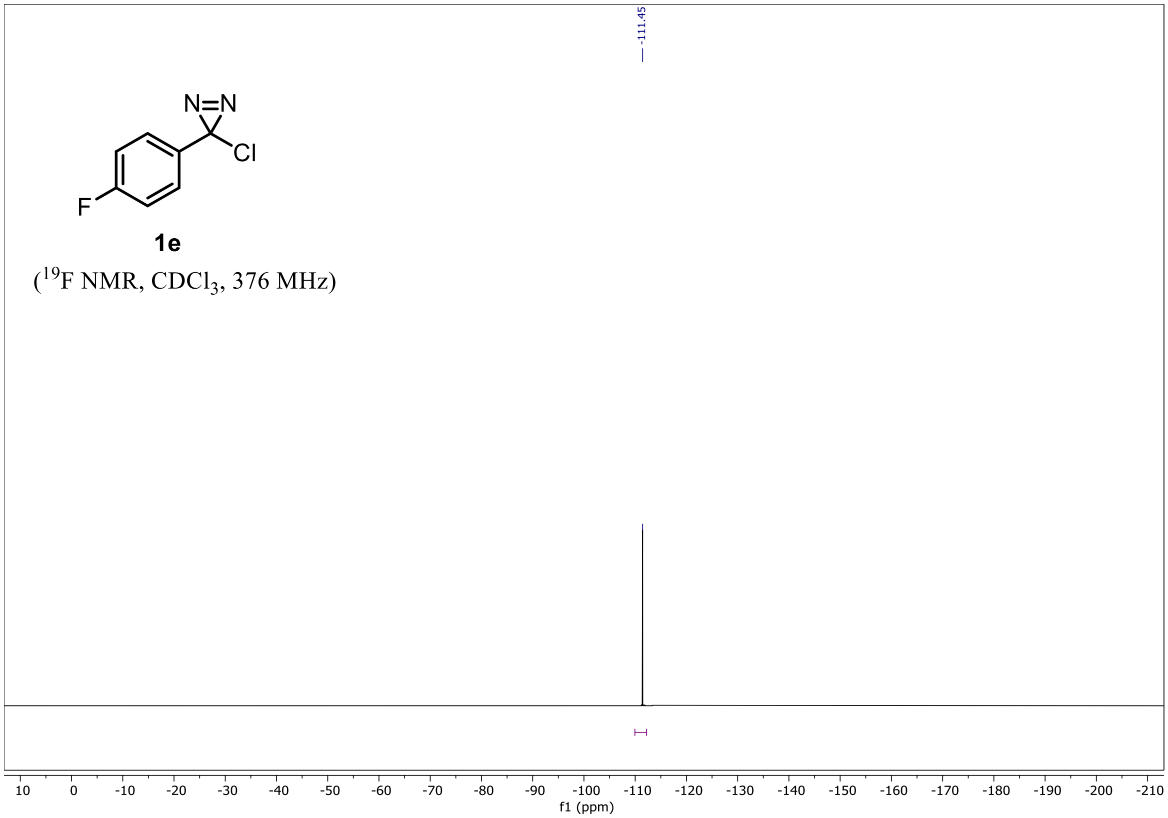
-111.45
1e
(19F NMR, CDCl3, 376 MHz)
f1 (ppm)

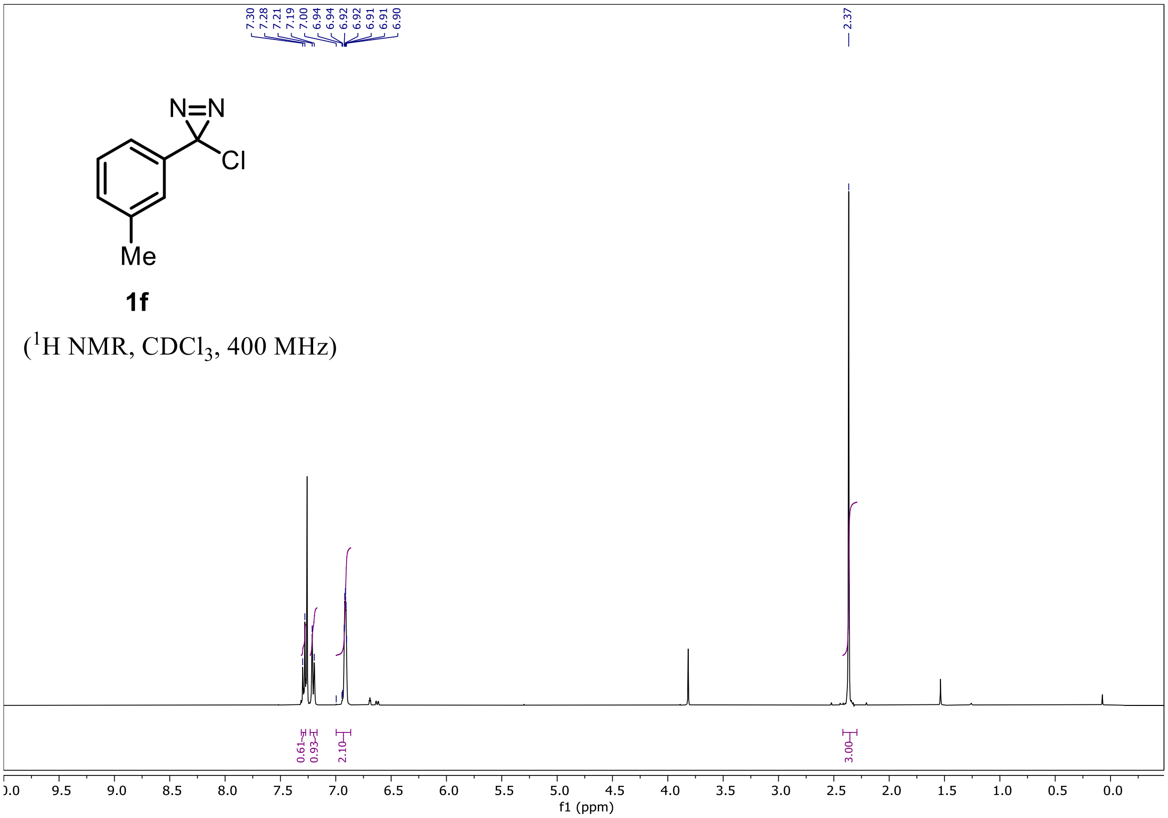
7.30
7.28
7.21
7.19
7.00
6.94
6.94
6.92
6.92
6.91
6.91
6.90
2.37
N=N
Cl
Me
1f
($^{1}$H NMR, $CDCl_3$, 400 MHz)
0.61
0.93
2.10
3.00
f1 (ppm)

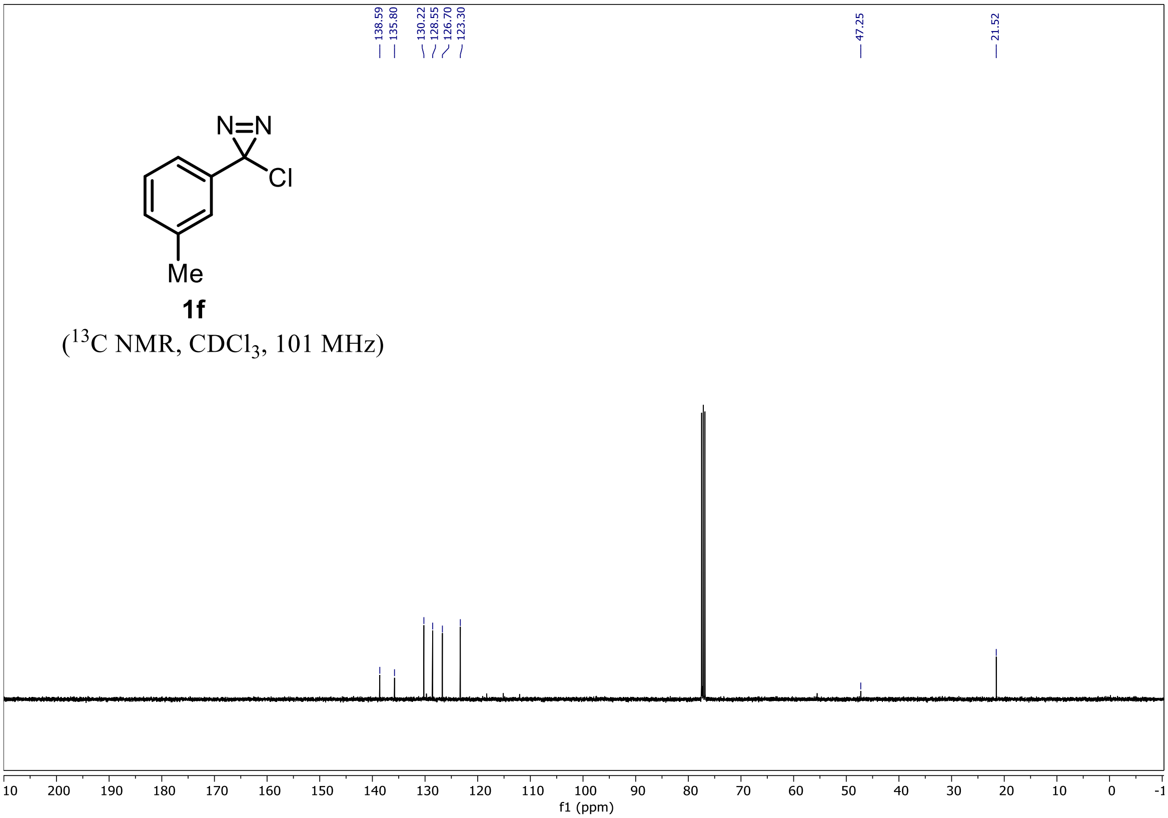
138.59
135.80
130.22
128.55
126.70
123.30
47.25
21.52
N=N
Cl
Me
1f
($^{13}$C NMR, $CDCl_3$, 101 MHz)
f1 (ppm)

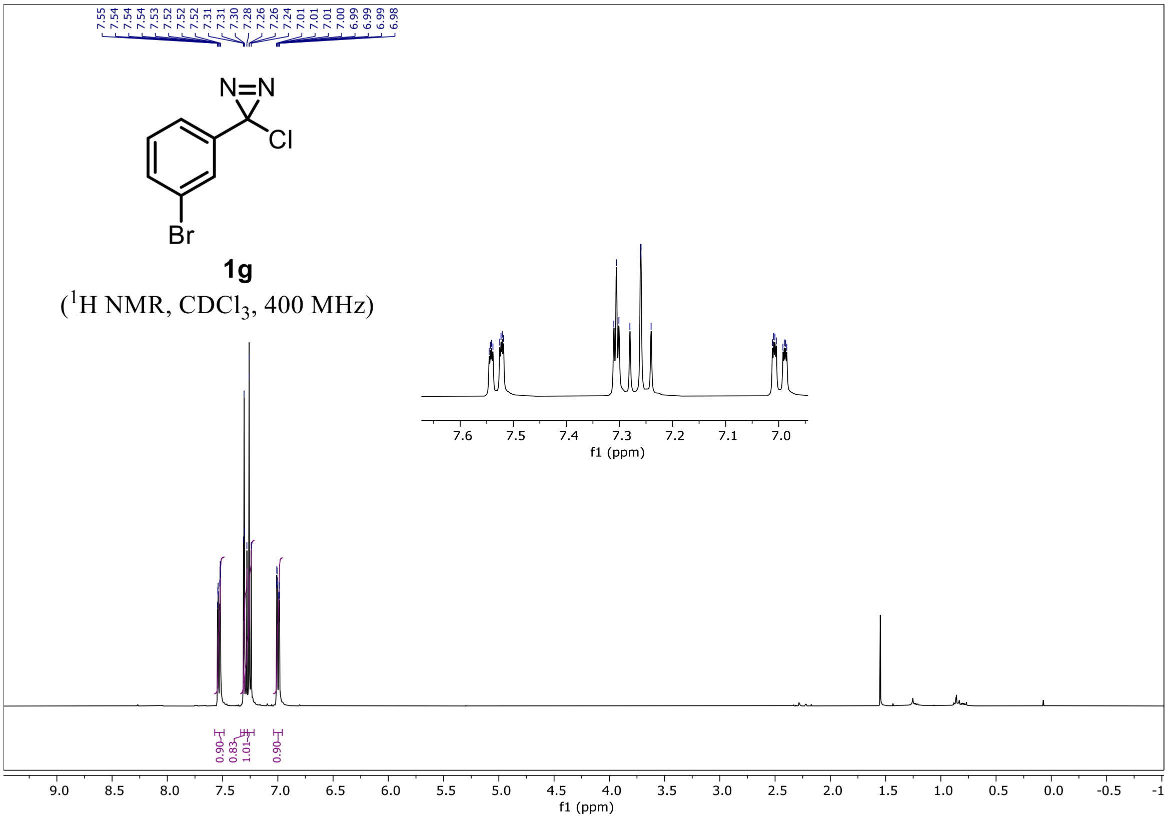

**1g**

($^{1}H$ NMR, $CDCl_3$, 400 MHz)

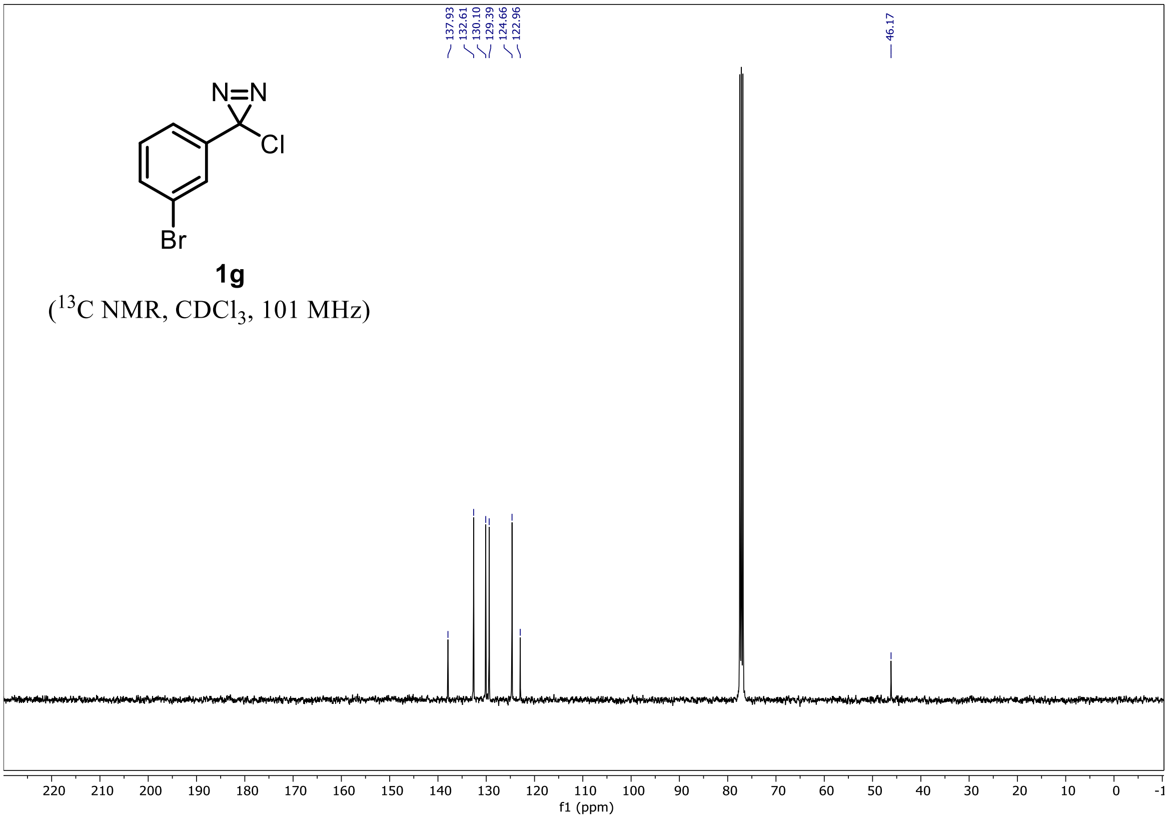

**1g**

($^{13}C$ NMR, $CDCl_3$, 101 MHz)

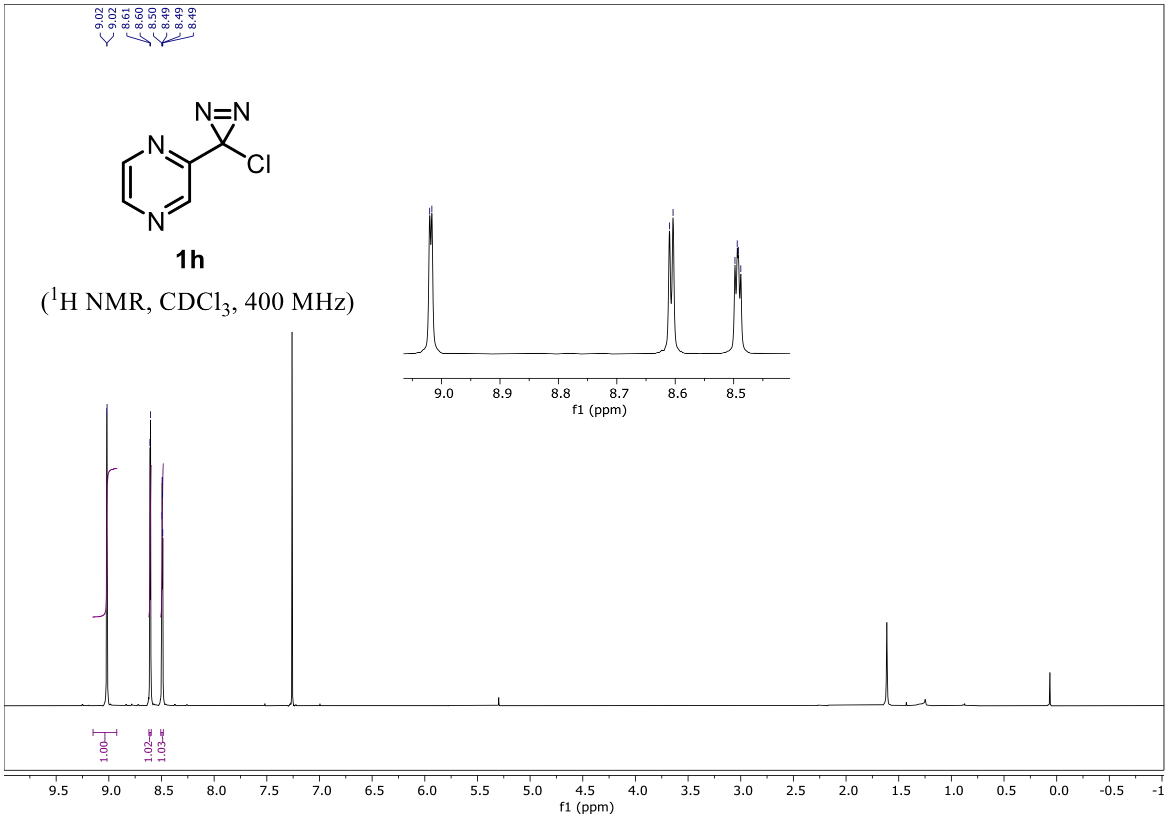
9.02
9.02
8.61
8.60
8.50
8.49
8.49
8.49
1h
(¹H NMR, CDCl3, 400 MHz)
1.00
1.02
1.03
f1 (ppm)

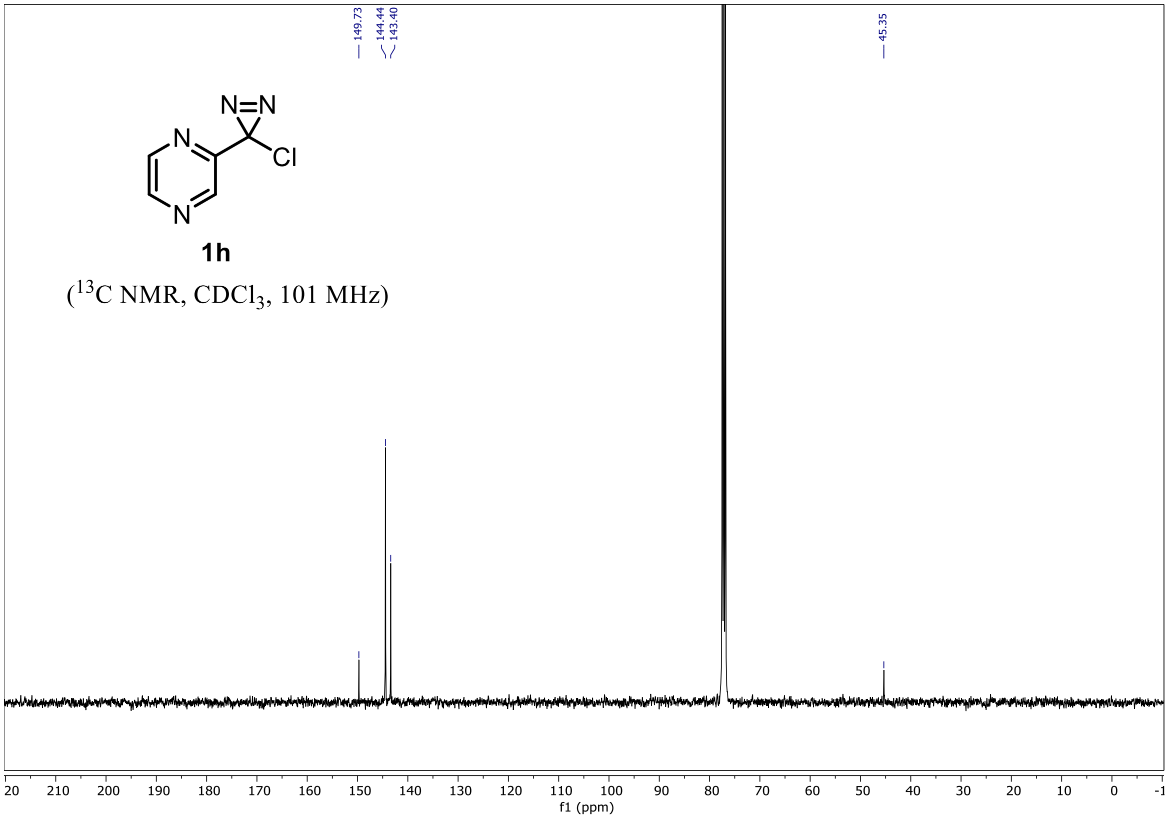
149.73
144.44
143.40
45.35
1h
(¹³C NMR, CDCl3, 101 MHz)
f1 (ppm)

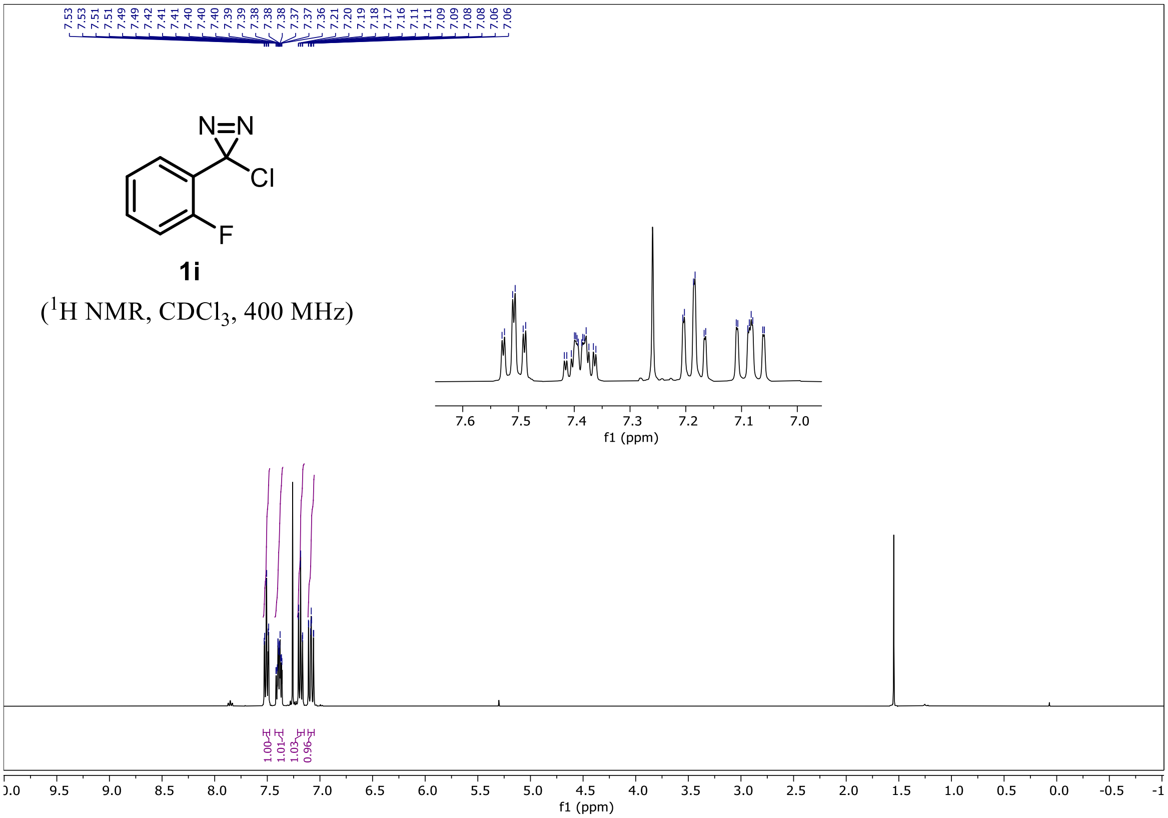
7.53 7.53 7.51 7.51 7.49 7.49 7.42 7.41 7.41 7.40 7.40 7.39 7.39 7.38 7.38 7.38 7.37 7.37 7.36 7.21 7.20 7.19 7.18 7.17 7.16 7.11 7.11 7.09 7.09 7.08 7.08 7.06 7.06
N=N
Cl
F
1i
(1H NMR, CDCl3, 400 MHz)
1.00
1.01
1.03
0.96
f1 (ppm)

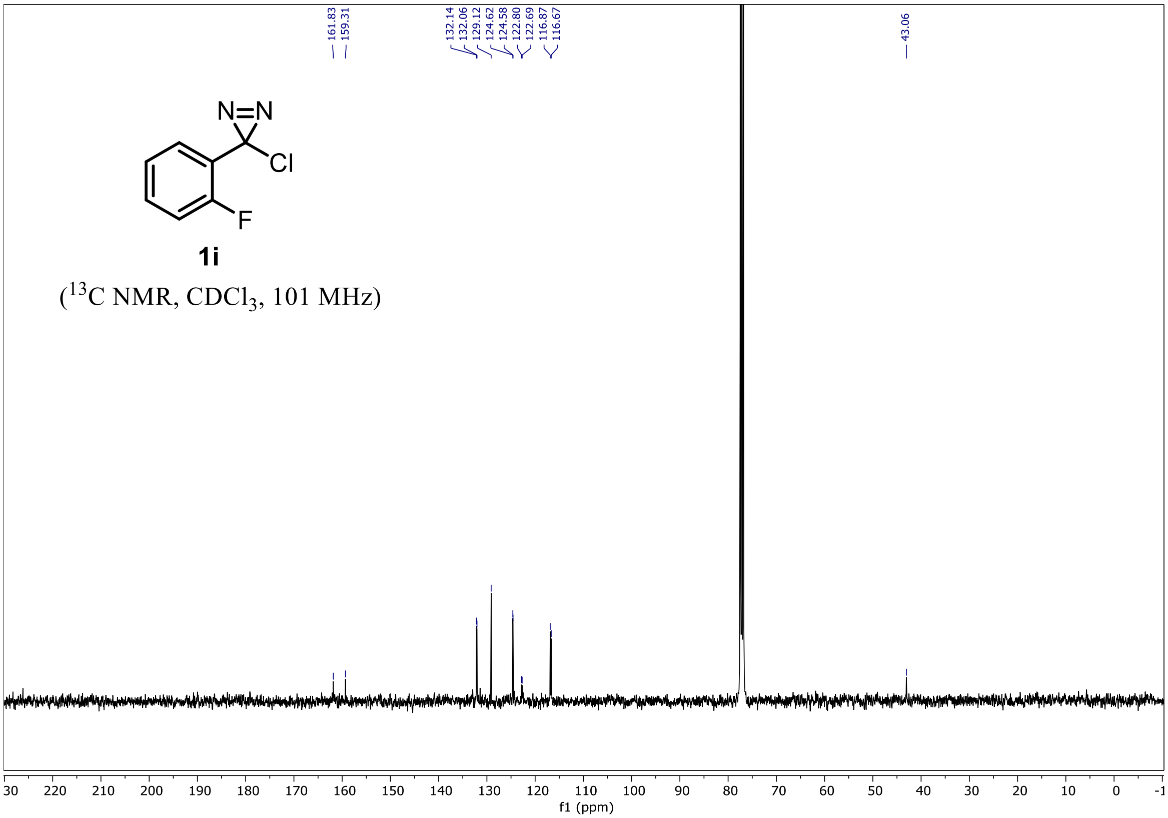
161.83
159.31
132.14 132.06 129.12 124.62 124.58 122.80 122.69 116.87 116.67
43.06
N=N
Cl
F
1i
(13C NMR, CDCl3, 101 MHz)
f1 (ppm)

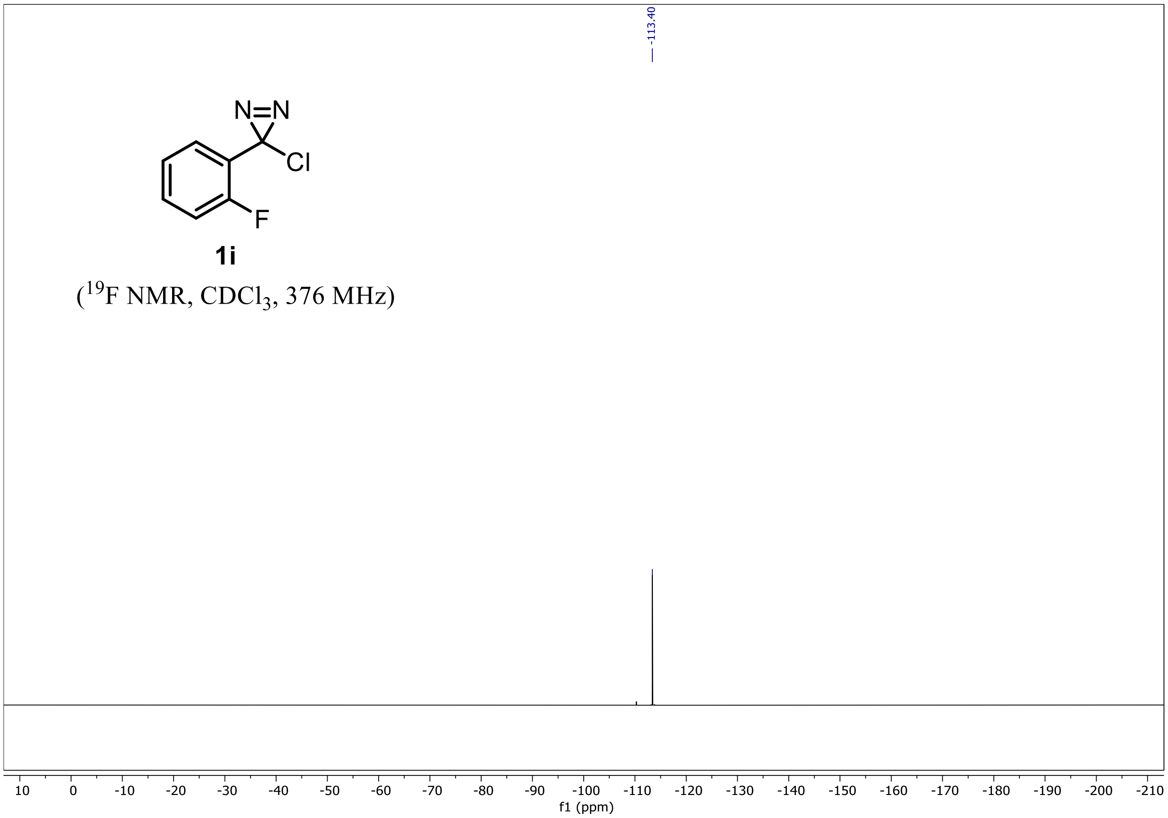


**1i**

($^{19}$F NMR, $CDCl_3$, 376 MHz)

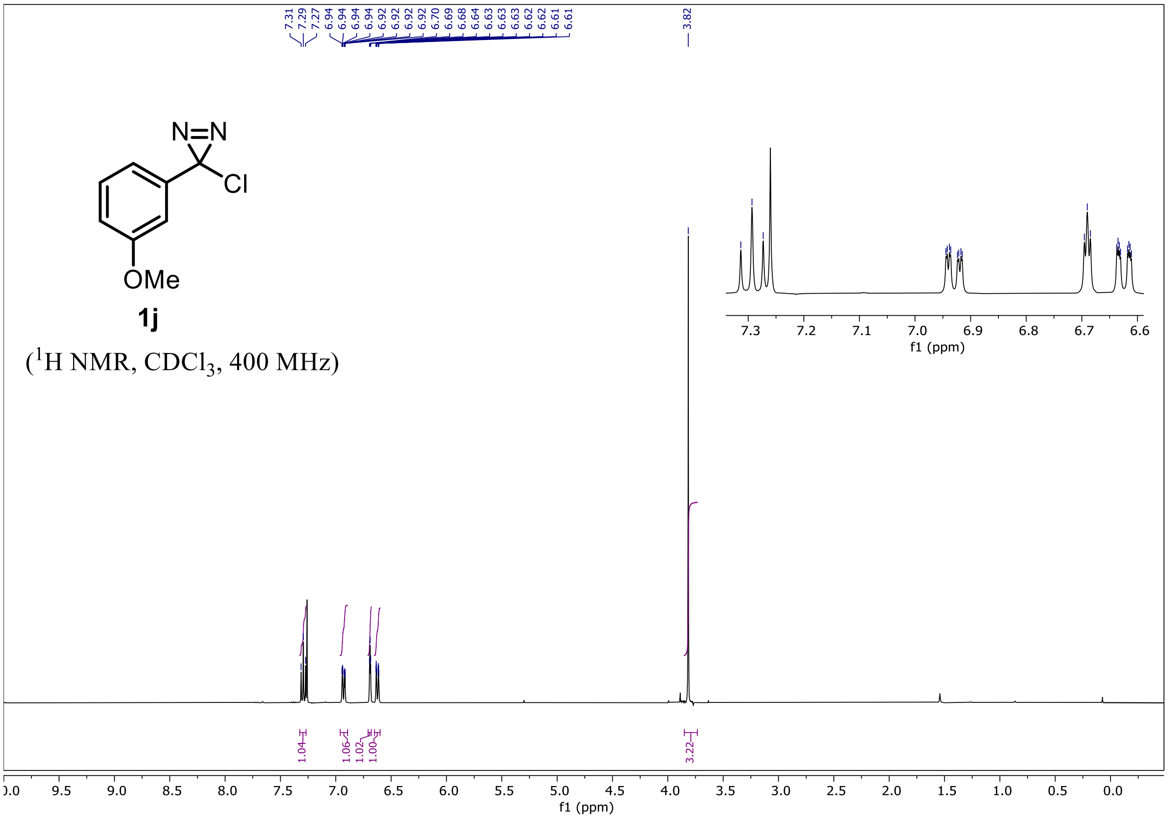


**1j**

($^{1}$H NMR, $CDCl_3$, 400 MHz)

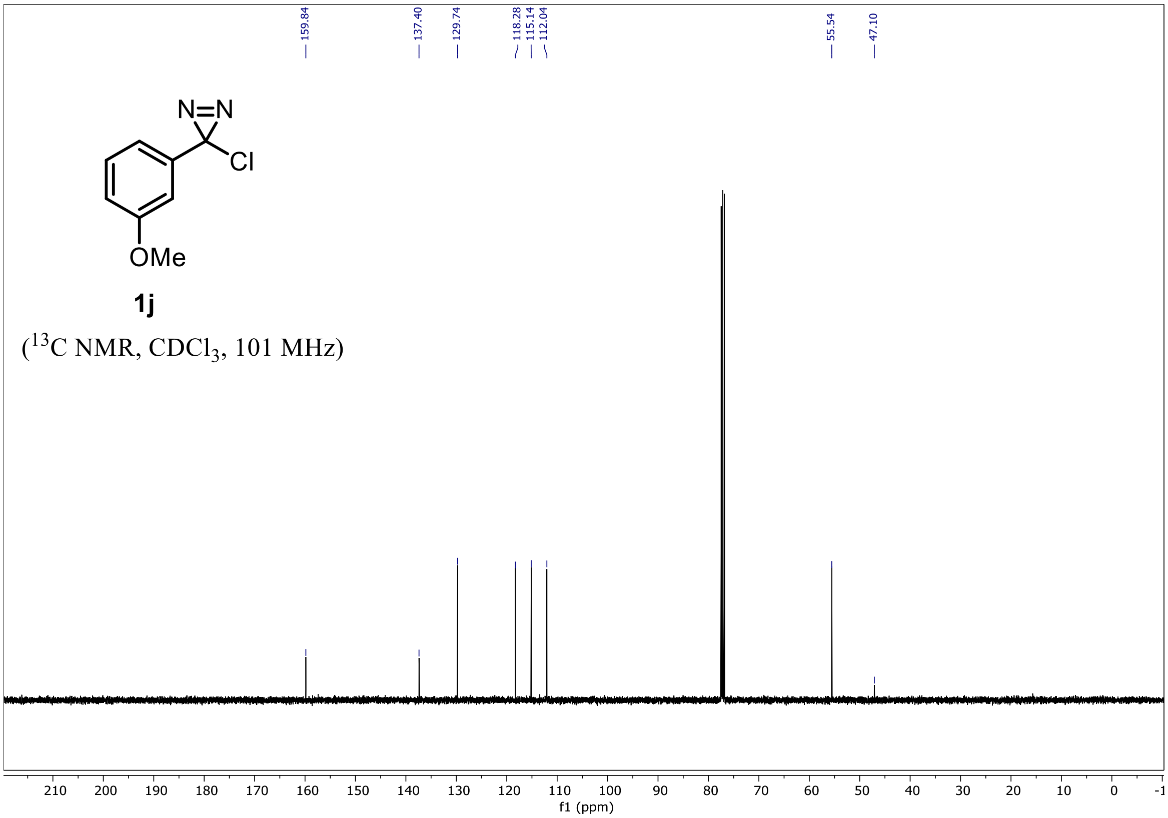
159.84
137.40
129.74
118.28
115.14
112.04
55.54
47.10
N=N
Cl
OMe
1j
(13C NMR, CDCl3, 101 MHz)
210 200 190 180 170 160 150 140 130 120 110 100 90 80 70 60 50 40 30 20 10 0
f1 (ppm)

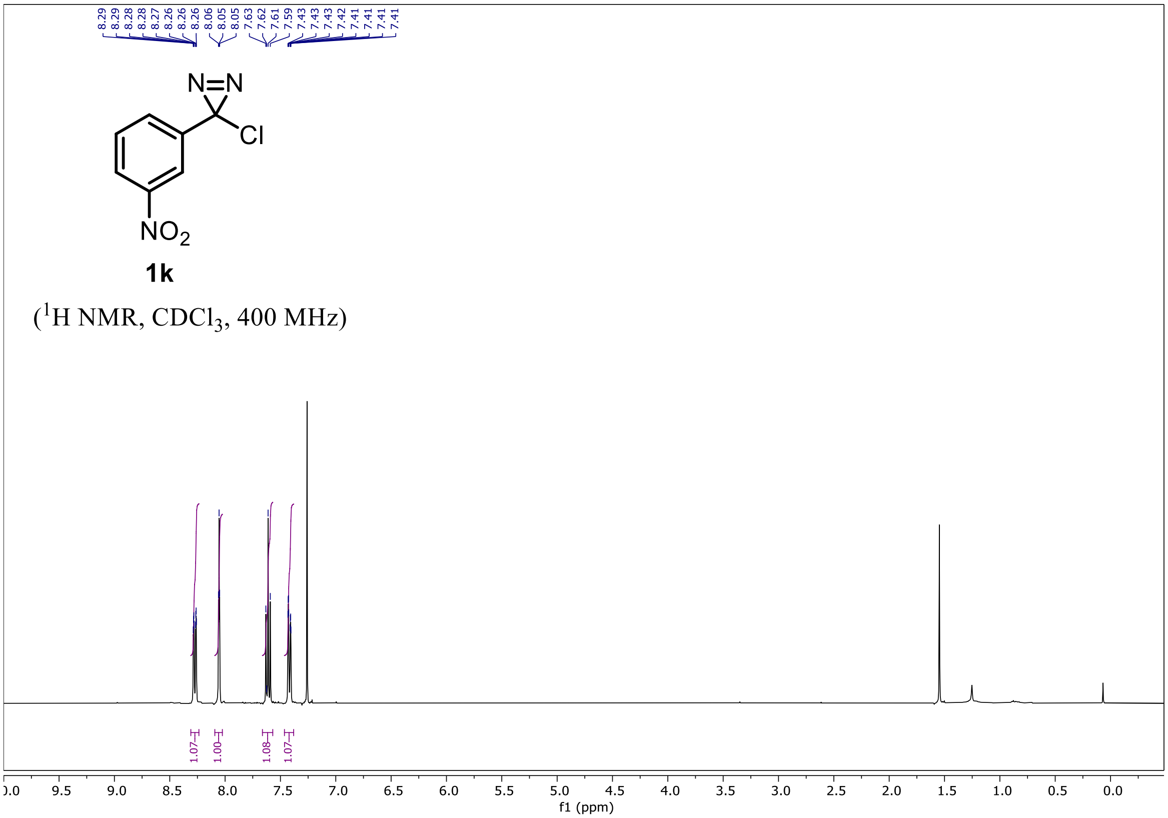
8.29
8.29
8.28
8.28
8.27
8.26
8.26
8.26
8.06
8.05
8.05
7.63
7.62
7.61
7.59
7.43
7.43
7.43
7.42
7.41
7.41
7.41
7.41
N=N
Cl
NO2
1k
(1H NMR, CDCl3, 400 MHz)
1.07
1.00
1.08
1.07
9.5 9.0 8.5 8.0 7.5 7.0 6.5 6.0 5.5 5.0 4.5 4.0 3.5 3.0 2.5 2.0 1.5 1.0 0.5 0.0
f1 (ppm)

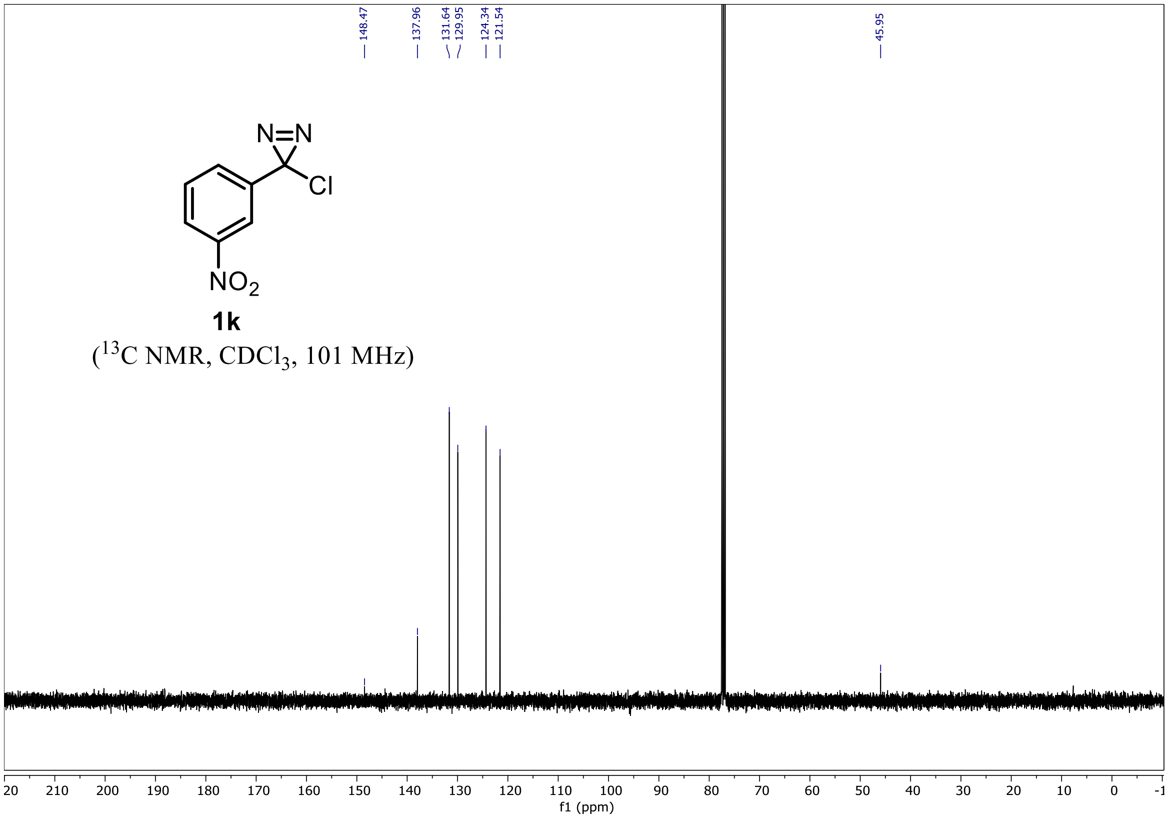
148.47
137.96
131.64
129.95
124.34
121.54
45.95
N=N
Cl
NO2
1k
(13C NMR, CDCl3, 101 MHz)
f1 (ppm)

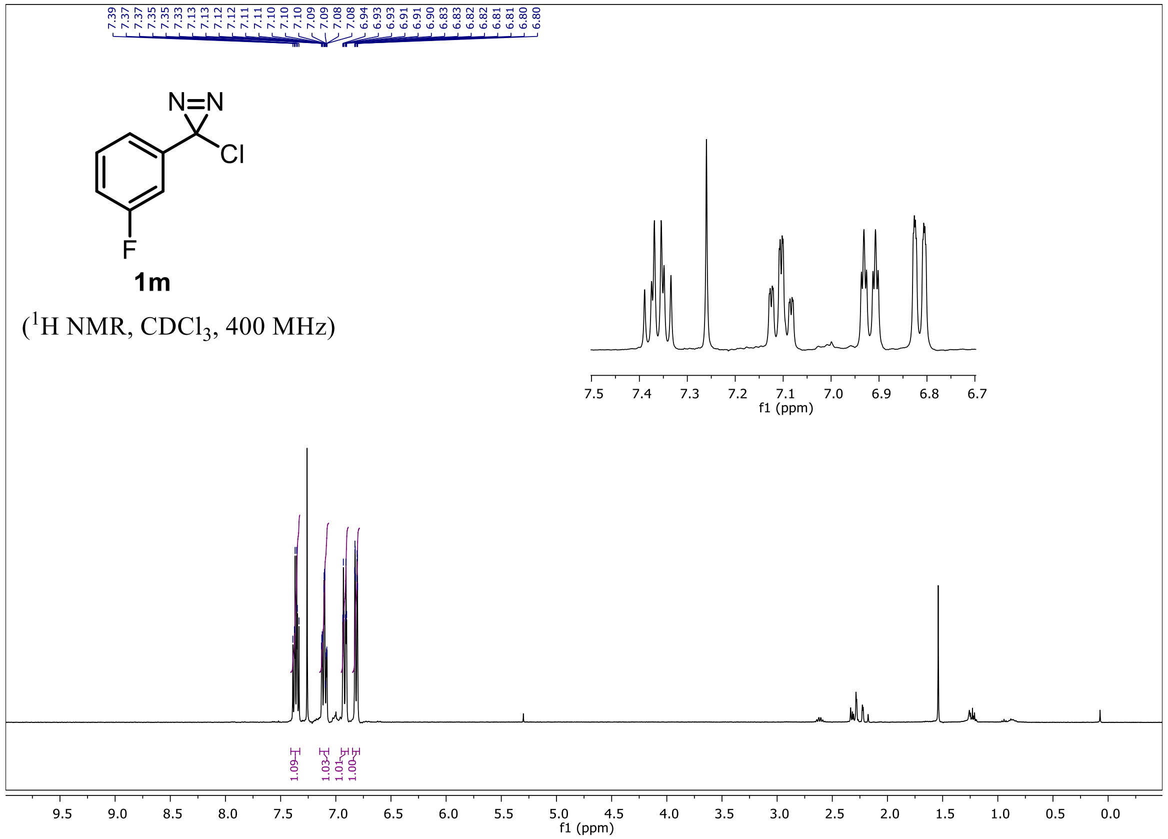
7.39
7.37
7.37
7.35
7.35
7.33
7.13
7.13
7.12
7.12
7.11
7.11
7.10
7.10
7.09
7.09
7.08
7.08
6.94
6.93
6.93
6.91
6.91
6.90
6.83
6.83
6.82
6.82
6.81
6.81
6.80
6.80
N=N
Cl
F
1m
(1H NMR, CDCl3, 400 MHz)
1.09
1.03
1.01
1.00
f1 (ppm)

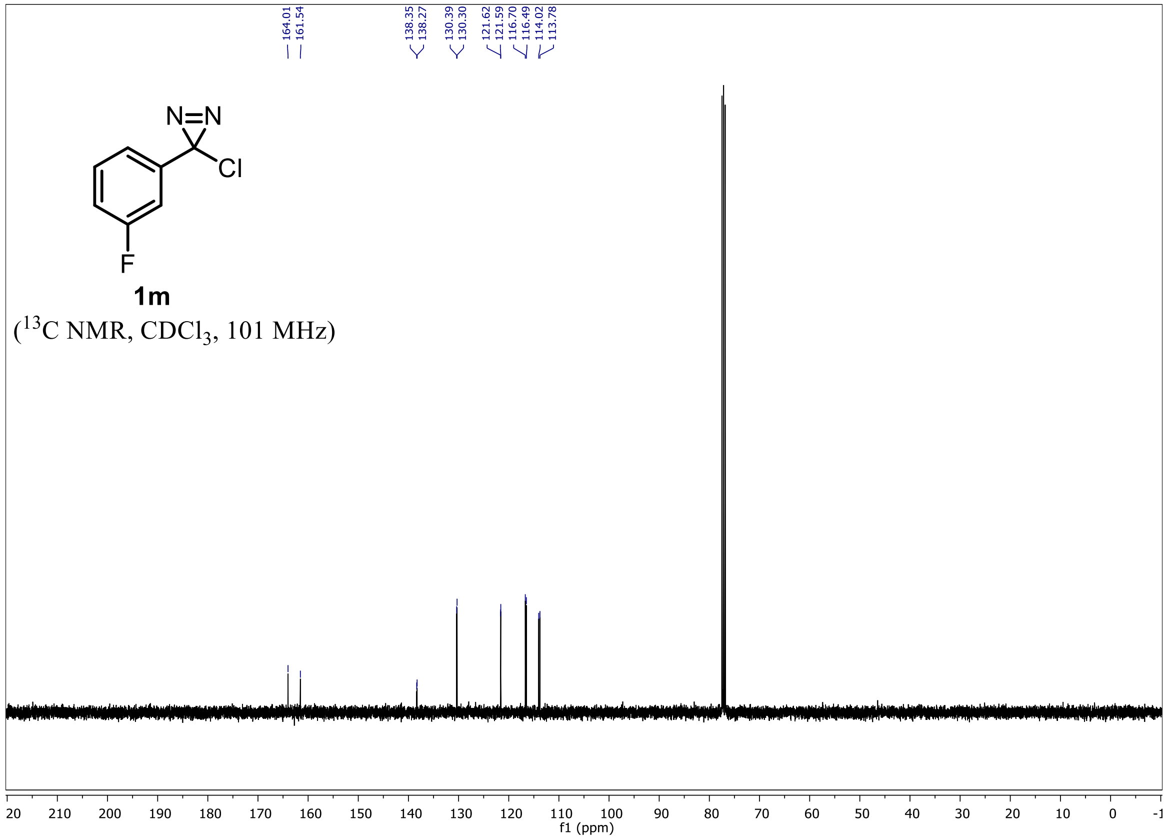
164.01
161.54
138.35
138.27
130.39
130.30
121.62
121.59
116.70
116.49
114.02
113.78
N=N
Cl
F
1m
($^{13}$C NMR, $CDCl_3$, 101 MHz)
f1 (ppm)

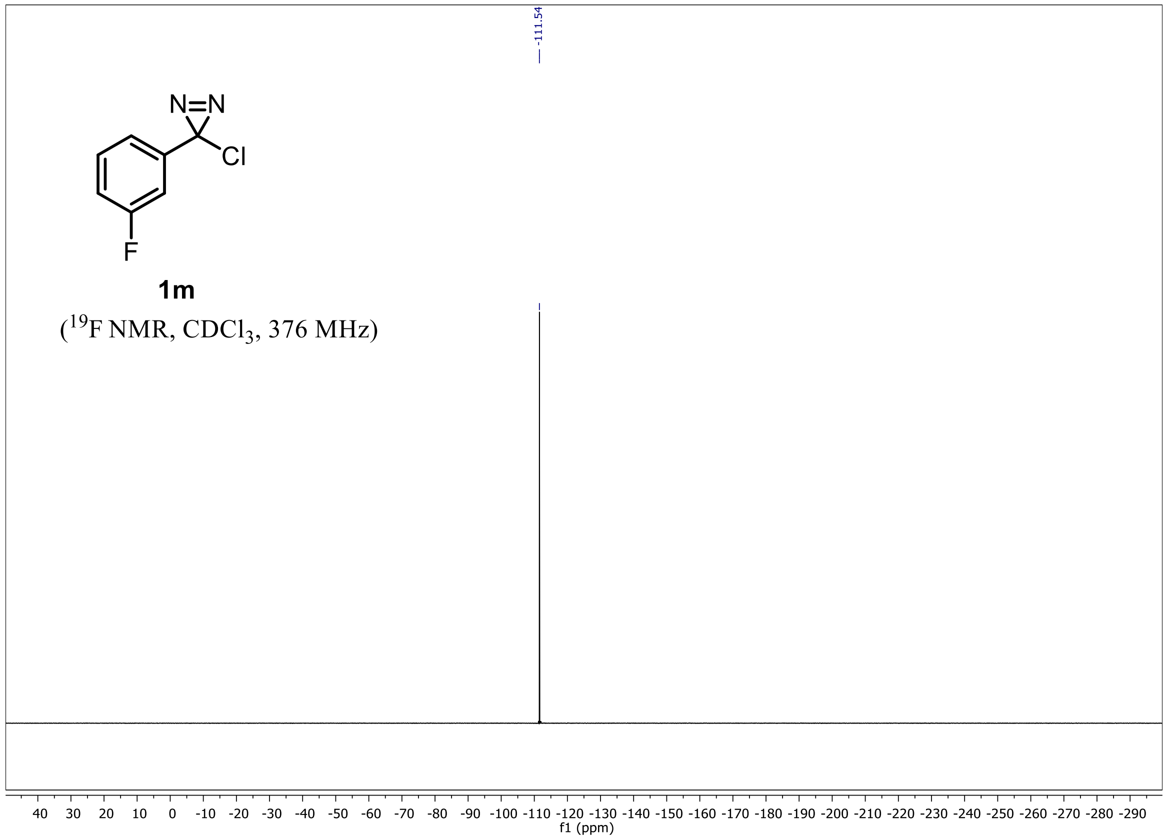
-111.54
N=N
Cl
F
1m
($^{19}$F NMR, $CDCl_3$, 376 MHz)
f1 (ppm)

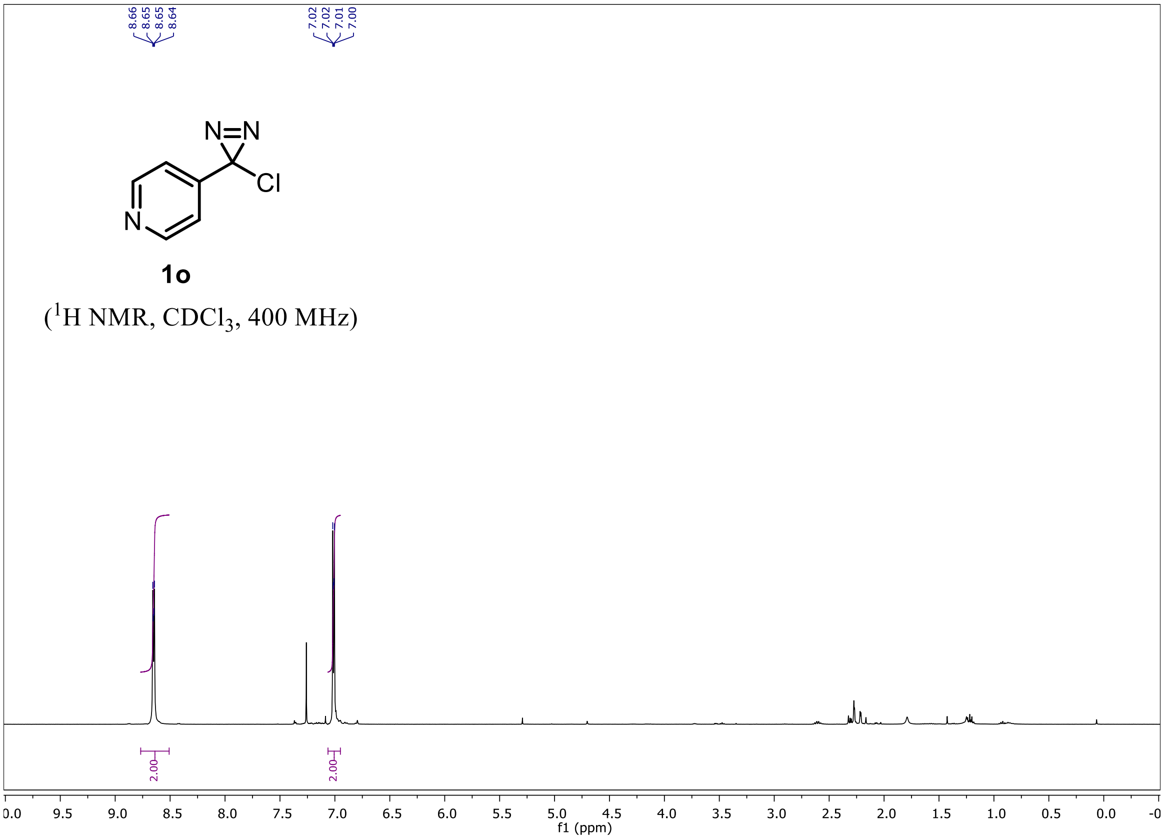
8.66
8.65
8.65
8.64
7.02
7.02
7.01
7.00
N=N
Cl
N
1o
($^1$H NMR, $CDCl_3$, 400 MHz)
2.00
2.00
f1 (ppm)

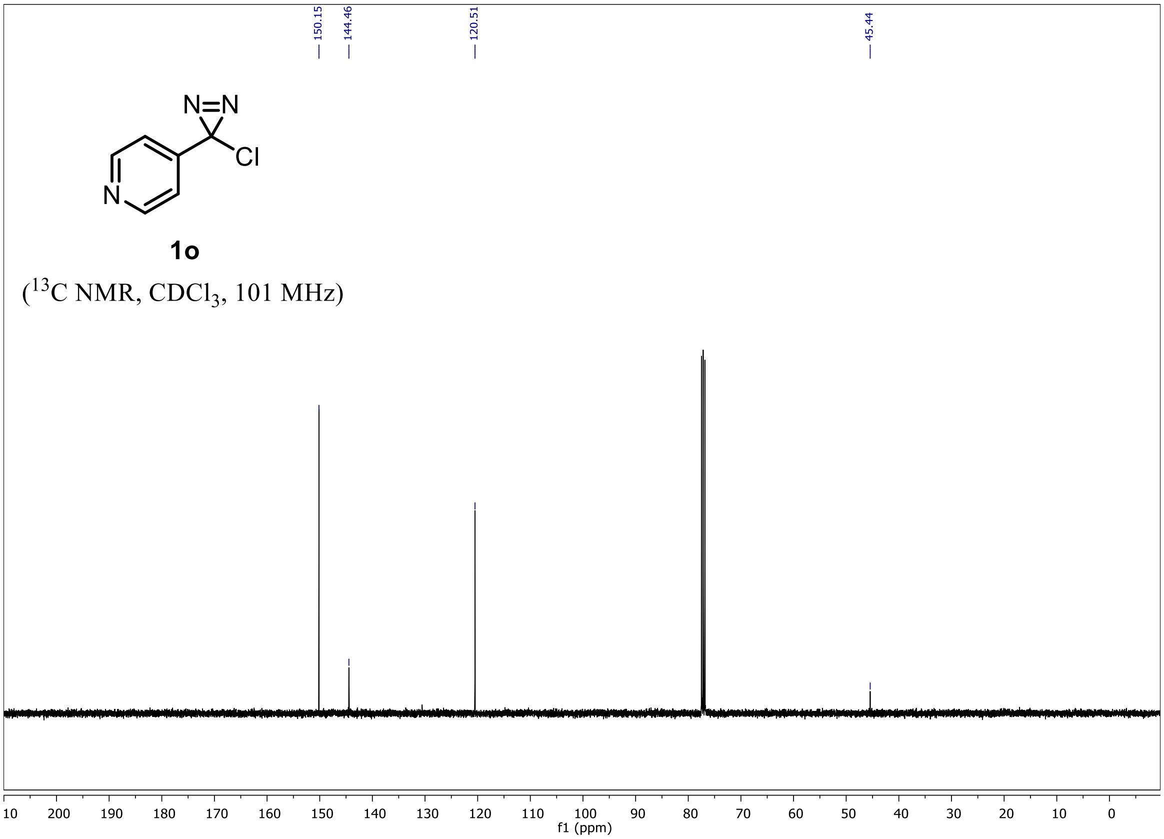
150.15
144.46
120.51
45.44
N=N
Cl
N
1o
($^{13}$C NMR, $CDCl_3$, 101 MHz)
f1 (ppm)

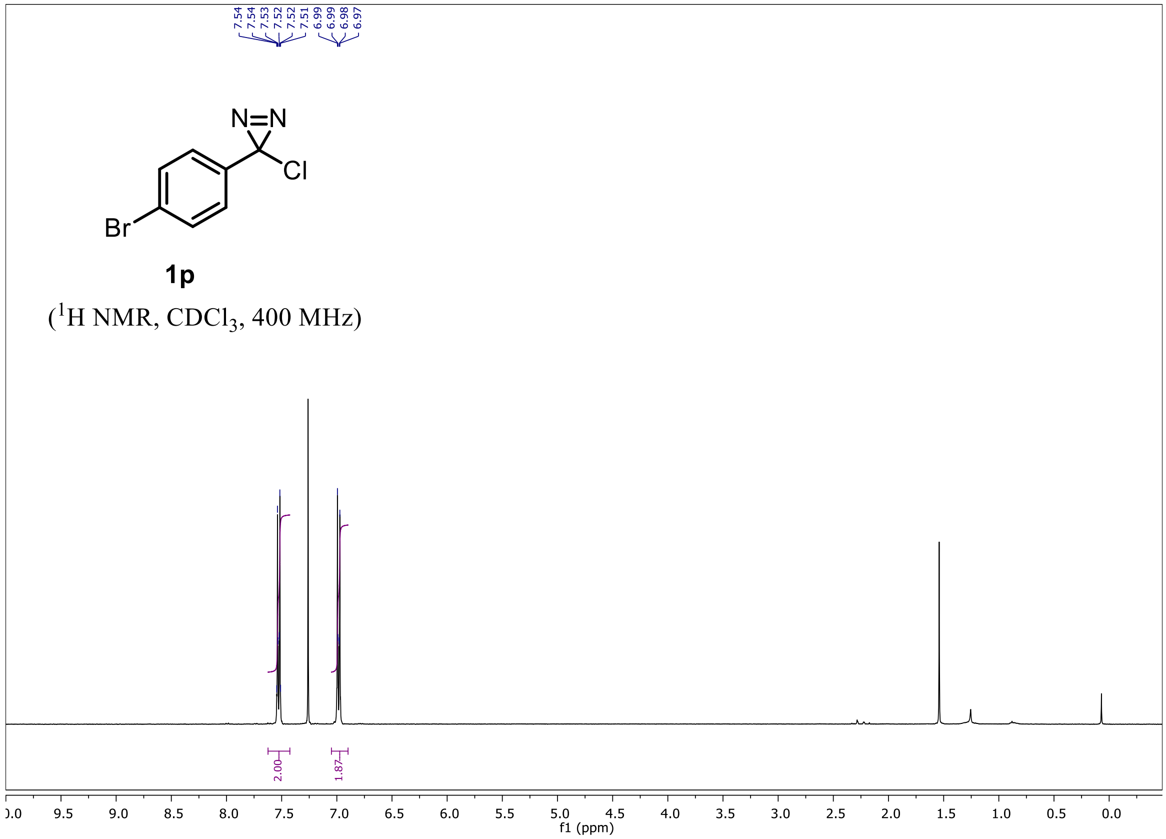

7.54
7.54
7.53
7.52
7.52
7.51
6.99
6.99
6.98
6.97
N=N
Cl
Br
1p
(1H NMR, CDCl3, 400 MHz)
2.00
1.87
f1 (ppm)


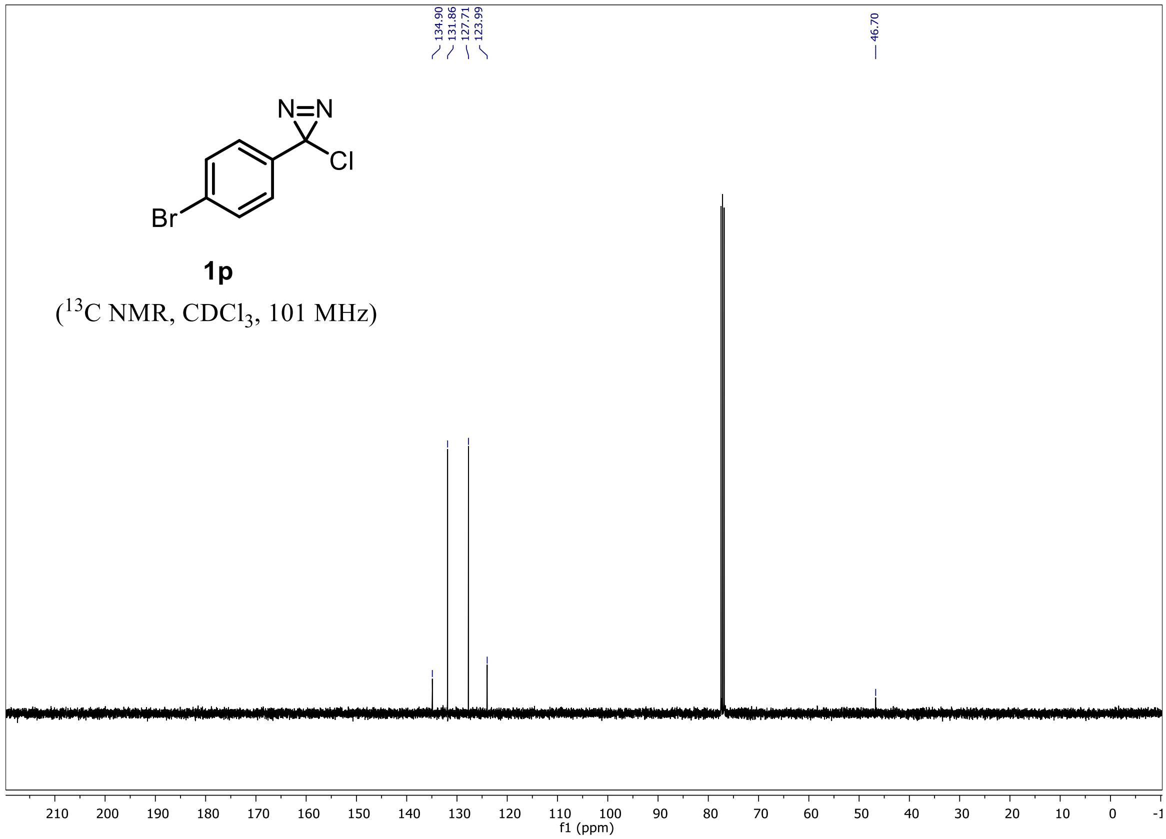

134.90
131.86
127.71
123.99
46.70
N=N
Cl
Br
1p
(13C NMR, CDCl3, 101 MHz)
f1 (ppm)

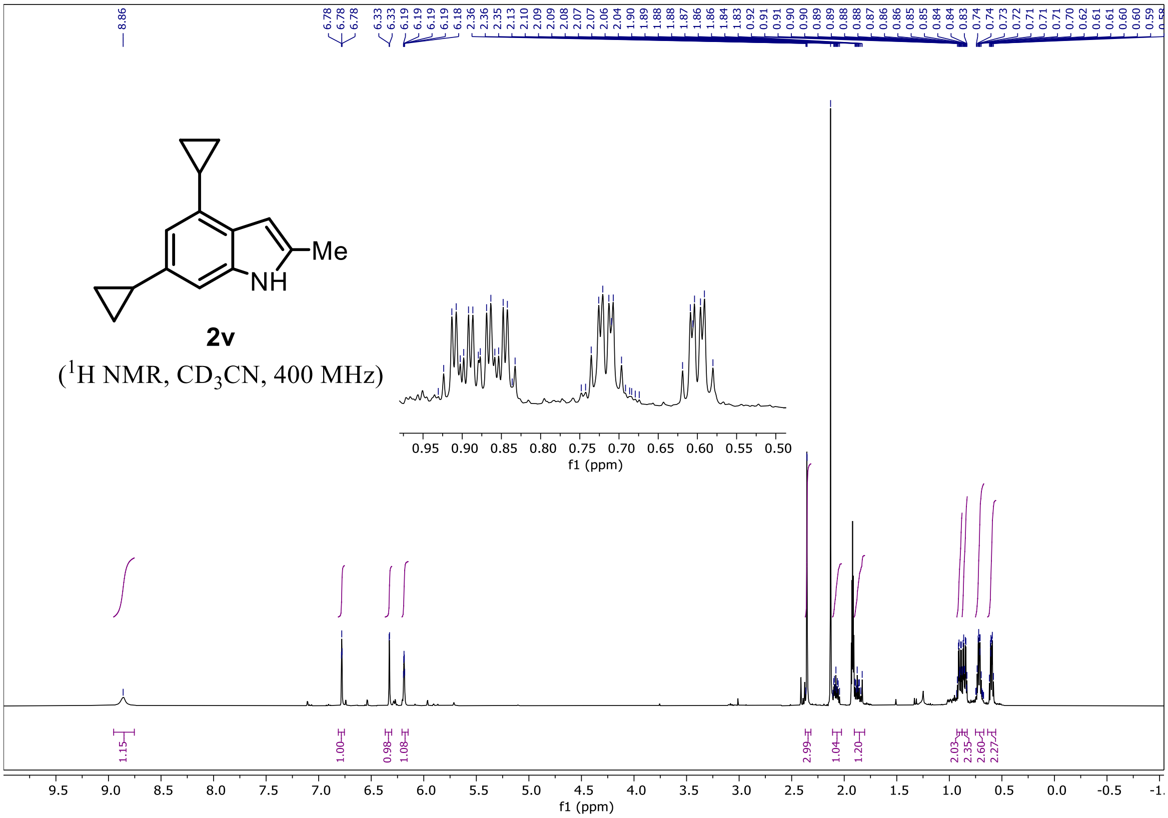

**2v**

($^{1}$H NMR, $CD_3CN$, 400 MHz)

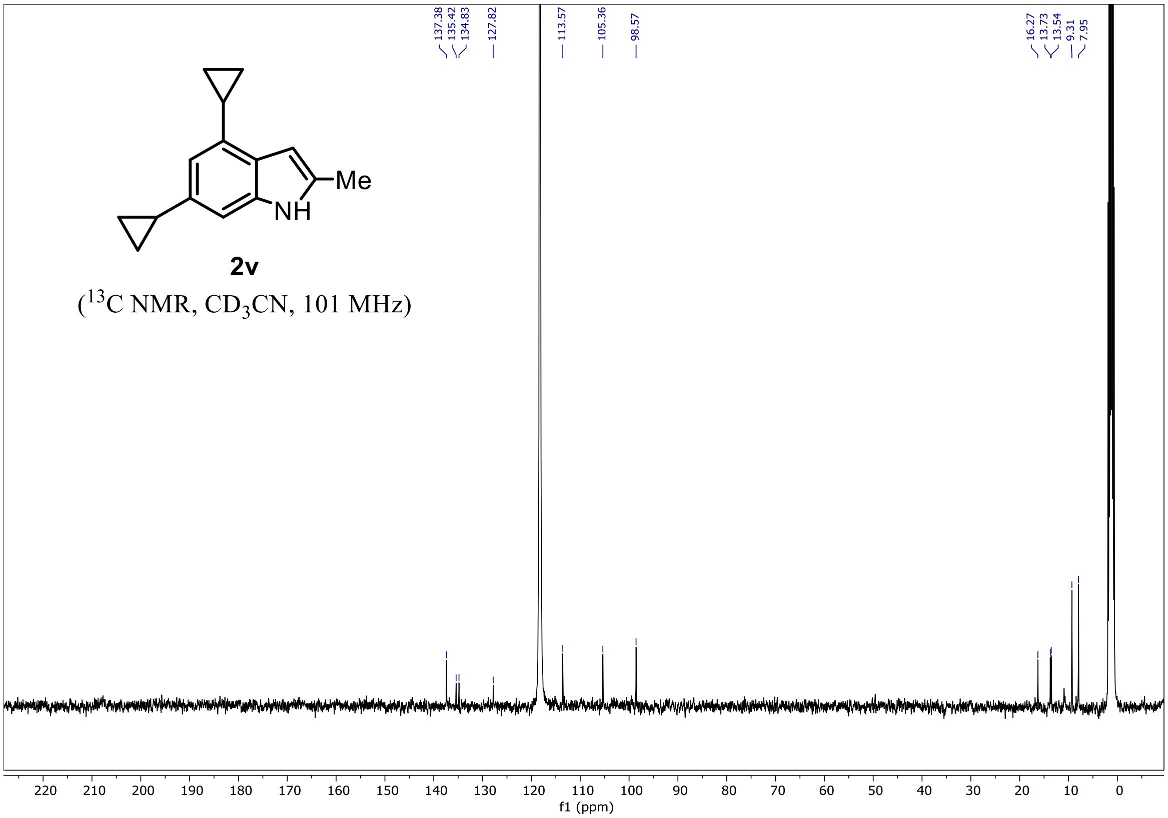

**2v**

($^{13}$C NMR, $CD_3CN$, 101 MHz)

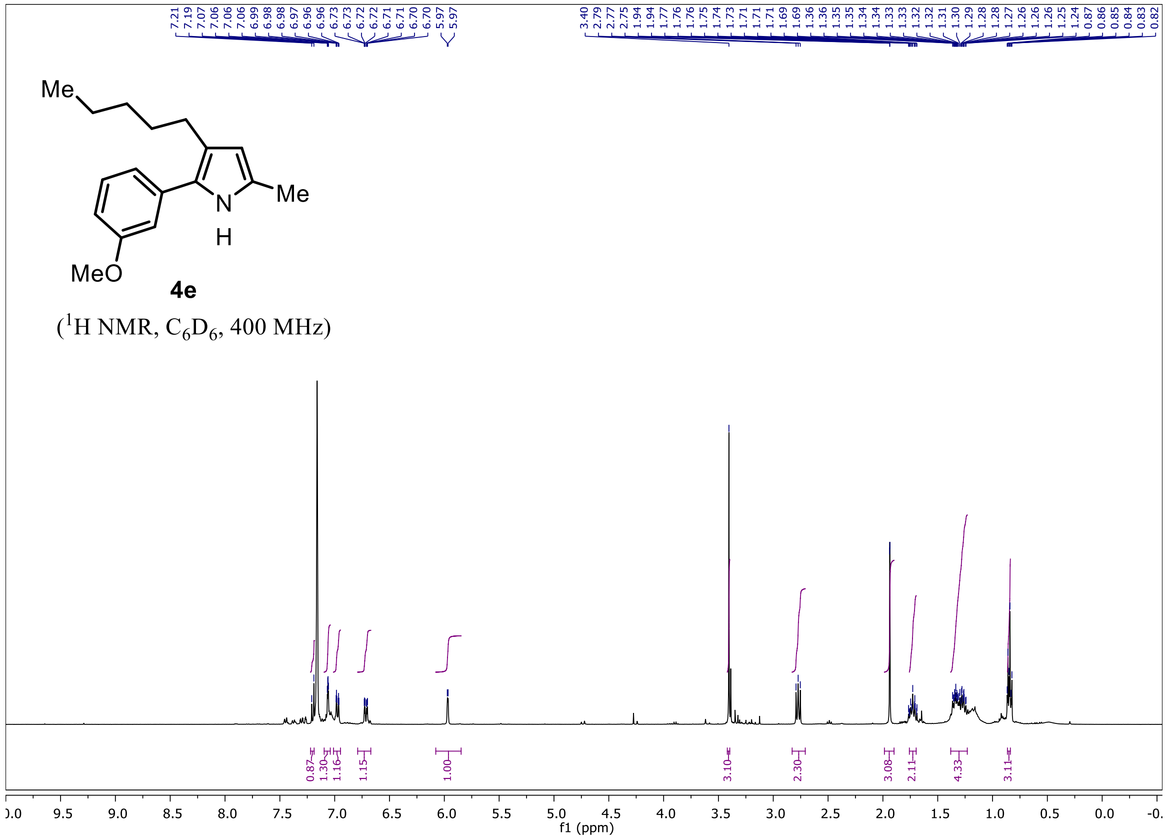

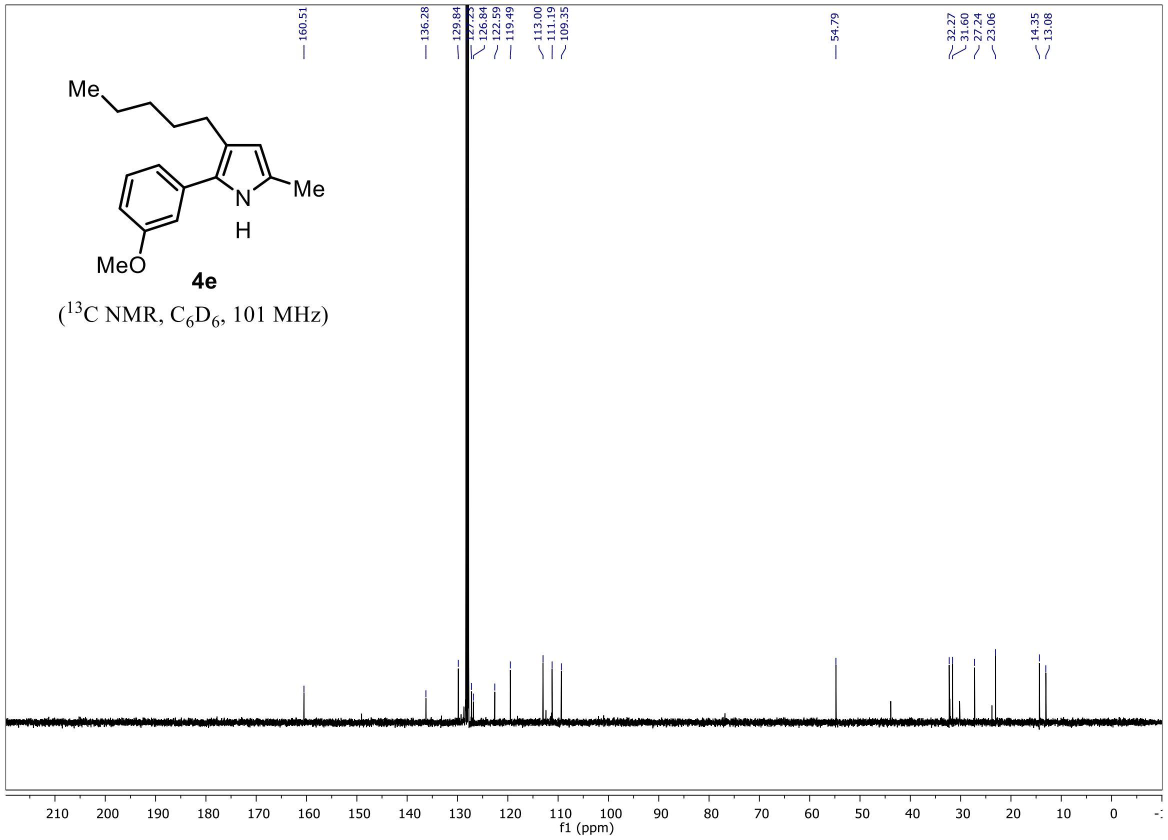

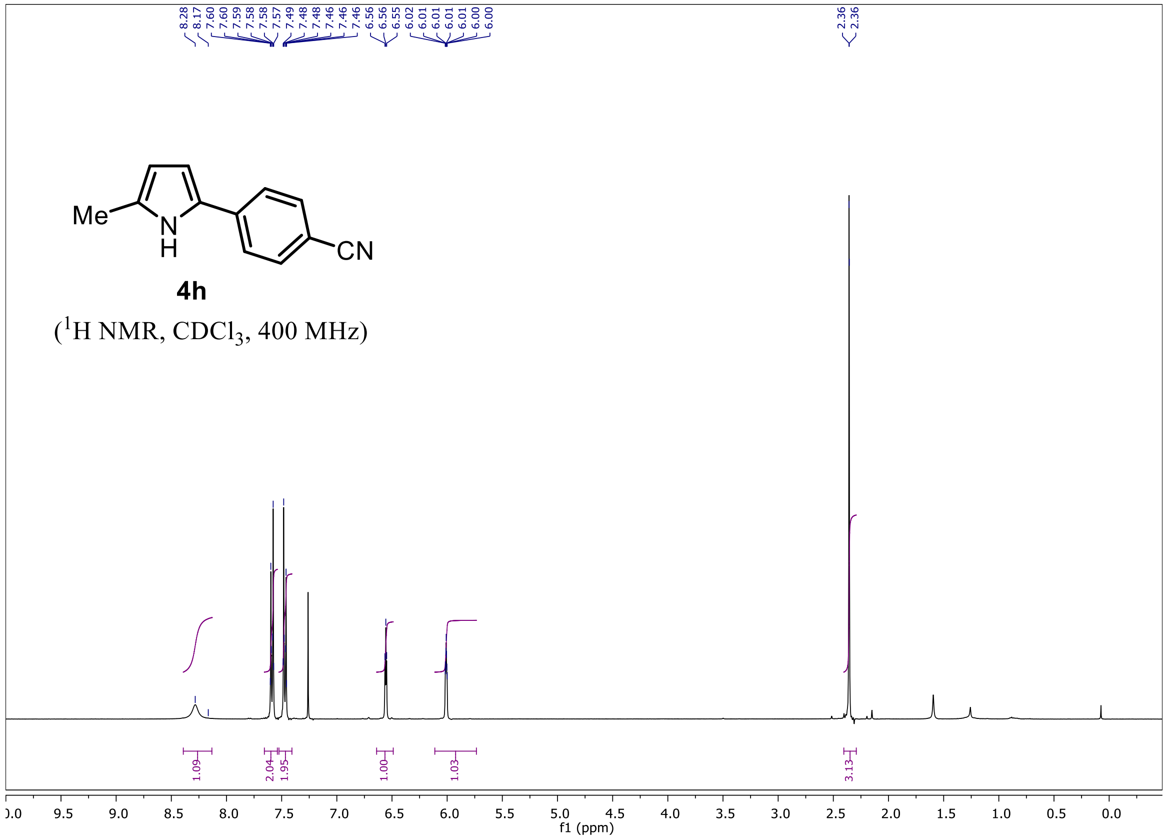
8.28
8.17
7.60
7.60
7.59
7.58
7.58
7.57
7.49
7.48
7.48
7.46
7.46
7.46
6.56
6.56
6.55
6.02
6.01
6.01
6.01
6.01
6.00
6.00
2.36
2.36
Me
N
H
CN
4h
(1H NMR, CDCl3, 400 MHz)
1.09
2.04
1.95
1.00
1.03
3.13
f1 (ppm)

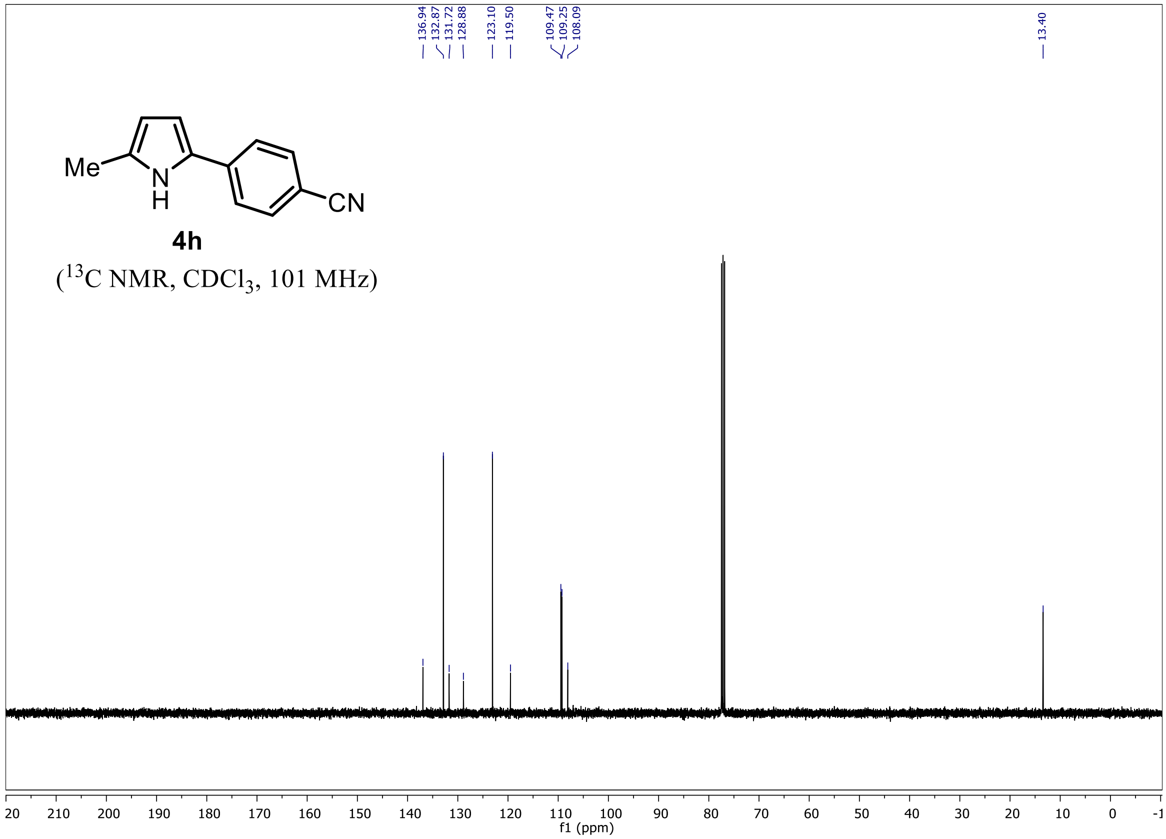
136.94
132.87
131.72
128.88
123.10
119.50
109.47
109.25
108.09
13.40
Me
N
H
CN
4h
(13C NMR, CDCl3, 101 MHz)
f1 (ppm)

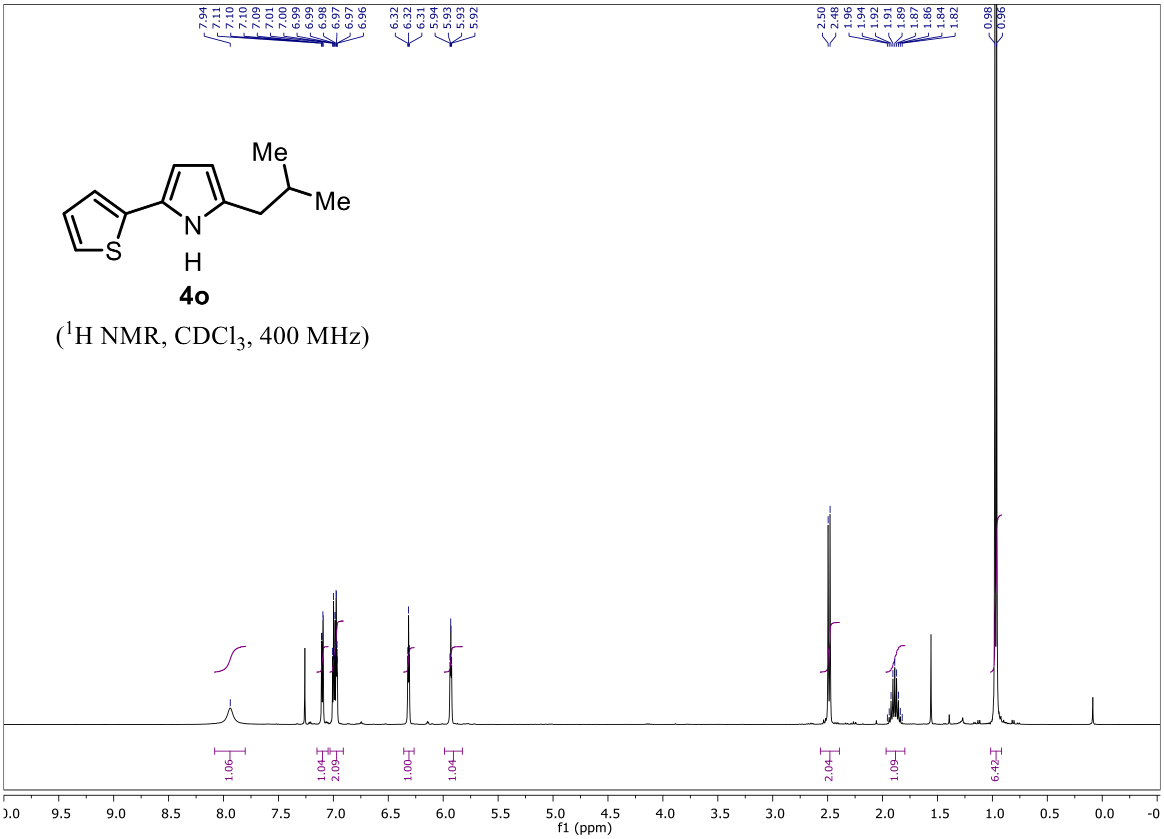

(¹H NMR, $CDCl_3$, 400 MHz)

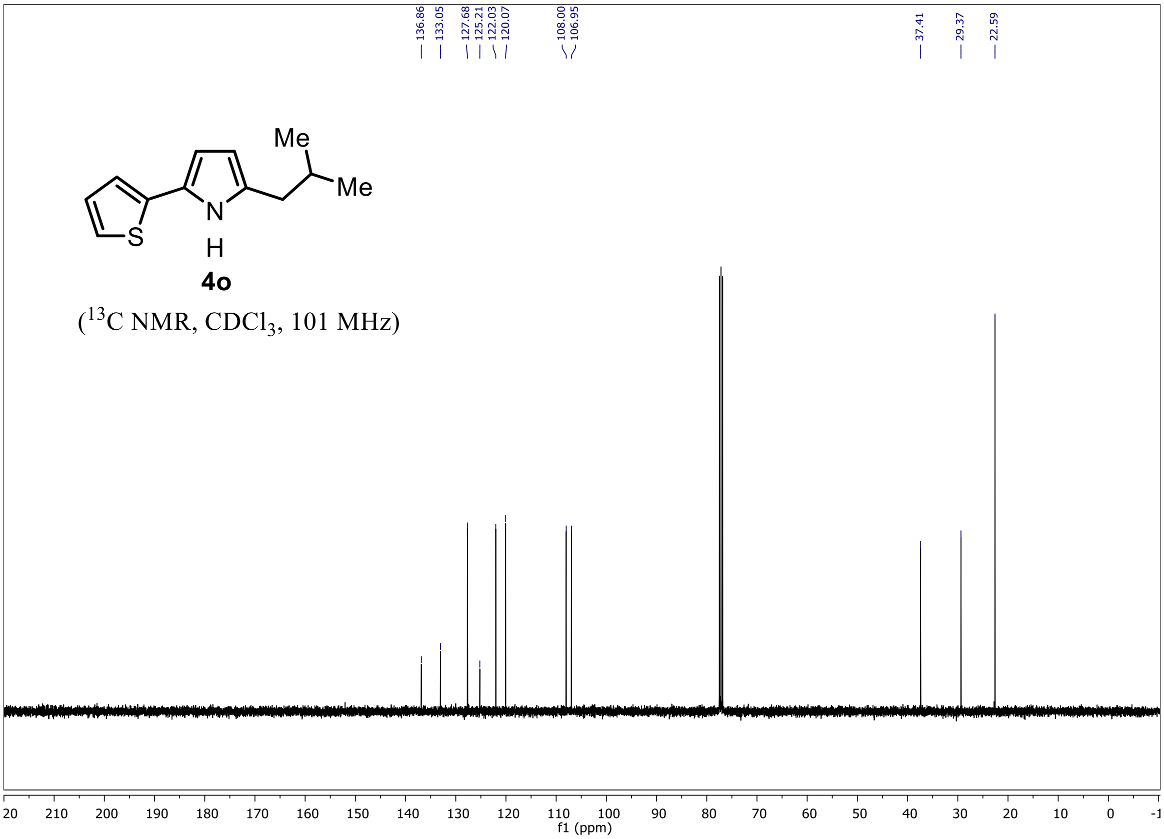

(¹³C NMR, $CDCl_3$, 101 MHz)

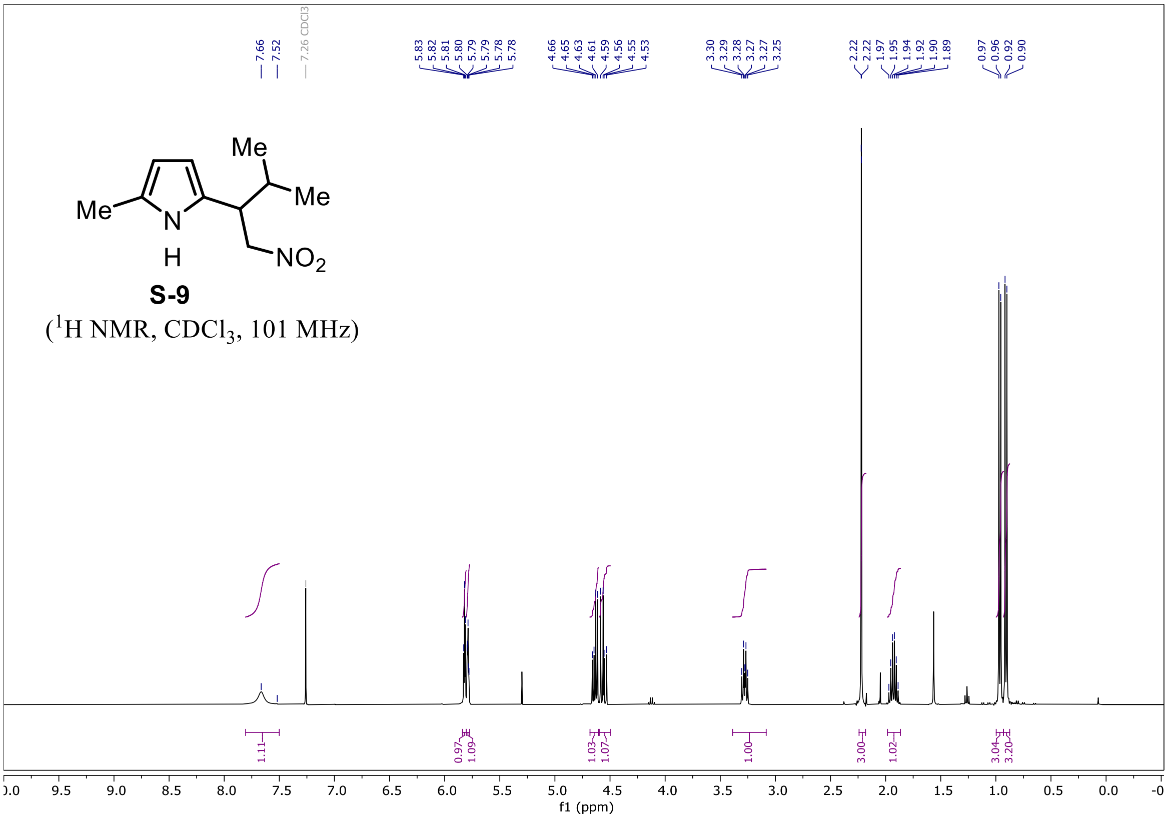
S-9
(1H NMR, CDCl3, 101 MHz)
f1 (ppm)

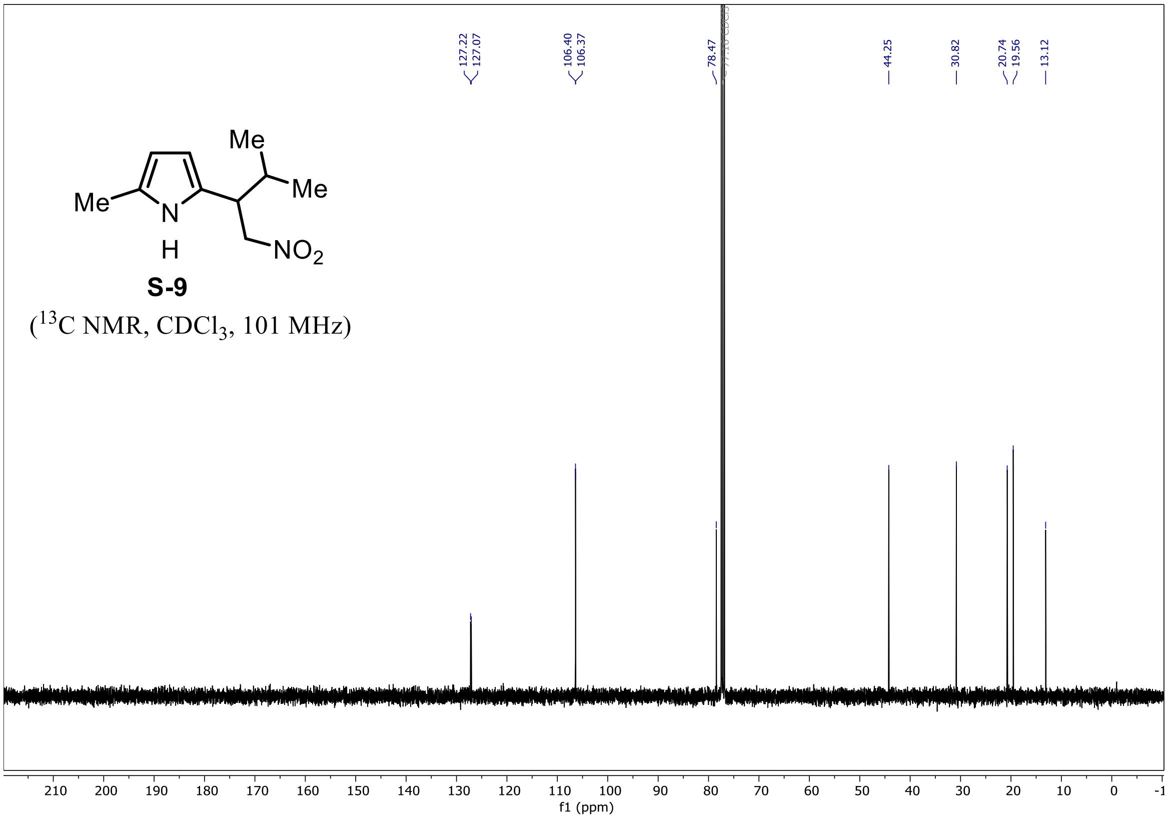
S-9
(13C NMR, CDCl3, 101 MHz)
f1 (ppm)

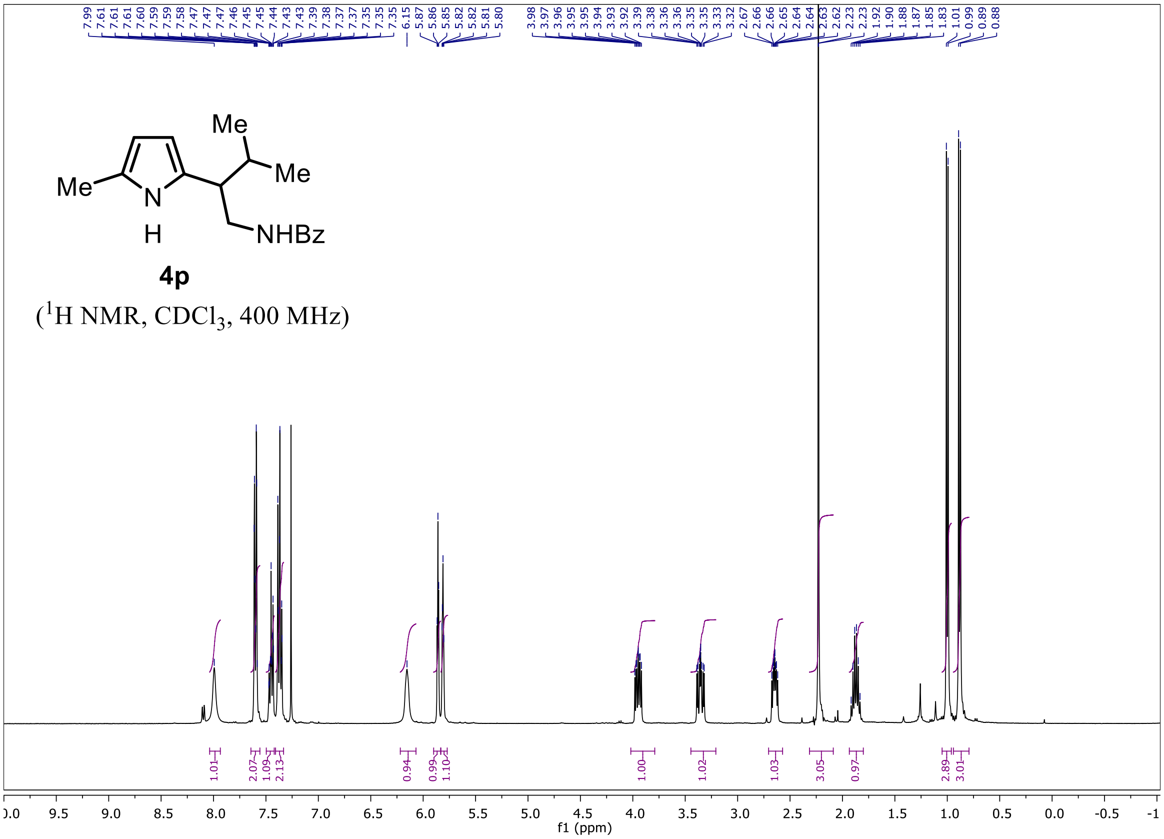
Me
Me
Me
N
H
NHBz
4p
(1H NMR, CDCl3, 400 MHz)
f1 (ppm)

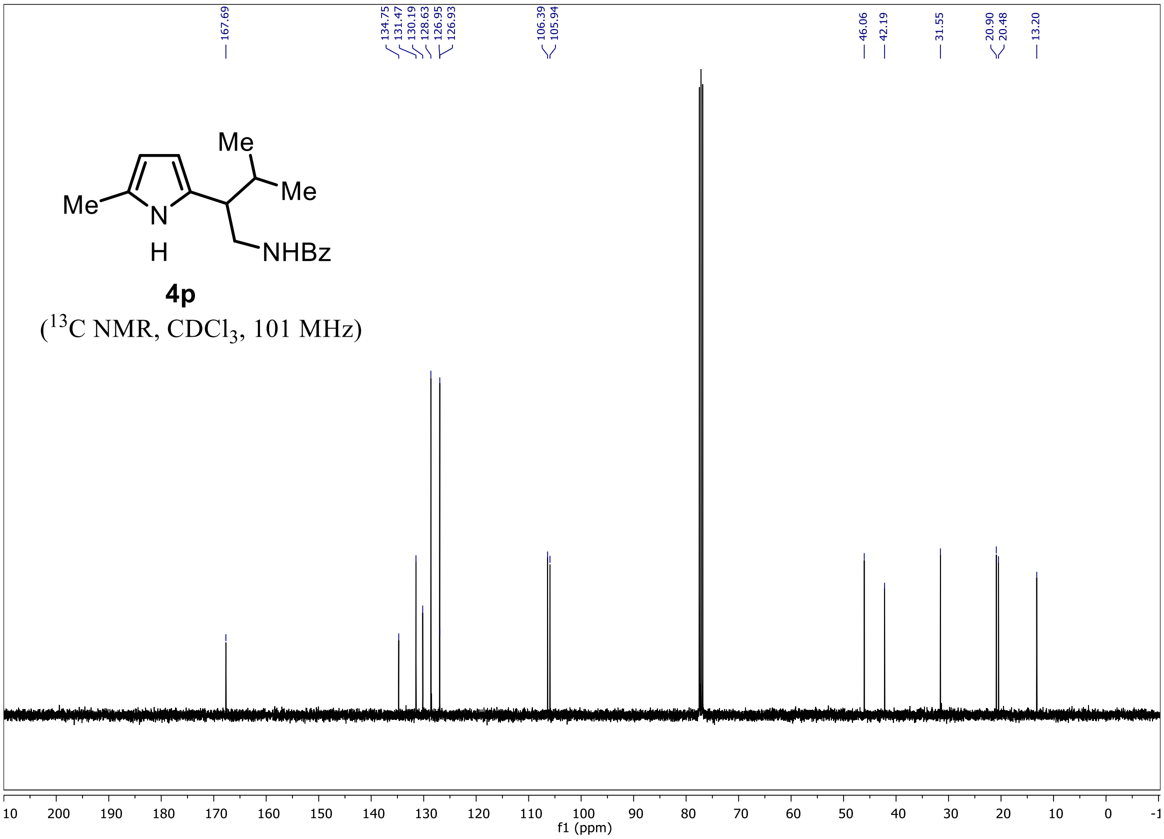
167.69
134.75
131.47
130.19
128.63
126.95
126.93
106.39
105.94
46.06
42.19
31.55
20.90
20.48
13.20
Me
Me
Me
N
H
NHBz
4p
(13C NMR, CDCl3, 101 MHz)
f1 (ppm)

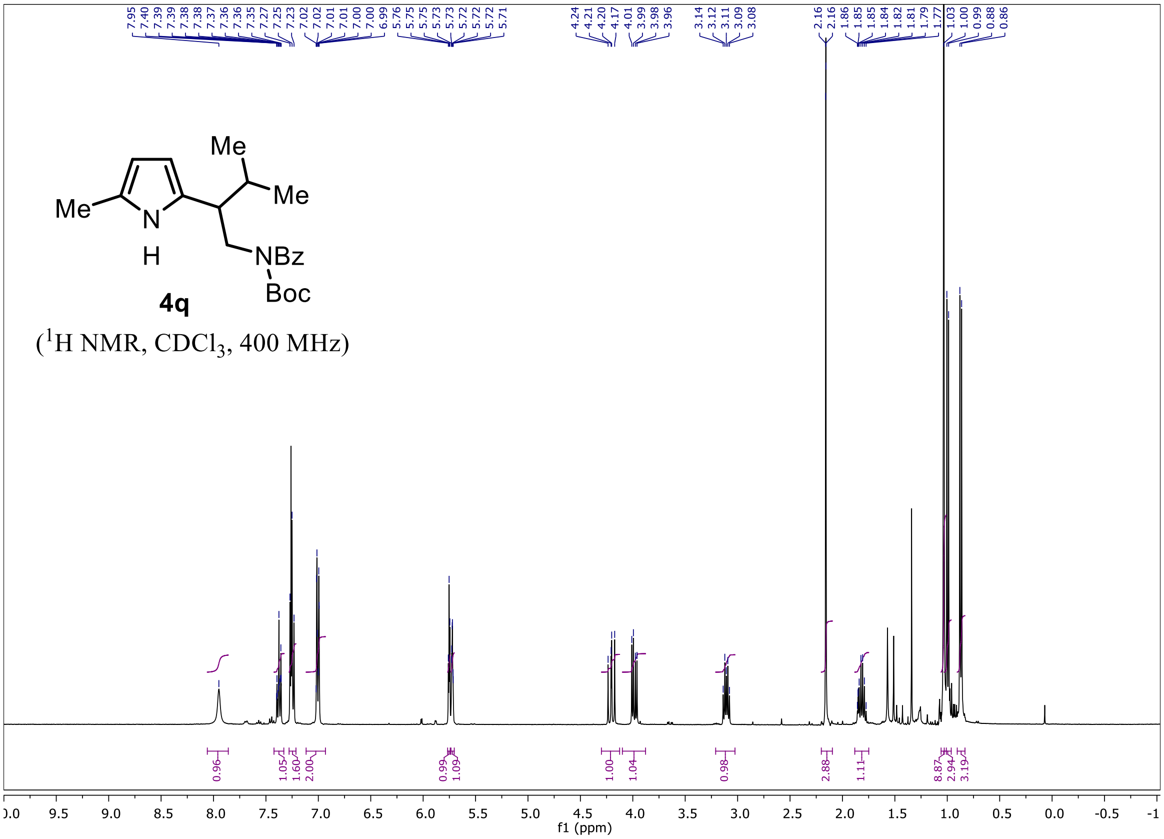
7.95 7.40 7.39 7.39 7.38 7.38 7.37 7.36 7.36 7.35 7.27 7.25 7.23 7.02 7.01 7.01 7.00 7.00 6.99 5.76 5.75 5.75 5.73 5.73 5.72 5.72 5.72 5.71
4.24 4.21 4.20 4.17 4.01 3.99 3.98 3.96 3.14 3.12 3.11 3.09 3.08
2.16 2.16 1.86 1.85 1.85 1.84 1.82 1.81 1.79 1.77 1.03 1.00 0.99 0.88 0.86
Me
Me
Me
N
H
NBz
Boc
4q
(1H NMR, CDCl3, 400 MHz)
0.96 1.05 1.60 2.00 0.99 1.09 1.00 1.04 0.98 2.88 1.11 8.87 2.94 3.19
f1 (ppm)

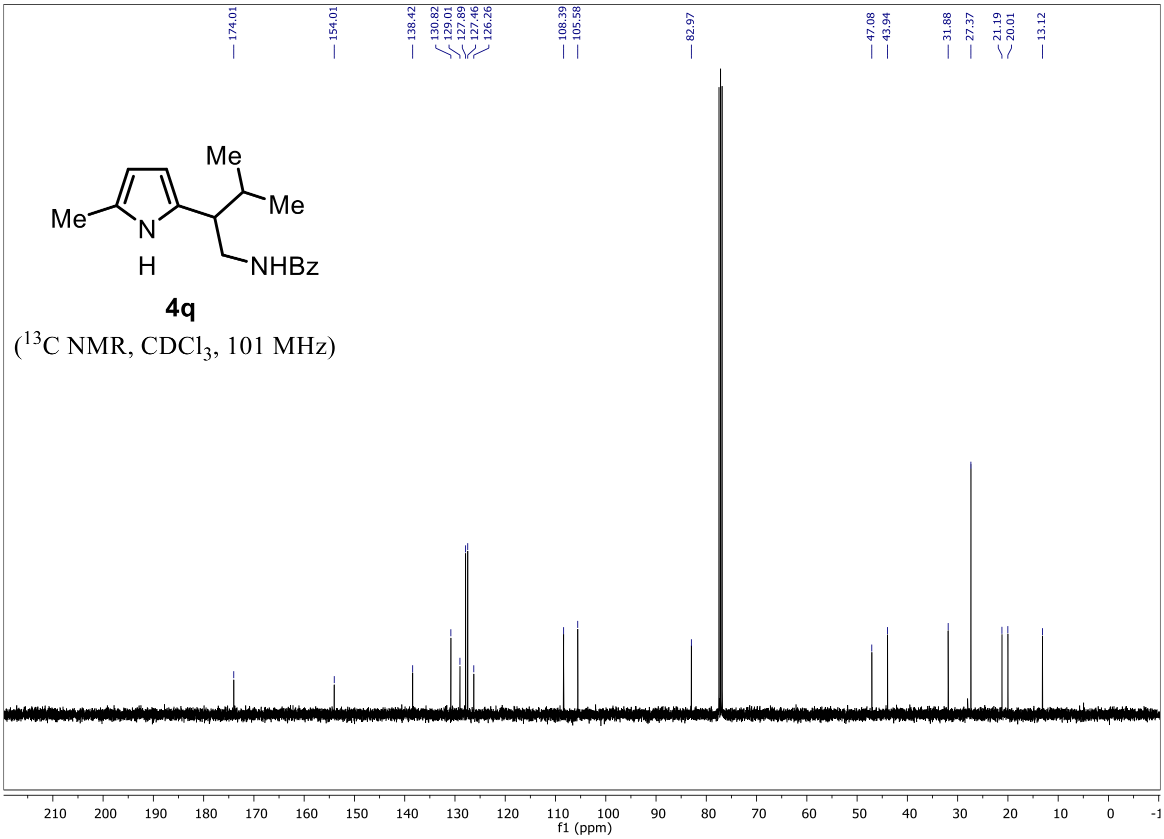
174.01
154.01
138.42 130.82 129.01 127.89 127.46 126.26
108.39 105.58
82.97
47.08 43.94
31.88 27.37 21.19 20.01 13.12
Me
Me
Me
N
H
NHBz
4q
(13C NMR, CDCl3, 101 MHz)
f1 (ppm)

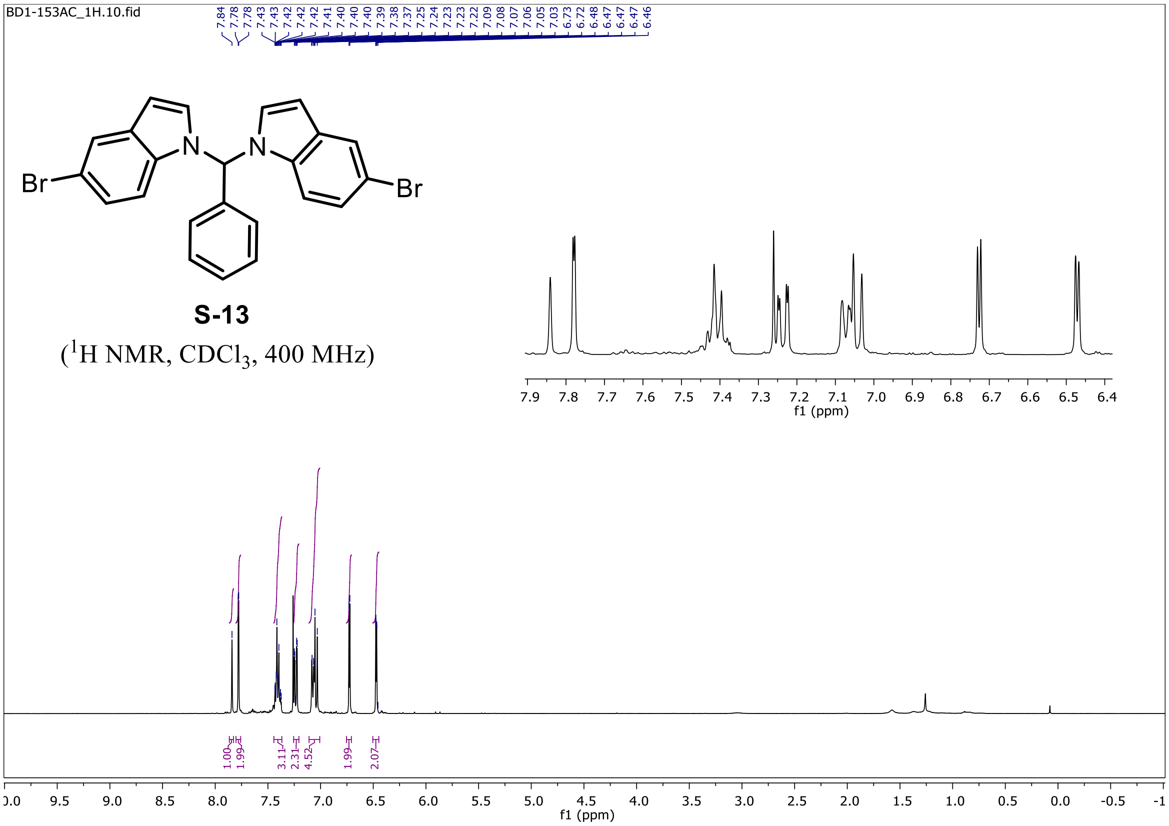
BD1-153AC_1H.10.fid
7.84 7.78 7.78 7.43 7.43 7.42 7.42 7.42 7.41 7.40 7.40 7.40 7.39 7.38 7.37 7.25 7.24 7.23 7.23 7.22 7.09 7.08 7.07 7.06 7.05 7.03 6.73 6.72 6.48 6.47 6.47 6.46
Br
N
N
Br
S-13
(1H NMR, CDCl3, 400 MHz)
1.00 1.99 3.11 2.31 4.52 1.99 2.07
f1 (ppm)

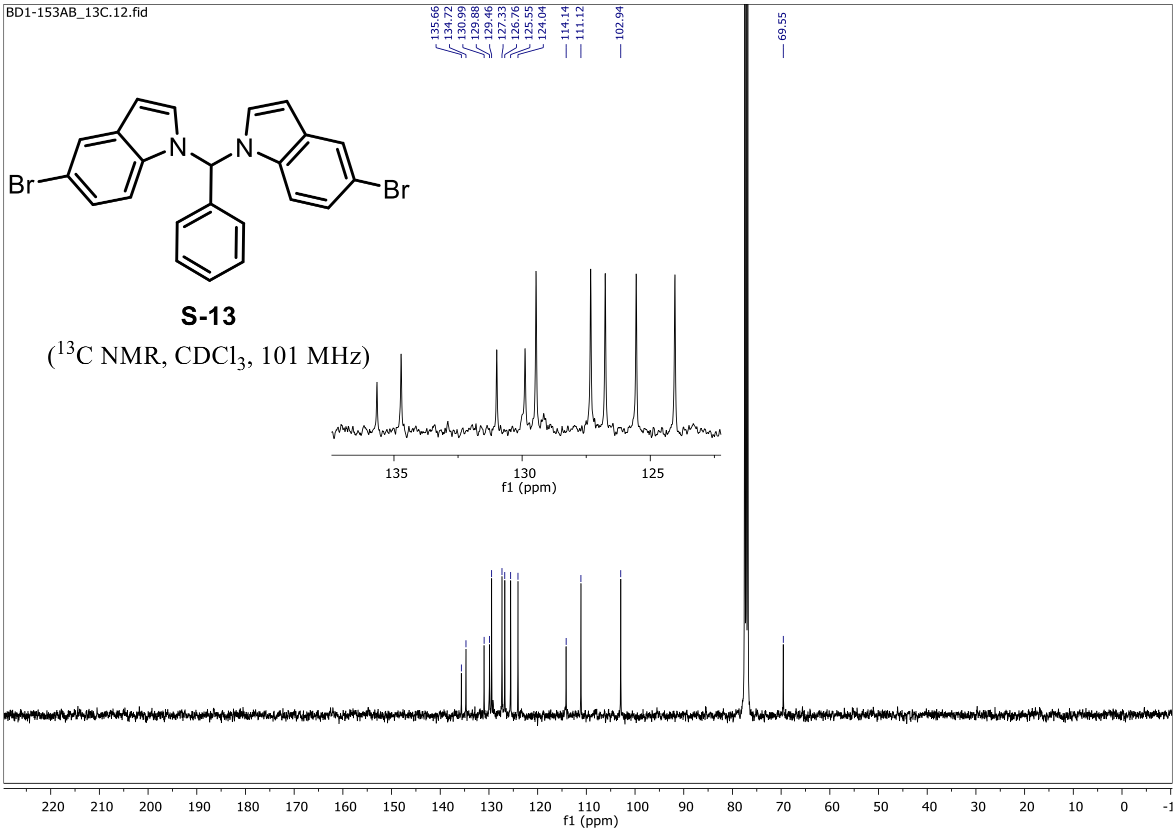
BD1-153AB_13C.12.fid
135.66 134.72 130.99 129.88 129.46 127.33 126.76 125.55 124.04 114.14 111.12 102.94 69.55
Br
N
N
Br
S-13
(13C NMR, CDCl3, 101 MHz)
f1 (ppm)
