# Supplementary material for: Carbon Atom Insertion into Pyrroles and Indoles Promoted by Chlorodiazirines
Source: J Am Chem Soc. 2021 Jul 21;143(30):11337–44. doi: 10.1021/jacs.1c06287 (PMC8343525; doi:10.1021/jacs.1c06287)
Supplement: Supplementary file 2 — ja1c06287_si_002.pdf [file ja1c06287_si_002.pdf]

Supporting Information 2

Carbon Atom Insertion into Pyrroles and Indoles Promoted by Chlorodiazirines

Computational Details

Balu D. Dherange[1], Patrick Q. Kelly[1], Jordan P. Liles[2], Matthew S. Sigman[2], and Mark D. Levin[1,*]

[1]Department of Chemistry, University of Chicago, Chicago, IL 60637, United States

[2] Department of Chemistry, University of Utah, Salt Lake City, Utah 84112, United States

Contents

1. Computational Methods....S2-2
2. Triplet energies and azabenzvalene pathway for 2,5-dimethylpyrrole (4a) S2-3
3. Energy pathway and predicted selectivity for 2-phenyl-5-methylpyrrole (4f)....S2-4
4. Plausible mechanism for para-insertion (5t')....S2-4
5. Select computed parameters....S2-6
6. Scan of V_{bur} radius....S2-7
7. References....S2-7
8. Coordinates for transition states on the energy pathway of product 5a....S2-9
9. Coordinates for compounds on the energy pathway of product 5f/5f'....S2-22
10. Coordinates for compounds on the energy pathway of product 5t/5t' S2-40
11. Coordinates for pyrroles used in the steric regioselectivity model....S2-80

1. Computational Methods

Conformational searches were conducted for the pyrroles used in the steric model with Macromodel version 11.8.012[1] and the OPLS3e force field.[2] Conformers within 10 kcal/mol of the minimum (typically 1-100) were used as starting coordinates for geometry optimization conducted using density functional theory (DFT) in Gaussian16 (Revision A.03).[3] Each of the **pyrroles in the steric model** were optimized using the polarizable continuum model (PCM) in acetonitrile (MeCN) at the B3LYPD3BJ/6-31g(d) level of theory. Single point energy calculations were conducted using PCM(MeCN) at the M062x/def2TZVP level and used to collect molecular descriptors.

Energies for the **transition states** were calculated using PCM(MeCN)-B3LYPD3BJ/6-311+g(d,p)//PCM(MeCN)-B3LYPD3BJ/6-31g(d) and were optimized without constraints except TS1-exoC2 for 2,5-dimethylpyrrole (5a). This transition state was optimized constraining the carbene-C2 distance taken from the B3LYPD3/6-31g(d) optimized geometry.

All of the optimized geometries were verified by frequency calculations as ground states (zero imaginary frequencies) or transition states (one imaginary frequency). Any conformers that did not optimize to true minima were discarded (<0.5% of all conformers). Transition states were verified by intrinsic reaction coordinate (IRC) calculations. Quasi-harmonic corrected free energies (Gtz) for each compound were computed using Grimme entropic corrections[4] and Head-Gordon enthalpic corrections[5] as implemented in Paton's GoodVibes python package.[6] Nucleus independent chemical shift (NICS0)[7] values were computed by the negative calculated chemical shift of a ghost atom placed at the center of the five pyrrole atoms (calculated as the cartesian coordinate average) using the Gauge-Independent Atomic Orbital (GIAO) method[8–12] with B3LYP/6-311+g(d,p). Buried volume parameters were computed using Paton's DBStep[13] program with C3 and C4 defined using IUPAC naming conventions. Differences in buried volume were visualized using Blender.[14]

2. Triplet energies and azabenzvalene pathway for 2,5-dimethylpyrrole (4a)

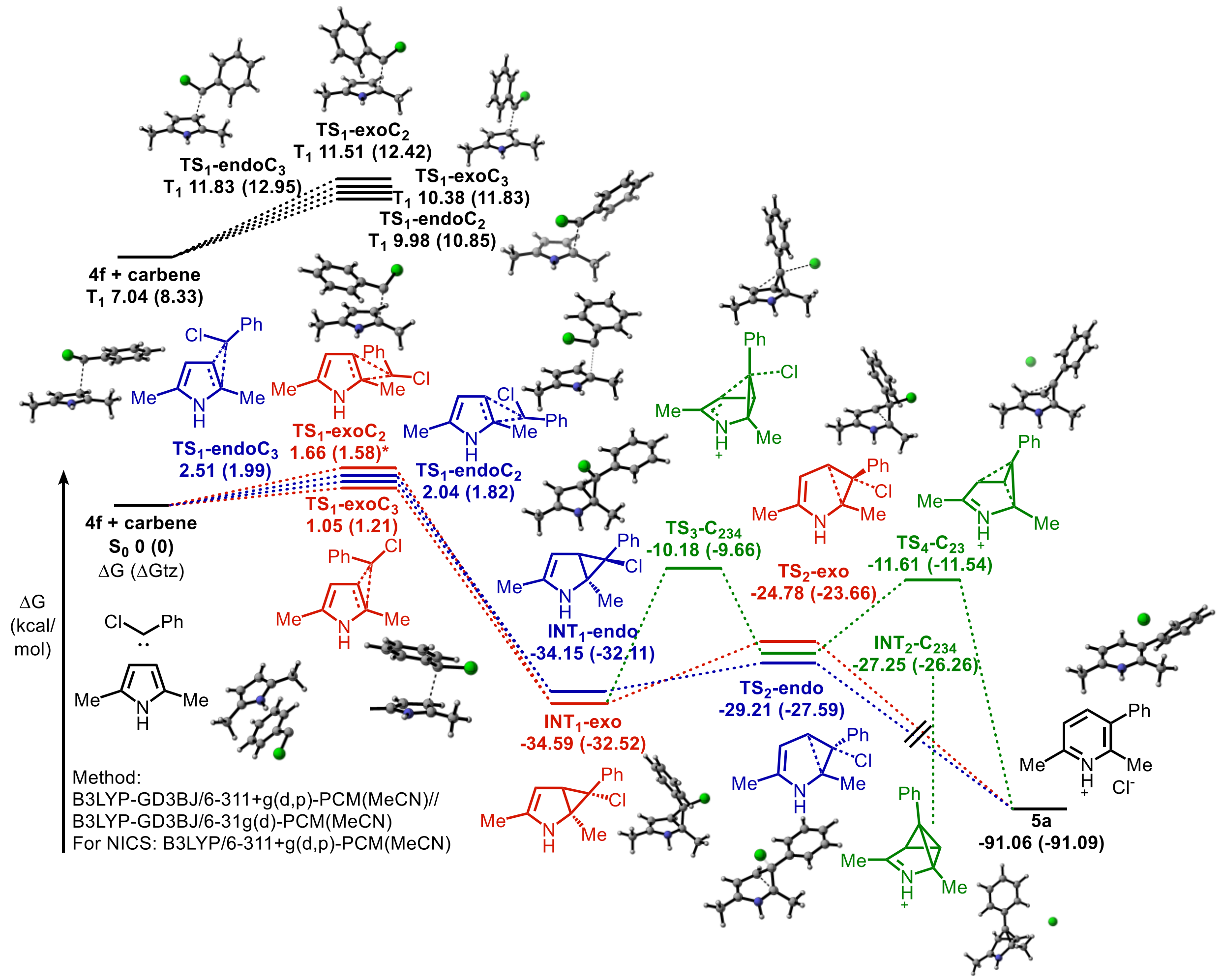

Fig S1: Triplet energies for the α-halocarbene and triplet cyclopropanation energies are significantly higher than the singlet pathway, as supported by previous works.[15–17] Azabenzvalene ring walking is not energetically competitive with ring opening, even when the ring opening is electronically forbidden.

3. Energy pathway and predicted selectivity for 2-phenyl-5-methylpyrrole (4f)

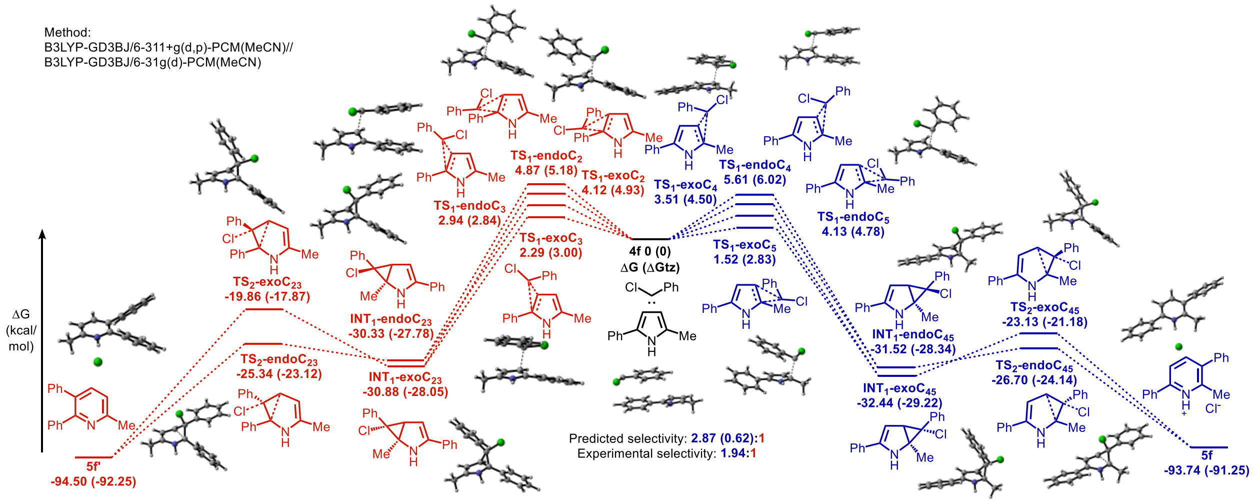

Fig. S2: Boltzmann weighting of the cyclopropanation transition states predicts the selectivity of 2-phenyl-5-methylpyrrole to be 2.87:1, near the experimental value of 1.94:1.

4. Plausible mechanism for para-insertion (5t')

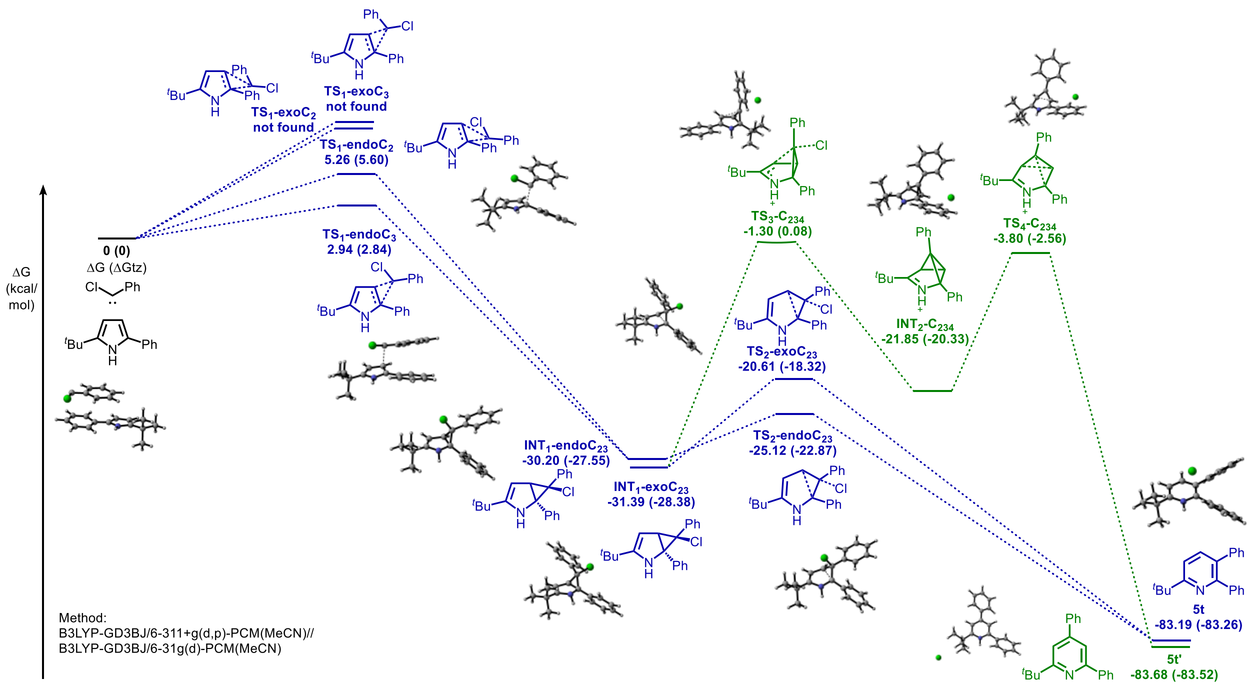

Fig. S3: formation of meta-insertion product 5t follows same pathway as others, although exo-Cl cyclopropanation appears difficult due to steric interaction between carbene aryl ring and pyrrole *tert*-butyl group. Azabenzvalene walking appears noncompetitive with ring opening to meta insertion.

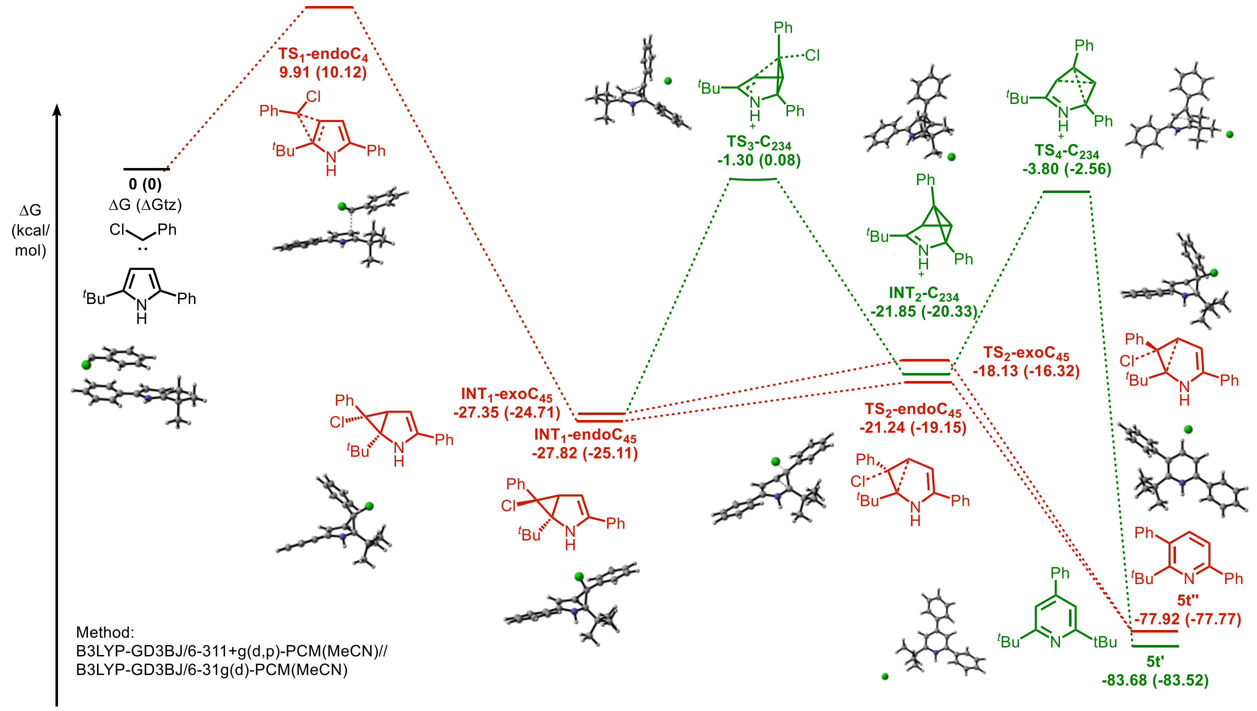

Fig. S4: The azabenzvalene formation appears noncompetitive with electrocyclic ring opening.

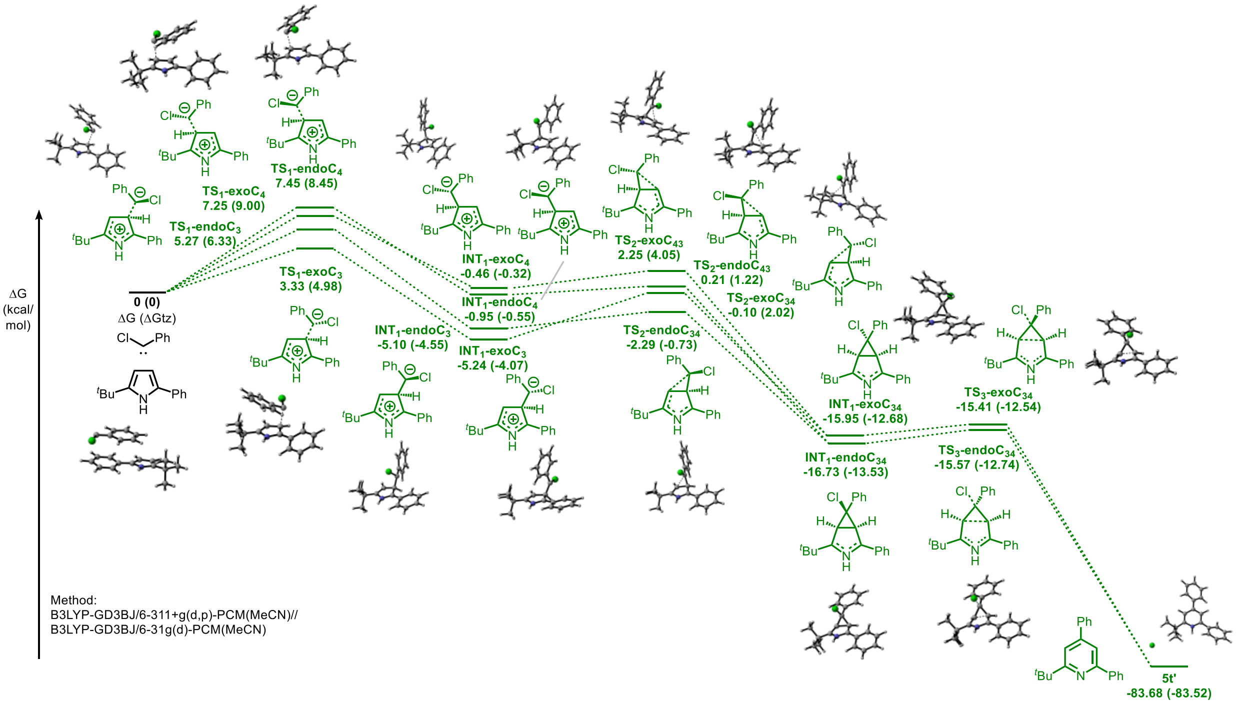

Fig. S6: Direct cyclopropanation between C3 and C4 is energetically competitive with cyclopropanation at the other positions of the pyrrole.

5. Select computed parameters

Substrate	Regioselectivity (C5/C2)	log (Regioselectivity)	C3R3.75_Boltz-C4R3.75_Boltz	ChelpG ((C2+C3)-(C4+C5))	calculated NMR ((C2+C3)-(C4+C5))	Sterimol L ((C2+C3)-(C4+C5))	Sterimol B1 ((C2+C3)-(C4+C5))	Sterimol B5 ((C2+C3)-(C4+C5))
4a	1.00	0.000	0.000	0.000	0.000	0.000	0.000	0.000
4b	0.05	-1.301	-6.873	-0.218	13.825	11.410	4.260	11.460
4c	20.00	1.301	10.094	0.137	-12.208	7.570	0.610	3.320
4d	20.00	1.301	6.032	0.087	-15.563	3.802	0.631	3.317
4e	20.00	1.301	8.378	0.170	-13.362	7.680	0.687	7.262
4f	1.90	0.279	2.684	-0.100	2.717	3.270	0.000	1.160
4g	1.50	0.176	2.655	-0.065	2.691	5.358	0.104	1.156
4h	2.10	0.322	2.686	-0.136	6.193	5.230	0.000	1.170
4i	2.00	0.301	2.684	-0.100	2.717	3.270	0.000	1.160
4j	1.30	0.114	2.684	-0.100	2.717	3.270	0.000	1.160
4k	1.20	0.079	2.684	-0.100	2.717	3.270	0.000	1.160
4l	2.40	0.380	4.547	0.197	-12.201	1.030	0.610	1.030
4m	1.10	0.041	0.694	-0.032	7.502	2.175	0.000	0.025
4n	0.38	-0.415	-1.467	-0.019	11.659	2.208	-0.302	0.002
4o	1.00	0.000	-0.534	0.056	13.007	0.703	0.027	-1.216
4q	9.00	0.954	6.219	-0.010	-4.445	4.030	0.965	6.050
4s	0.05	-1.301	-6.380	0.049	17.481	14.409	4.934	14.266
4u	1.00	0.000	-0.001	0.000	0.001	0.000	0.000	0.000
4t	0.10	-1.018	-3.233	0.038	14.081	2.240	-1.150	0.000
4p	0.33	-0.477	7.395	0.050	-4.589	2.733	1.074	4.831
4r	0.05	-1.301	1.664	0.120	16.073	1.129	-0.193	-2.404

Table S1: Select computed molecular descriptors from pyrrole ground states. Verloop sterimol values[18] were computed using a modified version of Paton's sterimol package.[19]

6. Scan of V_{bur} radius

Using Paton's DBStep[13] python package, buried volume parameters were computed using spheres of various radii. Initially, V_{bur} was calculated about C3 and C4 of the pyrrole using radii from 2.0 to 5.0 angstroms with 0.25 Å increments. A finer scan with a smaller increment (0.1 Å) was also performed from 3.4 to 4.2 Å in an attempt to further optimize the correlation. Ultimately, it was decided that a radius of 3.75 Å would be used.

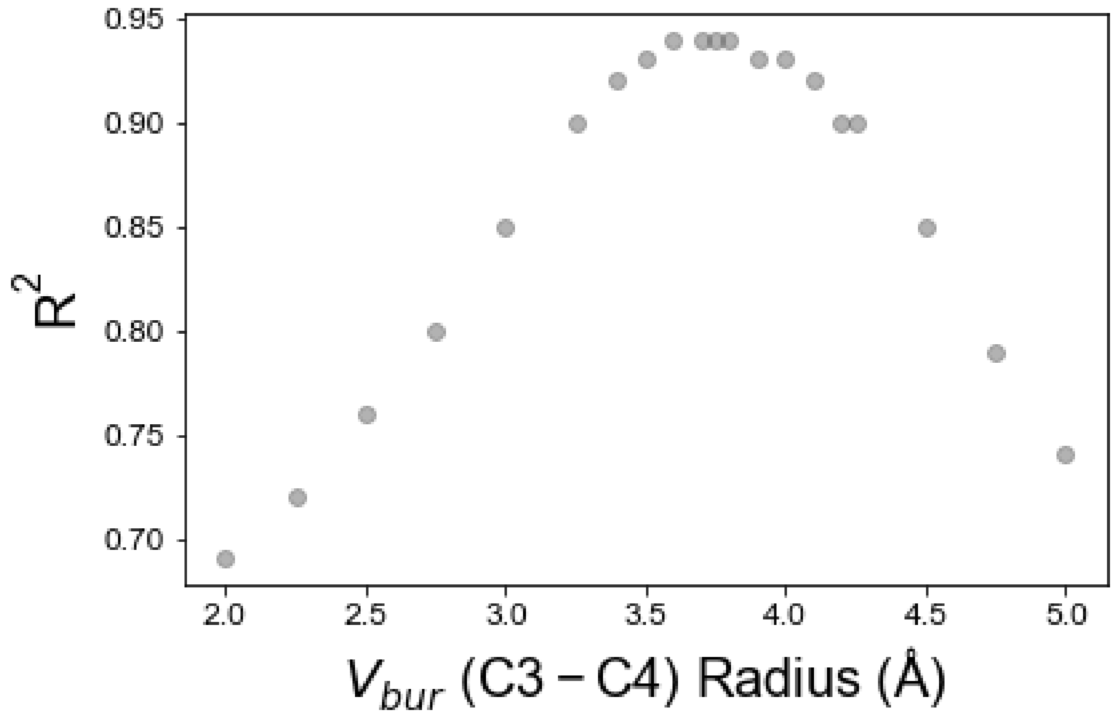

7. References

(1) *MacroModel*; Schrödinger, LLC, New York, NY, 2021.

(2) Roos, K.; Wu, C.; Damm, W.; Reboul, M.; Stevenson, J. M.; Lu, C.; Dahlgren, M. K.; Mondal, S.; Chen, W.; Wang, L.; Abel, R.; Friesner, R. A.; Harder, E. D. OPLS3e: Extending Force Field Coverage for Drug-Like Small Molecules. *J. Chem. Theory Comput.* **2019**, *15* (3), 1863–1874. https://doi.org/10.1021/acs.jctc.8b01026.

(3) M. J. Frisch, G. W. Trucks, H. B. Schlegel, G. E. Scuseria,; M. A. Robb, J. R. Cheeseman, G. Scalmani, V. Barone,; G. A. Petersson, H. Nakatsuji, X. Li, M. Caricato, A. V. Marenich,; J. Bloino, B. G. Janesko, R. Gomperts, B. Mennucci, H. P. Hratchian,; J. V. Ortiz, A. F. Izmaylov, J. L. Sonnenberg, D. Williams-Young,; F. Ding, F. Lipparini, F. Egidi, J. Goings, B. Peng, A. Petrone,; T. Henderson, D. Ranasinghe, V. G. Zakrzewski, J. Gao, N. Rega,; G. Zheng, W. Liang, M. Hada, M. Ehara, K. Toyota, R. Fukuda,; J. Hasegawa, M. Ishida, T. Nakajima, Y. Honda, O. Kitao, H. Nakai,; T. Vreven, K. Throssell, J. A. Montgomery, Jr., J. E. Peralta,; F. Ogliaro, M. J. Bearpark, J.

J. Heyd, E. N. Brothers, K. N. Kudin,; V. N. Staroverov, T. A. Keith, R. Kobayashi, J. Normand,; K. Raghavachari, A. P. Rendell, J. C. Burant, S. S. Iyengar,; J. Tomasi, M. Cossi, J. M. Millam, M. Klene, C. Adamo, R. Cammi,; J. W. Ochterski, R. L. Martin, K. Morokuma, O. Farkas,; J. B. Foresman, and D. J. Fox. *Gaussian 16, Revision A.03*; Gaussian Inc.: Wallingford CT, 2016.

(4) Grimme, S. Supramolecular Binding Thermodynamics by Dispersion-Corrected Density Functional Theory. *Chem. – Eur. J.* **2012**, *18* (32), 9955–9964. https://doi.org/10.1002/chem.201200497.

(5) Li, Y.-P.; Gomes, J.; Mallikarjun Sharada, S.; Bell, A. T.; Head-Gordon, M. Improved Force-Field Parameters for QM/MM Simulations of the Energies of Adsorption for Molecules in Zeolites and a Free Rotor Correction to the Rigid Rotor Harmonic Oscillator Model for Adsorption Enthalpies. *J. Phys. Chem. C* **2015**, *119* (4), 1840–1850. https://doi.org/10.1021/jp509921r.

(6) Luchini, G.; Alegre-Requena, J. V.; Funes-Ardoiz, I.; Paton, R. S. GoodVibes: Automated Thermochemistry for Heterogeneous Computational Chemistry Data. *F1000Research* **2020**, *9*, 291. https://doi.org/10.12688/f1000research.22758.1.

(7) Chen, Z.; Wannere, C. S.; Corminboeuf, C.; Puchta, R.; Schleyer, P. von R. Nucleus-Independent Chemical Shifts (NICS) as an Aromaticity Criterion. *Chem. Rev.* **2005**, *105* (10), 3842–3888. https://doi.org/10.1021/cr030088+.

(8) London, F. Théorie Quantique Des Courants Interatomiques Dans Les Combinaisons Aromatiques. *J. Phys. Radium* **1937**, *8* (10), 397–409. https://doi.org/10.1051/jphysrad:01937008010039700.

(9) McWeeny, R. Perturbation Theory for the Fock-Dirac Density Matrix. *Phys Rev* **1962**, *126* (3), 1028–1034. https://doi.org/10.1103/PhysRev.126.1028.

(10) Ditchfield, R. Self-Consistent Perturbation Theory of Diamagnetism. *Mol. Phys.* **1974**, *27* (4), 789–807. https://doi.org/10.1080/00268977400100711.

(11) Wolinski, K.; Hinton, J. F.; Pulay, P. Efficient Implementation of the Gauge-Independent Atomic Orbital Method for NMR Chemical Shift Calculations. *J. Am. Chem. Soc.* **1990**, *112* (23), 8251–8260. https://doi.org/10.1021/ja00179a005.

(12) Cheeseman, J. R.; Trucks, G. W.; Keith, T. A.; Frisch, M. J. A Comparison of Models for Calculating Nuclear Magnetic Resonance Shielding Tensors. *J. Chem. Phys.* **1996**, *104* (14), 5497–5509. https://doi.org/10.1063/1.471789.

(13) Guilian Luchini; Lab, R. P. / P. *Bobbypaton/DBSTEP: 1.2-Alpha Release*; Zenodo, 2021. https://doi.org/10.5281/ZENODO.4702097.

(14) Community, B. O. *Blender - a 3D Modelling and Rendering Package*; Blender Foundation: Stichting Blender Foundation, Amsterdam, 2018.

(15) Moss, R. A.; Lu, Z.; Sauers, R. R. Triplet Halocarbene Chemistry: P-Nitrophenylchlorocarbene and p-Nitrophenylbromocarbene. *Tetrahedron Lett.* **2010**, *51* (45), 5940–5942. https://doi.org/10.1016/j.tetlet.2010.09.018.

(16) Moss, R. A.; Turro, N. J. Laser Flash Photolytic Studies of Arylhalocarbenes. In *Kinetics and Spectroscopy of Carbenes and Biradicals*; Platz, M. S., Ed.; Springer US: Boston, MA, 1990; pp 213–238. https://doi.org/10.1007/978-1-4899-3707-0_7.

(17) Kable, S. H.; Reid, S. A.; Sears, T. J. The Halocarbenes: Model Systems for Understanding the Spectroscopy, Dynamics and Chemistry of Carbenes. *Int. Rev. Phys. Chem.* **2009**, *28* (3), 435–480. https://doi.org/10.1080/01442350903087792.

(18) Verloop, A. In *Drug Design*; Ariens, E. J., Ed.; Academic Press, 1976; Vol. 3.

(19) Brethomé, A. V.; Fletcher, S. P.; Paton, R. S. Conformational Effects on Physical-Organic Descriptors: The Case of Sterimol Steric Parameters. *ACS Catal.* **2019**, *9* (3), 2313–2323. https://doi.org/10.1021/acscatal.8b04043.

8. Coordinates for transition states on the energy pathway of product 5a

2Me-5Me-234azabenzvalene_23insertion_ring-opening_TS

29

C	2.03250	0.80490	-0.35629
C	2.83056	-0.25610	-0.65480
N	2.46794	-1.36535	0.08206
H	3.17095	-1.99381	0.45668
H	1.98395	1.74224	-0.89416
C	1.29797	-1.11214	0.82342
C	1.05942	0.54531	0.76554
H	0.80454	1.30758	1.49790
C	0.16736	-0.39037	0.20619
C	-1.19745	-0.56908	-0.06726
C	-2.06302	0.55652	-0.06430
C	-1.70149	-1.86088	-0.37029
C	-3.40740	0.37760	-0.35033
H	-1.64788	1.54195	0.14524
C	-3.05004	-2.02338	-0.63681
H	-1.02312	-2.70761	-0.38326
C	-3.89850	-0.90559	-0.62801
H	-4.08121	1.22797	-0.35611
H	-3.45048	-3.00661	-0.85898
H	-4.95425	-1.03784	-0.84462
Cl	-0.33090	3.73226	0.31867
C	0.99457	-2.02073	1.98984

H	1.80434	-1.96981	2.72458
H	0.06586	-1.72607	2.48198
H	0.89306	-3.05659	1.65041
C	3.94687	-0.33516	-1.64385
H	3.69847	-1.01751	-2.46421
H	4.15860	0.65173	-2.05982
H	4.85888	-0.71204	-1.16557

2Me-5Me-azabenzvalene

29

C	1.54291	-0.13575	-1.09303
C	2.78632	-0.67471	-0.51670
N	2.68823	-0.47933	0.76597
H	3.36538	-0.75133	1.47029
H	1.32849	-0.15588	-2.15311
C	1.38766	0.17181	1.02926
C	1.04539	0.99637	-0.18567
H	0.66681	1.99990	-0.37094
C	0.41668	-0.30127	-0.04109
C	-0.95277	-0.82879	-0.07774
C	-2.04103	0.04716	-0.19962
C	-1.17547	-2.20953	0.02922
C	-3.34083	-0.45958	-0.21253
H	-1.85983	1.11668	-0.28038
C	-2.47534	-2.71039	0.01248
H	-0.32770	-2.88214	0.12282
C	-3.55918	-1.83518	-0.10785
H	-4.18324	0.21965	-0.30537
H	-2.64493	-3.77991	0.09360
H	-4.57225	-2.22672	-0.11993
Cl	-1.01467	3.82903	-0.41677
C	3.91414	-1.32045	-1.22082
H	4.70946	-1.62132	-0.53735
H	3.54451	-2.19872	-1.76255
H	4.31596	-0.63029	-1.97097
C	1.01258	0.49719	2.44139
H	0.97858	-0.40730	3.05607
H	1.72297	1.20082	2.88524
H	0.02128	0.95702	2.45201

2Me-5Me-endoCl_C2_cyclopropanation_TS

29

C	-1.86270	-0.27119	1.58479
C	-2.84290	-0.12139	0.60639
N	-2.44539	0.90051	-0.21281
H	-2.93319	1.18451	-1.05111
H	-1.88430	-1.00829	2.37629
C	-1.19689	1.37381	0.15379
C	-0.84700	0.66530	1.31539
H	0.06600	0.82110	1.87289
C	0.23590	0.00570	-1.22541
C	1.50380	-0.17161	-0.48801
C	1.90749	-1.25281	0.32789
C	2.41641	0.88949	-0.68081
C	3.16139	-1.26352	0.92629
H	1.22019	-2.07521	0.49759
C	3.67491	0.87958	-0.08041
H	2.12001	1.71179	-1.32491
C	4.04970	-0.19692	0.72639
H	3.45399	-2.10022	1.55509
H	4.36231	1.70457	-0.24531
H	5.02930	-0.21123	1.19629
Cl	-0.69611	-1.54050	-1.29291
C	-0.68158	2.68180	-0.35581
H	0.37352	2.79020	-0.09331
H	-0.76798	2.74330	-1.44441
H	-1.23048	3.52251	0.08889
C	-4.10810	-0.87838	0.37329
H	-4.98080	-0.21317	0.36789
H	-4.08811	-1.40178	-0.59061
H	-4.25141	-1.62238	1.16109

2Me-5Me-endoCl_C2_cyclopropanation_TS_triplet

29

C	-2.61619	-1.10109	-1.25542
C	-3.18149	-0.15159	-0.41022
N	-2.46246	-0.17388	0.76146
H	-2.59076	0.48446	1.51701
H	-2.96870	-1.33348	-2.25144
C	-1.37810	-1.02849	0.66541
C	-1.52024	-1.67888	-0.58404
H	-0.87850	-2.47140	-0.94362
C	0.23818	0.50314	0.00405
C	1.62451	0.23230	-0.11388
C	2.64467	1.21916	0.01919
C	2.04120	-1.10711	-0.36965

C	3.98592	0.87731	-0.08810
H	2.36090	2.24837	0.21070
C	3.38551	-1.43225	-0.47359
H	1.28686	-1.87435	-0.49838
C	4.37218	-0.44672	-0.33235
H	4.74232	1.65037	0.02003
H	3.67165	-2.46172	-0.67139
H	5.42329	-0.70598	-0.41448
Cl	-0.36154	2.13642	0.17675
C	-0.66974	-1.49399	1.90273
H	0.28534	-1.95776	1.64775
H	-0.46704	-0.65854	2.57998
H	-1.27501	-2.23233	2.44416
C	-4.31832	0.79027	-0.62068
H	-4.81112	0.57776	-1.57274
H	-5.06631	0.70659	0.17758
H	-3.97804	1.83378	-0.64081

2Me-5Me-endoCl_C3_cyclopropanation_TS

29

C	2.46235	-0.60094	1.23284
C	3.02384	-0.37107	0.00361
N	2.09852	-0.78280	-0.95202
H	2.23406	-0.70227	-1.95036
H	2.91946	-0.37099	2.18530
C	0.96430	-1.25891	-0.36358
C	1.13462	-1.10416	1.02205
H	0.48097	-1.52890	1.76798
C	-0.18026	0.88766	1.08020
C	-1.45754	0.49971	0.46330
C	-2.24575	-0.35219	1.27224
C	-2.00570	0.93987	-0.76094
C	-3.51011	-0.76411	0.86927
H	-1.83733	-0.67687	2.22478
C	-3.28283	0.54740	-1.15288
H	-1.42502	1.59987	-1.39582
C	-4.03473	-0.31177	-0.34700
H	-4.09467	-1.42466	1.50280
H	-3.69203	0.90514	-2.09346
H	-5.02775	-0.62106	-0.65987
Cl	0.74923	2.01085	0.00202
C	-0.15415	-1.83370	-1.15810
H	-0.62177	-1.07416	-1.79891
H	0.19669	-2.64714	-1.80434

H	-0.92755	-2.22732	-0.49676
C	4.33786	0.21417	-0.39070
H	4.21089	1.15427	-0.94221
H	4.93449	0.42454	0.50058
H	4.91156	-0.46703	-1.03173

2Me-5Me-endoCl_C3_cyclopropanation_TS_triplet

29

C	-2.29905	-0.12861	1.28467
C	-3.25808	-0.04817	0.31006
N	-2.82949	0.89418	-0.61886
H	-3.34429	1.15509	-1.44842
H	-2.33281	-0.77949	2.14730
C	-1.62911	1.43979	-0.24850
C	-1.19887	0.73526	0.90854
H	-0.48657	1.14618	1.61227
C	0.30630	-0.55327	0.10056
C	1.69659	-0.26555	0.09970
C	2.13297	1.03635	0.48754
C	2.70396	-1.20180	-0.27744
C	3.47947	1.36942	0.49649
H	1.39804	1.78092	0.77012
C	4.04732	-0.85247	-0.26421
H	2.40782	-2.20077	-0.57809
C	4.45139	0.43174	0.12219
H	3.77850	2.37065	0.79483
H	4.79141	-1.58852	-0.55759
H	5.50394	0.69805	0.12993
Cl	-0.28800	-2.11187	-0.43651
C	-0.90848	2.44064	-1.08249
H	-1.60016	3.00415	-1.71712
H	-0.36681	3.15532	-0.45450
H	-0.16809	1.95695	-1.73554
C	-4.55542	-0.76638	0.14348
H	-4.55939	-1.39048	-0.75934
H	-4.73396	-1.41756	1.00297
H	-5.39918	-0.06959	0.06242

2Me-5Me-endoCl_cyclopropane

29

C	-2.28819	-0.49912	1.29868
C	-3.02618	0.03234	0.30114

N	-2.25835	0.88223	-0.51665
H	-2.69207	1.75951	-0.78718
H	-2.63732	-1.23000	2.01632
C	-0.92995	1.00231	0.04107
C	-0.89783	0.00737	1.23087
H	-0.33351	0.19392	2.14216
C	-0.07174	-0.24257	-0.02177
C	1.41834	-0.13630	0.04206
C	2.12613	-0.64240	1.13747
C	2.12849	0.44572	-1.01785
C	3.51930	-0.56104	1.18006
H	1.58420	-1.10344	1.95768
C	3.51881	0.52733	-0.97801
H	1.58279	0.83184	-1.87396
C	4.21808	0.02495	0.12355
H	4.05697	-0.95702	2.03675
H	4.05743	0.98392	-1.80336
H	5.30177	0.09035	0.15648
Cl	-0.56918	-1.57811	-1.15547
C	-0.33205	2.38570	0.02786
H	0.64634	2.39635	0.51119
H	-0.20621	2.75026	-0.99860
H	-0.98868	3.08092	0.56293
C	-4.45186	-0.22432	-0.06152
H	-4.52515	-0.73649	-1.02865
H	-4.93787	-0.84323	0.69683
H	-5.01233	0.71554	-0.15065

2Me-5Me-endoCl_ring_opening_TS

29

C	2.26958	-0.79635	-1.19254
C	2.99561	-0.05231	-0.31690
N	2.25149	0.98956	0.22750
H	2.72083	1.87519	0.37704
H	2.70209	-1.57770	-1.80598
C	0.90970	1.04272	-0.15013
C	0.86814	-0.45874	-1.25801
H	0.30049	-0.64121	-2.16676
C	0.11456	-0.18446	-0.02497
C	-1.38213	-0.10717	-0.08698
C	-2.10492	-0.88214	-0.99962
C	-2.08122	0.69906	0.82290
C	-3.50010	-0.83501	-1.02065
H	-1.58115	-1.53327	-1.69168

C	-3.47272	0.74815	0.80334
H	-1.52817	1.27996	1.55442
C	-4.18715	-0.01726	-0.12309
H	-4.04790	-1.44057	-1.73670
H	-4.00038	1.38030	1.51145
H	-5.27245	0.02047	-0.13989
Cl	0.52753	-1.29539	1.52127
C	0.32087	2.41442	-0.33659
H	-0.66170	2.36267	-0.80578
H	0.20457	2.92801	0.62723
H	0.97528	3.02381	-0.96836
C	4.42547	-0.20928	0.08567
H	4.51394	-0.57467	1.11641
H	4.92749	-0.92367	-0.57198
H	4.96449	0.74579	0.03181

2Me-5Me-exoCl-azabenzvalene-forming_TS

29

C	1.93780	-0.43794	-1.38127
C	2.64540	-0.96331	-0.33191
N	2.40287	-0.28501	0.84550
H	3.19828	-0.04836	1.43373
H	1.82334	-0.89987	-2.35306
C	1.43805	0.76256	0.55248
C	1.24398	0.84104	-1.00408
H	1.14472	1.69942	-1.66112
C	0.22680	0.29240	-0.14651
C	-0.96950	-0.43080	-0.01485
C	-1.82167	-0.64125	-1.12650
C	-1.30152	-0.98134	1.24602
C	-2.97335	-1.39287	-0.97407
H	-1.55635	-0.20587	-2.08356
C	-2.46800	-1.71903	1.39104
H	-0.63645	-0.81466	2.08676
C	-3.29856	-1.92585	0.28365
H	-3.63032	-1.56391	-1.82036
H	-2.73297	-2.13868	2.35570
H	-4.20876	-2.50660	0.39807
Cl	-1.39533	2.93373	-0.37395
C	1.40440	1.93074	1.50647
H	2.38429	2.41932	1.52389
H	0.64509	2.64547	1.18683
H	1.16680	1.58429	2.51779
C	3.57671	-2.12866	-0.35126

H	3.19132	-2.93910	0.27765
H	3.71166	-2.50157	-1.36807
H	4.55664	-1.84224	0.04930

2Me-5Me-exoCl_C2_cyclopropanation_TS

29

C	-1.81624	-0.30244	1.58535
C	-2.80524	-0.14085	0.60887
N	-2.40529	0.86552	-0.21725
H	-2.89019	1.13362	-1.06258
H	-1.84591	-1.03319	2.38165
C	-1.12070	1.30599	0.10783
C	-0.79113	0.60608	1.30021
H	0.12561	0.75258	1.85264
C	0.17009	0.10231	-1.14940
C	1.46509	-0.11934	-0.45392
C	1.88436	-1.26464	0.25394
C	2.38480	0.94121	-0.59139
C	3.15768	-1.33707	0.80778
H	1.19461	-2.09297	0.37605
C	3.65814	0.87473	-0.02744
H	2.08686	1.81013	-1.16955
C	4.04976	-0.26585	0.67538
H	3.45911	-2.22801	1.35218
H	4.34737	1.70574	-0.14792
H	5.04328	-0.32665	1.11026
Cl	-0.73892	-1.46357	-1.31405
C	-4.08527	-0.87604	0.40295
H	-4.10534	-1.36841	-0.57626
H	-4.21000	-1.63962	1.17393
H	-4.94566	-0.19715	0.44823
C	-0.68043	2.67904	-0.29989
H	0.37343	2.81930	-0.05098
H	-0.79906	2.82183	-1.37746
H	-1.26085	3.44606	0.22785

2Me-5Me-exoCl_C2_cyclopropanation_TS_triplet

29

C	-1.51434	1.29785	-1.57606
C	-1.05573	2.04274	-0.48914
N	-1.45790	1.38521	0.64541
H	-1.24694	1.68788	1.58533

H	-1.35628	1.55901	-2.61403
C	-2.06470	0.17597	0.33667
C	-2.18143	0.16725	-1.07630
H	-2.64639	-0.62615	-1.64474
C	-0.27583	-1.24129	0.38194
C	1.04225	-0.72943	0.29915
C	1.99827	-1.18026	-0.65771
C	1.44126	0.32128	1.17525
C	3.25166	-0.58991	-0.74564
H	1.72837	-1.98600	-1.33236
C	2.69857	0.89976	1.07542
H	0.75480	0.64964	1.94644
C	3.61484	0.45741	0.11095
H	3.95688	-0.94799	-1.49138
H	2.97578	1.69417	1.76334
H	4.59707	0.91388	0.03581
Cl	-0.70835	-2.79302	-0.28501
C	-2.91576	-0.53194	1.34860
H	-3.88642	-0.03336	1.46396
H	-3.09763	-1.56127	1.03034
H	-2.42760	-0.56074	2.32748
C	-0.22294	3.27682	-0.43162
H	-0.65770	4.02987	0.23707
H	0.78641	3.05132	-0.06162
H	-0.12981	3.71716	-1.42753

2Me-5Me-exoCl_C3_cyclopropanation_TS

29

C	-0.79029	-2.14046	-0.96619
C	-1.67555	-1.65548	-0.04282
N	-0.93186	-1.27731	1.07766
H	-1.32335	-0.84238	1.90214
H	-1.04609	-2.50145	-1.95250
C	0.39332	-1.50444	0.88628
C	0.54077	-1.97766	-0.43731
H	1.43545	-2.45341	-0.80970
C	1.21589	-0.01790	-1.24594
C	0.37317	0.99108	-0.58698
C	-0.93104	1.08177	-1.12640
C	0.72254	1.88783	0.44832
C	-1.85282	2.00384	-0.63420
H	-1.19328	0.42865	-1.94965
C	-0.19520	2.81391	0.93022
H	1.72501	1.85798	0.86031

C	-1.49005	2.86869	0.39969
H	-2.84820	2.05615	-1.06597
H	0.09450	3.49776	1.72323
H	-2.20401	3.59286	0.78139
Cl	2.95919	0.08839	-0.62074
C	1.39210	-1.23660	1.95335
H	1.04652	-1.62941	2.91589
H	2.35007	-1.69689	1.71121
H	1.55553	-0.15654	2.07392
C	-3.15829	-1.50276	-0.06869
H	-3.45130	-0.45519	0.07018
H	-3.55115	-1.84091	-1.03073
H	-3.64007	-2.08975	0.72313

2Me-5Me-exoCl_C3_cyclopropanation_TS_triplet

29

C	1.99095	0.20737	-1.33418
C	2.52497	-0.88562	-0.70427
N	2.53524	-0.61245	0.66139
H	2.86010	-1.25509	1.37084
H	1.84673	0.30943	-2.40092
C	2.03055	0.63620	0.91595
C	1.60134	1.16975	-0.32293
H	1.50369	2.23412	-0.49306
C	-0.57005	1.16645	-0.21865
C	-1.42198	0.04740	-0.03380
C	-0.84080	-1.20585	0.31818
C	-2.83870	0.09471	-0.18016
C	-1.63081	-2.33046	0.50572
H	0.23139	-1.27134	0.44434
C	-3.61406	-1.04112	0.01115
H	-3.31181	1.03362	-0.44633
C	-3.02261	-2.26343	0.35389
H	-1.16014	-3.27245	0.77481
H	-4.69253	-0.97559	-0.10754
H	-3.63514	-3.14772	0.50182
Cl	-1.22821	2.75133	-0.60664
C	1.86897	1.17558	2.29391
H	1.74752	2.26176	2.26139
H	0.97826	0.75900	2.78530
H	2.73434	0.94439	2.92516
C	3.01973	-2.18885	-1.23655
H	2.40467	-3.02809	-0.88690
H	2.98801	-2.18141	-2.32901

H	4.05290	-2.38885	-0.92644

2Me-5Me-exoCl_cyclopropane

29

C	-1.15135	1.35484	-1.37990
C	-1.08506	2.03376	-0.21402
N	-1.31942	1.19808	0.89417
H	-1.92082	1.57373	1.62150
H	-0.93755	1.76762	-2.35697
C	-1.68127	-0.12459	0.41171
C	-1.49644	-0.06460	-1.11725
H	-2.12412	-0.63392	-1.79840
C	-0.52657	-0.85372	-0.24451
C	0.89580	-0.46267	-0.04297
C	1.71861	-0.18777	-1.14051
C	1.42751	-0.39204	1.24929
C	3.05907	0.14749	-0.95044
H	1.30025	-0.23610	-2.14142
C	2.76779	-0.05678	1.44253
H	0.78358	-0.59628	2.09922
C	3.58549	0.21401	0.34237
H	3.69177	0.35791	-1.80804
H	3.17344	-0.00424	2.44891
H	4.62887	0.47716	0.49185
Cl	-0.70677	-2.66472	-0.36968
C	-2.80545	-0.80402	1.14688
H	-3.70287	-0.17630	1.11853
H	-3.04704	-1.76833	0.69825
H	-2.53596	-0.97411	2.19624
C	-0.73156	3.46464	0.02562
H	0.21468	3.54688	0.57416
H	-0.63303	4.00249	-0.92054
H	-1.49959	3.96595	0.62925

2Me-5Me-exoCl_ring_opening_TS

29

C	-1.30116	1.28272	-1.42355
C	-1.30419	1.91663	-0.20543
N	-1.53738	1.04940	0.84962
H	-2.04400	1.40055	1.65429
H	-1.35375	1.84373	-2.35080
C	-1.59114	-0.30537	0.58251

C	-1.32170	-0.14030	-1.38938
H	-1.72295	-0.75110	-2.19139
C	-0.50398	-0.78136	-0.31295
C	0.91751	-0.34176	-0.06138
C	1.75558	-0.01980	-1.13207
C	1.41024	-0.27248	1.24467
C	3.07748	0.35956	-0.90149
H	1.36100	-0.06048	-2.14312
C	2.73151	0.10995	1.47919
H	0.75408	-0.51336	2.07623
C	3.56791	0.42600	0.40579
H	3.72372	0.60817	-1.73866
H	3.10719	0.16387	2.49710
H	4.59604	0.72646	0.58688
Cl	-0.52113	-2.61969	-0.49429
C	-2.48800	-1.16730	1.41918
H	-3.33190	-0.58423	1.80070
H	-2.89099	-1.99697	0.83529
H	-1.95183	-1.60406	2.27360
C	-1.15978	3.37690	0.07866
H	-0.17449	3.61961	0.49759
H	-1.28039	3.95218	-0.84370
H	-1.91256	3.72432	0.79928

4a-plus-carbene-singlet

29

C	-1.46742	-2.02388	0.27383
C	-2.11497	-0.98747	-0.36572
N	-1.92875	0.13703	0.41328
H	-2.29796	1.05088	0.19313
H	-1.41088	-3.04218	-0.08820
C	-1.18495	-0.15996	1.54087
C	-0.88283	-1.50303	1.47203
H	-0.29612	-2.04811	2.19983
C	0.08649	1.26051	-1.35410
C	1.07061	0.39479	-0.75978
C	1.08582	-0.89561	-1.34635
C	1.98889	0.67368	0.28680
C	1.99087	-1.86358	-0.92079
H	0.38128	-1.10044	-2.14377
C	2.88552	-0.29129	0.70738
H	1.98718	1.65453	0.74822
C	2.88831	-1.55984	0.10188
H	1.99599	-2.84708	-1.37931

H	3.58963	-0.07244	1.50408
H	3.59858	-2.31039	0.43667
Cl	0.06951	2.88523	-0.58343
C	-0.83320	0.87868	2.55381
H	-0.24322	1.69366	2.11620
H	-1.72383	1.33116	3.00948
H	-0.23971	0.42799	3.35412
C	-2.83798	-0.92503	-1.66951
H	-2.32193	-0.25420	-2.36830
H	-2.88337	-1.92027	-2.12076
H	-3.86703	-0.55894	-1.55819

4a-plus-carbene-triplet

29

C	-3.07911	-0.43037	-0.82139
C	-2.50619	0.70976	-0.29023
N	-1.74229	0.30453	0.78582
H	-1.14217	0.91688	1.31961
H	-3.75437	-0.45261	-1.66725
C	-1.79122	-1.06505	0.93821
C	-2.63163	-1.54548	-0.04701
H	-2.88503	-2.58710	-0.19706
C	1.02434	1.55973	-0.34183
C	1.45365	0.22515	-0.41474
C	0.64578	-0.72673	-1.09872
C	2.66576	-0.21915	0.18992
C	1.03861	-2.05408	-1.16735
H	-0.29243	-0.40452	-1.53681
C	3.03715	-1.55330	0.10728
H	3.29024	0.49494	0.71658
C	2.23187	-2.48009	-0.56871
H	0.40254	-2.76936	-1.68036
H	3.96238	-1.87806	0.57497
H	2.52972	-3.52255	-0.62419
Cl	1.72345	2.91704	0.38891
C	-0.97967	-1.76923	1.97521
H	0.09584	-1.67377	1.77631
H	-1.16628	-1.37824	2.98366
H	-1.22328	-2.83544	1.97867
C	-2.60964	2.14651	-0.68750
H	-1.62778	2.57219	-0.93307
H	-3.24728	2.24365	-1.57071
H	-3.04272	2.76654	0.10855

5a_fromIRC

29

C	2.01791	1.73651	-0.96604
C	2.81814	1.03666	-0.07881
N	2.18819	0.23106	0.81329
H	2.77131	-0.27773	1.47336
H	2.47724	2.39863	-1.68926
C	0.85212	0.02710	0.90315
C	0.63568	1.56724	-0.92317
H	0.01070	2.10262	-1.63022
C	0.02625	0.70181	-0.00665
C	-1.44527	0.51823	-0.01212
C	-2.28956	1.63734	0.02953
C	-2.00394	-0.76585	-0.10253
C	-3.67396	1.47516	-0.00384
H	-1.85996	2.63217	0.10581
C	-3.38911	-0.92168	-0.13981
H	-1.34712	-1.62857	-0.17696
C	-4.22632	0.19515	-0.08684
H	-4.31952	2.34730	0.03816
H	-3.81478	-1.91783	-0.21761
H	-5.30470	0.06913	-0.11328
Cl	1.00294	-3.04125	-0.79573
C	0.38679	-0.90025	1.97749
H	-0.61672	-0.63139	2.30950
H	0.36462	-1.91785	1.56919
H	1.06641	-0.87813	2.83446
C	4.31126	1.10001	-0.02779
H	4.74665	0.12154	-0.25991
H	4.68218	1.82487	-0.75347
H	4.65634	1.39455	0.96927

9. Coordinates for compounds on the energy pathway of product 5f/5f’

2Ph-5Me-endoCl-C23_ring_opening_TS

36

C	-2.67089	-0.82715	1.31262
C	-3.20642	0.02768	0.40140
N	-2.23457	0.76758	-0.26776
H	-2.43241	1.75093	-0.41485
H	-3.27160	-1.39516	2.01269
C	-0.91342	0.44971	0.06104

C	-1.23155	-0.91277	1.29664
H	-0.68260	-1.17975	2.19620
C	-0.51526	-0.96826	0.01290
C	0.94342	-1.31886	0.00590
C	1.46294	-2.22459	0.93527
C	1.79155	-0.78407	-0.97351
C	2.81508	-2.57195	0.90521
H	0.81202	-2.66728	1.68233
C	3.13931	-1.13015	-1.00603
H	1.39070	-0.09096	-1.70554
C	3.65694	-2.02247	-0.06189
H	3.20688	-3.27355	1.63576
H	3.78752	-0.70348	-1.76581
H	4.70936	-2.28996	-0.08464
Cl	-1.33066	-2.00451	-1.39863
C	-4.64460	0.26946	0.07888
H	-4.90425	-0.12941	-0.90949
H	-5.28537	-0.21479	0.82019
H	-4.87823	1.34207	0.06886
C	0.06079	1.56679	0.09307
C	0.05396	2.53047	-0.92778
C	0.99955	1.68137	1.12738
C	0.97071	3.58197	-0.91376
H	-0.65255	2.44472	-1.74859
C	1.91938	2.72781	1.13487
H	1.00421	0.94573	1.92486
C	1.90827	3.68287	0.11540
H	0.95639	4.31605	-1.71407
H	2.64079	2.80169	1.94340
H	2.62380	4.49968	0.12409

2Ph-5Me-endoCl-C23cyclopropane

36

C	-2.67957	-0.69188	1.31781
C	-3.24004	0.10107	0.38190
N	-2.26737	0.74962	-0.40589
H	-2.41887	1.74312	-0.55426
H	-3.21864	-1.33553	2.00029
C	-0.95640	0.42467	0.11724
C	-1.20231	-0.61903	1.23949
H	-0.60367	-0.68519	2.14505
C	-0.51002	-1.02023	-0.04894
C	0.94960	-1.34372	-0.02627
C	1.49306	-2.11046	1.00911

C	1.78706	-0.89681	-1.05764
C	2.85513	-2.41761	1.02295
H	0.84850	-2.46797	1.80660
C	3.14569	-1.20283	-1.04651
H	1.36824	-0.30025	-1.86177
C	3.68404	-1.96258	-0.00329
H	3.26617	-3.01202	1.83377
H	3.78656	-0.84561	-1.84743
H	4.74444	-2.19759	0.00796
Cl	-1.39417	-2.06028	-1.24750
C	-4.68204	0.30642	0.05394
H	-4.91674	-0.08604	-0.94287
H	-5.31956	-0.19690	0.78497
H	-4.93769	1.37406	0.04972
C	0.04864	1.52433	0.11298
C	0.18330	2.32991	-1.02735
C	0.88346	1.75771	1.21056
C	1.13403	3.34925	-1.06601
H	-0.44858	2.14715	-1.89204
C	1.84023	2.77207	1.16918
H	0.78808	1.14167	2.09882
C	1.96809	3.57177	0.03197
H	1.22798	3.96396	-1.95654
H	2.48255	2.94003	2.02885
H	2.71215	4.36228	0.00094

2Ph-5Me-endoCl-C2_cyclopropanation_TS

36

C	-2.42890	-0.21946	1.57560
C	-3.04504	0.61458	0.63163
N	-2.06448	1.27633	-0.03227
H	-2.22393	1.91027	-0.80251
H	-2.95202	-0.88540	2.24746
C	-0.79528	0.83892	0.36995
C	-1.04900	-0.06035	1.44222
H	-0.27965	-0.56953	2.00341
C	-0.34478	-0.67696	-1.11137
C	0.67224	-1.56492	-0.49946
C	0.48496	-2.84813	0.05497
C	1.98573	-1.04797	-0.54179
C	1.56081	-3.57689	0.54945
H	-0.51628	-3.26324	0.10182
C	3.06214	-1.77377	-0.03499
H	2.14382	-0.07306	-0.98904

C	2.85410	-3.04101	0.51237
H	1.39566	-4.56413	0.97247
H	4.06351	-1.35455	-0.07624
H	3.69174	-3.61274	0.90166
Cl	-1.91680	-1.56030	-1.36443
C	-4.48788	0.79907	0.30767
H	-4.76841	1.85864	0.32715
H	-4.71686	0.41471	-0.69349
H	-5.10860	0.26393	1.02962
C	0.39165	1.69369	0.17765
C	0.59928	2.36948	-1.03467
C	1.35287	1.81020	1.19007
C	1.73687	3.14921	-1.22462
H	-0.11300	2.24594	-1.84411
C	2.50306	2.57589	0.99180
H	1.20029	1.30091	2.13571
C	2.69790	3.25087	-0.21332
H	1.88370	3.66554	-2.16898
H	3.24225	2.64847	1.78436
H	3.59042	3.85037	-0.36692

2Ph-5Me-endoCl-C3_cyclopropanation_TS

36

C	-2.90108	-0.43832	1.36916
C	-3.37099	0.43882	0.42942
N	-2.28383	1.17107	-0.03598
H	-2.34903	1.89938	-0.73237
H	-3.49453	-1.16730	1.90258
C	-1.12931	0.79125	0.58914
C	-1.46938	-0.29953	1.43175
H	-0.83999	-0.66944	2.22570
C	-0.71333	-1.88241	0.08049
C	0.72622	-1.60986	-0.05081
C	1.48071	-1.97565	1.08743
C	1.42475	-1.11343	-1.17115
C	2.86608	-1.85871	1.10558
H	0.95066	-2.36865	1.95069
C	2.81096	-1.00378	-1.15647
H	0.86882	-0.81591	-2.05239
C	3.53679	-1.37237	-0.02029
H	3.42431	-2.14893	1.99099
H	3.32997	-0.61787	-2.02914
H	4.61925	-1.28215	-0.01302
Cl	-1.58393	-1.59095	-1.50308

C	-4.74227	0.66956	-0.10852
H	-4.79826	0.42498	-1.17644
H	-5.46178	0.04067	0.42133
H	-5.05176	1.71571	0.00629
C	0.15464	1.41040	0.32425
C	1.20028	1.30298	1.25937
C	0.40124	2.08988	-0.88479
C	2.44996	1.84954	0.99058
H	1.02596	0.79166	2.19917
C	1.64959	2.64775	-1.14339
H	-0.37414	2.15295	-1.64253
C	2.68112	2.52584	-0.20953
H	3.24762	1.74593	1.71988
H	1.82252	3.16306	-2.08362
H	3.65793	2.95284	-0.41639

2Ph-5Me-endoCl-C45_cyclopropane

36

C	0.89785	1.40229	0.48597
C	1.65439	0.27835	0.46099
N	0.87781	-0.87398	0.70568
H	1.27363	-1.53263	1.36997
H	1.24084	2.39905	0.24412
C	-0.48013	-0.47304	0.99999
C	-0.51011	1.06412	0.78302
H	-1.12684	1.73879	1.37243
C	-1.25708	0.12208	-0.15119
C	-2.75236	0.10971	-0.13911
C	-3.47724	1.30318	-0.05588
C	-3.44622	-1.10376	-0.24894
C	-4.87315	1.28634	-0.07191
H	-2.94685	2.24770	0.01864
C	-4.83926	-1.12276	-0.26566
H	-2.88621	-2.03142	-0.32324
C	-5.55660	0.07386	-0.17528
H	-5.42472	2.21966	-0.00584
H	-5.36594	-2.06916	-0.34706
H	-6.64259	0.05960	-0.18604
Cl	-0.62810	-0.12913	-1.84009
C	-1.15544	-1.22043	2.12069
H	-2.15209	-0.82063	2.31443
H	-1.25828	-2.28443	1.87734
H	-0.56352	-1.13112	3.03840
C	3.08228	0.13156	0.16105

C	3.59578	-1.09773	-0.28419
C	3.96563	1.21327	0.32298
C	4.95504	-1.23990	-0.56307
H	2.91932	-1.93367	-0.43011
C	5.32036	1.07035	0.03778
H	3.58629	2.16212	0.68997
C	5.82216	-0.15774	-0.40533
H	5.33483	-2.19672	-0.90992
H	5.98945	1.91581	0.16999
H	6.88048	-0.26844	-0.62271

2Ph-5Me-endoCl-C45_ring_opening_TS

36

C	0.88392	-1.39248	-0.63109
C	1.62930	-0.26048	-0.46793
N	0.87125	0.89674	-0.66483
H	1.31006	1.63942	-1.19754
H	1.30305	-2.39067	-0.62174
C	-0.48711	0.69540	-0.89568
C	-0.52780	-1.17190	-0.79639
H	-1.13811	-1.89352	-1.33210
C	-1.21694	-0.17081	0.03774
C	-2.71728	-0.14471	0.04721
C	-3.45871	-1.31983	-0.11078
C	-3.39438	1.05999	0.28515
C	-4.85348	-1.28942	-0.05704
H	-2.95040	-2.26556	-0.26670
C	-4.78543	1.09247	0.33832
H	-2.82436	1.97093	0.43909
C	-5.52021	-0.08389	0.16298
H	-5.41647	-2.20938	-0.18399
H	-5.29655	2.03349	0.51889
H	-6.60523	-0.05985	0.20350
Cl	-0.66299	-0.05009	1.88823
C	-1.14603	1.62348	-1.87787
H	-2.14336	1.27308	-2.14351
H	-1.24616	2.63294	-1.45769
H	-0.54724	1.69347	-2.79155
C	3.05660	-0.13147	-0.16572
C	3.56826	1.06123	0.37408
C	3.94844	-1.18946	-0.42011
C	4.92999	1.19345	0.64580
H	2.89002	1.87717	0.60335
C	5.30509	-1.05663	-0.14105

H	3.57671	-2.11236	-0.85435
C	5.80461	0.13647	0.39142
H	5.30458	2.12256	1.06593
H	5.97801	-1.88407	-0.34740
H	6.86442	0.23826	0.60545

2Ph-5Me-endoCl-C4_cyclopropanation_TS

36

C	-0.94892	-1.56651	-0.68335
C	-1.64612	-0.76244	0.18484
N	-0.76377	-0.40978	1.21184
H	-1.01445	0.14730	2.01667
H	-1.32522	-1.96640	-1.61367
C	0.45694	-0.97029	1.02264
C	0.41216	-1.63969	-0.22312
H	1.14596	-2.36440	-0.53988
C	1.44007	-0.03268	-1.34824
C	2.72561	0.17702	-0.65587
C	3.64348	-0.89054	-0.79629
C	3.16444	1.33432	0.02119
C	4.92554	-0.81615	-0.26676
H	3.32601	-1.77664	-1.33843
C	4.45963	1.41899	0.52910
H	2.48496	2.17129	0.13707
C	5.34088	0.34388	0.39840
H	5.60907	-1.65238	-0.38176
H	4.77954	2.32435	1.03743
H	6.34690	0.41030	0.80224
Cl	0.35239	1.41729	-1.16533
C	-3.01876	-0.26591	0.14108
C	-3.39126	0.91986	0.79781
C	-3.99907	-0.97154	-0.57948
C	-4.70579	1.38033	0.74116
H	-2.64689	1.50443	1.33059
C	-5.30839	-0.50336	-0.64233
H	-3.73033	-1.89842	-1.07689
C	-5.67006	0.67284	0.02104
H	-4.97314	2.30016	1.25313
H	-6.05174	-1.06330	-1.20245
H	-6.69318	1.03387	-0.02423
C	1.55024	-0.83155	2.01976
H	1.92128	0.20173	2.05860
H	1.20172	-1.10297	3.02261
H	2.39160	-1.47378	1.75718

2Ph-5Me-endoCl-C5_cyclopropanation_TS

36

C	0.56799	-0.05043	-1.55366
C	1.50896	0.48708	-0.66596
N	0.91144	1.56271	-0.05686
H	1.35381	2.15411	0.63149
H	0.71516	-0.93259	-2.16029
C	-0.40196	1.69949	-0.45765
C	-0.60743	0.70454	-1.43563
H	-1.53631	0.55231	-1.96573
C	-1.52267	0.40760	1.18009
C	-2.68715	-0.23244	0.53982
C	-2.80076	-1.55075	0.04762
C	-3.82064	0.60541	0.46201
C	-3.99230	-2.00243	-0.50483
H	-1.93954	-2.20865	0.09152
C	-5.01569	0.15375	-0.09395
H	-3.74349	1.61108	0.86288
C	-5.10347	-1.15180	-0.58056
H	-4.06246	-3.01803	-0.88412
H	-5.87673	0.81381	-0.14194
H	-6.03270	-1.51060	-1.01367
Cl	-0.26645	-0.81553	1.59391
C	-1.15445	2.96843	-0.22130
H	-2.21161	2.81949	-0.45127
H	-1.07486	3.28755	0.82129
H	-0.77673	3.77253	-0.86606
C	2.86301	0.04874	-0.34987
C	3.48048	0.38826	0.86774
C	3.58067	-0.73686	-1.27069
C	4.77680	-0.03727	1.14929
H	2.93619	0.96054	1.61305
C	4.87260	-1.16688	-0.98219
H	3.12476	-0.99448	-2.22163
C	5.47927	-0.81669	0.22746
H	5.23478	0.23208	2.09657
H	5.41115	-1.77056	-1.70696
H	6.48850	-1.15002	0.44987

2Ph-5Me-exoCl-C23_cyclopropane

36

C	0.80924	2.02290	-1.26990
C	0.73576	2.38015	0.03009
N	-0.05355	1.48520	0.77937
H	-0.71856	1.90705	1.42126
H	1.41100	2.51635	-2.02140
C	-0.63423	0.51232	-0.13635
C	-0.00802	0.80746	-1.51060
H	-0.54517	0.66037	-2.44395
C	0.38454	-0.44211	-0.73318
C	1.73516	-0.65398	-0.14588
C	2.88272	-0.48074	-0.92732
C	1.86626	-1.05148	1.18911
C	4.14665	-0.70789	-0.38343
H	2.77748	-0.16471	-1.96087
C	3.12922	-1.27905	1.73588
H	0.97508	-1.17821	1.79605
C	4.27151	-1.10688	0.95007
H	5.03264	-0.57286	-0.99722
H	3.22264	-1.58750	2.77323
H	5.25566	-1.28169	1.37550
Cl	-0.32212	-1.98037	-1.39701
C	1.41773	3.50243	0.74051
H	2.14458	3.11983	1.46722
H	1.94148	4.14748	0.03079
H	0.69583	4.11458	1.29662
C	-2.04713	0.12684	0.13684
C	-3.07907	0.47920	-0.73789
C	-2.35487	-0.56770	1.31468
C	-4.40010	0.13461	-0.44682
H	-2.84445	1.02221	-1.64826
C	-3.67463	-0.90614	1.61040
H	-1.55527	-0.84624	1.99564
C	-4.70044	-0.55742	0.72789
H	-5.19364	0.40982	-1.13555
H	-3.90200	-1.44658	2.52474
H	-5.72832	-0.82495	0.95515

2Ph-5Me-exoCl-C23_ring_opening_TS

36

C	0.99434	2.01453	-1.42844
C	0.83490	2.38214	-0.11494
N	-0.08830	1.60085	0.55934
H	-0.67467	2.05753	1.24900
H	1.50717	2.65781	-2.13520

C	-0.62213	0.51217	-0.11632
C	0.37073	0.79700	-1.82113
H	0.04922	0.59045	-2.83678
C	0.40503	-0.31064	-0.82013
C	1.67992	-0.65093	-0.08697
C	2.91440	-0.56980	-0.73525
C	1.62487	-1.07389	1.24415
C	4.08578	-0.91451	-0.06082
H	2.95128	-0.22683	-1.76488
C	2.79470	-1.41701	1.92172
H	0.66305	-1.13287	1.74600
C	4.02811	-1.33743	1.26980
H	5.04290	-0.84909	-0.57052
H	2.74515	-1.74360	2.95658
H	4.94044	-1.60119	1.79718
Cl	-0.28921	-1.86240	-1.51900
C	1.46861	3.52877	0.60434
H	2.25060	3.19268	1.29715
H	1.92890	4.21398	-0.11278
H	0.73254	4.09168	1.19359
C	-2.02238	0.14139	0.16748
C	-2.95469	-0.08545	-0.85635
C	-2.44913	0.03429	1.50282
C	-4.27088	-0.42948	-0.55341
H	-2.64092	0.02051	-1.88904
C	-3.77094	-0.29457	1.80309
H	-1.73844	0.19169	2.30978
C	-4.68623	-0.53275	0.77642
H	-4.97783	-0.60255	-1.35983
H	-4.08036	-0.37534	2.84120
H	-5.71444	-0.79298	1.00981

2Ph-5Me-exoCl-C2_cyclopropanation_TS

36

C	1.05313	1.25370	-1.67836
C	1.01899	2.08971	-0.55542
N	-0.07676	1.74593	0.17412
H	-0.35613	2.19241	1.03639
H	1.82429	1.26494	-2.43550
C	-0.72890	0.63785	-0.37715
C	-0.03683	0.38437	-1.59374
H	-0.28919	-0.40895	-2.28151
C	0.17912	-1.04379	0.66016
C	1.62846	-0.92849	0.48100

C	2.47494	-1.68462	-0.36194
C	2.22499	0.05969	1.29505
C	3.84456	-1.46376	-0.38028
H	2.03973	-2.44326	-1.00292
C	3.60153	0.28878	1.26892
H	1.59090	0.61780	1.97467
C	4.41459	-0.47091	0.43058
H	4.47870	-2.05765	-1.03275
H	4.03472	1.05315	1.90737
H	5.48710	-0.30054	0.40799
Cl	-0.48546	-2.51575	-0.24737
C	1.92349	3.20925	-0.16484
H	1.47564	4.18063	-0.41011
H	2.13448	3.20061	0.90870
H	2.87151	3.12823	-0.70144
C	-2.14887	0.38537	-0.05786
C	-2.60081	0.42464	1.26948
C	-3.06723	0.11315	-1.07982
C	-3.94553	0.21455	1.56455
H	-1.88513	0.58122	2.07023
C	-4.41121	-0.11657	-0.78152
H	-2.72892	0.09111	-2.11085
C	-4.85636	-0.05991	0.53987
H	-4.28187	0.24984	2.59688
H	-5.11059	-0.33030	-1.58473
H	-5.90354	-0.23117	0.77188

2Ph-5Me-exoCl-C3_cyclopropanation_TS

36

C	-1.53124	2.39243	0.60274
C	-1.87318	1.97077	-0.65240
N	-0.77905	1.28246	-1.17213
H	-0.75670	0.87093	-2.09402
H	-2.17597	2.93142	1.28231
C	0.25848	1.26603	-0.28645
C	-0.21992	1.87511	0.90920
H	0.42253	2.23771	1.69748
C	-0.57517	0.03034	1.94581
C	-1.11749	-0.91929	0.95848
C	-2.48634	-0.72371	0.66452
C	-0.45464	-1.99592	0.32895
C	-3.15590	-1.54901	-0.23680
H	-3.01275	0.07579	1.17231
C	-1.12795	-2.82602	-0.56069

H	0.59382	-2.16878	0.54083
C	-2.47765	-2.60077	-0.85616
H	-4.20852	-1.37977	-0.44551
H	-0.59976	-3.64940	-1.03382
H	-2.99779	-3.24980	-1.55489
Cl	1.11534	-0.47393	2.47823
C	-3.12461	2.13105	-1.44592
H	-3.56416	1.15610	-1.68793
H	-3.85845	2.70429	-0.87449
H	-2.93892	2.65575	-2.39109
C	1.54373	0.67095	-0.59706
C	2.69843	1.07235	0.09982
C	1.66361	-0.32735	-1.58387
C	3.93394	0.49983	-0.18710
H	2.62302	1.84632	0.85551
C	2.90179	-0.89668	-1.86752
H	0.78333	-0.68617	-2.10695
C	4.04169	-0.48517	-1.17216
H	4.81554	0.82540	0.35696
H	2.97524	-1.66952	-2.62683
H	5.00665	-0.93048	-1.39517

2Ph-5Me-exoCl-C45_cyclopropane

36

C	-0.19610	-1.16572	-1.39845
C	-0.91983	-1.00524	-0.26305
N	-0.13939	-1.22666	0.89150
H	-0.57081	-1.80968	1.60315
H	-0.54529	-0.95900	-2.40083
C	1.18732	-1.65346	0.47946
C	1.19331	-1.55053	-1.05971
H	1.76905	-2.22377	-1.68955
C	1.97487	-0.55704	-0.20315
C	1.61056	0.88279	-0.09266
C	1.41302	1.65529	-1.24208
C	1.47941	1.47748	1.16697
C	1.09199	3.00822	-1.13524
H	1.50819	1.18829	-2.21787
C	1.15765	2.83041	1.27665
H	1.62354	0.87264	2.05681
C	0.96244	3.59743	0.12528
H	0.94045	3.60149	-2.03247
H	1.05609	3.28506	2.25796
H	0.70884	4.65037	0.20980

Cl	3.78104	-0.79187	-0.24390
C	1.80069	-2.75547	1.30055
H	1.94637	-2.43033	2.33751
H	1.14219	-3.63087	1.30160
H	2.76932	-3.05527	0.89909
C	-2.31396	-0.57758	-0.11130
C	-2.74986	0.01620	1.08478
C	-3.23956	-0.75581	-1.15418
C	-4.07563	0.42357	1.23212
H	-2.03690	0.17682	1.88687
C	-4.56033	-0.34233	-1.00664
H	-2.92050	-1.23510	-2.07473
C	-4.98525	0.24781	0.18816
H	-4.39568	0.88486	2.16208
H	-5.26364	-0.48873	-1.82143
H	-6.01751	0.56542	0.30282

2Ph-5Me-exoCl-C45_ring_opening_TS

36

C	-0.25721	-1.14736	-1.47847
C	-0.95875	-0.93587	-0.31061
N	-0.21995	-1.27289	0.82336
H	-0.71970	-1.72900	1.57875
H	-0.73723	-1.12837	-2.44991
C	1.10679	-1.60505	0.64226
C	1.12590	-1.43264	-1.33093
H	1.70357	-1.98137	-2.06713
C	1.83750	-0.67270	-0.25277
C	1.63257	0.81399	-0.10192
C	1.53401	1.63048	-1.23136
C	1.55532	1.38467	1.17156
C	1.36580	3.00744	-1.09055
H	1.57934	1.17848	-2.21779
C	1.38301	2.76173	1.31551
H	1.62313	0.74707	2.04830
C	1.28816	3.57558	0.18413
H	1.28929	3.63657	-1.97282
H	1.31964	3.19839	2.30812
H	1.15062	4.64743	0.29471
Cl	3.64611	-1.02090	-0.29418
C	1.72218	-2.62688	1.54833
H	0.97608	-3.36379	1.86012
H	2.53245	-3.15700	1.04571
H	2.14757	-2.15915	2.44722

C	-2.34051	-0.48987	-0.13901
C	-2.76979	0.05554	1.08502
C	-3.27909	-0.60226	-1.18289
C	-4.09296	0.45943	1.26335
H	-2.05322	0.19093	1.88940
C	-4.59547	-0.18950	-1.00408
H	-2.97662	-1.03050	-2.13351
C	-5.01319	0.34070	0.22145
H	-4.39977	0.88001	2.21697
H	-5.30284	-0.28897	-1.82272
H	-6.04204	0.66033	0.35796

2Ph-5Me-exoCl-C4_cyclopropanation_TS

36

C	-0.19157	-1.94754	-0.71530
C	-0.91025	-1.18107	0.16582
N	-0.05138	-0.86867	1.22881
H	-0.31837	-0.33345	2.04338
H	-0.54607	-2.31228	-1.66832
C	1.17151	-1.41833	1.04786
C	1.16904	-2.02498	-0.23768
H	1.89441	-2.76500	-0.54239
C	2.19737	-0.46005	-1.21862
C	1.66465	0.81146	-0.69573
C	0.40051	1.16737	-1.21971
C	2.28173	1.71312	0.20011
C	-0.23361	2.34772	-0.83857
H	-0.06390	0.50763	-1.94224
C	1.65358	2.89829	0.56764
H	3.26381	1.47980	0.59589
C	0.38765	3.21538	0.06143
H	-1.20973	2.59078	-1.24732
H	2.14838	3.57893	1.25505
H	-0.10175	4.13909	0.35658
Cl	3.95050	-0.71184	-0.65148
C	2.23944	-1.29614	2.07216
H	1.82807	-1.43226	3.07773
H	3.02170	-2.03723	1.90836
H	2.70235	-0.30024	2.03144
C	-2.28470	-0.68827	0.11536
C	-2.66932	0.47539	0.80328
C	-3.24721	-1.36871	-0.65041
C	-3.98144	0.94000	0.73091
H	-1.93534	1.04371	1.36584

C	-4.55462	-0.89654	-0.72743
H	-2.96724	-2.27801	-1.17337
C	-4.92935	0.25800	-0.03429
H	-4.25944	1.84342	1.26580
H	-5.28556	-1.43607	-1.32265
H	-5.95064	0.62248	-0.09144

2Ph-5Me-exoCl-C5_cyclopropanation_TS

36

C	-0.37133	-0.89648	1.67539
C	0.45028	-1.15718	0.56787
N	-0.34163	-1.74636	-0.38534
H	-0.04479	-1.92973	-1.33298
H	-0.04346	-0.45955	2.60732
C	-1.67617	-1.75027	0.00314
C	-1.67050	-1.28687	1.34150
H	-2.54854	-1.22465	1.96715
C	-2.41874	0.18427	-0.82152
C	-1.31541	1.11326	-0.58226
C	-1.13206	2.01323	0.49073
C	-0.30272	1.04629	-1.56553
C	0.00021	2.81499	0.56327
H	-1.88668	2.06635	1.26767
C	0.82943	1.85155	-1.49811
H	-0.43961	0.35667	-2.39300
C	0.98696	2.73305	-0.42744
H	0.12759	3.49764	1.39859
H	1.59456	1.78011	-2.26501
H	1.87542	3.35391	-0.36029
Cl	-3.90598	0.66374	0.18374
C	-2.66154	-2.65326	-0.67006
H	-2.49602	-3.69874	-0.38070
H	-3.67635	-2.36916	-0.38327
H	-2.58157	-2.57061	-1.75707
C	1.86258	-0.86628	0.36657
C	2.61616	-1.51482	-0.62845
C	2.49414	0.10396	1.16566
C	3.95790	-1.19695	-0.82027
H	2.16015	-2.28476	-1.24413
C	3.83825	0.41285	0.97574
H	1.91774	0.63577	1.91441
C	4.57589	-0.23236	-0.01957
H	4.52478	-1.70906	-1.59218
H	4.30739	1.16899	1.59844

H	5.62260	0.01369	-0.17108

2Ph-6Me-3Ph-pyridine

36

C	0.95792	-3.16287	0.08739
C	2.01175	-2.25757	0.10897
N	1.71017	-0.93928	0.07268
H	2.52264	-0.26656	-0.00647
H	1.16905	-4.22506	0.10799
C	0.45164	-0.43103	0.03381
C	-0.34924	-2.69291	0.05768
H	-1.17363	-3.39817	0.07088
C	-0.63490	-1.32013	0.04676
C	-2.04584	-0.86060	0.08002
C	-2.97402	-1.39628	-0.82366
C	-2.47902	0.07411	1.03254
C	-4.30933	-0.99437	-0.78524
H	-2.64429	-2.11318	-1.57001
C	-3.81383	0.46916	1.07273
H	-1.76977	0.48600	1.74270
C	-4.73190	-0.06062	0.16203
H	-5.01636	-1.40873	-1.49756
H	-4.13836	1.18942	1.81769
H	-5.77120	0.25263	0.19232
Cl	4.20965	0.80948	-0.32820
C	3.45311	-2.65288	0.18063
H	3.82434	-2.53461	1.20558
H	3.56920	-3.69922	-0.10756
H	4.06655	-2.01617	-0.46138
C	0.33109	1.04043	-0.03175
C	1.03825	1.84630	0.87108
C	-0.49005	1.63739	-1.00049
C	0.91383	3.23337	0.81354
H	1.67234	1.38793	1.62194
C	-0.59978	3.02338	-1.06216
H	-1.03377	1.01558	-1.70312
C	0.09680	3.82337	-0.15197
H	1.45622	3.85090	1.52258
H	-1.22956	3.47942	-1.81964
H	0.00261	4.90419	-0.19673

2Ph-6Me-5Ph-pyridine

36

C	0.38192	-1.85312	0.11069
C	1.18805	-0.71757	0.07280
N	0.56870	0.48866	0.00115
H	1.16175	1.34727	-0.15314
H	0.84004	-2.83060	0.18857
C	-0.77442	0.67286	-0.00642
C	-0.99827	-1.71488	0.09623
H	-1.62440	-2.59943	0.14658
C	-1.60932	-0.45244	0.05841
C	-3.08856	-0.34819	0.06043
C	-3.83845	-1.13469	-0.82785
C	-3.76220	0.49084	0.96083
C	-5.23101	-1.07414	-0.82356
H	-3.32615	-1.77978	-1.53563
C	-5.15503	0.54696	0.96617
H	-3.19735	1.08274	1.67342
C	-5.89280	-0.23252	0.07285
H	-5.79784	-1.68123	-1.52292
H	-5.66313	1.19598	1.67295
H	-6.97763	-0.18536	0.07630
Cl	2.12870	3.07098	-0.71137
C	-1.25636	2.08770	-0.11669
H	-2.21882	2.12273	-0.62909
H	-1.39097	2.52540	0.87922
H	-0.52320	2.69933	-0.64690
C	2.65682	-0.76950	0.12686
C	3.38976	0.20269	0.82612
C	3.33657	-1.82776	-0.49944
C	4.77798	0.11514	0.89424
H	2.87661	1.01694	1.32373
C	4.72465	-1.90700	-0.43175
H	2.78025	-2.57121	-1.06103
C	5.44808	-0.93682	0.26643
H	5.33557	0.86775	1.44278
H	5.24139	-2.72214	-0.92847
H	6.53058	-1.00105	0.32093

4f_plus_carbene_singlet_GS

36

C	3.15492	-0.67327	-0.49996
C	1.79432	-1.11576	-0.63294
C	1.25071	-0.83814	-1.91430
C	0.96093	-1.75578	0.32352

C	-0.05328	-1.19182	-2.23411
H	1.88813	-0.33748	-2.63505
C	-0.33825	-2.10479	0.00265
H	1.35423	-1.96542	1.31164
C	-0.85132	-1.81231	-1.27122
H	-0.45914	-0.96414	-3.21433
H	-0.97329	-2.58556	0.73980
H	-1.88201	-2.06115	-1.50192
Cl	3.84008	-1.09970	1.11183
C	-1.80022	0.93283	0.03929
C	-2.72072	0.99760	-1.00422
C	-3.88772	0.29787	-0.60473
C	-3.67161	-0.19121	0.67476
N	-2.41358	0.21074	1.04922
H	-1.97355	-0.05731	1.91667
H	-2.56598	1.50018	-1.94871
H	-4.79387	0.16188	-1.17990
C	-4.54072	-1.00696	1.57402
H	-4.07208	-1.96492	1.83369
H	-4.75974	-0.48574	2.51464
H	-5.49129	-1.22149	1.07888
C	-0.43965	1.43464	0.13902
C	0.17823	2.03110	-0.98003
C	0.31670	1.31812	1.32243
C	1.49695	2.46379	-0.92461
H	-0.37902	2.13232	-1.90525
C	1.63911	1.74604	1.37204
H	-0.12235	0.88442	2.21563
C	2.24583	2.30545	0.24523
H	1.95231	2.90551	-1.80648
H	2.20500	1.62519	2.29075
H	3.28106	2.62916	0.28334

4f_plus_carbene_triplet_GS

36

C	-0.75180	-2.18040	-1.14005
C	-1.05057	-1.72321	0.15174
C	-2.40682	-1.46838	0.50295
C	-0.04158	-1.48645	1.12832
C	-2.72672	-0.98821	1.76338
H	-3.18343	-1.64489	-0.23382
C	-0.38345	-0.99608	2.38041
H	0.99696	-1.66128	0.87290
C	-1.72103	-0.73862	2.70946

H	-3.76614	-0.79971	2.01582
H	0.40194	-0.80091	3.10430
H	-1.97823	-0.35566	3.69197
Cl	0.72207	-2.54865	-1.87802
C	0.16335	1.37293	-0.75429
C	-0.21989	1.45477	-2.08677
C	-1.61573	1.72172	-2.12227
C	-2.06756	1.79399	-0.81481
N	-0.97510	1.60459	-0.00080
H	-1.04313	1.41586	0.99017
H	0.44390	1.36831	-2.93641
H	-2.22878	1.86292	-3.00282
C	-3.43082	2.04973	-0.26232
H	-3.75719	1.24104	0.40195
H	-3.46975	2.98249	0.31522
H	-4.15410	2.13000	-1.07837
C	1.46357	1.11622	-0.14781
C	2.47798	0.47751	-0.88532
C	1.73873	1.47250	1.18549
C	3.71267	0.19514	-0.30724
H	2.28011	0.17704	-1.90900
C	2.97095	1.17855	1.76550
H	0.98614	1.99035	1.77253
C	3.96594	0.53653	1.02471
H	4.47673	-0.30590	-0.89534
H	3.15604	1.45897	2.79880
H	4.92598	0.30698	1.47750

10. Coordinates for compounds on the energy pathway of product 5t/5t'

2Ph-3Ph-6tBu-pyridine

45

C	1.86671	-2.00096	-0.41987
C	2.36808	-0.70707	-0.44112
N	1.45179	0.29005	-0.44853
H	1.79542	1.24407	-0.50460
H	2.54334	-2.84348	-0.43152
C	0.10406	0.15082	-0.39025
C	0.49083	-2.21003	-0.36261
H	0.10921	-3.22444	-0.31186
C	-0.41816	-1.14746	-0.32119
C	-1.86785	-1.39962	-0.14547
C	-2.54438	-2.28299	-0.99656
C	-2.56114	-0.78031	0.90544

C	-3.90493	-2.53065	-0.81128
H	-2.01009	-2.75876	-1.81402
C	-3.91791	-1.03584	1.08994
H	-2.01973	-0.12392	1.58022
C	-4.59385	-1.90615	0.22997
H	-4.42515	-3.20807	-1.48178
H	-4.44824	-0.55909	1.90903
H	-5.65286	-2.09926	0.37370
Cl	0.61500	0.26950	2.98771
C	3.83993	-0.31874	-0.41647
C	-0.67860	1.39822	-0.46763
C	-0.32195	2.50116	0.32278
C	-1.74818	1.50204	-1.37034
C	-1.03133	3.69565	0.20825
H	0.46012	2.39070	1.06728
C	-2.44760	2.70007	-1.48257
H	-2.02064	0.65159	-1.98527
C	-2.09143	3.79804	-0.69419
H	-0.76240	4.54164	0.83308
H	-3.26942	2.77799	-2.18747
H	-2.64329	4.72918	-0.78044
C	4.21263	0.01143	1.04896
H	5.26182	0.32137	1.09372
H	3.59197	0.81890	1.44914
H	4.08037	-0.86423	1.69151
C	4.09952	0.91478	-1.30821
H	5.17498	1.11049	-1.33630
H	3.75420	0.74964	-2.33381
H	3.63131	1.82807	-0.92240
C	4.71152	-1.48362	-0.91708
H	5.75749	-1.16417	-0.93200
H	4.64593	-2.35522	-0.25932
H	4.43241	-1.78722	-1.93135

2Ph-4Ph-6tBu-pyridine

45

C	1.69038	-0.55094	0.06740
C	0.56781	-1.36222	-0.00384
N	-0.64244	-0.75666	-0.13599
H	-1.45389	-1.36027	-0.22619
H	2.65523	-1.01268	0.22725
C	-0.84239	0.58749	-0.19495
C	0.27668	1.39602	-0.12288
H	0.15515	2.46637	-0.19663

C	1.56671	0.84488	0.00428
C	2.75469	1.71332	0.08097
C	2.67507	2.98215	0.68052
C	3.98522	1.28456	-0.44632
C	3.80061	3.79826	0.75488
H	1.74096	3.31709	1.11965
C	5.10570	2.10814	-0.38030
H	4.05753	0.32005	-0.93853
C	5.01731	3.36520	0.22254
H	3.72894	4.77020	1.23282
H	6.04644	1.77067	-0.80386
H	5.89300	4.00467	0.27712
Cl	-6.74475	1.78058	0.27189
C	-2.27758	1.06203	-0.36758
C	0.60261	-2.82950	0.07300
C	1.67528	-3.53024	-0.50184
C	-0.42485	-3.54161	0.71421
C	1.71321	-4.92034	-0.44159
H	2.45995	-2.98892	-1.01990
C	-0.37966	-4.93222	0.77224
H	-1.23740	-3.01550	1.20663
C	0.68670	-5.62364	0.19352
H	2.54014	-5.45489	-0.89786
H	-1.17187	-5.47366	1.27901
H	0.71913	-6.70768	0.23953
C	-2.81248	0.55407	-1.72756
H	-2.82380	-0.53966	-1.78971
H	-3.84208	0.90120	-1.85478
H	-2.20888	0.93785	-2.55614
C	-2.35632	2.59643	-0.34278
H	-1.80031	3.04665	-1.17147
H	-3.40381	2.89470	-0.43901
H	-1.97646	3.00459	0.59945
C	-3.15287	0.50324	0.77795
H	-2.78608	0.83986	1.75300
H	-4.17537	0.86765	0.63412
H	-3.18971	-0.59197	0.78397

2Ph-5Ph-6tBu-pyridine

45

C	1.04653	-1.34473	-0.76648
C	1.78352	-0.24733	-0.35010
N	1.08294	0.84911	0.04364
H	1.63281	1.65217	0.32968

H	1.54959	-2.26091	-1.04560
C	-0.26851	0.99445	0.07206
C	-0.34006	-1.26193	-0.77003
H	-0.92131	-2.12612	-1.06445
C	-1.02408	-0.10062	-0.37469
C	-2.51490	-0.14264	-0.45454
C	-3.18764	0.39705	-1.55759
C	-3.24316	-0.82335	0.53054
C	-4.57705	0.30088	-1.65019
H	-2.62320	0.88146	-2.34813
C	-4.63152	-0.91570	0.43636
H	-2.71589	-1.28446	1.35921
C	-5.30202	-0.34712	-0.64904
H	-5.08902	0.72690	-2.50776
H	-5.18842	-1.43695	1.20928
H	-6.38323	-0.41956	-0.72002
Cl	-0.94197	-3.87826	1.33883
C	-0.79307	2.34247	0.58960
C	3.25076	-0.19334	-0.28823
C	3.90745	0.52414	0.72566
C	4.00918	-0.87307	-1.25511
C	5.29896	0.56570	0.76349
H	3.34159	1.01349	1.51313
C	5.39975	-0.82697	-1.21172
H	3.50975	-1.41249	-2.05311
C	6.04722	-0.10689	-0.20484
H	5.79739	1.11336	1.55680
H	5.97776	-1.34742	-1.96863
H	7.13176	-0.07333	-0.17275
C	-1.84401	2.11192	1.69829
H	-1.42981	1.50978	2.51360
H	-2.13983	3.08345	2.10652
H	-2.73860	1.61688	1.32260
C	-1.41744	3.13954	-0.57875
H	-0.70113	3.26880	-1.39677
H	-2.31078	2.65177	-0.96868
H	-1.70409	4.13168	-0.21533
C	0.33703	3.20134	1.19656
H	0.83429	2.70347	2.03665
H	1.08602	3.50921	0.45617
H	-0.10432	4.12262	1.58433

2Ph-5tBu-234azabenzvalene-C23insertion_ring-opening_TS

45

C	-1.32971	-1.07087	0.52643
C	-2.35788	-0.17523	0.14909
N	-1.78360	0.98678	-0.23351
H	-2.29041	1.70365	-0.74015
H	-1.55655	-1.95223	1.11723
C	-0.46145	1.10501	0.06146
C	0.02035	-0.01018	0.93721
H	0.62975	0.11824	1.83660
C	0.06210	-1.03885	-0.00208
C	0.97963	-2.02966	-0.43744
C	2.36101	-1.88134	-0.16871
C	0.52514	-3.14395	-1.17990
C	3.25849	-2.83170	-0.63795
H	2.69912	-1.03491	0.42290
C	1.43110	-4.09603	-1.62582
H	-0.53569	-3.24474	-1.38840
C	2.79791	-3.93838	-1.36172
H	4.31883	-2.72148	-0.43271
H	1.08224	-4.95736	-2.18675
H	3.50461	-4.68057	-1.72061
Cl	2.99396	0.23029	2.90193
C	-3.84579	-0.35369	0.32503
C	-4.25129	-1.76582	-0.14168
H	-4.01750	-1.91100	-1.20130
H	-3.73890	-2.54423	0.43284
H	-5.32835	-1.90429	-0.00298
C	-4.18971	-0.18101	1.82539
H	-3.90484	0.81448	2.18049
H	-5.26828	-0.30608	1.97120
H	-3.67239	-0.92667	2.43729
C	-4.61452	0.69753	-0.49695
H	-5.68851	0.52142	-0.38854
H	-4.41914	1.71754	-0.14670
H	-4.37137	0.63317	-1.56332
C	0.38778	2.17504	-0.41066
C	1.66974	2.34031	0.15727
C	-0.03307	3.07072	-1.41747
C	2.48932	3.38343	-0.26225
H	2.02442	1.66086	0.92913
C	0.79229	4.11313	-1.82118
H	-0.99771	2.94961	-1.90058
C	2.05729	4.27510	-1.24698
H	3.47185	3.49944	0.18527
H	0.45254	4.79495	-2.59465
H	2.70179	5.08645	-1.57101

2Ph-5tBu-234azabenzvalene-forming_TS

45

C	-0.81705	-0.12371	-1.60316
C	-1.60734	-0.36405	-0.50685
N	-0.92587	-1.03810	0.50478
H	-1.40686	-1.85499	0.87022
H	-1.07098	0.52556	-2.43001
C	0.44646	-1.23723	0.04167
C	0.52495	-0.76034	-1.46099
H	1.09024	-1.18111	-2.28600
C	1.06543	0.02704	-0.37269
C	1.44632	1.29725	0.08427
C	1.85729	2.30510	-0.82460
C	1.36472	1.58480	1.46887
C	2.16531	3.56804	-0.35368
H	1.92965	2.06169	-1.87768
C	1.69130	2.85127	1.93154
H	1.03803	0.80868	2.15097
C	2.08827	3.83888	1.02257
H	2.47632	4.34726	-1.04144
H	1.63313	3.07806	2.99073
H	2.34101	4.83029	1.38612
Cl	4.08401	-0.26332	-1.33035
C	1.25596	-2.41928	0.64736
C	-3.01375	-0.01770	-0.31493
C	-3.55762	0.02238	0.98100
C	-3.84277	0.26592	-1.41525
C	-4.90170	0.33571	1.17027
H	-2.91578	-0.16763	1.83485
C	-5.18214	0.58528	-1.22026
H	-3.43816	0.21637	-2.42101
C	-5.71633	0.61880	0.07216
H	-5.31035	0.36786	2.17559
H	-5.81418	0.80044	-2.07633
H	-6.76313	0.86612	0.22067
C	2.19444	-1.89774	1.74947
H	2.74070	-2.73455	2.19820
H	2.92332	-1.19292	1.33972
H	1.63004	-1.40225	2.54756
C	2.07037	-3.11441	-0.45512
H	2.65071	-3.93158	-0.01231
H	1.40813	-3.54268	-1.21554
H	2.76669	-2.41997	-0.93255
C	0.29228	-3.45491	1.25890

H	0.87683	-4.30921	1.61378
H	-0.25322	-3.05735	2.12224
H	-0.42562	-3.83136	0.52131

2Ph-5tBu-234azabenzvalene

45

C	1.49636	0.73918	0.91069
C	2.43943	-0.16705	0.22773
N	1.69730	-1.10081	-0.29420
H	2.01940	-1.90415	-0.82081
H	1.80724	1.62148	1.45270
C	0.28862	-0.81095	0.03594
C	0.27346	-0.07866	1.35398
H	-0.30005	-0.14421	2.27593
C	0.15493	0.71208	0.14898
C	-0.76668	1.71772	-0.38521
C	-2.01228	1.91931	0.22680
C	-0.42152	2.44887	-1.53087
C	-2.90601	2.84445	-0.31263
H	-2.26707	1.35396	1.12026
C	-1.31495	3.37772	-2.05977
H	0.54595	2.28687	-1.99737
C	-2.55915	3.57360	-1.45251
H	-3.87159	2.99959	0.15966
H	-1.04463	3.94646	-2.94442
H	-3.25604	4.29601	-1.86747
Cl	-2.43931	-0.13320	3.56380
C	3.92696	-0.04472	0.12786
C	4.22749	1.29981	-0.58085
H	3.81585	1.31164	-1.59487
H	3.81702	2.14958	-0.02777
H	5.31255	1.42552	-0.64578
C	4.49932	-0.01865	1.56540
H	4.28885	-0.95539	2.09043
H	5.58440	0.11001	1.50788
H	4.08793	0.80908	2.15013
C	4.53921	-1.20872	-0.66527
H	5.62411	-1.08010	-0.71114
H	4.34005	-2.17328	-0.18570
H	4.16590	-1.23737	-1.69470
C	-0.78113	-1.68404	-0.49268
C	-1.94164	-1.90252	0.26091
C	-0.66350	-2.24832	-1.76988
C	-2.96995	-2.68560	-0.26339

H	-2.04538	-1.46090	1.24950
C	-1.68920	-3.04203	-2.28117
H	0.21856	-2.05833	-2.37529
C	-2.84542	-3.26147	-1.52928
H	-3.86789	-2.85058	0.32471
H	-1.58803	-3.47975	-3.26961
H	-3.64605	-3.87590	-1.93021

2Ph-5tBu-345azabenzvalene-C45insertion_ring-opening_TS

45

C	1.29373	0.82352	0.68829
C	1.80459	-0.39753	0.18180
N	0.74180	-1.22788	-0.04180
H	0.79730	-2.02566	-0.66674
H	1.94543	1.51755	1.20801
C	-0.42440	-0.82150	0.48950
C	-0.25058	0.36673	1.37435
H	-0.65429	0.44746	2.38511
C	-0.06860	1.35932	0.40948
C	-0.57910	2.65415	0.12749
C	-1.84632	3.04933	0.61569
C	0.16550	3.54758	-0.67617
C	-2.34685	4.30724	0.30941
H	-2.42416	2.35940	1.22300
C	-0.33503	4.81139	-0.95772
H	1.13260	3.23536	-1.05799
C	-1.59175	5.19153	-0.47109
H	-3.32170	4.60704	0.68053
H	0.24373	5.50081	-1.56410
H	-1.98542	6.17605	-0.70416
Cl	-5.41963	-3.37919	-0.58806
C	-1.75496	-1.46315	0.17685
C	3.18031	-0.81628	0.03231
C	4.19859	0.14902	-0.10477
C	3.52635	-2.18212	-0.00693
C	5.52056	-0.24381	-0.27750
H	3.94501	1.20445	-0.09328
C	4.84936	-2.56707	-0.19890
H	2.76451	-2.94156	0.14039
C	5.85100	-1.60180	-0.33166
H	6.29502	0.50964	-0.38205
H	5.10166	-3.62249	-0.22766
H	6.88379	-1.90521	-0.47209
C	-2.35618	-0.81882	-1.09607

H	-1.69011	-0.95095	-1.95497
H	-3.31408	-1.30212	-1.31255
H	-2.52486	0.25327	-0.95456
C	-2.72040	-1.25713	1.36011
H	-2.91690	-0.19505	1.54406
H	-3.67079	-1.74211	1.11934
H	-2.32394	-1.69976	2.28042
C	-1.56879	-2.97705	-0.06379
H	-1.09075	-3.46325	0.79306
H	-2.55813	-3.41880	-0.21613
H	-0.97511	-3.18386	-0.96216

2Ph-5tBu-345azabenzvalene-forming_TS

45

C	-1.67501	-0.09416	-1.50287
C	-2.15663	-0.78362	-0.42292
N	-1.12984	-1.25551	0.38160
H	-1.16419	-2.22898	0.67236
H	-2.25867	0.51278	-2.18013
C	0.12073	-0.80057	-0.20283
C	-0.17598	-0.17397	-1.60925
H	0.36363	-0.24341	-2.54810
C	0.17944	0.64831	-0.48524
C	0.28552	1.92073	0.09397
C	0.26671	3.08836	-0.70897
C	0.35999	2.03174	1.50361
C	0.30793	4.33327	-0.10730
H	0.22640	2.98303	-1.78667
C	0.41867	3.28527	2.09483
H	0.36995	1.13048	2.10618
C	0.38899	4.43108	1.29123
H	0.28839	5.23356	-0.71225
H	0.48057	3.37836	3.17377
H	0.43055	5.41149	1.75583
Cl	2.94970	1.06739	-1.85682
C	-3.59657	-1.02380	-0.02639
C	1.33985	-1.56532	0.19495
C	2.11251	-2.23615	-0.75587
C	1.66965	-1.66074	1.55244
C	3.21365	-2.99094	-0.35302
H	1.85593	-2.15810	-1.80628
C	2.76963	-2.41793	1.95406
H	1.06098	-1.15057	2.29323
C	3.54458	-3.08218	1.00081

H	3.81211	-3.50846	-1.09670
H	3.02102	-2.48662	3.00815
H	4.40353	-3.66928	1.31220
C	-3.87952	-0.22528	1.26695
H	-3.73046	0.84717	1.10267
H	-4.91701	-0.38579	1.57965
H	-3.21749	-0.54365	2.07757
C	-4.54890	-0.55909	-1.13888
H	-4.35661	-1.09072	-2.07666
H	-5.58350	-0.75556	-0.84067
H	-4.45307	0.51586	-1.32400
C	-3.81488	-2.52940	0.23774
H	-4.86472	-2.70479	0.49322
H	-3.57392	-3.12609	-0.64837
H	-3.21170	-2.89147	1.07737

2Ph-5tBu-345azabenzvalene

45

C	-1.31125	0.73964	-1.11074
C	-1.90117	-0.38988	-0.36044
N	-0.87630	-1.13923	-0.03268
H	-0.90199	-2.00254	0.49344
H	-1.88773	1.55969	-1.51412
C	0.35401	-0.50133	-0.54605
C	-0.03275	0.26380	-1.79038
H	0.33354	0.35416	-2.80401
C	0.11273	0.99949	-0.54951
C	0.78160	2.21144	-0.06209
C	1.38472	3.10520	-0.95681
C	0.81353	2.47874	1.31431
C	2.01291	4.25461	-0.47967
H	1.36429	2.89106	-2.02114
C	1.44791	3.62487	1.78903
H	0.34169	1.78520	2.00388
C	2.04689	4.51428	0.89261
H	2.48015	4.94355	-1.17674
H	1.47466	3.82532	2.85574
H	2.54112	5.40726	1.26346
Cl	5.31189	-3.34964	0.46417
C	1.67492	-1.20409	-0.29302
C	-3.28777	-0.62737	-0.03194
C	-4.27702	0.22989	-0.55180
C	-3.66957	-1.70284	0.79682
C	-5.61705	0.01350	-0.25091

H	-3.99993	1.05713	-1.19433
C	-5.00889	-1.91109	1.09402
H	-2.92462	-2.37003	1.21734
C	-5.98421	-1.05442	0.57070
H	-6.37378	0.67643	-0.65658
H	-5.29615	-2.73805	1.73461
H	-7.03068	-1.22082	0.80612
C	1.94107	-1.26950	1.22331
H	1.15361	-1.81587	1.75591
H	2.88856	-1.78814	1.39804
H	2.00530	-0.26379	1.65167
C	2.81757	-0.42948	-0.97337
H	2.91059	0.58627	-0.57878
H	3.75524	-0.96054	-0.78482
H	2.67144	-0.36750	-2.05734
C	1.61693	-2.62634	-0.88441
H	1.40808	-2.59517	-1.95939
H	2.58189	-3.11686	-0.72538
H	0.84601	-3.24306	-0.40766

2Ph-5tBu-endoCl-C23_ring_opening_TS

45

C	1.89250	-0.97614	-1.32486
C	2.47629	-0.14607	-0.41857
N	1.53585	0.64145	0.24779
H	1.77082	1.61777	0.37799
H	2.45079	-1.57841	-2.02876
C	0.20080	0.38528	-0.07336
C	0.45062	-0.99269	-1.30736
H	-0.10911	-1.23890	-2.20618
C	-0.26658	-1.01008	-0.02430
C	-1.74113	-1.28702	-0.01687
C	-2.30692	-2.16345	-0.94730
C	-2.56060	-0.71066	0.96340
C	-3.67535	-2.44001	-0.91793
H	-1.68005	-2.63865	-1.69493
C	-3.92455	-0.98606	0.99521
H	-2.12442	-0.04045	1.69656
C	-4.48780	-1.84871	0.04960
H	-4.10285	-3.11926	-1.64959
H	-4.54978	-0.52729	1.75555
H	-5.55276	-2.06089	0.07161
Cl	0.49273	-2.08680	1.39148
C	3.94277	0.02874	-0.08260

C	-0.71603	1.55008	-0.10136
C	-0.65385	2.51222	0.91916
C	-1.65314	1.71266	-1.13069
C	-1.51567	3.60927	0.91001
H	0.05205	2.39006	1.73592
C	-2.51824	2.80479	-1.13319
H	-1.69968	0.97821	-1.92790
C	-2.45252	3.75799	-0.11396
H	-1.45937	4.34163	1.71002
H	-3.23935	2.91575	-1.93772
H	-3.12538	4.61032	-0.11884
C	4.19495	-0.47622	1.35500
H	5.25041	-0.34685	1.62141
H	3.58615	0.07700	2.07721
H	3.94228	-1.53787	1.44232
C	4.33847	1.51906	-0.17434
H	5.41059	1.63175	0.01918
H	4.12563	1.92375	-1.16985
H	3.81471	2.13225	0.56839
C	4.80999	-0.77738	-1.06185
H	5.86819	-0.64771	-0.81206
H	4.57888	-1.84640	-1.00861
H	4.66077	-0.44393	-2.09449

2Ph-5tBu-endoCl-C34_ring_opening_TS

45

Cl	0.90883	-0.03966	2.05649
C	1.13766	-0.28100	0.25334
C	2.40240	-1.02749	-0.02433
C	3.61082	-0.33798	-0.17960
C	4.79600	-1.03520	-0.41280
C	4.78216	-2.43000	-0.49188
C	3.58022	-3.12422	-0.33446
C	2.39589	-2.42516	-0.10092
C	-0.06523	-0.58278	-0.57492
H	0.02797	-1.39775	-1.28884
C	-1.38583	-0.15528	-0.13923
C	-2.59575	-0.92525	-0.13621
C	-2.53784	-2.33753	-0.23721
C	-3.69592	-3.10788	-0.25569
C	-4.95527	-2.51044	-0.14424
C	-5.03180	-1.11800	-0.02851
C	-3.88182	-0.33669	-0.03551
N	-1.34088	1.19386	0.08577

H	-2.12753	1.69192	0.48519
C	-0.20781	1.83047	-0.24737
C	-0.04155	3.32951	-0.24858
C	1.11922	3.71725	0.69549
C	0.29624	3.79829	-1.68316
C	-1.32807	4.03176	0.21734
C	0.77869	0.85070	-0.66260
H	1.51970	1.11227	-1.41376
H	3.61943	0.74641	-0.11420
H	5.72856	-0.49156	-0.53308
H	5.70440	-2.97365	-0.67536
H	3.56521	-4.20862	-0.39358
H	1.46085	-2.96335	0.02616
H	-1.56892	-2.82599	-0.28190
H	-3.61293	-4.18860	-0.33971
H	-5.85708	-3.11514	-0.14486
H	-6.00115	-0.63246	0.05156
H	-3.98675	0.74423	0.01265
H	2.05387	3.24062	0.38106
H	0.90740	3.39990	1.72178
H	1.27046	4.80323	0.68987
H	1.21085	3.32342	-2.05383
H	0.45538	4.88258	-1.69596
H	-0.51905	3.55934	-2.37407
H	-1.59273	3.75162	1.24414
H	-2.17558	3.80420	-0.43987
H	-1.18098	5.11604	0.20219

2Ph-5tBu-endoCl-C45_ring_opening_TS

45

C	1.08705	-0.78089	-1.49507
C	1.79662	-0.15203	-0.51128
N	0.99989	0.71633	0.23216
H	1.42699	1.59134	0.50497
H	1.54057	-1.40106	-2.25764
C	-0.35806	0.74258	-0.08015
C	-0.33193	-0.58601	-1.43266
H	-0.93795	-0.69433	-2.32663
C	-1.01380	-0.57809	-0.12649
C	-2.50518	-0.73256	-0.04908
C	-3.25456	-1.20601	-1.12875
C	-3.16536	-0.43416	1.15291
C	-4.64207	-1.33468	-1.02706
H	-2.76820	-1.47398	-2.05957

C	-4.54632	-0.56237	1.25732
H	-2.58446	-0.11034	2.00974
C	-5.29316	-1.00592	0.16064
H	-5.21007	-1.69489	-1.87970
H	-5.04122	-0.31946	2.19295
H	-6.37197	-1.10344	0.23821
Cl	-0.31820	-1.85832	1.18009
C	-1.01153	2.13540	-0.09380
C	3.22221	-0.25182	-0.19383
C	3.69767	0.10313	1.08054
C	4.14868	-0.69052	-1.15756
C	5.05810	0.02765	1.37943
H	2.99350	0.41395	1.84611
C	5.50402	-0.77106	-0.85354
H	3.80415	-0.95197	-2.15331
C	5.96746	-0.41014	0.41601
H	5.40455	0.30316	2.37146
H	6.20407	-1.10897	-1.61237
H	7.02627	-0.47156	0.64940
C	-1.21534	2.62855	1.35607
H	-0.27591	2.62564	1.91988
H	-1.59905	3.65509	1.34897
H	-1.93470	2.00185	1.88946
C	-2.35673	2.17648	-0.84505
H	-2.26601	1.73281	-1.84170
H	-3.15707	1.66611	-0.31204
H	-2.65313	3.22362	-0.96649
C	-0.06683	3.11237	-0.83610
H	0.87449	3.29329	-0.30732
H	0.17053	2.73720	-1.83702
H	-0.56075	4.08355	-0.93898

2Ph-5tBu-endoCl_C23-cyclopropane

45

C	-1.89809	-0.84681	1.33271
C	-2.50595	-0.08450	0.39901
N	-1.56332	0.61417	-0.38806
H	-1.75637	1.59989	-0.53161
H	-2.38985	-1.51912	2.02086
C	-0.23674	0.35535	0.12979
C	-0.42650	-0.69777	1.25203
H	0.17548	-0.73495	2.15707
C	0.28280	-1.06426	-0.03717
C	1.75725	-1.31189	-0.01601

C	2.34047	-2.05107	1.01776
C	2.56945	-0.82150	-1.04783
C	3.71648	-2.28819	1.02958
H	1.71593	-2.44212	1.81546
C	3.94197	-1.05765	-1.03872
H	2.11952	-0.24598	-1.85050
C	4.51974	-1.79018	0.00288
H	4.15843	-2.86180	1.83913
H	4.56276	-0.66717	-1.83993
H	5.59078	-1.97072	0.01254
Cl	-0.54659	-2.14960	-1.23361
C	-3.97641	0.05183	0.07055
C	0.70861	1.50653	0.12124
C	0.79666	2.31637	-1.02063
C	1.53186	1.78643	1.21660
C	1.69085	3.38554	-1.06305
H	0.17337	2.09809	-1.88334
C	2.43217	2.85108	1.17155
H	1.47148	1.16739	2.10584
C	2.51397	3.65467	0.03277
H	1.74945	4.00302	-1.95468
H	3.06635	3.05507	2.02945
H	3.21408	4.48424	-0.00120
C	-4.38835	1.53917	0.14275
H	-3.86370	2.14698	-0.60329
H	-5.46122	1.63902	-0.05503
H	-4.18232	1.95680	1.13440
C	-4.82180	-0.75009	1.07166
H	-4.66113	-0.40073	2.09728
H	-5.88528	-0.63709	0.83571
H	-4.57712	-1.81675	1.03025
C	-4.23104	-0.47865	-1.35660
H	-3.63704	0.07270	-2.09162
H	-3.96191	-1.53762	-1.42925
H	-5.29089	-0.37158	-1.61502

2Ph-5tBu-endoCl_C2attack_C23-cyclopropanation_TS

45

C	1.68027	-0.59113	-1.49830
C	2.38888	0.13439	-0.52327
N	1.49572	0.93569	0.10808
H	1.71581	1.51992	0.90090
H	2.10777	-1.32739	-2.16283
C	0.18731	0.69477	-0.33394

C	0.33853	-0.22626	-1.40731
H	-0.47931	-0.61623	-1.99500
C	-0.51344	-0.73522	1.14264
C	-1.62124	-1.47469	0.49167
C	-1.59474	-2.77182	-0.06119
C	-2.85141	-0.78128	0.49251
C	-2.74398	-3.34537	-0.59367
H	-0.65974	-3.32182	-0.07602
C	-4.00042	-1.35193	-0.05194
H	-2.88804	0.20634	0.93837
C	-3.95153	-2.63628	-0.59693
H	-2.70271	-4.34649	-1.01432
H	-4.93514	-0.79837	-0.04186
H	-4.84689	-3.08744	-1.01508
Cl	0.91479	-1.82684	1.43656
C	3.86341	0.12840	-0.19402
C	-0.86457	1.71495	-0.16738
C	-0.99490	2.42290	1.03761
C	-1.77986	1.96275	-1.19885
C	-2.01046	3.36095	1.20143
H	-0.32368	2.20441	1.86198
C	-2.81006	2.88875	-1.02677
H	-1.68553	1.43011	-2.13918
C	-2.92758	3.59361	0.17121
H	-2.09817	3.90015	2.14032
H	-3.51572	3.06200	-1.83415
H	-3.72621	4.31747	0.30456
C	4.58645	1.07596	-1.18060
H	4.20775	2.09965	-1.08994
H	5.66148	1.08621	-0.96803
H	4.44234	0.74736	-2.21499
C	4.41895	-1.29941	-0.35640
H	5.48894	-1.30764	-0.12260
H	3.90942	-1.99551	0.31704
H	4.29775	-1.66253	-1.38178
C	4.10921	0.60559	1.24920
H	3.55900	-0.00995	1.96834
H	5.17650	0.53453	1.48140
H	3.82001	1.65369	1.39057

2Ph-5tBu-endoCl_C3-carbanion_cyclopropanation-intermediate

45

Cl	0.46984	-0.41471	1.94113
C	1.06605	-0.72270	0.25475

C	2.47998	-0.67216	0.05934
C	3.42007	-0.88901	1.11034
C	4.78071	-0.99897	0.85975
C	5.29443	-0.87870	-0.43826
C	4.39803	-0.63331	-1.48358
C	3.03260	-0.51688	-1.25026
C	0.12877	-0.10349	-0.80414
H	0.35004	-0.58070	-1.76835
C	-1.33076	-0.23042	-0.46774
C	-2.06195	-1.45533	-0.37036
C	-1.36618	-2.66465	-0.60156
C	-2.01731	-3.88587	-0.44743
C	-3.36511	-3.92438	-0.08857
C	-4.06720	-2.72973	0.13238
C	-3.42698	-1.50829	-0.00237
N	-1.79606	0.99659	-0.25112
H	-2.75106	1.19446	0.02204
C	-0.80744	2.00258	-0.38942
C	-1.13186	3.43455	-0.04273
C	-1.47044	3.53469	1.46188
C	0.09089	4.31459	-0.34687
C	-2.33288	3.92253	-0.88171
C	0.32549	1.38994	-0.80233
H	1.28282	1.85579	-0.97694
H	3.05644	-0.98131	2.12778
H	5.45648	-1.17243	1.69456
H	6.36122	-0.95640	-0.62518
H	4.76922	-0.51126	-2.49882
H	2.38157	-0.29847	-2.09006
H	-0.31703	-2.63112	-0.86990
H	-1.47309	-4.80961	-0.61858
H	-3.87435	-4.87770	0.01449
H	-5.11574	-2.75948	0.41170
H	-3.98469	-0.59508	0.18227
H	-0.62967	3.18755	2.07112
H	-2.34859	2.93387	1.72353
H	-1.68747	4.57504	1.72808
H	0.95760	4.00645	0.24708
H	-0.13495	5.35748	-0.10277
H	0.36261	4.26259	-1.40650
H	-3.24123	3.34258	-0.68104
H	-2.11526	3.85386	-1.95260
H	-2.55316	4.96772	-0.63996

2Ph-5tBu-endoCl_C34-cyclopropanation_step1_C3attack_TS

45

Cl	0.24170	-0.44483	2.01858
C	0.47781	-1.54337	0.56850
C	1.92253	-1.66897	0.25628
C	3.00554	-1.00352	0.86410
C	4.31682	-1.29071	0.49500
C	4.57960	-2.23894	-0.49884
C	3.51880	-2.90787	-1.11697
C	2.20939	-2.62888	-0.73810
C	-0.18894	-0.36440	-1.10833
H	-0.17214	-1.22473	-1.75970
C	-1.39348	0.18608	-0.59049
C	-2.67759	-0.45117	-0.36964
C	-2.74770	-1.85667	-0.31845
C	-3.96750	-2.48973	-0.09447
C	-5.13332	-1.73847	0.07664
C	-5.07214	-0.34258	0.02607
C	-3.85685	0.29830	-0.19599
N	-1.11509	1.46417	-0.23100
H	-1.73456	2.03895	0.32382
C	0.20834	1.80770	-0.52948
C	0.76805	3.15999	-0.16449
C	0.64858	3.38316	1.35964
C	2.25083	3.21755	-0.56524
C	-0.00982	4.26660	-0.91024
C	0.76934	0.71616	-1.13546
H	1.78708	0.63819	-1.48459
H	2.80999	-0.25554	1.62433
H	5.13906	-0.76921	0.97724
H	5.60398	-2.45724	-0.78664
H	3.71534	-3.64972	-1.88564
H	1.37917	-3.15520	-1.20126
H	-1.82906	-2.42705	-0.40146
H	-4.00853	-3.57422	-0.04734
H	-6.08381	-2.23578	0.24583
H	-5.97522	0.24749	0.15127
H	-3.83018	1.38229	-0.26290
H	1.17764	2.59829	1.90877
H	-0.39604	3.38015	1.69132
H	1.07844	4.35416	1.63005
H	2.82825	2.44277	-0.04932
H	2.67057	4.19287	-0.29822
H	2.37600	3.07736	-1.64427
H	-1.07363	4.25960	-0.64709
H	0.07198	4.13633	-1.99454

H	0.39266	5.25161	-0.64882

2Ph-5tBu-endoCl_C34-cyclopropanation_step1_C4attack_TS

45

Cl	0.61638	0.00169	2.05972
C	1.85959	0.35390	0.73313
C	2.48585	-0.92160	0.27945
C	3.58281	-0.75036	-0.59279
C	4.32233	-1.83657	-1.04929
C	3.99417	-3.13056	-0.63186
C	2.91820	-3.32039	0.23892
C	2.16792	-2.23259	0.68220
C	-0.14012	-0.32854	-1.09253
H	0.14721	-1.29707	-1.47322
C	-1.36321	0.00388	-0.57645
C	-2.57565	-0.78199	-0.37240
C	-2.48658	-2.18008	-0.24472
C	-3.63256	-2.95002	-0.06847
C	-4.88905	-2.34066	-0.00103
C	-4.98806	-0.95309	-0.11940
C	-3.84407	-0.17941	-0.30896
N	-1.28616	1.36311	-0.21140
H	-2.02429	1.85347	0.27675
C	-0.06871	1.87636	-0.44499
C	0.29586	3.30731	-0.14182
C	0.27251	3.53082	1.38836
C	1.71068	3.59505	-0.67111
C	-0.71904	4.25247	-0.82132
C	0.74343	0.80476	-0.92766
H	1.61753	0.98256	-1.53496
H	3.85078	0.25863	-0.89587
H	5.16122	-1.67629	-1.72077
H	4.57430	-3.98093	-0.97852
H	2.65708	-4.32273	0.56791
H	1.32536	-2.39426	1.34528
H	-1.50984	-2.65393	-0.26358
H	-3.54426	-4.02808	0.03042
H	-5.78135	-2.94200	0.14478
H	-5.95958	-0.46968	-0.07378
H	-3.94567	0.89509	-0.43356
H	0.98772	2.86892	1.88120
H	-0.72069	3.33941	1.80926
H	0.53787	4.57087	1.60742
H	2.43039	2.90957	-0.21469

H	1.99385	4.62314	-0.42291
H	1.75802	3.48392	-1.76010
H	-1.73585	4.10405	-0.44099
H	-0.73452	4.10032	-1.90566
H	-0.43853	5.29241	-0.62323

2Ph-5tBu-endoCl_C34-cyclopropanation_step2formingC3bond_TS

45

Cl	0.79270	0.25268	1.99023
C	1.43580	0.10842	0.35256
C	2.50199	-0.79872	0.08332
C	2.96645	-1.00566	-1.25086
C	4.05509	-1.82820	-1.51905
C	4.73239	-2.49369	-0.49274
C	4.28323	-2.31573	0.82173
C	3.19606	-1.50123	1.10925
C	-0.16797	-0.40204	-0.96766
H	0.21133	-1.31621	-1.39577
C	-1.44844	-0.16622	-0.49230
C	-2.56941	-1.06150	-0.27511
C	-2.35708	-2.45631	-0.27788
C	-3.41612	-3.34055	-0.09959
C	-4.71510	-2.86329	0.10015
C	-4.93856	-1.48431	0.11120
C	-3.88492	-0.59365	-0.07943
N	-1.49157	1.19702	-0.16106
H	-2.32232	1.65091	0.20165
C	-0.34201	1.83486	-0.30380
C	-0.14239	3.31455	-0.16776
C	1.14353	3.57448	0.64662
C	0.02790	3.90765	-1.59062
C	-1.34175	3.98279	0.52389
C	0.67076	0.82856	-0.75259
H	1.33276	1.18355	-1.54985
H	2.46415	-0.52275	-2.08234
H	4.37380	-1.95503	-2.55120
H	5.57858	-3.13833	-0.71006
H	4.78621	-2.82697	1.63956
H	2.87289	-1.38718	2.13796
H	-1.34943	-2.84047	-0.40303
H	-3.22396	-4.40996	-0.10425
H	-5.53923	-3.55492	0.24612
H	-5.94212	-1.09608	0.26112
H	-4.10098	0.47122	-0.09436

H	2.00486	3.05947	0.20910
H	1.02990	3.22745	1.67723
H	1.35619	4.64836	0.65870
H	0.88470	3.46465	-2.10815
H	0.19875	4.98703	-1.51565
H	-0.86850	3.73887	-2.19578
H	-1.50990	3.56639	1.52332
H	-2.26235	3.88058	-0.06279
H	-1.14685	5.05341	0.63597

2Ph-5tBu-endoCl_C34-cyclopropanation_step2formingC4bond_TS

45

Cl	0.42686	-0.36102	1.95006
C	0.97989	-0.63622	0.30320
C	2.32238	-1.07499	0.06778
C	3.23555	-1.35423	1.11996
C	4.50887	-1.84441	0.85964
C	4.94555	-2.06614	-0.45159
C	4.06870	-1.78406	-1.50298
C	2.78829	-1.29884	-1.26014
C	-0.01314	-0.37340	-0.81514
H	0.06308	-1.08495	-1.64356
C	-1.40607	-0.03571	-0.39768
C	-2.51105	-0.93282	-0.29267
C	-2.27981	-2.32388	-0.42889
C	-3.32374	-3.23413	-0.30395
C	-4.62330	-2.79337	-0.03810
C	-4.86733	-1.42053	0.09856
C	-3.83520	-0.50134	-0.02998
N	-1.43085	1.29447	-0.16406
H	-2.23860	1.76754	0.22343
C	-0.24730	1.94658	-0.43165
C	-0.05693	3.41158	-0.11898
C	-0.07606	3.62243	1.41259
C	1.30062	3.87372	-0.67359
C	-1.18089	4.24637	-0.76913
C	0.63851	0.99154	-0.91097
H	1.62315	1.16467	-1.31237
H	2.92700	-1.18970	2.14600
H	5.17395	-2.05078	1.69472
H	5.94501	-2.44203	-0.64793
H	4.38483	-1.94094	-2.53135
H	2.14433	-1.08499	-2.10613
H	-1.27231	-2.68185	-0.61481

H	-3.12091	-4.29616	-0.41018
H	-5.43640	-3.50619	0.05841
H	-5.87456	-1.06587	0.29844
H	-4.05914	0.55816	0.05855
H	0.71467	3.03721	1.89282
H	-1.03396	3.31499	1.84696
H	0.07852	4.68107	1.65162
H	2.12571	3.31921	-0.21427
H	1.44595	4.93712	-0.45773
H	1.35403	3.73547	-1.75893
H	-2.17099	3.97302	-0.38585
H	-1.18882	4.11264	-1.85592
H	-1.03052	5.30955	-0.55233

2Ph-5tBu-endoCl_C34-cyclopropane

45

Cl	0.88981	-0.04237	2.04883
C	1.13256	-0.28445	0.25096
C	2.39486	-1.03638	-0.02093
C	3.60737	-0.35264	-0.17001
C	4.79029	-1.05543	-0.39797
C	4.77005	-2.45010	-0.47811
C	3.56398	-3.13863	-0.32732
C	2.38194	-2.43397	-0.09903
C	-0.06670	-0.57007	-0.59225
H	0.02759	-1.38092	-1.31069
C	-1.39061	-0.14305	-0.15656
C	-2.59846	-0.91411	-0.14395
C	-2.53954	-2.32597	-0.25543
C	-3.69623	-3.09838	-0.26443
C	-4.95551	-2.50449	-0.13390
C	-5.03298	-1.11292	-0.00789
C	-3.88487	-0.32915	-0.02308
N	-1.34128	1.20595	0.06787
H	-2.12723	1.70782	0.46407
C	-0.20259	1.83591	-0.25594
C	-0.02094	3.33254	-0.24590
C	1.14372	3.70059	0.70135
C	0.32245	3.80893	-1.67675
C	-1.30015	4.04435	0.22545
C	0.77658	0.84522	-0.67221
H	1.51953	1.10028	-1.42363
H	3.62072	0.73168	-0.10413
H	5.72606	-0.51627	-0.51337

H	5.69053	-2.99809	-0.65749
H	3.54400	-4.22289	-0.38759
H	1.44348	-2.96748	0.02265
H	-1.57054	-2.81249	-0.31652
H	-3.61201	-4.17835	-0.35681
H	-5.85609	-3.11095	-0.12773
H	-6.00214	-0.62976	0.08764
H	-3.99193	0.75107	0.03590
H	2.07304	3.21518	0.38452
H	0.92746	3.37935	1.72544
H	1.30763	4.78473	0.70245
H	1.23226	3.32724	-2.05050
H	0.49284	4.89159	-1.68129
H	-0.49489	3.58363	-2.36983
H	-1.56836	3.75767	1.24951
H	-2.14960	3.83181	-0.43433
H	-1.14157	5.12710	0.21995

2Ph-5tBu-endoCl_C3attack_C23-cyclopropanation_TS

45

C	2.07942	-0.74162	-1.43824
C	2.64415	0.10339	-0.51968
N	1.62629	0.92830	-0.04407
H	1.75838	1.65644	0.64198
H	2.59017	-1.52447	-1.97845
C	0.43181	0.63933	-0.64116
C	0.66304	-0.48415	-1.47663
H	-0.00826	-0.80733	-2.25652
C	-0.19744	-1.99095	-0.10337
C	-1.60701	-1.59683	0.04767
C	-2.40853	-1.90697	-1.07480
C	-2.24256	-1.03446	1.17431
C	-3.77934	-1.67403	-1.07176
H	-1.92802	-2.35045	-1.94261
C	-3.61468	-0.80834	1.18085
H	-1.64906	-0.77773	2.04379
C	-4.38782	-1.12379	0.06004
H	-4.37483	-1.92357	-1.94520
H	-4.08466	-0.37305	2.05806
H	-5.45884	-0.94281	0.06923
Cl	0.71842	-1.76280	1.46318
C	4.06140	0.25168	-0.02204
C	-0.79121	1.36421	-0.35602
C	-1.85814	1.34118	-1.27264

C	-0.95875	2.06633	0.85345
C	-3.05210	1.99325	-0.98633
H	-1.74375	0.81207	-2.21184
C	-2.15081	2.72900	1.12983
H	-0.16850	2.06522	1.59824
C	-3.20442	2.69186	0.21363
H	-3.86803	1.95477	-1.70162
H	-2.26354	3.26046	2.07021
H	-4.13765	3.20141	0.43428
C	4.94794	-0.82271	-0.67116
H	5.97805	-0.71950	-0.31464
H	4.59772	-1.82896	-0.41809
H	4.95477	-0.72518	-1.76202
C	4.10020	0.08139	1.51246
H	5.13067	0.17655	1.87255
H	3.49847	0.84215	2.02228
H	3.71674	-0.90132	1.80300
C	4.59882	1.65040	-0.39962
H	5.62980	1.76462	-0.04649
H	4.58805	1.79057	-1.48574
H	4.00144	2.45068	0.05130

2Ph-5tBu-endoCl_C4-carbanion_cyclopropanation-intermediate

45

Cl	0.91341	0.15330	2.02792
C	1.68674	0.28448	0.37489
C	2.65437	-0.72403	0.05957
C	3.01045	-1.01353	-1.29387
C	4.06025	-1.87047	-1.60770
C	4.79644	-2.51418	-0.60844
C	4.44340	-2.27508	0.72603
C	3.40255	-1.41783	1.05645
C	-0.27174	-0.52641	-0.87968
H	0.08957	-1.50346	-1.16331
C	-1.52358	-0.21383	-0.46329
C	-2.72709	-1.01136	-0.25431
C	-2.61105	-2.39875	-0.04974
C	-3.74476	-3.18519	0.12885
C	-5.01656	-2.60441	0.11873
C	-5.14250	-1.22843	-0.07956
C	-4.01060	-0.43754	-0.26939
N	-1.51172	1.18892	-0.18675
H	-2.33494	1.70450	0.10551
C	-0.32840	1.73992	-0.29818

C	-0.06432	3.21005	-0.15490
C	1.01751	3.43651	0.92558
C	0.47017	3.71546	-1.52028
C	-1.34312	3.98027	0.21157
C	0.63699	0.65566	-0.69464
H	1.18484	0.94556	-1.59871
H	2.45521	-0.56280	-2.11008
H	4.29247	-2.05208	-2.65507
H	5.60673	-3.19200	-0.85975
H	4.98595	-2.77413	1.52650
H	3.15498	-1.26053	2.10053
H	-1.62500	-2.85125	-0.01363
H	-3.63519	-4.25380	0.28877
H	-5.89967	-3.21876	0.26534
H	-6.12528	-0.76673	-0.09490
H	-4.13945	0.62501	-0.45556
H	1.88848	2.80025	0.74376
H	0.62792	3.20131	1.91932
H	1.32157	4.48781	0.90454
H	1.42722	3.24885	-1.77024
H	0.62422	4.79735	-1.45900
H	-0.24080	3.51324	-2.32796
H	-1.75805	3.64292	1.16842
H	-2.11293	3.88998	-0.56384
H	-1.10462	5.04245	0.31570

2Ph-5tBu-endoCl_C45-cyclopropane

45

C	-1.08440	-0.57112	1.49089
C	-1.82126	-0.06417	0.47330
N	-1.01776	0.64536	-0.43662
H	-1.40928	1.50683	-0.79657
H	-1.44527	-1.22752	2.27059
C	0.33841	0.72833	0.07260
C	0.33361	-0.19284	1.31415
H	0.96048	-0.00816	2.18222
C	1.06896	-0.60854	0.04330
C	2.55753	-0.75238	0.03180
C	3.25854	-1.11359	1.18573
C	3.26923	-0.55993	-1.16209
C	4.64898	-1.24263	1.16040
H	2.71942	-1.28941	2.11090
C	4.65492	-0.69010	-1.19066
H	2.72743	-0.30705	-2.06827

C	5.35143	-1.02510	-0.02471
H	5.18022	-1.51662	2.06727
H	5.19325	-0.52866	-2.12008
H	6.43296	-1.12208	-0.04452
Cl	0.33667	-1.93059	-0.97825
C	0.97142	2.13071	-0.01051
C	-3.25155	-0.22705	0.19504
C	-3.74863	-0.07048	-1.10929
C	-4.15319	-0.53194	1.22957
C	-5.11067	-0.21768	-1.37187
H	-3.05866	0.14438	-1.91897
C	-5.51076	-0.68454	0.96359
H	-3.78463	-0.63163	2.24599
C	-5.99649	-0.52629	-0.33851
H	-5.47797	-0.09680	-2.38699
H	-6.19442	-0.91823	1.77474
H	-7.05696	-0.64083	-0.54302
C	2.33639	2.23422	0.69515
H	2.28823	1.86624	1.72517
H	3.12256	1.69030	0.17276
H	2.62883	3.28911	0.73395
C	0.02373	3.12060	0.70560
H	0.46996	4.12047	0.70837
H	-0.95452	3.20429	0.22246
H	-0.13908	2.81541	1.74506
C	1.13768	2.53503	-1.48782
H	0.18597	2.51417	-2.03048
H	1.53663	3.55312	-1.55822
H	1.83240	1.86209	-1.99978

2Ph-5tBu-endoCl_C4attack_C45-cyclopropanation_TS

45

C	1.08852	-0.41007	-1.58394
C	1.89624	0.14114	-0.63087
N	1.10889	1.05897	0.08528
H	1.46832	1.65626	0.81656
H	1.35508	-1.21438	-2.25356
C	-0.15372	1.11737	-0.39546
C	-0.25769	0.09554	-1.38252
H	-1.02528	0.10423	-2.14146
C	-1.08545	-1.55137	-0.37645
C	-2.50423	-1.23055	-0.11697
C	-3.31862	-1.18970	-1.27421
C	-3.14613	-1.12924	1.13409

C	-4.69325	-1.01156	-1.18779
H	-2.84549	-1.30531	-2.24529
C	-4.52820	-0.96923	1.22172
H	-2.55423	-1.18634	2.04041
C	-5.30722	-0.89630	0.06543
H	-5.29170	-0.97311	-2.09350
H	-4.99830	-0.89597	2.19856
H	-6.38288	-0.76509	0.13730
Cl	-0.13785	-1.68340	1.19004
C	3.29032	-0.11489	-0.27746
C	3.77271	0.12233	1.02096
C	4.17802	-0.61967	-1.24412
C	5.10564	-0.13311	1.34035
H	3.10033	0.47916	1.79575
C	5.50555	-0.88301	-0.91890
H	3.82309	-0.78913	-2.25616
C	5.97750	-0.63738	0.37394
H	5.45931	0.05379	2.35011
H	6.17711	-1.27121	-1.67921
H	7.01495	-0.83759	0.62442
C	-1.10496	2.19594	0.07768
C	-1.41805	2.00856	1.57827
H	-0.50797	2.04076	2.18717
H	-2.07892	2.81301	1.91894
H	-1.91668	1.05435	1.75966
C	-2.41031	2.17807	-0.73301
H	-3.04048	3.01611	-0.41720
H	-2.21193	2.29137	-1.80442
H	-2.97192	1.25640	-0.58176
C	-0.42198	3.56972	-0.13070
H	-0.15831	3.72113	-1.18275
H	-1.11008	4.36669	0.17070
H	0.48902	3.67422	0.46797

2Ph-5tBu-exoCl-C23_ring_opening_TS

45

C	1.03996	0.88397	-1.80496
C	1.09529	1.47948	-0.56607
N	0.00948	1.15119	0.23384
H	-0.36763	1.87299	0.83530
H	1.67824	1.20104	-2.62009
C	-0.87452	0.19429	-0.23647
C	0.04969	-0.12067	-1.98169
H	-0.38146	-0.37688	-2.94391

C	-0.19238	-1.00555	-0.80309
C	0.96886	-1.58586	-0.03000
C	2.14186	-1.96524	-0.68569
C	0.86865	-1.74748	1.35461
C	3.20778	-2.50300	0.03610
H	2.21928	-1.82256	-1.75922
C	1.93255	-2.28400	2.07941
H	-0.04393	-1.44705	1.86184
C	3.10613	-2.66047	1.42091
H	4.11837	-2.79429	-0.47980
H	1.84873	-2.40414	3.15578
H	3.93810	-3.07318	1.98442
Cl	-1.35984	-2.36373	-1.21023
C	2.15762	2.40258	-0.00509
C	-2.30012	0.33442	0.11578
C	-3.31906	0.23169	-0.84370
C	-2.65589	0.60505	1.44905
C	-4.65585	0.37564	-0.47682
H	-3.05281	0.05029	-1.87919
C	-3.99356	0.76441	1.81032
H	-1.88300	0.67226	2.21006
C	-4.99975	0.64560	0.85005
H	-5.43030	0.29195	-1.23397
H	-4.24777	0.97011	2.84618
H	-6.04169	0.76568	1.13171
C	2.99476	1.61371	1.02844
H	2.36240	1.24391	1.84186
H	3.47253	0.74826	0.55793
H	3.77441	2.25455	1.45767
C	1.51783	3.62586	0.68565
H	2.30069	4.30318	1.04324
H	0.87549	4.18098	-0.00691
H	0.92129	3.34314	1.56125
C	3.08154	2.89490	-1.13011
H	2.52312	3.45143	-1.89072
H	3.84773	3.55864	-0.71594
H	3.59070	2.05866	-1.62037

2Ph-5tBu-exoCl-C34_ring_opening_TS

45

Cl	-1.50402	-1.87017	2.62690
C	-0.90396	-0.89381	1.20799
C	-1.19941	-1.56095	-0.09389
C	-0.23462	-2.35972	-0.71518

C	-0.51623	-2.97947	-1.93363
C	-1.76262	-2.79893	-2.53838
C	-2.72947	-2.00045	-1.92077
C	-2.44575	-1.38151	-0.70359
C	0.40441	-0.23672	1.48334
H	0.92783	-0.48943	2.40077
C	1.10773	0.35883	0.37398
C	2.52038	0.29370	0.10689
C	3.18170	1.20228	-0.75427
C	4.54678	1.09591	-0.99806
C	5.30998	0.10130	-0.37722
C	4.67591	-0.79653	0.48662
C	3.30549	-0.71571	0.71347
N	0.25297	1.19219	-0.29851
H	0.52900	1.63973	-1.16442
C	-0.95054	1.43682	0.26487
C	-1.82418	2.57601	-0.21718
C	-1.05360	3.91486	-0.15163
C	-2.25798	2.30924	-1.67794
C	-3.08100	2.67855	0.66237
C	-1.13224	0.57769	1.40349
H	-1.73430	0.88468	2.25157
H	0.73638	-2.48593	-0.24630
H	0.23645	-3.59977	-2.41169
H	-1.98018	-3.27840	-3.48855
H	-3.70001	-1.85921	-2.38755
H	-3.19013	-0.75725	-0.21778
H	2.63126	2.01630	-1.21841
H	5.02344	1.80837	-1.66629
H	6.37668	0.02685	-0.56574
H	5.25033	-1.58046	0.97335
H	2.82263	-1.44834	1.35340
H	-0.15587	3.89436	-0.77970
H	-0.74200	4.13332	0.87539
H	-1.68653	4.73776	-0.50329
H	-1.39559	2.25294	-2.35247
H	-2.90339	3.12086	-2.03332
H	-2.80681	1.36589	-1.75618
H	-2.82519	2.91003	1.70196
H	-3.65488	1.74580	0.64943
H	-3.72629	3.48109	0.29034

2Ph-5tBu-exoCl-C45_ring_opening_TS

45

C	-0.46575	-0.42314	-1.86662
C	-1.06344	-0.67958	-0.65153
N	-0.14272	-1.03045	0.33111
H	-0.43938	-1.73164	0.99624
H	-1.02991	-0.34551	-2.78829
C	1.20693	-0.94408	0.02780
C	0.94731	-0.31127	-1.83086
H	1.58601	-0.49409	-2.68802
C	1.52498	0.36190	-0.62268
C	0.87986	1.61420	-0.07317
C	0.38506	2.59016	-0.94167
C	0.80210	1.81718	1.30706
C	-0.18001	3.76068	-0.43650
H	0.43719	2.42166	-2.01313
C	0.23475	2.98588	1.81587
H	1.18096	1.05523	1.98136
C	-0.25838	3.95999	0.94454
H	-0.56383	4.51422	-1.11840
H	0.17526	3.13470	2.89029
H	-0.70377	4.86875	1.33929
Cl	3.31002	0.73794	-0.88202
C	2.15846	-1.99779	0.60872
C	-2.48504	-0.69418	-0.31102
C	-2.90185	-0.55609	1.02582
C	-3.47129	-0.85704	-1.30284
C	-4.25551	-0.59615	1.36036
H	-2.16133	-0.38276	1.80073
C	-4.82065	-0.88737	-0.96603
H	-3.17478	-0.97650	-2.34034
C	-5.22241	-0.76142	0.36830
H	-4.55321	-0.48440	2.39923
H	-5.56411	-1.01790	-1.74746
H	-6.27665	-0.78724	0.62761
C	3.00613	-1.39405	1.75174
H	2.36104	-1.02511	2.55723
H	3.66215	-2.16428	2.17362
H	3.62928	-0.56832	1.40483
C	1.36004	-3.18414	1.19157
H	0.76762	-2.90109	2.07019
H	0.69780	-3.63775	0.44665
H	2.06438	-3.95249	1.52441
C	3.07388	-2.57884	-0.49177
H	2.47261	-3.05034	-1.27552
H	3.70063	-1.81604	-0.95341
H	3.72960	-3.34004	-0.05316

2Ph-5tBu-exoCl_C23-cyclopropane

45

C	0.86960	0.97798	-1.67765
C	1.00542	1.53647	-0.45465
N	0.01371	1.07674	0.44040
H	-0.45835	1.78923	0.98747
H	1.54305	1.11284	-2.51161
C	-0.88703	0.19458	-0.28445
C	-0.29074	0.05370	-1.69503
H	-0.89992	-0.07976	-2.58507
C	-0.23145	-1.10844	-0.71168
C	1.03418	-1.59693	-0.09892
C	2.15799	-1.85088	-0.89203
C	1.10794	-1.80303	1.28265
C	3.34242	-2.30469	-0.31185
H	2.10043	-1.68220	-1.96303
C	2.29012	-2.25812	1.86587
H	0.23655	-1.59937	1.89742
C	3.41074	-2.50637	1.06940
H	4.21067	-2.49952	-0.93492
H	2.33932	-2.41396	2.93977
H	4.33382	-2.85630	1.52289
Cl	-1.39262	-2.45642	-1.07711
C	2.10054	2.43543	0.07358
C	-2.32759	0.29925	0.07748
C	-3.29517	0.66643	-0.86219
C	-2.71848	0.05262	1.40108
C	-4.63583	0.77662	-0.48958
H	-2.99642	0.86530	-1.88665
C	-4.05610	0.17006	1.77637
H	-1.97024	-0.23470	2.13470
C	-5.01902	0.53010	0.83005
H	-5.37912	1.05978	-1.22928
H	-4.34758	-0.02492	2.80439
H	-6.06197	0.61761	1.12039
C	2.94236	1.63280	1.09102
H	2.32349	1.28986	1.92479
H	3.38293	0.74963	0.61716
H	3.75033	2.25860	1.48824
C	3.00456	2.90793	-1.07501
H	2.43319	3.45680	-1.83146
H	3.78400	3.57207	-0.68650
H	3.49669	2.06091	-1.56458
C	1.48574	3.66650	0.77295

H	2.28165	4.32895	1.13038
H	0.85076	4.23575	0.08485
H	0.88411	3.38354	1.64417

2Ph-5tBu-exoCl_C3-carbanion_cyclopropanation-intermediate

45

Cl	-0.79515	-2.29327	-2.56836
C	-0.69221	-1.20538	-1.13971
C	-0.91477	-1.77099	0.13880
C	-0.45882	-1.13622	1.33782
C	-0.87828	-1.56652	2.59354
C	-1.71206	-2.67661	2.74016
C	-2.10420	-3.36825	1.58182
C	-1.71343	-2.94562	0.32295
C	0.21695	-0.01755	-1.45556
H	-0.00036	0.30574	-2.48667
C	0.07078	1.16342	-0.51969
C	-1.13130	1.89768	-0.29307
C	-1.23615	2.91121	0.69092
C	-2.44827	3.53956	0.92025
C	-3.58681	3.17454	0.18315
C	-3.49722	2.17937	-0.78964
C	-2.28064	1.54923	-1.04254
N	1.27614	1.40809	0.00105
H	1.46682	2.16561	0.64517
C	2.24656	0.44499	-0.36917
C	3.60906	0.46496	0.27870
C	4.29030	1.82822	0.03132
C	3.45402	0.22587	1.79804
C	4.47256	-0.65324	-0.32553
C	1.67226	-0.37754	-1.27534
H	2.13138	-1.22833	-1.75497
H	0.26508	-0.33812	1.28798
H	-0.51189	-1.04242	3.47384
H	-2.02055	-3.01818	3.72361
H	-2.72614	-4.25666	1.66861
H	-2.04232	-3.49804	-0.55043
H	-0.37350	3.18811	1.28980
H	-2.51784	4.31317	1.67869
H	-4.53321	3.67371	0.36712
H	-4.37449	1.90094	-1.36567
H	-2.20737	0.78834	-1.80926
H	3.72132	2.65883	0.46470
H	4.40614	2.01820	-1.04073

H	5.28324	1.83542	0.49322
H	2.86628	1.01671	2.27779
H	4.43925	0.20813	2.27665
H	2.95598	-0.72997	1.99070
H	4.59888	-0.51688	-1.40472
H	4.02369	-1.63678	-0.15210
H	5.46410	-0.64528	0.13814

2Ph-5tBu-exoCl_C34-cyclopropanation_step1_C3attack_TS

45

Cl	0.81691	-2.96829	-1.89335
C	-0.20169	-2.01627	-0.65079
C	0.62554	-1.69380	0.53265
C	-0.11314	-1.21364	1.63677
C	0.50196	-0.94779	2.85699
C	1.87447	-1.16316	3.00821
C	2.62587	-1.63127	1.92546
C	2.01228	-1.88100	0.70125
C	-0.39875	-0.18918	-1.71924
H	-0.91387	-0.68251	-2.52967
C	-1.06721	0.62657	-0.76336
C	-2.44735	0.58951	-0.32091
C	-3.02787	1.66162	0.38409
C	-4.34877	1.58820	0.81515
C	-5.11360	0.44785	0.55080
C	-4.54594	-0.62090	-0.14745
C	-3.22509	-0.55525	-0.58362
N	-0.12543	1.43384	-0.21549
H	-0.27992	2.00262	0.60572
C	1.12726	1.24795	-0.80791
C	2.32730	2.05710	-0.37661
C	2.22784	3.47485	-0.98479
C	2.38337	2.16400	1.16131
C	3.60782	1.37088	-0.88006
C	0.96124	0.29649	-1.77931
H	1.73495	-0.09938	-2.41855
H	-1.18361	-1.07697	1.52253
H	-0.08909	-0.58477	3.69282
H	2.35606	-0.96712	3.96188
H	3.69454	-1.79281	2.03584
H	2.60365	-2.23317	-0.13612
H	-2.45636	2.56627	0.57066
H	-4.78569	2.42533	1.35134
H	-6.14532	0.39511	0.88543

H	-5.13328	-1.51219	-0.34830
H	-2.76091	-1.39878	-1.08180
H	1.31503	3.98384	-0.65570
H	2.21601	3.42842	-2.07884
H	3.08594	4.08128	-0.67283
H	1.53311	2.72646	1.56571
H	3.29073	2.70023	1.45967
H	2.39495	1.17478	1.62648
H	3.61697	1.29533	-1.97254
H	3.69886	0.36278	-0.46326
H	4.48460	1.95274	-0.57684

2Ph-5tBu-exoCl_C34-cyclopropanation_step1_C4attack_TS

45

Cl	-1.66678	2.98806	-1.53240
C	-1.65723	1.76613	-0.10774
C	-0.36558	1.84500	0.61099
C	0.72627	2.69297	0.32808
C	1.85861	2.70018	1.13548
C	1.94664	1.84982	2.24257
C	0.87501	1.00963	2.54823
C	-0.26605	1.01500	1.74979
C	-0.20969	0.04587	-1.63305
H	0.21764	0.73113	-2.34963
C	0.48693	-0.83315	-0.84684
C	1.91577	-1.09237	-0.70174
C	2.39690	-2.32855	-0.23621
C	3.76590	-2.55052	-0.09719
C	4.67960	-1.54889	-0.43154
C	4.21131	-0.31850	-0.90103
C	2.84436	-0.08782	-1.02783
N	-0.46904	-1.49299	-0.05213
H	-0.23488	-2.18386	0.64859
C	-1.71396	-1.04976	-0.28299
C	-2.95207	-1.64857	0.34207
C	-3.94274	-1.98104	-0.79807
C	-2.61871	-2.93789	1.11191
C	-3.61257	-0.63245	1.30136
C	-1.59655	0.02957	-1.21648
H	-2.42313	0.32923	-1.84282
H	0.68275	3.34082	-0.53961
H	2.68583	3.36206	0.89362
H	2.83839	1.84915	2.86262
H	0.92388	0.35316	3.41246

H	-1.10444	0.38011	2.00773
H	1.70373	-3.13216	-0.00269
H	4.11850	-3.51224	0.26407
H	5.74597	-1.72377	-0.32429
H	4.91345	0.47084	-1.15386
H	2.48340	0.88294	-1.34944
H	-3.49461	-2.66758	-1.52403
H	-4.25751	-1.07652	-1.32732
H	-4.83594	-2.45627	-0.37858
H	-2.16995	-3.69601	0.46023
H	-3.53815	-3.35782	1.53099
H	-1.93961	-2.74673	1.95146
H	-3.68978	0.35525	0.83962
H	-3.02886	-0.52609	2.22171
H	-4.61087	-0.98956	1.57631

2Ph-5tBu-exoCl_C34-cyclopropanation_step2formingC3bond_TS

45

Cl	-1.84980	-1.41333	2.65471
C	-1.35212	-0.66642	1.11324
C	-1.57995	-1.38379	-0.09970
C	-2.31780	-2.60428	-0.11735
C	-2.60763	-3.26438	-1.30147
C	-2.17372	-2.76442	-2.53731
C	-1.42457	-1.58933	-2.54527
C	-1.11253	-0.91856	-1.36315
C	0.51281	0.09694	1.57004
H	0.77026	-0.49907	2.43091
C	1.37778	0.51031	0.55659
C	2.71801	0.07425	0.21664
C	3.57159	0.82977	-0.61351
C	4.85653	0.38294	-0.91045
C	5.33229	-0.81893	-0.37955
C	4.49890	-1.57309	0.45182
C	3.20899	-1.13955	0.74183
N	0.68512	1.47402	-0.17365
H	1.08822	1.95140	-0.97248
C	-0.58391	1.64084	0.18651
C	-1.50176	2.70758	-0.33371
C	-1.54217	3.87001	0.69151
C	-1.01859	3.24040	-1.69314
C	-2.91675	2.10944	-0.48278
C	-0.83515	0.73811	1.35862
H	-1.34699	1.23530	2.19061

H	-2.67863	-3.01678	0.81714
H	-3.18442	-4.18537	-1.26101
H	-2.40168	-3.28792	-3.46084
H	-1.05041	-1.18809	-3.48423
H	-0.48357	-0.04802	-1.42709
H	3.24533	1.78504	-1.01651
H	5.49301	0.98389	-1.55398
H	6.33598	-1.16256	-0.61061
H	4.85142	-2.51300	0.86768
H	2.56094	-1.74958	1.36359
H	-0.55111	4.31922	0.81208
H	-1.88752	3.52474	1.67140
H	-2.23450	4.64393	0.34231
H	-0.04507	3.73908	-1.61711
H	-1.73069	3.98087	-2.06944
H	-0.94684	2.43501	-2.43282
H	-3.27847	1.70140	0.46678
H	-2.92190	1.30266	-1.22168
H	-3.61400	2.88951	-0.80569

2Ph-5tBu-exoCl_C34-cyclopropanation_step2formingC4bond_TS

45

Cl	-1.34165	-1.83574	2.63214
C	-0.65877	-1.11569	1.15022
C	-1.00631	-1.70557	-0.10971
C	-0.48720	-1.22528	-1.34505
C	-0.79652	-1.84920	-2.55152
C	-1.65215	-2.94936	-2.60094
C	-2.19142	-3.42608	-1.39960
C	-1.88453	-2.82391	-0.18832
C	0.26339	0.03527	1.50343
H	0.78191	-0.11512	2.45469
C	1.09718	0.67418	0.43860
C	2.48084	0.43758	0.17498
C	3.24603	1.23407	-0.71293
C	4.57779	0.93412	-0.96618
C	5.19726	-0.15543	-0.34082
C	4.45772	-0.94602	0.54360
C	3.12072	-0.66147	0.79908
N	0.32164	1.61517	-0.15305
H	0.62951	2.15410	-0.95379
C	-0.94098	1.73146	0.37139
C	-1.98724	2.62263	-0.25428
C	-1.43341	4.05087	-0.44278

C	-2.40290	2.04391	-1.62739
C	-3.22006	2.67500	0.66316
C	-1.01551	0.85110	1.44787
H	-1.80059	0.80737	2.18390
H	0.17677	-0.37828	-1.36296
H	-0.36582	-1.45288	-3.46777
H	-1.90100	-3.41977	-3.54738
H	-2.86402	-4.28017	-1.40664
H	-2.31845	-3.22173	0.72069
H	2.80077	2.09988	-1.19530
H	5.14340	1.56063	-1.65028
H	6.24055	-0.38066	-0.53923
H	4.92499	-1.79535	1.03427
H	2.55304	-1.29779	1.47055
H	-0.56619	4.07188	-1.11337
H	-1.13160	4.48376	0.51666
H	-2.20150	4.69378	-0.88612
H	-1.55588	2.01642	-2.32191
H	-3.18877	2.66271	-2.07619
H	-2.77985	1.02219	-1.51889
H	-2.96444	3.07666	1.64971
H	-3.65560	1.67938	0.79806
H	-3.98413	3.32136	0.21950

2Ph-5tBu-exoCl_C34-cyclopropane

45

Cl	-1.33342	-2.05342	2.58765
C	-0.81450	-0.97936	1.22042
C	-0.91066	-1.63213	-0.11264
C	0.20998	-2.24915	-0.67966
C	0.11849	-2.85479	-1.93353
C	-1.09287	-2.84166	-2.62990
C	-2.21445	-2.22493	-2.06903
C	-2.12051	-1.61936	-0.81571
C	0.31112	-0.04833	1.57733
H	0.80569	-0.19244	2.53386
C	1.01591	0.60131	0.46582
C	2.41711	0.59144	0.18271
C	3.01868	1.43917	-0.78276
C	4.38401	1.38399	-1.03597
C	5.21184	0.50141	-0.33209
C	4.63821	-0.33517	0.63045
C	3.26998	-0.30345	0.87815
N	0.08493	1.32288	-0.22955

H	0.32545	1.80803	-1.08668
C	-1.16535	1.34843	0.26284
C	-2.22213	2.29612	-0.25533
C	-1.83875	3.75776	0.08111
C	-2.35781	2.14465	-1.78754
C	-3.57653	1.97159	0.39781
C	-1.19373	0.47225	1.43551
H	-1.83600	0.69448	2.28061
H	1.15162	-2.24543	-0.13959
H	0.99119	-3.33414	-2.36759
H	-1.16283	-3.31068	-3.60725
H	-3.15822	-2.21483	-2.60657
H	-2.98738	-1.13887	-0.37231
H	2.41663	2.16123	-1.32875
H	4.81025	2.04706	-1.78460
H	6.27842	0.46618	-0.53198
H	5.26137	-1.03203	1.18539
H	2.84144	-0.98421	1.60786
H	-0.88085	4.03059	-0.37549
H	-1.74940	3.89592	1.16406
H	-2.60200	4.45066	-0.29262
H	-1.43199	2.41467	-2.30948
H	-3.14526	2.80869	-2.16119
H	-2.61081	1.11441	-2.05658
H	-3.54140	2.10622	1.48392
H	-3.88371	0.94133	0.19043
H	-4.34689	2.64244	0.00333

2Ph-5tBu-exoCl_C4-carbanion_cyclopropanation-intermediate

45

Cl	-2.79108	-0.22498	2.51914
C	-1.99988	0.00316	0.87519
C	-2.10618	-1.11738	-0.00951
C	-3.14311	-2.09355	0.07460
C	-3.30204	-3.08309	-0.88850
C	-2.44162	-3.17960	-1.98886
C	-1.39914	-2.25075	-2.08396
C	-1.22300	-1.26167	-1.12391
C	0.56124	-0.12610	1.35397
H	0.58797	-0.91612	2.09015
C	1.56861	0.24712	0.54079
C	2.92859	-0.24962	0.34195
C	3.96281	0.59377	-0.09613
C	5.25303	0.09480	-0.26874

C	5.52973	-1.24670	-0.00062
C	4.50636	-2.09088	0.44048
C	3.21470	-1.60013	0.60592
N	1.07298	1.34506	-0.24570
H	1.64162	1.83096	-0.93264
C	-0.18787	1.61648	-0.02944
C	-0.95108	2.70410	-0.72715
C	-1.83566	3.42437	0.31456
C	-0.00502	3.70639	-1.40949
C	-1.86490	2.03952	-1.79053
C	-0.66565	0.70747	1.07680
H	-0.80675	1.39684	1.92688
H	-3.83034	-2.05568	0.91299
H	-4.11411	-3.79895	-0.77336
H	-2.56965	-3.95757	-2.73570
H	-0.69552	-2.30617	-2.91257
H	-0.38299	-0.58612	-1.23779
H	3.77554	1.64859	-0.27652
H	6.04318	0.75875	-0.60583
H	6.53520	-1.63340	-0.13581
H	4.71277	-3.13732	0.64385
H	2.41485	-2.26294	0.92104
H	-1.22923	3.90023	1.09242
H	-2.52531	2.71469	0.77920
H	-2.41651	4.20227	-0.19026
H	0.68476	4.16625	-0.69351
H	-0.60000	4.50347	-1.86410
H	0.57532	3.24176	-2.21525
H	-2.52828	1.31385	-1.31681
H	-1.27232	1.53593	-2.55953
H	-2.46206	2.82376	-2.26700

2Ph-5tBu-exoCl_C45-cyclopropane

45

C	-0.37380	-0.57368	-1.74130
C	-0.99775	-0.79304	-0.55812
N	-0.07923	-0.92023	0.49826
H	-0.28884	-1.63660	1.18090
H	-0.85964	-0.35435	-2.68177
C	1.27812	-0.90631	-0.03345
C	1.08958	-0.54633	-1.52296
H	1.78533	-0.88227	-2.28612
C	1.61611	0.49027	-0.53226
C	0.81659	1.67542	-0.10307

C	0.20741	2.50254	-1.05122
C	0.69369	1.98194	1.25657
C	-0.51612	3.62534	-0.64712
H	0.30029	2.25956	-2.10539
C	-0.02818	3.10339	1.66366
H	1.16194	1.33347	1.99059
C	-0.63677	3.92605	0.71153
H	-0.98634	4.26261	-1.39066
H	-0.11928	3.33411	2.72130
H	-1.20275	4.79775	1.02783
Cl	3.35743	0.97412	-0.76020
C	2.25012	-1.94566	0.56472
C	-2.43339	-0.82237	-0.26292
C	-2.90249	-0.50663	1.02300
C	-3.36479	-1.16681	-1.25751
C	-4.26822	-0.53270	1.30504
H	-2.19253	-0.21532	1.79035
C	-4.72749	-1.18632	-0.97444
H	-3.01124	-1.43461	-2.24864
C	-5.18554	-0.87146	0.30906
H	-4.61543	-0.28139	2.30322
H	-5.43464	-1.45689	-1.75335
H	-6.24891	-0.89193	0.52907
C	2.85923	-1.38172	1.86224
H	2.07239	-1.09756	2.57086
H	3.49123	-2.13501	2.34654
H	3.47151	-0.49844	1.66061
C	3.36195	-2.33905	-0.42644
H	2.93634	-2.80256	-1.32295
H	3.97367	-1.49204	-0.73502
H	4.02017	-3.07394	0.04988
C	1.48156	-3.24697	0.89101
H	0.81012	-3.14406	1.75085
H	0.89672	-3.59213	0.03153
H	2.19928	-4.03205	1.14985

4t_plus-carbene

45

C	-3.81132	-0.85034	0.44125
C	-2.44129	-1.03513	0.83438
C	-2.11338	-0.33441	2.02415
C	-1.42007	-1.79138	0.19911
C	-0.83412	-0.39036	2.56183
H	-2.89493	0.25032	2.49705

C	-0.14750	-1.84556	0.73769
H	-1.64858	-2.32547	-0.71611
C	0.15118	-1.13443	1.91103
H	-0.59345	0.16142	3.46454
H	0.62969	-2.42197	0.24715
H	1.16223	-1.15472	2.30512
Cl	-4.21232	-1.79007	-1.04252
C	0.92655	1.32381	0.03009
C	1.67182	1.83163	1.09042
C	2.95747	1.23314	1.05012
C	2.99033	0.36149	-0.02975
N	1.75911	0.44245	-0.63764
H	1.46997	-0.13849	-1.40937
H	1.32756	2.56118	1.81016
H	3.77397	1.41973	1.73283
C	4.07438	-0.55169	-0.55318
C	-0.45329	1.55753	-0.36395
C	-1.30306	2.32632	0.45820
C	-0.99747	1.00911	-1.54210
C	-2.63989	2.51332	0.13023
H	-0.91105	2.75761	1.37324
C	-2.33864	1.19104	-1.86286
H	-0.37473	0.43145	-2.21837
C	-3.17452	1.92925	-1.02190
H	-3.27575	3.09752	0.78934
H	-2.73738	0.74022	-2.76653
H	-4.22287	2.06185	-1.26954
C	5.31682	-0.44412	0.34513
H	6.10546	-1.10583	-0.02818
H	5.70862	0.57854	0.35790
H	5.08626	-0.73582	1.37537
C	4.45349	-0.14391	-1.99427
H	5.24444	-0.79855	-2.37782
H	3.59730	-0.22043	-2.67380
H	4.81638	0.88906	-2.02235
C	3.57667	-2.01430	-0.55058
H	4.36484	-2.68366	-0.91355
H	3.29730	-2.32856	0.46091
H	2.70534	-2.14744	-1.20196

11. Coordinates for pyrroles used in the steric regioselectivity model

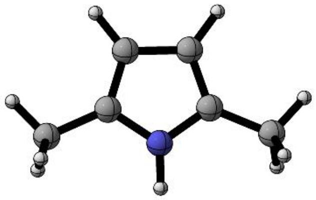

4a_conformer-001

C	-1.13704	0.02342	0.00000
C	-0.71443	-1.29153	0.00000
C	0.71443	-1.29153	-0.00000
C	1.13704	0.02342	-0.00000
N	0.00000	0.80657	-0.00000
H	0.00000	1.81672	0.00000
H	1.36667	-2.15555	-0.00000
H	-1.36667	-2.15555	0.00000
C	2.50768	0.61927	0.00000
H	2.68577	1.24671	-0.88344
H	2.68578	1.24668	0.88346
H	3.25818	-0.17622	-0.00002
C	-2.50768	0.61927	-0.00000
H	-2.68576	1.24673	0.88343
H	-2.68579	1.24666	-0.88347
H	-3.25818	-0.17622	0.00004

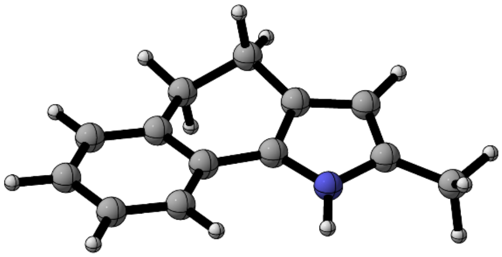

4b_conformer-001

C	-2.85424	-0.44143	0.01662
C	-2.58950	0.90872	-0.16817
N	-1.64133	-1.08620	0.12374
C	-4.15000	-1.17685	0.12093
H	-1.53497	-2.06976	0.32633
H	-3.34044	1.67842	-0.29369
H	-4.23365	-1.96786	-0.63536
H	-4.27548	-1.65168	1.10290
H	-4.98337	-0.48427	-0.02421
C	-1.17871	1.08213	-0.15204
C	-0.61070	-0.17088	0.03345
C	1.03525	2.06871	0.38532
C	-0.29650	2.28003	-0.36086
H	0.83510	2.16213	1.46410

H	1.74983	2.85816	0.12918
H	-0.09991	2.42674	-1.43452
H	-0.77582	3.19918	-0.00584
C	0.81611	-0.42976	0.02645
C	1.65868	0.70642	0.14046
C	3.03990	0.53049	0.09462
C	3.60515	-0.74173	-0.04872
C	2.77423	-1.85808	-0.15093
C	1.38813	-1.70244	-0.12029
H	3.68610	1.40130	0.17960
H	4.68479	-0.85635	-0.08124
H	3.20202	-2.85020	-0.26560
H	0.74834	-2.57448	-0.22897

4b_conformer-002

C	2.85424	-0.44143	0.01662
C	2.58950	0.90872	-0.16817
N	1.64133	-1.08620	0.12374
C	4.15000	-1.17685	0.12093
H	1.53497	-2.06976	0.32633
H	3.34044	1.67842	-0.29369
H	4.27548	-1.65168	1.10290
H	4.23365	-1.96786	-0.63535
H	4.98337	-0.48427	-0.02421
C	1.17871	1.08213	-0.15204
C	0.61070	-0.17088	0.03345
C	-1.03525	2.06871	0.38532
C	0.29650	2.28003	-0.36086
H	-1.74983	2.85816	0.12918
H	-0.83510	2.16213	1.46410
H	0.77582	3.19918	-0.00584
H	0.09991	2.42674	-1.43452
C	-0.81611	-0.42976	0.02645
C	-1.65868	0.70642	0.14046
C	-3.03990	0.53049	0.09462
C	-3.60515	-0.74173	-0.04872
C	-2.77423	-1.85808	-0.15093
C	-1.38813	-1.70244	-0.12029
H	-3.68610	1.40130	0.17960
H	-4.68479	-0.85635	-0.08124
H	-3.20202	-2.85020	-0.26560
H	-0.74834	-2.57448	-0.22897

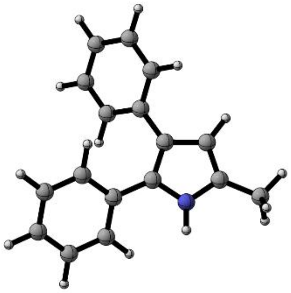

4c_conformer-001

C	0.39756	3.06233	-0.00086
C	-0.83376	2.43810	-0.00080
C	-0.62602	1.02180	-0.00290
C	0.75664	0.81538	0.00796
N	1.34641	2.06647	-0.00851
H	2.33955	2.22150	0.09424
H	-1.79184	2.93911	-0.04213
C	0.76653	4.50956	-0.00033
H	1.35927	4.77997	0.88298
H	1.35671	4.78115	-0.88467
H	-0.13795	5.12347	0.00250
C	1.57838	-0.39503	0.07718
C	2.81526	-0.46181	-0.58991
C	1.16914	-1.50702	0.83626
C	3.61514	-1.60055	-0.50059
H	3.14106	0.37583	-1.20059
C	1.96314	-2.64816	0.91178
H	0.22729	-1.46458	1.37218
C	3.19170	-2.70125	0.24647
H	4.56562	-1.63067	-1.02591
H	1.62810	-3.49543	1.50367
H	3.81168	-3.59060	0.31123
C	-1.69631	0.01459	-0.07805
C	-1.57437	-1.13810	-0.87497
C	-2.89875	0.20859	0.62405
C	-2.60803	-2.06936	-0.95031
H	-0.66303	-1.29545	-1.44256
C	-3.93741	-0.71869	0.54140
H	-3.00936	1.09050	1.24877
C	-3.79602	-1.86536	-0.24279
H	-2.49021	-2.95229	-1.57282
H	-4.85595	-0.54771	1.09635
H	-4.60268	-2.59017	-0.30524

4c_conformer-002

C	-0.39756	3.06233	-0.00086

C	0.83376	2.43810	-0.00080
C	0.62602	1.02180	-0.00290
C	-0.75664	0.81538	0.00796
N	-1.34641	2.06647	-0.00851
H	-2.33955	2.22150	0.09424
H	1.79184	2.93911	-0.04213
C	-0.76653	4.50956	-0.00033
H	-1.35670	4.78115	-0.88467
H	-1.35928	4.77997	0.88297
H	0.13795	5.12347	0.00251
C	-1.57838	-0.39503	0.07718
C	-1.16914	-1.50702	0.83626
C	-2.81526	-0.46181	-0.58991
C	-1.96314	-2.64816	0.91178
H	-0.22729	-1.46458	1.37218
C	-3.61514	-1.60055	-0.50058
H	-3.14106	0.37583	-1.20059
C	-3.19171	-2.70125	0.24647
H	-1.62810	-3.49543	1.50367
H	-4.56562	-1.63067	-1.02591
H	-3.81168	-3.59060	0.31123
C	1.69631	0.01459	-0.07805
C	1.57437	-1.13810	-0.87497
C	2.89875	0.20859	0.62405
C	2.60803	-2.06936	-0.95031
H	0.66303	-1.29545	-1.44257
C	3.93741	-0.71869	0.54140
H	3.00936	1.09050	1.24877
C	3.79602	-1.86536	-0.24279
H	2.49021	-2.95229	-1.57283
H	4.85595	-0.54771	1.09635
H	4.60268	-2.59017	-0.30524

4c_conformer-003

C	-0.39730	3.06232	-0.00083
C	0.83397	2.43800	-0.00082
C	0.62612	1.02170	-0.00294
C	-0.75655	0.81540	0.00793
N	-1.34623	2.06654	-0.00850
H	-2.33936	2.22163	0.09432
H	1.79209	2.93893	-0.04215
C	-0.76613	4.50959	-0.00026
H	-1.35626	4.78127	-0.88460
H	-1.35887	4.78003	0.88304
H	0.13841	5.12341	0.00263

C	-1.57842	-0.39494	0.07712
C	-1.16939	-1.50690	0.83635
C	-2.81524	-0.46160	-0.59009
C	-1.96355	-2.64792	0.91190
H	-0.22758	-1.46451	1.37235
C	-3.61529	-1.60023	-0.50071
H	-3.14085	0.37604	-1.20086
C	-3.19207	-2.70090	0.24649
H	-1.62868	-3.49518	1.50391
H	-4.56573	-1.63027	-1.02611
H	-3.81217	-3.59016	0.31130
C	1.69635	0.01443	-0.07809
C	1.57430	-1.13834	-0.87486
C	2.89884	0.20847	0.62390
C	2.60790	-2.06967	-0.95015
H	0.66292	-1.29573	-1.44238
C	3.93745	-0.71887	0.54131
H	3.00952	1.09045	1.24851
C	3.79595	-1.86564	-0.24272
H	2.49000	-2.95267	-1.57255
H	4.85603	-0.54788	1.09618
H	4.60257	-2.59050	-0.30514

4c_conformer-004

C	-0.39764	3.06233	-0.00084
C	0.83370	2.43814	-0.00078
C	0.62600	1.02182	-0.00294
C	-0.75665	0.81537	0.00787
N	-1.34646	2.06645	-0.00856
H	-2.33961	2.22144	0.09420
H	1.79176	2.93919	-0.04207
C	-0.76663	4.50955	-0.00025
H	-1.35680	4.78118	-0.88459
H	-1.35939	4.77992	0.88306
H	0.13784	5.12348	0.00263
C	-1.57835	-0.39508	0.07710
C	-1.16911	-1.50697	0.83633
C	-2.81519	-0.46195	-0.59005
C	-1.96309	-2.64812	0.91193
H	-0.22729	-1.46444	1.37228
C	-3.61505	-1.60071	-0.50063
H	-3.14096	0.37562	-1.20082
C	-3.19162	-2.70131	0.24657
H	-1.62805	-3.49532	1.50392
H	-4.56551	-1.63090	-1.02599

H	-3.81158	-3.59066	0.31141
C	1.69631	0.01463	-0.07809
C	2.89869	0.20860	0.62411
C	1.57443	-1.13801	-0.87508
C	3.93736	-0.71867	0.54149
H	3.00925	1.09048	1.24888
C	2.60809	-2.06927	-0.95039
H	0.66313	-1.29534	-1.44276
C	3.79603	-1.86530	-0.24277
H	4.85585	-0.54773	1.09652
H	2.49032	-2.95217	-1.57297
H	4.60269	-2.59010	-0.30519

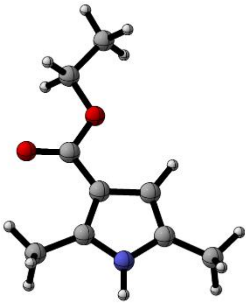

4d_conformer-001

C	-1.49173	1.05056	-0.00002
C	-0.37184	0.21595	0.00001
C	-0.84532	-1.14142	0.00003
C	-2.21714	-1.10813	0.00001
N	-2.58439	0.23072	-0.00002
C	-1.62589	2.53753	-0.00002
H	-0.23141	-2.03018	0.00005
C	-3.23500	-2.20057	0.00000
H	-3.53992	0.56147	-0.00004
H	-3.88383	-2.15477	0.88391
H	-3.88380	-2.15479	-0.88393
H	-2.73519	-3.17276	0.00002
H	-2.17389	2.88804	-0.88372
H	-2.17323	2.88812	0.88406
H	-0.63500	2.99066	-0.00039
C	1.01716	0.65689	0.00002
O	1.41520	1.81698	0.00002
O	1.87264	-0.39681	0.00001
C	3.27972	-0.07564	0.00001
C	4.04201	-1.38604	-0.00004
H	3.50687	0.52841	-0.88414
H	3.50689	0.52833	0.88421
H	5.11883	-1.18783	-0.00004
H	3.79995	-1.97802	0.88848

H	3.79994	-1.97795	-0.88861

4d_conformer-002

C	-1.30943	1.11817	0.04811
C	-0.34213	0.12318	-0.11260
C	-1.02249	-1.14330	-0.11556
C	-2.36398	-0.90027	0.03941
N	-2.51220	0.47687	0.13619
C	-1.20554	2.60576	0.12106
H	-0.56080	-2.11428	-0.21926
C	-3.53618	-1.82243	0.10992
H	-3.39792	0.94927	0.25877
H	-4.07390	-1.72792	1.06192
H	-4.25815	-1.62845	-0.69354
H	-3.19889	-2.85795	0.01579
H	-1.69106	3.08263	-0.74016
H	-1.68988	2.99380	1.02547
H	-0.15548	2.89627	0.12915
C	1.09139	0.34669	-0.24453
O	1.66010	1.43343	-0.24771
O	1.76471	-0.82743	-0.37275
C	3.20318	-0.74278	-0.47859
C	3.85171	-0.64605	0.89324
H	3.49229	-1.66224	-0.99244
H	3.46390	0.11506	-1.10241
H	4.94252	-0.65397	0.79105
H	3.55735	0.28133	1.39240
H	3.55768	-1.49353	1.52092

4d_conformer-003

C	1.30935	1.11818	0.04817
C	0.34213	0.12313	-0.11270
C	1.02259	-1.14330	-0.11569
C	2.36405	-0.90018	0.03939
N	2.51216	0.47696	0.13630
C	1.20536	2.60576	0.12122
H	0.56098	-2.11432	-0.21949
C	3.53631	-1.82226	0.10994
H	3.39783	0.94942	0.25904
H	4.07397	-1.72774	1.06196
H	3.19910	-2.85780	0.01576
H	4.25830	-1.62820	-0.69348
H	1.68918	3.08267	-0.74094
H	0.15527	2.89611	0.13118

H	1.69134	2.99391	1.02469
C	-1.09139	0.34657	-0.24473
O	-1.66012	1.43329	-0.24833
O	-1.76469	-0.82762	-0.37250
C	-3.20317	-0.74303	-0.47840
C	-3.85171	-0.64573	0.89338
H	-3.46388	0.11455	-1.10258
H	-3.49225	-1.66271	-0.99187
H	-3.55734	0.28185	1.39217
H	-4.94252	-0.65368	0.79119
H	-3.55770	-1.49297	1.52141

4d_conformer-004

C	0.81432	1.01364	0.00010
C	0.47062	-0.34090	0.00005
C	1.69031	-1.09699	-0.00006
C	2.73724	-0.20927	-0.00008
N	2.17937	1.06185	0.00002
C	-0.00945	2.26063	0.00018
H	1.76511	-2.17523	-0.00014
C	4.21600	-0.41681	-0.00018
H	2.71387	1.92026	0.00004
H	4.69156	0.02682	-0.88420
H	4.69167	0.02676	0.88380
H	4.43945	-1.48687	-0.00023
H	-0.66079	2.30532	-0.87838
H	-0.66114	2.30495	0.87851
H	0.63177	3.14744	0.00049
C	-0.85890	-0.93144	0.00010
O	-1.09086	-2.13458	0.00012
O	-1.84640	-0.00009	0.00013
C	-3.19915	-0.50245	0.00015
C	-4.12545	0.69749	-0.00043
H	-3.34534	-1.13105	-0.88391
H	-3.34556	-1.13036	0.88467
H	-3.96202	1.31622	0.88783
H	-3.96182	1.31552	-0.88914
H	-5.16746	0.36126	-0.00042

4d_conformer-005

C	-0.91941	1.05402	-0.03599
C	-0.40580	-0.24343	-0.11453
C	-1.51256	-1.15289	-0.02881
C	-2.66024	-0.41081	0.09656

N	-2.27305	0.92236	0.08971
C	-0.26691	2.39823	-0.06750
H	-1.44717	-2.23127	-0.05858
C	-4.09310	-0.81128	0.22441
H	-2.91104	1.70297	0.16880
H	-4.53615	-0.45299	1.16237
H	-4.70356	-0.41545	-0.59722
H	-4.17547	-1.90119	0.20897
H	-1.00765	3.19138	0.07385
H	0.49061	2.48740	0.71697
H	0.24358	2.56996	-1.02092
C	0.98154	-0.65722	-0.25337
O	1.35869	-1.82205	-0.30230
O	1.83667	0.39704	-0.33009
C	3.24401	0.08579	-0.43287
C	3.85172	-0.17536	0.93600
H	3.36973	-0.77430	-1.09421
H	3.68342	0.96843	-0.90276
H	3.68973	0.67959	1.60051
H	3.40609	-1.06469	1.39018
H	4.93085	-0.33857	0.83987

4d_conformer-006

C	0.91941	1.05402	-0.03599
C	0.40580	-0.24343	-0.11453
C	1.51256	-1.15289	-0.02881
C	2.66024	-0.41081	0.09656
N	2.27305	0.92236	0.08971
C	0.26691	2.39823	-0.06750
H	1.44717	-2.23127	-0.05858
C	4.09310	-0.81128	0.22441
H	2.91104	1.70297	0.16880
H	4.70356	-0.41545	-0.59722
H	4.53614	-0.45299	1.16237
H	4.17547	-1.90119	0.20897
H	1.00766	3.19138	0.07384
H	-0.24358	2.56996	-1.02091
H	-0.49061	2.48740	0.71697
C	-0.98154	-0.65722	-0.25337
O	-1.35869	-1.82205	-0.30231
O	-1.83667	0.39704	-0.33009
C	-3.24401	0.08579	-0.43287
C	-3.85172	-0.17536	0.93600
H	-3.68342	0.96843	-0.90276
H	-3.36973	-0.77430	-1.09421

H	-4.93085	-0.33857	0.83987
H	-3.40609	-1.06469	1.39018
H	-3.68973	0.67959	1.60051

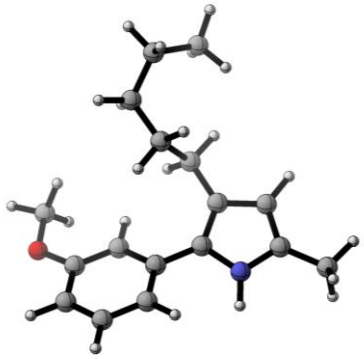

4e_conformer-001

C	2.73853	-2.16716	-0.35096
C	0.68049	-1.31637	0.10900
N	1.39026	-2.28963	-0.58173
H	0.97815	-2.91536	-1.25905
C	2.89741	-1.11576	0.53225
C	3.74030	-3.06078	-1.00630
H	3.55527	-4.11852	-0.77957
H	3.73075	-2.95625	-2.09916
H	4.74580	-2.81260	-0.65599
C	-0.78125	-1.27782	0.03629
C	-1.52095	-2.47509	-0.07518
C	-1.47877	-0.06371	0.05751
C	-2.90788	-2.43552	-0.15623
H	-1.00617	-3.43085	-0.06755
C	-2.87719	-0.03324	-0.00567
H	-0.94800	0.87884	0.09152
C	-3.60506	-1.22351	-0.11754
H	-3.46766	-3.36314	-0.23803
H	-4.68643	-1.22045	-0.17594
O	-3.43593	1.21274	0.02963
C	-4.85375	1.30917	-0.05750
H	-5.08429	2.37455	-0.01726
H	-5.22538	0.89040	-1.00053
H	-5.34129	0.79851	0.78168
C	1.61088	-0.57084	0.83116
C	1.36235	0.54445	1.80989
H	0.28779	0.65859	1.99029
H	1.80857	0.27486	2.77730
C	1.95997	1.89606	1.36953
H	1.86623	2.61350	2.19625
H	3.03694	1.76554	1.19406
C	1.31375	2.48348	0.11318

H	1.33126	1.73308	-0.68921
H	0.25363	2.69634	0.31548
C	1.99302	3.76558	-0.37712
H	1.98697	4.51187	0.42980
H	3.05029	3.55363	-0.58969
C	1.32616	4.35237	-1.62365
H	1.83105	5.26555	-1.95873
H	1.34475	3.63495	-2.45331
H	0.27640	4.60263	-1.42682
H	3.84300	-0.77792	0.93754

4e_conformer-002

C	1.02251	3.30288	-0.26397
C	-0.28398	1.48347	0.12780
N	-0.21770	2.74356	-0.45106
H	-0.94071	3.13275	-1.03912
C	1.75878	2.39924	0.47911
C	1.38210	4.64049	-0.82355
H	1.33624	4.65229	-1.92040
H	0.71030	5.42930	-0.46172
H	2.40116	4.90541	-0.52914
C	-1.52649	0.71122	0.07581
C	-2.77229	1.36081	0.11539
C	-1.49531	-0.69246	-0.03546
C	-3.95321	0.61931	0.04889
H	-2.81684	2.43971	0.22736
C	-2.68303	-1.42553	-0.08152
H	-0.54010	-1.18824	-0.12061
C	-3.92176	-0.76820	-0.04310
H	-4.91127	1.13056	0.08281
H	-4.83235	-1.35674	-0.08846
O	-2.73503	-2.78629	-0.18415
C	-1.50301	-3.49939	-0.21281
H	-0.91726	-3.31980	0.69705
H	-1.76906	-4.55552	-0.27179
H	-0.90189	-3.22543	-1.08839
C	0.94972	1.24947	0.73398
C	1.37467	0.07696	1.57595
H	1.69924	0.44504	2.55915
H	0.52177	-0.58407	1.76538
C	2.53464	-0.73706	0.96672
H	3.38424	-0.06295	0.78761
H	2.87277	-1.47150	1.70814
C	2.17288	-1.44406	-0.34288
H	1.40863	-2.20931	-0.14108

H	1.71290	-0.71482	-1.02238
C	3.36225	-2.10713	-1.05070
H	3.02421	-2.49600	-2.02043
H	4.11864	-1.34138	-1.27379
C	4.00697	-3.24506	-0.25281
H	3.26635	-4.01535	-0.00292
H	4.80859	-3.72504	-0.82555
H	4.44117	-2.88593	0.68668
H	2.77474	2.55252	0.82100

4e_conformer-003

C	3.36248	-1.14003	-0.10871
C	1.11076	-0.82047	-0.11922
N	2.16604	-1.52589	0.44139
H	2.05102	-2.30008	1.07999
C	3.07263	-0.14195	-1.02242
C	4.65892	-1.76897	0.28492
H	5.48303	-1.28670	-0.24763
H	4.85052	-1.67223	1.36124
H	4.68616	-2.84002	0.04500
C	-0.25854	-1.03577	0.34592
C	-0.52044	-1.36368	1.68668
C	-1.33928	-0.91025	-0.54841
C	-1.83516	-1.54905	2.11903
H	0.29727	-1.43666	2.39699
C	-2.65093	-1.07540	-0.10068
H	-1.13402	-0.69302	-1.58788
C	-2.90337	-1.40125	1.24111
H	-2.02900	-1.79523	3.15928
H	-3.93040	-1.52977	1.56704
O	-3.75221	-0.95337	-0.89851
C	-3.55158	-0.57485	-2.25662
H	-4.54692	-0.49689	-2.69584
H	-3.04197	0.39368	-2.32880
H	-2.97123	-1.32962	-2.80088
C	1.66228	0.06828	-1.04454
C	0.97201	1.14273	-1.84810
H	1.74616	1.71314	-2.37736
H	0.34007	0.70355	-2.63287
C	0.11352	2.11498	-1.01557
H	-0.31557	2.87053	-1.68840
H	-0.73168	1.56476	-0.59320
C	0.88161	2.81307	0.11036
H	1.42505	2.05911	0.69476
H	1.64400	3.47289	-0.32758

C	-0.01689	3.62130	1.05800
H	0.61739	4.21493	1.72976
H	-0.60794	4.34398	0.47725
C	-0.95599	2.75143	1.90270
H	-0.38726	2.01508	2.48402
H	-1.53463	3.36125	2.60593
H	-1.66854	2.19569	1.28347
H	3.80181	0.39908	-1.61270

4e_conformer-004

C	-3.36206	-1.14088	-0.10831
C	-1.11043	-0.82072	-0.11888
N	-2.16555	-1.52613	0.44207
H	-2.05039	-2.29979	1.08128
C	-3.07243	-0.14324	-1.02258
C	-4.65836	-1.76988	0.28565
H	-4.68515	-2.84118	0.04681
H	-4.85028	-1.67210	1.36182
H	-5.48252	-1.28845	-0.24761
C	0.25889	-1.03534	0.34646
C	0.52085	-1.36185	1.68754
C	1.33960	-0.91049	-0.54800
C	1.83560	-1.54655	2.12007
H	-0.29687	-1.43425	2.39790
C	2.65128	-1.07497	-0.10012
H	1.13428	-0.69427	-1.58769
C	2.90378	-1.39943	1.24200
H	2.02949	-1.79164	3.16057
H	3.93083	-1.52745	1.56804
O	3.75252	-0.95356	-0.89807
C	3.55179	-0.57658	-2.25658
H	3.04205	0.39180	-2.32983
H	4.54711	-0.49897	-2.69592
H	2.97152	-1.33203	-2.79998
C	-1.66213	0.06734	-1.04473
C	-0.97204	1.14154	-1.84876
H	-0.33976	0.70212	-2.63313
H	-1.74626	1.71140	-2.37853
C	-0.11408	2.11450	-1.01653
H	0.73134	1.56481	-0.59388
H	0.31474	2.87000	-1.68959
C	-0.88262	2.81262	0.10907
H	-1.64546	3.47172	-0.32918
H	-1.42555	2.05856	0.69381
C	0.01532	3.62189	1.05633

H	0.60597	4.34462	0.47524
H	-0.61937	4.21549	1.72774
C	0.95490	2.75304	1.90155
H	0.38658	2.01663	2.48321
H	1.66783	2.19742	1.28267
H	1.53311	3.36358	2.60451
H	-3.80172	0.39725	-1.61322

4e_conformer-005

C	-3.32983	-1.16034	0.29135
C	-1.08427	-0.82787	0.17719
N	-2.13455	-1.67367	-0.15046
H	-2.01422	-2.59782	-0.54005
C	-3.04598	0.05517	0.88585
C	-4.62069	-1.89139	0.11563
H	-4.62395	-2.85331	0.64489
H	-5.44511	-1.29084	0.50940
H	-4.83086	-2.10133	-0.94090
C	0.28468	-1.14716	-0.23003
C	0.53507	-1.76159	-1.46809
C	1.37186	-0.83951	0.61135
C	1.84533	-2.04675	-1.85695
H	-0.29042	-1.98310	-2.13742
C	2.68014	-1.10376	0.20016
H	1.17492	-0.41783	1.58740
C	2.92051	-1.71571	-1.03950
H	2.03066	-2.51748	-2.81840
H	3.94451	-1.91946	-1.33541
O	3.78786	-0.81836	0.94512
C	3.60149	-0.14392	2.18537
H	3.02425	-0.75403	2.89050
H	4.60126	0.02531	2.58721
H	3.09572	0.81878	2.04210
C	-1.63564	0.27699	0.82339
C	-0.94551	1.54303	1.25151
H	-1.18705	1.75983	2.30111
H	0.13953	1.41876	1.20907
C	-1.34349	2.76654	0.39980
H	-2.42275	2.93781	0.50457
H	-0.84985	3.65509	0.81423
C	-1.01013	2.63123	-1.09280
H	-1.59878	1.80561	-1.51275
H	-1.34278	3.54445	-1.60654
C	0.47315	2.39057	-1.41580
H	0.78393	1.41570	-1.02414

H	0.58141	2.32506	-2.50683
C	1.41456	3.47410	-0.88177
H	1.41769	3.50211	0.21388
H	2.44550	3.29622	-1.20864
H	1.11698	4.46901	-1.23677
H	-3.77905	0.72623	1.31590

4e_conformer-006

C	-1.94561	2.69623	-0.19186
C	-0.11517	1.39206	0.15438
N	-0.60574	2.54168	-0.45040
H	-0.07674	3.10789	-1.09819
C	-2.31788	1.64369	0.62308
C	-2.73790	3.82899	-0.75802
H	-2.32758	4.80309	-0.46268
H	-2.76129	3.80541	-1.85539
H	-3.76987	3.77649	-0.40079
C	1.30120	1.04197	0.03518
C	2.28171	2.04692	-0.03058
C	1.70396	-0.30577	-0.03598
C	3.62922	1.70584	-0.15992
H	1.99375	3.09059	0.04930
C	3.05649	-0.63577	-0.14496
H	0.94822	-1.07667	-0.04108
C	4.02775	0.37427	-0.21151
H	4.37975	2.48998	-0.20716
H	5.07243	0.09562	-0.30443
O	3.52520	-1.91660	-0.21384
C	2.57908	-2.97735	-0.13125
H	1.86972	-2.94770	-0.96731
H	3.15831	-3.90028	-0.17998
H	2.02379	-2.94328	0.81414
C	-1.17650	0.81491	0.85045
C	-1.16839	-0.39370	1.74723
H	-0.14463	-0.75665	1.88992
H	-1.52992	-0.09965	2.74241
C	-2.06185	-1.54195	1.23582
H	-2.13142	-2.31570	2.01281
H	-3.07761	-1.15208	1.09507
C	-1.56344	-2.17930	-0.06441
H	-1.39999	-1.39248	-0.81389
H	-0.58325	-2.63952	0.12349
C	-2.50309	-3.24452	-0.64743
H	-1.99005	-3.75170	-1.47523
H	-2.69448	-4.01541	0.11245

C	-3.83519	-2.68466	-1.15725
H	-3.66723	-1.90759	-1.91341
H	-4.44492	-3.47178	-1.61511
H	-4.42553	-2.23814	-0.34975
H	-3.30926	1.48941	1.03035

4e_conformer-007

C	1.94562	2.69622	-0.19186
C	0.11517	1.39206	0.15438
N	0.60576	2.54168	-0.45039
H	0.07676	3.10789	-1.09819
C	2.31789	1.64368	0.62308
C	2.73792	3.82898	-0.75801
H	2.76131	3.80540	-1.85538
H	2.32760	4.80308	-0.46268
H	3.76988	3.77648	-0.40078
C	-1.30120	1.04198	0.03518
C	-2.28170	2.04692	-0.03059
C	-1.70396	-0.30576	-0.03597
C	-3.62922	1.70585	-0.15993
H	-1.99374	3.09060	0.04929
C	-3.05649	-0.63576	-0.14495
H	-0.94822	-1.07666	-0.04107
C	-4.02775	0.37428	-0.21152
H	-4.37974	2.49000	-0.20717
H	-5.07242	0.09564	-0.30444
O	-3.52520	-1.91658	-0.21383
C	-2.57909	-2.97734	-0.13124
H	-2.02379	-2.94326	0.81415
H	-3.15832	-3.90027	-0.17997
H	-1.86972	-2.94770	-0.96730
C	1.17650	0.81490	0.85046
C	1.16838	-0.39371	1.74724
H	1.52991	-0.09966	2.74241
H	0.14463	-0.75665	1.88992
C	2.06184	-1.54197	1.23582
H	3.07760	-1.15209	1.09507
H	2.13140	-2.31571	2.01281
C	1.56343	-2.17931	-0.06441
H	0.58323	-2.63953	0.12349
H	1.39998	-1.39248	-0.81389
C	2.50308	-3.24453	-0.64744
H	2.69447	-4.01542	0.11244
H	1.99004	-3.75170	-1.47524
C	3.83518	-2.68466	-1.15726

H	3.66720	-1.90759	-1.91342
H	4.42552	-2.23814	-0.34977
H	4.44490	-3.47178	-1.61513
H	3.30926	1.48940	1.03036

4e_conformer-008

C	-3.14279	-1.23825	-0.23697
C	-0.92503	-1.00024	0.20519
N	-1.88711	-1.74314	-0.46701
H	-1.67369	-2.47106	-1.13372
C	-2.99032	-0.16850	0.62562
C	-4.36204	-1.82312	-0.87159
H	-5.25186	-1.28493	-0.53414
H	-4.32557	-1.75762	-1.96687
H	-4.48990	-2.88214	-0.61351
C	0.48850	-1.36649	0.11036
C	0.86764	-2.71449	-0.01288
C	1.49164	-0.37700	0.11502
C	2.21577	-3.05832	-0.12455
H	0.11145	-3.49310	0.00816
C	2.83862	-0.73416	0.02116
H	1.20239	0.66211	0.16045
C	3.20622	-2.08219	-0.10300
H	2.49783	-4.10349	-0.21640
H	4.25828	-2.33608	-0.18215
O	3.86813	0.16302	0.02303
C	3.54946	1.54441	0.14880
H	3.01432	1.74756	1.08444
H	4.50322	2.07351	0.15304
H	2.94350	1.89317	-0.69644
C	-1.60066	-0.00508	0.90994
C	-1.04047	1.01150	1.86712
H	-1.52674	0.87002	2.84281
H	0.02664	0.82976	2.03555
C	-1.25046	2.48699	1.46248
H	-2.31281	2.64748	1.23248
H	-1.03126	3.10488	2.34325
C	-0.39555	2.99995	0.29415
H	0.66328	2.78096	0.49926
H	-0.46761	4.09685	0.27124
C	-0.77005	2.46873	-1.09543
H	-0.66361	1.38007	-1.12460
H	-1.83434	2.67242	-1.27805
C	0.07432	3.09786	-2.20636
H	1.13976	2.87480	-2.06615

H	-0.21551	2.72038	-3.19358
H	-0.03398	4.18960	-2.21845
H	-3.79788	0.43281	1.02395

4e_conformer-009

C	3.14280	-1.23824	-0.23700
C	0.92504	-1.00023	0.20518
N	1.88711	-1.74313	-0.46703
H	1.67369	-2.47107	-1.13372
C	2.99033	-0.16849	0.62559
C	4.36204	-1.82312	-0.87162
H	4.48990	-2.88213	-0.61354
H	4.32556	-1.75763	-1.96690
H	5.25186	-1.28493	-0.53419
C	-0.48849	-1.36649	0.11037
C	-0.86761	-2.71450	-0.01286
C	-1.49164	-0.37701	0.11502
C	-2.21575	-3.05834	-0.12452
H	-0.11142	-3.49311	0.00818
C	-2.83861	-0.73418	0.02117
H	-1.20239	0.66209	0.16043
C	-3.20620	-2.08222	-0.10297
H	-2.49779	-4.10352	-0.21636
H	-4.25826	-2.33612	-0.18212
O	-3.86813	0.16299	0.02303
C	-3.54946	1.54438	0.14875
H	-3.01432	1.74757	1.08437
H	-2.94351	1.89312	-0.69651
H	-4.50322	2.07348	0.15299
C	1.60067	-0.00507	0.90992
C	1.04049	1.01150	1.86711
H	-0.02662	0.82976	2.03555
H	1.52677	0.87001	2.84280
C	1.25048	2.48700	1.46249
H	1.03130	3.10487	2.34327
H	2.31282	2.64748	1.23247
C	0.39554	2.99998	0.29418
H	-0.66329	2.78099	0.49931
H	0.46760	4.09687	0.27129
C	0.77000	2.46877	-1.09542
H	1.83429	2.67247	-1.27805
H	0.66356	1.38011	-1.12458
C	-0.07440	3.09791	-2.20631
H	-1.13983	2.87486	-2.06606
H	0.03392	4.18966	-2.21840

H	0.21540	2.72044	-3.19354
H	3.79789	0.43282	1.02391

4e_conformer-010

C	-3.72691	-0.83878	0.32804
C	-1.68247	0.13506	0.11284
N	-3.05585	0.32987	0.06899
H	-3.49475	1.23576	-0.01489
C	-2.76363	-1.81500	0.51377
C	-5.21819	-0.90309	0.38629
H	-5.53915	-1.93141	0.57315
H	-5.62584	-0.27380	1.18832
H	-5.67904	-0.57134	-0.55290
C	-0.76534	1.24021	-0.16481
C	-1.13099	2.26997	-1.04892
C	0.49665	1.29781	0.45969
C	-0.25238	3.32522	-1.30046
H	-2.08571	2.22935	-1.56402
C	1.37250	2.35107	0.19076
H	0.76728	0.52792	1.16796
C	0.99760	3.37440	-0.69346
H	-0.54325	4.11359	-1.98915
H	1.69248	4.18574	-0.88440
O	2.60953	2.48089	0.75429
C	3.02385	1.49411	1.69414
H	3.06623	0.49925	1.23700
H	4.02344	1.78927	2.01589
H	2.35446	1.46495	2.56250
C	-1.47499	-1.21850	0.38682
C	-0.17124	-1.97109	0.41493
H	0.51528	-1.54663	1.15627
H	-0.36865	-2.99790	0.74893
C	0.52060	-2.02381	-0.96054
H	-0.17102	-2.49388	-1.67150
H	0.68424	-0.99966	-1.31953
C	1.85429	-2.78167	-0.96283
H	1.70636	-3.78840	-0.54446
H	2.17841	-2.92775	-2.00279
C	2.97896	-2.07430	-0.19742
H	3.09956	-1.05957	-0.60265
H	2.69538	-1.95301	0.85614
C	4.31323	-2.82006	-0.27543
H	4.22562	-3.82772	0.14908
H	4.64387	-2.92689	-1.31585
H	5.10073	-2.29237	0.27450

H	-2.96049	-2.86082	0.71464

4e_conformer-011

C	2.70096	-1.93678	-0.60326
C	0.95659	-0.94822	0.46844
N	1.34891	-1.98766	-0.36243
H	0.74432	-2.74909	-0.63695
C	3.18008	-0.82159	0.05929
C	3.40012	-2.97030	-1.42468
H	3.33859	-3.96821	-0.97086
H	2.97591	-3.04596	-2.43406
H	4.45832	-2.71357	-1.52347
C	-0.45320	-0.76267	0.82067
C	-0.84001	-0.36462	2.10892
C	-1.44612	-0.98429	-0.15453
C	-2.19080	-0.17262	2.40524
H	-0.08913	-0.23133	2.87933
C	-2.79585	-0.80263	0.15646
H	-1.14260	-1.26239	-1.15630
C	-3.17179	-0.38817	1.44296
H	-2.48313	0.13642	3.40494
H	-4.22504	-0.24550	1.66204
O	-3.81818	-0.98804	-0.72915
C	-3.48819	-1.38883	-2.05501
H	-2.86364	-0.63828	-2.55463
H	-2.96841	-2.35450	-2.06282
H	-4.43727	-1.48247	-2.58429
C	2.09417	-0.19097	0.74022
C	2.18018	1.13125	1.45331
H	1.40938	1.20965	2.22653
H	3.14585	1.19414	1.97109
C	2.04630	2.34341	0.50659
H	2.24464	3.26086	1.07868
H	2.82443	2.28222	-0.26710
C	0.67204	2.44780	-0.15921
H	0.47303	1.52974	-0.72610
H	-0.10422	2.49462	0.61833
C	0.53205	3.65491	-1.09023
H	0.72150	4.57849	-0.52505
H	1.30974	3.60640	-1.86569
C	-0.84686	3.73108	-1.75123
H	-1.63942	3.81279	-0.99714
H	-0.92878	4.59639	-2.41889
H	-1.04787	2.82952	-2.34324
H	4.20875	-0.48265	0.05229

4e_conformer-012

C	-2.70110	-1.93666	-0.60322
C	-0.95668	-0.94815	0.46845
N	-1.34905	-1.98762	-0.36236
H	-0.74450	-2.74910	-0.63682
C	-3.18015	-0.82141	0.05926
C	-3.40030	-2.97019	-1.42459
H	-4.45847	-2.71337	-1.52346
H	-2.97605	-3.04597	-2.43394
H	-3.33890	-3.96807	-0.97069
C	0.45311	-0.76267	0.82068
C	0.83994	-0.36455	2.10891
C	1.44603	-0.98440	-0.15450
C	2.19073	-0.17261	2.40522
H	0.08906	-0.23117	2.87930
C	2.79577	-0.80279	0.15649
H	1.14251	-1.26256	-1.15625
C	3.17172	-0.38827	1.44297
H	2.48307	0.13648	3.40491
H	4.22498	-0.24565	1.66205
O	3.81810	-0.98831	-0.72910
C	3.48811	-1.38917	-2.05494
H	2.86360	-0.63862	-2.55461
H	4.43719	-1.48289	-2.58420
H	2.96828	-2.35482	-2.06269
C	-2.09422	-0.19081	0.74017
C	-2.18017	1.13144	1.45321
H	-3.14587	1.19442	1.97092
H	-1.40941	1.20982	2.22648
C	-2.04613	2.34355	0.50646
H	-2.82423	2.28240	-0.26729
H	-2.24445	3.26103	1.07850
C	-0.67183	2.44784	-0.15926
H	0.10439	2.49460	0.61831
H	-0.47286	1.52975	-0.72613
C	-0.53171	3.65492	-1.09030
H	-1.30935	3.60645	-1.86580
H	-0.72114	4.57853	-0.52513
C	0.84724	3.73100	-1.75121
H	1.63977	3.81268	-0.99707
H	1.04824	2.82943	-2.34320
H	0.92925	4.59630	-2.41888
H	-4.20881	-0.48240	0.05223

4e_conformer-013

C	3.39387	-0.79462	-0.41278
C	1.17730	-0.87123	0.09122
N	2.23074	-1.51064	-0.54918
H	2.11948	-2.32014	-1.14288
C	3.09180	0.31076	0.35952
C	4.67379	-1.23091	-1.04763
H	4.96597	-2.23897	-0.72704
H	4.60458	-1.24560	-2.14325
H	5.47877	-0.54386	-0.77320
C	-0.14607	-1.50147	0.11104
C	-0.24942	-2.89980	0.20874
C	-1.32459	-0.73629	0.00596
C	-1.50188	-3.51555	0.20368
H	0.64843	-3.50010	0.31706
C	-2.57427	-1.36306	0.01878
H	-1.24400	0.33449	-0.12122
C	-2.66500	-2.75999	0.11521
H	-1.57066	-4.59693	0.28234
H	-3.64659	-3.22267	0.11537
O	-3.76733	-0.70692	-0.08005
C	-3.74806	0.71287	-0.16804
H	-3.21179	1.05179	-1.06178
H	-4.79152	1.02411	-0.23234
H	-3.28877	1.15967	0.72169
C	1.69971	0.27720	0.68434
C	1.01231	1.26406	1.59087
H	-0.05568	1.03541	1.66285
H	1.41057	1.15511	2.61000
C	1.21852	2.72996	1.15584
H	0.83129	3.39451	1.94036
H	2.29658	2.92345	1.09572
C	0.56068	3.08473	-0.18450
H	1.01856	3.99719	-0.58988
H	0.77331	2.28639	-0.90924
C	-0.95286	3.30164	-0.08545
H	-1.41785	2.44438	0.41674
H	-1.14829	4.16728	0.56239
C	-1.61617	3.51606	-1.44734
H	-1.47008	2.64409	-2.09705
H	-2.69450	3.68573	-1.34957
H	-1.18604	4.38447	-1.96121
H	3.80371	1.06398	0.67203

4e_conformer-014

C	-3.39375	-0.79505	-0.41262
C	-1.17714	-0.87133	0.09122
N	-2.23052	-1.51089	-0.54912
H	-2.11918	-2.32034	-1.14286
C	-3.09179	0.31037	0.35969
C	-4.67364	-1.23151	-1.04740
H	-4.96561	-2.23964	-0.72688
H	-5.47871	-0.54461	-0.77285
H	-4.60451	-1.24609	-2.14304
C	0.14631	-1.50142	0.11099
C	0.24984	-2.89975	0.20840
C	1.32475	-0.73607	0.00612
C	1.50237	-3.51535	0.20327
H	-0.64794	-3.50020	0.31659
C	2.57450	-1.36270	0.01886
H	1.24405	0.33472	-0.12088
C	2.66541	-2.75963	0.11500
H	1.57127	-4.59674	0.28169
H	3.64705	-3.22219	0.11511
O	3.76749	-0.70639	-0.07978
C	3.74806	0.71341	-0.16747
H	3.21183	1.05246	-1.06118
H	3.28864	1.15998	0.72231
H	4.79149	1.02478	-0.23162
C	-1.69968	0.27701	0.68440
C	-1.01238	1.26396	1.59092
H	-1.41059	1.15494	2.61007
H	0.05564	1.03544	1.66286
C	-1.21880	2.72982	1.15590
H	-2.29691	2.92320	1.09595
H	-0.83152	3.39443	1.94035
C	-0.56125	3.08463	-0.18456
H	-0.77397	2.28627	-0.90925
H	-1.01926	3.99706	-0.58986
C	0.95230	3.30164	-0.08581
H	1.14780	4.16733	0.56195
H	1.41744	2.44443	0.41632
C	1.61532	3.51604	-1.44785
H	1.18503	4.38438	-1.96168
H	2.69366	3.68579	-1.35030
H	1.46917	2.64401	-2.09748
H	-3.80379	1.06347	0.67227

4e_conformer-015

C	1.28830	2.96446	-0.09381
C	-0.29402	1.34622	0.12806
N	-0.00702	2.60763	-0.37479
H	-0.63258	3.13826	-0.96381
C	1.83998	1.92242	0.62924
C	1.87363	4.25950	-0.55364
H	2.89774	4.35700	-0.18351
H	1.90496	4.33017	-1.64880
H	1.29917	5.12042	-0.18871
C	-1.63355	0.77680	-0.00526
C	-2.76377	1.61034	-0.08272
C	-1.81770	-0.61901	-0.06439
C	-4.03797	1.05713	-0.21607
H	-2.65114	2.68728	-0.00586
C	-3.09917	-1.16192	-0.17706
H	-0.95208	-1.26399	-0.05327
C	-4.21912	-0.32146	-0.25901
H	-4.90355	1.71129	-0.27279
H	-5.20524	-0.76416	-0.35441
O	-3.35575	-2.50213	-0.23145
C	-2.25190	-3.39666	-0.13522
H	-1.70790	-3.25930	0.80718
H	-2.67669	-4.40074	-0.16791
H	-1.55741	-3.26714	-0.97412
C	0.85928	0.89539	0.77394
C	1.04815	-0.39057	1.53081
H	1.68377	-0.19811	2.40580
H	0.08349	-0.73544	1.92113
C	1.69158	-1.53153	0.71409
H	1.67901	-2.44613	1.32339
H	1.07801	-1.74125	-0.17317
C	3.12616	-1.24464	0.26449
H	3.14036	-0.33579	-0.35089
H	3.74608	-1.02704	1.14719
C	3.75228	-2.39970	-0.52273
H	3.73874	-3.30965	0.09365
H	3.12839	-2.61678	-1.40132
C	5.18563	-2.10622	-0.97267
H	5.22138	-1.21851	-1.61615
H	5.83713	-1.91613	-0.11080
H	5.61098	-2.94513	-1.53522
H	2.84561	1.90118	1.02917

4e_conformer-016

C	-1.71931	2.52761	0.10703

C	-0.23584	0.87319	0.59595
N	-0.40904	2.12865	0.02916
H	0.35008	2.71860	-0.28074
C	-2.41113	1.49386	0.71331
C	-2.17667	3.85874	-0.39291
H	-1.70180	4.68712	0.14900
H	-1.95031	3.99508	-1.45810
H	-3.25837	3.95246	-0.26455
C	1.07961	0.23491	0.59986
C	1.45379	-0.66942	1.61571
C	2.01027	0.52327	-0.40895
C	2.71254	-1.25731	1.59877
H	0.76534	-0.88876	2.42294
C	3.28218	-0.06177	-0.41071
H	1.75568	1.18444	-1.23126
C	3.64301	-0.96459	0.59587
H	2.98981	-1.95098	2.38778
H	4.61872	-1.43407	0.60958
O	4.09303	0.30264	-1.44765
C	5.39151	-0.27814	-1.51249
H	5.98914	-0.01981	-0.63003
H	5.33658	-1.36952	-1.60502
H	5.86157	0.13889	-2.40407
C	-1.49363	0.44963	1.03111
C	-1.88946	-0.88342	1.61101
H	-1.37399	-1.07437	2.56089
H	-2.95556	-0.83804	1.86146
C	-1.63806	-2.08051	0.66964
H	-0.55675	-2.22212	0.55749
H	-2.02383	-2.98881	1.15352
C	-2.26540	-1.93554	-0.72215
H	-1.82689	-1.06264	-1.22392
H	-1.98832	-2.81087	-1.32660
C	-3.79104	-1.79611	-0.71987
H	-4.23304	-2.64330	-0.17584
H	-4.07643	-0.89214	-0.16782
C	-4.37744	-1.72728	-2.13212
H	-4.13614	-2.63149	-2.70465
H	-5.46850	-1.62462	-2.11169
H	-3.97096	-0.86955	-2.68234
H	-3.47649	1.48008	0.90672

4e_conformer-017

C	1.71911	2.52765	0.10700
C	0.23569	0.87319	0.59595

N	0.40884	2.12865	0.02915
H	-0.35030	2.71857	-0.28075
C	2.41096	1.49392	0.71327
C	2.17642	3.85879	-0.39295
H	1.70152	4.68715	0.14895
H	3.25812	3.95255	-0.26460
H	1.95005	3.99512	-1.45815
C	-1.07977	0.23490	0.59988
C	-1.45402	-0.66925	1.61586
C	-2.01035	0.52309	-0.40904
C	-2.71277	-1.25714	1.59893
H	-0.76563	-0.88843	2.42320
C	-3.28226	-0.06193	-0.41079
H	-1.75569	1.18413	-1.23144
C	-3.64317	-0.96458	0.59592
H	-2.99011	-1.95066	2.38805
H	-4.61888	-1.43405	0.60964
O	-4.09304	0.30231	-1.44785
C	-5.39153	-0.27847	-1.51267
H	-5.98921	-0.01997	-0.63030
H	-5.86152	0.13842	-2.40435
H	-5.33661	-1.36986	-1.60502
C	1.49350	0.44966	1.03109
C	1.88938	-0.88340	1.61094
H	2.95544	-0.83793	1.86156
H	1.37378	-1.07448	2.56072
C	1.63822	-2.08043	0.66945
H	2.02392	-2.98876	1.15336
H	0.55694	-2.22208	0.55706
C	2.26586	-1.93538	-0.72221
H	1.98883	-2.81063	-1.32679
H	1.82751	-1.06240	-1.22398
C	3.79151	-1.79608	-0.71961
H	4.07688	-0.89222	-0.16738
H	4.23333	-2.64338	-0.17563
C	4.37818	-1.72710	-2.13175
H	3.97188	-0.86925	-2.68192
H	5.46924	-1.62455	-2.11110
H	4.13689	-2.63120	-2.70445
H	3.47633	1.48016	0.90666

4e_conformer-018

C	-1.75917	3.15345	-0.20837
C	-1.36408	0.93282	0.07859
N	-2.27106	1.88823	-0.35798

H	-3.13768	1.67338	-0.82980
C	-0.50702	3.01201	0.36272
C	-2.51509	4.37035	-0.63174
H	-1.93552	5.26781	-0.39947
H	-2.71866	4.37253	-1.71054
H	-3.48185	4.45082	-0.11845
C	-1.69486	-0.49029	0.04751
C	-3.02706	-0.92603	0.16038
C	-0.67871	-1.45491	-0.10385
C	-3.32593	-2.28890	0.12316
H	-3.82452	-0.20461	0.30939
C	-0.98687	-2.81616	-0.11826
H	0.34018	-1.12694	-0.24187
C	-2.31942	-3.24035	-0.00937
H	-4.35867	-2.61401	0.21364
H	-2.53892	-4.30286	-0.03079
O	-0.05398	-3.80482	-0.25182
C	1.31522	-3.42209	-0.33814
H	1.88219	-4.35140	-0.40550
H	1.50624	-2.81324	-1.23018
H	1.63048	-2.86449	0.55229
C	-0.24532	1.62230	0.54915
C	0.99729	1.04400	1.16465
H	1.33813	1.69877	1.97783
H	0.77115	0.07453	1.62552
C	2.15653	0.86754	0.16573
H	1.81081	0.28244	-0.69826
H	2.43159	1.85233	-0.23152
C	3.37442	0.17878	0.78852
H	3.76865	0.80345	1.60359
H	3.04592	-0.76092	1.25551
C	4.50026	-0.13096	-0.20762
H	5.27721	-0.71217	0.30638
H	4.10613	-0.78108	-1.00186
C	5.13776	1.11268	-0.83520
H	5.52447	1.78692	-0.06066
H	5.97352	0.84052	-1.48971
H	4.41730	1.67763	-1.43674
H	0.15344	3.82610	0.63437

4e_conformer-019

C	1.75921	3.15343	-0.20837
C	1.36409	0.93281	0.07858
N	2.27108	1.88821	-0.35798
H	3.13770	1.67334	-0.82980

C	0.50706	3.01201	0.36272
C	2.51514	4.37032	-0.63174
H	3.48190	4.45078	-0.11845
H	2.71871	4.37250	-1.71054
H	1.93558	5.26779	-0.39948
C	1.69485	-0.49031	0.04751
C	3.02705	-0.92606	0.16039
C	0.67870	-1.45492	-0.10386
C	3.32591	-2.28893	0.12317
H	3.82452	-0.20465	0.30939
C	0.98684	-2.81617	-0.11826
H	-0.34019	-1.12694	-0.24188
C	2.31938	-3.24037	-0.00936
H	4.35864	-2.61406	0.21365
H	2.53887	-4.30289	-0.03079
O	0.05394	-3.80482	-0.25182
C	-1.31526	-3.42207	-0.33815
H	-1.50627	-2.81323	-1.23019
H	-1.88223	-4.35138	-0.40551
H	-1.63052	-2.86447	0.55227
C	0.24534	1.62230	0.54915
C	-0.99728	1.04402	1.16465
H	-0.77115	0.07455	1.62552
H	-1.33811	1.69879	1.97783
C	-2.15652	0.86756	0.16573
H	-2.43157	1.85235	-0.23152
H	-1.81081	0.28246	-0.69826
C	-3.37441	0.17882	0.78852
H	-3.04592	-0.76089	1.25551
H	-3.76864	0.80348	1.60359
C	-4.50026	-0.13092	-0.20762
H	-4.10614	-0.78104	-1.00186
H	-5.27721	-0.71213	0.30638
C	-5.13775	1.11273	-0.83520
H	-4.41729	1.67767	-1.43674
H	-5.97351	0.84058	-1.48970
H	-5.52445	1.78697	-0.06065
H	-0.15340	3.82611	0.63437

4e_conformer-020

C	2.08195	-2.52924	-0.52358
C	0.51626	-1.17499	0.41624
N	0.74297	-2.22885	-0.45732
H	0.00861	-2.78001	-0.87859
C	2.73200	-1.62856	0.29988

C	2.60167	-3.65873	-1.35168
H	2.21202	-4.62874	-1.01588
H	2.33060	-3.54847	-2.40945
H	3.69243	-3.69660	-1.28663
C	-0.83605	-0.64846	0.61340
C	-1.27993	-0.21499	1.87903
C	-1.72510	-0.57825	-0.46756
C	-2.56689	0.28753	2.03142
H	-0.62500	-0.30323	2.73814
C	-3.02602	-0.08667	-0.30292
H	-1.41208	-0.87799	-1.46284
C	-3.45467	0.35926	0.95264
H	-2.90117	0.61837	3.01087
H	-4.45355	0.75052	1.10080
O	-3.79403	-0.06954	-1.43194
C	-5.11967	0.43987	-1.32759
H	-5.12031	1.48799	-1.00494
H	-5.54750	0.36759	-2.32832
H	-5.72193	-0.15384	-0.62941
C	1.75922	-0.77221	0.90054
C	2.08487	0.42758	1.74778
H	1.22670	0.71666	2.36213
H	2.89202	0.16620	2.44443
C	2.52541	1.65062	0.91602
H	2.87175	2.43872	1.59997
H	3.38911	1.36606	0.30194
C	1.40772	2.20885	0.02954
H	1.06905	1.42602	-0.66166
H	0.54263	2.44643	0.66431
C	1.80279	3.45689	-0.77180
H	0.90843	3.85321	-1.27102
H	2.14057	4.24210	-0.08035
C	2.88753	3.20203	-1.82371
H	2.57855	2.41081	-2.51844
H	3.08884	4.10456	-2.41202
H	3.83211	2.88906	-1.36552
H	3.80307	-1.58028	0.45323

4e_conformer-021

C	-2.08178	-2.52928	-0.52357
C	-0.51612	-1.17500	0.41624
N	-0.74281	-2.22885	-0.45735
H	-0.00844	-2.77996	-0.87865
C	-2.73184	-1.62865	0.29992
C	-2.60148	-3.65877	-1.35170

H	-3.69224	-3.69668	-1.28662
H	-2.33044	-3.54848	-2.40947
H	-2.21179	-4.62877	-1.01593
C	0.83617	-0.64843	0.61339
C	1.28008	-0.21501	1.87903
C	1.72519	-0.57812	-0.46760
C	2.56702	0.28756	2.03141
H	0.62517	-0.30332	2.73815
C	3.02610	-0.08650	-0.30297
H	1.41215	-0.87782	-1.46288
C	3.45476	0.35938	0.95260
H	2.90131	0.61835	3.01086
H	4.45363	0.75067	1.10075
O	3.79407	-0.06926	-1.43201
C	5.11969	0.44021	-1.32767
H	5.72200	-0.15352	-0.62955
H	5.54749	0.36800	-2.32843
H	5.12028	1.48831	-1.00497
C	-1.75909	-0.77229	0.90058
C	-2.08475	0.42748	1.74786
H	-2.89178	0.16602	2.44463
H	-1.22653	0.71664	2.36210
C	-2.52552	1.65046	0.91615
H	-3.38927	1.36581	0.30219
H	-2.87186	2.43853	1.60014
C	-1.40800	2.20879	0.02953
H	-0.54287	2.44651	0.66419
H	-1.06930	1.42597	-0.66168
C	-1.80331	3.45675	-0.77183
H	-2.14113	4.24194	-0.08038
H	-0.90905	3.85317	-1.27115
C	-2.88812	3.20170	-1.82362
H	-3.83261	2.88861	-1.36533
H	-3.08961	4.10419	-2.41194
H	-2.57910	2.41051	-2.51836
H	-3.80291	-1.58042	0.45330

4e_conformer-022

C	3.37668	-1.33890	-0.02128
C	1.15715	-0.86433	-0.16557
N	2.12526	-1.64346	0.45232
H	1.91901	-2.41034	1.07664
C	3.21236	-0.31918	-0.94218
C	4.60067	-2.05689	0.44528
H	4.57026	-3.12603	0.19738

H	5.48687	-1.62914	-0.03125
H	4.73160	-1.97905	1.53218
C	-0.24927	-0.98594	0.21611
C	-0.60969	-1.28988	1.54588
C	-1.26836	-0.79582	-0.72534
C	-1.95029	-1.38202	1.90224
H	0.16086	-1.41462	2.30027
C	-2.61586	-0.87294	-0.35565
H	-1.03095	-0.59046	-1.76311
C	-2.96801	-1.17058	0.96684
H	-2.21825	-1.60732	2.93077
H	-4.00469	-1.23809	1.27239
O	-3.51309	-0.65446	-1.36186
C	-4.89931	-0.72878	-1.04536
H	-5.17269	-1.72570	-0.67911
H	-5.43005	-0.52577	-1.97642
H	-5.17827	0.02109	-0.29529
C	1.82367	-0.01189	-1.04764
C	1.25715	1.11073	-1.88103
H	2.09777	1.62882	-2.36046
H	0.64245	0.71868	-2.70323
C	0.42075	2.13542	-1.08998
H	0.08115	2.92080	-1.77948
H	-0.48140	1.64099	-0.71933
C	1.17040	2.77602	0.08167
H	1.62950	1.98531	0.68940
H	1.99815	3.38485	-0.30902
C	0.27752	3.63953	0.98486
H	-0.22980	4.40343	0.37828
H	0.91235	4.18551	1.69543
C	-0.76418	2.83231	1.76924
H	-0.28038	2.05592	2.37483
H	-1.33792	3.47722	2.44467
H	-1.47746	2.32977	1.10696
H	4.01165	0.17277	-1.48251

4e_conformer-023

C	3.63683	-1.26937	0.14805
C	1.73952	-0.01335	0.09292
N	3.10809	-0.03738	-0.14089
H	3.65755	0.77778	-0.37200
C	2.58152	-2.06723	0.55383
C	5.09644	-1.55891	0.01852
H	5.45715	-1.39468	-1.00493
H	5.70097	-0.92651	0.68172

H	5.29328	-2.60188	0.28067
C	0.96116	1.19506	-0.17313
C	1.40843	2.15323	-1.10047
C	-0.25621	1.42625	0.49915
C	0.65788	3.30513	-1.34123
H	2.32236	1.98609	-1.66160
C	-1.01097	2.56893	0.23183
H	-0.59296	0.71380	1.23738
C	-0.55055	3.52270	-0.68839
H	1.01442	4.03541	-2.06232
H	-1.15024	4.40700	-0.87759
O	-2.20740	2.84816	0.82890
C	-2.75377	1.87600	1.71426
H	-3.71680	2.27317	2.03767
H	-2.90540	0.91545	1.20623
H	-2.11072	1.72338	2.58944
C	1.38510	-1.29381	0.53034
C	0.01544	-1.83359	0.84754
H	0.12635	-2.86391	1.20856
H	-0.44350	-1.28270	1.68028
C	-0.95414	-1.82850	-0.34695
H	-1.00341	-0.81863	-0.77130
H	-0.54893	-2.47643	-1.13602
C	-2.36496	-2.28454	0.03034
H	-2.32538	-3.29997	0.45161
H	-2.74989	-1.63746	0.83334
C	-3.34453	-2.25911	-1.14664
H	-3.37664	-1.24305	-1.56459
H	-2.96397	-2.90763	-1.94811
C	-4.75837	-2.69827	-0.75811
H	-5.17261	-2.04510	0.01985
H	-5.43886	-2.66938	-1.61671
H	-4.75834	-3.72230	-0.36481
H	2.65521	-3.10982	0.83713

4e_conformer-024

C	-0.15197	3.62902	-0.21232
C	-0.31211	1.41847	0.30329
N	-0.99828	2.55497	-0.10279
H	-2.00371	2.61021	-0.18160
C	1.11393	3.16832	0.10388
C	-0.63772	4.98871	-0.59522
H	-1.13252	4.98565	-1.57485
H	-1.35771	5.38749	0.13134
H	0.20464	5.68377	-0.64645

C	-1.01252	0.14468	0.46120
C	-0.55714	-0.83934	1.36402
C	-2.17144	-0.12363	-0.28249
C	-1.24515	-2.03907	1.49628
H	0.31925	-0.65132	1.97189
C	-2.86725	-1.32905	-0.13268
H	-2.54087	0.58447	-1.01753
C	-2.40456	-2.30300	0.75945
H	-0.88447	-2.78770	2.19632
H	-2.92307	-3.24526	0.88536
O	-3.97889	-1.46453	-0.91473
C	-4.71480	-2.67982	-0.81981
H	-5.10676	-2.83138	0.19313
H	-5.54568	-2.58185	-1.51969
H	-4.09925	-3.54248	-1.10198
C	1.03008	1.78453	0.43519
C	2.21306	0.91006	0.75612
H	3.10266	1.54845	0.83095
H	2.10523	0.44048	1.74311
C	2.47657	-0.18643	-0.29019
H	1.57837	-0.80694	-0.39814
H	2.64436	0.28861	-1.26648
C	3.66854	-1.07722	0.06593
H	4.56968	-0.45678	0.18145
H	3.49142	-1.54334	1.04673
C	3.93703	-2.17220	-0.97103
H	3.03406	-2.78818	-1.08599
H	4.11562	-1.70617	-1.95022
C	5.12421	-3.06611	-0.60431
H	4.95594	-3.56879	0.35611
H	5.29447	-3.83970	-1.36175
H	6.04583	-2.47821	-0.51277
H	2.01901	3.76290	0.09453

4e_conformer-025

C	0.15198	3.62901	-0.21232
C	0.31211	1.41847	0.30329
N	0.99828	2.55497	-0.10279
H	2.00371	2.61020	-0.18161
C	-1.11393	3.16831	0.10389
C	0.63772	4.98870	-0.59522
H	1.35773	5.38748	0.13133
H	1.13251	4.98565	-1.57485
H	-0.20464	5.68377	-0.64642
C	1.01253	0.14467	0.46120

C	0.55713	-0.83936	1.36400
C	2.17145	-0.12363	-0.28248
C	1.24515	-2.03909	1.49626
H	-0.31927	-0.65135	1.97186
C	2.86726	-1.32904	-0.13267
H	2.54089	0.58448	-1.01751
C	2.40456	-2.30300	0.75944
H	0.88446	-2.78773	2.19629
H	2.92308	-3.24526	0.88535
O	3.97891	-1.46452	-0.91471
C	4.71482	-2.67981	-0.81981
H	4.09926	-3.54246	-1.10200
H	5.54570	-2.58183	-1.51968
H	5.10677	-2.83139	0.19313
C	-1.03008	1.78452	0.43519
C	-2.21307	0.91006	0.75612
H	-2.10523	0.44048	1.74312
H	-3.10266	1.54845	0.83095
C	-2.47658	-0.18643	-0.29019
H	-2.64436	0.28861	-1.26648
H	-1.57838	-0.80695	-0.39813
C	-3.66856	-1.07721	0.06593
H	-3.49144	-1.54333	1.04674
H	-4.56969	-0.45677	0.18145
C	-3.93705	-2.17219	-0.97102
H	-4.11564	-1.70616	-1.95022
H	-3.03408	-2.78818	-1.08598
C	-5.12424	-3.06609	-0.60431
H	-6.04585	-2.47818	-0.51278
H	-5.29450	-3.83968	-1.36175
H	-4.95598	-3.56877	0.35611
H	-2.01901	3.76290	0.09454

4e_conformer-026

C	2.15732	2.26633	-0.62767
C	0.23750	1.09642	-0.27690
N	0.90709	2.29377	-0.06382
H	0.48140	3.11474	0.34239
C	2.30712	1.01276	-1.19424
C	3.07826	3.44128	-0.57952
H	3.29724	3.74733	0.45146
H	4.02581	3.19100	-1.06410
H	2.65802	4.31447	-1.09552
C	-1.10679	0.89255	0.25990
C	-1.53016	1.56585	1.41900

C	-2.00849	0.02302	-0.38548
C	-2.82082	1.37041	1.91386
H	-0.84333	2.21479	1.95329
C	-3.28915	-0.18124	0.13015
H	-1.70037	-0.46931	-1.29671
C	-3.70421	0.50032	1.28456
H	-3.13557	1.89370	2.81246
H	-4.70612	0.33086	1.66530
O	-4.21341	-1.01588	-0.43034
C	-3.82678	-1.76241	-1.57962
H	-2.96269	-2.40340	-1.36627
H	-4.68633	-2.38275	-1.83671
H	-3.58762	-1.10354	-2.42312
C	1.10946	0.26724	-0.98719
C	0.93014	-1.17596	-1.38276
H	0.08896	-1.29893	-2.07752
H	1.81883	-1.48078	-1.94722
C	0.72789	-2.14136	-0.19555
H	-0.24868	-1.94455	0.26176
H	0.68793	-3.16724	-0.58791
C	1.80822	-2.04889	0.88884
H	1.79410	-1.04046	1.32340
H	1.54920	-2.73976	1.70365
C	3.22634	-2.36377	0.40218
H	3.23493	-3.35278	-0.07826
H	3.51801	-1.64233	-0.37099
C	4.25706	-2.33053	1.53338
H	4.01248	-3.06320	2.31245
H	5.26562	-2.55442	1.16726
H	4.28507	-1.34137	2.00714
H	3.19334	0.65639	-1.70436

4e_conformer-027

C	-2.15728	2.26636	-0.62767
C	-0.23747	1.09643	-0.27690
N	-0.90705	2.29379	-0.06383
H	-0.48134	3.11475	0.34237
C	-2.30710	1.01279	-1.19422
C	-3.07821	3.44132	-0.57953
H	-4.02576	3.19104	-1.06409
H	-3.29716	3.74740	0.45145
H	-2.65796	4.31449	-1.09555
C	1.10681	0.89254	0.25990
C	1.53020	1.56586	1.41898
C	2.00850	0.02299	-0.38548

C	2.82086	1.37040	1.91384
H	0.84339	2.21481	1.95327
C	3.28917	-0.18127	0.13014
H	1.70037	-0.46935	-1.29670
C	3.70424	0.50029	1.28455
H	3.13563	1.89370	2.81243
H	4.70615	0.33082	1.66528
O	4.21341	-1.01594	-0.43034
C	3.82676	-1.76248	-1.57961
H	3.58761	-1.10361	-2.42311
H	4.68630	-2.38283	-1.83670
H	2.96266	-2.40345	-1.36625
C	-1.10945	0.26725	-0.98718
C	-0.93015	-1.17596	-1.38273
H	-1.81884	-1.48077	-1.94720
H	-0.08896	-1.29895	-2.07747
C	-0.72793	-2.14134	-0.19550
H	-0.68797	-3.16723	-0.58786
H	0.24862	-1.94453	0.26183
C	-1.80830	-2.04886	0.88886
H	-1.79419	-1.04043	1.32340
H	-1.54929	-2.73972	1.70368
C	-3.22640	-2.36375	0.40216
H	-3.51804	-1.64233	-0.37103
H	-3.23498	-3.35277	-0.07826
C	-4.25716	-2.33050	1.53333
H	-4.28518	-1.34132	2.00707
H	-5.26570	-2.55439	1.16718
H	-4.01260	-3.06315	2.31242
H	-3.19333	0.65642	-1.70433

4e_conformer-028

C	3.27044	-1.48578	-0.19251
C	1.07445	-0.90153	-0.19530
N	2.00764	-1.84363	0.21386
H	1.76859	-2.73107	0.63303
C	3.15056	-0.27366	-0.84668
C	4.46219	-2.34694	0.07168
H	4.60039	-2.53546	1.14399
H	4.38122	-3.32417	-0.42219
H	5.36537	-1.85755	-0.30270
C	-0.33825	-1.04688	0.15881
C	-0.70834	-1.55745	1.42078
C	-1.34967	-0.68217	-0.73871
C	-2.05138	-1.67715	1.75853

H	0.05996	-1.82534	2.13940
C	-2.70012	-0.78617	-0.38330
H	-1.10549	-0.33131	-1.73485
C	-3.06134	-1.28926	0.87228
H	-2.32767	-2.06512	2.73507
H	-4.10013	-1.38390	1.16321
O	-3.58991	-0.38774	-1.33934
C	-4.97837	-0.48847	-1.03987
H	-5.24686	0.13307	-0.17718
H	-5.50234	-0.12634	-1.92533
H	-5.26996	-1.52702	-0.84205
C	1.77269	0.10565	-0.85733
C	1.24074	1.41856	-1.36199
H	1.51111	1.55021	-2.41880
H	0.14812	1.42146	-1.32558
C	1.77437	2.63033	-0.56980
H	2.86543	2.67757	-0.68174
H	1.37922	3.54704	-1.02636
C	1.43588	2.60188	0.92760
H	1.93535	1.73833	1.38494
H	1.86675	3.49701	1.39846
C	-0.06295	2.53680	1.26096
H	-0.48097	1.58887	0.90369
H	-0.17244	2.52132	2.35375
C	-0.88094	3.69926	0.69076
H	-1.92477	3.64637	1.02084
H	-0.47508	4.66598	1.01503
H	-0.88212	3.69284	-0.40516
H	3.97230	0.29245	-1.26725

4e_conformer-029

C	1.95397	3.19771	-0.23256
C	1.38080	1.01837	0.06995
N	2.38419	1.89649	-0.31633
H	3.26678	1.61017	-0.71495
C	0.65647	3.15872	0.24526
C	2.82343	4.34732	-0.62483
H	3.75534	4.37190	-0.04565
H	3.10139	4.30838	-1.68621
H	2.29547	5.28903	-0.45231
C	1.61379	-0.42445	0.09370
C	2.90505	-0.93974	0.34168
C	0.56966	-1.33089	-0.13535
C	3.12040	-2.31236	0.35640
H	3.72709	-0.26299	0.55307

C	0.79165	-2.71281	-0.10075
H	-0.42754	-0.98271	-0.37444
C	2.07472	-3.21641	0.14200
H	4.11766	-2.69680	0.55188
H	2.26787	-4.28167	0.16513
O	-0.30939	-3.48672	-0.33462
C	-0.14064	-4.90047	-0.33758
H	0.21201	-5.26285	0.63557
H	-1.12627	-5.31904	-0.54522
H	0.56242	-5.21713	-1.11748
C	0.28338	1.79552	0.44059
C	-1.03890	1.32368	0.97444
H	-0.92754	0.33350	1.43297
H	-1.36823	1.99932	1.77556
C	-2.14657	1.26266	-0.09311
H	-2.28878	2.26592	-0.51782
H	-1.81519	0.62710	-0.92624
C	-3.47558	0.73741	0.45383
H	-3.32159	-0.26715	0.87519
H	-3.80188	1.37239	1.29073
C	-4.58562	0.67806	-0.59993
H	-4.74002	1.68235	-1.01877
H	-4.25543	0.04557	-1.43591
C	-5.90874	0.14457	-0.04515
H	-5.78782	-0.87116	0.35112
H	-6.27604	0.77700	0.77245
H	-6.68506	0.11220	-0.81806
H	0.03755	4.02369	0.44905

4e_conformer-030

C	-1.95539	3.19686	-0.23253
C	-1.38119	1.01778	0.06993
N	-2.38496	1.89542	-0.31640
H	-3.26724	1.60861	-0.71535
C	-0.65792	3.15848	0.24540
C	-2.82541	4.34607	-0.62476
H	-3.10358	4.30687	-1.68607
H	-3.75721	4.37037	-0.04538
H	-2.29778	5.28801	-0.45250
C	-1.61355	-0.42513	0.09364
C	-2.90463	-0.94097	0.34142
C	-0.56900	-1.33113	-0.13523
C	-3.11939	-2.31369	0.35614
H	-3.72701	-0.26459	0.55266
C	-0.79041	-2.71314	-0.10069

H	0.42810	-0.98253	-0.37413
C	-2.07329	-3.21730	0.14190
H	-4.11651	-2.69854	0.55149
H	-2.26599	-4.28264	0.16502
O	0.31100	-3.48656	-0.33446
C	0.14286	-4.90038	-0.33738
H	-0.20956	-5.26288	0.63580
H	-0.56012	-5.21735	-1.11722
H	1.12866	-5.31853	-0.54509
C	-0.28417	1.79545	0.44070
C	1.03830	1.32416	0.97454
H	1.36725	1.99976	1.77586
H	0.92738	0.33381	1.43283
C	2.14608	1.26394	-0.09296
H	1.81492	0.62872	-0.92644
H	2.28808	2.26745	-0.51714
C	3.47518	0.73873	0.45379
H	3.80128	1.37333	1.29105
H	3.32141	-0.26609	0.87460
C	4.58528	0.68022	-0.59995
H	4.25526	0.04813	-1.43630
H	4.73948	1.68478	-1.01821
C	5.90850	0.14668	-0.04543
H	6.27562	0.77870	0.77257
H	5.78778	-0.86931	0.35022
H	6.68485	0.11495	-0.81833
H	-0.03945	4.02374	0.44931

4e_conformer-031

C	1.80113	3.15762	0.19934
C	1.36207	0.94229	-0.06431
N	2.34309	1.89655	0.16722
H	3.30267	1.67963	0.39539
C	0.44691	3.01586	-0.04509
C	2.62999	4.37100	0.46806
H	3.09599	4.33796	1.46157
H	3.43774	4.48574	-0.26604
H	2.00447	5.26650	0.42219
C	1.70150	-0.47504	-0.17229
C	2.96359	-0.88080	-0.64097
C	0.76805	-1.46302	0.20023
C	3.27618	-2.23791	-0.73254
H	3.68760	-0.13903	-0.96346
C	1.08378	-2.81791	0.08336
H	-0.18395	-1.15873	0.60877

C	2.34754	-3.21207	-0.38052
H	4.25379	-2.53980	-1.09800
H	2.57543	-4.27035	-0.45581
O	0.22857	-3.83000	0.41436
C	-1.07746	-3.47986	0.86108
H	-1.04152	-2.90470	1.79417
H	-1.61635	-2.90020	0.10162
H	-1.59699	-4.42277	1.03615
C	0.15647	1.62930	-0.21317
C	-1.19788	1.05743	-0.52335
H	-1.71482	1.72694	-1.22129
H	-1.09230	0.09559	-1.04062
C	-2.07831	0.86094	0.72713
H	-1.51081	0.29460	1.47746
H	-2.27699	1.84234	1.17961
C	-3.40310	0.14006	0.44899
H	-3.19257	-0.84993	0.01664
H	-3.91325	-0.04715	1.40430
C	-4.35765	0.89920	-0.47965
H	-4.53176	1.90485	-0.07105
H	-3.88701	1.04409	-1.46035
C	-5.69613	0.17958	-0.66402
H	-6.36374	0.73650	-1.33120
H	-5.54903	-0.81862	-1.09468
H	-6.21055	0.05265	0.29651
H	-0.26638	3.82803	-0.11098

4e_conformer-032

C	-0.19531	3.53681	0.02730
C	0.18207	1.29472	0.01719
N	0.74104	2.54990	0.21373
H	1.67741	2.70622	0.55830
C	-1.37515	2.90587	-0.32400
C	0.12883	4.98333	0.21170
H	-0.74992	5.59328	-0.01431
H	0.43642	5.20708	1.24157
H	0.94351	5.30759	-0.44836
C	0.99221	0.08291	0.12903
C	0.42999	-1.14191	0.54685
C	2.36183	0.11707	-0.17267
C	1.22358	-2.27941	0.63798
H	-0.61458	-1.19059	0.82731
C	3.15762	-1.02889	-0.06094
H	2.83157	1.02829	-0.52957
C	2.58958	-2.24375	0.34089

H	0.77914	-3.21640	0.96231
H	3.18547	-3.14385	0.42671
O	4.47474	-0.86166	-0.38138
C	5.32740	-1.99973	-0.30772
H	6.31771	-1.65108	-0.60355
H	5.37125	-2.40081	0.71199
H	4.99854	-2.79009	-0.99317
C	-1.15245	1.49667	-0.33508
C	-2.17389	0.45514	-0.69255
H	-2.77834	0.81180	-1.53791
H	-1.66874	-0.45293	-1.03742
C	-3.12732	0.11581	0.46991
H	-2.53810	-0.18437	1.34790
H	-3.65572	1.03276	0.76151
C	-4.14561	-0.98280	0.13925
H	-4.88799	-1.03686	0.94778
H	-4.70154	-0.70402	-0.76825
C	-3.52875	-2.37401	-0.05211
H	-2.80647	-2.35435	-0.87809
H	-2.95633	-2.63812	0.84872
C	-4.57861	-3.45358	-0.32680
H	-5.14487	-3.22752	-1.23874
H	-4.11745	-4.43941	-0.45517
H	-5.29669	-3.52376	0.49951
H	-2.30555	3.40559	-0.56353

4e_conformer-033

C	0.19529	3.53681	0.02729
C	-0.18208	1.29470	0.01719
N	-0.74106	2.54989	0.21373
H	-1.67743	2.70620	0.55829
C	1.37513	2.90587	-0.32399
C	-0.12886	4.98332	0.21169
H	-0.94355	5.30757	-0.44835
H	-0.43644	5.20707	1.24156
H	0.74989	5.59328	-0.01433
C	-0.99222	0.08290	0.12903
C	-0.43000	-1.14192	0.54687
C	-2.36184	0.11705	-0.17268
C	-1.22359	-2.27941	0.63800
H	0.61457	-1.19059	0.82734
C	-3.15762	-1.02890	-0.06094
H	-2.83157	1.02828	-0.52958
C	-2.58959	-2.24376	0.34090
H	-0.77916	-3.21640	0.96234

H	-3.18548	-3.14386	0.42673
O	-4.47474	-0.86167	-0.38140
C	-5.32740	-1.99974	-0.30773
H	-5.37126	-2.40081	0.71198
H	-6.31771	-1.65109	-0.60357
H	-4.99854	-2.79011	-0.99317
C	1.15244	1.49667	-0.33508
C	2.17389	0.45514	-0.69254
H	1.66876	-0.45293	-1.03743
H	2.77836	0.81182	-1.53790
C	3.12731	0.11582	0.46993
H	3.65569	1.03277	0.76155
H	2.53808	-0.18439	1.34790
C	4.14562	-0.98277	0.13926
H	4.70157	-0.70397	-0.76822
H	4.88799	-1.03683	0.94781
C	3.52880	-2.37399	-0.05213
H	2.95636	-2.63813	0.84869
H	2.80653	-2.35433	-0.87813
C	4.57868	-3.45353	-0.32682
H	5.29674	-3.52372	0.49950
H	4.11753	-4.43937	-0.45522
H	5.14495	-3.22745	-1.23875
H	2.30553	3.40559	-0.56353

4e_conformer-034

C	-2.52947	-2.27046	-0.19430
C	-0.53479	-1.18219	-0.10667
N	-1.30483	-2.20689	0.42479
H	-0.95692	-2.88994	1.08281
C	-2.55881	-1.24463	-1.12095
C	-3.53971	-3.31612	0.14865
H	-3.17306	-4.32865	-0.06536
H	-3.81306	-3.28758	1.21119
H	-4.44990	-3.16011	-0.43670
C	0.79515	-0.89604	0.43537
C	1.04549	-1.02794	1.81113
C	1.84422	-0.49304	-0.41310
C	2.31519	-0.75236	2.32288
H	0.24057	-1.31426	2.48095
C	3.10430	-0.19901	0.11199
H	1.66564	-0.44213	-1.47823
C	3.34478	-0.33220	1.48828
H	2.49891	-0.85276	3.38896
H	4.33349	-0.10494	1.87356

O	4.17007	0.20725	-0.63849
C	3.97088	0.38873	-2.03664
H	3.19174	1.13496	-2.23436
H	4.92390	0.74344	-2.43118
H	3.70110	-0.55382	-2.52815
C	-1.30963	-0.55340	-1.08035
C	-1.01312	0.70793	-1.84624
H	0.06366	0.84006	-1.99012
H	-1.45057	0.62341	-2.84937
C	-1.57455	1.97568	-1.16832
H	-1.44772	2.82786	-1.85143
H	-2.65465	1.84743	-1.02356
C	-0.89682	2.29729	0.16726
H	-1.03111	1.45199	0.85456
H	0.18597	2.38145	-0.00022
C	-1.40463	3.58133	0.83763
H	-0.77639	3.79429	1.71290
H	-1.26981	4.42904	0.15061
C	-2.86954	3.51250	1.28197
H	-3.54706	3.38907	0.43000
H	-3.03187	2.66462	1.95940
H	-3.16664	4.42537	1.81072
H	-3.39668	-1.00504	-1.76410

4e_conformer-035

C	2.53035	-2.26979	-0.19415
C	0.53536	-1.18209	-0.10664
N	1.30568	-2.20654	0.42489
H	0.95795	-2.88963	1.08296
C	2.55941	-1.24400	-1.12087
C	3.54089	-3.31513	0.14888
H	3.17454	-4.32778	-0.06506
H	4.45104	-3.15890	-0.43647
H	3.81422	-3.28644	1.21142
C	-0.79468	-0.89629	0.43537
C	-1.04501	-1.02822	1.81112
C	-1.84384	-0.49359	-0.41314
C	-2.31480	-0.75297	2.32283
H	-0.24003	-1.31431	2.48096
C	-3.10401	-0.19989	0.11192
H	-1.66525	-0.44266	-1.47826
C	-3.34448	-0.33311	1.48821
H	-2.49851	-0.85339	3.38892
H	-4.33326	-0.10610	1.87347
O	-4.16987	0.20606	-0.63860

C	-3.97070	0.38756	-2.03674
H	-3.19176	1.13400	-2.23446
H	-3.70065	-0.55493	-2.52822
H	-4.92381	0.74200	-2.43131
C	1.31004	-0.55313	-1.08035
C	1.01318	0.70805	-1.84634
H	1.45098	0.62370	-2.84934
H	-0.06361	0.83969	-1.99056
C	1.57383	1.97609	-1.16830
H	2.65395	1.84834	-1.02323
H	1.44680	2.82818	-1.85148
C	0.89557	2.29744	0.16708
H	-0.18722	2.38102	-0.00070
H	1.03012	1.45227	0.85447
C	1.40253	3.58180	0.83748
H	1.26745	4.42939	0.15036
H	0.77396	3.79449	1.71257
C	2.86736	3.51377	1.28220
H	3.54517	3.39063	0.43042
H	3.16385	4.42684	1.81097
H	3.02997	2.66603	1.95975
H	3.39722	-1.00421	-1.76401

4e_conformer-036

C	-1.86151	-2.80934	-0.47310
C	-0.44890	-1.26166	0.40814
N	-0.55231	-2.40483	-0.37123
H	0.24102	-2.93565	-0.70197
C	-2.61765	-1.88850	0.22823
C	-2.25462	-4.04497	-1.21496
H	-3.34193	-4.15860	-1.19876
H	-1.93473	-4.01087	-2.26426
H	-1.81657	-4.94855	-0.77103
C	0.84982	-0.61926	0.62220
C	1.19212	-0.04854	1.86473
C	1.78878	-0.57306	-0.41704
C	2.42936	0.56223	2.03352
H	0.49945	-0.11445	2.69589
C	3.04003	0.02868	-0.23432
H	1.55243	-0.97931	-1.39557
C	3.36658	0.61043	0.99618
H	2.68548	0.99896	2.99486
H	4.32523	1.08794	1.15668
O	3.86518	0.00918	-1.32217
C	5.14235	0.62706	-1.19997

H	5.62830	0.50342	-2.16863
H	5.04851	1.69565	-0.97208
H	5.74745	0.14350	-0.42367
C	-1.74162	-0.91025	0.79097
C	-2.19350	0.33390	1.50518
H	-2.98079	0.07231	2.22434
H	-1.37385	0.76632	2.08718
C	-2.73679	1.41752	0.54943
H	-3.56071	0.99515	-0.04307
H	-3.16683	2.22765	1.15201
C	-1.66751	1.97620	-0.39442
H	-0.86677	2.43766	0.20261
H	-1.20189	1.14130	-0.93233
C	-2.19511	2.99670	-1.41216
H	-1.38556	3.25043	-2.10963
H	-2.98366	2.52655	-2.01709
C	-2.73482	4.28639	-0.78517
H	-1.97000	4.76761	-0.16248
H	-3.03906	5.00420	-1.55555
H	-3.60640	4.09489	-0.14967
H	-3.69633	-1.90692	0.32352

4e_conformer-037

C	3.60807	-0.74301	-0.37874
C	1.38645	-0.75537	0.10413
N	2.43604	-1.45213	-0.47862
H	2.32137	-2.29991	-1.01553
C	3.31327	0.41751	0.31145
C	4.88971	-1.24272	-0.96124
H	5.16769	-2.22412	-0.55629
H	4.83191	-1.34606	-2.05274
H	5.69920	-0.54365	-0.73458
C	0.04246	-1.33478	0.13381
C	-0.14020	-2.72656	0.28016
C	-1.09035	-0.52546	-0.00371
C	-1.42248	-3.26383	0.29199
H	0.72079	-3.37468	0.41158
C	-2.37872	-1.07040	0.02754
H	-0.98834	0.53698	-0.16847
C	-2.55533	-2.45122	0.17269
H	-1.55389	-4.33600	0.40910
H	-3.54296	-2.89517	0.19113
O	-3.39587	-0.16927	-0.11210
C	-4.73003	-0.66611	-0.12049
H	-5.37260	0.20577	-0.24916

H	-4.89063	-1.36449	-0.95069
H	-4.97626	-1.16504	0.82456
C	1.91826	0.42435	0.62304
C	1.23702	1.48412	1.44761
H	0.21856	1.17158	1.70148
H	1.77077	1.58153	2.40315
C	1.21489	2.87359	0.77303
H	0.95155	3.63185	1.52337
H	2.23369	3.10930	0.44085
C	0.24645	2.98332	-0.41325
H	0.53007	3.83661	-1.04395
H	0.35272	2.09213	-1.04834
C	-1.21925	3.15990	0.00713
H	-1.49775	2.39532	0.74407
H	-1.32189	4.12405	0.52379
C	-2.19326	3.09732	-1.17162
H	-3.22442	3.27991	-0.84872
H	-1.94025	3.84651	-1.93193
H	-2.16741	2.11145	-1.65028
H	4.03096	1.18136	0.58362

4e_conformer-038

C	-3.60859	-0.74179	-0.37879
C	-1.38700	-0.75492	0.10422
N	-2.43683	-1.45142	-0.47838
H	-2.32242	-2.29928	-1.01523
C	-3.31334	0.41879	0.31111
C	-4.89038	-1.24115	-0.96128
H	-5.69969	-0.54184	-0.73468
H	-4.83260	-1.34455	-2.05276
H	-5.16865	-2.22243	-0.55628
C	-0.04321	-1.33479	0.13403
C	0.13905	-2.72659	0.28054
C	1.08981	-0.52582	-0.00374
C	1.42118	-3.26423	0.29236
H	-0.72211	-3.37446	0.41208
C	2.37802	-1.07109	0.02747
H	0.98806	0.53660	-0.16868
C	2.55424	-2.45194	0.17283
H	1.55230	-4.33641	0.40961
H	3.54174	-2.89618	0.19125
O	3.39539	-0.17023	-0.11245
C	4.72943	-0.66738	-0.12095
H	4.97570	-1.16617	0.82416
H	4.88975	-1.36596	-0.95102

H	5.37224	0.20428	-0.24989
C	-1.91836	0.42516	0.62280
C	-1.23677	1.48479	1.44728
H	-1.77083	1.58276	2.40258
H	-0.21860	1.17176	1.70165
C	-1.21353	2.87409	0.77237
H	-2.23204	3.11034	0.43970
H	-0.95009	3.63234	1.52267
C	-0.24447	2.98309	-0.41350
H	-0.35096	2.09192	-1.04856
H	-0.52733	3.83648	-1.04441
C	1.22114	3.15893	0.00756
H	1.32395	4.12292	0.52446
H	1.49897	2.39406	0.74446
C	2.19574	3.09620	-1.17070
H	1.94331	3.84555	-1.93105
H	3.22675	3.27854	-0.84720
H	2.17002	2.11037	-1.64942
H	-4.03073	1.18299	0.58308

4e_conformer-039

C	-2.28033	2.27340	-0.43987
C	-0.34958	1.08094	-0.27499
N	-1.00348	2.25856	0.06170
H	-0.55419	3.04742	0.50451
C	-2.46239	1.06604	-1.09115
C	-3.19376	3.44118	-0.25782
H	-3.35444	3.67324	0.80275
H	-2.80047	4.34923	-0.73337
H	-4.16741	3.22480	-0.70561
C	1.01875	0.83788	0.17898
C	1.49297	1.41612	1.37645
C	1.89635	0.03842	-0.56531
C	2.79901	1.18926	1.79441
H	0.82733	2.01319	1.99198
C	3.20501	-0.19800	-0.12981
H	1.58507	-0.40101	-1.50538
C	3.67026	0.38187	1.05671
H	3.15069	1.63570	2.72044
H	4.68063	0.21442	1.40833
O	3.95627	-0.99855	-0.94272
C	5.30314	-1.25808	-0.56100
H	5.88843	-0.33220	-0.50647
H	5.35224	-1.77415	0.40542
H	5.71536	-1.90309	-1.33806

C	-1.25832	0.30772	-1.00080
C	-1.10083	-1.10309	-1.50553
H	-2.00934	-1.36563	-2.05965
H	-0.28290	-1.17772	-2.23351
C	-0.86281	-2.14745	-0.39416
H	-0.83416	-3.14443	-0.85581
H	0.12725	-1.97870	0.04531
C	-1.91018	-2.13081	0.72589
H	-1.62350	-2.86964	1.48764
H	-1.88824	-1.15132	1.22212
C	-3.34075	-2.42446	0.26289
H	-3.65853	-1.66002	-0.45686
H	-3.35927	-3.38292	-0.27565
C	-4.33773	-2.46529	1.42370
H	-4.06674	-3.24137	2.15023
H	-4.35639	-1.50625	1.95621
H	-5.35561	-2.67358	1.07457
H	-3.37444	0.74854	-1.58100

4e_conformer-040

C	3.89347	-0.37588	-0.20683
C	1.62381	-0.49953	-0.14613
N	2.81381	-1.19693	0.00408
H	2.87161	-2.19766	0.12911
C	3.38690	0.88526	-0.46947
C	5.29847	-0.88012	-0.15121
H	5.53187	-1.33557	0.81968
H	5.49453	-1.63791	-0.92114
H	5.99652	-0.05395	-0.31106
C	0.33487	-1.15518	0.07089
C	0.20769	-2.21086	0.99950
C	-0.80616	-0.74712	-0.63123
C	-1.02803	-2.81164	1.20964
H	1.06884	-2.52978	1.57854
C	-2.04996	-1.34449	-0.40168
H	-0.74948	0.03591	-1.37653
C	-2.17124	-2.38745	0.52409
H	-1.11629	-3.61790	1.93260
H	-3.12455	-2.86415	0.71546
O	-3.08307	-0.84356	-1.14143
C	-4.38505	-1.36867	-0.90595
H	-4.43381	-2.43879	-1.14082
H	-4.69436	-1.21097	0.13417
H	-5.05343	-0.82053	-1.57114
C	1.96315	0.82294	-0.43884

C	1.03697	2.00219	-0.57184
H	1.61823	2.87052	-0.90709
H	0.28985	1.82925	-1.35469
C	0.32390	2.36413	0.74667
H	-0.10495	1.45593	1.18461
H	1.08051	2.71791	1.45867
C	-0.77065	3.42848	0.57589
H	-1.01581	3.85792	1.55737
H	-0.37104	4.25529	-0.02831
C	-2.06881	2.90743	-0.06452
H	-2.69416	3.76512	-0.34552
H	-1.83963	2.38174	-1.00094
C	-2.87477	1.98123	0.85195
H	-2.30038	1.09334	1.13360
H	-3.16517	2.50208	1.77305
H	-3.78738	1.63280	0.35650
H	3.97901	1.77210	-0.65872

4e_conformer-041

C	-3.89346	-0.37600	-0.20689
C	-1.62382	-0.49950	-0.14606
N	-2.81377	-1.19697	0.00412
H	-2.87151	-2.19770	0.12921
C	-3.38696	0.88516	-0.46958
C	-5.29844	-0.88032	-0.15131
H	-5.49443	-1.63812	-0.92124
H	-5.53185	-1.33577	0.81958
H	-5.99653	-0.05418	-0.31119
C	-0.33483	-1.15507	0.07099
C	-0.20751	-2.21053	0.99982
C	0.80608	-0.74711	-0.63136
C	1.02828	-2.81118	1.20998
H	-1.06858	-2.52938	1.57902
C	2.04995	-1.34435	-0.40180
H	0.74926	0.03574	-1.37684
C	2.17139	-2.38707	0.52421
H	1.11666	-3.61727	1.93312
H	3.12474	-2.86368	0.71559
O	3.08295	-0.84354	-1.14179
C	4.38500	-1.36848	-0.90628
H	4.43383	-2.43865	-1.14089
H	5.05327	-0.82044	-1.57165
H	4.69437	-1.21050	0.13378
C	-1.96320	0.82292	-0.43885
C	-1.03707	2.00222	-0.57185

H	-0.28997	1.82932	-1.35472
H	-1.61836	2.87053	-0.90706
C	-0.32398	2.36417	0.74667
H	-1.08057	2.71811	1.45861
H	0.10472	1.45594	1.18470
C	0.77074	3.42833	0.57585
H	0.37129	4.25515	-0.02843
H	1.01592	3.85780	1.55732
C	2.06886	2.90703	-0.06443
H	1.83968	2.38133	-1.00084
H	2.69439	3.76459	-0.34541
C	2.87456	1.98073	0.85217
H	3.16490	2.50155	1.77331
H	2.30001	1.09292	1.13375
H	3.78719	1.63217	0.35686
H	-3.97911	1.77195	-0.65890

4e_conformer-042

C	-3.86440	0.26074	-0.15755
C	-1.69934	-0.39473	0.06548
N	-2.99943	-0.80529	-0.19292
H	-3.25321	-1.73966	-0.47996
C	-3.10988	1.37673	0.15828
C	-5.32401	0.10719	-0.43590
H	-5.80053	-0.59973	0.25531
H	-5.82665	1.07220	-0.32890
H	-5.51173	-0.25743	-1.45429
C	-0.59851	-1.35483	0.11557
C	-0.81994	-2.68954	0.51971
C	0.70344	-0.97524	-0.23697
C	0.23734	-3.59039	0.56651
H	-1.81235	-3.00619	0.82571
C	1.76621	-1.88297	-0.16789
H	0.91330	0.02296	-0.59774
C	1.54018	-3.20499	0.23211
H	0.05593	-4.61388	0.88292
H	2.34727	-3.92510	0.28497
O	2.98449	-1.38103	-0.52926
C	4.10367	-2.26038	-0.49480
H	3.96809	-3.10631	-1.17951
H	4.96053	-1.66670	-0.81583
H	4.28184	-2.64044	0.51837
C	-1.74744	0.97936	0.30216
C	-0.60276	1.87885	0.67173
H	-0.93839	2.61158	1.41814

H	0.18364	1.28803	1.15309
C	-0.01576	2.64859	-0.52837
H	0.21446	1.94196	-1.33788
H	-0.79235	3.31541	-0.92493
C	1.24177	3.46063	-0.19103
H	1.48253	4.11514	-1.04037
H	1.03003	4.12725	0.65814
C	2.47419	2.60535	0.13161
H	2.27226	1.96701	1.00106
H	2.66867	1.92233	-0.70729
C	3.72260	3.44761	0.40549
H	3.56543	4.11699	1.26027
H	4.59082	2.81740	0.62920
H	3.97477	4.07136	-0.46096
H	-3.49847	2.37916	0.28815

4e_conformer-043

C	3.86448	0.26037	-0.15764
C	1.69933	-0.39482	0.06542
N	2.99936	-0.80553	-0.19307
H	3.25299	-1.73990	-0.48028
C	3.11014	1.37644	0.15834
C	5.32406	0.10664	-0.43604
H	5.80047	-0.60053	0.25500
H	5.51170	-0.25777	-1.45452
H	5.82686	1.07154	-0.32882
C	0.59837	-1.35478	0.11550
C	0.81961	-2.68948	0.51982
C	-0.70352	-0.97509	-0.23715
C	-0.23778	-3.59018	0.56670
H	1.81197	-3.00621	0.82592
C	-1.76641	-1.88267	-0.16796
H	-0.91324	0.02306	-0.59810
C	-1.54057	-3.20466	0.23223
H	-0.05651	-4.61366	0.88325
H	-2.34776	-3.92466	0.28518
O	-2.98463	-1.38059	-0.52936
C	-4.10391	-2.25981	-0.49483
H	-3.96845	-3.10579	-1.17951
H	-4.28210	-2.63980	0.51836
H	-4.96070	-1.66603	-0.81587
C	1.74764	0.97925	0.30224
C	0.60313	1.87891	0.67191
H	-0.18331	1.28820	1.15336
H	0.93894	2.61159	1.41827

C	0.01610	2.64874	-0.52813
H	0.79266	3.31568	-0.92457
H	-0.21401	1.94220	-1.33774
C	-1.24152	3.46061	-0.19074
H	-1.02997	4.12693	0.65871
H	-1.48216	4.11542	-1.03989
C	-2.47396	2.60514	0.13134
H	-2.66826	1.92247	-0.70789
H	-2.27216	1.96642	1.00054
C	-3.72245	3.44722	0.40538
H	-3.56549	4.11620	1.26051
H	-3.97450	4.07137	-0.46082
H	-4.59069	2.81686	0.62863
H	3.49888	2.37880	0.28830

4e_conformer-044

C	-0.85600	3.04558	-0.19981
C	0.05042	0.98547	0.12463
N	0.23999	2.24817	-0.42076
H	1.02173	2.49653	-1.01007
C	-1.75547	2.29250	0.53119
C	-0.93767	4.44401	-0.71871
H	-0.11610	5.06797	-0.34435
H	-0.89771	4.47782	-1.81532
H	-1.87877	4.90323	-0.40442
C	1.10780	-0.02361	0.03757
C	0.81241	-1.39467	-0.10732
C	2.45405	0.36774	0.07806
C	1.84231	-2.32489	-0.18998
H	-0.21755	-1.71762	-0.18837
C	3.48537	-0.57346	-0.02342
H	2.72626	1.40933	0.21771
C	3.18463	-1.93463	-0.15186
H	1.60365	-3.37877	-0.30412
H	3.96664	-2.68020	-0.22498
O	4.75160	-0.06431	0.02807
C	5.84043	-0.97862	-0.05085
H	5.83456	-1.52590	-1.00117
H	6.74507	-0.37260	0.01343
H	5.82106	-1.69516	0.77915
C	-1.19862	0.99388	0.74354
C	-1.84966	-0.09190	1.55625
H	-1.15655	-0.92851	1.69604
H	-2.06672	0.29614	2.56129
C	-3.17002	-0.60915	0.94973

H	-3.64174	-1.29030	1.66878
H	-3.85942	0.23834	0.82735
C	-2.99508	-1.31035	-0.40068
H	-2.40178	-0.66287	-1.05928
H	-2.40822	-2.23004	-0.25763
C	-4.31419	-1.66329	-1.10145
H	-4.88792	-0.74082	-1.26990
H	-4.08859	-2.06973	-2.09637
C	-5.17910	-2.66892	-0.33470
H	-4.62230	-3.59408	-0.13904
H	-5.50574	-2.26846	0.63127
H	-6.07748	-2.93320	-0.90408
H	-2.71483	2.64197	0.89173

4e_conformer-045

C	-1.71644	2.78306	-0.12160
C	0.01341	1.32806	0.12936
N	-0.39036	2.55088	-0.38900
H	0.18518	3.13453	-0.97873
C	-2.17061	1.69953	0.60827
C	-2.41968	4.01152	-0.59880
H	-1.93524	4.92706	-0.23610
H	-2.44621	4.06999	-1.69481
H	-3.45227	4.01225	-0.23955
C	1.40483	0.89431	0.01585
C	2.44706	1.84536	-0.05553
C	1.74394	-0.46467	-0.02926
C	3.76830	1.43075	-0.16741
H	2.21991	2.90477	0.01050
C	3.07953	-0.87422	-0.12436
H	0.97877	-1.23040	-0.02054
C	4.10623	0.07375	-0.19915
H	4.56055	2.17262	-0.21748
H	5.14446	-0.22307	-0.28047
O	3.27404	-2.22600	-0.15434
C	4.61002	-2.70376	-0.27275
H	5.07604	-2.36017	-1.20418
H	5.22495	-2.38571	0.57778
H	4.53883	-3.79217	-0.28216
C	-1.09598	0.77409	0.77102
C	-1.16403	-0.51764	1.53758
H	-0.17222	-0.76414	1.93436
H	-1.82027	-0.38121	2.40797
C	-1.68816	-1.72077	0.72533
H	-1.04749	-1.87640	-0.15370

H	-1.59444	-2.62539	1.34240
C	-3.13935	-1.57590	0.26082
H	-3.78571	-1.41100	1.13602
H	-3.23462	-0.67778	-0.36302
C	-3.64522	-2.79195	-0.52105
H	-2.99461	-2.95666	-1.39159
H	-3.55197	-3.69116	0.10413
C	-5.09522	-2.63967	-0.98739
H	-5.77069	-2.50425	-0.13367
H	-5.20817	-1.76524	-1.64009
H	-5.43349	-3.52018	-1.54548
H	-3.17286	1.58449	1.00063

4e_conformer-046

C	1.71625	2.78308	-0.12187
C	-0.01351	1.32802	0.12937
N	0.39014	2.55083	-0.38911
H	-0.18552	3.13445	-0.97874
C	2.17055	1.69961	0.60800
C	2.41935	4.01156	-0.59921
H	2.44583	4.06995	-1.69522
H	1.93485	4.92709	-0.23655
H	3.45197	4.01243	-0.24000
C	-1.40491	0.89422	0.01601
C	-2.44719	1.84524	-0.05498
C	-1.74397	-0.46476	-0.02946
C	-3.76843	1.43062	-0.16676
H	-2.22004	2.90465	0.01126
C	-3.07955	-0.87434	-0.12446
H	-0.97874	-1.23045	-0.02113
C	-4.10630	0.07361	-0.19882
H	-4.56072	2.17247	-0.21653
H	-5.14454	-0.22323	-0.28005
O	-3.27401	-2.22612	-0.15480
C	-4.60999	-2.70389	-0.27320
H	-5.22484	-2.38613	0.57750
H	-5.07613	-2.36004	-1.20446
H	-4.53877	-3.79230	-0.28294
C	1.09598	0.77414	0.77094
C	1.16417	-0.51751	1.53763
H	1.82040	-0.38092	2.40801
H	0.17238	-0.76405	1.93444
C	1.68841	-1.72071	0.72555
H	1.59483	-2.62523	1.34278
H	1.04771	-1.87658	-0.15342

C	3.13956	-1.57574	0.26094
H	3.23470	-0.67768	-0.36302
H	3.78595	-1.41066	1.13608
C	3.64553	-2.79182	-0.52081
H	3.55241	-3.69097	0.10448
H	2.99488	-2.95672	-1.39129
C	5.09548	-2.63944	-0.98726
H	5.77098	-2.50384	-0.13360
H	5.43381	-3.51997	-1.54526
H	5.20829	-1.76508	-1.64007
H	3.17285	1.58464	1.00024

4e_conformer-047

C	-1.62276	2.60189	-0.15116
C	0.15912	1.21112	0.09735
N	-0.26423	2.47253	-0.29780
H	0.33232	3.15854	-0.73754
C	-2.08162	1.40831	0.37580
C	-2.34928	3.84790	-0.53934
H	-1.96721	4.72745	-0.00562
H	-2.26200	4.05621	-1.61372
H	-3.41222	3.74704	-0.30418
C	1.57805	0.86029	0.07876
C	2.56458	1.84846	0.24721
C	1.98472	-0.47505	-0.11380
C	3.91666	1.50390	0.22301
H	2.27612	2.87882	0.43002
C	3.33994	-0.81132	-0.11524
H	1.23527	-1.23151	-0.29197
C	4.31580	0.18280	0.04866
H	4.66941	2.27586	0.35659
H	5.36359	-0.09919	0.03605
O	3.80868	-2.08250	-0.28643
C	2.85551	-3.12931	-0.44000
H	2.19181	-3.19557	0.43064
H	2.25055	-2.99020	-1.34419
H	3.43371	-4.04993	-0.52861
C	-0.97381	0.52156	0.53488
C	-1.04244	-0.86293	1.11841
H	-0.07931	-1.12224	1.57304
H	-1.77426	-0.86797	1.93547
C	-1.41895	-1.96967	0.10748
H	-0.64998	-2.01671	-0.67489
H	-1.39207	-2.93730	0.62814
C	-2.78555	-1.79426	-0.56713

H	-2.80547	-0.83437	-1.09962
H	-2.90186	-2.57408	-1.33277
C	-3.98030	-1.86497	0.39009
H	-3.95407	-2.82136	0.93150
H	-3.89557	-1.08113	1.15342
C	-5.32127	-1.71738	-0.33321
H	-5.45309	-2.50592	-1.08434
H	-6.16418	-1.77465	0.36486
H	-5.38136	-0.75290	-0.85257
H	-3.11049	1.19523	0.63578

4e_conformer-048

C	1.62275	2.60190	-0.15115
C	-0.15913	1.21113	0.09736
N	0.26422	2.47254	-0.29780
H	-0.33232	3.15855	-0.73753
C	2.08162	1.40833	0.37580
C	2.34927	3.84792	-0.53933
H	3.41221	3.74706	-0.30416
H	2.26201	4.05622	-1.61371
H	1.96720	4.72746	-0.00562
C	-1.57805	0.86030	0.07875
C	-2.56459	1.84845	0.24719
C	-1.98471	-0.47505	-0.11379
C	-3.91667	1.50389	0.22299
H	-2.27614	2.87883	0.43000
C	-3.33993	-0.81132	-0.11524
H	-1.23525	-1.23151	-0.29196
C	-4.31580	0.18278	0.04865
H	-4.66943	2.27584	0.35657
H	-5.36358	-0.09921	0.03604
O	-3.80865	-2.08251	-0.28642
C	-2.85548	-3.12932	-0.43999
H	-2.19178	-3.19556	0.43066
H	-3.43366	-4.04994	-0.52859
H	-2.25052	-2.99021	-1.34417
C	0.97380	0.52157	0.53488
C	1.04243	-0.86292	1.11841
H	1.77425	-0.86796	1.93546
H	0.07930	-1.12223	1.57303
C	1.41894	-1.96965	0.10747
H	1.39205	-2.93729	0.62812
H	0.64997	-2.01668	-0.67491
C	2.78554	-1.79425	-0.56714
H	2.80547	-0.83435	-1.09961

H	2.90184	-2.57406	-1.33279
C	3.98029	-1.86498	0.39008
H	3.89557	-1.08116	1.15343
H	3.95404	-2.82138	0.93148
C	5.32126	-1.71740	-0.33321
H	5.38136	-0.75291	-0.85256
H	6.16417	-1.77468	0.36486
H	5.45307	-2.50593	-1.08435
H	3.11048	1.19525	0.63579

4e_conformer-049

C	2.59257	-2.29025	-0.09341
C	0.60484	-1.18839	-0.16038
N	1.32742	-2.21174	0.43654
H	0.92848	-2.88600	1.07429
C	2.69543	-1.27516	-1.02662
C	3.56819	-3.33852	0.33222
H	4.52031	-3.19244	-0.18503
H	3.76198	-3.30243	1.41187
H	3.21248	-4.35087	0.09962
C	-0.75798	-0.88601	0.28260
C	-1.10127	-0.98890	1.64701
C	-1.74832	-0.49948	-0.62846
C	-2.39520	-0.70018	2.06565
H	-0.34352	-1.26539	2.37359
C	-3.04438	-0.19216	-0.19716
H	-1.53681	-0.45418	-1.69050
C	-3.37883	-0.29370	1.15875
H	-2.64900	-0.77700	3.11931
H	-4.37734	-0.06665	1.51113
O	-3.91638	0.17955	-1.18036
C	-5.25537	0.48296	-0.80288
H	-5.29221	1.33089	-0.10838
H	-5.77382	0.74681	-1.72556
H	-5.74659	-0.38288	-0.34267
C	1.45125	-0.57577	-1.08238
C	1.21364	0.67952	-1.87711
H	1.68140	0.57120	-2.86429
H	0.14525	0.83344	-2.05661
C	1.78268	1.94204	-1.19601
H	2.85532	1.79327	-1.01899
H	1.69305	2.79017	-1.88990
C	1.07381	2.28910	0.11688
H	1.17310	1.44775	0.81515
H	-0.00211	2.39145	-0.08210

C	1.58620	3.56993	0.78987
H	1.48465	4.41368	0.09235
H	0.93885	3.80166	1.64625
C	3.03743	3.47957	1.27352
H	3.73490	3.33739	0.44080
H	3.33632	4.39161	1.80272
H	3.16709	2.63472	1.96170
H	3.57911	-1.04813	-1.61025

4e_conformer-050

C	-2.95177	-1.89578	-0.27149
C	-0.79564	-1.17727	-0.15518
N	-1.70937	-2.13793	0.25696
H	-1.46085	-2.96922	0.77393
C	-2.84304	-0.73717	-1.02023
C	-4.11423	-2.79873	-0.01755
H	-3.93907	-3.81219	-0.40162
H	-4.33456	-2.89073	1.05367
H	-5.00633	-2.40416	-0.51128
C	0.58914	-1.22414	0.31102
C	0.91522	-1.82213	1.54104
C	1.62644	-0.67792	-0.47106
C	2.24259	-1.86987	1.97040
H	0.13149	-2.21891	2.17871
C	2.94726	-0.71178	-0.02175
H	1.38740	-0.24691	-1.43247
C	3.26284	-1.31705	1.20428
H	2.48115	-2.33173	2.92443
H	4.29710	-1.33671	1.53202
O	4.00251	-0.19185	-0.71555
C	3.72848	0.47664	-1.94257
H	4.68943	0.84508	-2.30394
H	3.04500	1.32115	-1.79192
H	3.29859	-0.20758	-2.68407
C	-1.49548	-0.27611	-0.96269
C	-1.00613	1.00316	-1.59176
H	-0.20157	0.80741	-2.31263
H	-1.83033	1.42674	-2.18013
C	-0.52452	2.06506	-0.58376
H	0.31871	1.66354	-0.00775
H	-0.13639	2.92341	-1.14655
C	-1.62228	2.52150	0.38168
H	-2.43717	2.98992	-0.19085
H	-2.05552	1.63630	0.86400
C	-1.13758	3.49435	1.46537

H	-1.96156	3.68209	2.16677
H	-0.34176	3.01123	2.05016
C	-0.62893	4.83395	0.92295
H	0.25554	4.70521	0.28945
H	-1.39986	5.32941	0.31925
H	-0.35512	5.51302	1.73851
H	-3.65303	-0.25623	-1.55435

4e_conformer-051

C	2.95177	-1.89578	-0.27149
C	0.79564	-1.17727	-0.15518
N	1.70937	-2.13793	0.25697
H	1.46085	-2.96922	0.77393
C	2.84304	-0.73718	-1.02024
C	4.11423	-2.79873	-0.01755
H	3.93906	-3.81220	-0.40162
H	5.00632	-2.40417	-0.51128
H	4.33456	-2.89073	1.05367
C	-0.58914	-1.22414	0.31103
C	-0.91522	-1.82212	1.54105
C	-1.62644	-0.67793	-0.47105
C	-2.24259	-1.86985	1.97041
H	-0.13149	-2.21889	2.17872
C	-2.94726	-0.71178	-0.02175
H	-1.38740	-0.24692	-1.43247
C	-3.26284	-1.31704	1.20429
H	-2.48115	-2.33170	2.92445
H	-4.29710	-1.33669	1.53203
O	-4.00251	-0.19186	-0.71556
C	-3.72848	0.47662	-1.94258
H	-3.29859	-0.20760	-2.68407
H	-4.68943	0.84506	-2.30395
H	-3.04500	1.32114	-1.79194
C	1.49548	-0.27611	-0.96269
C	1.00613	1.00315	-1.59176
H	1.83032	1.42674	-2.18013
H	0.20156	0.80740	-2.31263
C	0.52452	2.06506	-0.58376
H	0.13639	2.92341	-1.14656
H	-0.31871	1.66354	-0.00775
C	1.62228	2.52149	0.38168
H	2.05552	1.63630	0.86400
H	2.43717	2.98992	-0.19085
C	1.13758	3.49435	1.46536
H	0.34177	3.01123	2.05016

H	1.96157	3.68209	2.16676
C	0.62893	4.83395	0.92295
H	1.39987	5.32941	0.31925
H	-0.25554	4.70521	0.28944
H	0.35512	5.51302	1.73850
H	3.65302	-0.25623	-1.55435

4e_conformer-052

C	-1.24764	2.46369	-0.05109
C	0.06624	0.60972	0.03616
N	0.01192	1.96310	-0.26741
H	0.76736	2.47843	-0.69595
C	-2.01460	1.41772	0.42869
C	-1.59664	3.88779	-0.33604
H	-0.96200	4.58498	0.22572
H	-1.48572	4.13266	-1.40052
H	-2.63611	4.07899	-0.05626
C	1.32486	-0.12751	-0.06525
C	1.35289	-1.50680	-0.35967
C	2.54684	0.53574	0.12463
C	2.56702	-2.17868	-0.44378
H	0.42901	-2.03782	-0.55107
C	3.76549	-0.14430	0.02077
H	2.57495	1.58858	0.38721
C	3.78426	-1.51583	-0.25949
H	2.57542	-3.24066	-0.67338
H	4.71548	-2.06307	-0.33710
O	4.88108	0.61677	0.22500
C	6.14875	-0.02775	0.15250
H	6.32241	-0.45717	-0.84154
H	6.89030	0.74808	0.34732
H	6.23900	-0.81731	0.90822
C	-1.20362	0.24361	0.48613
C	-1.64902	-1.10125	0.99014
H	-0.78485	-1.66029	1.36684
H	-2.31536	-0.95700	1.84957
C	-2.37314	-1.97196	-0.06144
H	-1.68253	-2.17570	-0.89029
H	-2.60679	-2.94385	0.39572
C	-3.65751	-1.36169	-0.63718
H	-3.42127	-0.39891	-1.10888
H	-4.02765	-2.01536	-1.43945
C	-4.77955	-1.15941	0.38693
H	-5.00820	-2.12111	0.86795
H	-4.43896	-0.49196	1.18870

C	-6.05010	-0.58084	-0.24085
H	-6.43666	-1.24014	-1.02780
H	-6.84280	-0.44690	0.50400
H	-5.85063	0.39668	-0.69722
H	-3.05368	1.48939	0.72318

4e_conformer-053

C	1.24765	2.46369	-0.05114
C	-0.06625	0.60973	0.03612
N	-0.01192	1.96311	-0.26745
H	-0.76735	2.47844	-0.69600
C	2.01460	1.41772	0.42865
C	1.59666	3.88779	-0.33609
H	1.48574	4.13267	-1.40056
H	0.96202	4.58498	0.22568
H	2.63613	4.07899	-0.05631
C	-1.32487	-0.12749	-0.06530
C	-1.35290	-1.50677	-0.35980
C	-2.54685	0.53574	0.12467
C	-2.56703	-2.17864	-0.44392
H	-0.42903	-2.03776	-0.55128
C	-3.76550	-0.14430	0.02080
H	-2.57494	1.58856	0.38732
C	-3.78426	-1.51582	-0.25955
H	-2.57543	-3.24061	-0.67359
H	-4.71549	-2.06306	-0.33716
O	-4.88108	0.61675	0.22513
C	-6.14875	-0.02777	0.15263
H	-6.23897	-0.81738	0.90830
H	-6.89030	0.74804	0.34752
H	-6.32244	-0.45713	-0.84144
C	1.20361	0.24361	0.48609
C	1.64899	-1.10126	0.99010
H	2.31529	-0.95700	1.84956
H	0.78481	-1.66029	1.36677
C	2.37315	-1.97197	-0.06145
H	2.60678	-2.94386	0.39574
H	1.68257	-2.17571	-0.89031
C	3.65755	-1.36170	-0.63714
H	4.02771	-2.01538	-1.43940
H	3.42132	-0.39893	-1.10885
C	4.77955	-1.15942	0.38701
H	4.43893	-0.49197	1.18877
H	5.00818	-2.12112	0.86804
C	6.05011	-0.58085	-0.24072

H	6.43671	-1.24015	-1.02766
H	5.85067	0.39667	-0.69709
H	6.84279	-0.44691	0.50415
H	3.05368	1.48938	0.72313

4e_conformer-054

C	-1.68288	-3.21574	0.02596
C	-0.53848	-1.26818	0.29668
N	-0.44060	-2.64310	0.13197
H	0.42286	-3.16123	0.21095
C	-2.60131	-2.18294	0.09493
C	-1.86918	-4.69112	-0.11498
H	-2.93306	-4.92410	-0.21091
H	-1.35630	-5.08548	-1.00150
H	-1.48308	-5.23908	0.75453
C	0.66505	-0.44440	0.40224
C	0.67021	0.75965	1.13711
C	1.85589	-0.84683	-0.22010
C	1.82968	1.52075	1.22454
H	-0.22501	1.08098	1.65470
C	3.02407	-0.08227	-0.11455
H	1.89309	-1.74724	-0.82519
C	3.01823	1.11647	0.60775
H	1.81956	2.44561	1.79456
H	3.90814	1.72713	0.69535
O	4.11392	-0.58521	-0.76577
C	5.32552	0.16092	-0.70939
H	6.05420	-0.40777	-1.28850
H	5.20249	1.15609	-1.15335
H	5.68164	0.26676	0.32240
C	-1.89936	-0.95462	0.27027
C	-2.54616	0.40485	0.28806
H	-3.63328	0.26969	0.35962
H	-2.25922	0.96742	1.18360
C	-2.23566	1.23955	-0.96858
H	-1.15093	1.37579	-1.05077
H	-2.53698	0.65667	-1.84853
C	-2.94696	2.59986	-1.00111
H	-2.78370	3.06012	-1.98598
H	-4.03034	2.43348	-0.91602
C	-2.50840	3.59831	0.08213
H	-3.15308	4.48491	0.01749
H	-2.68441	3.17131	1.07823
C	-1.04444	4.03442	-0.03545
H	-0.84575	4.47237	-1.02192

H	-0.79546	4.78780	0.72086
H	-0.35730	3.19289	0.09851
H	-3.67617	-2.29279	0.02125

4e_conformer-055

C	1.68104	-3.21669	0.02606
C	0.53777	-1.26842	0.29645
N	0.43910	-2.64330	0.13190
H	-0.42466	-3.16091	0.21087
C	2.60008	-2.18443	0.09499
C	1.86648	-4.69219	-0.11470
H	1.47982	-5.23984	0.75477
H	1.35357	-5.08633	-1.00131
H	2.93024	-4.92584	-0.21034
C	-0.66530	-0.44394	0.40188
C	-0.66966	0.76047	1.13616
C	-1.85651	-0.84606	-0.21995
C	-1.82873	1.52219	1.22353
H	0.22586	1.08165	1.65331
C	-3.02428	-0.08086	-0.11446
H	-1.89435	-1.74673	-0.82461
C	-3.01766	1.11821	0.60726
H	-1.81799	2.44733	1.79309
H	-3.90723	1.72936	0.69481
O	-4.11455	-0.58358	-0.76514
C	-5.32579	0.16314	-0.70875
H	-5.20242	1.15806	-1.15318
H	-6.05489	-0.40547	-1.28739
H	-5.68156	0.26960	0.32310
C	1.89885	-0.95567	0.27013
C	2.54654	0.40338	0.28797
H	2.26021	0.96597	1.18371
H	3.63358	0.26748	0.35925
C	2.23633	1.23852	-0.96845
H	2.53709	0.65560	-1.84856
H	1.15167	1.37550	-1.05040
C	2.94854	2.59836	-1.00086
H	4.03180	2.43123	-0.91577
H	2.78560	3.05882	-1.98568
C	2.51064	3.59700	0.08247
H	2.68612	3.16970	1.07853
H	3.15609	4.48307	0.01808
C	1.04708	4.03439	-0.03531
H	0.35920	3.19342	0.09829
H	0.79859	4.78780	0.72113

H	0.84898	4.47275	-1.02171
H	3.67488	-2.29492	0.02144

4e_conformer-056

C	3.85486	0.52235	-0.12812
C	1.72104	-0.26810	-0.11461
N	3.05111	-0.54902	0.16788
H	3.39246	-1.46147	0.43402
C	3.02104	1.52718	-0.58652
C	5.33775	0.47573	0.04533
H	5.80126	-0.29350	-0.58616
H	5.77466	1.43994	-0.22800
H	5.62190	0.25885	1.08300
C	0.68124	-1.26149	0.14946
C	0.86053	-2.24335	1.14871
C	-0.51429	-1.27258	-0.58196
C	-0.12955	-3.18863	1.38872
H	1.75890	-2.24240	1.75803
C	-1.51317	-2.21758	-0.32310
H	-0.68715	-0.55790	-1.37707
C	-1.32636	-3.19099	0.66556
H	0.01952	-3.93528	2.16384
H	-2.08558	-3.93318	0.87831
O	-2.63114	-2.11347	-1.10140
C	-3.67663	-3.05738	-0.89425
H	-4.45849	-2.79971	-1.60987
H	-3.33316	-4.08188	-1.08199
H	-4.07819	-2.99169	0.12416
C	1.67970	1.04570	-0.58935
C	0.47476	1.87317	-0.95061
H	0.82332	2.83731	-1.34223
H	-0.09275	1.41205	-1.77017
C	-0.47739	2.13214	0.22961
H	-0.78884	1.17290	0.66102
H	0.07271	2.66279	1.01873
C	-1.71787	2.93424	-0.16933
H	-1.40981	3.89547	-0.60689
H	-2.25587	2.39574	-0.96378
C	-2.67320	3.19232	0.99979
H	-2.97587	2.22993	1.43595
H	-2.13642	3.73185	1.79268
C	-3.91792	3.98457	0.59233
H	-4.48919	3.45070	-0.17711
H	-4.58389	4.15477	1.44596
H	-3.64379	4.96379	0.18079

H	3.33831	2.51801	-0.88714

4e_conformer-057

C	-3.83348	0.34911	0.09949
C	-1.65650	-0.31332	0.10306
N	-2.96877	-0.68434	-0.15659
H	-3.25601	-1.62491	-0.38617
C	-3.05899	1.42048	0.50904
C	-5.31169	0.20778	-0.06188
H	-5.58746	-0.06248	-1.08926
H	-5.72486	-0.56489	0.59955
H	-5.80428	1.15328	0.18033
C	-0.55785	-1.24917	-0.13063
C	-0.67720	-2.28047	-1.07899
C	0.64765	-1.13209	0.59065
C	0.38258	-3.16241	-1.29704
H	-1.58063	-2.37320	-1.67360
C	1.70979	-2.00328	0.34615
H	0.73583	-0.36444	1.34541
C	1.57826	-3.03189	-0.59916
H	0.27884	-3.95239	-2.03566
H	2.41454	-3.70187	-0.77002
O	2.91301	-1.93816	0.98915
C	3.10975	-0.88927	1.93175
H	2.99658	0.09507	1.46173
H	2.41079	-0.97006	2.77300
H	4.13130	-1.00284	2.29687
C	-1.69143	1.02071	0.52148
C	-0.53515	1.92933	0.84728
H	-0.93525	2.90359	1.15548
H	0.02519	1.55727	1.71664
C	0.44448	2.13457	-0.32098
H	0.75946	1.15528	-0.69369
H	-0.08389	2.62553	-1.14965
C	1.67348	2.95925	0.07100
H	1.34935	3.96019	0.38853
H	2.14949	2.49986	0.95093
C	2.71730	3.09158	-1.04753
H	3.47627	3.82393	-0.74226
H	2.23573	3.50509	-1.94490
C	3.41351	1.77238	-1.40073
H	4.16927	1.92008	-2.18047
H	3.91861	1.35071	-0.52256
H	2.70501	1.02068	-1.76514
H	-3.43367	2.40240	0.77016

4e_conformer-058

C	-0.41326	3.35693	-0.23236
C	0.17639	1.17759	0.03834
N	0.58329	2.42839	-0.40525
H	1.44923	2.60226	-0.89494
C	-1.47415	2.69705	0.36126
C	-0.25481	4.77950	-0.65980
H	0.60350	5.25974	-0.17281
H	-0.10505	4.86831	-1.74377
H	-1.15117	5.34855	-0.39894
C	1.08108	0.03047	-0.01392
C	0.59528	-1.28430	-0.17604
C	2.46768	0.21895	0.08728
C	1.48053	-2.35520	-0.21932
H	-0.46656	-1.45788	-0.29877
C	3.35309	-0.86313	0.02524
H	2.88572	1.20803	0.24645
C	2.86264	-2.16603	-0.12289
H	1.09467	-3.36288	-0.34695
H	3.52957	-3.01808	-0.16606
O	4.67688	-0.54486	0.13379
C	5.62229	-1.60915	0.09934
H	5.57750	-2.15290	-0.85197
H	6.60236	-1.14206	0.20453
H	5.46225	-2.31225	0.92559
C	-1.11756	1.32686	0.53777
C	-1.98683	0.27798	1.17047
H	-1.36641	-0.52873	1.57946
H	-2.52305	0.71678	2.02292
C	-3.02197	-0.32435	0.20285
H	-3.67658	0.47761	-0.16618
H	-2.50000	-0.71734	-0.67867
C	-3.86762	-1.42390	0.85149
H	-3.20461	-2.23236	1.19444
H	-4.34560	-1.01680	1.75362
C	-4.94828	-2.01349	-0.06579
H	-5.56992	-2.70418	0.51921
H	-5.61670	-1.20742	-0.40003
C	-4.39168	-2.75459	-1.28596
H	-3.83926	-2.08307	-1.95220
H	-3.70596	-3.55411	-0.97810
H	-5.19715	-3.21170	-1.87182
H	-2.41164	3.15355	0.65363

4e_conformer-059

C	-2.88917	-2.00175	-0.17710
C	-0.77380	-1.17102	-0.10571
N	-1.63997	-2.14140	0.37705
H	-1.35030	-2.91832	0.95402
C	-2.83267	-0.89915	-1.00956
C	-4.00374	-2.94824	0.12898
H	-3.77475	-3.97211	-0.19468
H	-4.22231	-2.98862	1.20372
H	-4.91375	-2.63074	-0.38725
C	0.60456	-1.09693	0.38353
C	0.89829	-1.38211	1.72713
C	1.65651	-0.74886	-0.48549
C	2.21442	-1.31001	2.18819
H	0.09512	-1.62874	2.41464
C	2.96602	-0.65838	-0.00896
H	1.43792	-0.58151	-1.53110
C	3.24979	-0.94382	1.33553
H	2.43214	-1.52817	3.23000
H	4.27564	-0.87404	1.68253
O	4.03939	-0.31798	-0.78104
C	3.80213	0.01042	-2.14607
H	3.11456	0.86005	-2.23778
H	4.77346	0.28112	-2.56194
H	3.39476	-0.84491	-2.69851
C	-1.50730	-0.36746	-0.97755
C	-1.08898	0.91322	-1.64776
H	-0.00831	0.93419	-1.81846
H	-1.55871	0.96525	-2.63847
C	-1.47809	2.17685	-0.85088
H	-1.26757	3.05558	-1.47376
H	-2.56283	2.17038	-0.67293
C	-0.74463	2.29634	0.48855
H	-0.92150	1.38371	1.07029
H	0.33919	2.33258	0.30327
C	-1.15845	3.51346	1.32711
H	-2.23986	3.46681	1.52007
H	-0.67006	3.44796	2.30863
C	-0.81259	4.86244	0.68811
H	0.26127	4.92803	0.47186
H	-1.34977	5.01487	-0.25445
H	-1.07109	5.69397	1.35373
H	-3.66220	-0.50374	-1.58278

4e_conformer-060

C	2.89139	-1.99908	-0.17691
C	0.77527	-1.17024	-0.10560
N	1.64230	-2.13984	0.37720
H	1.35330	-2.91700	0.95418
C	2.83392	-0.89653	-1.00937
C	4.00679	-2.94458	0.12918
H	4.22542	-2.98473	1.20391
H	3.77872	-3.96866	-0.19444
H	4.91651	-2.62628	-0.38707
C	-0.60321	-1.09744	0.38356
C	-0.89676	-1.38277	1.72716
C	-1.65542	-0.75044	-0.48557
C	-2.21300	-1.31190	2.18811
H	-0.09341	-1.62856	2.41476
C	-2.96505	-0.66118	-0.00915
H	-1.43690	-0.58293	-1.53116
C	-3.24866	-0.94679	1.33534
H	-2.43059	-1.53018	3.22993
H	-4.27460	-0.87796	1.68226
O	-4.03869	-0.32185	-0.78133
C	-3.80164	0.00675	-2.14635
H	-3.39336	-0.84820	-2.69873
H	-4.77320	0.27647	-2.56231
H	-3.11490	0.85706	-2.23801
C	1.50806	-0.36603	-0.97741
C	1.08864	0.91424	-1.64773
H	1.55885	0.96684	-2.63817
H	0.00804	0.93399	-1.81904
C	1.47589	2.17827	-0.85059
H	2.56054	2.17301	-0.67204
H	1.26474	3.05679	-1.47358
C	0.74157	2.29694	0.48843
H	-0.34220	2.33190	0.30257
H	0.91920	1.38454	1.07029
C	1.15347	3.51458	1.32718
H	0.66467	3.44848	2.30845
H	2.23483	3.46928	1.52069
C	0.80625	4.86313	0.68800
H	-0.26759	4.92737	0.47120
H	1.06337	5.69498	1.35374
H	1.34372	5.01623	-0.25429
H	3.66310	-0.50038	-1.58258

4e_conformer-061

C	2.73283	-1.94273	-0.31880
C	0.98106	-0.77973	0.54760
N	1.37848	-1.94380	-0.09498
H	0.76957	-2.73274	-0.26081
C	3.21083	-0.73630	0.16171
C	3.43461	-3.09620	-0.95789
H	3.35406	-4.01136	-0.35666
H	3.02742	-3.32345	-1.95143
H	4.49713	-2.86646	-1.07482
C	-0.42998	-0.53613	0.84603
C	-0.81973	0.14725	2.00809
C	-1.42213	-0.98326	-0.04977
C	-2.17093	0.39440	2.25461
H	-0.06704	0.47279	2.71756
C	-2.77267	-0.74127	0.21291
H	-1.11989	-1.47890	-0.96396
C	-3.15122	-0.04385	1.36977
H	-2.46449	0.92724	3.15473
H	-4.20511	0.14068	1.55099
O	-3.79394	-1.12936	-0.60584
C	-3.46173	-1.81524	-1.80847
H	-2.93539	-2.75462	-1.60053
H	-4.41029	-2.03152	-2.30152
H	-2.84254	-1.19168	-2.46492
C	2.12347	0.00452	0.71245
C	2.22471	1.41491	1.23798
H	2.03831	1.44661	2.32043
H	3.26194	1.74873	1.10517
C	1.27991	2.41953	0.54914
H	0.24790	2.15540	0.79588
H	1.45404	3.41730	0.97546
C	1.44253	2.48052	-0.97251
H	2.43903	2.87752	-1.21320
H	1.41107	1.46062	-1.37759
C	0.36871	3.32505	-1.67428
H	0.35198	4.33455	-1.23891
H	0.64912	3.45177	-2.72851
C	-1.03611	2.71348	-1.60507
H	-1.04150	1.69943	-2.02371
H	-1.40152	2.64044	-0.57510
H	-1.75746	3.31501	-2.17000
H	4.24087	-0.40547	0.11214

4e_conformer-062

C	1.32733	2.87611	0.31009

C	0.15865	1.12197	-0.54726
N	0.10499	2.25519	0.25347
H	-0.74942	2.64178	0.62884
C	2.19507	2.10900	-0.44695
C	1.54076	4.14360	1.07104
H	0.93265	4.96794	0.67595
H	1.28565	4.03139	2.13258
H	2.59033	4.44313	1.00767
C	-1.02662	0.28516	-0.73039
C	-1.23760	-0.43626	-1.91665
C	-1.99178	0.20281	0.29536
C	-2.38035	-1.22326	-2.06514
H	-0.52223	-0.36396	-2.72710
C	-3.13937	-0.57559	0.12932
H	-1.81535	0.72239	1.22892
C	-3.33564	-1.29826	-1.05683
H	-2.53377	-1.77656	-2.98752
H	-4.23028	-1.90319	-1.16280
O	-4.11839	-0.70761	1.07202
C	-3.97194	0.00892	2.29360
H	-3.91823	1.09021	2.11820
H	-4.86045	-0.22032	2.88325
H	-3.07788	-0.31303	2.84121
C	1.47618	1.00713	-0.99622
C	2.09816	-0.10343	-1.80283
H	1.63234	-0.18272	-2.79338
H	3.14758	0.15942	-1.98848
C	2.04741	-1.48253	-1.11719
H	1.00010	-1.77751	-0.97662
H	2.49286	-2.22754	-1.79119
C	2.76878	-1.51998	0.23173
H	3.82245	-1.23540	0.09089
H	2.33374	-0.76040	0.89474
C	2.69895	-2.88875	0.91482
H	1.64478	-3.16938	1.05007
H	3.13460	-3.64965	0.25186
C	3.41403	-2.91785	2.26795
H	2.97575	-2.18752	2.95932
H	3.34776	-3.90574	2.73800
H	4.47700	-2.67054	2.15649
H	3.24994	2.30898	-0.58816

4e_conformer-063

C	0.49262	3.23585	-0.12042
C	-0.21956	1.08295	0.04628

N	-0.55802	2.37629	-0.32721
H	-1.41697	2.62408	-0.79696
C	1.51934	2.48636	0.42463
C	0.41284	4.68558	-0.47171
H	1.34427	5.18710	-0.19561
H	0.25534	4.83939	-1.54728
H	-0.40969	5.18890	0.05232
C	-1.18968	-0.00620	-0.05264
C	-0.78211	-1.33442	-0.29808
C	-2.56127	0.25379	0.08673
C	-1.72738	-2.35009	-0.38334
H	0.26621	-1.55863	-0.45216
C	-3.50766	-0.77209	-0.01714
H	-2.91938	1.25419	0.30896
C	-3.09458	-2.08972	-0.24796
H	-1.40198	-3.36887	-0.57522
H	-3.80947	-2.89945	-0.32511
O	-4.80911	-0.38740	0.13674
C	-5.81371	-1.39399	0.06436
H	-6.76362	-0.88002	0.21692
H	-5.81784	-1.88474	-0.91634
H	-5.67801	-2.15048	0.84667
C	1.08568	1.13138	0.53553
C	1.89893	0.00445	1.10572
H	2.45386	0.36640	1.98216
H	1.23830	-0.79263	1.46756
C	2.90846	-0.58933	0.10636
H	2.37393	-0.93712	-0.78919
H	3.57734	0.21132	-0.23294
C	3.71676	-1.74682	0.69975
H	4.28172	-1.38428	1.57138
H	3.01787	-2.50455	1.08157
C	4.68364	-2.41410	-0.28858
H	5.12872	-3.29492	0.19291
H	4.11411	-2.79075	-1.15006
C	5.80531	-1.49404	-0.78127
H	6.50092	-2.03385	-1.43377
H	5.41258	-0.64350	-1.34907
H	6.38059	-1.09251	0.06248
H	2.48445	2.87278	0.72795

4e_conformer-064

C	-0.49265	3.23581	-0.12041
C	0.21957	1.08292	0.04627
N	0.55800	2.37627	-0.32720

H	1.41695	2.62408	-0.79696
C	-1.51936	2.48629	0.42463
C	-0.41289	4.68555	-0.47168
H	0.40962	5.18888	0.05236
H	-0.25538	4.83936	-1.54725
H	-1.34434	5.18704	-0.19559
C	1.18971	-0.00621	-0.05265
C	0.78217	-1.33444	-0.29811
C	2.56129	0.25380	0.08674
C	1.72746	-2.35009	-0.38338
H	-0.26615	-1.55867	-0.45222
C	3.50770	-0.77206	-0.01713
H	2.91939	1.25420	0.30899
C	3.09466	-2.08970	-0.24797
H	1.40209	-3.36887	-0.57527
H	3.80956	-2.89941	-0.32512
O	4.80914	-0.38734	0.13677
C	5.81377	-1.39391	0.06438
H	5.81792	-1.88465	-0.91633
H	6.76366	-0.87992	0.21696
H	5.67807	-2.15041	0.84668
C	-1.08568	1.13132	0.53551
C	-1.89892	0.00438	1.10569
H	-1.23828	-0.79273	1.46747
H	-2.45382	0.36629	1.98215
C	-2.90848	-0.58935	0.10633
H	-3.57737	0.21131	-0.23290
H	-2.37399	-0.93711	-0.78925
C	-3.71677	-1.74687	0.69970
H	-3.01788	-2.50464	1.08141
H	-4.28166	-1.38438	1.57139
C	-4.68375	-2.41405	-0.28862
H	-4.11429	-2.79065	-1.15017
H	-5.12881	-3.29489	0.19284
C	-5.80544	-1.49392	-0.78114
H	-6.38063	-1.09244	0.06269
H	-5.41272	-0.64334	-1.34891
H	-6.50110	-2.03367	-1.43363
H	-2.48448	2.87270	0.72795

4e_conformer-065

C	-0.16416	3.66129	0.04032
C	0.09437	1.41304	0.27698
N	0.73998	2.63515	0.14690
H	1.73740	2.76099	0.24348

C	-1.42210	3.08532	0.07509
C	0.26233	5.08860	-0.07010
H	-0.61552	5.73232	-0.17153
H	0.82083	5.42015	0.81505
H	0.90656	5.25682	-0.94256
C	0.86098	0.17170	0.37479
C	0.34585	-0.96254	1.03778
C	2.14250	0.08339	-0.18935
C	1.09542	-2.12986	1.11280
H	-0.62855	-0.91954	1.50709
C	2.89774	-1.09160	-0.09604
H	2.56926	0.91480	-0.74148
C	2.37531	-2.21503	0.55475
H	0.68598	-2.99516	1.62686
H	2.93903	-3.13644	0.63098
O	4.12826	-1.04501	-0.68726
C	4.93132	-2.22008	-0.64476
H	5.85392	-1.97111	-1.17068
H	5.16561	-2.50674	0.38752
H	4.43796	-3.05930	-1.14975
C	-1.27748	1.67593	0.22865
C	-2.41441	0.68925	0.22778
H	-2.40744	0.08623	1.14309
H	-3.35980	1.24629	0.25058
C	-2.41685	-0.23661	-1.00354
H	-2.54472	0.38583	-1.89857
H	-1.43411	-0.71470	-1.09760
C	-3.50086	-1.32153	-0.96641
H	-4.48469	-0.85666	-0.80344
H	-3.55188	-1.80639	-1.95146
C	-3.26658	-2.40215	0.09687
H	-2.26468	-2.83112	-0.04503
H	-3.26516	-1.95017	1.09716
C	-4.31614	-3.51536	0.05078
H	-4.31731	-4.01490	-0.92589
H	-4.12890	-4.27669	0.81659
H	-5.32317	-3.11342	0.21760
H	-2.36010	3.62036	-0.00651

4e_conformer-066

C	1.51122	2.72566	0.06939
C	0.19541	0.92387	0.51521
N	0.23156	2.23165	0.05054
H	-0.59075	2.78185	-0.15295
C	2.32195	1.70343	0.53033

C	1.83020	4.12572	-0.34183
H	1.33397	4.86585	0.29951
H	2.90816	4.29603	-0.27589
H	1.51912	4.32978	-1.37435
C	-1.06723	0.18802	0.56380
C	-1.29318	-0.81859	1.52581
C	-2.09492	0.48269	-0.34388
C	-2.50489	-1.49780	1.55483
H	-0.52749	-1.04680	2.25750
C	-3.31850	-0.19623	-0.29885
H	-1.95511	1.22408	-1.12426
C	-3.53186	-1.19992	0.65275
H	-2.66782	-2.26999	2.30165
H	-4.46782	-1.74238	0.70019
O	-4.23480	0.18468	-1.23738
C	-5.48989	-0.48771	-1.25289
H	-5.36539	-1.56120	-1.43943
H	-6.03122	-0.34507	-0.30985
H	-6.05871	-0.04061	-2.06917
C	1.51114	0.56646	0.81868
C	2.04628	-0.77231	1.25682
H	3.12571	-0.66643	1.41743
H	1.63400	-1.06547	2.23099
C	1.79238	-1.91753	0.25530
H	2.25570	-2.83085	0.65029
H	0.71560	-2.11646	0.20667
C	2.30179	-1.64163	-1.16621
H	2.06991	-2.51355	-1.79422
H	1.73706	-0.79873	-1.58413
C	3.80237	-1.33344	-1.27670
H	4.04088	-1.14426	-2.33190
H	4.02422	-0.39899	-0.74606
C	4.70891	-2.44955	-0.74929
H	4.49475	-3.40109	-1.25221
H	5.76600	-2.21319	-0.91635
H	4.57240	-2.60716	0.32654
H	3.39722	1.75877	0.64571

4e_conformer-067

C	2.85395	-2.15303	-0.15839
C	0.78014	-1.21668	-0.17702
N	1.56866	-2.25017	0.31099
H	1.21061	-3.03532	0.83577
C	2.90209	-1.01534	-0.94458
C	3.90324	-3.16031	0.18150

H	4.05681	-3.24156	1.26518
H	3.64585	-4.16222	-0.18634
H	4.85553	-2.87270	-0.27209
C	-0.62400	-1.10857	0.21622
C	-1.06421	-1.61697	1.45783
C	-1.56936	-0.51093	-0.62803
C	-2.40258	-1.52074	1.81986
H	-0.35120	-2.05494	2.14928
C	-2.91211	-0.40302	-0.24921
H	-1.28418	-0.13240	-1.60203
C	-3.34257	-0.91382	0.98125
H	-2.72739	-1.91170	2.78016
H	-4.37799	-0.84422	1.29043
O	-3.72877	0.20682	-1.15884
C	-5.10972	0.33103	-0.83544
H	-5.57727	-0.65109	-0.69547
H	-5.25559	0.93300	0.06961
H	-5.57177	0.83544	-1.68508
C	1.60776	-0.41875	-0.97097
C	1.28079	0.88316	-1.65552
H	2.16636	1.20069	-2.22117
H	0.48676	0.74894	-2.40114
C	0.87575	2.01849	-0.69485
H	0.59689	2.89469	-1.29360
H	-0.02348	1.72063	-0.14066
C	1.98084	2.38996	0.29836
H	2.30596	1.47997	0.81811
H	2.85781	2.75738	-0.25599
C	1.56175	3.43766	1.33870
H	0.70111	3.05496	1.90579
H	2.37624	3.56089	2.06508
C	1.21203	4.80589	0.74442
H	2.05019	5.20305	0.15788
H	0.34165	4.74938	0.08154
H	0.98103	5.53235	1.53193
H	3.78285	-0.63681	-1.44822

4e_conformer-068

C	-2.25828	2.49352	-0.21996
C	-0.34537	1.32206	0.15335
N	-0.90802	2.43197	-0.46298
H	-0.41250	3.03221	-1.10659
C	-2.56529	1.42133	0.59661
C	-3.12129	3.56563	-0.80075
H	-2.78116	4.56742	-0.50896

H	-3.13359	3.53339	-1.89809
H	-4.15033	3.44487	-0.45168
C	1.09409	1.07381	0.05451
C	2.00136	2.15435	0.00559
C	1.60508	-0.22843	-0.01137
C	3.36675	1.91714	-0.09988
H	1.63279	3.17280	0.08006
C	2.98335	-0.45943	-0.09826
H	0.94120	-1.08319	-0.02755
C	3.87783	0.61601	-0.14732
H	4.05656	2.75596	-0.13287
H	4.94649	0.45775	-0.22250
O	3.35324	-1.77342	-0.14905
C	4.74107	-2.07018	-0.26321
H	4.81305	-3.15819	-0.29196
H	5.16463	-1.65166	-1.18422
H	5.30310	-1.69000	0.59835
C	-1.37109	0.67581	0.84108
C	-1.28479	-0.52612	1.74207
H	-0.23923	-0.82014	1.88465
H	-1.66511	-0.25398	2.73661
C	-2.10015	-1.73192	1.23322
H	-2.10896	-2.51137	2.00767
H	-3.14200	-1.41377	1.10267
C	-1.56956	-2.32727	-0.07411
H	-1.47498	-1.52850	-0.82273
H	-0.55455	-2.71119	0.10021
C	-2.43426	-3.45852	-0.64900
H	-1.89675	-3.92237	-1.48665
H	-2.55580	-4.24482	0.10958
C	-3.81252	-2.99965	-1.13692
H	-4.36732	-3.82897	-1.59018
H	-4.42329	-2.60261	-0.31879
H	-3.71534	-2.20887	-1.89133
H	-3.54815	1.19991	0.99344

4e_conformer-069

C	2.25847	2.49350	-0.21992
C	0.34548	1.32215	0.15331
N	0.90821	2.43205	-0.46296
H	0.41270	3.03242	-1.10646
C	2.56541	1.42123	0.59658
C	3.12160	3.56550	-0.80073
H	3.13434	3.53294	-1.89806
H	2.78125	4.56735	-0.50938

H	4.15051	3.44495	-0.45121
C	-1.09399	1.07396	0.05448
C	-2.00125	2.15451	0.00555
C	-1.60500	-0.22828	-0.01135
C	-3.36665	1.91730	-0.09991
H	-1.63267	3.17295	0.08002
C	-2.98326	-0.45927	-0.09824
H	-0.94111	-1.08304	-0.02749
C	-3.87774	0.61617	-0.14733
H	-4.05644	2.75612	-0.13292
H	-4.94639	0.45793	-0.22251
O	-3.35316	-1.77327	-0.14900
C	-4.74099	-2.07002	-0.26318
H	-5.16455	-1.65149	-1.18419
H	-4.81297	-3.15803	-0.29197
H	-5.30303	-1.68986	0.59838
C	1.37115	0.67579	0.84102
C	1.28474	-0.52613	1.74202
H	1.66510	-0.25400	2.73654
H	0.23915	-0.82005	1.88462
C	2.09996	-1.73203	1.23319
H	3.14182	-1.41396	1.10251
H	2.10879	-2.51141	2.00770
C	1.56921	-2.32746	-0.07404
H	0.55420	-2.71132	0.10041
H	1.47458	-1.52877	-0.82273
C	2.43382	-3.45880	-0.64889
H	2.55541	-4.24500	0.10978
H	1.89622	-3.92274	-1.48642
C	3.81204	-3.00001	-1.13699
H	3.71480	-2.20937	-1.89154
H	4.42286	-2.60281	-0.31898
H	4.36682	-3.82941	-1.59014
H	3.54825	1.19972	0.99339

4e_conformer-070

C	-0.30166	3.31917	0.02144
C	0.27690	1.12048	0.06029
N	0.73006	2.42722	0.17942
H	1.66713	2.67907	0.45925
C	-1.43803	2.57293	-0.23341
C	-0.09981	4.79463	0.13815
H	0.24587	5.08386	1.13923
H	0.64222	5.16418	-0.58111
H	-1.04187	5.31542	-0.05315

C	1.20162	-0.00728	0.15986
C	0.78351	-1.26088	0.65422
C	2.54141	0.14072	-0.22932
C	1.68539	-2.31564	0.73457
H	-0.23360	-1.39338	1.00177
C	3.44709	-0.92158	-0.12916
H	2.90090	1.07640	-0.64607
C	3.02156	-2.16625	0.35018
H	1.35249	-3.27571	1.11933
H	3.70394	-3.00337	0.42937
O	4.72076	-0.64704	-0.53891
C	5.68058	-1.69713	-0.47772
H	5.82363	-2.04819	0.55132
H	5.38781	-2.54319	-1.11104
H	6.61369	-1.27137	-0.84895
C	-1.08932	1.18945	-0.21249
C	-2.03784	0.05377	-0.47165
H	-2.71269	0.33485	-1.28929
H	-1.48623	-0.82949	-0.81649
C	-2.87760	-0.32794	0.76307
H	-2.19998	-0.52488	1.60435
H	-3.48480	0.53815	1.06106
C	-3.78432	-1.54633	0.54896
H	-3.16693	-2.41116	0.26323
H	-4.25535	-1.81060	1.50626
C	-4.88361	-1.34364	-0.50031
H	-5.46826	-0.44990	-0.23937
H	-4.43225	-1.13923	-1.47950
C	-5.81634	-2.55183	-0.61792
H	-5.25845	-3.45246	-0.90273
H	-6.31392	-2.75957	0.33741
H	-6.59400	-2.38770	-1.37249
H	-2.42299	2.97809	-0.42951

4e_conformer-071

C	0.30167	3.31917	0.02144
C	-0.27690	1.12049	0.06029
N	-0.73006	2.42722	0.17942
H	-1.66713	2.67908	0.45925
C	1.43803	2.57293	-0.23341
C	0.09981	4.79463	0.13816
H	-0.64222	5.16419	-0.58110
H	-0.24586	5.08386	1.13923
H	1.04188	5.31542	-0.05314
C	-1.20162	-0.00728	0.15986

C	-0.78351	-1.26088	0.65421
C	-2.54141	0.14072	-0.22932
C	-1.68539	-2.31563	0.73457
H	0.23360	-1.39338	1.00177
C	-3.44709	-0.92157	-0.12916
H	-2.90090	1.07641	-0.64607
C	-3.02156	-2.16625	0.35018
H	-1.35249	-3.27571	1.11933
H	-3.70394	-3.00337	0.42937
O	-4.72076	-0.64704	-0.53891
C	-5.68058	-1.69713	-0.47772
H	-5.82363	-2.04819	0.55132
H	-6.61369	-1.27137	-0.84895
H	-5.38781	-2.54319	-1.11104
C	1.08932	1.18945	-0.21249
C	2.03784	0.05377	-0.47165
H	1.48623	-0.82949	-0.81651
H	2.71270	0.33485	-1.28929
C	2.87759	-0.32796	0.76306
H	3.48479	0.53813	1.06107
H	2.19996	-0.52491	1.60434
C	3.78431	-1.54634	0.54895
H	4.25533	-1.81063	1.50625
H	3.16693	-2.41117	0.26320
C	4.88361	-1.34364	-0.50030
H	4.43227	-1.13921	-1.47949
H	5.46826	-0.44990	-0.23934
C	5.81635	-2.55182	-0.61792
H	6.31392	-2.75958	0.33742
H	5.25845	-3.45245	-0.90274
H	6.59401	-2.38768	-1.37248
H	2.42299	2.97809	-0.42951

4e_conformer-072

C	-0.80301	3.40214	-0.09927
C	-0.06530	1.25309	-0.03879
N	0.28050	2.57626	-0.27379
H	1.17396	2.87149	-0.64072
C	-1.85997	2.59717	0.28480
C	-0.72127	4.87654	-0.32522
H	0.04160	5.34598	0.30891
H	-0.47252	5.11920	-1.36667
H	-1.68322	5.34220	-0.09468
C	0.92815	0.18543	-0.13763
C	0.56714	-1.10736	-0.56914

C	2.27033	0.42964	0.18671
C	1.52519	-2.11092	-0.64907
H	-0.45324	-1.30523	-0.87231
C	3.23337	-0.58153	0.08681
H	2.58736	1.40156	0.55195
C	2.86402	-1.86739	-0.32660
H	1.23484	-3.10301	-0.98390
H	3.59214	-2.66530	-0.40331
O	4.50396	-0.21721	0.43010
C	5.52132	-1.21172	0.36864
H	5.64326	-1.59581	-0.65125
H	6.44163	-0.71786	0.68308
H	5.30488	-2.04656	1.04602
C	-1.41037	1.24286	0.32789
C	-2.24513	0.06582	0.74823
H	-1.59503	-0.75392	1.07211
H	-2.84645	0.34692	1.62448
C	-3.20386	-0.43045	-0.35065
H	-3.77833	0.42712	-0.72403
H	-2.62025	-0.79318	-1.20715
C	-4.18457	-1.51203	0.12524
H	-4.80663	-1.09017	0.92734
H	-4.86933	-1.75515	-0.69948
C	-3.54282	-2.81323	0.63202
H	-2.89824	-2.59982	1.49462
H	-4.34084	-3.46734	1.00737
C	-2.73950	-3.56849	-0.43097
H	-1.87034	-2.99455	-0.76900
H	-2.36914	-4.52326	-0.04102
H	-3.35733	-3.78225	-1.31208
H	-2.85627	2.94546	0.52749

4e_conformer-073

C	0.80302	3.40214	-0.09925
C	0.06530	1.25309	-0.03879
N	-0.28049	2.57626	-0.27378
H	-1.17396	2.87150	-0.64070
C	1.85997	2.59716	0.28480
C	0.72128	4.87654	-0.32519
H	0.47252	5.11922	-1.36663
H	-0.04159	5.34598	0.30895
H	1.68323	5.34220	-0.09465
C	-0.92815	0.18544	-0.13764
C	-0.56715	-1.10735	-0.56917
C	-2.27033	0.42965	0.18671

C	-1.52520	-2.11091	-0.64910
H	0.45323	-1.30523	-0.87234
C	-3.23337	-0.58152	0.08681
H	-2.58735	1.40156	0.55196
C	-2.86402	-1.86739	-0.32662
H	-1.23486	-3.10300	-0.98394
H	-3.59215	-2.66528	-0.40333
O	-4.50396	-0.21721	0.43011
C	-5.52132	-1.21172	0.36864
H	-5.64327	-1.59579	-0.65125
H	-5.30487	-2.04656	1.04601
H	-6.44162	-0.71786	0.68310
C	1.41037	1.24285	0.32787
C	2.24514	0.06581	0.74820
H	2.84647	0.34690	1.62445
H	1.59504	-0.75393	1.07209
C	3.20385	-0.43047	-0.35069
H	2.62022	-0.79322	-1.20717
H	3.77830	0.42710	-0.72410
C	4.18457	-1.51203	0.12520
H	4.86930	-1.75517	-0.69955
H	4.80668	-1.09015	0.92725
C	3.54285	-2.81321	0.63204
H	4.34089	-3.46733	1.00735
H	2.89833	-2.59978	1.49467
C	2.73946	-3.56849	-0.43088
H	1.87027	-2.99456	-0.76886
H	3.35722	-3.78227	-1.31203
H	2.36913	-4.52326	-0.04088
H	2.85628	2.94544	0.52749

4e_conformer-074

C	-1.75973	-3.20381	-0.25308
C	-0.56811	-1.34608	0.30050
N	-0.50396	-2.66897	-0.11560
H	0.35294	-3.19883	-0.18723
C	-2.65512	-2.19485	0.05569
C	-1.97913	-4.62645	-0.65201
H	-1.52029	-4.85477	-1.62249
H	-1.55794	-5.32818	0.07980
H	-3.05051	-4.82954	-0.73081
C	0.65285	-0.56303	0.48610
C	0.70462	0.51447	1.39577
C	1.81218	-0.87306	-0.24034
C	1.87620	1.24515	1.54872

H	-0.16575	0.76185	1.99087
C	2.99355	-0.14175	-0.06899
H	1.81342	-1.66808	-0.97930
C	3.03333	0.93199	0.82770
H	1.90205	2.07265	2.25239
H	3.93384	1.51648	0.96921
O	4.04865	-0.54568	-0.83661
C	5.27032	0.17663	-0.72118
H	5.14007	1.22886	-1.00141
H	5.67264	0.12098	0.29749
H	5.96618	-0.30010	-1.41277
C	-1.92378	-1.02405	0.40968
C	-2.54398	0.30970	0.73188
H	-3.63421	0.18717	0.76117
H	-2.25979	0.64588	1.73850
C	-2.19081	1.41551	-0.27748
H	-1.10133	1.48302	-0.36005
H	-2.55995	1.12363	-1.27049
C	-2.76817	2.77917	0.11043
H	-3.86064	2.69515	0.19803
H	-2.40041	3.05243	1.11104
C	-2.42580	3.90706	-0.87350
H	-2.79335	3.63849	-1.87405
H	-2.97293	4.81247	-0.57909
C	-0.92949	4.23035	-0.94818
H	-0.53477	4.48302	0.04405
H	-0.34832	3.38302	-1.32781
H	-0.74187	5.08305	-1.61054
H	-3.73412	-2.28207	0.02541

4e_conformer-075

C	2.23342	-2.69220	-0.27239
C	0.38076	-1.43545	0.12636
N	0.87581	-2.59483	-0.45584
H	0.33008	-3.20940	-1.04287
C	2.61485	-1.59119	0.47125
C	3.03149	-3.82301	-0.83445
H	2.67550	-4.79545	-0.47098
H	2.98865	-3.85050	-1.93114
H	4.08030	-3.71883	-0.54378
C	-1.05270	-1.14107	0.08216
C	-1.99693	-2.18945	0.13149
C	-1.52255	0.17382	-0.02623
C	-3.35714	-1.90898	0.07801
H	-1.65971	-3.21554	0.24114

C	-2.89491	0.44934	-0.06083
H	-0.83240	1.00270	-0.11681
C	-3.82619	-0.59439	-0.01260
H	-4.07539	-2.72313	0.12057
H	-4.89136	-0.40194	-0.04606
O	-3.22255	1.77158	-0.16215
C	-4.60346	2.11188	-0.22864
H	-4.64017	3.19894	-0.31075
H	-5.08369	1.66287	-1.10647
H	-5.13679	1.79615	0.67612
C	1.45941	-0.79183	0.73075
C	1.45504	0.45614	1.57078
H	0.42668	0.78628	1.75411
H	1.88193	0.22351	2.55636
C	2.26948	1.61525	0.96085
H	2.33112	2.42488	1.69856
H	3.29982	1.27218	0.79000
C	1.69491	2.14774	-0.35522
H	1.51398	1.30034	-1.02919
H	0.71410	2.60751	-0.16226
C	2.59366	3.16919	-1.06568
H	3.56425	2.70134	-1.28376
H	2.14755	3.41710	-2.03801
C	2.81690	4.46154	-0.27367
H	3.34397	4.27346	0.66813
H	3.41257	5.17991	-0.84834
H	1.86016	4.93986	-0.02855
H	3.62233	-1.38518	0.81047

4e_conformer-076

C	-3.93742	0.20752	-0.24690
C	-1.72935	-0.33068	-0.13733
N	-3.03157	-0.80930	-0.08305
H	-3.27536	-1.78875	-0.05130
C	-3.20388	1.37322	-0.38355
C	-5.41088	-0.03669	-0.26801
H	-5.76092	-0.50842	0.65907
H	-5.94338	0.91103	-0.38453
H	-5.70677	-0.69180	-1.09783
C	-0.59303	-1.23098	0.04979
C	-0.73153	-2.43145	0.78114
C	0.66683	-0.92457	-0.48238
C	0.36051	-3.27146	0.96383
H	-1.68329	-2.68590	1.23684
C	1.76713	-1.76144	-0.27089

H	0.81938	-0.03258	-1.07512
C	1.62198	-2.95178	0.45097
H	0.24013	-4.18941	1.53252
H	2.45983	-3.61652	0.62072
O	2.94191	-1.32732	-0.81858
C	4.10015	-2.13471	-0.63510
H	3.97129	-3.12635	-1.08536
H	4.91320	-1.61002	-1.13844
H	4.34239	-2.24908	0.42829
C	-1.81707	1.05262	-0.32205
C	-0.69750	2.05847	-0.36803
H	-0.01865	1.84221	-1.20134
H	-1.12371	3.04548	-0.58820
C	0.10477	2.14912	0.94476
H	-0.56301	2.53072	1.72790
H	0.40332	1.14171	1.25789
C	1.35605	3.03243	0.85221
H	1.07928	4.03113	0.48253
H	1.75831	3.18264	1.86401
C	2.46756	2.45349	-0.03340
H	2.70212	1.43301	0.29880
H	2.11171	2.35921	-1.06763
C	3.73893	3.30583	-0.01847
H	4.14600	3.38768	0.99697
H	4.51808	2.87594	-0.65826
H	3.53579	4.32299	-0.37586
H	-3.61945	2.36506	-0.51113

4e_conformer-077

C	2.06439	2.47940	-0.54722
C	0.21648	1.18807	-0.23894
N	0.77194	2.45151	-0.08833
H	0.25039	3.26653	0.20135
C	2.35751	1.19765	-0.97859
C	2.88558	3.72708	-0.53859
H	3.88705	3.51569	-0.92288
H	2.44494	4.51351	-1.16523
H	2.99260	4.13938	0.47290
C	-1.15176	0.92270	0.20227
C	-1.72490	1.65695	1.25509
C	-1.92654	-0.06850	-0.43248
C	-3.03711	1.40171	1.65746
H	-1.13755	2.40269	1.78165
C	-3.22998	-0.32996	-0.00780
H	-1.50314	-0.61067	-1.26583

C	-3.79500	0.41263	1.04040
H	-3.46833	1.97324	2.47465
H	-4.81203	0.19649	1.35098
O	-4.03741	-1.28108	-0.56314
C	-3.49700	-2.08914	-1.60363
H	-4.28268	-2.79657	-1.87192
H	-3.23323	-1.48732	-2.48174
H	-2.61044	-2.63768	-1.26275
C	1.20728	0.37517	-0.79555
C	1.16950	-1.10641	-1.06966
H	0.40697	-1.35348	-1.82002
H	2.12601	-1.38709	-1.52617
C	0.92767	-1.98117	0.17748
H	-0.08667	-1.79941	0.55068
H	0.95741	-3.03465	-0.12943
C	1.91894	-1.73902	1.32429
H	1.78391	-0.71519	1.69521
H	1.65789	-2.40822	2.15639
C	3.39839	-1.94362	0.96630
H	3.69957	-1.20197	0.21581
H	4.00346	-1.73221	1.85808
C	3.72805	-3.35014	0.45765
H	3.21756	-3.56865	-0.48708
H	4.80414	-3.46684	0.28563
H	3.42002	-4.11325	1.18360
H	3.30886	0.87330	-1.38148

4e_conformer-078

C	-2.06486	2.47941	-0.54685
C	-0.21672	1.18829	-0.23909
N	-0.77229	2.45166	-0.08833
H	-0.25081	3.26670	0.20138
C	-2.35795	1.19764	-0.97817
C	-2.88618	3.72701	-0.53802
H	-2.99300	4.13930	0.47350
H	-2.44578	4.51349	-1.16477
H	-3.88773	3.51551	-0.92207
C	1.15165	0.92313	0.20187
C	1.72506	1.65799	1.25413
C	1.92628	-0.06846	-0.43246
C	3.03735	1.40295	1.65637
H	1.13789	2.40410	1.78036
C	3.22979	-0.32972	-0.00788
H	1.50273	-0.61113	-1.26542
C	3.79508	0.41347	1.03974

H	3.46876	1.97496	2.47312
H	4.81214	0.19745	1.35026
O	4.03705	-1.28125	-0.56279
C	3.49627	-2.09022	-1.60239
H	2.60985	-2.63849	-1.26071
H	3.23217	-1.48918	-2.48094
H	4.28186	-2.79788	-1.87035
C	-1.20758	0.37528	-0.79547
C	-1.16966	-1.10621	-1.07004
H	-2.12633	-1.38689	-1.52623
H	-0.40744	-1.35279	-1.82088
C	-0.92711	-1.98152	0.17657
H	-0.95709	-3.03486	-0.13081
H	0.08744	-1.79999	0.54928
C	-1.91768	-1.73984	1.32409
H	-1.65611	-2.40936	2.15577
H	-1.78233	-0.71615	1.69530
C	-3.39736	-1.94426	0.96696
H	-4.00187	-1.73304	1.85917
H	-3.69896	-1.20240	0.21685
C	-3.72742	-3.35063	0.45816
H	-3.41905	-4.11394	1.18376
H	-4.80361	-3.46720	0.28671
H	-3.21747	-3.56895	-0.48691
H	-3.30934	0.87320	-1.38089

4e_conformer-079

C	0.53985	2.96650	-0.31780
C	-0.14249	0.84677	0.14381
N	-0.43458	2.02302	-0.53432
H	-1.18558	2.11665	-1.20329
C	1.45892	2.39911	0.54469
C	0.49952	4.31177	-0.96601
H	1.34574	4.91452	-0.62551
H	0.55636	4.24234	-2.06025
H	-0.42213	4.85549	-0.72244
C	-1.06414	-0.28939	0.08452
C	-0.60144	-1.61460	0.07931
C	-2.45309	-0.05218	0.01310
C	-1.51004	-2.67330	0.02103
H	0.46259	-1.81336	0.08541
C	-3.35137	-1.11869	-0.06375
H	-2.81437	0.96814	0.05122
C	-2.87871	-2.43984	-0.05309
H	-1.14211	-3.69561	0.01615

H	-3.59372	-3.25442	-0.10673
O	-4.70730	-0.97584	-0.13691
C	-5.24031	0.34428	-0.15927
H	-4.86867	0.91009	-1.02213
H	-6.32203	0.22886	-0.23836
H	-4.99796	0.88882	0.76136
C	1.04254	1.06536	0.84423
C	1.74441	0.14571	1.80603
H	1.83234	0.64745	2.77988
H	1.14188	-0.75279	1.97843
C	3.15992	-0.25977	1.34877
H	3.75378	0.65068	1.18724
H	3.65320	-0.81019	2.16160
C	3.18364	-1.10683	0.07478
H	2.66179	-2.05733	0.26024
H	2.61716	-0.59218	-0.71339
C	4.59845	-1.40545	-0.42974
H	5.11861	-0.45676	-0.62312
H	5.16832	-1.91048	0.36290
C	4.60971	-2.26444	-1.69663
H	5.63137	-2.46100	-2.04113
H	4.12362	-3.23186	-1.51997
H	4.07028	-1.76771	-2.51251
H	2.33774	2.89617	0.93599

4e_conformer-080

C	-0.53985	2.96650	-0.31779
C	0.14249	0.84677	0.14381
N	0.43458	2.02302	-0.53431
H	1.18558	2.11665	-1.20329
C	-1.45892	2.39911	0.54469
C	-0.49951	4.31177	-0.96601
H	0.42214	4.85548	-0.72244
H	-0.55636	4.24234	-2.06025
H	-1.34573	4.91453	-0.62550
C	1.06413	-0.28939	0.08452
C	0.60144	-1.61460	0.07933
C	2.45308	-0.05218	0.01310
C	1.51004	-2.67330	0.02105
H	-0.46259	-1.81337	0.08543
C	3.35137	-1.11869	-0.06375
H	2.81436	0.96814	0.05121
C	2.87871	-2.43984	-0.05309
H	1.14211	-3.69561	0.01617
H	3.59372	-3.25442	-0.10672

O	4.70730	-0.97584	-0.13691
C	5.24031	0.34428	-0.15928
H	4.86866	0.91009	-1.02214
H	4.99796	0.88882	0.76135
H	6.32202	0.22886	-0.23838
C	-1.04255	1.06536	0.84424
C	-1.74442	0.14571	1.80604
H	-1.14189	-0.75279	1.97844
H	-1.83235	0.64745	2.77988
C	-3.15992	-0.25977	1.34876
H	-3.65321	-0.81018	2.16160
H	-3.75378	0.65069	1.18723
C	-3.18364	-1.10683	0.07478
H	-2.61716	-0.59218	-0.71340
H	-2.66179	-2.05733	0.26024
C	-4.59844	-1.40544	-0.42975
H	-5.16832	-1.91048	0.36289
H	-5.11860	-0.45676	-0.62314
C	-4.60970	-2.26444	-1.69663
H	-4.07027	-1.76772	-2.51252
H	-4.12362	-3.23186	-1.51998
H	-5.63136	-2.46100	-2.04114
H	-2.33774	2.89617	0.93599

4e_conformer-081

C	2.25081	-2.42282	-0.05314
C	0.68387	-0.90035	0.57219
N	0.90622	-2.18966	0.10892
H	0.18835	-2.89694	0.03900
C	2.90228	-1.24953	0.27899
C	2.77747	-3.74956	-0.49360
H	2.54100	-4.54535	0.22493
H	2.36133	-4.05289	-1.46276
H	3.86518	-3.70345	-0.59441
C	-0.67646	-0.40390	0.78626
C	-0.98687	0.43374	1.86896
C	-1.70008	-0.76911	-0.11173
C	-2.28804	0.91379	2.03104
H	-0.21932	0.68487	2.59212
C	-3.00209	-0.29773	0.07071
H	-1.45256	-1.39065	-0.96344
C	-3.29783	0.55575	1.14456
H	-2.52025	1.56382	2.86999
H	-4.31403	0.91763	1.26317
O	-4.04887	-0.59856	-0.75225

C	-3.80106	-1.45579	-1.86197
H	-3.07295	-1.01390	-2.55289
H	-3.43875	-2.43779	-1.53462
H	-4.75927	-1.57228	-2.36982
C	1.92768	-0.28253	0.67639
C	2.21404	1.16007	0.98924
H	1.30020	1.65991	1.32175
H	2.92209	1.22949	1.82665
C	2.80361	1.93126	-0.21008
H	3.01584	2.96041	0.10739
H	3.77112	1.48777	-0.47919
C	1.90551	1.94061	-1.45556
H	2.41564	2.50685	-2.24788
H	1.80393	0.91188	-1.82396
C	0.50137	2.52934	-1.24781
H	-0.03913	2.47695	-2.20257
H	-0.06278	1.90205	-0.54838
C	0.49861	3.97702	-0.74819
H	0.94009	4.06020	0.25145
H	1.07363	4.62852	-1.41849
H	-0.52194	4.37263	-0.69071
H	3.97299	-1.09524	0.23062

4e_conformer-082

C	1.07237	2.82066	-0.15280
C	-0.02551	0.85409	0.16190
N	-0.08564	2.12446	-0.39415
H	-0.83378	2.44736	-0.99069
C	1.88566	1.99162	0.59821
C	1.29059	4.20371	-0.67317
H	0.51675	4.89904	-0.32371
H	2.25931	4.58033	-0.33395
H	1.28432	4.23520	-1.77057
C	-1.16994	-0.05209	0.07866
C	-1.00497	-1.45306	0.06355
C	-2.47305	0.46351	0.00403
C	-2.11497	-2.28652	-0.01271
H	-0.01074	-1.88091	0.08033
C	-3.58404	-0.38221	-0.08972
H	-2.65300	1.53305	0.04804
C	-3.41243	-1.77165	-0.09277
H	-1.97436	-3.36385	-0.02633
H	-4.25849	-2.44438	-0.15769
O	-4.79510	0.24564	-0.15810
C	-5.96228	-0.56641	-0.23551

H	-5.95673	-1.18892	-1.13834
H	-6.80522	0.12466	-0.27632
H	-6.06026	-1.21014	0.64693
C	1.21126	0.74990	0.80135
C	1.73820	-0.41051	1.59970
H	2.29929	-0.02486	2.46193
H	0.90053	-0.98702	2.00901
C	2.66107	-1.36452	0.81193
H	2.89378	-2.23064	1.44779
H	2.11665	-1.75328	-0.05759
C	3.97019	-0.71691	0.35006
H	3.74315	0.12776	-0.31339
H	4.48024	-0.29100	1.22606
C	4.92630	-1.68091	-0.36669
H	5.16020	-2.52320	0.29996
H	5.87698	-1.16283	-0.55034
C	4.38684	-2.21746	-1.69689
H	4.12896	-1.39360	-2.37427
H	5.13057	-2.84508	-2.20091
H	3.48537	-2.82360	-1.55522
H	2.86508	2.25219	0.97827

4e_conformer-083

C	-3.68398	-0.55034	-0.29047
C	-1.45443	-0.82009	0.06461
N	-2.61922	-1.40928	-0.40779
H	-2.65278	-2.30127	-0.88011
C	-3.20105	0.60566	0.29613
C	-5.05478	-0.91743	-0.75689
H	-5.43058	-1.81813	-0.25479
H	-5.75041	-0.10058	-0.54675
H	-5.08247	-1.11205	-1.83701
C	-0.19099	-1.55505	0.07361
C	-0.17858	-2.96107	0.20632
C	1.03590	-0.88977	-0.05535
C	1.02592	-3.65382	0.20880
H	-1.11098	-3.50122	0.33851
C	2.24586	-1.59367	-0.03408
H	1.07612	0.18187	-0.20464
C	2.25046	-2.98729	0.09441
H	1.02247	-4.73510	0.31537
H	3.17546	-3.55025	0.10572
O	3.36878	-0.82661	-0.16353
C	4.62923	-1.48856	-0.17569
H	4.80698	-2.02679	0.76313

H	4.70335	-2.19106	-1.01462
H	5.37808	-0.70397	-0.29158
C	-1.80168	0.45006	0.52546
C	-0.90504	1.46927	1.16791
H	-1.44856	1.97235	1.97909
H	-0.04625	0.97141	1.63498
C	-0.39097	2.54018	0.18814
H	0.09049	2.04305	-0.66319
H	-1.24802	3.08793	-0.22800
C	0.58198	3.52279	0.84555
H	0.08668	3.98696	1.70997
H	1.43974	2.96318	1.24794
C	1.09612	4.62504	-0.09125
H	0.24091	5.18969	-0.48882
H	1.68870	5.33931	0.49558
C	1.94940	4.10406	-1.25257
H	2.79939	3.51819	-0.88041
H	1.37203	3.45909	-1.92385
H	2.34871	4.93046	-1.85138
H	-3.79497	1.47496	0.54975

4e_conformer-084

C	-0.61833	3.42010	0.24289
C	0.47889	1.44962	-0.04757
N	0.60898	2.80526	0.22141
H	1.47749	3.25163	0.47845
C	-1.55706	2.44608	-0.04629
C	-0.77199	4.87504	0.54420
H	-0.41808	5.12504	1.55292
H	-0.21049	5.50059	-0.16144
H	-1.82584	5.15898	0.48013
C	1.65415	0.58745	-0.15191
C	2.89272	1.10339	-0.57286
C	1.56748	-0.78036	0.17661
C	4.00797	0.26904	-0.66192
H	2.97762	2.14663	-0.86098
C	2.68458	-1.60991	0.06340
H	0.63321	-1.17310	0.54886
C	3.91646	-1.08446	-0.35375
H	4.95917	0.67838	-0.99069
H	4.77426	-1.74501	-0.42733
O	2.67418	-2.94419	0.35485
C	1.44031	-3.52848	0.75989
H	0.67112	-3.41158	-0.01332
H	1.64640	-4.58882	0.91111

H	1.07762	-3.09011	1.69750
C	-0.88237	1.20294	-0.23145
C	-1.54733	-0.09676	-0.58625
H	-2.36198	0.10458	-1.29270
H	-0.84231	-0.75433	-1.10990
C	-2.12011	-0.83992	0.63611
H	-1.32962	-0.95581	1.38933
H	-2.88478	-0.20894	1.10648
C	-2.69824	-2.22329	0.30750
H	-1.88825	-2.86624	-0.06602
H	-3.05315	-2.68588	1.23916
C	-3.84424	-2.22811	-0.71629
H	-3.47471	-1.87842	-1.68902
H	-4.16536	-3.26646	-0.87169
C	-5.05212	-1.38444	-0.29783
H	-5.87118	-1.48272	-1.01912
H	-5.42963	-1.69785	0.68360
H	-4.79823	-0.32058	-0.22998
H	-2.62431	2.60924	-0.13092

4e_conformer-085

C	0.61830	3.42010	0.24289
C	-0.47890	1.44962	-0.04757
N	-0.60900	2.80526	0.22141
H	-1.47752	3.25162	0.47846
C	1.55703	2.44610	-0.04629
C	0.77195	4.87505	0.54420
H	0.21048	5.50059	-0.16146
H	0.41799	5.12505	1.55291
H	1.82580	5.15899	0.48018
C	-1.65416	0.58744	-0.15191
C	-2.89272	1.10337	-0.57288
C	-1.56748	-0.78037	0.17662
C	-4.00797	0.26901	-0.66193
H	-2.97763	2.14661	-0.86099
C	-2.68458	-1.60992	0.06340
H	-0.63320	-1.17310	0.54887
C	-3.91645	-1.08448	-0.35375
H	-4.95917	0.67834	-0.99072
H	-4.77424	-1.74504	-0.42734
O	-2.67417	-2.94421	0.35486
C	-1.44030	-3.52849	0.75990
H	-0.67111	-3.41158	-0.01330
H	-1.07762	-3.09013	1.69751
H	-1.64638	-4.58884	0.91111

C	0.88236	1.20294	-0.23144
C	1.54733	-0.09674	-0.58625
H	0.84231	-0.75433	-1.10989
H	2.36197	0.10460	-1.29271
C	2.12013	-0.83990	0.63611
H	2.88480	-0.20891	1.10646
H	1.32966	-0.95579	1.38934
C	2.69827	-2.22327	0.30750
H	3.05317	-2.68585	1.23916
H	1.88827	-2.86621	-0.06601
C	3.84426	-2.22809	-0.71630
H	4.16538	-3.26643	-0.87171
H	3.47473	-1.87838	-1.68902
C	5.05215	-1.38443	-0.29783
H	4.79827	-0.32056	-0.22997
H	5.42966	-1.69786	0.68359
H	5.87120	-1.48270	-1.01913
H	2.62429	2.60927	-0.13091

4e_conformer-086

C	0.91151	3.23627	-0.15456
C	-0.40979	1.40494	0.11267
N	-0.33471	2.70341	-0.37136
H	-1.06243	3.14953	-0.91101
C	1.64722	2.27035	0.50805
C	1.27856	4.61017	-0.61167
H	2.30198	4.84292	-0.30547
H	1.22520	4.70739	-1.70393
H	0.61638	5.37407	-0.18456
C	-1.66018	0.65163	0.03829
C	-2.90051	1.31443	0.05476
C	-1.64590	-0.75405	-0.05813
C	-4.08816	0.58590	-0.02438
H	-2.93855	2.39396	0.16220
C	-2.84081	-1.47391	-0.11728
H	-0.69770	-1.26658	-0.11794
C	-4.07240	-0.80278	-0.10550
H	-5.04033	1.10889	-0.00851
H	-4.98906	-1.38094	-0.16041
O	-2.90529	-2.83521	-0.20510
C	-1.68012	-3.56115	-0.21232
H	-1.10011	-3.37471	0.69967
H	-1.95699	-4.61510	-0.25912
H	-1.06877	-3.30539	-1.08628
C	0.83201	1.11123	0.68028

C	1.24094	-0.14815	1.39368
H	1.89600	0.11619	2.23497
H	0.35704	-0.62712	1.83106
C	1.98220	-1.18107	0.51735
H	2.11148	-2.09769	1.10654
H	1.35616	-1.45094	-0.34490
C	3.34073	-0.69501	0.00361
H	3.19695	0.24606	-0.54099
H	3.98721	-0.45908	0.86241
C	4.05855	-1.69680	-0.91203
H	3.41641	-1.91676	-1.77691
H	4.96183	-1.22036	-1.31575
C	4.45192	-3.00758	-0.22300
H	5.08056	-2.81412	0.65537
H	3.57377	-3.56835	0.11547
H	5.01644	-3.65690	-0.90185
H	2.66723	2.38636	0.85130

4e_conformer-087

C	-3.67711	0.70697	-0.18588
C	-1.61817	-0.26024	-0.11208
N	-2.96669	-0.40600	0.18485
H	-3.38446	-1.26445	0.51395
C	-2.76254	1.60265	-0.71150
C	-5.15780	0.79935	-0.01208
H	-5.68822	0.03475	-0.59461
H	-5.45522	0.67321	1.03680
H	-5.51122	1.77851	-0.34615
C	-0.66501	-1.31630	0.22410
C	-0.93584	-2.23230	1.25638
C	0.54768	-1.43915	-0.48396
C	-0.01891	-3.23869	1.56398
H	-1.84314	-2.14006	1.84493
C	1.46825	-2.43347	-0.15122
H	0.75094	-0.76118	-1.29948
C	1.18366	-3.34774	0.87427
H	-0.23999	-3.93761	2.36589
H	1.91104	-4.11664	1.11336
O	2.66993	-2.59958	-0.77902
C	3.03661	-1.65301	-1.77750
H	3.06445	-0.63492	-1.36965
H	4.03508	-1.93997	-2.10991
H	2.34720	-1.68116	-2.62995
C	-1.46721	1.00989	-0.67807
C	-0.20112	1.70434	-1.10567

H	0.30627	1.14680	-1.90523
H	-0.47203	2.66929	-1.55273
C	0.79550	1.95181	0.04004
H	0.31308	2.59212	0.78860
H	1.01764	1.00178	0.54133
C	2.10486	2.58114	-0.44432
H	2.51930	1.95394	-1.24713
H	1.89525	3.56065	-0.89935
C	3.16612	2.74569	0.65239
H	3.39061	1.75973	1.08374
H	4.09882	3.09858	0.19266
C	2.76260	3.71203	1.77055
H	2.51733	4.70097	1.36313
H	1.88514	3.35288	2.31929
H	3.57546	3.84055	2.49427
H	-2.99540	2.59318	-1.08210

4e_conformer-088

C	1.25793	3.39129	-0.22053
C	-0.11412	1.60328	0.07986
N	-0.02480	2.91948	-0.35218
H	-0.77435	3.41814	-0.80971
C	2.00404	2.36772	0.33525
C	1.64351	4.77052	-0.64549
H	1.49130	4.92609	-1.72157
H	1.06100	5.53848	-0.12061
H	2.70099	4.94446	-0.42919
C	-1.39221	0.89418	0.06520
C	-2.60589	1.59818	0.22564
C	-1.44857	-0.49451	-0.11532
C	-3.81768	0.91859	0.20430
H	-2.59211	2.67003	0.39747
C	-2.67280	-1.17382	-0.11759
H	-0.54780	-1.07064	-0.28722
C	-3.87202	-0.46932	0.03848
H	-4.74464	1.47073	0.33241
H	-4.82921	-0.97572	0.03202
O	-2.58976	-2.52538	-0.29736
C	-3.80401	-3.26798	-0.33251
H	-4.44844	-2.94117	-1.15757
H	-3.51429	-4.30804	-0.48786
H	-4.35381	-3.17961	0.61225
C	1.15456	1.23799	0.52996
C	1.57484	-0.07181	1.13305
H	2.26749	0.11786	1.96442

H	0.70651	-0.58323	1.56614
C	2.26819	-1.01344	0.13134
H	1.60257	-1.18885	-0.72587
H	3.15253	-0.50504	-0.27247
C	2.65973	-2.35594	0.75541
H	3.35582	-2.17865	1.58871
H	1.76291	-2.81281	1.19742
C	3.29113	-3.34998	-0.22955
H	3.43036	-4.31240	0.28049
H	2.58625	-3.53807	-1.05181
C	4.63492	-2.89033	-0.80432
H	4.53028	-1.97749	-1.40085
H	5.35278	-2.68135	-0.00113
H	5.07150	-3.65953	-1.45143
H	3.05477	2.42576	0.59093

4e_conformer-089

C	-1.65896	2.67993	-0.28982
C	-0.35787	0.88815	0.22756
N	-0.43336	2.08781	-0.46840
H	0.27054	2.40966	-1.11708
C	-2.37634	1.86156	0.56292
C	-2.02492	3.96362	-0.96041
H	-1.33311	4.77415	-0.69790
H	-2.01737	3.87143	-2.05437
H	-3.03036	4.26834	-0.65782
C	0.86562	0.08608	0.18789
C	0.82811	-1.32347	0.21727
C	2.11492	0.71666	0.09221
C	2.00946	-2.05451	0.16512
H	-0.12339	-1.83749	0.24981
C	3.29887	-0.02695	0.02342
H	2.19292	1.79942	0.09658
C	3.25434	-1.42562	0.06593
H	1.96776	-3.14007	0.18529
H	4.15837	-2.02007	0.02037
O	4.44751	0.70590	-0.06923
C	5.68390	0.00149	-0.12358
H	5.73522	-0.64793	-1.00578
H	6.46031	0.76479	-0.18907
H	5.83979	-0.60162	0.77897
C	-1.57176	0.72925	0.89412
C	-1.97773	-0.35422	1.85555
H	-1.12711	-1.01034	2.07027
H	-2.24332	0.11737	2.81237

C	-3.18601	-1.21107	1.41735
H	-3.53343	-1.76597	2.29886
H	-4.01175	-0.54581	1.12932
C	-2.92710	-2.22010	0.28862
H	-2.05239	-2.83510	0.54957
H	-3.77540	-2.91860	0.24845
C	-2.73302	-1.62433	-1.11136
H	-3.61435	-1.01882	-1.36541
H	-1.88316	-0.93528	-1.11221
C	-2.51894	-2.70253	-2.17643
H	-3.36554	-3.39963	-2.21323
H	-2.40166	-2.26421	-3.17414
H	-1.61679	-3.28948	-1.96231
H	-3.37772	2.05982	0.92399

4e_conformer-090

C	-3.95503	-0.16957	-0.06287
C	-1.69735	-0.43921	-0.14318
N	-2.91645	-1.04508	0.12822
H	-3.02890	-2.02833	0.32947
C	-3.39162	1.03680	-0.44159
C	-5.38083	-0.57386	0.12412
H	-5.57195	-0.94304	1.13985
H	-6.03632	0.28352	-0.05059
H	-5.67695	-1.36952	-0.57200
C	-0.44189	-1.16685	0.03665
C	-0.34965	-2.23564	0.95510
C	0.71044	-0.80788	-0.67615
C	0.85964	-2.89348	1.14623
H	-1.21327	-2.52147	1.54730
C	1.93046	-1.45491	-0.45668
H	0.68606	-0.01358	-1.41128
C	2.01514	-2.51248	0.45623
H	0.91800	-3.70916	1.86162
H	2.94920	-3.02927	0.63800
O	2.98260	-0.97929	-1.18843
C	4.25708	-1.58212	-0.98858
H	4.59145	-1.46982	0.04962
H	4.24122	-2.64661	-1.25161
H	4.94452	-1.05442	-1.65080
C	-1.97663	0.88400	-0.50012
C	-0.99699	1.98008	-0.82766
H	-0.43785	1.73278	-1.74104
H	-1.55639	2.89236	-1.06933
C	0.00961	2.27910	0.30014

H	-0.50773	2.79191	1.12239
H	0.37087	1.33218	0.70872
C	1.20396	3.11273	-0.17269
H	1.62536	2.64526	-1.07551
H	0.85767	4.11001	-0.47820
C	2.32289	3.25236	0.86983
H	3.07464	3.95564	0.48760
H	1.91775	3.70522	1.78590
C	3.01051	1.92455	1.21313
H	3.36665	1.42015	0.30641
H	3.87096	2.08537	1.87271
H	2.33072	1.23239	1.72126
H	-3.93984	1.94670	-0.65204

4e_conformer-091

C	-0.77416	3.45676	-0.26589
C	-0.05026	1.38493	0.33542
N	0.31303	2.62564	-0.17052
H	1.26811	2.91522	-0.32554
C	-1.86851	2.72636	0.16270
C	-0.65758	4.86729	-0.74350
H	-0.01476	5.47086	-0.08940
H	-0.23571	4.92326	-1.75512
H	-1.64534	5.33545	-0.76504
C	0.94679	0.32765	0.49763
C	0.80041	-0.67862	1.47575
C	2.08731	0.29779	-0.31879
C	1.76487	-1.66972	1.60905
H	-0.05559	-0.66705	2.13918
C	3.06152	-0.69620	-0.16864
H	2.22788	1.02704	-1.11043
C	2.90465	-1.69548	0.79849
H	1.64062	-2.43799	2.36728
H	3.64212	-2.47799	0.92648
O	4.12140	-0.61195	-1.02614
C	5.13112	-1.61188	-0.93567
H	4.72261	-2.61061	-1.13161
H	5.86729	-1.36380	-1.70136
H	5.61294	-1.60532	0.04945
C	-1.43073	1.42596	0.54873
C	-2.34424	0.31396	0.99148
H	-2.06798	-0.05697	1.98766
H	-3.35648	0.72379	1.10151
C	-2.39404	-0.87298	0.01398
H	-2.71029	-0.50103	-0.96832

H	-1.38204	-1.27639	-0.11772
C	-3.32604	-1.99096	0.49000
H	-3.02545	-2.29158	1.50385
H	-4.35063	-1.59961	0.57752
C	-3.33287	-3.23164	-0.41425
H	-2.31156	-3.63283	-0.48065
H	-3.93893	-4.01371	0.06215
C	-3.87128	-2.97204	-1.82496
H	-3.24274	-2.26280	-2.37443
H	-3.91258	-3.89852	-2.40890
H	-4.88562	-2.55523	-1.78581
H	-2.88994	3.08493	0.19282

4e_conformer-092

C	3.95504	-0.16972	-0.06291
C	1.69733	-0.43912	-0.14307
N	2.91638	-1.04511	0.12828
H	3.02874	-2.02837	0.32953
C	3.39173	1.03670	-0.44165
C	5.38080	-0.57415	0.12400
H	5.67682	-1.36984	-0.57213
H	6.03637	0.28317	-0.05073
H	5.57194	-0.94336	1.13972
C	0.44181	-1.16669	0.03673
C	0.34951	-2.23552	0.95513
C	-0.71049	-0.80768	-0.67610
C	-0.85980	-2.89334	1.14620
H	1.21309	-2.52141	1.54735
C	-1.93052	-1.45470	-0.45671
H	-0.68607	-0.01338	-1.41122
C	-2.01528	-2.51229	0.45617
H	-0.91821	-3.70905	1.86155
H	-2.94935	-3.02908	0.63787
O	-2.98262	-0.97905	-1.18851
C	-4.25710	-1.58193	-0.98884
H	-4.94448	-1.05422	-1.65113
H	-4.24116	-2.64641	-1.25191
H	-4.59160	-1.46969	0.04932
C	1.97673	0.88404	-0.50007
C	0.99718	1.98022	-0.82757
H	1.55668	2.89249	-1.06906
H	0.43813	1.73307	-1.74104
C	-0.00951	2.27919	0.30019
H	-0.37052	1.33225	0.70896
H	0.50770	2.79230	1.12234

C	-1.20407	3.11242	-0.17280
H	-0.85802	4.10970	-0.47859
H	-1.62542	2.64462	-1.07547
C	-2.32296	3.25206	0.86976
H	-1.91787	3.70533	1.78566
H	-3.07494	3.95501	0.48737
C	-3.01016	1.92416	1.21353
H	-2.33010	1.23234	1.72178
H	-3.87058	2.08492	1.87317
H	-3.36625	1.41939	0.30700
H	3.94003	1.94653	-0.65215

4e_conformer-093

C	0.77436	3.45683	-0.26592
C	0.05041	1.38502	0.33540
N	-0.31287	2.62575	-0.17050
H	-1.26795	2.91538	-0.32544
C	1.86870	2.72638	0.16259
C	0.65781	4.86737	-0.74350
H	1.64559	5.33549	-0.76512
H	0.23584	4.92339	-1.75508
H	0.01508	5.47098	-0.08933
C	-0.94664	0.32776	0.49762
C	-0.80017	-0.67859	1.47564
C	-2.08724	0.29797	-0.31870
C	-1.76462	-1.66971	1.60895
H	0.05591	-0.66709	2.13897
C	-3.06143	-0.69604	-0.16854
H	-2.22788	1.02728	-1.11027
C	-2.90448	-1.69539	0.79849
H	-1.64029	-2.43805	2.36710
H	-3.64193	-2.47792	0.92648
O	-4.12139	-0.61173	-1.02594
C	-5.13109	-1.61168	-0.93548
H	-5.61283	-1.60521	0.04967
H	-5.86732	-1.36355	-1.70109
H	-4.72257	-2.61038	-1.13154
C	1.43089	1.42600	0.54863
C	2.34435	0.31394	0.99134
H	3.35665	0.72366	1.10119
H	2.06818	-0.05687	1.98759
C	2.39387	-0.87311	0.01396
H	1.38182	-1.27644	-0.11752
H	2.70999	-0.50130	-0.96845
C	3.32585	-1.99112	0.48993

H	4.35051	-1.59988	0.57715
H	3.02546	-2.29155	1.50389
C	3.33231	-3.23195	-0.41412
H	3.93841	-4.01400	0.06224
H	2.31094	-3.63304	-0.48018
C	3.87037	-2.97264	-1.82501
H	4.88476	-2.55591	-1.78621
H	3.91143	-3.89922	-2.40882
H	3.24175	-2.26342	-2.37443
H	2.89014	3.08491	0.19266

4e_conformer-094

C	-0.54752	3.63068	-0.18027
C	0.40437	1.57924	0.05510
N	0.63417	2.93191	-0.15676
H	1.53723	3.32597	-0.37848
C	-1.55747	2.71391	0.04999
C	-0.59457	5.10304	-0.42810
H	-1.62716	5.45739	-0.36886
H	-0.00331	5.66116	0.30906
H	-0.20680	5.36502	-1.42125
C	1.51612	0.63570	0.15530
C	2.77137	1.05483	0.64844
C	1.37026	-0.70137	-0.23884
C	3.82556	0.15401	0.73664
H	2.90546	2.07639	0.99033
C	2.43244	-1.60696	-0.12968
H	0.44072	-1.05828	-0.66469
C	3.67475	-1.18372	0.35655
H	4.78540	0.48763	1.12128
H	4.51044	-1.86718	0.44126
O	2.15829	-2.88015	-0.54180
C	3.20609	-3.84176	-0.47429
H	2.78102	-4.77415	-0.84815
H	4.05670	-3.55051	-1.10229
H	3.55056	-3.98549	0.55691
C	-0.97387	1.42075	0.20036
C	-1.72889	0.15499	0.48975
H	-1.07186	-0.57166	0.98389
H	-2.53463	0.37596	1.20023
C	-2.33468	-0.49238	-0.77119
H	-3.06370	0.20187	-1.21181
H	-1.54266	-0.61383	-1.52191
C	-3.00019	-1.85039	-0.51633
H	-3.30644	-2.28202	-1.47967

H	-2.25701	-2.54312	-0.09371
C	-4.22130	-1.79629	0.40904
H	-3.92424	-1.42420	1.39779
H	-4.94163	-1.06752	0.01066
C	-4.90181	-3.15846	0.56685
H	-4.20862	-3.89730	0.98770
H	-5.77129	-3.09926	1.23136
H	-5.24506	-3.54170	-0.40201
H	-2.61263	2.94794	0.11806

4e_conformer-095

C	0.54760	3.63064	-0.18027
C	-0.40435	1.57923	0.05511
N	-0.63411	2.93190	-0.15676
H	-1.53716	3.32599	-0.37846
C	1.55753	2.71385	0.04999
C	0.59468	5.10300	-0.42810
H	0.20693	5.36499	-1.42126
H	0.00343	5.66114	0.30905
H	1.62728	5.45733	-0.36885
C	-1.51612	0.63573	0.15530
C	-2.77137	1.05489	0.64843
C	-1.37030	-0.70135	-0.23883
C	-3.82559	0.15410	0.73663
H	-2.90543	2.07646	0.99031
C	-2.43250	-1.60692	-0.12967
H	-0.44077	-1.05829	-0.66466
C	-3.67481	-1.18364	0.35655
H	-4.78542	0.48775	1.12125
H	-4.51052	-1.86707	0.44126
O	-2.15840	-2.88012	-0.54178
C	-3.20623	-3.84169	-0.47428
H	-3.55071	-3.98541	0.55691
H	-4.05682	-3.55041	-1.10230
H	-2.78118	-4.77409	-0.84814
C	0.97389	1.42070	0.20036
C	1.72888	0.15492	0.48975
H	2.53461	0.37588	1.20026
H	1.07183	-0.57172	0.98386
C	2.33470	-0.49242	-0.77119
H	1.54270	-0.61387	-1.52193
H	3.06372	0.20184	-1.21179
C	3.00021	-1.85043	-0.51633
H	2.25704	-2.54317	-0.09373
H	3.30647	-2.28205	-1.47968

C	4.22132	-1.79634	0.40904
H	4.94164	-1.06755	0.01067
H	3.92425	-1.42426	1.39779
C	4.90184	-3.15850	0.56685
H	5.77132	-3.09930	1.23136
H	4.20866	-3.89735	0.98768
H	5.24510	-3.54173	-0.40203
H	2.61269	2.94786	0.11806

4e_conformer-096

C	2.64806	2.57415	0.28472
C	1.53146	0.59836	0.11346
N	2.76688	1.22854	0.04759
H	3.64707	0.74122	-0.03981
C	1.30146	2.82292	0.48345
C	3.83182	3.48469	0.30981
H	3.50693	4.51204	0.49487
H	4.54385	3.21069	1.09926
H	4.37953	3.47027	-0.64116
C	1.41672	-0.83815	-0.13146
C	2.37090	-1.51662	-0.91069
C	0.34669	-1.57489	0.41556
C	2.25383	-2.89015	-1.12966
H	3.18489	-0.96980	-1.37604
C	0.22718	-2.94350	0.17133
H	-0.37347	-1.06961	1.04172
C	1.18953	-3.61197	-0.60043
H	2.99668	-3.40097	-1.73597
H	1.08034	-4.67754	-0.77385
O	-0.78943	-3.71740	0.65421
C	-1.82011	-3.07581	1.39813
H	-2.53938	-3.85670	1.64836
H	-1.43039	-2.63008	2.32131
H	-2.31539	-2.29811	0.80360
C	0.58718	1.59380	0.38468
C	-0.91112	1.47189	0.47538
H	-1.20816	0.78366	1.27917
H	-1.31182	2.44907	0.76745
C	-1.57650	1.01882	-0.83793
H	-1.40083	1.78615	-1.60444
H	-1.07982	0.10845	-1.19161
C	-3.07992	0.74674	-0.70856
H	-3.45092	0.34279	-1.66104
H	-3.23993	-0.04384	0.04076
C	-3.92109	1.97142	-0.33126

H	-3.60595	2.35292	0.64835
H	-3.72415	2.77759	-1.05236
C	-5.42112	1.66820	-0.29528
H	-5.64534	0.88233	0.43663
H	-6.00475	2.55497	-0.02309
H	-5.77587	1.31986	-1.27313
H	0.86423	3.79465	0.67665

4e_conformer-097

C	-1.69349	-2.67070	-0.39216
C	-0.30209	-1.13560	0.54395
N	-0.39303	-2.23729	-0.29514
H	0.40620	-2.73118	-0.66658
C	-2.45754	-1.80918	0.37307
C	-2.07072	-3.87520	-1.19117
H	-3.15449	-4.01665	-1.16185
H	-1.77162	-3.77819	-2.24268
H	-1.60285	-4.78965	-0.80343
C	0.98281	-0.46791	0.76362
C	1.34082	0.03730	2.02288
C	1.88754	-0.33445	-0.30908
C	2.56818	0.67994	2.19564
H	0.67431	-0.09878	2.86684
C	3.12027	0.29522	-0.12090
H	1.59770	-0.69892	-1.28691
C	3.46148	0.81244	1.13776
H	2.83804	1.06885	3.17355
H	4.42080	1.30460	1.26104
O	4.05098	0.46813	-1.10466
C	3.75247	-0.03547	-2.40265
H	2.86515	0.45112	-2.82538
H	4.62164	0.19476	-3.02006
H	3.59467	-1.12058	-2.38327
C	-1.59481	-0.83991	0.97063
C	-2.06276	0.35598	1.75392
H	-2.84895	0.04410	2.45387
H	-1.25001	0.76492	2.36199
C	-2.61669	1.48166	0.85532
H	-3.43800	1.07756	0.25028
H	-3.05167	2.26424	1.49342
C	-1.55077	2.10524	-0.05124
H	-0.72486	2.46757	0.57673
H	-1.12174	1.32529	-0.69396
C	-2.06304	3.25799	-0.92566
H	-2.49228	4.04000	-0.28307

H	-1.20812	3.71882	-1.43841
C	-3.09877	2.83111	-1.97121
H	-2.69713	2.04036	-2.61743
H	-4.01145	2.44456	-1.50458
H	-3.38605	3.67311	-2.61128
H	-3.53324	-1.86064	0.48817

4e_conformer-098

C	-0.83843	2.92964	-0.08793
C	0.13373	0.88371	0.11312
N	0.29239	2.18991	-0.32869
H	1.08777	2.51633	-0.85850
C	-1.73501	2.09009	0.54769
C	-0.95439	4.36073	-0.49978
H	-0.16446	4.97963	-0.05547
H	-0.88772	4.48113	-1.58899
H	-1.91844	4.76353	-0.17781
C	1.23116	-0.07713	0.01177
C	0.99235	-1.45978	-0.13406
C	2.56213	0.36612	0.05127
C	2.05858	-2.34728	-0.22433
H	-0.02288	-1.82842	-0.20693
C	3.62966	-0.53222	-0.05719
H	2.79647	1.41578	0.19833
C	3.38423	-1.90405	-0.19099
H	1.86141	-3.40960	-0.33930
H	4.19535	-2.61716	-0.26991
O	4.87458	0.02735	-0.00623
C	5.99908	-0.84232	-0.08922
H	6.01433	-1.38616	-1.04140
H	6.87878	-0.20075	-0.02365
H	6.00928	-1.56192	0.73829
C	-1.14046	0.79862	0.67759
C	-1.76936	-0.39032	1.35005
H	-0.98566	-1.04289	1.75206
H	-2.35455	-0.04336	2.21271
C	-2.69903	-1.22980	0.44733
H	-2.13871	-1.58134	-0.43045
H	-2.99756	-2.12594	1.00585
C	-3.94342	-0.47602	-0.03183
H	-4.52219	-0.14262	0.84292
H	-3.62626	0.43389	-0.55541
C	-4.85329	-1.29388	-0.95961
H	-5.64609	-0.63784	-1.34310
H	-4.27438	-1.61824	-1.83617

C	-5.49626	-2.51443	-0.29294
H	-6.06898	-2.21807	0.59495
H	-6.18265	-3.02370	-0.97901
H	-4.74587	-3.24600	0.02616
H	-2.71794	2.37540	0.89995

4e_conformer-099

C	1.69476	-2.67031	-0.39252
C	0.30296	-1.13573	0.54385
N	0.39416	-2.23736	-0.29528
H	-0.40496	-2.73150	-0.66663
C	2.45862	-1.80854	0.37261
C	2.07228	-3.87465	-1.19163
H	1.60471	-4.78927	-0.80390
H	1.77307	-3.77767	-2.24310
H	3.15610	-4.01578	-1.16239
C	-0.98214	-0.46846	0.76369
C	-1.34018	0.03655	2.02302
C	-1.88701	-0.33521	-0.30891
C	-2.56771	0.67880	2.19594
H	-0.67354	-0.09938	2.86691
C	-3.11991	0.29407	-0.12057
H	-1.59714	-0.69953	-1.28679
C	-3.46116	0.81110	1.13816
H	-2.83760	1.06757	3.17390
H	-4.42061	1.30296	1.26155
O	-4.05077	0.46678	-1.10423
C	-3.75221	-0.03662	-2.40229
H	-3.59407	-1.12168	-2.38303
H	-4.62149	0.19340	-3.01962
H	-2.86506	0.45028	-2.82505
C	1.59564	-0.83959	0.97033
C	2.06331	0.35646	1.75357
H	1.25070	0.76472	2.36229
H	2.85016	0.04493	2.45291
C	2.61590	1.48272	0.85487
H	3.05079	2.26542	1.49287
H	3.43708	1.07930	0.24919
C	1.54897	2.10584	-0.05084
H	1.12008	1.32578	-0.69352
H	0.72321	2.46741	0.57775
C	2.05992	3.25919	-0.92524
H	1.20436	3.71959	-1.43732
H	2.48902	4.04131	-0.28268
C	3.09529	2.83331	-1.97156

H	2.69377	2.04249	-2.61776
H	3.38161	3.67570	-2.61155
H	4.00851	2.44724	-1.50561
H	3.53435	-1.85962	0.48755

4e_conformer-100

C	0.29926	3.44513	-0.03125
C	-0.20582	1.25177	0.30409
N	-0.71041	2.52028	0.05186
H	-1.69424	2.74865	0.05533
C	1.48433	2.75295	0.14485
C	0.03099	4.89722	-0.25616
H	-0.51751	5.07224	-1.19053
H	-0.56284	5.33592	0.55638
H	0.97522	5.44541	-0.31318
C	-1.10383	0.10261	0.40936
C	-0.76551	-1.02784	1.18301
C	-2.33978	0.10541	-0.25458
C	-1.64066	-2.10339	1.26967
H	0.16949	-1.04929	1.72899
C	-3.22232	-0.97615	-0.15027
H	-2.63071	0.93440	-0.89202
C	-2.87579	-2.09672	0.61312
H	-1.36843	-2.96690	1.87037
H	-3.54021	-2.94718	0.70127
O	-4.39176	-0.84689	-0.84417
C	-5.31928	-1.92602	-0.79144
H	-4.88412	-2.84684	-1.19818
H	-5.66039	-2.10989	0.23446
H	-6.16703	-1.62214	-1.40688
C	1.18507	1.37629	0.36153
C	2.22029	0.29350	0.51343
H	3.20259	0.77386	0.58553
H	2.09100	-0.25468	1.45664
C	2.22833	-0.71642	-0.64937
H	1.21760	-1.12291	-0.76988
H	2.46047	-0.18021	-1.57998
C	3.21361	-1.87604	-0.45871
H	2.95983	-2.41725	0.46521
H	3.07811	-2.59446	-1.27957
C	4.68808	-1.45928	-0.40899
H	4.92659	-0.87583	-1.30979
H	4.85837	-0.78949	0.44359
C	5.63649	-2.65654	-0.30603
H	5.43416	-3.24115	0.59998

H	5.51783	-3.32773	-1.16561
H	6.68461	-2.33815	-0.27130
H	2.47575	3.18777	0.11616

4e_conformer-101

C	-0.29927	3.44513	-0.03125
C	0.20582	1.25177	0.30409
N	0.71040	2.52028	0.05186
H	1.69423	2.74866	0.05533
C	-1.48434	2.75294	0.14485
C	-0.03101	4.89722	-0.25617
H	0.56283	5.33592	0.55637
H	0.51749	5.07223	-1.19054
H	-0.97523	5.44540	-0.31319
C	1.10383	0.10261	0.40937
C	0.76552	-1.02783	1.18303
C	2.33978	0.10542	-0.25459
C	1.64067	-2.10338	1.26968
H	-0.16947	-1.04928	1.72901
C	3.22233	-0.97614	-0.15028
H	2.63071	0.93440	-0.89203
C	2.87580	-2.09672	0.61312
H	1.36846	-2.96689	1.87039
H	3.54022	-2.94717	0.70127
O	4.39176	-0.84688	-0.84418
C	5.31928	-1.92601	-0.79146
H	4.88412	-2.84683	-1.19818
H	6.16703	-1.62214	-1.40690
H	5.66040	-2.10988	0.23445
C	-1.18508	1.37628	0.36154
C	-2.22028	0.29349	0.51343
H	-2.09099	-0.25470	1.45664
H	-3.20258	0.77385	0.58555
C	-2.22833	-0.71642	-0.64938
H	-2.46048	-0.18020	-1.57998
H	-1.21760	-1.12291	-0.76989
C	-3.21361	-1.87604	-0.45872
H	-3.07812	-2.59446	-1.27959
H	-2.95983	-2.41726	0.46520
C	-4.68808	-1.45928	-0.40899
H	-4.85837	-0.78950	0.44360
H	-4.92660	-0.87583	-1.30978
C	-5.63649	-2.65654	-0.30604
H	-5.51783	-3.32773	-1.16562
H	-5.43416	-3.24116	0.59997

H	-6.68461	-2.33816	-0.27130
H	-2.47576	3.18775	0.11617

4e_conformer-102

C	2.76836	-1.70772	-0.05050
C	0.60503	-1.08010	0.26191
N	1.45639	-2.01615	-0.31140
H	1.15216	-2.74323	-0.94288
C	2.76146	-0.56902	0.73393
C	3.89787	-2.52539	-0.58604
H	3.83523	-3.57199	-0.26184
H	3.91883	-2.52565	-1.68368
H	4.85033	-2.12027	-0.23393
C	-0.84488	-1.21766	0.12087
C	-1.43755	-2.49085	0.05694
C	-1.67009	-0.08006	0.02061
C	-2.81861	-2.61625	-0.10230
H	-0.82324	-3.37988	0.16072
C	-3.05243	-0.21741	-0.12198
H	-1.21547	0.89886	0.02185
C	-3.63366	-1.49236	-0.18751
H	-3.26628	-3.60512	-0.14790
H	-4.70908	-1.57608	-0.30580
O	-3.91573	0.83557	-0.22535
C	-3.37389	2.15046	-0.15803
H	-2.67295	2.33732	-0.98080
H	-4.22310	2.82951	-0.24392
H	-2.86333	2.32268	0.79739
C	1.40797	-0.15983	0.93415
C	0.98662	0.99871	1.79697
H	-0.10214	1.00899	1.91707
H	1.39803	0.84386	2.80465
C	1.46229	2.38720	1.31858
H	1.31216	3.09083	2.14806
H	2.54508	2.35442	1.14374
C	0.74979	2.96260	0.08387
H	-0.33326	2.96782	0.27490
H	1.03808	4.01951	-0.01021
C	1.02097	2.27566	-1.26504
H	0.66791	1.23998	-1.24011
H	0.42348	2.78931	-2.03087
C	2.49390	2.29048	-1.68294
H	3.10284	1.67814	-1.01041
H	2.61751	1.88991	-2.69584
H	2.89847	3.31079	-1.67477

H	3.64086	-0.08199	1.13621

4e_conformer-103

C	-2.76842	-1.70765	-0.05046
C	-0.60508	-1.08008	0.26192
N	-1.45646	-2.01612	-0.31137
H	-1.15225	-2.74321	-0.94285
C	-2.76148	-0.56895	0.73396
C	-3.89796	-2.52530	-0.58597
H	-4.85041	-2.12015	-0.23386
H	-3.91893	-2.52558	-1.68362
H	-3.83534	-3.57190	-0.26176
C	0.84484	-1.21768	0.12086
C	1.43746	-2.49088	0.05689
C	1.67007	-0.08010	0.02064
C	2.81852	-2.61631	-0.10237
H	0.82314	-3.37990	0.16064
C	3.05241	-0.21748	-0.12197
H	1.21548	0.89884	0.02191
C	3.63360	-1.49244	-0.18755
H	3.26617	-3.60520	-0.14800
H	4.70902	-1.57618	-0.30585
O	3.91573	0.83548	-0.22531
C	3.37393	2.15038	-0.15791
H	2.86340	2.32257	0.79753
H	4.22315	2.82942	-0.24380
H	2.67297	2.33730	-0.98065
C	-1.40798	-0.15979	0.93416
C	-0.98660	0.99876	1.79696
H	-1.39801	0.84395	2.80464
H	0.10217	1.00900	1.91705
C	-1.46222	2.38725	1.31852
H	-2.54500	2.35450	1.14368
H	-1.31208	3.09090	2.14800
C	-0.74968	2.96261	0.08382
H	0.33336	2.96782	0.27487
H	-1.03795	4.01953	-0.01029
C	-1.02085	2.27565	-1.26508
H	-0.42333	2.78927	-2.03091
H	-0.66781	1.23996	-1.24012
C	-2.49377	2.29050	-1.68302
H	-2.61736	1.88991	-2.69591
H	-3.10274	1.67818	-1.01049
H	-2.89832	3.31081	-1.67488
H	-3.64087	-0.08189	1.13625

4e_conformer-104

C	2.08041	2.63789	-0.19510
C	0.23228	1.35045	0.11899
N	0.73862	2.50900	-0.45430
H	0.21542	3.11133	-1.07337
C	2.44085	1.55435	0.58521
C	2.88653	3.77721	-0.72730
H	2.48850	4.74710	-0.40270
H	3.91775	3.70104	-0.37219
H	2.90983	3.78627	-1.82488
C	-1.19101	1.03095	0.02152
C	-2.15052	2.06207	-0.08750
C	-1.64217	-0.29582	0.02912
C	-3.50202	1.75484	-0.18401
H	-1.83576	3.10064	-0.06273
C	-3.00742	-0.59614	-0.05081
H	-0.94325	-1.12160	0.06834
C	-3.95174	0.43048	-0.16321
H	-4.22996	2.55754	-0.26348
H	-5.01125	0.21814	-0.23355
O	-3.31404	-1.92710	-0.02800
C	-4.68558	-2.29586	-0.12813
H	-5.26935	-1.89406	0.70880
H	-4.70561	-3.38592	-0.09435
H	-5.12380	-1.95159	-1.07271
C	1.29085	0.73357	0.78836
C	1.24809	-0.52291	1.61325
H	1.91374	-0.40395	2.47909
H	0.23852	-0.66497	2.01636
C	1.66591	-1.80152	0.85643
H	1.49175	-2.66796	1.51031
H	1.01251	-1.93255	-0.01503
C	3.12955	-1.80144	0.40454
H	3.29902	-0.95785	-0.27730
H	3.76838	-1.62108	1.28105
C	3.57300	-3.10236	-0.27957
H	3.41125	-3.94660	0.40575
H	4.65562	-3.05576	-0.45764
C	2.86145	-3.37998	-1.60809
H	2.99401	-2.54226	-2.30434
H	3.25892	-4.28108	-2.08882
H	1.78483	-3.52759	-1.46952
H	3.43016	1.37191	0.98472

4e_conformer-105

C	1.41768	2.52363	-0.48707
C	0.08637	0.69817	-0.75865
N	0.14385	2.02852	-0.36487
H	-0.66845	2.58885	-0.14922
C	2.20436	1.48019	-0.94214
C	1.75312	3.94396	-0.16878
H	2.82517	4.11169	-0.30263
H	1.21972	4.64865	-0.82016
H	1.49809	4.20201	0.86703
C	-1.17152	-0.04367	-0.68660
C	-1.45437	-1.09339	-1.57563
C	-2.13502	0.30222	0.28421
C	-2.66556	-1.78068	-1.48681
H	-0.73966	-1.35514	-2.34668
C	-3.35016	-0.38262	0.35378
H	-1.90714	1.08256	0.99960
C	-3.61850	-1.43471	-0.53436
H	-2.87414	-2.59057	-2.18037
H	-4.56562	-1.95890	-0.45870
O	-4.33194	-0.11024	1.26289
C	-4.11230	0.94993	2.18751
H	-5.01384	1.00517	2.79896
H	-3.24742	0.74661	2.83059
H	-3.96164	1.90556	1.67088
C	1.38311	0.32842	-1.12227
C	1.89863	-1.03228	-1.51288
H	1.41989	-1.39122	-2.43290
H	2.96231	-0.93146	-1.75737
C	1.72677	-2.10965	-0.42103
H	0.65862	-2.32509	-0.30102
H	2.19543	-3.03879	-0.77461
C	2.31149	-1.72871	0.94455
H	1.79163	-0.83789	1.32172
H	2.09606	-2.53697	1.65776
C	3.81996	-1.46116	0.93775
H	4.34201	-2.33155	0.51477
H	4.04017	-0.61634	0.27355
C	4.36974	-1.15795	2.33378
H	4.19333	-1.99664	3.01866
H	5.44856	-0.96583	2.30987
H	3.88186	-0.27346	2.76199
H	3.27081	1.53081	-1.12301

4e_conformer-106

C	-3.23238	-1.30439	-0.29469
C	-1.04515	-0.94178	0.21191
N	-1.94558	-1.74821	-0.47249
H	-1.67380	-2.47717	-1.11633
C	-3.16313	-0.20909	0.54614
C	-4.39862	-1.96753	-0.95169
H	-4.47462	-3.02736	-0.67702
H	-5.32625	-1.47422	-0.64926
H	-4.33473	-1.91928	-2.04663
C	0.38834	-1.22974	0.15809
C	0.84833	-2.56013	0.04560
C	1.33728	-0.20001	0.18318
C	2.21013	-2.82509	-0.03774
H	0.13778	-3.38102	0.05146
C	2.70747	-0.47548	0.10993
H	1.03124	0.83563	0.22796
C	3.15707	-1.79574	-0.00395
H	2.55233	-3.85302	-0.12011
H	4.21249	-2.02974	-0.06808
O	3.52278	0.62048	0.13667
C	4.92681	0.40759	0.03293
H	5.30218	-0.19504	0.86874
H	5.38288	1.39787	0.06581
H	5.18714	-0.08354	-0.91246
C	-1.79375	0.03279	0.87078
C	-1.32142	1.09738	1.82343
H	-1.87419	0.97915	2.76595
H	-0.26557	0.94362	2.07297
C	-1.52698	2.55580	1.35319
H	-2.55989	2.67676	0.99851
H	-1.42798	3.20263	2.23479
C	-0.55160	3.06015	0.27938
H	-0.62308	4.15602	0.22783
H	0.47794	2.85037	0.60691
C	-0.76468	2.50085	-1.13315
H	-1.76126	2.80043	-1.48652
H	-0.76980	1.40709	-1.10882
C	0.30541	2.98433	-2.11496
H	1.30100	2.64269	-1.80410
H	0.12398	2.60475	-3.12700
H	0.33302	4.08004	-2.16702
H	-4.01276	0.35751	0.90619

4e_conformer-107

C	-0.74126	3.09479	0.09020
C	0.13601	0.99695	0.07670
N	0.40351	2.35029	0.23299
H	1.29479	2.72156	0.52906
C	-1.76210	2.20571	-0.19340
C	-0.74679	4.58025	0.24731
H	-1.75125	4.97024	0.06215
H	-0.44899	4.88715	1.25847
H	-0.06017	5.06870	-0.45587
C	1.20696	0.00510	0.15540
C	0.96295	-1.30666	0.61421
C	2.51556	0.34617	-0.21832
C	2.00122	-2.22892	0.67616
H	-0.02809	-1.58704	0.94910
C	3.55853	-0.58355	-0.13659
H	2.74468	1.33276	-0.60891
C	3.30610	-1.88694	0.30775
H	1.80183	-3.23545	1.03358
H	4.09689	-2.62393	0.37187
O	4.78440	-0.12580	-0.52793
C	5.87967	-1.03466	-0.48309
H	5.71035	-1.89681	-1.13938
H	6.74726	-0.47506	-0.83509
H	6.06359	-1.38864	0.53846
C	-1.22527	0.88371	-0.20525
C	-2.00806	-0.36378	-0.50049
H	-2.71118	-0.15542	-1.31647
H	-1.34059	-1.15491	-0.86358
C	-2.79552	-0.88469	0.71669
H	-2.10259	-1.02196	1.55727
H	-3.50215	-0.10923	1.03862
C	-3.53472	-2.20531	0.46076
H	-4.03553	-2.51259	1.38969
H	-2.79535	-2.98719	0.23611
C	-4.57369	-2.16608	-0.67112
H	-4.07248	-1.97825	-1.62954
H	-5.02763	-3.16193	-0.75904
C	-5.67827	-1.12575	-0.46261
H	-5.27939	-0.10511	-0.47123
H	-6.43576	-1.19047	-1.25183
H	-6.18293	-1.27562	0.50012
H	-2.79292	2.47554	-0.38695

4e_conformer-108

C	0.74128	3.09479	0.09020

C	-0.13601	0.99694	0.07671
N	-0.40350	2.35029	0.23299
H	-1.29479	2.72156	0.52906
C	1.76211	2.20570	-0.19339
C	0.74681	4.58025	0.24731
H	0.06017	5.06869	-0.45586
H	0.44904	4.88715	1.25847
H	1.75127	4.97023	0.06212
C	-1.20696	0.00511	0.15541
C	-0.96296	-1.30665	0.61425
C	-2.51555	0.34617	-0.21833
C	-2.00123	-2.22890	0.67620
H	0.02807	-1.58703	0.94915
C	-3.55853	-0.58354	-0.13660
H	-2.74466	1.33276	-0.60894
C	-3.30611	-1.88692	0.30777
H	-1.80185	-3.23543	1.03364
H	-4.09691	-2.62391	0.37189
O	-4.78439	-0.12580	-0.52797
C	-5.87966	-1.03465	-0.48313
H	-5.71034	-1.89682	-1.13940
H	-6.06360	-1.38861	0.53842
H	-6.74724	-0.47506	-0.83515
C	1.22527	0.88370	-0.20524
C	2.00805	-0.36379	-0.50047
H	1.34058	-1.15493	-0.86354
H	2.71117	-0.15544	-1.31647
C	2.79554	-0.88469	0.71671
H	3.50217	-0.10922	1.03862
H	2.10262	-1.02194	1.55730
C	3.53473	-2.20531	0.46078
H	4.03557	-2.51257	1.38971
H	2.79536	-2.98720	0.23618
C	4.57366	-2.16610	-0.67113
H	5.02762	-3.16195	-0.75903
H	4.07242	-1.97831	-1.62954
C	5.67824	-1.12575	-0.46269
H	6.43570	-1.19049	-1.25192
H	5.27935	-0.10512	-0.47133
H	6.18293	-1.27558	0.50003
H	2.79293	2.47552	-0.38694

4e_conformer-109

C	2.73768	-1.83858	-0.32631
C	0.95647	-0.76689	0.59374

N	1.37372	-1.85460	-0.15934
H	0.77046	-2.61917	-0.42800
C	3.20115	-0.69782	0.30293
C	3.46126	-2.92848	-1.04744
H	3.36591	-3.89522	-0.53567
H	3.08287	-3.06288	-2.06874
H	4.52606	-2.68885	-1.11210
C	-0.46854	-0.52165	0.82255
C	-0.94342	-0.01459	2.04882
C	-1.39396	-0.79148	-0.19443
C	-2.30068	0.23084	2.22157
H	-0.24987	0.15866	2.86379
C	-2.76115	-0.55236	-0.00908
H	-1.06255	-1.15669	-1.16110
C	-3.22476	-0.03120	1.20437
H	-2.65895	0.62139	3.17004
H	-4.27690	0.16713	1.36685
O	-3.55791	-0.84476	-1.07902
C	-4.95411	-0.59283	-0.95718
H	-5.39925	-1.19052	-0.15265
H	-5.15296	0.46946	-0.77098
H	-5.39494	-0.88327	-1.91166
C	2.09122	-0.01368	0.88513
C	2.15651	1.35180	1.51009
H	1.29406	1.51704	2.16300
H	3.04550	1.41081	2.15218
C	2.21365	2.51373	0.49297
H	2.34160	3.44265	1.06429
H	3.11219	2.40936	-0.13074
C	0.96967	2.63755	-0.40831
H	0.07975	2.33664	0.16186
H	0.81834	3.69425	-0.67021
C	1.03014	1.83057	-1.71210
H	1.87719	2.19205	-2.31286
H	1.23596	0.77941	-1.48939
C	-0.26229	1.93794	-2.52506
H	-0.49151	2.98256	-2.77195
H	-0.19315	1.37958	-3.46610
H	-1.11025	1.53418	-1.95913
H	4.23464	-0.37541	0.33332

4e_conformer-110

C	1.08616	2.68540	0.06187
C	-0.07170	0.72893	0.01373
N	-0.13934	2.09568	0.24589

H	-0.95267	2.56799	0.61347
C	1.95648	1.68233	-0.32444
C	1.30584	4.14609	0.28401
H	0.65315	4.75910	-0.35059
H	1.11304	4.43653	1.32508
H	2.34195	4.40614	0.05124
C	-1.27229	-0.10145	0.09843
C	-1.20694	-1.46184	0.46610
C	-2.53200	0.45091	-0.18024
C	-2.36772	-2.22371	0.53482
H	-0.25479	-1.90678	0.72582
C	-3.69755	-0.31852	-0.09151
H	-2.63003	1.48318	-0.50141
C	-3.62283	-1.67085	0.26284
H	-2.30423	-3.26974	0.82183
H	-4.51128	-2.28619	0.33072
O	-4.85818	0.33901	-0.38504
C	-6.07436	-0.39942	-0.32745
H	-6.25898	-0.78701	0.68170
H	-6.86397	0.30340	-0.59639
H	-6.06956	-1.23329	-1.03972
C	1.24441	0.44490	-0.35617
C	1.81523	-0.88515	-0.76438
H	1.01536	-1.52550	-1.15343
H	2.51503	-0.73465	-1.59608
C	2.54224	-1.64473	0.36697
H	1.83619	-1.82217	1.18866
H	2.82952	-2.63489	-0.00961
C	3.77398	-0.92939	0.94067
H	3.45793	0.02494	1.37988
H	4.17198	-1.53492	1.76713
C	4.90693	-0.66455	-0.06284
H	4.55727	0.01440	-0.85123
H	5.71164	-0.12965	0.45871
C	5.47970	-1.93145	-0.70569
H	4.73465	-2.44411	-1.32432
H	6.33583	-1.69615	-1.34806
H	5.81920	-2.64095	0.05945
H	2.99998	1.82368	-0.57408

4e_conformer-111

C	3.67531	-0.91231	-0.13543
C	1.40019	-0.85930	-0.13015
N	2.53265	-1.58133	0.22237
H	2.51305	-2.52553	0.58011

C	3.27148	0.28111	-0.70795
C	5.03640	-1.48345	0.09378
H	5.79841	-0.77405	-0.23998
H	5.21689	-1.69787	1.15490
H	5.18669	-2.42094	-0.45742
C	0.06728	-1.37353	0.18041
C	-0.12702	-2.26513	1.25842
C	-1.04940	-1.00550	-0.58258
C	-1.39548	-2.75637	1.54287
H	0.70925	-2.54445	1.89159
C	-2.32597	-1.49176	-0.27972
H	-0.94830	-0.34644	-1.43592
C	-2.51002	-2.37916	0.78731
H	-1.53090	-3.43784	2.37820
H	-3.48911	-2.76971	1.03514
O	-3.32795	-1.04905	-1.09609
C	-4.64741	-1.52214	-0.84692
H	-5.28036	-1.05543	-1.60282
H	-4.70592	-2.61293	-0.94457
H	-4.99501	-1.22950	0.15122
C	1.84719	0.32772	-0.71728
C	1.03415	1.50277	-1.19166
H	1.71566	2.22951	-1.65210
H	0.33755	1.20905	-1.98853
C	0.24499	2.20394	-0.07254
H	-0.41202	1.47568	0.41946
H	0.95459	2.54690	0.69041
C	-0.59629	3.37467	-0.58903
H	0.06521	4.12820	-1.04204
H	-1.24515	3.01049	-1.39831
C	-1.46813	4.04387	0.48283
H	-2.11242	4.79044	-0.00029
H	-2.14231	3.29212	0.91735
C	-0.67070	4.72141	1.60191
H	-1.33553	5.23480	2.30584
H	-0.07942	3.99790	2.17366
H	0.02278	5.46655	1.19200
H	3.93221	1.05361	-1.08140

4e_conformer-112

C	-1.25165	-3.01822	-0.36489
C	-0.17500	-1.22373	0.52359
N	-0.03869	-2.38964	-0.21655
H	0.85070	-2.78623	-0.48532
C	-2.19113	-2.22216	0.26377

C	-1.38581	-4.32523	-1.07590
H	-2.43733	-4.62248	-1.11288
H	-1.01457	-4.26929	-2.10719
H	-0.82861	-5.12661	-0.57307
C	0.98072	-0.35928	0.77310
C	1.15880	0.29098	2.00377
C	1.94435	-0.18102	-0.24024
C	2.26632	1.11706	2.20455
H	0.45008	0.12635	2.80730
C	3.05774	0.63407	-0.02251
H	1.79044	-0.66021	-1.19926
C	3.21748	1.29391	1.20525
H	2.39714	1.61738	3.16001
H	4.08548	1.92861	1.35160
O	4.03423	0.86165	-0.94909
C	3.91915	0.21593	-2.21296
H	4.79679	0.51943	-2.78505
H	3.91266	-0.87535	-2.10440
H	3.01164	0.53173	-2.74162
C	-1.52752	-1.09067	0.82998
C	-2.22282	0.07735	1.47380
H	-2.99642	-0.29507	2.15805
H	-1.52294	0.65667	2.08381
C	-2.88212	1.02853	0.45233
H	-3.58579	0.45742	-0.16977
H	-3.47815	1.76840	1.00164
C	-1.86743	1.73769	-0.44994
H	-1.19353	2.34358	0.17393
H	-1.23601	0.98137	-0.93201
C	-2.49751	2.62767	-1.52992
H	-1.70202	2.99804	-2.19043
H	-3.15801	2.01529	-2.16044
C	-3.28316	3.82194	-0.97849
H	-2.64862	4.44206	-0.33270
H	-3.65814	4.45764	-1.78878
H	-4.14592	3.50084	-0.38449
H	-3.25425	-2.42428	0.30776

4e_conformer-113

C	2.25556	-2.22488	-0.03178
C	0.57738	-0.84801	0.64880
N	0.90173	-2.00108	-0.05204
H	0.21991	-2.65333	-0.41269
C	2.81818	-1.17528	0.67341
C	2.87250	-3.42695	-0.66852

H	2.54523	-4.35977	-0.19069
H	2.61815	-3.50055	-1.73343
H	3.96140	-3.37406	-0.58564
C	-0.80605	-0.38208	0.71456
C	-1.28064	0.35266	1.82089
C	-1.69915	-0.66642	-0.32865
C	-2.60060	0.78474	1.85747
H	-0.61824	0.56292	2.65223
C	-3.03173	-0.23997	-0.27758
H	-1.36912	-1.19776	-1.21574
C	-3.49328	0.49641	0.81940
H	-2.95576	1.34909	2.71535
H	-4.51779	0.84255	0.87563
O	-3.79765	-0.58056	-1.35593
C	-5.15566	-0.15258	-1.36922
H	-5.71603	-0.57498	-0.52647
H	-5.22982	0.94120	-1.34064
H	-5.57624	-0.52091	-2.30583
C	1.77774	-0.30210	1.10563
C	2.00502	1.01846	1.79258
H	1.51140	1.04022	2.77330
H	3.07887	1.10887	2.00183
C	1.55798	2.26544	1.00151
H	0.47585	2.21971	0.82794
H	1.72861	3.13780	1.64667
C	2.27822	2.49678	-0.33719
H	2.14743	3.55193	-0.61812
H	3.36081	2.35477	-0.19896
C	1.79922	1.63210	-1.51196
H	2.00967	0.57787	-1.31089
H	0.70646	1.71669	-1.59501
C	2.44971	2.04111	-2.83619
H	3.54132	1.94031	-2.78560
H	2.09655	1.41853	-3.66651
H	2.22678	3.08693	-3.08298
H	3.87723	-1.03265	0.84886

4e_conformer-114

C	-2.25556	-2.22488	-0.03179
C	-0.57737	-0.84802	0.64880
N	-0.90172	-2.00108	-0.05204
H	-0.21990	-2.65334	-0.41270
C	-2.81817	-1.17528	0.67341
C	-2.87249	-3.42695	-0.66852
H	-2.61815	-3.50056	-1.73343

H	-2.54522	-4.35977	-0.19070
H	-3.96140	-3.37407	-0.58564
C	0.80605	-0.38209	0.71456
C	1.28064	0.35265	1.82089
C	1.69915	-0.66642	-0.32865
C	2.60061	0.78474	1.85747
H	0.61825	0.56291	2.65223
C	3.03173	-0.23996	-0.27758
H	1.36912	-1.19775	-1.21575
C	3.49328	0.49642	0.81940
H	2.95577	1.34909	2.71535
H	4.51779	0.84255	0.87563
O	3.79765	-0.58055	-1.35594
C	5.15566	-0.15257	-1.36922
H	5.22982	0.94121	-1.34064
H	5.71603	-0.57498	-0.52648
H	5.57624	-0.52090	-2.30583
C	-1.77773	-0.30210	1.10563
C	-2.00501	1.01845	1.79259
H	-3.07887	1.10886	2.00184
H	-1.51139	1.04021	2.77331
C	-1.55798	2.26544	1.00151
H	-1.72859	3.13780	1.64668
H	-0.47584	2.21970	0.82794
C	-2.27823	2.49679	-0.33717
H	-3.36081	2.35479	-0.19893
H	-2.14742	3.55193	-0.61811
C	-1.79924	1.63210	-1.51195
H	-0.70649	1.71666	-1.59500
H	-2.00972	0.57787	-1.31088
C	-2.44974	2.04113	-2.83617
H	-3.54135	1.94036	-2.78558
H	-2.22678	3.08694	-3.08296
H	-2.09660	1.41854	-3.66650
H	-3.87722	-1.03266	0.84886

4e_conformer-115

C	-1.00433	3.35806	-0.12116
C	-0.61130	1.17531	0.38953
N	-1.56464	2.11305	0.01487
H	-2.55714	1.92852	-0.01738
C	0.34624	3.21450	0.14312
C	-1.81568	4.56073	-0.47685
H	-2.57887	4.78045	0.28120
H	-1.16612	5.43620	-0.56002

H	-2.33610	4.43455	-1.43485
C	-0.97907	-0.22857	0.56915
C	-0.27717	-1.06319	1.45461
C	-2.06730	-0.76716	-0.15013
C	-0.65213	-2.39849	1.60749
H	0.54412	-0.66464	2.03741
C	-2.44318	-2.10095	0.02274
H	-2.58700	-0.14486	-0.86787
C	-1.72997	-2.92642	0.90459
H	-0.10142	-3.03356	2.29587
H	-2.03341	-3.96200	1.01862
O	-3.48163	-2.69544	-0.63524
C	-4.24247	-1.90253	-1.54045
H	-5.01927	-2.55960	-1.93359
H	-4.70788	-1.05022	-1.03072
H	-3.62317	-1.53471	-2.36754
C	0.60845	1.85229	0.47062
C	1.97769	1.28645	0.73928
H	2.69151	2.11893	0.78365
H	2.02422	0.80693	1.72624
C	2.45302	0.28183	-0.32433
H	1.72595	-0.53620	-0.39938
H	2.46148	0.78019	-1.30339
C	3.83769	-0.29567	-0.02376
H	4.56761	0.52335	0.05842
H	3.81893	-0.78738	0.96035
C	4.31743	-1.29764	-1.07841
H	3.58539	-2.11349	-1.15898
H	4.33636	-0.80599	-2.06118
C	5.69963	-1.87768	-0.76848
H	5.69900	-2.40197	0.19512
H	6.01835	-2.59076	-1.53730
H	6.45561	-1.08471	-0.71297
H	1.08206	4.00796	0.10086

4e_conformer-116

C	0.71378	2.67540	-0.01799
C	-0.24053	0.61734	0.14376
N	-0.39468	1.92012	-0.31080
H	-1.16260	2.23172	-0.88801
C	1.58796	1.85057	0.66602
C	0.83163	4.10599	-0.43107
H	0.81343	4.22082	-1.52278
H	0.01507	4.71744	-0.02632
H	1.77518	4.52168	-0.06743

C	-1.31817	-0.35921	-0.00685
C	-1.05459	-1.73145	-0.14880
C	-2.66024	0.07847	-0.01629
C	-2.10807	-2.63784	-0.28410
H	-0.03194	-2.08512	-0.18526
C	-3.70432	-0.83525	-0.17093
H	-2.87319	1.12951	0.13413
C	-3.42920	-2.20520	-0.30038
H	-1.89205	-3.69690	-0.39454
H	-4.25553	-2.89962	-0.41252
O	-5.02589	-0.49265	-0.19246
C	-5.36156	0.88600	-0.07491
H	-5.03290	1.29687	0.88740
H	-4.92079	1.47471	-0.88853
H	-6.44935	0.93551	-0.13777
C	1.00418	0.55193	0.77235
C	1.61338	-0.62651	1.48013
H	2.14045	-0.27144	2.37633
H	0.81915	-1.29469	1.83286
C	2.60918	-1.44378	0.63015
H	2.90651	-2.33388	1.20225
H	2.10384	-1.80993	-0.27430
C	3.86303	-0.66847	0.21833
H	3.57099	0.21475	-0.36425
H	4.36675	-0.28809	1.11975
C	4.85020	-1.50903	-0.59711
H	5.14515	-2.39233	-0.01324
H	4.34280	-1.89152	-1.49407
C	6.09941	-0.72674	-1.01027
H	5.83313	0.14397	-1.62211
H	6.64188	-0.35896	-0.13056
H	6.78909	-1.34763	-1.59338
H	2.54833	2.15018	1.06543

4e_conformer-117

C	1.66731	2.96171	-0.16222
C	0.01783	1.42013	0.11146
N	0.34123	2.67913	-0.37555
H	-0.28819	3.25811	-0.91255
C	2.20421	1.87302	0.50082
C	2.29169	4.23812	-0.62297
H	2.26390	4.33772	-1.71599
H	1.78431	5.11625	-0.20363
H	3.33899	4.27375	-0.31120
C	-1.35717	0.92776	0.04744

C	-2.44254	1.83180	0.06880
C	-1.63780	-0.44239	-0.04044
C	-3.74821	1.36155	0.00352
H	-2.25918	2.89697	0.16960
C	-2.95710	-0.90894	-0.08852
H	-0.83991	-1.17135	-0.10239
C	-4.02706	-0.00714	-0.07138
H	-4.57394	2.06742	0.02468
H	-5.05392	-0.34815	-0.11493
O	-3.09321	-2.26585	-0.16858
C	-4.41110	-2.79912	-0.24561
H	-4.99732	-2.54615	0.64604
H	-4.93655	-2.43803	-1.13797
H	-4.29272	-3.88171	-0.30690
C	1.18089	0.89330	0.67655
C	1.34139	-0.42216	1.38678
H	2.03562	-0.29067	2.22801
H	0.38205	-0.72417	1.82321
C	1.86984	-1.57424	0.50506
H	1.82683	-2.50027	1.09217
H	1.19836	-1.71743	-0.35319
C	3.29336	-1.35229	-0.01505
H	3.32615	-0.40244	-0.56234
H	3.97619	-1.23903	0.84064
C	3.80828	-2.47285	-0.92971
H	3.13056	-2.57484	-1.78935
H	4.78082	-2.17215	-1.34170
C	3.95961	-3.83063	-0.23606
H	4.62015	-3.75208	0.63669
H	2.99617	-4.21908	0.11178
H	4.38988	-4.57554	-0.91514
H	3.22838	1.78992	0.84117

4e_conformer-118

C	-1.66729	2.96172	-0.16224
C	-0.01781	1.42013	0.11145
N	-0.34120	2.67914	-0.37555
H	0.28822	3.25811	-0.91256
C	-2.20419	1.87303	0.50079
C	-2.29165	4.23813	-0.62299
H	-1.78434	5.11626	-0.20356
H	-2.26377	4.33778	-1.71599
H	-3.33899	4.27373	-0.31130
C	1.35719	0.92776	0.04744
C	2.44257	1.83178	0.06884

C	1.63780	-0.44239	-0.04047
C	3.74823	1.36152	0.00358
H	2.25922	2.89695	0.16967
C	2.95710	-0.90896	-0.08853
H	0.83991	-1.17135	-0.10245
C	4.02707	-0.00717	-0.07135
H	4.57397	2.06738	0.02477
H	5.05393	-0.34819	-0.11489
O	3.09320	-2.26586	-0.16862
C	4.41108	-2.79915	-0.24564
H	4.29269	-3.88174	-0.30696
H	4.93655	-2.43804	-1.13798
H	4.99729	-2.54621	0.64603
C	-1.18088	0.89331	0.67653
C	-1.34139	-0.42215	1.38677
H	-0.38205	-0.72417	1.82318
H	-2.03560	-0.29065	2.22800
C	-1.86986	-1.57423	0.50506
H	-1.19840	-1.71744	-0.35320
H	-1.82685	-2.50025	1.09217
C	-3.29338	-1.35227	-0.01503
H	-3.97621	-1.23901	0.84067
H	-3.32618	-0.40242	-0.56232
C	-3.80833	-2.47282	-0.92968
H	-4.78088	-2.17211	-1.34165
H	-3.13063	-2.57481	-1.78934
C	-3.95965	-3.83060	-0.23604
H	-4.62017	-3.75206	0.63672
H	-4.38994	-4.57551	-0.91512
H	-2.99621	-4.21906	0.11178
H	-3.22836	1.78993	0.84114

4e_conformer-119

C	-1.60112	3.37511	-0.24983
C	-0.14944	1.62682	-0.12751
N	-0.30160	3.00260	-0.01723
H	0.46556	3.64977	0.09557
C	-2.30901	2.21055	-0.48983
C	-2.03452	4.80449	-0.23150
H	-1.83239	5.28133	0.73613
H	-1.52281	5.39872	-0.99986
H	-3.10949	4.86950	-0.41996
C	1.15295	1.00433	0.10466
C	2.10918	1.62549	0.93798
C	1.49220	-0.21582	-0.49590

C	3.34868	1.03281	1.14666
H	1.86674	2.55301	1.44695
C	2.73657	-0.81391	-0.26836
H	0.80385	-0.71789	-1.16433
C	3.68141	-0.18955	0.55463
H	4.07289	1.51959	1.79402
H	4.65112	-0.63433	0.74004
O	2.93783	-2.00414	-0.90813
C	4.19275	-2.65223	-0.72875
H	4.36411	-2.90666	0.32414
H	4.14397	-3.56761	-1.31994
H	5.01970	-2.02846	-1.08911
C	-1.41224	1.10496	-0.42158
C	-1.82454	-0.33727	-0.55175
H	-2.87615	-0.36190	-0.85892
H	-1.27154	-0.83999	-1.35702
C	-1.65150	-1.14505	0.74824
H	-0.62157	-1.02547	1.10335
H	-2.29932	-0.70991	1.52182
C	-1.95587	-2.64026	0.59570
H	-1.28472	-3.06912	-0.16363
H	-1.71311	-3.14931	1.53923
C	-3.40778	-2.96154	0.22320
H	-4.07892	-2.49016	0.95542
H	-3.65182	-2.51356	-0.74856
C	-3.68335	-4.46633	0.16730
H	-4.72525	-4.67527	-0.10090
H	-3.04187	-4.95564	-0.57609
H	-3.48500	-4.93916	1.13711
H	-3.37170	2.15168	-0.68991

4e_conformer-120

C	-3.02014	-1.59097	-0.29909
C	-0.83306	-1.11472	0.10394
N	-1.72759	-2.03301	-0.43010
H	-1.44969	-2.84618	-0.96090
C	-2.96100	-0.37935	0.36424
C	-4.18145	-2.36080	-0.83780
H	-4.13926	-2.45676	-1.93070
H	-4.22593	-3.37623	-0.42397
H	-5.11532	-1.85245	-0.58324
C	0.60116	-1.41683	0.11478
C	1.04374	-2.74152	0.27393
C	1.56072	-0.40008	-0.05493
C	2.40908	-3.03380	0.26459

H	0.32247	-3.53691	0.43353
C	2.92412	-0.69998	-0.04391
H	1.22796	0.61256	-0.22595
C	3.35500	-2.02583	0.11348
H	2.73897	-4.06119	0.39119
H	4.41922	-2.23804	0.11230
O	3.91030	0.23187	-0.19780
C	3.52238	1.59383	-0.34683
H	2.92297	1.74217	-1.25321
H	2.95338	1.94459	0.52241
H	4.44942	2.16262	-0.42846
C	-1.59143	-0.06398	0.62175
C	-1.11493	1.12356	1.41691
H	-1.26365	0.92695	2.48928
H	-0.03671	1.25341	1.29469
C	-1.83824	2.44319	1.08443
H	-2.88632	2.36587	1.40162
H	-1.39728	3.23684	1.70070
C	-1.81419	2.85655	-0.39436
H	-2.40142	2.13437	-0.97464
H	-2.32819	3.82355	-0.48846
C	-0.42019	2.97149	-1.02713
H	0.03169	1.97396	-1.08967
H	-0.53737	3.30735	-2.06587
C	0.52867	3.92494	-0.29548
H	0.08729	4.92528	-0.20520
H	0.75673	3.57338	0.71710
H	1.47873	4.02677	-0.83222
H	-3.81839	0.22004	0.64167

4e_conformer-121

C	3.02024	-1.59081	-0.29908
C	0.83313	-1.11469	0.10394
N	1.72771	-2.03292	-0.43010
H	1.44986	-2.84610	-0.96091
C	2.96102	-0.37919	0.36426
C	4.18159	-2.36056	-0.83780
H	4.22613	-3.37599	-0.42397
H	4.13940	-2.45651	-1.93070
H	5.11543	-1.85216	-0.58323
C	-0.60109	-1.41685	0.11477
C	-1.04363	-2.74156	0.27392
C	-1.56068	-0.40014	-0.05494
C	-2.40896	-3.03388	0.26457
H	-0.32233	-3.53693	0.43353

C	-2.92406	-0.70007	-0.04392
H	-1.22795	0.61251	-0.22595
C	-3.35491	-2.02593	0.11346
H	-2.73882	-4.06128	0.39116
H	-4.41912	-2.23817	0.11228
O	-3.91027	0.23176	-0.19780
C	-3.52239	1.59373	-0.34678
H	-2.92298	1.74212	-1.25315
H	-4.44944	2.16250	-0.42840
H	-2.95340	1.94447	0.52248
C	1.59143	-0.06391	0.62177
C	1.11485	1.12360	1.41694
H	0.03662	1.25337	1.29471
H	1.26358	0.92699	2.48930
C	1.83807	2.44327	1.08446
H	1.39703	3.23690	1.70071
H	2.88614	2.36604	1.40169
C	1.81405	2.85662	-0.39433
H	2.32794	3.82368	-0.48841
H	2.40139	2.13451	-0.97458
C	0.42006	2.97140	-1.02718
H	0.53726	3.30731	-2.06590
H	-0.03168	1.97381	-1.08978
C	-0.52897	3.92470	-0.29555
H	-0.08771	4.92509	-0.20519
H	-1.47899	4.02645	-0.83237
H	-0.75706	3.57307	0.71699
H	3.81837	0.22024	0.64170

4e_conformer-122

C	0.91924	3.12216	-0.18998
C	0.06200	1.14568	0.54302
N	-0.20733	2.34351	-0.10588
H	-1.13517	2.65380	-0.35716
C	1.94419	2.39741	0.39195
C	0.90156	4.48228	-0.80696
H	0.55705	4.45432	-1.84862
H	0.24096	5.17137	-0.26460
H	1.90846	4.90817	-0.79730
C	-0.98986	0.14563	0.72182
C	-0.98587	-0.73470	1.81612
C	-2.04368	0.06161	-0.21271
C	-2.00543	-1.67604	1.96317
H	-0.20112	-0.66848	2.55998
C	-3.06695	-0.87426	-0.04684

H	-2.03172	0.70748	-1.08183
C	-3.04830	-1.75345	1.04604
H	-1.99221	-2.35134	2.81420
H	-3.84882	-2.47825	1.15254
O	-4.11909	-1.01906	-0.90518
C	-4.18985	-0.14915	-2.03004
H	-4.24655	0.90117	-1.71948
H	-3.32748	-0.28350	-2.69421
H	-5.10267	-0.42111	-2.56156
C	1.42206	1.15682	0.86182
C	2.25089	0.05758	1.47431
H	3.27263	0.43664	1.60608
H	1.89531	-0.19120	2.48229
C	2.30721	-1.23330	0.63377
H	2.88484	-1.98262	1.18957
H	1.29311	-1.63860	0.52555
C	2.91746	-1.02216	-0.75490
H	2.37750	-0.20851	-1.25522
H	3.95760	-0.67932	-0.64549
C	2.88471	-2.26858	-1.64992
H	1.84160	-2.59304	-1.77411
H	3.24096	-1.99429	-2.65195
C	3.72308	-3.43894	-1.12562
H	4.76719	-3.13582	-0.97650
H	3.34602	-3.81008	-0.16634
H	3.71595	-4.27822	-1.83047
H	2.97590	2.71781	0.46693

4e_conformer-123

C	-2.61377	-1.80677	-0.67800
C	-0.52727	-0.96299	-0.34580
N	-1.42239	-1.97158	-0.01737
H	-1.18028	-2.78593	0.52899
C	-2.49284	-0.64892	-1.42652
C	-3.73900	-2.77973	-0.54333
H	-4.60417	-2.42883	-1.11226
H	-3.47022	-3.77471	-0.92188
H	-4.04970	-2.90264	0.50192
C	0.79269	-0.90888	0.27818
C	1.00404	-1.43629	1.56389
C	1.88059	-0.32952	-0.40446
C	2.27215	-1.38289	2.14548
H	0.17351	-1.85661	2.12226
C	3.13891	-0.26222	0.19527
H	1.72947	0.04761	-1.40597

C	3.34157	-0.79753	1.47674
H	2.42331	-1.79027	3.14127
H	4.32936	-0.73902	1.92212
O	4.23728	0.29446	-0.39528
C	4.07257	0.89423	-1.67621
H	3.77254	0.15519	-2.42895
H	3.32937	1.70029	-1.64576
H	5.04704	1.30638	-1.94136
C	-1.18972	-0.10695	-1.22755
C	-0.72484	1.21353	-1.78354
H	0.15075	1.07883	-2.43306
H	-1.51679	1.60025	-2.43804
C	-0.38664	2.29811	-0.73962
H	0.42932	1.94670	-0.09645
H	0.00657	3.16498	-1.28772
C	-1.55974	2.76490	0.13756
H	-1.29034	3.73506	0.57985
H	-2.43693	2.95788	-0.49849
C	-1.95954	1.81888	1.27875
H	-2.33535	0.87691	0.86910
H	-1.06349	1.56417	1.86217
C	-3.01599	2.43532	2.19961
H	-2.65336	3.36577	2.65463
H	-3.93068	2.67574	1.64296
H	-3.29074	1.75085	3.01064
H	-3.26967	-0.21681	-2.04500

4e_conformer-124

C	2.61348	-1.80703	-0.67802
C	0.52709	-0.96299	-0.34579
N	1.42209	-1.97169	-0.01736
H	1.17989	-2.78599	0.52903
C	2.49268	-0.64918	-1.42654
C	3.73859	-2.78014	-0.54335
H	4.60378	-2.42937	-1.11233
H	4.04932	-2.90304	0.50190
H	3.46966	-3.77510	-0.92184
C	-0.79285	-0.90872	0.27824
C	-1.00418	-1.43602	1.56399
C	-1.88074	-0.32937	-0.40441
C	-2.27225	-1.38250	2.14564
H	-0.17364	-1.85634	2.12236
C	-3.13904	-0.26197	0.19536
H	-1.72964	0.04767	-1.40596
C	-3.34167	-0.79714	1.47689

H	-2.42339	-1.78979	3.14147
H	-4.32944	-0.73855	1.92229
O	-4.23741	0.29469	-0.39520
C	-4.07277	0.89422	-1.67625
H	-3.77278	0.15503	-2.42886
H	-5.04724	1.30633	-1.94143
H	-3.32956	1.70027	-1.64600
C	1.18964	-0.10704	-1.22755
C	0.72491	1.21349	-1.78354
H	1.51683	1.60003	-2.43820
H	-0.15081	1.07892	-2.43292
C	0.38708	2.29819	-0.73963
H	-0.00601	3.16512	-1.28773
H	-0.42888	1.94699	-0.09634
C	1.56040	2.76475	0.13738
H	2.43763	2.95724	-0.49876
H	1.29140	3.73512	0.57946
C	1.95988	1.81884	1.27877
H	1.06376	1.56472	1.86235
H	2.33519	0.87658	0.86933
C	3.01673	2.43501	2.19935
H	2.65460	3.36577	2.65416
H	3.29122	1.75062	3.01054
H	3.93148	2.67483	1.64254
H	3.26956	-0.21718	-2.04505

4e_conformer-125

C	-1.62886	3.11603	-0.22078
C	-0.05761	1.49365	0.04267
N	-0.28373	2.84127	-0.20321
H	0.43868	3.50155	-0.45231
C	-2.28220	1.92692	0.04885
C	-2.15546	4.48557	-0.50078
H	-1.77571	5.22467	0.21621
H	-1.87906	4.83458	-1.50435
H	-3.24688	4.48485	-0.43645
C	1.30201	0.96646	0.14127
C	2.35811	1.78452	0.59943
C	1.59486	-0.35547	-0.22080
C	3.64933	1.27849	0.68535
H	2.15646	2.80318	0.91597
C	2.89540	-0.86281	-0.11397
H	0.82701	-1.00657	-0.61988
C	3.93786	-0.04533	0.33754
H	4.45284	1.91656	1.04278

H	4.95153	-0.41722	0.41993
O	3.04618	-2.16656	-0.49288
C	4.35159	-2.73142	-0.42932
H	4.73879	-2.72828	0.59671
H	4.24934	-3.76077	-0.77541
H	5.05126	-2.19463	-1.08131
C	-1.30716	0.89898	0.21692
C	-1.60795	-0.53502	0.54673
H	-0.74684	-0.99868	1.04387
H	-2.43428	-0.56723	1.26764
C	-1.98950	-1.37241	-0.68882
H	-2.89468	-0.94556	-1.13916
H	-1.20304	-1.26723	-1.44772
C	-2.19447	-2.86389	-0.38991
H	-2.43424	-3.38048	-1.33000
H	-1.24286	-3.28810	-0.03923
C	-3.28823	-3.18127	0.64191
H	-3.33482	-4.27035	0.77330
H	-3.00580	-2.77197	1.62055
C	-4.67566	-2.66098	0.25437
H	-4.97560	-3.03769	-0.73158
H	-5.43338	-2.97884	0.97940
H	-4.69934	-1.56624	0.21104
H	-3.35521	1.80582	0.13147

4e_conformer-126

C	1.62885	3.11603	-0.22077
C	0.05760	1.49365	0.04267
N	0.28372	2.84127	-0.20321
H	-0.43868	3.50155	-0.45231
C	2.28219	1.92692	0.04886
C	2.15545	4.48558	-0.50077
H	1.87905	4.83459	-1.50434
H	1.77570	5.22467	0.21622
H	3.24687	4.48486	-0.43644
C	-1.30201	0.96646	0.14126
C	-2.35812	1.78452	0.59942
C	-1.59486	-0.35547	-0.22079
C	-3.64933	1.27848	0.68534
H	-2.15647	2.80318	0.91596
C	-2.89540	-0.86282	-0.11397
H	-0.82701	-1.00658	-0.61987
C	-3.93786	-0.04533	0.33753
H	-4.45285	1.91656	1.04276
H	-4.95154	-0.41722	0.41992

O	-3.04618	-2.16656	-0.49287
C	-4.35158	-2.73143	-0.42931
H	-5.05126	-2.19465	-1.08131
H	-4.24933	-3.76078	-0.77539
H	-4.73879	-2.72829	0.59672
C	1.30715	0.89899	0.21693
C	1.60795	-0.53501	0.54674
H	2.43427	-0.56722	1.26764
H	0.74684	-0.99868	1.04387
C	1.98950	-1.37240	-0.68882
H	1.20305	-1.26723	-1.44772
H	2.89469	-0.94555	-1.13916
C	2.19448	-2.86389	-0.38991
H	1.24287	-3.28810	-0.03923
H	2.43425	-3.38047	-1.33000
C	3.28824	-3.18126	0.64191
H	3.00582	-2.77196	1.62055
H	3.33483	-4.27034	0.77330
C	4.67567	-2.66097	0.25437
H	4.97561	-3.03768	-0.73159
H	4.69935	-1.56623	0.21103
H	5.43340	-2.97883	0.97939
H	3.35520	1.80582	0.13148

4e_conformer-127

C	2.37987	-2.17656	0.05981
C	0.71913	-0.72808	0.62686
N	1.02372	-1.96991	0.08565
H	0.33478	-2.67629	-0.12996
C	2.96346	-1.02969	0.56849
C	2.98028	-3.45259	-0.43230
H	2.68208	-3.67246	-1.46526
H	4.07116	-3.38559	-0.40424
H	2.68130	-4.31175	0.18245
C	-0.66953	-0.28394	0.72846
C	-1.08080	0.61917	1.73069
C	-1.63309	-0.75813	-0.17433
C	-2.40718	1.02676	1.80250
H	-0.36447	0.98072	2.45832
C	-2.97012	-0.35279	-0.08720
H	-1.35791	-1.42413	-0.98599
C	-3.36839	0.55097	0.90445
H	-2.71179	1.72213	2.57983
H	-4.39578	0.88335	0.98537
O	-3.80587	-0.88519	-1.02703

C	-5.17249	-0.48586	-1.00477
H	-5.65610	-0.76821	-0.06188
H	-5.27462	0.59584	-1.15372
H	-5.65168	-1.01327	-1.83059
C	1.93467	-0.10982	0.92632
C	2.18040	1.28966	1.42701
H	1.74049	1.42507	2.42400
H	3.26242	1.41343	1.56597
C	1.67364	2.42988	0.51914
H	0.59760	2.31219	0.35012
H	1.79106	3.36795	1.07821
C	2.40914	2.58214	-0.82314
H	2.17445	3.57735	-1.22821
H	3.49197	2.58138	-0.63103
C	2.09438	1.54171	-1.91147
H	2.73199	1.75881	-2.78000
H	2.37256	0.54189	-1.56444
C	0.62992	1.53827	-2.35943
H	0.32134	2.53248	-2.70824
H	0.47323	0.83253	-3.18384
H	-0.04083	1.24475	-1.54649
H	4.02807	-0.85378	0.65975

4e_conformer-128

C	2.37992	2.17656	0.05970
C	0.71917	0.72812	0.62682
N	1.02377	1.96993	0.08558
H	0.33483	2.67631	-0.13007
C	2.96351	1.02970	0.56840
C	2.98034	3.45258	-0.43246
H	2.68206	3.67245	-1.46539
H	2.68142	4.31174	0.18231
H	4.07122	3.38553	-0.40448
C	-0.66950	0.28399	0.72846
C	-1.08076	-0.61908	1.73072
C	-1.63307	0.75817	-0.17433
C	-2.40714	-1.02666	1.80256
H	-0.36442	-0.98062	2.45835
C	-2.97010	0.35283	-0.08717
H	-1.35790	1.42415	-0.98601
C	-3.36836	-0.55090	0.90451
H	-2.71174	-1.72201	2.57992
H	-4.39575	-0.88327	0.98545
O	-3.80586	0.88521	-1.02701
C	-5.17247	0.48587	-1.00473

H	-5.27459	-0.59584	-1.15366
H	-5.65608	0.76824	-0.06185
H	-5.65167	1.01324	-1.83057
C	1.93471	0.10985	0.92628
C	2.18045	-1.28960	1.42704
H	3.26247	-1.41335	1.56601
H	1.74053	-1.42497	2.42403
C	1.67372	-2.42987	0.51922
H	1.79124	-3.36793	1.07830
H	0.59766	-2.31227	0.35026
C	2.40914	-2.58211	-0.82310
H	3.49199	-2.58117	-0.63107
H	2.17459	-3.57739	-1.22808
C	2.09413	-1.54182	-1.91149
H	2.37222	-0.54193	-1.56458
H	2.73169	-1.75891	-2.78007
C	0.62963	-1.53863	-2.35930
H	0.32116	-2.53291	-2.70799
H	-0.04108	-1.24512	-1.54631
H	0.47275	-0.83298	-3.18375
H	4.02812	0.85378	0.65965

4e_conformer-129

C	-1.79481	2.69057	-0.17001
C	-0.04971	1.25459	0.08002
N	-0.43119	2.55060	-0.23886
H	0.20045	3.25833	-0.58525
C	-2.30104	1.46688	0.22903
C	-2.47957	3.97573	-0.50271
H	-2.12031	4.80414	0.12107
H	-2.32282	4.26561	-1.54998
H	-3.55634	3.87584	-0.34175
C	1.36418	0.88530	0.12694
C	2.34398	1.84728	0.45866
C	1.78647	-0.42002	-0.15835
C	3.68740	1.49509	0.49696
H	2.04528	2.85864	0.71625
C	3.14081	-0.77088	-0.10049
H	1.07779	-1.18124	-0.45928
C	4.10628	0.18857	0.22459
H	4.43082	2.24355	0.75707
H	5.15901	-0.06158	0.26731
O	3.41861	-2.07517	-0.39643
C	4.78166	-2.48611	-0.37803
H	5.22297	-2.36207	0.61823

H	4.77892	-3.54383	-0.64453
H	5.37745	-1.92673	-1.10943
C	-1.21631	0.55189	0.38737
C	-1.32998	-0.87426	0.85018
H	-0.39445	-1.17993	1.33274
H	-2.10597	-0.93958	1.62326
C	-1.65549	-1.88744	-0.26897
H	-0.86496	-1.84548	-1.02952
H	-1.61755	-2.89832	0.15697
C	-3.00478	-1.66789	-0.96835
H	-2.99738	-0.68369	-1.45304
H	-3.10230	-2.40998	-1.77323
C	-4.23949	-1.76539	-0.05904
H	-4.20984	-0.97139	0.69825
H	-5.13215	-1.56717	-0.66693
C	-4.39601	-3.12068	0.63738
H	-3.57487	-3.31374	1.33671
H	-5.33138	-3.16670	1.20654
H	-4.40738	-3.93891	-0.09366
H	-3.34711	1.25210	0.40466

4e_conformer-130

C	-0.58680	3.16553	-0.38222
C	-0.02604	1.00495	0.05270
N	0.37024	2.19381	-0.54543
H	1.18397	2.28317	-1.13716
C	-1.60257	2.60162	0.36598
C	-0.43797	4.53180	-0.96771
H	0.47496	5.02963	-0.61688
H	-0.39321	4.50596	-2.06441
H	-1.29097	5.15367	-0.68311
C	0.85597	-0.16376	0.02569
C	0.34540	-1.46368	-0.10580
C	2.25245	0.01441	0.11122
C	1.21057	-2.55895	-0.13259
H	-0.71908	-1.61176	-0.21963
C	3.11051	-1.08682	0.06461
H	2.64790	1.01380	0.24456
C	2.58760	-2.38397	-0.05124
H	0.80331	-3.56090	-0.23633
H	3.27191	-3.22577	-0.07890
O	4.47105	-1.00194	0.13961
C	5.05517	0.29192	0.25045
H	4.79952	0.92026	-0.61131
H	4.73839	0.79581	1.17161

H	6.13375	0.13189	0.27691
C	-1.26406	1.24174	0.64816
C	-2.08469	0.32261	1.51292
H	-1.54023	-0.60918	1.69900
H	-2.22481	0.79322	2.49606
C	-3.48103	0.01724	0.92821
H	-4.10043	-0.45389	1.70387
H	-3.96584	0.97053	0.68330
C	-3.45474	-0.88284	-0.31484
H	-4.39016	-0.76519	-0.87823
H	-2.65298	-0.54259	-0.98576
C	-3.26919	-2.37087	0.01042
H	-2.42028	-2.50452	0.69389
H	-4.15210	-2.72518	0.55981
C	-3.05544	-3.23178	-1.23670
H	-2.14352	-2.93267	-1.76851
H	-2.96110	-4.29347	-0.98217
H	-3.89413	-3.12680	-1.93588
H	-2.49584	3.11781	0.69482

4e_conformer-131

C	0.58676	3.16553	-0.38221
C	0.02604	1.00494	0.05271
N	-0.37026	2.19380	-0.54542
H	-1.18401	2.28315	-1.13713
C	1.60255	2.60163	0.36598
C	0.43791	4.53179	-0.96770
H	1.29090	5.15369	-0.68309
H	0.39315	4.50596	-2.06440
H	-0.47503	5.02961	-0.61687
C	-0.85597	-0.16377	0.02571
C	-0.34539	-1.46369	-0.10576
C	-2.25244	0.01440	0.11122
C	-1.21056	-2.55897	-0.13255
H	0.71909	-1.61177	-0.21959
C	-3.11051	-1.08683	0.06462
H	-2.64790	1.01379	0.24454
C	-2.58760	-2.38399	-0.05122
H	-0.80330	-3.56092	-0.23628
H	-3.27190	-3.22579	-0.07887
O	-4.47105	-1.00195	0.13960
C	-5.05517	0.29191	0.25041
H	-6.13375	0.13188	0.27686
H	-4.73840	0.79581	1.17156
H	-4.79951	0.92023	-0.61136

C	1.26406	1.24175	0.64815
C	2.08471	0.32263	1.51291
H	2.22484	0.79325	2.49604
H	1.54027	-0.60916	1.69900
C	3.48105	0.01727	0.92819
H	3.96585	0.97056	0.68326
H	4.10047	-0.45385	1.70383
C	3.45477	-0.88282	-0.31487
H	2.65300	-0.54257	-0.98579
H	4.39018	-0.76518	-0.87826
C	3.26921	-2.37085	0.01040
H	4.15212	-2.72515	0.55980
H	2.42029	-2.50449	0.69388
C	3.05546	-3.23176	-1.23671
H	3.89414	-3.12679	-1.93589
H	2.96112	-4.29345	-0.98216
H	2.14353	-2.93266	-1.76851
H	2.49581	3.11783	0.69481

4e_conformer-132

C	0.78143	2.78368	-0.11763
C	-0.11421	0.71303	0.16986
N	-0.29101	1.97079	-0.39225
H	-1.03794	2.20307	-1.03120
C	1.64932	2.04683	0.66600
C	0.87258	4.18018	-0.64005
H	0.89095	4.20748	-1.73743
H	0.02407	4.79516	-0.31400
H	1.78948	4.65361	-0.27881
C	-1.15181	-0.31081	0.03161
C	-0.83266	-1.67143	-0.09998
C	-2.50863	0.07560	0.01182
C	-1.84930	-2.61984	-0.22980
H	0.20426	-1.98030	-0.13491
C	-3.51603	-0.87992	-0.13766
H	-2.75940	1.12053	0.14670
C	-3.18638	-2.23895	-0.25257
H	-1.59211	-3.67052	-0.33196
H	-3.98455	-2.96621	-0.36077
O	-4.84986	-0.58964	-0.16792
C	-5.23950	0.77575	-0.06225
H	-4.92955	1.20665	0.89748
H	-4.82003	1.37481	-0.87956
H	-6.32822	0.78215	-0.12802
C	1.09864	0.74198	0.85597

C	1.71836	-0.33011	1.71051
H	1.86722	0.06389	2.72565
H	1.03379	-1.17952	1.80940
C	3.08236	-0.81897	1.18326
H	3.75075	0.04694	1.09837
H	3.53560	-1.48838	1.92748
C	2.99734	-1.54519	-0.16218
H	2.38531	-2.44938	-0.03598
H	2.46518	-0.90887	-0.88279
C	4.35801	-1.94629	-0.75005
H	4.90430	-2.55828	-0.01826
H	4.18900	-2.59129	-1.62249
C	5.22629	-0.75664	-1.17314
H	4.69703	-0.12676	-1.89912
H	5.49448	-0.12378	-0.32015
H	6.15887	-1.09427	-1.63934
H	2.58257	2.41148	1.07655

4e_conformer-133

C	1.34265	3.28204	-0.34095
C	-0.07243	1.51100	-0.13794
N	0.05947	2.89079	-0.05545
H	-0.71423	3.52657	0.07544
C	2.06203	2.12633	-0.58929
C	1.75065	4.71879	-0.36023
H	1.57336	5.20757	0.60628
H	2.81712	4.79949	-0.58698
H	1.20205	5.29129	-1.11954
C	-1.35287	0.86897	0.15326
C	-2.30101	1.49326	0.98342
C	-1.66586	-0.38952	-0.39933
C	-3.52388	0.87393	1.24629
H	-2.07308	2.44489	1.45300
C	-2.88257	-1.00940	-0.11195
H	-0.95783	-0.86240	-1.06357
C	-3.82472	-0.37362	0.71072
H	-4.24592	1.36639	1.89167
H	-4.76663	-0.87126	0.91717
O	-3.24844	-2.23223	-0.59814
C	-2.30625	-2.94220	-1.39519
H	-2.07411	-2.40037	-2.32002
H	-1.37665	-3.12725	-0.84276
H	-2.77912	-3.89373	-1.64164
C	1.18854	1.00658	-0.47152
C	1.62601	-0.42841	-0.60164

H	1.06313	-0.94978	-1.38848
H	2.66850	-0.43286	-0.94020
C	1.51369	-1.22968	0.70804
H	2.17608	-0.77441	1.45505
H	0.49603	-1.13004	1.10184
C	1.83347	-2.72213	0.54737
H	1.71270	-3.21435	1.52263
H	1.08434	-3.17354	-0.11962
C	3.23433	-3.03964	0.00155
H	3.33744	-4.12984	-0.07812
H	3.33048	-2.65266	-1.02133
C	4.37366	-2.48741	0.86342
H	4.29268	-2.84460	1.89773
H	5.34971	-2.80068	0.47602
H	4.36521	-1.39195	0.89056
H	3.11739	2.08245	-0.82816

4e_conformer-134

C	-1.34262	3.28205	-0.34096
C	0.07245	1.51100	-0.13793
N	-0.05945	2.89079	-0.05545
H	0.71425	3.52656	0.07545
C	-2.06202	2.12633	-0.58930
C	-1.75062	4.71880	-0.36024
H	-1.57333	5.20759	0.60626
H	-1.20202	5.29129	-1.11956
H	-2.81709	4.79950	-0.58700
C	1.35288	0.86896	0.15327
C	2.30102	1.49323	0.98344
C	1.66586	-0.38952	-0.39933
C	3.52388	0.87389	1.24630
H	2.07310	2.44487	1.45302
C	2.88257	-1.00941	-0.11196
H	0.95782	-0.86239	-1.06358
C	3.82472	-0.37366	0.71073
H	4.24593	1.36634	1.89169
H	4.76662	-0.87131	0.91717
O	3.24842	-2.23224	-0.59815
C	2.30623	-2.94219	-1.39524
H	2.07413	-2.40035	-2.32006
H	2.77909	-3.89372	-1.64169
H	1.37663	-3.12723	-0.84282
C	-1.18853	1.00659	-0.47152
C	-1.62601	-0.42841	-0.60162
H	-2.66848	-0.43285	-0.94021

H	-1.06311	-0.94979	-1.38844
C	-1.51372	-1.22965	0.70807
H	-0.49607	-1.13001	1.10190
H	-2.17613	-0.77437	1.45505
C	-1.83349	-2.72211	0.54742
H	-1.08434	-3.17353	-0.11955
H	-1.71275	-3.21431	1.52269
C	-3.23434	-3.03963	0.00156
H	-3.33045	-2.65269	-1.02134
H	-3.33744	-4.12984	-0.07808
C	-4.37370	-2.48738	0.86338
H	-5.34973	-2.80066	0.47596
H	-4.29274	-2.84453	1.89770
H	-4.36525	-1.39192	0.89048
H	-3.11737	2.08247	-0.82818

4e_conformer-135

C	0.57921	3.35171	-0.07752
C	0.44647	1.08180	-0.06354
N	1.26771	2.18042	-0.27838
H	2.20315	2.12439	-0.65499
C	-0.70300	3.00038	0.30425
C	1.21372	4.68909	-0.27800
H	2.09543	4.82370	0.36152
H	1.53934	4.83762	-1.31591
H	0.49887	5.48011	-0.03595
C	0.96195	-0.28083	-0.18475
C	0.13783	-1.34245	-0.59374
C	2.31684	-0.54618	0.10759
C	0.65609	-2.63505	-0.69132
H	-0.89360	-1.15461	-0.86440
C	2.82833	-1.83984	-0.01220
H	2.94776	0.26020	0.46056
C	1.99234	-2.89507	-0.40830
H	0.00929	-3.44836	-1.00864
H	2.40829	-3.89421	-0.48772
O	4.12482	-2.17886	0.24984
C	5.01904	-1.14506	0.64829
H	4.70444	-0.68691	1.59379
H	5.98957	-1.62432	0.78280
H	5.09839	-0.36755	-0.12113
C	-0.80048	1.57695	0.31780
C	-2.01882	0.78824	0.70393
H	-1.71930	-0.21133	1.03558
H	-2.50651	1.26644	1.56461

C	-3.05376	0.67522	-0.43259
H	-3.37323	1.68809	-0.71022
H	-2.56885	0.25676	-1.32586
C	-4.28142	-0.17214	-0.07478
H	-4.73786	0.21762	0.84722
H	-5.03804	-0.05537	-0.86325
C	-3.98386	-1.66662	0.09999
H	-3.50711	-2.04553	-0.81544
H	-3.25462	-1.81254	0.90696
C	-5.23972	-2.48827	0.40131
H	-5.97594	-2.39259	-0.40628
H	-5.00555	-3.55256	0.51740
H	-5.71880	-2.14789	1.32760
H	-1.49206	3.69547	0.56302

4e_conformer-136

C	-0.57920	3.35173	-0.07752
C	-0.44647	1.08181	-0.06353
N	-1.26771	2.18044	-0.27839
H	-2.20315	2.12442	-0.65500
C	0.70299	3.00039	0.30426
C	-1.21371	4.68910	-0.27800
H	-0.49887	5.48013	-0.03593
H	-1.53931	4.83765	-1.31593
H	-2.09544	4.82372	0.36150
C	-0.96195	-0.28082	-0.18475
C	-0.13781	-1.34243	-0.59374
C	-2.31683	-0.54619	0.10759
C	-0.65606	-2.63504	-0.69132
H	0.89361	-1.15457	-0.86440
C	-2.82830	-1.83985	-0.01221
H	-2.94777	0.26018	0.46056
C	-1.99231	-2.89507	-0.40830
H	-0.00925	-3.44834	-1.00864
H	-2.40824	-3.89422	-0.48772
O	-4.12479	-2.17889	0.24983
C	-5.01903	-1.14510	0.64829
H	-5.09839	-0.36759	-0.12113
H	-5.98955	-1.62437	0.78279
H	-4.70443	-0.68695	1.59378
C	0.80048	1.57696	0.31781
C	2.01881	0.78826	0.70394
H	2.50651	1.26645	1.56462
H	1.71929	-0.21132	1.03559
C	3.05375	0.67523	-0.43258

H	2.56884	0.25677	-1.32585
H	3.37324	1.68809	-0.71021
C	4.28140	-0.17215	-0.07477
H	4.73784	0.21759	0.84723
H	5.03803	-0.05538	-0.86324
C	3.98382	-1.66663	0.09998
H	3.25457	-1.81255	0.90694
H	3.50707	-2.04553	-0.81546
C	5.23967	-2.48830	0.40130
H	5.71875	-2.14793	1.32760
H	5.00548	-3.55259	0.51738
H	5.97590	-2.39263	-0.40628
H	1.49206	3.69549	0.56303

4e_conformer-137

C	-0.84758	3.21997	0.01915
C	0.01569	1.14156	0.35678
N	0.29984	2.46990	0.06961
H	1.23354	2.85321	0.03246
C	-1.89837	2.35057	0.25271
C	-0.82478	4.69189	-0.23414
H	-0.27947	5.23509	0.54876
H	-0.34636	4.93483	-1.19154
H	-1.84628	5.08063	-0.26068
C	1.08933	0.15186	0.43211
C	0.96745	-1.00893	1.22500
C	2.28125	0.34355	-0.28280
C	2.00621	-1.92992	1.28087
H	0.07055	-1.17301	1.80932
C	3.32878	-0.58209	-0.20981
H	2.40993	1.20011	-0.93694
C	3.19704	-1.73432	0.57347
H	1.89979	-2.81866	1.89685
H	3.99167	-2.46708	0.63827
O	4.43304	-0.27619	-0.95328
C	5.52253	-1.19279	-0.93450
H	5.93094	-1.30736	0.07682
H	5.22365	-2.17612	-1.31715
H	6.28452	-0.76512	-1.58739
C	-1.37368	1.04358	0.47229
C	-2.21562	-0.18739	0.67834
H	-1.96282	-0.69415	1.61962
H	-3.25813	0.13263	0.78895
C	-2.11323	-1.20013	-0.47630
H	-2.45227	-0.71406	-1.39988

H	-1.05855	-1.45312	-0.63412
C	-2.89912	-2.49623	-0.23446
H	-2.75089	-3.16200	-1.09647
H	-2.46617	-3.01482	0.63289
C	-4.40754	-2.31706	-0.00175
H	-4.85309	-3.30666	0.16494
H	-4.57601	-1.75168	0.92414
C	-5.13488	-1.62564	-1.15880
H	-4.78786	-0.59531	-1.29603
H	-4.96806	-2.15937	-2.10289
H	-6.21558	-1.58914	-0.98086
H	-2.94713	2.62050	0.26078

4e_conformer-138

C	0.24138	-3.48556	0.12364
C	0.18735	-1.22494	0.37462
N	0.99628	-2.34687	0.25301
H	1.99816	-2.33773	0.38179
C	-1.08312	-3.08482	0.13109
C	0.85950	-4.84156	0.02137
H	1.42739	-5.10390	0.92367
H	0.08008	-5.59637	-0.11328
H	1.54934	-4.91354	-0.82915
C	0.77618	0.10835	0.49473
C	0.13612	1.13017	1.21468
C	2.02077	0.38208	-0.11065
C	0.72363	2.39235	1.31483
H	-0.80404	0.93245	1.71469
C	2.60694	1.64412	0.00982
H	2.50060	-0.38831	-0.70128
C	1.95348	2.66011	0.72305
H	0.21847	3.17461	1.87443
H	2.42202	3.63594	0.79820
O	3.80596	1.98768	-0.54533
C	4.51492	0.99230	-1.27613
H	4.75404	0.12808	-0.64467
H	3.94352	0.65479	-2.14931
H	5.43898	1.46681	-1.60873
C	-1.13398	-1.66925	0.29320
C	-2.39302	-0.84519	0.24580
H	-3.25414	-1.52483	0.28836
H	-2.47861	-0.20353	1.12983
C	-2.50478	0.01348	-1.02800
H	-1.64691	0.69425	-1.07807
H	-2.41737	-0.65125	-1.89714

C	-3.81728	0.80321	-1.13162
H	-3.86558	1.27838	-2.12176
H	-4.65873	0.09697	-1.08875
C	-4.02022	1.88658	-0.06034
H	-5.02383	2.31417	-0.18597
H	-4.01091	1.43120	0.93868
C	-2.98452	3.01411	-0.11721
H	-2.97850	3.48927	-1.10645
H	-3.20532	3.79013	0.62482
H	-1.97241	2.64686	0.08051
H	-1.93802	-3.74094	0.02390

4e_conformer-139

C	-0.24039	-3.48561	0.12358
C	-0.18703	-1.22500	0.37476
N	-0.99563	-2.34716	0.25302
H	-1.99752	-2.33831	0.38173
C	1.08399	-3.08449	0.13110
C	-0.85811	-4.84179	0.02116
H	-0.07846	-5.59636	-0.11344
H	-1.42604	-5.10433	0.92337
H	-1.54782	-4.91392	-0.82945
C	-0.77626	0.10812	0.49495
C	-0.13665	1.13000	1.21522
C	-2.02077	0.38161	-0.11070
C	-0.72452	2.39201	1.31542
H	0.80342	0.93245	1.71545
C	-2.60731	1.64347	0.00982
H	-2.50025	-0.38882	-0.70156
C	-1.95430	2.65952	0.72337
H	-0.21973	3.17432	1.87527
H	-2.42313	3.63522	0.79856
O	-3.80629	1.98680	-0.54556
C	-4.51481	0.99133	-1.27668
H	-4.75385	0.12695	-0.64541
H	-5.43892	1.46565	-1.60942
H	-3.94312	0.65411	-2.14977
C	1.13443	-1.66891	0.29335
C	2.39322	-0.84447	0.24601
H	2.47839	-0.20252	1.12986
H	3.25454	-1.52381	0.28896
C	2.50498	0.01387	-1.02802
H	2.41804	-0.65113	-1.89699
H	1.64686	0.69431	-1.07850
C	3.81721	0.80408	-1.13152

H	4.65892	0.09817	-1.08826
H	3.86557	1.27901	-2.12177
C	4.01947	1.88780	-0.06046
H	4.01017	1.43266	0.93866
H	5.02292	2.31580	-0.18602
C	2.98329	3.01486	-0.11779
H	1.97131	2.64722	0.07990
H	3.20365	3.79118	0.62404
H	2.97719	3.48974	-1.10718
H	1.93908	-3.74034	0.02389

4e_conformer-140

C	2.70722	-1.87410	-0.42512
C	0.62233	-0.96889	-0.33056
N	1.46664	-1.95749	0.15570
H	1.16603	-2.71373	0.75412
C	2.66928	-0.78834	-1.28257
C	3.79606	-2.84966	-0.11894
H	4.71190	-2.56071	-0.64155
H	4.01838	-2.88959	0.95488
H	3.53716	-3.86863	-0.43562
C	-0.73786	-0.83384	0.18633
C	-1.04699	-1.22251	1.50777
C	-1.76732	-0.32250	-0.61462
C	-2.34469	-1.09478	1.98925
H	-0.26308	-1.59113	2.16187
C	-3.06779	-0.18212	-0.11744
H	-1.58034	-0.03805	-1.64333
C	-3.36898	-0.57346	1.19292
H	-2.56975	-1.39210	3.00979
H	-4.36987	-0.47660	1.59462
O	-3.97753	0.33492	-0.99559
C	-5.32204	0.48293	-0.55117
H	-5.38754	1.16767	0.30298
H	-5.87164	0.90197	-1.39505
H	-5.75995	-0.48364	-0.27402
C	1.36794	-0.20901	-1.23279
C	0.97463	1.06496	-1.93283
H	1.81556	1.37488	-2.56681
H	0.13638	0.89111	-2.62062
C	0.60276	2.24391	-1.00949
H	0.27540	3.07079	-1.65399
H	-0.26553	1.96964	-0.39791
C	1.72916	2.75160	-0.09432
H	2.65750	2.84919	-0.67737

H	1.47183	3.76947	0.23294
C	2.00361	1.90724	1.15818
H	1.05814	1.74975	1.69625
H	2.35987	0.91452	0.86880
C	3.02335	2.56653	2.09062
H	2.67712	3.55243	2.42593
H	3.20690	1.95519	2.98190
H	3.98517	2.71074	1.58226
H	3.49993	-0.42782	-1.87652

4e_conformer-141

C	-2.70736	-1.87399	-0.42501
C	-0.62242	-0.96889	-0.33052
N	-1.46679	-1.95738	0.15584
H	-1.16621	-2.71361	0.75430
C	-2.66934	-0.78832	-1.28258
C	-3.79625	-2.84946	-0.11875
H	-3.53742	-3.86846	-0.43538
H	-4.01855	-2.88931	0.95507
H	-4.71208	-2.56049	-0.64138
C	0.73775	-0.83381	0.18640
C	1.04682	-1.22219	1.50794
C	1.76727	-0.32270	-0.61464
C	2.34451	-1.09442	1.98943
H	0.26286	-1.59059	2.16209
C	3.06772	-0.18227	-0.11745
H	1.58033	-0.03847	-1.64342
C	3.36885	-0.57333	1.19301
H	2.56952	-1.39151	3.01005
H	4.36974	-0.47644	1.59472
O	3.97752	0.33454	-0.99568
C	5.32203	0.48254	-0.55125
H	5.38756	1.16745	0.30276
H	5.75987	-0.48401	-0.27391
H	5.87168	0.90138	-1.39521
C	-1.36797	-0.20906	-1.23285
C	-0.97458	1.06484	-1.93296
H	-0.13638	0.89089	-2.62079
H	-1.81551	1.37481	-2.56691
C	-0.60257	2.24379	-1.00968
H	0.26574	1.96946	-0.39814
H	-0.27517	3.07063	-1.65421
C	-1.72887	2.75160	-0.09445
H	-1.47138	3.76942	0.23284
H	-2.65721	2.84934	-0.67748

C	-2.00342	1.90723	1.15802
H	-2.35994	0.91460	0.86861
H	-1.05794	1.74948	1.69599
C	-3.02292	2.56670	2.09057
H	-2.67642	3.55250	2.42592
H	-3.98475	2.71118	1.58230
H	-3.20656	1.95536	2.98183
H	-3.49996	-0.42781	-1.87657

4e_conformer-142

C	2.92423	-1.60159	0.09014
C	0.76107	-0.90167	0.11458
N	1.71169	-1.65995	-0.55364
H	1.50009	-2.27749	-1.32466
C	2.75759	-0.76007	1.17456
C	4.11118	-2.37608	-0.38190
H	3.94441	-3.45994	-0.32713
H	4.36543	-2.13864	-1.42269
H	4.97995	-2.14052	0.23873
C	-0.58556	-0.74513	-0.43780
C	-0.77561	-0.64915	-1.82627
C	-1.70849	-0.69076	0.41008
C	-2.06071	-0.48931	-2.34896
H	0.08292	-0.66528	-2.48994
C	-2.98704	-0.50955	-0.12188
H	-1.56641	-0.81733	1.47463
C	-3.16700	-0.41091	-1.51026
H	-2.19821	-0.41061	-3.42378
H	-4.17016	-0.27715	-1.90195
O	-4.12468	-0.43495	0.62923
C	-3.99446	-0.49902	2.04575
H	-3.58006	-1.46246	2.36639
H	-3.35956	0.31120	2.42443
H	-5.00368	-0.38792	2.44429
C	1.40214	-0.31201	1.20226
C	0.87073	0.74362	2.13166
H	-0.21922	0.68123	2.20499
H	1.25815	0.55854	3.14245
C	1.23734	2.18916	1.72739
H	0.86333	2.85450	2.51674
H	2.33034	2.30120	1.72211
C	0.66450	2.63652	0.36852
H	-0.31183	2.15659	0.21327
H	0.46945	3.71780	0.39718
C	1.56572	2.34897	-0.83959

H	2.51071	2.89709	-0.71449
H	1.82562	1.28649	-0.86398
C	0.90937	2.74559	-2.16408
H	-0.00871	2.16883	-2.32759
H	0.64245	3.81019	-2.17297
H	1.57602	2.56112	-3.01478
H	3.53450	-0.47996	1.87521

4e_conformer-143

C	2.73743	-1.83882	-0.32668
C	0.95627	-0.76712	0.59346
N	1.37346	-1.85470	-0.15983
H	0.77012	-2.61913	-0.42874
C	3.20098	-0.69827	0.30288
C	3.46093	-2.92863	-1.04801
H	3.36549	-3.89547	-0.53643
H	3.08256	-3.06280	-2.06934
H	4.52576	-2.68909	-1.11260
C	-0.46874	-0.52185	0.82234
C	-0.94359	-0.01520	2.04878
C	-1.39416	-0.79129	-0.19474
C	-2.30085	0.23022	2.22162
H	-0.25004	0.15776	2.86381
C	-2.76134	-0.55219	-0.00931
H	-1.06276	-1.15619	-1.16152
C	-3.22493	-0.03143	1.20433
H	-2.65910	0.62047	3.17022
H	-4.27706	0.16688	1.36687
O	-3.55812	-0.84421	-1.07933
C	-4.95431	-0.59227	-0.95741
H	-5.15312	0.46997	-0.77086
H	-5.39517	-0.88238	-1.91198
H	-5.39946	-1.19020	-0.15307
C	2.09109	-0.01413	0.88513
C	2.15654	1.35113	1.51057
H	1.29407	1.51633	2.16346
H	3.04547	1.40975	2.15278
C	2.21407	2.51338	0.49384
H	2.34217	3.44209	1.06548
H	3.11267	2.40901	-0.12978
C	0.97023	2.63775	-0.40756
H	0.08018	2.33673	0.16234
H	0.81909	3.69459	-0.66902
C	1.03080	1.83133	-1.71169
H	1.87798	2.19297	-2.31218

H	1.23648	0.78005	-1.48939
C	-0.26151	1.93918	-2.52479
H	-0.49061	2.98393	-2.77123
H	-0.19227	1.38125	-3.46608
H	-1.10958	1.53523	-1.95918
H	4.23451	-0.37600	0.33347

4e_conformer-144

C	0.98203	2.42845	-0.12095
C	-0.17901	0.47616	-0.00891
N	-0.23125	1.82393	-0.33794
H	-1.01738	2.26630	-0.79202
C	1.82540	1.45736	0.38709
C	1.21754	3.87015	-0.43270
H	1.10006	4.08319	-1.50333
H	0.52120	4.52444	0.10718
H	2.23443	4.15144	-0.14611
C	-1.37230	-0.36322	-0.10658
C	-1.28647	-1.73572	-0.39171
C	-2.64734	0.21197	0.08351
C	-2.44595	-2.50958	-0.47155
H	-0.32193	-2.18945	-0.58187
C	-3.80062	-0.56827	-0.01704
H	-2.71914	1.26063	0.34389
C	-3.70171	-1.94098	-0.29093
H	-2.36742	-3.57011	-0.69419
H	-4.61005	-2.53087	-0.35829
O	-5.06790	-0.08853	0.15110
C	-5.22705	1.30027	0.42130
H	-4.81630	1.91401	-0.38951
H	-6.30198	1.46913	0.49684
H	-4.74649	1.58137	1.36624
C	1.11102	0.22270	0.46035
C	1.65369	-1.07207	0.99855
H	2.29108	-0.85909	1.86565
H	0.82934	-1.69069	1.37165
C	2.46272	-1.90312	-0.02256
H	2.76385	-2.84427	0.45882
H	1.80519	-2.17725	-0.85797
C	3.70547	-1.20512	-0.59008
H	4.14109	-1.84419	-1.37093
H	3.40293	-0.27433	-1.08750
C	4.78848	-0.89286	0.44836
H	4.38112	-0.23759	1.22873
H	5.08289	-1.82301	0.95500

C	6.02132	-0.22911	-0.17022
H	5.75430	0.71954	-0.65226
H	6.78651	-0.01688	0.58513
H	6.47368	-0.87247	-0.93497
H	2.85157	1.61881	0.69111

4e_conformer-145

C	-2.89011	-1.44328	-0.73125
C	-0.72663	-0.81473	-0.41133
N	-1.67961	-1.80105	-0.19370
H	-1.47236	-2.71281	0.18815
C	-2.72096	-0.18554	-1.28257
C	-4.08154	-2.34233	-0.67664
H	-3.91163	-3.28323	-1.21629
H	-4.34945	-2.60318	0.35511
H	-4.94277	-1.84661	-1.13251
C	0.63453	-0.97470	0.09558
C	0.89380	-1.76641	1.22843
C	1.71559	-0.34070	-0.54828
C	2.19952	-1.91490	1.69905
H	0.07411	-2.23535	1.76350
C	3.01438	-0.47935	-0.05675
H	1.52724	0.24180	-1.43848
C	3.26325	-1.27629	1.07109
H	2.38650	-2.52555	2.57796
H	4.28135	-1.37373	1.43365
O	4.10933	0.11376	-0.61727
C	3.90344	0.97071	-1.73577
H	3.49317	0.42077	-2.59136
H	3.23101	1.79978	-1.48384
H	4.88670	1.36471	-1.99600
C	-1.36963	0.22461	-1.08764
C	-0.82678	1.57510	-1.47842
H	-0.01224	1.46611	-2.20731
H	-1.62044	2.11637	-2.00990
C	-0.32224	2.46520	-0.32319
H	0.44084	1.92679	0.24938
H	0.19010	3.32566	-0.77445
C	-1.41132	3.00433	0.61938
H	-0.97448	3.82382	1.20862
H	-2.20669	3.45923	0.01122
C	-2.04389	2.00066	1.59801
H	-2.82579	2.52755	2.16280
H	-2.54654	1.20300	1.04229
C	-1.04737	1.38533	2.58464

H	-1.55891	0.73811	3.30691
H	-0.29698	0.77534	2.07304
H	-0.51941	2.16422	3.15025
H	-3.49512	0.39832	-1.76472

4e_conformer-146

C	1.67363	2.49431	0.03984
C	0.29270	0.70478	0.28895
N	0.42746	1.98604	-0.23075
H	-0.25210	2.42407	-0.83601
C	2.34384	1.53497	0.77626
C	2.10379	3.84082	-0.44282
H	1.44198	4.63697	-0.07870
H	3.11583	4.05675	-0.09004
H	2.11117	3.90001	-1.53906
C	-0.96566	-0.02656	0.13541
C	-0.99515	-1.42763	-0.02452
C	-2.18303	0.67027	0.12199
C	-2.20943	-2.08724	-0.17695
H	-0.06930	-1.98703	-0.05943
C	-3.40022	0.00050	-0.04879
H	-2.20992	1.74508	0.27196
C	-3.42237	-1.39177	-0.19392
H	-2.21953	-3.16649	-0.30227
H	-4.35299	-1.93083	-0.32079
O	-4.51192	0.79371	-0.04510
C	-5.77985	0.16313	-0.19511
H	-5.85422	-0.36081	-1.15560
H	-5.97199	-0.54571	0.61931
H	-6.51857	0.96492	-0.16075
C	1.48942	0.40183	0.93598
C	1.83533	-0.82224	1.73930
H	2.08050	-0.50465	2.76297
H	0.96175	-1.47671	1.82988
C	3.03542	-1.63898	1.21448
H	3.89226	-0.96783	1.07480
H	3.32853	-2.34039	2.00692
C	2.79421	-2.45464	-0.06613
H	3.64715	-3.13616	-0.19778
H	1.91820	-3.10110	0.08982
C	2.61040	-1.66608	-1.37353
H	2.42880	-2.38925	-2.18056
H	1.71563	-1.03870	-1.31223
C	3.81113	-0.79161	-1.74497
H	4.73719	-1.38032	-1.77261

H	3.67423	-0.33766	-2.73342
H	3.94736	0.02190	-1.02555
H	3.34622	1.63870	1.17227

4e_conformer-147

C	-1.67364	2.49431	0.03984
C	-0.29270	0.70478	0.28896
N	-0.42746	1.98603	-0.23075
H	0.25210	2.42407	-0.83600
C	-2.34384	1.53497	0.77626
C	-2.10378	3.84082	-0.44282
H	-1.44198	4.63697	-0.07870
H	-2.11117	3.90001	-1.53906
H	-3.11583	4.05675	-0.09004
C	0.96566	-0.02656	0.13542
C	0.99515	-1.42764	-0.02450
C	2.18303	0.67027	0.12199
C	2.20943	-2.08724	-0.17693
H	0.06930	-1.98704	-0.05940
C	3.40022	0.00050	-0.04879
H	2.20991	1.74509	0.27195
C	3.42237	-1.39177	-0.19390
H	2.21953	-3.16649	-0.30224
H	4.35299	-1.93083	-0.32078
O	4.51191	0.79371	-0.04510
C	5.77984	0.16314	-0.19512
H	5.85421	-0.36081	-1.15561
H	6.51856	0.96493	-0.16077
H	5.97199	-0.54570	0.61930
C	-1.48942	0.40183	0.93599
C	-1.83534	-0.82223	1.73930
H	-0.96176	-1.47671	1.82990
H	-2.08052	-0.50465	2.76297
C	-3.03543	-1.63898	1.21447
H	-3.32855	-2.34039	2.00691
H	-3.89226	-0.96782	1.07477
C	-2.79420	-2.45464	-0.06614
H	-1.91820	-3.10110	0.08982
H	-3.64714	-3.13616	-0.19780
C	-2.61038	-1.66608	-1.37353
H	-1.71561	-1.03870	-1.31223
H	-2.42877	-2.38925	-2.18056
C	-3.81110	-0.79161	-1.74499
H	-3.94734	0.02190	-1.02557
H	-3.67419	-0.33766	-2.73344

H	-4.73716	-1.38032	-1.77264
H	-3.34622	1.63871	1.17227

4e_conformer-148

C	0.36969	3.26553	0.13143
C	-0.29504	1.09753	0.26704
N	-0.70908	2.42103	0.21438
H	-1.66468	2.72115	0.34396
C	1.50035	2.46883	0.10036
C	0.21479	4.75099	0.09760
H	-0.39364	5.07983	-0.75464
H	-0.26560	5.13484	1.00709
H	1.19612	5.22547	0.01261
C	-1.26606	0.00827	0.34883
C	-0.97169	-1.18345	1.04401
C	-2.52619	0.13510	-0.25404
C	-1.91359	-2.20404	1.10816
H	-0.02166	-1.28892	1.55455
C	-3.47488	-0.89041	-0.17027
H	-2.78352	1.02208	-0.82456
C	-3.17084	-2.07624	0.50938
H	-1.67816	-3.11706	1.64812
H	-3.88780	-2.88477	0.58006
O	-4.66275	-0.64385	-0.79767
C	-5.65741	-1.66226	-0.76365
H	-5.96469	-1.88646	0.26491
H	-6.50909	-1.26719	-1.31891
H	-5.30335	-2.58240	-1.24393
C	1.09726	1.10125	0.18460
C	2.00489	-0.09832	0.12168
H	2.39413	-0.33166	1.12318
H	1.42568	-0.97858	-0.18343
C	3.18503	0.07422	-0.84824
H	2.79185	0.38200	-1.82580
H	3.82908	0.89457	-0.50358
C	4.03286	-1.19285	-1.01984
H	3.39326	-2.01121	-1.38264
H	4.77817	-1.01552	-1.80798
C	4.76137	-1.64911	0.24986
H	5.36082	-0.81398	0.63972
H	4.03158	-1.89360	1.03214
C	5.66472	-2.86102	0.00732
H	6.17546	-3.17244	0.92561
H	5.08403	-3.71655	-0.35924
H	6.43187	-2.63616	-0.74390

H	2.51426	2.83609	0.01192

4e_conformer-149

C	-0.36972	3.26552	0.13141
C	0.29503	1.09753	0.26709
N	0.70906	2.42103	0.21437
H	1.66466	2.72117	0.34392
C	-1.50037	2.46881	0.10039
C	-0.21483	4.75098	0.09753
H	-1.19617	5.22545	0.01254
H	0.26558	5.13487	1.00700
H	0.39358	5.07980	-0.75473
C	1.26607	0.00829	0.34889
C	0.97175	-1.18340	1.04417
C	2.52615	0.13509	-0.25407
C	1.91367	-2.20397	1.10833
H	0.02176	-1.28883	1.55477
C	3.47487	-0.89040	-0.17029
H	2.78344	1.02204	-0.82466
C	3.17089	-2.07619	0.50946
H	1.67829	-3.11695	1.64837
H	3.88786	-2.88471	0.58015
O	4.66270	-0.64387	-0.79778
C	5.65736	-1.66227	-0.76375
H	6.50901	-1.26721	-1.31909
H	5.96471	-1.88640	0.26480
H	5.30328	-2.58244	-1.24396
C	-1.09727	1.10124	0.18467
C	-2.00490	-0.09834	0.12178
H	-1.42565	-0.97865	-0.18309
H	-2.39431	-0.33151	1.12326
C	-3.18488	0.07407	-0.84835
H	-3.82896	0.89451	-0.50393
H	-2.79153	0.38168	-1.82590
C	-4.03271	-1.19300	-1.01988
H	-4.77789	-1.01578	-1.80818
H	-3.39308	-2.01145	-1.38243
C	-4.76146	-1.64900	0.24978
H	-4.03182	-1.89338	1.03223
H	-5.36096	-0.81379	0.63938
C	-5.66481	-2.86094	0.00731
H	-5.08407	-3.71656	-0.35900
H	-6.17572	-3.17218	0.92555
H	-6.43182	-2.63619	-0.74410
H	-2.51429	2.83606	0.01196

4e_conformer-150

C	1.16086	2.74202	-0.31704
C	0.04407	0.78644	-0.64294
N	-0.06302	2.12275	-0.28061
H	-0.94142	2.60213	-0.14269
C	2.08128	1.77553	-0.68203
C	1.32671	4.19478	-0.01235
H	2.38363	4.46848	-0.07014
H	0.77712	4.82890	-0.72032
H	0.96810	4.44516	0.99424
C	-1.13643	-0.07633	-0.65604
C	-1.24310	-1.15598	-1.54769
C	-2.20285	0.18006	0.23108
C	-2.38340	-1.96033	-1.54196
H	-0.44716	-1.35102	-2.25651
C	-3.34611	-0.62201	0.21697
H	-2.11009	0.98566	0.94874
C	-3.43787	-1.70333	-0.67188
H	-2.45559	-2.79274	-2.23645
H	-4.33151	-2.31883	-0.66152
O	-4.41899	-0.44085	1.04200
C	-4.37999	0.64559	1.96137
H	-4.28414	1.60604	1.44061
H	-5.32841	0.61654	2.49928
H	-3.55326	0.53685	2.67380
C	1.39612	0.54295	-0.89386
C	2.07284	-0.76475	-1.21426
H	1.72102	-1.16972	-2.17196
H	3.14111	-0.56364	-1.35771
C	1.90168	-1.85207	-0.13360
H	0.84948	-2.15672	-0.09787
H	2.46773	-2.73958	-0.44466
C	2.33204	-1.42091	1.27544
H	1.67030	-0.61339	1.61332
H	2.16923	-2.26399	1.96186
C	3.78875	-0.95091	1.40128
H	3.93238	-0.04027	0.80624
H	3.97066	-0.66047	2.44469
C	4.82147	-2.00276	0.98561
H	4.68804	-2.93148	1.55474
H	4.73841	-2.25305	-0.07802
H	5.84277	-1.64616	1.16165
H	3.14831	1.93093	-0.78159

4e_conformer-151

C	-1.16085	2.74190	-0.31723
C	-0.04414	0.78626	-0.64292
N	0.06300	2.12260	-0.28072
H	0.94139	2.60196	-0.14278
C	-2.08130	1.77542	-0.68218
C	-1.32667	4.19469	-0.01264
H	-0.96827	4.44509	0.99402
H	-0.77687	4.82873	-0.72052
H	-2.38356	4.46847	-0.07069
C	1.13632	-0.07656	-0.65587
C	1.24275	-1.15662	-1.54705
C	2.20294	0.18018	0.23091
C	2.38302	-1.96103	-1.54120
H	0.44666	-1.35196	-2.25561
C	3.34616	-0.62196	0.21692
H	2.11038	0.98608	0.94825
C	3.43769	-1.70368	-0.67147
H	2.45502	-2.79375	-2.23532
H	4.33131	-2.31921	-0.66102
O	4.41922	-0.44048	1.04165
C	4.38045	0.64636	1.96056
H	3.55384	0.53799	2.67318
H	5.32896	0.61747	2.49832
H	4.28458	1.60659	1.43940
C	-1.39619	0.54280	-0.89389
C	-2.07297	-0.76486	-1.21427
H	-3.14123	-0.56371	-1.35769
H	-1.72121	-1.16983	-2.17201
C	-1.90183	-1.85221	-0.13364
H	-2.46809	-2.73962	-0.44460
H	-0.84968	-2.15706	-0.09806
C	-2.33189	-1.42095	1.27546
H	-2.16925	-2.26409	1.96185
H	-1.66987	-0.61363	1.61327
C	-3.78844	-0.95051	1.40148
H	-3.97016	-0.66006	2.44492
H	-3.93185	-0.03980	0.80649
C	-4.82153	-2.00202	0.98587
H	-5.84270	-1.64511	1.16202
H	-4.73865	-2.25231	-0.07778
H	-4.68833	-2.93080	1.55496
H	-3.14832	1.93085	-0.78180

4e_conformer-152

C	-2.99849	-1.62295	-0.10065
C	-0.84872	-0.89623	-0.24076
N	-1.73697	-1.72033	0.43556
H	-1.45947	-2.39966	1.12987
C	-2.92612	-0.69030	-1.11885
C	-4.13814	-2.45023	0.39765
H	-3.96891	-3.52332	0.23824
H	-4.30930	-2.30433	1.47175
H	-5.05578	-2.17541	-0.12945
C	0.53861	-0.77246	0.21024
C	0.84102	-0.79090	1.58827
C	1.59070	-0.63705	-0.70348
C	2.15764	-0.66230	2.01623
H	0.03833	-0.87345	2.31405
C	2.91222	-0.49025	-0.26453
H	1.40390	-0.66487	-1.77090
C	3.20605	-0.50421	1.10444
H	2.38035	-0.67112	3.07962
H	4.22216	-0.39798	1.46349
O	3.84612	-0.35756	-1.25204
C	5.21024	-0.22645	-0.86541
H	5.36791	0.67103	-0.25527
H	5.55416	-1.10686	-0.30922
H	5.77697	-0.13869	-1.79324
C	-1.58006	-0.22424	-1.21747
C	-1.13000	0.91511	-2.08859
H	-0.04693	0.87914	-2.23956
H	-1.58372	0.80901	-3.08306
C	-1.48775	2.31312	-1.53627
H	-1.17502	3.05187	-2.28596
H	-2.57934	2.40410	-1.45054
C	-0.83253	2.64930	-0.18255
H	0.15696	2.17369	-0.13338
H	-0.65138	3.73195	-0.12732
C	-1.64991	2.24137	1.05042
H	-2.60461	2.78681	1.03836
H	-1.90116	1.17804	0.99401
C	-0.90975	2.52341	2.36002
H	0.02092	1.94567	2.40938
H	-0.64929	3.58590	2.44925
H	-1.51732	2.25388	3.23199
H	-3.75966	-0.36108	-1.72695

4e_conformer-153

C	0.15042	3.23226	-0.25501
C	0.37065	0.98502	0.01503
N	0.97743	2.15051	-0.43384
H	1.85146	2.17623	-0.93923
C	-1.00008	2.75891	0.34934
C	0.53961	4.60832	-0.68671
H	1.46632	4.94232	-0.20277
H	0.69933	4.66867	-1.77124
H	-0.25045	5.31752	-0.42561
C	1.07010	-0.29739	-0.04225
C	0.37488	-1.50604	-0.21329
C	2.47677	-0.33237	0.06697
C	1.07182	-2.71495	-0.26019
H	-0.70044	-1.49918	-0.34198
C	3.16404	-1.54577	-0.00025
H	3.01504	0.59168	0.23820
C	2.45813	-2.74810	-0.15913
H	0.52419	-3.64371	-0.39430
H	3.01089	-3.68095	-0.20218
O	4.52100	-1.66454	0.09450
C	5.28747	-0.47446	0.24748
H	5.03543	0.04637	1.17917
H	6.33032	-0.79237	0.28046
H	5.13934	0.20714	-0.59888
C	-0.87537	1.34846	0.52573
C	-1.89932	0.45865	1.17017
H	-1.41687	-0.44316	1.56653
H	-2.34071	0.97717	2.03226
C	-3.03612	0.04324	0.21849
H	-3.55295	0.94513	-0.13838
H	-2.60135	-0.42768	-0.67211
C	-4.04356	-0.90182	0.87920
H	-3.52008	-1.81157	1.20949
H	-4.43164	-0.42446	1.79000
C	-5.22269	-1.29779	-0.02065
H	-5.94125	-1.87786	0.57342
H	-5.75247	-0.38971	-0.34159
C	-4.81808	-2.11526	-1.25191
H	-4.17178	-1.54225	-1.92571
H	-4.27107	-3.01991	-0.95763
H	-5.69815	-2.42796	-1.82520
H	-1.84508	3.36586	0.64993

4e_conformer-154

C	2.60131	-1.88400	-0.03978

C	0.89106	-0.61670	0.75866
N	1.25029	-1.83924	0.20945
H	0.62646	-2.63074	0.13781
C	3.11287	-0.65157	0.32247
C	3.26892	-3.10861	-0.57462
H	3.19931	-3.95390	0.12270
H	2.82490	-3.43347	-1.52418
H	4.32918	-2.90776	-0.75046
C	-0.51460	-0.29759	1.01653
C	-0.90931	0.42791	2.15071
C	-1.49655	-0.72144	0.09884
C	-2.25595	0.74059	2.34613
H	-0.16868	0.72338	2.88531
C	-2.84223	-0.41142	0.30900
H	-1.18501	-1.25323	-0.79132
C	-3.22553	0.32823	1.43777
H	-2.55477	1.30321	3.22619
H	-4.27525	0.56382	1.58017
O	-3.85173	-0.77040	-0.53731
C	-3.51040	-1.50333	-1.70975
H	-3.03843	-2.46092	-1.45884
H	-4.45037	-1.68709	-2.23161
H	-2.83871	-0.92804	-2.35821
C	2.04761	0.15590	0.82511
C	2.16265	1.61450	1.16939
H	1.34739	1.91717	1.83383
H	3.09327	1.77729	1.72932
C	2.16027	2.56154	-0.05157
H	2.35502	3.57562	0.32187
H	3.00202	2.30943	-0.71129
C	0.84953	2.56526	-0.86215
H	0.00164	2.42690	-0.17674
H	0.71257	3.55662	-1.31675
C	0.76880	1.51774	-1.98053
H	1.58289	1.69953	-2.69685
H	0.93780	0.51889	-1.56782
C	-0.57660	1.54929	-2.70983
H	-0.76376	2.53307	-3.15892
H	-0.61788	0.80236	-3.51160
H	-1.39765	1.33864	-2.01429
H	4.14875	-0.35057	0.22582

4e_conformer-155

C	-2.60117	-1.88407	-0.03975
C	-0.89092	-0.61677	0.75870

N	-1.25014	-1.83928	0.20943
H	-0.62627	-2.63074	0.13767
C	-3.11275	-0.65168	0.32262
C	-3.26875	-3.10866	-0.57464
H	-3.19913	-3.95399	0.12263
H	-4.32902	-2.90783	-0.75047
H	-2.82473	-3.43347	-1.52422
C	0.51474	-0.29763	1.01654
C	0.90948	0.42776	2.15078
C	1.49666	-0.72135	0.09876
C	2.25612	0.74047	2.34618
H	0.16887	0.72313	2.88544
C	2.84234	-0.41130	0.30889
H	1.18510	-1.25305	-0.79144
C	3.22567	0.32823	1.43773
H	2.55496	1.30300	3.22628
H	4.27539	0.56385	1.58011
O	3.85182	-0.77015	-0.53750
C	3.51044	-1.50290	-1.71005
H	2.83870	-0.92753	-2.35838
H	4.45039	-1.68657	-2.23198
H	3.03850	-2.46055	-1.45926
C	-2.04749	0.15579	0.82526
C	-2.16257	1.61437	1.16964
H	-3.09310	1.77709	1.72973
H	-1.34720	1.91705	1.83394
C	-2.16044	2.56146	-0.05129
H	-3.00230	2.30934	-0.71087
H	-2.35515	3.57551	0.32221
C	-0.84984	2.56522	-0.86211
H	-0.71300	3.55660	-1.31673
H	-0.00183	2.42688	-0.17685
C	-0.76929	1.51771	-1.98051
H	-0.93822	0.51887	-1.56776
H	-1.58351	1.69949	-2.69668
C	0.57598	1.54927	-2.71005
H	1.39716	1.33864	-2.01466
H	0.61713	0.80235	-3.51183
H	0.76304	2.53306	-3.15917
H	-4.14864	-0.35070	0.22604

4e_conformer-156

C	1.11673	3.34578	-0.12062
C	0.54655	1.15379	-0.34234
N	1.59062	2.06422	-0.24486

H	2.56253	1.82754	-0.38412
C	-0.26428	3.25748	-0.10852
C	2.02877	4.52592	-0.03947
H	1.44330	5.44043	0.08783
H	2.72285	4.44906	0.80721
H	2.63520	4.63987	-0.94757
C	0.81408	-0.27974	-0.45114
C	-0.08492	-1.14816	-1.09276
C	2.00106	-0.81595	0.09251
C	0.19461	-2.51262	-1.17669
H	-0.98916	-0.75753	-1.54168
C	2.27814	-2.18068	-0.01239
H	2.68008	-0.16339	0.62702
C	1.36800	-3.03899	-0.64694
H	-0.50980	-3.17298	-1.67492
H	1.59822	-4.09752	-0.71001
O	3.40189	-2.77452	0.48707
C	4.36377	-1.94893	1.13521
H	5.17578	-2.61328	1.43354
H	4.75132	-1.17941	0.45662
H	3.94187	-1.46650	2.02523
C	-0.63808	1.88978	-0.25214
C	-2.05145	1.37399	-0.20411
H	-2.73620	2.23173	-0.20186
H	-2.29110	0.80979	-1.11277
C	-2.34196	0.50736	1.03597
H	-1.59291	-0.29127	1.10123
H	-2.20693	1.13197	1.92844
C	-3.74322	-0.11684	1.04638
H	-3.93026	-0.55334	2.03761
H	-4.50021	0.67016	0.91143
C	-3.94901	-1.20727	-0.01277
H	-3.81939	-0.78389	-1.01717
H	-3.16380	-1.96786	0.10099
C	-5.32613	-1.86912	0.07899
H	-6.12664	-1.13185	-0.05922
H	-5.45104	-2.64541	-0.68450
H	-5.47340	-2.33677	1.06034
H	-0.94579	4.09222	-0.00113

4e_conformer-157

C	-0.86971	3.43265	-0.36740
C	-1.07928	1.19104	-0.06458
N	-1.75317	2.39632	-0.20067
H	-2.75633	2.47928	-0.28201

C	0.40155	2.88832	-0.31071
C	-1.32520	4.84065	-0.57130
H	-0.45942	5.50189	-0.66354
H	-1.92886	4.94950	-1.48181
H	-1.93535	5.19909	0.26769
C	-1.80337	-0.05960	0.14504
C	-3.05559	-0.07246	0.78500
C	-1.25758	-1.28010	-0.30184
C	-3.73662	-1.27605	0.97133
H	-3.48172	0.85100	1.16481
C	-1.93863	-2.48063	-0.09033
H	-0.31859	-1.27086	-0.83629
C	-3.18895	-2.48204	0.54555
H	-4.70219	-1.27474	1.46944
H	-3.70416	-3.42554	0.69365
O	-1.47013	-3.70201	-0.48300
C	-0.18984	-3.75696	-1.10407
H	0.59050	-3.35649	-0.44544
H	0.00351	-4.81273	-1.29827
H	-0.18036	-3.20454	-2.05146
C	0.28584	1.47854	-0.12110
C	1.42231	0.50390	0.04539
H	1.10318	-0.32047	0.69694
H	1.66439	0.03678	-0.92026
C	2.69883	1.12743	0.62825
H	3.10104	1.86899	-0.07543
H	2.43807	1.67865	1.54093
C	3.78911	0.09835	0.95385
H	4.62711	0.61403	1.44355
H	3.40026	-0.62074	1.69008
C	4.32623	-0.66832	-0.26060
H	3.51779	-1.24875	-0.72332
H	4.66144	0.05143	-1.02113
C	5.47832	-1.60969	0.09990
H	6.32072	-1.05430	0.53035
H	5.16018	-2.35391	0.84047
H	5.84683	-2.14970	-0.77968
H	1.32075	3.45381	-0.38479

4e_conformer-158

C	0.86992	3.43261	-0.36740
C	1.07954	1.19102	-0.06458
N	1.75341	2.39633	-0.20055
H	2.75657	2.47936	-0.28176
C	-0.40133	2.88824	-0.31091

C	1.32539	4.84063	-0.57124
H	1.93513	5.19921	0.26798
H	1.92946	4.94941	-1.48149
H	0.45959	5.50178	-0.66397
C	1.80349	-0.05971	0.14506
C	3.05567	-0.07287	0.78508
C	1.25745	-1.28008	-0.30190
C	3.73642	-1.27662	0.97140
H	3.48200	0.85046	1.16498
C	1.93823	-2.48077	-0.09039
H	0.31851	-1.27060	-0.83644
C	3.18852	-2.48248	0.54555
H	4.70197	-1.27554	1.46956
H	3.70350	-3.42611	0.69364
O	1.46947	-3.70204	-0.48312
C	0.18919	-3.75669	-1.10424
H	-0.59108	-3.35596	-0.44567
H	0.17989	-3.20434	-2.05167
H	-0.00443	-4.81243	-1.29837
C	-0.28558	1.47847	-0.12132
C	-1.42208	0.50388	0.04525
H	-1.66442	0.03698	-0.92044
H	-1.10291	-0.32065	0.69657
C	-2.69839	1.12749	0.62848
H	-2.43737	1.67845	1.54125
H	-3.10056	1.86930	-0.07497
C	-3.78881	0.09854	0.95402
H	-3.40002	-0.62072	1.69013
H	-4.62670	0.61427	1.44385
C	-4.32612	-0.66790	-0.26050
H	-4.66123	0.05201	-1.02092
H	-3.51780	-1.24840	-0.72331
C	-5.47837	-1.60910	0.09995
H	-5.16033	-2.35345	0.84043
H	-6.32064	-1.05360	0.53048
H	-5.84701	-2.14894	-0.77967
H	-1.32055	3.45369	-0.38508

4e_conformer-159

C	0.05269	3.12802	-0.14697
C	-0.37351	0.90034	0.01706
N	-0.87511	2.13844	-0.36167
H	-1.75126	2.27085	-0.84635
C	1.16294	2.51958	0.40978
C	-0.21189	4.55451	-0.50265

H	0.64416	5.17324	-0.22038
H	-0.37905	4.68498	-1.57984
H	-1.09714	4.94751	0.01349
C	-1.19065	-0.30708	-0.08926
C	-0.61339	-1.56351	-0.33707
C	-2.59223	-0.21862	0.05000
C	-1.41980	-2.69958	-0.42856
H	0.45562	-1.64717	-0.48997
C	-3.38982	-1.35906	-0.06186
H	-3.03968	0.74051	0.27955
C	-2.80115	-2.61100	-0.29709
H	-0.96312	-3.66633	-0.62174
H	-3.43849	-3.48592	-0.37393
O	-4.74992	-1.35808	0.05859
C	-5.39989	-0.11271	0.29056
H	-6.46682	-0.33536	0.33271
H	-5.08249	0.33282	1.24115
H	-5.20594	0.59638	-0.52331
C	0.90899	1.11963	0.51931
C	1.85497	0.10844	1.10113
H	2.34516	0.53818	1.98556
H	1.29886	-0.76906	1.45265
C	2.94785	-0.34627	0.11658
H	2.47645	-0.75951	-0.78659
H	3.51103	0.53562	-0.21305
C	3.89209	-1.38879	0.72208
H	4.38758	-0.95857	1.60513
H	3.29347	-2.23468	1.08881
C	4.95755	-1.91619	-0.24943
H	5.50584	-2.73354	0.23747
H	4.45959	-2.35924	-1.12356
C	5.95705	-0.85279	-0.71592
H	6.45772	-0.38440	0.14083
H	6.73046	-1.29171	-1.35639
H	5.46657	-0.05780	-1.28817
H	2.06579	3.02923	0.72266

4e_conformer-160

C	1.12676	3.15408	-0.31844
C	-0.28360	1.38822	-0.08916
N	-0.16585	2.77070	-0.06225
H	-0.94811	3.40499	0.01356
C	1.86278	1.99455	-0.48521
C	1.53135	4.59059	-0.38481
H	1.00324	5.12975	-1.18210

H	1.32525	5.11790	0.55547
H	2.60370	4.66610	-0.58415
C	-1.56705	0.73913	0.16900
C	-2.51057	1.32906	1.03831
C	-1.89755	-0.47493	-0.44822
C	-3.73196	0.70839	1.26952
H	-2.26938	2.25477	1.55169
C	-3.12366	-1.10262	-0.19576
H	-1.22145	-0.93937	-1.15633
C	-4.05527	-0.51123	0.66527
H	-4.44911	1.16896	1.94339
H	-5.01153	-0.97734	0.86766
O	-3.32280	-2.28283	-0.85403
C	-4.56186	-2.95530	-0.65461
H	-5.40814	-2.33796	-0.97942
H	-4.51448	-3.85738	-1.26601
H	-4.70043	-3.23411	0.39692
C	0.98820	0.87453	-0.34387
C	1.38543	-0.57703	-0.39098
H	0.64679	-1.17423	0.15784
H	1.35642	-0.95174	-1.42644
C	2.77996	-0.85946	0.18610
H	3.52940	-0.29416	-0.38042
H	2.82631	-0.48500	1.21833
C	3.13005	-2.35059	0.16517
H	2.34322	-2.90600	0.69480
H	3.11314	-2.71174	-0.87398
C	4.48876	-2.68861	0.79511
H	4.49821	-2.34166	1.83811
H	4.59752	-3.78056	0.83474
C	5.68788	-2.09446	0.04895
H	5.69263	-2.41405	-1.00080
H	5.67203	-0.99915	0.06164
H	6.63304	-2.41619	0.50082
H	2.92727	1.95989	-0.67549

4e_conformer-161

C	-2.92412	-1.60170	-0.09018
C	-0.76099	-0.90166	-0.11460
N	-1.71157	-1.66001	0.55360
H	-1.49996	-2.27749	1.32466
C	-2.75752	-0.76019	-1.17460
C	-4.11103	-2.37626	0.38186
H	-3.94421	-3.46011	0.32705
H	-4.36526	-2.13887	1.42267

H	-4.97982	-2.14072	-0.23874
C	0.58563	-0.74508	0.43778
C	0.77566	-0.64911	1.82625
C	1.70857	-0.69069	-0.41009
C	2.06075	-0.48927	2.34897
H	-0.08287	-0.66525	2.48991
C	2.98711	-0.50948	0.12190
H	1.56651	-0.81725	-1.47464
C	3.16705	-0.41086	1.51028
H	2.19824	-0.41058	3.42378
H	4.17020	-0.27708	1.90198
O	4.12476	-0.43484	-0.62921
C	3.99455	-0.49881	-2.04573
H	3.35966	0.31145	-2.42435
H	5.00377	-0.38770	-2.44426
H	3.58013	-1.46222	-2.36645
C	-1.40210	-0.31204	-1.20229
C	-0.87076	0.74364	-2.13168
H	0.21919	0.68133	-2.20501
H	-1.25819	0.55856	-3.14246
C	-1.23749	2.18914	-1.72735
H	-0.86354	2.85455	-2.51668
H	-2.33049	2.30107	-1.72207
C	-0.66469	2.63650	-0.36846
H	0.31162	2.15653	-0.21314
H	-0.46958	3.71776	-0.39712
C	-1.56600	2.34900	0.83959
H	-2.51091	2.89724	0.71446
H	-1.82604	1.28655	0.86390
C	-0.90969	2.74546	2.16414
H	0.00831	2.16858	2.32767
H	-0.64263	3.81003	2.17310
H	-1.57641	2.56103	3.01479
H	-3.53443	-0.48012	-1.87526

4e_conformer-162

C	-2.93751	-1.60563	-0.49344
C	-0.79992	-0.83472	-0.37646
N	-1.68045	-1.83874	0.00452
H	-1.39975	-2.69054	0.46904
C	-2.87227	-0.40952	-1.18602
C	-4.06984	-2.55375	-0.27014
H	-3.88046	-3.53524	-0.72432
H	-4.25801	-2.72074	0.79803
H	-4.98459	-2.15165	-0.71383

C	0.59420	-0.86799	0.06103
C	0.95470	-1.50726	1.26760
C	1.60755	-0.27616	-0.70432
C	2.28341	-1.53945	1.67373
H	0.18856	-1.94455	1.89998
C	2.94119	-0.29986	-0.28109
H	1.38253	0.20103	-1.65037
C	3.29212	-0.93837	0.91448
H	2.54674	-2.02919	2.60715
H	4.31885	-0.97047	1.25710
O	3.82974	0.31816	-1.11443
C	5.20499	0.30887	-0.74598
H	5.59278	-0.71457	-0.67587
H	5.73067	0.84300	-1.53855
H	5.36622	0.82273	0.20935
C	-1.53819	0.08860	-1.11912
C	-1.09462	1.41575	-1.67740
H	-0.30635	1.27292	-2.42849
H	-1.94124	1.85330	-2.22255
C	-0.59463	2.44864	-0.64572
H	0.22969	2.01825	-0.06650
H	-0.16335	3.28825	-1.20729
C	-1.66948	3.01301	0.29853
H	-1.26350	3.91914	0.77155
H	-2.52653	3.34500	-0.30539
C	-2.17572	2.08234	1.41357
H	-2.96329	2.61484	1.96515
H	-2.64740	1.19691	0.97629
C	-1.08739	1.64684	2.39899
H	-1.51274	1.05106	3.21532
H	-0.32262	1.03473	1.91177
H	-0.58805	2.51649	2.84591
H	-3.70456	0.07714	-1.67912

4e_conformer-163

C	-2.79859	-1.76139	-0.07387
C	-0.67388	-1.02309	0.26031
N	-1.47007	-2.00023	-0.32392
H	-1.12322	-2.70977	-0.95339
C	-2.85793	-0.62686	0.71441
C	-3.87926	-2.63446	-0.62265
H	-3.89464	-2.62645	-1.72038
H	-3.76190	-3.67887	-0.30672
H	-4.85412	-2.28443	-0.27271
C	0.78287	-1.08773	0.13766

C	1.43777	-2.33752	0.08306
C	1.56065	0.07429	0.05042
C	2.81956	-2.39902	-0.05154
H	0.86243	-3.25339	0.17615
C	2.95413	0.00392	-0.06742
H	1.09884	1.05298	0.04293
C	3.59658	-1.23818	-0.12366
H	3.31261	-3.36651	-0.08877
H	4.67237	-1.31329	-0.22229
O	3.59490	1.20810	-0.13876
C	5.01086	1.20068	-0.28771
H	5.31280	2.24798	-0.32974
H	5.49979	0.71405	0.56484
H	5.31229	0.69611	-1.21364
C	-1.52940	-0.14864	0.92801
C	-1.17391	1.02833	1.79482
H	-1.58668	0.85370	2.79878
H	-0.08795	1.09096	1.92363
C	-1.71171	2.39204	1.31112
H	-2.79138	2.30872	1.13339
H	-1.59685	3.10389	2.13931
C	-1.02279	2.99605	0.07663
H	-1.35763	4.03908	-0.01955
H	0.05894	3.04789	0.26832
C	-1.26212	2.29517	-1.27115
H	-0.69223	2.83716	-2.03840
H	-0.85699	1.27871	-1.24483
C	-2.73447	2.23697	-1.68758
H	-3.18878	3.23620	-1.67963
H	-2.83919	1.83045	-2.70030
H	-3.31231	1.59582	-1.01453
H	-3.76511	-0.18754	1.10989

4e_conformer-164

C	2.14400	2.88644	-0.28263
C	0.38144	1.45327	-0.14706
N	0.80440	2.76893	-0.01208
H	0.18320	3.55043	0.14058
C	2.60155	1.61322	-0.57320
C	2.84984	4.20239	-0.24808
H	2.44542	4.90516	-0.98829
H	2.76988	4.68406	0.73485
H	3.91151	4.06080	-0.46740
C	-1.00969	1.09067	0.11880
C	-1.80019	1.86347	0.99788

C	-1.59902	-0.02374	-0.49384
C	-3.12454	1.51757	1.23815
H	-1.36624	2.71241	1.51690
C	-2.92849	-0.37454	-0.23439
H	-1.04303	-0.63190	-1.19649
C	-3.70761	0.39947	0.63376
H	-3.71958	2.11844	1.92036
H	-4.73935	0.14697	0.84432
O	-3.37742	-1.48574	-0.89026
C	-4.72832	-1.88275	-0.67958
H	-5.42885	-1.10153	-0.99855
H	-4.87669	-2.77465	-1.28977
H	-4.91360	-2.12627	0.37355
C	1.50740	0.70320	-0.49782
C	1.62569	-0.78715	-0.67463
H	0.96072	-1.15058	-1.46999
H	2.64234	-1.00575	-1.02120
C	1.34448	-1.58204	0.61330
H	2.08014	-1.28944	1.37316
H	0.36526	-1.28664	1.00698
C	1.35566	-3.10405	0.41534
H	1.12741	-3.58453	1.37728
H	0.53756	-3.37671	-0.26662
C	2.66928	-3.68664	-0.12922
H	2.54995	-4.77299	-0.23499
H	2.85355	-3.30434	-1.14173
C	3.88754	-3.39730	0.75279
H	3.72375	-3.75244	1.77801
H	4.78359	-3.89483	0.36488
H	4.10241	-2.32403	0.80538
H	3.62531	1.35290	-0.81192

4e_conformer-165

C	-0.07063	3.35371	-0.15138
C	-0.24767	1.08825	-0.09198
N	-0.91021	2.28268	-0.33890
H	-1.83989	2.34467	-0.72876
C	1.14701	2.83947	0.25514
C	-0.51507	4.76039	-0.38626
H	-1.37621	5.02739	0.23959
H	0.29856	5.45184	-0.15126
H	-0.80766	4.92959	-1.43082
C	-0.93914	-0.19495	-0.20067
C	-0.26426	-1.34829	-0.63057
C	-2.30939	-0.28738	0.12118

C	-0.94002	-2.56665	-0.71625
H	0.77385	-1.28113	-0.93087
C	-2.98038	-1.50746	0.01353
H	-2.82545	0.59264	0.48506
C	-2.29112	-2.65786	-0.40054
H	-0.40739	-3.45303	-1.04963
H	-2.83052	-3.59692	-0.46997
O	-4.30266	-1.68353	0.30426
C	-5.05285	-0.54762	0.72174
H	-4.66233	-0.13321	1.65909
H	-5.05313	0.23470	-0.04685
H	-6.07179	-0.90348	0.87916
C	1.05011	1.41568	0.29798
C	2.14351	0.48513	0.74204
H	2.64142	0.91223	1.62416
H	1.71075	-0.46815	1.06424
C	3.21384	0.23361	-0.33690
H	2.75568	-0.28083	-1.19196
H	3.55564	1.20326	-0.72140
C	4.43137	-0.55306	0.16983
H	5.15847	-0.64320	-0.64955
H	4.92505	0.03564	0.95595
C	4.13286	-1.95629	0.72058
H	5.07112	-2.38493	1.09679
H	3.46658	-1.88114	1.58981
C	3.52350	-2.91462	-0.30700
H	4.15662	-2.98869	-1.19999
H	3.41272	-3.92178	0.11033
H	2.53110	-2.58483	-0.63161
H	2.01959	3.42645	0.51379

4e_conformer-166

C	-0.63505	3.23433	-0.38764
C	0.57887	1.33954	-0.07678
N	0.61208	2.72595	-0.12518
H	1.45945	3.27425	-0.08996
C	-1.49554	2.15489	-0.48315
C	-0.88253	4.70071	-0.52881
H	-1.94481	4.88246	-0.71285
H	-0.59771	5.25295	0.37591
H	-0.31843	5.13408	-1.36504
C	1.78668	0.56748	0.20416
C	2.81453	1.10103	1.00175
C	1.94585	-0.72621	-0.33261
C	3.96665	0.35532	1.25404

H	2.70014	2.08330	1.44958
C	3.09407	-1.47138	-0.05522
H	1.18109	-1.12078	-0.98652
C	4.11507	-0.92833	0.73877
H	4.75362	0.77614	1.87373
H	5.00186	-1.52207	0.93539
O	3.31794	-2.73293	-0.52798
C	2.29976	-3.34059	-1.31644
H	2.66255	-4.34145	-1.55397
H	1.35666	-3.41466	-0.76148
H	2.12854	-2.78494	-2.24637
C	-0.74603	0.95529	-0.29021
C	-1.29933	-0.44538	-0.25855
H	-1.26712	-0.89558	-1.26357
H	-0.65521	-1.07748	0.36566
C	-2.73965	-0.53806	0.26347
H	-2.79248	-0.08550	1.26353
H	-3.39892	0.05702	-0.37950
C	-3.24374	-1.98323	0.32694
H	-3.21808	-2.41998	-0.68260
H	-2.54436	-2.57515	0.93408
C	-4.65740	-2.13402	0.90642
H	-4.88175	-3.20339	1.01525
H	-4.67736	-1.71190	1.92116
C	-5.75274	-1.47809	0.05930
H	-5.74290	-1.87127	-0.96512
H	-6.74587	-1.66921	0.48140
H	-5.62358	-0.39196	-0.00152
H	-2.56036	2.22715	-0.65985

4e_conformer-167

C	0.64370	3.26678	-0.42543
C	-0.53816	1.35533	-0.09382
N	-0.57233	2.74220	-0.06597
H	-1.41055	3.28561	0.08235
C	1.48722	2.19602	-0.66278
C	0.87943	4.73928	-0.51325
H	0.23005	5.21556	-1.25941
H	0.69535	5.24133	0.44526
H	1.91637	4.93342	-0.80030
C	-1.71447	0.56663	0.26471
C	-2.64913	1.04907	1.19781
C	-1.93720	-0.69061	-0.33244
C	-3.77306	0.28859	1.52296
H	-2.48181	2.00165	1.69098

C	-3.05486	-1.45191	0.01682
H	-1.24759	-1.04232	-1.08685
C	-3.98301	-0.96000	0.94659
H	-4.48791	0.66924	2.24721
H	-4.84819	-1.56495	1.19772
O	-3.33623	-2.68066	-0.50855
C	-2.41232	-3.23629	-1.43879
H	-2.33666	-2.62111	-2.34359
H	-1.41617	-3.34728	-0.99354
H	-2.80605	-4.21919	-1.70076
C	0.75745	0.98602	-0.45830
C	1.30896	-0.41226	-0.55270
H	0.71280	-1.08206	0.07968
H	1.20128	-0.80215	-1.57738
C	2.78379	-0.53059	-0.14323
H	3.40456	0.08581	-0.80768
H	2.90582	-0.11597	0.86503
C	3.28644	-1.97690	-0.18933
H	2.69926	-2.58628	0.51372
H	3.08879	-2.38808	-1.18942
C	4.77980	-2.13638	0.12869
H	5.06596	-3.18380	-0.03495
H	5.36568	-1.54304	-0.58761
C	5.15938	-1.73204	1.55694
H	6.22074	-1.92247	1.75258
H	4.97552	-0.66766	1.73959
H	4.57590	-2.30037	2.29223
H	2.52923	2.27949	-0.94145

4e_conformer-168

C	0.63512	3.23431	-0.38775
C	-0.57878	1.33954	-0.07674
N	-0.61199	2.72595	-0.12512
H	-1.45934	3.27425	-0.08980
C	1.49559	2.15488	-0.48337
C	0.88259	4.70069	-0.52894
H	1.94485	4.88244	-0.71314
H	0.31838	5.13408	-1.36509
H	0.59792	5.25293	0.37583
C	-1.78658	0.56749	0.20428
C	-2.81426	1.10097	1.00213
C	-1.94592	-0.72610	-0.33268
C	-3.96638	0.35528	1.25450
H	-2.69973	2.08316	1.45010
C	-3.09413	-1.47126	-0.05520

H	-1.18129	-1.12058	-0.98681
C	-4.11496	-0.92828	0.73906
H	-4.75322	0.77604	1.87439
H	-5.00174	-1.52200	0.93573
O	-3.31815	-2.73272	-0.52813
C	-2.30013	-3.34033	-1.31684
H	-2.66302	-4.34113	-1.55446
H	-2.12902	-2.78455	-2.24671
H	-1.35695	-3.41455	-0.76203
C	0.74609	0.95527	-0.29034
C	1.29935	-0.44541	-0.25880
H	0.65513	-1.07756	0.36526
H	1.26728	-0.89549	-1.26387
C	2.73960	-0.53819	0.26344
H	3.39899	0.05700	-0.37931
H	2.79226	-0.08579	1.26358
C	3.24367	-1.98337	0.32674
H	2.54418	-2.57539	0.93363
H	3.21821	-2.41993	-0.68289
C	4.65722	-2.13427	0.90647
H	4.67697	-1.71237	1.92130
H	4.88155	-3.20366	1.01511
C	5.75272	-1.47814	0.05971
H	5.62357	-0.39200	-0.00089
H	6.74577	-1.66935	0.48197
H	5.74308	-1.87110	-0.96480
H	2.56039	2.22713	-0.66022

4e_conformer-169

C	-3.54475	-0.93459	-0.29896
C	-1.30817	-0.87176	0.10273
N	-2.36252	-1.62801	-0.39016
H	-2.24865	-2.51015	-0.86884
C	-3.25023	0.27763	0.29580
C	-4.83497	-1.49696	-0.79961
H	-5.65084	-0.79968	-0.59095
H	-4.81342	-1.67288	-1.88313
H	-5.07748	-2.45375	-0.31992
C	0.04692	-1.42557	0.13376
C	0.25731	-2.79399	0.40688
C	1.16013	-0.61750	-0.12230
C	1.54790	-3.31048	0.42730
H	-0.58882	-3.43677	0.62948
C	2.45813	-1.13958	-0.07917
H	1.03493	0.42261	-0.38969

C	2.66203	-2.49723	0.19361
H	1.70119	-4.36437	0.64259
H	3.65726	-2.92320	0.22379
O	3.45646	-0.24346	-0.33638
C	4.79759	-0.72165	-0.33139
H	5.07488	-1.12291	0.65084
H	4.94835	-1.49553	-1.09372
H	5.42377	0.14148	-0.56083
C	-1.84549	0.33197	0.55547
C	-1.15582	1.45628	1.28154
H	-1.62796	1.58226	2.26595
H	-0.11084	1.19508	1.47997
C	-1.23446	2.80849	0.54072
H	-2.28309	2.99594	0.27796
H	-0.94595	3.61093	1.23149
C	-0.37062	2.87702	-0.72784
H	-0.51169	1.94749	-1.29573
H	-0.73399	3.68634	-1.37584
C	1.12735	3.10715	-0.46242
H	1.48934	2.39950	0.29476
H	1.69160	2.88301	-1.37731
C	1.45539	4.53393	-0.01087
H	0.94548	4.79039	0.92463
H	2.53136	4.65889	0.15548
H	1.14511	5.26428	-0.76832
H	-3.97409	1.04568	0.53802

4e_conformer-170

C	0.15538	3.19467	-0.10220
C	0.43063	0.93731	-0.12049
N	1.05435	2.16945	-0.26516
H	2.00863	2.28895	-0.57325
C	-1.06601	2.61204	0.18391
C	0.55237	4.62780	-0.24382
H	-0.30839	5.27329	-0.04955
H	1.34705	4.90394	0.46103
H	0.92017	4.85409	-1.25318
C	1.19120	-0.30712	-0.22046
C	0.60438	-1.48901	-0.70229
C	2.54871	-0.33075	0.16481
C	1.35613	-2.66285	-0.78208
H	-0.42364	-1.48320	-1.04324
C	3.29518	-1.50618	0.06301
H	2.99619	0.56661	0.57379
C	2.69519	-2.68390	-0.40829

H	0.89262	-3.57101	-1.15742
H	3.29120	-3.58841	-0.47326
O	4.61090	-1.61139	0.41262
C	5.27132	-0.44430	0.89114
H	6.30150	-0.74331	1.08868
H	5.26012	0.35629	0.14161
H	4.81388	-0.07782	1.81814
C	-0.90783	1.19386	0.17620
C	-1.99307	0.19779	0.46960
H	-1.55476	-0.74852	0.81006
H	-2.60460	0.57189	1.29973
C	-2.90550	-0.07829	-0.74147
H	-3.40579	0.85663	-1.02998
H	-2.27954	-0.36217	-1.59781
C	-3.95296	-1.17169	-0.49740
H	-4.47976	-1.37520	-1.44038
H	-3.44222	-2.10739	-0.22472
C	-4.98851	-0.83088	0.58053
H	-4.48793	-0.68375	1.54597
H	-5.46313	0.12904	0.33156
C	-6.06202	-1.91207	0.72918
H	-5.61397	-2.87533	1.00281
H	-6.79178	-1.65068	1.50391
H	-6.60797	-2.05678	-0.21125
H	-1.98292	3.14916	0.39261

4e_conformer-171

C	-0.94950	3.21779	-0.20888
C	-0.10099	1.12385	0.04376
N	0.14540	2.41276	-0.40874
H	0.96616	2.67815	-0.93442
C	-1.90621	2.44043	0.41573
C	-0.97445	4.64631	-0.64523
H	-0.16497	5.22831	-0.18649
H	-0.86921	4.74507	-1.73359
H	-1.92381	5.10656	-0.35846
C	0.94259	0.09968	-0.04087
C	0.63343	-1.24010	-0.34813
C	2.28554	0.44609	0.16349
C	1.64547	-2.19002	-0.42486
H	-0.39037	-1.51893	-0.55852
C	3.30164	-0.51259	0.06739
H	2.56312	1.46174	0.42788
C	2.98553	-1.84555	-0.22143
H	1.39629	-3.22008	-0.66467

H	3.75524	-2.60389	-0.29389
O	4.56757	-0.04989	0.28682
C	5.63929	-0.98517	0.21965
H	5.52609	-1.77664	0.97030
H	5.71163	-1.43885	-0.77610
H	6.54645	-0.41580	0.42603
C	-1.38642	1.11902	0.58213
C	-2.09760	0.00780	1.30747
H	-1.44249	-0.86503	1.39927
H	-2.30987	0.33486	2.33510
C	-3.43506	-0.39617	0.65122
H	-4.00396	-1.01833	1.35371
H	-4.03135	0.51199	0.49827
C	-3.27512	-1.13250	-0.68746
H	-4.21380	-1.07051	-1.25493
H	-2.52142	-0.60458	-1.28705
C	-2.88965	-2.61550	-0.55184
H	-2.55166	-2.98759	-1.52805
H	-2.03189	-2.71973	0.12570
C	-4.03416	-3.50176	-0.04976
H	-4.89391	-3.45085	-0.72920
H	-3.72224	-4.55006	0.01936
H	-4.37893	-3.19451	0.94400
H	-2.87945	2.78884	0.73804

4e_conformer-172

C	-2.99845	-1.62294	-0.10077
C	-0.84868	-0.89621	-0.24084
N	-1.73693	-1.72035	0.43544
H	-1.45941	-2.39978	1.12963
C	-2.92609	-0.69023	-1.11892
C	-4.13808	-2.45027	0.39748
H	-3.96887	-3.52335	0.23789
H	-4.30916	-2.30454	1.47162
H	-5.05576	-2.17537	-0.12951
C	0.53865	-0.77247	0.21017
C	0.84107	-0.79107	1.58820
C	1.59075	-0.63694	-0.70353
C	2.15769	-0.66251	2.01617
H	0.03838	-0.87369	2.31397
C	2.91226	-0.49017	-0.26457
H	1.40396	-0.66465	-1.77095
C	3.20610	-0.50430	1.10440
H	2.38040	-0.67147	3.07956
H	4.22221	-0.39810	1.46346

O	3.84615	-0.35733	-1.25206
C	5.21028	-0.22624	-0.86541
H	5.55420	-1.10671	-0.30933
H	5.77700	-0.13835	-1.79323
H	5.36793	0.67117	-0.25516
C	-1.58003	-0.22417	-1.21751
C	-1.13001	0.91527	-2.08854
H	-0.04694	0.87938	-2.23950
H	-1.58373	0.80924	-3.08302
C	-1.48788	2.31320	-1.53607
H	-1.17528	3.05207	-2.28570
H	-2.57948	2.40402	-1.45028
C	-0.83264	2.64928	-0.18234
H	0.15680	2.17358	-0.13316
H	-0.65140	3.73191	-0.12705
C	-1.65008	2.24134	1.05059
H	-2.60466	2.78699	1.03864
H	-1.90157	1.17807	0.99401
C	-0.90983	2.52302	2.36021
H	-0.64916	3.58544	2.44960
H	-1.51744	2.25349	3.23216
H	0.02073	1.94508	2.40946
H	-3.75963	-0.36097	-1.72699

4e_conformer-173

C	-1.13865	2.78160	-0.27224
C	-0.15519	0.79293	0.22764
N	-0.03066	1.99290	-0.46157
H	0.70998	2.19218	-1.11874
C	-1.97816	2.09112	0.58195
C	-1.28869	4.11191	-0.93485
H	-0.46982	4.79420	-0.67335
H	-1.30292	4.02564	-2.02924
H	-2.22742	4.57851	-0.62443
C	0.91465	-0.20431	0.17599
C	0.64077	-1.58150	0.18504
C	2.25715	0.22148	0.09046
C	1.68666	-2.50460	0.12387
H	-0.38391	-1.92844	0.20831
C	3.29373	-0.71085	0.01234
H	2.47488	1.28202	0.11804
C	3.00905	-2.08478	0.03467
H	1.46324	-3.56785	0.12957
H	3.82958	-2.79279	-0.02096
O	4.61598	-0.38181	-0.07341

C	4.96067	0.99934	-0.10542
H	6.04734	1.03452	-0.19234
H	4.50815	1.50371	-0.96777
H	4.65156	1.50970	0.81479
C	-1.37396	0.83748	0.90228
C	-1.94866	-0.16805	1.86205
H	-1.21924	-0.96052	2.06254
H	-2.12101	0.33433	2.82456
C	-3.28876	-0.80456	1.43219
H	-3.71436	-1.30279	2.31328
H	-3.99431	-0.00698	1.16105
C	-3.21559	-1.83020	0.29125
H	-2.45214	-2.58473	0.53459
H	-4.16872	-2.37744	0.25683
C	-2.94239	-1.25960	-1.10604
H	-3.71331	-0.51264	-1.34197
H	-1.98941	-0.72209	-1.11289
C	-2.92515	-2.34654	-2.18354
H	-3.87731	-2.89100	-2.21520
H	-2.74753	-1.92317	-3.17878
H	-2.13219	-3.07913	-1.98677
H	-2.92920	2.45370	0.95139

4e_conformer-174

C	-0.25355	3.40716	0.14667
C	-0.33606	1.20764	-0.43219
N	-1.05684	2.30109	0.02977
H	-2.06148	2.31333	0.13300
C	1.02028	3.01088	-0.22058
C	-0.78453	4.73287	0.58450
H	0.02921	5.46133	0.63426
H	-1.54089	5.12223	-0.10953
H	-1.25064	4.67965	1.57668
C	-0.98629	-0.09005	-0.60955
C	-0.52498	-1.01861	-1.55734
C	-2.10971	-0.42562	0.17603
C	-1.16736	-2.24831	-1.70624
H	0.31851	-0.77204	-2.19036
C	-2.75426	-1.65284	0.00690
H	-2.44634	0.26421	0.93979
C	-2.27956	-2.57496	-0.93759
H	-0.80105	-2.95767	-2.44319
H	-2.78996	-3.52618	-1.04785
O	-3.84474	-2.05081	0.72613
C	-4.37431	-1.14992	1.69304

H	-5.23921	-1.65237	2.12801
H	-3.64317	-0.93460	2.48169
H	-4.69310	-0.20866	1.22907
C	0.98476	1.63512	-0.59179
C	2.19341	0.82118	-0.97092
H	2.07647	0.37294	-1.96670
H	3.05265	1.49911	-1.05348
C	2.53321	-0.28874	0.03868
H	2.69330	0.17254	1.02097
H	1.66961	-0.95719	0.14617
C	3.75863	-1.10878	-0.37497
H	3.59296	-1.50178	-1.38823
H	4.63509	-0.44704	-0.44243
C	4.07854	-2.27921	0.56565
H	3.20806	-2.94878	0.61430
H	4.89559	-2.87003	0.13062
C	4.47182	-1.85041	1.98297
H	3.65125	-1.33361	2.49250
H	4.74822	-2.71620	2.59541
H	5.33078	-1.16786	1.96077
H	1.89952	3.64312	-0.21864

4e_conformer-175

C	0.25354	3.40714	0.14667
C	0.33605	1.20762	-0.43221
N	1.05683	2.30107	0.02976
H	2.06147	2.31331	0.13299
C	-1.02029	3.01086	-0.22058
C	0.78452	4.73285	0.58451
H	-0.02922	5.46130	0.63429
H	1.25065	4.67961	1.57668
H	1.54087	5.12221	-0.10953
C	0.98629	-0.09006	-0.60957
C	0.52502	-1.01862	-1.55737
C	2.10970	-0.42561	0.17603
C	1.16742	-2.24831	-1.70627
H	-0.31846	-0.77206	-2.19042
C	2.75428	-1.65282	0.00691
H	2.44631	0.26422	0.93980
C	2.27962	-2.57494	-0.93759
H	0.80115	-2.95767	-2.44322
H	2.79004	-3.52615	-1.04785
O	3.84475	-2.05077	0.72616
C	4.37427	-1.14987	1.69309
H	5.23918	-1.65231	2.12807

H	4.69306	-0.20860	1.22913
H	3.64312	-0.93458	2.48172
C	-0.98477	1.63511	-0.59181
C	-2.19343	0.82118	-0.97093
H	-3.05266	1.49911	-1.05348
H	-2.07650	0.37293	-1.96671
C	-2.53323	-0.28874	0.03868
H	-1.66964	-0.95720	0.14615
H	-2.69330	0.17253	1.02097
C	-3.75866	-1.10877	-0.37496
H	-4.63512	-0.44703	-0.44240
H	-3.59302	-1.50176	-1.38823
C	-4.07856	-2.27921	0.56566
H	-4.89562	-2.87001	0.13063
H	-3.20809	-2.94878	0.61428
C	-4.47182	-1.85041	1.98299
H	-5.33076	-1.16786	1.96081
H	-4.74821	-2.71620	2.59543
H	-3.65123	-1.33362	2.49251
H	-1.89953	3.64310	-0.21863

4e_conformer-176

C	-1.30376	2.86261	-0.22349
C	-0.20062	0.89472	0.06636
N	-0.12420	2.19119	-0.42615
H	0.64677	2.53974	-0.97782
C	-2.14656	1.99374	0.44438
C	-1.51450	4.26092	-0.70494
H	-0.76955	4.95227	-0.29088
H	-1.45184	4.33257	-1.79870
H	-2.50464	4.61319	-0.40347
C	0.97462	0.02040	0.00322
C	0.86681	-1.37068	-0.20144
C	2.25748	0.57444	0.12622
C	2.00968	-2.15918	-0.26471
H	-0.10591	-1.82524	-0.33932
C	3.40498	-0.22371	0.04513
H	2.38798	1.63520	0.31557
C	3.28821	-1.60512	-0.14601
H	1.91237	-3.22920	-0.42634
H	4.16174	-2.24239	-0.20500
O	4.59119	0.43948	0.17900
C	5.79101	-0.32557	0.12344
H	5.89818	-0.83061	-0.84406
H	6.60643	0.38714	0.25329

H	5.82645	-1.07149	0.92657
C	-1.46817	0.75029	0.63113
C	-2.01126	-0.42333	1.40294
H	-1.40308	-1.31137	1.21414
H	-1.91410	-0.22795	2.48146
C	-3.49324	-0.73923	1.12098
H	-3.76620	-1.63002	1.70078
H	-4.11452	0.07583	1.51452
C	-3.85432	-0.94734	-0.35743
H	-4.91749	-1.22023	-0.41640
H	-3.74852	0.00761	-0.88639
C	-3.02481	-2.00674	-1.09613
H	-3.39554	-2.08129	-2.12707
H	-1.98768	-1.65918	-1.17841
C	-3.05486	-3.39465	-0.44998
H	-4.08521	-3.75725	-0.34603
H	-2.50261	-4.12384	-1.05360
H	-2.60607	-3.38633	0.54971
H	-3.15129	2.22927	0.77056

4e_conformer-177

C	2.27162	2.19329	-0.06284
C	0.59366	0.77655	-0.64658
N	0.91055	2.00932	-0.09333
H	0.22920	2.72177	0.12700
C	2.84091	1.04168	-0.57409
C	2.89038	3.45690	0.43882
H	2.60081	4.32591	-0.16650
H	2.59844	3.67075	1.47492
H	3.98023	3.37581	0.40683
C	-0.79839	0.33666	-0.74679
C	-1.25666	-0.40235	-1.85643
C	-1.71007	0.65336	0.27002
C	-2.58229	-0.81673	-1.91756
H	-0.57933	-0.61899	-2.67462
C	-3.04779	0.24690	0.19301
H	-1.38846	1.19739	1.15260
C	-3.49254	-0.50092	-0.90368
H	-2.92795	-1.38443	-2.77704
H	-4.52095	-0.83159	-0.97957
O	-3.83545	0.61709	1.24509
C	-5.19993	0.20963	1.23090
H	-5.28992	-0.88325	1.21858
H	-5.63810	0.59996	2.15034
H	-5.73227	0.62612	0.36734

C	1.79564	0.13841	-0.94021
C	2.00663	-1.26461	-1.44256
H	1.04486	-1.73070	-1.68094
H	2.56612	-1.22202	-2.38762
C	2.77612	-2.18833	-0.47008
H	3.24207	-2.99050	-1.05746
H	3.60121	-1.62876	-0.01264
C	1.91633	-2.85430	0.61684
H	1.14870	-3.46560	0.12058
H	2.55394	-3.55842	1.17147
C	1.22385	-1.92633	1.62676
H	0.50333	-1.28516	1.10913
H	0.63904	-2.55355	2.31405
C	2.18579	-1.05530	2.43901
H	2.71224	-0.34165	1.79780
H	1.64603	-0.48121	3.20149
H	2.93657	-1.66939	2.95335
H	3.90435	0.85834	-0.66371

4e_conformer-178

C	-0.73743	3.06735	0.13366
C	0.16548	0.98489	0.22635
N	0.42664	2.34789	0.23694
H	1.33469	2.74989	0.42087
C	-1.76711	2.14968	0.02489
C	-0.75290	4.56092	0.15660
H	-0.36272	4.96150	1.10139
H	-0.14705	4.98890	-0.65236
H	-1.77719	4.92424	0.03751
C	1.25011	0.00958	0.31436
C	1.06066	-1.23999	0.94140
C	2.51578	0.30575	-0.21318
C	2.10928	-2.14995	1.01393
H	0.10504	-1.47681	1.39398
C	3.57068	-0.60931	-0.12074
H	2.69826	1.24212	-0.73130
C	3.37177	-1.85294	0.49108
H	1.95370	-3.10860	1.50117
H	4.17239	-2.57813	0.56698
O	4.75195	-0.20069	-0.67135
C	5.85478	-1.10024	-0.62448
H	6.13384	-1.33678	0.40936
H	5.63422	-2.03076	-1.16130
H	6.68182	-0.58525	-1.11495
C	-1.21380	0.83447	0.08157

C	-1.97544	-0.45706	-0.05579
H	-1.29682	-1.24713	-0.40087
H	-2.33745	-0.78813	0.92866
C	-3.16634	-0.36988	-1.02210
H	-3.89649	0.35469	-0.64106
H	-2.81422	0.03129	-1.98116
C	-3.85636	-1.71897	-1.26981
H	-4.67082	-1.56772	-1.99221
H	-3.14058	-2.40063	-1.75060
C	-4.42812	-2.40480	-0.01873
H	-4.88973	-3.35292	-0.32459
H	-3.61169	-2.67424	0.66389
C	-5.46185	-1.56110	0.73327
H	-5.01639	-0.64868	1.14547
H	-6.28082	-1.25843	0.06867
H	-5.89736	-2.12169	1.56807
H	-2.81181	2.40318	-0.09716

4e_conformer-179

C	0.73764	3.06728	0.13360
C	-0.16543	0.98490	0.22651
N	-0.42650	2.34793	0.23686
H	-1.33454	2.75000	0.42065
C	1.76726	2.14952	0.02508
C	0.75321	4.56086	0.15631
H	0.14747	4.98876	-0.65277
H	0.36297	4.96161	1.10101
H	1.77754	4.92409	0.03726
C	-1.25015	0.00969	0.31462
C	-1.06097	-1.23962	0.94225
C	-2.51565	0.30573	-0.21341
C	-2.10967	-2.14948	1.01487
H	-0.10549	-1.47630	1.39521
C	-3.57063	-0.60922	-0.12088
H	-2.69790	1.24190	-0.73196
C	-3.37199	-1.85261	0.49153
H	-1.95430	-3.10793	1.50256
H	-4.17268	-2.57771	0.56751
O	-4.75173	-0.20076	-0.67200
C	-5.85463	-1.10021	-0.62505
H	-6.13400	-1.33630	0.40880
H	-6.68149	-0.58537	-1.11598
H	-5.63397	-2.03098	-1.16142
C	1.21385	0.83436	0.08191
C	1.97541	-0.45724	-0.05524

H	2.33807	-0.78776	0.92916
H	1.29660	-1.24750	-0.39947
C	3.16569	-0.37053	-1.02237
H	2.81291	0.03004	-1.98144
H	3.89602	0.35434	-0.64224
C	3.85568	-1.71970	-1.26973
H	3.13966	-2.40171	-1.74967
H	4.66967	-1.56880	-1.99274
C	4.42830	-2.40475	-0.01861
H	3.61232	-2.67386	0.66468
H	4.88980	-3.35300	-0.32421
C	5.46243	-1.56051	0.73223
H	6.28096	-1.25817	0.06696
H	5.01715	-0.64789	1.14417
H	5.89850	-2.12057	1.56710
H	2.81200	2.40293	-0.09688

4e_conformer-180

C	-0.46548	2.86706	-0.09155
C	0.26474	0.72130	0.09849
N	0.56962	2.00150	-0.34404
H	1.38831	2.23325	-0.88811
C	-1.44665	2.13629	0.55337
C	-0.41855	4.30236	-0.50235
H	-1.32257	4.81586	-0.16419
H	0.44663	4.82278	-0.07218
H	-0.35694	4.41558	-1.59267
C	1.24285	-0.35978	-0.01179
C	0.84794	-1.70025	-0.15380
C	2.62195	-0.05906	0.01957
C	1.80894	-2.70857	-0.25015
H	-0.20303	-1.95106	-0.22033
C	3.57343	-1.07403	-0.09579
H	2.93491	0.96645	0.17112
C	3.16664	-2.41065	-0.22664
H	1.49118	-3.74162	-0.36116
H	3.92228	-3.18521	-0.30805
O	4.92279	-0.86595	-0.07567
C	5.39170	0.47273	0.04738
H	5.07482	0.92164	0.99654
H	6.48041	0.41203	0.02064
H	5.03953	1.09681	-0.78288
C	-1.00476	0.78429	0.67646
C	-1.76002	-0.32515	1.35420
H	-1.05246	-1.06319	1.74975

H	-2.29561	0.08552	2.22108
C	-2.78551	-1.05234	0.45770
H	-2.27500	-1.46250	-0.42500
H	-3.17909	-1.91048	1.01699
C	-3.94023	-0.16159	-0.01065
H	-4.47508	0.22776	0.86892
H	-3.52588	0.71112	-0.52971
C	-4.93849	-0.86657	-0.94009
H	-5.65718	-0.12452	-1.31271
H	-4.40273	-1.24331	-1.82312
C	-5.70687	-2.01671	-0.28100
H	-6.23875	-1.66924	0.61374
H	-6.44860	-2.44092	-0.96732
H	-5.03875	-2.82895	0.02557
H	-2.38584	2.53331	0.91659

4e_conformer-181

C	-2.03811	-2.23288	-0.44805
C	-0.45946	-0.64932	-0.86683
N	-0.69746	-1.93957	-0.41385
H	0.02992	-2.61408	-0.22272
C	-2.67951	-1.09403	-0.90291
C	-2.56801	-3.57527	-0.06312
H	-3.66057	-3.56962	-0.10396
H	-2.21196	-4.36697	-0.73549
H	-2.26966	-3.85516	0.95515
C	0.89340	-0.09653	-0.86457
C	1.29767	0.84859	-1.82082
C	1.82039	-0.51746	0.11164
C	2.59440	1.36340	-1.79229
H	0.60583	1.16327	-2.59341
C	3.12006	-0.00594	0.12165
H	1.50025	-1.21502	0.87558
C	3.51127	0.94368	-0.83388
H	2.89798	2.09415	-2.53681
H	4.52347	1.33374	-0.80420
O	4.07462	-0.35799	1.03233
C	3.72802	-1.31529	2.02759
H	2.91363	-0.95106	2.66559
H	3.43406	-2.27107	1.57715
H	4.62546	-1.45818	2.63081
C	-1.70183	-0.09108	-1.16842
C	-2.01859	1.32985	-1.55334
H	-1.56951	1.58235	-2.52319
H	-3.10359	1.40517	-1.70252

C	-1.59215	2.40539	-0.53264
H	-0.50233	2.38173	-0.41138
H	-1.83094	3.38326	-0.97213
C	-2.25874	2.31361	0.84981
H	-2.15235	3.28966	1.34524
H	-3.34086	2.15675	0.72448
C	-1.69679	1.24076	1.79377
H	-1.88911	0.24480	1.38481
H	-0.60384	1.34600	1.84369
C	-2.28910	1.33941	3.20214
H	-3.37903	1.21321	3.18022
H	-1.87869	0.56904	3.86544
H	-2.08001	2.31716	3.65448
H	-3.75035	-0.97954	-1.01681

4e_conformer-182

C	2.03775	-2.23314	-0.44794
C	0.45921	-0.64948	-0.86678
N	0.69712	-1.93972	-0.41370
H	-0.03029	-2.61414	-0.22245
C	2.67923	-1.09439	-0.90294
C	2.56755	-3.57553	-0.06289
H	2.26923	-3.85530	0.95543
H	2.21140	-4.36727	-0.73516
H	3.66011	-3.56999	-0.10378
C	-0.89361	-0.09658	-0.86452
C	-1.29791	0.84839	-1.82089
C	-1.82055	-0.51728	0.11184
C	-2.59460	1.36329	-1.79235
H	-0.60613	1.16290	-2.59361
C	-3.12018	-0.00568	0.12188
H	-1.50039	-1.21474	0.87587
C	-3.51142	0.94381	-0.83378
H	-2.89820	2.09393	-2.53696
H	-4.52359	1.33394	-0.80408
O	-4.07469	-0.35752	1.03269
C	-3.72807	-1.31469	2.02807
H	-3.43420	-2.27055	1.57775
H	-2.91362	-0.95041	2.66595
H	-4.62547	-1.45743	2.63138
C	1.70162	-0.09138	-1.16849
C	2.01850	1.32945	-1.55366
H	3.10347	1.40459	-1.70312
H	1.56922	1.58189	-2.52343
C	1.59248	2.40519	-0.53299

H	1.83128	3.38296	-0.97269
H	0.50268	2.38170	-0.41143
C	2.25945	2.31350	0.84928
H	3.34146	2.15613	0.72366
H	2.15362	3.28971	1.34451
C	1.69732	1.24112	1.79367
H	0.60444	1.34695	1.84400
H	1.88894	0.24497	1.38483
C	2.29022	1.33973	3.20179
H	2.08185	2.31769	3.65400
H	1.87965	0.56973	3.86540
H	3.38008	1.21292	3.17947
H	3.75007	-0.98001	-1.01691

4e_conformer-183

C	2.91868	-1.86074	-0.28568
C	0.80883	-1.10595	0.10542
N	1.58058	-2.13253	-0.42414
H	1.20372	-2.90391	-0.95637
C	3.01262	-0.65148	0.37789
C	3.97456	-2.77420	-0.81699
H	3.88807	-3.78547	-0.39962
H	3.92461	-2.86821	-1.90973
H	4.96452	-2.38774	-0.56014
C	-0.65242	-1.22491	0.11426
C	-1.25431	-2.49297	0.26883
C	-1.48751	-0.11195	-0.04633
C	-2.63857	-2.61916	0.26157
H	-0.63301	-3.36952	0.42321
C	-2.88110	-0.24416	-0.03551
H	-1.07694	0.87592	-0.21121
C	-3.47035	-1.50474	0.11547
H	-3.08837	-3.60043	0.38530
H	-4.54618	-1.62812	0.12016
O	-3.57816	0.91922	-0.19770
C	-4.99980	0.84512	-0.22493
H	-5.39763	0.45261	0.71864
H	-5.34993	1.86811	-0.36866
H	-5.35116	0.21881	-1.05381
C	1.69385	-0.16230	0.62755
C	1.36721	1.08017	1.41303
H	0.31305	1.33826	1.28635
H	1.49028	0.87632	2.48731
C	2.24511	2.29999	1.06994
H	1.88860	3.15131	1.66353

H	3.27199	2.10861	1.40776
C	2.29274	2.68320	-0.41676
H	2.89313	3.59854	-0.51647
H	2.82645	1.89957	-0.96822
C	0.92874	2.90372	-1.08537
H	1.09726	3.20294	-2.12838
H	0.39439	1.94726	-1.13433
C	0.04929	3.95185	-0.39791
H	0.56756	4.91655	-0.33016
H	-0.88287	4.11014	-0.95203
H	-0.22015	3.65032	0.62059
H	3.93836	-0.16690	0.66005

4e_conformer-184

C	-2.14048	-2.22111	-0.35398
C	-0.60103	-0.59742	-0.76804
N	-0.80213	-1.91958	-0.39372
H	-0.06161	-2.60142	-0.30900
C	-2.81685	-1.05853	-0.67871
C	-2.63682	-3.59112	-0.02596
H	-3.72987	-3.59524	-0.00269
H	-2.31460	-4.33398	-0.76749
H	-2.27697	-3.93199	0.95311
C	0.75000	-0.04580	-0.85039
C	1.06628	0.98569	-1.74884
C	1.76754	-0.55656	-0.01689
C	2.36431	1.49543	-1.80265
H	0.30532	1.37301	-2.41566
C	3.06554	-0.04654	-0.08751
H	1.51989	-1.32068	0.70918
C	3.36850	0.98870	-0.98442
H	2.59805	2.29325	-2.50214
H	4.38168	1.37573	-1.01883
O	4.10170	-0.48090	0.68832
C	3.84267	-1.51744	1.62942
H	4.79264	-1.71290	2.12842
H	3.09766	-1.20539	2.37143
H	3.49614	-2.43134	1.13189
C	-1.86460	-0.02959	-0.93847
C	-2.21628	1.40645	-1.22742
H	-1.82698	1.70870	-2.20864
H	-3.30869	1.47693	-1.31155
C	-1.74109	2.43883	-0.18360
H	-0.65336	2.37103	-0.07148
H	-1.94079	3.43632	-0.59768

C	-2.42466	2.35038	1.19156
H	-2.23616	3.29286	1.72615
H	-3.51268	2.30167	1.03894
C	-1.99704	1.19208	2.10916
H	-2.61123	1.23873	3.01945
H	-2.22248	0.23372	1.63138
C	-0.51836	1.23050	2.50597
H	-0.26303	2.18399	2.98656
H	-0.28023	0.42679	3.21292
H	0.13629	1.10626	1.63838
H	-3.89344	-0.94558	-0.70853

4e_conformer-185

C	2.14010	-2.22133	-0.35415
C	0.60088	-0.59735	-0.76799
N	0.80179	-1.91957	-0.39377
H	0.06117	-2.60131	-0.30906
C	2.81663	-1.05884	-0.67887
C	2.63623	-3.59144	-0.02625
H	2.27640	-3.93231	0.95283
H	2.31384	-4.33420	-0.76780
H	3.72928	-3.59574	-0.00307
C	-0.75009	-0.04558	-0.85026
C	-1.06628	0.98609	-1.74854
C	-1.76769	-0.55639	-0.01686
C	-2.36427	1.49594	-1.80229
H	-0.30528	1.37347	-2.41528
C	-3.06565	-0.04627	-0.08742
H	-1.52012	-1.32066	0.70909
C	-3.36852	0.98915	-0.98416
H	-2.59794	2.29389	-2.50165
H	-4.38167	1.37626	-1.01853
O	-4.10185	-0.48068	0.68831
C	-3.84292	-1.51741	1.62924
H	-3.49646	-2.43125	1.13156
H	-3.09791	-1.20554	2.37132
H	-4.79292	-1.71287	2.12818
C	1.86453	-0.02973	-0.93849
C	2.21646	1.40626	-1.22742
H	3.30889	1.47653	-1.31157
H	1.82721	1.70858	-2.20864
C	1.74148	2.43872	-0.18359
H	1.94128	3.43617	-0.59770
H	0.65375	2.37107	-0.07139
C	2.42515	2.35023	1.19152

H	3.51316	2.30158	1.03882
H	2.23664	3.29268	1.72616
C	1.99765	1.19188	2.10911
H	2.22319	0.23354	1.63133
H	2.61185	1.23859	3.01939
C	0.51898	1.23013	2.50593
H	0.26353	2.18361	2.98648
H	-0.13566	1.10576	1.63836
H	0.28095	0.42642	3.21292
H	3.89324	-0.94607	-0.70876

4e_conformer-186

C	-0.77659	2.64238	-0.14996
C	0.19276	0.58688	-0.06962
N	0.38642	1.93635	-0.33238
H	1.23397	2.32176	-0.72369
C	-1.73226	1.73437	0.26810
C	-0.86202	4.11174	-0.40442
H	-1.86551	4.47316	-0.16418
H	-0.14496	4.67511	0.20622
H	-0.65922	4.35886	-1.45472
C	1.30756	-0.35613	-0.14619
C	1.11213	-1.70209	-0.49677
C	2.61805	0.08910	0.13148
C	2.19886	-2.57691	-0.55476
H	0.12175	-2.05605	-0.75398
C	3.69909	-0.79062	0.05219
H	2.77138	1.11492	0.44254
C	3.49004	-2.13591	-0.28788
H	2.03571	-3.61564	-0.82842
H	4.34300	-2.80494	-0.33724
O	4.99410	-0.43821	0.30354
C	5.26438	0.91775	0.64343
H	6.34464	0.98242	0.77963
H	4.95518	1.59931	-0.15828
H	4.76164	1.20426	1.57511
C	-1.13928	0.43629	0.32055
C	-1.82383	-0.82573	0.76742
H	-1.07862	-1.53059	1.15354
H	-2.48878	-0.59471	1.60926
C	-2.64214	-1.53544	-0.33344
H	-1.97333	-1.79196	-1.16538
H	-3.01050	-2.48801	0.06870
C	-3.81568	-0.72019	-0.89584
H	-3.42311	0.19106	-1.36386

H	-4.28831	-1.30295	-1.69901
C	-4.89414	-0.32949	0.12645
H	-4.46422	0.33261	0.88910
H	-5.65963	0.26478	-0.38983
C	-5.56337	-1.52314	0.81461
H	-4.85252	-2.08674	1.42906
H	-6.37815	-1.19537	1.47009
H	-5.98503	-2.21660	0.07612
H	-2.75443	1.97934	0.52565

4e_conformer-187

C	3.65781	0.62813	0.18593
C	1.59993	-0.30370	-0.07052
N	2.94342	-0.54488	0.18135
H	3.31703	-1.44590	0.44356
C	2.76103	1.64192	-0.09794
C	5.12440	0.66348	0.46763
H	5.35754	0.30144	1.47754
H	5.69174	0.04389	-0.23856
H	5.49400	1.68928	0.38721
C	0.64427	-1.40785	-0.14880
C	1.04593	-2.66533	-0.63185
C	-0.68884	-1.23561	0.27591
C	0.13474	-3.72091	-0.68671
H	2.06015	-2.80799	-0.99167
C	-1.59438	-2.29741	0.20756
H	-0.98880	-0.28356	0.69093
C	-1.18247	-3.54859	-0.27553
H	0.45426	-4.68788	-1.06517
H	-1.90195	-4.36005	-0.31374
O	-2.89690	-2.22123	0.60977
C	-3.36502	-0.98236	1.13239
H	-3.29735	-0.18474	0.38492
H	-4.41082	-1.14464	1.39641
H	-2.80404	-0.68753	2.02749
C	1.46343	1.07046	-0.26264
C	0.21873	1.83024	-0.62496
H	0.46757	2.58845	-1.38024
H	-0.49939	1.15365	-1.09489
C	-0.42449	2.54996	0.57778
H	-0.75605	1.80839	1.31891
H	0.36541	3.12997	1.07272
C	-1.58832	3.49815	0.24301
H	-1.70532	4.19215	1.08587
H	-1.31181	4.11826	-0.62269

C	-2.96029	2.85188	-0.01322
H	-3.20294	2.18700	0.82810
H	-3.71559	3.64880	0.00793
C	-3.11100	2.08719	-1.33324
H	-2.79783	2.70625	-2.18283
H	-2.51330	1.17115	-1.35578
H	-4.15581	1.79870	-1.49720
H	3.01185	2.69095	-0.19523

4e_conformer-188

C	-2.71478	-1.80893	-0.50477
C	-0.64346	-0.88759	-0.34814
N	-1.44748	-1.96336	0.00229
H	-1.10556	-2.80141	0.45050
C	-2.73481	-0.59362	-1.16366
C	-3.77508	-2.84485	-0.32021
H	-3.50284	-3.80045	-0.78729
H	-3.96906	-3.04557	0.74117
H	-4.70997	-2.50540	-0.77422
C	0.74201	-0.81103	0.11631
C	1.08836	-1.27083	1.40479
C	1.75154	-0.28920	-0.70204
C	2.40574	-1.19531	1.84200
H	0.31700	-1.65680	2.06396
C	3.07303	-0.19949	-0.24743
H	1.53281	0.03050	-1.71449
C	3.41078	-0.65745	1.03182
H	2.66297	-1.54739	2.83716
H	4.42824	-0.60350	1.39865
O	3.96314	0.33358	-1.13526
C	5.32716	0.42540	-0.73697
H	5.74707	-0.56413	-0.51960
H	5.85644	0.86701	-1.58229
H	5.44391	1.06909	0.14319
C	-1.43837	0.00035	-1.06764
C	-1.08265	1.37333	-1.57124
H	-0.01996	1.57664	-1.40263
H	-1.22732	1.39940	-2.66039
C	-1.91176	2.52021	-0.94727
H	-1.92098	3.36107	-1.65323
H	-2.95552	2.19950	-0.84347
C	-1.38190	3.05240	0.39439
H	-0.36883	3.44750	0.23073
H	-2.00030	3.91345	0.68782
C	-1.33490	2.06312	1.56887

H	-0.63792	1.24991	1.34210
H	-0.91754	2.59160	2.43740
C	-2.69582	1.47294	1.94775
H	-3.09670	0.85072	1.14169
H	-2.61495	0.84528	2.84320
H	-3.42486	2.26562	2.16110
H	-3.59943	-0.16446	-1.65438

4e_conformer-189

C	2.72542	-2.11480	-0.24012
C	0.93158	-0.89984	0.44181
N	1.35606	-2.09452	-0.12029
H	0.75041	-2.88433	-0.29312
C	3.18487	-0.89586	0.22324
C	3.45692	-3.30370	-0.77242
H	3.31302	-4.19148	-0.14265
H	3.12637	-3.56715	-1.78531
H	4.52911	-3.09345	-0.81300
C	-0.49185	-0.62290	0.64495
C	-0.95046	0.01236	1.81578
C	-1.42663	-0.98657	-0.33302
C	-2.30305	0.29186	1.97142
H	-0.24562	0.26459	2.60008
C	-2.79020	-0.71639	-0.16218
H	-1.10593	-1.45828	-1.25674
C	-3.23713	-0.06415	0.99322
H	-2.64956	0.78493	2.87543
H	-4.28589	0.16141	1.14149
O	-3.60129	-1.11636	-1.18513
C	-4.99420	-0.84044	-1.07712
H	-5.43449	-1.34202	-0.20697
H	-5.18307	0.23757	-1.00719
H	-5.44792	-1.23049	-1.98910
C	2.06480	-0.12070	0.65697
C	2.11157	1.31592	1.09820
H	1.12718	1.62586	1.45401
H	2.79664	1.42346	1.95084
C	2.58697	2.26949	-0.02219
H	2.67265	3.28610	0.38718
H	3.60350	1.96814	-0.30536
C	1.70642	2.29555	-1.28646
H	2.34476	2.53289	-2.14771
H	1.31152	1.28727	-1.46994
C	0.54818	3.30673	-1.28028
H	0.95766	4.31453	-1.11936

H	0.10297	3.31850	-2.28482
C	-0.56185	3.04203	-0.25834
H	-0.96252	2.02781	-0.36005
H	-0.20349	3.15158	0.77021
H	-1.38967	3.74859	-0.39315
H	4.22215	-0.58513	0.23752

4e_conformer-190

C	-2.21243	-2.38282	-0.22707
C	-0.61557	-0.92201	0.46345
N	-0.86226	-2.14736	-0.13936
H	-0.14531	-2.82312	-0.36171
C	-2.84686	-1.27366	0.30153
C	-2.76133	-3.64950	-0.79785
H	-3.85414	-3.61584	-0.79586
H	-2.43070	-3.80887	-1.83218
H	-2.45192	-4.52941	-0.21869
C	0.75172	-0.44002	0.65176
C	1.11033	0.31518	1.78715
C	1.73878	-0.72442	-0.30211
C	2.41284	0.78006	1.93153
H	0.37422	0.50889	2.55880
C	3.05167	-0.26681	-0.14124
H	1.49613	-1.27730	-1.20427
C	3.39716	0.49899	0.97903
H	2.68058	1.36152	2.80944
H	4.40517	0.86926	1.11881
O	3.91861	-0.60681	-1.14016
C	5.26191	-0.14469	-1.04178
H	5.30695	0.95095	-1.02668
H	5.75556	-0.53931	-0.14558
H	5.77220	-0.51707	-1.93099
C	-1.85325	-0.34443	0.73996
C	-2.11983	1.02617	1.30170
H	-2.46472	0.94678	2.34367
H	-1.18853	1.59675	1.34314
C	-3.18068	1.82145	0.51585
H	-4.14242	1.29906	0.60542
H	-3.31796	2.79429	1.00834
C	-2.89918	2.05201	-0.97720
H	-2.73492	1.08422	-1.46936
H	-3.81692	2.46507	-1.41686
C	-1.73678	3.00370	-1.31963
H	-1.91991	3.41673	-2.32075
H	-1.75786	3.86271	-0.63325

C	-0.33832	2.37493	-1.31701
H	-0.30775	1.48859	-1.96188
H	0.40694	3.08676	-1.69225
H	-0.01936	2.05843	-0.32175
H	-3.91946	-1.13789	0.35268

4e_conformer-191

C	-3.26274	-1.51076	-0.15337
C	-1.07477	-0.90052	-0.17028
N	-1.99746	-1.83590	0.27436
H	-1.74996	-2.69937	0.73650
C	-3.15408	-0.32608	-0.85775
C	-4.44577	-2.37353	0.14302
H	-5.35304	-1.91028	-0.25408
H	-4.58563	-2.51868	1.22188
H	-4.35259	-3.36964	-0.30928
C	0.34024	-1.00602	0.19035
C	0.71962	-1.39948	1.49038
C	1.34231	-0.70916	-0.74040
C	2.06548	-1.46715	1.83278
H	-0.04400	-1.61749	2.23068
C	2.69479	-0.75886	-0.38244
H	1.08791	-0.44395	-1.76033
C	3.06659	-1.14210	0.91169
H	2.35075	-1.76336	2.83846
H	4.10739	-1.19268	1.20651
O	3.57593	-0.42954	-1.37240
C	4.96592	-0.46955	-1.06645
H	5.28257	-1.47888	-0.77701
H	5.48224	-0.17527	-1.98109
H	5.21797	0.23286	-0.26283
C	-1.78058	0.06935	-0.87722
C	-1.25195	1.36671	-1.42364
H	-0.16139	1.36990	-1.38115
H	-1.51955	1.46132	-2.48543
C	-1.80689	2.60120	-0.67697
H	-1.44713	3.51173	-1.17688
H	-2.89792	2.59906	-0.79539
C	-1.47066	2.67132	0.82494
H	-2.27665	3.21601	1.33415
H	-1.47969	1.65545	1.24250
C	-0.14703	3.36427	1.18897
H	-0.15629	4.38473	0.77937
H	-0.11371	3.47782	2.28140
C	1.12904	2.64781	0.73564

H	1.22019	2.62591	-0.35503
H	2.01862	3.15536	1.12768
H	1.14853	1.61082	1.08733
H	-3.98101	0.21152	-1.30479

4e_conformer-192

C	3.26277	-1.51072	-0.15337
C	1.07480	-0.90050	-0.17028
N	1.99750	-1.83588	0.27436
H	1.75002	-2.69935	0.73650
C	3.15410	-0.32603	-0.85775
C	4.44582	-2.37348	0.14302
H	4.35266	-3.36958	-0.30931
H	5.35308	-1.91020	-0.25407
H	4.58567	-2.51865	1.22187
C	-0.34021	-1.00602	0.19036
C	-0.71959	-1.39948	1.49039
C	-1.34228	-0.70918	-0.74040
C	-2.06544	-1.46717	1.83279
H	0.04404	-1.61747	2.23069
C	-2.69476	-0.75891	-0.38244
H	-1.08789	-0.44397	-1.76033
C	-3.06655	-1.14215	0.91169
H	-2.35071	-1.76338	2.83846
H	-4.10735	-1.19274	1.20651
O	-3.57591	-0.42960	-1.37240
C	-4.96590	-0.46964	-1.06645
H	-5.21795	0.23277	-0.26283
H	-5.48222	-0.17537	-1.98109
H	-5.28253	-1.47897	-0.77701
C	1.78059	0.06938	-0.87721
C	1.25195	1.36673	-1.42363
H	1.51959	1.46137	-2.48540
H	0.16139	1.36990	-1.38117
C	1.80684	2.60122	-0.67692
H	2.89788	2.59910	-0.79530
H	1.44708	3.51175	-1.17684
C	1.47055	2.67131	0.82498
H	1.47954	1.65544	1.24252
H	2.27652	3.21598	1.33424
C	0.14691	3.36427	1.18895
H	0.11353	3.47779	2.28139
H	0.15621	4.38475	0.77939
C	-1.12914	2.64785	0.73554
H	-1.22023	2.62598	-0.35514

H	-1.14868	1.61085	1.08720
H	-2.01874	3.15541	1.12753
H	3.98103	0.21158	-1.30478

4e_conformer-193

C	0.33098	3.17840	-0.16962
C	-0.20766	0.98505	-0.45076
N	-0.69209	2.26546	-0.21730
H	-1.67238	2.50869	-0.21974
C	1.50407	2.46675	-0.34801
C	0.08477	4.63828	0.02816
H	1.03665	5.17523	0.05862
H	-0.51624	5.06736	-0.78425
H	-0.44654	4.84027	0.96701
C	-1.12064	-0.15522	-0.51596
C	-0.81604	-1.29698	-1.27559
C	-2.34322	-0.11675	0.18801
C	-1.70814	-2.36899	-1.32076
H	0.10358	-1.33783	-1.84619
C	-3.23529	-1.18906	0.12254
H	-2.56800	0.73948	0.81147
C	-2.91660	-2.32688	-0.63348
H	-1.46116	-3.24611	-1.91248
H	-3.62004	-3.15252	-0.66504
O	-4.43513	-1.22780	0.77345
C	-4.81273	-0.09540	1.54944
H	-5.79727	-0.32730	1.95762
H	-4.10868	0.07829	2.37227
H	-4.87648	0.80858	0.93171
C	1.18351	1.08996	-0.53107
C	2.20160	-0.01016	-0.67176
H	2.05281	-0.57887	-1.59968
H	3.18924	0.45537	-0.76853
C	2.21358	-0.98919	0.51602
H	2.45077	-0.42935	1.42954
H	1.20279	-1.38882	0.65663
C	3.18673	-2.16315	0.34057
H	3.10986	-2.81669	1.22107
H	2.86113	-2.76585	-0.51932
C	4.65969	-1.77249	0.14206
H	5.24926	-2.69091	0.02152
H	4.77336	-1.21675	-0.79791
C	5.24212	-0.94763	1.29367
H	5.12495	-1.47134	2.25077
H	6.31113	-0.75891	1.14359

H	4.74495	0.02454	1.38606
H	2.50158	2.88839	-0.34194

4e_conformer-194

C	1.28365	2.55679	0.03831
C	0.13920	0.60482	0.26831
N	0.11664	1.89364	-0.25044
H	-0.60159	2.24025	-0.87015
C	2.05918	1.69075	0.78685
C	1.54509	3.94749	-0.44009
H	2.51519	4.29169	-0.07166
H	1.56243	4.00727	-1.53618
H	0.78063	4.65185	-0.08810
C	-1.01246	-0.28185	0.09682
C	-0.86071	-1.66802	-0.06908
C	-2.31236	0.26683	0.07565
C	-1.98361	-2.48093	-0.23664
H	0.12951	-2.10389	-0.09810
C	-3.42658	-0.55378	-0.11086
H	-2.43633	1.33032	0.23829
C	-3.26382	-1.93929	-0.26237
H	-1.85462	-3.55199	-0.36563
H	-4.14310	-2.56028	-0.39971
O	-4.71448	-0.10189	-0.14540
C	-4.93615	1.29777	-0.00666
H	-6.01509	1.43867	-0.08225
H	-4.58800	1.66309	0.96706
H	-4.43675	1.86176	-0.80373
C	1.35428	0.45740	0.93483
C	1.83784	-0.71217	1.74796
H	2.02301	-0.36506	2.77467
H	1.05180	-1.47114	1.82483
C	3.14014	-1.37266	1.24744
H	3.90877	-0.59971	1.12106
H	3.50404	-2.03110	2.04736
C	3.02643	-2.21322	-0.03471
H	3.95947	-2.78408	-0.14800
H	2.23420	-2.96288	0.10657
C	2.77182	-1.45423	-1.34770
H	2.69697	-2.19464	-2.15605
H	1.80507	-0.94274	-1.30539
C	3.86207	-0.43798	-1.69836
H	4.85418	-0.90765	-1.70668
H	3.68925	-0.00470	-2.69044
H	3.88293	0.38642	-0.97867

H	3.03337	1.92186	1.19902

4e_conformer-195

C	-1.28365	2.55679	0.03830
C	-0.13919	0.60482	0.26830
N	-0.11664	1.89365	-0.25044
H	0.60159	2.24025	-0.87015
C	-2.05918	1.69076	0.78684
C	-1.54509	3.94749	-0.44009
H	-0.78064	4.65185	-0.08809
H	-1.56242	4.00727	-1.53618
H	-2.51519	4.29169	-0.07167
C	1.01246	-0.28185	0.09682
C	0.86071	-1.66801	-0.06909
C	2.31236	0.26682	0.07566
C	1.98360	-2.48093	-0.23664
H	-0.12951	-2.10388	-0.09811
C	3.42658	-0.55379	-0.11086
H	2.43633	1.33032	0.23830
C	3.26382	-1.93929	-0.26237
H	1.85461	-3.55199	-0.36564
H	4.14309	-2.56028	-0.39972
O	4.71448	-0.10189	-0.14539
C	4.93616	1.29777	-0.00666
H	4.43675	1.86176	-0.80372
H	4.58800	1.66308	0.96707
H	6.01510	1.43866	-0.08224
C	-1.35428	0.45740	0.93482
C	-1.83784	-0.71217	1.74796
H	-1.05181	-1.47115	1.82482
H	-2.02300	-0.36506	2.77467
C	-3.14015	-1.37266	1.24744
H	-3.50405	-2.03109	2.04736
H	-3.90877	-0.59969	1.12106
C	-3.02645	-2.21322	-0.03470
H	-2.23423	-2.96290	0.10659
H	-3.95950	-2.78407	-0.14799
C	-2.77182	-1.45424	-1.34770
H	-1.80506	-0.94278	-1.30538
H	-2.69699	-2.19466	-2.15604
C	-3.86205	-0.43797	-1.69836
H	-4.85416	-0.90762	-1.70669
H	-3.88289	0.38643	-0.97867
H	-3.68922	-0.00470	-2.69045
H	-3.03337	1.92187	1.19902

4e_conformer-196

C	-0.22570	3.20027	-0.24962
C	0.15324	0.97355	0.01223
N	0.66591	2.17583	-0.45629
H	1.51484	2.25316	-0.99825
C	-1.31781	2.65284	0.39635
C	0.05194	4.59696	-0.70125
H	0.97835	4.99138	-0.26465
H	0.15293	4.66337	-1.79249
H	-0.76805	5.25503	-0.40139
C	0.94960	-0.25246	-0.07559
C	0.35797	-1.49141	-0.36327
C	2.34592	-0.18991	0.11233
C	1.14410	-2.64274	-0.43995
H	-0.70435	-1.54589	-0.55999
C	3.12549	-1.34473	0.01430
H	2.80000	0.76074	0.36358
C	2.52108	-2.58200	-0.25610
H	0.67634	-3.59756	-0.66361
H	3.14426	-3.46808	-0.32103
O	4.48032	-1.37147	0.18075
C	5.14508	-0.14101	0.44835
H	4.80317	0.30082	1.39213
H	6.20555	-0.38436	0.52476
H	4.99257	0.57973	-0.36409
C	-1.09388	1.25135	0.56888
C	-2.01700	0.32547	1.31524
H	-1.56477	-0.66784	1.40587
H	-2.13732	0.69750	2.34248
C	-3.42038	0.21648	0.68188
H	-4.09832	-0.26650	1.39702
H	-3.80982	1.23117	0.53192
C	-3.44427	-0.54111	-0.65431
H	-4.35677	-0.28036	-1.20780
H	-2.60453	-0.18948	-1.26867
C	-3.38444	-2.07188	-0.51504
H	-3.14918	-2.51089	-1.49361
H	-2.55895	-2.35598	0.15094
C	-4.68492	-2.68981	0.00875
H	-5.52375	-2.45774	-0.65902
H	-4.60445	-3.78039	0.08057
H	-4.94065	-2.31206	1.00511
H	-2.18716	3.20596	0.72909

4e_conformer-197

C	-2.27581	2.10197	0.15877
C	-0.22574	1.12135	0.25754
N	-0.96063	2.19761	-0.22137
H	-0.59526	2.89963	-0.84844
C	-2.38628	0.95395	0.92152
C	-3.29755	3.11025	-0.25380
H	-3.03093	4.12244	0.07618
H	-3.42119	3.14555	-1.34406
H	-4.26701	2.85786	0.18408
C	1.21497	1.04169	0.02703
C	1.98043	2.20869	-0.14860
C	1.86541	-0.20683	-0.02650
C	3.35491	2.12136	-0.37301
H	1.50982	3.18424	-0.07703
C	3.24420	-0.28148	-0.23305
H	1.27780	-1.10752	0.06255
C	3.99722	0.88806	-0.41295
H	3.93549	3.03016	-0.50500
H	5.06636	0.80715	-0.58000
O	3.94267	-1.45361	-0.29125
C	3.22287	-2.66808	-0.10546
H	2.47078	-2.81149	-0.89090
H	3.96341	-3.46685	-0.16251
H	2.73040	-2.69438	0.87416
C	-1.10880	0.32051	0.98505
C	-0.81507	-0.94386	1.74516
H	0.21410	-0.92286	2.12469
H	-1.46777	-0.96830	2.62779
C	-1.02673	-2.27080	0.97803
H	-0.25649	-2.38796	0.20384
H	-0.86097	-3.08649	1.69338
C	-2.40959	-2.42547	0.32001
H	-2.69104	-3.48728	0.30527
H	-3.16725	-1.92144	0.93648
C	-2.47845	-1.88721	-1.11531
H	-2.09737	-0.86122	-1.13893
H	-1.80585	-2.48434	-1.74756
C	-3.89537	-1.92040	-1.69095
H	-4.30883	-2.93678	-1.67370
H	-4.56705	-1.27981	-1.10592
H	-3.91739	-1.56776	-2.72860
H	-3.29440	0.60077	1.39294

4e_conformer-198

C	2.27582	2.10186	0.15895
C	0.22568	1.12136	0.25751
N	0.96066	2.19761	-0.22127
H	0.59538	2.89970	-0.84831
C	2.38616	0.95378	0.92162
C	3.29764	3.11013	-0.25346
H	4.26713	2.85749	0.18420
H	3.42114	3.14570	-1.34373
H	3.03121	4.12226	0.07683
C	-1.21501	1.04173	0.02695
C	-1.98047	2.20872	-0.14870
C	-1.86543	-0.20681	-0.02654
C	-3.35495	2.12136	-0.37312
H	-1.50985	3.18426	-0.07716
C	-3.24422	-0.28147	-0.23309
H	-1.27779	-1.10749	0.06257
C	-3.99724	0.88806	-0.41302
H	-3.93554	3.03015	-0.50513
H	-5.06638	0.80714	-0.58007
O	-3.94270	-1.45361	-0.29126
C	-3.22295	-2.66807	-0.10525
H	-2.73048	-2.69422	0.87438
H	-3.96352	-3.46683	-0.16214
H	-2.47087	-2.81166	-0.89067
C	1.10864	0.32041	0.98500
C	0.81472	-0.94405	1.74487
H	1.46721	-0.96868	2.62765
H	-0.21454	-0.92308	2.12416
C	1.02648	-2.27084	0.97751
H	0.86041	-3.08670	1.69260
H	0.25648	-2.38772	0.20304
C	2.40953	-2.42549	0.31987
H	2.69089	-3.48731	0.30497
H	3.16705	-1.92167	0.93669
C	2.47886	-1.88689	-1.11530
H	1.80637	-2.48379	-1.74789
H	2.09794	-0.86085	-1.13877
C	3.89594	-1.92012	-1.69056
H	4.30926	-2.93655	-1.67348
H	3.91829	-1.56720	-2.72810
H	4.56755	-1.27978	-1.10517
H	3.29424	0.60048	1.39305

4e_conformer-199

C	-2.04118	2.23046	-0.39198
C	-0.48306	0.62828	-0.80428
N	-0.69698	1.94948	-0.43576
H	0.03686	2.63869	-0.35275
C	-2.70369	1.05739	-0.70247
C	-2.55587	3.59588	-0.07275
H	-3.64895	3.58606	-0.05110
H	-2.20222	3.94666	0.90517
H	-2.24187	4.33890	-0.81760
C	0.87264	0.08271	-0.87809
C	1.23972	-0.83529	-1.87421
C	1.83641	0.48738	0.06846
C	2.53818	-1.34783	-1.90832
H	0.52100	-1.12079	-2.63393
C	3.13675	-0.01964	0.01485
H	1.54242	1.17108	0.85526
C	3.48976	-0.94808	-0.97626
H	2.81544	-2.05745	-2.68286
H	4.50351	-1.33487	-0.99628
O	4.12736	0.31655	0.89182
C	3.81842	1.24972	1.92211
H	3.03103	0.86798	2.58322
H	4.73894	1.38176	2.49206
H	3.50436	2.21447	1.50575
C	-1.73445	0.03856	-0.95854
C	-2.05611	-1.40662	-1.22874
H	-2.65735	-1.46981	-2.14650
H	-1.13788	-1.96860	-1.42918
C	-2.83376	-2.11341	-0.09370
H	-3.58103	-1.42420	0.31815
H	-3.39804	-2.94750	-0.53116
C	-1.96220	-2.68994	1.03420
H	-1.29021	-3.44310	0.59788
H	-2.61941	-3.23443	1.72812
C	-1.11723	-1.69387	1.84295
H	-0.54673	-2.26455	2.58906
H	-0.37737	-1.21891	1.19089
C	-1.93282	-0.61202	2.55583
H	-2.69775	-1.05727	3.20543
H	-1.28788	0.01532	3.18266
H	-2.43647	0.04288	1.83811
H	-3.77948	0.93681	-0.72889

4e_conformer-200

C	-1.56166	2.33722	0.28056

C	-0.13993	0.56195	0.33071
N	-0.31920	1.87714	-0.07741
H	0.33714	2.38789	-0.65006
C	-2.18702	1.30640	0.95730
C	-2.03210	3.71095	-0.06931
H	-1.36835	4.48644	0.33378
H	-2.08609	3.86134	-1.15542
H	-3.03189	3.87714	0.34069
C	1.13694	-0.11692	0.11775
C	1.21699	-1.51861	-0.01816
C	2.32610	0.62505	0.04210
C	2.44849	-2.13336	-0.21265
H	0.31456	-2.11538	-0.00106
C	3.55988	-0.00000	-0.17040
H	2.32275	1.70183	0.17881
C	3.63145	-1.39261	-0.29483
H	2.49534	-3.21373	-0.31859
H	4.57635	-1.89730	-0.45321
O	4.63872	0.83593	-0.22577
C	5.92347	0.25237	-0.41674
H	6.17559	-0.43420	0.40041
H	5.98040	-0.28510	-1.37093
H	6.63087	1.08261	-0.42501
C	-1.31091	0.17998	0.98824
C	-1.62220	-1.13218	1.65336
H	-0.69927	-1.58750	2.03306
H	-2.24952	-0.92937	2.53158
C	-2.36249	-2.17710	0.78579
H	-1.69619	-2.54850	-0.00429
H	-2.57682	-3.03866	1.43113
C	-3.66557	-1.67620	0.13640
H	-4.37350	-2.51154	0.04686
H	-4.14740	-0.94345	0.79918
C	-3.47216	-1.05723	-1.25436
H	-2.69450	-0.28823	-1.20719
H	-3.10109	-1.83501	-1.93703
C	-4.75961	-0.44933	-1.81412
H	-5.56229	-1.19575	-1.86751
H	-5.11203	0.37034	-1.17557
H	-4.60991	-0.04625	-2.82242
H	-3.17766	1.35274	1.39108

4e_conformer-201

C	1.56176	2.33715	0.28080
C	0.14005	0.56185	0.33095

N	0.31931	1.87705	-0.07718
H	-0.33702	2.38778	-0.64984
C	2.18712	1.30634	0.95756
C	2.03219	3.71088	-0.06907
H	2.08630	3.86123	-1.15519
H	1.36838	4.48636	0.33391
H	3.03193	3.87711	0.34103
C	-1.13685	-0.11699	0.11799
C	-1.21702	-1.51870	-0.01754
C	-2.32595	0.62506	0.04199
C	-2.44856	-2.13339	-0.21201
H	-0.31466	-2.11557	-0.00017
C	-3.55976	0.00006	-0.17050
H	-2.32254	1.70188	0.17843
C	-3.63144	-1.39257	-0.29454
H	-2.49549	-3.21380	-0.31765
H	-4.57637	-1.89723	-0.45290
O	-4.63852	0.83608	-0.22624
C	-5.92330	0.25259	-0.41722
H	-5.98015	-0.28513	-1.37128
H	-6.17557	-0.43375	0.40008
H	-6.63062	1.08289	-0.42578
C	1.31104	0.17990	0.98849
C	1.62245	-1.13226	1.65352
H	2.24998	-0.92947	2.53159
H	0.69962	-1.58762	2.03341
C	2.36257	-2.17710	0.78571
H	2.57711	-3.03869	1.43094
H	1.69609	-2.54847	-0.00424
C	3.66544	-1.67606	0.13600
H	4.14727	-0.94311	0.79856
H	4.37351	-2.51128	0.04644
C	3.47163	-1.05734	-1.25481
H	3.10060	-1.83529	-1.93730
H	2.69382	-0.28849	-1.20759
C	4.75883	-0.44923	-1.81491
H	5.11120	0.37062	-1.17654
H	5.56167	-1.19548	-1.86835
H	4.60885	-0.04633	-2.82324
H	3.17777	1.35269	1.39134

4e_conformer-202

C	3.65775	-0.62806	-0.18607
C	1.59988	0.30375	0.07058
N	2.94335	0.54495	-0.18137

H	3.31696	1.44598	-0.44354
C	2.76098	-1.64187	0.09781
C	5.12432	-0.66340	-0.46785
H	5.35740	-0.30127	-1.47773
H	5.69170	-0.04388	0.23837
H	5.49392	-1.68921	-0.38754
C	0.64421	1.40790	0.14891
C	1.04579	2.66528	0.63228
C	-0.68883	1.23575	-0.27605
C	0.13460	3.72085	0.68722
H	2.05996	2.80786	0.99229
C	-1.59437	2.29756	-0.20766
H	-0.98874	0.28377	-0.69126
C	-1.18253	3.54863	0.27576
H	0.45407	4.68774	1.06592
H	-1.90201	4.36009	0.31401
O	-2.89681	2.22148	-0.61010
C	-3.36490	0.98269	-1.13295
H	-3.29743	0.18499	-0.38554
H	-4.41064	1.14507	-1.39716
H	-2.80376	0.68793	-2.02797
C	1.46339	-1.07042	0.26262
C	0.21871	-1.83024	0.62493
H	0.46757	-2.58843	1.38022
H	-0.49944	-1.15367	1.09486
C	-0.42449	-2.54999	-0.57780
H	-0.75621	-1.80845	-1.31889
H	0.36545	-3.12988	-1.07281
C	-1.58816	-3.49837	-0.24295
H	-1.70515	-4.19235	-1.08583
H	-1.31148	-4.11847	0.62270
C	-2.96020	-2.85231	0.01345
H	-3.20308	-2.18748	-0.82786
H	-3.71536	-3.64936	-0.00757
C	-3.11084	-2.08762	1.33347
H	-2.79739	-2.70658	2.18302
H	-2.51332	-1.17145	1.35587
H	-4.15568	-1.79933	1.49760
H	3.01180	-2.69090	0.19502

4e_conformer-203

C	-1.66606	3.18159	-0.20483
C	-1.25246	0.95969	0.04564
N	-2.22706	1.93009	-0.14212
H	-3.20031	1.72923	-0.32194

C	-0.30419	3.01736	-0.02549
C	-2.48600	4.40843	-0.43718
H	-2.99982	4.38120	-1.40698
H	-3.25537	4.53873	0.33460
H	-1.84374	5.29308	-0.42421
C	-1.61052	-0.45115	0.17547
C	-2.85875	-0.83320	0.69829
C	-0.71010	-1.45689	-0.22986
C	-3.19031	-2.18425	0.81000
H	-3.55632	-0.07795	1.04673
C	-1.04383	-2.80555	-0.09265
H	0.22924	-1.17124	-0.67909
C	-2.29375	-3.17587	0.42519
H	-4.15690	-2.46751	1.21729
H	-2.53616	-4.22972	0.51558
O	-0.22070	-3.83395	-0.45348
C	1.07323	-3.50893	-0.95122
H	1.01212	-2.93462	-1.88353
H	1.65151	-2.93828	-0.21424
H	1.56813	-4.46158	-1.14403
C	-0.02957	1.62661	0.13322
C	1.32922	1.03285	0.37646
H	1.89309	1.70065	1.03812
H	1.23523	0.07769	0.90707
C	2.13432	0.81922	-0.92085
H	1.50209	0.28854	-1.64563
H	2.34004	1.80118	-1.36897
C	3.45205	0.04718	-0.76187
H	3.23848	-0.96769	-0.39461
H	3.88017	-0.08017	-1.76457
C	4.50632	0.70352	0.15264
H	5.50645	0.43686	-0.21253
H	4.43486	1.79670	0.06194
C	4.41597	0.29740	1.62858
H	5.20930	0.77353	2.21664
H	3.45739	0.57549	2.07656
H	4.52688	-0.78860	1.73663
H	0.42526	3.81728	0.00262

4e_conformer-204

C	1.66936	3.17993	-0.20493
C	1.25348	0.95848	0.04561
N	2.22908	1.92786	-0.14218
H	3.20216	1.72600	-0.32182
C	0.30732	3.01710	-0.02560

C	2.49052	4.40594	-0.43733
H	3.26015	4.53538	0.33434
H	3.00416	4.37825	-1.40722
H	1.84917	5.29125	-0.42419
C	1.61003	-0.45274	0.17551
C	2.85773	-0.83612	0.69858
C	0.70861	-1.45751	-0.23000
C	3.18782	-2.18753	0.81039
H	3.55603	-0.08161	1.04715
C	1.04086	-2.80653	-0.09269
H	-0.23032	-1.17085	-0.67944
C	2.29027	-3.17819	0.42541
H	4.15402	-2.47183	1.21787
H	2.53152	-4.23229	0.51587
O	0.21670	-3.83405	-0.45367
C	-1.07675	-3.50765	-0.95175
H	-1.65461	-2.93638	-0.21492
H	-1.01478	-2.93340	-1.88403
H	-1.57262	-4.45977	-1.14469
C	0.03127	1.62664	0.13316
C	-1.32812	1.03428	0.37647
H	-1.23507	0.07908	0.90720
H	-1.89130	1.70271	1.03807
C	-2.13345	0.82127	-0.92079
H	-2.33829	1.80339	-1.36898
H	-1.50173	0.28997	-1.64554
C	-3.45188	0.05045	-0.76172
H	-3.88015	-0.07659	-1.76439
H	-3.23922	-0.96459	-0.39437
C	-4.50553	0.70781	0.15277
H	-4.43311	1.80092	0.06196
H	-5.50591	0.44200	-0.21233
C	-4.41549	0.30177	1.62876
H	-3.45666	0.57907	2.07668
H	-5.20839	0.77864	2.21679
H	-4.52734	-0.78413	1.73693
H	-0.42130	3.81778	0.00246

4e_conformer-205

C	2.39772	2.03667	0.11192
C	0.35354	1.04957	0.26000
N	1.07566	2.12255	-0.24594
H	0.69711	2.81522	-0.87553
C	2.52625	0.89927	0.88757
C	3.40796	3.04327	-0.33192

H	3.51312	3.06373	-1.42451
H	3.14259	4.05892	-0.01170
H	4.38576	2.80102	0.09295
C	-1.09178	0.96614	0.05976
C	-1.85893	2.13975	-0.11516
C	-1.75539	-0.26765	0.03399
C	-3.23210	2.05802	-0.30905
H	-1.38128	3.11320	-0.06556
C	-3.14205	-0.34038	-0.14528
H	-1.20702	-1.19619	0.12344
C	-3.89431	0.82597	-0.32386
H	-3.81015	2.96882	-0.43883
H	-4.96648	0.78998	-0.47142
O	-3.66407	-1.60282	-0.14723
C	-5.06660	-1.74276	-0.34812
H	-5.63572	-1.24396	0.44562
H	-5.37303	-1.33916	-1.32075
H	-5.26747	-2.81452	-0.31999
C	1.25258	0.26205	0.98168
C	0.97579	-0.99600	1.75713
H	1.64046	-1.01115	2.63110
H	-0.04863	-0.97598	2.14937
C	1.18192	-2.32594	0.99449
H	1.01949	-3.13911	1.71349
H	0.40644	-2.44218	0.22563
C	2.56151	-2.48478	0.32990
H	3.32221	-1.97478	0.93780
H	2.84418	-3.54638	0.32290
C	2.62278	-1.95941	-1.11055
H	1.94721	-2.56239	-1.73398
H	2.24138	-0.93374	-1.14144
C	4.03670	-1.99751	-1.69332
H	4.71145	-1.35160	-1.11772
H	4.45047	-3.01363	-1.66908
H	4.05323	-1.65430	-2.73424
H	3.44413	0.55520	1.34669

4e_conformer-206

C	-2.39787	2.03656	0.11196
C	-0.35363	1.04957	0.25998
N	-1.07583	2.12250	-0.24593
H	-0.69732	2.81521	-0.87553
C	-2.52631	0.89915	0.88762
C	-3.40817	3.04310	-0.33185
H	-3.14284	4.05877	-0.01166

H	-3.51339	3.06354	-1.42444
H	-4.38595	2.80083	0.09307
C	1.09168	0.96620	0.05974
C	1.85879	2.13982	-0.11522
C	1.75532	-0.26758	0.03400
C	3.23197	2.05812	-0.30910
H	1.38111	3.11326	-0.06565
C	3.14198	-0.34028	-0.14527
H	1.20697	-1.19613	0.12349
C	3.89421	0.82609	-0.32388
H	3.80999	2.96893	-0.43890
H	4.96638	0.79013	-0.47143
O	3.66405	-1.60270	-0.14717
C	5.06658	-1.74260	-0.34806
H	5.37299	-1.33902	-1.32070
H	5.63568	-1.24377	0.44567
H	5.26748	-2.81435	-0.31990
C	-1.25261	0.26200	0.98169
C	-0.97569	-0.99601	1.75715
H	0.04874	-0.97591	2.14934
H	-1.64032	-1.01120	2.63115
C	-1.18176	-2.32599	0.99455
H	-0.40622	-2.44225	0.22575
H	-1.01935	-3.13914	1.71359
C	-2.56130	-2.48486	0.32987
H	-2.84394	-3.54646	0.32286
H	-3.32204	-1.97487	0.93771
C	-2.62245	-1.95950	-1.11059
H	-2.24102	-0.93385	-1.14146
H	-1.94685	-2.56251	-1.73397
C	-4.03632	-1.99756	-1.69346
H	-4.45013	-3.01367	-1.66925
H	-4.71110	-1.35163	-1.11791
H	-4.05278	-1.65434	-2.73438
H	-3.44416	0.55504	1.34676

4e_conformer-207

C	-1.70788	2.53889	0.09769
C	-0.26138	0.83919	0.53895
N	-0.39661	2.13709	0.06461
H	0.38191	2.74313	-0.15201
C	-2.43759	1.46552	0.57735
C	-2.13146	3.90876	-0.32074
H	-3.21838	4.00059	-0.24636
H	-1.68513	4.68825	0.31058

H	-1.84476	4.12651	-1.35748
C	1.05196	0.19740	0.57693
C	1.36582	-0.77493	1.54940
C	2.04096	0.55207	-0.35193
C	2.62420	-1.36372	1.56781
H	0.63039	-1.04629	2.29734
C	3.31150	-0.03500	-0.31758
H	1.83489	1.26912	-1.14038
C	3.61249	-1.00562	0.64465
H	2.85483	-2.11023	2.32284
H	4.58627	-1.47761	0.68452
O	4.18268	0.39718	-1.27661
C	5.48354	-0.18129	-1.30282
H	6.02705	0.01562	-0.37090
H	5.43576	-1.26382	-1.47167
H	6.00539	0.29354	-2.13468
C	-1.54414	0.39134	0.86267
C	-1.97663	-0.97802	1.31902
H	-1.52372	-1.23053	2.28700
H	-3.05439	-0.94586	1.50365
C	-1.64446	-2.11693	0.32929
H	-0.55471	-2.23461	0.31124
H	-2.04723	-3.05763	0.73093
C	-2.12612	-1.93271	-1.11936
H	-1.88337	-0.91481	-1.45238
H	-1.53341	-2.60783	-1.75128
C	-3.60943	-2.22393	-1.40231
H	-3.73692	-2.28150	-2.49204
H	-3.85921	-3.22294	-1.01675
C	-4.61226	-1.20079	-0.85687
H	-4.33287	-0.18274	-1.15186
H	-5.61920	-1.40030	-1.24216
H	-4.67077	-1.22239	0.23543
H	-3.51225	1.44388	0.70413

4e_conformer-208

C	2.71543	-1.78591	0.05847
C	0.56264	-1.07212	0.24486
N	1.40920	-2.04335	-0.27373
H	1.11271	-2.77946	-0.89799
C	2.70900	-0.63814	0.82927
C	3.83890	-2.65590	-0.40175
H	3.71373	-3.69494	-0.07135
H	3.92374	-2.67046	-1.49614
H	4.78522	-2.28596	0.00179

C	-0.88438	-1.18458	0.07451
C	-1.49037	-2.43937	-0.12011
C	-1.70375	-0.03891	0.09916
C	-2.87184	-2.53550	-0.29189
H	-0.89035	-3.34365	-0.10402
C	-3.08655	-0.14806	-0.05858
H	-1.24518	0.92981	0.21561
C	-3.67903	-1.40298	-0.26093
H	-3.32644	-3.51116	-0.43960
H	-4.75478	-1.46526	-0.38865
O	-3.93936	0.91834	-0.04462
C	-3.38209	2.21414	0.15019
H	-2.85924	2.28388	1.11178
H	-2.68765	2.47572	-0.65764
H	-4.22459	2.90679	0.14289
C	1.36647	-0.16904	0.94525
C	0.95697	1.03957	1.74287
H	-0.00932	0.85399	2.22991
H	1.68720	1.16768	2.55279
C	0.85765	2.38846	0.99326
H	-0.00172	2.38335	0.31368
H	0.62775	3.15094	1.74931
C	2.10419	2.83791	0.21281
H	2.02705	3.92362	0.05895
H	2.99635	2.68241	0.83603
C	2.33106	2.18298	-1.16251
H	3.20511	2.66550	-1.62146
H	2.58994	1.12820	-1.03632
C	1.13964	2.29862	-2.11878
H	0.28834	1.69888	-1.77827
H	0.79947	3.33812	-2.21269
H	1.40764	1.94360	-3.12057
H	3.58191	-0.17190	1.26848

4e_conformer-209

C	0.74295	3.39594	0.05655
C	0.04029	1.23642	-0.04973
N	-0.33492	2.56920	-0.14493
H	-1.24918	2.88110	-0.43948
C	1.82912	2.57939	0.31496
C	0.62820	4.88312	-0.02577
H	-0.10778	5.27668	0.68693
H	1.59384	5.34387	0.19914
H	0.32247	5.21664	-1.02608
C	-0.94345	0.16454	-0.19465

C	-0.58752	-1.09329	-0.72539
C	-2.27677	0.37170	0.18869
C	-1.54216	-2.09673	-0.84351
H	0.42371	-1.26742	-1.07171
C	-3.23591	-0.63823	0.04988
H	-2.58961	1.31228	0.63122
C	-2.87170	-1.88893	-0.46321
H	-1.25637	-3.06093	-1.25491
H	-3.59617	-2.68649	-0.57165
O	-4.49699	-0.30942	0.45832
C	-5.50986	-1.30537	0.35868
H	-6.42279	-0.84212	0.73514
H	-5.66149	-1.61830	-0.68136
H	-5.26756	-2.18370	0.96898
C	1.40173	1.21930	0.25224
C	2.26959	0.01948	0.50556
H	2.92841	0.21841	1.36218
H	1.64287	-0.83162	0.78812
C	3.14420	-0.35634	-0.70491
H	2.49133	-0.57545	-1.56181
H	3.73094	0.52590	-0.99363
C	4.09596	-1.54087	-0.48457
H	4.70232	-1.64932	-1.39301
H	4.80222	-1.29853	0.32284
C	3.40975	-2.88870	-0.17432
H	2.43179	-2.92268	-0.67543
H	4.00047	-3.70055	-0.61752
C	3.23561	-3.18830	1.31931
H	2.62683	-2.43183	1.82388
H	2.75345	-4.16088	1.47264
H	4.20966	-3.21551	1.82299
H	2.83092	2.92261	0.54159

4e_conformer-210

C	-0.61792	3.26529	0.01837
C	0.10785	1.11278	-0.08551
N	0.47544	2.45026	-0.14093
H	1.40074	2.77420	-0.38327
C	-1.70861	2.43610	0.20888
C	-0.51206	4.75443	-0.03471
H	0.18011	5.14266	0.72339
H	-0.15483	5.10614	-1.01143
H	-1.49264	5.20375	0.14382
C	1.10826	0.05279	-0.19712
C	0.78825	-1.20737	-0.74531

C	2.42431	0.27455	0.23567
C	1.76056	-2.19677	-0.83513
H	-0.20799	-1.39473	-1.12629
C	3.40143	-0.72137	0.12577
H	2.70919	1.21611	0.69467
C	3.07303	-1.97351	-0.40743
H	1.50288	-3.16245	-1.26136
H	3.81170	-2.76050	-0.49501
O	4.64251	-0.37814	0.58116
C	5.67169	-1.35986	0.51253
H	5.86482	-1.66461	-0.52307
H	6.56419	-0.88672	0.92407
H	5.41964	-2.24499	1.10891
C	-1.26730	1.08072	0.14650
C	-2.14307	-0.12589	0.33127
H	-1.54459	-0.97935	0.67179
H	-2.86797	0.08254	1.12692
C	-2.90526	-0.51595	-0.95002
H	-3.58982	0.30291	-1.21173
H	-2.18741	-0.58195	-1.77874
C	-3.69263	-1.83206	-0.87537
H	-4.12627	-2.01116	-1.86781
H	-2.99533	-2.66378	-0.69606
C	-4.81915	-1.87815	0.17745
H	-5.26422	-0.87785	0.27640
H	-5.62259	-2.52852	-0.19178
C	-4.38765	-2.39321	1.55589
H	-3.98199	-3.40941	1.47771
H	-3.61457	-1.76535	2.00882
H	-5.23781	-2.42612	2.24735
H	-2.72377	2.76725	0.38936

4e_conformer-211

C	3.78597	-0.39687	-0.12015
C	1.53382	-0.65170	0.04633
N	2.74848	-1.29688	-0.13658
H	2.84260	-2.27572	-0.36696
C	3.23020	0.84966	0.10722
C	5.20414	-0.81669	-0.33024
H	5.86501	0.05016	-0.24585
H	5.52529	-1.55929	0.41135
H	5.35842	-1.26058	-1.32245
C	0.27808	-1.39704	0.10468
C	0.23922	-2.71898	0.59894
C	-0.92103	-0.81358	-0.32489

C	-0.96709	-3.40678	0.65954
H	1.14756	-3.18892	0.96339
C	-2.13491	-1.50360	-0.23830
H	-0.93104	0.17702	-0.76008
C	-2.16745	-2.81384	0.25245
H	-0.98627	-4.42219	1.04570
H	-3.09490	-3.36879	0.32064
O	-3.22941	-0.81143	-0.67425
C	-4.49378	-1.46410	-0.62150
H	-5.21766	-0.74591	-1.00863
H	-4.50390	-2.36574	-1.24576
H	-4.76097	-1.73354	0.40740
C	1.81518	0.70432	0.21469
C	0.83347	1.80632	0.49546
H	-0.06684	1.37898	0.94633
H	1.25551	2.49645	1.23883
C	0.45264	2.61473	-0.75916
H	1.36823	3.05468	-1.17654
H	0.07582	1.92615	-1.52841
C	-0.58366	3.72563	-0.53466
H	-0.19658	4.44697	0.19969
H	-0.68078	4.27898	-1.47755
C	-1.98346	3.24565	-0.09033
H	-2.74087	3.90738	-0.52971
H	-2.18123	2.24846	-0.50814
C	-2.20174	3.21930	1.42718
H	-2.06582	4.22043	1.85440
H	-3.21720	2.88666	1.67235
H	-1.50138	2.54872	1.93453
H	3.78529	1.77466	0.20205

4e_conformer-212

C	0.82871	3.45946	-0.09055
C	0.14936	1.29559	-0.27123
N	-0.25235	2.62294	-0.20847
H	-1.20148	2.93403	-0.35847
C	1.95159	2.65148	-0.04807
C	0.68300	4.94542	-0.04473
H	0.23918	5.34360	-0.96659
H	0.04600	5.26846	0.78855
H	1.66385	5.41150	0.08186
C	-0.83835	0.22111	-0.36456
C	-0.53510	-1.00767	-0.98885
C	-2.12633	0.39582	0.16307
C	-1.49368	-2.01077	-1.06170

H	0.43937	-1.16277	-1.43437
C	-3.09139	-0.61431	0.07226
H	-2.39765	1.30856	0.68412
C	-2.77917	-1.83378	-0.53928
H	-1.24683	-2.95128	-1.54657
H	-3.50805	-2.63119	-0.61329
O	-4.30419	-0.31667	0.62540
C	-5.31723	-1.31654	0.58460
H	-5.57671	-1.58048	-0.44771
H	-5.00732	-2.22090	1.12198
H	-6.18733	-0.88111	1.07763
C	1.54256	1.29136	-0.16568
C	2.46183	0.10311	-0.07094
H	2.34356	-0.54864	-0.94244
H	3.50011	0.45749	-0.10350
C	2.25164	-0.71470	1.21619
H	2.40614	-0.04693	2.07410
H	1.20354	-1.03645	1.26196
C	3.15469	-1.94761	1.36356
H	4.20985	-1.63748	1.35747
H	2.97141	-2.37237	2.35890
C	2.93536	-3.04603	0.29971
H	3.06007	-4.03036	0.76872
H	1.89283	-3.00919	-0.04693
C	3.88454	-2.96899	-0.90202
H	3.79593	-2.01834	-1.43752
H	4.92760	-3.06569	-0.57658
H	3.68230	-3.77455	-1.61770
H	2.97135	2.99899	0.06189

4e_conformer-213

C	-0.77035	3.04575	-0.14313
C	0.14033	0.96257	-0.10361
N	0.38740	2.31838	-0.27056
H	1.27457	2.70079	-0.56481
C	-1.77873	2.14309	0.14118
C	-0.79815	4.52946	-0.31399
H	-0.12448	5.03504	0.38961
H	-0.49772	4.83180	-1.32574
H	-1.80997	4.90521	-0.13972
C	1.22851	-0.01184	-0.16501
C	1.01166	-1.32900	-0.62143
C	2.52641	0.35243	0.22346
C	2.06543	-2.23452	-0.66627
H	0.02929	-1.62615	-0.96749

C	3.58538	-0.56047	0.15892
H	2.73412	1.34437	0.61247
C	3.35977	-1.86965	-0.28281
H	1.88714	-3.24562	-1.02194
H	4.16322	-2.59390	-0.33375
O	4.79853	-0.08109	0.56369
C	5.90846	-0.97277	0.53913
H	6.11241	-1.32909	-0.47779
H	6.76209	-0.39775	0.90028
H	5.74332	-1.83403	1.19764
C	-1.22086	0.82964	0.16961
C	-1.98724	-0.42544	0.47561
H	-1.30616	-1.21009	0.82579
H	-2.67855	-0.22964	1.30301
C	-2.78235	-0.96091	-0.73264
H	-3.42869	-0.16118	-1.11990
H	-2.07003	-1.18070	-1.53828
C	-3.62715	-2.21443	-0.42845
H	-3.62967	-2.86874	-1.30956
H	-3.14436	-2.79091	0.37421
C	-5.09008	-1.93636	-0.04783
H	-5.58735	-1.44614	-0.89679
H	-5.60199	-2.89813	0.09119
C	-5.28638	-1.08120	1.20750
H	-4.77392	-1.52173	2.07162
H	-4.89624	-0.06687	1.07018
H	-6.34985	-0.99285	1.45795
H	-2.81511	2.39823	0.32474

4e_conformer-214

C	-2.63514	-2.11672	-0.35434
C	-0.57530	-1.25313	0.07256
N	-1.28282	-2.31723	-0.47092
H	-0.85563	-3.06596	-0.99742
C	-2.80316	-0.91600	0.30997
C	-3.63271	-3.08133	-0.90743
H	-4.64570	-2.74877	-0.66521
H	-3.56035	-3.16666	-1.99964
H	-3.50022	-4.08901	-0.49341
C	0.88976	-1.29894	0.10584
C	1.54601	-2.52529	0.30856
C	1.66370	-0.13721	-0.08575
C	2.94063	-2.58450	0.32204
H	0.96490	-3.42524	0.48364
C	3.05896	-0.20535	-0.05143

H	1.16392	0.79868	-0.29220
C	3.70347	-1.43544	0.15040
H	3.43714	-3.53748	0.48231
H	4.78815	-1.46349	0.16626
O	3.88525	0.86796	-0.22265
C	3.29107	2.14678	-0.41534
H	4.11857	2.85250	-0.49882
H	2.69336	2.17589	-1.33390
H	2.65765	2.42427	0.43518
C	-1.51589	-0.35819	0.58347
C	-1.26438	0.88786	1.39509
H	-1.27774	0.63138	2.46545
H	-0.25989	1.26883	1.20106
C	-2.31032	1.99860	1.17265
H	-3.28353	1.61305	1.50026
H	-2.08087	2.83499	1.84696
C	-2.44210	2.52357	-0.27162
H	-2.22562	1.70803	-0.97432
H	-3.48853	2.80382	-0.44831
C	-1.57081	3.74139	-0.61379
H	-1.84411	4.09226	-1.61810
H	-1.81755	4.56249	0.07412
C	-0.06248	3.48588	-0.57494
H	0.20553	2.64933	-1.23189
H	0.49923	4.36544	-0.90986
H	0.28026	3.24372	0.43643
H	-3.75907	-0.48306	0.57337

4e_conformer-215

C	3.83997	-0.20684	-0.30288
C	1.59126	-0.51190	-0.14556
N	2.83418	-1.12974	-0.16471
H	2.96910	-2.13018	-0.19790
C	3.23358	1.03673	-0.34646
C	5.27727	-0.60374	-0.39216
H	5.60159	-1.16853	0.49121
H	5.47649	-1.23158	-1.27051
H	5.90503	0.28798	-0.46991
C	0.36856	-1.29256	0.03363
C	0.38982	-2.52701	0.71927
C	-0.85924	-0.83325	-0.46169
C	-0.78380	-3.25042	0.89623
H	1.31749	-2.89813	1.14377
C	-2.03987	-1.55396	-0.25672
H	-0.92281	0.08886	-1.02439

C	-2.01188	-2.77820	0.42117
H	-0.75414	-4.19621	1.43021
H	-2.91390	-3.35438	0.58566
O	-3.16910	-0.97658	-0.76644
C	-4.40425	-1.66314	-0.59200
H	-4.38694	-2.64561	-1.07898
H	-4.64344	-1.79034	0.47061
H	-5.16343	-1.03742	-1.06295
C	1.82256	0.86229	-0.25397
C	0.81406	1.97863	-0.18999
H	1.32429	2.92772	-0.39825
H	0.06710	1.86660	-0.98209
C	0.11030	2.07894	1.17604
H	-0.32587	1.10360	1.42383
H	0.87641	2.27513	1.93795
C	-0.98846	3.14746	1.27422
H	-1.29435	3.20521	2.32682
H	-0.57083	4.13466	1.02760
C	-2.24040	2.88670	0.40645
H	-2.38094	1.80385	0.28277
H	-3.12695	3.24266	0.94700
C	-2.22337	3.56554	-0.96841
H	-2.13839	4.65395	-0.86139
H	-1.38331	3.23127	-1.58543
H	-3.14590	3.35562	-1.52241
H	3.75068	1.98441	-0.43172

4e_conformer-216

C	-2.89547	2.17086	-0.30602
C	-1.51835	0.36895	-0.11526
N	-2.83402	0.81236	-0.12722
H	-3.63749	0.20052	-0.12983
C	-1.58855	2.61837	-0.38620
C	-4.19402	2.90365	-0.39425
H	-4.00930	3.97442	-0.51504
H	-4.79718	2.56887	-1.24829
H	-4.80373	2.76620	0.50791
C	-1.21353	-1.04635	0.08678
C	-2.11231	-1.88894	0.76512
C	-0.01168	-1.59715	-0.40229
C	-1.81355	-3.24083	0.94216
H	-3.02785	-1.48527	1.18587
C	0.28636	-2.94536	-0.20074
H	0.67032	-0.96564	-0.95192
C	-0.62153	-3.77896	0.46964

H	-2.51564	-3.87980	1.47054
H	-0.37213	-4.82548	0.61121
O	1.43499	-3.54467	-0.63319
C	2.41207	-2.73000	-1.27259
H	3.25058	-3.38979	-1.49913
H	2.02673	-2.29747	-2.20384
H	2.74982	-1.92149	-0.61250
C	-0.71197	1.50051	-0.27384
C	0.79087	1.59780	-0.24900
H	1.24356	0.99639	-1.04904
H	1.06321	2.63493	-0.47309
C	1.40965	1.18973	1.10086
H	1.07408	1.90442	1.86499
H	1.00389	0.21442	1.39323
C	2.94225	1.10330	1.12169
H	3.23687	0.75072	2.11864
H	3.27208	0.32443	0.41741
C	3.69395	2.41430	0.81324
H	3.11647	3.26357	1.20534
H	4.64303	2.41659	1.36465
C	4.00729	2.64038	-0.67078
H	3.10137	2.69819	-1.28136
H	4.56740	3.57141	-0.81708
H	4.61781	1.81866	-1.06506
H	-1.28455	3.65054	-0.50862

4e_conformer-217

C	-1.63636	2.48976	0.08670
C	-0.28749	0.66976	0.30997
N	-0.40333	1.95861	-0.19555
H	0.28821	2.40255	-0.78201
C	-2.32275	1.53317	0.81121
C	-2.03915	3.85229	-0.37371
H	-1.35911	4.62837	0.00004
H	-2.04898	3.92814	-1.46882
H	-3.04506	4.08425	-0.01379
C	0.97667	-0.05618	0.19913
C	1.03392	-1.46524	0.23649
C	2.18238	0.64849	0.05237
C	2.25517	-2.12066	0.13066
H	0.12376	-2.04176	0.32416
C	3.40598	-0.01903	-0.06900
H	2.20235	1.73351	0.06075
C	3.45296	-1.41751	-0.02717
H	2.28142	-3.20656	0.15779

H	4.38940	-1.95435	-0.11351
O	4.50063	0.78580	-0.20945
C	5.77593	0.16162	-0.31728
H	6.00802	-0.42970	0.57665
H	5.83131	-0.48389	-1.20209
H	6.49869	0.97281	-0.41406
C	-1.49619	0.37838	0.94802
C	-1.88961	-0.86176	1.70360
H	-1.01624	-1.26607	2.23172
H	-2.60761	-0.56861	2.48107
C	-2.52601	-2.01387	0.89314
H	-1.78737	-2.44535	0.20869
H	-2.76298	-2.80958	1.61207
C	-3.80390	-1.68468	0.10310
H	-4.30819	-2.63608	-0.11854
H	-4.49235	-1.12014	0.74826
C	-3.62508	-0.92689	-1.22577
H	-4.60870	-0.87069	-1.71243
H	-3.31610	0.10349	-1.03075
C	-2.62685	-1.57444	-2.19052
H	-2.85724	-2.63504	-2.35555
H	-2.64554	-1.07356	-3.16537
H	-1.60160	-1.51071	-1.80980
H	-3.32556	1.64290	1.20444

4e_conformer-218

C	3.65070	-0.32150	-0.26183
C	1.42925	-0.62746	0.11730
N	2.59116	-1.18331	-0.40230
H	2.61720	-2.04197	-0.93347
C	3.16940	0.79728	0.39107
C	5.01472	-0.65082	-0.77402
H	5.02295	-0.78170	-1.86401
H	5.70936	0.15716	-0.52925
H	5.40456	-1.57683	-0.33255
C	0.17827	-1.38632	0.08606
C	0.18860	-2.79087	0.20771
C	-1.05740	-0.74757	-0.10812
C	-0.99482	-3.52180	0.13030
H	1.12774	-3.30741	0.38213
C	-2.23777	-1.48647	-0.17242
H	-1.10108	0.32161	-0.25849
C	-2.21992	-2.87693	-0.05349
H	-0.96492	-4.60355	0.22511
H	-3.15115	-3.43060	-0.11624

O	-3.43684	-0.84569	-0.42398
C	-4.00708	-0.20773	0.72161
H	-4.25127	-0.94465	1.49738
H	-3.32564	0.54121	1.14172
H	-4.92040	0.28442	0.38190
C	1.77275	0.62046	0.63648
C	0.92065	1.59826	1.40069
H	-0.05917	1.16126	1.62146
H	1.39274	1.78710	2.37458
C	0.75032	2.95586	0.68239
H	0.37739	3.69814	1.40148
H	1.74168	3.30311	0.36628
C	-0.19546	2.91021	-0.52629
H	-0.00074	3.76749	-1.18435
H	0.02680	2.01538	-1.12503
C	-1.67780	2.92936	-0.12889
H	-1.86114	2.18869	0.66079
H	-1.90571	3.90439	0.32280
C	-2.62837	2.66594	-1.29880
H	-2.45010	3.37363	-2.11764
H	-2.49653	1.65267	-1.69563
H	-3.67551	2.76494	-0.99108
H	3.76333	1.65481	0.68160

4e_conformer-219

C	-0.91473	3.16060	-0.01669
C	0.00747	1.11425	0.35878
N	0.25317	2.44332	0.04115
H	1.17585	2.85106	-0.00901
C	-1.93961	2.26795	0.24258
C	-0.93376	4.62701	-0.30037
H	-0.39929	5.20117	0.46776
H	-0.46756	4.86296	-1.26551
H	-1.96569	4.98705	-0.32870
C	1.10924	0.15684	0.44666
C	1.03017	-0.98383	1.27325
C	2.28633	0.36217	-0.28858
C	2.09524	-1.87346	1.34101
H	0.14569	-1.15552	1.87422
C	3.36068	-0.53125	-0.20353
H	2.38250	1.20378	-0.96727
C	3.27131	-1.66427	0.61303
H	2.02196	-2.74701	1.98300
H	4.08729	-2.37218	0.68799
O	4.44679	-0.21519	-0.96915

C	5.56213	-1.09985	-0.93911
H	5.28685	-2.10144	-1.29087
H	6.30379	-0.66914	-1.61306
H	5.98561	-1.17479	0.06971
C	-1.37770	0.98059	0.48644
C	-2.18664	-0.26802	0.71586
H	-1.90180	-0.76482	1.65259
H	-3.23316	0.02451	0.84797
C	-2.07456	-1.29116	-0.43167
H	-2.32862	-0.79446	-1.37815
H	-1.02303	-1.58720	-0.51723
C	-2.95151	-2.54546	-0.24600
H	-2.42597	-3.41574	-0.65988
H	-3.07093	-2.74869	0.82843
C	-4.33664	-2.47723	-0.90859
H	-4.20231	-2.37230	-1.99453
H	-4.84313	-3.43986	-0.75588
C	-5.24550	-1.35100	-0.40714
H	-4.83640	-0.36410	-0.64908
H	-6.23872	-1.41631	-0.86627
H	-5.37547	-1.40104	0.68103
H	-2.99535	2.50911	0.25244

4e_conformer-220

C	-1.30299	3.28725	-0.29673
C	0.10179	1.50418	-0.12970
N	-0.01822	2.88436	-0.03572
H	0.76215	3.51358	0.08679
C	-2.03540	2.13878	-0.54041
C	-1.70001	4.72720	-0.29996
H	-2.76907	4.81769	-0.51018
H	-1.50457	5.20787	0.66713
H	-1.15828	5.30072	-1.06342
C	1.38186	0.85046	0.13628
C	2.35246	1.46497	0.94765
C	1.67147	-0.41147	-0.42102
C	3.57312	0.83229	1.18821
H	2.14440	2.41937	1.42078
C	2.88504	-1.04595	-0.15366
H	0.94694	-0.87686	-1.07242
C	3.84975	-0.41977	0.64994
H	4.31257	1.31758	1.81917
H	4.78913	-0.92824	0.84092
O	3.22500	-2.27534	-0.64212
C	2.25300	-2.97975	-1.40832

H	2.70448	-3.94221	-1.65250
H	1.33295	-3.14142	-0.83316
H	2.00974	-2.44635	-2.33521
C	-1.16869	1.01155	-0.44541
C	-1.62112	-0.41864	-0.57779
H	-2.66154	-0.41556	-0.91736
H	-1.06239	-0.94276	-1.36497
C	-1.50153	-1.22830	0.72866
H	-0.46161	-1.17002	1.06751
H	-2.10320	-0.73969	1.50736
C	-1.91018	-2.70875	0.59812
H	-1.67424	-3.06055	-0.41733
H	-1.28970	-3.30964	1.27568
C	-3.38145	-3.01943	0.91570
H	-3.52850	-4.10573	0.84631
H	-3.57951	-2.75158	1.96322
C	-4.40729	-2.32038	0.01858
H	-4.21208	-2.52513	-1.04136
H	-5.42308	-2.66482	0.24460
H	-4.39131	-1.23404	0.15691
H	-3.09482	2.10461	-0.76231

4e_conformer-221

C	1.30357	3.28702	-0.29676
C	-0.10154	1.50422	-0.12974
N	0.01874	2.88437	-0.03573
H	-0.76151	3.51373	0.08679
C	2.03577	2.13841	-0.54048
C	1.70087	4.72689	-0.29999
H	2.76996	4.81718	-0.51014
H	1.15930	5.30051	-1.06349
H	1.50546	5.20761	0.66708
C	-1.38170	0.85069	0.13629
C	-2.35212	1.46529	0.94781
C	-1.67155	-0.41115	-0.42108
C	-3.57286	0.83277	1.18843
H	-2.14387	2.41962	1.42099
C	-2.88518	-1.04547	-0.15365
H	-0.94714	-0.87658	-1.07260
C	-3.84972	-0.41921	0.65009
H	-4.31217	1.31811	1.81951
H	-4.78916	-0.92755	0.84112
O	-3.22538	-2.27477	-0.64219
C	-2.25359	-2.97925	-1.40858
H	-2.70524	-3.94161	-1.65281

H	-2.01037	-2.44578	-2.33544
H	-1.33349	-3.14112	-0.83356
C	1.16884	1.01134	-0.44548
C	1.62096	-0.41894	-0.57785
H	1.06207	-0.94298	-1.36497
H	2.66136	-0.41611	-0.91748
C	1.50127	-1.22853	0.72864
H	2.10304	-0.73998	1.50730
H	0.46137	-1.17004	1.06752
C	1.90966	-2.70905	0.59816
H	1.28906	-3.30981	1.27573
H	1.67368	-3.06084	-0.41728
C	3.38088	-3.01996	0.91578
H	3.57895	-2.75214	1.96329
H	3.52775	-4.10629	0.84640
C	4.40684	-2.32108	0.01867
H	4.39102	-1.23474	0.15698
H	5.42258	-2.66567	0.24473
H	4.21164	-2.52583	-1.04126
H	3.09517	2.10405	-0.76240

4e_conformer-222

C	-1.60037	2.71199	0.23713
C	0.12056	1.25270	-0.04872
N	-0.24064	2.54331	0.31245
H	0.40146	3.22744	0.68591
C	-2.12551	1.51201	-0.20743
C	-2.26365	3.99808	0.60750
H	-3.34004	3.92596	0.43002
H	-2.11462	4.24707	1.66633
H	-1.87980	4.84058	0.01825
C	1.52846	0.86329	-0.10728
C	2.52543	1.81780	-0.37862
C	1.91534	-0.47419	0.10931
C	3.86759	1.43907	-0.42822
H	2.25080	2.84774	-0.58377
C	3.25945	-0.84553	0.03689
H	1.16201	-1.20442	0.36316
C	4.24599	0.11552	-0.22806
H	4.62794	2.18546	-0.64078
H	5.28535	-0.19329	-0.27189
O	3.70774	-2.12142	0.22687
C	2.74199	-3.13595	0.48353
H	3.30247	-4.06760	0.57063
H	2.20178	-2.94653	1.41904

H	2.02064	-3.21756	-0.33859
C	-1.05740	0.58161	-0.38651
C	-1.20268	-0.82367	-0.90258
H	-1.99433	-0.83941	-1.65812
H	-0.28226	-1.12184	-1.41890
C	-1.50878	-1.88452	0.18226
H	-1.49113	-2.87891	-0.28624
H	-0.68403	-1.87526	0.90619
C	-2.82727	-1.71167	0.95898
H	-2.70401	-2.17584	1.94641
H	-3.00100	-0.64483	1.14920
C	-4.07669	-2.33482	0.31559
H	-3.90712	-3.41296	0.18315
H	-4.90583	-2.24463	1.03053
C	-4.51659	-1.72938	-1.02142
H	-3.79495	-1.93277	-1.81900
H	-5.47979	-2.14604	-1.33833
H	-4.63315	-0.64130	-0.94597
H	-3.17299	1.32305	-0.40333

4e_conformer-223

C	-0.68960	3.31837	0.00867
C	0.03015	1.17610	0.27637
N	0.40793	2.50211	0.11363
H	1.35916	2.83289	0.19007
C	-1.80086	2.49697	0.07914
C	-0.56794	4.80020	-0.13481
H	-0.07601	5.25810	0.73333
H	0.01405	5.07886	-1.02257
H	-1.56106	5.24734	-0.23009
C	1.03961	0.12369	0.38367
C	0.78077	-1.07771	1.07698
C	2.30501	0.29329	-0.19816
C	1.76098	-2.05818	1.16733
H	-0.17717	-1.22844	1.55898
C	3.29195	-0.69348	-0.09037
H	2.54138	1.18200	-0.77478
C	3.02482	-1.88498	0.59342
H	1.54887	-2.97774	1.70586
H	3.77127	-2.66437	0.68261
O	4.47901	-0.40345	-0.70064
C	5.51276	-1.38098	-0.64294
H	5.81151	-1.58590	0.39214
H	5.20360	-2.31774	-1.12213
H	6.35715	-0.95592	-1.18708

C	-1.36705	1.15034	0.25234
C	-2.28608	-0.04185	0.29277
H	-2.15321	-0.61860	1.21779
H	-3.31616	0.32983	0.32168
C	-2.11752	-0.98181	-0.91509
H	-2.38383	-0.42642	-1.82512
H	-1.05657	-1.24048	-1.01207
C	-2.93213	-2.28177	-0.85489
H	-2.67194	-2.87248	-1.74291
H	-2.60310	-2.87678	0.00994
C	-4.46425	-2.11018	-0.80050
H	-4.74943	-1.21727	-1.37486
H	-4.93534	-2.95913	-1.31257
C	-5.04943	-2.02579	0.61443
H	-4.81207	-2.93272	1.18409
H	-4.65441	-1.17354	1.17542
H	-6.14097	-1.92766	0.58402
H	-2.83030	2.82559	0.00783

4e_conformer-224

C	1.26568	2.34532	0.29528
C	0.01745	0.44318	0.30722
N	0.07864	1.77305	-0.08919
H	-0.60834	2.22193	-0.67769
C	1.97241	1.37321	0.97863
C	1.61167	3.75915	-0.03957
H	1.68714	3.91711	-1.12339
H	0.86232	4.46513	0.34051
H	2.57634	4.02072	0.40347
C	-1.18500	-0.35244	0.06736
C	-1.12719	-1.74608	-0.09573
C	-2.44221	0.28637	-0.00378
C	-2.29664	-2.47670	-0.31444
H	-0.17088	-2.25241	-0.08302
C	-3.60257	-0.45256	-0.24063
H	-2.50204	1.35427	0.16504
C	-3.53298	-1.84583	-0.39275
H	-2.23841	-3.55427	-0.44072
H	-4.44759	-2.40243	-0.56955
O	-4.85304	0.08958	-0.32455
C	-4.98159	1.50060	-0.18487
H	-4.65076	1.83620	0.80552
H	-4.41021	2.03169	-0.95579
H	-6.04375	1.71759	-0.30503
C	1.20626	0.16875	0.98577

C	1.62395	-1.11293	1.65158
H	2.20839	-0.85848	2.54580
H	0.73855	-1.65539	2.00556
C	2.47938	-2.07818	0.79752
H	2.76490	-2.91615	1.44633
H	1.86761	-2.51143	-0.00504
C	3.73864	-1.45006	0.17290
H	4.13343	-0.67472	0.84466
H	4.52717	-2.21116	0.09600
C	3.50981	-0.84985	-1.22070
H	3.23421	-1.65943	-1.91155
H	2.65613	-0.16536	-1.18765
C	4.73815	-0.11027	-1.75403
H	4.99050	0.73982	-1.10793
H	5.61485	-0.76926	-1.79158
H	4.56773	0.27778	-2.76495
H	2.94520	1.51020	1.43313

4e_conformer-225

C	3.53970	-0.47870	0.43653
C	1.34358	-0.68414	-0.11136
N	2.45593	-1.32448	0.41470
H	2.43161	-2.24586	0.82833
C	3.12226	0.71608	-0.11739
C	4.86160	-0.89690	0.99327
H	5.58858	-0.08926	0.87172
H	4.80105	-1.13444	2.06352
H	5.25711	-1.78575	0.48528
C	0.07432	-1.41345	-0.24790
C	0.05309	-2.68131	-0.84945
C	-1.12246	-0.87474	0.25952
C	-1.14788	-3.38593	-0.95587
H	0.97135	-3.10001	-1.24943
C	-2.31832	-1.59144	0.15665
H	-1.09246	0.08327	0.75979
C	-2.33264	-2.84995	-0.46266
H	-1.16027	-4.36347	-1.42968
H	-3.27149	-3.39029	-0.52921
O	-3.51727	-1.16085	0.64905
C	-3.52393	-0.01562	1.49731
H	-4.54669	0.07689	1.86516
H	-2.83922	-0.14631	2.34377
H	-3.25411	0.89417	0.95160
C	1.74036	0.59972	-0.47054
C	0.95002	1.63583	-1.22304

H	1.44878	1.82652	-2.18409
H	-0.03855	1.23663	-1.46770
C	0.81117	2.98236	-0.47852
H	1.79736	3.25760	-0.08570
H	0.54256	3.76772	-1.19786
C	-0.21971	2.96393	0.66464
H	-0.25704	1.95371	1.09290
H	0.11878	3.61623	1.47961
C	-1.63180	3.42252	0.25774
H	-2.30410	3.28912	1.11636
H	-1.60222	4.50366	0.06273
C	-2.23469	2.72917	-0.96970
H	-2.27107	1.64083	-0.85136
H	-3.25804	3.07950	-1.14640
H	-1.65437	2.93962	-1.87441
H	3.75113	1.58342	-0.27546

4e_conformer-226

C	2.72140	-1.85338	0.04539
C	0.61589	-1.01123	0.24146
N	1.40131	-2.02983	-0.28385
H	1.06093	-2.74391	-0.91111
C	2.78670	-0.71068	0.82086
C	3.78873	-2.78731	-0.42291
H	3.59981	-3.81932	-0.10086
H	3.87218	-2.79812	-1.51746
H	4.75632	-2.47986	-0.01731
C	-0.83722	-1.04524	0.08551
C	-1.50482	-2.27328	-0.12508
C	-1.61027	0.12230	0.14139
C	-2.88488	-2.30626	-0.27944
H	-0.94549	-3.20324	-0.13320
C	-3.00252	0.07875	0.00041
H	-1.14810	1.09095	0.27157
C	-3.65433	-1.13982	-0.21820
H	-3.38288	-3.25882	-0.43803
H	-4.72934	-1.19335	-0.33687
O	-3.63346	1.28793	0.07784
C	-5.04807	1.31131	-0.08108
H	-5.34538	0.93273	-1.06651
H	-5.54746	0.72269	0.69791
H	-5.34227	2.35770	0.00998
C	1.47565	-0.16131	0.94202
C	1.13919	1.06839	1.74046
H	0.16733	0.93431	2.23337

H	1.88014	1.16065	2.54569
C	1.10276	2.41628	0.98489
H	0.26779	2.42785	0.27579
H	0.86770	3.18776	1.73030
C	2.38714	2.83378	0.24894
H	2.33165	3.91816	0.07682
H	3.25045	2.67602	0.91126
C	2.66296	2.15660	-1.10650
H	3.55299	2.63192	-1.54174
H	2.91638	1.10391	-0.95478
C	1.50601	2.25477	-2.10576
H	0.64352	1.66193	-1.78197
H	1.17027	3.29241	-2.23112
H	1.80839	1.88072	-3.09075
H	3.68744	-0.29975	1.25918

4e_conformer-227

C	-0.02917	3.48364	-0.13846
C	-0.13392	1.22945	-0.41776
N	-0.86313	2.40186	-0.27997
H	-1.86387	2.46208	-0.40515
C	1.26373	2.99090	-0.15267
C	-0.55020	4.87865	-0.02039
H	0.28071	5.57517	0.12058
H	-1.09968	5.19022	-0.91844
H	-1.23189	4.99005	0.83249
C	-0.80919	-0.06170	-0.55033
C	-0.28443	-1.07644	-1.36562
C	-2.01645	-0.29653	0.13916
C	-0.94245	-2.30305	-1.46989
H	0.61667	-0.89398	-1.93860
C	-2.67639	-1.52149	0.01330
H	-2.40656	0.47374	0.79301
C	-2.13345	-2.53548	-0.79024
H	-0.52703	-3.08257	-2.10240
H	-2.65770	-3.48248	-0.86717
O	-3.84580	-1.83061	0.64609
C	-4.44317	-0.83593	1.47161
H	-5.36106	-1.28025	1.85853
H	-3.78809	-0.56495	2.30840
H	-4.68721	0.06565	0.89689
C	1.21442	1.57633	-0.33194
C	2.40457	0.65697	-0.26049
H	2.41429	-0.05760	-1.08906
H	3.31555	1.25857	-0.37388

C	2.48501	-0.10121	1.08052
H	2.46702	0.64847	1.88147
H	1.57512	-0.70275	1.20816
C	3.73166	-0.99591	1.23760
H	4.55952	-0.57120	0.65092
H	4.06042	-0.96821	2.28449
C	3.53804	-2.46993	0.84883
H	4.46901	-3.01092	1.06620
H	2.77050	-2.90914	1.50186
C	3.14779	-2.71153	-0.61200
H	3.86445	-2.24190	-1.29719
H	3.12206	-3.78394	-0.83818
H	2.15544	-2.30682	-0.83239
H	2.16237	3.58423	-0.03691

4e_conformer-228

C	-2.02962	2.91616	-0.25751
C	-0.30541	1.43546	-0.14037
N	-0.69388	2.75976	0.01263
H	-0.05212	3.52231	0.17572
C	-2.52005	1.65943	-0.56543
C	-2.70025	4.24993	-0.20690
H	-3.76584	4.13889	-0.42483
H	-2.60506	4.71851	0.78098
H	-2.27922	4.94980	-0.94058
C	1.07602	1.03454	0.12199
C	1.88015	1.76898	1.02120
C	1.64263	-0.07756	-0.51555
C	3.19593	1.38867	1.25681
H	1.46281	2.61484	1.55853
C	2.96346	-0.46316	-0.26116
H	1.07542	-0.65558	-1.23466
C	3.75635	0.27284	0.62743
H	3.80180	1.96007	1.95463
H	4.78173	-0.00701	0.83440
O	3.39090	-1.56729	-0.94274
C	4.73257	-1.99764	-0.73847
H	5.45027	-1.22389	-1.03680
H	4.90966	-2.27027	0.30891
H	4.86394	-2.87749	-1.36967
C	-1.44993	0.71991	-0.50223
C	-1.61184	-0.76468	-0.69136
H	-2.62500	-0.95180	-1.06115
H	-0.94118	-1.14511	-1.47305
C	-1.37039	-1.57740	0.59602

H	-0.34360	-1.38642	0.92747
H	-2.02330	-1.18938	1.38990
C	-1.58697	-3.09513	0.43388
H	-1.35053	-3.38667	-0.60006
H	-0.86877	-3.63011	1.06861
C	-2.99423	-3.59638	0.79527
H	-3.01064	-4.68995	0.69358
H	-3.18051	-3.38623	1.85804
C	-4.13325	-3.00148	-0.03815
H	-3.96443	-3.15955	-1.11054
H	-5.09170	-3.46552	0.22184
H	-4.23482	-1.92356	0.12806
H	-3.55001	1.42923	-0.80848

4e_conformer-229

C	2.02960	2.91617	-0.25751
C	0.30540	1.43545	-0.14038
N	0.69385	2.75976	0.01263
H	0.05209	3.52231	0.17572
C	2.52004	1.65944	-0.56543
C	2.70022	4.24994	-0.20690
H	3.76581	4.13891	-0.42483
H	2.27918	4.94982	-0.94058
H	2.60503	4.71852	0.78098
C	-1.07603	1.03453	0.12199
C	-1.88016	1.76897	1.02120
C	-1.64263	-0.07757	-0.51555
C	-3.19594	1.38865	1.25682
H	-1.46282	2.61482	1.55853
C	-2.96346	-0.46318	-0.26116
H	-1.07543	-0.65558	-1.23466
C	-3.75635	0.27282	0.62743
H	-3.80181	1.96004	1.95464
H	-4.78174	-0.00704	0.83441
O	-3.39091	-1.56731	-0.94274
C	-4.73257	-1.99766	-0.73847
H	-4.86394	-2.87751	-1.36967
H	-4.90966	-2.27030	0.30891
H	-5.45028	-1.22391	-1.03679
C	1.44992	0.71991	-0.50224
C	1.61184	-0.76468	-0.69136
H	0.94118	-1.14511	-1.47304
H	2.62500	-0.95179	-1.06116
C	1.37041	-1.57739	0.59603
H	2.02333	-1.18936	1.38990

H	0.34363	-1.38642	0.92748
C	1.58700	-3.09512	0.43389
H	0.86882	-3.63010	1.06863
H	1.35056	-3.38667	-0.60005
C	2.99427	-3.59635	0.79526
H	3.18056	-3.38620	1.85803
H	3.01070	-4.68993	0.69359
C	4.13328	-3.00145	-0.03817
H	3.96445	-3.15952	-1.11055
H	4.23484	-1.92352	0.12803
H	5.09174	-3.46548	0.22182
H	3.54999	1.42925	-0.80848

4e_conformer-230

C	0.81157	3.44390	-0.39485
C	-0.34607	1.51555	-0.07459
N	-0.43408	2.89094	-0.23645
H	-1.30257	3.39576	-0.34004
C	1.72432	2.40729	-0.30636
C	1.00887	4.90712	-0.62190
H	0.59892	5.50792	0.20007
H	2.07619	5.13045	-0.70126
H	0.52518	5.24846	-1.54646
C	-1.54040	0.70150	0.13406
C	-2.68655	1.24202	0.74398
C	-1.56557	-0.64512	-0.28248
C	-3.82182	0.45242	0.93031
H	-2.67989	2.26694	1.10171
C	-2.69935	-1.43197	-0.06952
H	-0.70669	-1.05191	-0.79633
C	-3.83899	-0.88148	0.53526
H	-4.70074	0.87983	1.40492
H	-4.71175	-1.50876	0.68474
O	-2.79507	-2.74524	-0.43241
C	-1.64694	-3.36558	-1.00213
H	-1.36979	-2.89902	-1.95522
H	-0.79090	-3.32373	-0.31778
H	-1.92381	-4.40623	-1.17577
C	1.01083	1.18782	-0.10550
C	1.61422	-0.17847	0.08960
H	0.95782	-0.77857	0.73267
H	1.65354	-0.71049	-0.87143
C	3.02319	-0.15508	0.69839
H	3.71852	0.31227	-0.01230
H	3.01125	0.49243	1.58505

C	3.57607	-1.53043	1.10098
H	4.54975	-1.36383	1.57965
H	2.93015	-1.97018	1.87507
C	3.75611	-2.54676	-0.04558
H	4.05169	-2.01370	-0.96045
H	4.59644	-3.20868	0.20049
C	2.53159	-3.42573	-0.33026
H	2.23643	-3.98054	0.56883
H	1.66669	-2.83907	-0.65330
H	2.74774	-4.15741	-1.11747
H	2.79832	2.52137	-0.36735

4e_conformer-231

C	1.56170	2.33707	0.28107
C	0.14004	0.56171	0.33123
N	0.31926	1.87693	-0.07688
H	-0.33716	2.38773	-0.64939
C	2.18714	1.30627	0.95774
C	2.03205	3.71084	-0.06877
H	2.08632	3.86117	-1.15488
H	1.36807	4.48626	0.33408
H	3.03169	3.87719	0.34150
C	-1.13686	-0.11712	0.11826
C	-1.21719	-1.51888	-0.01685
C	-2.32589	0.62501	0.04186
C	-2.44876	-2.13349	-0.21135
H	-0.31494	-2.11589	0.00094
C	-3.55973	0.00008	-0.17063
H	-2.32242	1.70186	0.17795
C	-3.63156	-1.39258	-0.29429
H	-2.49578	-3.21392	-0.31667
H	-4.57652	-1.89717	-0.45265
O	-4.63839	0.83621	-0.22678
C	-5.92320	0.25280	-0.41773
H	-5.98001	-0.28517	-1.37166
H	-6.17563	-0.43330	0.39972
H	-6.63044	1.08317	-0.42659
C	1.31110	0.17978	0.98867
C	1.62272	-1.13244	1.65348
H	2.25041	-0.92978	2.53147
H	0.69998	-1.58790	2.03350
C	2.36266	-2.17708	0.78529
H	2.57724	-3.03887	1.43024
H	1.69605	-2.54817	-0.00468
C	3.66549	-1.67591	0.13559

H	4.14727	-0.94295	0.79818
H	4.37362	-2.51108	0.04599
C	3.47162	-1.05711	-1.25516
H	3.10053	-1.83500	-1.93768
H	2.69382	-0.28825	-1.20785
C	4.75880	-0.44899	-1.81529
H	5.56161	-1.19527	-1.86887
H	4.60876	-0.04598	-2.82357
H	5.11125	0.37078	-1.17687
H	3.17782	1.35263	1.39142

4e_conformer-232

C	-0.59193	3.37038	-0.39949
C	0.51080	1.41394	-0.05896
N	0.63958	2.77712	-0.28329
H	1.51877	3.24398	-0.45343
C	-1.53533	2.37357	-0.22001
C	-0.74836	4.82973	-0.67735
H	-0.27222	5.44703	0.09525
H	-0.30441	5.11475	-1.64015
H	-1.80964	5.09057	-0.70880
C	1.68411	0.56271	0.11641
C	2.88258	1.07963	0.64011
C	1.63485	-0.79810	-0.24824
C	3.99610	0.25284	0.79379
H	2.93499	2.11674	0.95669
C	2.74903	-1.62054	-0.06984
H	0.73245	-1.18969	-0.69499
C	3.94099	-1.09410	0.44954
H	4.91611	0.66210	1.20188
H	4.79707	-1.74931	0.57360
O	2.77430	-2.94859	-0.38793
C	1.57926	-3.53610	-0.89259
H	0.75251	-3.43474	-0.17895
H	1.28608	-3.08792	-1.84969
H	1.80496	-4.59284	-1.04129
C	-0.85679	1.13655	-0.00536
C	-1.49576	-0.19735	0.28234
H	-1.57400	-0.78852	-0.63968
H	-0.83964	-0.77852	0.94334
C	-2.88124	-0.09824	0.93937
H	-2.78104	0.51313	1.84508
H	-3.57027	0.44774	0.28082
C	-3.50182	-1.46123	1.30517
H	-2.70034	-2.16318	1.57854

H	-4.11543	-1.33884	2.20691
C	-4.38963	-2.09856	0.22428
H	-5.23603	-1.42709	0.02160
H	-4.82411	-3.01936	0.63627
C	-3.68686	-2.42998	-1.09584
H	-4.35777	-2.97707	-1.76828
H	-3.36383	-1.52498	-1.62123
H	-2.79979	-3.05324	-0.92744
H	-2.60599	2.52723	-0.22954

4e_conformer-233

C	0.59196	3.37037	-0.39952
C	-0.51079	1.41394	-0.05897
N	-0.63956	2.77713	-0.28330
H	-1.51874	3.24398	-0.45347
C	1.53535	2.37355	-0.22004
C	0.74840	4.82972	-0.67738
H	0.30445	5.11474	-1.64018
H	0.27226	5.44702	0.09522
H	1.80968	5.09055	-0.70883
C	-1.68411	0.56272	0.11641
C	-2.88254	1.07964	0.64019
C	-1.63488	-0.79807	-0.24827
C	-3.99607	0.25285	0.79389
H	-2.93493	2.11673	0.95680
C	-2.74905	-1.62051	-0.06985
H	-0.73249	-1.18967	-0.69507
C	-3.94099	-1.09407	0.44959
H	-4.91606	0.66211	1.20202
H	-4.79706	-1.74928	0.57366
O	-2.77435	-2.94856	-0.38797
C	-1.57932	-3.53606	-0.89268
H	-0.75254	-3.43467	-0.17909
H	-1.80502	-4.59280	-1.04136
H	-1.28620	-3.08789	-1.84981
C	0.85680	1.13654	-0.00538
C	1.49576	-0.19737	0.28231
H	0.83961	-0.77855	0.94329
H	1.57403	-0.78852	-0.63971
C	2.88121	-0.09827	0.93940
H	3.57026	0.44772	0.28088
H	2.78098	0.51309	1.84511
C	3.50178	-1.46127	1.30520
H	4.11537	-1.33889	2.20695
H	2.70030	-2.16322	1.57854

C	4.38962	-2.09858	0.22433
H	5.23603	-1.42712	0.02167
H	4.82409	-3.01939	0.63632
C	3.68689	-2.43000	-1.09582
H	2.79982	-3.05327	-0.92744
H	3.36386	-1.52501	-1.62121
H	4.35781	-2.97709	-1.76824
H	2.60601	2.52720	-0.22957

4e_conformer-234

C	0.27882	3.41037	-0.17574
C	0.24505	1.14134	-0.34040
N	1.04194	2.27809	-0.31284
H	2.03325	2.27861	-0.50680
C	-1.03711	2.99211	-0.08236
C	0.88060	4.77745	-0.16290
H	1.62133	4.89081	0.63892
H	1.38640	5.01312	-1.10846
H	0.09934	5.52621	-0.00694
C	0.84416	-0.18879	-0.44490
C	0.16463	-1.25444	-1.05851
C	2.13665	-0.41792	0.07275
C	0.76037	-2.51376	-1.13922
H	-0.81449	-1.09460	-1.49242
C	2.72961	-1.67838	-0.02874
H	2.65054	0.38633	0.58435
C	2.03685	-2.73737	-0.63405
H	0.22444	-3.32961	-1.61632
H	2.51217	-3.71094	-0.69519
O	3.97365	-1.97996	0.44652
C	4.72454	-0.93999	1.06442
H	4.90120	-0.10903	0.37080
H	4.21970	-0.56096	1.96127
H	5.67843	-1.38714	1.34701
C	-1.07535	1.57135	-0.18920
C	-2.31205	0.72603	-0.04149
H	-3.19142	1.38280	-0.04798
H	-2.43155	0.06116	-0.90270
C	-2.31658	-0.10551	1.25402
H	-1.41572	-0.73174	1.27329
H	-2.23012	0.58462	2.10377
C	-3.54711	-1.00229	1.45088
H	-3.46750	-1.45651	2.44690
H	-4.45661	-0.38432	1.47291
C	-3.71086	-2.12137	0.39849

H	-2.72022	-2.40209	0.01352
H	-4.10736	-3.02007	0.88790
C	-4.63953	-1.76733	-0.76907
H	-4.71733	-2.59937	-1.47877
H	-5.64969	-1.54400	-0.40446
H	-4.29024	-0.88967	-1.32239
H	-1.89355	3.64041	0.05551

4e_conformer-235

C	-0.27880	3.41038	-0.17574
C	-0.24505	1.14135	-0.34040
N	-1.04193	2.27809	-0.31284
H	-2.03324	2.27862	-0.50681
C	1.03713	2.99211	-0.08236
C	-0.88058	4.77746	-0.16291
H	-0.09931	5.52621	-0.00696
H	-1.38639	5.01312	-1.10846
H	-1.62130	4.89083	0.63893
C	-0.84415	-0.18879	-0.44490
C	-0.16463	-1.25443	-1.05852
C	-2.13665	-0.41791	0.07275
C	-0.76038	-2.51375	-1.13923
H	0.81448	-1.09459	-1.49244
C	-2.72961	-1.67837	-0.02874
H	-2.65053	0.38633	0.58435
C	-2.03686	-2.73736	-0.63406
H	-0.22445	-3.32961	-1.61633
H	-2.51218	-3.71093	-0.69520
O	-3.97365	-1.97995	0.44652
C	-4.72454	-0.93998	1.06443
H	-5.67843	-1.38713	1.34703
H	-4.21968	-0.56096	1.96128
H	-4.90119	-0.10901	0.37082
C	1.07536	1.57135	-0.18919
C	2.31205	0.72602	-0.04148
H	2.43154	0.06114	-0.90269
H	3.19143	1.38278	-0.04799
C	2.31659	-0.10551	1.25404
H	2.23014	0.58463	2.10378
H	1.41571	-0.73171	1.27332
C	3.54709	-1.00231	1.45088
H	4.45661	-0.38436	1.47291
H	3.46748	-1.45653	2.44690
C	3.71082	-2.12138	0.39848
H	4.10727	-3.02010	0.88788

H	2.72017	-2.40206	0.01349
C	4.63952	-1.76736	-0.76907
H	5.64968	-1.54408	-0.40444
H	4.71728	-2.59939	-1.47878
H	4.29026	-0.88967	-1.32237
H	1.89357	3.64040	0.05551

4e_conformer-236

C	-1.25088	2.53661	0.11476
C	-0.13468	0.56071	0.29246
N	-0.09627	1.86191	-0.19331
H	0.63229	2.22232	-0.79225
C	-2.03941	1.66161	0.83838
C	-1.48895	3.94445	-0.32355
H	-0.70919	4.62431	0.04274
H	-1.51169	4.03454	-1.41741
H	-2.45020	4.29629	0.06044
C	1.02899	-0.31398	0.15695
C	0.90912	-1.71317	0.13952
C	2.31730	0.25382	0.04280
C	2.04471	-2.51553	0.01352
H	-0.06741	-2.17347	0.19793
C	3.44375	-0.55795	-0.09925
H	2.42925	1.32881	0.10489
C	3.31043	-1.95478	-0.11128
H	1.93583	-3.59646	-0.00105
H	4.19929	-2.56822	-0.21682
O	4.71916	-0.08468	-0.21705
C	4.91275	1.32583	-0.21769
H	4.37578	1.80204	-1.04699
H	4.58718	1.77453	0.72866
H	5.98527	1.48048	-0.34216
C	-1.36018	0.41130	0.94654
C	-1.88402	-0.78215	1.69773
H	-1.05151	-1.29898	2.19288
H	-2.53800	-0.41594	2.50040
C	-2.67899	-1.83564	0.89339
H	-2.01925	-2.33859	0.17780
H	-2.98473	-2.61096	1.60867
C	-3.93464	-1.34481	0.15333
H	-4.55164	-2.22728	-0.06850
H	-4.53294	-0.71998	0.83198
C	-3.71231	-0.58300	-1.16664
H	-4.69768	-0.40675	-1.61993
H	-3.28350	0.40190	-0.96339

C	-2.82254	-1.31680	-2.17475
H	-3.17432	-2.34177	-2.35041
H	-2.81531	-0.79677	-3.13968
H	-1.78543	-1.37466	-1.82662
H	-3.01413	1.88948	1.25106

4e_conformer-237

C	0.37010	2.97795	-0.24316
C	-0.28334	0.80116	-0.16337
N	-0.68970	2.11320	-0.36702
H	-1.60946	2.37777	-0.68959
C	1.47507	2.21066	0.07755
C	0.22138	4.44957	-0.45210
H	-0.09316	4.68917	-1.47632
H	-0.52238	4.88609	0.22670
H	1.17658	4.94960	-0.27080
C	-1.24504	-0.29881	-0.21235
C	-0.86966	-1.58267	-0.64085
C	-2.58509	-0.07411	0.16899
C	-1.81005	-2.61414	-0.67185
H	0.14311	-1.76557	-0.97854
C	-3.52091	-1.10892	0.11562
H	-2.87130	0.90343	0.53708
C	-3.13158	-2.39088	-0.30173
H	-1.50937	-3.60329	-1.00598
H	-3.87297	-3.18283	-0.32918
O	-4.83409	-0.97401	0.46455
C	-5.28597	0.30888	0.88545
H	-4.77045	0.63509	1.79683
H	-5.14127	1.06038	0.09987
H	-6.35135	0.19816	1.09134
C	1.07916	0.84036	0.13150
C	1.98376	-0.30696	0.47951
H	2.63257	-0.01242	1.31206
H	1.39366	-1.15893	0.83765
C	2.85702	-0.77112	-0.70397
H	2.18902	-1.08472	-1.51646
H	3.41636	0.08923	-1.09678
C	3.83163	-1.91572	-0.36116
H	3.40058	-2.53037	0.44266
H	3.92606	-2.57943	-1.23011
C	5.24580	-1.46827	0.04154
H	5.85970	-2.36391	0.20694
H	5.70200	-0.93824	-0.80655
C	5.31928	-0.57731	1.28515

H	4.82148	0.38488	1.12279
H	4.84133	-1.05912	2.14703
H	6.36083	-0.36679	1.55413
H	2.46980	2.59360	0.26926

4e_conformer-238

C	0.48195	3.15660	-0.23002
C	-0.05007	0.95915	0.00947
N	-0.47852	2.19871	-0.44639
H	-1.31909	2.33998	-0.98863
C	1.53371	2.52851	0.40950
C	0.30265	4.57330	-0.66870
H	0.21312	4.65743	-1.75973
H	-0.59758	5.02516	-0.23309
H	1.16353	5.17114	-0.35753
C	-0.93082	-0.20701	-0.08803
C	-0.42772	-1.48257	-0.38340
C	-2.31962	-0.04802	0.09818
C	-1.29169	-2.57559	-0.46940
H	0.62946	-1.60909	-0.57652
C	-3.17797	-1.14488	-0.00868
H	-2.70645	0.93038	0.35528
C	-2.66143	-2.41963	-0.28687
H	-0.89147	-3.55934	-0.69899
H	-3.34512	-3.25933	-0.35860
O	-4.53158	-1.07785	0.15590
C	-5.10911	0.19386	0.43305
H	-4.73724	0.60378	1.37988
H	-4.90703	0.90826	-0.37415
H	-6.18393	0.02425	0.50826
C	1.21363	1.14417	0.56750
C	2.06605	0.14775	1.30782
H	2.15341	0.46506	2.35716
H	1.57462	-0.82968	1.32661
C	3.49144	0.01456	0.73242
H	3.93420	1.01678	0.69096
H	4.10708	-0.55522	1.44138
C	3.56612	-0.64651	-0.66148
H	2.58529	-0.57884	-1.15142
H	4.25433	-0.07933	-1.30050
C	4.04129	-2.10838	-0.63993
H	4.12300	-2.47129	-1.67332
H	5.05879	-2.13727	-0.22493
C	3.15249	-3.06706	0.15809
H	3.05852	-2.75490	1.20424

H	2.14268	-3.12193	-0.26401
H	3.56744	-4.08145	0.15269
H	2.43922	3.01772	0.74561

4e_conformer-239

C	-0.43260	3.12902	-0.13720
C	0.18940	0.96283	-0.45502
N	0.62412	2.25567	-0.19365
H	1.59471	2.53500	-0.18530
C	-1.57700	2.37770	-0.33770
C	-0.24225	4.59323	0.08867
H	0.27671	4.79738	1.03393
H	0.34607	5.05952	-0.71245
H	-1.21372	5.09354	0.12384
C	1.14570	-0.14072	-0.53358
C	0.89497	-1.27428	-1.32431
C	2.35660	-0.07258	0.18800
C	1.82800	-2.31020	-1.38255
H	-0.01472	-1.33536	-1.90901
C	3.29006	-1.10825	0.10933
H	2.53972	0.77650	0.83454
C	3.02497	-2.23868	-0.67778
H	1.62275	-3.18140	-1.99846
H	3.76002	-3.03585	-0.71927
O	4.48210	-1.11715	0.77546
C	4.80656	0.01001	1.58249
H	5.79392	-0.19426	1.99856
H	4.84388	0.93005	0.98669
H	4.08605	0.13761	2.39947
C	-1.20395	1.01732	-0.54462
C	-2.18162	-0.11591	-0.70587
H	-3.18230	0.31242	-0.82199
H	-1.99548	-0.67859	-1.63003
C	-2.17389	-1.10545	0.47613
H	-1.16982	-1.53855	0.54562
H	-2.32976	-0.54724	1.40952
C	-3.21721	-2.23451	0.36021
H	-3.39486	-2.45636	-0.70243
H	-2.80184	-3.15343	0.79374
C	-4.56015	-1.95581	1.05388
H	-5.19645	-2.84513	0.95027
H	-4.38070	-1.83322	2.13143
C	-5.32278	-0.73403	0.53292
H	-4.77655	0.19546	0.72664
H	-5.49114	-0.80315	-0.54892

H	-6.30137	-0.64736	1.01913
H	-2.58961	2.76171	-0.33173

4e_conformer-240

C	-1.26544	2.34558	0.29492
C	-0.01738	0.44334	0.30701
N	-0.07847	1.77317	-0.08952
H	0.60855	2.22193	-0.67807
C	-1.97224	1.37360	0.97837
C	-1.61129	3.75943	-0.04001
H	-2.57600	4.02107	0.40290
H	-0.86194	4.46537	0.34016
H	-1.68661	3.91737	-1.12385
C	1.18501	-0.35241	0.06718
C	1.12704	-1.74602	-0.09606
C	2.44230	0.28626	-0.00378
C	2.29643	-2.47676	-0.31473
H	0.17067	-2.25223	-0.08350
C	3.60259	-0.45278	-0.24060
H	2.50223	1.35414	0.16513
C	3.53285	-1.84603	-0.39286
H	2.23808	-3.55431	-0.44114
H	4.44741	-2.40271	-0.56963
O	4.85313	0.08921	-0.32433
C	4.98185	1.50020	-0.18446
H	6.04406	1.71706	-0.30451
H	4.41061	2.03146	-0.95538
H	4.65098	1.83572	0.80593
C	-1.20621	0.16908	0.98561
C	-1.62393	-1.11245	1.65173
H	-0.73852	-1.65485	2.00576
H	-2.20827	-0.85776	2.54595
C	-2.47947	-2.07792	0.79803
H	-1.86783	-2.51150	-0.00443
H	-2.76496	-2.91567	1.44716
C	-3.73881	-1.45002	0.17336
H	-4.52731	-2.21117	0.09671
H	-4.13359	-0.67453	0.84497
C	-3.51014	-0.85012	-1.22039
H	-2.65654	-0.16551	-1.18756
H	-3.23446	-1.65983	-1.91106
C	-4.73861	-0.11084	-1.75385
H	-4.99105	0.73937	-1.10793
H	-4.56829	0.27699	-2.76486
H	-5.61522	-0.76997	-1.79119

H	-2.94498	1.51074	1.43295

4e_conformer-241

C	3.10587	-1.65820	-0.38292
C	1.20369	-0.72788	0.43949
N	1.75687	-1.83511	-0.18496
H	1.25710	-2.69683	-0.35327
C	3.41606	-0.39759	0.09341
C	3.96459	-2.72052	-0.98800
H	3.60629	-3.01903	-1.98149
H	4.98893	-2.35385	-1.09654
H	3.99716	-3.62660	-0.36868
C	-0.23412	-0.64924	0.70879
C	-0.72072	-0.13891	1.92862
C	-1.15363	-1.07068	-0.25895
C	-2.09048	-0.03290	2.14082
H	-0.02144	0.15424	2.70387
C	-2.53231	-0.96943	-0.03492
H	-0.81258	-1.44650	-1.21849
C	-3.01093	-0.44087	1.16949
H	-2.45987	0.36368	3.08246
H	-4.07284	-0.34717	1.35993
O	-3.32612	-1.39315	-1.06178
C	-4.73615	-1.26499	-0.90926
H	-5.10308	-1.86228	-0.06592
H	-5.02723	-0.21759	-0.76481
H	-5.17254	-1.63939	-1.83612
C	2.22724	0.19881	0.61655
C	2.10049	1.62430	1.07797
H	1.19847	1.75198	1.68274
H	2.94673	1.87622	1.73055
C	2.07504	2.63110	-0.09532
H	2.05977	3.65099	0.31485
H	3.01422	2.53316	-0.65553
C	0.89498	2.45057	-1.05985
H	1.05516	3.08826	-1.94085
H	0.88636	1.41514	-1.42525
C	-0.47512	2.78001	-0.45600
H	-0.64228	2.17381	0.44049
H	-0.48164	3.83008	-0.12926
C	-1.62473	2.53588	-1.43687
H	-1.50888	3.14027	-2.34550
H	-1.65975	1.48184	-1.73582
H	-2.59375	2.78523	-0.98889
H	4.39788	0.05861	0.06219

4e_conformer-242

C	3.47872	-0.94639	0.10834
C	1.22126	-0.67796	0.13697
N	2.27677	-1.44964	-0.32722
H	2.16170	-2.33146	-0.80659
C	3.19087	0.19065	0.84040
C	4.77851	-1.61363	-0.20310
H	4.83708	-2.62210	0.22707
H	4.93868	-1.71202	-1.28438
H	5.60525	-1.02737	0.20704
C	-0.15660	-1.00106	-0.23963
C	-0.45049	-1.46629	-1.53176
C	-1.20479	-0.85825	0.68928
C	-1.76717	-1.76883	-1.88561
H	0.34490	-1.56083	-2.26452
C	-2.52140	-1.13844	0.31704
H	-0.97002	-0.54844	1.69853
C	-2.80603	-1.60150	-0.97673
H	-1.98673	-2.12426	-2.88849
H	-3.83526	-1.82058	-1.24186
O	-3.59560	-1.00898	1.14948
C	-3.36700	-0.49492	2.45755
H	-2.91741	0.50476	2.41901
H	-4.34758	-0.43577	2.93149
H	-2.71959	-1.15995	3.04174
C	1.77389	0.37108	0.87006
C	1.08116	1.57614	1.44606
H	0.03709	1.35366	1.68101
H	1.55830	1.84205	2.39819
C	1.14061	2.80741	0.51274
H	0.73329	3.67686	1.04825
H	2.19488	3.03265	0.30559
C	0.39049	2.62820	-0.81363
H	0.65258	3.45516	-1.48862
H	0.74003	1.70887	-1.30156
C	-1.13512	2.57410	-0.67167
H	-1.41392	1.79746	0.04903
H	-1.49221	3.52793	-0.25729
C	-1.84004	2.28605	-1.99929
H	-1.53316	1.31021	-2.39322
H	-2.92967	2.26975	-1.88034
H	-1.59591	3.04588	-2.75251
H	3.92399	0.83856	1.30493

4e_conformer-243

C	3.09645	-1.54051	-0.34102
C	0.89209	-1.21302	0.11158
N	1.81920	-1.99008	-0.56964
H	1.57228	-2.70296	-1.24111
C	2.99246	-0.47321	0.53117
C	4.28703	-2.17053	-0.98690
H	4.36277	-3.23950	-0.75022
H	4.25593	-2.08130	-2.08071
H	5.20136	-1.68308	-0.63801
C	-0.53486	-1.53108	0.03383
C	-0.96325	-2.86555	-0.07272
C	-1.50046	-0.50601	0.04108
C	-2.32459	-3.16062	-0.16514
H	-0.23540	-3.67084	-0.05396
C	-2.86049	-0.81384	-0.03459
H	-1.17045	0.52128	0.06819
C	-3.27853	-2.14866	-0.14130
H	-2.64589	-4.19548	-0.24412
H	-4.33996	-2.36490	-0.20651
O	-3.85487	0.12221	-0.03288
C	-3.48074	1.49101	0.08354
H	-2.86012	1.81065	-0.76253
H	-4.41268	2.05773	0.08416
H	-2.93864	1.67752	1.01869
C	1.61133	-0.25531	0.82557
C	1.10084	0.77392	1.79758
H	0.03426	0.61678	1.99354
H	1.61215	0.63567	2.76029
C	1.33360	2.22788	1.33850
H	1.09103	2.90678	2.16740
H	2.40327	2.36545	1.12809
C	0.52118	2.62624	0.10500
H	0.69566	1.89596	-0.69722
H	-0.55035	2.57326	0.34691
C	0.83804	4.03188	-0.41259
H	0.68061	4.76074	0.39481
H	1.90408	4.08876	-0.67332
C	-0.01070	4.41868	-1.62633
H	-1.07980	4.39584	-1.38069
H	0.22971	5.42684	-1.98243
H	0.15209	3.72141	-2.45738
H	3.82623	0.08872	0.93321

4e_conformer-244

C	-3.09713	-1.53918	-0.34106
C	-0.89265	-1.21268	0.11165
N	-1.82008	-1.98936	-0.56958
H	-1.57346	-2.70241	-1.24098
C	-2.99270	-0.47190	0.53111
C	-4.28797	-2.16859	-0.98705
H	-4.36426	-3.23753	-0.75043
H	-5.20209	-1.68070	-0.63820
H	-4.25676	-2.07932	-2.08086
C	0.53417	-1.53133	0.03394
C	0.96204	-2.86598	-0.07260
C	1.50017	-0.50664	0.04112
C	2.32326	-3.16156	-0.16512
H	0.23389	-3.67100	-0.05377
C	2.86007	-0.81499	-0.03462
H	1.17054	0.52078	0.06818
C	3.27759	-2.14997	-0.14136
H	2.64416	-4.19655	-0.24410
H	4.33894	-2.36662	-0.20666
O	3.85482	0.12068	-0.03298
C	3.48125	1.48961	0.08366
H	4.41342	2.05594	0.08434
H	2.86072	1.80964	-0.76232
H	2.93927	1.67619	1.01887
C	-1.61149	-0.25463	0.82558
C	-1.10063	0.77438	1.79762
H	-1.61210	0.63640	2.76027
H	-0.03414	0.61675	1.99369
C	-1.33267	2.22844	1.33846
H	-2.40227	2.36650	1.12798
H	-1.08982	2.90726	2.16734
C	-0.52000	2.62635	0.10498
H	0.55149	2.57282	0.34695
H	-0.69482	1.89614	-0.69723
C	-0.83611	4.03214	-0.41266
H	-1.90209	4.08955	-0.67349
H	-0.67839	4.76093	0.39475
C	0.01296	4.41849	-1.62632
H	-0.15009	3.72128	-2.45737
H	-0.22692	5.42676	-1.98247
H	1.08202	4.39512	-1.38056
H	-3.82625	0.09043	0.93307

4e_conformer-245

C	3.62111	-0.96587	-0.09602
C	1.37221	-0.64211	-0.20015
N	2.39065	-1.47823	0.23550
H	2.23720	-2.39779	0.62448
C	3.38955	0.24058	-0.73028
C	4.89140	-1.69041	0.20920
H	5.74808	-1.08696	-0.10286
H	4.99686	-1.89637	1.28201
H	4.95181	-2.65373	-0.31412
C	-0.02561	-0.96989	0.08879
C	-0.37429	-1.58930	1.30783
C	-1.04290	-0.68370	-0.82826
C	-1.70286	-1.89203	1.58350
H	0.39482	-1.80082	2.04418
C	-2.38176	-0.96283	-0.53100
H	-0.81279	-0.24768	-1.79331
C	-2.72227	-1.57881	0.67895
H	-1.96156	-2.36581	2.52641
H	-3.75142	-1.81064	0.92335
O	-3.28104	-0.60584	-1.49494
C	-4.66023	-0.85240	-1.24153
H	-5.00423	-0.31203	-0.35138
H	-5.19579	-0.48659	-2.11851
H	-4.85823	-1.92345	-1.11354
C	1.97964	0.45745	-0.80637
C	1.34728	1.72505	-1.31548
H	1.94269	2.09758	-2.15872
H	0.34613	1.53759	-1.71229
C	1.27038	2.83972	-0.24662
H	2.27930	3.00080	0.15476
H	0.97361	3.77859	-0.73563
C	0.30295	2.53995	0.90602
H	0.52612	1.54542	1.31310
H	0.48177	3.25398	1.72225
C	-1.17778	2.60514	0.51291
H	-1.42605	3.63431	0.21627
H	-1.35760	1.97668	-0.36654
C	-2.10663	2.14666	1.63912
H	-1.90561	1.10201	1.90128
H	-1.96702	2.75527	2.54169
H	-3.16044	2.21983	1.34558
H	4.15523	0.91090	-1.10107

4e_conformer-246

C	-3.62051	-0.96632	-0.09660

C	-1.37172	-0.64199	-0.20089
N	-2.38990	-1.47858	0.23438
H	-2.23610	-2.39818	0.62312
C	-3.38932	0.24054	-0.73025
C	-4.89063	-1.69127	0.20837
H	-5.74742	-1.08763	-0.10300
H	-4.95111	-2.65419	-0.31568
H	-4.99580	-1.89808	1.28106
C	0.02618	-0.96960	0.08785
C	0.37493	-1.59092	1.30591
C	1.04358	-0.68148	-0.82847
C	1.70355	-1.89363	1.58132
H	-0.39417	-1.80397	2.04183
C	2.38249	-0.96049	-0.53137
H	0.81356	-0.24401	-1.79288
C	2.72302	-1.57847	0.67754
H	1.96223	-2.36892	2.52348
H	3.75223	-1.81029	0.92173
O	3.28179	-0.60142	-1.49453
C	4.66095	-0.84864	-1.24164
H	4.85878	-1.91995	-1.11553
H	5.19652	-0.48139	-2.11800
H	5.00510	-0.30991	-0.35055
C	-1.97952	0.45779	-0.80645
C	-1.34764	1.72587	-1.31496
H	-0.34636	1.53900	-1.71172
H	-1.94307	2.09856	-2.15811
C	-1.27111	2.84000	-0.24550
H	-0.97345	3.77900	-0.73372
H	-2.28028	3.00130	0.15517
C	-0.30481	2.53871	0.90765
H	-0.48432	3.25143	1.72486
H	-0.52859	1.54357	1.31289
C	1.17635	2.60422	0.51608
H	1.35646	1.97914	-0.36571
H	1.42579	3.63435	0.22375
C	2.10371	2.14022	1.64125
H	1.90130	1.09474	1.89891
H	3.15787	2.21349	1.34899
H	1.96389	2.74531	2.54616
H	-4.15524	0.91087	-1.10052

4e_conformer-247

C	2.79628	-1.98791	-0.66082
C	1.01505	-1.01442	0.36320

N	1.43247	-2.01857	-0.49807
H	0.82747	-2.74728	-0.84947
C	3.26071	-0.92119	0.08662
C	3.51871	-2.99301	-1.49720
H	3.40541	-4.01245	-1.10551
H	3.15479	-3.00067	-2.53258
H	4.58686	-2.76051	-1.51839
C	-0.40864	-0.81993	0.64720
C	-0.85927	-0.45162	1.93060
C	-1.35670	-1.00341	-0.36815
C	-2.21569	-0.25691	2.16326
H	-0.14758	-0.34736	2.74138
C	-2.72321	-0.82048	-0.12257
H	-1.04622	-1.26101	-1.37596
C	-3.16286	-0.43644	1.14948
H	-2.55465	0.02639	3.15591
H	-4.21399	-0.28188	1.35922
O	-3.54473	-1.02603	-1.19385
C	-4.94267	-0.82896	-1.00762
H	-5.16405	0.20308	-0.70999
H	-5.40525	-1.03586	-1.97351
H	-5.34619	-1.51678	-0.25482
C	2.15185	-0.30077	0.73901
C	2.23281	0.97600	1.53138
H	1.42526	1.03349	2.26786
H	3.17117	0.98033	2.10064
C	2.18194	2.24457	0.65342
H	2.37973	3.11989	1.28828
H	2.99478	2.20306	-0.08510
C	0.84568	2.43415	-0.06840
H	0.64755	1.56026	-0.70176
H	0.03411	2.45778	0.67337
C	0.78847	3.70199	-0.92464
H	0.97997	4.58028	-0.29194
H	1.60078	3.67601	-1.66486
C	-0.55356	3.86921	-1.64217
H	-1.37889	3.92877	-0.92188
H	-0.57531	4.77908	-2.25286
H	-0.75389	3.01644	-2.30259
H	4.29523	-0.60818	0.15639

4e_conformer-248

C	-2.95144	-1.68350	-0.24084
C	-1.09500	-0.64308	0.55522
N	-1.60857	-1.81625	0.02275

H	-1.09363	-2.68363	-0.03420
C	-3.29702	-0.38775	0.09594
C	-3.77156	-2.81535	-0.76822
H	-3.37150	-3.20445	-1.71324
H	-3.81519	-3.65606	-0.06331
H	-4.79599	-2.47930	-0.95012
C	0.33012	-0.51585	0.86936
C	0.76309	0.12130	2.04175
C	1.28769	-1.03020	-0.02636
C	2.12870	0.26612	2.29428
H	0.03287	0.48468	2.75632
C	2.65168	-0.89034	0.24170
H	0.94797	-1.49810	-0.94205
C	3.07583	-0.23222	1.40581
H	2.45851	0.76412	3.20174
H	4.13995	-0.12733	1.59115
O	3.64446	-1.34430	-0.57776
C	3.26674	-1.99484	-1.78680
H	2.67229	-2.89460	-1.58761
H	2.69733	-1.32270	-2.44023
H	4.19860	-2.27629	-2.27877
C	-2.13773	0.27816	0.60118
C	-2.05045	1.74415	0.92366
H	-2.91791	2.04054	1.52795
H	-1.16708	1.94910	1.53449
C	-2.01346	2.63149	-0.34193
H	-2.93819	2.46418	-0.90970
H	-2.02166	3.68673	-0.03370
C	-0.80976	2.37584	-1.25872
H	-0.78331	1.31100	-1.52570
H	-0.95520	2.92702	-2.19867
C	0.54294	2.77473	-0.65769
H	0.53236	3.85007	-0.42764
H	0.69559	2.25401	0.29348
C	1.71600	2.45574	-1.58777
H	1.76378	1.37949	-1.79127
H	1.61628	2.97575	-2.54894
H	2.67322	2.75161	-1.14303
H	-4.28293	0.04600	-0.01741

4e_conformer-249

C	-2.88342	-2.37834	-0.22246
C	-1.68706	-0.46749	0.07366
N	-2.90350	-1.00884	-0.31809
H	-3.65733	-0.47450	-0.72555

C	-1.63701	-2.72553	0.26744
C	-4.05416	-3.21884	-0.61542
H	-4.95432	-2.95956	-0.04360
H	-4.30138	-3.10671	-1.67918
H	-3.83248	-4.27383	-0.43341
C	-1.47440	0.97829	0.08566
C	-2.54727	1.86557	0.28366
C	-0.18444	1.51118	-0.11013
C	-2.32831	3.24401	0.28487
H	-3.54519	1.47999	0.46812
C	0.02637	2.89074	-0.08566
H	0.63616	0.84021	-0.31303
C	-1.05143	3.76733	0.10759
H	-3.16479	3.91956	0.44095
H	-0.86765	4.83677	0.11514
O	1.24845	3.47567	-0.25887
C	2.37742	2.62380	-0.42667
H	2.50984	1.96243	0.43834
H	2.28985	2.01512	-1.33482
H	3.24066	3.28456	-0.51520
C	-0.87462	-1.53545	0.45871
C	0.52499	-1.47503	1.00167
H	0.69380	-0.50993	1.49512
H	0.64660	-2.24218	1.77798
C	1.61367	-1.68863	-0.06689
H	1.50459	-2.69469	-0.49430
H	1.45097	-0.99125	-0.90043
C	3.03087	-1.50404	0.47953
H	3.11659	-0.50297	0.92852
H	3.20494	-2.21923	1.29676
C	4.11888	-1.67070	-0.58520
H	4.03765	-2.67116	-1.03259
H	3.93623	-0.95548	-1.39966
C	5.53177	-1.46756	-0.03273
H	5.75075	-2.18980	0.76329
H	6.29045	-1.58993	-0.81403
H	5.64668	-0.46249	0.39158
H	-1.30671	-3.73433	0.48177

4e_conformer-250

C	-3.31908	-1.28801	-0.06882
C	-1.07690	-0.91887	0.02163
N	-2.10319	-1.58150	-0.63618
H	-1.95587	-2.28174	-1.34921
C	-3.07203	-0.39332	0.95614

C	-4.59030	-1.90705	-0.55073
H	-4.76870	-1.69986	-1.61364
H	-4.58911	-2.99813	-0.42820
H	-5.43631	-1.50855	0.01573
C	0.30494	-1.04113	-0.44770
C	0.58294	-1.13561	-1.82124
C	1.37462	-1.06699	0.46749
C	1.90281	-1.24190	-2.26453
H	-0.22869	-1.09146	-2.54078
C	2.69204	-1.15110	0.01242
H	1.15832	-1.04530	1.52653
C	2.96041	-1.24290	-1.36208
H	2.10884	-1.30908	-3.32915
H	3.99152	-1.31356	-1.69290
O	3.78458	-1.16898	0.83118
C	3.57011	-1.03585	2.23247
H	4.56104	-1.03586	2.68830
H	3.05768	-0.09535	2.46911
H	2.98638	-1.87498	2.62979
C	-1.66600	-0.15353	1.02794
C	-1.02614	0.86489	1.93338
H	-1.57730	0.88433	2.88218
H	0.00219	0.58522	2.18253
C	-1.01243	2.29088	1.34286
H	-2.04156	2.58572	1.09430
H	-0.66541	2.98846	2.11832
C	-0.12688	2.43248	0.10271
H	0.89824	2.12255	0.35264
H	-0.47051	1.73484	-0.67131
C	-0.10328	3.85181	-0.47068
H	-1.12893	4.15839	-0.72068
H	0.24531	4.55281	0.30098
C	0.78463	3.97205	-1.71201
H	0.43935	3.30011	-2.50747
H	0.78546	4.99267	-2.11153
H	1.82222	3.70173	-1.48001
H	-3.82532	0.05433	1.59271

4e_conformer-251

C	3.31857	-1.28898	-0.06881
C	1.07649	-0.91921	0.02161
N	2.10261	-1.58209	-0.63621
H	1.95511	-2.28224	-1.34930
C	3.07174	-0.39427	0.95620
C	4.58962	-1.90836	-0.55071

H	4.58812	-2.99944	-0.42823
H	4.76810	-1.70117	-1.61360
H	5.43573	-1.51011	0.01579
C	-0.30537	-1.04104	-0.44776
C	-0.58336	-1.13535	-1.82132
C	-1.37508	-1.06670	0.46740
C	-1.90325	-1.24128	-2.26464
H	0.22829	-1.09135	-2.54084
C	-2.69251	-1.15045	0.01229
H	-1.15880	-1.04513	1.52644
C	-2.96087	-1.24209	-1.36222
H	-2.10927	-1.30832	-3.32927
H	-3.99199	-1.31247	-1.69307
O	-3.78508	-1.16812	0.83103
C	-3.57061	-1.03517	2.23234
H	-3.05796	-0.09482	2.46908
H	-4.56154	-1.03501	2.68814
H	-2.98707	-1.87447	2.62958
C	1.66579	-0.15409	1.02798
C	1.02617	0.86446	1.93345
H	-0.00231	0.58515	2.18238
H	1.57718	0.88356	2.88235
C	1.01313	2.29053	1.34312
H	0.66618	2.98813	2.11859
H	2.04242	2.58502	1.09483
C	0.12792	2.43262	0.10278
H	0.47149	1.73497	-0.67126
H	-0.89737	2.12302	0.35241
C	0.10496	3.85204	-0.47040
H	-0.24358	4.55305	0.30128
H	1.13077	4.15830	-0.72011
C	-0.78260	3.97279	-1.71193
H	-1.82034	3.70278	-1.48024
H	-0.78299	4.99347	-2.11128
H	-0.43736	3.30087	-2.50742
H	3.82515	0.05312	1.59282

4e_conformer-252

C	1.77964	-2.73889	-0.47174
C	0.36707	-1.19147	0.41552
N	0.48760	-2.27884	-0.43909
H	-0.29584	-2.74257	-0.87662
C	2.51404	-1.91174	0.35965
C	2.18108	-3.92727	-1.28272
H	1.66756	-4.84085	-0.95560

H	1.95463	-3.79097	-2.34792
H	3.25696	-4.09654	-1.18690
C	-0.92034	-0.51875	0.58313
C	-1.25693	0.13530	1.78680
C	-1.86142	-0.52159	-0.45696
C	-2.48915	0.76327	1.91983
H	-0.56093	0.12990	2.61687
C	-3.10666	0.10104	-0.30988
H	-1.63444	-0.98042	-1.41418
C	-3.42956	0.75565	0.88419
H	-2.73734	1.26246	2.85245
H	-4.38390	1.24989	1.01655
O	-3.93097	0.02643	-1.39640
C	-5.20235	0.66209	-1.31170
H	-5.81298	0.22901	-0.51031
H	-5.09892	1.74099	-1.14449
H	-5.68831	0.48821	-2.27263
C	1.64157	-0.93849	0.92855
C	2.08933	0.19397	1.81617
H	1.57643	0.16428	2.78599
H	3.15413	0.04830	2.03944
C	1.90121	1.59184	1.19552
H	0.83286	1.76774	1.01823
H	2.22407	2.34747	1.92527
C	2.66982	1.78626	-0.11317
H	3.74291	1.61971	0.06512
H	2.35744	1.01786	-0.83271
C	2.46644	3.17240	-0.73149
H	1.39307	3.33490	-0.90406
H	2.77970	3.94198	-0.01183
C	3.22979	3.35805	-2.04525
H	2.91150	2.61998	-2.79185
H	3.06612	4.35539	-2.46929
H	4.30897	3.23113	-1.89450
H	3.57928	-1.98746	0.53911

4e_conformer-253

C	3.29380	-1.66546	-0.29805
C	1.68636	-0.09939	0.06645
N	2.96948	-0.33569	-0.40660
H	3.53758	0.35595	-0.87430
C	2.20762	-2.29455	0.28353
C	4.60587	-2.20279	-0.76819
H	5.45128	-1.71277	-0.26854
H	4.66616	-3.27404	-0.55822

H	4.74264	-2.06475	-1.84875
C	1.12586	1.25038	0.08046
C	1.95695	2.37637	0.21669
C	-0.26327	1.44666	-0.05227
C	1.40672	3.65911	0.22046
H	3.02675	2.24941	0.35111
C	-0.80453	2.73320	-0.02802
H	-0.90214	0.59027	-0.20645
C	0.03349	3.85017	0.10492
H	2.05806	4.52189	0.32894
H	-0.40647	4.84209	0.11370
O	-2.13911	2.99878	-0.14519
C	-3.02811	1.89394	-0.27565
H	-2.82428	1.32079	-1.18791
H	-2.96290	1.22448	0.59070
H	-4.03058	2.32015	-0.33148
C	1.18993	-1.32289	0.51855
C	-0.14011	-1.59351	1.16297
H	-0.01192	-2.33836	1.95987
H	-0.51300	-0.68377	1.64972
C	-1.20961	-2.10950	0.18236
H	-1.29988	-1.40479	-0.65363
H	-0.86683	-3.05532	-0.25941
C	-2.57248	-2.31558	0.84971
H	-2.46001	-3.04438	1.66440
H	-2.88831	-1.37409	1.32415
C	-3.67675	-2.79055	-0.10569
H	-3.34504	-3.70662	-0.61446
H	-4.55997	-3.07001	0.48357
C	-4.08642	-1.74452	-1.14791
H	-4.89633	-2.11663	-1.78540
H	-4.44065	-0.82764	-0.66018
H	-3.25108	-1.47122	-1.80194
H	2.15152	-3.34745	0.53051

4e_conformer-254

C	-3.29347	-1.66605	-0.29792
C	-1.68630	-0.09965	0.06645
N	-2.96940	-0.33623	-0.40654
H	-3.53765	0.35529	-0.87425
C	-2.20714	-2.29491	0.28365
C	-4.60545	-2.20365	-0.76799
H	-5.45093	-1.71377	-0.26833
H	-4.74228	-2.06568	-1.84855
H	-4.66553	-3.27491	-0.55797

C	-1.12609	1.25022	0.08039
C	-1.95744	2.37606	0.21638
C	0.26302	1.44680	-0.05219
C	-1.40749	3.65891	0.22006
H	-3.02723	2.24887	0.35068
C	0.80398	2.73347	-0.02800
H	0.90211	0.59055	-0.20619
C	-0.03430	3.85027	0.10469
H	-2.05905	4.52156	0.32836
H	0.40545	4.84229	0.11341
O	2.13853	2.99933	-0.14501
C	3.02781	1.89467	-0.27507
H	4.03019	2.32110	-0.33081
H	2.96259	1.22537	0.59141
H	2.82428	1.32129	-1.18724
C	-1.18962	-1.32305	0.51859
C	0.14048	-1.59343	1.16296
H	0.51326	-0.68362	1.64966
H	0.01245	-2.33827	1.95989
C	1.21002	-2.10930	0.18232
H	0.86738	-3.05521	-0.25936
H	1.30014	-1.40464	-0.65373
C	2.57296	-2.31510	0.84962
H	2.88872	-1.37348	1.32385
H	2.46062	-3.04375	1.66446
C	3.67722	-2.79015	-0.10575
H	4.56053	-3.06934	0.48351
H	3.34560	-3.70640	-0.61426
C	4.08666	-1.74433	-1.14827
H	4.44076	-0.82727	-0.66080
H	4.89659	-2.11648	-1.78571
H	3.25124	-1.47134	-1.80232
H	-2.15084	-3.34779	0.53067

4e_conformer-255

C	-3.26324	-1.57775	0.13034
C	-1.07400	-1.00382	-0.08796
N	-1.99282	-1.76735	0.61763
H	-1.73854	-2.46281	1.30482
C	-3.16218	-0.64868	-0.88882
C	-4.44125	-2.31798	0.67446
H	-4.35245	-3.40184	0.52318
H	-4.56804	-2.14971	1.75155
H	-5.35385	-1.98492	0.17278
C	0.34000	-1.00258	0.29404

C	0.70877	-1.06345	1.65420
C	1.35208	-0.94145	-0.67103
C	2.05126	-1.04974	2.01524
H	-0.06024	-1.08849	2.42003
C	2.70152	-0.91012	-0.29940
H	1.11077	-0.93778	-1.72762
C	3.06192	-0.96649	1.05253
H	2.32585	-1.09015	3.06570
H	4.10014	-0.94875	1.36000
O	3.59226	-0.84120	-1.33237
C	4.98018	-0.82527	-1.01485
H	5.27901	-1.73715	-0.48388
H	5.50490	-0.77238	-1.96964
H	5.24067	0.04917	-0.40644
C	-1.79055	-0.28026	-1.03982
C	-1.29631	0.80825	-1.95406
H	-0.25516	0.63688	-2.24449
H	-1.88298	0.78358	-2.88128
C	-1.40494	2.21983	-1.33897
H	-1.15672	2.96052	-2.11234
H	-2.44896	2.40593	-1.05048
C	-0.49520	2.43005	-0.12628
H	-0.73991	1.68834	0.64448
H	0.54651	2.22966	-0.41628
C	-0.59424	3.83469	0.47465
H	-0.34458	4.58021	-0.29361
H	-1.63620	4.03195	0.76425
C	0.31946	4.02305	1.68850
H	1.37023	3.86212	1.41742
H	0.23221	5.03187	2.10796
H	0.07057	3.30687	2.48137
H	-3.99124	-0.26182	-1.46854

4e_conformer-256

C	3.26321	-1.57778	0.13034
C	1.07398	-1.00383	-0.08796
N	1.99279	-1.76735	0.61764
H	1.73851	-2.46281	1.30483
C	3.16216	-0.64872	-0.88883
C	4.44122	-2.31801	0.67447
H	4.56803	-2.14970	1.75155
H	4.35239	-3.40187	0.52324
H	5.35382	-1.98500	0.17276
C	-0.34002	-1.00256	0.29405
C	-0.70878	-1.06341	1.65421

C	-1.35210	-0.94145	-0.67102
C	-2.05128	-1.04967	2.01525
H	0.06023	-1.08844	2.42004
C	-2.70154	-0.91009	-0.29939
H	-1.11078	-0.93780	-1.72762
C	-3.06194	-0.96643	1.05254
H	-2.32587	-1.09006	3.06571
H	-4.10016	-0.94868	1.36001
O	-3.59228	-0.84119	-1.33237
C	-4.98019	-0.82525	-1.01484
H	-5.24068	0.04920	-0.40645
H	-5.50491	-0.77236	-1.96963
H	-5.27903	-1.73711	-0.48386
C	1.79054	-0.28029	-1.03983
C	1.29631	0.80822	-1.95407
H	1.88297	0.78354	-2.88130
H	0.25515	0.63686	-2.24450
C	1.40496	2.21980	-1.33900
H	2.44899	2.40591	-1.05054
H	1.15672	2.96049	-2.11237
C	0.49525	2.43004	-0.12629
H	-0.54646	2.22963	-0.41626
H	0.73999	1.68833	0.64447
C	0.59430	3.83468	0.47463
H	1.63627	4.03194	0.76421
H	0.34462	4.58020	-0.29363
C	-0.31937	4.02305	1.68849
H	-1.37015	3.86210	1.41744
H	-0.07046	3.30687	2.48136
H	-0.23212	5.03187	2.10794
H	3.99123	-0.26188	-1.46856

4e_conformer-257

C	3.79624	-0.02762	0.28714
C	1.56389	-0.42510	0.12104
N	2.83517	-0.97005	0.01618
H	3.01780	-1.95498	-0.11493
C	3.12813	1.15763	0.54130
C	5.25341	-0.35669	0.29210
H	5.83769	0.54587	0.49071
H	5.50602	-1.09568	1.06385
H	5.58350	-0.76692	-0.67083
C	0.37558	-1.22958	-0.16441
C	0.40363	-2.22919	-1.15148
C	-0.81748	-1.01500	0.55305

C	-0.74045	-2.98397	-1.41769
H	1.30637	-2.39178	-1.73209
C	-1.95992	-1.76511	0.26832
H	-0.82918	-0.27538	1.34093
C	-1.92372	-2.75769	-0.72299
H	-0.71130	-3.75071	-2.18688
H	-2.82180	-3.33218	-0.92538
O	-3.15461	-1.61285	0.91184
C	-3.22274	-0.67656	1.98299
H	-3.01355	0.34229	1.63900
H	-4.24496	-0.72843	2.35997
H	-2.52272	-0.93907	2.78532
C	1.72490	0.92269	0.44504
C	0.65860	1.98265	0.52308
H	-0.13585	1.69576	1.22030
H	1.10049	2.89846	0.93634
C	0.04367	2.30547	-0.85275
H	0.84449	2.67601	-1.50562
H	-0.31452	1.37579	-1.30994
C	-1.09016	3.33938	-0.80134
H	-0.73281	4.23301	-0.27017
H	-1.32195	3.66379	-1.82564
C	-2.39011	2.84806	-0.14333
H	-2.18676	2.51745	0.88429
H	-3.07843	3.69863	-0.05282
C	-3.08483	1.72283	-0.91736
H	-3.30557	2.03677	-1.94528
H	-2.46437	0.82261	-0.96918
H	-4.03227	1.44207	-0.44330
H	3.59842	2.10674	0.76699

4e_conformer-258

C	2.37835	2.42113	-0.29322
C	0.42825	1.25805	-0.14441
N	1.16102	2.33615	0.33337
H	0.79406	3.03244	0.96644
C	2.44269	1.35764	-1.17627
C	3.35294	3.51307	0.00501
H	2.94735	4.50394	-0.23789
H	3.63755	3.52932	1.06484
H	4.26240	3.37087	-0.58489
C	-0.89390	0.96006	0.40346
C	-1.22606	1.32309	1.72038
C	-1.86539	0.31193	-0.38521
C	-2.49605	1.04372	2.22808

H	-0.48433	1.79213	2.35921
C	-3.12505	0.01922	0.13942
H	-1.62758	0.06093	-1.40885
C	-3.44874	0.39154	1.45316
H	-2.73971	1.32615	3.24850
H	-4.43578	0.15861	1.83914
O	-4.11324	-0.61549	-0.55748
C	-3.82266	-1.04582	-1.88333
H	-3.60092	-0.19607	-2.54028
H	-2.97717	-1.74430	-1.89941
H	-4.72121	-1.55287	-2.23723
C	1.22511	0.62055	-1.09930
C	0.95561	-0.65863	-1.84962
H	0.06789	-0.56563	-2.48873
H	1.79510	-0.83087	-2.53544
C	0.79343	-1.89783	-0.94807
H	-0.07364	-1.75730	-0.29080
H	0.56568	-2.76626	-1.58193
C	2.02934	-2.19815	-0.09731
H	2.89834	-2.34817	-0.75557
H	2.26324	-1.32185	0.52173
C	1.85712	-3.42398	0.80421
H	0.98603	-3.27063	1.45688
H	1.62373	-4.30171	0.18502
C	3.09291	-3.71405	1.65966
H	3.97185	-3.90102	1.03039
H	3.32795	-2.86322	2.31103
H	2.94470	-4.59310	2.29728
H	3.28581	1.12037	-1.81306

4e_conformer-259

C	-2.37932	2.42042	-0.29306
C	-0.42888	1.25792	-0.14435
N	-1.16197	2.33575	0.33354
H	-0.79522	3.03210	0.96666
C	-2.44333	1.35701	-1.17623
C	-3.35425	3.51203	0.00528
H	-4.26365	3.36963	-0.58467
H	-3.63891	3.52803	1.06511
H	-2.94895	4.50305	-0.23745
C	0.89337	0.96027	0.40349
C	1.22546	1.32341	1.72040
C	1.86501	0.31238	-0.38519
C	2.49554	1.04440	2.22807
H	0.48361	1.79223	2.35925

C	3.12478	0.02009	0.13939
H	1.62720	0.06123	-1.40879
C	3.44841	0.39252	1.45311
H	2.73915	1.32691	3.24848
H	4.43554	0.15991	1.83906
O	4.11316	-0.61430	-0.55755
C	3.82280	-1.04435	-1.88354
H	2.97751	-1.74306	-1.89989
H	3.60088	-0.19448	-2.54027
H	4.72152	-1.55107	-2.23750
C	-1.22553	0.62028	-1.09932
C	-0.95561	-0.65872	-1.84980
H	-1.79521	-0.83132	-2.53540
H	-0.06812	-0.56524	-2.48916
C	-0.79259	-1.89789	-0.94836
H	-0.56452	-2.76617	-1.58231
H	0.07455	-1.75694	-0.29127
C	-2.02818	-2.19888	-0.09738
H	-2.26250	-1.32267	0.52164
H	-2.89720	-2.34945	-0.75549
C	-1.85510	-3.42455	0.80420
H	-1.62129	-4.30219	0.18504
H	-0.98400	-3.27064	1.45673
C	-3.09059	-3.71528	1.65985
H	-3.32603	-2.86454	2.31121
H	-3.96952	-3.90279	1.03073
H	-2.94178	-4.59420	2.29751
H	-3.28637	1.11956	-1.81305

4e_conformer-260

C	-2.89712	-1.90495	-0.44902
C	-1.08241	-0.85217	0.42821
N	-1.53450	-1.94885	-0.29154
H	-0.95012	-2.72823	-0.55920
C	-3.32651	-0.73721	0.15675
C	-3.65194	-2.98615	-1.15083
H	-3.29435	-3.13439	-2.17781
H	-3.56500	-3.95098	-0.63407
H	-4.71329	-2.72795	-1.19863
C	0.34602	-0.66146	0.67818
C	0.80469	-0.07426	1.87479
C	1.29065	-1.05583	-0.27890
C	2.16501	0.12349	2.07735
H	0.09150	0.21184	2.63986
C	2.66105	-0.86655	-0.06163

H	0.97733	-1.48298	-1.22637
C	3.10900	-0.26644	1.12078
H	2.50949	0.57957	3.00136
H	4.16345	-0.10289	1.30510
O	3.47843	-1.28614	-1.07189
C	4.88009	-1.08593	-0.92040
H	5.27011	-1.63058	-0.05214
H	5.33810	-1.47695	-1.82984
H	5.12207	-0.02120	-0.81796
C	-2.19920	-0.06614	0.71638
C	-2.24968	1.29192	1.37130
H	-3.28439	1.65356	1.31219
H	-2.02085	1.22053	2.44377
C	-1.31333	2.34116	0.74034
H	-1.45389	3.29806	1.26213
H	-0.27762	2.04133	0.92202
C	-1.53050	2.54379	-0.76194
H	-1.52508	1.56547	-1.26008
H	-2.53048	2.96926	-0.92800
C	-0.47314	3.44138	-1.42149
H	-0.78802	3.66499	-2.44955
H	-0.43182	4.40709	-0.89743
C	0.92707	2.81654	-1.45664
H	0.90748	1.84424	-1.96429
H	1.63456	3.46128	-1.99060
H	1.32691	2.64896	-0.45076
H	-4.35062	-0.38626	0.18691

4e_conformer-261

C	-2.52192	-2.38710	-0.13955
C	-0.55290	-1.24702	-0.16860
N	-1.26425	-2.29484	0.40059
H	-0.86296	-2.97229	1.03318
C	-2.63346	-1.35784	-1.05777
C	-3.48669	-3.45274	0.26662
H	-3.69746	-3.42529	1.34334
H	-4.43302	-3.31926	-0.26438
H	-3.11104	-4.45788	0.03436
C	0.80658	-0.94696	0.27830
C	1.21570	-1.25703	1.59367
C	1.73958	-0.35661	-0.58458
C	2.51275	-0.97865	2.00809
H	0.50712	-1.68388	2.29652
C	3.03900	-0.06493	-0.15493
H	1.47980	-0.12718	-1.61089

C	3.43932	-0.37917	1.14943
H	2.81354	-1.21759	3.02444
H	4.44140	-0.16520	1.49966
O	3.84757	0.51531	-1.09073
C	5.18717	0.81924	-0.71638
H	5.65031	1.26500	-1.59763
H	5.21735	1.53563	0.11341
H	5.73790	-0.08577	-0.43283
C	-1.40472	-0.63616	-1.09215
C	-1.16735	0.60738	-1.90947
H	-0.31929	0.47549	-2.59355
H	-2.04393	0.76319	-2.55146
C	-0.93770	1.87906	-1.07007
H	-0.03676	1.75048	-0.45727
H	-0.73413	2.71778	-1.75037
C	-2.11912	2.23346	-0.16444
H	-3.02139	2.37267	-0.77889
H	-2.33127	1.38629	0.50126
C	-1.87806	3.49054	0.67624
H	-0.97452	3.34755	1.28574
H	-1.66575	4.33893	0.01034
C	-3.05965	3.83614	1.58593
H	-3.27148	3.01596	2.28299
H	-2.86245	4.73683	2.17850
H	-3.96917	4.01389	0.99885
H	-3.51483	-1.13350	-1.64560

4e_conformer-262

C	-0.07197	3.40197	-0.13110
C	0.35452	1.17354	0.01797
N	0.86875	2.41895	-0.31566
H	1.77097	2.56267	-0.74604
C	-1.20644	2.78091	0.35909
C	0.20445	4.83498	-0.44974
H	1.06166	5.22196	0.11588
H	0.42413	4.98369	-1.51507
H	-0.66682	5.44640	-0.20001
C	1.18196	-0.02859	-0.06748
C	0.61908	-1.29069	-0.35190
C	2.56944	0.05303	0.12383
C	1.43091	-2.41679	-0.42401
H	-0.44280	-1.37790	-0.54614
C	3.38183	-1.08297	0.03228
H	3.04278	0.99649	0.37744
C	2.81426	-2.33403	-0.23743

H	0.98653	-3.38313	-0.64611
H	3.42353	-3.22662	-0.30569
O	4.71508	-0.86886	0.23715
C	5.58682	-1.99319	0.17619
H	5.56242	-2.46436	-0.81377
H	6.58810	-1.60604	0.36937
H	5.33039	-2.73920	0.93805
C	-0.95279	1.38023	0.45825
C	-1.92585	0.35695	0.97090
H	-1.38733	-0.52297	1.34292
H	-2.46872	0.77253	1.83083
C	-2.95634	-0.08930	-0.08219
H	-3.51776	0.79005	-0.42679
H	-2.42955	-0.47584	-0.96593
C	-3.92678	-1.15067	0.44056
H	-3.35606	-2.02703	0.78212
H	-4.44862	-0.76207	1.32753
C	-4.95894	-1.59445	-0.60052
H	-5.52859	-0.71811	-0.94017
H	-4.43471	-1.98020	-1.48615
C	-5.92294	-2.65923	-0.07134
H	-6.64976	-2.95878	-0.83495
H	-5.38031	-3.55832	0.24578
H	-6.48220	-2.28735	0.79600
H	-2.12641	3.28222	0.63312

4e_conformer-263

C	3.31955	-1.72273	0.16475
C	1.68689	-0.15815	-0.07962
N	3.03338	-0.38012	0.17719
H	3.68281	0.34132	0.45578
C	2.13952	-2.37842	-0.13459
C	4.69018	-2.24465	0.44798
H	5.43421	-1.84071	-0.25040
H	5.02473	-1.99027	1.46222
H	4.69986	-3.33408	0.35661
C	1.15804	1.20325	-0.14643
C	1.97910	2.26643	-0.56238
C	-0.17576	1.47784	0.21790
C	1.47708	3.56757	-0.60747
H	2.99887	2.07217	-0.87973
C	-0.67282	2.78175	0.14866
H	-0.79713	0.67291	0.58457
C	0.15752	3.83589	-0.26041
H	2.12046	4.38073	-0.93183

H	-0.24888	4.84134	-0.29834
O	-1.95051	3.13204	0.47912
C	-2.85550	2.09269	0.83577
H	-2.96114	1.36391	0.02429
H	-2.53270	1.57353	1.74647
H	-3.81482	2.57897	1.01756
C	1.10450	-1.40794	-0.29098
C	-0.32041	-1.72579	-0.64730
H	-0.80849	-0.84347	-1.07405
H	-0.33140	-2.49423	-1.43305
C	-1.13468	-2.25716	0.54914
H	-0.58272	-3.09602	0.99216
H	-1.18585	-1.48353	1.32903
C	-2.55124	-2.71963	0.18505
H	-2.49145	-3.45551	-0.63056
H	-2.98642	-3.24990	1.04360
C	-3.50186	-1.58874	-0.22276
H	-3.56751	-0.86683	0.60300
H	-3.08710	-1.04099	-1.07909
C	-4.90439	-2.08987	-0.57535
H	-4.87084	-2.78966	-1.41935
H	-5.35880	-2.61617	0.27307
H	-5.56838	-1.26309	-0.85192
H	2.02873	-3.44999	-0.24516

4e_conformer-264

C	2.56746	-2.27824	-0.19907
C	0.85101	-0.94068	0.45521
N	1.20282	-2.17272	-0.07747
H	0.55175	-2.93081	-0.22530
C	3.10047	-1.07787	0.23283
C	3.22529	-3.52235	-0.70019
H	3.03381	-4.38185	-0.04441
H	2.87364	-3.79472	-1.70350
H	4.30761	-3.37625	-0.75150
C	-0.55484	-0.58052	0.64626
C	-0.97515	0.16237	1.76843
C	-1.51593	-0.97452	-0.29460
C	-2.31329	0.51114	1.91117
H	-0.25565	0.43713	2.53127
C	-2.86484	-0.63500	-0.13436
H	-1.22687	-1.52423	-1.18495
C	-3.27248	0.12126	0.97100
H	-2.62873	1.08483	2.77824
H	-4.30920	0.40181	1.10958

O	-3.70204	-1.07463	-1.11929
C	-5.08119	-0.73311	-1.02248
H	-5.53314	-1.14959	-0.11427
H	-5.22466	0.35408	-1.03206
H	-5.55977	-1.17054	-1.89960
C	2.03084	-0.22650	0.64962
C	2.17975	1.21123	1.06454
H	1.21886	1.60590	1.40552
H	2.86232	1.28472	1.92243
C	2.72216	2.11104	-0.06556
H	2.84399	3.12901	0.32663
H	3.72695	1.76724	-0.34380
C	1.84581	2.13546	-1.32614
H	2.32385	2.78936	-2.06944
H	1.82842	1.12856	-1.76227
C	0.39791	2.60288	-1.11025
H	-0.12177	2.57235	-2.07742
H	-0.12845	1.89135	-0.46401
C	0.27746	4.01090	-0.51995
H	0.69500	4.06181	0.49206
H	0.81241	4.74546	-1.13532
H	-0.77090	4.32488	-0.45915
H	4.15443	-0.82941	0.23918

4e_conformer-265

C	-2.56755	-2.27815	-0.19915
C	-0.85105	-0.94071	0.45520
N	-1.20290	-2.17269	-0.07757
H	-0.55187	-2.93082	-0.22539
C	-3.10050	-1.07780	0.23284
C	-3.22543	-3.52223	-0.70031
H	-2.87366	-3.79467	-1.70356
H	-3.03412	-4.38172	-0.04448
H	-4.30773	-3.37604	-0.75178
C	0.55483	-0.58061	0.64626
C	0.97517	0.16210	1.76855
C	1.51589	-0.97450	-0.29466
C	2.31331	0.51080	1.91133
H	0.25567	0.43675	2.53142
C	2.86481	-0.63503	-0.13439
H	1.22680	-1.52407	-1.18508
C	3.27248	0.12105	0.97108
H	2.62878	1.08435	2.77849
H	4.30921	0.40155	1.10970
O	3.70200	-1.07454	-1.11941

C	5.08115	-0.73305	-1.02258
H	5.53311	-1.14967	-0.11443
H	5.55973	-1.17035	-1.89976
H	5.22464	0.35413	-1.03199
C	-2.03084	-0.22649	0.64968
C	-2.17969	1.21127	1.06457
H	-2.86226	1.28481	1.92245
H	-1.21878	1.60589	1.40556
C	-2.72206	2.11106	-0.06556
H	-3.72683	1.76724	-0.34383
H	-2.84393	3.12903	0.32662
C	-1.84565	2.13549	-1.32610
H	-1.82819	1.12859	-1.76220
H	-2.32370	2.78935	-2.06944
C	-0.39778	2.60301	-1.11017
H	0.12860	1.89151	-0.46390
H	0.12192	2.57248	-2.07732
C	-0.27745	4.01104	-0.51989
H	0.77089	4.32511	-0.45911
H	-0.81247	4.74555	-1.13525
H	-0.69496	4.06193	0.49214
H	-4.15445	-0.82930	0.23922

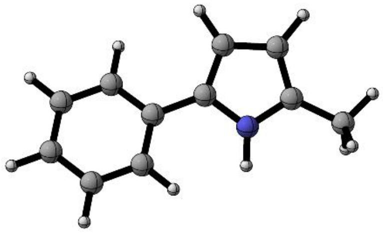

*** Note: the conformers for 4f were also used for 4i, 4j, and 4k
4f_conformer-001

C	-0.55411	0.34176	-0.04717
C	-1.27294	1.51364	-0.24960
C	-2.65606	1.19157	-0.19955
C	-2.76770	-0.16874	0.03650
N	-1.48866	-0.66742	0.11442
H	-1.26719	-1.62197	0.35783
H	-0.84305	2.48627	-0.44716
H	-3.48752	1.87204	-0.32816
C	-3.96831	-1.04200	0.20131
H	-3.99722	-1.84414	-0.54724
H	-3.99660	-1.51825	1.18997
H	-4.87864	-0.44677	0.09087
C	0.88430	0.09756	-0.01714
C	1.42268	-1.18845	-0.21188

C	1.77910	1.16245	0.20428
C	2.79994	-1.40086	-0.17842
H	0.76470	-2.02840	-0.41638
C	3.15446	0.94931	0.22419
H	1.38573	2.15973	0.37648
C	3.67523	-0.33464	0.03720
H	3.18956	-2.40334	-0.33238
H	3.82327	1.78792	0.39747
H	4.74818	-0.50075	0.05942

4f_conformer-002

C	0.55411	0.34176	-0.04717
C	1.27294	1.51364	-0.24960
C	2.65606	1.19157	-0.19955
C	2.76770	-0.16874	0.03650
N	1.48866	-0.66742	0.11442
H	1.26719	-1.62197	0.35783
H	0.84305	2.48627	-0.44716
H	3.48752	1.87204	-0.32816
C	3.96831	-1.04200	0.20131
H	3.99660	-1.51825	1.18997
H	3.99722	-1.84414	-0.54724
H	4.87864	-0.44677	0.09087
C	-0.88430	0.09756	-0.01714
C	-1.42268	-1.18845	-0.21188
C	-1.77910	1.16245	0.20428
C	-2.79994	-1.40086	-0.17842
H	-0.76470	-2.02840	-0.41638
C	-3.15446	0.94931	0.22419
H	-1.38573	2.15973	0.37648
C	-3.67523	-0.33464	0.03720
H	-3.18956	-2.40334	-0.33238
H	-3.82327	1.78792	0.39747
H	-4.74818	-0.50075	0.05942

4f_conformer-003

C	-0.55413	0.34176	0.04715
C	-1.27296	1.51366	0.24949
C	-2.65610	1.19159	0.19944
C	-2.76773	-0.16874	-0.03650
N	-1.48869	-0.66744	-0.11439
H	-1.26726	-1.62206	-0.35749
H	-0.84307	2.48632	0.44693
H	-3.48758	1.87206	0.32796

C	-3.96833	-1.04201	-0.20120
H	-3.99642	-1.51868	-1.18967
H	-3.99745	-1.84384	0.54768
H	-4.87864	-0.44667	-0.09120
C	0.88428	0.09755	0.01714
C	1.42271	-1.18847	0.21177
C	1.77912	1.16245	-0.20418
C	2.79998	-1.40086	0.17833
H	0.76477	-2.02848	0.41611
C	3.15448	0.94933	-0.22408
H	1.38577	2.15976	-0.37628
C	3.67529	-0.33463	-0.03717
H	3.18960	-2.40337	0.33220
H	3.82328	1.78797	-0.39728
H	4.74824	-0.50073	-0.05939

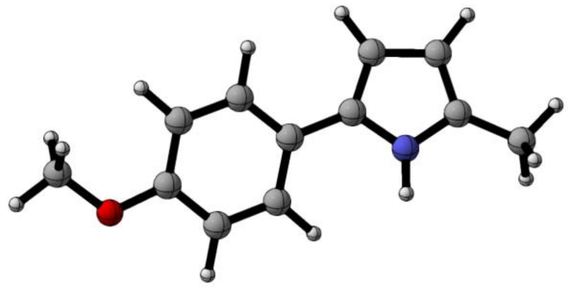

4g_conformer-001

27

C	1.45176	0.35045	-0.06892
C	2.17252	1.51653	-0.29147
C	3.55632	1.19770	-0.20410
C	3.66515	-0.15422	0.07193
N	2.38342	-0.65224	0.13987
H	2.15641	-1.59866	0.40865
H	1.74457	2.48181	-0.52656
H	4.38941	1.87561	-0.33668
C	0.01291	0.10211	-0.05362
C	-0.52495	-1.18300	-0.26714
C	-0.89174	1.15224	0.16946
C	-1.89517	-1.40409	-0.24954
H	0.13409	-2.02149	-0.47402
C	-2.27064	0.94657	0.17525
H	-0.51044	2.15150	0.35703
C	-2.78235	-0.34075	-0.03049

H	-2.30136	-2.39676	-0.41713
H	-2.92988	1.78766	0.35313
O	-4.11034	-0.65788	-0.03610
C	-5.04536	0.39511	0.17447
H	-4.96378	1.16307	-0.60430
H	-6.03234	-0.06677	0.12720
H	-4.90715	0.86119	1.15760
C	4.86241	-1.02232	0.28263
H	4.87237	-1.47352	1.28349
H	4.90716	-1.84357	-0.44434
H	5.77465	-0.42934	0.17486

4g_conformer-002

27

C	-1.45176	0.35057	-0.06901
C	-2.17262	1.51653	-0.29178
C	-3.55642	1.19756	-0.20431
C	-3.66511	-0.15430	0.07201
N	-2.38331	-0.65219	0.13995
H	-2.15616	-1.59841	0.40938
H	-1.74477	2.48178	-0.52717
H	-4.38959	1.87536	-0.33704
C	-0.01290	0.10227	-0.05371
C	0.52490	-1.18282	-0.26750
C	0.89177	1.15231	0.16964
C	1.89510	-1.40399	-0.24987
H	-0.13423	-2.02118	-0.47466
C	2.27066	0.94658	0.17544
H	0.51048	2.15154	0.35745
C	2.78231	-0.34073	-0.03054
H	2.30125	-2.39663	-0.41766
H	2.92993	1.78759	0.35354
O	4.11029	-0.65793	-0.03613
C	5.04538	0.39494	0.17472
H	4.90711	0.86085	1.15792
H	6.03232	-0.06702	0.12745
H	4.96394	1.16305	-0.60391
C	-4.86227	-1.02245	0.28302
H	-4.87238	-1.47300	1.28417
H	-5.77458	-0.42970	0.17466
H	-4.90672	-1.84419	-0.44342

4g_conformer-003

27

C	-1.47280	0.37735	-0.06105
C	-2.31588	1.45698	-0.29028
C	-3.65651	0.98482	-0.22778
C	-3.61773	-0.37233	0.04102
N	-2.28957	-0.72447	0.12798
H	-1.96245	-1.64077	0.39824
H	-1.99523	2.46564	-0.51367
H	-4.55802	1.56623	-0.37071
C	-0.01568	0.29302	-0.02353
C	0.66840	-0.91603	-0.22151
C	0.76026	1.44684	0.21122
C	2.06207	-0.98869	-0.17988
H	0.11424	-1.82523	-0.43722
C	2.14495	1.39111	0.24112
H	0.26344	2.39622	0.38667
C	2.81085	0.17017	0.05045
H	2.54584	-1.94472	-0.33985
H	2.73523	2.28347	0.42520
O	4.17411	0.21751	0.10781
C	4.88805	-1.00129	-0.07102
H	4.69860	-1.43327	-1.06117
H	5.94469	-0.74564	0.01749
H	4.62370	-1.73523	0.69996
C	-4.71383	-1.36929	0.23054
H	-4.65353	-2.18901	-0.49698
H	-4.69076	-1.82068	1.23114
H	-5.68462	-0.88181	0.10668

4g_conformer-004

27

C	1.47280	0.37735	-0.06104
C	2.31587	1.45699	-0.29027
C	3.65651	0.98483	-0.22777
C	3.61773	-0.37233	0.04101
N	2.28957	-0.72448	0.12798
H	1.96245	-1.64078	0.39821
H	1.99523	2.46564	-0.51364
H	4.55801	1.56624	-0.37070
C	0.01568	0.29302	-0.02353
C	-0.66840	-0.91604	-0.22149
C	-0.76026	1.44684	0.21121
C	-2.06207	-0.98869	-0.17986

H	-0.11424	-1.82524	-0.43718
C	-2.14495	1.39111	0.24111
H	-0.26344	2.39622	0.38665
C	-2.81085	0.17017	0.05045
H	-2.54584	-1.94472	-0.33983
H	-2.73523	2.28347	0.42518
O	-4.17411	0.21751	0.10781
C	-4.88805	-1.00129	-0.07102
H	-4.62371	-1.73522	0.69996
H	-5.94469	-0.74564	0.01748
H	-4.69860	-1.43328	-1.06116
C	4.71383	-1.36929	0.23053
H	4.69077	-1.82069	1.23111
H	4.65354	-2.18901	-0.49700
H	5.68462	-0.88180	0.10667

4g_conformer-005

27

C	-1.45179	0.35052	-0.06884
C	-2.17261	1.51657	-0.29131
C	-3.55636	1.19764	-0.20398
C	-3.66511	-0.15431	0.07196
N	-2.38336	-0.65224	0.13992
H	-2.15627	-1.59872	0.40841
H	-1.74471	2.48189	-0.52631
H	-4.38951	1.87550	-0.33651
C	-0.01293	0.10223	-0.05362
C	0.52494	-1.18289	-0.26707
C	0.89173	1.15236	0.16926
C	1.89514	-1.40397	-0.24960
H	-0.13412	-2.02139	-0.47389
C	2.27064	0.94673	0.17496
H	0.51043	2.15166	0.35669
C	2.78235	-0.34060	-0.03063
H	2.30133	-2.39662	-0.41721
H	2.92984	1.78790	0.35257
O	4.11024	-0.65793	-0.03613
C	5.04546	0.39480	0.17474
H	4.90723	0.86079	1.15790
H	6.03232	-0.06733	0.12759
H	4.96419	1.16288	-0.60394
C	-4.86233	-1.02249	0.28255
H	-4.90706	-1.84365	-0.44452
H	-4.87226	-1.47383	1.28335

H	-5.77459	-0.42953	0.17486

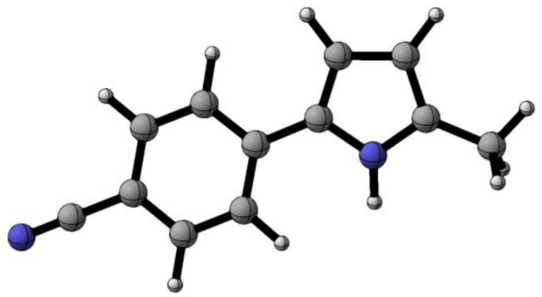

4h_conformer-001

24

C	-1.29192	0.35258	-0.03138
C	-2.06714	1.50209	-0.16902
C	-3.42863	1.11611	-0.13436
C	-3.47642	-0.26174	0.02963
N	-2.18050	-0.70597	0.07802
H	-1.92124	-1.66462	0.26031
H	-1.68588	2.50398	-0.31024
H	-4.29090	1.76313	-0.22326
C	0.14830	0.18249	-0.01946
C	0.75262	-1.08493	-0.16143
C	0.99316	1.30394	0.13357
C	2.13138	-1.23028	-0.14471
H	0.14231	-1.96979	-0.30900
C	2.37085	1.17027	0.14290
H	0.55628	2.28843	0.26038
C	2.95717	-0.10274	0.00707
H	2.57642	-2.21311	-0.25731
H	3.00284	2.04360	0.26400
C	-4.63734	-1.19270	0.15184
H	-5.57276	-0.63359	0.06903
H	-4.64055	-1.71483	1.11705
H	-4.62920	-1.95819	-0.63407
C	4.37695	-0.24702	0.02295
N	5.53604	-0.36453	0.03591

4h_conformer-002

24

C	1.29192	0.35258	-0.03138

C	2.06714	1.50209	-0.16903
C	3.42863	1.11611	-0.13436
C	3.47642	-0.26174	0.02964
N	2.18050	-0.70597	0.07802
H	1.92124	-1.66462	0.26032
H	1.68588	2.50398	-0.31025
H	4.29090	1.76312	-0.22326
C	-0.14830	0.18249	-0.01947
C	-0.75262	-1.08493	-0.16144
C	-0.99316	1.30394	0.13357
C	-2.13138	-1.23027	-0.14472
H	-0.14231	-1.96979	-0.30902
C	-2.37085	1.17027	0.14291
H	-0.55628	2.28843	0.26038
C	-2.95717	-0.10274	0.00707
H	-2.57642	-2.21311	-0.25732
H	-3.00284	2.04360	0.26401
C	4.63734	-1.19270	0.15185
H	4.64051	-1.71488	1.11704
H	4.62923	-1.95816	-0.63409
H	5.57276	-0.63359	0.06910
C	-4.37695	-0.24703	0.02295
N	-5.53604	-0.36453	0.03592

4h_conformer-003

24

C	1.29192	0.35255	0.03129
C	2.06713	1.50213	0.16857
C	3.42861	1.11619	0.13403
C	3.47644	-0.26172	-0.02951
N	2.18055	-0.70600	-0.07778
H	1.92133	-1.66476	-0.25956
H	1.68585	2.50406	0.30947
H	4.29086	1.76326	0.22270
C	-0.14829	0.18244	0.01937
C	-0.75264	-1.08502	0.16092
C	-0.99316	1.30395	-0.13326
C	-2.13140	-1.23034	0.14426
H	-0.14236	-1.96996	0.30814
C	-2.37085	1.17030	-0.14253
H	-0.55631	2.28848	-0.25981
C	-2.95719	-0.10275	-0.00706
H	-2.57644	-2.21321	0.25655
H	-3.00284	2.04368	-0.26330

C	4.63739	-1.19268	-0.15142
H	4.62927	-1.95793	0.63473
H	4.64061	-1.71512	-1.11647
H	5.57279	-0.63352	-0.06878
C	-4.37697	-0.24700	-0.02285
N	-5.53606	-0.36447	-0.03572

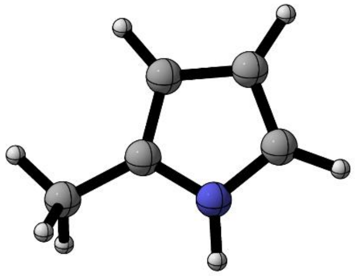

4l_conformer-001

C	0.65692	0.08280	0.00004
C	-0.20086	1.16780	0.00001
C	-1.53951	0.67088	-0.00008
C	-1.46391	-0.70614	-0.00010
N	-0.13023	-1.04859	-0.00002
H	0.22329	-1.99449	-0.00002
H	0.10849	2.20511	0.00004
H	-2.44995	1.25566	-0.00012
H	-2.23272	-1.46499	-0.00015
C	2.15015	0.00950	0.00012
H	2.53727	-0.51454	0.88375
H	2.53738	-0.51439	-0.88355
H	2.57111	1.01875	0.00023

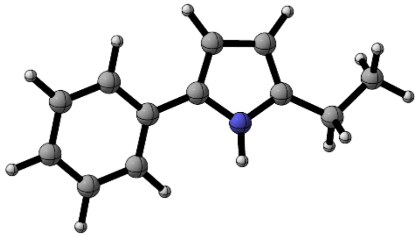

4m_conformer-001

C	0.07451	0.36356	-0.05276
C	0.77603	1.54482	-0.25823
C	2.16460	1.24340	-0.21252
C	2.29664	-0.11575	0.02431
N	1.02427	-0.63176	0.10625
H	0.81758	-1.58896	0.35252
H	0.33257	2.51145	-0.45547
H	2.98077	1.93994	-0.34526
C	3.50619	-0.98639	0.18865

H	3.47393	-1.48086	1.17036
H	3.47909	-1.79786	-0.55270
C	-1.36013	0.09862	-0.01812
C	-1.88053	-1.19460	-0.21384
C	-2.26937	1.15002	0.20885
C	-3.25446	-1.42698	-0.17591
H	-1.21109	-2.02434	-0.42269
C	-3.64143	0.91698	0.23317
H	-1.88967	2.15246	0.38187
C	-4.14425	-0.37398	0.04523
H	-3.63013	-2.43465	-0.33075
H	-4.32170	1.74543	0.41072
H	-5.21460	-0.55563	0.07097
C	4.82206	-0.21653	0.05196
H	4.90278	0.25290	-0.93436
H	4.89620	0.57315	0.80734
H	5.67528	-0.88978	0.17899

4m_conformer-002

C	-0.07451	0.36356	-0.05276
C	-0.77603	1.54482	-0.25823
C	-2.16460	1.24340	-0.21252
C	-2.29664	-0.11575	0.02431
N	-1.02427	-0.63176	0.10625
H	-0.81758	-1.58896	0.35252
H	-0.33257	2.51145	-0.45547
H	-2.98077	1.93994	-0.34526
C	-3.50619	-0.98639	0.18865
H	-3.47909	-1.79786	-0.55270
H	-3.47393	-1.48086	1.17036
C	1.36013	0.09862	-0.01812
C	1.88053	-1.19460	-0.21384
C	2.26937	1.15002	0.20885
C	3.25446	-1.42698	-0.17591
H	1.21109	-2.02434	-0.42269
C	3.64143	0.91698	0.23317
H	1.88967	2.15246	0.38187
C	4.14425	-0.37398	0.04523
H	3.63013	-2.43465	-0.33075
H	4.32170	1.74543	0.41072
H	5.21460	-0.55563	0.07097
C	-4.82206	-0.21653	0.05196
H	-4.90278	0.25290	-0.93436
H	-5.67528	-0.88978	0.17899
H	-4.89620	0.57315	0.80734

4m_conformer-003

C	0.12429	0.55312	-0.00579
C	0.70880	1.77499	-0.31689
C	2.11516	1.63635	-0.16772
C	2.37490	0.33709	0.23681
N	1.16102	-0.30627	0.31626
H	1.03827	-1.24317	0.67228
H	0.17767	2.65757	-0.64686
H	2.86578	2.39595	-0.34263
C	3.66397	-0.36615	0.52629
H	4.46054	0.38497	0.55488
H	3.62948	-0.82009	1.52665
C	-1.27424	0.13611	-0.00408
C	-1.64262	-1.22044	-0.07836
C	-2.30060	1.09778	0.06652
C	-2.98411	-1.59920	-0.07238
H	-0.87822	-1.98734	-0.16704
C	-3.63949	0.71708	0.05898
H	-2.03929	2.14885	0.14367
C	-3.99128	-0.63447	-0.00558
H	-3.24181	-2.65306	-0.13097
H	-4.41254	1.47854	0.11527
H	-5.03610	-0.93057	-0.00439
C	4.01290	-1.45076	-0.50814
H	4.09619	-1.01676	-1.51028
H	4.96528	-1.93170	-0.25993
H	3.24163	-2.22833	-0.54240

4m_conformer-004

C	-0.12430	0.55318	-0.00583
C	-0.70878	1.77507	-0.31696
C	-2.11515	1.63645	-0.16785
C	-2.37493	0.33720	0.23668
N	-1.16107	-0.30618	0.31618
H	-1.03835	-1.24309	0.67219
H	-0.17762	2.65764	-0.64692
H	-2.86575	2.39607	-0.34278
C	-3.66401	-0.36601	0.52618
H	-3.62974	-0.81947	1.52676
H	-4.46064	0.38507	0.55422
C	1.27420	0.13613	-0.00409
C	2.30060	1.09777	0.06663
C	1.64255	-1.22042	-0.07846

C	3.63947	0.71703	0.05913
H	2.03931	2.14884	0.14385
C	2.98402	-1.59922	-0.07245
H	0.87812	-1.98729	-0.16725
C	3.99123	-0.63453	-0.00552
H	4.41255	1.47846	0.11551
H	3.24169	-2.65309	-0.13110
H	5.03603	-0.93066	-0.00430
C	-4.01259	-1.45114	-0.50782
H	-3.24127	-2.22868	-0.54149
H	-4.96501	-1.93203	-0.25962
H	-4.09565	-1.01764	-1.51019

4m_conformer-005

C	0.12971	0.52435	-0.13946
C	0.72265	1.78060	-0.18787
C	2.12723	1.59624	-0.29581
C	2.37865	0.23376	-0.30860
N	1.15918	-0.39757	-0.22276
H	1.04184	-1.39707	-0.14081
H	0.19317	2.72375	-0.18381
H	2.88147	2.36920	-0.36427
C	3.66556	-0.52919	-0.35262
H	3.63316	-1.27572	-1.15827
H	4.46600	0.17218	-0.61145
C	-1.27378	0.13465	-0.04871
C	-1.71415	-1.14752	-0.42762
C	-2.23353	1.05081	0.42366
C	-3.05903	-1.50081	-0.32923
H	-1.00762	-1.86973	-0.82693
C	-3.57768	0.69935	0.50830
H	-1.91396	2.03917	0.73987
C	-4.00003	-0.58062	0.13686
H	-3.37271	-2.49700	-0.62868
H	-4.29803	1.42456	0.87677
H	-5.04799	-0.85587	0.20981
C	4.00636	-1.23180	0.97370
H	4.96029	-1.76470	0.89614
H	4.08294	-0.50363	1.78821
H	3.23548	-1.96114	1.24700

4m_conformer-006

C	-0.12971	0.52435	-0.13946
C	-0.72265	1.78061	-0.18785

C	-2.12723	1.59625	-0.29579
C	-2.37865	0.23377	-0.30859
N	-1.15919	-0.39756	-0.22276
H	-1.04184	-1.39706	-0.14081
H	-0.19317	2.72376	-0.18379
H	-2.88147	2.36921	-0.36425
C	-3.66556	-0.52918	-0.35262
H	-4.46601	0.17220	-0.61142
H	-3.63318	-1.27568	-1.15829
C	1.27378	0.13465	-0.04871
C	1.71415	-1.14752	-0.42762
C	2.23353	1.05082	0.42365
C	3.05902	-1.50081	-0.32922
H	1.00761	-1.86973	-0.82691
C	3.57768	0.69934	0.50829
H	1.91397	2.03917	0.73986
C	4.00002	-0.58062	0.13686
H	3.37270	-2.49701	-0.62868
H	4.29804	1.42455	0.87675
H	5.04799	-0.85588	0.20980
C	-4.00634	-1.23182	0.97369
H	-4.08291	-0.50369	1.78822
H	-4.96027	-1.76474	0.89612
H	-3.23546	-1.96117	1.24695

4m_conformer-007

C	-0.07451	0.36360	-0.05273
C	-0.77604	1.54485	-0.25826
C	-2.16461	1.24338	-0.21255
C	-2.29661	-0.11576	0.02434
N	-1.02423	-0.63173	0.10633
H	-0.81749	-1.58895	0.35252
H	-0.33257	2.51146	-0.45555
H	-2.98081	1.93989	-0.34533
C	-3.50615	-0.98639	0.18870
H	-3.47900	-1.79793	-0.55259
H	-3.47392	-1.48079	1.17045
C	1.36011	0.09866	-0.01810
C	2.26937	1.15003	0.20890
C	1.88047	-1.19458	-0.21386
C	3.64143	0.91695	0.23317
H	1.88968	2.15246	0.38199
C	3.25440	-1.42700	-0.17596
H	1.21097	-2.02428	-0.42270
C	4.14423	-0.37402	0.04518

H	4.32174	1.74537	0.41075
H	3.63005	-2.43467	-0.33083
H	5.21458	-0.55569	0.07089
C	-4.82201	-0.21656	0.05190
H	-4.90270	0.25279	-0.93447
H	-5.67524	-0.88981	0.17895
H	-4.89619	0.57319	0.80721

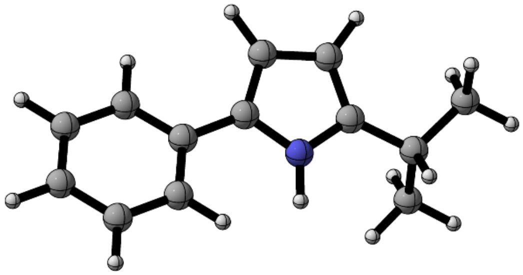

4n_conformer-001

C	0.25011	0.50119	-0.07225
C	-0.37011	1.74459	-0.06987
C	-1.77403	1.53366	-0.13623
C	-1.99853	0.16602	-0.17395
N	-0.76294	-0.43950	-0.14405
H	-0.62227	-1.43799	-0.09292
H	0.13839	2.69917	-0.05975
H	-2.53933	2.29675	-0.16322
C	1.66343	0.13885	-0.03934
C	2.11543	-1.12599	-0.46042
C	2.62108	1.06510	0.41736
C	3.46988	-1.45285	-0.41839
H	1.40945	-1.85505	-0.84812
C	3.97423	0.74019	0.44610
H	2.29341	2.04005	0.76532
C	4.40852	-0.52275	0.03252
H	3.79245	-2.43601	-0.74979
H	4.69277	1.47268	0.80354
H	5.46381	-0.77736	0.06160
C	-3.47034	-1.40295	1.12779
H	-3.58004	-0.69660	1.95876
H	-4.37315	-2.02237	1.08036
H	-2.62241	-2.05903	1.35275
C	-4.48255	0.23620	-0.51205
H	-4.63326	0.98696	0.27242
H	-4.36032	0.76103	-1.46515
H	-5.38840	-0.37599	-0.56889
C	-3.26630	-0.64253	-0.19796
H	-3.17544	-1.39215	-0.99842

4n_conformer-002

C	-0.25008	0.50130	-0.07216
C	0.37009	1.74472	-0.06982
C	1.77401	1.53383	-0.13624
C	1.99857	0.16620	-0.17397
N	0.76299	-0.43936	-0.14400
H	0.62233	-1.43784	-0.09272
H	-0.13843	2.69930	-0.05969
H	2.53930	2.29694	-0.16331
C	-1.66339	0.13891	-0.03926
C	-2.62110	1.06513	0.41736
C	-2.11531	-1.12597	-0.46031
C	-3.97424	0.74017	0.44603
H	-2.29350	2.04011	0.76529
C	-3.46975	-1.45289	-0.41834
H	-1.40928	-1.85501	-0.84796
C	-4.40846	-0.52281	0.03247
H	-4.69283	1.47264	0.80340
H	-3.79226	-2.43607	-0.74973
H	-5.46374	-0.77746	0.06150
C	4.48272	0.23689	-0.51071
H	4.63300	0.98667	0.27478
H	5.38862	-0.37519	-0.56788
H	4.36094	0.76291	-1.46321
C	3.46981	-1.40437	1.12657
H	2.62197	-2.06105	1.35014
H	4.37284	-2.02345	1.07888
H	3.57877	-0.69908	1.95853
C	3.26637	-0.64229	-0.19832
H	3.17596	-1.39093	-0.99976

4n_conformer-003

C	-0.25307	-0.50745	0.06205
C	0.36457	-1.70033	0.41620
C	1.76947	-1.51673	0.30139
C	1.99640	-0.21725	-0.12474
N	0.76244	0.38018	-0.25062
H	0.61675	1.30291	-0.63404
H	-0.14350	-2.59430	0.75183
H	2.53422	-2.25104	0.51283
C	-1.66447	-0.13986	0.01173
C	-2.08111	1.20432	0.04223
C	-2.65480	-1.13833	-0.06281
C	-3.43415	1.53566	-0.00877

H	-1.34621	1.99937	0.13240
C	-4.00567	-0.80466	-0.10028
H	-2.35543	-2.18113	-0.10692
C	-4.40533	0.53490	-0.07838
H	-3.72944	2.58094	0.01675
H	-4.75030	-1.59381	-0.15835
H	-5.45929	0.79409	-0.11458
C	3.48416	1.65973	0.63709
H	4.38424	2.23681	0.39654
H	2.63602	2.35199	0.66942
H	3.60737	1.23022	1.63804
C	4.47631	-0.39598	-0.45527
H	4.63638	-0.88093	0.51476
H	4.34174	-1.17934	-1.20823
H	5.38184	0.16781	-0.70173
C	3.26479	0.54166	-0.40161
H	3.16578	1.02113	-1.38737

4n_conformer-004

C	0.25309	-0.50733	0.06212
C	-0.36457	-1.70017	0.41636
C	-1.76947	-1.51656	0.30152
C	-1.99637	-0.21710	-0.12470
N	-0.76240	0.38030	-0.25062
H	-0.61669	1.30298	-0.63418
H	0.14348	-2.59412	0.75206
H	-2.53423	-2.25083	0.51302
C	1.66450	-0.13980	0.01173
C	2.08120	1.20437	0.04196
C	2.65479	-1.13833	-0.06259
C	3.43426	1.53563	-0.00909
H	1.34634	1.99947	0.13196
C	4.00568	-0.80473	-0.10011
H	2.35539	-2.18112	-0.10650
C	4.40540	0.53482	-0.07848
H	3.72959	2.58091	0.01622
H	4.75027	-1.59393	-0.15801
H	5.45937	0.79396	-0.11472
C	-4.47612	-0.39590	-0.45642
H	-4.63658	-0.88161	0.51317
H	-5.38161	0.16796	-0.70286
H	-4.34110	-1.17868	-1.20991
C	-3.48470	1.65901	0.63807
H	-4.38469	2.23626	0.39757
H	-3.60840	1.22862	1.63858

H	-2.63659	2.35125	0.67144
C	-3.26475	0.54185	-0.40149
H	-3.16537	1.02216	-1.38679

4n_conformer-005

C	0.25010	0.50126	-0.07209
C	-0.37007	1.74468	-0.06979
C	-1.77400	1.53378	-0.13622
C	-1.99855	0.16616	-0.17390
N	-0.76298	-0.43940	-0.14390
H	-0.62230	-1.43788	-0.09256
H	0.13845	2.69925	-0.05971
H	-2.53928	2.29690	-0.16333
C	1.66341	0.13888	-0.03922
C	2.11534	-1.12599	-0.46028
C	2.62112	1.06512	0.41735
C	3.46979	-1.45289	-0.41836
H	1.40931	-1.85505	-0.84789
C	3.97427	0.74018	0.44598
H	2.29351	2.04009	0.76529
C	4.40849	-0.52279	0.03241
H	3.79229	-2.43607	-0.74975
H	4.69286	1.47267	0.80331
H	5.46378	-0.77743	0.06139
C	-3.47007	-1.40408	1.12676
H	-4.37315	-2.02308	1.07907
H	-2.62233	-2.06079	1.35060
H	-3.57909	-0.69860	1.95855
C	-4.48265	0.23682	-0.51109
H	-4.63305	0.98679	0.27419
H	-4.36069	0.76259	-1.46369
H	-5.38855	-0.37526	-0.56826
C	-3.26636	-0.64232	-0.19827
H	-3.17583	-1.39115	-0.99951

4n_conformer-006

C	-0.25308	0.50736	-0.06208
C	0.36457	1.70022	-0.41628
C	1.76947	1.51661	-0.30146
C	1.99638	0.21715	0.12472
N	0.76241	-0.38027	0.25062
H	0.61672	-1.30297	0.63412
H	-0.14349	2.59418	-0.75196
H	2.53423	2.25089	-0.51294

C	-1.66449	0.13982	-0.01172
C	-2.65479	1.13833	0.06263
C	-2.08117	-1.20435	-0.04201
C	-4.00567	0.80471	0.10012
H	-2.35540	2.18113	0.10658
C	-3.43423	-1.53564	0.00902
H	-1.34630	-1.99945	-0.13204
C	-4.40538	-0.53484	0.07844
H	-4.75027	1.59390	0.15805
H	-3.72954	-2.58092	-0.01633
H	-5.45934	-0.79399	0.11466
C	3.48452	-1.65921	-0.63781
H	3.60812	-1.22905	-1.63844
H	4.38451	-2.23645	-0.39730
H	2.63637	-2.35141	-0.67092
C	4.47618	0.39591	0.45608
H	4.34131	1.17884	1.20944
H	5.38168	-0.16794	0.70249
H	4.63650	0.88143	-0.51363
C	3.26476	-0.54180	0.40151
H	3.16549	-1.02188	1.38694

4n_conformer-007

C	0.21052	0.64029	-0.09211
C	-0.29468	1.91941	-0.29158
C	-1.71331	1.83513	-0.27573
C	-2.06021	0.51107	-0.06370
N	-0.88444	-0.19817	0.03317
H	-0.82792	-1.17785	0.26959
H	0.29816	2.80760	-0.46352
H	-2.41468	2.64790	-0.41185
C	1.58519	0.15409	-0.03282
C	1.90103	-1.20200	-0.23949
C	2.64198	1.04689	0.23061
C	3.22038	-1.64746	-0.17653
H	1.11519	-1.91372	-0.47661
C	3.95973	0.60121	0.27987
H	2.41992	2.09408	0.41241
C	4.25851	-0.75004	0.08109
H	3.43725	-2.69941	-0.34036
H	4.75693	1.31026	0.48555
H	5.28634	-1.09763	0.12640
C	-3.64399	-1.16260	-1.07923
H	-4.64991	-1.59102	-1.00685
H	-3.53966	-0.68750	-2.06014

H	-2.92466	-1.98873	-1.02652
C	-3.60301	-0.80551	1.43262
H	-2.89170	-1.62642	1.58600
H	-3.45909	-0.07961	2.23953
H	-4.61252	-1.22277	1.51772
C	-3.40484	-0.14752	0.05425
H	-4.14641	0.65433	-0.04695

4n_conformer-008

C	-0.21052	0.64029	-0.09209
C	0.29468	1.91942	-0.29154
C	1.71331	1.83515	-0.27568
C	2.06021	0.51108	-0.06366
N	0.88444	-0.19817	0.03319
H	0.82792	-1.17784	0.26961
H	-0.29816	2.80761	-0.46348
H	2.41468	2.64792	-0.41177
C	-1.58519	0.15409	-0.03282
C	-1.90102	-1.20200	-0.23951
C	-2.64199	1.04689	0.23061
C	-3.22037	-1.64746	-0.17657
H	-1.11518	-1.91371	-0.47663
C	-3.95973	0.60120	0.27986
H	-2.41993	2.09407	0.41244
C	-4.25850	-0.75005	0.08106
H	-3.43723	-2.69941	-0.34041
H	-4.75694	1.31025	0.48555
H	-5.28634	-1.09764	0.12636
C	3.60295	-0.80566	1.43259
H	4.61247	-1.22292	1.51769
H	3.45898	-0.07985	2.23957
H	2.89164	-1.62660	1.58584
C	3.64404	-1.16247	-1.07929
H	2.92470	-1.98861	-1.02670
H	3.53975	-0.68727	-2.06016
H	4.64995	-1.59091	-1.00691
C	3.40484	-0.14751	0.05429
H	4.14641	0.65435	-0.04680

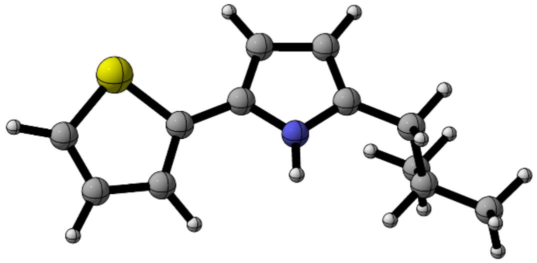

4o_conformer-001

C	0.69498	0.51587	-0.11014
C	0.33177	1.83203	0.15227
C	-1.06204	1.95244	-0.09284
C	-1.53355	0.71497	-0.50206
N	-0.45782	-0.14347	-0.50035
H	-0.49165	-1.10253	-0.81480
H	1.00079	2.60767	0.50075
H	-1.66782	2.84129	0.02263
C	1.97007	-0.15791	-0.02318
C	2.24492	-1.50878	0.00690
S	3.46207	0.76697	0.05256
C	3.63876	-1.80283	0.09727
H	1.47497	-2.27262	-0.00970
C	4.42211	-0.68351	0.14027
H	4.03702	-2.81068	0.13949
H	5.49805	-0.61266	0.21996
C	-2.90812	0.25163	-0.86201
H	-2.89399	-0.23575	-1.84730
C	-3.53810	-0.72274	0.16220
H	-2.86306	-1.58560	0.26019
C	-4.88823	-1.23058	-0.35401
C	-3.68100	-0.07010	1.54056
H	-2.71538	0.28230	1.91740
H	-4.09639	-0.77710	2.26756
H	-4.35575	0.79446	1.48883
H	-3.55365	1.13307	-0.95902
H	-4.78357	-1.72549	-1.32670
H	-5.33293	-1.94761	0.34540
H	-5.59436	-0.39891	-0.47458

4o_conformer-002

C	-0.69498	0.51586	-0.11015
C	-0.33176	1.83202	0.15226
C	1.06205	1.95243	-0.09285
C	1.53355	0.71496	-0.50207
N	0.45782	-0.14348	-0.50035
H	0.49165	-1.10254	-0.81480
H	-1.00079	2.60767	0.50074
H	1.66782	2.84128	0.02261
C	-1.97007	-0.15791	-0.02319
C	-2.24493	-1.50878	0.00690
S	-3.46207	0.76698	0.05256
C	-3.63877	-1.80282	0.09728

H	-1.47498	-2.27262	-0.00970
C	-4.42211	-0.68350	0.14028
H	-4.03703	-2.81067	0.13949
H	-5.49805	-0.61264	0.21997
C	2.90812	0.25161	-0.86202
H	3.55364	1.13306	-0.95904
C	3.53810	-0.72274	0.16221
H	2.86307	-1.58560	0.26022
C	3.68101	-0.07007	1.54056
C	4.88823	-1.23059	-0.35399
H	5.59436	-0.39892	-0.47459
H	5.33294	-1.94760	0.34542
H	4.78357	-1.72551	-1.32668
H	2.89399	-0.23578	-1.84729
H	4.35576	0.79449	1.48880
H	2.71539	0.28233	1.91739
H	4.09640	-0.77706	2.26757

4o_conformer-003

C	0.69349	0.48485	-0.31677
C	0.31957	1.82391	-0.33587
C	-1.08230	1.87754	-0.55709
C	-1.54824	0.57705	-0.67130
N	-0.45944	-0.25176	-0.52565
H	-0.50604	-1.26033	-0.51332
H	0.99393	2.66292	-0.22653
H	-1.69493	2.76606	-0.63183
C	1.98256	-0.14410	-0.14329
C	2.37135	-1.43558	-0.43022
S	3.32915	0.75899	0.53377
C	3.73891	-1.69958	-0.11797
H	1.70945	-2.17113	-0.87448
C	4.39044	-0.61542	0.40011
H	4.21732	-2.65846	-0.28478
H	5.42281	-0.53031	0.70990
C	-2.92813	0.03659	-0.86565
H	-2.94279	-0.65649	-1.71869
C	-3.50785	-0.69020	0.37180
H	-2.82288	-1.51162	0.62914
C	-4.87284	-1.29858	0.03407
C	-3.60180	0.24809	1.57883
H	-3.98292	-0.28253	2.45867
H	-2.62501	0.67360	1.83064
H	-4.28476	1.08106	1.36637
H	-3.58950	0.87098	-1.12926

H	-5.59060	-0.51317	-0.23575
H	-4.80249	-1.99343	-0.81103
H	-5.28283	-1.84605	0.89034

4o_conformer-004

C	-0.69349	0.48485	-0.31677
C	-0.31957	1.82391	-0.33587
C	1.08230	1.87754	-0.55709
C	1.54824	0.57704	-0.67130
N	0.45944	-0.25176	-0.52565
H	0.50604	-1.26033	-0.51332
H	-0.99393	2.66292	-0.22654
H	1.69493	2.76606	-0.63184
C	-1.98256	-0.14411	-0.14329
C	-2.37135	-1.43558	-0.43022
S	-3.32915	0.75899	0.53377
C	-3.73891	-1.69958	-0.11797
H	-1.70946	-2.17113	-0.87448
C	-4.39044	-0.61542	0.40011
H	-4.21732	-2.65846	-0.28478
H	-5.42281	-0.53031	0.70990
C	2.92813	0.03659	-0.86565
H	3.58950	0.87098	-1.12926
C	3.50785	-0.69020	0.37181
H	2.82288	-1.51162	0.62914
C	3.60180	0.24809	1.57883
C	4.87284	-1.29858	0.03407
H	5.59060	-0.51318	-0.23576
H	5.28283	-1.84605	0.89034
H	4.80248	-1.99343	-0.81103
H	2.94279	-0.65649	-1.71869
H	4.28477	1.08106	1.36637
H	2.62501	0.67361	1.83064
H	3.98292	-0.28252	2.45867

4o_conformer-005

C	0.74411	0.81413	-0.08980
C	0.27151	2.07884	0.24184
C	-1.12824	2.09254	0.00069
C	-1.49555	0.84226	-0.47133
N	-0.35074	0.07876	-0.51135
H	-0.30402	-0.85731	-0.88914
H	0.87454	2.88516	0.63665
H	-1.80704	2.91927	0.16295

C	2.08268	0.27184	-0.03444
C	3.27959	0.95473	-0.01514
S	2.37273	-1.46114	0.06489
C	4.41953	0.10163	0.06986
H	3.33605	2.03540	-0.08086
C	4.09748	-1.22688	0.10716
H	5.44114	0.46532	0.08698
H	4.75551	-2.08350	0.15266
C	-2.82722	0.28263	-0.85497
H	-2.77544	-0.15284	-1.86303
C	-3.36995	-0.79034	0.11947
H	-2.62562	-1.59829	0.17435
C	-4.67579	-1.38166	-0.42135
C	-3.56061	-0.22107	1.52865
H	-4.30427	0.58661	1.52005
H	-3.91364	-0.99515	2.21937
H	-2.62572	0.19029	1.92279
H	-3.54506	1.11017	-0.90917
H	-4.53498	-1.81639	-1.41793
H	-5.05646	-2.16748	0.24093
H	-5.44888	-0.60634	-0.49935

4o_conformer-006

C	-0.74411	0.81415	-0.08978
C	-0.27153	2.07886	0.24189
C	1.12822	2.09258	0.00076
C	1.49555	0.84232	-0.47128
N	0.35075	0.07880	-0.51132
H	0.30405	-0.85726	-0.88913
H	-0.87458	2.88516	0.63671
H	1.80701	2.91932	0.16304
C	-2.08267	0.27184	-0.03444
C	-3.27960	0.95471	-0.01516
S	-2.37270	-1.46114	0.06488
C	-4.41952	0.10159	0.06982
H	-3.33607	2.03539	-0.08089
C	-4.09746	-1.22691	0.10712
H	-5.44114	0.46528	0.08692
H	-4.75548	-2.08353	0.15261
C	2.82723	0.28272	-0.85493
H	3.54508	1.11026	-0.90902
C	3.36993	-0.79036	0.11941
H	2.62561	-1.59833	0.17416
C	3.56053	-0.22125	1.52867
C	4.67580	-1.38160	-0.42142

H	4.53505	-1.81622	-1.41806
H	5.05645	-2.16750	0.24079
H	5.44889	-0.60627	-0.49929
H	2.77549	-0.15265	-1.86304
H	2.62561	0.19004	1.92282
H	3.91354	-0.99541	2.21931
H	4.30417	0.58645	1.52019

4o_conformer-007

C	-0.72470	0.74989	-0.21254
C	-0.24361	2.05094	-0.30880
C	1.14853	1.97625	-0.57948
C	1.50198	0.63780	-0.65032
N	0.35434	-0.08968	-0.43006
H	0.32156	-1.09755	-0.36490
H	-0.84504	2.94543	-0.22026
H	1.82873	2.80621	-0.71762
C	-2.06120	0.26461	0.04630
C	-3.11401	0.93739	0.62817
S	-2.56095	-1.35859	-0.41458
C	-4.30774	0.16145	0.71216
H	-3.02381	1.95127	1.00136
C	-4.16747	-1.10088	0.20535
H	-5.23137	0.52675	1.14773
H	-4.89662	-1.89764	0.15675
C	2.82493	-0.02310	-0.86699
H	3.54049	0.74137	-1.19369
C	3.39312	-0.73863	0.38240
H	2.65525	-1.48854	0.70379
C	3.61086	0.24220	1.53857
C	4.69001	-1.47207	0.02519
H	5.45729	-0.76155	-0.30837
H	5.08863	-2.01195	0.89160
H	4.52996	-2.19650	-0.78201
H	2.75030	-0.75269	-1.68576
H	4.34944	1.00575	1.26125
H	2.68246	0.75750	1.80529
H	3.98334	-0.27775	2.42841

4o_conformer-008

C	0.72470	0.74989	-0.21253
C	0.24361	2.05095	-0.30879
C	-1.14853	1.97626	-0.57948
C	-1.50198	0.63781	-0.65032

N	-0.35434	-0.08968	-0.43006
H	-0.32156	-1.09755	-0.36490
H	0.84504	2.94543	-0.22025
H	-1.82873	2.80622	-0.71761
C	2.06120	0.26461	0.04630
C	3.11401	0.93739	0.62817
S	2.56095	-1.35859	-0.41458
C	4.30773	0.16145	0.71216
H	3.02381	1.95127	1.00136
C	4.16747	-1.10088	0.20534
H	5.23137	0.52675	1.14773
H	4.89661	-1.89764	0.15675
C	-2.82493	-0.02310	-0.86699
H	-2.75031	-0.75268	-1.68576
C	-3.39312	-0.73863	0.38240
H	-2.65525	-1.48855	0.70378
C	-4.69001	-1.47207	0.02519
C	-3.61085	0.24219	1.53857
H	-2.68246	0.75750	1.80529
H	-4.34944	1.00575	1.26126
H	-3.98334	-0.27776	2.42841
H	-3.54050	0.74137	-1.19368
H	-5.45729	-0.76154	-0.30836
H	-4.52997	-2.19650	-0.78201
H	-5.08862	-2.01195	0.89159

4o_conformer-009

C	0.68432	-0.55059	0.04225
C	0.31050	-1.81728	-0.39425
C	-1.08378	-1.95875	-0.16850
C	-1.54702	-0.78163	0.40071
N	-0.46378	0.05812	0.51691
H	-0.49631	0.97250	0.94402
H	0.97521	-2.54705	-0.83739
H	-1.69400	-2.82211	-0.39840
C	1.96376	0.11996	0.04890
C	2.24609	1.45985	0.21095
S	3.45029	-0.79234	-0.16326
C	3.64131	1.75663	0.15909
H	1.48015	2.21708	0.33927
C	4.41834	0.65096	-0.04573
H	4.04505	2.75813	0.25997
H	5.49365	0.58657	-0.13816
C	-2.92622	-0.36086	0.79757
H	-2.87133	0.36289	1.62317

C	-3.76194	0.25795	-0.34979
H	-3.80980	-0.48877	-1.15489
C	-3.11018	1.52587	-0.91131
C	-5.18832	0.54014	0.13454
H	-5.18354	1.27857	0.94674
H	-5.80748	0.94008	-0.67650
H	-5.66938	-0.36998	0.51162
H	-3.46093	-1.23561	1.18595
H	-3.01767	2.29262	-0.13048
H	-2.10884	1.32425	-1.30507
H	-3.71414	1.94784	-1.72259

4o_conformer-010

C	-0.68431	-0.55070	0.04227
C	-0.31057	-1.81746	-0.39409
C	1.08372	-1.95896	-0.16839
C	1.54704	-0.78179	0.40066
N	0.46384	0.05802	0.51681
H	0.49644	0.97247	0.94374
H	-0.97533	-2.54726	-0.83710
H	1.69389	-2.82238	-0.39819
C	-1.96371	0.11992	0.04892
C	-2.24594	1.45982	0.21108
S	-3.45029	-0.79226	-0.16339
C	-3.64114	1.75671	0.15919
H	-1.47995	2.21697	0.33952
C	-4.41824	0.65110	-0.04576
H	-4.04482	2.75822	0.26014
H	-5.49356	0.58681	-0.13824
C	2.92627	-0.36105	0.79742
H	3.46106	-1.23589	1.18548
C	3.76182	0.25810	-0.34988
H	3.80960	-0.48839	-1.15520
C	5.18825	0.54021	0.13435
C	3.10995	1.52616	-0.91097
H	3.71384	1.94841	-1.72216
H	2.10860	1.32462	-1.30474
H	3.01745	2.29267	-0.12989
H	2.87148	0.36248	1.62323
H	5.18354	1.27843	0.94675
H	5.66939	-0.36999	0.51113
H	5.80730	0.94037	-0.67666

4o_conformer-011

C	-0.68351	0.54642	-0.22615
C	-0.28663	1.85890	0.00776
C	1.11489	1.93151	-0.20535
C	1.55940	0.66735	-0.56412
N	0.45686	-0.15483	-0.57504
H	0.49009	-1.14949	-0.74673
H	-0.94767	2.67382	0.27181
H	1.74010	2.81002	-0.11597
C	-1.98276	-0.08303	-0.17569
C	-2.39210	-1.28577	-0.71146
S	-3.31394	0.68803	0.67314
C	-3.76328	-1.58576	-0.45189
H	-1.74163	-1.92713	-1.29626
C	-4.39712	-0.61624	0.27374
H	-4.25703	-2.48494	-0.80385
H	-5.42775	-0.57831	0.59839
C	2.93728	0.15451	-0.83973
H	3.49132	0.91468	-1.40340
C	3.74804	-0.20553	0.42890
H	3.80485	0.70248	1.04564
C	5.17229	-0.61845	0.04240
C	3.06019	-1.29969	1.25176
H	2.95806	-2.22121	0.66309
H	3.64422	-1.53963	2.14748
H	2.05959	-0.99264	1.57309
H	2.88011	-0.73257	-1.48603
H	5.15893	-1.52221	-0.58051
H	5.67718	0.17228	-0.52487
H	5.77450	-0.83512	0.93205

4o_conformer-012

C	0.68351	0.54648	-0.22600
C	0.28671	1.85896	0.00806
C	-1.11481	1.93168	-0.20502
C	-1.55940	0.66759	-0.56392
N	-0.45691	-0.15466	-0.57494
H	-0.49020	-1.14928	-0.74681
H	0.94780	2.67382	0.27222
H	-1.73996	2.81022	-0.11553
C	1.98271	-0.08305	-0.17560
C	2.39187	-1.28596	-0.71116
S	3.31411	0.68811	0.67280
C	3.76307	-1.58599	-0.45177
H	1.74125	-1.92743	-1.29567
C	4.39712	-0.61635	0.27353

H	4.25669	-2.48530	-0.80358
H	5.42780	-0.57843	0.59801
C	-2.93731	0.15488	-0.83961
H	-2.88022	-0.73194	-1.48630
C	-3.74799	-0.20568	0.42892
H	-3.80468	0.70206	1.04608
C	-3.06016	-1.30027	1.25123
C	-5.17230	-0.61831	0.04236
H	-5.15906	-1.52178	-0.58098
H	-5.67718	0.17272	-0.52450
H	-5.77445	-0.83536	0.93196
H	-3.49138	0.91530	-1.40292
H	-2.05951	-0.99346	1.57262
H	-3.64415	-1.54057	2.14688
H	-2.95817	-2.22153	0.66213

4o_conformer-013

C	-0.68434	0.55086	-0.04213
C	-0.31066	1.81761	0.39429
C	1.08364	1.95915	0.16867
C	1.54703	0.78201	-0.40038
N	0.46385	-0.05782	-0.51661
H	0.49645	-0.97218	-0.94375
H	-0.97544	2.54737	0.83734
H	1.69379	2.82257	0.39855
C	-1.96368	-0.11986	-0.04881
C	-2.24571	-1.45983	-0.21073
S	-3.45040	0.79219	0.16304
C	-3.64088	-1.75688	-0.15902
H	-1.47958	-2.21690	-0.33883
C	-4.41815	-0.65133	0.04557
H	-4.04441	-2.75847	-0.25982
H	-5.49349	-0.58714	0.13786
C	2.92626	0.36136	-0.79722
H	3.46107	1.23635	-1.18491
C	3.76175	-0.25829	0.34985
H	3.80951	0.48786	1.15549
C	5.18821	-0.54020	-0.13445
C	3.10989	-1.52660	0.91038
H	3.71376	-1.94917	1.72142
H	2.10852	-1.32527	1.30418
H	3.01746	-2.29279	0.12898
H	2.87151	-0.36184	-1.62332
H	5.18352	-1.27805	-0.94718
H	5.66937	0.37017	-0.51080

H	5.80722	-0.94073	0.67641

4o_conformer-014

C	0.68438	0.55080	-0.04235
C	0.31065	1.81760	0.39387
C	-1.08366	1.95904	0.16827
C	-1.54701	0.78178	-0.40057
N	-0.46379	-0.05801	-0.51671
H	-0.49638	-0.97247	-0.94362
H	0.97541	2.54748	0.83676
H	-1.69384	2.82248	0.39802
C	1.96375	-0.11985	-0.04896
C	2.24589	-1.45976	-0.21124
S	3.45038	0.79219	0.16356
C	3.64106	-1.75676	-0.15929
H	1.47985	-2.21682	-0.33983
C	4.41823	-0.65123	0.04584
H	4.04467	-2.75829	-0.26032
H	5.49355	-0.58702	0.13840
C	-2.92623	0.36101	-0.79733
H	-2.87147	-0.36252	-1.62315
C	-3.76182	-0.25815	0.34994
H	-3.80946	0.48825	1.15535
C	-3.11019	-1.52642	0.91085
C	-5.18832	-0.53997	-0.13428
H	-5.66934	0.37036	-0.51090
H	-5.18375	-1.27807	-0.94679
H	-5.80740	-0.94017	0.67670
H	-3.46097	1.23587	-1.18539
H	-3.71411	-1.94861	1.72204
H	-3.01792	-2.29286	0.12969
H	-2.10876	-1.32516	1.30456

4o_conformer-015

C	0.68356	-0.54623	-0.22653
C	0.28646	-1.85873	0.00686
C	-1.11507	-1.93102	-0.20633
C	-1.55936	-0.66665	-0.56463
N	-0.45667	0.15535	-0.57520
H	-0.48974	1.15013	-0.74626
H	0.94738	-2.67388	0.27053
H	-1.74041	-2.80947	-0.11734
C	1.98292	0.08295	-0.17589
C	2.39278	1.28522	-0.71234

S	3.31351	-0.68778	0.67416
C	3.76391	1.58508	-0.45234
H	1.74273	1.92620	-1.29801
C	4.39720	0.61594	0.27429
H	4.25805	2.48388	-0.80474
H	5.42767	0.57799	0.59940
C	-2.93715	-0.15342	-0.84001
H	-3.49109	-0.91287	-1.40477
C	-3.74827	0.20510	0.42882
H	-3.80538	-0.70370	1.04438
C	-5.17234	0.61869	0.04240
C	-3.06054	1.29810	1.25330
H	-3.64477	1.53690	2.14919
H	-2.06004	0.99056	1.57445
H	-2.95819	2.22039	0.66587
H	-2.87973	0.73443	-1.48522
H	-5.15866	1.52323	-0.57936
H	-5.67715	-0.17126	-0.52603
H	-5.77480	0.83432	0.93213

4o_conformer-016

C	-0.68354	-0.54634	-0.22641
C	-0.28653	-1.85885	0.00708
C	1.11500	-1.93125	-0.20610
C	1.55938	-0.66693	-0.56449
N	0.45674	0.15514	-0.57512
H	0.48986	1.14989	-0.74632
H	-0.94750	-2.67394	0.27081
H	1.74028	-2.80974	-0.11705
C	-1.98286	0.08293	-0.17586
C	-2.39258	1.28524	-0.71230
S	-3.31358	-0.68772	0.67407
C	-3.76371	1.58521	-0.45241
H	-1.74243	1.92620	-1.29790
C	-4.39713	0.61609	0.27415
H	-4.25775	2.48405	-0.80481
H	-5.42763	0.57822	0.59919
C	2.93720	-0.15382	-0.83988
H	2.87990	0.73373	-1.48552
C	3.74816	0.20530	0.42889
H	3.80517	-0.70321	1.04489
C	3.06034	1.29871	1.25276
C	5.17229	0.61868	0.04246
H	5.15870	1.52293	-0.57973
H	5.77463	0.83473	0.93217

H	5.67717	-0.17154	-0.52552
H	3.49121	-0.91355	-1.40419
H	2.95808	2.22071	0.66487
H	2.05980	0.99133	1.57396
H	3.64447	1.53793	2.14860

4o_conformer-017

C	0.72865	0.84606	-0.00375
C	0.25001	2.06347	-0.47636
C	-1.15000	2.09657	-0.24316
C	-1.51313	0.90300	0.36299
N	-0.36387	0.15792	0.49413
H	-0.31916	-0.73698	0.96119
H	0.85142	2.82640	-0.95179
H	-1.83065	2.89900	-0.49553
C	2.06992	0.30805	0.00871
C	3.26432	0.99038	-0.07421
S	2.36713	-1.42459	0.09462
C	4.40810	0.13815	-0.06020
H	3.31621	2.07222	-0.12252
C	4.09140	-1.18830	0.04070
H	5.42847	0.50265	-0.10796
H	4.75302	-2.04185	0.09145
C	-2.85100	0.38305	0.78277
H	-3.45899	1.22548	1.13274
C	-3.62978	-0.35733	-0.33214
H	-3.73950	0.34381	-1.17146
C	-5.02782	-0.73599	0.16875
C	-2.87253	-1.59098	-0.83484
H	-3.43797	-2.10084	-1.62311
H	-1.89148	-1.32468	-1.24096
H	-2.71615	-2.30899	-0.01860
H	-2.73500	-0.29511	1.64022
H	-4.96152	-1.43154	1.01537
H	-5.58493	0.14706	0.50310
H	-5.60961	-1.22545	-0.62058

4o_conformer-018

C	-0.72865	0.84607	-0.00375
C	-0.25001	2.06348	-0.47633
C	1.15000	2.09658	-0.24313
C	1.51313	0.90300	0.36299
N	0.36387	0.15792	0.49413
H	0.31916	-0.73700	0.96115

H	-0.85142	2.82642	-0.95174
H	1.83065	2.89902	-0.49549
C	-2.06991	0.30805	0.00871
C	-3.26432	0.99038	-0.07425
S	-2.36713	-1.42459	0.09466
C	-4.40810	0.13815	-0.06024
H	-3.31621	2.07221	-0.12259
C	-4.09140	-1.18830	0.04069
H	-5.42847	0.50265	-0.10803
H	-4.75302	-2.04185	0.09145
C	2.85100	0.38305	0.78277
H	2.73502	-0.29510	1.64022
C	3.62977	-0.35734	-0.33215
H	3.73948	0.34380	-1.17148
C	2.87253	-1.59099	-0.83484
C	5.02782	-0.73599	0.16872
H	5.58493	0.14706	0.50306
H	5.60961	-1.22546	-0.62061
H	4.96154	-1.43153	1.01535
H	3.45900	1.22549	1.13272
H	1.89146	-1.32470	-1.24094
H	3.43795	-2.10085	-1.62311
H	2.71616	-2.30900	-0.01859

4o_conformer-019

C	0.71143	0.78705	-0.08358
C	0.21527	2.07993	0.04842
C	-1.17575	2.03911	-0.23135
C	-1.51515	0.72832	-0.53262
N	-0.35920	-0.01243	-0.44351
H	-0.32039	-1.01680	-0.54809
H	0.80805	2.95266	0.28669
H	-1.86295	2.87494	-0.22617
C	2.05422	0.27913	0.08111
C	3.10244	0.84891	0.77115
S	2.56938	-1.22952	-0.66445
C	4.30487	0.08404	0.71172
H	3.00311	1.77851	1.32013
C	4.17593	-1.06878	-0.01234
H	5.22647	0.37606	1.20334
H	4.91336	-1.83567	-0.20443
C	-2.83889	0.10405	-0.84108
H	-2.68941	-0.80253	-1.44426
C	-3.67888	-0.26154	0.40696
H	-3.82724	0.66446	0.98020

C	-2.95443	-1.26883	1.30590
C	-5.05160	-0.79148	-0.02109
H	-4.94647	-1.71589	-0.60364
H	-5.67653	-1.01567	0.85085
H	-5.58492	-0.06249	-0.64246
H	-3.41979	0.79747	-1.46094
H	-2.76098	-2.20401	0.76346
H	-1.99384	-0.87765	1.65659
H	-3.56085	-1.51351	2.18531

4o_conformer-020

C	-0.71143	0.78706	-0.08357
C	-0.21527	2.07994	0.04844
C	1.17575	2.03912	-0.23133
C	1.51515	0.72834	-0.53261
N	0.35920	-0.01242	-0.44350
H	0.32039	-1.01678	-0.54810
H	-0.80806	2.95266	0.28671
H	1.86295	2.87495	-0.22614
C	-2.05422	0.27913	0.08111
C	-3.10245	0.84892	0.77113
S	-2.56936	-1.22953	-0.66444
C	-4.30488	0.08404	0.71169
H	-3.00314	1.77852	1.32009
C	-4.17592	-1.06879	-0.01234
H	-5.22648	0.37606	1.20330
H	-4.91335	-1.83569	-0.20444
C	2.83890	0.10407	-0.84108
H	3.41979	0.79751	-1.46090
C	3.67887	-0.26155	0.40696
H	3.82723	0.66443	0.98024
C	5.05160	-0.79147	-0.02109
C	2.95442	-1.26888	1.30586
H	3.56083	-1.51358	2.18527
H	1.99382	-0.87771	1.65656
H	2.76098	-2.20403	0.76340
H	2.68942	-0.80248	-1.44428
H	4.94647	-1.71587	-0.60368
H	5.58492	-0.06246	-0.64243
H	5.67652	-1.01569	0.85085

4o_conformer-021

C	0.72864	0.84606	0.00365
C	0.25000	2.06357	0.47600

C	-1.14999	2.09663	0.24278
C	-1.51312	0.90294	-0.36313
N	-0.36386	0.15785	-0.49415
H	-0.31920	-0.73730	-0.96074
H	0.85141	2.82661	0.95125
H	-1.83064	2.89911	0.49498
C	2.06990	0.30805	-0.00867
C	3.26429	0.99035	0.07475
S	2.36714	-1.42456	-0.09510
C	4.40807	0.13813	0.06074
H	3.31618	2.07216	0.12347
C	4.09140	-1.18829	-0.04065
H	5.42842	0.50262	0.10889
H	4.75303	-2.04183	-0.09150
C	-2.85101	0.38293	-0.78279
H	-2.73508	-0.29534	-1.64016
C	-3.62976	-0.35729	0.33226
H	-3.73948	0.34398	1.17147
C	-2.87249	-1.59084	0.83516
C	-5.02781	-0.73605	-0.16855
H	-5.60956	-1.22542	0.62087
H	-5.58494	0.14694	-0.50301
H	-4.96151	-1.43172	-1.01507
H	-3.45901	1.22533	-1.13284
H	-2.71614	-2.30901	0.01904
H	-1.89143	-1.32447	1.24120
H	-3.43791	-2.10055	1.62353

4o_conformer-022

C	-0.72861	0.84616	0.00368
C	-0.25005	2.06373	0.47596
C	1.14994	2.09687	0.24277
C	1.51316	0.90316	-0.36305
N	0.36395	0.15799	-0.49407
H	0.31938	-0.73723	-0.96051
H	-0.85153	2.82679	0.95112
H	1.83054	2.89941	0.49491
C	-2.06983	0.30806	-0.00866
C	-3.26427	0.99029	0.07470
S	-2.36695	-1.42457	-0.09505
C	-4.40799	0.13800	0.06068
H	-3.31623	2.07210	0.12338
C	-4.09123	-1.18841	-0.04066
H	-5.42837	0.50241	0.10878
H	-4.75280	-2.04199	-0.09150

C	2.85110	0.38320	-0.78260
H	3.45922	1.22571	-1.13217
C	3.62953	-0.35751	0.33233
H	3.73907	0.34341	1.17186
C	5.02768	-0.73615	-0.16828
C	2.87209	-1.59124	0.83454
H	2.71580	-2.30902	0.01807
H	1.89098	-1.32499	1.24056
H	3.43733	-2.10136	1.62277
H	2.73533	-0.29472	-1.64027
H	5.60923	-1.22585	0.62108
H	4.96155	-1.43148	-1.01510
H	5.58494	0.14694	-0.50226

4o_conformer-023

C	-0.71152	0.78722	-0.08336
C	-0.21553	2.08015	0.04883
C	1.17552	2.03952	-0.23074
C	1.51513	0.72879	-0.53208
N	0.35924	-0.01210	-0.44320
H	0.32055	-1.01646	-0.54794
H	-0.80843	2.95279	0.28711
H	1.86262	2.87543	-0.22542
C	-2.05428	0.27915	0.08113
C	-3.10273	0.84895	0.77079
S	-2.56908	-1.22971	-0.66427
C	-4.30506	0.08393	0.71121
H	-3.00366	1.77867	1.31960
C	-4.17581	-1.06903	-0.01257
H	-5.22681	0.37594	1.20255
H	-4.91310	-1.83605	-0.20469
C	2.83897	0.10480	-0.84077
H	3.41997	0.79878	-1.45990
C	3.67876	-0.26189	0.40710
H	3.82663	0.66350	0.98145
C	5.05179	-0.79083	-0.02121
C	2.95447	-1.27053	1.30467
H	2.76165	-2.20517	0.76111
H	3.56071	-1.51592	2.18400
H	1.99359	-0.88019	1.65550
H	2.68966	-0.80124	-1.44477
H	4.94715	-1.71458	-0.60490
H	5.58503	-0.06091	-0.64155
H	5.67655	-1.01585	0.85064

4o_conformer-024

C	0.71148	0.78721	-0.08345
C	0.21543	2.08012	0.04872
C	-1.17562	2.03943	-0.23091
C	-1.51515	0.72870	-0.53228
N	-0.35924	-0.01215	-0.44336
H	-0.32055	-1.01652	-0.54797
H	0.80830	2.95278	0.28701
H	-1.86276	2.87530	-0.22559
C	2.05424	0.27917	0.08109
C	3.10265	0.84895	0.77084
S	2.56908	-1.22968	-0.66429
C	4.30498	0.08392	0.71133
H	3.00353	1.77865	1.31968
C	4.17577	-1.06902	-0.01248
H	5.22670	0.37592	1.20274
H	4.91308	-1.83604	-0.20457
C	-2.83894	0.10458	-0.84086
H	-2.68953	-0.80162	-1.44461
C	-3.67876	-0.26179	0.40705
H	-3.82692	0.66381	0.98099
C	-2.95431	-1.26985	1.30512
C	-5.05162	-0.79124	-0.02117
H	-4.94669	-1.71522	-0.60445
H	-5.67642	-1.01604	0.85071
H	-5.58497	-0.06172	-0.64190
H	-3.41991	0.79839	-1.46021
H	-2.76111	-2.20465	0.76196
H	-1.99361	-0.87908	1.65595
H	-3.56062	-1.51509	2.18445

4o_conformer-025

C	-0.56301	-0.52177	-0.13550
C	-0.11479	-1.78954	0.21938
C	1.26907	-1.86042	-0.08918
C	1.65270	-0.64028	-0.62650
N	0.53060	0.15670	-0.64289
H	0.49891	1.08734	-1.03307
H	-0.72276	-2.56549	0.66544
H	1.92626	-2.70568	0.06512
C	-1.86642	0.09363	-0.03892
C	-2.20968	1.42700	-0.11361
S	-3.30179	-0.89048	0.20184
C	-3.61055	1.66141	0.02769

H	-1.48233	2.22154	-0.24069
C	-4.33085	0.51471	0.21350
H	-4.05848	2.64877	0.00222
H	-5.39613	0.40030	0.35875
C	2.98524	-0.14639	-1.09505
H	2.85045	0.49445	-1.97688
C	3.80716	0.63639	-0.03649
H	4.74292	0.92284	-0.53679
C	4.16158	-0.24415	1.16605
C	3.09872	1.92126	0.40748
H	2.84581	2.55587	-0.45094
H	2.17070	1.69439	0.94493
H	3.73804	2.50479	1.07953
H	3.57429	-1.01052	-1.42299
H	3.25565	-0.57007	1.68974
H	4.70815	-1.14199	0.85363
H	4.78791	0.30320	1.87967

4o_conformer-026

C	0.56301	-0.52177	-0.13552
C	0.11479	-1.78955	0.21934
C	-1.26907	-1.86044	-0.08922
C	-1.65270	-0.64027	-0.62651
N	-0.53061	0.15671	-0.64288
H	-0.49892	1.08736	-1.03302
H	0.72278	-2.56551	0.66536
H	-1.92625	-2.70569	0.06506
C	1.86642	0.09363	-0.03893
C	2.20969	1.42699	-0.11365
S	3.30179	-0.89048	0.20188
C	3.61055	1.66141	0.02766
H	1.48233	2.22154	-0.24078
C	4.33085	0.51471	0.21352
H	4.05848	2.64876	0.00217
H	5.39613	0.40031	0.35878
C	-2.98524	-0.14638	-1.09504
H	-3.57430	-1.01051	-1.42299
C	-3.80716	0.63639	-0.03647
H	-4.74293	0.92284	-0.53677
C	-3.09872	1.92126	0.40750
C	-4.16157	-0.24415	1.16606
H	-4.78791	0.30319	1.87969
H	-4.70814	-1.14199	0.85364
H	-3.25565	-0.57007	1.68975
H	-2.85046	0.49446	-1.97687

H	-2.84581	2.55587	-0.45093
H	-3.73804	2.50479	1.07955
H	-2.17070	1.69440	0.94495

4o_conformer-027

C	0.68446	-0.55061	-0.04203
C	0.31066	-1.81732	0.39443
C	-1.08364	-1.95875	0.16877
C	-1.54692	-0.78159	-0.40033
N	-0.46367	0.05816	-0.51654
H	-0.49612	0.97247	-0.94381
H	0.97538	-2.54711	0.83753
H	-1.69387	-2.82211	0.39866
C	1.96389	0.11994	-0.04877
C	2.24612	1.45989	-0.21055
S	3.45049	-0.79235	0.16287
C	3.64133	1.75672	-0.15890
H	1.48009	2.21708	-0.33853
C	4.41845	0.65103	0.04552
H	4.04502	2.75826	-0.25961
H	5.49378	0.58667	0.13773
C	-2.92615	-0.36104	-0.79730
H	-3.46070	-1.23603	-1.18538
C	-3.76212	0.25804	0.34972
H	-3.80983	-0.48831	1.15517
C	-5.18855	0.53955	-0.13488
C	-3.11088	1.52645	0.91073
H	-3.71526	1.94875	1.72153
H	-2.10966	1.32530	1.30503
H	-3.01825	2.29276	0.12947
H	-2.87138	0.36241	-1.62318
H	-5.18393	1.27759	-0.94744
H	-5.66929	-0.37090	-0.51157
H	-5.80792	0.93968	0.67591

4o_conformer-028

C	0.59014	0.82161	-0.08466
C	0.02184	2.02224	0.32693
C	-1.36076	1.97614	0.00783
C	-1.62529	0.75206	-0.58881
N	-0.43253	0.06560	-0.63026
H	-0.31002	-0.83224	-1.07708
H	0.55214	2.82529	0.82050
H	-2.09645	2.74637	0.19731

C	1.95524	0.35447	-0.00022
C	3.10515	1.10052	0.14189
S	2.34605	-1.36134	-0.02665
C	4.28972	0.30984	0.22048
H	3.09812	2.18433	0.16800
C	4.04866	-1.03326	0.13125
H	5.28478	0.72857	0.32435
H	4.75503	-1.85166	0.14311
C	-2.90347	0.15839	-1.09174
H	-2.70793	-0.41622	-2.00726
C	-3.64707	-0.75692	-0.08255
H	-4.55411	-1.09832	-0.60098
C	-4.07744	0.01650	1.16795
C	-2.82339	-1.99550	0.28823
H	-2.52367	-2.55881	-0.60414
H	-1.91413	-1.71627	0.83303
H	-3.40244	-2.66841	0.93075
H	-3.57269	0.97901	-1.37451
H	-3.20384	0.39105	1.71358
H	-4.70254	0.87863	0.90628
H	-4.65143	-0.62552	1.84580

4o_conformer-029

C	-0.59014	0.82161	-0.08466
C	-0.02186	2.02225	0.32691
C	1.36075	1.97615	0.00782
C	1.62529	0.75207	-0.58880
N	0.43253	0.06560	-0.63024
H	0.31003	-0.83225	-1.07705
H	-0.55216	2.82531	0.82047
H	2.09643	2.74640	0.19729
C	-1.95524	0.35447	-0.00022
C	-3.10515	1.10052	0.14191
S	-2.34605	-1.36135	-0.02666
C	-4.28972	0.30983	0.22050
H	-3.09812	2.18432	0.16802
C	-4.04866	-1.03327	0.13125
H	-5.28478	0.72857	0.32437
H	-4.75503	-1.85167	0.14310
C	2.90348	0.15840	-1.09173
H	3.57269	0.97904	-1.37448
C	3.64707	-0.75692	-0.08254
H	4.55412	-1.09831	-0.60099
C	2.82340	-1.99551	0.28820
C	4.07744	0.01648	1.16796

H	4.70254	0.87862	0.90631
H	3.20384	0.39102	1.71360
H	4.65143	-0.62555	1.84580
H	2.70795	-0.41619	-2.00725
H	2.52370	-2.55881	-0.60418
H	3.40245	-2.66842	0.93073
H	1.91412	-1.71629	0.83300

4o_conformer-030

C	0.56277	-0.49354	-0.31581
C	0.09282	-1.79604	-0.18496
C	-1.29921	-1.78282	-0.46181
C	-1.66663	-0.47769	-0.75631
N	-0.52502	0.28585	-0.66642
H	-0.49765	1.28738	-0.79136
H	0.69965	-2.65822	0.05812
H	-1.97033	-2.63116	-0.45653
C	1.88634	0.06457	-0.16306
C	2.36854	1.29290	-0.56307
S	3.15035	-0.84971	0.64548
C	3.74151	1.49938	-0.23193
H	1.76913	2.02134	-1.09833
C	4.30439	0.43410	0.41344
H	4.28723	2.40426	-0.47595
H	5.31948	0.31543	0.76631
C	-3.00077	0.12058	-1.07544
H	-3.61221	-0.64568	-1.56592
C	-3.78708	0.66791	0.14585
H	-4.72540	1.07198	-0.25948
C	-3.04369	1.81281	0.84300
C	-4.13917	-0.44681	1.13613
H	-4.70740	-1.24737	0.64758
H	-3.23171	-0.89187	1.55982
H	-4.74406	-0.05736	1.96292
H	-2.87315	0.93325	-1.80293
H	-2.10703	1.46022	1.29038
H	-2.80118	2.61972	0.14049
H	-3.65466	2.24035	1.64600

4o_conformer-031

C	-0.56277	-0.49354	-0.31583
C	-0.09281	-1.79604	-0.18501
C	1.29921	-1.78282	-0.46186
C	1.66663	-0.47768	-0.75633

N	0.52502	0.28586	-0.66641
H	0.49765	1.28739	-0.79131
H	-0.69965	-2.65823	0.05803
H	1.97034	-2.63116	-0.45660
C	-1.88634	0.06456	-0.16308
C	-2.36857	1.29286	-0.56314
S	-3.15032	-0.84969	0.64555
C	-3.74153	1.49935	-0.23197
H	-1.76918	2.02128	-1.09847
C	-4.30438	0.43410	0.41347
H	-4.28726	2.40420	-0.47603
H	-5.31946	0.31543	0.76637
C	3.00077	0.12061	-1.07543
H	2.87315	0.93328	-1.80291
C	3.78707	0.66791	0.14587
H	4.72539	1.07199	-0.25944
C	4.13916	-0.44682	1.13613
C	3.04368	1.81280	0.84303
H	2.80117	2.61972	0.14053
H	2.10702	1.46021	1.29040
H	3.65464	2.24034	1.64604
H	3.61221	-0.64565	-1.56592
H	3.23170	-0.89188	1.55982
H	4.70740	-1.24737	0.64758
H	4.74404	-0.05738	1.96294

4o_conformer-032

C	0.57122	0.74752	-0.17604
C	-0.01124	2.00935	-0.11834
C	-1.38184	1.86727	-0.45888
C	-1.62428	0.52693	-0.72232
N	-0.42801	-0.13289	-0.55172
H	-0.31775	-1.13460	-0.62338
H	0.51223	2.92678	0.11403
H	-2.12060	2.65533	-0.51877
C	1.93349	0.33520	0.07441
C	2.90949	1.00537	0.78016
S	2.57683	-1.17309	-0.56442
C	4.15842	0.31767	0.81662
H	2.72621	1.95333	1.27323
C	4.13668	-0.87607	0.14996
H	5.03517	0.69306	1.33300
H	4.92871	-1.60248	0.03222
C	-2.88566	-0.19292	-1.08302
H	-2.65592	-1.00528	-1.78546

C	-3.67503	-0.78321	0.11605
H	-4.55710	-1.27377	-0.31902
C	-4.16283	0.31541	1.06588
C	-2.86823	-1.84730	0.86862
H	-3.47656	-2.31460	1.65119
H	-1.98827	-1.40671	1.35172
H	-2.52346	-2.64047	0.19362
H	-3.53885	0.50706	-1.61680
H	-4.76619	-0.10898	1.87643
H	-4.77660	1.05422	0.53684
H	-3.31538	0.84551	1.51527

4o_conformer-033

C	-0.57130	0.74745	-0.17603
C	0.01118	2.00928	-0.11840
C	1.38177	1.86716	-0.45892
C	1.62422	0.52681	-0.72228
N	0.42794	-0.13299	-0.55164
H	0.31760	-1.13469	-0.62341
H	-0.51227	2.92673	0.11393
H	2.12054	2.65522	-0.51885
C	-1.93358	0.33517	0.07441
C	-2.90958	1.00541	0.78008
S	-2.57693	-1.17316	-0.56431
C	-4.15852	0.31775	0.81656
H	-2.72630	1.95341	1.27309
C	-4.13680	-0.87604	0.14999
H	-5.03528	0.69319	1.33291
H	-4.92883	-1.60244	0.03230
C	2.88559	-0.19304	-1.08303
H	3.53865	0.50688	-1.61704
C	3.67522	-0.78306	0.11601
H	4.55735	-1.27345	-0.31914
C	2.86878	-1.84728	0.86876
C	4.16293	0.31573	1.06569
H	4.76661	-0.10847	1.87610
H	4.77636	1.05471	0.53649
H	3.31544	0.84560	1.51528
H	2.65580	-1.00554	-1.78528
H	1.98858	-1.40692	1.35166
H	2.52441	-2.64076	0.19393
H	3.47722	-2.31413	1.65152

4o_conformer-034

C	-0.56275	-0.49357	-0.31596
C	-0.09281	-1.79610	-0.18526
C	1.29923	-1.78285	-0.46204
C	1.66667	-0.47769	-0.75636
N	0.52506	0.28585	-0.66642
H	0.49771	1.28740	-0.79120
H	-0.69966	-2.65831	0.05765
H	1.97034	-2.63120	-0.45690
C	-1.88631	0.06453	-0.16318
C	-2.36864	1.29271	-0.56347
S	-3.15015	-0.84957	0.64585
C	-3.74157	1.49923	-0.23217
H	-1.76935	2.02102	-1.09906
C	-4.30430	0.43411	0.41361
H	-4.28737	2.40401	-0.47637
H	-5.31933	0.31549	0.76667
C	3.00082	0.12060	-1.07539
H	2.87323	0.93328	-1.80287
C	3.78702	0.66792	0.14599
H	4.72541	1.07190	-0.25924
C	4.13887	-0.44678	1.13635
C	3.04362	1.81290	0.84300
H	2.80135	2.61985	0.14046
H	2.10683	1.46041	1.29019
H	3.65448	2.24036	1.64613
H	3.61232	-0.64563	-1.56585
H	4.70698	-1.24749	0.64790
H	3.23130	-0.89162	1.56004
H	4.74379	-0.05738	1.96314

4o_conformer-035

C	0.56278	-0.49363	-0.31589
C	0.09286	-1.79616	-0.18514
C	-1.29917	-1.78295	-0.46193
C	-1.66665	-0.47780	-0.75629
N	-0.52504	0.28576	-0.66639
H	-0.49771	1.28730	-0.79116
H	0.69973	-2.65835	0.05780
H	-1.97026	-2.63131	-0.45671
C	1.88632	0.06452	-0.16314
C	2.36854	1.29278	-0.56334
S	3.15027	-0.84956	0.64573
C	3.74146	1.49936	-0.23210
H	1.76917	2.02108	-1.09885
C	4.30431	0.43422	0.41353

H	4.28718	2.40420	-0.47625
H	5.31936	0.31564	0.76652
C	-3.00080	0.12046	-1.07538
H	-3.61225	-0.64586	-1.56575
C	-3.78705	0.66788	0.14591
H	-4.72548	1.07173	-0.25939
C	-3.04379	1.81304	0.84275
C	-4.13884	-0.44671	1.13642
H	-3.23126	-0.89147	1.56017
H	-4.74378	-0.05722	1.96317
H	-4.70693	-1.24750	0.64808
H	-2.87324	0.93306	-1.80295
H	-3.65469	2.24054	1.64583
H	-2.10695	1.46075	1.29000
H	-2.80163	2.61993	0.14010

4o_conformer-036

C	0.59015	0.82151	-0.08464
C	0.02176	2.02209	0.32697
C	-1.36082	1.97591	0.00781
C	-1.62525	0.75184	-0.58889
N	-0.43244	0.06545	-0.63031
H	-0.30984	-0.83232	-1.07723
H	0.55198	2.82515	0.82060
H	-2.09655	2.74611	0.19728
C	1.95528	0.35447	-0.00018
C	3.10513	1.10061	0.14205
S	2.34624	-1.36130	-0.02677
C	4.28976	0.31002	0.22060
H	3.09800	2.18441	0.16827
C	4.04882	-1.03309	0.13122
H	5.28478	0.72882	0.32455
H	4.75525	-1.85143	0.14303
C	-2.90332	0.15799	-1.09192
H	-2.70753	-0.41692	-2.00720
C	-3.64709	-0.75701	-0.08260
H	-4.55384	-1.09888	-0.60125
C	-4.07818	0.01688	1.16736
C	-2.82326	-1.99521	0.28908
H	-1.91434	-1.71549	0.83421
H	-3.40244	-2.66801	0.93160
H	-2.52294	-2.55881	-0.60290
H	-3.57253	0.97847	-1.37514
H	-4.65224	-0.62499	1.84529
H	-3.20491	0.39195	1.71315

H	-4.70345	0.87868	0.90503

4o_conformer-037

C	-0.59018	0.82151	-0.08465
C	-0.02183	2.02212	0.32694
C	1.36077	1.97596	0.00781
C	1.62524	0.75189	-0.58885
N	0.43245	0.06547	-0.63028
H	0.30988	-0.83233	-1.07716
H	-0.55208	2.82518	0.82054
H	2.09649	2.74617	0.19729
C	-1.95530	0.35445	-0.00021
C	-3.10516	1.10057	0.14193
S	-2.34621	-1.36134	-0.02667
C	-4.28978	0.30996	0.22052
H	-3.09806	2.18438	0.16806
C	-4.04880	-1.03315	0.13126
H	-5.28481	0.72875	0.32441
H	-4.75522	-1.85151	0.14311
C	2.90338	0.15817	-1.09184
H	3.57252	0.97876	-1.37489
C	3.64718	-0.75689	-0.08258
H	4.55411	-1.09843	-0.60113
C	2.82357	-1.99536	0.28867
C	4.07784	0.01684	1.16763
H	4.65196	-0.62503	1.84552
H	4.70290	0.87888	0.90561
H	3.20437	0.39154	1.71336
H	2.70772	-0.41664	-2.00720
H	2.52373	-2.55894	-0.60348
H	3.40273	-2.66808	0.93130
H	1.91439	-1.71598	0.83353

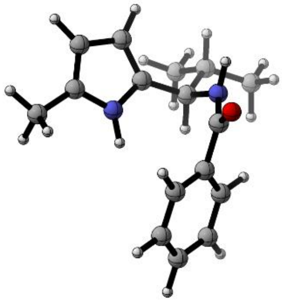

4p_conformer-001

C	1.59140	0.38292	-0.04842
C	2.83069	0.86060	0.33619
C	2.80200	2.28241	0.21749

C	1.54550	2.64652	-0.23132
N	0.82622	1.48242	-0.38765
H	3.61241	2.96817	0.42786
C	0.95610	3.98402	-0.54161
H	-0.14046	1.43935	-0.68102
H	0.68361	4.07829	-1.60086
H	0.04880	4.17623	0.04551
H	1.67803	4.77204	-0.31076
C	0.98135	-0.98935	-0.01924
H	3.66465	0.25521	0.66536
N	0.28723	-1.21221	1.27541
H	0.21729	-1.04842	-0.79776
C	1.98994	-2.12811	-0.27278
C	1.31336	-3.49984	-0.16252
C	2.65298	-1.96904	-1.64683
H	2.77061	-2.06367	0.49927
H	3.37311	-2.77665	-1.81654
H	1.90031	-2.01979	-2.44405
H	3.17759	-1.01471	-1.73610
H	2.04371	-4.29821	-0.33256
H	0.85861	-3.65317	0.81964
H	0.52481	-3.60195	-0.91933
H	0.91983	-1.30291	2.06631
C	-0.89594	-0.63597	1.64717
O	-1.15704	-0.39458	2.82875
C	-3.79465	0.29878	-1.38411
C	-3.18910	-0.95980	-1.38133
C	-2.23307	-1.27391	-0.41540
C	-1.88997	-0.33399	0.56517
C	-2.52218	0.91608	0.57695
C	-3.45897	1.23734	-0.40472
H	-4.52932	0.54631	-2.14453
H	-3.45869	-1.69626	-2.13230
H	-1.76301	-2.25254	-0.41122
H	-2.26492	1.63102	1.35211
H	-3.93145	2.21499	-0.40153

4p_conformer-002

C	-1.68156	0.06683	-0.47640
C	-2.71167	0.08106	-1.39908
C	-3.40199	-1.16366	-1.29689
C	-2.78472	-1.91276	-0.31315
N	-1.73936	-1.15425	0.16772
H	-4.26198	-1.47793	-1.87410
C	-3.08891	-3.27050	0.23176

H	-1.13956	-1.43168	0.93194
H	-2.24190	-3.95905	0.11621
H	-3.33860	-3.23883	1.30042
H	-3.94364	-3.70009	-0.29783
C	-0.58155	1.06249	-0.18956
H	-2.93908	0.89023	-2.07966
N	-0.02285	0.83645	1.15473
H	0.23981	0.89743	-0.89241
C	-1.05050	2.52667	-0.35830
C	-2.11156	2.92623	0.67452
C	0.13707	3.49375	-0.33483
H	-1.50911	2.57780	-1.35368
H	0.66114	3.44298	0.62633
H	0.85604	3.26465	-1.12967
H	-0.20375	4.52478	-0.47777
H	-2.50566	3.92226	0.44727
H	-1.68674	2.96929	1.68501
H	-2.94841	2.22101	0.67994
H	-0.69872	0.92805	1.90783
C	0.94449	-0.08331	1.47516
O	0.92756	-0.66370	2.56562
C	4.13498	-0.92945	-1.26762
C	3.70843	0.39008	-1.10323
C	2.65663	0.68160	-0.23483
C	2.03298	-0.34717	0.48353
C	2.48298	-1.66547	0.33568
C	3.52056	-1.95733	-0.54685
H	4.94820	-1.15616	-1.95081
H	4.19511	1.19307	-1.64856
H	2.33590	1.70789	-0.09531
H	2.00802	-2.45246	0.91246
H	3.85413	-2.98344	-0.66922

4p_conformer-003

C	-0.54005	1.17338	0.68891
C	-0.08764	1.80154	1.83445
C	1.17882	2.38449	1.54054
C	1.47251	2.10755	0.21686
N	0.41276	1.39063	-0.28697
H	1.80533	2.95646	2.21260
C	2.65540	2.45737	-0.62491
H	0.41770	0.94497	-1.19319
H	3.14750	1.56003	-1.02036
H	2.37967	3.08502	-1.48219
H	3.38745	3.00832	-0.02807

C	-1.79985	0.37941	0.47904
H	-0.61977	1.83716	2.77649
N	-1.71207	-1.00216	1.03354
H	-2.56442	0.84713	1.11003
C	-2.35065	0.42643	-0.96703
C	-3.56962	-0.48757	-1.12502
C	-2.70281	1.86970	-1.35188
H	-1.57190	0.06689	-1.65084
H	-3.48323	2.26476	-0.68923
H	-1.83624	2.53353	-1.28629
H	-3.08524	1.90766	-2.37744
H	-3.95567	-0.43385	-2.14855
H	-3.32189	-1.52988	-0.90510
H	-4.37572	-0.18110	-0.44581
H	-2.44044	-1.23544	1.69894
C	-0.61898	-1.80227	1.18633
O	-0.58244	-2.68334	2.05128
C	2.69671	-1.33200	-1.50062
C	1.42491	-1.60042	-2.01283
C	0.33974	-1.74311	-1.14624
C	0.52400	-1.61940	0.23600
C	1.80600	-1.39261	0.74720
C	2.88541	-1.23379	-0.11913
H	3.53954	-1.20982	-2.17444
H	1.27775	-1.70151	-3.08399
H	-0.64713	-1.96217	-1.54045
H	1.94371	-1.31176	1.82051
H	3.87422	-1.03104	0.28127

4p_conformer-004

C	-0.84913	-0.64210	-0.40012
C	-0.27436	-1.24257	-1.50107
C	0.00072	-2.60030	-1.15870
C	-0.40751	-2.79912	0.14741
N	-0.92844	-1.60109	0.58970
H	0.45887	-3.35105	-1.78943
C	-0.35004	-4.00680	1.02506
H	-1.28080	-1.43906	1.52238
H	0.10516	-4.83928	0.48162
H	0.24766	-3.82924	1.92859
H	-1.34792	-4.32599	1.35296
C	-1.32898	0.76275	-0.18956
H	-0.05734	-0.74669	-2.43781
N	-0.79344	1.33398	1.06772
H	-0.96897	1.35528	-1.03662

C	-2.87290	0.90395	-0.16121
C	-3.29287	2.37681	-0.05918
C	-3.50117	0.25445	-1.39827
H	-3.23654	0.36847	0.72892
H	-3.13184	0.73034	-2.31540
H	-3.26864	-0.81195	-1.45707
H	-4.58967	0.37196	-1.37403
H	-4.38444	2.46032	-0.03007
H	-2.89606	2.86768	0.83464
H	-2.93589	2.93562	-0.93333
H	-1.46100	1.44268	1.82369
C	0.48949	1.22211	1.52689
O	0.74988	1.30860	2.73035
C	3.65030	0.59301	-1.30664
C	2.70019	1.58772	-1.54984
C	1.65874	1.79429	-0.64595
C	1.57078	1.01421	0.51287
C	2.54529	0.04392	0.77146
C	3.57215	-0.17786	-0.14364
H	4.45362	0.42381	-2.01778
H	2.76937	2.20126	-2.44326
H	0.92322	2.57061	-0.83222
H	2.47768	-0.54327	1.68164
H	4.31349	-0.94739	0.05062

4p_conformer-005

C	-0.87496	-0.44254	-0.47945
C	-0.46237	-1.15377	-1.58831
C	-0.15501	-2.48050	-1.16186
C	-0.38048	-2.55030	0.20041
N	-0.82859	-1.30792	0.59476
H	0.20577	-3.29473	-1.77680
C	-0.20780	-3.67082	1.17307
H	-1.04476	-1.05179	1.54729
H	-1.15126	-3.93792	1.66663
H	0.16148	-4.55963	0.65424
H	0.51260	-3.41955	1.96245
C	-1.28543	0.99535	-0.33987
H	-0.36842	-0.75031	-2.58781
N	-0.75173	1.59597	0.89861
H	-0.87195	1.52892	-1.20173
C	-2.82101	1.23168	-0.37398
C	-3.37093	0.91198	-1.76696
C	-3.58168	0.44790	0.70290
H	-2.96220	2.30631	-0.18988

H	-3.50466	-0.63007	0.52616
H	-3.21202	0.64923	1.71524
H	-4.64248	0.71837	0.68432
H	-4.44473	1.12182	-1.81183
H	-2.87436	1.51089	-2.53893
H	-3.21849	-0.14537	-2.00950
H	-1.42892	1.90933	1.58439
C	0.50362	1.44835	1.41484
O	0.74126	1.67737	2.60433
C	3.66652	0.19082	-1.20007
C	2.86409	1.27461	-1.56387
C	1.82101	1.68071	-0.73223
C	1.58748	1.01408	0.47594
C	2.41619	-0.04762	0.85497
C	3.44086	-0.46991	0.01046
H	4.46937	-0.13479	-1.85515
H	3.04740	1.80034	-2.49632
H	1.19605	2.52231	-1.01512
H	2.23644	-0.54897	1.80060
H	4.06666	-1.30985	0.29754

4p_conformer-006

C	0.83484	0.70636	0.17261
C	0.89438	1.14169	1.48518
C	-0.05810	2.19212	1.63419
C	-0.68432	2.37501	0.41377
N	-0.11444	1.48057	-0.46163
H	-0.26885	2.75481	2.53448
C	-1.78654	3.29094	-0.00573
H	-0.46183	1.29710	-1.39265
H	-1.48237	3.95963	-0.82125
H	-2.66342	2.72823	-0.35231
H	-2.09810	3.91189	0.83865
C	1.50443	-0.43008	-0.55156
H	1.53331	0.73298	2.25562
N	1.27230	-1.72357	0.12691
H	1.05484	-0.48911	-1.54862
C	3.02566	-0.24966	-0.78740
C	3.26551	0.94117	-1.72121
C	3.84422	-0.09515	0.50011
H	3.35673	-1.16283	-1.30174
H	4.91240	-0.06854	0.26058
H	3.58733	0.83790	1.01091
H	3.68869	-0.91802	1.20738
H	4.33207	1.04220	-1.94768

H	2.92856	1.87301	-1.25312
H	2.72646	0.82319	-2.66819
H	2.07198	-2.14255	0.58785
C	0.08120	-2.12259	0.66360
O	0.02035	-2.96508	1.56235
C	-3.46534	-0.30102	-0.93753
C	-2.55393	-0.91178	-1.80309
C	-1.39954	-1.50687	-1.29399
C	-1.15651	-1.50279	0.08516
C	-2.09008	-0.92498	0.95126
C	-3.23260	-0.31215	0.44020
H	-4.35723	0.17478	-1.33463
H	-2.74087	-0.92329	-2.87286
H	-0.68853	-1.97847	-1.96583
H	-1.89733	-0.93065	2.01886
H	-3.94130	0.15811	1.11543

4p_conformer-007

C	0.83481	0.70629	0.17277
C	0.89434	1.14156	1.48535
C	-0.05795	2.19219	1.63431
C	-0.68403	2.37524	0.41384
N	-0.11425	1.48071	-0.46154
H	-0.26866	2.75491	2.53459
C	-1.78612	3.29132	-0.00567
H	-0.46164	1.29726	-1.39256
H	-1.48201	3.95960	-0.82154
H	-2.66329	2.72875	-0.35176
H	-2.09724	3.91269	0.83858
C	1.50431	-0.43018	-0.55146
H	1.53314	0.73272	2.25582
N	1.27222	-1.72367	0.12701
H	1.05464	-0.48922	-1.54848
C	3.02554	-0.24976	-0.78743
C	3.26531	0.94106	-1.72126
C	3.84419	-0.09522	0.50002
H	3.35656	-1.16295	-1.30177
H	4.91235	-0.06853	0.26038
H	3.58728	0.83779	1.01087
H	3.68881	-0.91812	1.20729
H	4.33183	1.04203	-1.94789
H	2.72609	0.82313	-2.66815
H	2.92849	1.87292	-1.25310
H	2.07187	-2.14264	0.58802
C	0.08107	-2.12283	0.66348

O	0.02013	-2.96547	1.56208
C	-3.46528	-0.30078	-0.93753
C	-3.23263	-0.31219	0.44022
C	-2.09018	-0.92521	0.95121
C	-1.15660	-1.50291	0.08506
C	-1.39954	-1.50669	-1.29410
C	-2.55387	-0.91142	-1.80315
H	-4.35712	0.17517	-1.33459
H	-3.94133	0.15799	1.11550
H	-1.89747	-0.93106	2.01883
H	-0.68853	-1.97822	-1.96601
H	-2.74074	-0.92271	-2.87294

4p_conformer-008

C	1.34488	0.80961	-0.16072
C	1.68513	0.22118	-1.36352
C	2.84769	-0.57540	-1.14326
C	3.19648	-0.45934	0.18978
N	2.28153	0.39649	0.76484
H	3.37505	-1.17030	-1.87751
C	4.31784	-1.06235	0.97173
H	2.23648	0.61910	1.74945
H	3.95413	-1.64081	1.83052
H	4.89401	-1.73774	0.33346
H	5.00538	-0.30004	1.36063
C	0.20586	1.69034	0.27162
H	1.14363	0.32160	-2.29305
N	-0.44459	1.16017	1.50044
H	0.62038	2.64654	0.61848
C	-0.77906	2.02968	-0.87594
C	-2.06739	2.67593	-0.35181
C	-0.10807	2.97689	-1.88353
H	-1.04143	1.09449	-1.38516
H	0.83614	2.58087	-2.26337
H	-0.77177	3.15745	-2.73596
H	0.09957	3.94578	-1.41172
H	-2.70475	2.96922	-1.19281
H	-1.84211	3.58016	0.22769
H	-2.64843	2.00558	0.28684
H	-0.57763	1.85121	2.23072
C	-1.38291	0.16814	1.60636
O	-2.17950	0.15874	2.54998
C	-1.65851	-3.02376	-1.25258
C	-0.43490	-2.76439	-0.63409
C	-0.31323	-1.70890	0.27067

C	-1.42692	-0.91888	0.57546
C	-2.66333	-1.20472	-0.02029
C	-2.77492	-2.23996	-0.94567
H	-1.74566	-3.83722	-1.96721
H	0.43325	-3.37592	-0.86115
H	0.64020	-1.51385	0.74610
H	-3.52967	-0.60505	0.24132
H	-3.73117	-2.44191	-1.41928

4p_conformer-009

C	-1.81332	0.07362	-0.41495
C	-2.85627	0.08443	-1.32217
C	-3.77436	-0.93788	-0.93342
C	-3.27649	-1.54927	0.20212
N	-2.08180	-0.92726	0.49470
H	-4.70264	-1.19738	-1.42599
C	-3.81908	-2.65640	1.04634
H	-1.50430	-1.10902	1.30380
H	-4.76524	-3.01181	0.62905
H	-3.13175	-3.51089	1.09512
H	-4.00884	-2.33290	2.07818
C	-0.52972	0.85820	-0.34596
H	-2.94679	0.74396	-2.17467
N	0.02989	0.78932	1.01167
H	0.21171	0.39192	-1.00074
C	-0.68255	2.32208	-0.83209
C	-1.64709	3.14445	0.02978
C	0.68900	2.99822	-0.93601
H	-1.10559	2.25162	-1.84277
H	1.14622	3.11626	0.05319
H	1.37769	2.41426	-1.55755
H	0.59371	3.99530	-1.37875
H	-2.62054	2.65291	0.11795
H	-1.24791	3.30482	1.03933
H	-1.80099	4.13396	-0.41339
H	-0.43246	1.37249	1.70233
C	1.33237	0.53213	1.35370
O	1.80985	0.96974	2.40339
C	3.78396	-1.97860	-1.12289
C	2.43613	-2.30041	-0.94933
C	1.61627	-1.48326	-0.17149
C	2.14464	-0.34140	0.44724
C	3.50304	-0.03820	0.29020
C	4.31630	-0.84517	-0.50248
H	4.41906	-2.61181	-1.73542

H	2.02224	-3.18838	-1.41761
H	0.57137	-1.74302	-0.03432
H	3.90761	0.83505	0.79123
H	5.36498	-0.59482	-0.63220

4p_conformer-010

C	0.84898	0.64231	-0.40017
C	0.27366	1.24253	-1.50097
C	-0.00198	2.60012	-1.15850
C	0.40659	2.79915	0.14748
N	0.92821	1.60136	0.58961
H	-0.46067	3.35067	-1.78908
C	0.34868	4.00676	1.02519
H	1.28096	1.43952	1.52217
H	-0.10706	4.83902	0.48185
H	-0.24876	3.82886	1.92883
H	1.34645	4.32644	1.35293
C	1.32930	-0.76238	-0.18964
H	0.05652	0.74655	-2.43764
N	0.79386	-1.33380	1.06760
H	0.96949	-1.35500	-1.03672
C	2.87324	-0.90317	-0.16123
C	3.29364	-2.37592	-0.05929
C	3.50129	-0.25344	-1.39826
H	3.23675	-0.36766	0.72896
H	4.58983	-0.37069	-1.37409
H	3.13203	-0.72938	-2.31540
H	3.26849	0.81290	-1.45700
H	4.38524	-2.45908	-0.03019
H	2.89700	-2.86697	0.83451
H	2.93683	-2.93478	-0.93347
H	1.46145	-1.44239	1.82356
C	-0.48908	-1.22242	1.52681
O	-0.74945	-1.30923	2.73026
C	-3.65007	-0.59364	-1.30654
C	-2.69973	-1.58810	-1.54991
C	-1.65822	-1.79458	-0.64609
C	-1.57041	-1.01463	0.51283
C	-2.54510	-0.04458	0.77157
C	-3.57204	0.17710	-0.14346
H	-4.45346	-0.42453	-2.01763
H	-2.76881	-2.20153	-2.44341
H	-0.92250	-2.57067	-0.83249
H	-2.47754	0.54251	1.68182
H	-4.31353	0.94645	0.05092

4p_conformer-011

C	-1.81309	0.07334	-0.41504
C	-2.85602	0.08369	-1.32228
C	-3.77385	-0.93878	-0.93334
C	-3.27585	-1.54979	0.20236
N	-2.08133	-0.92739	0.49484
H	-4.70203	-1.19864	-1.42588
C	-3.81814	-2.65692	1.04678
H	-1.50380	-1.10884	1.30398
H	-3.13065	-3.51128	1.09558
H	-4.00785	-2.33332	2.07859
H	-4.76428	-3.01255	0.62962
C	-0.52978	0.85842	-0.34609
H	-2.94669	0.74302	-2.17492
N	0.02996	0.78976	1.01150
H	0.21179	0.39251	-1.00096
C	-0.68328	2.32229	-0.83210
C	-1.64804	3.14418	0.02996
C	0.68798	2.99900	-0.93615
H	-1.10643	2.25173	-1.84272
H	0.59222	3.99608	-1.37878
H	1.14530	3.11712	0.05299
H	1.37682	2.41537	-1.55785
H	-1.80238	4.13368	-0.41308
H	-2.62129	2.65224	0.11818
H	-1.24883	3.30459	1.03949
H	-0.43228	1.37307	1.70209
C	1.33245	0.53254	1.35349
O	1.81001	0.97030	2.40308
C	3.78387	-1.97865	-1.12279
C	2.43597	-2.30024	-0.94937
C	1.61616	-1.48294	-0.17163
C	2.14465	-0.34115	0.44712
C	3.50312	-0.03818	0.29023
C	4.31633	-0.84530	-0.50234
H	4.41893	-2.61198	-1.73524
H	2.02199	-3.18816	-1.41766
H	0.57121	-1.74254	-0.03454
H	3.90777	0.83501	0.79130
H	5.36506	-0.59512	-0.63196

4p_conformer-012

C	-1.61751	-0.24082	-0.20432

C	-1.76776	-1.42280	-0.90320
C	-3.03548	-1.97720	-0.55188
C	-3.63334	-1.12303	0.35499
N	-2.76085	-0.07248	0.55173
H	-3.46655	-2.90023	-0.91694
C	-4.94710	-1.19972	1.06252
H	-2.94106	0.70450	1.17142
H	-4.82510	-1.23859	2.15277
H	-5.58594	-0.33602	0.83716
H	-5.48276	-2.10153	0.75392
C	-0.49640	0.75460	-0.21197
H	-1.04496	-1.83150	-1.59721
N	0.13960	0.86966	1.11496
H	0.26246	0.37230	-0.89713
C	-0.91185	2.15992	-0.72206
C	0.30020	3.09716	-0.78496
C	-1.59473	2.05800	-2.09064
H	-1.63715	2.57811	-0.00749
H	-1.87874	3.05325	-2.44883
H	-0.91446	1.61629	-2.82970
H	-2.49420	1.43756	-2.04898
H	0.76415	3.23683	0.19585
H	1.06259	2.69419	-1.46393
H	0.00308	4.08189	-1.16106
H	-0.32430	1.48882	1.77244
C	1.45625	0.69056	1.43100
O	1.95145	1.23226	2.42514
C	3.92600	-1.90129	-0.94105
C	2.60294	-2.26638	-0.68332
C	1.77749	-1.42537	0.06319
C	2.27639	-0.21547	0.56453
C	3.61177	0.13314	0.32571
C	4.42941	-0.69923	-0.43561
H	4.56515	-2.55334	-1.52927
H	2.21265	-3.20665	-1.06153
H	0.75047	-1.71212	0.26485
H	3.99479	1.06061	0.73876
H	5.45930	-0.41510	-0.63058

4p_conformer-013

C	0.70849	0.72843	-0.64148
C	0.10339	1.31591	-1.73368
C	-0.42090	2.57475	-1.31200
C	-0.13060	2.72654	0.03113
N	0.56561	1.60094	0.41812

H	-0.96482	3.28870	-1.91682
C	-0.45152	3.82411	0.99240
H	0.88126	1.41629	1.35946
H	-1.06443	3.46800	1.83070
H	0.45298	4.27625	1.41964
H	-1.01030	4.61318	0.48180
C	1.39524	-0.60156	-0.51196
H	0.02865	0.87405	-2.71873
N	1.10906	-1.23907	0.78731
H	1.00478	-1.23695	-1.31301
C	2.93150	-0.53588	-0.72855
C	3.65928	0.35820	0.28326
C	3.54513	-1.94109	-0.78342
H	3.04506	-0.07723	-1.71850
H	4.60136	-1.88469	-1.06735
H	3.49221	-2.44668	0.18771
H	3.02814	-2.57030	-1.51684
H	4.72233	0.42129	0.02855
H	3.25081	1.37296	0.28799
H	3.59904	-0.03613	1.30558
H	1.88366	-1.32982	1.43410
C	-0.10513	-1.32542	1.40753
O	-0.19207	-1.51509	2.62403
C	-3.66209	-0.92044	-0.96026
C	-2.66797	-1.81461	-1.36385
C	-1.49980	-1.94532	-0.61351
C	-1.32792	-1.19119	0.55284
C	-2.34120	-0.32248	0.97363
C	-3.49705	-0.17443	0.20990
H	-4.56544	-0.80883	-1.55286
H	-2.80074	-2.40793	-2.26372
H	-0.72808	-2.64211	-0.92585
H	-2.20492	0.24656	1.88761
H	-4.27082	0.51794	0.52831

4p_conformer-014

C	-0.87518	0.44246	0.47926
C	-0.46293	1.15407	1.58802
C	-0.15625	2.48088	1.16139
C	-0.38176	2.55037	-0.20089
N	-0.82923	1.30772	-0.59507
H	0.20412	3.29537	1.77622
C	-0.20967	3.67086	-1.17370
H	-1.04538	1.05141	-1.54755
H	0.15893	4.56000	-0.65496

H	0.51102	3.41996	-1.96293
H	-1.15324	3.93725	-1.66745
C	-1.28517	-0.99561	0.34002
H	-0.36876	0.75081	2.58758
N	-0.75113	-1.59641	-0.89822
H	-0.87166	-1.52878	1.20211
C	-2.82068	-1.23230	0.37394
C	-3.37087	-0.91282	1.76687
C	-3.58139	-0.44855	-0.70294
H	-2.96165	-2.30694	0.18976
H	-4.64209	-0.71944	-0.68473
H	-3.50484	0.62940	-0.52588
H	-3.21136	-0.64945	-1.71524
H	-4.44460	-1.12300	1.81162
H	-2.87419	-1.51158	2.53888
H	-3.21881	0.14458	2.00946
H	-1.42805	-1.91070	-1.58384
C	0.50433	-1.44916	-1.41420
O	0.74225	-1.67920	-2.60344
C	3.66699	-0.18916	1.19983
C	2.86475	-1.27283	1.56441
C	1.82169	-1.67966	0.73310
C	1.58797	-1.01388	-0.47552
C	2.41648	0.04773	-0.85527
C	3.44115	0.47072	-0.01112
H	4.46984	0.13699	1.85464
H	3.04820	-1.79792	2.49719
H	1.19691	-2.52120	1.01658
H	2.23663	0.54841	-1.80123
H	4.06680	1.31056	-0.29880

4p_conformer-015

C	-1.39634	-0.20997	-0.37381
C	-1.82618	-0.98033	-1.43849
C	-2.63958	-2.02844	-0.91103
C	-2.68477	-1.88072	0.46288
N	-1.93080	-0.76762	0.76773
H	-3.13245	-2.81266	-1.47100
C	-3.35954	-2.69140	1.52121
H	-1.71887	-0.44520	1.70130
H	-2.64218	-3.10565	2.24164
H	-4.08765	-2.09985	2.09125
H	-3.89400	-3.52833	1.06349
C	-0.53904	1.02725	-0.32155
H	-1.57424	-0.81170	-2.47686

N	0.10721	1.17754	0.99234
H	0.25442	0.92238	-1.06688
C	-1.30947	2.33649	-0.65992
C	-1.71881	2.36122	-2.13580
C	-2.52958	2.55774	0.24243
H	-0.59266	3.15165	-0.49077
H	-2.27378	2.58138	1.30860
H	-3.00511	3.51494	0.00442
H	-3.26947	1.76401	0.09381
H	-2.18648	3.32003	-2.38381
H	-2.43919	1.56601	-2.35418
H	-0.85211	2.22574	-2.79313
H	-0.33276	1.83033	1.63161
C	1.42330	0.97718	1.31126
O	1.91213	1.51031	2.31214
C	3.94562	-1.55777	-1.06994
C	2.61022	-1.91642	-0.87877
C	1.76325	-1.09762	-0.13114
C	2.25278	0.08540	0.43869
C	3.60147	0.42690	0.26467
C	4.44045	-0.38263	-0.49673
H	4.60088	-2.19350	-1.65833
H	2.22465	-2.83555	-1.31006
H	0.72931	-1.38682	0.01630
H	3.97716	1.33201	0.73020
H	5.47997	-0.10238	-0.63903

4p_conformer-016

C	-1.61765	-0.24107	-0.20416
C	-1.76770	-1.42367	-0.90204
C	-3.03572	-1.97754	-0.55092
C	-3.63396	-1.12243	0.35481
N	-2.76138	-0.07186	0.55110
H	-3.46674	-2.90082	-0.91538
C	-4.94816	-1.19821	1.06163
H	-2.94191	0.70586	1.16977
H	-5.48379	-2.10021	0.75356
H	-4.82680	-1.23608	2.15199
H	-5.58670	-0.33460	0.83508
C	-0.49636	0.75412	-0.21194
H	-1.04459	-1.83314	-1.59526
N	0.13954	0.86931	1.11505
H	0.26251	0.37154	-0.89692
C	-0.91155	2.15939	-0.72238
C	0.30057	3.09654	-0.78514

C	-1.59411	2.05724	-2.09110
H	-1.63699	2.57775	-0.00805
H	-1.87804	3.05244	-2.44952
H	-0.91366	1.61542	-2.82993
H	-2.49358	1.43681	-2.04955
H	0.00361	4.08120	-1.16155
H	0.76424	3.23640	0.19576
H	1.06313	2.69338	-1.46381
H	-0.32456	1.48835	1.77252
C	1.45624	0.69058	1.43107
O	1.95136	1.23247	2.42515
C	3.92633	-1.90100	-0.94093
C	2.60343	-2.26643	-0.68285
C	1.77789	-1.42552	0.06366
C	2.27653	-0.21537	0.56466
C	3.61176	0.13359	0.32549
C	4.42948	-0.69869	-0.43583
H	4.56555	-2.55298	-1.52915
H	2.21334	-3.20690	-1.06079
H	0.75099	-1.71252	0.26560
H	3.99459	1.06126	0.73826
H	5.45925	-0.41429	-0.63108

4p_conformer-017

C	-1.61784	-0.24129	-0.20404
C	-1.76775	-1.42442	-0.90106
C	-3.03602	-1.97780	-0.55010
C	-3.63457	-1.12186	0.35465
N	-2.76191	-0.07129	0.55054
H	-3.46702	-2.90131	-0.91402
C	-4.94914	-1.19683	1.06086
H	-2.94270	0.70709	1.16830
H	-4.82835	-1.23386	2.15131
H	-5.58740	-0.33328	0.83331
H	-5.48480	-2.09897	0.75324
C	-0.49635	0.75367	-0.21192
H	-1.04437	-1.83457	-1.59359
N	0.13945	0.86895	1.11511
H	0.26252	0.37082	-0.89674
C	-0.91127	2.15889	-0.72268
C	0.30092	3.09597	-0.78518
C	-1.59340	2.05658	-2.09161
H	-1.63691	2.57741	-0.00866
H	-0.91272	1.61468	-2.83017
H	-2.49288	1.43614	-2.05025

H	-1.87725	3.05173	-2.45024
H	0.00413	4.08059	-1.16186
H	0.76425	3.23598	0.19586
H	1.06370	2.69267	-1.46351
H	-0.32484	1.48778	1.77263
C	1.45619	0.69051	1.43115
O	1.95119	1.23251	2.42523
C	3.92674	-1.90065	-0.94083
C	2.60398	-2.26642	-0.68254
C	1.77830	-1.42565	0.06397
C	2.27667	-0.21530	0.56476
C	3.61177	0.13400	0.32541
C	4.42963	-0.69815	-0.43592
H	4.56607	-2.55252	-1.52906
H	2.21410	-3.20704	-1.06032
H	0.75151	-1.71290	0.26609
H	3.99439	1.06183	0.73802
H	5.45929	-0.41349	-0.63132

4p_conformer-018

C	1.84545	-0.53212	-0.10875
C	2.37286	-1.79048	-0.31949
C	3.77276	-1.64539	-0.55013
C	4.07485	-0.29875	-0.48181
N	2.89594	0.36442	-0.21291
H	4.48544	-2.43475	-0.74991
C	5.36910	0.43036	-0.64205
H	2.83024	1.36300	-0.08095
H	6.17225	-0.28126	-0.85148
H	5.33449	1.15110	-1.46911
H	5.64147	0.98708	0.26393
C	0.43038	-0.13101	0.17153
H	1.80407	-2.71111	-0.30989
N	-0.05461	0.76466	-0.90236
H	-0.15015	-1.05324	0.12011
C	0.17250	0.46877	1.58343
C	0.53697	-0.54983	2.66699
C	0.86278	1.81469	1.82739
H	-0.91166	0.63800	1.62789
H	1.94887	1.69803	1.91428
H	0.64911	2.53394	1.02880
H	0.50960	2.25294	2.76684
H	0.01401	-1.50073	2.51102
H	1.61328	-0.75490	2.66555
H	0.26361	-0.17145	3.65793

H	0.61982	1.42638	-1.27054
C	-1.34153	1.17435	-1.10828
O	-1.59419	2.22703	-1.70190
C	-4.59879	-1.30785	0.19039
C	-3.52716	-1.88559	-0.49374
C	-2.45049	-1.09714	-0.90009
C	-2.44586	0.27816	-0.63318
C	-3.53724	0.85774	0.02573
C	-4.60267	0.06546	0.44917
H	-5.43202	-1.92433	0.51468
H	-3.52976	-2.94899	-0.71358
H	-1.62635	-1.54431	-1.44742
H	-3.53752	1.92788	0.20689
H	-5.43812	0.51865	0.97447

4p_conformer-019

C	-1.91662	0.17316	-0.23786
C	-3.05789	0.74728	-0.76890
C	-4.11452	-0.20647	-0.66214
C	-3.60312	-1.34359	-0.06646
N	-2.27044	-1.09796	0.17511
H	-5.14042	-0.07332	-0.97990
C	-4.25071	-2.63657	0.31006
H	-1.64025	-1.72239	0.65869
H	-5.30567	-2.62123	0.02307
H	-3.77909	-3.49282	-0.18906
H	-4.19982	-2.82191	1.39087
C	-0.47975	0.60500	-0.10737
H	-3.13789	1.73659	-1.19304
N	0.01042	0.13685	1.20760
H	0.11324	0.07466	-0.85807
C	-0.18180	2.11229	-0.31209
C	-0.35028	2.53870	-1.77650
C	-0.94145	3.02844	0.65352
H	0.88854	2.20419	-0.08044
H	-0.75356	2.75487	1.69789
H	-0.61571	4.06598	0.52231
H	-2.02127	2.98799	0.48474
H	0.03626	3.55391	-1.91814
H	0.20605	1.87117	-2.44508
H	-1.39597	2.53489	-2.09468
H	-0.55370	0.43618	1.99747
C	1.32383	-0.03994	1.56025
O	1.69424	0.09098	2.72910
C	4.20430	-1.22962	-1.39569

C	2.95350	-1.85073	-1.37146
C	1.99672	-1.45878	-0.43563
C	2.29091	-0.44932	0.49110
C	3.55645	0.14994	0.48048
C	4.50443	-0.22833	-0.46855
H	4.94532	-1.52953	-2.13080
H	2.72295	-2.64142	-2.07908
H	1.03034	-1.95269	-0.41029
H	3.78466	0.91395	1.21667
H	5.47821	0.25210	-0.48131

4p_conformer-020

C	-1.84529	0.53219	-0.10796
C	-2.37247	1.79094	-0.31691
C	-3.77236	1.64643	-0.54798
C	-4.07467	0.29973	-0.48173
N	-2.89591	-0.36402	-0.21363
H	-4.48488	2.43619	-0.74669
C	-5.36900	-0.42895	-0.64333
H	-2.83041	-1.36281	-0.08312
H	-5.64144	-0.98729	0.26163
H	-6.17207	0.28313	-0.85148
H	-5.33444	-1.14819	-1.47170
C	-0.43032	0.13044	0.17195
H	-1.80353	2.71147	-0.30584
N	0.05464	-0.76393	-0.90304
H	0.15040	1.05261	0.12177
C	-0.17268	-0.47121	1.58310
C	-0.53721	0.54602	2.66793
C	-0.86309	-1.81739	1.82520
H	0.91147	-0.64058	1.62746
H	-1.94919	-1.70079	1.91191
H	-0.64922	-2.53566	1.02578
H	-0.51019	-2.25683	2.76420
H	-0.26406	0.16631	3.65842
H	-0.01411	1.49706	2.51330
H	-1.61349	0.75121	2.66657
H	-0.61972	-1.42541	-1.27178
C	1.34161	-1.17325	-1.10944
O	1.59440	-2.22528	-1.70415
C	4.59856	1.30813	0.19159
C	3.52689	1.88636	-0.49208
C	2.45032	1.09816	-0.89916
C	2.44583	-0.27738	-0.63347
C	3.53724	-0.85743	0.02496

C	4.60257	-0.06541	0.44915
H	5.43171	1.92441	0.51645
H	3.52939	2.94996	-0.71098
H	1.62614	1.54571	-1.44612
H	3.53762	-1.92773	0.20518
H	5.43804	-0.51897	0.97409

4p_conformer-021

C	-1.45763	-0.21570	0.09863
C	-1.59588	-0.98136	1.24093
C	-2.58581	-1.97639	0.97560
C	-3.02968	-1.79994	-0.32022
N	-2.33576	-0.72417	-0.83715
H	-2.93797	-2.73965	1.65724
C	-4.04585	-2.54312	-1.12480
H	-2.44230	-0.37734	-1.77972
H	-4.87283	-1.89499	-1.44290
H	-3.61090	-2.98357	-2.03141
H	-4.46887	-3.35604	-0.52835
C	-0.55223	0.94984	-0.20336
H	-1.04357	-0.84366	2.16004
N	0.24295	1.27973	0.98598
H	0.15084	0.66122	-0.98973
C	-1.30873	2.20291	-0.71368
C	-2.29763	2.75574	0.31955
C	-0.32620	3.27855	-1.18705
H	-1.88815	1.86923	-1.58507
H	-0.86443	4.14253	-1.59141
H	0.29896	3.62555	-0.35724
H	0.33528	2.89489	-1.97264
H	-2.90418	3.55205	-0.12400
H	-2.97203	1.97484	0.68571
H	-1.77648	3.19045	1.18148
H	-0.30584	1.63757	1.76211
C	1.30287	0.53879	1.44379
O	1.54822	0.45395	2.64984
C	3.92011	-1.39402	-1.35614
C	3.49124	-0.08910	-1.60891
C	2.62084	0.54430	-0.72186
C	2.18451	-0.12243	0.43089
C	2.63854	-1.42055	0.69330
C	3.49226	-2.05927	-0.20389
H	4.59074	-1.88951	-2.05206
H	3.83517	0.43646	-2.49473
H	2.29774	1.56334	-0.90986

H	2.31031	-1.92095	1.59860
H	3.82816	-3.07229	-0.00327

4p_conformer-022

C	-1.98161	0.30177	-0.12346
C	-3.18539	0.93309	-0.37578
C	-4.21601	-0.05221	-0.30460
C	-3.62487	-1.26408	-0.00260
N	-2.27010	-1.03250	0.09313
H	-5.27552	0.10579	-0.45820
C	-4.21189	-2.62172	0.20931
H	-1.59353	-1.71291	0.40871
H	-3.82093	-3.35485	-0.50787
H	-4.00204	-3.00718	1.21551
H	-5.29766	-2.58001	0.08750
C	-0.56163	0.79337	-0.04051
H	-3.31672	1.98299	-0.59590
N	-0.01250	0.37300	1.26258
H	0.04700	0.29503	-0.80062
C	-0.43533	2.31859	-0.29249
C	0.92953	2.86226	0.14636
C	-0.66093	2.62534	-1.78123
H	-1.21228	2.82123	0.29902
H	0.14741	2.19022	-2.38258
H	-1.60720	2.22180	-2.14915
H	-0.65922	3.70694	-1.95375
H	1.00207	3.92739	-0.09831
H	1.09269	2.75396	1.22233
H	1.74457	2.34430	-0.37348
H	-0.50975	0.74047	2.06870
C	1.27770	0.03472	1.56359
O	1.71402	0.15301	2.71248
C	3.89584	-1.59126	-1.43309
C	2.57504	-2.03938	-1.36825
C	1.70145	-1.50588	-0.42064
C	2.14610	-0.52665	0.47725
C	3.48016	-0.10255	0.42529
C	4.34705	-0.62089	-0.53448
H	4.57279	-2.00066	-2.17707
H	2.22379	-2.80534	-2.05301
H	0.68006	-1.86861	-0.37102
H	3.82404	0.63860	1.13933
H	5.37510	-0.27381	-0.57792

4p_conformer-023

C	0.33126	-1.30090	-0.12318
C	-0.19417	-1.31053	-1.40042
C	-1.49373	-1.89257	-1.33070
C	-1.74027	-2.22484	-0.01176
N	-0.61722	-1.87199	0.70307
H	-2.18158	-2.04438	-2.15224
C	-2.93489	-2.83709	0.64346
H	-0.51966	-1.96778	1.70400
H	-3.70481	-3.04250	-0.10526
H	-2.69239	-3.78319	1.14446
H	-3.37158	-2.17112	1.39920
C	1.61556	-0.77223	0.44985
H	0.28758	-0.91657	-2.28381
N	1.35123	0.19201	1.54900
H	2.13326	-1.59371	0.96515
C	2.59806	-0.25476	-0.62933
C	3.72862	0.57280	-0.01021
C	3.17854	-1.43484	-1.42235
H	2.03765	0.38682	-1.31878
H	3.82453	-1.07198	-2.22911
H	3.78755	-2.07240	-0.76872
H	2.39546	-2.05618	-1.86432
H	4.42542	0.90200	-0.78861
H	3.34842	1.45836	0.50698
H	4.29641	-0.02214	0.71693
H	1.85027	0.01223	2.41239
C	0.29424	1.03422	1.73468
O	0.01088	1.45892	2.86017
C	-2.04103	2.40408	-1.59502
C	-0.65905	2.60561	-1.59229
C	0.11292	2.13517	-0.53035
C	-0.49586	1.46876	0.53867
C	-1.88493	1.30196	0.55100
C	-2.65253	1.75225	-0.52085
H	-2.64089	2.76021	-2.42763
H	-0.18208	3.12857	-2.41617
H	1.18515	2.29975	-0.52271
H	-2.35264	0.80151	1.39271
H	-3.72773	1.59844	-0.51764

4p_conformer-024

C	-1.81299	0.07322	-0.41514
C	-2.85585	0.08336	-1.32246
C	-3.77367	-0.93909	-0.93342

C	-3.27574	-1.54988	0.20243
N	-2.08129	-0.92736	0.49490
H	-4.70179	-1.19908	-1.42601
C	-3.81805	-2.65688	1.04699
H	-1.50381	-1.10871	1.30411
H	-4.76408	-3.01272	0.62975
H	-3.13047	-3.51115	1.09610
H	-4.00799	-2.33308	2.07870
C	-0.52979	0.85845	-0.34614
H	-2.94649	0.74253	-2.17523
N	0.02996	0.78981	1.01144
H	0.21187	0.39268	-1.00102
C	-0.68351	2.32231	-0.83208
C	-1.64825	3.14408	0.03013
C	0.68765	2.99919	-0.93632
H	-1.10680	2.25175	-1.84265
H	1.14517	3.11721	0.05274
H	1.37641	2.41573	-1.55826
H	0.59168	3.99633	-1.37879
H	-1.80289	4.13350	-0.41297
H	-2.62138	2.65195	0.11860
H	-1.24883	3.30468	1.03955
H	-0.43226	1.37313	1.70205
C	1.33244	0.53260	1.35345
O	1.80999	0.97040	2.40303
C	3.78395	-1.97858	-1.12275
C	4.31640	-0.84527	-0.50220
C	3.50316	-0.03818	0.29035
C	2.14466	-0.34111	0.44712
C	1.61619	-1.48285	-0.17173
C	2.43604	-2.30014	-0.94944
H	4.41904	-2.61190	-1.73519
H	5.36516	-0.59513	-0.63172
H	3.90780	0.83498	0.79150
H	0.57123	-1.74245	-0.03470
H	2.02206	-3.18802	-1.41781

4p_conformer-025

C	0.83493	0.70638	0.17258
C	0.89474	1.14190	1.48506
C	-0.05757	2.19251	1.63404
C	-0.68392	2.37530	0.41367
N	-0.11431	1.48063	-0.46166
H	-0.26809	2.75540	2.53426
C	-1.78596	3.29143	-0.00585

H	-0.46192	1.29703	-1.39258
H	-1.48146	3.96048	-0.82094
H	-2.66269	2.72887	-0.35304
H	-2.09786	3.91200	0.83870
C	1.50428	-0.43029	-0.55147
H	1.53369	0.73317	2.25548
N	1.27206	-1.72365	0.12721
H	1.05460	-0.48940	-1.54849
C	3.02553	-0.25012	-0.78743
C	3.26547	0.94042	-1.72156
C	3.84414	-0.09533	0.50001
H	3.35649	-1.16346	-1.30152
H	4.91231	-0.06896	0.26043
H	3.58740	0.83791	1.01054
H	3.68852	-0.91796	1.20755
H	4.33202	1.04126	-1.94812
H	2.72634	0.82225	-2.66847
H	2.92865	1.87243	-1.25369
H	2.07171	-2.14270	0.58814
C	0.08093	-2.12261	0.66381
O	0.01996	-2.96505	1.56261
C	-3.46543	-0.30092	-0.93756
C	-2.55407	-0.91186	-1.80304
C	-1.39973	-1.50699	-1.29387
C	-1.15671	-1.50276	0.08528
C	-2.09023	-0.92477	0.95131
C	-3.23270	-0.31189	0.44018
H	-4.35728	0.17491	-1.33472
H	-2.74101	-0.92348	-2.87281
H	-0.68874	-1.97873	-1.96565
H	-1.89745	-0.93031	2.01891
H	-3.94135	0.15850	1.11535

4p_conformer-026

C	-1.61363	-0.12072	0.20516
C	-1.91280	-0.62854	1.45440
C	-3.08573	-1.43355	1.32394
C	-3.47709	-1.40213	-0.00019
N	-2.57320	-0.59787	-0.66506
H	-3.59138	-1.97716	2.11135
C	-4.61971	-2.05525	-0.70742
H	-2.60325	-0.40721	-1.65640
H	-5.21114	-2.63872	0.00344
H	-5.28730	-1.32044	-1.17588
H	-4.27929	-2.73594	-1.49858

C	-0.49684	0.77691	-0.25384
H	-1.34756	-0.43455	2.35514
N	0.32850	1.17159	0.88594
H	0.15614	0.22524	-0.93732
C	-0.99554	2.02461	-1.03621
C	-1.92634	2.91479	-0.20523
C	0.18738	2.81505	-1.60458
H	-1.57533	1.63351	-1.88333
H	-0.16661	3.63327	-2.24062
H	0.79176	3.25167	-0.80168
H	0.84145	2.17393	-2.20704
H	-2.34040	3.71483	-0.82761
H	-2.75835	2.34133	0.21540
H	-1.39202	3.39792	0.62249
H	-0.03349	1.92260	1.46262
C	1.65950	0.94925	1.08660
O	2.30703	1.64612	1.87491
C	3.66216	-2.29798	-0.88607
C	2.35941	-2.48284	-0.41832
C	1.68095	-1.43209	0.19954
C	2.30928	-0.18915	0.36018
C	3.62542	-0.01723	-0.08741
C	4.29444	-1.06245	-0.72038
H	4.18596	-3.11544	-1.37300
H	1.87089	-3.44614	-0.53174
H	0.67115	-1.57960	0.57068
H	4.11106	0.94119	0.06445
H	5.30929	-0.91737	-1.07880

4p_conformer-027

C	-1.56973	0.14545	0.35723
C	-2.29709	0.08038	1.52951
C	-3.23147	-0.99085	1.39805
C	-3.05613	-1.55855	0.14969
N	-2.04305	-0.85547	-0.46711
H	-3.95009	-1.32042	2.13721
C	-3.73911	-2.70851	-0.51631
H	-1.68902	-1.05279	-1.39258
H	-3.03899	-3.51848	-0.75922
H	-4.50663	-3.11817	0.14602
H	-4.22860	-2.41025	-1.45254
C	-0.48318	1.08478	-0.08870
H	-2.16271	0.72589	2.38727
N	0.44898	1.37902	1.00706
H	0.09322	0.59707	-0.88184

C	-1.02101	2.42254	-0.66601
C	0.12717	3.28841	-1.19883
C	-2.06653	2.17662	-1.75902
H	-1.51440	2.95230	0.16250
H	-1.63390	1.61058	-2.59410
H	-2.92687	1.61873	-1.37948
H	-2.42694	3.13101	-2.15726
H	-0.25702	4.23795	-1.58649
H	0.86673	3.51364	-0.42470
H	0.64502	2.77563	-2.01948
H	0.22265	2.18512	1.57961
C	1.74497	0.97942	1.14522
O	2.52116	1.57905	1.89703
C	3.17205	-2.48233	-0.96013
C	1.90015	-2.50835	-0.38445
C	1.40980	-1.38436	0.28055
C	2.19588	-0.22858	0.38093
C	3.48217	-0.21771	-0.17356
C	3.96292	-1.33458	-0.85358
H	3.54933	-3.35526	-1.48491
H	1.28885	-3.40358	-0.45078
H	0.42572	-1.40844	0.73601
H	4.09307	0.67310	-0.06918
H	4.95475	-1.31360	-1.29549

4p_conformer-028

C	-1.61358	-0.12066	0.20516
C	-1.91288	-0.62826	1.45447
C	-3.08573	-1.43339	1.32402
C	-3.47691	-1.40227	-0.00017
N	-2.57301	-0.59806	-0.66508
H	-3.59144	-1.97688	2.11147
C	-4.61939	-2.05562	-0.70742
H	-2.60292	-0.40764	-1.65647
H	-5.21087	-2.63899	0.00349
H	-5.28698	-1.32096	-1.17613
H	-4.27881	-2.73645	-1.49838
C	-0.49684	0.77700	-0.25388
H	-1.34777	-0.43404	2.35524
N	0.32853	1.17174	0.88585
H	0.15613	0.22535	-0.93738
C	-0.99562	2.02467	-1.03624
C	-1.92639	2.91484	-0.20522
C	0.18724	2.81513	-1.60470
H	-1.57546	1.63354	-1.88331

H	-0.16680	3.63334	-2.24071
H	0.79167	3.25177	-0.80183
H	0.84129	2.17402	-2.20720
H	-2.34053	3.71485	-0.82758
H	-2.75834	2.34137	0.21550
H	-1.39202	3.39802	0.62245
H	-0.03342	1.92279	1.46250
C	1.65951	0.94933	1.08654
O	2.30708	1.64619	1.87482
C	3.66202	-2.29809	-0.88597
C	2.35927	-2.48288	-0.41818
C	1.68087	-1.43207	0.19963
C	2.30924	-0.18914	0.36017
C	3.62538	-0.01729	-0.08745
C	4.29435	-1.06258	-0.72037
H	4.18579	-3.11560	-1.37286
H	1.87072	-3.44618	-0.53153
H	0.67107	-1.57953	0.57080
H	4.11106	0.94112	0.06434
H	5.30919	-0.91755	-1.07882

4p_conformer-029

C	-1.41680	0.02739	-0.28180
C	-1.92357	0.18829	-1.55680
C	-2.71413	1.37725	-1.56050
C	-2.67305	1.91987	-0.29003
N	-1.88221	1.08597	0.47267
H	-3.25194	1.79688	-2.40069
C	-3.29988	3.15111	0.27860
H	-1.64963	1.24627	1.44251
H	-2.54992	3.85326	0.66542
H	-3.87273	3.66755	-0.49641
H	-3.98527	2.91981	1.10434
C	-0.52418	-1.02933	0.31156
H	-1.73158	-0.47121	-2.39207
N	0.36622	-1.60665	-0.69969
H	0.11474	-0.55606	1.06435
C	-1.29193	-2.17435	1.03507
C	-2.02992	-1.64144	2.26844
C	-2.26019	-2.92294	0.11180
H	-0.51823	-2.87523	1.37641
H	-3.02929	-2.24527	-0.27319
H	-1.75875	-3.38234	-0.74819
H	-2.75650	-3.72923	0.66200
H	-2.47243	-2.46945	2.83210

H	-1.35209	-1.10194	2.94070
H	-2.84043	-0.96239	1.98230
H	0.04607	-2.44857	-1.16311
C	1.69662	-1.37587	-0.88606
O	2.38828	-2.15406	-1.55215
C	3.57434	2.11529	0.77316
C	2.31734	2.23464	0.17736
C	1.67807	1.10890	-0.34298
C	2.30057	-0.14500	-0.27780
C	3.57472	-0.25403	0.29508
C	4.20267	0.86790	0.83131
H	4.06667	2.99150	1.18505
H	1.83276	3.20465	0.11509
H	0.70633	1.20451	-0.81510
H	4.06062	-1.22407	0.31788
H	5.18312	0.77244	1.28859

4p_conformer-030

C	1.57001	-0.14550	0.35720
C	2.29759	-0.08079	1.52937
C	3.23223	0.99023	1.39790
C	3.05681	1.55816	0.14966
N	2.04344	0.85544	-0.46707
H	3.95105	1.31950	2.13699
C	3.73998	2.70804	-0.51628
H	1.68934	1.05295	-1.39247
H	3.04006	3.51829	-0.75883
H	4.50781	3.11730	0.14592
H	4.22911	2.40981	-1.45271
C	0.48318	-1.08452	-0.08867
H	2.16321	-0.72640	2.38705
N	-0.44890	-1.37866	1.00720
H	-0.09325	-0.59659	-0.88166
C	1.02061	-2.42235	-0.66620
C	-0.12786	-3.28791	-1.19889
C	2.06597	-2.17657	-1.75940
H	1.51403	-2.95229	0.16216
H	1.63329	-1.61040	-2.59436
H	2.92650	-1.61888	-1.37998
H	2.42612	-3.13101	-2.15775
H	0.25603	-4.23750	-1.58670
H	-0.86732	-3.51304	-0.42463
H	-0.64576	-2.77494	-2.01940
H	-0.22243	-2.18465	1.57986
C	-1.74496	-0.97925	1.14532

O	-2.52107	-1.57896	1.89714
C	-3.17252	2.48232	-0.96000
C	-1.90052	2.50842	-0.38452
C	-1.41002	1.38448	0.28044
C	-2.19603	0.22867	0.38100
C	-3.48241	0.21772	-0.17329
C	-3.96332	1.33454	-0.85327
H	-3.54992	3.35522	-1.48475
H	-1.28928	3.40368	-0.45099
H	-0.42586	1.40862	0.73575
H	-4.09325	-0.67312	-0.06876
H	-4.95522	1.31349	-1.29502

4p_conformer-031

C	-1.94631	-0.12756	-0.17785
C	-2.87456	-1.07472	-0.57392
C	-4.13993	-0.42337	-0.67252
C	-3.96308	0.90461	-0.33121
N	-2.62641	1.06423	-0.03877
H	-5.08058	-0.87398	-0.96093
C	-4.93074	2.04055	-0.25477
H	-2.20023	1.93630	0.24161
H	-4.98611	2.46538	0.75584
H	-4.66145	2.85716	-0.93702
H	-5.93139	1.69406	-0.52692
C	-0.47421	-0.21628	0.10019
H	-2.67069	-2.11849	-0.77248
N	0.18811	-0.85551	-1.05442
H	-0.09262	0.80450	0.18261
C	-0.12105	-0.92850	1.43770
C	-0.73714	-0.17336	2.61897
C	-0.50906	-2.40900	1.45489
H	0.97178	-0.86225	1.52444
H	-1.59593	-2.53060	1.39878
H	-0.05844	-2.95500	0.61889
H	-0.16516	-2.87787	2.38317
H	-0.40705	-0.60966	3.56783
H	-0.44517	0.88387	2.61481
H	-1.83146	-0.22204	2.58704
H	-0.35441	-1.57798	-1.51490
C	1.53192	-1.01658	-1.24067
O	1.97902	-1.93550	-1.93380
C	4.27489	1.80801	0.47779
C	3.12633	2.27343	-0.16592
C	2.21365	1.36849	-0.70828

C	2.45153	-0.00918	-0.61865
C	3.62058	-0.46949	0.00008
C	4.52131	0.43477	0.55940
H	4.98002	2.51313	0.90800
H	2.94181	3.34032	-0.24893
H	1.33131	1.73218	-1.22573
H	3.80937	-1.53744	0.04268
H	5.41758	0.07040	1.05260

4p_conformer-032

C	1.33776	0.96994	-0.22553
C	1.77246	0.47395	-1.43871
C	2.94977	-0.29520	-1.19509
C	3.21687	-0.24779	0.16041
N	2.23848	0.53998	0.72907
H	3.54101	-0.82612	-1.92963
C	4.30834	-0.86522	0.97270
H	2.14190	0.72077	1.71878
H	4.96382	-1.45479	0.32607
H	4.92436	-0.10863	1.47555
H	3.91420	-1.53285	1.74950
C	0.19559	1.85243	0.17750
H	1.29862	0.63668	-2.39602
N	-0.59195	1.30911	1.31818
H	0.64112	2.75122	0.62442
C	-0.65925	2.37016	-1.00647
C	-1.50854	1.30867	-1.71555
C	-1.54606	3.53647	-0.55265
H	0.07177	2.76118	-1.72656
H	-0.95489	4.34060	-0.09903
H	-2.09277	3.95683	-1.40350
H	-2.28641	3.20113	0.18348
H	-0.94206	0.40488	-1.94935
H	-2.36587	1.01628	-1.10202
H	-1.90361	1.72041	-2.65100
H	-0.86754	2.03121	1.97536
C	-1.48118	0.26440	1.42138
O	-2.39059	0.33228	2.25629
C	-1.28565	-3.36453	-0.87433
C	-0.10866	-2.83440	-0.34614
C	-0.13493	-1.64183	0.37922
C	-1.34614	-0.97547	0.58985
C	-2.53273	-1.53656	0.09346
C	-2.50241	-2.71240	-0.65009
H	-1.25938	-4.28546	-1.44982

H	0.83822	-3.34379	-0.49883
H	0.78344	-1.25079	0.79363
H	-3.47358	-1.03168	0.28686
H	-3.42476	-3.12467	-1.04869

4p_conformer-033

C	1.34501	0.80977	-0.16079
C	1.68547	0.22171	-1.36371
C	2.84793	-0.57505	-1.14345
C	3.19642	-0.45947	0.18971
N	2.28142	0.39626	0.76485
H	3.37541	-1.16973	-1.87778
C	4.31758	-1.06279	0.97171
H	2.23610	0.61852	1.74952
H	3.95366	-1.64135	1.83035
H	4.89374	-1.73815	0.33341
H	5.00518	-0.30066	1.36085
C	0.20592	1.69037	0.27163
H	1.14418	0.32247	-2.29332
N	-0.44429	1.16017	1.50053
H	0.62036	2.64665	0.61837
C	-0.77916	2.02947	-0.87587
C	-2.06768	2.67527	-0.35166
C	-0.10852	2.97695	-1.88342
H	-1.04122	1.09424	-1.38517
H	0.83577	2.58123	-2.26337
H	-0.77233	3.15740	-2.73577
H	0.09891	3.94586	-1.41154
H	-2.64853	2.00465	0.28688
H	-1.84268	3.57947	0.22798
H	-2.70512	2.96849	-1.19263
H	-0.57727	1.85117	2.23086
C	-1.38243	0.16798	1.60664
O	-2.17869	0.15835	2.55053
C	-1.65896	-3.02329	-1.25295
C	-0.43514	-2.76407	-0.63481
C	-0.31315	-1.70881	0.27017
C	-1.42673	-0.91886	0.57554
C	-2.66335	-1.20455	-0.01986
C	-2.77527	-2.23957	-0.94545
H	-1.74635	-3.83658	-1.96774
H	0.43293	-3.37554	-0.86232
H	0.64044	-1.51390	0.74534
H	-3.52961	-0.60497	0.24220
H	-3.73168	-2.44141	-1.41877

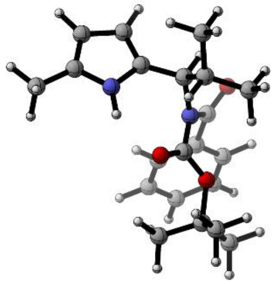

4q_conformer-001

C	-2.57340	-0.57115	-0.32656
C	-3.37850	-1.61085	-0.75418
C	-4.02614	-2.15254	0.39544
C	-3.59662	-1.43436	1.49864
N	-2.72768	-0.47211	1.04021
H	-4.72796	-2.97617	0.42105
C	-3.92334	-1.57535	2.95031
H	-2.16932	0.16330	1.59912
H	-4.63773	-2.39059	3.09467
H	-3.03258	-1.80002	3.55153
H	-4.36949	-0.66068	3.36160
C	-1.70099	0.35082	-1.11848
H	-3.49195	-1.93190	-1.78163
N	-0.25972	0.20439	-0.73172
H	-1.71894	-0.00309	-2.15009
C	-2.15401	1.83100	-1.14598
C	-1.17755	2.68933	-1.96037
C	-3.56992	1.91924	-1.72755
H	-2.18237	2.20751	-0.11843
H	-3.91122	2.95989	-1.73771
H	-3.59185	1.54775	-2.76007
H	-4.28150	1.32910	-1.14304
H	-1.54206	3.72000	-2.02639
H	-0.17771	2.71879	-1.51611
H	-1.07674	2.29872	-2.98030
C	0.58808	-0.48700	-1.62169
O	0.32006	-0.56069	-2.81344
C	3.91928	-2.66468	-0.04441
C	2.73205	-2.59506	0.68932
C	1.65009	-1.86758	0.19775
C	1.74826	-1.21890	-1.03969
C	2.92528	-1.31970	-1.78957
C	4.01405	-2.02723	-1.28486
H	4.76621	-3.22100	0.34597
H	2.65001	-3.10505	1.64430
H	0.72721	-1.81078	0.76671
H	2.98179	-0.82701	-2.75461

H	4.93417	-2.08547	-1.85830
C	0.21859	0.88808	0.38747
O	-0.49436	1.22228	1.32468
O	1.50894	1.18174	0.24921
C	2.31952	1.73425	1.36343
C	3.73085	1.68005	0.78327
C	1.87261	3.17083	1.62424
C	2.20634	0.84587	2.60117
H	4.00923	0.64807	0.54940
H	3.79368	2.27551	-0.13276
H	4.44384	2.08174	1.50948
H	0.85146	3.19902	2.00826
H	2.53915	3.63060	2.36119
H	1.92331	3.75790	0.70152
H	1.19760	0.86079	3.01572
H	2.48001	-0.18445	2.35736
H	2.90133	1.21453	3.36237

4q_conformer-002

C	2.64661	0.23256	-0.27151
C	3.48724	1.25239	-0.67641
C	4.28268	1.62321	0.44861
C	3.90330	0.82553	1.51505
N	2.92005	-0.01961	1.05623
H	5.04761	2.38828	0.48246
C	4.37841	0.78913	2.93172
H	2.36462	-0.66766	1.60389
H	4.79257	-0.19108	3.20105
H	5.16247	1.53651	3.08165
H	3.56925	1.00519	3.64158
C	1.62117	-0.53976	-1.04016
H	3.52627	1.67178	-1.67336
N	0.24140	-0.30799	-0.49922
H	1.57475	-0.10097	-2.03815
C	1.94020	-2.04272	-1.22118
C	0.82094	-2.77053	-1.97514
C	3.27228	-2.18167	-1.97003
H	2.05479	-2.50137	-0.23424
H	3.53171	-3.23935	-2.08521
H	3.20477	-1.74003	-2.97246
H	4.08693	-1.68304	-1.43731
H	1.12176	-3.80048	-2.19496
H	-0.10949	-2.81614	-1.40203
H	0.60443	-2.27239	-2.92866
C	-0.22624	-1.04504	0.59700

O	0.55367	-1.55753	1.39848
C	-4.41794	-1.82799	0.98429
C	-3.86209	-1.68769	-0.29052
C	-2.50416	-1.41324	-0.43248
C	-1.69134	-1.29082	0.70248
C	-2.24487	-1.46252	1.97657
C	-3.60737	-1.71618	2.11729
H	-5.47909	-2.03077	1.09379
H	-4.48656	-1.79049	-1.17261
H	-2.07299	-1.30121	-1.42176
H	-1.60238	-1.37820	2.84650
H	-4.03708	-1.82879	3.10793
C	-0.39255	0.86455	-0.95084
O	-0.09956	1.40394	-2.00238
O	-1.27311	1.29895	-0.05084
C	-2.23414	2.38687	-0.34890
C	-3.03516	2.05768	-1.60763
C	-3.13096	2.36502	0.88705
C	-1.46844	3.70312	-0.46408
H	-2.39944	2.04350	-2.49403
H	-3.52775	1.08670	-1.50237
H	-3.80805	2.82065	-1.74491
H	-3.62873	1.39526	0.98225
H	-2.54318	2.54678	1.79213
H	-3.89300	3.14579	0.80397
H	-0.86937	3.87371	0.43620
H	-0.80909	3.69702	-1.33364
H	-2.17939	4.52957	-0.56590

4q_conformer-003

C	-2.58225	-0.30122	0.59128
C	-3.18257	-0.22223	1.83432
C	-4.16932	0.80454	1.77411
C	-4.15093	1.33197	0.49437
N	-3.19469	0.64113	-0.21243
H	-4.82716	1.12464	2.57165
C	-4.94825	2.43317	-0.12629
H	-2.87630	0.86102	-1.14647
H	-4.30992	3.25666	-0.47207
H	-5.52562	2.08433	-0.99208
H	-5.65257	2.84056	0.60418
C	-1.52113	-1.23948	0.11277
H	-2.94157	-0.84881	2.68339
N	-0.19346	-0.55657	-0.03938
H	-1.34424	-1.93979	0.93018

C	-1.90561	-2.06545	-1.14113
C	-0.73793	-2.93569	-1.62015
C	-3.13461	-2.92624	-0.82524
H	-2.16335	-1.36726	-1.94314
H	-3.43403	-3.50184	-1.70773
H	-2.91476	-3.63752	-0.01870
H	-3.98499	-2.31368	-0.51112
H	-1.04388	-3.53911	-2.48144
H	0.12155	-2.33101	-1.92646
H	-0.40719	-3.62111	-0.83017
C	-0.07462	0.55939	-0.87624
O	-0.85786	0.73738	-1.80637
C	2.79677	3.59517	0.04957
C	2.15656	2.91269	1.08777
C	1.23911	1.90525	0.79803
C	0.94859	1.58627	-0.53452
C	1.56303	2.29542	-1.57240
C	2.49755	3.28656	-1.28049
H	3.52112	4.37166	0.27673
H	2.37309	3.16458	2.12146
H	0.73890	1.37348	1.60128
H	1.31498	2.05049	-2.59989
H	2.98996	3.82050	-2.08751
C	0.91585	-1.21502	0.52030
O	0.81364	-2.03266	1.41777
O	2.03444	-0.86777	-0.11450
C	3.37577	-1.25286	0.38414
C	3.55284	-0.80081	1.83271
C	3.55193	-2.75960	0.20902
C	4.29735	-0.46785	-0.54672
H	3.36709	0.27353	1.92094
H	2.88095	-1.33743	2.50399
H	4.58457	-0.99747	2.14134
H	2.86892	-3.31107	0.85721
H	3.36461	-3.04500	-0.83124
H	4.58035	-3.03737	0.46199
H	4.12788	-0.75627	-1.58876
H	4.11660	0.60670	-0.44786
H	5.34152	-0.67489	-0.29358

4q_conformer-004

C	2.54053	-0.55916	-0.55742
C	3.20819	-0.72532	-1.75611
C	4.12341	0.35925	-1.89539
C	3.99239	1.16779	-0.77973

N	3.03918	0.59013	0.02659
H	4.80654	0.53470	-2.71630
C	4.67732	2.44082	-0.40080
H	2.66027	0.99190	0.87374
H	3.96571	3.26844	-0.28241
H	5.22608	2.34674	0.54512
H	5.39345	2.72437	-1.17691
C	1.48718	-1.41249	0.07611
H	3.05189	-1.54344	-2.44710
N	0.12771	-0.77851	-0.00435
H	1.38517	-2.29189	-0.55996
C	1.83243	-1.90703	1.50246
C	0.65363	-2.64635	2.14230
C	3.07065	-2.80970	1.42786
H	2.07182	-1.04025	2.12460
H	3.36340	-3.13782	2.43094
H	2.86521	-3.70508	0.82718
H	3.92059	-2.28938	0.97593
H	0.95393	-3.08250	3.10124
H	-0.18730	-1.97335	2.33102
H	0.30040	-3.46206	1.49864
C	-0.79102	-1.33654	-0.91511
O	-0.40963	-2.07580	-1.81384
C	-4.99838	-0.74189	-0.36014
C	-4.17431	-0.89670	0.75806
C	-2.80126	-1.06576	0.59494
C	-2.24649	-1.09506	-0.69050
C	-3.07817	-0.97566	-1.80931
C	-4.44826	-0.78292	-1.64400
H	-6.06692	-0.59765	-0.23111
H	-4.60093	-0.88380	1.75641
H	-2.16307	-1.18307	1.46443
H	-2.63967	-1.01788	-2.80080
H	-5.08688	-0.66843	-2.51459
C	-0.04388	0.47534	0.59289
O	0.62839	0.84519	1.54504
O	-0.95962	1.19608	-0.04552
C	-1.45823	2.48929	0.48240
C	-2.59009	2.81251	-0.49039
C	-1.99459	2.31080	1.90165
C	-0.33508	3.52101	0.39906
H	-2.20865	2.88756	-1.51336
H	-3.35656	2.03243	-0.45931
H	-3.04789	3.76829	-0.21830
H	-1.19846	2.05216	2.60109
H	-2.45302	3.25029	2.22658

H	-2.76067	1.53037	1.92327
H	0.47765	3.27286	1.08358
H	0.06019	3.57008	-0.62067
H	-0.72940	4.50772	0.66298

4q_conformer-005

C	-2.62329	-0.18595	0.14442
C	-3.50430	0.45909	-0.70290
C	-4.12820	1.50311	0.04359
C	-3.60794	1.47921	1.32635
N	-2.70321	0.44453	1.37031
H	-4.87908	2.19659	-0.31243
C	-3.88827	2.33814	2.51672
H	-2.10359	0.19668	2.14873
H	-4.28041	1.75609	3.36075
H	-2.98664	2.85506	2.87021
H	-4.63104	3.09877	2.26090
C	-1.63456	-1.29375	-0.05011
H	-3.68162	0.20670	-1.73915
N	-0.23700	-0.74078	-0.01566
H	-1.65873	-1.93805	0.83245
C	-1.91230	-2.19628	-1.26998
C	-0.72472	-3.11629	-1.56999
C	-3.18035	-3.02004	-1.00162
H	-2.08234	-1.56096	-2.14242
H	-3.43120	-3.62615	-1.87884
H	-3.02999	-3.70201	-0.15485
H	-4.03612	-2.37853	-0.77286
H	-0.98920	-3.82739	-2.36030
H	0.14534	-2.54738	-1.90777
H	-0.43604	-3.69344	-0.68240
C	0.43511	-0.79521	1.21939
O	-0.19084	-0.91754	2.27018
C	4.71406	-0.83320	1.26743
C	4.03486	-1.40787	0.18936
C	2.64264	-1.38942	0.15263
C	1.92260	-0.80469	1.20255
C	2.60435	-0.25910	2.29666
C	3.99705	-0.26099	2.32197
H	5.79976	-0.83815	1.28902
H	4.58992	-1.86917	-0.62172
H	2.11347	-1.83753	-0.68160
H	2.03393	0.17828	3.10920
H	4.52360	0.18118	3.16222
C	0.13051	0.16681	-1.02230

O	-0.33533	0.13169	-2.14566
O	0.99135	1.07037	-0.54864
C	1.72538	1.99723	-1.43962
C	2.70514	2.66021	-0.47356
C	2.47229	1.21259	-2.51733
C	0.74035	3.00968	-2.02056
H	2.16601	3.17230	0.32950
H	3.36909	1.91353	-0.02766
H	3.31173	3.39616	-1.00999
H	1.78207	0.71545	-3.20040
H	3.09542	1.90432	-3.09319
H	3.12490	0.46426	-2.05792
H	0.02762	2.52260	-2.68788
H	0.19051	3.50807	-1.21563
H	1.29022	3.77055	-2.58417

4q_conformer-006

C	1.97640	-0.99827	0.47025
C	3.20128	-0.42217	0.19069
C	3.84764	-1.24218	-0.78282
C	3.00137	-2.29674	-1.08072
N	1.87589	-2.13465	-0.30700
H	4.82604	-1.08460	-1.21783
C	3.15660	-3.44571	-2.02345
H	1.05093	-2.72319	-0.30755
H	4.11852	-3.37731	-2.53891
H	3.12213	-4.41176	-1.50340
H	2.36716	-3.45914	-2.78604
C	0.82245	-0.59905	1.33762
H	3.59033	0.48184	0.63786
N	-0.36979	-0.25344	0.48529
H	0.47388	-1.48528	1.87383
C	1.16214	0.45717	2.41082
C	-0.10169	1.05875	3.03263
C	2.03815	-0.19660	3.49001
H	1.73164	1.26383	1.94386
H	2.35278	0.55032	4.22683
H	1.48278	-0.97952	4.02202
H	2.93533	-0.65028	3.05891
H	0.16578	1.71441	3.86850
H	-0.66230	1.65229	2.30576
H	-0.76511	0.27500	3.41945
C	-1.33062	-1.27041	0.31094
O	-1.02831	-2.44976	0.47852
C	-5.41900	-0.25156	-0.42519

C	-4.59485	0.66099	0.23932
C	-3.25386	0.35817	0.45941
C	-2.73157	-0.86753	0.02325
C	-3.56631	-1.78898	-0.62252
C	-4.90304	-1.47662	-0.85653
H	-6.46303	-0.01020	-0.60186
H	-4.99842	1.60689	0.58742
H	-2.61705	1.06064	0.98571
H	-3.15308	-2.73997	-0.94116
H	-5.54314	-2.18725	-1.37045
C	-0.29567	0.75706	-0.48727
O	-0.94679	0.74636	-1.51704
O	0.58062	1.68755	-0.11422
C	1.01969	2.75038	-1.04709
C	-0.15874	3.67220	-1.35588
C	2.08638	3.47783	-0.23182
C	1.62841	2.11756	-2.29808
H	-0.93143	3.14714	-1.91856
H	-0.59255	4.05618	-0.42660
H	0.19358	4.52377	-1.94711
H	2.91151	2.80190	0.01075
H	2.48246	4.31879	-0.80894
H	1.66248	3.86403	0.70035
H	2.40252	1.39701	-2.01596
H	0.87052	1.60985	-2.89640
H	2.08771	2.90227	-2.90796

4q_conformer-007

C	-2.13598	-0.68650	0.20659
C	-3.31105	0.02962	0.07128
C	-4.08192	-0.60336	-0.94955
C	-3.35553	-1.68532	-1.41761
N	-2.18453	-1.72643	-0.69672
H	-5.05898	-0.30560	-1.30756
C	-3.66060	-2.68400	-2.48685
H	-1.39444	-2.34422	-0.84713
H	-4.63823	-2.47111	-2.92804
H	-2.91641	-2.66126	-3.29361
H	-3.68414	-3.70955	-2.09631
C	-0.96732	-0.47090	1.11507
H	-3.58297	0.90056	0.65329
N	0.27578	-0.15243	0.32980
H	-1.16588	0.44123	1.67625
C	-0.73413	-1.58573	2.16241
C	0.50238	-1.29807	3.02243

C	-1.98481	-1.70654	3.04327
H	-0.58954	-2.53819	1.64454
H	-1.85156	-2.50651	3.77913
H	-2.17026	-0.77322	3.59003
H	-2.87501	-1.93034	2.44863
H	0.58637	-2.04112	3.82272
H	1.43173	-1.32851	2.44639
H	0.42995	-0.30754	3.48924
C	1.07405	-1.19359	-0.17533
O	0.60178	-2.30754	-0.39288
C	5.29683	-0.62715	-0.56732
C	4.58283	0.15667	0.34327
C	3.20365	0.00699	0.46114
C	2.53094	-0.93824	-0.32677
C	3.25345	-1.73614	-1.22354
C	4.63043	-1.57299	-1.35120
H	6.37166	-0.50426	-0.66223
H	5.10136	0.88248	0.96209
H	2.65064	0.60765	1.17529
H	2.72315	-2.47290	-1.81728
H	5.18466	-2.18372	-2.05732
C	0.39218	1.11639	-0.26343
O	1.04529	1.32519	-1.27030
O	-0.31397	2.01563	0.42322
C	-0.60002	3.35849	-0.13356
C	-1.30995	3.21073	-1.47894
C	-1.53889	3.94861	0.91602
C	0.69727	4.15929	-0.22640
H	-0.64102	2.80756	-2.24071
H	-2.17399	2.54623	-1.37624
H	-1.66354	4.19302	-1.80811
H	-1.04943	3.98477	1.89422
H	-1.82045	4.96639	0.62992
H	-2.44849	3.34594	1.00093
H	1.19424	4.19033	0.74880
H	1.37715	3.72139	-0.95806
H	0.46705	5.18682	-0.52649

4q_conformer-008

C	2.71539	0.28214	-0.43167
C	3.65238	1.21327	-0.84316
C	4.52356	1.46117	0.25821
C	4.09213	0.68321	1.31958
N	3.00344	-0.03121	0.87924
H	5.36793	2.13802	0.28259

C	4.60768	0.55502	2.71670
H	2.41462	-0.65563	1.42039
H	5.47592	1.20530	2.85521
H	3.85282	0.84072	3.46091
H	4.91756	-0.47243	2.94770
C	1.59812	-0.33712	-1.21384
H	3.70105	1.65828	-1.82881
N	0.26049	-0.17342	-0.55536
H	1.48440	0.28422	-2.10185
C	1.86971	-1.76448	-1.77259
C	2.41663	-2.82073	-0.80342
C	0.65338	-2.29432	-2.54161
H	2.66774	-1.57618	-2.50377
H	0.93272	-3.17065	-3.13623
H	-0.14675	-2.60276	-1.86006
H	0.24454	-1.53749	-3.22109
H	2.76515	-3.68638	-1.37852
H	3.26386	-2.43564	-0.22884
H	1.65851	-3.16720	-0.09834
C	-0.15686	-0.99435	0.50439
O	0.65243	-1.51053	1.27186
C	-4.30438	-1.98100	0.90593
C	-3.78721	-1.70259	-0.36228
C	-2.44490	-1.36139	-0.50856
C	-1.60797	-1.30986	0.61447
C	-2.12283	-1.61766	1.87969
C	-3.47006	-1.93897	2.02647
H	-5.35370	-2.23616	1.01960
H	-4.42962	-1.74963	-1.23615
H	-2.04539	-1.14239	-1.49280
H	-1.46225	-1.58539	2.73938
H	-3.86965	-2.15810	3.01185
C	-0.43121	1.00872	-0.88300
O	-0.22510	1.63312	-1.90817
O	-1.26341	1.34869	0.10037
C	-2.27161	2.42432	-0.05457
C	-3.14236	2.16471	-1.28283
C	-3.08643	2.28179	1.22916
C	-1.55038	3.76890	-0.11607
H	-2.56462	2.23684	-2.20533
H	-3.60102	1.17365	-1.22195
H	-3.94254	2.91113	-1.31323
H	-2.44726	2.41494	2.10745
H	-3.87359	3.04140	1.25308
H	-3.55006	1.29199	1.28093
H	-0.90104	3.89177	0.75670

H	-0.94693	3.84741	-1.02195
H	-2.28821	4.57784	-0.11247

4q_conformer-009

C	-2.42918	0.25032	0.23115
C	-3.13499	1.12759	-0.56917
C	-3.25394	2.35795	0.14540
C	-2.60966	2.21043	1.36092
N	-2.11978	0.92375	1.39790
H	-3.76100	3.25457	-0.18644
C	-2.41802	3.16321	2.49581
H	-1.55201	0.52192	2.13389
H	-2.87265	4.12745	2.25304
H	-2.87897	2.79747	3.42229
H	-1.35507	3.33704	2.70787
C	-1.84800	-1.10963	-0.00123
H	-3.52000	0.90952	-1.55600
N	-0.38395	-0.94051	-0.29723
H	-1.84795	-1.65402	0.94587
C	-2.60068	-1.98197	-1.02227
C	-1.83387	-3.27715	-1.30613
C	-4.00483	-2.29005	-0.48260
H	-2.69714	-1.42477	-1.95807
H	-4.56313	-2.89400	-1.20601
H	-3.94593	-2.85925	0.45407
H	-4.57256	-1.37586	-0.28830
H	-2.40798	-3.91510	-1.98707
H	-0.86460	-3.07134	-1.76646
H	-1.66438	-3.84268	-0.38078
C	0.01692	-0.31443	-1.49998
O	-0.50810	-0.60111	-2.56512
C	2.80053	2.93417	-1.34719
C	1.83241	2.80478	-0.34863
C	0.94652	1.72845	-0.36981
C	1.01597	0.78897	-1.40614
C	1.96147	0.94070	-2.42675
C	2.86440	2.00051	-2.38636
H	3.49972	3.76463	-1.32071
H	1.76879	3.53928	0.44853
H	0.19372	1.62700	0.40526
H	1.99053	0.21647	-3.23438
H	3.61341	2.10363	-3.16562
C	0.49489	-1.11829	0.77124
O	0.12527	-1.19424	1.93583
O	1.74424	-1.24876	0.32564

C	2.90921	-1.26497	1.24244
C	2.90589	-0.02671	2.13756
C	2.88769	-2.56772	2.03920
C	4.08235	-1.22469	0.26576
H	2.87150	0.88257	1.53129
H	2.05764	-0.03119	2.82339
H	3.82947	-0.01410	2.72530
H	2.84193	-3.42660	1.36185
H	3.80443	-2.64656	2.63267
H	2.02933	-2.59953	2.71245
H	4.05492	-2.08872	-0.40541
H	4.04658	-0.31224	-0.33722
H	5.02558	-1.24408	0.81992

4q_conformer-010

C	-2.61507	-0.40627	-0.15830
C	-3.26601	-0.80762	-1.30890
C	-4.11080	0.26495	-1.72158
C	-3.95358	1.30358	-0.81995
N	-3.04914	0.87583	0.12439
H	-4.76840	0.28003	-2.58119
C	-4.57963	2.65912	-0.75924
H	-2.68458	1.43203	0.88658
H	-5.16456	2.80227	0.15861
H	-3.82845	3.45900	-0.79341
H	-5.25304	2.79667	-1.60970
C	-1.56928	-1.04645	0.70429
H	-3.13785	-1.76182	-1.80024
N	-0.20077	-0.55040	0.33129
H	-1.68873	-0.66835	1.72283
C	-1.65533	-2.58258	0.79338
C	-0.48158	-3.16840	1.58832
C	-2.98603	-2.97168	1.45407
H	-1.63567	-2.99840	-0.21778
H	-3.07996	-4.06196	1.49828
H	-3.03809	-2.58877	2.48129
H	-3.84064	-2.57526	0.89956
H	-0.62993	-4.24321	1.73756
H	0.47891	-3.03783	1.08216
H	-0.40664	-2.69883	2.57728
C	0.50904	-1.17029	-0.71942
O	-0.07915	-1.79676	-1.58938
C	4.79305	-1.14689	-0.63367
C	4.08316	-1.13388	0.57058
C	2.69059	-1.12174	0.55868

C	1.99930	-1.13529	-0.66012
C	2.71252	-1.18030	-1.86314
C	4.10575	-1.17154	-1.85016
H	5.87896	-1.14464	-0.62337
H	4.61473	-1.13141	1.51731
H	2.13823	-1.10809	1.49246
H	2.16568	-1.20443	-2.79985
H	4.65555	-1.18586	-2.78634
C	0.10625	0.73457	0.79247
O	-0.48383	1.25354	1.72980
O	1.04674	1.30464	0.04341
C	1.71447	2.56709	0.43599
C	2.80255	2.69437	-0.62825
C	0.70860	3.71298	0.34778
C	2.32778	2.42166	1.82756
H	3.48027	1.83642	-0.58801
H	2.35913	2.74281	-1.62759
H	3.37950	3.60844	-0.45842
H	0.24714	3.73650	-0.64476
H	-0.07384	3.60724	1.10072
H	1.22730	4.66379	0.50824
H	3.00294	1.56104	1.85631
H	1.55858	2.30007	2.59177
H	2.90823	3.32094	2.05694

4q_conformer-011

C	0.37655	2.02084	0.01821
C	1.31667	2.76131	-0.67714
C	0.63022	3.82686	-1.32924
C	-0.71420	3.71401	-1.01865
N	-0.84698	2.62425	-0.19076
H	1.06431	4.59438	-1.95669
C	-1.89162	4.53323	-1.43846
H	-1.71446	2.22619	0.14658
H	-1.56302	5.36073	-2.07318
H	-2.62010	3.94208	-2.00863
H	-2.42229	4.95942	-0.57749
C	0.57567	0.84458	0.92152
H	2.37949	2.55861	-0.69683
N	-0.01782	-0.42585	0.37705
H	1.64976	0.65226	0.92950
C	0.16767	1.09346	2.39892
C	0.37648	-0.16286	3.25139
C	0.97628	2.27068	2.95748
H	-0.89318	1.35659	2.42043

H	0.69271	2.46482	3.99750
H	2.05155	2.05044	2.93840
H	0.81035	3.18472	2.38016
H	0.13296	0.04632	4.29856
H	-0.25056	-0.99482	2.91887
H	1.42279	-0.49382	3.21235
C	0.79299	-1.59782	0.33936
O	0.40393	-2.66398	0.78660
C	4.66640	-1.31090	-1.45660
C	3.64226	-0.46991	-1.89901
C	2.38296	-0.53235	-1.30584
C	2.14202	-1.44612	-0.26993
C	3.16737	-2.30118	0.15843
C	4.42818	-2.22615	-0.42654
H	5.64935	-1.25530	-1.91502
H	3.82373	0.23394	-2.70550
H	1.58757	0.12380	-1.64090
H	2.96250	-3.01132	0.95260
H	5.22462	-2.87925	-0.08323
C	-1.39859	-0.52109	0.23409
O	-2.17815	0.31892	0.66881
O	-1.73325	-1.58458	-0.49364
C	-3.14003	-2.02565	-0.61868
C	-3.71776	-2.29032	0.77110
C	-3.93856	-0.98877	-1.40759
C	-2.99954	-3.32571	-1.40777
H	-3.06783	-2.97642	1.32388
H	-3.82534	-1.36602	1.34077
H	-4.70348	-2.75551	0.67005
H	-4.03691	-0.05744	-0.84894
H	-3.44755	-0.78089	-2.36390
H	-4.93817	-1.38473	-1.61515
H	-2.53554	-3.13692	-2.38085
H	-2.38164	-4.04307	-0.85917
H	-3.98682	-3.76739	-1.57275

4q_conformer-012

C	2.64952	-0.60779	0.46173
C	3.57664	-1.55598	0.85747
C	4.32287	-1.93576	-0.29662
C	3.82778	-1.21809	-1.37225
N	2.82379	-0.41153	-0.89152
H	5.12722	-2.65851	-0.34457
C	4.20717	-1.22735	-2.81801
H	2.21592	0.19856	-1.42746

H	4.53879	-0.23912	-3.16218
H	5.02683	-1.93188	-2.98431
H	3.36974	-1.52984	-3.46032
C	1.65837	0.13256	1.30651
H	3.70122	-1.92526	1.86748
N	0.25573	0.02515	0.78961
H	1.59514	-0.42592	2.23983
C	2.06804	1.57092	1.74372
C	2.56969	2.52140	0.64900
C	0.95498	2.21826	2.57811
H	2.92163	1.38537	2.41003
H	1.31745	3.13934	3.04689
H	0.09251	2.48095	1.95461
H	0.60253	1.54368	3.36518
H	3.01546	3.40604	1.11817
H	3.33518	2.04758	0.02758
H	1.76391	2.85889	-0.00631
C	-0.65172	-0.74980	1.54115
O	-0.46431	-0.97201	2.73004
C	-3.89040	-2.67733	-0.49048
C	-2.65338	-2.55402	-1.12761
C	-1.60024	-1.90346	-0.48669
C	-1.77896	-1.38821	0.80324
C	-3.00822	-1.54389	1.45385
C	-4.06641	-2.17193	0.80127
H	-4.71384	-3.17286	-0.99598
H	-2.50917	-2.96173	-2.12346
H	-0.63862	-1.80474	-0.98087
H	-3.12725	-1.15593	2.46012
H	-5.02603	-2.27223	1.29914
C	-0.17742	0.81783	-0.27979
O	0.54556	1.16692	-1.20203
O	-1.44622	1.17865	-0.11058
C	-2.21986	1.88327	-1.16556
C	-3.62615	1.89004	-0.57120
C	-1.66520	3.29834	-1.31247
C	-2.18551	1.09618	-2.47410
H	-3.98065	0.86661	-0.41571
H	-3.63585	2.41368	0.38973
H	-4.31228	2.40085	-1.25326
H	-0.65366	3.28373	-1.72179
H	-2.30911	3.87091	-1.98792
H	-1.65043	3.80370	-0.34139
H	-1.18060	1.06339	-2.89696
H	-2.54239	0.07488	-2.31593
H	-2.84999	1.58497	-3.19387

4q_conformer-013

C	-2.58952	0.02464	-0.17189
C	-3.07748	-0.33243	-1.41465
C	-3.83022	0.76873	-1.91661
C	-3.78548	1.78172	-0.97440
N	-3.02931	1.31271	0.07542
H	-4.35108	0.82213	-2.86394
C	-4.39419	3.14631	-0.96596
H	-2.83657	1.83219	0.92058
H	-4.94453	3.31301	-1.89598
H	-5.09631	3.27904	-0.13257
H	-3.63477	3.93426	-0.87969
C	-1.72038	-0.67790	0.82524
H	-2.89109	-1.27454	-1.90910
N	-0.27565	-0.35472	0.58813
H	-1.90260	-0.24104	1.80995
C	-1.98307	-2.19255	0.96228
C	-0.96806	-2.84297	1.91124
C	-3.41350	-2.41887	1.46846
H	-1.88720	-2.65415	-0.02510
H	-3.62358	-3.49102	1.54657
H	-3.54902	-1.97752	2.46423
H	-4.15090	-1.97283	0.79531
H	-1.21091	-3.90093	2.05683
H	0.05616	-2.78698	1.52997
H	-0.98489	-2.35237	2.89210
C	0.35447	0.52097	1.48864
O	-0.06149	0.65185	2.63442
C	3.49863	3.06692	0.08542
C	2.42228	2.77223	-0.75591
C	1.40788	1.92293	-0.31899
C	1.45991	1.37629	0.96931
C	2.51892	1.70202	1.82337
C	3.54482	2.53192	1.37593
H	4.29538	3.71845	-0.26097
H	2.37471	3.20176	-1.75200
H	0.57167	1.69029	-0.97115
H	2.53687	1.28743	2.82596
H	4.37739	2.76517	2.03275
C	0.40507	-1.03733	-0.42778
O	-0.15798	-1.56412	-1.36955
O	1.71305	-1.08847	-0.15669
C	2.69848	-1.58898	-1.14090
C	2.56497	-0.83572	-2.46387

C	4.02520	-1.25731	-0.46084
C	2.51208	-3.09667	-1.30027
H	1.61160	-1.04668	-2.95005
H	2.65319	0.24178	-2.29826
H	3.37428	-1.14789	-3.13178
H	4.09467	-1.75616	0.51087
H	4.85653	-1.59633	-1.08638
H	4.11681	-0.17792	-0.30656
H	2.57120	-3.59136	-0.32522
H	1.54696	-3.32406	-1.75590
H	3.30712	-3.49730	-1.93772

4q_conformer-014

C	-2.53582	-0.74985	0.07143
C	-3.36227	-1.83499	0.31237
C	-4.11734	-1.55122	1.48691
C	-3.72968	-0.30220	1.94234
N	-2.78700	0.17446	1.06352
H	-4.85914	-2.18523	1.95521
C	-4.15884	0.48331	3.13949
H	-2.21852	1.00691	1.17061
H	-4.92134	-0.06957	3.69513
H	-3.32250	0.67955	3.82319
H	-4.58467	1.45591	2.86107
C	-1.60377	-0.55156	-1.08478
H	-3.41966	-2.72361	-0.30312
N	-0.17747	-0.30967	-0.68422
H	-1.53811	-1.53046	-1.55802
C	-2.08720	0.39340	-2.22336
C	-3.27892	-0.26803	-2.93086
C	-2.45397	1.82588	-1.81644
H	-1.25156	0.43439	-2.93494
H	-2.83587	2.36518	-2.69071
H	-3.24120	1.82475	-1.05466
H	-1.60468	2.38472	-1.42128
H	-3.59537	0.33408	-3.78914
H	-3.02483	-1.27061	-3.29408
H	-4.13100	-0.36381	-2.24871
C	0.76853	-1.25999	-1.14005
O	0.55300	-1.92235	-2.14546
C	4.20779	-2.09890	1.26699
C	3.00148	-1.75922	1.88501
C	1.88196	-1.45854	1.11168
C	1.96282	-1.51136	-0.28560
C	3.16232	-1.88579	-0.90177

C	4.28638	-2.16317	-0.12729
H	5.08283	-2.32061	1.87059
H	2.93335	-1.72672	2.96809
H	0.94384	-1.19347	1.58920
H	3.20630	-1.93927	-1.98449
H	5.22178	-2.43245	-0.60827
C	0.23013	0.89610	-0.10896
O	-0.50037	1.62137	0.55181
O	1.49443	1.16465	-0.42828
C	2.25760	2.26493	0.21234
C	3.66814	2.02117	-0.31943
C	1.69686	3.59876	-0.27612
C	2.22026	2.12935	1.73359
H	4.03046	1.03703	-0.00762
H	3.68129	2.06834	-1.41272
H	4.34599	2.78647	0.07036
H	2.33650	4.41310	0.07960
H	1.68244	3.62646	-1.37045
H	0.68416	3.75939	0.09727
H	2.58131	1.14290	2.03742
H	2.88059	2.88566	2.16993
H	1.21369	2.27954	2.12588

4q_conformer-015

C	1.99952	-0.99003	-0.50938
C	2.29554	-1.44730	-1.78034
C	3.57095	-0.92437	-2.14241
C	4.02749	-0.15764	-1.08396
N	3.07036	-0.22015	-0.09847
H	4.10472	-1.08944	-3.06932
C	5.28239	0.63636	-0.91549
H	3.06624	0.31751	0.75777
H	5.07487	1.70352	-0.76298
H	5.87222	0.29417	-0.05549
H	5.90591	0.54168	-1.80872
C	0.80751	-1.28872	0.34236
H	1.66370	-2.09856	-2.37056
N	-0.12818	-0.11213	0.43456
H	0.23332	-2.03451	-0.20423
C	1.13379	-1.88789	1.73402
C	-0.14217	-2.12499	2.54963
C	1.91524	-3.19501	1.55431
H	1.76145	-1.17483	2.27595
H	2.15857	-3.62997	2.52969
H	1.32108	-3.93091	0.99723

H	2.84947	-3.03399	1.00843
H	-0.82188	-2.80863	2.02517
H	-0.68175	-1.19323	2.74563
H	0.10349	-2.57713	3.51641
C	0.34675	1.10779	0.92719
O	1.34026	1.15507	1.64955
C	-1.32955	4.79577	-0.45320
C	-1.31107	3.68784	-1.30492
C	-0.80480	2.47092	-0.85542
C	-0.30254	2.36015	0.44851
C	-0.29693	3.47897	1.28986
C	-0.82272	4.68971	0.84478
H	-1.73252	5.74174	-0.80278
H	-1.68935	3.77310	-2.31905
H	-0.77884	1.61305	-1.51983
H	0.11394	3.38542	2.28964
H	-0.83271	5.55096	1.50591
C	-1.51325	-0.28890	0.25836
O	-2.34580	0.42606	0.78569
O	-1.75630	-1.33132	-0.53855
C	-3.13917	-1.79774	-0.80182
C	-3.80980	-2.20093	0.51027
C	-3.90456	-0.70772	-1.55020
C	-2.90330	-3.01509	-1.69250
H	-3.19941	-2.94055	1.03853
H	-3.96623	-1.33865	1.15936
H	-4.78116	-2.65498	0.28966
H	-4.05972	0.16886	-0.92022
H	-3.35547	-0.40880	-2.44910
H	-4.87942	-1.09921	-1.85827
H	-2.37156	-2.72792	-2.60483
H	-2.31226	-3.77018	-1.16482
H	-3.86329	-3.45824	-1.97348

4q_conformer-016

C	1.59397	-1.60111	-0.00257
C	1.87919	-2.92853	-0.26277
C	2.52096	-2.99065	-1.53544
C	2.60637	-1.69991	-2.02735
N	2.05129	-0.87135	-1.07979
H	2.88021	-3.87709	-2.04191
C	3.15609	-1.16757	-3.31108
H	1.89371	0.12135	-1.17685
H	3.56151	-1.98628	-3.91189
H	2.38595	-0.66274	-3.90897

H	3.96275	-0.44255	-3.14237
C	0.95413	-0.98268	1.20050
H	1.65829	-3.75542	0.39971
N	-0.34277	-0.31401	0.85045
H	0.63453	-1.80756	1.83929
C	1.88359	-0.09712	2.06603
C	1.12804	0.49845	3.26096
C	3.07467	-0.93560	2.54681
H	2.27434	0.71875	1.45064
H	3.75143	-0.31926	3.14805
H	2.73675	-1.77249	3.17104
H	3.63986	-1.34932	1.70693
H	1.81491	1.06652	3.89730
H	0.32310	1.17443	2.95776
H	0.68218	-0.29710	3.87067
C	-1.52158	-1.05216	1.07164
O	-1.54578	-1.97279	1.87833
C	-4.93217	-0.40375	-1.43455
C	-3.64970	-0.21758	-1.95739
C	-2.53283	-0.38904	-1.14314
C	-2.69451	-0.75889	0.19926
C	-3.97935	-0.97164	0.71236
C	-5.09562	-0.78048	-0.09877
H	-5.80226	-0.26158	-2.06870
H	-3.52003	0.05901	-2.99926
H	-1.53645	-0.25589	-1.55315
H	-4.09037	-1.27839	1.74714
H	-6.09144	-0.92977	0.30743
C	-0.44182	1.03591	0.49301
O	-1.40714	1.72783	0.75678
O	0.65885	1.44348	-0.15637
C	0.86083	2.87562	-0.50996
C	0.86388	3.72141	0.76159
C	2.24286	2.86095	-1.15968
C	-0.20984	3.30200	-1.51194
H	-0.11590	3.72454	1.24038
H	1.60820	3.34451	1.47042
H	1.12959	4.75127	0.50320
H	2.24439	2.24465	-2.06517
H	2.52391	3.87870	-1.44528
H	2.99719	2.47656	-0.46610
H	-0.21916	2.61971	-2.36824
H	-1.19917	3.31154	-1.05415
H	0.02199	4.30746	-1.87755

4q_conformer-017

C	-2.65173	0.31338	0.31118
C	-3.64726	1.17525	0.73631
C	-4.47895	1.45625	-0.38801
C	-3.96664	0.76620	-1.47379
N	-2.86548	0.07419	-1.02850
H	-5.34949	2.09913	-0.41072
C	-4.41423	0.70506	-2.89857
H	-2.24089	-0.50985	-1.57524
H	-4.66406	-0.31842	-3.20699
H	-5.30595	1.32286	-3.03649
H	-3.64235	1.07178	-3.58773
C	-1.54624	-0.32268	1.09523
H	-3.75626	1.55698	1.74322
N	-0.20050	-0.14718	0.46470
H	-1.44557	0.27192	2.00293
C	-1.80133	-1.78754	1.57288
C	-2.59343	-1.72459	2.88758
C	-2.53533	-2.70027	0.58157
H	-0.81505	-2.21950	1.78950
H	-2.66676	-3.69176	1.02934
H	-3.53008	-2.30050	0.35656
H	-1.99524	-2.81806	-0.35823
H	-2.80524	-2.73170	3.26206
H	-2.04366	-1.17970	3.66334
H	-3.55164	-1.21481	2.72917
C	0.25913	-0.98813	-0.56727
O	-0.50910	-1.44590	-1.40959
C	4.36288	-2.19624	-0.57539
C	3.73586	-1.90297	0.63878
C	2.40534	-1.49141	0.65425
C	1.69188	-1.38363	-0.54698
C	2.31377	-1.70747	-1.75918
C	3.64981	-2.09977	-1.77398
H	5.40367	-2.50572	-0.58697
H	4.28366	-1.99367	1.57166
H	1.91650	-1.26177	1.59537
H	1.74631	-1.63033	-2.68045
H	4.13557	-2.33122	-2.71692
C	0.49378	1.02271	0.81234
O	0.22483	1.68678	1.79770
O	1.41613	1.30470	-0.10920
C	2.45999	2.33289	0.10995
C	3.21485	2.05102	1.40810
C	3.36695	2.12808	-1.10162
C	1.80667	3.71308	0.09470

H	2.56900	2.16938	2.27942
H	3.62278	1.03603	1.39795
H	4.04854	2.75525	1.49383
H	3.78252	1.11586	-1.10274
H	2.80763	2.27713	-2.03043
H	4.19087	2.84745	-1.07219
H	1.23622	3.85286	-0.82920
H	1.13771	3.83784	0.94771
H	2.58336	4.48359	0.13920

4q_conformer-018

C	2.55045	-0.19402	-0.63491
C	3.20110	-0.00297	-1.84175
C	4.25046	0.93584	-1.62862
C	4.22009	1.30134	-0.29368
N	3.20264	0.59152	0.29759
H	4.95495	1.30558	-2.36221
C	5.05544	2.27031	0.47907
H	2.83097	0.75901	1.22229
H	5.56673	1.78857	1.32220
H	5.81823	2.70692	-0.17138
H	4.45488	3.09233	0.89000
C	1.45753	-1.17208	-0.34378
H	2.95643	-0.51014	-2.76627
N	0.13131	-0.54241	-0.02102
H	1.25656	-1.65013	-1.30399
C	1.79965	-2.32980	0.63161
C	2.99285	-3.12350	0.08565
C	2.03498	-1.92094	2.08817
H	0.92133	-2.98870	0.60684
H	2.16763	-2.81794	2.70375
H	2.94214	-1.31541	2.18986
H	1.19790	-1.34625	2.49017
H	3.16691	-4.01635	0.69587
H	2.82170	-3.44644	-0.94780
H	3.90563	-2.51798	0.10102
C	0.01404	0.64223	0.72559
O	0.81215	0.92373	1.61331
C	-2.99328	3.49137	-0.35406
C	-2.39384	2.71319	-1.34813
C	-1.43142	1.76571	-1.00612
C	-1.05467	1.60301	0.33305
C	-1.63105	2.40758	1.32265
C	-2.60935	3.33853	0.98142
H	-3.75246	4.22071	-0.62037

H	-2.67720	2.84349	-2.38810
H	-0.96393	1.15976	-1.77577
H	-1.31833	2.28229	2.35398
H	-3.07078	3.94635	1.75375
C	-0.99200	-1.28975	-0.40811
O	-0.93359	-2.19016	-1.22836
O	-2.06687	-0.91978	0.28892
C	-3.42938	-1.38505	-0.06071
C	-4.30112	-0.54342	0.86877
C	-3.73805	-1.07303	-1.52433
C	-3.54105	-2.87376	0.26134
H	-4.04051	-0.73055	1.91512
H	-4.16585	0.52211	0.66028
H	-5.35422	-0.80104	0.72085
H	-3.10360	-1.65017	-2.19845
H	-4.78367	-1.32588	-1.72742
H	-3.59800	-0.00671	-1.72377
H	-2.89338	-3.46384	-0.38912
H	-3.26308	-3.05838	1.30407
H	-4.57646	-3.20004	0.11881

4q_conformer-019

C	1.84512	-1.20377	-0.58547
C	2.05121	-1.27390	-1.95089
C	3.33029	-0.70952	-2.22726
C	3.88537	-0.31024	-1.02389
N	2.96992	-0.61744	-0.04257
H	3.80396	-0.61196	-3.19532
C	5.19805	0.32948	-0.70645
H	3.09321	-0.41146	0.93708
H	5.07296	1.31413	-0.23721
H	5.79964	-0.28354	-0.02334
H	5.77330	0.47030	-1.62545
C	0.63793	-1.60360	0.21403
H	1.34861	-1.68218	-2.66577
N	-0.52267	-0.70689	-0.09465
H	0.29605	-2.56819	-0.16637
C	0.87448	-1.76396	1.73285
C	-0.44234	-1.99474	2.48295
C	1.83915	-2.93220	1.98697
H	1.31753	-0.83855	2.12017
H	2.04439	-3.02913	3.05823
H	1.39495	-3.87481	1.64391
H	2.79393	-2.80510	1.46880
H	-0.97202	-2.86883	2.08563

H	-1.10896	-1.13145	2.40824
H	-0.24396	-2.17469	3.54472
C	-1.72119	-1.30481	-0.50135
O	-1.75956	-2.48350	-0.84048
C	-5.43407	0.81232	-0.17025
C	-4.40947	1.08289	0.74055
C	-3.18317	0.43274	0.62548
C	-2.97743	-0.50333	-0.39735
C	-4.01550	-0.79032	-1.29167
C	-5.23476	-0.12429	-1.18758
H	-6.38700	1.32597	-0.08351
H	-4.56602	1.79947	1.54108
H	-2.39346	0.64491	1.33896
H	-3.85264	-1.53242	-2.06603
H	-6.03037	-0.33913	-1.89459
C	-0.32114	0.67498	-0.27334
O	-0.89206	1.33178	-1.12219
O	0.56683	1.12237	0.61631
C	1.07498	2.51490	0.54900
C	1.71962	2.77306	-0.81246
C	-0.07119	3.47740	0.85764
C	2.12008	2.54056	1.66167
H	2.44933	1.99142	-1.04419
H	0.97332	2.80733	-1.60692
H	2.24084	3.73545	-0.78069
H	-0.83731	3.43729	0.08248
H	-0.52591	3.22950	1.82249
H	0.32001	4.49833	0.91515
H	1.67368	2.25052	2.61772
H	2.94545	1.85932	1.43710
H	2.52796	3.55084	1.76004

4q_conformer-020

C	2.60281	-0.70457	-0.56743
C	3.40365	-0.86838	-1.68491
C	4.47456	0.06634	-1.59602
C	4.30492	0.78707	-0.42645
N	3.18176	0.29437	0.19350
H	5.28202	0.20350	-2.30345
C	5.09661	1.91099	0.16012
H	2.72295	0.70456	0.99547
H	5.95132	2.14040	-0.48211
H	4.49717	2.82541	0.25966
H	5.48154	1.66493	1.15803
C	1.40339	-1.51917	-0.19131

H	3.23624	-1.59693	-2.46798
N	0.10403	-0.76062	-0.18101
H	1.26445	-2.18761	-1.04109
C	1.56404	-2.44712	1.04398
C	1.80143	-1.75601	2.38981
C	0.38396	-3.42373	1.14291
H	2.46324	-3.03273	0.80699
H	0.57524	-4.17210	1.91928
H	-0.54124	-2.90163	1.41245
H	0.21128	-3.94889	0.19750
H	1.95917	-2.51431	3.16539
H	2.68596	-1.11367	2.36724
H	0.94521	-1.14169	2.67970
C	-0.95098	-1.29636	-0.95132
O	-0.73133	-2.08426	-1.86207
C	-5.01080	-0.46622	0.14025
C	-4.04093	-0.57636	1.14000
C	-2.71186	-0.81898	0.79928
C	-2.34897	-0.96837	-0.54531
C	-3.32817	-0.89143	-1.54213
C	-4.65236	-0.62456	-1.20136
H	-6.04418	-0.26515	0.40642
H	-4.31873	-0.47102	2.18427
H	-1.95959	-0.89681	1.57767
H	-3.03799	-1.02662	-2.57869
H	-5.40522	-0.54428	-1.97956
C	0.03833	0.52723	0.36320
O	0.73557	0.89983	1.29435
O	-0.83291	1.27873	-0.30547
C	-1.21720	2.63772	0.14922
C	-1.72445	2.60129	1.58991
C	-0.01953	3.56808	-0.03193
C	-2.34912	2.98451	-0.81537
H	-2.54599	1.88616	1.68687
H	-0.93091	2.33334	2.28842
H	-2.10151	3.59447	1.85434
H	0.34023	3.52729	-1.06503
H	-0.32284	4.59715	0.18658
H	0.79570	3.29241	0.63926
H	-1.99447	2.95971	-1.85037
H	-3.17287	2.27184	-0.71075
H	-2.72320	3.98972	-0.59927

4q_conformer-021

C	2.62306	-0.46030	-0.66076

C	3.29575	-0.45590	-1.87070
C	4.35306	0.49383	-1.77837
C	4.30169	1.05682	-0.51474
N	3.26124	0.45676	0.15316
H	5.07437	0.74442	-2.54529
C	5.13985	2.11653	0.12439
H	2.90279	0.72757	1.05892
H	5.90958	2.45502	-0.57458
H	4.54316	2.99084	0.41543
H	5.64350	1.75159	1.02872
C	1.47620	-1.33682	-0.26451
H	3.05336	-1.08293	-2.71924
N	0.20131	-0.57929	-0.00970
H	1.22953	-1.88424	-1.17460
C	1.78145	-2.43231	0.79812
C	2.35226	-1.96113	2.13934
C	0.55741	-3.33046	1.02499
H	2.55699	-3.03924	0.30977
H	0.82125	-4.17104	1.67534
H	-0.25025	-2.77518	1.51719
H	0.16778	-3.73441	0.08544
H	2.61948	-2.83541	2.74429
H	3.25602	-1.35981	2.00684
H	1.62638	-1.36389	2.69521
C	0.15607	0.55193	0.80720
O	0.97548	0.73285	1.70471
C	-2.62945	3.68365	-0.06920
C	-2.02184	2.98853	-1.11789
C	-1.13705	1.94712	-0.84429
C	-0.84534	1.60852	0.48275
C	-1.42557	2.33038	1.53154
C	-2.32935	3.35388	1.25582
H	-3.32882	4.48618	-0.28367
H	-2.23836	3.25583	-2.14771
H	-0.66501	1.40394	-1.65719
H	-1.17555	2.07051	2.55490
H	-2.79622	3.89756	2.07149
C	-0.96328	-1.13306	-0.58406
O	-0.93795	-1.85561	-1.56395
O	-2.03423	-0.81042	0.13776
C	-3.41167	-1.12731	-0.31163
C	-3.66326	-0.56404	-1.70917
C	-3.61167	-2.63957	-0.24025
C	-4.25929	-0.39850	0.72849
H	-3.45523	0.50931	-1.73029
H	-3.04816	-1.06350	-2.45875

H	-4.71685	-0.71526	-1.96519
H	-3.37506	-3.00735	0.76339
H	-4.65815	-2.87820	-0.45647
H	-2.97767	-3.15253	-0.96547
H	-4.03683	-0.76847	1.73418
H	-4.05952	0.67695	0.69957
H	-5.32082	-0.56587	0.52291

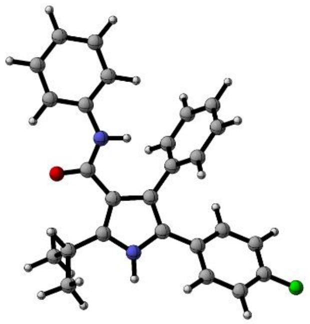

4r_conformer-001

C	1.79885	0.68867	-0.01129
C	0.51714	0.16471	0.08305
C	-0.40006	1.28164	0.06758
C	0.35914	2.45051	-0.03444
N	1.66697	2.06540	-0.09215
H	2.44777	2.70631	-0.10696
C	5.62737	-1.15694	-0.03902
C	5.43253	0.00148	-0.78001
C	4.17172	0.59446	-0.77228
C	3.11491	0.04747	-0.02249
C	3.35715	-1.12150	0.72209
C	4.60793	-1.73170	0.71151
F	6.84692	-1.74375	-0.04661
H	6.24971	0.41768	-1.35908
H	4.00437	1.48067	-1.37713
H	2.56189	-1.54841	1.32176
H	4.80181	-2.63152	1.28536
C	-0.54147	-3.99053	0.27373
C	-0.90065	-3.12676	1.31202
C	-0.54262	-1.78069	1.25828
C	0.17470	-1.27221	0.16154
C	0.52972	-2.15009	-0.87550
C	0.17733	-3.49883	-0.81795
H	-0.82159	-5.03894	0.31516
H	-1.45923	-3.50175	2.16459
H	-0.82499	-1.10819	2.06280
H	1.07796	-1.76370	-1.72920
H	0.45747	-4.16342	-1.63007
C	-0.05160	3.89682	-0.05908

C	0.90243	4.76557	-0.89220
H	0.50781	5.78402	-0.96499
H	1.89503	4.84062	-0.43033
H	1.02556	4.37226	-1.90678
C	-1.87559	1.27918	0.07374
O	-2.54232	2.25814	0.43302
N	-2.45143	0.11319	-0.37877
H	-1.80610	-0.61608	-0.65233
H	-1.04514	3.92742	-0.51560
C	-0.19382	4.44055	1.37600
H	-0.52802	5.48421	1.35545
H	-0.92783	3.84996	1.92986
H	0.76493	4.39937	1.90643
C	-6.42344	-1.28084	-0.25779
C	-6.16514	0.07156	-0.02931
C	-4.86391	0.57517	-0.06196
C	-3.79410	-0.29537	-0.32193
C	-4.05106	-1.65857	-0.54983
C	-5.35519	-2.14289	-0.52001
H	-7.44102	-1.65881	-0.23274
H	-6.98655	0.75335	0.17380
H	-4.66410	1.62224	0.11215
H	-3.22083	-2.33462	-0.73920
H	-5.53495	-3.19938	-0.69779

4r_conformer-002

C	-1.79883	0.68867	-0.01127
C	-0.51712	0.16472	0.08310
C	0.40007	1.28165	0.06766
C	-0.35913	2.45052	-0.03437
N	-1.66696	2.06539	-0.09212
H	-2.44777	2.70629	-0.10696
C	-5.62733	-1.15698	-0.03910
C	-5.43248	0.00143	-0.78010
C	-4.17168	0.59443	-0.77234
C	-3.11488	0.04745	-0.02251
C	-3.35714	-1.12151	0.72208
C	-4.60791	-1.73172	0.71147
F	-6.84687	-1.74380	-0.04672
H	-6.24965	0.41762	-1.35919
H	-4.00431	1.48063	-1.37719
H	-2.56189	-1.54841	1.32177
H	-4.80180	-2.63153	1.28532
C	0.54146	-3.99053	0.27383
C	0.90063	-3.12676	1.31213

C	0.54261	-1.78068	1.25837
C	-0.17468	-1.27220	0.16161
C	-0.52968	-2.15008	-0.87544
C	-0.17731	-3.49883	-0.81787
H	0.82157	-5.03894	0.31528
H	1.45918	-3.50175	2.16471
H	0.82496	-1.10818	2.06288
H	-1.07790	-1.76370	-1.72915
H	-0.45743	-4.16343	-1.62999
C	0.05157	3.89683	-0.05901
C	0.19338	4.44069	1.37606
H	0.52757	5.48437	1.35550
H	-0.76550	4.39955	1.90623
H	0.92725	3.85019	1.93020
C	1.87561	1.27919	0.07383
O	2.54235	2.25813	0.43312
N	2.45143	0.11320	-0.37872
H	1.80610	-0.61606	-0.65228
H	1.04522	3.92744	-0.51528
C	-0.90229	4.76548	-0.89244
H	-0.50769	5.78394	-0.96522
H	-1.02517	4.37207	-1.90701
H	-1.89500	4.84053	-0.43081
C	6.42345	-1.28082	-0.25791
C	6.16516	0.07156	-0.02936
C	4.86392	0.57517	-0.06195
C	3.79411	-0.29535	-0.32194
C	4.05107	-1.65855	-0.54991
C	5.35519	-2.14287	-0.52015
H	7.44103	-1.65879	-0.23291
H	6.98657	0.75335	0.17376
H	4.66411	1.62223	0.11223
H	3.22083	-2.33458	-0.73930
H	5.53495	-3.19935	-0.69799

4r_conformer-003

C	-1.84524	0.81425	-0.00021
C	-0.58115	0.25259	0.11269
C	0.37283	1.33939	0.11589
C	-0.34876	2.53362	0.00795
N	-1.66616	2.18386	-0.07523
H	-2.42212	2.85485	-0.10125
C	-5.72614	-0.91770	-0.07585
C	-5.48779	0.23332	-0.81561
C	-4.21044	0.78944	-0.79195

C	-3.18008	0.21311	-0.02754
C	-3.46625	-0.94689	0.71540
C	-4.73400	-1.52050	0.68908
F	-6.96201	-1.46890	-0.09918
H	-6.28475	0.67208	-1.40610
H	-4.00993	1.66971	-1.39544
H	-2.69162	-1.39583	1.32587
H	-4.96155	-2.41334	1.26147
C	0.36879	-3.92796	0.31445
C	0.74808	-3.07113	1.35157
C	0.42389	-1.71705	1.29519
C	-0.28042	-1.19309	0.19773
C	-0.65596	-2.06331	-0.83828
C	-0.33635	-3.42056	-0.77866
H	0.62352	-4.98276	0.35778
H	1.29732	-3.45827	2.20480
H	0.72290	-1.05041	2.09860
H	-1.19360	-1.66511	-1.69334
H	-0.63203	-4.07915	-1.59018
C	0.03554	3.98974	-0.04727
C	1.05084	4.28765	-1.16342
H	1.23107	5.36678	-1.22360
H	2.00278	3.79134	-0.96189
H	0.67706	3.94798	-2.13529
C	1.84710	1.23228	0.15984
O	2.57972	2.09667	0.65526
N	2.34438	0.08675	-0.42008
H	1.65182	-0.55011	-0.79107
H	-0.88631	4.53550	-0.29166
C	0.52717	4.49931	1.32004
H	0.73751	5.57380	1.26776
H	-0.23115	4.33640	2.09385
H	1.43884	3.97047	1.60565
C	6.15934	-1.68770	-0.24605
C	6.01777	-0.33559	0.06978
C	4.77149	0.29087	0.02251
C	3.63926	-0.45419	-0.34033
C	3.77804	-1.81644	-0.65702
C	5.02924	-2.42387	-0.61216
H	7.13530	-2.16213	-0.20844
H	6.88877	0.24889	0.35343
H	4.66174	1.33777	0.26463
H	2.89790	-2.39549	-0.92563
H	5.11857	-3.47805	-0.85873

4r_conformer-004

C	1.84513	0.81435	-0.00015
C	0.58112	0.25248	0.11270
C	-0.37304	1.33909	0.11592
C	0.34837	2.53347	0.00817
N	1.66583	2.18393	-0.07500
H	2.42167	2.85505	-0.10092
C	5.72634	-0.91696	-0.07633
C	4.73431	-1.52009	0.68848
C	3.46645	-0.94671	0.71498
C	3.18008	0.21343	-0.02765
C	4.21032	0.79013	-0.79195
C	5.48775	0.23419	-0.81581
F	6.96229	-1.46796	-0.09985
H	4.96200	-2.41304	1.26064
H	2.69192	-1.39595	1.32536
H	4.00967	1.67052	-1.39521
H	6.28461	0.67324	-1.40621
C	-0.36758	-3.92832	0.31470
C	-0.74648	-3.07170	1.35212
C	-0.42275	-1.71750	1.29561
C	0.28075	-1.19330	0.19776
C	0.65595	-2.06332	-0.83853
C	0.33675	-3.42066	-0.77882
H	-0.62196	-4.98320	0.35813
H	-1.29508	-3.45905	2.20565
H	-0.72149	-1.05100	2.09924
H	1.19303	-1.66493	-1.69384
H	0.63214	-4.07913	-1.59054
C	-0.03611	3.98956	-0.04679
C	-0.52766	4.49886	1.32065
H	-0.73817	5.57332	1.26857
H	-1.43923	3.96989	1.60632
H	0.23076	4.33594	2.09435
C	-1.84726	1.23179	0.15996
O	-2.57991	2.09605	0.65556
N	-2.34450	0.08640	-0.42026
H	-1.65192	-0.55056	-0.79106
H	0.88559	4.53555	-0.29118
C	-1.05154	4.28754	-1.16280
H	-1.23191	5.36666	-1.22282
H	-0.67780	3.94808	-2.13477
H	-2.00342	3.79112	-0.96132
C	-6.15976	-1.68745	-0.24624
C	-6.01825	-0.33491	0.06778
C	-4.77185	0.29130	0.02046

C	-3.63946	-0.45443	-0.34051
C	-3.77820	-1.81708	-0.65542
C	-5.02952	-2.42429	-0.61057
H	-7.13580	-2.16169	-0.20862
H	-6.88937	0.25008	0.34999
H	-4.66219	1.33854	0.26115
H	-2.89799	-2.39663	-0.92263
H	-5.11885	-3.47878	-0.85573

4r_conformer-005

C	-1.80248	0.70138	-0.08638
C	-0.52442	0.16445	-0.14652
C	0.40124	1.27310	-0.21390
C	-0.35296	2.45166	-0.20438
N	-1.66157	2.07924	-0.10899
H	-2.43823	2.72312	-0.15781
C	-5.64433	-1.11139	0.07259
C	-5.44480	0.10316	0.71597
C	-4.17993	0.68527	0.66585
C	-3.12295	0.07152	-0.03052
C	-3.37033	-1.15349	-0.67713
C	-4.62538	-1.75257	-0.62257
F	-6.86788	-1.68777	0.12266
H	-6.26172	0.57138	1.25426
H	-4.01064	1.61779	1.19590
H	-2.57619	-1.63406	-1.23610
H	-4.82261	-2.69545	-1.12121
C	0.46683	-4.01156	-0.07137
C	0.78869	-3.23484	-1.18775
C	0.45494	-1.88167	-1.21863
C	-0.20064	-1.27968	-0.13053
C	-0.51864	-2.07058	0.98529
C	-0.19020	-3.42634	1.01306
H	0.72783	-5.06541	-0.04711
H	1.29900	-3.68313	-2.03513
H	0.70859	-1.27600	-2.08351
H	-1.02004	-1.61115	1.83158
H	-0.44114	-4.02335	1.88497
C	0.06819	3.89786	-0.21397
C	0.62775	4.30616	1.16323
H	0.96151	5.34986	1.14155
H	-0.14080	4.20843	1.93882
H	1.48032	3.67853	1.43519
C	1.87777	1.26094	-0.22407
O	2.55001	2.23563	-0.58442

N	2.45054	0.09232	0.22448
H	1.80551	-0.63456	0.50474
H	0.88611	3.97737	-0.93448
C	-1.06393	4.83997	-0.64683
H	-0.68218	5.86238	-0.72756
H	-1.47993	4.55681	-1.61996
H	-1.87966	4.85990	0.08708
C	6.42863	-1.28931	0.18648
C	5.36013	-2.14628	0.46398
C	4.05456	-1.66522	0.46648
C	3.79542	-0.31030	0.19427
C	4.86566	0.55507	-0.08172
C	6.16856	0.05469	-0.08522
H	7.44747	-1.66468	0.18280
H	5.54096	-3.19631	0.67575
H	3.22461	-2.33767	0.66982
H	4.66485	1.59556	-0.29010
H	6.98997	0.73265	-0.30088

4r_conformer-006

C	1.80246	0.70143	-0.08633
C	0.52442	0.16445	-0.14630
C	-0.40129	1.27306	-0.21368
C	0.35287	2.45165	-0.20432
N	1.66150	2.07928	-0.10906
H	2.43813	2.72318	-0.15796
C	5.64438	-1.11122	0.07239
C	5.44491	0.10343	0.71562
C	4.18001	0.68549	0.66560
C	3.12295	0.07162	-0.03054
C	3.37028	-1.15348	-0.67702
C	4.62535	-1.75252	-0.62254
F	6.86795	-1.68756	0.12237
H	6.26189	0.57174	1.25374
H	4.01077	1.61808	1.19554
H	2.57608	-1.63415	-1.23582
H	4.82255	-2.69547	-1.12108
C	-0.46637	-4.01168	-0.07093
C	-0.78837	-3.23504	-1.18732
C	-0.45480	-1.88183	-1.21826
C	0.20073	-1.27970	-0.13020
C	0.51884	-2.07053	0.98565
C	0.19058	-3.42633	1.01347
H	-0.72723	-5.06557	-0.04663
H	-1.29863	-3.68343	-2.03469

H	-0.70854	-1.27622	-2.08317
H	1.02021	-1.61100	1.83190
H	0.44163	-4.02327	1.88540
C	-0.06828	3.89786	-0.21408
C	1.06404	4.83993	-0.64649
H	0.68235	5.86236	-0.72736
H	1.87947	4.85981	0.08777
H	1.48042	4.55680	-1.61947
C	-1.87783	1.26080	-0.22405
O	-2.55010	2.23546	-0.58442
N	-2.45061	0.09213	0.22441
H	-1.80560	-0.63479	0.50461
H	-0.88590	3.97739	-0.93494
C	-0.62833	4.30627	1.16290
H	-0.96196	5.35001	1.14101
H	-1.48104	3.67875	1.43460
H	0.13995	4.20854	1.93876
C	-6.42879	-1.28926	0.18616
C	-6.16867	0.05487	-0.08485
C	-4.86573	0.55516	-0.08126
C	-3.79551	-0.31045	0.19409
C	-4.05471	-1.66549	0.46562
C	-5.36031	-2.14646	0.46305
H	-7.44766	-1.66456	0.18241
H	-6.99005	0.73301	-0.30001
H	-4.66486	1.59574	-0.28909
H	-3.22477	-2.33810	0.66849
H	-5.54119	-3.19659	0.67428

4r_conformer-007

C	-1.68956	0.72518	-0.08770
C	-0.71658	-0.26391	-0.17849
C	0.55363	0.40772	-0.27194
C	0.31862	1.78154	-0.26155
N	-1.03746	1.94374	-0.15835
H	-1.50320	2.83547	-0.06474
C	-5.89971	0.55980	0.47192
C	-5.09846	-0.38573	1.10258
C	-3.72060	-0.32641	0.91739
C	-3.14185	0.65855	0.09681
C	-3.98691	1.59378	-0.52595
C	-5.36736	1.55443	-0.33787
F	-7.23973	0.50942	0.65460
H	-5.55159	-1.14105	1.73559
H	-3.08278	-1.04523	1.41837

H	-3.56627	2.34888	-1.18349
H	-6.02464	2.27105	-0.81824
C	-1.38065	-4.50215	-0.14429
C	-0.40203	-3.93617	0.67638
C	-0.18588	-2.55907	0.66306
C	-0.94002	-1.72090	-0.17360
C	-1.91531	-2.30263	-0.99967
C	-2.13672	-3.67892	-0.98211
H	-1.55108	-5.57488	-0.13230
H	0.19086	-4.56745	1.33249
H	0.56763	-2.12230	1.31190
H	-2.49768	-1.66658	-1.65908
H	-2.89603	-4.10975	-1.62892
C	1.24452	2.96095	-0.39627
C	1.39267	3.71994	0.93685
H	2.10225	4.54685	0.82725
H	0.43268	4.13814	1.26079
H	1.75397	3.06316	1.73613
C	1.85804	-0.26405	-0.48085
O	2.00630	-1.20621	-1.26060
N	2.87992	0.25651	0.28412
H	2.60001	0.94649	0.96830
H	2.22656	2.56153	-0.66891
C	0.79973	3.90732	-1.52564
H	1.53329	4.71021	-1.65286
H	0.70583	3.37030	-2.47473
H	-0.16595	4.37632	-1.30344
C	7.01559	-0.55006	0.36857
C	6.19212	-1.27480	-0.49390
C	4.81737	-1.04106	-0.54886
C	4.25235	-0.05578	0.27568
C	5.07911	0.67575	1.14440
C	6.44771	0.42776	1.18929
H	8.08342	-0.74343	0.40252
H	6.61960	-2.03931	-1.13686
H	4.17972	-1.60647	-1.21200
H	4.64368	1.44122	1.78213
H	7.07080	1.00355	1.86754

4r_conformer-008

C	1.68953	0.72519	-0.08765
C	0.71662	-0.26397	-0.17834
C	-0.55366	0.40757	-0.27172
C	-0.31873	1.78141	-0.26139
N	1.03734	1.94371	-0.15829

H	1.50302	2.83547	-0.06476
C	5.89977	0.56015	0.47144
C	5.09867	-0.38543	1.10222
C	3.72078	-0.32621	0.91722
C	3.14185	0.65868	0.09668
C	3.98676	1.59396	-0.52622
C	5.36723	1.55471	-0.33831
F	7.23982	0.50987	0.65393
H	5.55193	-1.14070	1.73519
H	3.08309	-1.04507	1.41830
H	3.56597	2.34900	-1.18372
H	6.02439	2.27136	-0.81880
C	1.38106	-4.50215	-0.14385
C	0.40252	-3.93617	0.67693
C	0.18624	-2.55909	0.66350
C	0.94016	-1.72094	-0.17336
C	1.91538	-2.30266	-0.99954
C	2.13692	-3.67894	-0.98187
H	1.55158	-5.57485	-0.13177
H	-0.19021	-4.56744	1.33320
H	-0.56721	-2.12232	1.31242
H	2.49758	-1.66663	-1.65909
H	2.89616	-4.10977	-1.62876
C	-1.24472	2.96077	-0.39592
C	-0.79981	3.90760	-1.52486
H	-0.70562	3.37093	-2.47411
H	-1.53345	4.71042	-1.65196
H	0.16575	4.37667	-1.30227
C	-1.85800	-0.26434	-0.48056
O	-2.00610	-1.20679	-1.25999
N	-2.88003	0.25644	0.28407
H	-2.60031	0.94682	0.96791
H	-2.22667	2.56135	-0.66892
C	-1.39321	3.71928	0.93744
H	-0.43334	4.13756	1.26161
H	-2.10295	4.54608	0.82803
H	-1.75443	3.06218	1.73648
C	-7.01573	-0.55001	0.36786
C	-6.44817	0.42878	1.18764
C	-5.07956	0.67674	1.14295
C	-4.25247	-0.05581	0.27541
C	-4.81717	-1.04208	-0.54817
C	-6.19194	-1.27576	-0.49344
H	-8.08358	-0.74335	0.40165
H	-7.07151	1.00538	1.86497
H	-4.64438	1.44300	1.77991

H	-4.17926	-1.60825	-1.21042
H	-6.61917	-2.04103	-1.13565

4r_conformer-009

C	1.78396	0.81549	-0.12017
C	0.77557	-0.13764	-0.22818
C	-0.46599	0.57820	-0.35421
C	-0.18420	1.94229	-0.34598
N	1.17649	2.05267	-0.21441
H	1.66386	2.93303	-0.11766
C	5.97053	0.49257	0.54119
C	5.11803	-0.41493	1.16041
C	3.74835	-0.30329	0.94163
C	3.22799	0.69535	0.09866
C	4.12391	1.59049	-0.51185
C	5.49688	1.49926	-0.28985
F	7.30297	0.39182	0.75657
H	5.52594	-1.18131	1.81068
H	3.07092	-0.99179	1.43329
H	3.74901	2.35480	-1.18608
H	6.19301	2.18498	-0.76053
C	1.21985	-4.40494	-0.21034
C	0.22432	-3.79684	0.55760
C	0.08371	-2.40968	0.55294
C	0.93111	-1.60275	-0.22377
C	1.92231	-2.22736	-0.99895
C	2.06882	-3.61334	-0.98799
H	1.33191	-5.48534	-0.20465
H	-0.44077	-4.40259	1.16684
H	-0.68309	-1.94195	1.16374
H	2.57540	-1.61687	-1.61459
H	2.84187	-4.07690	-1.59465
C	-1.02319	3.18721	-0.49890
C	-1.62234	3.66087	0.83967
H	-0.85125	3.74772	1.61175
H	-2.09689	4.64031	0.71492
H	-2.39028	2.96836	1.20008
C	-1.77980	-0.07516	-0.59718
O	-1.97108	-0.85315	-1.53135
N	-2.74040	0.25359	0.33125
H	-2.41578	0.82679	1.09860
H	-0.33310	3.97308	-0.83447
C	-2.10787	3.04769	-1.57906
H	-2.87630	2.32684	-1.28504
H	-2.60091	4.01273	-1.73701

H	-1.67720	2.72022	-2.53052
C	-6.83726	-0.70151	0.58346
C	-6.08496	-1.18001	-0.48998
C	-4.72490	-0.89000	-0.60643
C	-4.10295	-0.10021	0.37266
C	-4.85729	0.38244	1.45442
C	-6.21240	0.08242	1.55660
H	-7.89443	-0.93582	0.66263
H	-6.55750	-1.79194	-1.25336
H	-4.14156	-1.26608	-1.43371
H	-4.37672	0.99291	2.21490
H	-6.77986	0.46433	2.40030

4r_conformer-010

C	-1.78396	0.81549	-0.12017
C	-0.77556	-0.13764	-0.22817
C	0.46600	0.57822	-0.35421
C	0.18419	1.94230	-0.34601
N	-1.17650	2.05268	-0.21443
H	-1.66387	2.93303	-0.11768
C	-5.97053	0.49254	0.54120
C	-5.49688	1.49923	-0.28984
C	-4.12391	1.59048	-0.51184
C	-3.22799	0.69534	0.09866
C	-3.74834	-0.30329	0.94164
C	-5.11802	-0.41495	1.16042
F	-7.30296	0.39178	0.75658
H	-6.19302	2.18495	-0.76052
H	-3.74903	2.35479	-1.18607
H	-3.07091	-0.99180	1.43330
H	-5.52592	-1.18132	1.81069
C	-1.21983	-4.40494	-0.21034
C	-2.06880	-3.61333	-0.98800
C	-1.92230	-2.22735	-0.99895
C	-0.93110	-1.60274	-0.22376
C	-0.08370	-2.40967	0.55296
C	-0.22431	-3.79683	0.55761
H	-1.33189	-5.48533	-0.20466
H	-2.84185	-4.07689	-1.59466
H	-2.57538	-1.61686	-1.61460
H	0.68309	-1.94194	1.16377
H	0.44078	-4.40258	1.16685
C	1.02317	3.18723	-0.49894
C	2.10787	3.04770	-1.57906
H	1.67723	2.72019	-2.53053

H	2.60089	4.01275	-1.73703
H	2.87631	2.32687	-1.28501
C	1.77980	-0.07515	-0.59718
O	1.97107	-0.85314	-1.53136
N	2.74040	0.25358	0.33125
H	2.41580	0.82683	1.09857
H	0.33308	3.97308	-0.83454
C	1.62228	3.66092	0.83964
H	2.39019	2.96842	1.20010
H	2.09685	4.64035	0.71488
H	0.85116	3.74781	1.61169
C	6.83726	-0.70152	0.58347
C	6.08492	-1.18019	-0.48987
C	4.72485	-0.89018	-0.60632
C	4.10295	-0.10022	0.37266
C	4.85734	0.38259	1.45432
C	6.21244	0.08256	1.55651
H	7.89442	-0.93585	0.66265
H	6.55743	-1.79225	-1.25316
H	4.14149	-1.26638	-1.43352
H	4.37680	0.99319	2.21473
H	6.77993	0.46460	2.40013

4r_conformer-011

C	1.84521	0.81424	0.00033
C	0.58112	0.25256	-0.11258
C	-0.37286	1.33936	-0.11582
C	0.34871	2.53360	-0.00794
N	1.66612	2.18385	0.07528
H	2.42206	2.85485	0.10127
C	5.72618	-0.91758	0.07597
C	4.73414	-1.52024	-0.68920
C	3.46637	-0.94667	-0.71551
C	3.18007	0.21314	0.02766
C	4.21033	0.78932	0.79233
C	5.48770	0.23324	0.81600
F	6.96206	-1.46874	0.09930
H	4.96178	-2.41294	-1.26178
H	2.69181	-1.39551	-1.32616
H	4.00972	1.66942	1.39603
H	6.28458	0.67189	1.40667
C	-0.36867	-3.92801	-0.31483
C	0.33654	-3.42072	0.77829
C	0.65608	-2.06346	0.83807
C	0.28040	-1.19311	-0.19777

C	-0.42402	-1.71694	-1.29522
C	-0.74811	-3.07104	-1.35179
H	-0.62333	-4.98281	-0.35830
H	0.63233	-4.07941	1.58969
H	1.19377	-1.66537	1.69314
H	-0.72315	-1.05022	-2.09851
H	-1.29742	-3.45808	-2.20502
C	-0.03560	3.98972	0.04713
C	-1.05113	4.28771	1.16304
H	-1.23132	5.36686	1.22315
H	-2.00305	3.79145	0.96134
H	-0.67757	3.94807	2.13502
C	-1.84712	1.23222	-0.15981
O	-2.57975	2.09648	-0.65543
N	-2.34439	0.08678	0.42033
H	-1.65184	-0.54999	0.79147
H	0.88618	4.53551	0.29166
C	-0.52697	4.49917	-1.32032
H	-0.73739	5.57365	-1.26816
H	0.23153	4.33626	-2.09396
H	-1.43854	3.97026	-1.60610
C	-6.15934	-1.68769	0.24618
C	-6.01780	-0.33552	-0.06938
C	-4.77151	0.29093	-0.02209
C	-3.63925	-0.45421	0.34051
C	-3.77801	-1.81651	0.65695
C	-5.02921	-2.42394	0.61206
H	-7.13530	-2.16211	0.20855
H	-6.88882	0.24902	-0.35285
H	-4.66178	1.33788	-0.26402
H	-2.89785	-2.39561	0.92539
H	-5.11852	-3.47816	0.85842

4r_conformer-012

C	-1.84523	0.81428	0.00025
C	-0.58115	0.25258	-0.11257
C	0.37287	1.33935	-0.11575
C	-0.34870	2.53361	-0.00788
N	-1.66612	2.18388	0.07525
H	-2.42205	2.85490	0.10118
C	-5.72614	-0.91766	0.07581
C	-5.48783	0.23341	0.81550
C	-4.21049	0.78954	0.79186
C	-3.18007	0.21315	0.02755
C	-3.46620	-0.94690	-0.71532

C	-4.73396	-1.52052	-0.68903
F	-6.96200	-1.46887	0.09912
H	-6.28482	0.67221	1.40591
H	-4.01002	1.66986	1.39530
H	-2.69154	-1.39588	-1.32573
H	-4.96147	-2.41339	-1.26137
C	0.36862	-3.92801	-0.31430
C	-0.33618	-3.42052	0.77899
C	-0.65575	-2.06325	0.83861
C	-0.28049	-1.19312	-0.19756
C	0.42346	-1.71717	-1.29521
C	0.74759	-3.07127	-1.35160
H	0.62329	-4.98282	-0.35764
H	-0.63163	-4.07904	1.59065
H	-1.19312	-1.66498	1.69381
H	0.72224	-1.05060	-2.09876
H	1.29656	-3.45848	-2.20498
C	0.03556	3.98974	0.04723
C	0.52691	4.49925	-1.32021
H	0.73725	5.57374	-1.26803
H	1.43852	3.97041	-1.60600
H	-0.23157	4.33629	-2.09386
C	1.84713	1.23223	-0.15967
O	2.57977	2.09667	-0.65499
N	2.34442	0.08665	0.42016
H	1.65187	-0.55031	0.79100
H	-0.88626	4.53547	0.29175
C	1.05105	4.28777	1.16316
H	1.23117	5.36693	1.22328
H	0.67748	3.94810	2.13512
H	2.00300	3.79156	0.96147
C	6.15946	-1.68762	0.24578
C	6.01785	-0.33542	-0.06963
C	4.77154	0.29097	-0.02224
C	3.63932	-0.45426	0.34028
C	3.77814	-1.81659	0.65656
C	5.02937	-2.42396	0.61158
H	7.13544	-2.16200	0.20807
H	6.88885	0.24919	-0.35304
H	4.66177	1.33794	-0.26403
H	2.89801	-2.39576	0.92494
H	5.11873	-3.47821	0.85783

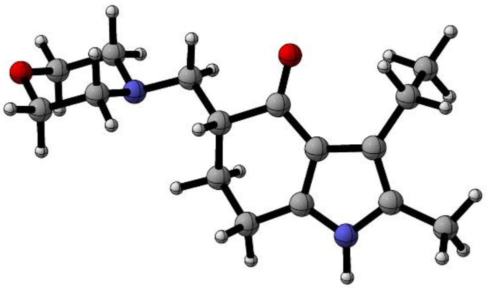

Note: This enantiomer was not used in the univariate correlation, but was verified to behave the same as the (S)-enantiomer
4s_Renantiomer_conformer-001

C	3.38947	0.30571	0.28133
N	3.21777	-1.87445	-0.20853
H	3.48534	-2.82583	-0.42266
C	1.94061	-1.41978	-0.15836
C	1.99608	-0.05976	0.14538
C	-0.43625	-1.25115	-0.75118
C	0.69824	-2.21568	-0.37486
H	-0.26167	-0.86607	-1.76487
H	-1.39579	-1.77428	-0.76926
H	0.84339	-2.97255	-1.15484
H	0.43147	-2.75979	0.54398
C	0.79123	0.72661	0.31299
O	0.79027	1.93914	0.55315
C	-0.52990	-0.06082	0.21965
C	-1.68770	0.88151	-0.13042
H	-1.71102	1.71231	0.59447
H	-1.48637	1.32715	-1.11229
C	-5.30921	0.31549	-0.96043
C	-3.98603	1.06180	-0.84092
N	-2.98045	0.20229	-0.21490
C	-3.46632	-0.24502	1.09138
C	-4.80452	-0.95806	0.93396
O	-5.77423	-0.11857	0.31470
H	-5.18236	-0.55213	-1.62754
H	-6.08514	0.96703	-1.37183
H	-4.14659	1.99028	-0.25872
H	-3.63674	1.35064	-1.83883
H	-2.74651	-0.94016	1.53475
H	-3.58460	0.60723	1.78900
H	-4.66353	-1.87200	0.33489
H	-5.20937	-1.23623	1.91092
C	3.92535	1.67717	0.57481
H	3.35339	2.12028	1.39755
H	4.96548	1.60221	0.91382
C	3.84982	2.61787	-0.63847

H	4.42222	2.21432	-1.48152
H	2.80927	2.73954	-0.95347
H	4.25271	3.60774	-0.39343
C	4.11987	-0.84056	0.05440
C	5.59127	-1.09480	0.04136
H	5.94431	-1.39800	-0.95285
H	6.13476	-0.18985	0.32349
H	5.87377	-1.88852	0.74457
H	-0.68812	-0.46598	1.23220

4s_Renantiomer_conformer-002

C	3.39087	0.34382	-0.19553
N	3.21763	-1.87964	0.02439
H	3.48260	-2.85365	0.08325
C	1.94105	-1.42121	0.03177
C	1.99741	-0.03371	-0.09759
C	-0.46757	-1.43365	-0.44235
C	0.69872	-2.23661	0.15244
H	-0.35594	-1.38962	-1.53410
H	-1.41918	-1.93110	-0.23865
H	0.80783	-3.20254	-0.35471
H	0.49250	-2.45942	1.21041
C	0.79406	0.77239	-0.11713
O	0.79211	1.99978	-0.26371
C	-0.52254	0.00285	0.10640
C	-1.70530	0.79431	-0.46546
H	-1.70507	1.80975	-0.03513
H	-1.55317	0.91112	-1.54544
C	-5.35333	0.00760	-0.91168
C	-4.03583	0.75227	-1.08990
N	-2.99315	0.12723	-0.27527
C	-3.41467	0.10747	1.12622
C	-4.74927	-0.61727	1.25873
O	-5.75601	-0.00953	0.45514
H	-5.24482	-1.02414	-1.28263
H	-6.15503	0.50073	-1.46838
H	-4.18255	1.81570	-0.81704
H	-3.73447	0.71714	-2.14308
H	-2.66726	-0.41764	1.72926
H	-3.51262	1.13399	1.53078
H	-4.62276	-1.67194	0.96593
H	-5.10724	-0.57878	2.29126
C	3.92890	1.74073	-0.31207
H	4.96768	1.70836	-0.66182
H	3.35467	2.28777	-1.06762

C	3.86075	2.51582	1.01361
H	2.82226	2.59732	1.34811
H	4.43621	2.00506	1.79408
H	4.26425	3.52870	0.89767
C	4.12038	-0.82245	-0.11334
C	5.59096	-1.08003	-0.14630
H	6.13540	-0.14860	-0.31934
H	5.95217	-1.50570	0.79880
H	5.86347	-1.78104	-0.94558
H	-0.62671	-0.06045	1.20174

4s_Renantiomer_conformer-003

C	-3.25658	0.21160	-0.02771
N	-2.68787	-1.95246	0.09269
H	-2.76710	-2.95099	0.23084
C	-1.57098	-1.31653	-0.34094
C	-1.87639	0.04302	-0.42736
C	0.52232	-1.00039	-1.59481
C	-0.25817	-1.94604	-0.66595
H	1.55300	-1.35054	-1.69527
H	0.06241	-1.02243	-2.59010
H	0.31701	-2.12571	0.25369
H	-0.39674	-2.92468	-1.14032
C	-0.88590	1.00973	-0.85159
O	-1.09902	2.22148	-0.97094
C	0.52618	0.45187	-1.08634
C	1.30208	0.61518	0.24062
H	1.32148	1.68918	0.50396
H	0.75162	0.10556	1.03968
C	4.58597	-0.73410	1.46749
C	3.21761	-0.06758	1.54359
N	2.64243	0.04047	0.20216
C	3.55388	0.78508	-0.66761
C	4.91805	0.10506	-0.69042
O	5.46833	-0.00512	0.61884
H	4.47161	-1.76387	1.09291
H	5.05592	-0.76688	2.45431
H	3.32517	0.92519	2.02188
H	2.55003	-0.67026	2.16988
H	3.15267	0.81046	-1.68542
H	3.67044	1.83155	-0.32466
H	4.81958	-0.89578	-1.14054
H	5.62951	0.68883	-1.28086
C	-3.99987	1.51238	0.06986
H	-3.81977	2.10139	-0.83613

H	-5.07826	1.31917	0.11545
C	-3.57813	2.34653	1.29037
H	-2.51356	2.59091	1.22843
H	-4.14320	3.28490	1.33864
H	-3.75292	1.79316	2.22009
C	-3.72965	-1.04267	0.29079
C	-5.06427	-1.51004	0.77072
H	-5.49148	-2.27198	0.10630
H	-5.00630	-1.94794	1.77557
H	-5.76593	-0.67356	0.81385
H	0.98144	1.09862	-1.84354

4s_Renantiomer_conformer-004

C	3.19111	0.07485	-0.39632
N	2.59700	-1.96683	0.31058
H	2.65421	-2.95251	0.52879
C	1.52034	-1.17962	0.55737
C	1.84400	0.10971	0.13063
C	-0.48854	-0.36335	1.71848
C	0.22518	-1.60694	1.16191
H	-1.51297	-0.62109	1.99963
H	0.03376	-0.03070	2.62352
H	-0.40709	-2.09456	0.40612
H	0.38535	-2.34686	1.95498
C	0.88386	1.19035	0.21037
O	1.10879	2.35226	-0.14654
C	-0.51523	0.79856	0.71006
C	-1.37333	0.48413	-0.53652
H	-1.40519	1.38620	-1.17558
H	-0.87780	-0.29867	-1.12209
C	-4.73527	-1.13103	-0.98375
C	-3.37255	-0.57662	-1.38135
N	-2.71099	0.00120	-0.21080
C	-3.56109	1.03825	0.37528
C	-4.92339	0.45205	0.72796
O	-5.55804	-0.11729	-0.41313
H	-4.60005	-1.95348	-0.26321
H	-5.26864	-1.51176	-1.85913
H	-3.50782	0.17327	-2.18467
H	-2.74930	-1.38529	-1.77979
H	-3.09451	1.42513	1.28640
H	-3.69574	1.88807	-0.32187
H	-4.79950	-0.31501	1.50915
H	-5.59285	1.23144	1.10211
C	3.96350	1.24294	-0.93733

H	4.82046	0.88187	-1.51831
H	3.32747	1.80901	-1.62690
C	4.45707	2.19180	0.16656
H	3.60533	2.59709	0.72066
H	5.10950	1.66424	0.87167
H	5.02016	3.03055	-0.25957
C	3.62985	-1.22495	-0.26822
C	4.92770	-1.87552	-0.61787
H	4.78843	-2.71848	-1.30667
H	5.59418	-1.15664	-1.10045
H	5.44275	-2.26161	0.27129
H	-0.91761	1.69174	1.19923

4s_Renantiomer_conformer-005

C	2.76812	0.58599	0.26594
N	3.14459	-1.22172	-1.00440
H	3.60093	-1.85498	-1.64722
C	1.94059	-1.44223	-0.42085
C	1.66097	-0.33818	0.38419
C	-0.37098	-2.24152	-0.27153
C	1.07987	-2.65025	-0.56695
H	-0.74198	-1.61968	-1.09187
H	-1.01197	-3.12949	-0.23629
H	1.16523	-3.07868	-1.57242
H	1.40230	-3.43265	0.13658
C	0.47100	-0.28640	1.20573
O	0.24805	0.60525	2.03233
C	-0.51296	-1.46786	1.05204
C	-1.96701	-1.05029	1.32707
H	-2.53696	-1.94747	1.59365
H	-1.97688	-0.37928	2.20229
C	-2.82245	1.36457	-1.45496
C	-2.06765	0.82985	-0.24344
N	-2.64366	-0.44903	0.17666
C	-4.07218	-0.28214	0.44542
C	-4.77712	0.27500	-0.78497
O	-4.21208	1.52065	-1.18331
H	-2.68350	0.67701	-2.30494
H	-2.44522	2.35187	-1.73579
H	-2.09445	1.57626	0.57186
H	-1.01981	0.68795	-0.52401
H	-4.50296	-1.25655	0.70344
H	-4.24740	0.39916	1.30099
H	-4.70317	-0.45048	-1.61098
H	-5.83350	0.46274	-0.57337

C	2.87110	1.93295	0.92062
H	2.64222	1.83614	1.98757
H	3.90078	2.30220	0.84648
C	1.91229	2.96469	0.30423
H	0.87610	2.63175	0.41719
H	2.01402	3.93882	0.79693
H	2.11717	3.09802	-0.76411
C	3.66683	0.01224	-0.60671
C	4.97691	0.48850	-1.14210
H	4.94346	0.63738	-2.22916
H	5.24907	1.44298	-0.68522
H	5.78476	-0.22477	-0.93467
H	-0.23651	-2.14984	1.87264

4s_Renantiomer_conformer-006

C	2.71185	-0.58547	0.27246
N	2.98791	1.57959	0.78146
H	3.39151	2.42292	1.16665
C	1.81993	1.52231	0.09503
C	1.60560	0.18771	-0.24949
C	-0.49337	2.12473	-0.44580
C	0.93187	2.66571	-0.25848
H	-0.90038	1.84179	0.52968
H	-1.14505	2.91144	-0.84182
H	0.95495	3.43984	0.51743
H	1.27893	3.14147	-1.18816
C	0.45291	-0.21502	-1.02642
O	0.27508	-1.36546	-1.44236
C	-0.54545	0.90817	-1.38794
C	-1.97680	0.38183	-1.58089
H	-2.52142	1.09559	-2.20915
H	-1.92624	-0.57258	-2.13118
C	-3.04978	-0.81347	1.84239
C	-2.20800	-0.76575	0.57248
N	-2.73902	0.25040	-0.33802
C	-4.14725	-0.02859	-0.62054
C	-4.94162	-0.08991	0.67820
O	-4.41909	-1.08271	1.55617
H	-2.96186	0.14674	2.37605
H	-2.70434	-1.61491	2.50139
H	-2.18906	-1.76528	0.10047
H	-1.17963	-0.51277	0.84683
H	-4.54838	0.76881	-1.25684
H	-4.27051	-0.98564	-1.16440
H	-4.91718	0.89537	1.17084

H	-5.98285	-0.36077	0.48210
C	2.91604	-2.06516	0.11983
H	3.61679	-2.42196	0.88425
H	1.96522	-2.58070	0.29388
C	3.43840	-2.45312	-1.27270
H	2.72057	-2.14744	-2.03946
H	4.39706	-1.96403	-1.48008
H	3.58330	-3.53728	-1.34965
C	3.54942	0.30628	0.90653
C	4.83856	0.11301	1.63488
H	5.66868	0.63356	1.14009
H	4.78487	0.48854	2.66475
H	5.09311	-0.94869	1.67904
H	-0.21210	1.24182	-2.38445

4s_Renantiomer_conformer-007

C	-3.04906	0.65753	0.26383
N	-3.62503	-1.34408	-0.56341
H	-4.19821	-2.09994	-0.91355
C	-2.28679	-1.41715	-0.35390
C	-1.87854	-0.18639	0.15803
C	-0.13419	-2.44580	0.23649
C	-1.40374	-2.58877	-0.61704
H	-0.38110	-2.63905	1.28873
H	0.60070	-3.19920	-0.06637
H	-1.91769	-3.53161	-0.39599
H	-1.12814	-2.62336	-1.68199
C	-0.49335	0.07251	0.49265
O	-0.10261	1.12583	1.00735
C	0.50132	-1.04465	0.11761
C	1.82862	-0.95142	0.89415
H	1.68926	-0.33815	1.80015
H	2.11482	-1.95588	1.22517
C	5.35544	-0.21778	-0.18925
C	4.22552	-0.67520	0.72664
N	2.93554	-0.45072	0.07403
C	2.80611	0.97030	-0.25844
C	3.96055	1.39156	-1.15903
O	5.22054	1.15786	-0.53379
H	5.35692	-0.83403	-1.10268
H	6.32371	-0.32250	0.30841
H	4.29755	-0.13158	1.68916
H	4.33894	-1.74478	0.93895
H	1.85692	1.14382	-0.77207
H	2.80408	1.59799	0.65174

H	3.90688	0.83776	-2.11020
H	3.91312	2.46327	-1.37199
C	-3.07786	2.08326	0.73357
H	-4.10851	2.37314	0.97026
H	-2.50074	2.17031	1.66058
C	-2.49661	3.06119	-0.30027
H	-3.05449	3.01044	-1.24227
H	-2.53916	4.09299	0.06814
H	-1.45059	2.81395	-0.50441
C	-4.11332	-0.08803	-0.19505
C	-5.56453	0.23353	-0.33827
H	-5.77144	1.23392	0.04937
H	-5.88615	0.21030	-1.38750
H	-6.19482	-0.47628	0.21249
H	0.73381	-0.88552	-0.94732

4s_Renantiomer_conformer-008

C	3.02644	-0.69252	-0.22957
N	3.56074	1.46497	-0.51583
H	4.11351	2.29350	-0.69032
C	2.23635	1.46219	-0.22266
C	1.85492	0.13435	-0.03509
C	0.10952	2.26549	0.70815
C	1.33877	2.64862	-0.12975
H	0.40357	2.17402	1.76204
H	-0.64010	3.06184	0.64996
H	1.85877	3.50742	0.31083
H	1.01597	2.95889	-1.13516
C	0.48320	-0.22523	0.25977
O	0.11298	-1.38561	0.46908
C	-0.52841	0.93859	0.24664
C	-1.81571	0.62155	1.03243
H	-1.63829	-0.22670	1.71455
H	-2.07217	1.48701	1.65334
C	-5.40100	0.22756	-0.03700
C	-4.22267	0.40748	0.91382
N	-2.96845	0.37508	0.16137
C	-2.87559	-0.89475	-0.56325
C	-4.07692	-1.04424	-1.48831
O	-5.30246	-0.99534	-0.76107
H	-5.43660	1.07629	-0.73882
H	-6.34312	0.18833	0.51710
H	-4.25723	-0.38552	1.68654
H	-4.31025	1.37392	1.42396
H	-1.95351	-0.91756	-1.15005

H	-2.84085	-1.75323	0.13284
H	-4.05994	-0.24466	-2.24636
H	-4.05535	-2.01245	-1.99653
C	3.09460	-2.18627	-0.09489
H	2.24178	-2.63551	-0.61562
H	4.00099	-2.56006	-0.58596
C	3.07731	-2.65252	1.36968
H	3.92585	-2.23371	1.92265
H	2.15302	-2.32618	1.85532
H	3.13218	-3.74568	1.43445
C	4.06603	0.16261	-0.52443
C	5.51002	-0.08953	-0.80952
H	6.16058	0.35840	-0.04721
H	5.71113	-1.16338	-0.82660
H	5.81159	0.32381	-1.78037
H	-0.81298	1.06822	-0.80948

4s_Renantiomer_conformer-009

C	-3.03681	0.60049	0.14866
N	-3.40928	-1.47846	-0.60031
H	-3.89970	-2.28908	-0.95337
C	-2.09402	-1.44970	-0.26966
C	-1.81126	-0.16724	0.19892
C	0.04877	-2.30178	0.57596
C	-1.11998	-2.57257	-0.38355
H	-0.28937	-2.45370	1.60959
H	0.85348	-3.02208	0.39257
H	-1.59507	-3.53490	-0.15936
H	-0.74096	-2.64195	-1.41459
C	-0.47879	0.20315	0.63018
O	-0.19466	1.31581	1.08778
C	0.60763	-0.87230	0.43616
C	1.83577	-0.63340	1.35517
H	1.55613	0.07446	2.14049
H	2.10441	-1.57950	1.84054
C	4.25419	1.69459	-0.36680
C	2.89680	1.20059	0.11987
N	3.04913	-0.12445	0.71821
C	3.64112	-1.05327	-0.24046
C	4.98111	-0.50376	-0.71987
O	4.82907	0.78666	-1.30511
H	4.93154	1.81972	0.49310
H	4.15636	2.65408	-0.88263
H	2.19397	1.19571	-0.73418
H	2.49034	1.88565	0.86935

H	3.79338	-2.02309	0.24824
H	2.99468	-1.22122	-1.12319
H	5.67989	-0.44877	0.13014
H	5.41187	-1.14828	-1.49137
C	-3.19873	2.04487	0.52526
H	-4.26197	2.27227	0.66754
H	-2.70253	2.22624	1.48482
C	-2.60784	3.00202	-0.52245
H	-1.53543	2.81828	-0.63682
H	-3.08908	2.85919	-1.49668
H	-2.74645	4.04727	-0.22171
C	-4.00636	-0.23978	-0.35452
C	-5.45543	-0.02556	-0.64510
H	-5.67532	-0.12037	-1.71638
H	-6.08789	-0.75005	-0.11636
H	-5.76125	0.97557	-0.33179
H	0.92753	-0.75291	-0.61131

4s_Renantiomer_conformer-010

C	-2.99298	0.58732	-0.33820
N	-3.32808	-1.62126	-0.51706
H	-3.79671	-2.50310	-0.67553
C	-2.03511	-1.48778	-0.12921
C	-1.77754	-0.12326	-0.00383
C	0.06708	-2.05110	1.00825
C	-1.05488	-2.58612	0.10581
H	-0.31943	-1.92336	2.02810
H	0.88016	-2.78319	1.06287
H	-1.53822	-3.45980	0.55892
H	-0.62838	-2.92415	-0.85091
C	-0.46414	0.36809	0.35935
O	-0.19988	1.56752	0.49976
C	0.63116	-0.70452	0.51427
C	1.81028	-0.20921	1.39504
H	1.49225	0.68389	1.94028
H	2.04870	-0.98295	2.13489
C	4.32948	1.58446	-0.76562
C	2.94515	1.22885	-0.23600
N	3.05810	0.11849	0.70796
C	3.69561	-1.03072	0.07135
C	5.06244	-0.62016	-0.46767
O	4.94918	0.46092	-1.38918
H	4.96008	1.94518	0.06270
H	4.26496	2.36636	-1.52769
H	2.28946	0.98476	-1.09268

H	2.50259	2.08846	0.27542
H	3.81589	-1.82942	0.81307
H	3.09640	-1.43840	-0.76534
H	5.71507	-0.32971	0.37111
H	5.53022	-1.44624	-1.01077
C	-3.19155	2.07529	-0.30921
H	-2.33733	2.56340	-0.79096
H	-4.08145	2.34022	-0.89260
C	-3.33285	2.62918	1.11769
H	-2.42848	2.41464	1.69473
H	-3.48172	3.71540	1.10423
H	-4.18699	2.17280	1.63085
C	-3.93436	-0.36973	-0.64965
C	-5.36747	-0.25984	-1.05488
H	-5.65179	0.78977	-1.16160
H	-5.55888	-0.75843	-2.01363
H	-6.03744	-0.71177	-0.31196
H	1.00461	-0.86695	-0.50937

4s_Renantiomer_conformer-011

C	2.77513	0.77968	0.12281
N	3.30602	-1.21106	-0.75844
H	3.83562	-1.93166	-1.23028
C	2.10921	-1.40080	-0.14876
C	1.73335	-0.18065	0.41386
C	0.32786	-2.56513	1.08584
C	1.34174	-2.67772	-0.06824
H	-0.37154	-3.40763	1.05321
H	0.86887	-2.62929	2.03739
H	0.81923	-2.86690	-1.01664
H	2.01048	-3.53184	0.09092
C	0.49517	-0.04113	1.15162
O	0.19657	0.96562	1.80479
C	-0.46420	-1.23917	1.04738
C	-1.34706	-1.16646	-0.22818
H	-0.78717	-0.67897	-1.04926
H	-1.55094	-2.19141	-0.55823
C	-4.90934	-0.17983	-0.88746
C	-3.53972	-0.79508	-1.15025
N	-2.64780	-0.53348	-0.01843
C	-2.55462	0.91215	0.20340
C	-3.94445	1.49280	0.42917
O	-4.81344	1.22412	-0.66867
H	-5.36662	-0.66551	-0.01043
H	-5.56678	-0.31924	-1.75025

H	-3.13325	-0.38001	-2.09335
H	-3.64567	-1.87835	-1.28104
H	-1.92902	1.10621	1.07698
H	-2.08465	1.41821	-0.66413
H	-4.37186	1.07229	1.35376
H	-3.89447	2.58076	0.52840
C	2.76377	2.23490	0.49125
H	2.50825	2.33831	1.55136
H	3.76707	2.65744	0.36105
C	1.75570	3.04634	-0.33913
H	0.74221	2.66414	-0.18325
H	1.77033	4.10389	-0.05049
H	1.98808	2.97943	-1.40804
C	3.73129	0.11172	-0.61067
C	5.01995	0.56541	-1.21338
H	5.86619	-0.04140	-0.86703
H	5.00145	0.50547	-2.30924
H	5.22099	1.60439	-0.94142
H	-1.13210	-1.19670	1.91356

4s_Renantiomer_conformer-012

C	-3.29298	0.42721	-0.37687
N	-3.34102	-1.79334	-0.07901
H	-3.69937	-2.73549	0.00051
C	-2.04586	-1.43735	0.10852
C	-1.96454	-0.05654	-0.06775
C	0.21364	-1.50128	1.06104
C	-0.90924	-2.34359	0.43816
H	-0.09231	-1.17707	2.06410
H	1.11130	-2.11434	1.18673
H	-1.22357	-3.14222	1.12037
H	-0.53839	-2.83666	-0.47332
C	-0.70178	0.64650	0.02517
O	-0.59357	1.87118	-0.09061
C	0.54395	-0.25330	0.21751
C	1.74327	0.54504	0.75378
H	1.49277	1.60804	0.70808
H	1.91204	0.29135	1.81889
C	5.23827	1.13918	-0.49337
C	3.99613	1.31164	0.37237
N	2.96363	0.35674	-0.03317
C	3.49679	-1.00318	0.05354
C	4.75428	-1.12697	-0.79986
O	5.74935	-0.18813	-0.40362
H	6.03812	1.80611	-0.15979

H	4.99214	1.37528	-1.54096
H	3.60536	2.32903	0.25576
H	4.27742	1.17959	1.43571
H	3.73594	-1.27744	1.10026
H	2.75093	-1.71414	-0.31514
H	5.19833	-2.12006	-0.68856
H	4.49354	-0.97141	-1.85899
C	-3.68882	1.85599	-0.61356
H	-2.96154	2.32663	-1.28412
H	-4.65980	1.89022	-1.12194
C	-3.76324	2.67535	0.68487
H	-2.78593	2.68945	1.17630
H	-4.05909	3.71090	0.47923
H	-4.49295	2.24206	1.37837
C	-4.12200	-0.67358	-0.37472
C	-5.59014	-0.81278	-0.60946
H	-6.02424	0.15305	-0.87894
H	-5.80753	-1.51702	-1.42256
H	-6.11358	-1.17454	0.28503
H	0.79962	-0.58356	-0.80216

4s_Renantiomer_conformer-013

C	3.32522	0.48975	-0.09821
N	3.39132	-1.73008	0.20225
H	3.76100	-2.66114	0.34005
C	2.08028	-1.43500	0.01810
C	1.98689	-0.05622	-0.16832
C	-0.22323	-1.75355	-0.76255
C	0.94095	-2.39622	0.00577
H	0.02840	-1.71246	-1.83019
H	-1.11722	-2.37804	-0.67206
H	1.23081	-3.35012	-0.45026
H	0.62300	-2.62332	1.03473
C	0.71196	0.59485	-0.38722
O	0.59327	1.80519	-0.60170
C	-0.52950	-0.32409	-0.27185
C	-1.76213	0.30025	-0.94561
H	-1.52330	1.33635	-1.19879
H	-1.97548	-0.23047	-1.89436
C	-4.68012	-0.89279	1.13715
C	-3.46244	-1.00278	0.22604
N	-2.94355	0.33049	-0.08118
C	-4.00515	1.14013	-0.68128
C	-5.20626	1.20642	0.25421
O	-5.70349	-0.09643	0.54803

H	-4.37585	-0.45714	2.10233
H	-5.11564	-1.87945	1.31768
H	-3.74318	-1.54940	-0.69612
H	-2.69221	-1.58747	0.73850
H	-3.62286	2.15158	-0.86077
H	-4.33068	0.72623	-1.65593
H	-4.91820	1.71612	1.18751
H	-6.02831	1.75824	-0.21006
C	3.70352	1.93875	-0.20613
H	4.77741	2.02523	-0.41016
H	3.17990	2.38762	-1.05705
C	3.35913	2.73835	1.06079
H	2.28191	2.70056	1.24849
H	3.87668	2.32643	1.93464
H	3.65166	3.78953	0.95323
C	4.17088	-0.57278	0.13592
C	5.65200	-0.64438	0.31220
H	6.11353	-1.33600	-0.40409
H	6.10007	0.34068	0.16127
H	5.92756	-0.98435	1.31887
H	-0.73350	-0.36624	0.81029

4s_Renantiomer_conformer-014

C	2.74204	0.67371	-0.36131
N	3.16008	-1.50849	-0.65407
H	3.63790	-2.35777	-0.92443
C	1.98384	-1.45997	0.01962
C	1.67978	-0.11379	0.22596
C	0.20592	-2.13999	1.57537
C	1.16683	-2.62283	0.47330
H	-0.52504	-2.92298	1.80488
H	0.78288	-1.95662	2.48958
H	0.59998	-3.04218	-0.37003
H	1.80478	-3.43175	0.84824
C	0.46437	0.28911	0.90199
O	0.21186	1.45758	1.21913
C	-0.53755	-0.84485	1.18196
C	-1.48153	-1.09685	-0.02615
H	-0.96580	-0.84507	-0.97270
H	-1.70098	-2.16959	-0.07004
C	-5.08011	-0.27022	-0.71317
C	-3.72741	-0.95095	-0.88884
N	-2.76982	-0.41479	0.08037
C	-2.66582	1.03677	-0.09108
C	-4.04015	1.67516	0.06153

O	-4.97541	1.14108	-0.87287
H	-5.48062	-0.50560	0.28600
H	-5.78929	-0.62007	-1.46866
H	-3.38088	-0.80132	-1.93020
H	-3.83820	-2.02936	-0.72584
H	-1.98318	1.44337	0.65833
H	-2.25548	1.29058	-1.08913
H	-4.40722	1.51563	1.08825
H	-3.98812	2.75065	-0.12985
C	2.84650	2.17140	-0.35531
H	3.54645	2.49566	-1.13465
H	1.87053	2.60221	-0.60547
C	3.29771	2.73151	1.00309
H	2.57632	2.45649	1.77801
H	4.27870	2.33146	1.28382
H	3.36981	3.82517	0.97220
C	3.64215	-0.22024	-0.89912
C	4.93069	-0.01699	-1.62593
H	4.92119	-0.49588	-2.61329
H	5.11820	1.04933	-1.77329
H	5.78218	-0.43045	-1.07009
H	-1.16095	-0.52403	2.02260

4s_Renantiomer_conformer-015

C	-3.25657	0.21163	-0.02768
N	-2.68791	-1.95243	0.09283
H	-2.76717	-2.95095	0.23110
C	-1.57101	-1.31657	-0.34087
C	-1.87642	0.04298	-0.42738
C	0.52220	-1.00050	-1.59490
C	-0.25827	-1.94615	-0.66602
H	1.55286	-1.35068	-1.69548
H	0.06222	-1.02249	-2.59016
H	0.31698	-2.12589	0.25356
H	-0.39693	-2.92475	-1.14043
C	-0.88590	1.00965	-0.85161
O	-1.09897	2.22141	-0.97094
C	0.52615	0.45173	-1.08639
C	1.30207	0.61498	0.24058
H	1.32151	1.68898	0.50394
H	0.75161	0.10536	1.03964
C	4.58609	-0.73388	1.46759
C	3.21758	-0.06767	1.54357
N	2.64242	0.04027	0.20212
C	3.55380	0.78486	-0.66774

C	4.91813	0.10519	-0.69037
O	5.46835	-0.00479	0.61893
H	4.47199	-1.76371	1.09309
H	5.05598	-0.76649	2.45444
H	3.32488	0.92512	2.02187
H	2.55010	-0.67049	2.16985
H	3.15265	0.80997	-1.68557
H	3.67010	1.83143	-0.32501
H	4.81998	-0.89570	-1.14045
H	5.62948	0.68910	-1.28079
C	-3.99975	1.51249	0.06978
H	-3.81943	2.10143	-0.83621
H	-5.07815	1.31934	0.11522
C	-3.57813	2.34681	1.29023
H	-3.75283	1.79356	2.22003
H	-2.51360	2.59138	1.22824
H	-4.14336	3.28509	1.33838
C	-3.72964	-1.04261	0.29092
C	-5.06423	-1.51001	0.77089
H	-5.00605	-1.94877	1.77535
H	-5.76566	-0.67339	0.81494
H	-5.49186	-2.27127	0.10596
H	0.98141	1.09849	-1.84358

4s_Renantiomer_conformer-016

C	2.27112	-0.98734	-0.37467
N	3.23480	0.57199	0.91306
H	3.90052	1.01843	1.52920
C	2.15717	1.18526	0.35737
C	1.52368	0.24613	-0.45532
C	0.70646	3.00221	-0.49972
C	1.73453	2.60015	0.57795
H	0.19481	3.92227	-0.19560
H	1.23984	3.22625	-1.43084
H	1.30047	2.70942	1.58165
H	2.59554	3.27862	0.54640
C	0.33967	0.58956	-1.21619
O	-0.12721	-0.10116	-2.12703
C	-0.33390	1.89924	-0.79123
C	-1.22635	1.64307	0.44314
H	-0.59088	1.54295	1.32878
H	-1.86402	2.53450	0.60492
C	-3.75874	-0.88058	-0.75616
C	-3.01567	0.44916	-0.70929
N	-2.01299	0.41798	0.35623

C	-2.64834	0.09942	1.63377
C	-3.38924	-1.22790	1.52655
O	-4.36972	-1.18936	0.49399
H	-3.05404	-1.68030	-1.03475
H	-4.56293	-0.84624	-1.49643
H	-3.74125	1.27155	-0.55455
H	-2.51400	0.60376	-1.66712
H	-1.87582	0.02625	2.40810
H	-3.36204	0.88797	1.94313
H	-2.66642	-2.03534	1.32700
H	-3.92067	-1.45094	2.45598
C	1.88764	-2.28322	-1.02790
H	1.67502	-2.10601	-2.08762
H	2.72586	-2.98854	-0.98168
C	0.64647	-2.91581	-0.37477
H	0.83971	-3.15019	0.67844
H	-0.20052	-2.22398	-0.41902
H	0.36160	-3.84332	-0.88534
C	3.32128	-0.75549	0.48707
C	4.41489	-1.64323	0.98293
H	4.35415	-1.79994	2.06768
H	4.35468	-2.62269	0.50217
H	5.40679	-1.22541	0.76828
H	-0.95765	2.22171	-1.63071

4s_Renantiomer_conformer-017

C	2.71201	-0.58543	0.27252
N	2.98806	1.57971	0.78116
H	3.39169	2.42310	1.16617
C	1.82003	1.52229	0.09484
C	1.60570	0.18764	-0.24943
C	-0.49332	2.12457	-0.44597
C	0.93192	2.66561	-0.25878
H	-1.14503	2.91120	-0.84209
H	-0.90029	1.84178	0.52956
H	0.95501	3.43986	0.51701
H	1.27892	3.14125	-1.18856
C	0.45293	-0.21528	-1.02616
O	0.27504	-1.36583	-1.44175
C	-0.54539	0.90787	-1.38792
C	-1.97674	0.38154	-1.58090
H	-2.52127	1.09520	-2.20935
H	-1.92617	-0.57297	-2.13100
C	-3.05009	-0.81305	1.84251
C	-2.20818	-0.76557	0.57269

N	-2.73908	0.25040	-0.33808
C	-4.14728	-0.02865	-0.62070
C	-4.94180	-0.08972	0.67796
O	-4.41937	-1.08235	1.55618
H	-2.96225	0.14726	2.37599
H	-2.70473	-1.61436	2.50170
H	-2.18916	-1.76519	0.10088
H	-1.17984	-0.51254	0.84711
H	-4.54835	0.76863	-1.25720
H	-4.27049	-0.98580	-1.16439
H	-4.91742	0.89565	1.17042
H	-5.98301	-0.36061	0.48179
C	2.91622	-2.06514	0.12012
H	3.61721	-2.42176	0.88441
H	1.96548	-2.58071	0.29452
C	3.43821	-2.45333	-1.27249
H	4.39677	-1.96420	-1.48024
H	3.58319	-3.53749	-1.34926
H	2.72012	-2.14788	-2.03910
C	3.54962	0.30643	0.90637
C	4.83882	0.11331	1.63467
H	5.09341	-0.94838	1.67901
H	5.66889	0.63379	1.13975
H	4.78517	0.48901	2.66448
H	-0.21196	1.24136	-2.38445

4s_Renantiomer_conformer-018

C	2.40351	0.85178	0.38950
N	3.29009	-0.84213	-0.77886
H	3.95461	-1.37994	-1.31885
C	2.10100	-1.31003	-0.32231
C	1.50394	-0.28086	0.40759
C	0.46318	-2.94259	0.52971
C	1.54917	-2.68062	-0.53179
H	-0.09513	-3.85021	0.27338
H	0.95265	-3.12835	1.49305
H	1.12897	-2.77264	-1.54292
H	2.33983	-3.43682	-0.45995
C	0.21855	-0.46490	1.04930
O	-0.29241	0.35631	1.82100
C	-0.51652	-1.76059	0.69163
C	-1.38665	-1.61902	-0.59386
H	-0.73552	-1.43549	-1.45531
H	-1.85531	-2.60124	-0.75050
C	-3.27007	1.58023	-1.25677

C	-2.02612	0.74689	-0.96823
N	-2.43884	-0.61547	-0.63324
C	-3.34822	-0.62282	0.50953
C	-4.55335	0.25818	0.19463
O	-4.15168	1.58621	-0.13530
H	-3.79152	1.17570	-2.13915
H	-3.00327	2.62378	-1.44792
H	-1.46201	1.22927	-0.15693
H	-1.38641	0.71813	-1.85802
H	-3.68558	-1.65061	0.69232
H	-2.85923	-0.25636	1.42741
H	-5.11949	-0.17850	-0.64382
H	-5.21309	0.34021	1.06330
C	2.16119	2.19361	1.01890
H	1.71432	2.05039	2.00765
H	3.11761	2.70890	1.16911
C	1.23242	3.08692	0.18071
H	0.25591	2.61010	0.05121
H	1.65672	3.26586	-0.81380
H	1.07602	4.05718	0.66658
C	3.49652	0.47392	-0.35977
C	4.73672	1.20994	-0.74656
H	5.64198	0.66203	-0.45560
H	4.79082	1.37912	-1.82979
H	4.76513	2.18664	-0.25729
H	-1.18568	-1.97426	1.53164

4s_Renantiomer_conformer-019

C	2.34808	-0.87547	0.19682
N	3.06701	1.10095	0.96913
H	3.64160	1.78653	1.44044
C	1.96216	1.38750	0.23390
C	1.47636	0.18000	-0.26875
C	0.42050	2.67897	-1.20101
C	1.38457	2.74375	0.00001
H	-0.18348	3.59246	-1.24083
H	1.01036	2.64441	-2.12431
H	0.85668	3.09194	0.89872
H	2.17510	3.47929	-0.19104
C	0.29078	0.13464	-1.09942
O	-0.09122	-0.86887	-1.71049
C	-0.50578	1.44491	-1.16820
C	-1.48896	1.53711	0.01843
H	-0.92548	1.73924	0.93555
H	-2.14928	2.41127	-0.15030

C	-3.86928	-1.33451	-0.52797
C	-3.14252	-0.03417	-0.84867
N	-2.25232	0.31866	0.25818
C	-3.01618	0.41125	1.50167
C	-3.73926	-0.90396	1.76638
O	-4.60669	-1.24229	0.68832
H	-3.13289	-2.15095	-0.45800
H	-4.59107	-1.57666	-1.31300
H	-3.88433	0.76579	-1.04095
H	-2.54739	-0.18124	-1.75246
H	-2.32852	0.62379	2.32865
H	-3.75987	1.23153	1.46354
H	-2.99817	-1.70530	1.91655
H	-4.36431	-0.82881	2.66065
C	2.21939	-2.33740	-0.12013
H	2.77301	-2.92654	0.62084
H	1.16712	-2.63226	-0.03844
C	2.72037	-2.68750	-1.53041
H	2.13744	-2.14212	-2.27812
H	3.77605	-2.41732	-1.64799
H	2.61736	-3.76099	-1.72871
C	3.32192	-0.27200	0.96286
C	4.49297	-0.83192	1.70119
H	4.46574	-0.57186	2.76703
H	4.50352	-1.92179	1.62311
H	5.44428	-0.46077	1.29839
H	-1.07363	1.41505	-2.10329

4s_Renantiomer_conformer-020

C	3.23532	0.54086	0.27637
N	3.43559	-1.67688	0.02440
H	3.86087	-2.58912	-0.07278
C	2.10183	-1.43345	-0.01837
C	1.92572	-0.05833	0.13528
C	-0.23367	-1.71410	-0.71345
C	1.01336	-2.43881	-0.18661
H	-0.06031	-1.41319	-1.75564
H	-1.09934	-2.38122	-0.70939
H	1.31776	-3.24204	-0.86811
H	0.78505	-2.91622	0.77852
C	0.60442	0.53423	0.17976
O	0.40156	1.74774	0.30304
C	-0.57008	-0.46115	0.11274
C	-1.84295	0.23447	-0.38947
H	-1.96924	1.17518	0.16209

H	-1.69308	0.51059	-1.43991
C	-4.41847	0.43590	1.43435
C	-3.58685	-0.78049	1.02010
N	-3.01408	-0.64893	-0.32735
C	-4.08020	-0.29093	-1.27036
C	-4.89731	0.92089	-0.81872
O	-5.44907	0.69662	0.47995
H	-3.77926	1.32671	1.54211
H	-4.91891	0.25794	2.39111
H	-4.23656	-1.66536	1.02583
H	-2.78640	-0.95556	1.74610
H	-3.64257	-0.11126	-2.25835
H	-4.75848	-1.15093	-1.35392
H	-4.27134	1.82802	-0.80703
H	-5.74435	1.09643	-1.48917
C	3.53265	2.00447	0.42957
H	2.85787	2.43491	1.17760
H	4.55365	2.13627	0.80727
C	3.36961	2.78410	-0.88506
H	4.03934	2.39017	-1.65802
H	2.33984	2.70088	-1.24500
H	3.59865	3.84685	-0.74242
C	4.14841	-0.48849	0.20123
C	5.64036	-0.50082	0.26395
H	6.01983	0.50405	0.46433
H	6.00885	-1.16307	1.05770
H	6.08570	-0.84233	-0.67950
H	-0.73088	-0.78192	1.15543

4s_Renantiomer_conformer-021

C	3.24011	0.52900	-0.25382
N	3.44204	-1.65203	0.22035
H	3.86680	-2.55474	0.38538
C	2.10642	-1.41694	0.19065
C	1.92935	-0.06358	-0.09671
C	-0.25966	-1.88738	-0.25419
C	1.01804	-2.41053	0.41820
H	-0.14213	-1.94916	-1.34465
H	-1.11944	-2.50649	0.01414
H	1.29297	-3.39623	0.02457
H	0.84263	-2.54109	1.49692
C	0.60764	0.52204	-0.19073
O	0.40268	1.70945	-0.46783
C	-0.56254	-0.42871	0.12766
C	-1.86252	0.07519	-0.51448

H	-1.96675	1.14704	-0.30133
H	-1.76697	-0.01513	-1.60298
C	-4.35353	0.90604	1.24702
C	-3.53220	-0.38545	1.23310
N	-3.02342	-0.72443	-0.10372
C	-4.13427	-0.69937	-1.06237
C	-4.93934	0.60043	-1.01249
O	-5.42884	0.83569	0.30903
H	-3.71707	1.77525	1.01650
H	-4.80826	1.06780	2.22913
H	-4.17354	-1.21036	1.56986
H	-2.69735	-0.30932	1.93720
H	-3.74377	-0.86972	-2.07151
H	-4.80885	-1.53110	-0.81775
H	-4.32066	1.45222	-1.33897
H	-5.81782	0.54456	-1.66285
C	3.53706	1.97312	-0.53863
H	4.56806	2.07355	-0.89844
H	2.88266	2.32497	-1.34353
C	3.33515	2.87645	0.68860
H	2.29549	2.82628	1.02550
H	3.98222	2.56188	1.51537
H	3.56800	3.92092	0.44967
C	4.15489	-0.48098	-0.04821
C	5.64800	-0.49001	-0.07049
H	6.02911	0.49442	-0.35255
H	6.06940	-0.74095	0.91154
H	6.03973	-1.22008	-0.79027
H	-0.67119	-0.38642	1.22430

4s_Renantiomer_conformer-022

C	3.25486	0.40968	0.18170
N	3.13946	-1.66650	-0.65380
H	3.42435	-2.53661	-1.08299
C	1.89914	-1.41021	-0.16911
C	1.91952	-0.11897	0.35948
C	-0.26814	-1.88753	0.89526
C	0.72875	-2.33300	-0.19002
H	-1.20078	-2.44847	0.78268
H	0.14689	-2.14878	1.87618
H	0.24996	-2.31765	-1.17915
H	1.04033	-3.36970	-0.01622
C	0.73878	0.46921	0.95078
O	0.70055	1.59965	1.44921
C	-0.54867	-0.37051	0.87305

C	-1.36635	0.10248	-0.36001
H	-0.96099	1.05204	-0.72475
H	-1.25606	-0.62496	-1.18668
C	-4.95587	-0.52909	0.63506
C	-3.50042	-0.86591	0.32932
N	-2.77635	0.34646	-0.05545
C	-3.44741	0.97725	-1.19438
C	-4.90073	1.27571	-0.84720
O	-5.59586	0.08832	-0.47717
H	-4.99952	0.13882	1.51011
H	-5.52095	-1.43842	0.85730
H	-3.46077	-1.63175	-0.47035
H	-3.04594	-1.29530	1.22685
H	-2.92939	1.91158	-1.43960
H	-3.41737	0.33008	-2.09267
H	-4.94032	2.00527	-0.02274
H	-5.42632	1.69086	-1.71154
C	3.72646	1.78087	0.57062
H	3.38824	2.00429	1.58835
H	4.82262	1.80362	0.58631
C	3.20566	2.87766	-0.37206
H	2.11206	2.89907	-0.35378
H	3.57338	3.86450	-0.06700
H	3.53170	2.69396	-1.40216
C	3.98460	-0.57201	-0.45189
C	5.40635	-0.61778	-0.90605
H	5.92796	0.29616	-0.61194
H	5.94584	-1.46743	-0.46850
H	5.48341	-0.70850	-1.99722
H	-1.12702	-0.09306	1.76064

4s_Renantiomer_conformer-023

C	-3.19152	0.42236	0.31288
N	-3.04258	-1.81190	0.39921
H	-3.30354	-2.77830	0.54231
C	-1.83868	-1.39431	-0.06504
C	-1.88286	-0.00150	-0.13650
C	0.26278	-1.45782	-1.34606
C	-0.67694	-2.25639	-0.42457
H	1.19643	-2.01243	-1.47913
H	-0.20579	-1.37705	-2.33430
H	-0.14439	-2.57457	0.48264
H	-1.00660	-3.17498	-0.92429
C	-0.73273	0.76663	-0.55731
O	-0.71677	1.99899	-0.65083

C	0.55371	-0.03381	-0.82770
C	1.43340	0.00661	0.45163
H	1.05477	0.77802	1.13035
H	1.35478	-0.95512	0.99347
C	4.96640	-0.23150	-0.86629
C	3.52361	-0.66094	-0.62292
N	2.83009	0.34735	0.17994
C	3.55953	0.56346	1.43156
C	4.99780	0.97080	1.13646
O	5.66347	-0.01889	0.35711
H	4.97554	0.69161	-1.46757
H	5.51170	-1.00981	-1.40708
H	3.51401	-1.65094	-0.12561
H	3.02365	-0.76714	-1.58996
H	3.06253	1.35768	2.00046
H	3.56510	-0.34801	2.06071
H	5.00546	1.93487	0.60350
H	5.56721	1.07547	2.06409
C	-3.69391	1.83565	0.37421
H	-4.57768	1.88617	1.02133
H	-2.92712	2.47123	0.83069
C	-4.04075	2.40363	-1.01131
H	-4.81591	1.80055	-1.49772
H	-3.15182	2.40355	-1.64899
H	-4.40712	3.43404	-0.93273
C	-3.88583	-0.72286	0.63550
C	-5.27524	-0.93422	1.14020
H	-5.76435	0.02667	1.31714
H	-5.88935	-1.49272	0.42195
H	-5.28477	-1.49456	2.08369
H	1.08857	0.53211	-1.59778

4s_Renantiomer_conformer-024

C	-3.37088	0.29653	-0.23637
N	-3.12329	-1.92098	-0.02458
H	-3.35710	-2.90021	0.06982
C	-1.86238	-1.42235	-0.04863
C	-1.96373	-0.03873	-0.17933
C	0.52192	-1.25969	0.51930
C	-0.59126	-2.19480	0.02067
H	0.36555	-1.04990	1.58558
H	1.48194	-1.77295	0.43062
H	-0.68964	-3.06510	0.68004
H	-0.32896	-2.58409	-0.97470
C	-0.78800	0.79796	-0.29401

O	-0.84120	2.02284	-0.45786
C	0.57614	0.07262	-0.24998
C	1.65195	1.05242	0.27622
H	1.51488	1.99090	-0.26961
H	1.44888	1.26952	1.33228
C	5.01659	0.23994	-1.20541
C	3.49083	0.24907	-1.15404
N	3.05659	0.67282	0.16933
C	3.61707	-0.17794	1.21271
C	5.13882	-0.14827	1.10497
O	5.56452	-0.58937	-0.18331
H	5.39121	1.26954	-1.09104
H	5.37235	-0.16405	-2.15757
H	3.11957	-0.75933	-1.41898
H	3.10481	0.95170	-1.90218
H	3.30568	0.20227	2.19210
H	3.29291	-1.23106	1.14218
H	5.50297	0.87488	1.28938
H	5.59514	-0.82638	1.83188
C	-3.95442	1.67587	-0.34097
H	-3.43790	2.22824	-1.13355
H	-5.00893	1.60874	-0.63382
C	-3.83753	2.47229	0.96849
H	-2.78522	2.59091	1.24283
H	-4.27875	3.47031	0.86150
H	-4.35266	1.95606	1.78662
C	-4.06166	-0.89141	-0.13403
C	-5.52393	-1.19381	-0.11316
H	-5.80516	-1.89827	-0.90631
H	-5.83533	-1.63594	0.84210
H	-6.10294	-0.27874	-0.25885
H	0.80977	-0.15589	-1.30273

4s_Renantiomer_conformer-025

C	3.36557	0.27069	-0.22832
N	3.11783	-1.89590	0.28936
H	3.34948	-2.86085	0.48318
C	1.85856	-1.39484	0.24094
C	1.96047	-0.04125	-0.07414
C	-0.55271	-1.39429	-0.22646
C	0.58971	-2.13360	0.48842
H	-0.45258	-1.53872	-1.31023
H	-1.50409	-1.84119	0.07030
H	0.65777	-3.17040	0.13873
H	0.38125	-2.17964	1.56797

C	0.78759	0.80054	-0.18050
O	0.84246	2.01162	-0.42550
C	-0.57390	0.11471	0.07729
C	-1.68075	0.88757	-0.67870
H	-1.51608	1.94993	-0.47486
H	-1.53848	0.74786	-1.75757
C	-4.95587	0.65975	1.16462
C	-3.43526	0.62741	1.03207
N	-3.07607	0.58563	-0.37808
C	-3.69112	-0.55194	-1.05184
C	-5.20469	-0.46462	-0.87933
O	-5.55725	-0.45084	0.50306
H	-5.33913	1.60025	0.73791
H	-5.25731	0.59685	2.21418
H	-3.04746	-0.24360	1.59409
H	-3.01021	1.53074	1.48567
H	-3.43507	-0.52025	-2.11682
H	-3.36105	-1.52815	-0.65543
H	-5.58119	0.44654	-1.37073
H	-5.69872	-1.33685	-1.31706
C	3.95267	1.61736	-0.53882
H	4.98063	1.49666	-0.90119
H	3.38180	2.08311	-1.34948
C	3.94676	2.56300	0.67296
H	4.52029	2.13628	1.50375
H	4.38729	3.53355	0.41555
H	2.92022	2.73134	1.01130
C	4.05502	-0.89958	0.00460
C	5.51520	-1.21239	-0.00591
H	6.08889	-0.33444	-0.31254
H	5.87532	-1.51488	0.98594
H	5.75164	-2.02730	-0.70194
H	-0.75066	0.23765	1.15846

4s_Renantiomer_conformer-026

C	2.64188	0.72054	0.28628
N	3.34231	-1.22896	-0.56992
H	3.94043	-1.91513	-1.01024
C	2.07455	-1.46124	-0.14982
C	1.59288	-0.27053	0.39430
C	-0.17390	-2.43098	-0.22895
C	1.32881	-2.75008	-0.21433
H	-0.43426	-1.98500	-1.19342
H	-0.75594	-3.35517	-0.14228
H	1.61264	-3.32760	-1.10193

H	1.57376	-3.37391	0.65855
C	0.28040	-0.19498	0.99625
O	-0.13519	0.79736	1.60709
C	-0.58565	-1.47131	0.90199
C	-2.08891	-1.14812	0.88597
H	-2.63331	-2.03807	1.22141
H	-2.26614	-0.36016	1.63164
C	-4.59698	0.58109	0.11221
C	-4.07650	-0.73572	-0.47150
N	-2.61142	-0.83103	-0.44998
C	-2.05339	0.37026	-1.08747
C	-2.59370	1.66874	-0.48998
O	-4.02304	1.69856	-0.56673
H	-4.37267	0.64303	1.18908
H	-5.68086	0.66352	-0.01633
H	-4.40449	-0.80340	-1.51765
H	-4.50285	-1.58716	0.07108
H	-0.96166	0.34969	-1.04043
H	-2.33290	0.33490	-2.14906
H	-2.26650	1.77381	0.55387
H	-2.23681	2.53625	-1.05424
C	2.54658	2.15996	0.70158
H	2.16096	2.21713	1.72541
H	3.54702	2.60832	0.71147
C	1.62685	2.98244	-0.21565
H	0.60996	2.57944	-0.18971
H	1.58719	4.02983	0.10585
H	1.98308	2.95525	-1.25169
C	3.70951	0.09689	-0.32149
C	5.05722	0.59768	-0.72478
H	5.19661	0.56196	-1.81300
H	5.18710	1.63520	-0.40784
H	5.86304	0.00573	-0.27253
H	-0.40170	-1.98377	1.86065

4s_Renantiomer_conformer-027

C	-2.35916	-0.04844	0.05947
N	-1.53874	-0.32222	-2.00765
H	-1.44681	-0.65314	-2.95870
C	-0.73971	0.61035	-1.43120
C	-1.21094	0.81250	-0.13301
C	0.78421	2.53712	-1.25454
C	0.42859	1.27990	-2.06869
H	1.76624	2.91364	-1.56366
H	0.05453	3.32071	-1.49355

H	1.27821	0.58964	-2.08725
H	0.20384	1.55290	-3.10753
C	-0.53142	1.69980	0.78372
O	-0.90296	1.91966	1.94402
C	0.77279	2.32549	0.27253
C	1.99345	1.52517	0.79296
H	2.89458	2.04309	0.44610
H	2.00247	1.57315	1.89440
C	1.37336	-2.18391	0.59159
C	1.45542	-0.81701	1.25677
N	2.04453	0.14121	0.32427
C	3.37333	-0.31200	-0.07704
C	3.26085	-1.69438	-0.71343
O	2.66126	-2.63339	0.17470
H	0.69480	-2.12458	-0.27422
H	0.99284	-2.93495	1.28963
H	2.05538	-0.89514	2.18467
H	0.45162	-0.49347	1.53428
H	3.78759	0.38934	-0.80967
H	4.07321	-0.36198	0.77970
H	2.66474	-1.62108	-1.63753
H	4.25019	-2.08897	-0.96206
C	-3.16735	-0.19519	1.31673
H	-3.39903	0.79763	1.71784
H	-4.12526	-0.67465	1.08234
C	-2.44586	-1.00789	2.40402
H	-1.53169	-0.49352	2.71365
H	-3.08251	-1.13251	3.28803
H	-2.17587	-2.00333	2.03316
C	-2.53281	-0.74088	-1.11861
C	-3.53125	-1.77245	-1.52907
H	-4.07328	-1.47579	-2.43608
H	-3.05635	-2.74063	-1.73430
H	-4.26671	-1.92610	-0.73572
H	0.83938	3.30762	0.75708

4s_Renantiomer_conformer-028

C	2.62453	0.69065	-0.18235
N	3.22914	-1.43564	-0.55263
H	3.78359	-2.23129	-0.83939
C	1.97538	-1.50741	-0.04124
C	1.55523	-0.20120	0.21142
C	-0.30523	-2.37610	0.19919
C	1.18650	-2.74257	0.23090
H	-0.60066	-2.16743	-0.83344

H	-0.90846	-3.22743	0.53323
H	1.41001	-3.52590	-0.50286
H	1.45219	-3.15112	1.21768
C	0.26193	0.07530	0.79654
O	-0.11369	1.20900	1.11934
C	-0.62999	-1.15467	1.07769
C	-2.12685	-0.80553	1.08013
H	-2.65205	-1.56644	1.66836
H	-2.24496	0.15071	1.60940
C	-4.68941	0.70705	0.07657
C	-4.20588	-0.71993	-0.19757
N	-2.74252	-0.82678	-0.25354
C	-2.23517	0.17061	-1.20669
C	-2.73819	1.58406	-0.91533
O	-4.16946	1.61349	-0.89650
H	-4.38980	1.03067	1.08625
H	-5.78001	0.76980	0.00732
H	-4.60567	-1.03915	-1.16961
H	-4.59009	-1.40537	0.56638
H	-1.14278	0.14886	-1.22820
H	-2.58663	-0.12412	-2.20469
H	-2.33726	1.94119	0.04365
H	-2.42720	2.27896	-1.70183
C	2.62394	2.18783	-0.06807
H	3.38239	2.60947	-0.73857
H	1.65433	2.57422	-0.40075
C	2.88264	2.67632	1.36620
H	3.85281	2.31979	1.73069
H	2.88037	3.77175	1.41402
H	2.10266	2.30200	2.03562
C	3.64634	-0.10548	-0.65175
C	4.99550	0.22933	-1.19703
H	5.13859	-0.17539	-2.20705
H	5.12400	1.31313	-1.24997
H	5.80075	-0.17066	-0.56743
H	-0.38483	-1.41886	2.11992

4s_Renantiomer_conformer-029

C	-2.15656	0.42250	-0.17918
N	-1.25311	1.11143	1.75171
H	-1.07770	1.67576	2.57212
C	-0.62929	-0.05934	1.46775
C	-1.16356	-0.53163	0.26827
C	0.62647	-2.15233	1.80711
C	0.45073	-0.69554	2.27293

H	1.56105	-2.56001	2.20966
H	-0.18914	-2.75137	2.23077
H	1.38298	-0.13791	2.13433
H	0.21056	-0.66777	3.34345
C	-0.63117	-1.70779	-0.38148
O	-1.06092	-2.17008	-1.44691
C	0.60433	-2.33371	0.27638
C	1.89093	-1.82766	-0.42479
H	2.74367	-2.31355	0.06256
H	1.88117	-2.17511	-1.47083
C	1.61291	1.83557	-1.24701
C	1.58042	0.33884	-1.52489
N	2.07418	-0.37944	-0.35212
C	3.43479	0.04941	-0.04428
C	3.43961	1.55567	0.20068
O	2.93226	2.27076	-0.92228
H	0.92362	2.06434	-0.41899
H	1.30822	2.40366	-2.13052
H	2.19549	0.11753	-2.41919
H	0.55526	0.03388	-1.74216
H	3.77924	-0.46408	0.86013
H	4.14338	-0.18874	-0.86136
H	2.83138	1.77966	1.09204
H	4.45766	1.91930	0.36716
C	-2.99491	0.31599	-1.41966
H	-3.44407	1.29037	-1.64594
H	-2.35252	0.05705	-2.26911
C	-4.10189	-0.74356	-1.30260
H	-4.77345	-0.51614	-0.46680
H	-3.65869	-1.72910	-1.13247
H	-4.70037	-0.78963	-2.22020
C	-2.18782	1.42806	0.76155
C	-3.00421	2.67425	0.86104
H	-3.64175	2.78170	-0.01982
H	-3.65607	2.66615	1.74431
H	-2.37313	3.56960	0.92914
H	0.55026	-3.40728	0.05707

4s_Renantiomer_conformer-030

C	-3.10921	0.44983	-0.06506
N	-2.92205	-1.70566	0.51788
H	-3.16656	-2.61962	0.87472
C	-1.73720	-1.39185	-0.06308
C	-1.80383	-0.04922	-0.43984
C	0.30622	-1.74083	-1.39001

C	-0.57519	-2.30549	-0.26247
H	1.25636	-2.28016	-1.42345
H	-0.20348	-1.89773	-2.34824
H	0.00764	-2.38763	0.66637
H	-0.90955	-3.32063	-0.50668
C	-0.68268	0.60755	-1.07771
O	-0.68077	1.78964	-1.44087
C	0.59019	-0.23897	-1.22179
C	1.48198	0.06676	0.00615
H	1.62433	1.15807	0.05491
H	0.94081	-0.21413	0.91614
C	4.45444	1.06839	-0.43155
C	3.73515	-0.17688	-0.95480
N	2.73294	-0.69218	-0.01106
C	3.35021	-0.85840	1.31036
C	4.07238	0.40052	1.79365
O	5.05301	0.81281	0.84025
H	3.75598	1.91646	-0.34908
H	5.26666	1.36042	-1.10411
H	4.47944	-0.96656	-1.11999
H	3.26025	0.03518	-1.91764
H	2.58318	-1.16177	2.03124
H	4.08483	-1.67168	1.23820
H	3.35297	1.21742	1.96695
H	4.60904	0.21137	2.72848
C	-3.60847	1.85438	-0.24439
H	-4.70045	1.87363	-0.14651
H	-3.37380	2.19582	-1.25862
C	-2.98800	2.83701	0.76187
H	-3.37489	3.85158	0.60977
H	-3.21328	2.53509	1.79106
H	-1.90122	2.86280	0.63927
C	-3.77611	-0.59995	0.52794
C	-5.14369	-0.70341	1.11864
H	-5.68647	0.23550	0.98543
H	-5.73169	-1.50045	0.64590
H	-5.10839	-0.91758	2.19464
H	1.09706	0.13336	-2.11886

4s_Renantiomer_conformer-031

C	-3.02541	0.32021	0.46314
N	-2.79235	-1.87118	0.05754
H	-3.00361	-2.85914	0.01671
C	-1.65900	-1.30012	-0.42143
C	-1.75806	0.07301	-0.18996

C	0.29891	-0.98335	-1.87979
C	-0.51054	-2.00452	-1.06184
H	1.24650	-1.42790	-2.19466
H	-0.26746	-0.72584	-2.78287
H	0.12860	-2.46465	-0.29442
H	-0.85824	-2.82297	-1.70337
C	-0.68027	0.97313	-0.54134
O	-0.70748	2.19666	-0.36482
C	0.58772	0.30674	-1.09411
C	1.54353	0.07876	0.10190
H	1.68389	1.04665	0.60900
H	1.05374	-0.58035	0.82678
C	4.22513	-0.31203	1.73576
C	3.48380	-1.26312	0.79457
N	2.79531	-0.56529	-0.29797
C	3.74237	0.33126	-0.97609
C	4.48332	1.25273	-0.00458
O	5.15089	0.49463	1.00574
H	4.81245	-0.86756	2.47340
H	3.51242	0.33253	2.27565
H	2.75811	-1.86601	1.35128
H	4.21736	-1.94722	0.34714
H	4.48023	-0.29128	-1.49844
H	3.21476	0.92133	-1.73173
H	5.25671	1.82607	-0.52467
H	3.78661	1.96492	0.46580
C	-3.56647	1.65893	0.87416
H	-4.38879	1.52172	1.58640
H	-2.78358	2.22019	1.39642
C	-4.05698	2.49484	-0.31876
H	-3.23003	2.68178	-1.01008
H	-4.85143	1.97076	-0.86226
H	-4.45084	3.46205	0.01523
C	-3.64256	-0.90406	0.59961
C	-4.96255	-1.29395	1.17877
H	-4.85223	-2.03408	1.98155
H	-5.46620	-0.41945	1.59766
H	-5.62732	-1.72960	0.42164
H	1.04282	1.04239	-1.76639

4s_Renantiomer_conformer-032

C	-3.24860	0.20079	-0.05768
N	-2.64835	-1.95744	-0.00374
H	-2.71684	-2.96202	0.08901
C	-1.52627	-1.28618	-0.36558

C	-1.85090	0.07108	-0.40736
C	0.60120	-0.87248	-1.51637
C	-0.19259	-1.88143	-0.66983
H	1.63315	-1.20984	-1.63956
H	0.15910	-0.84049	-2.52003
H	0.34584	-2.10908	0.26113
H	-0.29744	-2.83255	-1.20483
C	-0.86045	1.07283	-0.74401
O	-1.09092	2.28657	-0.80594
C	0.56988	0.55182	-0.93393
C	1.27867	0.72734	0.44495
H	1.14761	1.78242	0.71896
H	0.75508	0.13620	1.20494
C	5.00702	0.89731	-0.04510
C	3.54650	1.02282	-0.47008
N	2.69617	0.41299	0.54379
C	3.06347	-0.97636	0.79497
C	4.53384	-1.03239	1.19940
O	5.36205	-0.46376	0.18736
H	5.17390	1.48781	0.86966
H	5.67362	1.26323	-0.83111
H	3.42286	0.55372	-1.46375
H	3.28422	2.08280	-0.56854
H	2.44343	-1.36869	1.60877
H	2.92403	-1.63201	-0.08216
H	4.67669	-0.49085	2.14787
H	4.86292	-2.06763	1.32637
C	-4.01719	1.48464	0.06479
H	-3.80439	2.11808	-0.80326
H	-5.09326	1.27426	0.05050
C	-3.66561	2.26582	1.34121
H	-3.87658	1.66778	2.23508
H	-2.60269	2.52456	1.34116
H	-4.24504	3.19444	1.40547
C	-3.71225	-1.07296	0.18963
C	-5.05603	-1.57989	0.59855
H	-5.77508	-0.75816	0.63884
H	-5.44049	-2.32762	-0.10676
H	-5.03082	-2.04888	1.59065
H	1.05228	1.23479	-1.64151

4s_Renantiomer_conformer-033

C	2.79725	0.53737	0.15086
N	3.03622	-1.41327	-0.92664
H	3.43875	-2.13682	-1.50694

C	1.83410	-1.49541	-0.30460
C	1.63873	-0.29815	0.38310
C	-0.51663	-2.11020	-0.02795
C	0.89204	-2.65063	-0.31783
H	-0.83745	-1.51579	-0.88925
H	-1.22462	-2.93957	0.07497
H	0.91754	-3.17336	-1.28104
H	1.17772	-3.38657	0.44894
C	0.47563	-0.08991	1.21998
O	0.32291	0.90579	1.93779
C	-0.55787	-1.23705	1.23875
C	-1.97362	-0.74326	1.62818
H	-2.55032	-1.60348	1.98555
H	-1.84262	-0.06032	2.47429
C	-4.61071	-0.04179	-0.98420
C	-3.55391	-0.89998	-0.29587
N	-2.79786	-0.06612	0.63252
C	-2.19286	1.07702	-0.03861
C	-3.27494	1.87055	-0.76454
O	-4.00664	1.05094	-1.67357
H	-5.32508	0.33717	-0.23591
H	-5.15477	-0.62159	-1.73554
H	-2.92875	-1.36268	-1.07858
H	-4.04021	-1.70796	0.26265
H	-1.70522	1.71178	0.70618
H	-1.41797	0.77599	-0.77005
H	-3.96319	2.30945	-0.02439
H	-2.83283	2.67581	-1.35845
C	3.00009	1.93422	0.66192
H	2.79427	1.96022	1.73763
H	4.04840	2.22715	0.53002
C	2.09104	2.95826	-0.03668
H	1.03998	2.70458	0.13068
H	2.26571	3.96821	0.35265
H	2.27551	2.97094	-1.11693
C	3.64100	-0.18007	-0.66911
C	4.96702	0.15294	-1.26990
H	5.73248	-0.58624	-1.00133
H	4.92013	0.19157	-2.36594
H	5.30844	1.12952	-0.91839
H	-0.23290	-1.86952	2.08317

4s_Renantiomer_conformer-034

C	3.19209	0.10633	-0.35742
N	2.57468	-1.97763	0.18754

H	2.62512	-2.97562	0.34216
C	1.48709	-1.20772	0.44166
C	1.82452	0.10707	0.11518
C	-0.56278	-0.46059	1.56351
C	0.17121	-1.67343	0.96799
H	-1.58660	-0.73137	1.83253
H	-0.05827	-0.17497	2.49500
H	-0.42454	-2.12593	0.16260
H	0.30367	-2.45310	1.72736
C	0.85882	1.18125	0.22268
O	1.09673	2.36224	-0.05785
C	-0.56045	0.75887	0.62438
C	-1.35442	0.58062	-0.70639
H	-1.23241	1.52154	-1.25881
H	-0.88637	-0.20407	-1.31154
C	-5.04151	0.96900	-0.05335
C	-3.55523	1.16449	0.23343
N	-2.77826	0.28763	-0.63279
C	-3.17332	-1.10892	-0.48494
C	-4.66738	-1.23157	-0.77028
O	-5.42269	-0.39573	0.10399
H	-5.26314	1.30225	-1.07959
H	-5.65188	1.54567	0.64734
H	-3.37081	0.97071	1.30638
H	-3.27788	2.20592	0.03190
H	-2.61125	-1.71761	-1.20208
H	-2.98296	-1.51381	0.52424
H	-4.86695	-0.95495	-1.81763
H	-5.01327	-2.25492	-0.59921
C	3.98241	1.30682	-0.79107
H	4.86254	0.98368	-1.35965
H	3.37226	1.91553	-1.46753
C	4.42985	2.18348	0.38956
H	3.55614	2.55308	0.93431
H	5.05537	1.61228	1.08500
H	5.00729	3.04751	0.04001
C	3.62819	-1.19942	-0.29818
C	4.94023	-1.82612	-0.63842
H	5.62264	-1.07797	-1.04883
H	5.42211	-2.26755	0.24355
H	4.82935	-2.62382	-1.38388
H	-0.99336	1.61452	1.15389

4s_Renantiomer_conformer-035

C	2.87547	0.73248	-0.28418

N	3.64479	-1.32228	0.17347
H	4.29239	-2.08257	0.33215
C	2.29439	-1.44520	0.15018
C	1.76693	-0.18446	-0.12658
C	0.12064	-2.51406	-0.26297
C	1.50627	-2.68968	0.37718
H	0.21831	-2.55727	-1.35573
H	-0.53149	-3.34148	0.03611
H	2.01893	-3.56580	-0.03696
H	1.39166	-2.87339	1.45624
C	0.33890	0.03317	-0.22007
O	-0.16410	1.12260	-0.52019
C	-0.54640	-1.17952	0.13045
C	-1.97099	-1.06320	-0.44649
H	-1.95967	-0.35731	-1.28927
H	-2.26526	-2.03677	-0.85367
C	-3.39572	1.69877	0.18393
C	-2.85738	0.62694	1.13154
N	-2.96831	-0.73406	0.58310
C	-4.34913	-0.95350	0.13380
C	-4.86015	0.14298	-0.80393
O	-4.75113	1.42462	-0.18508
H	-2.76364	1.76053	-0.71306
H	-3.40194	2.67963	0.67008
H	-3.43882	0.65645	2.06292
H	-1.81456	0.84183	1.37434
H	-4.42005	-1.93368	-0.35160
H	-4.99292	-0.97071	1.02374
H	-4.29781	0.13603	-1.75176
H	-5.92037	-0.00226	-1.03457
C	2.78064	2.20578	-0.55745
H	3.75339	2.58093	-0.89699
H	2.07039	2.37773	-1.37355
C	2.32373	3.01049	0.67002
H	1.32969	2.68147	0.98706
H	3.01739	2.87237	1.50720
H	2.27297	4.08135	0.44018
C	4.02405	-0.00318	-0.08808
C	5.46715	0.37999	-0.11300
H	5.57600	1.43098	-0.39119
H	5.94236	0.24421	0.86708
H	6.03428	-0.21887	-0.83699
H	-0.63502	-1.17381	1.22830

4s_Renantiomer_conformer-036

C	2.72061	-0.48969	0.35012
N	2.86577	1.72079	0.69109
H	3.21447	2.61296	1.01496
C	1.71073	1.54189	0.00349
C	1.57753	0.17474	-0.23773
C	-0.62803	1.94370	-0.59080
C	0.75370	2.59732	-0.43480
H	-0.99451	1.67763	0.40575
H	-1.33794	2.66133	-1.01586
H	0.71471	3.42254	0.28570
H	1.07453	3.03019	-1.39443
C	0.46193	-0.35290	-0.99493
O	0.35681	-1.54374	-1.31296
C	-0.58055	0.68235	-1.47132
C	-1.96373	0.04155	-1.74573
H	-2.51916	0.70233	-2.42035
H	-1.77241	-0.89263	-2.28442
C	-4.77112	0.23824	0.77386
C	-3.67233	0.82581	-0.10625
N	-2.85108	-0.26180	-0.62764
C	-2.28855	-1.07277	0.44435
C	-3.41539	-1.58900	1.33400
O	-4.21245	-0.52189	1.84362
H	-5.43058	-0.40121	0.16551
H	-5.36807	1.03121	1.23378
H	-3.10390	1.55282	0.49865
H	-4.12178	1.36956	-0.94503
H	-1.74496	-1.91515	0.00837
H	-1.57029	-0.50740	1.06911
H	-4.04821	-2.28117	0.75557
H	-3.01290	-2.11858	2.20242
C	3.01237	-1.96221	0.31502
H	3.72632	-2.21578	1.10788
H	2.09189	-2.51735	0.52607
C	3.56833	-2.42777	-1.04026
H	4.49874	-1.90124	-1.28187
H	3.77663	-3.50425	-1.03029
H	2.84071	-2.22611	-1.83174
C	3.49843	0.49598	0.91816
C	4.78885	0.43533	1.66711
H	5.59371	0.96057	1.13682
H	4.70310	0.88904	2.66273
H	5.10202	-0.60319	1.79897
H	-0.20643	0.99048	-2.46328

4s_Renantiomer_conformer-037

C	-2.86582	0.75713	-0.19049
N	-3.62391	-1.35195	-0.19226
H	-4.26650	-2.13246	-0.21347
C	-2.27779	-1.45801	-0.06608
C	-1.75753	-0.16437	-0.06018
C	-0.12521	-2.38881	0.66707
C	-1.48592	-2.71663	0.03374
H	-0.26445	-2.17721	1.73541
H	0.53532	-3.25923	0.59363
H	-2.01686	-3.47470	0.62156
H	-1.33146	-3.14683	-0.96742
C	-0.33290	0.07741	0.02693
O	0.16222	1.20976	0.07860
C	0.56116	-1.17843	0.00005
C	1.96230	-0.92388	0.59043
H	1.92020	-0.04121	1.24435
H	2.23532	-1.77427	1.22489
C	3.42653	1.62200	-0.60075
C	2.92179	0.35863	-1.29773
N	3.00214	-0.83879	-0.44617
C	4.36245	-0.94355	0.09726
C	4.84130	0.34126	0.77761
O	4.76419	1.44583	-0.12313
H	2.75850	1.88562	0.23128
H	3.45794	2.46486	-1.29863
H	3.54147	0.17503	-2.18581
H	1.89111	0.50764	-1.62645
H	4.40836	-1.78551	0.79751
H	5.04147	-1.16248	-0.73807
H	4.24131	0.55018	1.67818
H	5.89058	0.25715	1.07807
C	-2.78174	2.25607	-0.19967
H	-1.96768	2.56819	-0.86305
H	-3.70747	2.67472	-0.61217
C	-2.53135	2.84680	1.19705
H	-3.33326	2.56585	1.88928
H	-1.58335	2.47459	1.59630
H	-2.48121	3.94137	1.15702
C	-4.00741	-0.01059	-0.26871
C	-5.44789	0.36092	-0.39873
H	-5.55221	1.44363	-0.50247
H	-5.91125	-0.10659	-1.27683
H	-6.02930	0.05362	0.48020
H	0.69266	-1.42710	-1.06478

4s_Renantiomer_conformer-038

C	-3.03672	0.60062	0.14859
N	-3.40949	-1.47830	-0.60031
H	-3.90003	-2.28886	-0.95335
C	-2.09422	-1.44971	-0.26967
C	-1.81131	-0.16727	0.19887
C	0.04852	-2.30191	0.57604
C	-1.12029	-2.57269	-0.38342
H	-0.28959	-2.45372	1.60969
H	0.85316	-3.02229	0.39267
H	-1.59548	-3.53494	-0.15909
H	-0.74131	-2.64226	-1.41445
C	-0.47884	0.20303	0.63019
O	-0.19473	1.31565	1.08789
C	0.60751	-0.87248	0.43613
C	1.83570	-0.63362	1.35506
H	1.55610	0.07415	2.14048
H	2.10439	-1.57975	1.84034
C	4.25428	1.69452	-0.36652
C	2.89683	1.20057	0.12003
N	3.04902	-0.12461	0.71809
C	3.64093	-1.05330	-0.24076
C	4.98098	-0.50385	-0.72006
O	4.82912	0.78674	-1.30499
H	4.93161	1.81943	0.49342
H	4.15655	2.65412	-0.88218
H	2.19401	1.19594	-0.73403
H	2.49044	1.88552	0.86963
H	3.79309	-2.02323	0.24776
H	2.99448	-1.22101	-1.12353
H	5.67979	-0.44915	0.12995
H	5.41165	-1.14824	-1.49172
C	-3.19832	2.04501	0.52521
H	-4.26152	2.27270	0.66724
H	-2.70218	2.22620	1.48484
C	-2.60694	3.00204	-0.52234
H	-3.08806	2.85936	-1.49666
H	-2.74530	4.04732	-0.22158
H	-1.53456	2.81800	-0.63652
C	-4.00639	-0.23952	-0.35458
C	-5.45541	-0.02507	-0.64519
H	-5.67538	-0.11995	-1.71646
H	-6.08798	-0.74938	-0.11633
H	-5.76099	0.97617	-0.33199
H	0.92735	-0.75316	-0.61136

4s_Renantiomer_conformer-039

C	-3.32517	0.48985	0.09823
N	-3.39148	-1.72996	-0.20231
H	-3.76125	-2.66098	-0.34018
C	-2.08041	-1.43500	-0.01814
C	-1.98689	-0.05625	0.16834
C	0.22307	-1.75378	0.76251
C	-0.94116	-2.39632	-0.00586
H	-0.02857	-1.71274	1.83015
H	1.11700	-2.37835	0.67199
H	-1.23110	-3.35023	0.45010
H	-0.62322	-2.62337	-1.03483
C	-0.71191	0.59473	0.38717
O	-0.59311	1.80509	0.60150
C	0.52948	-0.32433	0.27189
C	1.76211	0.29988	0.94578
H	1.52327	1.33592	1.19923
H	1.97552	-0.23107	1.89437
C	4.68008	-0.89258	-1.13735
C	3.46245	-1.00281	-0.22619
N	2.94350	0.33038	0.08130
C	4.00508	1.13995	0.68153
C	5.20614	1.20651	-0.25401
O	5.70343	-0.09625	-0.54814
H	4.37571	-0.45677	-2.10243
H	5.11565	-1.87918	-1.31809
H	3.74327	-1.54957	0.69585
H	2.69223	-1.58745	-0.73870
H	3.62274	2.15134	0.86125
H	4.33068	0.72586	1.65607
H	4.91801	1.71640	-1.18718
H	6.02818	1.75828	0.21034
C	-3.70330	1.93889	0.20616
H	-4.77720	2.02560	0.41009
H	-3.17972	2.38766	1.05715
C	-3.35856	2.73840	-1.06071
H	-2.28133	2.70019	-1.24834
H	-3.87620	2.32675	-1.93462
H	-3.65067	3.78970	-0.95311
C	-4.17093	-0.57259	-0.13596
C	-5.65206	-0.64402	-0.31217
H	-5.92770	-0.98418	-1.31875
H	-6.11369	-1.33540	0.40430
H	-6.09995	0.34116	-0.16143

H	0.73356	-0.36646	-0.81023

4s_Renantiomer_conformer-040

C	2.77522	0.77969	0.12276
N	3.30606	-1.21113	-0.75836
H	3.83567	-1.93179	-1.23010
C	2.10924	-1.40080	-0.14869
C	1.73340	-0.18059	0.41384
C	0.32785	-2.56500	1.08597
C	1.34174	-2.67769	-0.06810
H	-0.37157	-3.40748	1.05339
H	0.86885	-2.62910	2.03752
H	0.81923	-2.86692	-1.01649
H	2.01045	-3.53182	0.09113
C	0.49521	-0.04099	1.15155
O	0.19659	0.96584	1.80459
C	-0.46418	-1.23903	1.04740
C	-1.34706	-1.16639	-0.22816
H	-0.78719	-0.67890	-1.04926
H	-1.55088	-2.19136	-0.55819
C	-4.90938	-0.17998	-0.88743
C	-3.53975	-0.79517	-1.15022
N	-2.64782	-0.53347	-0.01842
C	-2.55471	0.91216	0.20336
C	-3.94456	1.49276	0.42913
O	-4.81355	1.22399	-0.66870
H	-5.36662	-0.66564	-0.01037
H	-5.56684	-0.31945	-1.75020
H	-3.13332	-0.38010	-2.09334
H	-3.64564	-1.87844	-1.28098
H	-1.92909	1.10629	1.07691
H	-2.08479	1.41822	-0.66420
H	-4.37195	1.07224	1.35373
H	-3.89464	2.58072	0.52832
C	2.76387	2.23492	0.49114
H	2.50853	2.33837	1.55129
H	3.76713	2.65748	0.36075
C	1.75563	3.04631	-0.33908
H	0.74218	2.66414	-0.18295
H	1.77034	4.10388	-0.05049
H	1.98778	2.97936	-1.40803
C	3.73138	0.11165	-0.61067
C	5.02005	0.56523	-1.21344
H	5.86635	-0.04118	-0.86655
H	5.00176	0.50458	-2.30927

H	5.22081	1.60441	-0.94210
H	-1.13209	-1.19647	1.91358

4s_Renantiomer_conformer-041

C	-2.40358	-0.85176	0.38948
N	-3.29013	0.84220	-0.77885
H	-3.95463	1.38003	-1.31883
C	-2.10102	1.31006	-0.32228
C	-1.50399	0.28086	0.40760
C	-0.46313	2.94254	0.52973
C	-1.54916	2.68063	-0.53175
H	0.09522	3.85014	0.27341
H	-0.95257	3.12831	1.49309
H	-1.12897	2.77265	-1.54288
H	-2.33979	3.43686	-0.45987
C	-0.21860	0.46485	1.04931
O	0.29231	-0.35637	1.82103
C	0.51653	1.76050	0.69162
C	1.38660	1.61890	-0.59391
H	0.73542	1.43527	-1.45532
H	1.85520	2.60112	-0.75065
C	3.27008	-1.58039	-1.25654
C	2.02615	-0.74703	-0.96800
N	2.43885	0.61540	-0.63330
C	3.34840	0.62299	0.50935
C	4.55351	-0.25801	0.19443
O	4.15185	-1.58612	-0.13518
H	3.79141	-1.17601	-2.13906
H	3.00329	-2.62398	-1.44745
H	1.46213	-1.22928	-0.15656
H	1.38633	-0.71845	-1.85773
H	3.68574	1.65083	0.69190
H	2.85956	0.25667	1.42736
H	5.11951	0.17852	-0.64418
H	5.21338	-0.33985	1.06302
C	-2.16128	-2.19362	1.01883
H	-1.71443	-2.05044	2.00760
H	-3.11770	-2.70893	1.16899
C	-1.23248	-3.08687	0.18062
H	-0.25598	-2.61001	0.05114
H	-1.65677	-3.26579	-0.81390
H	-1.07604	-4.05714	0.66646
C	-3.49659	-0.47386	-0.35978
C	-4.73681	-1.20985	-0.74657
H	-5.64206	-0.66195	-0.45555

H	-4.79095	-1.37895	-1.82982
H	-4.76521	-2.18658	-0.25737
H	1.18573	1.97414	1.53160

4s_Renantiomer_conformer-042

C	2.27061	-0.98753	-0.37486
N	3.23466	0.57134	0.91315
H	3.90048	1.01751	1.52937
C	2.15720	1.18498	0.35751
C	1.52353	0.24614	-0.45536
C	0.70688	3.00241	-0.49930
C	1.73495	2.59997	0.57823
H	0.19540	3.92249	-0.19496
H	1.24024	3.22655	-1.43042
H	1.30103	2.70929	1.58198
H	2.59614	3.27820	0.54664
C	0.33969	0.59002	-1.21630
O	-0.12714	-0.10030	-2.12747
C	-0.33368	1.89967	-0.79098
C	-1.22601	1.64325	0.44342
H	-0.59048	1.54301	1.32899
H	-1.86375	2.53459	0.60542
C	-3.75851	-0.88019	-0.75609
C	-3.01547	0.44955	-0.70899
N	-2.01256	0.41811	0.35630
C	-2.64756	0.09907	1.63390
C	-3.38848	-1.22822	1.52642
O	-4.36920	-1.18936	0.49410
H	-3.05385	-1.67981	-1.03508
H	-4.56287	-0.84565	-1.49617
H	-3.74104	1.27188	-0.55388
H	-2.51400	0.60445	-1.66689
H	-1.87481	0.02559	2.40797
H	-3.36116	0.88750	1.94378
H	-2.66571	-2.03561	1.32647
H	-3.91971	-1.45154	2.45590
C	1.88673	-2.28320	-1.02826
H	1.67445	-2.10585	-2.08803
H	2.72463	-2.98889	-0.98185
C	0.64512	-2.91525	-0.37544
H	0.83808	-3.14994	0.67775
H	-0.20151	-2.22297	-0.41966
H	0.35984	-3.84252	-0.88622
C	3.32080	-0.75610	0.48696
C	4.41407	-1.64427	0.98282

H	5.40611	-1.22671	0.76839
H	4.35313	-1.80118	2.06753
H	4.35363	-2.62360	0.50186
H	-0.95749	2.22243	-1.63030

4s_Renantiomer_conformer-043

C	-2.26383	-0.88092	-0.03848
N	-3.08638	0.99495	-0.94726
H	-3.70745	1.61736	-1.44648
C	-1.95516	1.37923	-0.30348
C	-1.40431	0.23778	0.28059
C	-0.36503	2.83172	0.90553
C	-1.40710	2.76555	-0.22861
H	0.21082	3.76074	0.82488
H	-0.89217	2.86409	1.86634
H	-0.94970	3.04411	-1.18808
H	-2.20650	3.49470	-0.05035
C	-0.16819	0.30439	1.03163
O	0.28410	-0.62778	1.70812
C	0.59712	1.62423	0.91077
C	1.50662	1.66749	-0.35400
H	0.87884	1.68579	-1.25114
H	2.03636	2.63012	-0.31408
C	3.21473	-1.47884	-1.53959
C	2.01390	-0.62812	-1.13930
N	2.49770	0.62117	-0.55123
C	3.36813	0.36641	0.59404
C	4.52565	-0.52440	0.15405
O	4.05240	-1.74321	-0.41564
H	3.79256	-0.95956	-2.32100
H	2.89217	-2.45142	-1.92252
H	1.38509	-1.21087	-0.44960
H	1.41653	-0.39467	-2.02835
H	3.76283	1.32176	0.96178
H	2.82796	-0.12041	1.42239
H	5.14600	0.01338	-0.58096
H	5.14941	-0.80253	1.00853
C	-2.06946	-2.30449	0.39703
H	-2.65955	-2.96974	-0.24462
H	-1.01850	-2.58448	0.25984
C	-2.45380	-2.53765	1.86677
H	-1.83128	-1.91649	2.51702
H	-3.50472	-2.27948	2.04062
H	-2.30772	-3.58713	2.14873
C	-3.29515	-0.37837	-0.80215

C	-4.48316	-1.03554	-1.42401
H	-5.42435	-0.65751	-1.00423
H	-4.51907	-0.87013	-2.50833
H	-4.45341	-2.11400	-1.25104
H	1.23894	1.69046	1.79546

4s_Renantiomer_conformer-044

C	3.24009	0.52904	-0.25373
N	3.44202	-1.65204	0.22024
H	3.86679	-2.55476	0.38523
C	2.10640	-1.41694	0.19057
C	1.92933	-0.06357	-0.09668
C	-0.25970	-1.88735	-0.25435
C	1.01802	-2.41057	0.41797
H	-0.14222	-1.94907	-1.34482
H	-1.11947	-2.50648	0.01397
H	1.29296	-3.39622	0.02421
H	0.84261	-2.54128	1.49667
C	0.60763	0.52207	-0.19067
O	0.40268	1.70951	-0.46766
C	-0.56257	-0.42871	0.12759
C	-1.86248	0.07531	-0.51460
H	-1.96678	1.14708	-0.30114
H	-1.76671	-0.01465	-1.60311
C	-4.35332	0.90511	1.24762
C	-3.53220	-0.38650	1.23269
N	-3.02344	-0.72449	-0.10439
C	-4.13431	-0.69852	-1.06301
C	-4.93919	0.60135	-1.01210
O	-5.42866	0.83564	0.30960
H	-3.71673	1.77441	1.01778
H	-4.80801	1.06618	2.22987
H	-4.17370	-1.21156	1.56874
H	-2.69738	-0.31110	1.93689
H	-3.74386	-0.86815	-2.07229
H	-4.80901	-1.53035	-0.81902
H	-4.32039	1.45331	-1.33791
H	-5.81768	0.54611	-1.66251
C	3.53701	1.97318	-0.53843
H	4.56805	2.07367	-0.89810
H	2.88272	2.32504	-1.34341
C	3.33491	2.87647	0.68880
H	2.29519	2.82629	1.02552
H	3.98185	2.56188	1.51565
H	3.56778	3.92094	0.44992

C	4.15487	-0.48097	-0.04820
C	5.64798	-0.49001	-0.07042
H	6.06933	-0.74081	0.91166
H	6.03973	-1.22019	-0.79007
H	6.02912	0.49438	-0.35261
H	-0.67125	-0.38647	1.22423

4s_Renantiomer_conformer-045

C	2.40353	0.85177	0.38955
N	3.29018	-0.84216	-0.77874
H	3.95475	-1.37998	-1.31865
C	2.10103	-1.31004	-0.32230
C	1.50394	-0.28086	0.40756
C	0.46318	-2.94259	0.52964
C	1.54918	-2.68059	-0.53185
H	-0.09512	-3.85021	0.27330
H	0.95265	-3.12835	1.49298
H	1.12896	-2.77254	-1.54298
H	2.33981	-3.43683	-0.46006
C	0.21851	-0.46488	1.04918
O	-0.29252	0.35636	1.82081
C	-0.51653	-1.76060	0.69156
C	-1.38671	-1.61904	-0.59390
H	-0.73563	-1.43548	-1.45539
H	-1.85536	-2.60126	-0.75053
C	-3.27020	1.58021	-1.25674
C	-2.02623	0.74685	-0.96833
N	-2.43891	-0.61549	-0.63321
C	-3.34821	-0.62280	0.50963
C	-4.55335	0.25820	0.19482
O	-4.15170	1.58622	-0.13517
H	-3.79176	1.17568	-2.13905
H	-3.00340	2.62375	-1.44793
H	-1.46199	1.22926	-0.15714
H	-1.38666	0.71804	-1.85822
H	-3.68556	-1.65058	0.69247
H	-2.85914	-0.25631	1.42746
H	-5.11959	-0.17847	-0.64355
H	-5.21300	0.34026	1.06356
C	2.16112	2.19363	1.01885
H	1.71394	2.05047	2.00747
H	3.11752	2.70887	1.16932
C	1.23263	3.08696	0.18036
H	1.65720	3.26576	-0.81406
H	0.25611	2.61023	0.05067

H	1.07622	4.05728	0.66611
C	3.49661	0.47388	-0.35960
C	4.73687	1.20987	-0.74629
H	4.79101	1.37920	-1.82950
H	4.76533	2.18650	-0.25689
H	5.64208	0.66185	-0.45539
H	-1.18566	-1.97424	1.53160

4s_Renantiomer_conformer-046

C	2.55409	0.69800	0.20559
N	3.00450	-1.12028	-1.02464
H	3.49052	-1.74976	-1.64923
C	1.86337	-1.41903	-0.35503
C	1.53909	-0.30901	0.42583
C	0.18878	-2.78717	0.81866
C	1.10018	-2.69929	-0.41976
H	-0.51454	-3.61960	0.70747
H	0.80883	-3.00141	1.69729
H	0.49815	-2.73561	-1.33872
H	1.77612	-3.56149	-0.46009
C	0.36652	-0.30140	1.27481
O	0.11825	0.59508	2.09020
C	-0.59841	-1.48180	1.06690
C	-1.58516	-1.20178	-0.09954
H	-1.08951	-0.55743	-0.84494
H	-1.79156	-2.14925	-0.61038
C	-3.68305	0.45621	-1.73093
C	-3.88336	-0.68973	-0.73508
N	-2.88804	-0.69500	0.34664
C	-2.86071	0.63973	0.96736
C	-2.66852	1.76236	-0.05175
O	-3.68083	1.71742	-1.06118
H	-4.50309	0.49101	-2.45493
H	-2.74059	0.32799	-2.28729
H	-3.85949	-1.65372	-1.25626
H	-4.87469	-0.57887	-0.27596
H	-3.82799	0.78788	1.46557
H	-2.07556	0.67806	1.72463
H	-1.67321	1.69792	-0.52088
H	-2.75303	2.74280	0.42708
C	2.57917	2.07517	0.80265
H	2.41028	2.00525	1.88264
H	3.57090	2.52086	0.66184
C	1.51217	3.00205	0.19731
H	1.65210	3.10364	-0.88484

H	0.51219	2.59624	0.37808
H	1.56049	4.00172	0.64483
C	3.44350	0.16675	-0.70272
C	4.67818	0.72555	-1.32957
H	5.54946	0.08173	-1.15463
H	4.56865	0.83959	-2.41576
H	4.90238	1.71076	-0.91370
H	-1.18637	-1.59094	1.98356

4s_Renantiomer_conformer-047

C	2.49792	-0.65554	0.27174
N	2.82699	1.44397	0.98278
H	3.25192	2.23605	1.44599
C	1.72086	1.50422	0.20038
C	1.47468	0.21311	-0.26877
C	0.07141	2.43321	-1.36825
C	0.91449	2.71977	-0.11210
H	-0.66022	3.23438	-1.51951
H	0.73349	2.42915	-2.24231
H	0.26220	2.97146	0.73611
H	1.55948	3.59157	-0.27365
C	0.33604	-0.07819	-1.11363
O	0.13592	-1.18197	-1.63571
C	-0.66427	1.07816	-1.29023
C	-1.71832	1.08165	-0.14886
H	-1.27124	0.64189	0.75854
H	-1.95130	2.12208	0.10516
C	-3.90667	-0.17514	1.70594
C	-4.04930	0.70336	0.45975
N	-2.99397	0.46820	-0.53609
C	-2.93116	-0.97410	-0.82472
C	-2.79952	-1.82507	0.43774
O	-3.86714	-1.55772	1.35143
H	-4.76673	-0.04993	2.37114
H	-2.99754	0.09179	2.26850
H	-4.05434	1.76285	0.74084
H	-5.01298	0.47557	-0.01487
H	-3.86649	-1.24712	-1.33109
H	-2.09980	-1.17842	-1.50211
H	-2.85912	-2.89106	0.19733
H	-1.83142	-1.64095	0.93103
C	2.64361	-2.12692	0.01191
H	3.27646	-2.57761	0.78571
H	1.66078	-2.60554	0.08617
C	3.23534	-2.43019	-1.37383

H	3.33179	-3.51093	-1.53159
H	2.58451	-2.02583	-2.15458
H	4.22805	-1.97841	-1.48141
C	3.31919	0.13773	1.04272
C	4.53934	-0.17807	1.84342
H	5.42606	0.34101	1.45724
H	4.42255	0.11244	2.89522
H	4.74464	-1.25085	1.81369
H	-1.19388	0.91082	-2.23331

4s_Renantiomer_conformer-048

C	-3.25482	-0.40964	0.18165
N	-3.13935	1.66649	-0.65393
H	-3.42419	2.53658	-1.08320
C	-1.89908	1.41024	-0.16908
C	-1.91950	0.11902	0.35957
C	0.26817	1.88756	0.89542
C	-0.72869	2.33305	-0.18988
H	1.20083	2.44847	0.78282
H	-0.14685	2.14884	1.87633
H	-0.24986	2.31776	-1.17899
H	-1.04030	3.36973	-0.01603
C	-0.73882	-0.46913	0.95104
O	-0.70070	-1.59949	1.44964
C	0.54866	0.37053	0.87324
C	1.36627	-0.10250	-0.35987
H	0.96087	-1.05207	-0.72455
H	1.25589	0.62492	-1.18655
C	4.95589	0.52912	0.63482
C	3.50040	0.86591	0.32925
N	2.77628	-0.34646	-0.05542
C	3.44724	-0.97737	-1.19434
C	4.90059	-1.27581	-0.84728
O	5.59575	-0.08839	-0.47742
H	4.99965	-0.13870	1.50992
H	5.52099	1.43849	0.85691
H	3.46066	1.63172	-0.47045
H	3.04602	1.29533	1.22681
H	2.92919	-1.91173	-1.43943
H	3.41712	-0.33029	-2.09269
H	4.94027	-2.00529	-0.02276
H	5.42610	-1.69103	-1.71164
C	-3.72650	-1.78082	0.57052
H	-3.38855	-2.00420	1.58836
H	-4.82267	-1.80355	0.58594

C	-3.20549	-2.87766	-0.37198
H	-2.11190	-2.89911	-0.35342
H	-3.57331	-3.86448	-0.06696
H	-3.53126	-2.69400	-1.40217
C	-3.98448	0.57200	-0.45208
C	-5.40619	0.61767	-0.90642
H	-5.92775	-0.29635	-0.61246
H	-5.94580	1.46724	-0.46886
H	-5.48311	0.70847	-1.99759
H	1.12705	0.09309	1.76080

4s_Renantiomer_conformer-049

C	-3.36560	-0.27058	-0.22832
N	-3.11760	1.89603	0.28913
H	-3.34913	2.86104	0.48283
C	-1.85839	1.39480	0.24083
C	-1.96046	0.04119	-0.07410
C	0.55291	1.39388	-0.22639
C	-0.58947	2.13343	0.48829
H	0.45291	1.53819	-1.31019
H	1.50433	1.84068	0.07040
H	-0.65737	3.17020	0.13845
H	-0.38107	2.17961	1.56785
C	-0.78767	-0.80075	-0.18032
O	-0.84265	-2.01183	-0.42530
C	0.57386	-0.11507	0.07758
C	1.68072	-0.88819	-0.67810
H	1.51618	-1.95047	-0.47373
H	1.53838	-0.74904	-1.75703
C	4.95591	-0.65877	1.16494
C	3.43529	-0.62664	1.03244
N	3.07603	-0.58591	-0.37773
C	3.69100	0.55116	-1.05240
C	5.20458	0.46406	-0.87987
O	5.55718	0.45136	0.50253
H	5.33921	-1.59957	0.73891
H	5.25739	-0.59507	2.21444
H	3.04744	0.24474	1.59384
H	3.01033	-1.52967	1.48671
H	3.43492	0.51861	-2.11734
H	3.36087	1.52767	-0.65676
H	5.58111	-0.44745	-1.37058
H	5.69853	1.33599	-1.31828
C	-3.95286	-1.61720	-0.53872
H	-4.98082	-1.49640	-0.90105

H	-3.38209	-2.08306	-1.34938
C	-3.94701	-2.56278	0.67311
H	-4.52042	-2.13593	1.50392
H	-4.38771	-3.53328	0.41579
H	-2.92047	-2.73125	1.01139
C	-4.05490	0.89981	0.00444
C	-5.51504	1.21283	-0.00623
H	-5.87515	1.51566	0.98551
H	-5.75131	2.02758	-0.70251
H	-6.08885	0.33488	-0.31263
H	0.75043	-0.23786	1.15879

4s_Renantiomer_conformer-050

C	-2.41543	0.81806	-0.41212
N	-3.25984	-0.77851	0.91578
H	-3.88602	-1.25639	1.54970
C	-2.11476	-1.30270	0.41088
C	-1.55104	-0.34082	-0.42495
C	-0.04370	-2.60623	0.48540
C	-1.57269	-2.67376	0.63002
H	0.35920	-2.09409	1.36314
H	0.37565	-3.61827	0.49676
H	-1.84795	-3.05921	1.61832
H	-1.99387	-3.37148	-0.10893
C	-0.36173	-0.62850	-1.19914
O	0.01607	0.05426	-2.15494
C	0.42101	-1.89875	-0.80891
C	1.94522	-1.63254	-0.82777
H	2.44077	-2.47356	-0.30380
H	2.29110	-1.65933	-1.86527
C	4.25807	1.19855	0.01229
C	3.82512	-0.19206	-0.43453
N	2.37837	-0.34201	-0.29097
C	1.98000	-0.06881	1.08759
C	2.46628	1.31583	1.50340
O	3.87699	1.44666	1.36251
H	3.80513	1.95449	-0.64882
H	5.34633	1.29743	-0.03043
H	4.37456	-0.94932	0.15962
H	4.09592	-0.33747	-1.48683
H	0.88943	-0.07910	1.16177
H	2.37961	-0.82453	1.79275
H	1.95729	2.07805	0.89192
H	2.23730	1.49981	2.55678
C	-2.15935	2.11315	-1.12650

H	-1.97691	1.91199	-2.18804
H	-3.05002	2.74981	-1.06970
C	-0.94905	2.87312	-0.55811
H	-0.03997	2.27393	-0.66724
H	-0.79764	3.82143	-1.08695
H	-1.09088	3.09324	0.50613
C	-3.45917	0.51950	0.43717
C	-4.63860	1.31862	0.88460
H	-5.58380	0.80557	0.66579
H	-4.61447	1.51269	1.96475
H	-4.65803	2.28455	0.37427
H	0.23187	-2.59999	-1.63585

4s_Renantiomer_conformer-051

C	2.45649	-0.78270	0.11691
N	3.11256	1.20063	0.92917
H	3.66336	1.89501	1.41576
C	1.95787	1.45213	0.26298
C	1.51004	0.24208	-0.26496
C	-0.20570	2.53757	-0.10676
C	1.30244	2.77576	0.06545
H	-0.62121	2.25665	0.86452
H	-0.69817	3.47138	-0.39935
H	1.49257	3.44591	0.91153
H	1.71096	3.27172	-0.82777
C	0.32207	0.17608	-1.09171
O	0.00565	-0.81142	-1.76063
C	-0.53054	1.45992	-1.16602
C	-2.04009	1.14214	-1.25973
H	-2.58973	2.08826	-1.08520
H	-2.26761	0.83101	-2.28383
C	-4.47443	-1.30315	0.20890
C	-3.97276	-0.11449	-0.60134
N	-2.53840	0.07360	-0.39322
C	-2.25380	0.23046	1.02991
C	-2.80065	-0.96694	1.80053
O	-4.20008	-1.13213	1.59655
H	-3.99604	-2.22612	-0.15574
H	-5.55901	-1.40584	0.11341
H	-4.54778	0.78789	-0.31172
H	-4.15770	-0.29552	-1.66674
H	-1.17188	0.26669	1.18484
H	-2.69040	1.16165	1.44275
H	-2.26647	-1.87634	1.48180
H	-2.65390	-0.82950	2.87541

C	2.39073	-2.23754	-0.24900
H	2.98129	-2.82561	0.46396
H	1.35466	-2.58386	-0.16506
C	2.88870	-2.51459	-1.67652
H	3.93003	-2.19409	-1.79566
H	2.83039	-3.58387	-1.91251
H	2.27284	-1.96858	-2.39686
C	3.43391	-0.15704	0.86071
C	4.65596	-0.68470	1.53736
H	5.57356	-0.25264	1.11760
H	4.65362	-0.46517	2.61270
H	4.71610	-1.76920	1.41762
H	-0.26444	1.89895	-2.14071

4s_Renantiomer_conformer-052

C	-3.28714	0.41631	-0.22959
N	-3.27288	-1.80908	0.03358
H	-3.60798	-2.75652	0.14473
C	-1.96769	-1.44078	0.04987
C	-1.92434	-0.05646	-0.11153
C	0.40168	-1.49426	0.68206
C	-0.78837	-2.33931	0.20467
H	0.23505	-1.19232	1.72412
H	1.31317	-2.10039	0.67091
H	-0.99826	-3.15243	0.90964
H	-0.54190	-2.81336	-0.75756
C	-0.66740	0.66017	-0.17695
O	-0.58788	1.88802	-0.28123
C	0.60186	-0.22706	-0.17393
C	1.85533	0.57505	0.21536
H	1.58485	1.63353	0.24644
H	2.15734	0.29065	1.23793
C	4.94106	1.05032	0.60559
C	4.01736	1.42343	-0.55654
N	2.93782	0.45027	-0.77112
C	3.52704	-0.89222	-0.87720
C	4.45347	-1.23379	0.29140
O	5.48810	-0.25620	0.41662
H	4.39903	1.09230	1.56384
H	5.79194	1.73601	0.66464
H	4.61911	1.46639	-1.47442
H	3.58061	2.41488	-0.39249
H	2.73358	-1.63947	-0.97038
H	4.11599	-0.92464	-1.80329
H	3.88748	-1.29960	1.23452

H	4.95228	-2.19371	0.12640
C	-3.72564	1.84354	-0.38788
H	-3.10439	2.33080	-1.14731
H	-4.75854	1.87297	-0.75483
C	-3.62519	2.64646	0.91899
H	-2.58848	2.66668	1.26778
H	-3.95800	3.68059	0.77070
H	-4.24545	2.19609	1.70235
C	-4.09752	-0.69424	-0.13442
C	-5.58249	-0.84766	-0.17199
H	-5.97724	-1.22986	0.77830
H	-6.05859	0.11688	-0.36396
H	-5.90026	-1.54152	-0.96064
H	0.72014	-0.53382	-1.22538

4s_Renantiomer_conformer-053

C	3.28969	0.41605	-0.23477
N	3.27967	-1.80355	0.07383
H	3.61558	-2.75211	0.17235
C	1.97278	-1.44145	0.05334
C	1.92702	-0.06004	-0.13089
C	-0.42361	-1.64138	-0.42871
C	0.79415	-2.34379	0.18963
H	-0.30825	-1.61222	-1.51989
H	-1.32893	-2.22109	-0.22215
H	0.97604	-3.31046	-0.29452
H	0.59710	-2.55572	1.25151
C	0.66887	0.65498	-0.19166
O	0.58482	1.86890	-0.40168
C	-0.59200	-0.19906	0.09059
C	-1.87022	0.48634	-0.42127
H	-1.61029	1.50218	-0.72926
H	-2.22226	-0.04248	-1.32355
C	-4.46415	-1.25908	0.07957
C	-3.47777	-0.65404	1.08032
N	-2.89880	0.61641	0.62056
C	-3.99067	1.51983	0.23371
C	-4.97406	0.88597	-0.75335
O	-5.50653	-0.33033	-0.22506
H	-4.95111	-2.14508	0.49834
H	-3.94887	-1.55755	-0.84752
H	-2.67839	-1.36449	1.31032
H	-4.01644	-0.45488	2.01613
H	-4.54265	1.78983	1.14415
H	-3.56603	2.43756	-0.18830

H	-5.82887	1.54549	-0.93219
H	-4.48375	0.68922	-1.72018
C	3.72517	1.84274	-0.40609
H	4.76775	1.87151	-0.74460
H	3.12227	2.31311	-1.19030
C	3.58524	2.66750	0.88355
H	4.18545	2.23331	1.69125
H	3.91778	3.70043	0.72671
H	2.53957	2.68997	1.20473
C	4.10276	-0.68842	-0.09978
C	5.58866	-0.83680	-0.11053
H	5.97130	-1.18562	0.85736
H	5.91991	-1.55531	-0.87108
H	6.06421	0.12258	-0.32807
H	-0.65458	-0.23283	1.18999

4s_Renantiomer_conformer-054

C	-3.10918	0.44990	-0.06501
N	-2.92212	-1.70561	0.51791
H	-3.16667	-2.61955	0.87476
C	-1.73728	-1.39186	-0.06309
C	-1.80385	-0.04924	-0.43984
C	0.30610	-1.74094	-1.39007
C	-0.57533	-2.30557	-0.26253
H	1.25623	-2.28030	-1.42351
H	-0.20360	-1.89783	-2.34830
H	0.00751	-2.38777	0.66630
H	-0.90975	-3.32068	-0.50676
C	-0.68270	0.60747	-1.07777
O	-0.68076	1.78955	-1.44095
C	0.59013	-0.23908	-1.22186
C	1.48196	0.06663	0.00605
H	1.62422	1.15795	0.05487
H	0.94086	-0.21435	0.91605
C	4.07226	0.40021	1.79374
C	3.35024	-0.85866	1.31013
N	2.73299	-0.69220	-0.01128
C	3.73518	-0.17658	-0.95486
C	4.45433	1.06864	-0.43129
O	5.05288	0.81282	0.84047
H	4.60889	0.21090	2.72855
H	3.35275	1.21700	1.96720
H	2.58321	-1.16229	2.03091
H	4.08493	-1.67186	1.23781
H	4.47956	-0.96615	-1.12021

H	3.26029	0.03566	-1.91766
H	5.26655	1.36090	-1.10375
H	3.75578	1.91662	-0.34865
C	-3.60833	1.85449	-0.24427
H	-3.37387	2.19586	-1.25858
H	-4.70028	1.87386	-0.14612
C	-2.98748	2.83714	0.76175
H	-3.21236	2.53524	1.79103
H	-1.90075	2.86293	0.63872
H	-3.37446	3.85171	0.60979
C	-3.77612	-0.59985	0.52801
C	-5.14368	-0.70326	1.11879
H	-5.10833	-0.91760	2.19476
H	-5.68640	0.23572	0.98577
H	-5.73178	-1.50017	0.64596
H	1.09701	0.13321	-2.11895

4s_Renantiomer_conformer-055

C	3.24856	0.20086	0.05745
N	2.64844	-1.95743	0.00371
H	2.71702	-2.96203	-0.08887
C	1.52636	-1.28622	0.36564
C	1.85091	0.07106	0.40735
C	-0.60115	-0.87269	1.51650
C	0.19271	-1.88155	0.66991
H	-1.63311	-1.21009	1.63957
H	-0.15912	-0.84080	2.52019
H	-0.34569	-2.10921	-0.26106
H	0.29762	-2.83268	1.20487
C	0.86050	1.07272	0.74436
O	1.09102	2.28643	0.80667
C	-0.56983	0.55168	0.93422
C	-1.27860	0.72736	-0.44466
H	-1.14760	1.78248	-0.71853
H	-0.75497	0.13635	-1.20470
C	-5.00706	0.89736	0.04485
C	-3.54661	1.02275	0.47016
N	-2.69609	0.41297	-0.54359
C	-3.06337	-0.97638	-0.79488
C	-4.53367	-1.03239	-1.19964
O	-5.36212	-0.46368	-0.18783
H	-5.17371	1.48795	-0.86988
H	-5.67382	1.26322	0.83076
H	-3.42324	0.55350	1.46378
H	-3.28429	2.08270	0.56881

H	-2.44314	-1.36874	-1.60853
H	-2.92417	-1.63199	0.08230
H	-4.67628	-0.49091	-2.14817
H	-4.86275	-2.06763	-1.32662
C	4.01696	1.48481	-0.06523
H	3.80443	2.11821	0.80291
H	5.09306	1.27448	-0.05121
C	3.66515	2.26618	-1.34145
H	2.60243	2.52567	-1.34082
H	4.24518	3.19441	-1.40599
H	3.87521	1.66800	-2.23543
C	3.71228	-1.07290	-0.18982
C	5.05604	-1.57990	-0.59871
H	5.77510	-0.75817	-0.63889
H	5.44045	-2.32766	0.10662
H	5.03089	-2.04886	-1.59082
H	-1.05223	1.23456	1.64188

4s_Renantiomer_conformer-056

C	2.87556	0.73249	-0.28424
N	3.64481	-1.32224	0.17366
H	4.29239	-2.08253	0.33241
C	2.29440	-1.44514	0.15028
C	1.76700	-0.18442	-0.12662
C	0.12067	-2.51396	-0.26305
C	1.50623	-2.68958	0.37726
H	0.21846	-2.55722	-1.35580
H	-0.53151	-3.34135	0.03600
H	2.01890	-3.56574	-0.03677
H	1.39149	-2.87322	1.45632
C	0.33899	0.03325	-0.22028
O	-0.16394	1.12268	-0.52054
C	-0.54636	-1.17939	0.13026
C	-1.97094	-1.06305	-0.44670
H	-1.95965	-0.35692	-1.28927
H	-2.26512	-2.03653	-0.85416
C	-3.39576	1.69869	0.18440
C	-2.85744	0.62661	1.13174
N	-2.96829	-0.73425	0.58296
C	-4.34910	-0.95361	0.13356
C	-4.86010	0.14306	-0.80394
O	-4.75112	1.42458	-0.18483
H	-2.76360	1.76077	-0.71252
H	-3.40210	2.67939	0.67085
H	-3.43890	0.65587	2.06311

H	-1.81463	0.84147	1.37464
H	-4.41997	-1.93368	-0.35206
H	-4.99292	-0.97104	1.02348
H	-4.29774	0.13631	-1.75176
H	-5.92031	-0.00214	-1.03463
C	2.78061	2.20578	-0.55747
H	3.75343	2.58100	-0.89671
H	2.07058	2.37771	-1.37380
C	2.32323	3.01041	0.66987
H	1.32901	2.68151	0.98645
H	3.01654	2.87218	1.50732
H	2.27268	4.08130	0.44006
C	4.02410	-0.00316	-0.08800
C	5.46722	0.37997	-0.11278
H	5.57613	1.43093	-0.39103
H	5.94236	0.24423	0.86735
H	6.03440	-0.21895	-0.83669
H	-0.63499	-1.17362	1.22811

4s_Renantiomer_conformer-057

C	-2.67539	0.69078	-0.21801
N	-3.11199	-1.22156	0.86393
H	-3.60331	-1.91103	1.41661
C	-1.92462	-1.42996	0.23770
C	-1.60932	-0.25738	-0.44828
C	-0.08824	-2.65531	-0.87887
C	-1.11925	-2.68666	0.26740
H	0.66384	-3.43655	-0.72094
H	-0.59981	-2.88943	-1.81970
H	-0.61201	-2.78750	1.23700
H	-1.76341	-3.56877	0.16981
C	-0.40578	-0.15650	-1.24924
O	-0.17531	0.75857	-2.04545
C	0.60804	-1.28586	-1.03197
C	1.48682	-0.98299	0.20135
H	0.89059	-1.13160	1.10750
H	2.28964	-1.74049	0.22852
C	4.01257	0.17489	1.67721
C	2.59390	0.73459	1.54110
N	1.96676	0.39894	0.25673
C	2.87122	0.79113	-0.83378
C	4.27953	0.22194	-0.66501
O	4.83490	0.61134	0.59390
H	3.99503	-0.92638	1.71159
H	4.48921	0.53695	2.59343

H	2.64535	1.82906	1.61364
H	1.96073	0.37216	2.35891
H	2.43637	0.50160	-1.79254
H	2.93889	1.88689	-0.82464
H	4.27053	-0.87734	-0.74323
H	4.95405	0.61183	-1.43350
C	-2.71491	2.10602	-0.71705
H	-2.47297	2.11924	-1.78504
H	-3.72874	2.51022	-0.61170
C	-1.72104	3.01622	0.02383
H	-0.70071	2.63884	-0.09355
H	-1.75602	4.03932	-0.36876
H	-1.95071	3.05220	1.09490
C	-3.58712	0.06590	0.60511
C	-4.87298	0.53564	1.20168
H	-5.11735	1.53582	0.83563
H	-5.70749	-0.12868	0.94349
H	-4.82165	0.58534	2.29706
H	1.23924	-1.31736	-1.92576

4s_Renantiomer_conformer-058

C	-3.15230	0.25350	-0.04126
N	-2.70924	-1.86492	0.54004
H	-2.84401	-2.80221	0.89477
C	-1.57123	-1.41186	-0.04237
C	-1.79733	-0.08705	-0.41792
C	0.50070	-1.50267	-1.36869
C	-0.31354	-2.18380	-0.25363
H	1.49525	-1.95629	-1.41754
H	0.01149	-1.70478	-2.32909
H	0.26988	-2.22523	0.67691
H	-0.52981	-3.22383	-0.52504
C	-0.76813	0.69794	-1.06245
O	-0.92103	1.86257	-1.44676
C	0.61045	0.02679	-1.19155
C	1.47414	0.46268	0.02421
H	1.00284	1.32147	0.51368
H	1.48086	-0.34763	0.77134
C	4.21701	-1.11906	0.02493
C	3.67019	-0.10778	-0.98409
N	2.81553	0.91547	-0.36542
C	3.54877	1.52638	0.75279
C	4.10628	0.49630	1.73873
O	4.93500	-0.45293	1.06521
H	4.92575	-1.80271	-0.45214

H	3.40206	-1.71988	0.46027
H	3.12578	-0.61373	-1.78545
H	4.52084	0.41010	-1.44584
H	4.38996	2.09282	0.33187
H	2.89200	2.23356	1.27180
H	4.73736	0.97862	2.49138
H	3.28924	-0.02688	2.26073
C	-3.81543	1.58821	-0.22234
H	-3.63460	1.94609	-1.24189
H	-4.90072	1.47922	-0.11022
C	-3.30297	2.64638	0.76798
H	-3.47476	2.32819	1.80258
H	-2.22933	2.80286	0.62760
H	-3.81208	3.60538	0.61544
C	-3.68802	-0.86775	0.55344
C	-5.03074	-1.13137	1.15112
H	-5.52062	-1.99549	0.68457
H	-4.96515	-1.33476	2.22776
H	-5.68256	-0.26485	1.01661
H	1.06749	0.46783	-2.08344

4s_Renantiomer_conformer-059

C	-3.08214	0.19365	0.40947
N	-2.62228	-1.98466	0.15440
H	-2.73749	-2.98918	0.16488
C	-1.52293	-1.33472	-0.30183
C	-1.76242	0.03289	-0.16243
C	0.46802	-0.90948	-1.68422
C	-0.28419	-1.95960	-0.84690
H	1.45447	-1.30026	-1.95199
H	-0.07885	-0.75646	-2.62233
H	0.35124	-2.32491	-0.02783
H	-0.52236	-2.83505	-1.46255
C	-0.76070	1.01191	-0.52153
O	-0.91997	2.23425	-0.43149
C	0.60105	0.45275	-0.97063
C	1.54894	0.43175	0.25999
H	1.11547	1.03825	1.06204
H	1.60840	-0.59617	0.65340
C	4.28090	-0.97619	-0.49150
C	3.66806	0.31891	-1.02714
N	2.86012	1.02862	-0.02507
C	3.67022	1.21022	1.18821
C	4.29177	-0.09384	1.69500
O	5.07010	-0.71567	0.67079

H	4.95379	-1.42413	-1.22894
H	3.49637	-1.71239	-0.25227
H	3.06834	0.12473	-1.92013
H	4.48576	0.98836	-1.32403
H	4.48170	1.90960	0.94733
H	3.05249	1.66664	1.96979
H	4.97417	0.09738	2.52878
H	3.51127	-0.78894	2.04325
C	-3.76926	1.49503	0.70585
H	-4.61027	1.32145	1.38767
H	-3.07142	2.16191	1.22419
C	-4.27537	2.20272	-0.56129
H	-3.43537	2.42763	-1.22501
H	-4.98661	1.56984	-1.10420
H	-4.77701	3.14487	-0.31057
C	-3.58793	-1.07463	0.59245
C	-4.89532	-1.55633	1.12967
H	-5.49848	-0.71239	1.47292
H	-5.47622	-2.09241	0.36805
H	-4.75979	-2.23917	1.97800
H	1.00230	1.19427	-1.66921

4s_Renantiomer_conformer-060

C	3.37957	0.28628	0.26492
N	3.16307	-1.90057	-0.17445
H	3.41097	-2.86176	-0.36713
C	1.89524	-1.42007	-0.12843
C	1.97862	-0.05486	0.14413
C	-0.47951	-1.21706	-0.71669
C	0.63649	-2.19588	-0.32263
H	-0.29943	-0.85620	-1.73820
H	-1.44902	-1.72192	-0.72292
H	0.76369	-2.97303	-1.08561
H	0.36196	-2.71338	0.60922
C	0.78957	0.75813	0.29916
O	0.81293	1.97594	0.50994
C	-0.54596	-0.00621	0.23078
C	-1.69253	0.94710	-0.12748
H	-1.69227	1.78939	0.58546
H	-1.49934	1.37067	-1.12079
C	-4.92418	-0.42872	1.18723
C	-3.39815	-0.24200	1.14056
N	-2.98624	0.27493	-0.17228
C	-4.03602	1.10378	-0.75102
C	-5.25518	0.21785	-1.05210

O	-5.42253	-0.82152	-0.08318
H	-5.41794	0.49578	1.52221
H	-5.19186	-1.22048	1.89156
H	-2.89573	-1.20010	1.31686
H	-3.10242	0.44028	1.96004
H	-4.31491	1.94849	-0.09287
H	-3.67315	1.54212	-1.68619
H	-6.16391	0.83726	-1.10517
H	-5.12604	-0.28986	-2.01300
C	3.94362	1.65331	0.52422
H	3.38539	2.12608	1.33987
H	4.98414	1.56577	0.85903
C	3.87890	2.56766	-0.70965
H	2.83889	2.70316	-1.02075
H	4.30313	3.55450	-0.48959
H	4.43753	2.13376	-1.54683
C	4.08643	-0.87891	0.06059
C	5.55245	-1.16273	0.04612
H	5.82310	-1.94466	0.76700
H	5.89369	-1.49704	-0.94222
H	6.11548	-0.26229	0.30324
H	-0.70914	-0.38846	1.25160

4s_Renantiomer_conformer-061

C	3.10921	0.44982	0.06513
N	2.92204	-1.70566	-0.51789
H	3.16655	-2.61960	-0.87476
C	1.73718	-1.39184	0.06303
C	1.80381	-0.04924	0.43984
C	-0.30629	-1.74085	1.38989
C	0.57515	-2.30548	0.26234
H	-1.25644	-2.28017	1.42326
H	0.20338	-1.89779	2.34812
H	-0.00766	-2.38756	-0.66652
H	0.90948	-3.32063	0.50652
C	0.68264	0.60753	1.07769
O	0.68073	1.78961	1.44088
C	-0.59024	-0.23898	1.22170
C	-1.48197	0.06678	-0.00627
H	-1.62439	1.15807	-0.05497
H	-0.94072	-0.21401	-0.91625
C	-4.07240	0.40100	-1.79353
C	-3.35020	-0.85807	-1.31068
N	-2.73287	-0.69228	0.01078
C	-3.73511	-0.17735	0.95470

C	-4.45444	1.06809	0.43191
O	-5.05302	0.81294	-0.83998
H	-4.60905	0.21217	-2.72842
H	-3.35300	1.21798	-1.96656
H	-2.58320	-1.16120	-2.03169
H	-4.08481	-1.67138	-1.23875
H	-4.47937	-0.96712	1.11958
H	-3.26023	0.03436	1.91763
H	-5.26666	1.35985	1.10458
H	-3.75601	1.91620	0.34973
C	3.60848	1.85435	0.24453
H	3.37371	2.19578	1.25874
H	4.70047	1.87359	0.14676
C	2.98813	2.83700	-0.76178
H	3.21353	2.53510	-1.79096
H	1.90133	2.86279	-0.63932
H	3.37501	3.85157	-0.60962
C	3.77612	-0.59995	-0.52789
C	5.14372	-0.70341	-1.11855
H	5.73162	-1.50060	-0.64595
H	5.10844	-0.91736	-2.19460
H	5.68658	0.23543	-0.98513
H	-1.09714	0.13333	2.11876

4s_Renantiomer_conformer-062

C	3.38007	0.31376	-0.20682
N	3.17032	-1.89950	0.07507
H	3.41933	-2.87578	0.16056
C	1.90125	-1.42051	0.06568
C	1.98050	-0.03827	-0.10231
C	-0.50498	-1.40615	-0.41794
C	0.64519	-2.21198	0.20392
H	-0.38649	-1.39227	-1.50968
H	-1.46558	-1.88336	-0.20728
H	0.74030	-3.19330	-0.27576
H	0.43099	-2.40174	1.26673
C	0.79038	0.78642	-0.14785
O	0.80916	2.00903	-0.32956
C	-0.53841	0.04476	0.09350
C	-1.71052	0.83810	-0.49728
H	-1.68306	1.86659	-0.09840
H	-1.57053	0.91381	-1.58269
C	-5.30894	-0.12151	-0.95606
C	-4.08234	0.80161	-1.02215
N	-2.99894	0.19765	-0.25694

C	-3.34143	0.14119	1.17151
C	-4.86215	-0.00046	1.35257
O	-5.42356	-0.78176	0.30809
H	-5.22305	-0.91878	-1.70092
H	-6.22282	0.45507	-1.16736
H	-4.33368	1.81795	-0.66389
H	-3.76992	0.90606	-2.06611
H	-2.82668	-0.71362	1.62520
H	-3.00724	1.04921	1.70837
H	-5.08994	-0.51393	2.29017
H	-5.34192	0.98904	1.38980
C	3.94049	1.69798	-0.36234
H	4.97951	1.63912	-0.70785
H	3.37672	2.23125	-1.13542
C	3.88104	2.51276	0.93974
H	2.84308	2.62038	1.26841
H	4.44630	2.01625	1.73669
H	4.30080	3.51531	0.79528
C	4.09044	-0.86127	-0.08968
C	5.55677	-1.14310	-0.11101
H	5.82015	-1.87004	-0.88991
H	6.11650	-0.22558	-0.30793
H	5.90865	-1.54830	0.84653
H	-0.65007	0.01151	1.18958

4s_Renantiomer_conformer-063

C	-2.61561	0.59548	0.36297
N	-2.92314	-1.61299	0.58015
H	-3.35019	-2.49385	0.83303
C	-1.77782	-1.48236	-0.13777
C	-1.54416	-0.11624	-0.29714
C	0.01649	-2.04063	-1.74368
C	-0.93616	-2.59613	-0.66638
H	0.79584	-2.77864	-1.96462
H	-0.54982	-1.88600	-2.66944
H	-0.36473	-3.06029	0.14944
H	-1.56023	-3.39222	-1.08997
C	-0.38464	0.36746	-1.01946
O	-0.21602	1.54333	-1.35737
C	0.66844	-0.70036	-1.34136
C	1.61960	-0.90265	-0.14200
H	1.08407	-1.43650	0.64994
H	2.43049	-1.57371	-0.47609
C	4.33220	0.65921	-0.58758
C	2.91042	1.19331	-0.41623

N	2.08724	0.34720	0.45956
C	2.80048	0.13657	1.72562
C	4.22991	-0.37549	1.52838
O	4.97104	0.50500	0.68241
H	4.94771	1.36055	-1.15933
H	4.32733	-0.30530	-1.12077
H	2.41257	1.31692	-1.38004
H	2.96929	2.18605	0.04927
H	2.84785	1.10082	2.24908
H	2.22961	-0.55875	2.35160
H	4.76633	-0.41388	2.48153
H	4.22388	-1.38938	1.09693
C	-2.78519	2.08590	0.42617
H	-3.45394	2.34703	1.25518
H	-1.81628	2.55011	0.64230
C	-3.33442	2.67887	-0.88115
H	-2.64520	2.46597	-1.70336
H	-4.31166	2.24696	-1.12558
H	-3.45085	3.76626	-0.80106
C	-3.45134	-0.36053	0.89883
C	-4.71558	-0.24567	1.68519
H	-4.94124	0.80412	1.88790
H	-5.57186	-0.67130	1.14604
H	-4.64482	-0.76593	2.64884
H	1.24124	-0.32557	-2.19565

4s_Renantiomer_conformer-064

C	3.02535	0.32035	-0.46312
N	2.79258	-1.87111	-0.05778
H	3.00395	-2.85905	-0.01709
C	1.65920	-1.30025	0.42136
C	1.75808	0.07292	0.19004
C	-0.29879	-0.98391	1.87973
C	0.51084	-2.00487	1.06168
H	-1.24635	-1.42861	2.19448
H	0.26750	-0.72650	2.78288
H	-0.12820	-2.46502	0.29418
H	0.85863	-2.82333	1.70314
C	0.68021	0.97289	0.54158
O	0.70731	2.19644	0.36525
C	-0.58770	0.30630	1.09427
C	-1.54362	0.07851	-0.10167
H	-1.68375	1.04639	-0.60881
H	-1.05411	-0.58081	-0.82656
C	-4.22492	-0.31254	-1.73568

C	-3.48406	-1.26334	-0.79383
N	-2.79557	-0.56511	0.29844
C	-3.74254	0.33199	0.97595
C	-4.48303	1.25320	0.00384
O	-5.15058	0.49481	-1.00628
H	-4.81228	-0.86828	-2.47313
H	-3.51190	0.33150	-2.27578
H	-2.75845	-1.86675	-1.35009
H	-4.21790	-1.94698	-0.34615
H	-4.48069	-0.29012	1.49841
H	-3.21493	0.92227	1.73144
H	-5.25639	1.82699	0.52348
H	-3.78603	1.96500	-0.46671
C	3.56621	1.65920	-0.87403
H	4.38852	1.52217	-1.58631
H	2.78322	2.22039	-1.39621
C	4.05662	2.49506	0.31896
H	4.85111	1.97102	0.86242
H	4.45037	3.46234	-0.01495
H	3.22963	2.68185	1.01028
C	3.64263	-0.90383	-0.59980
C	4.96257	-1.29353	-1.17920
H	4.85219	-2.03333	-1.98227
H	5.46624	-0.41888	-1.59779
H	5.62737	-1.72949	-0.42228
H	-1.04287	1.04179	1.76670

4s_Renantiomer_conformer-065

C	2.56003	0.40955	0.11838
N	2.29657	-1.21593	-1.40098
H	2.46103	-1.79404	-2.21410
C	1.31962	-1.42821	-0.48431
C	1.44123	-0.43240	0.48635
C	-0.27790	-2.69216	0.89694
C	0.29927	-2.51353	-0.51906
H	-1.17043	-3.32748	0.85601
H	0.46341	-3.22580	1.50466
H	-0.49953	-2.24299	-1.21734
H	0.74147	-3.45457	-0.86970
C	0.51799	-0.34407	1.59349
O	0.56510	0.52983	2.46989
C	-0.62113	-1.37202	1.61523
C	-1.93536	-0.71653	1.12341
H	-2.73853	-1.44767	1.26459
H	-2.16011	0.12865	1.79077

C	-4.26474	0.31786	-0.79285
C	-3.11254	-0.65358	-1.05183
N	-1.90626	-0.31903	-0.28830
C	-1.55530	1.08970	-0.51391
C	-2.73645	2.03045	-0.27114
O	-3.86065	1.65902	-1.07121
H	-4.60436	0.23921	0.25349
H	-5.11576	0.10788	-1.44840
H	-2.85847	-0.61223	-2.11972
H	-3.41413	-1.68071	-0.82154
H	-0.71405	1.36515	0.12521
H	-1.22667	1.19613	-1.55603
H	-3.02235	2.02319	0.79344
H	-2.48308	3.05802	-0.54965
C	3.03810	1.62814	0.85401
H	3.08979	1.40677	1.92576
H	4.05576	1.87774	0.53061
C	2.12507	2.84775	0.64895
H	1.12562	2.63741	1.04069
H	2.51963	3.72599	1.17377
H	2.03663	3.09546	-0.41493
C	3.06633	-0.10117	-1.05624
C	4.20447	0.33093	-1.92087
H	3.86442	0.64537	-2.91613
H	4.72623	1.17684	-1.46680
H	4.93494	-0.47541	-2.06528
H	-0.79132	-1.59327	2.67675

4s_Renantiomer_conformer-066

C	2.55361	-0.69784	-0.20563
N	3.00375	1.12005	1.02526
H	3.48962	1.74933	1.65019
C	1.86280	1.41903	0.35546
C	1.53870	0.30925	-0.42582
C	0.18865	2.78757	-0.81840
C	1.09973	2.69936	0.42023
H	-0.51462	3.62005	-0.70722
H	0.80892	3.00191	-1.69683
H	0.49748	2.73559	1.33906
H	1.77576	3.56148	0.46088
C	0.36641	0.30202	-1.27521
O	0.11851	-0.59399	-2.09122
C	-0.59861	1.48231	-1.06705
C	-1.58542	1.20217	0.09928
H	-1.08954	0.55840	0.84503

H	-1.79242	2.14974	0.60968
C	-3.68228	-0.45654	1.73108
C	-3.88330	0.68897	0.73488
N	-2.88799	0.69453	-0.34686
C	-2.85993	-0.64038	-0.96717
C	-2.66696	-1.76258	0.05227
O	-3.67926	-1.71796	1.06174
H	-4.50232	-0.49163	2.45506
H	-2.73993	-0.32755	2.28743
H	-3.86002	1.65314	1.25577
H	-4.87456	0.57738	0.27580
H	-3.82718	-0.78926	-1.46519
H	-2.07489	-0.67846	-1.72457
H	-1.67167	-1.69738	0.52133
H	-2.75088	-2.74322	-0.42626
C	2.57892	-2.07479	-0.80319
H	2.41030	-2.00447	-1.88320
H	3.57066	-2.52044	-0.66233
C	1.51189	-3.00203	-0.19849
H	0.51190	-2.59627	-0.37936
H	1.56044	-4.00151	-0.64638
H	1.65154	-3.10404	0.88367
C	3.44280	-0.16690	0.70308
C	4.67730	-0.72591	1.33007
H	4.56732	-0.84074	2.41613
H	4.90188	-1.71078	0.91361
H	5.54855	-0.08180	1.15599
H	-1.18649	1.59168	-1.98373

4s_Renantiomer_conformer-067

C	-2.41578	-0.49789	-0.28792
N	-2.05797	1.41967	-1.38993
H	-2.15504	2.15422	-2.07790
C	-1.18067	1.43819	-0.35582
C	-1.36814	0.25824	0.36589
C	0.27510	2.36852	1.39628
C	-0.18197	2.50543	-0.06709
H	1.15532	2.99840	1.57075
H	-0.52338	2.75334	2.04279
H	0.67470	2.39640	-0.74066
H	-0.61128	3.50057	-0.23906
C	-0.53377	-0.07223	1.49730
O	-0.63359	-1.11875	2.15287
C	0.58549	0.92255	1.83122
C	1.93824	0.38692	1.30048

H	2.71834	1.08578	1.62111
H	2.14466	-0.56706	1.80744
C	4.38850	-0.19556	-0.66072
C	3.20932	0.76935	-0.78904
N	1.98759	0.25877	-0.16005
C	1.70264	-1.09459	-0.65655
C	2.91170	-2.02407	-0.53798
O	4.05007	-1.47275	-1.20255
H	4.68151	-0.30387	0.39701
H	5.25643	0.16065	-1.22454
H	3.00142	0.92155	-1.85693
H	3.45878	1.74419	-0.35719
H	0.85113	-1.51144	-0.11393
H	1.41736	-1.01698	-1.71381
H	3.15513	-2.20556	0.52178
H	2.71264	-2.98972	-1.01294
C	-2.96418	-1.82281	0.15629
H	-3.53356	-2.27970	-0.66194
H	-2.13329	-2.50005	0.38356
C	-3.85809	-1.71069	1.40147
H	-4.24706	-2.69272	1.69565
H	-3.28329	-1.30486	2.23915
H	-4.70997	-1.04753	1.21230
C	-2.82370	0.25014	-1.36993
C	-3.86835	0.01039	-2.40964
H	-3.44703	0.03160	-3.42287
H	-4.33325	-0.96734	-2.26190
H	-4.66371	0.76604	-2.37085
H	0.67603	0.90552	2.92490

4s_Renantiomer_conformer-068

C	3.38112	0.28833	0.26770
N	3.17180	-1.89062	-0.21259
H	3.42292	-2.84650	-0.42640
C	1.90207	-1.41756	-0.14712
C	1.98089	-0.05751	0.15137
C	-0.47807	-1.21622	-0.71408
C	0.64556	-2.19590	-0.34478
H	-0.30790	-0.83859	-1.73134
H	-1.44555	-1.72447	-0.71912
H	0.77044	-2.95943	-1.12175
H	0.38098	-2.73051	0.58027
C	0.78966	0.74660	0.33225
O	0.80990	1.95892	0.57311
C	-0.54450	-0.02037	0.25206

C	-1.69090	0.93819	-0.09277
H	-1.69397	1.77778	0.62235
H	-1.49108	1.37291	-1.08035
C	-4.71154	-1.08922	0.87024
C	-3.52017	-0.15018	1.12265
N	-2.99279	0.28207	-0.16577
C	-3.97286	1.13458	-0.85107
C	-5.40497	0.66887	-0.52863
O	-5.44319	-0.73301	-0.30512
H	-5.37777	-1.09278	1.74703
H	-4.35843	-2.11100	0.70021
H	-2.75331	-0.69927	1.67519
H	-3.81463	0.70771	1.75716
H	-3.87691	2.19508	-0.55004
H	-3.77813	1.08285	-1.92908
H	-5.79625	1.20409	0.34971
H	-6.07528	0.87493	-1.36698
C	3.94052	1.65308	0.54818
H	3.38693	2.10740	1.37730
H	4.98435	1.56462	0.87227
C	3.86054	2.59022	-0.66758
H	4.41442	2.17527	-1.51743
H	2.81730	2.72595	-0.96761
H	4.28133	3.57504	-0.43242
C	4.09202	-0.86899	0.03488
C	5.55926	-1.14476	0.00254
H	5.89470	-1.45362	-0.99603
H	6.11969	-0.24778	0.27688
H	5.83925	-1.94223	0.70251
H	-0.70168	-0.41974	1.26731

4s_Renantiomer_conformer-069

C	3.37984	0.32737	-0.19400
N	3.17211	-1.89745	-0.02305
H	3.42192	-2.87658	0.01289
C	1.90301	-1.41918	0.00069
C	1.98099	-0.03011	-0.09744
C	-0.50672	-1.38213	-0.46464
C	0.64811	-2.21719	0.10786
H	-0.39665	-1.31447	-1.55539
H	-1.46615	-1.86767	-0.26947
H	0.74005	-3.17306	-0.42130
H	0.44180	-2.46043	1.16131
C	0.79079	0.79550	-0.09054
O	0.80866	2.02636	-0.20378

C	-0.53741	0.04175	0.11758
C	-1.70974	0.86417	-0.43169
H	-1.68658	1.87527	0.00821
H	-1.56377	0.99263	-1.51171
C	-4.65756	-0.78524	1.22758
C	-3.46457	0.18079	1.13737
N	-3.00674	0.22043	-0.24602
C	-4.02860	0.84381	-1.09703
C	-5.43806	0.49789	-0.58156
O	-5.45119	-0.77959	0.03854
H	-5.27861	-0.53253	2.10112
H	-4.30322	-1.81435	1.34147
H	-2.66571	-0.18950	1.78510
H	-3.73532	1.18670	1.51172
H	-3.92829	1.94560	-1.11976
H	-3.88766	0.48258	-2.12286
H	-5.79017	1.26571	0.12378
H	-6.15157	0.45656	-1.40851
C	3.93977	1.71756	-0.28556
H	4.97559	1.67534	-0.64298
H	3.36974	2.28860	-1.02642
C	3.89300	2.46759	1.05531
H	4.46442	1.93206	1.82204
H	4.31322	3.47547	0.95636
H	2.85816	2.56039	1.39789
C	4.09100	-0.85188	-0.14146
C	5.55712	-1.13197	-0.18748
H	5.91535	-1.58600	0.74549
H	5.81506	-1.81776	-1.00460
H	6.11571	-0.20542	-0.34049
H	-0.64050	-0.04656	1.21150

4s_Renantiomer_conformer-070

C	-2.34812	-0.87553	-0.19685
N	-3.06720	1.10094	-0.96887
H	-3.64188	1.78655	-1.44005
C	-1.96230	1.38746	-0.23372
C	-1.47637	0.17993	0.26876
C	-0.42057	2.67890	1.20112
C	-1.38475	2.74372	0.00019
H	0.18338	3.59241	1.24094
H	-1.01035	2.64425	2.12446
H	-0.85696	3.09199	-0.89855
H	-2.17530	3.47921	0.19135
C	-0.29064	0.13453	1.09922

O	0.09166	-0.86909	1.70992
C	0.50576	1.44490	1.16814
C	1.48891	1.53724	-0.01850
H	0.92541	1.73938	-0.93560
H	2.14921	2.41141	0.15027
C	3.86895	-1.33453	0.52797
C	3.14233	-0.03411	0.84865
N	2.25230	0.31882	-0.25829
C	3.01638	0.41153	-1.50164
C	3.73934	-0.90373	-1.76639
O	4.60654	-1.24234	-0.68821
H	3.13242	-2.15084	0.45787
H	4.59060	-1.57683	1.31308
H	3.88421	0.76576	1.04102
H	2.54706	-0.18113	1.75236
H	2.32888	0.62426	-2.32871
H	3.76016	1.23173	-1.46325
H	2.99816	-1.70494	-1.91678
H	4.36456	-0.82855	-2.66052
C	-2.21935	-2.33749	0.11994
H	-2.77338	-2.92655	-0.62080
H	-1.16714	-2.63241	0.03769
C	-2.71963	-2.68766	1.53045
H	-3.77524	-2.41743	1.64859
H	-2.61658	-3.76118	1.72862
H	-2.13628	-2.14237	2.27789
C	-3.32206	-0.27202	-0.96272
C	-4.49314	-0.83187	-1.70105
H	-5.44442	-0.46045	-1.29841
H	-4.46576	-0.57203	-2.76694
H	-4.50392	-1.92173	-1.62275
H	1.07363	1.41501	2.10322

4s_Renantiomer_conformer-071

C	2.41510	-0.81809	-0.41226
N	3.25970	0.77821	0.91586
H	3.88588	1.25587	1.54992
C	2.11474	1.30263	0.41094
C	1.55092	0.34093	-0.42502
C	0.04384	2.60644	0.48553
C	1.57285	2.67375	0.63020
H	-0.35916	2.09435	1.36325
H	-0.37535	3.61855	0.49693
H	1.84812	3.05908	1.61854
H	1.99414	3.37148	-0.10867

C	0.36169	0.62888	-1.19925
O	-0.01612	-0.05366	-2.15519
C	-0.42099	1.89909	-0.80880
C	-1.94519	1.63277	-0.82748
H	-2.44075	2.47363	-0.30327
H	-2.29122	1.65972	-1.86493
C	-4.25756	-1.19881	0.01225
C	-3.82484	0.19192	-0.43443
N	-2.37811	0.34208	-0.29086
C	-1.97974	0.06885	1.08770
C	-2.46582	-1.31588	1.50344
O	-3.87650	-1.44695	1.36248
H	-3.80444	-1.95461	-0.64889
H	-5.34580	-1.29789	-0.03054
H	-4.37439	0.94902	0.15980
H	-4.09568	0.33739	-1.48670
H	-0.88918	0.07930	1.16192
H	-2.37947	0.82449	1.79287
H	-1.95667	-2.07800	0.89197
H	-2.23686	-1.49986	2.55683
C	2.15889	-2.11306	-1.12680
H	1.97649	-1.91174	-2.18832
H	3.04948	-2.74984	-1.07008
C	0.94852	-2.87294	-0.55847
H	0.03948	-2.27370	-0.66770
H	0.79711	-3.82128	-1.08726
H	1.09028	-3.09298	0.50580
C	3.45883	-0.51980	0.43715
C	4.63801	-1.31927	0.88463
H	4.65690	-2.28542	0.37470
H	5.58339	-0.80673	0.66543
H	4.61401	-1.51288	1.96486
H	-0.23197	2.60040	-1.63569

4s_Renantiomer_conformer-072

C	-3.32905	0.40700	-0.33345
N	-3.32788	-1.78992	0.10860
H	-3.66829	-2.72546	0.28511
C	-2.02294	-1.42088	0.13794
C	-1.97196	-0.05393	-0.13385
C	0.32138	-1.42345	0.86542
C	-0.84971	-2.30258	0.40200
H	0.11272	-1.04765	1.87606
H	1.24177	-2.01003	0.92670
H	-1.08838	-3.06552	1.15257

H	-0.56663	-2.84245	-0.51449
C	-0.70988	0.65220	-0.21879
O	-0.61515	1.86620	-0.43165
C	0.54871	-0.22374	-0.07077
C	1.74718	0.63034	0.36182
H	1.85454	1.46940	-0.34662
H	1.52572	1.06577	1.34317
C	4.90280	-0.97663	-0.80653
C	3.40934	-0.68601	-0.81845
N	2.97586	-0.15300	0.48227
C	4.06513	0.57707	1.15730
C	5.04566	1.20501	0.17195
O	5.68281	0.20993	-0.63906
H	5.20816	-1.42013	-1.75896
H	5.13147	-1.70008	-0.00914
H	2.86189	-1.60824	-1.04913
H	3.20016	0.02705	-1.63763
H	3.63607	1.35351	1.79734
H	4.61747	-0.10915	1.81312
H	4.51992	1.92769	-0.47063
H	5.84043	1.74215	0.70090
C	-3.75742	1.81791	-0.61625
H	-3.11845	2.24032	-1.39961
H	-4.78262	1.82143	-1.00524
C	-3.67787	2.72283	0.62371
H	-2.64779	2.76669	0.98934
H	-4.00223	3.74307	0.38681
H	-4.31531	2.33903	1.42843
C	-4.14459	-0.69248	-0.17489
C	-5.62807	-0.84874	-0.24685
H	-5.92305	-1.59821	-0.99234
H	-6.05238	-1.16109	0.71615
H	-6.09624	0.09915	-0.52293
H	0.74169	-0.61925	-1.08131

4s_Renantiomer_conformer-073

C	2.75746	0.54786	0.21834
N	3.05679	-1.30159	-1.01181
H	3.48118	-1.96286	-1.64820
C	1.86604	-1.47885	-0.38713
C	1.63456	-0.34758	0.39443
C	-0.45801	-2.21538	-0.15144
C	0.97359	-2.66935	-0.47313
H	-1.11960	-3.08573	-0.07578
H	-0.83570	-1.60455	-0.97687

H	1.02040	-3.12932	-1.46725
H	1.29679	-3.43856	0.24447
C	0.47425	-0.24980	1.25370
O	0.30803	0.65879	2.07548
C	-0.54956	-1.40151	1.15213
C	-1.98919	-0.92656	1.42682
H	-2.59060	-1.80341	1.69074
H	-1.97423	-0.25579	2.30252
C	-2.97698	1.76242	-1.02599
C	-2.01379	0.97294	-0.12209
N	-2.62254	-0.30495	0.26905
C	-4.06602	-0.16377	0.39965
C	-4.65808	0.11854	-0.99202
O	-3.77232	0.88597	-1.81115
H	-2.41924	2.39404	-1.72228
H	-3.62030	2.42054	-0.42227
H	-1.75809	1.59213	0.75465
H	-1.07605	0.76721	-0.65210
H	-4.48801	-1.09739	0.78487
H	-4.34782	0.63091	1.11644
H	-4.82175	-0.81945	-1.53152
H	-5.62556	0.63481	-0.89032
C	2.90782	1.91016	0.83077
H	2.71886	1.84689	1.90799
H	3.93971	2.25947	0.70697
C	1.94246	2.94095	0.22309
H	0.90636	2.62872	0.38503
H	2.07896	3.92635	0.68401
H	2.10773	3.04145	-0.85567
C	3.61698	-0.06917	-0.66437
C	4.91974	0.36323	-1.25248
H	5.71815	-0.36216	-1.05038
H	4.85573	0.48375	-2.34170
H	5.22671	1.32316	-0.83042
H	-0.28657	-2.06736	1.99007

4s_Renantiomer_conformer-074

C	-3.25206	0.20325	-0.03895
N	-2.67566	-1.96086	0.03468
H	-2.75269	-2.96287	0.14646
C	-1.55541	-1.30950	-0.36651
C	-1.86559	0.05050	-0.42269
C	0.55819	-0.95237	-1.57642
C	-0.23478	-1.92503	-0.68655
H	1.59279	-1.29466	-1.66424

H	0.11859	-0.95341	-2.58108
H	0.32507	-2.12793	0.23766
H	-0.36118	-2.89098	-1.18941
C	-0.87342	1.03199	-0.80724
O	-1.09114	2.24554	-0.89846
C	0.54426	0.48754	-1.03409
C	1.30508	0.62343	0.30492
H	1.28816	1.68894	0.60344
H	0.76857	0.06647	1.08153
C	5.00091	0.64285	-0.32193
C	3.52060	0.86573	-0.67413
N	2.65970	0.09785	0.23647
C	3.28747	-0.05258	1.54336
C	4.54916	-0.91566	1.38594
O	5.21421	-0.68275	0.14083
H	5.33357	1.36998	0.43382
H	5.62888	0.77463	-1.20667
H	3.32397	0.54269	-1.70265
H	3.30490	1.94955	-0.62273
H	3.53125	0.92151	2.00727
H	2.59117	-0.55781	2.22018
H	5.23712	-0.72838	2.22481
H	4.28304	-1.97726	1.38674
C	-4.00179	1.49837	0.08174
H	-3.80897	2.11235	-0.80478
H	-5.08009	1.30064	0.10465
C	-3.60248	2.30025	1.33109
H	-3.79117	1.72170	2.24259
H	-2.53761	2.54825	1.29335
H	-4.17055	3.23586	1.39525
C	-3.72413	-1.06060	0.24079
C	-5.06334	-1.54516	0.68986
H	-5.76962	-0.71309	0.74209
H	-5.47702	-2.29328	0.00161
H	-5.01807	-2.00619	1.68498
H	1.01108	1.15402	-1.76710

4s_Renantiomer_conformer-075

C	3.19104	0.08051	-0.37882
N	2.58901	-1.97628	0.27561
H	2.64412	-2.96630	0.47378
C	1.50736	-1.19567	0.52174
C	1.83566	0.10265	0.12730
C	-0.52355	-0.40761	1.66536
C	0.20303	-1.63766	1.09510

H	-1.55316	-0.67319	1.91925
H	-0.02038	-0.09574	2.58845
H	-0.41461	-2.11100	0.31848
H	0.35113	-2.39380	1.87512
C	0.87257	1.18006	0.21411
O	1.10157	2.34919	-0.11630
C	-0.53293	0.77762	0.68415
C	-1.37893	0.49371	-0.57815
H	-1.37670	1.40475	-1.20633
H	-0.89803	-0.29830	-1.16352
C	-4.69550	-1.22597	-0.90211
C	-3.44342	-0.49210	-1.40752
N	-2.72878	0.05394	-0.26047
C	-3.52301	1.09604	0.40504
C	-5.02459	0.80928	0.23749
O	-5.27325	-0.58895	0.24119
H	-4.43499	-2.23839	-0.57818
H	-5.43802	-1.29947	-1.71166
H	-3.71344	0.28735	-2.14443
H	-2.79630	-1.20570	-1.92733
H	-3.25858	1.11661	1.46832
H	-3.30709	2.09968	-0.00721
H	-5.59072	1.23420	1.07020
H	-5.40510	1.26300	-0.68981
C	3.96999	1.26051	-0.88336
H	4.83653	0.91257	-1.45812
H	3.34389	1.83976	-1.57107
C	4.44474	2.18728	0.24724
H	3.58384	2.58065	0.79572
H	5.08645	1.64616	0.95193
H	5.01362	3.03493	-0.15281
C	3.62973	-1.22115	-0.27105
C	4.93388	-1.86267	-0.61391
H	4.80683	-2.69050	-1.32314
H	5.60704	-1.13275	-1.06992
H	5.43516	-2.26762	0.27473
H	-0.94608	1.65860	1.18681

4s_Renantiomer_conformer-076

C	2.70365	-0.50956	0.32689
N	2.89174	1.69535	0.68128
H	3.25752	2.57891	1.00975
C	1.74009	1.54479	-0.01891
C	1.58009	0.18193	-0.26780
C	-0.58182	2.01861	-0.64298

C	0.81896	2.62477	-0.47244
H	-0.99885	1.79208	0.34287
H	-1.25283	2.74973	-1.10768
H	0.79836	3.45483	0.24346
H	1.16778	3.04307	-1.42875
C	0.45913	-0.31866	-1.03444
O	0.33777	-1.50121	-1.37458
C	-0.57612	0.73323	-1.49041
C	-1.99057	0.14332	-1.64235
H	-2.56387	0.80664	-2.29927
H	-1.90996	-0.83554	-2.14461
C	-4.84698	0.16947	0.78709
C	-4.14934	-0.15661	-0.54473
N	-2.71829	0.05657	-0.38132
C	-2.15505	-0.91845	0.56093
C	-3.19450	-1.27059	1.63956
O	-4.03559	-0.16024	1.91740
H	-5.81190	-0.35833	0.84442
H	-5.03593	1.24481	0.86218
H	-4.52933	0.51539	-1.32071
H	-4.38749	-1.18636	-0.87285
H	-1.83686	-1.84509	0.05358
H	-1.26017	-0.48327	1.02219
H	-3.79856	-2.13543	1.32555
H	-2.70010	-1.53623	2.57751
C	2.96435	-1.98767	0.28579
H	3.66269	-2.26200	1.08565
H	2.02962	-2.52438	0.48138
C	3.52718	-2.45533	-1.06595
H	2.81443	-2.23058	-1.86474
H	4.47235	-1.94829	-1.29142
H	3.71077	-3.53636	-1.06225
C	3.49611	0.45628	0.90834
C	4.77716	0.36405	1.67017
H	5.59924	0.87228	1.14976
H	4.69175	0.81658	2.66636
H	5.06521	-0.68178	1.80175
H	-0.24952	0.99990	-2.50902

4s_Renantiomer_conformer-077

C	3.35319	0.43274	0.27229
N	3.35960	-1.79533	0.03209
H	3.70366	-2.74151	-0.06114
C	2.05217	-1.43696	-0.01351
C	1.99632	-0.05088	0.13260

C	-0.30079	-1.51938	-0.70381
C	0.88135	-2.34613	-0.17740
H	-1.22092	-2.10948	-0.69297
H	-0.10671	-1.23969	-1.74809
H	1.11476	-3.17417	-0.85731
H	0.61521	-2.79985	0.78945
C	0.73100	0.65391	0.17093
O	0.63229	1.88150	0.27841
C	-0.52411	-0.23815	0.11813
C	-1.73844	0.55463	-0.37925
H	-1.79850	1.51191	0.16281
H	-1.57672	0.80471	-1.43511
C	-5.36994	-0.21862	-0.84098
C	-3.99816	0.32304	-1.21109
N	-2.98308	-0.20932	-0.29446
C	-3.50538	-0.34456	1.08180
C	-4.71867	0.54594	1.33176
O	-5.79916	0.23327	0.44833
H	-5.34503	-1.31885	-0.86297
H	-6.12043	0.11799	-1.56316
H	-4.04049	1.42796	-1.19551
H	-3.74857	0.02764	-2.23758
H	-3.78545	-1.38892	1.27308
H	-2.72310	-0.08830	1.80300
H	-5.09551	0.40638	2.35018
H	-4.43404	1.60354	1.22146
C	3.77804	1.86493	0.42325
H	3.14694	2.35267	1.17433
H	4.80784	1.90628	0.79771
C	3.68018	2.65725	-0.89026
H	2.64573	2.66627	-1.24610
H	4.00351	3.69518	-0.74757
H	4.30949	2.20610	-1.66593
C	4.17310	-0.67277	0.20390
C	5.65815	-0.81523	0.27080
H	5.96628	-1.49393	1.07633
H	6.07260	-1.21021	-0.66580
H	6.12442	0.15552	0.45550
H	-0.70374	-0.54139	1.16304

4s_Renantiomer_conformer-078

C	3.36261	0.41665	-0.23156
N	3.36419	-1.76142	0.29550
H	3.70567	-2.69138	0.49775
C	2.05484	-1.41663	0.21377

C	2.00208	-0.06044	-0.10820
C	-0.32781	-1.69618	-0.29995
C	0.88011	-2.31152	0.42171
H	-0.18320	-1.78829	-1.38486
H	-1.24448	-2.23634	-0.04907
H	1.08337	-3.32411	0.05357
H	0.66063	-2.40650	1.49609
C	0.73789	0.63098	-0.26020
O	0.64105	1.82188	-0.57744
C	-0.51558	-0.20982	0.04979
C	-1.75676	0.38418	-0.62600
H	-1.79287	1.46912	-0.43784
H	-1.65117	0.25986	-1.71086
C	-4.66006	0.96772	1.14045
C	-3.43998	0.05166	1.14197
N	-2.99159	-0.28945	-0.22488
C	-4.05628	-0.08729	-1.21376
C	-5.40003	-0.47742	-0.61859
O	-5.77474	0.36953	0.47395
H	-4.40548	1.92757	0.66554
H	-4.98381	1.17817	2.16487
H	-3.68468	-0.86558	1.69392
H	-2.62919	0.54453	1.68771
H	-4.11499	0.96078	-1.56180
H	-3.85073	-0.70525	-2.09639
H	-6.18944	-0.39509	-1.37246
H	-5.35793	-1.52443	-0.28165
C	3.78890	1.82412	-0.53445
H	4.83810	1.83127	-0.85311
H	3.19844	2.20848	-1.37321
C	3.61376	2.76995	0.66455
H	4.20163	2.42466	1.52268
H	3.93886	3.78654	0.41340
H	2.56144	2.81069	0.96101
C	4.18179	-0.66078	0.02785
C	5.66884	-0.79491	0.05874
H	6.03510	-1.05022	1.06155
H	6.02099	-1.57654	-0.62650
H	6.14080	0.14530	-0.23662
H	-0.64176	-0.13992	1.14317

4s_Renantiomer_conformer-079

C	-2.66965	-0.17597	0.08095
N	-2.08505	1.17995	-1.60464
H	-2.11969	1.66814	-2.48951

C	-1.10419	1.32061	-0.67806
C	-1.42348	0.48972	0.39474
C	0.65521	2.48186	0.59749
C	0.13062	2.13984	-0.80907
H	1.66010	2.91175	0.52518
H	0.00793	3.27109	0.99730
H	0.88000	1.58200	-1.38678
H	-0.06220	3.06220	-1.36936
C	-0.55953	0.38109	1.54830
O	-0.74443	-0.42265	2.47178
C	0.65817	1.31957	1.61818
C	1.98375	0.50403	1.76084
H	2.75432	1.19367	2.12416
H	1.79069	-0.22107	2.56088
C	4.15428	-0.43285	-1.17702
C	3.34621	0.53884	-0.32136
N	2.55711	-0.21754	0.64018
C	1.70318	-1.20847	-0.00116
C	2.55608	-2.12044	-0.87768
O	3.30029	-1.36751	-1.83318
H	4.87714	-0.96947	-0.54216
H	4.69664	0.09967	-1.96352
H	2.73430	1.16142	-0.99688
H	4.02584	1.20676	0.22001
H	1.20118	-1.80085	0.77171
H	0.91733	-0.74894	-0.62498
H	3.24465	-2.70173	-0.24399
H	1.92741	-2.81142	-1.44652
C	-3.37437	-1.19447	0.92935
H	-3.37473	-0.86017	1.97253
H	-4.42338	-1.27233	0.61923
C	-2.71554	-2.58152	0.85621
H	-1.68287	-2.52256	1.21232
H	-3.25380	-3.30600	1.47891
H	-2.70669	-2.95502	-0.17413
C	-3.05388	0.27564	-1.16280
C	-4.24205	-0.03843	-2.01079
H	-4.81534	0.86396	-2.25890
H	-3.95312	-0.51263	-2.95764
H	-4.91075	-0.72554	-1.48675
H	0.56486	1.77710	2.61263

4s_Renantiomer_conformer-080

C	-2.55132	0.24832	-0.41891
N	-1.89764	1.72321	1.13632

H	-1.87269	2.55906	1.70468
C	-1.01710	0.69542	1.22848
C	-1.38577	-0.25411	0.27686
C	0.57493	-0.84503	2.30808
C	0.18083	0.62983	2.10855
H	1.55941	-0.90577	2.78445
H	-0.14095	-1.27473	3.01844
H	1.00107	1.20131	1.65339
H	-0.01468	1.09805	3.08045
C	-0.60844	-1.45461	0.06889
O	-0.82564	-2.26317	-0.84340
C	0.55644	-1.72591	1.03696
C	1.90390	-1.88058	0.26081
H	2.61056	-2.38566	0.92925
H	1.68325	-2.57042	-0.56303
C	4.34861	0.94612	-0.23737
C	3.42953	0.05783	0.59676
N	2.59624	-0.73376	-0.29650
C	1.84246	0.09351	-1.22892
C	2.80524	0.99045	-2.00097
O	3.59502	1.78053	-1.11449
H	5.03804	0.31641	-0.82189
H	4.93155	1.61362	0.40370
H	2.85032	0.70742	1.27591
H	4.03138	-0.61658	1.21645
H	1.30031	-0.55583	-1.92508
H	1.09551	0.72791	-0.72163
H	3.46155	0.36969	-2.63145
H	2.25792	1.69065	-2.63850
C	-3.30773	-0.45526	-1.50811
H	-3.96885	0.25656	-2.01654
H	-2.59908	-0.82480	-2.25728
C	-4.13652	-1.64119	-0.98954
H	-4.68061	-2.12727	-1.80794
H	-3.48121	-2.38469	-0.52616
H	-4.86651	-1.31039	-0.24197
C	-2.84715	1.47117	0.14279
C	-3.93991	2.45237	-0.12736
H	-4.61266	2.56136	0.73319
H	-3.54290	3.44860	-0.36008
H	-4.54167	2.12545	-0.97874
H	0.36768	-2.75483	1.37269

4s_Renantiomer_conformer-081

C	-2.76831	0.57929	-0.26112

N	-3.11411	-1.26455	0.96537
H	-3.55950	-1.91984	1.59363
C	-1.90838	-1.45253	0.37425
C	-1.64773	-0.32504	-0.40454
C	0.41572	-2.20979	0.20223
C	-1.02745	-2.64924	0.49145
H	0.77721	-1.59884	1.03501
H	1.07190	-3.08552	0.14728
H	-1.10351	-3.10166	1.48712
H	-1.33830	-3.42077	-0.22911
C	-0.45952	-0.23417	-1.22492
O	-0.24765	0.68520	-2.02388
C	0.53980	-1.40674	-1.10530
C	1.98449	-0.96201	-1.38283
H	2.56580	-1.84398	-1.67711
H	1.97758	-0.27277	-2.24425
C	4.72648	0.80280	0.45485
C	4.09881	-0.20963	-0.51728
N	2.66858	-0.38181	-0.23014
C	2.09597	0.85420	0.28918
C	2.73692	1.15245	1.65511
O	4.10179	0.73303	1.72774
H	4.65240	1.82355	0.05022
H	5.78643	0.58357	0.60739
H	4.58800	-1.18780	-0.42986
H	4.26953	0.15036	-1.54962
H	2.22019	1.70243	-0.40916
H	1.02156	0.73339	0.44091
H	2.65787	2.22850	1.87533
H	2.21556	0.60043	2.44357
C	-2.89413	1.94016	-0.88225
H	-2.67011	1.87311	-1.95254
H	-3.92869	2.29223	-0.79377
C	-1.94728	2.97082	-0.24587
H	-2.14594	3.07258	0.82711
H	-0.90680	2.65807	-0.37565
H	-2.06879	3.95576	-0.71188
C	-3.65602	-0.02934	0.59901
C	-4.97189	0.41331	1.14885
H	-4.93786	0.53587	2.23917
H	-5.26000	1.37439	0.71628
H	-5.76916	-0.30708	0.92569
H	0.26530	-2.07455	-1.93818

4s_Renantiomer_conformer-082

C	-3.24751	0.18117	-0.05116
N	-2.64177	-1.96452	0.17272
H	-2.70463	-2.95714	0.35491
C	-1.53187	-1.32865	-0.27864
C	-1.86047	0.01984	-0.42938
C	0.56853	-1.03092	-1.52512
C	-0.20458	-1.94804	-0.56200
H	1.60658	-1.36511	-1.59950
H	0.11943	-1.10626	-2.52274
H	0.36370	-2.07554	0.37051
H	-0.32046	-2.94945	-0.99290
C	-0.88265	0.98455	-0.88611
O	-1.11559	2.18606	-1.06021
C	0.54120	0.44233	-1.08266
C	1.30238	0.68059	0.24169
H	1.29500	1.76478	0.46133
H	0.75448	0.19519	1.05809
C	4.84673	-0.04895	-0.77826
C	3.58664	0.82061	-0.63384
N	2.65080	0.13179	0.24642
C	3.19023	0.06164	1.61110
C	4.72342	-0.07577	1.57063
O	5.13729	-0.78523	0.41237
H	5.70619	0.58093	-1.05562
H	4.70069	-0.79927	-1.56135
H	3.13811	0.95524	-1.62132
H	3.83851	1.83066	-0.25861
H	2.93783	0.96312	2.20048
H	2.73344	-0.79780	2.11634
H	5.20027	0.91550	1.59997
H	5.08204	-0.64114	2.43450
C	-4.01426	1.47157	-0.01901
H	-3.83278	2.02415	-0.94738
H	-5.08970	1.26165	0.02224
C	-3.62189	2.36467	1.16916
H	-2.56047	2.62316	1.10990
H	-4.20209	3.29501	1.16939
H	-3.80020	1.84938	2.11985
C	-3.70155	-1.06581	0.31941
C	-5.03245	-1.53499	0.80791
H	-5.43673	-2.33792	0.17836
H	-4.97803	-1.92035	1.83426
H	-5.75073	-0.71162	0.80087
H	0.99158	1.06220	-1.86511

4s_Renantiomer_conformer-083

C	2.72889	-0.52515	0.35211
N	2.96362	1.69818	0.50776
H	3.35183	2.59938	0.75191
C	1.79146	1.51006	-0.14750
C	1.60250	0.13359	-0.27335
C	-0.53816	1.97042	-0.75647
C	0.87499	2.56536	-0.66514
H	-0.93470	1.83140	0.25398
H	-1.20961	2.67194	-1.26368
H	0.88328	3.45016	-0.01790
H	1.20657	2.90005	-1.65976
C	0.45000	-0.41087	-0.95854
O	0.28268	-1.61843	-1.16458
C	-0.56301	0.62286	-1.50035
C	-1.98221	0.04656	-1.62163
H	-2.52933	0.63897	-2.36457
H	-1.91057	-0.98251	-2.01296
C	-4.89453	-0.59941	0.64277
C	-4.17301	-0.20942	-0.65831
N	-2.76425	0.10631	-0.38988
C	-2.26112	-0.69722	0.71725
C	-3.00211	-0.27786	1.99818
O	-4.35605	0.10856	1.74930
H	-4.82252	-1.68415	0.81390
H	-5.95565	-0.34319	0.58631
H	-4.64232	0.67100	-1.11473
H	-4.27737	-1.04510	-1.37624
H	-2.36228	-1.78221	0.52952
H	-1.19606	-0.50698	0.86283
H	-2.96998	-1.09806	2.73229
H	-2.52096	0.59999	2.44099
C	2.96213	-2.00611	0.43304
H	3.68385	-2.22336	1.22961
H	2.02552	-2.50388	0.70705
C	3.46771	-2.60197	-0.89060
H	4.41331	-2.13680	-1.19172
H	3.63161	-3.68215	-0.79778
H	2.73151	-2.43312	-1.68188
C	3.55309	0.47199	0.82635
C	4.85322	0.42175	1.55905
H	5.66831	0.86840	0.97510
H	4.80303	0.95926	2.51453
H	5.12793	-0.61410	1.77243
H	-0.22298	0.80501	-2.53326

4s_Renantiomer_conformer-084

C	3.18424	0.06480	-0.37665
N	2.55146	-1.98563	0.26870
H	2.59191	-2.97720	0.46257
C	1.48200	-1.18990	0.51937
C	1.82964	0.10516	0.13050
C	-0.53269	-0.37507	1.67108
C	0.17208	-1.61423	1.09352
H	-1.56484	-0.62422	1.93034
H	-0.01900	-0.07268	2.59156
H	-0.45491	-2.07387	0.31618
H	0.31015	-2.37624	1.86964
C	0.88253	1.19633	0.22217
O	1.12777	2.36336	-0.10358
C	-0.52871	0.81168	0.69197
C	-1.37463	0.54204	-0.57348
H	-1.38073	1.45749	-1.19444
H	-0.88262	-0.23468	-1.17101
C	-4.87581	-0.59563	-1.28323
C	-3.35011	-0.51120	-1.47645
N	-2.72050	0.06908	-0.28334
C	-3.59707	1.06674	0.31751
C	-4.84053	0.34939	0.86992
O	-5.20225	-0.78957	0.08506
H	-5.28697	-1.44686	-1.83174
H	-5.36364	0.31388	-1.66494
H	-3.14711	0.08938	-2.38311
H	-2.92054	-1.50672	-1.64061
H	-3.08297	1.56498	1.14321
H	-3.87868	1.85657	-0.40459
H	-4.64018	-0.03586	1.87437
H	-5.68417	1.05444	0.92932
C	3.98105	1.23517	-0.87573
H	4.84272	0.87669	-1.45130
H	3.36433	1.82659	-1.56157
C	4.46889	2.14999	0.25911
H	3.61370	2.55439	0.80849
H	5.10154	1.59616	0.96212
H	5.05114	2.99037	-0.13700
C	3.60315	-1.24389	-0.27525
C	4.89688	-1.90391	-0.62241
H	4.75665	-2.72467	-1.33737
H	5.58185	-1.18186	-1.07334
H	5.39134	-2.32303	0.26347
H	-0.92522	1.69782	1.19880

4s_Renantiomer_conformer-085

C	-3.21865	0.35314	-0.08656
N	-2.89155	-1.78224	0.50854
H	-3.07968	-2.71116	0.86081
C	-1.71685	-1.38381	-0.04052
C	-1.87092	-0.05027	-0.42378
C	0.38365	-1.58123	-1.30788
C	-0.48549	-2.20975	-0.20505
H	1.36905	-2.05487	-1.31493
H	-0.08643	-1.77187	-2.28012
H	0.07668	-2.24762	0.73899
H	-0.73658	-3.24682	-0.45688
C	-0.78490	0.68617	-1.03492
O	-0.86358	1.86124	-1.41128
C	0.55254	-0.06223	-1.13188
C	1.37212	0.30279	0.12756
H	1.47658	1.40404	0.15539
H	0.80486	0.01150	1.01795
C	5.00638	-0.18173	-0.54231
C	3.55184	0.07230	-0.90922
N	2.66123	-0.37716	0.17200
C	3.32193	-0.30414	1.48848
C	4.37380	0.79865	1.55082
O	5.41840	0.58174	0.59411
H	5.65988	0.11099	-1.36941
H	5.15821	-1.25592	-0.35673
H	3.31257	-0.45779	-1.83902
H	3.42605	1.15205	-1.11138
H	2.56664	-0.13972	2.26243
H	3.80496	-1.26472	1.71126
H	3.90189	1.77661	1.37067
H	4.84678	0.83071	2.53835
C	-3.81169	1.71815	-0.28407
H	-3.57787	2.07157	-1.29434
H	-4.90426	1.66065	-0.21098
C	-3.28485	2.74602	0.73042
H	-2.20022	2.84879	0.63126
H	-3.73949	3.72988	0.56460
H	-3.51084	2.43273	1.75607
C	-3.82351	-0.74139	0.49182
C	-5.19477	-0.94224	1.04767
H	-5.70783	-1.78630	0.56938
H	-5.17274	-1.14228	2.12675
H	-5.80305	-0.04853	0.88882

H	1.06477	0.34757	-2.00887

4s_Renantiomer_conformer-086

C	3.15231	-0.25351	-0.04126
N	2.70928	1.86491	0.54005
H	2.84406	2.80221	0.89477
C	1.57126	1.41186	-0.04235
C	1.79734	0.08705	-0.41790
C	-0.50067	1.50269	-1.36866
C	0.31357	2.18381	-0.25361
H	-1.49522	1.95633	-1.41752
H	-0.01146	1.70480	-2.32906
H	-0.26985	2.22525	0.67694
H	0.52985	3.22385	-0.52501
C	0.76813	-0.69793	-1.06242
O	0.92102	-1.86256	-1.44671
C	-0.61044	-0.02676	-1.19152
C	-1.47415	-0.46263	0.02423
H	-1.00285	-1.32140	0.51374
H	-1.48089	0.34771	0.77134
C	-4.21706	1.11905	0.02491
C	-3.67020	0.10778	-0.98410
N	-2.81552	-0.91544	-0.36541
C	-3.54877	-1.52638	0.75279
C	-4.10634	-0.49631	1.73870
O	-4.93508	0.45288	1.06516
H	-4.92581	1.80268	-0.45217
H	-3.40214	1.71987	0.46029
H	-3.12577	0.61375	-1.78544
H	-4.52081	-0.41012	-1.44587
H	-4.38993	-2.09285	0.33184
H	-2.89199	-2.23353	1.27181
H	-4.73743	-0.97866	2.49134
H	-3.28934	0.02689	2.26073
C	3.81544	-1.58822	-0.22237
H	3.63463	-1.94607	-1.24193
H	4.90073	-1.47923	-0.11024
C	3.30297	-2.64642	0.76792
H	3.47474	-2.32825	1.80253
H	2.22934	-2.80289	0.62751
H	3.81209	-3.60541	0.61536
C	3.68805	0.86774	0.55343
C	5.03077	1.13134	1.15112
H	4.96520	1.33460	2.22779
H	5.68262	0.26486	1.01650

H	5.52060	1.99553	0.68466
H	-1.06749	-0.46780	-2.08342

4s_Renantiomer_conformer-087

C	2.67539	-0.69088	-0.21793
N	3.11207	1.22145	0.86396
H	3.60339	1.91090	1.41669
C	1.92472	1.42990	0.23770
C	1.60940	0.25733	-0.44827
C	0.08839	2.65527	-0.87893
C	1.11939	2.68662	0.26735
H	-0.66367	3.43653	-0.72104
H	0.59998	2.88935	-1.81976
H	0.61213	2.78750	1.23694
H	1.76357	3.56872	0.16974
C	0.40588	0.15645	-1.24927
O	0.17545	-0.75862	-2.04549
C	-0.60792	1.28582	-1.03202
C	-1.48669	0.98297	0.20131
H	-0.89042	1.13151	1.10745
H	-2.28945	1.74053	0.22853
C	-4.01243	-0.17482	1.67730
C	-2.59380	-0.73460	1.54106
N	-1.96673	-0.39892	0.25666
C	-2.87130	-0.79100	-0.83381
C	-4.27955	-0.22171	-0.66490
O	-4.83485	-0.61115	0.59402
H	-3.99481	0.92644	1.71175
H	-4.48902	-0.53690	2.59353
H	-2.64531	-1.82908	1.61353
H	-1.96055	-0.37227	2.35884
H	-2.43649	-0.50145	-1.79258
H	-2.93904	-1.88675	-0.82473
H	-4.27047	0.87757	-0.74306
H	-4.95416	-0.61151	-1.43337
C	2.71474	-2.10614	-0.71692
H	2.47330	-2.11936	-1.78503
H	3.72838	-2.51067	-0.61106
C	1.72015	-3.01591	0.02354
H	0.70005	-2.63805	-0.09420
H	1.75479	-4.03903	-0.36905
H	1.94941	-3.05202	1.09470
C	3.58713	-0.06605	0.60520
C	4.87297	-0.53585	1.20178
H	5.70758	0.12826	0.94334

H	4.82172	-0.58525	2.29717
H	5.11715	-1.53617	0.83599
H	-1.23913	1.31733	-1.92581

4s_Renantiomer_conformer-088

C	-3.06121	0.62990	0.23126
N	-3.57615	-1.39285	-0.58470
H	-4.12551	-2.16466	-0.93805
C	-2.24186	-1.43552	-0.34266
C	-1.87132	-0.19040	0.16274
C	-0.08284	-2.41014	0.31380
C	-1.32971	-2.59328	-0.56549
H	-0.34855	-2.59158	1.36361
H	0.67415	-3.15233	0.03903
H	-1.83001	-3.54253	-0.34034
H	-1.02856	-2.64014	-1.62305
C	-0.50036	0.10056	0.53056
O	-0.14954	1.16328	1.05391
C	0.52525	-0.99769	0.18353
C	1.84259	-0.86543	0.97344
H	1.67776	-0.23888	1.86649
H	2.14259	-1.85879	1.32509
C	4.10291	1.64163	-0.70976
C	2.75841	1.04296	-0.25534
N	2.94284	-0.35899	0.15302
C	4.24511	-0.52113	0.78745
C	5.33633	-0.31031	-0.27702
O	4.94659	0.64365	-1.26722
H	4.60923	2.13541	0.13380
H	3.94733	2.39411	-1.48728
H	2.02711	1.07745	-1.07048
H	2.35157	1.65598	0.56517
H	4.38648	0.17093	1.63992
H	4.32905	-1.53578	1.19009
H	6.27556	0.00325	0.20541
H	5.52160	-1.24190	-0.82052
C	-3.13122	2.05943	0.68500
H	-4.17392	2.33145	0.88773
H	-2.58421	2.16644	1.62808
C	-2.53790	3.03895	-0.34037
H	-1.48171	2.81022	-0.51082
H	-3.06615	2.96886	-1.29810
H	-2.61129	4.07304	0.01660
C	-4.09868	-0.14305	-0.24355
C	-5.55242	0.14628	-0.42482

H	-5.78937	1.14637	-0.05396
H	-5.84844	0.10457	-1.48099
H	-6.18081	-0.57039	0.11923
H	0.76433	-0.85029	-0.88114

4s_Renantiomer_conformer-089

C	3.02762	-0.65399	-0.25788
N	3.50506	1.51968	-0.52125
H	4.03425	2.36398	-0.69302
C	2.18760	1.48056	-0.19967
C	1.84164	0.14142	-0.02543
C	0.06004	2.21677	0.78579
C	1.26663	2.64493	-0.06359
H	0.37167	2.11185	1.83334
H	-0.70856	2.99624	0.75383
H	1.77625	3.50436	0.38771
H	0.91938	2.97108	-1.05567
C	0.48590	-0.25527	0.29876
O	0.15562	-1.42507	0.52038
C	-0.55346	0.88456	0.30550
C	-1.83387	0.52947	1.08815
H	-1.63417	-0.32362	1.75851
H	-2.10696	1.38140	1.72059
C	-4.19084	-1.42410	-1.11111
C	-2.82092	-0.95625	-0.58651
N	-2.97619	0.27140	0.21130
C	-4.24502	0.23943	0.92838
C	-5.38885	0.32497	-0.09731
O	-5.05922	-0.32083	-1.32925
H	-4.65145	-2.13354	-0.40686
H	-4.08206	-1.93603	-2.07080
H	-2.14147	-0.74876	-1.42067
H	-2.36533	-1.76852	0.00223
H	-4.34827	-0.66356	1.56018
H	-4.30319	1.10092	1.60152
H	-6.30696	-0.11056	0.32732
H	-5.58960	1.36944	-0.35496
C	3.13522	-2.14786	-0.15030
H	2.27718	-2.60958	-0.65110
H	4.03365	-2.49178	-0.67653
C	3.17726	-2.63800	1.30590
H	4.03269	-2.20683	1.83828
H	2.26157	-2.34326	1.82670
H	3.26148	-3.73028	1.35030
C	4.04018	0.23033	-0.56193

C	5.48316	0.01665	-0.88236
H	5.75357	0.45083	-1.85321
H	6.13981	0.46872	-0.12784
H	5.70915	-1.05172	-0.91984
H	-0.83971	1.02709	-0.74792

4s_Renantiomer_conformer-090

C	-3.38009	-0.31375	-0.20682
N	-3.17034	1.89952	0.07510
H	-3.41932	2.87579	0.16067
C	-1.90126	1.42051	0.06566
C	-1.98053	0.03827	-0.10235
C	0.50492	1.40613	-0.41809
C	-0.64518	2.21196	0.20387
H	0.38632	1.39222	-1.50982
H	1.46554	1.88337	-0.20755
H	-0.74032	3.19331	-0.27575
H	-0.43091	2.40167	1.26668
C	-0.79041	-0.78643	-0.14788
O	-0.80921	-2.00906	-0.32952
C	0.53839	-0.04476	0.09342
C	1.71053	-0.83814	-0.49730
H	1.68304	-1.86662	-0.09840
H	1.57059	-0.91387	-1.58272
C	4.86217	0.00054	1.35263
C	3.34146	-0.14140	1.17154
N	2.99896	-0.19767	-0.25692
C	4.08236	-0.80156	-1.02220
C	5.30897	0.12158	-0.95601
O	5.42348	0.78189	0.30813
H	5.34213	-0.98888	1.38992
H	5.08986	0.51408	2.29021
H	2.82655	0.71325	1.62536
H	3.00743	-1.04958	1.70823
H	4.33372	-1.81792	-0.66400
H	3.76994	-0.90594	-2.06616
H	6.22289	-0.45498	-1.16719
H	5.22312	0.91883	-1.70090
C	-3.94053	-1.69795	-0.36237
H	-4.97960	-1.63910	-0.70773
H	-3.37687	-2.23119	-1.13556
C	-3.88084	-2.51278	0.93967
H	-4.44609	-2.01638	1.73669
H	-4.30047	-3.51539	0.79521
H	-2.84284	-2.62027	1.26824

C	-4.09047	0.86129	-0.08961
C	-5.55680	1.14310	-0.11086
H	-5.90853	1.54884	0.84652
H	-5.82036	1.86958	-0.89013
H	-6.11654	0.22544	-0.30715
H	0.65005	-0.01145	1.18949

4s_Renantiomer_conformer-091

C	3.10922	0.44983	0.06502
N	2.92203	-1.70568	-0.51785
H	3.16652	-2.61965	-0.87467
C	1.73720	-1.39184	0.06314
C	1.80385	-0.04921	0.43985
C	-0.30619	-1.74079	1.39011
C	0.57519	-2.30547	0.26256
H	-1.25635	-2.28009	1.42357
H	0.20352	-1.89768	2.34833
H	-0.00766	-2.38761	-0.66627
H	0.90954	-3.32061	0.50677
C	0.68273	0.60758	1.07774
O	0.68083	1.78968	1.44087
C	-0.59015	-0.23892	1.22187
C	-1.48199	0.06680	-0.00603
H	-1.62430	1.15811	-0.05482
H	-0.94088	-0.21414	-0.91604
C	-4.07239	0.40014	-1.79374
C	-3.35022	-0.85864	-1.31010
N	-2.73299	-0.69208	0.01129
C	-3.73521	-0.17650	0.95487
C	-4.45448	1.06862	0.43125
O	-5.05305	0.81266	-0.84047
H	-4.60902	0.21074	-2.72854
H	-3.35299	1.21701	-1.96724
H	-2.58316	-1.16219	-2.03088
H	-4.08482	-1.67192	-1.23776
H	-4.47950	-0.96613	1.12028
H	-3.26031	0.03584	1.91765
H	-5.26669	1.36087	1.10372
H	-3.75600	1.91665	0.34852
C	3.60849	1.85438	0.24429
H	3.37381	2.19588	1.25850
H	4.70048	1.87362	0.14644
C	2.98805	2.83697	-0.76202
H	3.21328	2.53494	-1.79120
H	1.90128	2.86284	-0.63940

H	3.37502	3.85153	-0.61003
C	3.77610	-0.59997	-0.52797
C	5.14367	-0.70348	-1.11869
H	5.73164	-1.50055	-0.64597
H	5.10834	-0.91763	-2.19470
H	5.68648	0.23542	-0.98547
H	-1.09699	0.13342	2.11895

4s_Renantiomer_conformer-092

C	2.69163	0.68684	0.31115
N	3.27813	-1.13290	-0.85843
H	3.81971	-1.75532	-1.44300
C	2.05747	-1.42000	-0.34197
C	1.64784	-0.31156	0.39808
C	-0.19806	-2.37223	-0.31780
C	1.29432	-2.69100	-0.49207
H	-0.78007	-3.30043	-0.29270
H	-0.54756	-1.80564	-1.18614
H	1.48159	-3.15220	-1.46892
H	1.61680	-3.41983	0.26676
C	0.40803	-0.32088	1.14456
O	0.08236	0.56885	1.93898
C	-0.49265	-1.56182	0.95758
C	-1.99022	-1.22077	1.08479
H	-2.52648	-2.14947	1.30473
H	-2.11709	-0.54658	1.94856
C	-2.93868	1.35365	-1.47604
C	-2.02484	0.65503	-0.48178
N	-2.56380	-0.66878	-0.14155
C	-4.03833	-0.67957	-0.16572
C	-4.63378	0.70230	0.08470
O	-4.22623	1.63869	-0.91880
H	-3.04671	0.73272	-2.37848
H	-2.50581	2.31276	-1.77674
H	-1.90999	1.29395	0.40937
H	-1.02471	0.54756	-0.92051
H	-4.39141	-1.03990	-1.14207
H	-4.41191	-1.37996	0.58729
H	-5.72799	0.66510	0.05622
H	-4.33387	1.06387	1.08025
C	2.65347	2.06175	0.91227
H	2.37099	1.98845	1.96810
H	3.65444	2.50792	0.87914
C	1.65600	2.99004	0.20008
H	0.64370	2.58017	0.26822

H	1.65267	3.98707	0.65614
H	1.91307	3.09888	-0.85972
C	3.68362	0.15018	-0.48049
C	4.98678	0.70383	-0.95501
H	5.82895	0.05838	-0.67486
H	5.00955	0.81519	-2.04685
H	5.16213	1.68966	-0.51770
H	-0.25494	-2.19572	1.82731

4s_Renantiomer_conformer-093

C	3.04297	0.64433	-0.26648
N	3.59773	-1.36056	0.56741
H	4.16288	-2.12075	0.92122
C	2.25905	-1.42040	0.35806
C	1.86351	-0.18702	-0.15801
C	0.09977	-2.43098	-0.23490
C	1.36309	-2.58078	0.62637
H	0.35170	-2.63174	-1.28457
H	-0.64329	-3.17668	0.06702
H	1.86832	-3.53051	0.41503
H	1.08214	-2.60415	1.69020
C	0.48116	0.08618	-0.49056
O	0.09908	1.14897	-0.99360
C	-0.52562	-1.02443	-0.12859
C	-1.83874	-0.92414	-0.92863
H	-1.67607	-0.33044	-1.84448
H	-2.13285	-1.93021	-1.24979
C	-3.88310	1.30676	1.30803
C	-2.84543	1.00905	0.21468
N	-2.95393	-0.40185	-0.14069
C	-4.23463	-0.64763	-0.81439
C	-5.33267	0.25220	-0.21504
O	-5.07296	0.52684	1.15368
H	-4.13066	2.37997	1.30910
H	-3.48357	1.04354	2.29250
H	-1.84416	1.22266	0.59202
H	-2.99001	1.66774	-0.66217
H	-4.17555	-0.44557	-1.90123
H	-4.48866	-1.70808	-0.69434
H	-5.41215	1.19191	-0.78233
H	-6.30564	-0.24399	-0.25960
C	3.08769	2.06760	-0.74232
H	4.12085	2.34324	-0.98505
H	2.50757	2.15792	-1.66714
C	2.52391	3.05722	0.29002

H	3.08643	3.00414	1.22913
H	2.57680	4.08672	-0.08348
H	1.47623	2.82327	0.50102
C	4.09930	-0.11103	0.19438
C	5.55411	0.19497	0.33484
H	5.87674	0.17139	1.38373
H	6.17578	-0.52349	-0.21452
H	5.77152	1.19184	-0.05612
H	-0.77623	-0.85984	0.93145

4s_Renantiomer_conformer-094

C	2.65617	-0.68535	0.18789
N	3.13037	1.41621	0.80593
H	3.61925	2.20001	1.21732
C	1.93277	1.49314	0.17384
C	1.59530	0.20323	-0.23526
C	-0.34570	2.30489	-0.21970
C	1.12554	2.72117	-0.07389
H	-0.73058	1.99602	0.75690
H	-0.94843	3.16289	-0.53796
H	1.24485	3.44558	0.74042
H	1.46879	3.22125	-0.99216
C	0.38429	-0.05841	-0.98342
O	0.10723	-1.16060	-1.47054
C	-0.54359	1.15347	-1.22198
C	-2.02510	0.74971	-1.34635
H	-2.54591	1.55425	-1.87532
H	-2.08454	-0.15723	-1.97150
C	-4.73784	-0.83892	-0.01060
C	-4.16059	0.56713	-0.13891
N	-2.68856	0.58034	-0.05463
C	-2.17736	-0.56799	0.70611
C	-3.16679	-0.95919	1.79262
O	-4.40599	-1.42885	1.25120
H	-4.36562	-1.47206	-0.83059
H	-5.83118	-0.81767	-0.07196
H	-4.58417	1.19247	0.65939
H	-4.47668	1.00629	-1.08986
H	-1.99222	-1.44060	0.05842
H	-1.21504	-0.30949	1.16629
H	-2.75942	-1.77385	2.39919
H	-3.34585	-0.09958	2.45648
C	2.73098	-2.16525	-0.05524
H	3.43312	-2.62064	0.65355
H	1.74975	-2.61261	0.13777

C	3.15679	-2.51306	-1.49055
H	4.14279	-2.09190	-1.71795
H	3.20726	-3.59914	-1.63277
H	2.43313	-2.10638	-2.20279
C	3.59033	0.09711	0.83130
C	4.88859	-0.24255	1.48632
H	5.73770	0.23783	0.98318
H	4.90969	0.07389	2.53698
H	5.05549	-1.32206	1.45906
H	-0.24743	1.51758	-2.21956

4s_Renantiomer_conformer-095

C	-3.23574	0.33575	0.00076
N	-2.85872	-1.86287	0.21140
H	-3.02226	-2.84268	0.40042
C	-1.70346	-1.35119	-0.28235
C	-1.88857	0.02521	-0.42565
C	0.37473	-1.28278	-1.59953
C	-0.46304	-2.11200	-0.61061
H	1.36986	-1.72328	-1.70343
H	-0.10970	-1.31266	-2.58291
H	0.11600	-2.30799	0.30339
H	-0.70351	-3.09246	-1.03828
C	-0.82752	0.87899	-0.91647
O	-0.93267	2.10038	-1.07800
C	0.51979	0.18607	-1.16606
C	1.36445	0.34406	0.11927
H	1.41678	1.41799	0.37584
H	0.84263	-0.14762	0.94811
C	4.72386	1.16679	-0.03703
C	3.62724	0.48148	-0.84666
N	2.68391	-0.26532	0.01121
C	3.25402	-0.56522	1.32894
C	4.73296	-0.89010	1.18913
O	5.49270	0.23033	0.72197
H	4.27875	1.92024	0.63058
H	5.42516	1.68229	-0.70095
H	4.09149	-0.20003	-1.57176
H	3.08656	1.23943	-1.42216
H	3.14064	0.27417	2.03965
H	2.72771	-1.42457	1.76194
H	5.15247	-1.18024	2.15768
H	4.85385	-1.73720	0.49690
C	-3.85795	1.70107	0.05215
H	-3.64390	2.23116	-0.88233

H	-4.94819	1.60805	0.12390
C	-3.33853	2.54546	1.22700
H	-3.81242	3.53411	1.23998
H	-3.54713	2.05289	2.18355
H	-2.25706	2.68508	1.13892
C	-3.80873	-0.85448	0.39222
C	-5.16379	-1.17590	0.93115
H	-5.79176	-0.28170	0.93633
H	-5.67110	-1.93878	0.32697
H	-5.11467	-1.55377	1.96060
H	1.00498	0.74986	-1.97063

4s_Renantiomer_conformer-096

C	-3.01183	-0.67955	0.24536
N	-3.53227	1.48689	0.48683
H	-4.07937	2.32222	0.64618
C	-2.20968	1.47008	0.18678
C	-1.83676	0.13623	0.02620
C	-0.08282	2.24061	-0.77155
C	-1.30559	2.64867	0.06406
H	-0.38292	2.12892	-1.82180
H	0.67147	3.03370	-0.73389
H	-1.82274	3.50092	-0.39227
H	-0.97607	2.97835	1.06104
C	-0.46901	-0.23728	-0.26744
O	-0.10631	-1.40468	-0.45205
C	0.55026	0.91967	-0.28661
C	1.82743	0.57654	-1.07774
H	1.62975	-0.26477	-1.76379
H	2.09865	1.43763	-1.69962
C	3.98551	-0.89216	1.63193
C	2.89964	-0.93027	0.54551
N	2.98033	0.30960	-0.21928
C	4.22902	0.33760	-0.99040
C	5.35915	-0.34878	-0.19905
O	5.16199	-0.20147	1.19947
H	4.24083	-1.91694	1.94440
H	3.62648	-0.34322	2.50821
H	1.91726	-1.01689	1.01266
H	3.01264	-1.82146	-0.09998
H	4.12515	-0.17822	-1.96438
H	4.47926	1.38453	-1.20208
H	5.42109	-1.41528	-0.46338
H	6.32529	0.10617	-0.43246
C	-3.08915	-2.17559	0.14537

H	-2.23571	-2.61767	0.67123
H	-3.99456	-2.53269	0.65048
C	-3.08370	-2.67564	-1.30807
H	-3.14513	-3.76967	-1.34710
H	-3.93334	-2.26486	-1.86534
H	-2.16068	-2.36598	-1.80688
C	-4.04487	0.18785	0.52673
C	-5.48873	-0.04965	0.82450
H	-5.78314	0.38676	1.78741
H	-6.14056	0.38497	0.05562
H	-5.69567	-1.12171	0.86633
H	0.84698	1.06892	0.76364

4s_Renantiomer_conformer-097

C	-2.41600	-0.49765	-0.28829
N	-2.05833	1.42093	-1.38857
H	-2.15547	2.15612	-2.07585
C	-1.18085	1.43846	-0.35461
C	-1.36821	0.25783	0.36603
C	0.27511	2.36718	1.39817
C	-0.18204	2.50536	-0.06506
H	1.15534	2.99691	1.57314
H	-0.52333	2.75144	2.04506
H	0.67460	2.39680	-0.73875
H	-0.61126	3.50068	-0.23616
C	-0.53367	-0.07371	1.49699
O	-0.63330	-1.12091	2.15149
C	0.58554	0.92083	1.83182
C	1.93832	0.38572	1.30066
H	2.71839	1.08428	1.62197
H	2.14470	-0.56874	1.80671
C	4.38874	-0.19471	-0.66065
C	3.20947	0.77018	-0.78841
N	1.98771	0.25895	-0.16001
C	1.70298	-1.09402	-0.65771
C	2.91210	-2.02349	-0.53970
O	4.05056	-1.47149	-1.20357
H	4.68156	-0.30383	0.39706
H	5.25674	0.16204	-1.22402
H	3.00173	0.92318	-1.85620
H	3.45874	1.74470	-0.35574
H	0.85142	-1.51140	-0.11557
H	1.41789	-1.01553	-1.71495
H	3.15534	-2.20590	0.51994
H	2.71322	-2.98874	-1.01555

C	-2.96440	-1.82296	0.15477
H	-3.53393	-2.27905	-0.66380
H	-2.13348	-2.50042	0.38124
C	-3.85811	-1.71200	1.40020
H	-4.71007	-1.04873	1.21178
H	-4.24699	-2.69432	1.69357
H	-3.28321	-1.30689	2.23815
C	-2.82412	0.25142	-1.36951
C	-3.86906	0.01266	-2.40916
H	-4.33393	-0.96522	-2.26223
H	-4.66440	0.76827	-2.36940
H	-3.44805	0.03484	-3.42250
H	0.67605	0.90282	2.92550

4s_Renantiomer_conformer-098

C	2.73319	0.61491	-0.22788
N	3.25581	-1.38907	0.62917
H	3.80513	-2.14317	1.01909
C	1.94343	-1.47523	0.29636
C	1.56876	-0.24370	-0.23824
C	-0.13813	-2.51303	-0.50032
C	1.05525	-2.66166	0.45685
H	0.19969	-2.68397	-1.53112
H	-0.88724	-3.28008	-0.27592
H	1.59855	-3.59400	0.26260
H	0.68828	-2.72216	1.49275
C	0.21883	-0.00174	-0.70496
O	-0.12023	1.03743	-1.28344
C	-0.79625	-1.11967	-0.40632
C	-2.06723	-1.03353	-1.29790
H	-1.77496	-0.62937	-2.27292
H	-2.41133	-2.06158	-1.47002
C	-3.59854	-0.14622	1.62950
C	-3.97328	-0.80563	0.29631
N	-3.23128	-0.28608	-0.84782
C	-3.18865	1.17263	-0.85273
C	-2.80587	1.76206	0.50559
O	-3.67525	1.27464	1.53253
H	-2.58593	-0.44111	1.94689
H	-4.30180	-0.44115	2.41533
H	-5.04554	-0.61785	0.12882
H	-3.83340	-1.89044	0.35920
H	-2.48324	1.50770	-1.61399
H	-4.19214	1.54737	-1.10943
H	-1.76218	1.51642	0.74955

H	-2.91537	2.85152	0.50471
C	2.78526	2.04721	-0.67530
H	3.82950	2.35611	-0.80330
H	2.30274	2.13819	-1.65436
C	2.08727	3.00256	0.30627
H	2.55004	2.94920	1.29832
H	2.14833	4.03937	-0.04501
H	1.03028	2.73717	0.40435
C	3.75976	-0.12296	0.32071
C	5.18684	0.21468	0.60237
H	5.41493	1.22295	0.24850
H	5.41015	0.18063	1.67652
H	5.87535	-0.47911	0.10323
H	-1.09152	-0.97727	0.64219

4s_Renantiomer_conformer-099

C	3.12834	0.32916	-0.11518
N	2.92751	-1.85965	0.32191
H	3.18128	-2.81386	0.53960
C	1.67005	-1.43600	0.04162
C	1.74252	-0.07100	-0.23325
C	-0.60683	-1.55510	-0.85910
C	0.43091	-2.26353	0.02404
H	-0.29092	-1.62038	-1.90858
H	-1.56539	-2.07646	-0.78552
H	0.63404	-3.27520	-0.34646
H	0.03268	-2.37602	1.04393
C	0.55966	0.70072	-0.55309
O	0.58528	1.91028	-0.80583
C	-0.78174	-0.06936	-0.49259
C	-1.86864	0.63736	-1.34559
H	-1.55903	1.67953	-1.45950
H	-1.85614	0.19020	-2.34933
C	-4.00115	-1.24789	0.54590
C	-4.00034	-0.59627	-0.84059
N	-3.25099	0.65389	-0.89157
C	-3.59234	1.53647	0.22016
C	-3.62317	0.83056	1.58028
O	-4.46290	-0.32324	1.53165
H	-2.99512	-1.60370	0.81764
H	-4.68847	-2.09932	0.57585
H	-5.04910	-0.38417	-1.10099
H	-3.60780	-1.28752	-1.59342
H	-2.88823	2.37469	0.24569
H	-4.59760	1.94879	0.04109

H	-2.61016	0.53753	1.89760
H	-4.04615	1.48724	2.34718
C	3.67339	1.71721	-0.29026
H	4.75731	1.67055	-0.44980
H	3.23750	2.16619	-1.18929
C	3.37330	2.62941	0.91008
H	2.29223	2.72384	1.04896
H	3.80565	2.21886	1.82970
H	3.78810	3.63235	0.75439
C	3.83527	-0.80162	0.23225
C	5.28756	-1.02374	0.49965
H	5.85154	-0.10587	0.31691
H	5.46995	-1.32455	1.53944
H	5.70460	-1.80770	-0.14528
H	-1.07614	-0.00933	0.56527

4s_Renantiomer_conformer-100

C	-3.37978	-0.32734	-0.19413
N	-3.17212	1.89747	-0.02292
H	-3.42198	2.87659	0.01309
C	-1.90300	1.41924	0.00076
C	-1.98094	0.03017	-0.09750
C	0.50674	1.38227	-0.46441
C	-0.64811	2.21725	0.10818
H	0.39663	1.31471	-1.55515
H	1.46615	1.86784	-0.26921
H	-0.74000	3.17321	-0.42081
H	-0.44189	2.46029	1.16169
C	-0.79074	-0.79544	-0.09029
O	-0.80861	-2.02635	-0.20303
C	0.53749	-0.04167	0.11767
C	1.70972	-0.86406	-0.43190
H	1.68645	-1.87528	0.00771
H	1.56370	-0.99218	-1.51195
C	4.65752	0.78475	1.22784
C	3.46473	-0.18150	1.13733
N	3.00681	-0.22049	-0.24605
C	4.02863	-0.84373	-1.09722
C	5.43814	-0.49737	-0.58205
O	5.45093	0.77981	0.03864
H	5.27881	0.53174	2.10112
H	4.30298	1.81373	1.34227
H	2.66584	0.18820	1.78537
H	3.73581	-1.18755	1.51103
H	3.92853	-1.94554	-1.11979

H	3.88741	-0.48257	-2.12304
H	5.79085	-1.26538	0.12280
H	6.15135	-0.45529	-1.40921
C	-3.93976	-1.71751	-0.28588
H	-4.97549	-1.67521	-0.64354
H	-3.36955	-2.28852	-1.02663
C	-3.89332	-2.46762	1.05496
H	-4.31388	-3.47535	0.95596
H	-2.85852	-2.56078	1.39756
H	-4.46457	-1.93191	1.82170
C	-4.09097	0.85189	-0.14145
C	-5.55711	1.13189	-0.18763
H	-5.91564	1.58511	0.74560
H	-5.81486	1.81834	-1.00425
H	-6.11559	0.20541	-0.34159
H	0.64079	0.04652	1.21157

4s_Renantiomer_conformer-101

C	2.88642	0.62860	0.20395
N	3.33018	-1.35143	-0.74780
H	3.82614	-2.07742	-1.24717
C	2.07201	-1.46829	-0.25620
C	1.74794	-0.25356	0.34834
C	-0.26360	-2.21205	-0.17286
C	1.19480	-2.67190	-0.31744
H	-0.57062	-1.68786	-1.08326
H	-0.92330	-3.08069	-0.07046
H	1.33998	-3.22037	-1.25571
H	1.45085	-3.36792	0.49590
C	0.48373	-0.06006	1.02389
O	0.17903	0.98329	1.61568
C	-0.46474	-1.27803	1.03384
C	-1.94410	-0.90677	1.24401
H	-2.44554	-1.78796	1.65921
H	-2.00742	-0.11077	2.00459
C	-4.84850	-0.05770	-0.95872
C	-4.10837	-0.73640	0.18335
N	-2.66166	-0.56520	0.01694
C	-2.32582	0.74964	-0.56812
C	-3.43414	1.77632	-0.35853
O	-4.67050	1.36234	-0.94885
H	-5.92425	-0.24398	-0.88002
H	-4.50008	-0.47417	-1.91639
H	-4.34912	-1.80699	0.19441
H	-4.47556	-0.31518	1.13821

H	-1.40867	1.13179	-0.11917
H	-2.14403	0.63913	-1.64615
H	-3.57404	1.95744	0.71827
H	-3.16704	2.73044	-0.82498
C	2.97458	2.05201	0.67337
H	2.61697	2.11506	1.70692
H	4.02266	2.37420	0.67803
C	2.14812	3.01689	-0.19191
H	1.09075	2.73748	-0.16267
H	2.24015	4.04701	0.17211
H	2.48364	2.99101	-1.23493
C	3.84721	-0.08018	-0.48357
C	5.21642	0.29262	-0.94849
H	5.29340	0.27710	-2.04338
H	5.46922	1.30127	-0.61302
H	5.97984	-0.39193	-0.55739
H	-0.16344	-1.83221	1.93892

4s_Renantiomer_conformer-102

C	-2.27092	-0.98729	0.37480
N	-3.23445	0.57181	-0.91331
H	-3.90012	1.01816	-1.52956
C	-2.15692	1.18521	-0.35753
C	-1.52351	0.24620	0.45537
C	-0.70647	3.00237	0.49967
C	-1.73459	2.60022	-0.57794
H	-0.19484	3.92241	0.19546
H	-1.23980	3.22647	1.43081
H	-1.30074	2.70981	-1.58168
H	-2.59577	3.27847	-0.54611
C	-0.33958	0.58979	1.21632
O	0.12723	-0.10074	2.12733
C	0.33393	1.89944	0.79117
C	1.22619	1.64310	-0.44331
H	0.59058	1.54290	-1.32882
H	1.86390	2.53446	-0.60530
C	3.75860	-0.88054	0.75606
C	3.01559	0.44924	0.70914
N	2.01282	0.41800	-0.35627
C	2.64792	0.09916	-1.63387
C	3.38885	-1.22814	-1.52657
O	4.36943	-1.18948	-0.49411
H	3.05388	-1.68018	1.03484
H	4.56288	-0.84614	1.49623
H	3.74119	1.27159	0.55430

H	2.51400	0.60393	1.66700
H	1.87522	0.02582	-2.40800
H	3.36154	0.88765	-1.94357
H	2.66607	-2.03558	-1.32691
H	3.92022	-1.45124	-2.45603
C	-1.88768	-2.28312	1.02828
H	-1.67525	-2.10590	2.08804
H	-2.72600	-2.98830	0.98192
C	-0.64650	-2.91592	0.37537
H	0.20054	-2.22416	0.41962
H	-0.36178	-3.84341	0.88608
H	-0.83966	-3.15039	-0.67784
C	-3.32094	-0.75560	-0.48715
C	-4.41432	-1.64351	-0.98323
H	-5.40632	-1.22574	-0.76896
H	-4.35320	-1.80034	-2.06793
H	-4.35419	-2.62289	-0.50233
H	0.95779	2.22201	1.63053

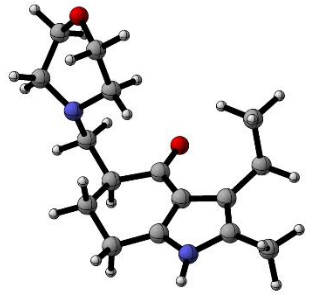

4s_Senantiomer_conformer-001

C	-2.69152	0.68690	0.31116
N	-3.27811	-1.13279	-0.85846
H	-3.81972	-1.75518	-1.44303
C	-2.05749	-1.41998	-0.34196
C	-1.64780	-0.31157	0.39812
C	0.19797	-2.37235	-0.31778
C	-1.29443	-2.69104	-0.49201
H	0.54746	-1.80579	-1.18614
H	0.77993	-3.30058	-0.29268
H	-1.61695	-3.41982	0.26686
H	-1.48174	-3.15227	-1.46883
C	-0.40801	-0.32097	1.14462
O	-0.08233	0.56873	1.93906
C	0.49266	-1.56192	0.95757
H	0.25502	-2.19580	1.82734
C	1.99024	-1.22086	1.08466
H	2.11716	-0.54665	1.94840
H	2.52652	-2.14956	1.30457

C	2.93851	1.35386	-1.47597
C	2.02466	0.65494	-0.48190
N	2.56371	-0.66888	-0.14173
C	4.03823	-0.67959	-0.16593
C	4.63351	0.70229	0.08479
O	4.22609	1.63873	-0.91870
H	2.50559	2.31304	-1.77637
H	3.04653	0.73320	-2.37859
H	1.02460	0.54743	-0.92075
H	1.90963	1.29370	0.40933
H	4.41182	-1.38011	0.58695
H	4.39135	-1.03970	-1.14235
H	4.33334	1.06369	1.08032
H	5.72774	0.66523	0.05654
C	-2.65327	2.06181	0.91229
H	-3.65416	2.50811	0.87898
H	-2.37096	1.98847	1.96816
C	-1.65552	2.98994	0.20027
H	-1.91239	3.09884	-0.85957
H	-0.64330	2.57990	0.26856
H	-1.65209	3.98696	0.65635
C	-3.68351	0.15032	-0.48053
C	-4.98665	0.70406	-0.95506
H	-5.00971	0.81454	-2.04698
H	-5.82897	0.05914	-0.67413
H	-5.16153	1.69031	-0.51849

4s_Senantiomer_conformer-002

C	-2.65617	-0.68535	0.18789
N	-3.13037	1.41621	0.80593
H	-3.61925	2.20000	1.21732
C	-1.93277	1.49314	0.17384
C	-1.59530	0.20323	-0.23526
C	0.34570	2.30489	-0.21970
C	-1.12554	2.72117	-0.07389
H	0.73058	1.99603	0.75690
H	0.94843	3.16289	-0.53796
H	-1.46879	3.22125	-0.99216
H	-1.24485	3.44557	0.74043
C	-0.38429	-0.05840	-0.98342
O	-0.10723	-1.16060	-1.47054
C	0.54359	1.15348	-1.22198
H	0.24743	1.51759	-2.21956
C	2.02510	0.74971	-1.34634
H	2.08454	-0.15722	-1.97150

H	2.54591	1.55425	-1.87532
C	3.16679	-0.95919	1.79262
C	2.17736	-0.56799	0.70611
N	2.68856	0.58034	-0.05463
C	4.16059	0.56713	-0.13891
C	4.73784	-0.83892	-0.01060
O	4.40599	-1.42885	1.25120
H	2.75942	-1.77386	2.39918
H	3.34585	-0.09959	2.45648
H	1.21504	-0.30949	1.16629
H	1.99222	-1.44060	0.05842
H	4.47668	1.00630	-1.08985
H	4.58416	1.19247	0.65940
H	4.36562	-1.47205	-0.83059
H	5.83118	-0.81766	-0.07196
C	-2.73098	-2.16526	-0.05524
H	-1.74974	-2.61261	0.13777
H	-3.43312	-2.62064	0.65354
C	-3.15678	-2.51306	-1.49055
H	-2.43312	-2.10637	-2.20279
H	-4.14278	-2.09190	-1.71795
H	-3.20725	-3.59914	-1.63277
C	-3.59033	0.09711	0.83130
C	-4.88859	-0.24255	1.48631
H	-4.90970	0.07391	2.53697
H	-5.73771	0.23781	0.98317
H	-5.05548	-1.32207	1.45907

4s_Senantiomer_conformer-003

C	3.23575	0.33572	0.00076
N	2.85867	-1.86289	0.21136
H	3.02219	-2.84272	0.40035
C	1.70342	-1.35118	-0.28238
C	1.88857	0.02522	-0.42565
C	-0.37476	-1.28270	-1.59956
C	0.46299	-2.11195	-0.61066
H	-1.36990	-1.72317	-1.70347
H	0.10967	-1.31256	-2.58294
H	0.70344	-3.09240	-1.03835
H	-0.11605	-2.30795	0.30334
C	0.82753	0.87904	-0.91645
O	0.93271	2.10043	-1.07795
C	-0.51979	0.18615	-1.16605
H	-1.00496	0.74996	-1.97061
C	-1.36445	0.34413	0.11928

H	-1.41677	1.41805	0.37586
H	-0.84263	-0.14756	0.94812
C	-4.72393	1.16675	-0.03698
C	-3.62725	0.48156	-0.84663
N	-2.68391	-0.26525	0.01122
C	-3.25401	-0.56517	1.32895
C	-4.73292	-0.89017	1.18911
O	-5.49274	0.23019	0.72192
H	-5.42525	1.68226	-0.70088
H	-4.27888	1.92018	0.63070
H	-3.08660	1.23957	-1.42206
H	-4.09145	-0.19993	-1.57179
H	-2.72764	-1.42448	1.76196
H	-3.14070	0.27424	2.03965
H	-4.85372	-1.73729	0.49689
H	-5.15242	-1.18035	2.15765
C	3.85800	1.70103	0.05216
H	4.94824	1.60797	0.12386
H	3.64392	2.23114	-0.88229
C	3.33866	2.54540	1.22706
H	3.81258	3.53404	1.24005
H	2.25719	2.68505	1.13904
H	3.54730	2.05280	2.18359
C	3.80871	-0.85453	0.39220
C	5.16377	-1.17600	0.93111
H	5.67110	-1.93879	0.32684
H	5.79172	-0.28179	0.93641
H	5.11463	-1.55400	1.96051

4s_Senantiomer_conformer-004

C	3.01182	-0.67956	0.24535
N	3.53228	1.48687	0.48684
H	4.07938	2.32221	0.64619
C	2.20969	1.47008	0.18679
C	1.83676	0.13623	0.02620
C	0.08283	2.24062	-0.77155
C	1.30561	2.64867	0.06407
H	0.38293	2.12894	-1.82180
H	-0.67145	3.03373	-0.73388
H	0.97608	2.97836	1.06105
H	1.82277	3.50092	-0.39226
C	0.46900	-0.23727	-0.26746
O	0.10630	-1.40467	-0.45210
C	-0.55026	0.91969	-0.28661
H	-0.84697	1.06893	0.76364

C	-1.82743	0.57658	-1.07774
H	-2.09866	1.43768	-1.69960
H	-1.62976	-0.26472	-1.76381
C	-3.98548	-0.89216	1.63193
C	-2.89962	-0.93026	0.54551
N	-2.98032	0.30961	-0.21927
C	-4.22902	0.33761	-0.99038
C	-5.35914	-0.34879	-0.19904
O	-5.16198	-0.20150	1.19949
H	-3.62644	-0.34319	2.50820
H	-4.24076	-1.91694	1.94444
H	-3.01263	-1.82145	-0.10000
H	-1.91723	-1.01689	1.01264
H	-4.47928	1.38454	-1.20204
H	-4.12515	-0.17820	-1.96437
H	-6.32528	0.10615	-0.43244
H	-5.42107	-1.41529	-0.46339
C	3.08913	-2.17560	0.14536
H	3.99454	-2.53270	0.65045
H	2.23570	-2.61767	0.67125
C	3.08364	-2.67564	-1.30808
H	3.93327	-2.26487	-1.86537
H	2.16061	-2.36598	-1.80686
H	3.14505	-3.76967	-1.34711
C	4.04487	0.18783	0.52674
C	5.48872	-0.04968	0.82451
H	6.14056	0.38495	0.05564
H	5.78313	0.38671	1.78743
H	5.69566	-1.12174	0.86632

4s_Senantiomer_conformer-005

C	-3.15865	0.19751	-0.44973
N	-2.75632	-1.93166	0.12100
H	-2.89760	-2.92386	0.25557
C	-1.64319	-1.24977	0.49019
C	-1.84764	0.08934	0.15268
C	0.35665	-0.68371	1.80981
C	-0.41898	-1.82123	1.12289
H	1.34793	-1.03688	2.10602
H	-0.18086	-0.38949	2.71922
H	-0.67753	-2.60223	1.84758
H	0.21200	-2.30342	0.36231
C	-0.82064	1.08545	0.37280
O	-0.94378	2.28555	0.10206
C	0.51607	0.55321	0.90902

H	0.95156	1.36903	1.49688
C	1.43213	0.28498	-0.30687
H	0.96366	-0.47527	-0.94205
H	1.49109	1.20670	-0.91413
C	4.86882	-1.13997	-0.71675
C	3.39457	-0.93566	-1.02842
N	2.74817	-0.21369	0.07305
C	3.63532	0.80976	0.66343
C	4.76112	1.21282	-0.28408
O	5.58318	0.09875	-0.64164
H	5.34499	-1.73667	-1.50117
H	4.96628	-1.68575	0.23408
H	2.90638	-1.90945	-1.15683
H	3.30740	-0.40110	-1.99232
H	3.05426	1.70032	0.92257
H	4.07271	0.43063	1.59652
H	4.33904	1.67678	-1.18860
H	5.41853	1.94751	0.19182
C	-3.80915	1.45895	-0.93899
H	-3.08746	2.02929	-1.53429
H	-4.64504	1.20983	-1.60350
C	-4.31425	2.35096	0.20635
H	-5.04776	1.81800	0.82203
H	-4.78915	3.25909	-0.18346
H	-3.47784	2.64911	0.84543
C	-3.69602	-1.07117	-0.45234
C	-5.01240	-1.59242	-0.92708
H	-4.89044	-2.38778	-1.67336
H	-5.60843	-2.00585	-0.10318
H	-5.59442	-0.78979	-1.38637

4s_Senantiomer_conformer-006

C	2.41592	-0.49777	-0.28812
N	2.05824	1.42039	-1.38913
H	2.15538	2.15530	-2.07670
C	1.18084	1.43836	-0.35511
C	1.36822	0.25803	0.36598
C	-0.27504	2.36779	1.39737
C	0.18209	2.50543	-0.06592
H	-1.15524	2.99761	1.57212
H	0.52343	2.75227	2.04410
H	0.61137	3.50066	-0.23739
H	-0.67456	2.39669	-0.73958
C	0.53372	-0.07306	1.49711
O	0.63340	-1.11998	2.15205

C	-0.58549	0.92161	1.83155
H	-0.67602	0.90401	2.92523
C	-1.93827	0.38631	1.30056
H	-2.14475	-0.56789	1.80708
H	-2.71833	1.08507	1.62151
C	-2.91204	-2.02374	-0.53886
C	-1.70289	-1.09434	-0.65716
N	-1.98764	0.25884	-0.16004
C	-3.20935	0.76983	-0.78873
C	-4.38864	-0.19500	-0.66074
O	-4.05040	-1.47201	-1.20311
H	-3.15539	-2.20564	0.52084
H	-2.71312	-2.98921	-1.01423
H	-1.41774	-1.01629	-1.71442
H	-0.85135	-1.51149	-0.11482
H	-3.45866	1.74451	-0.35645
H	-3.00151	0.92246	-1.85657
H	-4.68160	-0.30370	0.39697
H	-5.25656	0.16154	-1.22437
C	2.96421	-1.82294	0.15545
H	2.13325	-2.50020	0.38235
H	3.53359	-2.27949	-0.66297
C	3.85808	-1.71152	1.40072
H	4.71005	-1.04836	1.21191
H	4.24694	-2.69373	1.69445
H	3.28330	-1.30603	2.23857
C	2.82397	0.25084	-1.36968
C	3.86871	0.01163	-2.40942
H	4.66371	0.76765	-2.37058
H	4.33407	-0.96589	-2.26173
H	3.44739	0.03270	-3.42265

4s_Senantiomer_conformer-007

C	-2.73320	0.61489	-0.22787
N	-3.25577	-1.38910	0.62919
H	-3.80507	-2.14322	1.01910
C	-1.94340	-1.47524	0.29635
C	-1.56876	-0.24370	-0.23825
C	0.13817	-2.51299	-0.50036
C	-1.05519	-2.66165	0.45683
H	-0.19964	-2.68393	-1.53116
H	0.88731	-3.28003	-0.27597
H	-0.68821	-2.72216	1.49272
H	-1.59848	-3.59400	0.26257
C	-0.21884	-0.00171	-0.70500

O	0.12019	1.03746	-1.28350
C	0.79626	-1.11962	-0.40636
H	1.09153	-0.97722	0.64216
C	2.06725	-1.03346	-1.29793
H	2.41135	-2.06150	-1.47009
H	1.77497	-0.62927	-2.27294
C	2.80586	1.76205	0.50568
C	3.18865	1.17270	-0.85266
N	3.23130	-0.28602	-0.84784
C	3.97331	-0.80560	0.29626
C	3.59853	-0.14630	1.62948
O	3.67521	1.27456	1.53261
H	1.76216	1.51640	0.74963
H	2.91536	2.85151	0.50487
H	4.19212	1.54747	-1.10935
H	2.48324	1.50780	-1.61391
H	3.83349	-1.89043	0.35907
H	5.04557	-0.61776	0.12879
H	2.58592	-0.44123	1.94682
H	4.30178	-0.44127	2.41531
C	-2.78532	2.04719	-0.67529
H	-2.30280	2.13817	-1.65436
H	-3.82956	2.35607	-0.80328
C	-2.08734	3.00256	0.30627
H	-1.03033	2.73718	0.40433
H	-2.14841	4.03936	-0.04501
H	-2.55009	2.94918	1.29832
C	-3.75975	-0.12301	0.32073
C	-5.18684	0.21461	0.60241
H	-5.41496	1.22286	0.24851
H	-5.87534	-0.47922	0.10332
H	-5.41012	0.18060	1.67657

4s_Senantiomer_conformer-008

C	3.37973	-0.32743	-0.19426
N	3.17218	1.89738	-0.02306
H	3.42205	2.87650	0.01292
C	1.90304	1.41919	0.00062
C	1.98092	0.03012	-0.09763
C	-0.50671	1.38219	-0.46465
C	0.64815	2.21721	0.10790
H	-0.39661	1.31456	-1.55539
H	-1.46612	1.86777	-0.26950
H	0.44187	2.46035	1.16137
H	0.74008	3.17312	-0.42118

C	0.79074	-0.79548	-0.09058
O	0.80869	-2.02636	-0.20363
C	-0.53746	-0.04171	0.11754
H	-0.64057	0.04656	1.21145
C	-1.70978	-0.86413	-0.43178
H	-1.56382	-0.99255	-1.51180
H	-1.68658	-1.87525	0.00809
C	-5.43814	-0.49794	-0.58147
C	-4.02870	-0.84386	-1.09702
N	-3.00681	-0.22046	-0.24604
C	-3.46456	-0.18081	1.13738
C	-4.65760	0.78519	1.22763
O	-5.45116	0.77957	0.03853
H	-5.79021	-1.26571	0.12393
H	-6.15170	-0.45666	-1.40837
H	-3.88783	-0.48256	-2.12283
H	-3.92837	-1.94564	-1.11983
H	-3.73530	-1.18670	1.51174
H	-2.66568	0.18948	1.78509
H	-5.27866	0.53242	2.10114
H	-4.30330	1.81430	1.34156
C	3.93966	-1.71760	-0.28568
H	3.36934	-2.28899	-1.02605
H	4.97532	-1.67549	-0.64352
C	3.89337	-2.46695	1.05562
H	4.31389	-3.47475	0.95726
H	4.46467	-1.93067	1.82193
H	2.85858	-2.55986	1.39839
C	4.09098	0.85175	-0.14159
C	5.55715	1.13159	-0.18746
H	5.91555	1.58469	0.74589
H	6.11551	0.20503	-0.34130
H	5.81520	1.81802	-1.00399

4s_Senantiomer_conformer-009

C	-2.88645	0.62844	0.20400
N	-3.32995	-1.35150	-0.74803
H	-3.82580	-2.07749	-1.24754
C	-2.07183	-1.46832	-0.25631
C	-1.74791	-0.25363	0.34839
C	0.26386	-2.21185	-0.17294
C	-1.19448	-2.67183	-0.31768
H	0.57088	-1.68751	-1.08326
H	0.92364	-3.08044	-0.07063
H	-1.45050	-3.36800	0.49555

H	-1.33955	-3.22017	-1.25604
C	-0.48375	-0.06009	1.02403
O	-0.17915	0.98323	1.61590
C	0.46481	-1.27799	1.03389
H	0.16345	-1.83232	1.93886
C	1.94412	-0.90666	1.24428
H	2.00730	-0.11066	2.00487
H	2.44555	-1.78782	1.65955
C	3.43389	1.77639	-0.35931
C	2.32563	0.74950	-0.56812
N	2.66181	-0.56508	0.01730
C	4.10852	-0.73587	0.18397
C	4.84861	-0.05773	-0.95845
O	4.67016	1.36226	-0.94966
H	3.57398	1.95813	0.71737
H	3.16657	2.73021	-0.82624
H	2.14348	0.63857	-1.64606
H	1.40860	1.13176	-0.11901
H	4.47555	-0.31404	1.13863
H	4.34947	-1.80641	0.19565
H	5.92441	-0.24363	-0.87943
H	4.50048	-0.47498	-1.91587
C	-2.97481	2.05179	0.67357
H	-4.02295	2.37382	0.67829
H	-2.61720	2.11478	1.70713
C	-2.14852	3.01692	-0.19162
H	-2.48406	2.99110	-1.23463
H	-1.09111	2.73769	-0.16243
H	-2.24073	4.04697	0.17253
C	-3.84711	-0.08033	-0.48370
C	-5.21633	0.29241	-0.94864
H	-5.29341	0.27638	-2.04352
H	-5.97980	-0.39188	-0.55715
H	-5.46899	1.30124	-0.61363

4s_Senantiomer_conformer-010

C	2.04961	-0.66591	0.51734
N	2.47579	0.50326	-1.34569
H	2.90467	0.78011	-2.21841
C	1.52590	1.21681	-0.68553
C	1.22056	0.51823	0.48245
C	0.27637	3.19110	0.08889
C	0.92939	2.51156	-1.12630
H	-0.34310	4.03185	-0.24364
H	1.06961	3.60989	0.71998

H	1.69173	3.16688	-1.56449
H	0.18215	2.34583	-1.91530
C	0.21715	1.00037	1.41145
O	-0.01785	0.48574	2.50825
C	-0.57355	2.23463	0.94840
H	-0.84238	2.76851	1.86578
C	-1.91193	1.78427	0.26910
H	-2.72698	2.00799	0.96222
H	-2.09575	2.39950	-0.62657
C	-1.66375	-1.46745	-1.64849
C	-1.69131	0.04317	-1.44999
N	-1.98677	0.35665	-0.05568
C	-3.24659	-0.26555	0.34694
C	-3.17702	-1.77004	0.10999
O	-2.89467	-2.06848	-1.25449
H	-0.83285	-1.89451	-1.06633
H	-1.51975	-1.71407	-2.70423
H	-2.43940	0.49093	-2.13315
H	-0.71094	0.44410	-1.71333
H	-3.41182	-0.07669	1.41313
H	-4.10986	0.14769	-0.20998
H	-2.39970	-2.20630	0.75668
H	-4.13609	-2.24198	0.34160
C	2.04752	-1.72377	1.58328
H	2.95569	-2.33259	1.49940
H	2.07632	-1.24335	2.56733
C	0.81365	-2.63762	1.52056
H	0.75495	-3.14882	0.55279
H	-0.09688	-2.04697	1.65272
H	0.84754	-3.39942	2.30852
C	2.80979	-0.64907	-0.63294
C	3.83217	-1.60025	-1.16232
H	3.51616	-2.05147	-2.11178
H	4.79561	-1.10562	-1.34032
H	4.00026	-2.41167	-0.45010

4s_Senantiomer_conformer-011

C	3.04905	0.65750	0.26387
N	3.62504	-1.34411	-0.56339
H	4.19823	-2.09996	-0.91349
C	2.28679	-1.41717	-0.35388
C	1.87853	-0.18641	0.15803
C	0.13417	-2.44580	0.23641
C	1.40371	-2.58874	-0.61714
H	0.38111	-2.63905	1.28864

H	-0.60072	-3.19920	-0.06644
H	1.12810	-2.62321	-1.68210
H	1.91763	-3.53162	-0.39620
C	0.49334	0.07248	0.49265
O	0.10261	1.12579	1.00739
C	-0.50133	-1.04465	0.11756
H	-0.73383	-0.88550	-0.94737
C	-1.82864	-0.95146	0.89410
H	-2.11486	-1.95593	1.22504
H	-1.68927	-0.33825	1.80013
C	-3.96064	1.39170	-1.15890
C	-2.80613	0.97033	-0.25844
N	-2.93556	-0.45070	0.07401
C	-4.22556	-0.67531	0.72655
C	-5.35549	-0.21776	-0.18926
O	-5.22058	1.15793	-0.53359
H	-3.90706	0.83802	-2.11013
H	-3.91323	2.46345	-1.37172
H	-2.80401	1.59799	0.65177
H	-1.85698	1.14385	-0.77214
H	-4.33895	-1.74493	0.93869
H	-4.29763	-0.13182	1.68915
H	-5.35698	-0.83387	-1.10280
H	-6.32375	-0.32254	0.30840
C	3.07790	2.08326	0.73353
H	2.50067	2.17040	1.66046
H	4.10856	2.37305	0.97032
C	2.49688	3.06117	-0.30045
H	1.45086	2.81401	-0.50472
H	2.53947	4.09300	0.06789
H	3.05488	3.01031	-1.24238
C	4.11332	-0.08806	-0.19499
C	5.56454	0.23352	-0.33809
H	5.77127	1.23422	0.04886
H	6.19476	-0.47582	0.21336
H	5.88642	0.20953	-1.38721

4s_Senantiomer_conformer-012

C	-3.02645	-0.69258	-0.22955
N	-3.56086	1.46490	-0.51573
H	-4.11368	2.29340	-0.69015
C	-2.23643	1.46216	-0.22272
C	-1.85494	0.13434	-0.03519
C	-0.10959	2.26558	0.70797
C	-1.33892	2.64864	-0.12986

H	-0.40356	2.17414	1.76188
H	0.64000	3.06195	0.64970
H	-1.01619	2.95893	-1.13528
H	-1.85894	3.50740	0.31076
C	-0.48318	-0.22519	0.25955
O	-0.11286	-1.38556	0.46872
C	0.52838	0.93868	0.24649
H	0.81302	1.06832	-0.80962
C	1.81566	0.62169	1.03235
H	2.07210	1.48721	1.65319
H	1.63821	-0.22650	1.71455
C	4.07695	-1.04429	-1.48821
C	2.87557	-0.89470	-0.56322
N	2.96842	0.37515	0.16135
C	4.22261	0.40756	0.91387
C	5.40099	0.22755	-0.03686
O	5.30246	-0.99536	-0.76092
H	4.06002	-0.24476	-2.24632
H	4.05538	-2.01253	-1.99635
H	2.84074	-1.75315	0.13289
H	1.95353	-0.91750	-1.15009
H	4.31019	1.37403	1.42396
H	4.25709	-0.38540	1.68664
H	5.43670	1.07626	-0.73869
H	6.34307	0.18829	0.51731
C	-3.09451	-2.18633	-0.09488
H	-4.00101	-2.56015	-0.58573
H	-2.24181	-2.63554	-0.61582
C	-3.07686	-2.65260	1.36968
H	-2.15247	-2.32620	1.85510
H	-3.92530	-2.23384	1.92286
H	-3.13165	-3.74576	1.43446
C	-4.06611	0.16251	-0.52428
C	-5.51012	-0.08967	-0.80916
H	-5.71116	-1.16353	-0.82630
H	-6.16059	0.35816	-0.04671
H	-5.81187	0.32373	-1.77993

4s_Senantiomer_conformer-013

C	3.03671	0.60063	0.14860
N	3.40940	-1.47828	-0.60036
H	3.89990	-2.28885	-0.95342
C	2.09415	-1.44967	-0.26966
C	1.81126	-0.16724	0.19892
C	-0.04855	-2.30195	0.57604

C	1.12024	-2.57267	-0.38344
H	0.28958	-2.45381	1.60968
H	-0.85318	-3.02233	0.39265
H	0.74124	-2.64219	-1.41448
H	1.59546	-3.53492	-0.15918
C	0.47878	0.20299	0.63030
O	0.19462	1.31557	1.08809
C	-0.60754	-0.87253	0.43619
H	-0.92736	-0.75318	-0.61131
C	-1.83576	-0.63374	1.35510
H	-2.10448	-1.57991	1.84027
H	-1.55618	0.07396	2.14059
C	-4.98092	-0.50372	-0.72020
C	-3.64096	-1.05328	-0.24078
N	-3.04905	-0.12465	0.71812
C	-2.89674	1.20054	0.12012
C	-4.25412	1.69458	-0.36656
O	-4.82890	0.78685	-1.30511
H	-5.67980	-0.44897	0.12974
H	-5.41157	-1.14808	-1.49191
H	-2.99445	-1.22106	-1.12349
H	-3.79324	-2.02320	0.24772
H	-2.49040	1.88544	0.86979
H	-2.19382	1.19591	-0.73386
H	-4.93154	1.81950	0.49331
H	-4.15628	2.65419	-0.88217
C	3.19843	2.04504	0.52518
H	2.70252	2.22626	1.48492
H	4.26166	2.27269	0.66710
C	2.60694	3.00208	-0.52228
H	1.53453	2.81808	-0.63630
H	2.74539	4.04735	-0.22155
H	3.08788	2.85941	-1.49669
C	4.00634	-0.23953	-0.35461
C	5.45536	-0.02514	-0.64530
H	5.76110	0.97600	-0.33194
H	6.08794	-0.74960	-0.11666
H	5.67518	-0.11984	-1.71661

4s_Senantiomer_conformer-014

C	2.99296	0.58734	-0.33819
N	3.32809	-1.62124	-0.51708
H	3.79673	-2.50307	-0.67556
C	2.03512	-1.48778	-0.12923
C	1.77753	-0.12326	-0.00382

C	-0.06707	-2.05114	1.00824
C	1.05491	-2.58614	0.10580
H	0.31944	-1.92341	2.02809
H	-0.88013	-2.78324	1.06286
H	0.62841	-2.92418	-0.85092
H	1.53826	-3.45982	0.55890
C	0.46413	0.36806	0.35937
O	0.19986	1.56749	0.49982
C	-0.63116	-0.70456	0.51428
H	-1.00461	-0.86699	-0.50937
C	-1.81028	-0.20927	1.39504
H	-2.04873	-0.98304	2.13486
H	-1.49226	0.68379	1.94033
C	-5.06244	-0.62013	-0.46771
C	-3.69562	-1.03072	0.07131
N	-3.05810	0.11846	0.70795
C	-2.94511	1.22885	-0.23597
C	-4.32943	1.58449	-0.76560
O	-4.94915	0.46098	-1.38919
H	-5.71507	-0.32969	0.37107
H	-5.53022	-1.44619	-1.01084
H	-3.09640	-1.43839	-0.76538
H	-3.81592	-1.82943	0.81301
H	-2.50254	2.08843	0.27546
H	-2.28943	0.98476	-1.09266
H	-4.96003	1.94521	0.06272
H	-4.26489	2.36640	-1.52765
C	3.19150	2.07530	-0.30919
H	4.08140	2.34025	-0.89259
H	2.33728	2.56340	-0.79093
C	3.33283	2.62919	1.11771
H	2.42846	2.41462	1.69476
H	4.18698	2.17281	1.63085
H	3.48168	3.71540	1.10425
C	3.93435	-0.36971	-0.64966
C	5.36745	-0.25978	-1.05490
H	6.03744	-0.71167	-0.31197
H	5.55887	-0.75839	-2.01364
H	5.65174	0.78983	-1.16165

4s_Senantiomer_conformer-015

C	-2.77500	0.77976	0.12280
N	-3.30597	-1.21088	-0.75864
H	-3.83559	-1.93141	-1.23056
C	-2.10920	-1.40075	-0.14892

C	-1.73330	-0.18066	0.41384
C	-0.32806	-2.56530	1.08576
C	-1.34184	-2.67772	-0.06842
H	0.37127	-3.40785	1.05314
H	-0.86916	-2.62947	2.03725
H	-2.01065	-3.53181	0.09062
H	-0.81925	-2.86688	-1.01678
C	-0.49518	-0.04130	1.15172
O	-0.19656	0.96535	1.80503
C	0.46412	-1.23940	1.04746
H	1.13194	-1.19704	1.91371
C	1.34711	-1.16674	-0.22800
H	1.55119	-2.19172	-0.55782
H	0.78720	-0.67951	-1.04923
C	3.94406	1.49311	0.42880
C	2.55433	0.91218	0.20312
N	2.64773	-0.53350	-0.01829
C	3.53979	-0.79529	-1.14996
C	4.90928	-0.17976	-0.88726
O	4.81315	1.22425	-0.66891
H	4.37147	1.07293	1.35353
H	3.89390	2.58109	0.52771
H	2.08431	1.41793	-0.66456
H	1.92869	1.10640	1.07664
H	3.64592	-1.87858	-1.28041
H	3.13332	-0.38058	-2.09322
H	5.36658	-0.66509	-0.01005
H	5.56680	-0.31933	-1.74996
C	-2.76355	2.23494	0.49138
H	-3.76676	2.65763	0.36098
H	-2.50827	2.33820	1.55156
C	-1.75517	3.04632	-0.33868
H	-1.98730	2.97954	-1.40765
H	-0.74178	2.66398	-0.18259
H	-1.76974	4.10385	-0.04995
C	-3.73117	0.11191	-0.61079
C	-5.01974	0.56571	-1.21358
H	-5.22093	1.60457	-0.94126
H	-5.00102	0.50620	-2.30946
H	-5.86599	-0.04131	-0.86763

4s_Senantiomer_conformer-016

C	3.29299	0.42721	-0.37686
N	3.34102	-1.79334	-0.07901
H	3.69936	-2.73549	0.00051

C	2.04585	-1.43735	0.10851
C	1.96454	-0.05654	-0.06775
C	-0.21364	-1.50127	1.06104
C	0.90924	-2.34358	0.43815
H	0.09231	-1.17706	2.06409
H	-1.11130	-2.11433	1.18673
H	0.53839	-2.83665	-0.47333
H	1.22356	-3.14222	1.12036
C	0.70178	0.64650	0.02516
O	0.59358	1.87119	-0.09061
C	-0.54395	-0.25330	0.21751
H	-0.79962	-0.58356	-0.80217
C	-1.74327	0.54505	0.75377
H	-1.91204	0.29137	1.81888
H	-1.49277	1.60804	0.70806
C	-5.23828	1.13918	-0.49337
C	-3.99613	1.31164	0.37235
N	-2.96363	0.35675	-0.03317
C	-3.49679	-1.00318	0.05355
C	-4.75429	-1.12698	-0.79985
O	-5.74935	-0.18814	-0.40360
H	-4.99215	1.37526	-1.54096
H	-6.03812	1.80611	-0.15980
H	-4.27741	1.17961	1.43570
H	-3.60536	2.32903	0.25573
H	-2.75093	-1.71414	-0.31513
H	-3.73593	-1.27744	1.10027
H	-4.49355	-0.97142	-1.85898
H	-5.19834	-2.12006	-0.68853
C	3.68884	1.85599	-0.61355
H	4.65982	1.89021	-1.12193
H	2.96156	2.32663	-1.28412
C	3.76325	2.67534	0.68487
H	2.78593	2.68945	1.17630
H	4.49295	2.24205	1.37838
H	4.05911	3.71089	0.47923
C	4.12200	-0.67359	-0.37471
C	5.59014	-0.81279	-0.60946
H	6.11357	-1.17460	0.28502
H	5.80752	-1.51699	-1.42259
H	6.02425	0.15304	-0.87889

4s_Senantiomer_conformer-017

C	-3.32522	0.48975	-0.09821
N	-3.39132	-1.73008	0.20225

H	-3.76100	-2.66114	0.34005
C	-2.08028	-1.43500	0.01810
C	-1.98689	-0.05622	-0.16832
C	0.22324	-1.75354	-0.76255
C	-0.94095	-2.39622	0.00577
H	-0.02840	-1.71246	-1.83020
H	1.11722	-2.37804	-0.67207
H	-0.62299	-2.62331	1.03473
H	-1.23081	-3.35012	-0.45026
C	-0.71196	0.59485	-0.38723
O	-0.59327	1.80519	-0.60170
C	0.52950	-0.32409	-0.27185
H	0.73350	-0.36624	0.81029
C	1.76213	0.30025	-0.94561
H	1.97548	-0.23046	-1.89436
H	1.52330	1.33636	-1.19878
C	5.20626	1.20642	0.25422
C	4.00515	1.14013	-0.68127
N	2.94355	0.33049	-0.08118
C	3.46244	-1.00278	0.22604
C	4.68012	-0.89280	1.13715
O	5.70349	-0.09643	0.54803
H	4.91821	1.71612	1.18752
H	6.02831	1.75824	-0.21005
H	4.33068	0.72623	-1.65593
H	3.62286	2.15158	-0.86076
H	2.69221	-1.58747	0.73850
H	3.74318	-1.54940	-0.69612
H	4.37585	-0.45714	2.10233
H	5.11564	-1.87946	1.31767
C	-3.70352	1.93875	-0.20613
H	-3.17991	2.38762	-1.05705
H	-4.77741	2.02523	-0.41015
C	-3.35913	2.73834	1.06080
H	-3.87667	2.32642	1.93464
H	-2.28190	2.70055	1.24849
H	-3.65165	3.78953	0.95323
C	-4.17087	-0.57278	0.13592
C	-5.65200	-0.64438	0.31220
H	-6.10007	0.34068	0.16128
H	-6.11353	-1.33600	-0.40409
H	-5.92756	-0.98436	1.31887

4s_Senantiomer_conformer-018

C	-2.74207	0.67370	-0.36134

N	-3.16010	-1.50852	-0.65401
H	-3.63793	-2.35780	-0.92433
C	-1.98387	-1.45996	0.01967
C	-1.67980	-0.11377	0.22596
C	-0.20592	-2.13990	1.57542
C	-1.16683	-2.62280	0.47338
H	0.52505	-2.92288	1.80496
H	-0.78288	-1.95650	2.48963
H	-1.80476	-3.43171	0.84835
H	-0.59999	-3.04216	-0.36995
C	-0.46438	0.28917	0.90194
O	-0.21187	1.45766	1.21902
C	0.53755	-0.84478	1.18195
H	1.16097	-0.52393	2.02257
C	1.48153	-1.09683	-0.02616
H	1.70098	-2.16958	-0.07001
H	0.96580	-0.84509	-0.97271
C	4.04023	1.67511	0.06157
C	2.66588	1.03678	-0.09105
N	2.76983	-0.41479	0.08035
C	3.72741	-0.95097	-0.88887
C	5.08013	-0.27028	-0.71319
O	4.97547	1.14102	-0.87285
H	4.40729	1.51554	1.08828
H	3.98824	2.75061	-0.12978
H	2.25555	1.29062	-1.08910
H	1.98325	1.44337	0.65836
H	3.83815	-2.02939	-0.72591
H	3.38087	-0.80128	-1.93023
H	5.48064	-0.50571	0.28597
H	5.78929	-0.62013	-1.46870
C	-2.84656	2.17139	-0.35535
H	-1.87061	2.60221	-0.60555
H	-3.54656	2.49564	-1.13465
C	-3.29770	2.73149	1.00308
H	-3.36979	3.82514	0.97222
H	-2.57628	2.45644	1.77796
H	-4.27868	2.33144	1.28386
C	-3.64218	-0.22027	-0.89912
C	-4.93072	-0.01706	-1.62593
H	-5.78221	-0.43053	-1.07010
H	-5.11825	1.04926	-1.77331
H	-4.92120	-0.49596	-2.61329

4s_Senantiomer_conformer-019

C	3.25656	0.21168	-0.02769
N	2.68800	-1.95241	0.09285
H	2.76727	-2.95092	0.23116
C	1.57108	-1.31659	-0.34089
C	1.87642	0.04297	-0.42739
C	-0.52219	-1.00061	-1.59487
C	0.25834	-1.94622	-0.66599
H	-1.55284	-1.35082	-1.69539
H	-0.06222	-1.02261	-2.59014
H	0.39702	-2.92482	-1.14037
H	-0.31688	-2.12596	0.25361
C	0.88587	1.00961	-0.85165
O	1.09891	2.22137	-0.97101
C	-0.52617	0.45164	-1.08640
H	-0.98147	1.09836	-1.84360
C	-1.30208	0.61491	0.24058
H	-0.75164	0.10523	1.03961
H	-1.32145	1.68890	0.50397
C	-4.91817	0.10527	-0.69036
C	-3.55382	0.78492	-0.66771
N	-2.64245	0.04025	0.20211
C	-3.21759	-0.06774	1.54357
C	-4.58614	-0.73391	1.46756
O	-5.46837	-0.00473	0.61895
H	-4.82006	-0.89560	-1.14048
H	-5.62953	0.68925	-1.28074
H	-3.67009	1.83148	-0.32494
H	-3.15267	0.81004	-1.68555
H	-2.55012	-0.67062	2.16978
H	-3.32488	0.92503	2.02192
H	-4.47207	-1.76372	1.09300
H	-5.05602	-0.76654	2.45440
C	3.99974	1.51254	0.06975
H	5.07815	1.31945	0.11520
H	3.81942	2.10150	-0.83622
C	3.57803	2.34670	1.29027
H	3.75274	1.79333	2.22000
H	4.14315	3.28504	1.33856
H	2.51347	2.59115	1.22826
C	3.72970	-1.04253	0.29094
C	5.06436	-1.50976	0.77089
H	5.49199	-2.27114	0.10609
H	5.76573	-0.67308	0.81470
H	5.00630	-1.94832	1.77544

4s_Senantiomer_conformer-020

C	-2.27078	-0.98755	-0.37474
N	-3.23472	0.57149	0.91314
H	-3.90049	1.01775	1.52936
C	-2.15725	1.18503	0.35743
C	-1.52364	0.24609	-0.45537
C	-0.70682	3.00229	-0.49951
C	-1.73487	2.59999	0.57808
H	-0.19530	3.92238	-0.19527
H	-1.24019	3.22638	-1.43064
H	-2.59600	3.27831	0.54654
H	-1.30087	2.70928	1.58180
C	-0.33974	0.58981	-1.21629
O	0.12713	-0.10066	-2.12732
C	0.33368	1.89947	-0.79111
H	0.95748	2.22212	-1.63048
C	1.22600	1.64315	0.44331
H	1.86367	2.53454	0.60528
H	0.59044	1.54290	1.32887
C	3.38879	-1.22798	1.52653
C	2.64780	0.09928	1.63387
N	2.01262	0.41804	0.35629
C	3.01544	0.44942	-0.70909
C	3.75861	-0.88026	-0.75606
O	4.36943	-1.18920	0.49412
H	2.66605	-2.03544	1.32673
H	3.92009	-1.45114	2.45600
H	3.36140	0.88781	1.94349
H	1.87516	0.02592	2.40806
H	2.51389	0.60414	-1.66697
H	3.74095	1.27183	-0.55415
H	3.05401	-1.67999	-1.03489
H	4.56290	-0.84574	-1.49621
C	-1.88696	-2.28327	-1.02807
H	-2.72482	-2.98900	-0.98143
H	-1.67490	-2.10603	-2.08789
C	-0.64518	-2.91518	-0.37543
H	-0.83787	-3.14967	0.67785
H	0.20140	-2.22286	-0.42003
H	-0.36000	-3.84253	-0.88613
C	-3.32093	-0.75599	0.48708
C	-4.41432	-1.64400	0.98296
H	-4.35352	-1.80069	2.06771
H	-5.40632	-1.22643	0.76833
H	-4.35388	-2.62344	0.50220

4s_Senantiomer_conformer-021

C	-2.71203	-0.58543	0.27253
N	-2.98810	1.57970	0.78122
H	-3.39169	2.42306	1.16633
C	-1.82006	1.52231	0.09490
C	-1.60573	0.18766	-0.24942
C	0.49330	2.12462	-0.44589
C	-0.93194	2.66565	-0.25864
H	0.90032	1.84182	0.52962
H	1.14499	2.91126	-0.84203
H	-1.27895	3.14136	-1.18837
H	-0.95502	3.43983	0.51721
C	-0.45293	-0.21524	-1.02611
O	-0.27498	-1.36580	-1.44165
C	0.54536	0.90792	-1.38787
H	0.21191	1.24143	-2.38439
C	1.97672	0.38162	-1.58088
H	1.92615	-0.57286	-2.13106
H	2.52125	1.09533	-2.20927
C	3.05017	-0.81333	1.84240
C	2.20826	-0.76577	0.57257
N	2.73906	0.25038	-0.33806
C	4.14731	-0.02843	-0.62068
C	4.94181	-0.08961	0.67799
O	4.41948	-1.08245	1.55604
H	2.70488	-1.61477	2.50148
H	2.96222	0.14691	2.37601
H	1.17989	-0.51290	0.84701
H	2.18937	-1.76533	0.10064
H	4.27066	-0.98549	-1.16452
H	4.54829	0.76900	-1.25704
H	5.98305	-0.36035	0.48181
H	4.91729	0.89568	1.17061
C	-2.91623	-2.06512	0.12002
H	-1.96545	-2.58066	0.29433
H	-3.61716	-2.42181	0.88432
C	-3.43829	-2.45327	-1.27259
H	-3.58315	-3.53744	-1.34943
H	-4.39692	-1.96421	-1.48021
H	-2.72032	-2.14768	-2.03924
C	-3.54964	0.30640	0.90641
C	-4.83887	0.11321	1.63460
H	-5.09323	-0.94852	1.67914
H	-4.78542	0.48916	2.66433
H	-5.66900	0.63342	1.13947

4s_Senantiomer_conformer-022

C	-2.34820	-0.87550	0.19685
N	-3.06718	1.10100	0.96891
H	-3.64181	1.78664	1.44009
C	-1.96228	1.38748	0.23374
C	-1.47642	0.17993	-0.26876
C	-0.42052	2.67885	-1.20114
C	-1.38467	2.74371	-0.00019
H	0.18346	3.59233	-1.24099
H	-1.01033	2.64420	-2.12447
H	-2.17518	3.47924	-0.19132
H	-0.85684	3.09195	0.89854
C	-0.29071	0.13449	-1.09925
O	0.09151	-0.86914	-1.71000
C	0.50577	1.44481	-1.16817
H	1.07364	1.41489	-2.10324
C	1.48890	1.53712	0.01849
H	2.14917	2.41132	-0.15023
H	0.92538	1.73920	0.93559
C	3.73939	-0.90381	1.76638
C	3.01632	0.41141	1.50166
N	2.25235	0.31872	0.25824
C	3.14252	-0.03405	-0.84864
C	3.86923	-1.33442	-0.52799
O	4.60671	-1.24225	0.68826
H	2.99828	-1.70510	1.91665
H	4.36453	-0.82862	2.66058
H	3.76001	1.23168	1.46341
H	2.32872	0.62400	2.32869
H	2.54736	-0.18106	-1.75243
H	3.88435	0.76591	-1.04088
H	3.13278	-2.15081	-0.45801
H	4.59097	-1.57660	-1.31306
C	-2.21951	-2.33746	-0.11995
H	-1.16731	-2.63242	-0.03780
H	-2.77350	-2.92650	0.62083
C	-2.71993	-2.68761	-1.53042
H	-2.61694	-3.76113	-1.72860
H	-2.13662	-2.14234	-2.27791
H	-3.77554	-2.41733	-1.64847
C	-3.32210	-0.27195	0.96275
C	-4.49319	-0.83176	1.70109
H	-4.46579	-0.57192	2.76698
H	-5.44445	-0.46032	1.29846

H	-4.50400	-1.92162	1.62281

4s_Senantiomer_conformer-023

C	-3.23533	0.54087	0.27635
N	-3.43560	-1.67688	0.02444
H	-3.86089	-2.58912	-0.07272
C	-2.10184	-1.43346	-0.01833
C	-1.92573	-0.05832	0.13528
C	0.23365	-1.71412	-0.71340
C	-1.01337	-2.43882	-0.18654
H	0.06029	-1.41322	-1.75559
H	1.09933	-2.38124	-0.70932
H	-0.78506	-2.91620	0.77860
H	-1.31777	-3.24206	-0.86802
C	-0.60443	0.53423	0.17975
O	-0.40157	1.74775	0.30300
C	0.57007	-0.46116	0.11277
H	0.73085	-0.78191	1.15547
C	1.84296	0.23443	-0.38942
H	1.69317	0.51045	-1.43990
H	1.96921	1.17519	0.16207
C	4.89739	0.92061	-0.81898
C	4.08019	-0.29128	-1.27026
N	3.01408	-0.64896	-0.32711
C	3.58687	-0.78018	1.02036
C	4.41857	0.43628	1.43425
O	5.44916	0.69667	0.47975
H	4.27149	1.82778	-0.80753
H	5.74443	1.09590	-1.48950
H	4.75841	-1.15134	-1.35361
H	3.64254	-0.11186	-2.25827
H	2.78642	-0.95500	1.74643
H	4.23654	-1.66509	1.02633
H	3.77939	1.32716	1.54174
H	4.91901	0.25859	2.39105
C	-3.53267	2.00449	0.42951
H	-4.55365	2.13629	0.80726
H	-2.85784	2.43496	1.17746
C	-3.36973	2.78405	-0.88518
H	-3.59877	3.84680	-0.74257
H	-2.33998	2.70083	-1.24519
H	-4.03951	2.39007	-1.65808
C	-4.14842	-0.48848	0.20123
C	-5.64037	-0.50081	0.26395
H	-6.00886	-1.16304	1.05773

H	-6.01984	0.50407	0.46430
H	-6.08571	-0.84236	-0.67949

4s_Senantiomer_conformer-024

C	-3.24009	0.52902	-0.25375
N	-3.44202	-1.65203	0.22030
H	-3.86678	-2.55474	0.38533
C	-2.10639	-1.41694	0.19056
C	-1.92933	-0.06358	-0.09674
C	0.25969	-1.88736	-0.25440
C	-1.01801	-2.41057	0.41796
H	0.14219	-1.94911	-1.34487
H	1.11948	-2.50649	0.01392
H	-0.84258	-2.54126	1.49666
H	-1.29295	-3.39623	0.02423
C	-0.60763	0.52205	-0.19082
O	-0.40269	1.70947	-0.46790
C	0.56257	-0.42871	0.12750
H	0.67122	-0.38645	1.22414
C	1.86251	0.07528	-0.51466
H	1.76681	-0.01476	-1.60317
H	1.96679	1.14707	-0.30126
C	4.93927	0.60123	-1.01206
C	4.13437	-0.69863	-1.06286
N	3.02345	-0.72447	-0.10429
C	3.53214	-0.38632	1.23277
C	4.35329	0.90527	1.24759
O	5.42868	0.83565	0.30964
H	4.32051	1.45317	-1.33799
H	5.81779	0.54590	-1.66242
H	4.80904	-1.53045	-0.81875
H	3.74396	-0.86837	-2.07214
H	2.69728	-0.31082	1.93692
H	4.17360	-1.21136	1.56897
H	3.71673	1.77455	1.01758
H	4.80792	1.06646	2.22985
C	-3.53703	1.97316	-0.53847
H	-2.88270	2.32504	-1.34340
H	-4.56806	2.07364	-0.89817
C	-3.33498	2.87642	0.68880
H	-3.56780	3.92091	0.44994
H	-3.98200	2.56184	1.51561
H	-2.29530	2.82618	1.02561
C	-4.15487	-0.48097	-0.04815
C	-5.64798	-0.48997	-0.07029

H	-6.03979	-1.22029	-0.78976
H	-6.06931	-0.74053	0.91187
H	-6.02909	0.49437	-0.35268

4s_Senantiomer_conformer-025

C	-2.26398	0.88091	-0.03851
N	-3.08648	-0.99502	-0.94723
H	-3.70755	-1.61747	-1.44640
C	-1.95526	-1.37924	-0.30340
C	-1.40443	-0.23775	0.28060
C	-0.36501	-2.83160	0.90563
C	-1.40713	-2.76553	-0.22848
H	0.21086	-3.76059	0.82500
H	-0.89212	-2.86392	1.86645
H	-2.20650	-3.49471	-0.05014
H	-0.94976	-3.04414	-1.18795
C	-0.16824	-0.30424	1.03154
O	0.28404	0.62806	1.70786
C	0.59710	-1.62408	0.91076
H	1.23897	-1.69019	1.79544
C	1.50663	-1.66743	-0.35399
H	2.03637	-2.63006	-0.31401
H	0.87886	-1.68575	-1.25114
C	4.52580	0.52425	0.15407
C	3.36824	-0.36652	0.59404
N	2.49777	-0.62118	-0.55123
C	2.01405	0.62813	-1.13934
C	3.21496	1.47880	-1.53956
O	4.05258	1.74310	-0.41556
H	5.14614	-0.01351	-0.58096
H	5.14958	0.80233	1.00856
H	2.82811	0.12031	1.42240
H	3.76289	-1.32191	0.96174
H	1.41671	0.39470	-2.02841
H	1.38526	1.21092	-0.44966
H	3.79281	0.95949	-2.32094
H	2.89247	2.45141	-1.92250
C	-2.06965	2.30448	0.39700
H	-1.01873	2.58454	0.25967
H	-2.65989	2.96970	-0.24454
C	-2.45380	2.53756	1.86680
H	-1.83116	1.91642	2.51695
H	-2.30774	3.58704	2.14878
H	-3.50468	2.27933	2.04079
C	-3.29528	0.37830	-0.80217

C	-4.48329	1.03544	-1.42408
H	-4.45355	2.11392	-1.25116
H	-4.51918	0.87001	-2.50839
H	-5.42448	0.65742	-1.00432

4s_Senantiomer_conformer-026

C	-3.25486	0.40967	0.18170
N	-3.13945	-1.66650	-0.65381
H	-3.42434	-2.53661	-1.08300
C	-1.89914	-1.41021	-0.16911
C	-1.91952	-0.11897	0.35948
C	0.26815	-1.88753	0.89528
C	-0.72874	-2.33301	-0.19001
H	1.20079	-2.44847	0.78270
H	-0.14689	-2.14879	1.87619
H	-1.04032	-3.36970	-0.01621
H	-0.24995	-2.31765	-1.17913
C	-0.73878	0.46921	0.95080
O	-0.70056	1.59963	1.44924
C	0.54867	-0.37051	0.87307
H	1.12702	-0.09306	1.76065
C	1.36635	0.10249	-0.36000
H	1.25604	-0.62495	-1.18667
H	0.96098	1.05204	-0.72473
C	4.90072	1.27572	-0.84721
C	3.44740	0.97725	-1.19438
N	2.77635	0.34646	-0.05545
C	3.50041	-0.86591	0.32932
C	4.95587	-0.52909	0.63504
O	5.59585	0.08833	-0.47719
H	4.94031	2.00527	-0.02274
H	5.42631	1.69087	-1.71155
H	3.41735	0.33009	-2.09267
H	2.92938	1.91159	-1.43960
H	3.04595	-1.29530	1.22684
H	3.46076	-1.63174	-0.47036
H	4.99952	0.13882	1.51009
H	5.52095	-1.43842	0.85728
C	-3.72646	1.78087	0.57062
H	-4.82262	1.80362	0.58630
H	-3.38824	2.00428	1.58835
C	-3.20566	2.87766	-0.37206
H	-3.57338	3.86450	-0.06700
H	-2.11206	2.89907	-0.35378
H	-3.53169	2.69396	-1.40216

C	-3.98459	-0.57201	-0.45190
C	-5.40634	-0.61778	-0.90608
H	-5.48338	-0.70846	-1.99725
H	-5.94582	-1.46745	-0.46856
H	-5.92795	0.29614	-0.61194

4s_Senantiomer_conformer-027

C	3.19151	0.42236	0.31290
N	3.04255	-1.81191	0.39922
H	3.30350	-2.77831	0.54233
C	1.83866	-1.39431	-0.06506
C	1.88285	-0.00150	-0.13651
C	-0.26278	-1.45779	-1.34611
C	0.67692	-2.25638	-0.42462
H	-1.19644	-2.01240	-1.47919
H	0.20580	-1.37701	-2.33434
H	1.00659	-3.17496	-0.92434
H	0.14436	-2.57457	0.48259
C	0.73273	0.76664	-0.55734
O	0.71679	1.99900	-0.65087
C	-0.55371	-0.03379	-0.82773
H	-1.08858	0.53213	-1.59780
C	-1.43339	0.00661	0.45161
H	-1.35476	-0.95512	0.99343
H	-1.05475	0.77800	1.13034
C	-4.96640	-0.23147	-0.86627
C	-3.52361	-0.66092	-0.62292
N	-2.83008	0.34736	0.17994
C	-3.55951	0.56344	1.43157
C	-4.99779	0.97079	1.13650
O	-5.66346	-0.01889	0.35714
H	-5.51171	-1.00978	-1.40707
H	-4.97555	0.69164	-1.46755
H	-3.02366	-0.76712	-1.58997
H	-3.51401	-1.65093	-0.12562
H	-3.56507	-0.34804	2.06070
H	-3.06250	1.35765	2.00049
H	-5.56718	1.07544	2.06414
H	-5.00545	1.93487	0.60357
C	3.69391	1.83564	0.37422
H	2.92710	2.47124	0.83064
H	4.57764	1.88617	1.02139
C	4.04083	2.40358	-1.01130
H	3.15193	2.40347	-1.64904
H	4.40718	3.43399	-0.93273

H	4.81601	1.80049	-1.49764
C	3.88580	-0.72287	0.63553
C	5.27520	-0.93424	1.14026
H	5.76431	0.02666	1.31720
H	5.28471	-1.49457	2.08375
H	5.88933	-1.49273	0.42202

4s_Senantiomer_conformer-028

C	3.37088	0.29653	-0.23638
N	3.12329	-1.92098	-0.02458
H	3.35710	-2.90021	0.06982
C	1.86238	-1.42235	-0.04864
C	1.96373	-0.03873	-0.17933
C	-0.52192	-1.25969	0.51929
C	0.59125	-2.19480	0.02066
H	-0.36555	-1.04990	1.58557
H	-1.48194	-1.77296	0.43061
H	0.32896	-2.58409	-0.97470
H	0.68964	-3.06510	0.68003
C	0.78800	0.79796	-0.29401
O	0.84120	2.02284	-0.45787
C	-0.57615	0.07262	-0.24998
H	-0.80978	-0.15589	-1.30274
C	-1.65195	1.05242	0.27621
H	-1.44888	1.26952	1.33228
H	-1.51489	1.99090	-0.26962
C	-5.13882	-0.14826	1.10497
C	-3.61707	-0.17794	1.21271
N	-3.05659	0.67282	0.16933
C	-3.49083	0.24907	-1.15404
C	-5.01660	0.23994	-1.20541
O	-5.56453	-0.58937	-0.18330
H	-5.50297	0.87488	1.28938
H	-5.59513	-0.82637	1.83189
H	-3.29291	-1.23106	1.14219
H	-3.30568	0.20227	2.19210
H	-3.10482	0.95170	-1.90218
H	-3.11958	-0.75933	-1.41898
H	-5.39122	1.26954	-1.09103
H	-5.37236	-0.16405	-2.15756
C	3.95442	1.67587	-0.34097
H	5.00893	1.60875	-0.63384
H	3.43789	2.22825	-1.13353
C	3.83756	2.47227	0.96850
H	4.27878	3.47030	0.86151

H	2.78525	2.59090	1.24286
H	4.35271	1.95604	1.78662
C	4.06165	-0.89141	-0.13403
C	5.52393	-1.19381	-0.11316
H	6.10294	-0.27873	-0.25885
H	5.83533	-1.63593	0.84211
H	5.80516	-1.89827	-0.90630

4s_Senantiomer_conformer-029

C	-3.36557	0.27069	-0.22832
N	-3.11783	-1.89590	0.28936
H	-3.34948	-2.86085	0.48318
C	-1.85856	-1.39484	0.24094
C	-1.96047	-0.04125	-0.07414
C	0.55270	-1.39429	-0.22645
C	-0.58972	-2.13360	0.48842
H	0.45257	-1.53873	-1.31023
H	1.50409	-1.84119	0.07030
H	-0.38125	-2.17964	1.56798
H	-0.65777	-3.17040	0.13874
C	-0.78759	0.80054	-0.18049
O	-0.84245	2.01161	-0.42550
C	0.57390	0.11471	0.07729
H	0.75066	0.23765	1.15846
C	1.68075	0.88757	-0.67870
H	1.53848	0.74784	-1.75758
H	1.51608	1.94993	-0.47487
C	5.20469	-0.46462	-0.87932
C	3.69112	-0.55195	-1.05184
N	3.07607	0.58562	-0.37808
C	3.43526	0.62741	1.03207
C	4.95587	0.65976	1.16462
O	5.55725	-0.45083	0.50307
H	5.58119	0.44653	-1.37074
H	5.69872	-1.33686	-1.31705
H	3.36105	-1.52816	-0.65542
H	3.43507	-0.52027	-2.11682
H	3.01021	1.53075	1.48565
H	3.04746	-0.24358	1.59409
H	5.33913	1.60026	0.73790
H	5.25731	0.59687	2.21418
C	-3.95267	1.61736	-0.53882
H	-3.38180	2.08311	-1.34948
H	-4.98062	1.49666	-0.90119
C	-3.94676	2.56300	0.67296

H	-4.38729	3.53356	0.41555
H	-4.52029	2.13629	1.50375
H	-2.92021	2.73134	1.01130
C	-4.05502	-0.89958	0.00460
C	-5.51520	-1.21239	-0.00591
H	-6.08889	-0.33443	-0.31253
H	-5.75164	-2.02729	-0.70195
H	-5.87532	-1.51487	0.98593

4s_Senantiomer_conformer-030

C	3.04902	-0.65739	-0.26384
N	3.62487	1.34425	0.56344
H	4.19800	2.10013	0.91360
C	2.28661	1.41723	0.35394
C	1.87843	0.18644	-0.15800
C	0.13399	2.44583	-0.23639
C	1.40350	2.58877	0.61720
H	0.38094	2.63910	-1.28861
H	-0.60093	3.19920	0.06646
H	1.12786	2.62322	1.68214
H	1.91742	3.53166	0.39628
C	0.49323	-0.07245	-0.49265
O	0.10249	-1.12572	-1.00745
C	-0.50147	1.04465	-0.11755
H	-0.73397	0.88549	0.94737
C	-1.82877	0.95142	-0.89411
H	-2.11501	1.95588	-1.22507
H	-1.68936	0.33820	-1.80013
C	-3.96066	-1.39173	1.15901
C	-2.80615	-0.97035	0.25855
N	-2.93568	0.45064	-0.07402
C	-4.22566	0.67507	-0.72666
C	-5.35560	0.21753	0.18914
O	-5.22060	-1.15812	0.53363
H	-3.90715	-0.83794	2.11019
H	-3.91317	-2.46344	1.37194
H	-2.80395	-1.59809	-0.65160
H	-1.85701	-1.14376	0.77231
H	-4.33913	1.74466	-0.93892
H	-4.29763	0.13148	-1.68920
H	-5.35719	0.83374	1.10261
H	-6.32385	0.32217	-0.30857
C	3.07822	-2.08311	-0.73363
H	2.50070	-2.17042	-1.66036
H	4.10891	-2.37242	-0.97089

C	2.49811	-3.06141	0.30051
H	2.54134	-4.09321	-0.06781
H	3.05632	-3.01014	1.24230
H	1.45199	-2.81495	0.50506
C	4.11323	0.08824	0.19503
C	5.56448	-0.23324	0.33796
H	5.77113	-1.23420	-0.04839
H	6.19459	0.47574	-0.21408
H	5.88658	-0.20859	1.38700

4s_Senantiomer_conformer-031

C	-2.64187	0.72054	0.28627
N	-3.34231	-1.22895	-0.56992
H	-3.94043	-1.91513	-1.01024
C	-2.07455	-1.46123	-0.14982
C	-1.59288	-0.27053	0.39430
C	0.17390	-2.43098	-0.22894
C	-1.32881	-2.75008	-0.21433
H	0.43425	-1.98501	-1.19342
H	0.75593	-3.35518	-0.14228
H	-1.57377	-3.37391	0.65855
H	-1.61265	-3.32760	-1.10193
C	-0.28040	-0.19498	0.99625
O	0.13519	0.79735	1.60710
C	0.58565	-1.47131	0.90199
H	0.40171	-1.98378	1.86065
C	2.08890	-1.14812	0.88597
H	2.26615	-0.36017	1.63164
H	2.63331	-2.03808	1.22140
C	2.59370	1.66874	-0.48997
C	2.05338	0.37026	-1.08746
N	2.61142	-0.83103	-0.44998
C	4.07650	-0.73573	-0.47151
C	4.59698	0.58108	0.11221
O	4.02304	1.69856	-0.56672
H	2.26650	1.77381	0.55388
H	2.23681	2.53625	-1.05422
H	2.33289	0.33492	-2.14906
H	0.96165	0.34970	-1.04042
H	4.50285	-1.58716	0.07106
H	4.40448	-0.80339	-1.51766
H	4.37267	0.64302	1.18908
H	5.68085	0.66351	-0.01634
C	-2.54657	2.15996	0.70157
H	-3.54701	2.60833	0.71146

H	-2.16096	2.21713	1.72541
C	-1.62683	2.98244	-0.21565
H	-0.60995	2.57944	-0.18970
H	-1.58718	4.02983	0.10585
H	-1.98306	2.95524	-1.25169
C	-3.70951	0.09689	-0.32149
C	-5.05721	0.59769	-0.72478
H	-5.19661	0.56196	-1.81300
H	-5.86304	0.00574	-0.27253
H	-5.18710	1.63521	-0.40785

4s_Senantiomer_conformer-032

C	2.35918	-0.04845	0.05948
N	1.53877	-0.32222	-2.00765
H	1.44684	-0.65314	-2.95870
C	0.73973	0.61035	-1.43120
C	1.21096	0.81249	-0.13300
C	-0.78418	2.53711	-1.25453
C	-0.42857	1.27990	-2.06869
H	-1.76621	2.91364	-1.56365
H	-0.05450	3.32071	-1.49354
H	-0.20381	1.55290	-3.10753
H	-1.27819	0.58964	-2.08724
C	0.53143	1.69979	0.78373
O	0.90296	1.91964	1.94404
C	-0.77277	2.32549	0.27254
H	-0.83935	3.30761	0.75708
C	-1.99344	1.52518	0.79296
H	-2.00246	1.57315	1.89440
H	-2.89456	2.04311	0.44610
C	-3.26090	-1.69435	-0.71344
C	-3.37336	-0.31197	-0.07704
N	-2.04454	0.14122	0.32426
C	-1.45544	-0.81701	1.25675
C	-1.37341	-2.18392	0.59157
O	-2.66132	-2.63336	0.17469
H	-2.66480	-1.62105	-1.63754
H	-4.25025	-2.08892	-0.96206
H	-4.07322	-0.36194	0.77970
H	-3.78761	0.38938	-0.80966
H	-0.45164	-0.49349	1.53425
H	-2.05540	-0.89513	2.18466
H	-0.69486	-2.12460	-0.27425
H	-0.99290	-2.93496	1.28960
C	3.16736	-0.19520	1.31674

H	4.12527	-0.67466	1.08235
H	3.39904	0.79761	1.71786
C	2.44587	-1.00792	2.40402
H	2.17588	-2.00335	2.03315
H	1.53169	-0.49355	2.71365
H	3.08251	-1.13255	3.28804
C	2.53283	-0.74088	-1.11861
C	3.53127	-1.77245	-1.52907
H	3.05638	-2.74063	-1.73432
H	4.07332	-1.47578	-2.43607
H	4.26673	-1.92611	-0.73571

4s_Senantiomer_conformer-033

C	-2.62454	0.69065	-0.18235
N	-3.22914	-1.43564	-0.55263
H	-3.78359	-2.23129	-0.83939
C	-1.97538	-1.50741	-0.04124
C	-1.55523	-0.20120	0.21142
C	0.30523	-2.37610	0.19919
C	-1.18650	-2.74257	0.23090
H	0.60066	-2.16743	-0.83344
H	0.90846	-3.22743	0.53323
H	-1.45219	-3.15112	1.21768
H	-1.41001	-3.52590	-0.50286
C	-0.26193	0.07530	0.79654
O	0.11369	1.20900	1.11934
C	0.62999	-1.15467	1.07769
H	0.38483	-1.41886	2.11992
C	2.12685	-0.80553	1.08013
H	2.24496	0.15071	1.60940
H	2.65205	-1.56643	1.66836
C	2.73820	1.58406	-0.91533
C	2.23517	0.17061	-1.20669
N	2.74252	-0.82678	-0.25354
C	4.20588	-0.71993	-0.19757
C	4.68941	0.70705	0.07657
O	4.16946	1.61349	-0.89650
H	2.33727	1.94119	0.04365
H	2.42720	2.27896	-1.70183
H	2.58664	-0.12412	-2.20469
H	1.14278	0.14886	-1.22820
H	4.59009	-1.40537	0.56638
H	4.60567	-1.03915	-1.16961
H	4.38980	1.03067	1.08625
H	5.78001	0.76980	0.00732

C	-2.62394	2.18783	-0.06807
H	-1.65433	2.57422	-0.40075
H	-3.38239	2.60947	-0.73858
C	-2.88265	2.67632	1.36620
H	-3.85282	2.31979	1.73069
H	-2.88038	3.77175	1.41402
H	-2.10267	2.30199	2.03562
C	-3.64634	-0.10548	-0.65175
C	-4.99550	0.22933	-1.19704
H	-5.13859	-0.17539	-2.20705
H	-5.80075	-0.17067	-0.56743
H	-5.12400	1.31313	-1.24997

4s_Senantiomer_conformer-034

C	2.15654	0.42251	-0.17917
N	1.25304	1.11146	1.75169
H	1.07761	1.67581	2.57208
C	0.62925	-0.05933	1.46775
C	1.16355	-0.53163	0.26829
C	-0.62649	-2.15233	1.80710
C	-0.45077	-0.69553	2.27292
H	-1.56109	-2.56001	2.20964
H	0.18911	-2.75136	2.23079
H	-0.21060	-0.66776	3.34344
H	-1.38301	-0.13790	2.13432
C	0.63119	-1.70781	-0.38146
O	1.06095	-2.17010	-1.44689
C	-0.60433	-2.33372	0.27638
H	-0.55025	-3.40730	0.05708
C	-1.89091	-1.82767	-0.42482
H	-1.88111	-2.17511	-1.47087
H	-2.74365	-2.31356	0.06249
C	-3.43958	1.55566	0.20067
C	-3.43476	0.04940	-0.04430
N	-2.07415	-0.37945	-0.35215
C	-1.58039	0.33884	-1.52490
C	-1.61287	1.83557	-1.24701
O	-2.93222	2.27075	-0.92228
H	-2.83136	1.77963	1.09204
H	-4.45763	1.91929	0.36714
H	-4.14335	-0.18875	-0.86139
H	-3.77921	-0.46410	0.86011
H	-0.55523	0.03389	-1.74218
H	-2.19545	0.11755	-2.41921
H	-0.92358	2.06432	-0.41898

H	-1.30818	2.40367	-2.13051
C	2.99492	0.31597	-1.41963
H	2.35256	0.05699	-2.26909
H	3.44407	1.29035	-1.64592
C	4.10192	-0.74356	-1.30251
H	3.65873	-1.72910	-1.13235
H	4.70042	-0.78965	-2.22008
H	4.77345	-0.51611	-0.46669
C	2.18777	1.42809	0.76154
C	3.00413	2.67430	0.86101
H	3.64169	2.78172	-0.01983
H	2.37302	3.56964	0.92906
H	3.65595	2.66625	1.74429

4s_Senantiomer_conformer-035

C	3.10921	0.44984	-0.06505
N	2.92206	-1.70567	0.51786
H	3.16656	-2.61963	0.87469
C	1.73721	-1.39184	-0.06310
C	1.80384	-0.04922	-0.43983
C	-0.30621	-1.74081	-1.39004
C	0.57521	-2.30549	-0.26250
H	-1.25635	-2.28014	-1.42349
H	0.20350	-1.89770	-2.34827
H	0.90957	-3.32062	-0.50674
H	-0.00763	-2.38765	0.66633
C	0.68269	0.60756	-1.07770
O	0.68078	1.78966	-1.44083
C	-0.59018	-0.23895	-1.22179
H	-1.09705	0.13339	-2.11886
C	-1.48198	0.06675	0.00614
H	-0.94084	-0.21418	0.91613
H	-1.62431	1.15806	0.05495
C	-4.07238	0.40041	1.79367
C	-3.35023	-0.85847	1.31029
N	-2.73295	-0.69218	-0.01112
C	-3.73516	-0.17679	-0.95482
C	-4.45443	1.06845	-0.43149
O	-5.05301	0.81278	0.84030
H	-3.35296	1.21730	1.96702
H	-4.60904	0.21121	2.72849
H	-4.08486	-1.67175	1.23807
H	-2.58320	-1.16191	2.03114
H	-3.26025	0.03534	-1.91765
H	-4.47945	-0.96645	-1.12007

H	-3.75596	1.91650	-0.34895
H	-5.26664	1.36054	-1.10403
C	3.60847	1.85439	-0.24436
H	4.70045	1.87365	-0.14645
H	3.37383	2.19583	-1.25860
C	2.98795	2.83701	0.76187
H	3.37485	3.85159	0.60979
H	3.21320	2.53510	1.79108
H	1.90118	2.86280	0.63924
C	3.77611	-0.59995	0.52794
C	5.14369	-0.70342	1.11865
H	5.73167	-1.50050	0.64594
H	5.68650	0.23547	0.98540
H	5.10839	-0.91755	2.19466

4s_Senantiomer_conformer-036

C	3.02541	0.32022	0.46314
N	2.79237	-1.87117	0.05756
H	3.00363	-2.85914	0.01674
C	1.65901	-1.30013	-0.42142
C	1.75807	0.07300	-0.18996
C	-0.29890	-0.98339	-1.87979
C	0.51056	-2.00454	-1.06182
H	-1.24649	-1.42795	-2.19464
H	0.26747	-0.72589	-2.78286
H	0.85826	-2.82300	-1.70335
H	-0.12858	-2.46467	-0.29439
C	0.68027	0.97312	-0.54134
O	0.70747	2.19664	-0.36484
C	-0.58771	0.30671	-1.09412
H	-1.04281	1.04235	-1.76642
C	-1.54354	0.07875	0.10189
H	-1.05376	-0.58038	0.82677
H	-1.68388	1.04663	0.60898
C	-4.22513	-0.31207	1.73575
C	-3.48383	-1.26313	0.79452
N	-2.79534	-0.56528	-0.29800
C	-3.74238	0.33131	-0.97608
C	-4.48331	1.25276	-0.00454
O	-5.15088	0.49463	1.00577
H	-3.51241	0.33245	2.27566
H	-4.81246	-0.86761	2.47337
H	-4.21740	-1.94721	0.34707
H	-2.75814	-1.86606	1.35120
H	-3.21476	0.92140	-1.73171

H	-4.48025	-0.29119	-1.49845
H	-3.78659	1.96493	0.46586
H	-5.25669	1.82613	-0.52460
C	3.56646	1.65895	0.87415
H	2.78357	2.22021	1.39640
H	4.38878	1.52175	1.58639
C	4.05697	2.49485	-0.31877
H	4.45082	3.46207	0.01520
H	3.23002	2.68178	-1.01010
H	4.85142	1.97077	-0.86227
C	3.64257	-0.90405	0.59962
C	4.96256	-1.29392	1.17878
H	5.46621	-0.41941	1.59766
H	4.85225	-2.03404	1.98158
H	5.62733	-1.72958	0.42166

4s_Senantiomer_conformer-037

C	3.24859	0.20079	-0.05768
N	2.64835	-1.95744	-0.00375
H	2.71684	-2.96202	0.08901
C	1.52626	-1.28618	-0.36557
C	1.85089	0.07108	-0.40736
C	-0.60120	-0.87248	-1.51636
C	0.19259	-1.88143	-0.66983
H	-1.63316	-1.20984	-1.63955
H	-0.15911	-0.84049	-2.52002
H	0.29743	-2.83255	-1.20482
H	-0.34584	-2.10908	0.26114
C	0.86044	1.07283	-0.74401
O	1.09092	2.28657	-0.80595
C	-0.56988	0.55181	-0.93392
H	-1.05229	1.23479	-1.64150
C	-1.27867	0.72733	0.44496
H	-0.75509	0.13620	1.20495
H	-1.14761	1.78242	0.71897
C	-4.53385	-1.03238	1.19940
C	-3.06348	-0.97636	0.79497
N	-2.69617	0.41300	0.54380
C	-3.54649	1.02282	-0.47008
C	-5.00702	0.89731	-0.04511
O	-5.36205	-0.46375	0.18736
H	-4.67670	-0.49085	2.14787
H	-4.86293	-2.06763	1.32636
H	-2.92404	-1.63201	-0.08216
H	-2.44344	-1.36870	1.60877

H	-3.28421	2.08280	-0.56854
H	-3.42285	0.55371	-1.46374
H	-5.17390	1.48782	0.86965
H	-5.67361	1.26324	-0.83113
C	4.01719	1.48463	0.06478
H	5.09326	1.27425	0.05047
H	3.80438	2.11809	-0.80325
C	3.66565	2.26580	1.34122
H	4.24509	3.19442	1.40548
H	2.60273	2.52455	1.34119
H	3.87663	1.66775	2.23508
C	3.71225	-1.07296	0.18963
C	5.05604	-1.57988	0.59854
H	5.44050	-2.32760	-0.10679
H	5.77508	-0.75815	0.63883
H	5.03083	-2.04889	1.59064

4s_Senantiomer_conformer-038

C	-2.79725	0.53737	0.15086
N	-3.03623	-1.41327	-0.92664
H	-3.43875	-2.13682	-1.50694
C	-1.83411	-1.49541	-0.30459
C	-1.63873	-0.29815	0.38310
C	0.51663	-2.11020	-0.02794
C	-0.89205	-2.65063	-0.31782
H	0.83744	-1.51579	-0.88925
H	1.22462	-2.93957	0.07497
H	-1.17772	-3.38656	0.44895
H	-0.91754	-3.17336	-1.28104
C	-0.47563	-0.08991	1.21997
O	-0.32291	0.90579	1.93779
C	0.55787	-1.23705	1.23876
H	0.23290	-1.86951	2.08318
C	1.97361	-0.74326	1.62818
H	1.84262	-0.06032	2.47429
H	2.55032	-1.60348	1.98555
C	3.27494	1.87055	-0.76454
C	2.19286	1.07702	-0.03861
N	2.79786	-0.06612	0.63252
C	3.55391	-0.89999	-0.29587
C	4.61070	-0.04179	-0.98420
O	4.00664	1.05093	-1.67358
H	3.96319	2.30945	-0.02439
H	2.83284	2.67581	-1.35845
H	1.41797	0.77599	-0.77005

H	1.70522	1.71178	0.70618
H	4.04021	-1.70796	0.26265
H	2.92875	-1.36268	-1.07858
H	5.32508	0.33717	-0.23592
H	5.15477	-0.62159	-1.73554
C	-3.00009	1.93422	0.66192
H	-4.04840	2.22716	0.53001
H	-2.79428	1.96022	1.73763
C	-2.09103	2.95826	-0.03668
H	-2.27550	2.97094	-1.11693
H	-1.03998	2.70457	0.13069
H	-2.26570	3.96821	0.35265
C	-3.64100	-0.18007	-0.66911
C	-4.96702	0.15294	-1.26990
H	-5.30843	1.12952	-0.91839
H	-4.92013	0.19156	-2.36594
H	-5.73249	-0.58624	-1.00133

4s_Senantiomer_conformer-039

C	-3.19209	0.10633	-0.35742
N	-2.57467	-1.97763	0.18754
H	-2.62512	-2.97562	0.34216
C	-1.48709	-1.20772	0.44166
C	-1.82452	0.10707	0.11518
C	0.56278	-0.46059	1.56351
C	-0.17121	-1.67343	0.96799
H	1.58660	-0.73137	1.83253
H	0.05827	-0.17497	2.49500
H	-0.30367	-2.45310	1.72736
H	0.42454	-2.12593	0.16260
C	-0.85882	1.18125	0.22268
O	-1.09673	2.36224	-0.05785
C	0.56045	0.75888	0.62437
H	0.99336	1.61452	1.15389
C	1.35442	0.58062	-0.70639
H	0.88637	-0.20407	-1.31154
H	1.23241	1.52154	-1.25881
C	4.66738	-1.23157	-0.77028
C	3.17332	-1.10892	-0.48494
N	2.77826	0.28763	-0.63279
C	3.55523	1.16449	0.23343
C	5.04151	0.96900	-0.05335
O	5.42269	-0.39573	0.10399
H	4.86694	-0.95495	-1.81763
H	5.01327	-2.25492	-0.59921

H	2.98295	-1.51381	0.52424
H	2.61125	-1.71761	-1.20208
H	3.27788	2.20592	0.03190
H	3.37081	0.97072	1.30638
H	5.26314	1.30224	-1.07959
H	5.65188	1.54567	0.64734
C	-3.98241	1.30682	-0.79107
H	-3.37226	1.91553	-1.46753
H	-4.86254	0.98368	-1.35965
C	-4.42985	2.18348	0.38956
H	-5.00729	3.04751	0.04001
H	-5.05537	1.61229	1.08501
H	-3.55613	2.55308	0.93431
C	-3.62819	-1.19942	-0.29818
C	-4.94023	-1.82612	-0.63842
H	-5.62264	-1.07797	-1.04883
H	-4.82935	-2.62382	-1.38389
H	-5.42211	-2.26756	0.24355

4s_Senantiomer_conformer-040

C	-2.87547	0.73249	-0.28417
N	-3.64480	-1.32227	0.17347
H	-4.29240	-2.08256	0.33214
C	-2.29440	-1.44519	0.15018
C	-1.76693	-0.18446	-0.12658
C	-0.12065	-2.51406	-0.26298
C	-1.50628	-2.68968	0.37717
H	-0.21832	-2.55727	-1.35573
H	0.53147	-3.34148	0.03610
H	-1.39168	-2.87339	1.45623
H	-2.01894	-3.56579	-0.03697
C	-0.33890	0.03317	-0.22007
O	0.16411	1.12260	-0.52018
C	0.54639	-1.17953	0.13045
H	0.63501	-1.17382	1.22830
C	1.97099	-1.06321	-0.44648
H	2.26525	-2.03678	-0.85366
H	1.95966	-0.35733	-1.28927
C	4.86015	0.14297	-0.80393
C	4.34913	-0.95350	0.13381
N	2.96830	-0.73406	0.58310
C	2.85737	0.62694	1.13153
C	3.39571	1.69877	0.18391
O	4.75112	1.42462	-0.18509
H	4.29781	0.13601	-1.75175

H	5.92037	-0.00227	-1.03456
H	4.99291	-0.97070	1.02375
H	4.42005	-1.93368	-0.35158
H	1.81455	0.84184	1.37432
H	3.43881	0.65647	2.06291
H	2.76364	1.76052	-0.71308
H	3.40193	2.67963	0.67005
C	-2.78063	2.20579	-0.55744
H	-2.07040	2.37774	-1.37356
H	-3.75338	2.58095	-0.89695
C	-2.32367	3.01048	0.67002
H	-2.27290	4.08135	0.44019
H	-1.32962	2.68146	0.98702
H	-3.01730	2.87237	1.50723
C	-4.02405	-0.00317	-0.08808
C	-5.46715	0.38001	-0.11300
H	-6.03428	-0.21884	-0.83700
H	-5.57599	1.43100	-0.39118
H	-5.94237	0.24422	0.86707

4s_Senantiomer_conformer-041

C	-2.72047	-0.48969	0.35003
N	-2.86562	1.72074	0.69130
H	-3.21431	2.61287	1.01529
C	-1.71063	1.54195	0.00357
C	-1.57745	0.17483	-0.23783
C	0.62809	1.94380	-0.59070
C	-0.75364	2.59742	-0.43468
H	0.99453	1.67760	0.40583
H	1.33803	2.66148	-1.01564
H	-1.07447	3.03030	-1.39430
H	-0.71466	3.42262	0.28584
C	-0.46189	-0.35273	-0.99514
O	-0.35682	-1.54352	-1.31337
C	0.58063	0.68256	-1.47138
H	0.20656	0.99082	-2.46332
C	1.96381	0.04178	-1.74579
H	1.77251	-0.89231	-2.28464
H	2.51932	0.70265	-2.42024
C	3.41502	-1.58920	1.33393
C	2.28832	-1.07278	0.44422
N	2.85103	-0.26177	-0.62765
C	3.67236	0.82571	-0.10611
C	4.77099	0.23795	0.77408
O	4.21212	-0.52219	1.84373

H	4.04785	-2.28136	0.75550
H	3.01241	-2.11883	2.20227
H	1.57007	-0.50740	1.06897
H	1.74468	-1.91506	0.00812
H	4.12197	1.36945	-0.94481
H	3.10396	1.55276	0.49876
H	5.43045	-0.40153	0.16576
H	5.36798	1.03082	1.23413
C	-3.01221	-1.96221	0.31479
H	-2.09165	-2.51736	0.52549
H	-3.72593	-2.21591	1.10782
C	-3.56855	-2.42758	-1.04039
H	-2.84117	-2.22579	-1.83206
H	-4.49904	-1.90103	-1.28166
H	-3.77683	-3.50406	-1.03052
C	-3.49825	0.49589	0.91826
C	-4.78855	0.43507	1.66741
H	-5.59332	0.96103	1.13769
H	-5.10208	-0.60345	1.79845
H	-4.70249	0.88790	2.66339

4s_Senantiomer_conformer-042

C	2.86586	0.75705	-0.19053
N	3.62379	-1.35208	-0.19247
H	4.26632	-2.13264	-0.21380
C	2.27767	-1.45805	-0.06614
C	1.75752	-0.16437	-0.06012
C	0.12508	-2.38871	0.66717
C	1.48572	-2.71661	0.03373
H	0.26440	-2.17717	1.73551
H	-0.53553	-3.25907	0.59373
H	1.33116	-3.14680	-0.96742
H	2.01665	-3.47472	0.62152
C	0.33292	0.07753	0.02717
O	-0.16211	1.20991	0.07899
C	-0.56124	-1.17824	0.00024
H	-0.69275	-1.42685	-1.06460
C	-1.96235	-0.92363	0.59064
H	-2.23530	-1.77388	1.22531
H	-1.92026	-0.04081	1.24436
C	-3.42673	1.62191	-0.60125
C	-2.92196	0.35835	-1.29785
N	-3.00224	-0.83882	-0.44593
C	-4.36253	-0.94346	0.09759
C	-4.84136	0.34155	0.77758

O	-4.76436	1.44583	-0.12354
H	-3.45820	2.46455	-1.29941
H	-2.75868	1.88585	0.23067
H	-1.89128	0.50730	-1.62664
H	-3.54164	0.17446	-2.18587
H	-5.04159	-1.16265	-0.73764
H	-4.40838	-1.78520	0.79810
H	-5.89062	0.25751	1.07814
H	-4.24130	0.55079	1.67802
C	2.78192	2.25600	-0.19962
H	3.70748	2.67457	-0.61259
H	1.96753	2.56822	-0.86253
C	2.53236	2.84669	1.19727
H	3.33465	2.56567	1.88904
H	1.58457	2.47452	1.59708
H	2.48226	3.94127	1.15732
C	4.00738	-0.01076	-0.26892
C	5.44787	0.36067	-0.39910
H	6.02935	0.05347	0.47981
H	5.91113	-0.10697	-1.27719
H	5.55222	1.44337	-0.50300

4s_Senantiomer_conformer-043

C	3.03674	0.60060	0.14860
N	3.40941	-1.47832	-0.60030
H	3.89990	-2.28892	-0.95333
C	2.09414	-1.44968	-0.26967
C	1.81127	-0.16724	0.19889
C	-0.04859	-2.30190	0.57603
C	1.12021	-2.57265	-0.38345
H	0.28954	-2.45375	1.60967
H	-0.85323	-3.02227	0.39264
H	0.74121	-2.64217	-1.41448
H	1.59540	-3.53491	-0.15917
C	0.47880	0.20304	0.63024
O	0.19466	1.31563	1.08801
C	-0.60755	-0.87247	0.43615
H	-0.92738	-0.75312	-0.61135
C	-1.83576	-0.63365	1.35507
H	-2.10444	-1.57980	1.84030
H	-1.55617	0.07409	2.14052
C	-4.25429	1.69456	-0.36651
C	-2.89688	1.20060	0.12014
N	-3.04908	-0.12461	0.71811
C	-3.64097	-1.05327	-0.24078

C	-4.98097	-0.50379	-0.72015
O	-4.82904	0.78680	-1.30506
H	-4.15652	2.65417	-0.88213
H	-4.93170	1.81944	0.49338
H	-2.49059	1.88552	0.86982
H	-2.19397	1.19605	-0.73384
H	-2.99448	-1.22100	-1.12352
H	-3.79318	-2.02320	0.24772
H	-5.41160	-1.14816	-1.49186
H	-5.67983	-0.44909	0.12981
C	3.19849	2.04500	0.52518
H	2.70237	2.22630	1.48480
H	4.26171	2.27257	0.66733
C	2.60730	3.00205	-0.52244
H	1.53492	2.81808	-0.63674
H	3.08849	2.85936	-1.49672
H	2.74569	4.04732	-0.22169
C	4.00637	-0.23959	-0.35455
C	5.45541	-0.02523	-0.64518
H	5.67524	-0.11984	-1.71650
H	5.76119	0.97586	-0.33171
H	6.08794	-0.74979	-0.11661

4s_Senantiomer_conformer-044

C	3.32518	0.48979	0.09818
N	3.39138	-1.73003	-0.20229
H	3.76112	-2.66108	-0.34006
C	2.08032	-1.43501	-0.01819
C	1.98686	-0.05624	0.16823
C	-0.22316	-1.75373	0.76249
C	0.94106	-2.39631	-0.00585
H	0.02847	-1.71268	1.83014
H	-1.11711	-2.37828	0.67198
H	0.62310	-2.62342	-1.03481
H	1.23100	-3.35020	0.45016
C	0.71189	0.59474	0.38715
O	0.59310	1.80507	0.60159
C	-0.52952	-0.32428	0.27184
H	-0.73359	-0.36643	-0.81028
C	-1.76214	0.29999	0.94569
H	-1.97553	-0.23088	1.89434
H	-1.52328	1.33604	1.19904
C	-5.20615	1.20657	-0.25414
C	-4.00510	1.14005	0.68142
N	-2.94355	0.33041	0.08124

C	-3.46256	-1.00280	-0.22604
C	-4.68018	-0.89265	-1.13722
O	-5.70350	-0.09618	-0.54814
H	-4.91798	1.71636	-1.18736
H	-6.02817	1.75843	0.21015
H	-4.33071	0.72604	1.65600
H	-3.62274	2.15144	0.86107
H	-2.69236	-1.58755	-0.73847
H	-3.74340	-1.54940	0.69608
H	-4.37580	-0.45700	-2.10237
H	-5.11580	-1.87926	-1.31780
C	3.70346	1.93880	0.20620
H	3.17984	2.38760	1.05716
H	4.77734	2.02524	0.41030
C	3.35918	2.73858	-1.06063
H	3.65187	3.78971	-0.95292
H	3.87669	2.32671	-1.93452
H	2.28196	2.70099	-1.24834
C	4.17088	-0.57270	-0.13592
C	5.65201	-0.64427	-0.31217
H	6.10004	0.34082	-0.16135
H	6.11355	-1.33579	0.40421
H	5.92760	-0.98436	-1.31880

4s_Senantiomer_conformer-045

C	-2.77512	0.77965	0.12283
N	-3.30599	-1.21106	-0.75850
H	-3.83557	-1.93165	-1.23038
C	-2.10920	-1.40083	-0.14879
C	-1.73335	-0.18069	0.41388
C	-0.32786	-2.56517	1.08580
C	-1.34172	-2.67774	-0.06830
H	0.37155	-3.40766	1.05316
H	-0.86888	-2.62936	2.03733
H	-2.01044	-3.53188	0.09082
H	-0.81918	-2.86688	-1.01669
C	-0.49519	-0.04117	1.15165
O	-0.19660	0.96558	1.80482
C	0.46420	-1.23920	1.04740
H	1.13208	-1.19675	1.91359
C	1.34709	-1.16647	-0.22814
H	1.55103	-2.19141	-0.55815
H	0.78720	-0.67904	-1.04926
C	3.94438	1.49295	0.42903
C	2.55458	0.91224	0.20325

N	2.64780	-0.53342	-0.01839
C	3.53981	-0.79516	-1.15011
C	4.90939	-0.17984	-0.88731
O	4.81344	1.22414	-0.66873
H	4.37174	1.07256	1.35369
H	3.89437	2.58092	0.52811
H	2.08465	1.41817	-0.66438
H	1.92893	1.10640	1.07676
H	3.64579	-1.87844	-1.28076
H	3.13340	-0.38022	-2.09330
H	5.36662	-0.66538	-0.01018
H	5.56690	-0.31935	-1.75003
C	-2.76380	2.23485	0.49132
H	-3.76712	2.65735	0.36121
H	-2.50820	2.33822	1.55142
C	-1.75581	3.04636	-0.33909
H	-1.98819	2.97939	-1.40799
H	-0.74228	2.66427	-0.18320
H	-1.77054	4.10392	-0.05049
C	-3.73126	0.11171	-0.61071
C	-5.01992	0.56544	-1.21338
H	-5.22066	1.60459	-0.94184
H	-5.00161	0.50503	-2.30922
H	-5.86626	-0.04100	-0.86663

4s_Senantiomer_conformer-046

C	2.40359	-0.85177	0.38947
N	3.29013	0.84220	-0.77885
H	3.95463	1.38003	-1.31883
C	2.10102	1.31006	-0.32228
C	1.50399	0.28085	0.40759
C	0.46314	2.94254	0.52975
C	1.54916	2.68063	-0.53174
H	-0.09521	3.85013	0.27343
H	0.95258	3.12829	1.49310
H	2.33979	3.43685	-0.45986
H	1.12896	2.77266	-1.54287
C	0.21860	0.46483	1.04930
O	-0.29231	-0.35639	1.82102
C	-0.51653	1.76049	0.69162
H	-1.18572	1.97413	1.53161
C	-1.38660	1.61890	-0.59390
H	-1.85521	2.60113	-0.75062
H	-0.73543	1.43529	-1.45531
C	-4.55351	-0.25801	0.19445

C	-3.34838	0.62297	0.50937
N	-2.43885	0.61541	-0.63329
C	-2.02616	-0.74702	-0.96802
C	-3.27011	-1.58036	-1.25657
O	-4.15186	-1.58611	-0.13520
H	-5.11952	0.17855	-0.64414
H	-5.21337	-0.33987	1.06304
H	-2.85954	0.25663	1.42737
H	-3.68572	1.65081	0.69195
H	-1.38636	-0.71842	-1.85776
H	-1.46214	-1.22929	-0.15659
H	-3.79143	-1.17596	-2.13908
H	-3.00332	-2.62395	-1.44751
C	2.16129	-2.19362	1.01883
H	3.11772	-2.70894	1.16896
H	1.71449	-2.05044	2.00761
C	1.23246	-3.08687	0.18065
H	1.07605	-4.05715	0.66648
H	0.25596	-2.61001	0.05121
H	1.65671	-3.26578	-0.81389
C	3.49659	-0.47385	-0.35979
C	4.73682	-1.20984	-0.74658
H	5.64206	-0.66194	-0.45556
H	4.76522	-2.18658	-0.25739
H	4.79096	-1.37893	-1.82983

4s_Senantiomer_conformer-047

C	-2.27068	-0.98754	-0.37483
N	-3.23466	0.57137	0.91317
H	-3.90047	1.01758	1.52938
C	-2.15721	1.18498	0.35750
C	-1.52357	0.24612	-0.45537
C	-0.70687	3.00236	-0.49938
C	-1.73493	2.59997	0.57818
H	-0.19537	3.92244	-0.19507
H	-1.24024	3.22649	-1.43050
H	-2.59610	3.27822	0.54659
H	-1.30099	2.70930	1.58192
C	-0.33973	0.58995	-1.21633
O	0.12708	-0.10040	-2.12748
C	0.33367	1.89959	-0.79104
H	0.95749	2.22233	-1.63036
C	1.22597	1.64320	0.44338
H	1.86368	2.53456	0.60538
H	0.59041	1.54295	1.32894

C	3.38858	-1.22811	1.52648
C	2.64762	0.09915	1.63390
N	2.01255	0.41807	0.35630
C	3.01543	0.44951	-0.70902
C	3.75856	-0.88019	-0.75605
O	4.36929	-1.18926	0.49415
H	2.66585	-2.03554	1.32657
H	3.91983	-1.45137	2.45597
H	3.36121	0.88763	1.94368
H	1.87490	0.02571	2.40801
H	2.51393	0.60430	-1.66691
H	3.74096	1.27189	-0.55397
H	3.05395	-1.67988	-1.03500
H	4.56292	-0.84563	-1.49614
C	-1.88677	-2.28319	-1.02825
H	-2.72465	-2.98892	-0.98183
H	-1.67454	-2.10581	-2.08802
C	-0.64511	-2.91525	-0.37552
H	-0.83805	-3.15015	0.67762
H	0.20145	-2.22287	-0.41962
H	-0.35975	-3.84239	-0.88649
C	-3.32084	-0.75607	0.48701
C	-4.41409	-1.64420	0.98299
H	-5.40613	-1.22634	0.76911
H	-4.35405	-2.62337	0.50163
H	-4.35275	-1.80152	2.06762

4s_Senantiomer_conformer-048

C	2.26396	-0.88088	-0.03851
N	3.08653	0.99505	-0.94713
H	3.70761	1.61751	-1.44630
C	1.95528	1.37927	-0.30336
C	1.40442	0.23777	0.28059
C	0.36500	2.83165	0.90557
C	1.40714	2.76556	-0.22851
H	-0.21089	3.76064	0.82490
H	0.89210	2.86402	1.86640
H	2.20649	3.49475	-0.05019
H	0.94978	3.04412	-1.18800
C	0.16821	0.30427	1.03150
O	-0.28414	-0.62804	1.70775
C	-0.59710	1.62412	0.91074
H	-1.23892	1.69023	1.79545
C	-1.50672	1.66745	-0.35397
H	-2.03646	2.63008	-0.31395

H	-0.87900	1.68579	-1.25116
C	-4.52579	-0.52434	0.15423
C	-3.36820	0.36641	0.59416
N	-2.49784	0.62118	-0.55118
C	-2.01415	-0.62808	-1.13941
C	-3.21509	-1.47875	-1.53959
O	-4.05261	-1.74314	-0.41554
H	-5.14621	0.01348	-0.58070
H	-5.14949	-0.80249	1.00876
H	-2.82795	-0.12050	1.42240
H	-3.76281	1.32176	0.96198
H	-1.41689	-0.39460	-2.02851
H	-1.38532	-1.21092	-0.44981
H	-3.79301	-0.95940	-2.32090
H	-2.89261	-2.45133	-1.92262
C	2.06964	-2.30448	0.39694
H	1.01867	-2.58447	0.25983
H	2.65969	-2.96969	-0.24480
C	2.45412	-2.53770	1.86663
H	2.30806	-3.58719	2.14857
H	1.83168	-1.91657	2.51698
H	3.50505	-2.27955	2.04039
C	3.29532	-0.37827	-0.80210
C	4.48333	-1.03542	-1.42400
H	4.51901	-0.87035	-2.50837
H	5.42453	-0.65710	-1.00452
H	4.45381	-2.11385	-1.25070

4s_Senantiomer_conformer-049

C	-3.38947	0.30571	0.28133
N	-3.21777	-1.87445	-0.20854
H	-3.48533	-2.82583	-0.42267
C	-1.94061	-1.41978	-0.15835
C	-1.99608	-0.05976	0.14539
C	0.43625	-1.25114	-0.75117
C	-0.69823	-2.21567	-0.37486
H	0.26168	-0.86606	-1.76485
H	1.39580	-1.77426	-0.76924
H	-0.43147	-2.75979	0.54399
H	-0.84338	-2.97254	-1.15483
C	-0.79123	0.72661	0.31301
O	-0.79027	1.93914	0.55318
C	0.52989	-0.06081	0.21967
H	0.68812	-0.46598	1.23222
C	1.68769	0.88152	-0.13039

H	1.48636	1.32718	-1.11225
H	1.71103	1.71230	0.59451
C	5.30920	0.31551	-0.96044
C	3.98603	1.06182	-0.84090
N	2.98044	0.20230	-0.21490
C	3.46632	-0.24504	1.09136
C	4.80451	-0.95809	0.93392
O	5.77423	-0.11858	0.31468
H	6.08512	0.96706	-1.37183
H	5.18234	-0.55209	-1.62757
H	3.63673	1.35069	-1.83880
H	4.14659	1.99029	-0.25868
H	3.58461	0.60719	1.78901
H	2.74651	-0.94020	1.53472
H	5.20937	-1.23629	1.91087
H	4.66352	-1.87201	0.33482
C	-3.92535	1.67717	0.57480
H	-4.96549	1.60222	0.91380
H	-3.35340	2.12028	1.39755
C	-3.84980	2.61787	-0.63848
H	-4.42220	2.21432	-1.48153
H	-2.80925	2.73952	-0.95348
H	-4.25268	3.60775	-0.39344
C	-4.11987	-0.84057	0.05439
C	-5.59127	-1.09481	0.04133
H	-6.13476	-0.18985	0.32345
H	-5.94430	-1.39801	-0.95288
H	-5.87378	-1.88851	0.74455

4s_Senantiomer_conformer-050

C	-3.24010	0.52903	-0.25368
N	-3.44200	-1.65201	0.22040
H	-3.86675	-2.55470	0.38555
C	-2.10637	-1.41695	0.19049
C	-1.92932	-0.06359	-0.09685
C	0.25969	-1.88739	-0.25460
C	-1.01798	-2.41060	0.41780
H	0.14216	-1.94913	-1.34507
H	1.11949	-2.50651	0.01368
H	-0.84250	-2.54132	1.49649
H	-1.29297	-3.39624	0.02406
C	-0.60762	0.52201	-0.19109
O	-0.40267	1.70939	-0.46833
C	0.56256	-0.42874	0.12730
H	0.67114	-0.38648	1.22394

C	1.86254	0.07523	-0.51478
H	1.76692	-0.01484	-1.60329
H	1.96681	1.14702	-0.30140
C	4.93929	0.60137	-1.01184
C	4.13448	-0.69854	-1.06281
N	3.02347	-0.72451	-0.10434
C	3.53205	-0.38644	1.23279
C	4.35310	0.90521	1.24777
O	5.42859	0.83573	0.30991
H	4.32050	1.45330	-1.33776
H	5.81787	0.54616	-1.66214
H	4.80919	-1.53032	-0.81868
H	3.74419	-0.86823	-2.07214
H	2.69714	-0.31107	1.93689
H	4.17356	-1.21147	1.56894
H	3.71651	1.77446	1.01777
H	4.80764	1.06636	2.23008
C	-3.53714	1.97315	-0.53836
H	-2.88307	2.32502	-1.34351
H	-4.56828	2.07358	-0.89778
C	-3.33479	2.87649	0.68880
H	-3.98154	2.56193	1.51582
H	-2.29500	2.82633	1.02531
H	-3.56773	3.92095	0.44994
C	-4.15487	-0.48095	-0.04796
C	-5.64799	-0.48991	-0.07000
H	-6.02917	0.49425	-0.35294
H	-6.03984	-1.22065	-0.78902
H	-6.06923	-0.73991	0.91233

4s_Senantiomer_conformer-051

C	-2.55409	0.69800	0.20559
N	-3.00451	-1.12028	-1.02463
H	-3.49053	-1.74977	-1.64922
C	-1.86338	-1.41903	-0.35503
C	-1.53910	-0.30900	0.42583
C	-0.18878	-2.78717	0.81867
C	-1.10018	-2.69929	-0.41975
H	0.51454	-3.61959	0.70747
H	-0.80882	-3.00141	1.69729
H	-1.77612	-3.56149	-0.46008
H	-0.49816	-2.73562	-1.33872
C	-0.36652	-0.30140	1.27480
O	-0.11824	0.59509	2.09019
C	0.59841	-1.48180	1.06689

H	1.18637	-1.59093	1.98356
C	1.58516	-1.20178	-0.09955
H	1.79155	-2.14924	-0.61040
H	1.08951	-0.55742	-0.84494
C	3.68305	0.45621	-1.73093
C	3.88336	-0.68974	-0.73509
N	2.88804	-0.69501	0.34664
C	2.86073	0.63971	0.96737
C	2.66854	1.76235	-0.05173
O	3.68085	1.71741	-1.06117
H	2.74059	0.32801	-2.28729
H	4.50309	0.49102	-2.45493
H	4.87469	-0.57889	-0.27597
H	3.85948	-1.65372	-1.25628
H	2.07557	0.67805	1.72463
H	3.82800	0.78786	1.46558
H	1.67323	1.69793	-0.52087
H	2.75306	2.74279	0.42711
C	-2.57917	2.07517	0.80264
H	-3.57090	2.52086	0.66184
H	-2.41027	2.00526	1.88264
C	-1.51219	3.00206	0.19729
H	-0.51220	2.59625	0.37806
H	-1.56050	4.00173	0.64480
H	-1.65213	3.10364	-0.88486
C	-3.44351	0.16675	-0.70272
C	-4.67819	0.72555	-1.32956
H	-4.56868	0.83955	-2.41576
H	-5.54948	0.08174	-1.15459
H	-4.90236	1.71077	-0.91371

4s_Senantiomer_conformer-052

C	-2.49792	-0.65555	0.27174
N	-2.82701	1.44398	0.98269
H	-3.25195	2.23606	1.44589
C	-1.72088	1.50421	0.20031
C	-1.47467	0.21308	-0.26879
C	-0.07142	2.43315	-1.36834
C	-0.91455	2.71977	-0.11224
H	0.66018	3.23434	-1.51964
H	-0.73347	2.42900	-2.24242
H	-1.55955	3.59154	-0.27386
H	-0.26228	2.97153	0.73598
C	-0.33598	-0.07823	-1.11356
O	-0.13579	-1.18206	-1.63554

C	0.66431	1.07813	-1.29020
H	1.19396	0.91073	-2.23325
C	1.71830	1.08174	-0.14877
H	1.95116	2.12219	0.10528
H	1.27120	0.64194	0.75860
C	3.90644	-0.17534	1.70610
C	4.04920	0.70345	0.46013
N	2.99404	0.46843	-0.53592
C	2.93135	-0.97381	-0.82488
C	2.79961	-1.82508	0.43736
O	3.86706	-1.55784	1.35129
H	2.99720	0.09140	2.26858
H	4.76639	-0.05021	2.37147
H	5.01297	0.47584	-0.01439
H	4.05411	1.76287	0.74146
H	2.10008	-1.17805	-1.50239
H	3.86676	-1.24666	-1.33120
H	1.83141	-1.64117	0.93054
H	2.85934	-2.89101	0.19671
C	-2.64361	-2.12695	0.01196
H	-1.66079	-2.60557	0.08630
H	-3.27650	-2.57761	0.78575
C	-3.23529	-2.43028	-1.37378
H	-4.22801	-1.97853	-1.48140
H	-3.33172	-3.51103	-1.53149
H	-2.58447	-2.02595	-2.15454
C	-3.31919	0.13773	1.04269
C	-4.53936	-0.17802	1.84338
H	-4.42260	0.11259	2.89516
H	-5.42608	0.34102	1.45714
H	-4.74465	-1.25080	1.81374

4s_Senantiomer_conformer-053

C	3.25481	-0.40967	0.18157
N	3.13935	1.66651	-0.65391
H	3.42418	2.53660	-1.08319
C	1.89908	1.41025	-0.16906
C	1.91951	0.11901	0.35954
C	-0.26816	1.88754	0.89545
C	0.72870	2.33306	-0.18984
H	-1.20082	2.44846	0.78288
H	0.14687	2.14877	1.87637
H	1.04031	3.36974	-0.01597
H	0.24987	2.31779	-1.17895
C	0.73881	-0.46917	0.95094

O	0.70065	-1.59956	1.44946
C	-0.54867	0.37051	0.87320
H	-1.12705	0.09304	1.76075
C	-1.36630	-0.10245	-0.35992
H	-1.25596	0.62503	-1.18655
H	-0.96089	-1.05198	-0.72467
C	-4.90062	-1.27578	-0.84731
C	-3.44727	-0.97733	-1.19437
N	-2.77630	-0.34645	-0.05545
C	-3.50042	0.86589	0.32931
C	-4.95590	0.52908	0.63490
O	-5.59578	-0.08837	-0.47737
H	-4.94029	-2.00530	-0.02283
H	-5.42615	-1.69094	-1.71169
H	-3.41716	-0.33023	-2.09271
H	-2.92924	-1.91169	-1.43949
H	-3.04603	1.29525	1.22690
H	-3.46070	1.63175	-0.47033
H	-4.99965	-0.13880	1.50996
H	-5.52101	1.43842	0.85704
C	3.72654	-1.78081	0.57054
H	4.82271	-1.80352	0.58602
H	3.38856	-2.00409	1.58839
C	3.20560	-2.87774	-0.37189
H	3.57345	-3.86451	-0.06678
H	2.11201	-2.89922	-0.35336
H	3.53140	-2.69415	-1.40209
C	3.98448	0.57200	-0.45213
C	5.40623	0.61777	-0.90633
H	5.94585	1.46718	-0.46847
H	5.92774	-0.29636	-0.61261
H	5.48322	0.70890	-1.99745

4s_Senantiomer_conformer-054

C	3.36556	-0.27081	-0.22826
N	3.11806	1.89574	0.28966
H	3.34983	2.86064	0.48363
C	1.85872	1.39488	0.24096
C	1.96048	0.04132	-0.07427
C	-0.55246	1.39474	-0.22683
C	0.58996	2.13381	0.48830
H	-0.45211	1.53924	-1.31058
H	-1.50382	1.84177	0.06978
H	0.38132	2.17980	1.56782
H	0.65823	3.17064	0.13871

C	0.78749	-0.80029	-0.18091
O	0.84223	-2.01135	-0.42604
C	-0.57393	-0.11429	0.07678
H	-0.75083	-0.23732	1.15791
C	-1.68082	-0.88689	-0.67941
H	-1.53875	-0.74660	-1.75824
H	-1.51600	-1.94933	-0.47613
C	-5.20488	0.46517	-0.87858
C	-3.69134	0.55281	-1.05112
N	-3.07613	-0.58530	-0.37841
C	-3.43519	-0.62831	1.03174
C	-4.95579	-0.66083	1.16443
O	-5.55732	0.45025	0.50382
H	-5.58132	-0.44562	-1.37070
H	-5.69903	1.33771	-1.31558
H	-3.36135	1.52869	-0.65383
H	-3.43534	0.52211	-2.11614
H	-3.01006	-1.53202	1.48451
H	-3.04736	0.24221	1.59448
H	-5.33905	-1.60101	0.73702
H	-5.25712	-0.59878	2.21407
C	3.95250	-1.61753	-0.53886
H	3.38154	-2.08317	-1.34951
H	4.98046	-1.49692	-0.90127
C	3.94656	-2.56323	0.67288
H	4.38691	-3.53384	0.41537
H	4.52024	-2.13665	1.50363
H	2.92002	-2.73142	1.01132
C	4.05515	0.89933	0.00492
C	5.51538	1.21189	-0.00524
H	6.08897	0.33398	-0.31220
H	5.75205	2.02708	-0.70087
H	5.87542	1.51386	0.98679

4s_Senantiomer_conformer-055

C	2.41543	0.81798	-0.41221
N	3.25973	-0.77849	0.91590
H	3.88586	-1.25632	1.54989
C	2.11467	-1.30270	0.41098
C	1.55102	-0.34089	-0.42498
C	0.04362	-2.60624	0.48545
C	1.57260	-2.67376	0.63013
H	-0.37572	-3.61828	0.49684
H	-0.35929	-2.09407	1.36316
H	1.99383	-3.37150	-0.10878

H	1.84783	-3.05918	1.61845
C	0.36178	-0.62865	-1.19925
O	-0.01591	0.05397	-2.15518
C	-0.42103	-1.89882	-0.80892
H	-0.23195	-2.60013	-1.63580
C	-1.94521	-1.63245	-0.82786
H	-2.29097	-1.65907	-1.86540
H	-2.44091	-2.47346	-0.30403
C	-2.46662	1.31544	1.50392
C	-1.98036	-0.06913	1.08784
N	-2.37825	-0.34190	-0.29096
C	-3.82491	-0.19159	-0.43508
C	-4.25777	1.19897	0.01201
O	-3.87725	1.44652	1.36249
H	-1.95728	2.07782	0.89295
H	-2.23806	1.49900	2.55747
H	-2.38030	-0.82503	1.79261
H	-0.88982	-0.07956	1.16242
H	-4.09530	-0.33660	-1.48754
H	-4.37477	-0.94891	0.15859
H	-3.80437	1.95504	-0.64863
H	-5.34599	1.29809	-0.03118
C	2.15941	2.11304	-1.12666
H	3.05032	2.74943	-1.07038
H	1.97640	1.91176	-2.18808
C	0.94962	2.87346	-0.55782
H	0.79822	3.82172	-1.08674
H	0.04031	2.27450	-0.66640
H	1.09200	3.09370	0.50632
C	3.45910	0.51947	0.43719
C	4.63849	1.31867	0.88463
H	4.61407	1.51318	1.96469
H	5.58369	0.80543	0.66629
H	4.65815	2.28438	0.37391

4s_Senantiomer_conformer-056

C	-2.45651	-0.78269	0.11692
N	-3.11257	1.20064	0.92915
H	-3.66337	1.89503	1.41573
C	-1.95787	1.45213	0.26296
C	-1.51005	0.24207	-0.26496
C	0.20570	2.53756	-0.10677
C	-1.30244	2.77576	0.06543
H	0.62120	2.25664	0.86450
H	0.69817	3.47136	-0.39937

H	-1.71096	3.27171	-0.82780
H	-1.49257	3.44592	0.91150
C	-0.32208	0.17606	-1.09170
O	-0.00564	-0.81145	-1.76059
C	0.53054	1.45990	-1.16602
H	0.26443	1.89892	-2.14072
C	2.04008	1.14212	-1.25974
H	2.26760	0.83097	-2.28384
H	2.58973	2.08824	-1.08523
C	2.80068	-0.96691	1.80054
C	2.25382	0.23047	1.02990
N	2.53841	0.07359	-0.39322
C	3.97277	-0.11449	-0.60135
C	4.47445	-1.30314	0.20890
O	4.20012	-1.13210	1.59655
H	2.26651	-1.87631	1.48183
H	2.65394	-0.82945	2.87542
H	2.69042	1.16168	1.44273
H	1.17190	0.26670	1.18485
H	4.15769	-0.29554	-1.66676
H	4.54778	0.78789	-0.31176
H	3.99606	-2.22611	-0.15571
H	5.55903	-1.40582	0.11340
C	-2.39075	-2.23754	-0.24897
H	-1.35468	-2.58386	-0.16502
H	-2.98132	-2.82560	0.46399
C	-2.88871	-2.51461	-1.67649
H	-2.83040	-3.58389	-1.91247
H	-2.27285	-1.96861	-2.39683
H	-3.93004	-2.19410	-1.79564
C	-3.43393	-0.15703	0.86070
C	-4.65598	-0.68467	1.53735
H	-4.65366	-0.46512	2.61269
H	-4.71612	-1.76917	1.41764
H	-5.57358	-0.25262	1.11758

4s_Senantiomer_conformer-057

C	2.62453	0.69067	0.18230
N	3.22913	-1.43560	0.55266
H	3.78357	-2.23124	0.83948
C	1.97542	-1.50741	0.04115
C	1.55527	-0.20120	-0.21155
C	-0.30518	-2.37611	-0.19927
C	1.18655	-2.74257	-0.23100
H	-0.60061	-2.16744	0.83336

H	-0.90840	-3.22745	-0.53331
H	1.45224	-3.15112	-1.21778
H	1.41006	-3.52589	0.50276
C	0.26199	0.07527	-0.79669
O	-0.11363	1.20898	-1.11952
C	-0.62997	-1.15468	-1.07777
H	-0.38487	-1.41889	-2.12002
C	-2.12682	-0.80550	-1.08014
H	-2.24491	0.15079	-1.60932
H	-2.65207	-1.56635	-1.66839
C	-4.68932	0.70705	-0.07645
C	-4.20581	-0.71994	0.19764
N	-2.74245	-0.82684	0.25356
C	-2.23506	0.17052	1.20673
C	-2.73807	1.58399	0.91541
O	-4.16933	1.61344	0.89665
H	-5.77991	0.76982	-0.00717
H	-4.38973	1.03070	-1.08613
H	-4.59006	-1.40536	-0.56632
H	-4.60557	-1.03918	1.16969
H	-2.58653	-0.12422	2.20472
H	-1.14268	0.14875	1.22823
H	-2.42702	2.27888	1.70190
H	-2.33718	1.94112	-0.04359
C	2.62381	2.18786	0.06799
H	1.65422	2.57416	0.40087
H	3.38233	2.60954	0.73839
C	2.88218	2.67646	-1.36629
H	2.87975	3.77189	-1.41401
H	3.85233	2.32012	-1.73101
H	2.10210	2.30209	-2.03558
C	3.64629	-0.10544	0.65182
C	4.99536	0.22935	1.19731
H	5.80072	-0.17025	0.56759
H	5.12369	1.31314	1.25074
H	5.13844	-0.17580	2.20716

4s_Senantiomer_conformer-058

C	3.28714	0.41631	-0.22959
N	3.27287	-1.80908	0.03358
H	3.60797	-2.75652	0.14473
C	1.96768	-1.44078	0.04987
C	1.92434	-0.05646	-0.11153
C	-0.40168	-1.49425	0.68206
C	0.78836	-2.33931	0.20467

H	-0.23505	-1.19231	1.72411
H	-1.31318	-2.10038	0.67092
H	0.54189	-2.81335	-0.75757
H	0.99825	-3.15242	0.90963
C	0.66740	0.66017	-0.17695
O	0.58788	1.88803	-0.28124
C	-0.60186	-0.22705	-0.17393
H	-0.72014	-0.53382	-1.22538
C	-1.85533	0.57505	0.21535
H	-2.15734	0.29067	1.23792
H	-1.58485	1.63353	0.24643
C	-4.94107	1.05033	0.60559
C	-4.01737	1.42343	-0.55654
N	-2.93782	0.45027	-0.77112
C	-3.52704	-0.89223	-0.87720
C	-4.45347	-1.23379	0.29141
O	-5.48810	-0.25620	0.41663
H	-5.79195	1.73601	0.66463
H	-4.39903	1.09230	1.56384
H	-3.58062	2.41488	-0.39250
H	-4.61912	1.46638	-1.47442
H	-4.11599	-0.92465	-1.80328
H	-2.73358	-1.63947	-0.97038
H	-4.95228	-2.19371	0.12641
H	-3.88747	-1.29959	1.23453
C	3.72565	1.84354	-0.38788
H	4.75854	1.87297	-0.75485
H	3.10438	2.33081	-1.14729
C	3.62522	2.64645	0.91900
H	4.24549	2.19607	1.70234
H	3.95803	3.68058	0.77072
H	2.58853	2.66667	1.26782
C	4.09752	-0.69424	-0.13442
C	5.58248	-0.84767	-0.17199
H	5.90025	-1.54152	-0.96063
H	6.05859	0.11687	-0.36396
H	5.97724	-1.22987	0.77830

4s_Senantiomer_conformer-059

C	-3.39087	0.34382	-0.19553
N	-3.21763	-1.87964	0.02439
H	-3.48260	-2.85365	0.08325
C	-1.94105	-1.42121	0.03177
C	-1.99741	-0.03371	-0.09759
C	0.46757	-1.43365	-0.44235

C	-0.69872	-2.23661	0.15243
H	1.41918	-1.93110	-0.23865
H	0.35595	-1.38963	-1.53410
H	-0.49250	-2.45942	1.21040
H	-0.80783	-3.20254	-0.35472
C	-0.79406	0.77239	-0.11714
O	-0.79211	1.99978	-0.26371
C	0.52254	0.00285	0.10640
H	0.62671	-0.06046	1.20174
C	1.70530	0.79430	-0.46546
H	1.55317	0.91112	-1.54544
H	1.70507	1.80975	-0.03513
C	4.74927	-0.61727	1.25873
C	3.41467	0.10747	1.12622
N	2.99315	0.12723	-0.27527
C	4.03583	0.75227	-1.08989
C	5.35333	0.00760	-0.91168
O	5.75601	-0.00953	0.45514
H	4.62276	-1.67194	0.96593
H	5.10724	-0.57878	2.29127
H	3.51262	1.13399	1.53078
H	2.66726	-0.41764	1.72926
H	3.73448	0.71715	-2.14308
H	4.18255	1.81570	-0.81704
H	5.24483	-1.02414	-1.28263
H	6.15503	0.50073	-1.46837
C	-3.92890	1.74073	-0.31207
H	-3.35466	2.28777	-1.06761
H	-4.96768	1.70836	-0.66183
C	-3.86075	2.51582	1.01361
H	-4.26426	3.52869	0.89767
H	-4.43622	2.00505	1.79407
H	-2.82228	2.59731	1.34812
C	-4.12038	-0.82245	-0.11334
C	-5.59096	-1.08003	-0.14630
H	-5.95217	-1.50570	0.79881
H	-6.13540	-0.14859	-0.31933
H	-5.86347	-1.78103	-0.94558

4s_Senantiomer_conformer-060

C	-3.28969	0.41605	-0.23477
N	-3.27967	-1.80355	0.07383
H	-3.61558	-2.75211	0.17235
C	-1.97278	-1.44145	0.05334
C	-1.92702	-0.06004	-0.13089

C	0.42361	-1.64139	-0.42871
C	-0.79415	-2.34379	0.18962
H	0.30825	-1.61222	-1.51989
H	1.32893	-2.22109	-0.22216
H	-0.59710	-2.55573	1.25150
H	-0.97604	-3.31046	-0.29453
C	-0.66887	0.65498	-0.19167
O	-0.58482	1.86890	-0.40169
C	0.59200	-0.19906	0.09059
H	0.65458	-0.23284	1.18999
C	1.87022	0.48634	-0.42127
H	2.22226	-0.04248	-1.32355
H	1.61029	1.50218	-0.72926
C	4.46416	-1.25908	0.07957
C	3.47777	-0.65404	1.08032
N	2.89880	0.61641	0.62056
C	3.99067	1.51983	0.23371
C	4.97406	0.88597	-0.75335
O	5.50653	-0.33032	-0.22505
H	3.94888	-1.55755	-0.84752
H	4.95111	-2.14508	0.49834
H	4.01644	-0.45488	2.01613
H	2.67839	-1.36450	1.31031
H	3.56603	2.43756	-0.18829
H	4.54265	1.78983	1.14415
H	4.48375	0.68923	-1.72018
H	5.82887	1.54550	-0.93218
C	-3.72517	1.84274	-0.40609
H	-3.12227	2.31311	-1.19030
H	-4.76775	1.87151	-0.74460
C	-3.58523	2.66750	0.88355
H	-2.53957	2.68998	1.20474
H	-3.91778	3.70043	0.72671
H	-4.18545	2.23331	1.69125
C	-4.10276	-0.68841	-0.09978
C	-5.58866	-0.83680	-0.11052
H	-6.06421	0.12259	-0.32808
H	-5.91992	-1.55532	-0.87106
H	-5.97130	-1.18561	0.85737

4s_Senantiomer_conformer-061

C	3.10926	0.44979	-0.06511
N	2.92201	-1.70570	0.51784
H	3.16648	-2.61966	0.87469
C	1.73715	-1.39181	-0.06306

C	1.80384	-0.04920	-0.43982
C	-0.30628	-1.74069	-1.38998
C	0.57510	-2.30540	-0.26244
H	-1.25643	-2.27999	-1.42345
H	0.20343	-1.89756	-2.34820
H	0.90941	-3.32055	-0.50666
H	-0.00775	-2.38750	0.66639
C	0.68270	0.60764	-1.07764
O	0.68082	1.78975	-1.44075
C	-0.59021	-0.23882	-1.22170
H	-1.09710	0.13354	-2.11874
C	-1.48193	0.06691	0.00628
H	-0.94065	-0.21381	0.91626
H	-1.62441	1.15820	0.05494
C	-4.07251	0.40099	1.79350
C	-3.35009	-0.85799	1.31077
N	-2.73278	-0.69222	-0.01070
C	-3.73504	-0.17746	-0.95469
C	-4.45455	1.06790	-0.43198
O	-5.05317	0.81269	0.83988
H	-3.35326	1.21811	1.96649
H	-4.60917	0.21214	2.72838
H	-4.08456	-1.67144	1.23892
H	-2.58303	-1.16093	2.03180
H	-3.26014	0.03427	-1.91760
H	-4.47920	-0.96733	-1.11957
H	-3.75622	1.91610	-0.34976
H	-5.26676	1.35956	-1.10470
C	3.60853	1.85433	-0.24443
H	4.70051	1.87357	-0.14652
H	3.37390	2.19580	-1.25866
C	2.98802	2.83694	0.76183
H	3.37494	3.85151	0.60979
H	3.21326	2.53497	1.79102
H	1.90125	2.86274	0.63920
C	3.77613	-0.60002	0.52787
C	5.14373	-0.70358	1.11851
H	5.68652	0.23535	0.98540
H	5.10846	-0.91787	2.19448
H	5.73169	-1.50058	0.64565

4s_Senantiomer_conformer-062

C	-3.24854	0.20088	0.05752
N	-2.64846	-1.95741	0.00370
H	-2.71705	-2.96200	-0.08890

C	-1.52636	-1.28622	0.36564
C	-1.85089	0.07106	0.40740
C	0.60116	-0.87274	1.51650
C	-0.19272	-1.88158	0.66990
H	1.63311	-1.21016	1.63957
H	0.15912	-0.84087	2.52019
H	-0.29763	-2.83272	1.20483
H	0.34568	-2.10923	-0.26108
C	-0.86047	1.07270	0.74444
O	-1.09099	2.28641	0.80681
C	0.56985	0.55164	0.93426
H	1.05227	1.23450	1.64193
C	1.27860	0.72735	-0.44462
H	0.75496	0.13635	-1.20467
H	1.14758	1.78247	-0.71848
C	4.53366	-1.03238	-1.19967
C	3.06338	-0.97637	-0.79486
N	2.69609	0.41297	-0.54359
C	3.54662	1.02276	0.47015
C	5.00707	0.89739	0.04480
O	5.36214	-0.46364	-0.18790
H	4.67623	-0.49090	-2.14821
H	4.86275	-2.06761	-1.32665
H	2.92423	-1.63196	0.08236
H	2.44312	-1.36878	-1.60846
H	3.28429	2.08270	0.56881
H	3.42328	0.55350	1.46377
H	5.17367	1.48799	-0.86994
H	5.67384	1.26327	0.83068
C	-4.01691	1.48485	-0.06522
H	-5.09302	1.27458	-0.05101
H	-3.80419	2.11837	0.80278
C	-3.66526	2.26599	-1.34163
H	-4.24519	3.19427	-1.40621
H	-2.60249	2.52535	-1.34125
H	-3.87556	1.66771	-2.23548
C	-3.71228	-1.07285	-0.18978
C	-5.05607	-1.57976	-0.59872
H	-5.03099	-2.04838	-1.59100
H	-5.44040	-2.32777	0.10637
H	-5.77515	-0.75804	-0.63857

4s_Senantiomer_conformer-063

C	2.72054	-0.48971	-0.35007
N	2.86572	1.72074	-0.69119

H	3.21443	2.61289	-1.01513
C	1.71070	1.54192	-0.00353
C	1.57749	0.17478	0.23778
C	-0.62804	1.94379	0.59074
C	0.75370	2.59739	0.43472
H	-0.99450	1.67767	-0.40580
H	-1.33795	2.66146	1.01574
H	1.07454	3.03030	1.39433
H	0.71473	3.42258	-0.28583
C	0.46191	-0.35279	0.99505
O	0.35681	-1.54359	1.31323
C	-0.58058	0.68249	1.47134
H	-0.20650	0.99069	2.46329
C	-1.96378	0.04172	1.74576
H	-1.77248	-0.89240	2.28456
H	-2.51924	0.70258	2.42027
C	-3.41524	-1.58915	-1.33390
C	-2.28846	-1.07283	-0.44423
N	-2.85107	-0.26176	0.62766
C	-3.67230	0.82580	0.10611
C	-4.77103	0.23813	-0.77402
O	-4.21228	-0.52209	-1.84367
H	-4.04809	-2.28128	-0.75547
H	-3.01268	-2.11879	-2.20226
H	-1.57017	-0.50751	-1.06898
H	-1.74490	-1.91516	-0.00813
H	-4.12181	1.36962	0.94481
H	-3.10383	1.55275	-0.49882
H	-5.43053	-0.40126	-0.16566
H	-5.36795	1.03106	-1.23406
C	3.01231	-1.96223	-0.31491
H	2.09181	-2.51740	-0.52588
H	3.72622	-2.21583	-1.10780
C	3.56833	-2.42772	1.04034
H	3.77686	-3.50416	1.03030
H	4.49862	-1.90101	1.28200
H	2.84063	-2.22627	1.83180
C	3.49835	0.49591	-0.91821
C	4.78870	0.43517	-1.66727
H	5.10200	-0.60335	-1.79881
H	4.70281	0.88852	-2.66303
H	5.59355	0.96068	-1.13722

4s_Senantiomer_conformer-064

C	-2.87549	0.73266	-0.28395

N	-3.64491	-1.32210	0.17358
H	-4.29256	-2.08236	0.33221
C	-2.29452	-1.44511	0.15022
C	-1.76700	-0.18439	-0.12649
C	-0.12083	-2.51409	-0.26306
C	-1.50646	-2.68966	0.37712
H	-0.21853	-2.55728	-1.35582
H	0.53126	-3.34154	0.03599
H	-1.39183	-2.87342	1.45616
H	-2.01918	-3.56572	-0.03705
C	-0.33897	0.03314	-0.22021
O	0.16405	1.12250	-0.52057
C	0.54628	-1.17958	0.13036
H	0.63485	-1.17386	1.22821
C	1.97089	-1.06332	-0.44654
H	2.26514	-2.03690	-0.85370
H	1.95961	-0.35743	-1.28932
C	4.86007	0.14288	-0.80387
C	4.34902	-0.95363	0.13381
N	2.96818	-0.73419	0.58307
C	2.85725	0.62678	1.13157
C	3.39563	1.69866	0.18402
O	4.75105	1.42450	-0.18496
H	4.29775	0.13598	-1.75170
H	5.92030	-0.00236	-1.03449
H	4.99278	-0.97088	1.02377
H	4.41994	-1.93379	-0.35162
H	1.81442	0.84167	1.37434
H	3.43866	0.65626	2.06297
H	2.76359	1.76047	-0.71299
H	3.40186	2.67949	0.67021
C	-2.78033	2.20594	-0.55726
H	-2.07047	2.37759	-1.37377
H	-3.75312	2.58139	-0.89638
C	-2.32248	3.01071	0.66980
H	-2.27171	4.08152	0.43972
H	-1.32825	2.68161	0.98616
H	-3.01558	2.87287	1.50748
C	-4.02410	-0.00295	-0.08786
C	-5.46725	0.38005	-0.11293
H	-5.57620	1.43109	-0.39085
H	-5.94265	0.24388	0.86700
H	-6.03412	-0.21869	-0.83723

4s_Senantiomer_conformer-065

C	2.67541	0.69077	-0.21800
N	3.11199	-1.22160	0.86390
H	3.60331	-1.91108	1.41657
C	1.92461	-1.42996	0.23768
C	1.60933	-0.25737	-0.44828
C	0.08822	-2.65528	-0.87890
C	1.11923	-2.68666	0.26736
H	-0.66386	-3.43652	-0.72100
H	0.59979	-2.88938	-1.81974
H	1.76339	-3.56877	0.16976
H	0.61198	-2.78751	1.23696
C	0.40577	-0.15646	-1.24922
O	0.17530	0.75864	-2.04540
C	-0.60804	-1.28582	-1.03198
H	-1.23925	-1.31730	-1.92578
C	-1.48684	-0.98298	0.20134
H	-2.28965	-1.74049	0.22849
H	-0.89061	-1.13162	1.10750
C	-4.27953	0.22196	-0.66503
C	-2.87123	0.79117	-0.83377
N	-1.96678	0.39895	0.25675
C	-2.59395	0.73455	1.54113
C	-4.01262	0.17485	1.67719
O	-4.83493	0.61133	0.59388
H	-4.27052	-0.87732	-0.74328
H	-4.95404	0.61188	-1.43353
H	-2.93890	1.88692	-0.82459
H	-2.43635	0.50167	-1.79252
H	-1.96080	0.37210	2.35893
H	-2.64540	1.82902	1.61369
H	-3.99508	-0.92642	1.71153
H	-4.48928	0.53687	2.59341
C	2.71496	2.10601	-0.71702
H	3.72881	2.51018	-0.61169
H	2.47300	2.11925	-1.78501
C	1.72113	3.01624	0.02388
H	1.95082	3.05219	1.09496
H	1.75614	4.03934	-0.36869
H	0.70079	2.63890	-0.09348
C	3.58714	0.06586	0.60511
C	4.87301	0.53557	1.20166
H	4.82170	0.58525	2.29704
H	5.70751	-0.12875	0.94345
H	5.11739	1.53576	0.83564

4s_Senantiomer_conformer-066

C	2.40339	-0.85181	0.38957
N	3.29006	0.84202	-0.77885
H	3.95462	1.37979	-1.31882
C	2.10098	1.31000	-0.32232
C	1.50388	0.28089	0.40762
C	0.46322	2.94266	0.52960
C	1.54923	2.68060	-0.53186
H	-0.09507	3.85027	0.27320
H	0.95267	3.12848	1.49294
H	2.33993	3.43677	-0.46002
H	1.12906	2.77261	-1.54300
C	0.21853	0.46502	1.04937
O	-0.29240	-0.35608	1.82120
C	-0.51651	1.76070	0.69157
H	-1.18570	1.97445	1.53153
C	-1.38659	1.61906	-0.59394
H	-1.85528	2.60125	-0.75063
H	-0.73543	1.43553	-1.45538
C	-4.55325	-0.25821	0.19457
C	-3.34817	0.62286	0.50944
N	-2.43876	0.61548	-0.63332
C	-2.02597	-0.74689	-0.96818
C	-3.26986	-1.58031	-1.25670
O	-4.15151	-1.58625	-0.13525
H	-5.11939	0.17837	-0.64391
H	-5.21300	-0.34021	1.06324
H	-2.85916	0.25650	1.42735
H	-3.68559	1.65065	0.69214
H	-1.38621	-0.71818	-1.85794
H	-1.46188	-1.22917	-0.15681
H	-3.79131	-1.17587	-2.13912
H	-3.00301	-2.62386	-1.44777
C	2.16087	-2.19361	1.01897
H	3.11723	-2.70895	1.16942
H	1.71376	-2.05031	2.00759
C	1.23226	-3.08691	0.18060
H	1.07577	-4.05718	0.66644
H	0.25576	-2.61011	0.05092
H	1.65674	-3.26584	-0.81384
C	3.49642	-0.47402	-0.35970
C	4.73659	-1.21010	-0.74651
H	5.64188	-0.66230	-0.45544
H	4.76486	-2.18684	-0.25734
H	4.79075	-1.37916	-1.82977

4s_Senantiomer_conformer-067

C	3.15229	0.25350	-0.04125
N	2.70923	-1.86492	0.54003
H	2.84399	-2.80222	0.89477
C	1.57123	-1.41186	-0.04238
C	1.79733	-0.08705	-0.41792
C	-0.50070	-1.50266	-1.36870
C	0.31353	-2.18379	-0.25365
H	-1.49526	-1.95628	-1.41756
H	-0.01149	-1.70476	-2.32910
H	0.52980	-3.22383	-0.52506
H	-0.26989	-2.22523	0.67690
C	0.76814	0.69794	-1.06246
O	0.92104	1.86257	-1.44677
C	-0.61045	0.02680	-1.19156
H	-1.06749	0.46784	-2.08345
C	-1.47414	0.46269	0.02420
H	-1.48085	-0.34762	0.77134
H	-1.00284	1.32149	0.51367
C	-4.21699	-1.11906	0.02493
C	-3.67019	-0.10777	-0.98408
N	-2.81553	0.91547	-0.36542
C	-3.54877	1.52638	0.75280
C	-4.10626	0.49629	1.73873
O	-4.93499	-0.45294	1.06522
H	-3.40205	-1.71988	0.46027
H	-4.92575	-1.80271	-0.45214
H	-4.52084	0.41011	-1.44583
H	-3.12578	-0.61372	-1.78545
H	-2.89199	2.23356	1.27181
H	-4.38996	2.09282	0.33189
H	-3.28923	-0.02689	2.26073
H	-4.73734	0.97861	2.49139
C	3.81543	1.58821	-0.22234
H	4.90072	1.47921	-0.11021
H	3.63461	1.94609	-1.24188
C	3.30297	2.64638	0.76799
H	3.81208	3.60537	0.61545
H	3.47475	2.32818	1.80259
H	2.22934	2.80285	0.62761
C	3.68801	-0.86775	0.55344
C	5.03073	-1.13138	1.15113
H	5.52060	-1.99551	0.68458
H	5.68255	-0.26486	1.01662
H	4.96514	-1.33476	2.22777

4s_Senantiomer_conformer-068

C	3.08203	0.19359	0.40950
N	2.62205	-1.98468	0.15432
H	2.73718	-2.98920	0.16477
C	1.52277	-1.33464	-0.30196
C	1.76235	0.03295	-0.16249
C	-0.46810	-0.90920	-1.68440
C	0.28403	-1.95943	-0.84715
H	-1.45457	-1.29992	-1.95224
H	0.07880	-0.75614	-2.62249
H	0.52220	-2.83482	-1.46287
H	-0.35145	-2.32479	-0.02814
C	0.76070	1.01205	-0.52161
O	0.92005	2.23437	-0.43156
C	-0.60108	0.45298	-0.97071
H	-1.00234	1.19457	-1.66923
C	-1.54892	0.43191	0.25995
H	-1.60829	-0.59601	0.65336
H	-1.11546	1.03845	1.06197
C	-4.28082	-0.97621	-0.49146
C	-3.66811	0.31896	-1.02709
N	-2.86016	1.02870	-0.02504
C	-3.67022	1.21019	1.18829
C	-4.29164	-0.09394	1.69507
O	-5.06997	-0.71579	0.67088
H	-3.49621	-1.71236	-0.25229
H	-4.95371	-1.42418	-1.22888
H	-4.48588	0.98835	-1.32390
H	-3.06843	0.12486	-1.92012
H	-3.05248	1.66663	1.96985
H	-4.48176	1.90952	0.94748
H	-3.51107	-0.78899	2.04325
H	-4.97401	0.09720	2.52889
C	3.76926	1.49490	0.70597
H	3.07126	2.16201	1.22381
H	4.60990	1.32131	1.38825
C	4.27615	2.20224	-0.56106
H	4.77780	3.14436	-0.31028
H	3.43654	2.42713	-1.22528
H	4.98757	1.56912	-1.10346
C	3.58772	-1.07473	0.59247
C	4.89504	-1.55651	1.12978
H	5.49822	-0.71261	1.47308
H	4.75943	-2.23936	1.97808

H	5.47598	-2.09260	0.36818

4s_Senantiomer_conformer-069

C	-3.37957	0.28628	0.26491
N	-3.16306	-1.90057	-0.17445
H	-3.41097	-2.86176	-0.36714
C	-1.89524	-1.42007	-0.12842
C	-1.97862	-0.05486	0.14413
C	0.47951	-1.21706	-0.71666
C	-0.63648	-2.19588	-0.32261
H	0.29944	-0.85621	-1.73818
H	1.44903	-1.72192	-0.72289
H	-0.36196	-2.71337	0.60924
H	-0.76368	-2.97303	-1.08559
C	-0.78958	0.75813	0.29917
O	-0.81293	1.97594	0.50995
C	0.54596	-0.00621	0.23080
H	0.70914	-0.38846	1.25162
C	1.69253	0.94710	-0.12746
H	1.49933	1.37068	-1.12077
H	1.69227	1.78939	0.58549
C	5.25517	0.21787	-1.05211
C	4.03601	1.10380	-0.75100
N	2.98624	0.27494	-0.17227
C	3.39816	-0.24200	1.14056
C	4.92418	-0.42875	1.18720
O	5.42249	-0.82154	-0.08323
H	6.16391	0.83727	-1.10514
H	5.12604	-0.28981	-2.01303
H	3.67314	1.54216	-1.68615
H	4.31491	1.94849	-0.09284
H	3.10246	0.44031	1.96005
H	2.89572	-1.20008	1.31688
H	5.41798	0.49573	1.52218
H	5.19186	-1.22053	1.89151
C	-3.94363	1.65331	0.52421
H	-4.98415	1.56577	0.85899
H	-3.38541	2.12607	1.33988
C	-3.87887	2.56767	-0.70965
H	-2.83885	2.70317	-1.02072
H	-4.43748	2.13378	-1.54685
H	-4.30310	3.55450	-0.48960
C	-4.08643	-0.87891	0.06058
C	-5.55245	-1.16274	0.04609
H	-5.89367	-1.49709	-0.94224

H	-5.82311	-1.94463	0.76701
H	-6.11548	-0.26228	0.30317

4s_Senantiomer_conformer-070

C	3.25658	0.21159	-0.02769
N	2.68785	-1.95246	0.09269
H	2.76707	-2.95100	0.23084
C	1.57097	-1.31653	-0.34094
C	1.87639	0.04302	-0.42736
C	-0.52232	-1.00036	-1.59483
C	0.25816	-1.94603	-0.66597
H	-1.55301	-1.35051	-1.69529
H	-0.06241	-1.02241	-2.59011
H	0.39672	-2.92466	-1.14034
H	-0.31703	-2.12569	0.25366
C	0.88591	1.00974	-0.85159
O	1.09905	2.22149	-0.97095
C	-0.52617	0.45189	-1.08636
H	-0.98143	1.09865	-1.84355
C	-1.30207	0.61520	0.24061
H	-0.75160	0.10559	1.03967
H	-1.32149	1.68920	0.50394
C	-4.91803	0.10501	-0.69042
C	-3.55387	0.78504	-0.66763
N	-2.64241	0.04047	0.20216
C	-3.21759	-0.06753	1.54360
C	-4.58595	-0.73407	1.46753
O	-5.46832	-0.00513	0.61884
H	-4.81955	-0.89585	-1.14050
H	-5.62950	0.68875	-1.28088
H	-3.67043	1.83152	-0.32473
H	-3.15266	0.81038	-1.68544
H	-2.55001	-0.67019	2.16992
H	-3.32516	0.92526	2.02185
H	-4.47158	-1.76385	1.09298
H	-5.05589	-0.76682	2.45434
C	3.99988	1.51236	0.06988
H	5.07825	1.31915	0.11553
H	3.81982	2.10135	-0.83613
C	3.57807	2.34655	1.29035
H	4.14315	3.28491	1.33863
H	2.51351	2.59095	1.22833
H	3.75280	1.79320	2.22009
C	3.72963	-1.04268	0.29080
C	5.06425	-1.51007	0.77075

H	5.76591	-0.67359	0.81390
H	5.00626	-1.94799	1.77559
H	5.49146	-2.27200	0.10632

4s_Senantiomer_conformer-071

C	-3.10921	0.44990	0.06513
N	-2.92221	-1.70559	-0.51792
H	-3.16680	-2.61951	-0.87477
C	-1.73731	-1.39186	0.06298
C	-1.80384	-0.04925	0.43979
C	0.30617	-1.74106	1.38977
C	-0.57535	-2.30558	0.26223
H	1.25629	-2.28042	1.42308
H	-0.20347	-1.89803	2.34801
H	-0.90975	-3.32072	0.50640
H	0.00742	-2.38770	-0.66665
C	-0.68260	0.60742	1.07762
O	-0.68056	1.78951	1.44077
C	0.59021	-0.23919	1.22167
H	1.09706	0.13303	2.11879
C	1.48206	0.06659	-0.00621
H	0.94094	-0.21431	-0.91623
H	1.62438	1.15789	-0.05496
C	4.07242	0.40069	-1.79356
C	3.35041	-0.85835	-1.31037
N	2.73305	-0.69231	0.01105
C	3.73521	-0.17696	0.95483
C	4.45436	1.06843	0.43171
O	5.05297	0.81305	-0.84012
H	3.35290	1.21751	-1.96684
H	4.60911	0.21167	-2.72840
H	4.08514	-1.67153	-1.23822
H	2.58345	-1.16180	-2.03129
H	3.26029	0.03497	1.91769
H	4.47958	-0.96658	1.11994
H	3.75581	1.91644	0.34932
H	5.26654	1.36048	1.10430
C	-3.60839	1.85446	0.24456
H	-4.70038	1.87377	0.14679
H	-3.37361	2.19585	1.25878
C	-2.98799	2.83710	-0.76173
H	-3.37484	3.85168	-0.60955
H	-3.21339	2.53523	-1.79091
H	-1.90120	2.86285	-0.63924
C	-3.77621	-0.59982	-0.52788

C	-5.14384	-0.70319	-1.11848
H	-5.73190	-1.50011	-0.64562
H	-5.68651	0.23580	-0.98535
H	-5.10862	-0.91749	-2.19447

4s_Senantiomer_conformer-072

C	-3.38007	0.31377	-0.20681
N	-3.17034	-1.89950	0.07510
H	-3.41935	-2.87576	0.16061
C	-1.90126	-1.42052	0.06569
C	-1.98050	-0.03827	-0.10231
C	0.50496	-1.40618	-0.41796
C	-0.64520	-2.21199	0.20393
H	0.38645	-1.39231	-1.50970
H	1.46556	-1.88340	-0.20731
H	-0.43099	-2.40174	1.26673
H	-0.74033	-3.19332	-0.27574
C	-0.79038	0.78641	-0.14788
O	-0.80914	2.00902	-0.32960
C	0.53841	0.04474	0.09347
H	0.65008	0.01150	1.18954
C	1.71053	0.83806	-0.49733
H	1.57055	0.91373	-1.58275
H	1.68305	1.86657	-0.09849
C	4.86215	-0.00036	1.35259
C	3.34143	0.14125	1.17151
N	2.99895	0.19762	-0.25695
C	4.08235	0.80154	-1.02219
C	5.30896	-0.12156	-0.95603
O	5.42359	-0.78173	0.30817
H	5.08995	-0.51377	2.29022
H	5.34191	0.98915	1.38976
H	3.00722	1.04930	1.70832
H	2.82669	-0.71354	1.62525
H	3.76994	0.90592	-2.06616
H	4.33368	1.81791	-0.66400
H	5.22307	-0.91888	-1.70084
H	6.22283	0.45501	-1.16737
C	-3.94048	1.69798	-0.36235
H	-3.37671	2.23125	-1.13543
H	-4.97950	1.63912	-0.70786
C	-3.88104	2.51278	0.93972
H	-2.84308	2.62040	1.26840
H	-4.44631	2.01628	1.73667
H	-4.30080	3.51533	0.79525

C	-4.09045	-0.86125	-0.08965
C	-5.55678	-1.14307	-0.11097
H	-5.82020	-1.86993	-0.88994
H	-5.90864	-1.54837	0.84654
H	-6.11652	-0.22553	-0.30778

4s_Senantiomer_conformer-073

C	2.61563	0.59547	0.36298
N	2.92316	-1.61300	0.58011
H	3.35021	-2.49386	0.83298
C	1.77782	-1.48236	-0.13779
C	1.54417	-0.11623	-0.29713
C	-0.01648	-2.04059	-1.74369
C	0.93616	-2.59612	-0.66641
H	-0.79584	-2.77859	-1.96465
H	0.54982	-1.88595	-2.66945
H	1.56022	-3.39220	-1.09001
H	0.36473	-3.06028	0.14940
C	0.38464	0.36748	-1.01943
O	0.21602	1.54336	-1.35732
C	-0.66844	-0.70033	-1.34135
H	-1.24123	-0.32552	-2.19564
C	-1.61960	-0.90264	-0.14200
H	-2.43049	-1.57370	-0.47611
H	-1.08407	-1.43650	0.64993
C	-4.33219	0.65922	-0.58759
C	-2.91043	1.19332	-0.41620
N	-2.08725	0.34720	0.45958
C	-2.80051	0.13653	1.72563
C	-4.22993	-0.37554	1.52835
O	-4.97105	0.50497	0.68239
H	-4.32731	-0.30529	-1.12080
H	-4.94770	1.36056	-1.15933
H	-2.96931	2.18605	0.04933
H	-2.41256	1.31696	-1.38000
H	-2.22964	-0.55880	2.35160
H	-2.84789	1.10077	2.24911
H	-4.22388	-1.38941	1.09688
H	-4.76636	-0.41394	2.48150
C	2.78522	2.08590	0.42621
H	1.81632	2.55010	0.64241
H	3.45402	2.34700	1.25518
C	3.33437	2.67889	-0.88113
H	2.64509	2.46602	-1.70330
H	4.31158	2.24698	-1.12563

H	3.45081	3.76627	-0.80103
C	3.45136	-0.36055	0.89882
C	4.71561	-0.24571	1.68517
H	4.94130	0.80408	1.88787
H	4.64485	-0.76596	2.64881
H	5.57188	-0.67136	1.14601

4s_Senantiomer_conformer-074

C	-3.02536	0.32024	-0.46314
N	-2.79236	-1.87117	-0.05760
H	-3.00365	-2.85913	-0.01680
C	-1.65903	-1.30015	0.42146
C	-1.75805	0.07299	0.19002
C	0.29888	-0.98347	1.87986
C	-0.51060	-2.00459	1.06187
H	1.24648	-1.42804	2.19468
H	-0.26748	-0.72600	2.78295
H	-0.85832	-2.82305	1.70338
H	0.12853	-2.46472	0.29444
C	-0.68026	0.97309	0.54147
O	-0.70745	2.19662	0.36500
C	0.58771	0.30666	1.09424
H	1.04281	1.04228	1.76656
C	1.54355	0.07873	-0.10176
H	1.05382	-0.58046	-0.82662
H	1.68383	1.04661	-0.60888
C	4.22497	-0.31234	-1.73575
C	3.48383	-1.26324	-0.79423
N	2.79538	-0.56519	0.29818
C	3.74243	0.33160	0.97600
C	4.48321	1.25290	0.00420
O	5.15069	0.49466	-1.00607
H	3.51213	0.33196	-2.27578
H	4.81231	-0.86801	-2.47328
H	4.21751	-1.94715	-0.34668
H	2.75814	-1.86637	-1.35070
H	3.21483	0.92182	1.73153
H	4.48038	-0.29075	1.49843
H	3.78640	1.96497	-0.46623
H	5.25663	1.82639	0.52408
C	-3.56636	1.65898	-0.87417
H	-2.78342	2.22023	-1.39636
H	-4.38863	1.52180	-1.58647
C	-4.05692	2.49488	0.31873
H	-4.45076	3.46210	-0.01526

H	-3.23000	2.68179	1.01009
H	-4.85140	1.97080	0.86219
C	-3.64252	-0.90402	-0.59968
C	-4.96246	-1.29389	-1.17899
H	-5.46634	-0.41927	-1.59738
H	-4.85203	-2.03356	-1.98218
H	-5.62707	-1.73010	-0.42205

4s_Senantiomer_conformer-075

C	-2.56003	0.40956	0.11839
N	-2.29658	-1.21594	-1.40097
H	-2.46105	-1.79405	-2.21408
C	-1.31963	-1.42822	-0.48430
C	-1.44123	-0.43240	0.48635
C	0.27789	-2.69215	0.89695
C	-0.29928	-2.51354	-0.51905
H	1.17043	-3.32748	0.85602
H	-0.46342	-3.22579	1.50468
H	-0.74148	-3.45457	-0.86968
H	0.49952	-2.24299	-1.21734
C	-0.51798	-0.34406	1.59349
O	-0.56508	0.52985	2.46988
C	0.62113	-1.37202	1.61523
H	0.79132	-1.59326	2.67675
C	1.93536	-0.71653	1.12341
H	2.16012	0.12865	1.79077
H	2.73853	-1.44768	1.26459
C	2.73646	2.03044	-0.27114
C	1.55530	1.08970	-0.51392
N	1.90626	-0.31903	-0.28831
C	3.11254	-0.65358	-1.05183
C	4.26475	0.31785	-0.79285
O	3.86066	1.65901	-1.07121
H	3.02236	2.02318	0.79344
H	2.48309	3.05801	-0.54965
H	1.22668	1.19614	-1.55604
H	0.71406	1.36515	0.12520
H	3.41413	-1.68071	-0.82155
H	2.85847	-0.61223	-2.11973
H	4.60436	0.23919	0.25349
H	5.11577	0.10787	-1.44840
C	-3.03810	1.62816	0.85400
H	-4.05576	1.87776	0.53061
H	-3.08977	1.40679	1.92576
C	-2.12506	2.84776	0.64892

H	-2.51961	3.72600	1.17375
H	-2.03663	3.09546	-0.41496
H	-1.12561	2.63741	1.04066
C	-3.06634	-0.10117	-1.05624
C	-4.20449	0.33092	-1.92085
H	-3.86445	0.64534	-2.91612
H	-4.93497	-0.47542	-2.06524
H	-4.72623	1.17685	-1.46679

4s_Senantiomer_conformer-076

C	2.04883	-0.66654	0.51754
N	2.47604	0.50280	-1.34516
H	2.90531	0.77964	-2.21769
C	1.52637	1.21670	-0.68506
C	1.22040	0.51803	0.48269
C	0.27757	3.19141	0.08953
C	0.93066	2.51188	-1.12564
H	-0.34131	4.03261	-0.24296
H	1.07078	3.60955	0.72110
H	1.69346	3.16694	-1.56342
H	0.18357	2.34673	-1.91490
C	0.21689	1.00044	1.41143
O	-0.01859	0.48582	2.50814
C	-0.57324	2.23499	0.94820
H	-0.84275	2.76861	1.86553
C	-1.91100	1.78512	0.26736
H	-2.72696	2.00997	0.95906
H	-2.09306	2.39979	-0.62902
C	-1.66356	-1.46765	-1.64854
C	-1.69112	0.04310	-1.45093
N	-1.98622	0.35736	-0.05673
C	-3.24581	-0.26466	0.34678
C	-3.17629	-1.76928	0.11060
O	-2.89438	-2.06843	-1.25381
H	-0.83249	-1.89435	-1.06637
H	-1.51989	-1.71490	-2.70417
H	-2.43942	0.49038	-2.13418
H	-0.71086	0.44399	-1.71478
H	-3.41057	-0.07524	1.41294
H	-4.10935	0.14824	-0.20996
H	-2.39878	-2.20521	0.75727
H	-4.13529	-2.24110	0.34275
C	2.04596	-1.72461	1.58326
H	2.95375	-2.33400	1.49936
H	2.07497	-1.24440	2.56741

C	0.81150	-2.63764	1.52031
H	-0.09864	-2.04637	1.65251
H	0.75251	-3.14865	0.55246
H	0.84484	-3.39959	2.30815
C	2.80928	-0.64985	-0.63257
C	3.83127	-1.60146	-1.16190
H	4.79483	-1.10716	-1.34022
H	3.99926	-2.41275	-0.44951
H	3.51493	-2.05281	-2.11119

4s_Senantiomer_conformer-077

C	-2.55406	-0.69793	-0.20561
N	-3.00439	1.12034	1.02467
H	-3.49038	1.74983	1.64928
C	-1.86324	1.41907	0.35508
C	-1.53901	0.30905	-0.42581
C	-0.18868	2.78721	-0.81864
C	-1.10003	2.69930	0.41981
H	0.51466	3.61963	-0.70746
H	-0.80875	3.00145	-1.69725
H	-1.77595	3.56152	0.46019
H	-0.49797	2.73560	1.33875
C	-0.36645	0.30145	-1.27481
O	-0.11819	-0.59500	-2.09024
C	0.59850	1.48184	-1.06690
H	1.18644	1.59097	-1.98358
C	1.58528	1.20185	0.09951
H	1.79177	2.14934	0.61025
H	1.08960	0.55760	0.84499
C	3.68292	-0.45634	1.73095
C	3.88339	0.68956	0.73510
N	2.88811	0.69493	-0.34665
C	2.86068	-0.63980	-0.96737
C	2.66832	-1.76240	0.05175
O	3.68060	-1.71756	1.06122
H	2.74045	-0.32803	2.28728
H	4.50294	-0.49123	2.45498
H	4.87472	0.57859	0.27601
H	3.85960	1.65356	1.25627
H	2.07556	-0.67806	-1.72468
H	3.82797	-0.78806	-1.46551
H	1.67300	-1.69785	0.52084
H	2.75275	-2.74286	-0.42707
C	-2.57926	-2.07507	-0.80273
H	-3.57109	-2.52061	-0.66206

H	-2.41026	-2.00512	-1.88271
C	-1.51250	-3.00217	-0.19733
H	-0.51242	-2.59653	-0.37799
H	-1.56095	-4.00180	-0.64490
H	-1.65254	-3.10379	0.88481
C	-3.44345	-0.16666	0.70271
C	-4.67812	-0.72544	1.32958
H	-5.54930	-0.08137	1.15500
H	-4.90259	-1.71046	0.91340
H	-4.56844	-0.83988	2.41572

4s_Senantiomer_conformer-078

C	2.41569	-0.49786	-0.28805
N	2.05756	1.41962	-1.39010
H	2.15449	2.15417	-2.07810
C	1.18046	1.43816	-0.35583
C	1.36811	0.25826	0.36591
C	-0.27504	2.36847	1.39651
C	0.18182	2.50541	-0.06693
H	-1.15523	2.99835	1.57112
H	0.52352	2.75326	2.04293
H	0.61114	3.50054	-0.23893
H	-0.67495	2.39642	-0.74038
C	0.53390	-0.07224	1.49742
O	0.63384	-1.11875	2.15299
C	-0.58538	0.92247	1.83141
H	-0.67596	0.90536	2.92509
C	-1.93811	0.38685	1.30060
H	-2.14466	-0.56704	1.80768
H	-2.71818	1.08584	1.62103
C	-2.91198	-2.02413	-0.53770
C	-1.70262	-1.09503	-0.65615
N	-1.98729	0.25847	-0.15992
C	-3.20871	0.76936	-0.78924
C	-4.38823	-0.19517	-0.66108
O	-4.05005	-1.47256	-1.20259
H	-3.15569	-2.20538	0.52203
H	-2.71312	-2.98990	-1.01249
H	-1.41707	-1.01767	-1.71335
H	-0.85134	-1.51201	-0.11327
H	-3.45795	1.74432	-0.35757
H	-3.00053	0.92134	-1.85711
H	-4.68157	-0.30321	0.39659
H	-5.25589	0.16121	-1.22521
C	2.96428	-1.82270	0.15616

H	2.13349	-2.50009	0.38335
H	3.53380	-2.27946	-0.66203
C	3.85806	-1.71047	1.40142
H	3.28312	-1.30476	2.23905
H	4.24717	-2.69245	1.69561
H	4.70986	-1.04717	1.21235
C	2.82337	0.25014	-1.37017
C	3.86787	0.01042	-2.41004
H	4.33303	-0.96717	-2.26218
H	3.44634	0.03130	-3.42320
H	4.66305	0.76627	-2.37157

4s_Senantiomer_conformer-079

C	-3.38111	0.28832	0.26770
N	-3.17180	-1.89062	-0.21259
H	-3.42291	-2.84650	-0.42640
C	-1.90206	-1.41756	-0.14712
C	-1.98089	-0.05751	0.15137
C	0.47807	-1.21621	-0.71409
C	-0.64555	-2.19590	-0.34478
H	0.30790	-0.83859	-1.73135
H	1.44555	-1.72446	-0.71913
H	-0.38097	-2.73050	0.58027
H	-0.77043	-2.95943	-1.12175
C	-0.78966	0.74660	0.33225
O	-0.80990	1.95893	0.57310
C	0.54450	-0.02036	0.25206
H	0.70167	-0.41974	1.26731
C	1.69090	0.93819	-0.09277
H	1.49109	1.37292	-1.08033
H	1.69398	1.77777	0.62237
C	4.71156	-1.08922	0.87025
C	3.52017	-0.15019	1.12265
N	2.99279	0.28207	-0.16577
C	3.97287	1.13458	-0.85107
C	5.40497	0.66888	-0.52865
O	5.44320	-0.73301	-0.30512
H	4.35846	-2.11100	0.70023
H	5.37779	-1.09275	1.74703
H	3.81461	0.70770	1.75717
H	2.75331	-0.69930	1.67518
H	3.77812	1.08286	-1.92908
H	3.87691	2.19508	-0.55003
H	6.07527	0.87492	-1.36701
H	5.79627	1.20410	0.34969

C	-3.94053	1.65307	0.54819
H	-4.98435	1.56460	0.87230
H	-3.38693	2.10740	1.37729
C	-3.86059	2.59021	-0.66758
H	-4.41448	2.17525	-1.51742
H	-2.81736	2.72595	-0.96763
H	-4.28140	3.57502	-0.43242
C	-4.09201	-0.86899	0.03488
C	-5.55926	-1.14477	0.00254
H	-6.11969	-0.24778	0.27685
H	-5.89469	-1.45365	-0.99602
H	-5.83925	-1.94222	0.70253

4s_Senantiomer_conformer-080

C	-3.37984	0.32737	-0.19401
N	-3.17211	-1.89745	-0.02306
H	-3.42192	-2.87658	0.01288
C	-1.90301	-1.41918	0.00069
C	-1.98099	-0.03011	-0.09744
C	0.50673	-1.38212	-0.46463
C	-0.64810	-2.21719	0.10787
H	0.39666	-1.31447	-1.55537
H	1.46615	-1.86767	-0.26945
H	-0.44180	-2.46043	1.16132
H	-0.74004	-3.17305	-0.42129
C	-0.79079	0.79550	-0.09053
O	-0.80866	2.02636	-0.20376
C	0.53741	0.04175	0.11760
H	0.64051	-0.04656	1.21151
C	1.70974	0.86418	-0.43168
H	1.56377	0.99264	-1.51170
H	1.68658	1.87528	0.00823
C	5.43806	0.49790	-0.58155
C	4.02860	0.84384	-1.09702
N	3.00674	0.22043	-0.24602
C	3.46457	0.18078	1.13737
C	4.65756	-0.78527	1.22755
O	5.45118	-0.77960	0.03850
H	5.79017	1.26570	0.12381
H	6.15157	0.45659	-1.40850
H	3.88765	0.48263	-2.12285
H	3.92830	1.94563	-1.11972
H	3.73534	1.18668	1.51173
H	2.66571	-0.18952	1.78510
H	5.27862	-0.53256	2.10109

H	4.30322	-1.81437	1.34144
C	-3.93977	1.71756	-0.28557
H	-3.36972	2.28861	-1.02642
H	-4.97558	1.67534	-0.64300
C	-3.89302	2.46758	1.05532
H	-4.31324	3.47546	0.95637
H	-4.46446	1.93203	1.82203
H	-2.85818	2.56037	1.39791
C	-4.09100	-0.85188	-0.14147
C	-5.55712	-1.13197	-0.18750
H	-6.11571	-0.20541	-0.34049
H	-5.81505	-1.81774	-1.00463
H	-5.91535	-1.58603	0.74546

4s_Senantiomer_conformer-081

C	-3.19111	0.07484	-0.39633
N	-2.59698	-1.96684	0.31059
H	-2.65418	-2.95251	0.52880
C	-1.52032	-1.17961	0.55737
C	-1.84399	0.10971	0.13063
C	0.48855	-0.36332	1.71848
C	-0.22516	-1.60692	1.16192
H	1.51298	-0.62105	1.99964
H	-0.03376	-0.03067	2.62352
H	-0.38532	-2.34684	1.95499
H	0.40711	-2.09453	0.40613
C	-0.88387	1.19036	0.21036
O	-1.10880	2.35227	-0.14655
C	0.51523	0.79858	0.71005
H	0.91761	1.69178	1.19922
C	1.37332	0.48416	-0.53653
H	0.87779	-0.29863	-1.12210
H	1.40520	1.38623	-1.17558
C	4.92337	0.45201	0.72799
C	3.56108	1.03822	0.37532
N	2.71098	0.00120	-0.21080
C	3.37255	-0.57658	-1.38138
C	4.73526	-1.13102	-0.98379
O	5.55803	-0.11730	-0.41312
H	4.79947	-0.31509	1.50914
H	5.59284	1.23137	1.10216
H	3.69574	1.88807	-0.32180
H	3.09450	1.42507	1.28645
H	2.74929	-1.38524	-1.77984
H	3.50782	0.17334	-2.18467

H	4.60003	-1.95349	-0.26327
H	5.26863	-1.51172	-1.85917
C	-3.96351	1.24292	-0.93733
H	-3.32748	1.80900	-1.62690
H	-4.82046	0.88184	-1.51832
C	-4.45710	2.19178	0.16655
H	-5.02020	3.03051	-0.25958
H	-3.60536	2.59708	0.72065
H	-5.10953	1.66420	0.87165
C	-3.62983	-1.22496	-0.26821
C	-4.92768	-1.87554	-0.61786
H	-5.59417	-1.15667	-1.10044
H	-4.78840	-2.71851	-1.30665
H	-5.44272	-2.26163	0.27131

4s_Senantiomer_conformer-082

C	2.34836	-0.87541	-0.19704
N	3.06719	1.10128	-0.96874
H	3.64177	1.78703	-1.43982
C	1.96227	1.38754	-0.23353
C	1.47649	0.17987	0.26875
C	0.42044	2.67857	1.20156
C	1.38453	2.74369	0.00057
H	-0.18360	3.59201	1.24156
H	1.01028	2.64382	2.12486
H	2.17497	3.47927	0.19176
H	0.85662	3.09197	-0.89810
C	0.29074	0.13420	1.09917
O	-0.09152	-0.86960	1.70961
C	-0.50577	1.44449	1.16841
H	-1.07357	1.41434	2.10354
C	-1.48902	1.53707	-0.01813
H	-2.14932	2.41118	0.15093
H	-0.92557	1.73948	-0.93521
C	-3.74008	-0.90293	-1.76651
C	-3.01695	0.41214	-1.50127
N	-2.25241	0.31871	-0.25825
C	-3.14207	-0.03481	0.84882
C	-3.86884	-1.33503	0.52765
O	-4.60689	-1.24211	-0.68821
H	-2.99901	-1.70410	-1.91766
H	-4.36564	-0.82719	-2.66037
H	-3.76066	1.23236	-1.46214
H	-2.32973	0.62532	-2.32847
H	-2.54647	-0.18238	1.75222

H	-3.88386	0.76497	1.04191
H	-3.13235	-2.15131	0.45677
H	-4.59019	-1.57779	1.31288
C	2.21979	-2.33745	0.11948
H	1.16761	-2.63249	0.03724
H	2.77386	-2.92630	-0.62139
C	2.72019	-2.68782	1.52990
H	2.13679	-2.14277	2.27748
H	3.77576	-2.41744	1.64805
H	2.61732	-3.76140	1.72786
C	3.32222	-0.27166	-0.96281
C	4.49336	-0.83124	-1.70125
H	4.46596	-0.57119	-2.76709
H	5.44460	-0.45980	-1.29854
H	4.50425	-1.92111	-1.62318

4s_Senantiomer_conformer-083

C	3.32905	0.40700	-0.33345
N	3.32787	-1.78992	0.10861
H	3.66829	-2.72546	0.28511
C	2.02294	-1.42088	0.13794
C	1.97196	-0.05393	-0.13385
C	-0.32139	-1.42345	0.86542
C	0.84971	-2.30258	0.40200
H	-0.11273	-1.04765	1.87605
H	-1.24178	-2.01003	0.92670
H	0.56663	-2.84245	-0.51449
H	1.08838	-3.06553	1.15257
C	0.70988	0.65220	-0.21879
O	0.61515	1.86620	-0.43166
C	-0.54871	-0.22374	-0.07077
H	-0.74169	-0.61925	-1.08131
C	-1.74718	0.63034	0.36181
H	-1.52572	1.06577	1.34316
H	-1.85454	1.46940	-0.34663
C	-5.04566	1.20501	0.17196
C	-4.06513	0.57706	1.15730
N	-2.97586	-0.15300	0.48227
C	-3.40934	-0.68602	-0.81845
C	-4.90280	-0.97663	-0.80653
O	-5.68280	0.20994	-0.63906
H	-4.51993	1.92770	-0.47061
H	-5.84044	1.74214	0.70091
H	-4.61746	-0.10915	1.81312
H	-3.63606	1.35351	1.79734

H	-3.20016	0.02704	-1.63763
H	-2.86189	-1.60825	-1.04913
H	-5.20816	-1.42013	-1.75895
H	-5.13147	-1.70007	-0.00913
C	3.75742	1.81791	-0.61625
H	4.78262	1.82143	-1.00525
H	3.11845	2.24032	-1.39961
C	3.67788	2.72283	0.62371
H	4.00224	3.74307	0.38681
H	2.64780	2.76669	0.98935
H	4.31532	2.33903	1.42843
C	4.14459	-0.69248	-0.17489
C	5.62807	-0.84874	-0.24685
H	6.05237	-1.16109	0.71616
H	5.92306	-1.59821	-0.99234
H	6.09624	0.09915	-0.52293

4s_Senantiomer_conformer-084

C	-3.28971	0.41608	-0.23470
N	-3.27972	-1.80352	0.07393
H	-3.61565	-2.75206	0.17253
C	-1.97281	-1.44146	0.05327
C	-1.92705	-0.06004	-0.13099
C	0.42355	-1.64141	-0.42898
C	-0.79419	-2.34384	0.18938
H	0.30811	-1.61215	-1.52015
H	1.32887	-2.22112	-0.22253
H	-0.59707	-2.55587	1.25122
H	-0.97614	-3.31046	-0.29485
C	-0.66887	0.65493	-0.19189
O	-0.58478	1.86882	-0.40207
C	0.59196	-0.19913	0.09042
H	0.65446	-0.23298	1.18982
C	1.87023	0.48628	-0.42130
H	2.22234	-0.04250	-1.32358
H	1.61035	1.50214	-0.72926
C	4.97413	0.88611	-0.75307
C	3.99060	1.51980	0.23397
N	2.89874	0.61628	0.62060
C	3.47772	-0.65420	1.08028
C	4.46421	-1.25907	0.07953
O	5.50657	-0.33024	-0.22489
H	5.82893	1.54570	-0.93170
H	4.48394	0.68949	-1.71998
H	3.56595	2.43756	-0.18798

H	4.54246	1.78973	1.14449
H	4.01630	-0.45512	2.01614
H	2.67835	-1.36471	1.31012
H	4.95116	-2.14511	0.49822
H	3.94901	-1.55743	-0.84764
C	-3.72522	1.84275	-0.40605
H	-3.12259	2.31304	-1.19051
H	-4.76790	1.87148	-0.74425
C	-3.58490	2.66765	0.88346
H	-3.91749	3.70056	0.72662
H	-4.18488	2.23355	1.69138
H	-2.53914	2.69015	1.20432
C	-4.10280	-0.68836	-0.09958
C	-5.58871	-0.83673	-0.11010
H	-5.97127	-1.18500	0.85802
H	-6.06426	0.12255	-0.32812
H	-5.92005	-1.55565	-0.87021

4s_Senantiomer_conformer-085

C	-2.75752	0.54782	0.21832
N	-3.05677	-1.30168	-1.01177
H	-3.48113	-1.96299	-1.64814
C	-1.86601	-1.47887	-0.38708
C	-1.63457	-0.34757	0.39443
C	0.45807	-2.21532	-0.15136
C	-0.97352	-2.66934	-0.47305
H	0.83576	-1.60451	-0.97681
H	1.11967	-3.08564	-0.07566
H	-1.29669	-3.43855	0.24457
H	-1.02031	-3.12933	-1.46717
C	-0.47426	-0.24970	1.25367
O	-0.30803	0.65897	2.07538
C	0.54958	-1.40139	1.15218
H	0.28657	-2.06721	1.99014
C	1.98920	-0.92643	1.42689
H	1.97422	-0.25561	2.30254
H	2.59060	-1.80326	1.69088
C	4.65815	0.11847	-0.99198
C	4.06607	-0.16376	0.39970
N	2.62259	-0.30490	0.26910
C	2.01387	0.97297	-0.12214
C	2.97708	1.76238	-1.02608
O	3.77242	0.88586	-1.81116
H	4.82182	-0.81956	-1.53142
H	5.62564	0.63473	-0.89029

H	4.34789	0.63093	1.11646
H	4.48803	-1.09739	0.78495
H	1.07613	0.76722	-0.65216
H	1.75816	1.59222	0.75456
H	2.41937	2.39397	-1.72242
H	3.62041	2.42052	-0.42239
C	-2.90796	1.91012	0.83072
H	-3.93989	2.25934	0.70697
H	-2.71895	1.84690	1.90793
C	-1.94273	2.94099	0.22296
H	-2.10808	3.04144	-0.85580
H	-0.90659	2.62887	0.38485
H	-2.07931	3.92639	0.68386
C	-3.61701	-0.06928	-0.66436
C	-4.91981	0.36305	-1.25247
H	-5.22684	1.32295	-0.83040
H	-4.85579	0.48358	-2.34169
H	-5.71816	-0.36240	-1.05039

4s_Senantiomer_conformer-086

C	3.25207	0.20316	-0.03879
N	2.67559	-1.96093	0.03465
H	2.75258	-2.96296	0.14636
C	1.55538	-1.30950	-0.36651
C	1.86561	0.05049	-0.42258
C	-0.55815	-0.95216	-1.57649
C	0.23473	-1.92494	-0.68667
H	-1.59277	-1.29440	-1.66440
H	-0.11851	-0.95314	-2.58113
H	0.36110	-2.89086	-1.18959
H	-0.32518	-2.12788	0.23750
C	0.87351	1.03206	-0.80709
O	1.09133	2.24559	-0.89822
C	-0.54420	0.48770	-1.03402
H	-1.01099	1.15427	-1.76696
C	-1.30501	0.62346	0.30500
H	-1.28814	1.68894	0.60360
H	-0.76849	0.06645	1.08156
C	-5.00084	0.64275	-0.32205
C	-3.52051	0.86571	-0.67412
N	-2.65961	0.09783	0.23649
C	-3.28740	-0.05269	1.54337
C	-4.54912	-0.91573	1.38589
O	-5.21411	-0.68285	0.14074
H	-5.62873	0.77446	-1.20686

H	-5.33362	1.36989	0.43364
H	-3.30487	1.94954	-0.62265
H	-3.32379	0.54274	-1.70264
H	-2.59111	-0.55798	2.22015
H	-3.53115	0.92138	2.00735
H	-4.28303	-1.97733	1.38671
H	-5.23710	-0.72843	2.22473
C	4.00185	1.49825	0.08192
H	5.08011	1.30041	0.10554
H	3.80965	2.11198	-0.80493
C	3.60186	2.30063	1.33073
H	4.17011	3.23612	1.39497
H	2.53708	2.54888	1.29214
H	3.78979	1.72233	2.24255
C	3.72409	-1.06072	0.24086
C	5.06329	-1.54539	0.68984
H	5.01783	-2.00739	1.68450
H	5.47738	-2.29273	0.00100
H	5.76936	-0.71319	0.74310

4s_Senantiomer_conformer-087

C	-2.70376	-0.50954	0.32690
N	-2.89194	1.69539	0.68106
H	-3.25776	2.57897	1.00943
C	-1.74020	1.54477	-0.01898
C	-1.58017	0.18190	-0.26771
C	0.58175	2.01850	-0.64293
C	-0.81902	2.62471	-0.47252
H	0.99871	1.79203	0.34297
H	1.25281	2.74958	-1.10763
H	-1.16777	3.04297	-1.42887
H	-0.79844	3.45481	0.24333
C	-0.45913	-0.31881	-1.03416
O	-0.33769	-1.50144	-1.37402
C	0.57608	0.73306	-1.49028
H	0.24938	0.99967	-2.50888
C	1.99053	0.14320	-1.64231
H	1.90991	-0.83573	-2.14442
H	2.56373	0.80645	-2.29938
C	3.19471	-1.27014	1.63979
C	2.15521	-0.91814	0.56117
N	2.71843	0.05665	-0.38135
C	4.14939	-0.15691	-0.54499
C	4.84736	0.16933	0.78662
O	4.03614	-0.15992	1.91718

H	3.79852	-2.13524	1.32603
H	2.70035	-1.53531	2.57790
H	1.26039	-0.48280	1.02241
H	1.83692	-1.84485	0.05402
H	4.52938	0.51483	-1.32120
H	4.38721	-1.18680	-0.87292
H	5.81216	-0.35871	0.84391
H	5.03661	1.24464	0.86139
C	-2.96442	-1.98767	0.28585
H	-2.02961	-2.52432	0.48125
H	-3.66260	-2.26204	1.08583
C	-3.52748	-2.45533	-1.06580
H	-3.71098	-3.53638	-1.06209
H	-4.47274	-1.94836	-1.29106
H	-2.81493	-2.23051	-1.86474
C	-3.49631	0.45633	0.90817
C	-4.77742	0.36411	1.66991
H	-4.69200	0.81634	2.66623
H	-5.59938	0.87262	1.14959
H	-5.06564	-0.68171	1.80116

4s_Senantiomer_conformer-088

C	-3.35324	0.43262	0.27231
N	-3.35946	-1.79544	0.03203
H	-3.70343	-2.74164	-0.06123
C	-2.05206	-1.43695	-0.01363
C	-1.99633	-0.05087	0.13254
C	0.30089	-1.51912	-0.70398
C	-0.88117	-2.34600	-0.17758
H	0.10674	-1.23938	-1.74823
H	1.22107	-2.10914	-0.69320
H	-0.61497	-2.79973	0.78925
H	-1.11452	-3.17404	-0.85751
C	-0.73107	0.65403	0.17088
O	-0.63245	1.88162	0.27841
C	0.52410	-0.23793	0.11804
H	0.70374	-0.54122	1.16294
C	1.73839	0.55495	-0.37926
H	1.57671	0.80507	-1.43512
H	1.79835	1.51221	0.16287
C	4.71893	0.54556	1.33184
C	3.50522	-0.34435	1.08187
N	2.98307	-0.20892	-0.29443
C	3.99819	0.32347	-1.21094
C	5.36984	-0.21871	-0.84111

O	5.79926	0.23252	0.44838
H	5.09574	0.40571	2.35024
H	4.43476	1.60330	1.22169
H	2.72306	-0.08761	1.80302
H	3.78475	-1.38885	1.27322
H	4.04082	1.42838	-1.19506
H	3.74846	0.02842	-2.23749
H	5.34462	-1.31892	-0.86354
H	6.12039	0.11799	-1.56319
C	-3.77821	1.86477	0.42334
H	-4.80803	1.90600	0.79778
H	-3.14719	2.35251	1.17448
C	-3.68039	2.65716	-0.89012
H	-4.30964	2.20600	-1.66583
H	-2.64593	2.66631	-1.24592
H	-4.00383	3.69506	-0.74738
C	-4.17305	-0.67295	0.20393
C	-5.65808	-0.81556	0.27089
H	-6.12442	0.15513	0.45571
H	-6.07255	-1.21049	-0.66572
H	-5.96610	-1.49436	1.07638

4s_Senantiomer_conformer-089

C	-3.36260	0.41667	-0.23155
N	-3.36423	-1.76139	0.29554
H	-3.70573	-2.69134	0.49781
C	-2.05487	-1.41663	0.21379
C	-2.00209	-0.06045	-0.10821
C	0.32776	-1.69624	-0.29995
C	-0.88017	-2.31155	0.42173
H	0.18314	-1.78836	-1.38486
H	1.24442	-2.23642	-0.04908
H	-0.66068	-2.40652	1.49610
H	-1.08345	-3.32414	0.05359
C	-0.73788	0.63094	-0.26022
O	-0.64102	1.82183	-0.57749
C	0.51557	-0.20988	0.04977
H	0.64176	-0.13997	1.14314
C	1.75676	0.38407	-0.62604
H	1.65120	0.25969	-1.71088
H	1.79288	1.46903	-0.43794
C	5.40004	-0.47746	-0.61848
C	4.05628	-0.08748	-1.21373
N	2.99158	-0.28954	-0.22484
C	3.43997	0.05171	1.14197

C	4.65998	0.96787	1.14037
O	5.77471	0.36969	0.47392
H	6.18947	-0.39521	-1.37235
H	5.35799	-1.52442	-0.28137
H	3.85078	-0.70557	-2.09628
H	4.11496	0.96055	-1.56191
H	2.62914	0.54454	1.68770
H	3.68475	-0.86547	1.69399
H	4.40533	1.92765	0.66537
H	4.98371	1.17844	2.16478
C	-3.78885	1.82415	-0.53446
H	-3.19847	2.20845	-1.37331
H	-4.83809	1.83133	-0.85302
C	-3.61356	2.77003	0.66447
H	-2.56120	2.81073	0.96082
H	-4.20135	2.42481	1.52268
H	-3.93863	3.78661	0.41330
C	-4.18181	-0.66073	0.02788
C	-5.66887	-0.79481	0.05880
H	-6.02105	-1.57638	-0.62650
H	-6.03510	-1.05019	1.06159
H	-6.14080	0.14543	-0.23649

4s_Senantiomer_conformer-090

C	2.66965	-0.17599	0.08094
N	2.08508	1.18014	-1.60449
H	2.11974	1.66844	-2.48930
C	1.10421	1.32068	-0.67792
C	1.42348	0.48966	0.39479
C	-0.65520	2.48179	0.59776
C	-0.13060	2.13993	-0.80883
H	-1.66010	2.91168	0.52548
H	-0.00793	3.27098	0.99767
H	0.06222	3.06236	-1.36901
H	-0.87998	1.58216	-1.38662
C	0.55952	0.38089	1.54833
O	0.74441	-0.42296	2.47171
C	-0.65817	1.31938	1.61831
H	-0.56485	1.77680	2.61282
C	-1.98376	0.50385	1.76089
H	-1.79072	-0.22133	2.56087
H	-2.75433	1.19346	2.12428
C	-2.55607	-2.12033	-0.87793
C	-1.70319	-1.20846	-0.00130
N	-2.55713	-0.21760	0.64016

C	-3.34622	0.53888	-0.32130
C	-4.15429	-0.43271	-1.17708
O	-3.30029	-1.36729	-1.83334
H	-3.24464	-2.70170	-0.24431
H	-1.92740	-2.81123	-1.44684
H	-0.91735	-0.74884	-0.62506
H	-1.20118	-1.80092	0.77151
H	-4.02586	1.20675	0.22015
H	-2.73431	1.16154	-0.99675
H	-4.87714	-0.96940	-0.54228
H	-4.69664	0.09991	-1.96352
C	3.37435	-1.19460	0.92922
H	4.42336	-1.27245	0.61908
H	3.37473	-0.86042	1.97243
C	2.71549	-2.58163	0.85593
H	3.25373	-3.30620	1.47854
H	2.70662	-2.95501	-0.17445
H	1.68283	-2.52268	1.21206
C	3.05391	0.27577	-1.16275
C	4.24210	-0.03818	-2.01076
H	3.95318	-0.51221	-2.95769
H	4.81542	0.86423	-2.25868
H	4.91076	-0.72541	-1.48681

4s_Senantiomer_conformer-091

C	-2.76813	0.58598	0.26594
N	-3.14459	-1.22174	-1.00440
H	-3.60092	-1.85501	-1.64722
C	-1.94058	-1.44224	-0.42084
C	-1.66097	-0.33818	0.38419
C	0.37100	-2.24150	-0.27152
C	-1.07985	-2.65024	-0.56694
H	0.74199	-1.61966	-1.09186
H	1.01199	-3.12947	-0.23628
H	-1.40227	-3.43265	0.13659
H	-1.16521	-3.07867	-1.57242
C	-0.47100	-0.28639	1.20573
O	-0.24806	0.60527	2.03232
C	0.51296	-1.46785	1.05205
H	0.23652	-2.14982	1.87266
C	1.96701	-1.05026	1.32708
H	1.97688	-0.37925	2.20229
H	2.53696	-1.94744	1.59368
C	2.82247	1.36457	-1.45496
C	2.06768	0.82987	-0.24343

N	2.64367	-0.44901	0.17667
C	4.07219	-0.28214	0.44542
C	4.77713	0.27499	-0.78497
O	4.21210	1.52064	-1.18332
H	2.44525	2.35187	-1.73579
H	2.68351	0.67701	-2.30493
H	1.01982	0.68798	-0.52399
H	2.09449	1.57628	0.57187
H	4.24742	0.39916	1.30099
H	4.50297	-1.25656	0.70345
H	5.83351	0.46271	-0.57337
H	4.70317	-0.45050	-1.61098
C	-2.87113	1.93294	0.92061
H	-3.90082	2.30217	0.84648
H	-2.64224	1.83614	1.98755
C	-1.91236	2.96470	0.30420
H	-2.01411	3.93884	0.79690
H	-0.87616	2.63180	0.41716
H	-2.11725	3.09802	-0.76414
C	-3.66684	0.01222	-0.60671
C	-4.97692	0.48847	-1.14210
H	-5.24907	1.44296	-0.68525
H	-4.94349	0.63730	-2.22916
H	-5.78477	-0.22479	-0.93463

4s_Senantiomer_conformer-092

C	2.55132	0.24833	-0.41890
N	1.89762	1.72320	1.13633
H	1.87267	2.55905	1.70470
C	1.01709	0.69541	1.22848
C	1.38577	-0.25412	0.27687
C	-0.57493	-0.84505	2.30807
C	-0.18084	0.62981	2.10855
H	-1.55942	-0.90579	2.78443
H	0.14094	-1.27476	3.01843
H	0.01466	1.09803	3.08045
H	-1.00107	1.20129	1.65339
C	0.60845	-1.45462	0.06888
O	0.82565	-2.26317	-0.84341
C	-0.55644	-1.72592	1.03695
H	-0.36768	-2.75485	1.37267
C	-1.90389	-1.88059	0.26079
H	-1.68324	-2.57041	-0.56307
H	-2.61056	-2.38567	0.92922
C	-2.80523	0.99047	-2.00096

C	-1.84246	0.09352	-1.22892
N	-2.59624	-0.73376	-0.29651
C	-3.42953	0.05781	0.59676
C	-4.34862	0.94612	-0.23736
O	-3.59502	1.78054	-1.11447
H	-3.46155	0.36971	-2.63145
H	-2.25792	1.69068	-2.63848
H	-1.09551	0.72792	-0.72162
H	-1.30031	-0.55581	-1.92508
H	-4.03138	-0.61660	1.21643
H	-2.85033	0.70740	1.27591
H	-5.03803	0.31640	-0.82189
H	-4.93156	1.61360	0.40371
C	3.30773	-0.45524	-1.50811
H	2.59909	-0.82478	-2.25728
H	3.96885	0.25658	-2.01653
C	4.13654	-1.64118	-0.98955
H	4.68063	-2.12724	-1.80795
H	4.86652	-1.31037	-0.24197
H	3.48123	-2.38468	-0.52616
C	2.84715	1.47118	0.14280
C	3.93989	2.45238	-0.12735
H	4.54169	2.12545	-0.97869
H	3.54287	3.44860	-0.36011
H	4.61261	2.56141	0.73323

4s_Senantiomer_conformer-093

C	2.76823	0.57931	-0.26118
N	3.11416	-1.26445	0.96539
H	3.55959	-1.91968	1.59368
C	1.90841	-1.45251	0.37433
C	1.64769	-0.32507	-0.40451
C	-0.41567	-2.20988	0.20239
C	1.02754	-2.64925	0.49162
H	-0.77719	-1.59891	1.03514
H	-1.07180	-3.08564	0.14749
H	1.33841	-3.42081	-0.22889
H	1.10363	-3.10160	1.48733
C	0.45946	-0.23428	-1.22487
O	0.24754	0.68505	-2.02386
C	-0.53980	-1.40690	-1.10518
H	-0.26527	-2.07474	-1.93802
C	-1.98451	-0.96226	-1.38271
H	-1.97765	-0.27312	-2.24421
H	-2.56580	-1.84428	-1.67686

C	-4.72642	0.80291	0.45474
C	-4.09878	-0.20971	-0.51722
N	-2.66856	-0.38192	-0.23006
C	-2.09587	0.85411	0.28913
C	-2.73687	1.15264	1.65498
O	-4.10176	0.73332	1.72765
H	-5.78638	0.58376	0.60728
H	-4.65228	1.82360	0.04995
H	-4.26948	0.15013	-1.54961
H	-4.58803	-1.18783	-0.42964
H	-1.02148	0.73320	0.44096
H	-2.21992	1.70224	-0.40934
H	-2.21556	0.60076	2.44357
H	-2.65778	2.22873	1.87500
C	2.89397	1.94016	-0.88239
H	3.92853	2.29223	-0.79408
H	2.66979	1.87305	-1.95264
C	1.94720	2.97083	-0.24593
H	2.14600	3.07265	0.82702
H	0.90671	2.65807	-0.37555
H	2.06863	3.95576	-0.71200
C	3.65600	-0.02923	0.59894
C	4.97189	0.41350	1.14870
H	5.76919	-0.30684	0.92548
H	5.25990	1.37459	0.71610
H	4.93792	0.53606	2.23902

4s_Senantiomer_conformer-094

C	3.24754	0.18117	-0.05116
N	2.64181	-1.96452	0.17269
H	2.70467	-2.95714	0.35487
C	1.53190	-1.32865	-0.27863
C	1.86048	0.01984	-0.42936
C	-0.56852	-1.03092	-1.52507
C	0.20460	-1.94804	-0.56198
H	-1.60657	-1.36512	-1.59945
H	-0.11944	-1.10625	-2.52271
H	0.32049	-2.94945	-0.99290
H	-0.36366	-2.07556	0.37054
C	0.88265	0.98455	-0.88604
O	1.11556	2.18608	-1.06010
C	-0.54120	0.44232	-1.08261
H	-0.99157	1.06220	-1.86506
C	-1.30241	0.68058	0.24172
H	-1.29501	1.76476	0.46140

H	-0.75454	0.19514	1.05812
C	-4.84677	-0.04887	-0.77829
C	-3.58668	0.82068	-0.63381
N	-2.65084	0.13181	0.24641
C	-3.19028	0.06155	1.61108
C	-4.72347	-0.07585	1.57059
O	-5.13734	-0.78523	0.41229
H	-4.70072	-0.79914	-1.56143
H	-5.70623	0.58103	-1.05561
H	-3.83854	1.83072	-0.25854
H	-3.13815	0.95536	-1.62128
H	-2.73350	-0.79794	2.11625
H	-2.93787	0.96297	2.20054
H	-5.08209	-0.64128	2.43443
H	-5.20032	0.91543	1.60000
C	4.01431	1.47157	-0.01903
H	5.08974	1.26162	0.02221
H	3.83283	2.02414	-0.94741
C	3.62198	2.36469	1.16913
H	4.20223	3.29500	1.16935
H	2.56058	2.62326	1.10986
H	3.80025	1.84940	2.11982
C	3.70159	-1.06581	0.31937
C	5.03249	-1.53500	0.80784
H	4.97813	-1.92023	1.83425
H	5.43668	-2.33804	0.17837
H	5.75082	-0.71168	0.80064

4s_Senantiomer_conformer-095

C	-2.72890	-0.52514	0.35213
N	-2.96363	1.69819	0.50771
H	-3.35183	2.59940	0.75183
C	-1.79146	1.51005	-0.14754
C	-1.60251	0.13358	-0.27334
C	0.53816	1.97039	-0.75652
C	-0.87499	2.56534	-0.66521
H	0.93469	1.83140	0.25393
H	1.20961	2.67190	-1.26375
H	-1.20658	2.89998	-1.65985
H	-0.88329	3.45016	-0.01801
C	-0.45000	-0.41090	-0.95851
O	-0.28268	-1.61848	-1.16450
C	0.56301	0.62281	-1.50036
H	0.22297	0.80492	-2.53327
C	1.98221	0.04651	-1.62163

H	1.91057	-0.98258	-2.01292
H	2.52932	0.63889	-2.36459
C	3.00212	-0.27772	1.99817
C	2.26116	-0.69720	0.71728
N	2.76426	0.10629	-0.38988
C	4.17301	-0.20947	-0.65832
C	4.89452	-0.59942	0.64278
O	4.35608	0.10865	1.74927
H	2.96997	-1.09782	2.73238
H	2.52098	0.60020	2.44087
H	1.19609	-0.50702	0.86285
H	2.36238	-1.78220	0.52961
H	4.64233	0.67092	-1.11478
H	4.27735	-1.04519	-1.37621
H	4.82247	-1.68414	0.81398
H	5.95565	-0.34324	0.58630
C	-2.96214	-2.00610	0.43310
H	-2.02554	-2.50387	0.70713
H	-3.68388	-2.22333	1.22966
C	-3.46770	-2.60198	-0.89054
H	-4.41330	-2.13682	-1.19169
H	-2.73149	-2.43314	-1.68182
H	-3.63160	-3.68216	-0.79771
C	-3.55310	0.47201	0.82633
C	-4.85324	0.42179	1.55903
H	-4.80308	0.95941	2.51445
H	-5.66834	0.86834	0.97502
H	-5.12791	-0.61405	1.77251

4s_Senantiomer_conformer-096

C	-3.18425	0.06480	-0.37665
N	-2.55146	-1.98564	0.26869
H	-2.59190	-2.97721	0.46257
C	-1.48199	-1.18990	0.51936
C	-1.82964	0.10515	0.13050
C	0.53269	-0.37508	1.67107
C	-0.17206	-1.61423	1.09350
H	1.56485	-0.62422	1.93032
H	0.01900	-0.07269	2.59155
H	-0.31013	-2.37625	1.86961
H	0.45492	-2.07386	0.31616
C	-0.88253	1.19633	0.22216
O	-1.12777	2.36336	-0.10359
C	0.52871	0.81169	0.69196
H	0.92521	1.69783	1.19881

C	1.37464	0.54206	-0.57348
H	1.38074	1.45752	-1.19444
H	0.88262	-0.23465	-1.17102
C	4.84052	0.34935	0.86994
C	3.59707	1.06673	0.31754
N	2.72050	0.06909	-0.28334
C	3.35012	-0.51116	-1.47647
C	4.87582	-0.59562	-1.28323
O	5.20223	-0.78959	0.08505
H	4.64017	-0.03591	1.87438
H	5.68417	1.05440	0.92935
H	3.87869	1.85659	-0.40452
H	3.08296	1.56493	1.14326
H	2.92053	-1.50666	-1.64068
H	3.14714	0.08946	-2.38311
H	5.28697	-1.44684	-1.83175
H	5.36367	0.31389	-1.66491
C	-3.98106	1.23516	-0.87572
H	-3.36435	1.82658	-1.56157
H	-4.84274	0.87668	-1.45127
C	-4.46888	2.15000	0.25912
H	-5.05113	2.99037	-0.13698
H	-5.10151	1.59617	0.96214
H	-3.61368	2.55440	0.80849
C	-3.60315	-1.24389	-0.27525
C	-4.89688	-1.90392	-0.62240
H	-4.75666	-2.72466	-1.33739
H	-5.39132	-2.32307	0.26348
H	-5.58186	-1.18187	-1.07330

4s_Senantiomer_conformer-097

C	3.21867	0.35309	-0.08656
N	2.89148	-1.78229	0.50852
H	3.07958	-2.71122	0.86077
C	1.71679	-1.38380	-0.04054
C	1.87091	-0.05026	-0.42379
C	-0.38371	-1.58112	-1.30791
C	0.48541	-2.20970	-0.20509
H	-1.36913	-2.05472	-1.31497
H	0.08636	-1.77176	-2.28015
H	0.73646	-3.24677	-0.45694
H	-0.07677	-2.24757	0.73894
C	0.78492	0.68623	-1.03491
O	0.86365	1.86130	-1.41125
C	-0.55254	-0.06212	-1.13188

H	-1.06476	0.34772	-2.00886
C	-1.37211	0.30290	0.12756
H	-1.47660	1.40416	0.15538
H	-0.80485	0.01163	1.01795
C	-5.00636	-0.18182	-0.54234
C	-3.55184	0.07237	-0.90919
N	-2.66122	-0.37707	0.17202
C	-3.32190	-0.30404	1.48850
C	-4.37388	0.79865	1.55078
O	-5.41850	0.58152	0.59414
H	-5.15810	-1.25603	-0.35684
H	-5.65986	0.11090	-1.36943
H	-3.42614	1.15214	-1.11131
H	-3.31250	-0.45765	-1.83902
H	-3.80484	-1.26466	1.71135
H	-2.56663	-0.13950	2.26244
H	-4.84684	0.83076	2.53832
H	-3.90208	1.77664	1.37052
C	3.81178	1.71807	-0.28404
H	4.90435	1.66050	-0.21100
H	3.57793	2.07154	-1.29429
C	3.28506	2.74594	0.73050
H	3.51105	2.43259	1.75613
H	3.73978	3.72977	0.56471
H	2.20043	2.84880	0.63138
C	3.82348	-0.74147	0.49180
C	5.19475	-0.94238	1.04763
H	5.17270	-1.14276	2.12664
H	5.70790	-1.78623	0.56909
H	5.80296	-0.04856	0.88910

4s_Senantiomer_conformer-098

C	-2.67540	-0.69081	-0.21804
N	-3.11205	1.22148	0.86394
H	-3.60340	1.91092	1.41664
C	-1.92466	1.42991	0.23778
C	-1.60933	0.25736	-0.44824
C	-0.08832	2.65536	-0.87874
C	-1.11932	2.68661	0.26755
H	0.66374	3.43661	-0.72078
H	-0.59992	2.88951	-1.81954
H	-1.76350	3.56872	0.17003
H	-0.61206	2.78740	1.23715
C	-0.40579	0.15655	-1.24921
O	-0.17531	-0.75847	-2.04548

C	0.60799	1.28594	-1.03194
H	1.23916	1.31751	-1.92575
C	1.48681	0.98300	0.20133
H	2.28960	1.74053	0.22858
H	0.89058	1.13149	1.10751
C	4.27965	-0.22174	-0.66491
C	2.87138	-0.79094	-0.83392
N	1.96680	-0.39892	0.25655
C	2.59379	-0.73477	1.54094
C	4.01243	-0.17507	1.67731
O	4.83490	-0.61128	0.59401
H	4.27063	0.87755	-0.74301
H	4.95428	-0.61154	-1.43337
H	2.93906	-1.88670	-0.82492
H	2.43662	-0.50128	-1.79268
H	1.96052	-0.37250	2.35873
H	2.64525	-1.82926	1.61329
H	3.99485	0.92619	1.71192
H	4.48898	-0.53731	2.59351
C	-2.71479	-2.10607	-0.71706
H	-3.72858	-2.51037	-0.61168
H	-2.47291	-2.11928	-1.78508
C	-1.72079	-3.01617	0.02376
H	-1.75550	-4.03920	-0.36903
H	-0.70053	-2.63856	-0.09337
H	-1.95062	-3.05244	1.09480
C	-3.58718	-0.06597	0.60504
C	-4.87305	-0.53573	1.20158
H	-5.70756	0.12858	0.94333
H	-5.11742	-1.53591	0.83555
H	-4.82177	-0.58541	2.29697

4s_Senantiomer_conformer-099

C	3.06121	0.62990	0.23126
N	3.57615	-1.39285	-0.58470
H	4.12551	-2.16467	-0.93805
C	2.24186	-1.43552	-0.34266
C	1.87132	-0.19040	0.16274
C	0.08284	-2.41013	0.31381
C	1.32971	-2.59328	-0.56549
H	0.34855	-2.59158	1.36361
H	-0.67415	-3.15233	0.03903
H	1.02856	-2.64014	-1.62305
H	1.83001	-3.54253	-0.34035
C	0.50036	0.10056	0.53056

O	0.14954	1.16328	1.05391
C	-0.52525	-0.99769	0.18353
H	-0.76433	-0.85029	-0.88114
C	-1.84259	-0.86543	0.97344
H	-2.14259	-1.85879	1.32509
H	-1.67776	-0.23888	1.86649
C	-4.10291	1.64163	-0.70976
C	-2.75841	1.04296	-0.25534
N	-2.94284	-0.35899	0.15302
C	-4.24511	-0.52113	0.78745
C	-5.33633	-0.31031	-0.27702
O	-4.94659	0.64364	-1.26722
H	-3.94734	2.39412	-1.48727
H	-4.60924	2.13541	0.13380
H	-2.35157	1.65599	0.56517
H	-2.02711	1.07746	-1.07048
H	-4.32905	-1.53578	1.19009
H	-4.38649	0.17093	1.63991
H	-5.52159	-1.24191	-0.82053
H	-6.27556	0.00324	0.20540
C	3.13122	2.05943	0.68500
H	2.58422	2.16644	1.62808
H	4.17392	2.33145	0.88772
C	2.53790	3.03895	-0.34038
H	1.48170	2.81022	-0.51081
H	3.06614	2.96886	-1.29810
H	2.61130	4.07304	0.01659
C	4.09868	-0.14305	-0.24355
C	5.55242	0.14628	-0.42482
H	5.78938	1.14637	-0.05395
H	6.18081	-0.57039	0.11922
H	5.84844	0.10458	-1.48099

4s_Senantiomer_conformer-100

C	-3.02744	-0.65411	-0.25785
N	-3.50506	1.51948	-0.52146
H	-4.03431	2.36372	-0.69337
C	-2.18764	1.48053	-0.19969
C	-1.84156	0.14144	-0.02532
C	-0.06023	2.21701	0.78587
C	-1.26681	2.64500	-0.06359
H	-0.37189	2.11211	1.83342
H	0.70830	2.99657	0.75389
H	-0.91954	2.97117	-1.05566
H	-1.77657	3.50438	0.38765

C	-0.48583	-0.25507	0.29911
O	-0.15549	-1.42479	0.52108
C	0.55342	0.88485	0.30565
H	0.83958	1.02734	-0.74780
C	1.83393	0.52998	1.08822
H	2.10722	1.38215	1.72025
H	1.63426	-0.32283	1.75896
C	4.19024	-1.42420	-1.11129
C	2.82050	-0.95630	-0.58626
N	2.97607	0.27146	0.21128
C	4.24502	0.23961	0.92816
C	5.38877	0.32457	-0.09770
O	5.05857	-0.32093	-1.32962
H	4.08116	-1.93605	-2.07099
H	4.65105	-2.13369	-0.40722
H	2.36507	-1.76849	0.00274
H	2.14079	-0.74889	-1.42023
H	4.30337	1.10135	1.60097
H	4.34825	-0.66315	1.56029
H	5.59012	1.36893	-0.35533
H	6.30667	-0.11155	0.32678
C	-3.13480	-2.14799	-0.15014
H	-4.03332	-2.49208	-0.67610
H	-2.27683	-2.60962	-0.65113
C	-3.17636	-2.63802	1.30612
H	-4.03170	-2.20692	1.83871
H	-2.26055	-2.34308	1.82660
H	-3.26040	-3.73031	1.35063
C	-4.04005	0.23006	-0.56210
C	-5.48295	0.01623	-0.88274
H	-5.70893	-1.05215	-0.91964
H	-6.13976	0.46877	-0.12863
H	-5.75315	0.44985	-1.85391

4s_Senantiomer_conformer-101

C	3.38010	-0.31376	-0.20682
N	3.17038	1.89950	0.07506
H	3.41938	2.87577	0.16058
C	1.90130	1.42052	0.06567
C	1.98054	0.03827	-0.10232
C	-0.50491	1.40617	-0.41806
C	0.64523	2.21199	0.20386
H	-0.38633	1.39226	-1.50979
H	-1.46551	1.88342	-0.20749
H	0.43097	2.40174	1.26666

H	0.74037	3.19332	-0.27580
C	0.79041	-0.78641	-0.14787
O	0.80919	-2.00903	-0.32954
C	-0.53838	-0.04473	0.09344
H	-0.65005	-0.01143	1.18951
C	-1.71052	-0.83809	-0.49732
H	-1.57057	-0.91378	-1.58274
H	-1.68304	-1.86658	-0.09845
C	-4.86215	0.00056	1.35263
C	-3.34145	-0.14137	1.17153
N	-2.99895	-0.19764	-0.25693
C	-4.08233	-0.80153	-1.02223
C	-5.30902	0.12150	-0.95598
O	-5.42344	0.78190	0.30811
H	-5.08984	0.51411	2.29021
H	-5.34212	-0.98885	1.38993
H	-3.00741	-1.04956	1.70822
H	-2.82653	0.71327	1.62535
H	-3.76992	-0.90584	-2.06620
H	-4.33361	-1.81794	-0.66409
H	-5.22332	0.91871	-1.70093
H	-6.22290	-0.45515	-1.16705
C	3.94054	-1.69797	-0.36232
H	3.37706	-2.23117	-1.13568
H	4.97968	-1.63909	-0.70746
C	3.88062	-2.51288	0.93965
H	4.44568	-2.01649	1.73682
H	4.30034	-3.51546	0.79522
H	2.84255	-2.62045	1.26798
C	4.09048	0.86127	-0.08966
C	5.55682	1.14307	-0.11082
H	5.82037	1.86980	-0.88986
H	5.90856	1.54849	0.84668
H	6.11656	0.22548	-0.30742

4s_Senantiomer_conformer-102

C	-2.71191	-0.58543	0.27249
N	-2.98791	1.57967	0.78136
H	-3.39150	2.42303	1.16649
C	-1.81992	1.52231	0.09496
C	-1.60562	0.18768	-0.24947
C	0.49340	2.12464	-0.44590
C	-0.93183	2.66566	-0.25860
H	0.90041	1.84176	0.52959
H	1.14509	2.91131	-0.84198

H	-1.27888	3.14139	-1.18831
H	-0.95489	3.43983	0.51727
C	-0.45292	-0.21514	-1.02635
O	-0.27508	-1.36562	-1.44216
C	0.54544	0.90802	-1.38796
H	0.21208	1.24161	-2.38448
C	1.97679	0.38165	-1.58091
H	1.92621	-0.57281	-2.13111
H	2.52139	1.09535	-2.20926
C	3.04991	-0.81333	1.84243
C	2.20809	-0.76574	0.57255
N	2.73905	0.25034	-0.33806
C	4.14729	-0.02863	-0.62060
C	4.94170	-0.08981	0.67812
O	4.41923	-1.08255	1.55619
H	2.70452	-1.61473	2.50151
H	2.96199	0.14692	2.37601
H	1.17973	-0.51276	0.84691
H	2.18915	-1.76531	0.10063
H	4.54837	0.76873	-1.25698
H	4.27057	-0.98572	-1.16439
H	5.98293	-0.36066	0.48201
H	4.91725	0.89551	1.17068
C	-2.91616	-2.06512	0.11994
H	-1.96534	-2.58069	0.29394
H	-3.61687	-2.42188	0.88441
C	-3.43863	-2.45311	-1.27255
H	-3.58355	-3.53727	-1.34946
H	-4.39729	-1.96402	-1.47987
H	-2.72085	-2.14747	-2.03937
C	-3.54947	0.30637	0.90649
C	-4.83863	0.11318	1.63483
H	-4.78493	0.48872	2.66470
H	-5.66872	0.63377	1.14003
H	-5.09323	-0.94851	1.67901

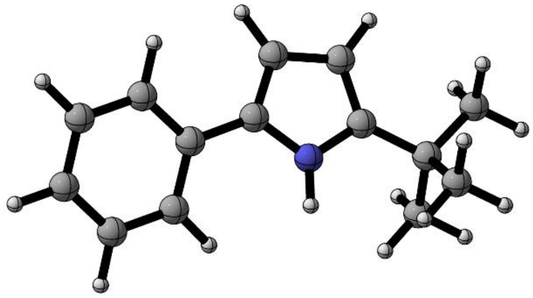

4t_conformer-001

C	-0.52004	0.60021	-0.08462
C	0.03611	1.85825	-0.27780

C	1.45045	1.71759	-0.26241
C	1.74640	0.37875	-0.05598
N	0.54047	-0.28078	0.03762
H	0.44212	-1.25830	0.26817
H	-0.51923	2.77083	-0.44735
H	2.17546	2.50783	-0.39630
C	-1.91287	0.16766	-0.03196
C	-2.93373	1.09816	0.24199
C	-2.28141	-1.17217	-0.25632
C	-4.26812	0.70423	0.28443
H	-2.67035	2.13328	0.43738
C	-3.61740	-1.56598	-0.20019
H	-1.52376	-1.91088	-0.50257
C	-4.61957	-0.63156	0.06809
H	-5.03710	1.44141	0.49851
H	-3.87549	-2.60629	-0.37795
H	-5.66040	-0.93884	0.10796
C	3.17106	-1.00329	1.46817
H	2.37142	-1.73271	1.64004
H	4.12773	-1.52873	1.56917
H	3.11000	-0.24333	2.25456
C	3.15681	-1.45240	-1.01223
H	3.09113	-1.01589	-2.01467
H	4.11090	-1.98561	-0.92961
H	2.35268	-2.19019	-0.91190
C	3.06207	-0.35444	0.07019
C	4.22344	0.63517	-0.11443
H	4.19734	1.42245	0.64660
H	5.17985	0.10884	-0.02658
H	4.18523	1.11055	-1.10044

4t_conformer-002

C	0.52004	0.60021	-0.08462
C	-0.03611	1.85826	-0.27780
C	-1.45045	1.71759	-0.26241
C	-1.74640	0.37875	-0.05598
N	-0.54047	-0.28077	0.03762
H	-0.44212	-1.25830	0.26816
H	0.51922	2.77083	-0.44735
H	-2.17546	2.50783	-0.39629
C	1.91287	0.16766	-0.03196
C	2.28141	-1.17217	-0.25632
C	2.93373	1.09816	0.24199
C	3.61739	-1.56598	-0.20019
H	1.52375	-1.91087	-0.50258

C	4.26813	0.70422	0.28443
H	2.67036	2.13328	0.43738
C	4.61957	-0.63157	0.06810
H	3.87548	-2.60630	-0.37795
H	5.03711	1.44140	0.49851
H	5.66039	-0.93885	0.10796
C	-3.15682	-1.45239	-1.01224
H	-2.35270	-2.19018	-0.91192
H	-4.11091	-1.98559	-0.92962
H	-3.09114	-1.01587	-2.01467
C	-3.17104	-1.00331	1.46816
H	-3.10998	-0.24336	2.25456
H	-4.12771	-1.52876	1.56916
H	-2.37141	-1.73274	1.64001
C	-3.06207	-0.35444	0.07019
C	-4.22344	0.63518	-0.11441
H	-4.18524	1.11056	-1.10041
H	-5.17985	0.10884	-0.02656
H	-4.19733	1.42244	0.64663

4t_conformer-003

C	0.52004	-0.60042	-0.08458
C	-0.03614	-1.85844	-0.27772
C	-1.45050	-1.71774	-0.26234
C	-1.74642	-0.37889	-0.05596
N	-0.54046	0.28059	0.03763
H	-0.44213	1.25813	0.26814
H	0.51916	-2.77106	-0.44719
H	-2.17552	-2.50798	-0.39621
C	1.91285	-0.16778	-0.03195
C	2.93381	-1.09818	0.24195
C	2.28121	1.17210	-0.25629
C	4.26816	-0.70408	0.28439
H	2.67057	-2.13334	0.43726
C	3.61714	1.56607	-0.20018
H	1.52343	1.91068	-0.50248
C	4.61943	0.63176	0.06807
H	5.03724	-1.44116	0.49842
H	3.87512	2.60641	-0.37794
H	5.66021	0.93916	0.10792
C	-3.17109	1.00300	1.46830
H	-3.11023	0.24284	2.25451
H	-2.37138	1.73226	1.64046
H	-4.12770	1.52854	1.56932
C	-3.15625	1.45275	-1.01197

H	-3.09056	1.01647	-2.01451
H	-4.11016	1.98624	-0.92936
H	-2.35188	2.19022	-0.91134
C	-3.06200	0.35448	0.07018
C	-4.22357	-0.63480	-0.11492
H	-4.19775	-1.42233	0.64587
H	-5.17987	-0.10829	-0.02703
H	-4.18533	-1.10989	-1.10106

4t_conformer-004

C	-0.52004	-0.60040	-0.08458
C	0.03614	-1.85843	-0.27773
C	1.45050	-1.71772	-0.26234
C	1.74642	-0.37888	-0.05595
N	0.54046	0.28061	0.03765
H	0.44211	1.25815	0.26816
H	-0.51916	-2.77104	-0.44721
H	2.17552	-2.50796	-0.39620
C	-1.91286	-0.16778	-0.03195
C	-2.93381	-1.09818	0.24195
C	-2.28122	1.17210	-0.25630
C	-4.26816	-0.70408	0.28439
H	-2.67057	-2.13334	0.43727
C	-3.61717	1.56607	-0.20018
H	-1.52345	1.91069	-0.50249
C	-4.61944	0.63175	0.06807
H	-5.03724	-1.44117	0.49843
H	-3.87514	2.60641	-0.37794
H	-5.66023	0.93915	0.10792
C	3.17104	1.00321	1.46822
H	2.37136	1.73255	1.64022
H	3.11009	0.24317	2.25453
H	4.12767	1.52871	1.56921
C	4.22355	-0.63488	-0.11468
H	4.18539	-1.11008	-1.10077
H	5.17987	-0.10840	-0.02676
H	4.19761	-1.42231	0.64620
C	3.06200	0.35449	0.07018
C	3.15639	1.45257	-1.01214
H	2.35203	2.19010	-0.91170
H	4.11032	1.98604	-0.92953
H	3.09076	1.01613	-2.01461

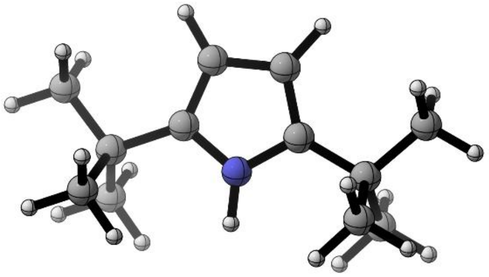

4u_conformer-001

C	-1.14118	-0.40363	-0.00005
C	-0.71454	-1.71841	-0.00003
C	0.71454	-1.71841	0.00003
C	1.14118	-0.40363	0.00005
N	-0.00000	0.37694	0.00001
H	-1.35661	-2.58833	-0.00006
H	1.35661	-2.58833	0.00005
H	-0.00000	1.38598	0.00000
C	2.52116	0.21362	0.00001
C	-2.52116	0.21362	-0.00001
C	-2.70789	1.08853	1.25929
H	-3.70997	1.53323	1.27051
H	-2.58535	0.49060	2.16889
H	-1.98020	1.90745	1.29281
C	-3.58288	-0.89738	-0.00056
H	-4.58609	-0.45752	-0.00053
H	-3.48921	-1.53274	-0.88788
H	-3.48945	-1.53339	0.88631
C	-2.70757	1.08951	-1.25867
H	-3.70969	1.53411	-1.26984
H	-1.97998	1.90854	-1.29133
H	-2.58471	0.49231	-2.16871
C	2.70784	1.08864	-1.25923
H	3.70993	1.53330	-1.27047
H	2.58523	0.49078	-2.16887
H	1.98018	1.90758	-1.29263
C	2.70763	1.08941	1.25873
H	3.70974	1.53402	1.26989
H	1.98002	1.90843	1.29149
H	2.58481	0.49213	2.16873
C	3.58288	-0.89738	0.00043
H	4.58609	-0.45752	0.00038
H	3.48925	-1.53280	0.88771
H	3.48941	-1.53333	-0.88648

4u_conformer-002

C	-1.14120	0.40354	0.00025
C	-0.71469	1.71834	0.00017
C	0.71473	1.71834	-0.00018
C	1.14123	0.40355	-0.00027
N	0.00002	-0.37697	-0.00004
H	-1.35679	2.58824	0.00030
H	1.35684	2.58824	-0.00030
H	0.00002	-1.38599	0.00010
C	2.52116	-0.21360	-0.00003
C	-2.52114	-0.21360	0.00002
C	-2.70843	-1.08701	-1.26027
H	-3.71069	-1.53125	-1.27202
H	-2.58568	-0.48815	-2.16922
H	-1.98110	-1.90618	-1.29472
C	-3.58273	0.89753	0.00229
H	-4.58603	0.45788	0.00216
H	-3.48861	1.53188	0.89026
H	-3.48960	1.53454	-0.88388
C	-2.70719	-1.09105	1.25768
H	-3.70961	-1.53493	1.26918
H	-1.98023	-1.91071	1.28858
H	-2.58311	-0.49525	2.16846
C	2.70836	-1.08702	1.26026
H	3.71073	-1.53100	1.27229
H	2.58519	-0.48825	2.16922
H	1.98120	-1.90635	1.29439
C	2.70723	-1.09113	-1.25762
H	3.70969	-1.53491	-1.26914
H	1.98035	-1.91086	-1.28836
H	2.58302	-0.49544	-2.16846
C	3.58270	0.89757	-0.00231
H	4.58603	0.45798	-0.00228
H	3.48845	1.53197	-0.89024
H	3.48959	1.53453	0.88390

4u_conformer-003

C	1.13994	-0.36503	-0.00001
C	0.71268	-1.68266	-0.00001
C	-0.71267	-1.68266	-0.00000
C	-1.13994	-0.36503	-0.00000
N	0.00000	0.41218	-0.00001
H	1.35768	-2.55198	-0.00000
H	-1.35768	-2.55198	0.00001
H	0.00000	1.42002	-0.00002
C	-2.54060	0.21543	0.00000

C	2.54060	0.21543	-0.00000
C	2.50821	1.75390	-0.00014
H	3.53021	2.14692	-0.00015
H	2.00332	2.14732	-0.89054
H	2.00327	2.14749	0.89015
C	3.29384	-0.26916	-1.25741
H	4.31877	0.12145	-1.26713
H	3.34531	-1.36266	-1.28528
H	2.78790	0.06895	-2.16857
C	3.29372	-0.26893	1.25758
H	4.31865	0.12169	1.26732
H	2.78769	0.06934	2.16862
H	3.34520	-1.36242	1.28564
C	-2.50821	1.75390	-0.00004
H	-3.53021	2.14692	-0.00004
H	-2.00329	2.14743	0.89029
H	-2.00331	2.14738	-0.89040
C	-3.29380	-0.26908	-1.25747
H	-4.31873	0.12154	-1.26719
H	-2.78783	0.06909	-2.16858
H	-3.34527	-1.36257	-1.28541
C	-3.29376	-0.26901	1.25752
H	-4.31869	0.12161	1.26725
H	-3.34523	-1.36251	1.28551
H	-2.78776	0.06920	2.16860
